# Abstract Supplement HIV Glasgow 10–13 November 2024, Glasgow, UK/Virtual

**DOI:** 10.1002/jia2.26370

**Published:** 2024-11-08

## Contents

Commonly‐used Abbreviations 1

Oral Presentations

Experience in the Implementation of Long‐Acting Treatment 3

Antiretroviral Treatment Strategies 5

Infection Prevention 6

Integrase Strand Transfer Inhibitor (INSTI) Resistance 7

Co‐morbidities and Co‐infections 9

PrEP‐ing for the Future 17

Poster Presentations

ARV‐based prevention—Vertical transmission 20

ARV‐based prevention—PEP 28

ARV‐based prevention—PrEP 29

Treatment strategies—Novel therapeutic targets (phase I and II) 45

Treatment strategies—RCTs: Oral and injectable therapy in first line and suppressed switch populations 47

Treatment strategies—Real‐world and implementation science studies oral and injectable therapy 66

Treatment strategies—Treatment experienced adults (second line and multi‐drug resistance studies) 122

Treatment strategies—Models of care for ageing/frail populations including virological failure and switching 147

Treatment strategies—Rapid ART initiation 149

Treatment strategies—Adherence 158

Clinical management considerations—Women 164

Clinical management considerations—Late presenters 170

Clinical management considerations—People who inject drugs (PWID) 180

Clinical management considerations—Transgender people 181

Clinical management considerations—Adolescents 182

Clinical management considerations—Paediatrics 183

Clinical management considerations—Drug‐drug interactions 187

Cure/post‐treatment control 194

Opportunistic infections and AIDS‐defining cancers 200

Clinical pharmacology 206

Community‐based treatment and prevention initiatives, including primary care screening 209

Public health strategies including of policy options 219

Cost and cost‐effectiveness 233

Models of care: evaluation of ARV delivery and coverage 237

Co‐morbidities and complications of disease and/or treatment—Ageing and frailty 245

Co‐morbidities and complications of disease and/or treatment—Cardiovascular/metabolic including weight gain 249

Co‐morbidities and complications of disease and/or treatment—Malignancies: non‐AIDS defining 273

Co‐morbidities and complications of disease and/or treatment—Neurological 280

Co‐morbidities and complications of disease and/or treatment—Renal 282

Co‐morbidities and complications of disease and/or treatment—Mental health disorders 287

Co‐morbidities and complications of disease and/or treatment—Other 291

People living with HIV and COVID‐19: Outcomes 304

People living with HIV and mpox virus 307

People living with HIV and sexually transmitted diseases 311

People living with HIV and tuberculosis 320

People living with HIV and viral hepatitis 323

People living with HIV and other diseases 329

Author Index 335

## COMMONLY‐USED ABBREVIATIONS

### HIV Glasgow Abbreviations List

### Common abbreviations used throughout this Supplement are noted here to avoid repetition of definitions

**Table:** 

2DR	two‐drug regimen
3DR	three‐drug regimen
3TC	lamivudine
ABC	abacavir
AE	adverse event
AHA	American Heart Association
aHR	adjusted hazard ratio
AI	artificial intelligence
AIDS	acquired immunodeficiency syndrome
ANOVA	analysis of variance
anti‐HBc	hepatitis B core antibody
aOR	adjusted odds ratio
ART	antiretroviral therapy
ARV	antiretroviral
ATV	atazanavir
ATV/r	atazanavir/ritonavir
AZT	zidovudine
B	bictegravir
BHIVA	British HIV Association
BIC	bictegravir
BID	twice‐daily
BL	baseline
BMI	body mass index
BP	blood pressure
c (cobi)	cobicistat
C	cobicistat
CAB	cabotegravir
cART	combination antiretroviral therapy
CDC	Centers for Disease Control and Prevention
CI	confidence interval
CNS	central nervous system
COBI	cobicistat
CT	computed tomography
DAA	direct‐acting antivirals
DDI	drug‐drug interaction
DNA	deoxyribonucleic acid
DOR	doravirine
doxyPEP	doxycycline post‐exposure prophylaxis
DRV	darunavir
DRV/b	boosted darunavir
DRV/c	cobicistat‐boosted darunavir
DRV/r	ritonavir‐boosted darunavir
DTG	dolutegravir
E	elvitegravir
EACS	European AIDS Clinical Society
ECDC	European Centre for Disease Prevention and Control
EEA	European Economic Area
EFV	efavirenz
ELISA	enzyme‐linked immunosorbent assay
EMA	European Medicines Agency
ETR	etravirine
EVG	elvitegravir
EVG/c	cobicistat‐boosted elvitegravir
EU	European Union
F	emtricitabine
FDA	United States Food and Drug Administration
FTC	emtricitabine
FU	follow‐up
GBMSM	gay, bisexual and other men who have sex with men
GFR	glomerular filtration rate
GP	general practitioner
HAART	highly active antiretroviral therapy
HbA1c	haemoglobin A1c
HBcAb	hepatitis B core antibody
HBeAg	hepatitis B e‐antigen
HBsAb	hepatitis B surface antibody
HBsAg	hepatitis B surface antigen
HBV	hepatitis B virus
HCV	hepatitis C virus
HCVAb	hepatitis C antibody
HDL	high‐density lipoprotein
HIRI	HIV incidence risk index
HIV	human immunodeficiency virus
HLA	human leukocyte antigen
HPV	human papillomavirus
HR	hazard ratio
HRQoL	health‐related quality of life
HSV	herpes simplex virus
IC	inhibitory concentration
IFN	interferon
IgG	immunoglobulin G
IM	intramuscular
INI	integrase inhibitor
INSTI	integrase strand transfer inhibitor
IQR	interquartile range
ITT	intention‐to‐treat
IV	intravenous
LA	long‐acting
LAR	long‐acting regimen
LDL	low‐density lipoprotein
LGBT	lesbian, gay, bisexual, transgender
LPV	lopinavir
LPV/r	ritonavir‐boosted lopinavir
MedDRA	Medical Dictionary for Regulatory Activities
MRI	magnetic resonance imaging
MSM	men who have sex with men
MSW	men who have sex with women
NEV	nevirapine
NGO	non‐governmental organisation
NHS	National Health Service
NNRTI	non‐nucleoside reverse transcriptase inhibitor
NRTI	nucleoside reverse transcriptase inhibitor
NVP	nevirapine
OR	odds ratio
PCR	polymerase chain reaction test
PD1	programmed death‐1
PDL1	programmed death‐ligand‐1
PEP	post‐exposure prophylaxis
PI	protease inhibitor
PI/b	boosted protease inhibitor
PI/r	ritonavir‐boosted protease inhibitor
PLWH	people living with HIV
PrEP	pre‐exposure prophylaxis
PRO	patient‐reported outcomes
PWID	persons who inject drugs
Q	quartile
QD	once‐daily
QoL	quality of life
/r	boosted with ritonavir
RAL	raltegravir
RAM	resistance‐associated mutation
RBV	ribavirin
RCT	randomized controlled trial
RNA	ribonucleic acid
RR	risk ratio
RTV	ritonavir
SC	subcutaneous
SD	standard deviation
SE	standard error
SQVr	ritonavir‐boosted saquinavir
STD	sexually transmitted disease
STI	sexually transmitted infection
TAF	tenofovir alafenamide
TB	tuberculosis
TDF	tenofovir disoproxil fumarate
TPVr	ritonavir‐boosted tipranavir
TXF	tenofovir alafenamide or tenofovir disoproxil fumarate
U = U	undetectable = untransmissible
VF	virological failure
VL	viral load
VS	virological suppression
WHO	World Health Organization
WHODD	WHO Drug Dictionary
XTC	lamivudine or emtricitabine

## ORAL PRESENTATIONS

### Experience in the Implementation of Long‐Acting Treatment

### O21 Once‐weekly islatravir plus lenacapavir in virologically suppressed PWH: week 48 safety, efficacy and metabolic changes

Amy E. Colson ^1^, Gordon E. Crofoot^2^, Peter J. Ruane^3^, Moti N. Ramgopal^4^, Alexandra W. Dretler^5^, Ronald G. Nahass^6^, Gary I. Sinclair^7^, Mezgebe Berhe^8^, Fadi Shihadeh^9^, Shan‐Yu Liu^9^, Stephanie Klopfer^10^, Sharline Madera^9^, Hadas Dvory‐Sobol^9^, Martin S. Rhee^9^, Elizabeth G. Rhee^10^, Jared Baeten^9^, Joseph Eron^11^

^1^Community Resource Initiative, Boston, MA, USA. ^2^The Crofoot Research Center, Houston, TX, USA. ^3^Ruane Clinical Research, Los Angeles, CA, USA. ^4^Midway Immunology & Research Center, Fort Pierce, FL, USA. ^5^Metro Infectious Disease Consultants, Decatur, GA, USA. ^6^ID Care, Hillsborough, NJ, USA. ^7^Prism Health North Texas, Dallas, TX, USA. ^8^North Texas Infectious Diseases Consultants, Dallas, TX, USA. ^9^Gilead Sciences, Foster City, CA, USA. ^10^Merck & Co., Inc., Rahway, NJ, USA. ^11^University of North Carolina, Chapel Hill, NC, USA

**Background**: Islatravir (ISL), a nucleoside reverse transcriptase translocation inhibitor, and lenacapavir (LEN), a capsid inhibitor, have potent anti‐HIV‐1 activity with pharmacokinetic profiles supporting once‐weekly oral dosing. In the current trial (NCT05052996), weekly oral ISL+LEN maintained a high rate of virological suppression at week (W) 24. Here, we report W48 results.

**Materials and methods**: In this phase II, randomized, open‐label, active‐controlled study, virologically suppressed adults on bictegravir/emtricitabine/tenofovir alafenamide (B/F/TAF) were randomized 1:1 to receive weekly oral ISL 2 mg + LEN 300 mg or to continue daily B/F/TAF. Virological outcomes, safety, lymphocyte counts, weight, body mass index (BMI) and adherence (by pill count) were assessed.

**Results**: Overall, 104 participants (52/group) were randomized and dosed. Median age was 40 years (range 26–76) and 19 (18.3%) were assigned female at birth. At W48, 49 (94.2%) participants assigned to ISL+LEN and 48 (92.3%) assigned to B/F/TAF had HIV‐1 RNA <50 copies/ml; no participants had HIV‐1 RNA ≥50 copies/ml; three (5.8%) and four (7.7%) participants, respectively, had no available data (Table 1). AEs occurring in ≥10% of the ISL+LEN group were upper respiratory tract infection (13.5%), COVID‐19 (11.5%) and diarrhoea (11.5%). No grade ≥3 AEs related to ISL+LEN were reported. Two participants (3.8%) discontinued ISL+LEN due to unrelated AEs. There were no significant differences between ISL+LEN and B/F/TAF groups in mean change from baseline in CD4^+^ T cells (−12 vs. −29/µl; *p* = 0.88) or lymphocytes (−0.07 vs. −0.03 × 103/µl; *p* = 0.23). Median (IQR) weight and BMI changes from baseline were −0.2 kg (−2.7, 3.0) and −0.05 kg/m^2^ (−0.95, 0.91) for ISL+LEN, and 0.1 kg (−1.9, 2.7) and 0.03 kg/m^2^ (−0.64, 0.84) for B/F/TAF. Mean adherence through W48 was 99.2% for ISL+LEN and 98.1% for B/F/TAF.

**O21: Table 1:** Week 48 virological outcome data by FDA Snapshot algorithm

*n* (%)	ISL+LEN (*n* = 52)	B/F/TAF (*n* = 52)
HIV‐1 RNA <50 copies/ml	49 (94.2)	48 (92.3)
ISL+LEN versus B/F/TAF difference in percentage (95% CI)	1.9 (−9.3, 13.6)	
HIV‐1 RNA ≥50 copies/ml	0	0
No virological data in week 48 window	3 (5.8)	4 (7.7)
Discontinued study drug due to unrelated AE	2 (3.8)^a^	0
Discontinued study drug due to other reasons	1 (1.9)^a^	3 (5.8)^a^
Missing data during window but on study drug	0	1 (1.9)

*Note*: AEs leading to study discontinuation included large intestine perforation and renal colic (*n* = 1), and hepatitis B (*n* = 1), both previously reported.

Abbreviations: AE, adverse event; B/F/TAF, bictegravir/emtricitabine/tenofovir alafenamide; FDA, Food and Drug Administration; ISL, islatravir; LEN, lenacapavir.

^a^ Last available on‐treatment HIV‐1 RNA <50 copies/ml.

**Conclusions**: Weekly oral ISL+LEN maintained a high rate of virological suppression at W48. Through week 48, ISL+LEN was well tolerated without significant changes in CD4^+^ T‐cell count, lymphocyte count or weight. Adherence to this weekly oral dosing regimen was high. ISL+LEN has the potential to become the first weekly oral complete regimen for the treatment of HIV‐1 infection.

### O22 Cabotegravir and rilpivirine concentrations and HIV‐1 RNA suppression in male and female genital fluids and rectal tissue in people with HIV on antiretroviral therapy with long‐acting intramuscular cabotegravir plus rilpivirine

Analuz Fernández^1^, Sofia Scévola^1^, Jordi Niubó^1^, Craig Sykes^2^, Amanda P. Schauer^2^, Camila Piatti^1^, Sandra Morenilla^1^, Alicia Sedó^1^, Irene Soriano^1^, Benito Garcia^1^, Daniel Medina^1^, Juan Tiraboschi^1^, Maria Saumoy^1^, Mackenzie L. Cottrell^2^, Arkaitz Imaz ^1^

^1^Infectious Diseases, Bellvitge University Hospital, L'Hospitalet de Llobregat, Barcelona, Spain. ^2^Eshelman School of Pharmacy, University of North Carolina at Chapel Hill, Chapel Hill, NC, USA

**Background**: This study evaluated the distribution of cabotegravir (CAB) and rilpivirine (RPV) and HIV‐RNA suppression in genital fluids and rectum in adults receiving intramuscular long‐acting (LA) CAB+RPV.

**Methods**: Virologically suppressed adults with HIV‐1, on stable ART, without resistance to CAB or RPV were included. ART was switched to oral CAB+RPV followed by intramuscular CAB 600 mg + RPV 900 mg at weeks 4 and 8, and every 8 weeks onwards. At week 16, pre‐dose total drug concentrations (C_trough_) and protein‐bound fractions were measured in blood plasma (BP) and seminal plasma (SP) and rectal tissue (RT) in males, and cervicovaginal fluid (CVF) in females. HIV‐1 RNA was assessed in BP, SP, rectal fluid (RF) and CVF at baseline and at week 16. Validated liquid chromatography‐tandem mass spectrometry (LC‐MS/MS) was used to quantify drug concentrations, and HIV‐1 RNA was determined by real‐time PCR. Data are presented as median (range).

**Results**: Sixteen males and 15 females completed the study. Age was 43 years (28–66), time on ART 132 months (12–311) and CD4‐count 729 cells/µl (380–1146). Total CAB and RPV C_trough_ in SP were 23.3 (10.10–130.00) and 2.65 (1.47–9.37) ng/ml, corresponding to 2% (1–11%) and 8% (4–12%) of BP concentrations, respectively. Total CAB and RPV C_trough_ in RT were 90.01 (54.35–138.77) and 38.57 (19.68–79.64) ng/g, corresponding to 9% (5–20%) and 112% (72–142%) of BP concentrations, respectively. Total CAB and RPV C_trough_ in CVF were 15.63 (1.79–428.23) and 21.41 (0.13–47.77) ng/ml, corresponding to 0.73% (0.14–16%) and 48% (0.74–107%) of BP concentrations, respectively (Table 1). The CAB protein‐bound fraction in SP, RT and CVF was 57%, 72% and 32%, respectively, compared to >99% in BP. For RPV, it was 97%, 94% and 95% in SP, RT and CVF, respectively, compared to >99% in BP (Table 1). HIV‐1 RNA remained <20 copies/ml in BP, SP, RF and CVF from baseline through week 16 in all subjects.

**O22: Table 1:** 

	**Blood plasma**			**SP**			**RT**			**CVF**		
	**Total drug C_trough_ (ng/ml)**	**Protein‐bound (%)**	**Estimated protein‐unbound C_trough_ (ng/ml)**	**Total drug C_trough_ (ng/ml)**	**Protein‐bound (%)**	**Estimated protein‐unbound C_trough_ (ng/ml)**	**Total drug C_trough_ (ng/g)**	**Protein‐bound (%)**	**Estimated protein‐unbound C_trough_ (ng/g)**	**Total drug C_trough_ (ng/ml)**	**Protein‐bound (%)**	**Estimated protein‐unbound C_trough_ (ng/ml)**
Cabotegravir												
Median (range)	Male 1063 (537−1900) Female 1470 (1170−2970)	Male 99.94 (99.85−99.96) Female 99.93 (99.88−99.94)	Male 0.66 (0.40−0.95) Female 1.03 (0.70−2.30)	23.30 (10.10−130.00)	56.56 (44.08−77.17)	9.01 (5.20−16.43)	90.01 (54.35−138.77)	71.66 (46.04−78.44)	27.10 (13.47−59.50)	15.63 (1.79−428.23)	31.86 (12.86−50.73)	13.73 (2.60−283.11)
Rilpivirine												
Median (range)	Male 36.40 (17.20−79.20) Female 37.50 (15.60−103.00)	Male 99.88 (99.78−99.90) Female 99.89 (99.74−99.92)	Male 0.04 (0.03−0.09) Female 0.04 (0.03−0.08)	2.65 (1.47−9.37)	97.03 (94.49−99.19)	0.08 (0.04−0.19)	38.57 (19.68−79.64)	93.68 (80.27−96.86)	2.70 (1.41−5.51)	21.41 (0.13−47.77)	94.93 (46.84−97.86)	0.81 (0.14−13.22)

Abbreviations: CVF, cervicovaginal fluid; RT, rectal tissue; SP, seminal plasma.

**Conclusions**: Despite the low distribution of total CAB in SP, RT and CVF, the estimated protein‐unbound (active fraction) concentrations highly exceeded the half maximal effective concentration (EC50) value for wild‐type HIV‐1 (0.10 ng/ml). Low RPV concentrations were observed in SP, whereas median total and protein‐unbound concentrations exceeded the protein‐adjusted IC90 (12 ng/ml) and the EC50 (0.27 ng/ml), respectively, in both RT and CVF. HIV‐RNA suppression <20 copies/ml was maintained in BP, genital and rectal fluids after switching to LA CAB+RPV.

### O23 Efficacy and safety analysis of lenacapavir with broadly neutralizing antibodies, teropavimab and zinlirvimab, in people with HIV‐1 highly sensitive to one or both broadly neutralizing antibodies

Susan J. Little^1^, Paul P. Cook ^2^, Kwad Mponponsuo^3^, Edwin DeJesus^4^, Gordon E. Crofoot^5^, Hailin Huang^6^, Linda Gorgos^7^, Sean E. Collins^3^, Joseph J. Eron^8^

^1^Division of Infectious Diseases, University of California, San Diego, CA, USA. ^2^Division of Infectious Diseases, East Carolina University, Greenville, NC, USA. ^3^Clinical Development, Gilead Sciences, Inc., Foster City, CA, USA. ^4^Infectious Diseases, Orlando Immunology Center, Orlando, FL, USA. ^5^Infectious Diseases, The Crofoot Research Center, Houston, TX, USA. ^6^Biostatistics, Gilead Sciences, Inc., Foster City, CA, USA. ^7^Infectious Diseases, AXCES Research Group, Santa Fe, NM, USA. ^8^Infectious Diseases, University of North Carolina, Chapel Hill, NC, USA

**Background**: Lenacapavir (LEN), the first‐in‐class HIV‐1 capsid inhibitor approved for the treatment of multidrug‐resistant HIV‐1 infection in adults, is being evaluated as part of a twice‐yearly combination treatment regimen with broadly neutralizing antibodies (bNAbs) teropavimab (TAB, GS‐5423) and zinlirvimab (ZAB, GS‐2872). In a randomized, phase Ib study (NCT04811040), participants received LEN + TAB with a low or high dose of ZAB. We evaluated pooled efficacy and safety of the combination regimen, stratified by dose of ZAB.

**Methods**: Virologically suppressed adults (HIV‐1 RNA <50 copies/ml) with HIV‐1, highly susceptible to both bNAbs (primary cohort) or only one bNAb (pilot cohort) by HIV‐1 proviral phenotype (PhenoSense monoclonal antibody assay IC_90_ ≤2 µg/ml) were enrolled. Cohorts were randomized 1:1 to two active treatment groups: LEN (927 mg subcutaneously after oral loading) + TAB (30 mg/kg intravenously [IV]) + ZAB (low dose, 10 mg/kg IV; or high dose, 30 mg/kg IV). Week 26 data for the primary cohort have been previously reported [1]. In this analysis, we assessed pooled virological outcomes (using US FDA Snapshot algorithm) and safety of both cohorts by treatment group through Week 26.

**Results**: Thirty‐two participants were randomized; 31 received the complete study regimen; one restarted antiretroviral therapy due to a major protocol violation and was excluded from analyses. Participants were: 19% female, 22% Black and 31% Hispanic/Latinx. At baseline, median age was 48 years and mean CD4 count was 992 cells/µl. At week 26, 3/14 (21%) in the low dose, and 0/16 (0%) in the high dose group had HIV‐1 RNA ≥50 copies/ml (Table 1). There were no serious adverse events (AEs) related to study drug; the most common AEs were injection site reactions related to subcutaneous LEN administration.

**O23: Table 1:** Efficacy as determined by US FDA‐defined Snapshot algorithm at W26

	LEN + TAB + ZAB 10 mg/kg	LEN + TAB + ZAB 30 mg/kg
	(*n* = 14)	(*n* = 16)
HIV‐1 RNA ≥50 copies/ml, *n* (% [95% CI])	3 (21.4 [4.7−50.8])	0 (0.0 [0.0−20.6])
HIV‐1 RNA <50 copies/ml, *n* (% [95% CI])	11 (78.6 [49.2−95.3])	15 (93.8 [69.8−99.8])
No virological data in W26 window, *n* (%) (discontinued study drug due to other reasons^a^ and last available HIV‐1 RNA <50 copies/ml)	0	1^b^ (6.3)

Abbreviations: AE, adverse event; LEN, lenacapavir; TAB, teropavimab; W, week; ZAB, zinlirvimab.

^a^ Reasons other than AE/death or lack of efficacy (e.g. discontinued study drug due to investigator's discretion, participant decision, lost to follow‐up, non‐compliance with study drug, protocol violation, pregnancy or study terminated by sponsor).

^b^ Withdrew from the study after W12.

**Conclusions**: All participants who received LEN, TAB and high‐dose ZAB maintained viral suppression with no difference in safety or tolerability between dose groups. These early phase results suggest that high treatment efficacy for the long‐acting regimen of LEN, TAB and high‐dose ZAB can be achieved when at least one antibody is highly active in people with HIV highly susceptible to one or both bNAbs.

**Reference**

1. Eron JJ, Little SJ, Crofoot G, Cook P, Ruane PJ, Jayaweera D, et al. Safety of teropavimab and zinlirvimab with lenacapavir once every 6 months for HIV treatment: a phase 1b, randomised, proof‐of‐concept study. Lancet HIV. 2024;11:e146‐55.

### Antiretroviral Treatment Strategies

### O24 Comparable efficacy and safety of dolutegravir/lamivudine to a three‐drug regimen among ARV naive people living with HIV with CD4 <200/mm^3^: the DOLCE study

Maria Ines Figueroa^1^, Carlos Brites^2^, Diego Cecchini^3^, Aline Santos Ramalho Teixeira Benvenuto^4^, Jose Luis Francos^5^, Marcus Lacerda^6^, Maria Jose Rolon^7^, Jose Valdez Madruga^8^, Eduardo Sprinz^9^, Tamara Newman Lobato Souza^10^, Pablo Parenti^11^, Demetrius Montenegro^12^, Daniela Converso^1^, Gissella Mernies^1^, Omar Sued^13^, Pedro Cahn ^1^

^1^Research Department, Fundacion Huesped, Buenos Aires, Argentina. ^2^Fundacao Bahiana de Infectologia, Salvador, Brazil. ^3^Hospital General de Agudos Dr Cosme Argerich, Buenos Aires, Argentina. ^4^Hospital Geral de Nova Iguacu, Nova Iguacu, Brazil. ^5^Hospital de Infecciosas Francisco J. Muñiz, Buenos Aires, Argentina. ^6^Fundacao de Medicina Tropical de Amazonas—FMT/IMT/AM, Manaus, Brazil. ^7^Hospital General de Agudos Juan A Fernandez, Buenos Aires, Argentina. ^8^Centro de Treinamiento e Referencia DST/AIDS, Sao Paulo, Brazil. ^9^Hospital de Clinicas de Porto Alegre, Porto Alegre, Brazil. ^10^Centro de Pesquisa Instituto de Infectologia Emilio Ribas, Sao Paulo, Brazil. ^11^Instituto CAICI, Rosario, Argentina. ^12^Hospital Universitario Oswaldo Cruz, Universidade de Pernambuco, Recife, Brazil. ^13^Panamerican Health Organization, Washington, DC, USA

**Background**: Dolutegravir/lamivudine dual therapy (DTG/3TC) (DT) has demonstrated non‐inferiority compared to triple therapy (TT) in the GEMINI trials. The population with fewer than 200 CD4 cells/mm^3^ had a lower response rate, though this was not related to virological failure.

**Materials and methods**: DOLCE is a randomized, exploratory, open‐label, multicentre study conducted in Argentina and Brazil, to assess the antiviral activity at week 48 of DTG/3TC among ART‐naïve HIV participants with CD4 counts ≤200 cells/mm^3^. Participants were randomly assigned in a 2:1 ratio to receive DTG/3TC single tablet regimen (STR) or DTG+TDF/XTC, stratified by country and by plasma viral load (pVL) at screening (> or ≤100,000 copies/ml). Primary endpoint: proportion of participants with pVL <50 copies/ml at week 48 (FDA Snapshot analysis for the intent‐to‐treat exposed [ITT‐E] population). Efficacy and safety results at week 48 are reported. NCT04880395.

**Results**: Baseline characteristics were similar in both arms (*n* = 230): median age 35 years, 77% male, 48.3% heterosexual, median CD4 cell count 116 (IQR 53–188), median pVL 151,000 copies/ml (IQR 49,027–446,947); 43.4% had CD4 <100 cells/mm^3^ and 69%, pVL >100,000 copies/ml. CDC stage C: 33%. At week 48, 82.2% in the DT (125/152) versus 80.5% (62/77) with TT achieved pVL <50 copies/ml, adjusted treatment difference 2% (95% CI −8.7, 12.8%) in the ITT‐E analysis. Participants with baseline pVL >100,000 copies/ml showed 80.9% response in DT and 76.6% in TT, adjusted treatment difference: 5.1% (95% CI −10.1, 20.3%). Per‐protocol analysis: 91.9% responded in the DT and 91.2% in the TT, adjusted treatment difference 1.8% (95% CI −6.3, 9.9%). No statistically significant differences were observed between arms in different analyses (Table 1). CD4 count change from baseline to week 48: +200 cells/mm^3^ in DT, +177 cells/mm^3^ in TT. Ten participants developed immune reconstitution inflammatory syndrome: DT: 6/153 (3.9%); TT: 4/77 (5.2%). Severe adverse events (*n* = 27) were reported: DT 17/153 (11.1%); TT: 10/77 (12.9%). No difference in safety parameters between arms was observed.

**O24: Table 1:** Efficacy analysis. Primary outcome viral load <50 copies/ml at week 48

	Total	Dual therapy (DT)	Triple therapy (TT)	Adjusted risk difference (95% CI)	Non‐inferiority *p*‐value^a^
ITT‐E (global *n* = 229; TT *n* = 77; DT *n* = 152) *n* (%) [95% CI]	187 (81.7%) [76−86%]	125 (82.2%) [75−88%]	62 (80.5%) [70−88%]	2.0% (−8.7, 12.8%)	0.016
ITT‐E, baseline VL >100,000 copies/ml (global *n* = 141; TT *n* = 47; DT *n* = 94) *n* (%) [95% CI]	112 (79.4%) [72−86%]	76 (80.9%) [71−88%]	36 (76.6%) [62−87%]	5.1% (−10.1, 20.3%)	0.026
Per protocol (global *n* = 204; TT *n* = 68; DT *n* = 136) *n* (%) [95% CI]	187 (91.7%) [87−95%]	125 (91.9%) [86−96%]	62 (91.2%) [81−96%]	1.8% (−6.3, 9.9%)	0.005

^a^One‐sided non‐inferiority test, with a significance level of 0.05. This gives a confidence interval of 90% for the difference between treatment arms (a narrower CI than 95% CI). The non‐inferiority margin (10%) is not included in the 90% CI, therefore, non‐inferiority can be claimed. For the ITT‐E, the CI is (−8.3, 11.7%); for the ITT‐E, baseline VL >100,000 copies/ml, the CI is (−9.5, 18.0%); and in the per protocol analysis, the CI for the difference is (−7.2, 8.7%).

**Conclusions**: DTG/3TC achieved comparable results to triple therapy, meeting non‐inferiority criteria in a severely immunosuppressed population with low CD4 counts and high viral load. pVL slope (Figure 1), time to viral suppression and CD4 recovery were also comparable between arms. Similar results were observed in patients with pVL >100,000 copies/ml. This study adds information regarding the efficacy and safety of dual therapy with DTG/3TC, regardless of baseline CD4 counts and viral load.

**O24: Figure 1:**
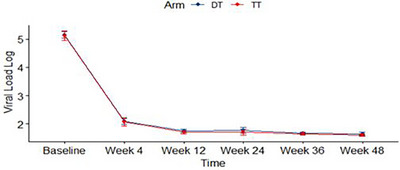
Mean viral load log over time on DT and TT arms.

### Infection Prevention

### O25 Post‐exposure prophylaxis with doxycycline (DoxyPEP) in a cohort of men who have sex with men (MSM) in high risk for sexually transmitted infections (STI): the PRIDOX study

Rosario Palacios ^1^, Cristina Gómez‐Ayerbe^1^, María López‐Jodar^1^, Salvador Martín‐Cortés^1^, Isabel Ascensión Pérez‐Hernández^1^, Andrea Prolo^1^, Marina Villalobos^1^, Victoria García‐López^2^, Jesús Santos^1^

^1^Infectious Diseases Unit, Hospital Universitario Virgen de la Victoria, Málaga, Spain. ^2^Microbiology Department, Hospital Universitario Virgen de la Victoria, Málaga, Spain

**Background**: Clinical trials have shown the high efficacy of doxycycline as STI post‐exposure prophylaxis (DoxyPEP), but real‐world data are limited. We aimed to analyse the impact of DoxyPEP on the incidence of *Chlamydia trachomatis* (CT), *Neisseria gonorrhoeae* (NG) and syphilis (SPL) in a high‐risk MSM cohort.

**Materials and methods**: PrEP users in follow‐up at our clinic, meeting criteria of high risk for STI similar to the IPERGAY and DoxiPEP trials [1,2], were offered to be included in a DoxyPEP programme. PrEP users who started DoxyPEP between March 2023 and June 2024 are included in this analysis. The pre‐DoxyPEP period was considered from the date starting PrEP to the date starting DoxyPEP, and the post‐DoxyPEP period from DoxyPEP start to 30 June 2024, or DoxyPEP discontinuation. Subjects were tested at baseline, each visit, and when symptomatic for NG and CT by PCR in pharynx, rectum and urine, and serological tests for SPL; NG culture was also conducted. Additionally, microbiological cultures for multidrug‐resistant bacteria were performed on nasal and rectal samples at DoxyPEP start and at 48 weeks. The incidence of the first episode of CT, NG and SPL was analysed and compared between periods using Cox proportional hazard models.

**Results**: Among 846 active PrEP users, 189 (22.3%) received DoxyPEP; all were MSM with a mean age of 39±8.9 years. Median follow‐up was 464±346 days and 257±142 days for the pre‐DoxyPEP and post‐DoxyPEP periods. The incidence rates per 100 PY of the first episode for each STI in the pre‐ and post‐DoxyPEP periods were: 31.8 and 8.53 (*p* = 0.0001) for CT, 41.7 and 26.6 (*p* = 0.0228) for NG, and 21.6 and 2.52 (*p* = 0.0001) for SPL. NG culture was positive in 33% of PCR‐positive samples; 25% of isolates were tetracycline‐resistant, similar to those not on DoxyPEP (42%; *p* = 0.2). DoxyPEP was discontinued in five (2.6%) participants (two adverse events, two due to reduced STI risk, one for medical reasons). Microbiological cultures for multidrug‐resistant bacteria were available in 150 (79.8%) participants, with 16 (10.6%) positive at baseline and 13 (8.6%) at 48 weeks.

**Conclusions**: DoxyPEP was well accepted as part of routine PrEP care, significantly reducing STI incidence with good tolerance. Our data demonstrate the efficacy and safety of this strategy in a real‐world setting. The impact of DoxyPEP on the emergence of multidrug‐resistant germs or resistant NG has not been evident. However, its impact on bacterial resistance and microbiota requires further evaluation.

**References**

1. Molina JM, Charreau I, Chidiac C, Pialoux G, Cua E, Delaugerre C, et al. Post‐exposure prophylaxis with doxycycline to prevent sexually transmitted infections in men who have sex with men: an open‐label randomised substudy of the ANRS IPERGAY trial. Lancet Infect Dis. 2018;18:308‐17.

2. Luetkemeyer AF, Donnell D, Dombrowski JC, Cohen S, Grabow C, Brown CE, et al. Postexposure doxycycline to prevent bacterial sexually transmitted infections. N Engl J Med. 2023;388:1296‐306.

### O26 Both smallpox MVA vaccine and mpox infection produce potent immune response to mpox in people living with HIV

Maryam Khan ^1^, Scott Jones^2^, Hafiza Rahman^3^, Helena Miras^3^, John Thornhill^1^, Ashley Otter^2^, Chloe Orkin^1^

^1^SHARE Collaborative, Immunobiology, Blizard Institute, Queen Mary University of London, London, UK. ^2^Emerging Pathogen Serology, UK Health Security Agency, London, UK. ^3^Grahame Hayton Unit, Barts Health NHS Trust, London, UK

**Background**: The 2022 mpox outbreak predominantly affected men who have sex with men (MSM) and up to 40% of those affected were people living with HIV (PLWH). The predominant control strategy applied during the outbreak was the use of modified vaccinia virus Ankara (MVA), a third‐generation smallpox vaccine. This vaccine produces a potent immune response against monkeypox virus (MPXV). However, the vaccine efficacy and stability over time in PLWH are unclear. We compare serological responses to MPXV in seven PLWH with prior mpox without vaccination and seven PLWH who received two doses of MVA >1 month apart.

**Methods**: Study participants were PLWH infected with MPXV “prior infection cohort” (*n* = 7) or PLWH vaccinated with MVA “vaccinated cohort” (*n* = 7) in 2022. Plasma samples were taken at single time‐point at least 2 months post‐convalescence or post‐vaccination, respectively. Serological responses to MPXV were measured using Luminex with nine MPXV (A5L, A27L, A29L, A35R, B2R, B6R, E8L, H3L, M1R) and three Vaccinia virus (VACV) (V A27L, V A33R, V B5R) recombinant antigens and reported as mean fluorescent intensity (MFI).

**Results**: All study participants were male and virally suppressed on ART (<20 copies HIV‐1 RNA copies/ml). Median time at point of sampling from infection was 18 months (IQR 17–19) and median time from vaccination was 15 months (IQR 14–16). All participants had CD4, CD8 counts (cells/mm^3^) and CD4:CD8 ratio within normal range (median CD4 686, IQR 686–1144; median CD8 500, IQR 390–802; median CD4:CD8 1.8, IQR 1–2). Twelve of 14 participants produced potent serological responses to MPXV and VACV antigens. No difference in MFI was observed in response to eight MPXV antigens three VACV antigens between “prior infection” cohort and “vaccinated” cohorts. Median MFI for A27L, antigen specific for infection, was significantly higher (*p* < 0.05) in prior infection cohort (median 2819, IQR 2069–3909) versus vaccinated cohort (median 113, IQR 33–2201). Notably, two PLWH in vaccinated cohort produced no response to any antigens.

**Conclusions**: Both MVA and prior infection produce potent serological responses to MPXV in PLWH. These findings are relevant to an immune‐competent cohort who are virally suppressed with no evidence of immune dysfunction. Humoral immunity responses to infection and vaccination are being evaluated and the finding of no response in certain PLWH requires further investigation.

### Integrase Strand Transfer Inhibitor (INSTI) Resistance

### O31 Clinical features and resistance patterns during second‐generation INSTI failure: the ROSETTA registry

Mafalda N. S. Miranda ^1^, Carole Seguin‐Devaux^2^, Ana B. Abecasis^3^, Fatima Bikhezar^1^, Ricardo Diaz^4^, Joseph Fokam^5^, Laurence Guilorit^2^, Thibault Mesplede^6^, Suzanne McCluskey^7^, Monique Nijhuis^1^, Dimitrios Paraskevis^8^, Karol Serwin^9^, Annelies Verbon^10^, Berend van Welzen^10^, Ferdinand Wit^11^, Ivailo Alexiev^12^, Josip Begovac^13^, Michael Böhm^14^, Federico Garcia^15^, Perpétua Gomes^16^, Itzchak Levi^17^, Rolf Kaiser^14^, Josep M. Llibre^18^, Nadine Lubke^24^, Kaja Mielczak^9^, Orna Mor^19^, Milosz Parczewski^9^, Murat Sayan^20^, Alessandra Simões Bassini^4^, Karolien Stoffels^21^, Margarida Veloso^22^, Jeroen van Kampen^6^, Annemarie M. J. Wensing^23^

^1^Translational Virology, Department of Medical Microbiology, University Medical Center Utrecht, Utrecht, Netherlands. ^2^Infection and Immunotherapy Research Group, Department of Infection and Immunity, Luxembourg Institute of Health, Alzette, Luxembourg. ^3^Global Health and Tropical Medicine (GHTM), Associate Laboratory in Translation and Innovation towards Global Health (LA‐REAL), Institute of Hygiene and Tropical Medicine, New University of Lisbon (IHMT/UNL), Lisbon, Portugal. ^4^University Federal de São Paulo, São Paulo, Brazil. ^5^Virology Laboratory, Chantal Biya International Reference Centre for Research on the Prevention and Management of HIV/AIDS (CIRCB), Yaoundé, Cameroon. ^6^Erasmus University Medical Center, Rotterdam, Netherlands. ^7^Division of Infectious Diseases, Harvard Medical School, Massachusetts General Hospital, Boston, MA, USA. ^8^Medical School of the National and Kapodistrian University of Athens, Athens, Greece. ^9^Pomeranian Medical University, Szczecin, Poland. ^10^Department of Infectious Diseases, University Medical Center Utrecht, Utrecht, Netherlands. ^11^Stichting HIV Monitoring, Amsterdam, Netherlands. ^12^National Center of Infectious and Parasitic Diseases, Sofia, Bulgaria. ^13^University Hospital for Infectious Diseases, Zagreb, Croatia. ^14^Institute of Virology, University Hospital of Cologne, University of Cologne, Cologne, Germany. ^15^University Hospital San Cecilio, Granada, Spain. ^16^Egas Moniz Center for Interdisciplinary Research (CiiEM), Egas Moniz School of Health & Science, Caparica, Portugal. ^17^Ministry of Health & Sheba Medical Center, Tel‐Aviv, Israel. ^18^University Hospital Germans Trias, Badalona, Spain. ^19^Central Virology Laboratory, Ministry of Health and School of Medicine, Tel Aviv University, Tel Aviv, Israel. ^20^Clinical Laboratory, PCR Unit, Kocaeli University, Kocaeli, Turkey. ^21^CHU Saint‐Pierre, UMC Sint‐Pieter, Brussels, Belgium. ^22^Laboratório de Biologia Molecular (LMCBM, SPC, ULSLO‐HEM), Lisbon, Portugal. ^23^Translational Virology, Department of Global Public Health & Bioethics, Julius Center for Health Sciences and Primary Care, University Medical Center Utrecht, Utrecht, Netherlands. ^24^Heinrich Heine University Hospital, Düsseldorf, Germany

**Background**: Second‐generation integrase inhibitors (2nd‐gen INSTI) are globally recommended for treatment of people with HIV (PWH) [1]. Although treatment failure may occur, selection of drug resistance is rare [2]. As such, there is limited information regarding risk factors and resistance patterns. The ROSETTA registry aims to systematically analyse otherwise scattered information related to 2nd‐gen INSTI therapy failure on a global scale.

**Methods**: In this planned interim analysis, we examine 125 cases of PWH experiencing virological failure to 2nd‐gen INSTI‐based antiretroviral therapy (ART) from Africa, America and Europe. Failure was defined as two consecutive plasma viral loads (VL) 50 cp/ml or one 200 cp/ml. Demographic, clinical and genotypic data were collected. Analysis of integrase sequences was performed with IAS‐USA‐tables 2022 and subtypes with COMET.

**Results**: One hundred and twenty‐five clinical datasets and 124 sequences passed quality control. Most participants were male (73.6%), with a median age of 46 years, harbouring different HIV‐1 subtypes: A1, A6, B, C, D, F1 and G; five circulating and two unique recombinant forms. The median time on ART was 23 months (IQR 7–91), median time to failure on 2nd‐gen INSTI was 13 months (IQR 6–26). One hundred and eleven cases experienced failure with dolutegravir (DTG), 10 cases with bictegravir (BIC) and four cases with cabotegravir (CAB). 91.2% had an NRTI‐backbone. Major INSTI resistance mutations were selected in 33 cases (26.6%): 28 cases with DTG failure, three with BIC, two with CAB. Median VL in cases without resistance (8951 cp/ml) was not statistically different from those with resistance (18,300 cp/ml) (*p* = 0.694). Seven cases with INSTI resistance had a VL <1000 cp/ml. INSTI resistance was detected in 39.1% (27/69) of cases with previous ART and in 10.9% (6/55) of cases without (*p* < 0.001). INSTI resistance was detected in 50% (12/24) of those with pre‐exposure to first‐generation (1st‐gen) INSTIs versus 21% (21/100) in those without (*p* = 0.004). Table 1 presents resistance patterns in relation to VL, subtype and previous INSTI usage.

**O31: Table 1:** Cases with virological failure to second‐generation INSTIs and major IAS‐USA INSTI resistance mutations

HIV‐1 subtype	*N*	Current failure to 2nd‐gen INSTI	Major and minor IAS‐USA INSTI resistance mutations at failure	Pre‐exposure to 1st‐gen INSTI	VL at failure (copies/ml)
A1	*n* = 1	DTG	155H	RAL	4360
A6	*n* = 1	DTG	140A+148R	EVG	7260
B	*n* = 1	BIC	97A+147G+155H	RAL	106
B	*n* = 2	CAB	140S+148K; 97A+138K+140S+148H	RAL; RAL	1490; 34,800
B	*n* = 2	DTG	97A+140S+148H; 138A+140S+148H	RAL; RAL	30,700; 18,300
B	*n* = 1	DTG	138K+140A+147G+148R+155H	EVG	371,000
D	*n* = 1	DTG	97A+140S+148H	RAL	345,365
F1	*n* = 1	DTG	74M+97A+138K+143R+147G	RAL	64
02_AG	*n* = 1	DTG	97A+147G+155H	RAL	13,309
95_02B	*n* = 1	DTG	92Q+138K+147G+155H	EVG	463,740
A1	*n* = 2	DTG	263K; 263K	−	99,003; 695
A6	*n* = 1	DTG	138K+147G	−	147,000
B	*n* = 2	BIC	74M+138A+140A+148R; 97A+138T+140S+148H	−	1170; 7000
B	*n* = 4	DTG	263K; 263K; 138K+147G+155H; 92Q+138A+147G+155H	−	33,308; 41,522; 699; 1176
C	*n* = 4	DTG	118R; 263K; 263K; 155H+263K	−	39,918; 38,190; 244; 16,541
D	*n* = 1	DTG	66I+74M+118R+138K	−	624
F1	*n* = 1	DTG	263K	−	311,894
G	*n* = 3	DTG	74M+118R; 263K; 263K	−	300; 450,000; 147,120
02_AG	*n* = 2	DTG	66I+74M+118R+138K+153Y; 138A+147G+155H	−	5812; 63,300
31_BC	*n* = 1	DTG	263K	−	38,190

*Note*: Data for each case is separated by “;”.

Abbreviations: BIC, bictegravir; CAB, cabotegravir; DTG, dolutegravir; EVG, elvitegravir; IAS‐USA, International Antiviral Society USA; *N*, number of cases; RAL, raltegravir.

**Conclusions**: The ROSETTA registry holds the largest collection of real‐life failures to 2nd‐gen INSTIs. The selection of INSTI resistance was already seen at relatively low VL. Notably, resistance mutations G118R and R263K were not observed in cases with previous 1st‐gen INSTI exposure, suggesting different resistance pathways based on past exposure.

**References**

1. Zhao AV, Crutchley RD, Guduru RC, Ton K, Lam T, Min AC. A clinical review of HIV integrase strand transfer inhibitors (INSTIs) for the prevention and treatment of HIV‐1 infection. Retrovirology. 2022;19(1):22.

2. Jeffrey JL, St Clair M, Wang P, Wang C, Li Z, Beloor J, et al. Impact of integrase sequences from HIV‐1 subtypes A6/A1 on the *in vitro* potency of cabotegravir or rilpivirine. Antimicrob Agents Chemother. 2022;66(3):e0170221.

### O32 Virological outcomes and associated factors among treatment‐naive patients with HIV‐1 on dolutegravir‐based regimen in a programmatic setting in Thailand

Napon Hiranburana ^1^, Opass Putcharoen^2^, Cheewanan Lertpiriyasuwat^3^, Sairat Noknoi^4^, Jiratchaya Sophonphan^5^, Supunnee Jirajariyave^6^, Stephen J. Kerr^7^, Ploenchan Chetchotisakd^8^, Chureeratana Bowonwatanuwong^9^, Kiat Ruxrungtham^9^, Anchalee Avihingsanon^5^

^1^HIV Netherlands Australia Thailand Research Collaboration (HIV‐NAT), Thai Red Cross AIDS Research Centre, Bangkok, Thailand. ^2^Infectious Disease, Medicine Faculty, Chulalongkorn University, Bangkok, Thailand. ^3^Disease Control, Division of AIDS and STI, Department of Disease Control, Ministry of Public Health, Bangkok, Thailand. ^4^Research, HIV‐NAT, Thai Red Cross AIDS Research Center, Bangkok, Thailand. ^5^Research, HIV Netherlands Australia Thailand Research Collaboration (HIV‐NAT), Thai Red Cross AIDS Research Centre, Bangkok, Thailand. ^6^Internal Medicine, Taksin Hospital, Bangkok, Thailand. ^7^Biostatistics Excellence Centre, Faculty of Medicine, Chulalongkorn University, Bangkok, Thailand. ^8^Faculty of Medicine, Srinagarind Hospital, Khon Kaen University, Khon Kaen, Thailand. ^9^Thai AIDS Society, Bangkok, Thailand

**Background**: Fixed dose combinations of tenofovir disoproxil fumarate/lamivudine/dolutegravir (TLD) have been scaled up as first‐line ART for people living with HIV (PLWH) in Thailand since 2020. There are limited data on the efficacy of TLD in programmatic settings where baseline plasma HIV‐1 RNA and drug resistance testing are not routinely performed. We assessed the efficacy of DTG‐based ART (mainly TLD) in the Thai National HIV/AIDS (NAP) and determined factors associated with virological failure (VF).

**Methods**: Thai PLWH aged ≥15 years initiating first‐line DTG‐based ART between 2020 and 2023, with at least one HIV viral load (VL) after DTG, were included. VF was defined as viral load (VL) ≥1000 copies/ml after ≥6 months of ART. The primary endpoint was the proportion of participants with VL ≤50 copies/ml. Factors associated with VF were analysed with death and loss to follow‐up (LTFU) as competing events. Vital status was confirmed with the Death Registry.

**Results**: Of 10,475 ART‐naïve PLWH initiating DTG‐based ART, 9472 (90.4%) started TLD. Most were male (69.9%), median (IQR) age was 34.3 (26.6–44.4) years, pre‐ART CD4 count was 203 (55–391) cells/mm^3^ and 39.9% had CD4 <200 cells/mm^3^. ART initiation timing was same‐day in 26.4%, 2–7 days in 13.3%, 8–29 days in 26.4% and ≥30 days in 33.8%. During a median follow‐up of 8.7 (IQR 6.8–12.4) months, HIV‐1RNA ≤1000, <200 and <50 cps/ml was achieved in 96.9%, 95.3% and 84.5% of participants, respectively. VF incidence was 3.6 (95% CI 3.2–4.0) per 100 person‐years (PY). In multivariate analysis, adolescent/young adults (15–24 years) (adjusted sub‐distribution hazard ratio [aSHR] 2.28 [95% CI 1.66–3.12], *p* < 0.001), ages 25–34 (aSHR 1.43 [95% CI 1.07–1.9], *p* = 0.02), CD4 <100 cells/mm^3^ (aSHR 2.11 [95% CI 1.36–3.27], *p* < 0.001) and living in Northern (aSHR 1.64 [95% CI 1.12–2.4], *p* = 0.01) or Southern Thailand (aSHR 1.99 [95% CI 1.3–3.04], *p* < 0.001) were significantly associated with VF.

**Conclusions**: DTG‐based ART achieved excellent viral suppression among ART‐naïve PLWH in a large HIV programmatic in Thailand. Notably, high VL failure were observed among adolescent/young adult, those with lower baseline CD4 cell counts and who lived in Northern/Southern parts of Thailand.

### Co‐morbidities and Co‐infections

### O41 Safety and tolerability of immune checkpoint inhibitors in people with HIV infection and cancer: insights from the national prospective real‐world OncoVIHAC ANRS CO24 cohort study

Raghiatou Baldé^1^, Lambert Assoumou^1^, Christine Katlama^2^, Baptiste Abbar^3^, Pierre Delobel^4^, Thierry Allegre^5^, Armelle Lavole^6^, Alain Makinson^7^, Olivia Zaegel‐Faucher^8^, Laurent Greillier^9^, Cathia Soulie^10^, Marianne Veyri^11^, Mathilde Bertheau^12^, Michèle Algarte Genin^1^, Séverine Gibowski^13^, Anne‐Geneviève Marcelin^10^, Kevin Bihan^14^, Marine Baron^3^, Dominique Costagliola^1^, Olivier Lambotte^15^, Jean‐Philippe Spano ^11^

^1^Sorbonne Université, INSERM, Institut Pierre Louis d'Epidémiologie et de Santé Publique, Paris, France. ^2^Sorbonne Université, INSERM, Institut Pierre Louis d'Epidémiologie et de Santé Publique, AP‐HP, Hôpital Pitié‐Salpêtrière, Service des Maladies Infectieuses, Paris, France. ^3^Sorbonne University, Department of Medical Oncology, AP‐HP, Pitié‐Salpêtrière Hospital, Centre d'Immunologie et des Maladies Infectieuses (CIMI‐Paris), Paris, France. ^4^CHU de Toulouse, Service des Maladies Infectieuses et Tropicales, INSERM, UMR1291, Université Toulouse III Paul Sabatier, Toulouse, France. ^5^Department of Hematology Oncology & Internal Médicine, Centre Hospitalier d'Aix en Provence, Aix‐en‐Provence, France. ^6^GRC#04 Theranoscan, Département de Pneumologie et Oncologie Thoracique, AP‐HP, Hôpital Tenon, Sorbonne Université, Paris, France. ^7^INSERM U1175, Département de Maladies Infectieuses, Centre Hospitalier Universitaire de Montpellier, Montpellier, France. ^8^Aix‐Marseille Université, APHM Sainte‐Marguerite, Service d'Immuno‐Hématologie Clinique, Marseille, France. ^9^Multidisciplinary Oncology and Therapeutic Innovations Department, Aix Marseille University, Assistance Publique‐Hôpitaux de Marseille, Marseille, France. ^10^Sorbonne Université, INSERM, Institut Pierre Louis d'Epidémiologie et de Santé Publique, AP‐HP, Hôpital Pitié‐Salpêtrière, Laboratoire de Virologie, Paris, France. ^11^Sorbonne Université, INSERM, Institut Pierre Louis d'Epidémiologie et de Santé Publique, AP‐HP, Hôpital Pitié‐Salpêtrière, Département d'Oncologie Médicale, Paris, France. ^12^Clinical Research, INSERM‐ANRS‐MIE, Paris, France. ^13^Safety, INSERM‐ANRS‐MIE, Paris, France. ^14^Sorbonne University, INSERM CIC Paris‐Est, AP‐HP, ICAN, Regional Pharmacovigilance Centre, Department of Pharmacology, AP‐HP, Pitié‐Salpêtrière Hospital, Paris, France. ^15^Département d'Immunologie Clinique, AP‐HP, Hôpital Bicêtre, Université Paris‐Saclay, Le Kremlin Bicêtre, France

**Background**: Immune checkpoint inhibitors (ICIs) have been a major advance in cancer management. However, we still lack prospective real‐world data regarding their usage in people with HIV infection (PWH).

**Materials and methods**: The ANRS‐CO24‐OncoVIHAC study (NCT03354936) is an ongoing prospective observational cohort study in France of PWH with cancer treated with ICI. We assessed the incidence of grade ≥3 immune‐related adverse events (irAEs). All grade ≥3 irAEs were reviewed by an Event Review.

**Results**: Between 17 January 2018 and 5 December 2023, 150 participants were recruited from 33 sites and 140 were included in this analysis. At the data cut‐off date of 5 December 2023, median follow‐up was 9.2 months (IQR 3.9–18.3), with a total of 126.2 person‐years. Median age was 59 years (IQR 54–64) and 111 (79.3%) were men. Median time since HIV diagnosis was 25 years (12–31), median duration on ARV was 19.5 years (7.7–25.4) and the CD4 nadir was 117/µl (51–240). ICI regimens comprised anti‐programmed cell death protein 1 (PD1) for 111 (79.3%) participants, anti‐programmed death‐ligand 1 (PDL1) for 25 (17.9%), a combination of anti‐PD1 and anti‐human cytotoxic T‐lymphocyte‐associated protein 4 (CTLA4) for three (2.1%), and anti‐PD1 along with anti‐vascular endothelial growth factor (VEGFR) for one (0.7%). The most frequent cancers were lung (*n* = 65), head/neck (*n* = 15), melanoma (*n* = 12), liver (*n* = 11) and Hodgkin lymphoma (*n* = 9). During follow‐up, a total of 34 grade ≥3 irAEs occurred in 20 participants, leading to an incidence rate of 26.9 per 100 person‐years. The Kaplan‐Meier estimates of the proportion of participants with at least one episode of grade ≥3 irAEs were 13.8% at 6 months, 15.0% at 12 months and 18.7% at 18 months. One treatment‐related death due to myocarditis was reported (0.7%). Multivariable analysis of cumulative incidence showed that participants with time since HIV diagnosis >17 years (incidence rate ratio [IRR] 4.66, *p* = 0.002), with CD4 <200 cells/µl (IRR 4.39, *p* < 0.0001), with positive cytomegalovirus (CMV) serology (IRR 2.76, *p* = 0.034), with history of cancer surgery (IRR 3.44, *p* = 0.001) had a higher risk of incidence of grade ≥3 irAEs.

**Conclusions**: This study showed that the incidence of a first episode of grade ≥3 irAE was 15.0% (95% CI 9.6–22.9) at 1 year and the cumulative incidence of all severe irAE episodes was 26.9 per 100 person‐years. Low CD4 count, positive CMV serology, history of cancer surgery and a longer time since HIV diagnosis were associated with the occurrence of severe irAEs.

### O42 Depression and anxiety symptoms among people with HIV in the UK: prevalence, correlates and treatment. Results from the Positive Voices 2022 study

Fiona Lampe ^1^, Colette Smith^1^, Annegret Pelchen‐Matthews^1^, Adamma Aghaizu^2^, Meaghan Kall^2^, Carole Kelly^2^, Hannah Kitt^2^, Clare Humphreys^2^, Alex Sparrowhawk^3^, Elbushra Herieka^4^, Nicola Fearnley^5^, Helena King^6^, Fumiyo Nakagawa^1^, Janey Sewell^1^, Alison Rodger^1^

^1^Institute for Global Health, University College London, London, UK. ^2^Blood Safety, Hepatitis, STI and HIV Division, UK Health Security Agency, London, UK. ^3^HIV Advice, Support and Information, George House Trust, Manchester, UK. ^4^GUM/HIV, University Hospitals Dorset NHS Foundation Trust, Bournemouth, UK. ^5^Genitourinary Medicine, Bradford Teaching Hospitals NHS Foundation Trust, Bradford, UK. ^6^Department for Public Leadership and Social Enterprise, The Open University, London, UK

**Background**: People with HIV are disproportionately affected by poor mental health. We examined the prevalence and correlates of depression and anxiety symptoms, and the proportion receiving mental health treatment, in a large UK study of people living with HIV.

**Methods**: In “Positive Voices 2022,” 4607 participants, from 101 HIV clinics in England, Wales and Scotland, self‐completed a questionnaire on demographic, socio‐economic and health‐related factors (April 2022–March 2023). Current symptoms of depression and anxiety were defined as PHQ‐9 ≥10 and GAD‐7 ≥10, respectively. Current mental health treatment was defined as receiving prescribed medication or professional therapy for a mental health condition in the past 3 months. Associations of factors with depression and anxiety symptoms, adjusted for core variables, were assessed using modified Poisson regression.

**Results**: Among 4228 participants, prevalence [95% CI] was 20.7% [19.5–21.9%] for depression symptoms; 15.1% [14.0–16.2%] for anxiety symptoms; 23.2% [21.9–24.5%] for either symptom. Patterns of association were similar for depression and anxiety (Table 1): prevalence decreased markedly with older age and was lowest for black African men and women, and highest for “other ethnicity women” and those whose gender was non‐binary/defined in another way. HIV diagnosis in an earlier calendar period, and other major comorbidity were associated with higher prevalence of symptoms. Unemployment, rented or unstable housing, financial hardship and lower social support had exceptionally strong associations with depression and anxiety symptoms, as did experiences of HIV‐related stigma. Strong belief in U═U was associated with lower symptom prevalence. Overall, 1003 (23.7%) participants were currently receiving mental health treatment (16.9% medication, 13.1% professional therapy). 51.7% of those with anxiety or depression symptoms were receiving mental health treatment, versus 15.3% of those without. In total, 1476 (34.9% [33.5–36.4%]) participants had evidence of a current mental health problem, defined as current treatment or depression or anxiety symptoms.

**O42: Table 1:** Association of factors with depression symptoms (PHQ‐9 ≥10) and anxiety symptoms (GAD‐7 ≥10)

	*N*	Depression symptoms %	Adjusted prevalence ratio^a^ (95% CI)	Anxiety symptoms %	Adjusted prevalence ratio^a^ (95% CI)
Age group (years)					
18−34	327	29.4	1	24.8	1
35−44	811	22.6	0.76 (0.61−0.95)	16.4	0.70 (0.54−0.90)
45−54	1254	21.0	0.67 (0.54−0.83)	16.0	0.65 (0.51−0.83)
55−64	1287	20.2	0.60 (0.48−0.75)	13.4	0.50 (0.38−0.65)
65	547	13.4	0.38 (0.29−0.51)	9.5	0.34 (0.24−0.48)
Demographic group					
GBMSM	2519	21.0	1	16.0	1
Black African heterosexual men	249	14.5	0.70 (0.51−0.96)	8.4	0.56 (0.37−0.85)
Other ethnicity heterosexual men	353	19.6	1.05 (0.84−1.31)	10.8	0.77 (0.56−1.05)
Black African women	554	15.0	0.69 (0.55−0.85)	12.6	0.79 (0.62−1.00)
Other ethnicity women	447	27.7	1.32 (1.11−1.55)	19.7	1.23 (1.00−1.51)
Non‐binary gender/in another way^b^	106	33.0	1.57 (1.19−2.07)	18.9	1.14 (0.76−1.70)
Year of HIV diagnosis					
2019−2021	179	25.1	1.16 (0.88−1.53)	21.8	1.34 (0.99−1.82)
2009−2018	1610	19.3	1	14.5	1
1999−2008	1661	20.1	1.22 (1.06−1.41)	13.1	1.07 (0.89−1.28)
1999	686	24.1	1.54 (1.28−1.84)	19.0	1.70 (1.36−2.11)
Major comorbidity^c^					
No	3013	18.1	1	13.5	1
Yes	1215	27.2	1.75 (1.54−1.98)	19.0	1.69 (1.45−1.98)
Employment					
Employed	2713	14.4	1	10.4	1
Unemployed	306	43.1	3.03 (2.59−3.55)	32.0	3.12 (2.56−3.80)
Retired	556	11.9	1.04 (0.79−1.37)	8.5	1.09 (0.76−1.56)
Not working (sickness/disability)	362	62.2	4.42 (3.89−5.03)	45.9	4.72 (3.99−5.57)
Other^d^	126	18.3	1.29 (0.88−1.90)	15.9	1.51 (0.99−2.30)
Housing status					
Homeowner	1849	11.3	1	8.5	1
Renting (private)	1002	22.7	1.94 (1.62−2.31)	15.6	1.67 (1.35−2.08)
Renting (social housing)	929	34.5	3.23 (2.76−3.79)	25.2	3.18 (2.62−3.00)
Other^e^	233	26.2	2.30 (1.79−2.96)	18.9	2.06 (1.52−2.81)
Temporary/homeless	43	55.8	6.21 (4.67−8.26)	46.5	6.65 (4.65−9.51)
Enough money for basic needs					
Yes	2216	11.4	1	7.9	1
Mostly	1056	26.4	2.44 (2.10−2.85)	18.3	2.48 (2.05−3.00)
Sometimes	524	32.1	3.38 (2.84−4.01)	25.2	3.90 (3.16−4.80)
No	273	49.5	5.37 (4.53−6.37)	38.1	6.09 (4.91−7.54)
Social support^f^					
Maximum (as much as desired)	1192	6.6	1	5.0	1
Higher	1317	12.5	1.88 (1.46−2.43)	9.3	1.86 (1.38−2.51)
Medium	667	26.4	3.86 (3.01−4.94)	16.9	3.30 (2.44−4.44)
Low	960	45.2	6.59 (5.26−8.25)	34.4	6.73 (5.17−8.75)
Felt excluded by friends/family due to HIV					
No^g^	3557	16.4	1	11.7	1
Yes, not in the last year	454	34.8	1.97 (1.70−2.28)	24.9	1.96 (1.63−2.36)
Yes, in the last year	217	61.8	3.51 (3.08−4.00)	51.2	4.08 (3.48−4.79)
Belief in U═U					
No	2723	19.3	1	13.8	1
Yes	1505	23.2	1.24 (1.10−1.40)	17.5	1.35 (1.16−1.56)

Abbreviations: GAD‐7, general anxiety disorder‐7; GBMSM, gay, bisexual and other men who have sex with men; PHQ‐9, patient health questionnaire‐9; U═U, undetectable = untransmissible.

^a^ Each factor adjusted for age, demographic group, year of HIV diagnosis only.

^b^ Includes missing gender.

^c^ Ever diagnosed with: cardiovascular disease, diabetes, cancer, kidney disease, liver disease.

^d^ Includes student, carer, other.

^e^ Includes staying with friends/family, sheltered accommodation, care home, other.

^f^ Derived from modified version of Duke Functional Social Support Questionnaire.

^g^ Includes no, don't know and all non‐affirmative responses.

**Conclusions**: There remains a significant burden of depression and anxiety symptoms among people living with HIV. Socio‐economic deprivation, lack of social support and experiences of stigma are among the factors most strongly associated with symptoms. Overall, including symptoms or treatment, about one in three people living with HIV have evidence of a current mental health problem.

### O43 Randomized, multicentre, double‐blind clinical trial designed to evaluate the safety and convenience of switching from dolutegravir/lamivudine to bictegravir/emtricitabine/tenofovir alafenamide in people with HIV, good virological control and neuropsychiatric comorbidities: week 24 results from the GESIDA 11920 ‐ MIND study

Diana Corona Mata^1^, María José Crusells‐Canales^2^, Maria Velasco Arribas^3^, Rafael Mican^4^, Pablo Ryan^5^, Marina Gallo^6^, Juan Tiraboschi^7^, Noemi Cabello Clotet^8^, Marta Molero^9^, Francisca Artigues Serra^10^, Alvaro Mena^11^, Sara de la Fuente Moral^12^, Javier Perez^13^, Mariana Diaz Almidon^14^, Pedro Gil^15^, Ignacio Perez Valero ^16^

^1^Enfermedades Infecciosas, Hospital Universitario Reina Sofia, Cordoba, Spain. ^2^Medicina Interna, Hospital Clínico Universitario Lozano Bless, Zaragoza, Spain. ^3^Enfermedades Infecciosas, Hospital Universitario Fundación Alcorcón, Alcorcon, Spain. ^4^Medicina Interna, Hospital Universitario La Paz, Madrid, Spain. ^5^Medicina Interna, Hospital Universitario Infanta Leonor, Madrid, Spain. ^6^Enfermedades Infecciosas, Hospital Universitario Virgen Macarena, Sevilla, Spain. ^7^Enfermedades Infecciosas, Hospital Universitari de Bellvitge, L'Hospitalet de Llobregat, Spain. ^8^Enfermedades Infecciosas, Hospital Clínico, Madrid, Spain. ^9^Hospital Son Llatzer, Bonilla, Palma, Spain. ^10^Medicina Interna, Hospital Universitario Son Espases, Palma de Mallorca, Spain. ^11^Medicina Interna, Hospital Universitario de A Coruña, A Coruña, Spain. ^12^Medicina Interna, Hospital Puerta de Hierro, Majadahonda, Spain. ^13^Stachowski, Hospital Costa de Sol, Marbella, Spain. ^14^Bioestadistica, Hospital Universitario La Paz, Madrid, Spain. ^15^Clinical Research Operations, Fundación SEIMC‐GESIDA, Madrid, Spain. ^16^Enfermedades Infecciosas, Hospital Universitario Reina Sofia, IMIBIC, CIBERINFEC, Cordoba, Spain

**Background**: People with HIV (PWH) and pre‐existing neuropsychiatric comorbidities (NPCs) have been systematically excluded from dolutegravir/lamivudine (DTG/3TC) clinical trials. Therefore, we do not know the neuropsychiatric safety profile of DTG/3TC in this population.

**Materials and methods**: The purpose of the MIND study, a double‐blind, multicentre, randomized clinical trial, was to compare the neuropsychiatric safety of continuing on DTG/3TC versus switching to bictegravir/emtricitabine/tenofovir alafenamide (BIC/FTC/TAF) in PWH with undetectable viral loads (HIV‐RNA <50 copies/ml) and pre‐existing NPCs. We compared the proportion of discontinuations and neuropsychiatric adverse events (AEs) between groups, as well as self‐reported changes in mood (Hospital Anxiety and Depression Scale; HADS), sleep (Pittsburgh Sleep Quality Index; PSQI), HIV‐related symptoms (HIV Symptom Index; HIV‐SI), quality of life (Medical Outcome Study‐HIV Health Survey; MOS‐HIV) and treatment satisfaction (Escala de Satisfacción con el Tratamiento Antirretroviral; ESTAR) after 24 (current analysis) and 48 weeks of follow‐up. Generalized estimating equations and linear general models were used to evaluate the treatment effect on outcome variables.

**Results**: The study was fully recruited, with 39 participants continuing on DTG/3TC (arm 1) and 41 switching to BIC/FTC/TAF (arm 2). Baseline characteristics were similar between arms. At week 24, five participants discontinued treatment in DTG/3TC and BIC/FTC/TAF arms (average: 12.8% [4.8–30.1] vs. 12.2% [4.6–28.8]). Neuropsychiatric AEs leading to ART discontinuation (3 vs. 2) and grade 2–4 neuropsychiatric AEs (17.9% vs. 14.5%) were also similar between groups. The most common AEs with DTG/3TC were headache (15.4%), insomnia (7.7%) and anxiety/depression (both 5.1%), and with BIC/FTC/TAF, they were anxiety (14.6%), depression (7.3%) and insomnia (7.3%). Suicidality was documented in one participant on DTG/3TC (2.6%) and in two participants on BIC/FTC/TAF (4.9%) (*p* = 0.59). Changes in the HADS, PSQI, HIV‐SI and MOS‐HIV questionnaires were also similar between groups (Figure 1). Participants who switched to BIC/FTC/TAF experienced improvements in gastrointestinal tolerability (nausea: 3.9% vs. 23.2%, *p* = 0.03; abdominal discomfort: 16.8% vs. 40.2%, *p* = 0.02) and lipid profile (change in total cholesterol: −11.1 vs. 5.4 mg/dl; LDL: −13.1 vs. 4.2 mg/dl).

**O43: Figure 1:**
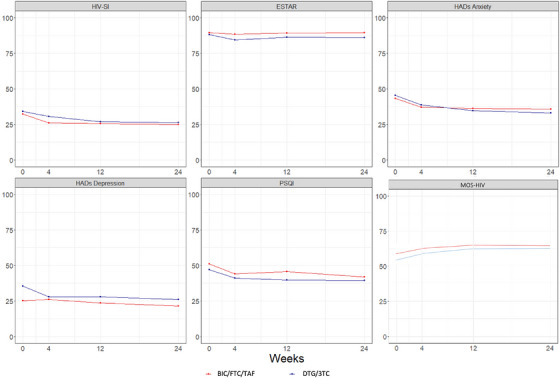
Evolution of normalized scores of self‐reported questionnaires (HIV‐SI, HADs, PSQI, ESTAR and MOS‐HIV) up to week 24.

**Conclusions**: In PWH with pre‐existing NPCs, switching from DTG/3TC to BIC/FTC/TAF did not show short‐term neuropsychiatric safety benefits. However, improvements in gastrointestinal tolerability and lipid profile may justify the switch in PWH experiencing gastrointestinal intolerance and/or dyslipidaemia.

### O44 A phase III randomized study to evaluate the safety, tolerability and immunogenicity of V116, an adult‐specific pneumococcal conjugate vaccine (PCV), followed by PCV15, in adults living with HIV (STRIDE‐7)

Jayani Pathirana^1^, Moti Ramgopal ^2^, Charlotte Martin^3^, Johannes J. Lombaard^4^, Carolina Chahin^5^, Odile Launay^6^, Winai Ratanasuwan^7^, David Greenberg^8^, Carlos G. Grijalva^9^, Walter A. Orenstein^10^, Angelika Shenkerman^11^, Doreen Fernsler^11^, Yeonil Kim^11^, Jianing Li^11^, Heather Loryn Platt^11^

^1^Global Clinical Development, MSD Zurich, Zurich, Switzerland. ^2^Infectious Diseases, Midway Immunology and Research Center, Fort Pierce, FL, USA. ^3^Infectious Diseases, CHU Saint‐Pierre, Brussels, Belgium. ^4^Clinical Development, Josha Research, Bloemfontein, South Africa. ^5^Specialty Care, Hospital Hernán Henriquez Aravena de Temuco‐Unidad de Investigación Clínica, Araucania, Chile. ^6^Department of Vaccinology, Université Paris Cité, Assistance Publique Hôpitaux de Paris, Hôpital Cochin, Inserm, F‐CRIN, CIC Cochin Pasteur, Paris, France. ^7^Department of Preventive and Social Medicine, Faculty of Medicine, Siriraj Hospital, Mahidol University, Bangkok, Thailand. ^8^Pediatric Infectious Disease Unit, Soroka University Medical Center, Beer‐Sheva, Israel. ^9^Health Policy, Vanderbilt University Medical Center, Nashville, TN, USA. ^10^Global Health, OrensteinVax, Atlanta, GA, USA. ^11^MRL, Merck & Co., Rahway, NJ, USA.

**Background: **Adults living with HIV are at greater risk of pneumococcal disease (PD). V116 is a 21‐valent adult‐specific PCV containing the most prevalent serotypes associated with PD in adults in regions with established paediatric vaccination programmes. This phase III (NCT05393037), double‐blind study evaluated the safety, tolerability and immunogenicity of V116 in adults living with HIV.

**Materials and methods**: In part A, adults with HIV (CD4^+^ cell count ≥50 cells/µL, plasma HIV RNA <50,000 copies/mL, on antiretroviral therapy ≥6 weeks) were randomised to receive either V116 followed by placebo, or a 15‐valent PCV followed by 23‐valent polysaccharide pneumococcal vaccine (PCV15 + PPSV23), with an 8‐week interval between doses. In part B, participants who received V116 in part A, received PCV15 open‐label approximately 10–18 months later. Opsonophagocytic activity geometric mean titres (OPA GMTs) and immunoglobulin G geometric mean concentrations (IgG GMCs) were evaluated 30 days post‐vaccination in parts A and B. Safety was evaluated as the proportion of participants with adverse events (AEs).

**Results**: Of 313 participants enrolled, 305 (97.1%) completed part A. V116 was immunogenic, as assessed by OPA GMTs and IgG GMCs, at 30 days post‐vaccination for all 21 vaccine serotypes. V116 elicited comparable immune responses to PCV15 + PPSV23 for the 13 common serotypes and higher immune responses for the 8 unique serotypes (Figure 1). Proportions of participants with AEs were lower in the V116 + placebo group (71.6%), primarily due to fewer injection‐site AEs, compared with the PCV15 + PPSV23 group (91.0%). Among 126 participants who received PCV15 in part B, at 30 days post vaccination, PCV15 was immunogenic for the 9 serotypes in PCV15 but not V116 and the 6 common serotypes to V116 and PCV15 (Figure 2). OPA GMTs to the 15 serotypes unique to V116 remained high at 30 days post vaccination. The proportion of participants with AEs post‐PCV15 (69.8%) was consistent with the established safety profile of PCV15. No vaccine‐related serious AEs or deaths occurred in part A or part B.

**O44: Figure 1:**
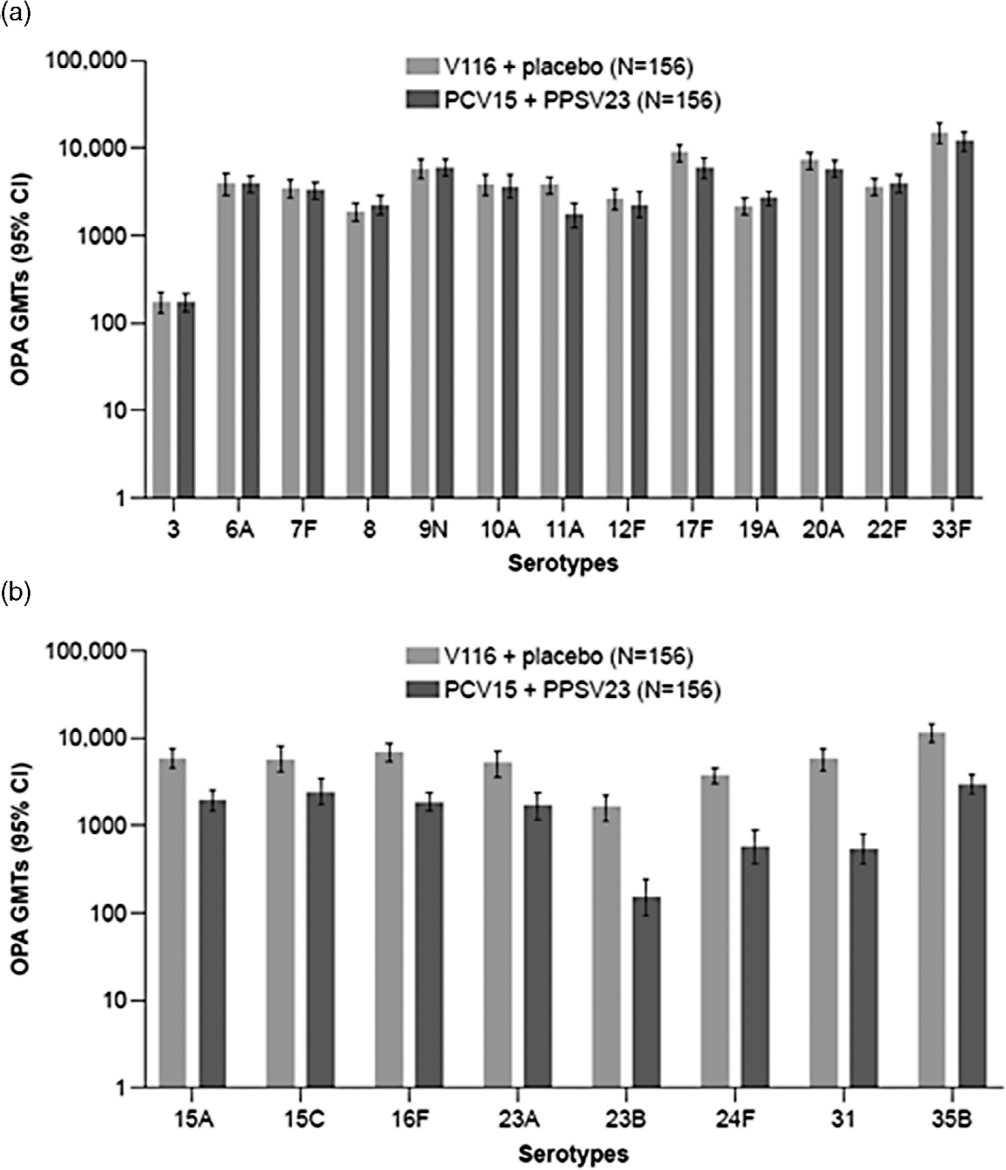
Part A: OPA GMTs for a) 13 common serotypes between V116 and PCV15 + PPSV23 and b) the eight unique serotypes in V116, at 30 days post‐vaccination (per‐protocol population). Serotype 15C represents the immune response to the deOAc15B polysaccharide, as the molecular structures for deOAc15B and 15C are similar; anti‐15C immune responses are assessed in this study. Bars show serotype‐specific OPA GMTs with 95% CIs for each serotype. The within‐group 95% CIs are obtained by exponentiating the CIs of the mean of the natural log values based on the *t*‐distribution. CI, confidence interval; GMC, geometric mean concentration; GMT, geometric mean titre; IgG, immunoglobulin G; *N*, number of participants; OPA, opsonophagocytic activity; PCV15, 15‐valent pneumococcal conjugate vaccine; V116, an investigational adult‐specific pneumococcal conjugate vaccine.

**Conclusions**: In adults living with HIV, V116 was well tolerated and induced comparable immune responses to PCV15 + PPSV23 for common serotypes and higher responses for V116‐unique serotypes.

### O45 Prevalence trends of active HCV infection among people with HIV in Spain (2002−2023): nearing elimination

Juan Berenguer ^1^, María M. Arcos^2^, Chiara Fanciulli^1^, María J. Vivancos^3^, Pere Domingo^4^, Asunción Hernando^5^, María N. Sánz‐Pérez^6^, Pablo Ryan^7^, Jordi Navarro^8^, Rosario Palacios^9^, Luis E. Morano^10^, José A. Iribarren^11^, Rosa Martínez^12^, María J. Galindo Puerto^13^, Ignacio Santos^14^, Ian López‐Cruz^15^, Antonio Rivero Roman^16^, Livia Giner^17^, Carmen Fariñas^18^, Coral García^19^, Marta Montero^20^, Oscar L. Ferrero^21^, Aroa Villoslada^22^, Josefa F. Soler‐González^23^, José Sanz^24^, Sergio Rodríguez^25^, Juan E. Losa^26^, Enrique Bernal^27^, Sergio Veloso^28^, Laura Pérez‐Martínez^29^, Fernando Mateos^30^, Laia Arbonés^31^, Raquel Franch^32^, Diana Corps^33^, Cristina Martín^34^, Gerardo Alonso^35^, Marta Clavero Olmos^36^, Rafael Silvariño^37^, Ramón Teira^38^, Olga Belinchon^39^, Marta De Miguel^40^, Inmaculada Jarrín^41^, Juan González‐García^2^

^1^Infectious Diseases, Hospital Gregorio Marañón, Madrid, Spain. ^2^HIV Unit/Internal Medicine, Hospital La Paz, Madrid, Spain. ^3^Infectious Diseases, Hospital Ramón y Cajal, Madrid, Spain. ^4^Infectious Diseases, Hospital Santa Creu i Sant Pau, Barcelona, Spain. ^5^HIV Unit, Hospital 12 Octubre, Madrid, Spain. ^6^Infectious Diseases/Internal Medicine, Hospital Clínico San Carlos, Madrid, Spain. ^7^Infectious Diseases/Internal Medicine, Hospital Infanta Leonor, Madrid, Spain. ^8^Infectious Diseases, Hospital Vall d'Hebrón, Barcelona, Spain. ^9^Infectious Diseases, Hospital Virgen de la Victoria, Málaga, Spain. ^10^Infectious Diseases/Internal Medicine, Hospital Alvaro Cunqueiro, Vigo, Spain. ^11^Infectious Diseases, Hospital Donostia, San Sebastián, Spain. ^12^Infectious Diseases/Internal Medicine, Hospital Miguel Servet, Zaragoza, Spain. ^13^Infectious Diseases/Internal Medicine, Hospital Clínico de Valencia, Valencia, Spain. ^14^Infectious Diseases, Hospital de la Princesa, Madrid, Spain. ^15^Infectious Diseases/Internal Medicine, Hospital Doctor Peset, Valencia, Spain. ^16^Infectious Diseases, Hospital Reina Sofía de Córdoba, Córdoba, Spain. ^17^Infectious Diseases, Hospital General de Alicante, Alicante, Spain. ^18^Infectious Diseases, Hospital Marqués de Valdecilla, Santander, Spain. ^19^Infectious Diseases, Hospital Virgen de las Nieves, Granada, Spain. ^20^Infectious Diseases, Hospital La Fe, Valencia, Spain. ^21^Infectious Diseases, Hospital de Basurto, Bilbao, Spain. ^22^Infectious Diseases/Internal Medicine, Hospital Son Llátzer, Palma de Mallorca, Spain. ^23^Infectious Diseases/Internal Medicine, Hospital de Cabueñes, Gijón, Spain. ^24^Infectious Diseases/Internal Medicine, Hospital Principe de Asturias, Alcalá de Henares, Spain. ^25^Infectious Diseases/Internal Medicine, Hospital de Getafe, Getafe, Spain. ^26^Infectious Diseases, Hospital Fundación Alcorcón, Alcorcón, Spain. ^27^Infectious Diseases, Hospital Reina Sofía de Murcia, Murcia, Spain. ^28^Infectious Diseases/Internal Medicine, Hospital Joan XXIII, Tarragona, Spain. ^29^Infectious Diseases/Internal Medicine, Hospital de la Rioja, Logroño, Spain. ^30^Infectious Diseases, Hospital General de Albacete, Albacete, Spain. ^31^Infectious Diseases/Internal Medicine, Hospital de Mataró, Mataró, Spain. ^32^Infectious Diseases/Internal Medicine, Hospital Virgen de la Cinta, Tortosa, Spain. ^33^Infectious Diseases/Internal Medicine, Hospital de Torrejón, Torrejón de Ardoz, Spain. ^34^Infectious Diseases/Internal Medicine, Hospital Virgen de la Concha, Zamora, Spain. ^35^Infectious Diseases/Internal Medicine, Hospital Rafael Méndez, Lorca, Spain. ^36^Infectious Diseases/Internal Medicine, Hospital Infanta Elena, Valdemoro, Spain. ^37^Infectious Diseases/Internal Medicine, Hospital San Eloy, Baracaldo, Spain. ^38^Infectious Diseases/Internal Medicine, Hospital de Sierrallana, Torrelavega, Spain. ^39^Infectious Diseases/Internal Medicine, Hospital Virgen de la Luz, Cuenca, Spain. ^40^Clinical Investigation, Fundación SEIMC/GESIDA, Madrid, Spain. ^41^Centro Nacional de Epidemiología, Instituto de Salud Carlos III, Madrid, Spain

**Background**: Hepatitis C virus (HCV) historically posed a severe health burden for people with HIV (PWH) in Spain. Over the past two decades, shifts in HIV acquisition modes, implementation of harm reduction programmes for people who inject drugs (PWID) and the introduction of all‐oral direct‐acting antiviral (DAA) therapies for HCV have significantly influenced the epidemiology of HIV/HCV coinfection. This study reports the prevalence of HCV in PWH in Spain in 2023 and compares the results with eight similar studies conducted since 2002.

**Materials and methods**: The primary objective of all studies was to determine the prevalence of both anti‐HCV antibodies and active HCV infection (HCV‐RNA+) among PWH. The target population included PWH in active follow‐up at participating centres (defined as having at least one outpatient visit or hospitalization in the previous 12 months). Sample size estimation accuracies varied from 3% in the first 2 years to less than 1% in the last 2 years. Patients were selected through random sampling with proportional allocation.

**Results**: Table 1 summarizes study metrics and patient characteristics across all nine prevalence studies. Significant trends include changes in the mechanisms of HIV acquisition among participants, with a decrease in PWID and an increase in men who have sex with men (MSM). HCV seroprevalence among PWH decreased from 60.8% in 2002 to 27.4% in 2023. The prevalence of active HCV infection decreased from 54.0% in 2002 to 0.9% in both 2021 and 2023. Anti‐HCV treatment uptake increased from 23.0% in 2002 to 48.0% in 2009 (with pegylated IFN + RBV), and following the introduction of all‐oral DAAs, saw a sharp increase from 59.3% in 2015 to 99.0% in 2023 (Figure 1).

**O45: Table 1:** Participating centres, reference population, sample size and baseline characteristics of participants in the nine prevalence studies of HCV among PWH in Spain

	2002	2009	2015	2016	2017	2018	2019	2021	2023
Sites, *n*	39	43	41	43	43	43	41	41	39
Reference population, *n*	31,800	36,450	35,791	38,904	40,322	40,650	41,973	46,059	47,006
Sample size, *n*	1260	1458	1867	1588	1690	1733	1325	1421	1431
Male sex, %	72	73	76	77	75	74	75	75	79
Age years, mean (SD)	40 (8)	45 (10)	47 (10)	49 (11)	49 (11)	49 (11	49 (12)	50 (12)	51 (12)
HIV transmission category, %									
PWID	55	44	31	30	30	29	26	24	21
MSM	17	24	35	35	34	36	40	43	46
Other/unknown	28	32	34	35	36	35	34	33	33
Anti‐HCV treatment uptake, %	23.0	48.0	59.3	74.7	82.4	92.2	95.0	97.9	99.0
Prevalence of HCV antibodies, %	60.8	50.4	37.7	34.6	34.0	33.6	28.6	28.4	27.4
Prevalence of active HCV (RNA+), %	54.0	34.0	22.0	11.8	8.0	3.7	2.2	0.9	0.9

**O45: Figure 1:**
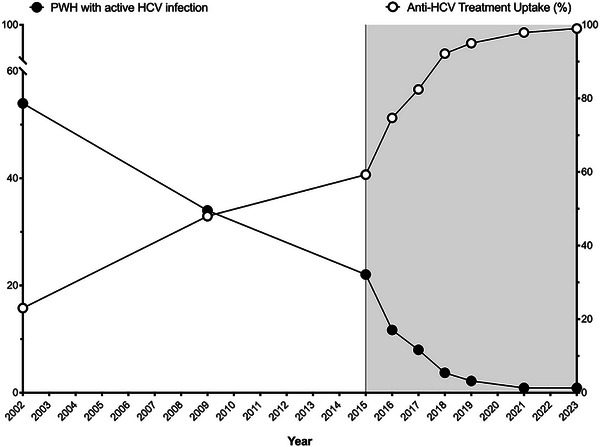
Prevalence of active HCV infection and anti‐HCV treatment uptake in the nine prevalence studies of HCV among PWH in Spain. The shaded area indicates the years during which DAAs were available for treatment.

**Conclusions**: Over the past two decades, HCV infection among PWH in Spain has sharply declined, now representing a marginal issue within this population. The introduction of all‐oral DAAs has been crucial in reducing active HCV infection prevalences to less than 1% since 2021. Despite this progress, the absence of an HCV vaccine and continued high‐risk transmission practices highlight the need for ongoing awareness, testing, characterization of new infections and transmission networks, and prevention efforts.

### O46A Long‐term outcomes following liver transplantation (LT) in patients with HIV (PWH): a retrospective single‐centre case‐control study

Sheila Blanco^1^, Lucia Serrano^2^, Pablo Ruiz^3^, Daniela Malano Barletta^1^, Montserrat Laguno^4^, Alejandro Forner^5^, Christian Manzardo^1^, Gonzalo Crespo^5^, Hugo Lopez^6^, Anna Lligoña^6^, Josep Fuster^7^, Constantino Fondevila^7^, Juan C. Garcia‐Valdecasas^7^, Montserrat Tuset^8^, Asuncion Moreno^1^, Antonio Rimola^5^, Jose M. Miró ^4^

^1^Infectious Diseases Service—HIV/AIDS Unit, Hospital Clinic‐IDIBAPS‐University of Barcelona, Barcelona, Spain. ^2^Statistics Department, Fundación SEIMC‐GESIDA, Madrid, Spain. ^3^Hepatology Service—Liver Transplant Unit, Hospital Clinic‐IDIBAPS‐University of Barcelona, Barcelona, Spain. ^4^Infectious Diseases Service—HIV/AIDS Unit, Hospital Clinic‐IDIBAPS‐University of Barcelona‐CIBERINFEC, Barcelona, Spain. ^5^Hepatology Service—Liver Transplant Unit, Hospital Clinic‐IDIBAPS‐University of Barcelona‐CIBEREHD, Barcelona, Spain. ^6^Psychiatry Service, Hospital Clinic‐IDIBAPS‐University of Barcelona, Barcelona, Spain. ^7^Hepatopancreatobiliary Surgery and Transplantation, General and Digestive Surgery Service, Hospital Clinic‐IDIBAPS‐University of Barcelona, Barcelona, Spain. ^8^Pharmacy Department, Hospital Clinic‐IDIBAPS‐University of Barcelona, Barcelona, Spain

**Background**: End‐stage liver disease and hepatocellular carcinoma has been common in PWH co‐infected with HCV and/or HBV, sometimes requiring LT. To date, LT in PWH has presented short/mid‐term outcomes (patient/graft survival) comparable to those of the general population, but data on the long‐term outcomes is scarce and no studies have been performed in Europe. We aimed to compare 15‐ to 20‐year LT outcomes between HIV‐positive/HIV‐negative recipients and to evaluate comorbidity prevalence in survivors.

**Methods**: This single‐centre, retrospective, case‐control study included 96 patients (24 HIV positive and 72 matched HIV negative) transplanted between 2003 and 2012 and followed until April 2024. HIV‐positive recipients were matched with HIV‐negative recipients (1:3 ratio) by calendar year (±1 year), age (±12 years), gender, presence of HCV/HBV co‐infection and presence of hepatocellular carcinoma (HCC). A descriptive analysis was performed. Survival time from LT was estimated using the Kaplan‐Meier product‐limit method; the curves obtained in HIV‐positive and HIV‐negative recipients were compared using the generalized log‐rank test (univariate Cox model analysis).

**Results**: Ninety‐two recipients (96%) had HCV infection at the time of LT. At the end of follow‐up, 49 patients (51%) remained alive with no differences between HIV‐positive and HIV‐negative groups (54.4 vs. 50%, *p* = 0.988). Median (range) follow‐up was 12.7 years (5.5–15.2). Few patients reached 20 years of follow‐up. HIV‐positive patient and graft survival rates (95% confidence interval) at 15 years were 65.2% (45.8–84.7) and 56.5% (36.3–76.8), respectively, compared to 57.5% (45.3–69.7) and 54.5% (42.4–66.8) for HIV‐negative recipients (*p*>0.05) (Figure 1a, b). The overall main cause of death was liver disease, mostly before the anti‐HCV direct acting antivirals era. The development of comorbidities and AIDS and non‐AIDS‐related events and cancer were similar in both groups, except chronic kidney disease which was more frequent in HIV positive (*p* = 0.047) and diabetes mellitus which was more frequent in HIV‐negative recipients (*p* = 0.042). Immuno‐virological evolution of HIV‐positive patients was favourable with integrase strand‐transfer inhibitors (InSTI)‐based antiretroviral therapy being the most common antiretroviral therapy in recent years.

**O46A: Figure 1:**
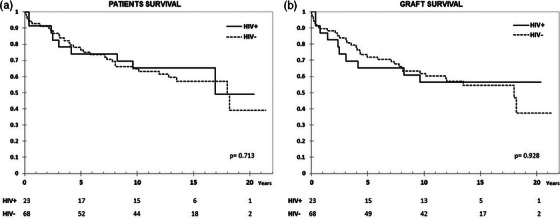
Probability of patient and graft survival in HIV‐positive (solid line) and HIV‐negative (dotted line) LT recipients at 15–20 years.

**Conclusions**: Long‐term outcomes (survival rates and prevalence of comorbidities) between HIV‐positive and HIV‐negative recipients were similar. These results are encouraging and support LT in PWH when clinically indicated.

### O46B HIV‐positive donor to positive recipient kidney transplantation: a nationwide survey

Nerea L. Amondarain^1^, Lucia Serrano^2^, David Paredes^3^, Beatriz Mahillo^4^, Gloria de la Rosa^4^, Daniela Malano Barletta^1^, Christian Manzardo^1^, Frederic Cofan^5^, Angela Gonzalez^5^, David Cucchiari^5^, Mireia Musquera^5^, Elisa de Lazzari^1^, Antonio Rimola^6^, Asuncion Moreno^1^, Jose M. Miró ^1^

^1^Infectious Diseases Service—HIV/AIDS Unit, Hospital Clinic‐Institut D'investigacions Biomèdiques August Pi I Sunyer (IDIBAPS)‐University of Barcelona, Barcelona, Spain. ^2^Statistical Department, Fundación Sociedad Española de Enfermedades Infecciosas y Microbiología Clínica (SEIMC)‐Grupo de Estudio del Sida (GeSIDA), Madrid, Spain. ^3^Transplant Coordination Department, Hospital Clinic‐IDIBAPS‐University of Barcelona, Barcelona, Spain. ^4^Transplant Coordination Department, Organización Nacional de Trasplantes (ONT), Madrid, Spain. ^5^Department of Nephrology and Urology, Hospital Clinic‐IDIBAPS‐University of Barcelona, Barcelona, Spain. ^6^Department of Hepatology and Liver Transplantation, Hospital Clinic‐IDIBAPS‐University of Barcelona, Barcelona, Spain

**Background**: HIV‐positive donor kidneys can now be transplanted into HIV‐positive recipients (HIV D+/R+) with end‐stage organ disease in some countries (South Africa [1], USA (HOPE) [2], Europe). Despite Spain having the highest donor rate, this practice has been prohibited by law since 1987 [3]. This survey canvassed Spanish kidney transplant (KT) teams on this strategy and their attitude towards HIV D+/R+ KT.

**Materials and methods**: The survey was conducted in 2019 and was sent to the four members of all KT teams (specialists in HIV/infectious diseases [HIV/ID], nephrology [NEPH], urology [URO] and transplant coordination [TC]) in the 39 Spanish KT centres. They answered standardized questions via an electronic questionnaire (REDcap software), using a 0−10 analogue scale (0 = fully disagree, 10 = fully agree). The responses were correlated according to the specialty of the KT team from each centre.

**Results**: At least one member of the 39 KT teams (100%) answered the questionnaire. The rate of specialists responding was 99/156 (64%). The rates of response by specialty were: HIV/ID 17 (44%), NEPH 33 (85%), URO 16 (49%) and TC 33 (85%). Mean (SD) age of specialists was 52.6 (8.7) years and 64 were males (65%). Mean (SD) time working in transplantation/donation was 18.2 (10) years. The main results of the survey are depicted in Table 1. Respondents agreed on using organs from various donor groups, including VS on ART donors (both deceased and living), high‐risk donors and serodifferent couples. However, urologists were least likely to agree on using organs from VS deceased donors on ART (*p* < 0.05) or HIV‐high risk deceased donors (*p* < 0.05). Participants were reluctant to use organs from non‐VS donors, whether they were not on ART or diagnosed with HIV during transplant assessment. There was unanimous support for a specialized consent form for HIV‐positive recipients and willingness to participate in an HIV D+/R+ trial.

**O46B: Table 1:** Survey questions and responses categorized by specialty

		Overall (*N* = 99)	HIV/ID (*N* = 17)	NEPH (*N* = 33)	URO (*N* = 16)	TC (*N* = 33)	*p*‐value
Deceased donor	VS on ART	9 (8−10)^a^	10 (9−10)	9 (7−10)	8 (7−10)	10 (8−10)	0.047
Deceased donor	No VS off ART	2 (0−5)	1 (0−7)	2 (0−5)	1 (0−4)	3 (1−8)	0.180
Deceased donor	HIV diagnosis at transplant evaluation	2 (0−5)	1 (0−5)	2 (0−5)	1 (0−5)	4 (1−8)	0.104
Deceased donor	High‐risk donor, HIV negative	9 (7−10)	9 (8−10)	8 (6−9)	7 (2−10)	10 (8−10)	0.008
Deceased donor	Serodifferent HIV donor but HIV‐positive couple	9 (6−10)	9 (8−10)	8 (6−9)	7.5 (5−10)	10 (8−10)	0.099
Living donor	VS on ART	9 (6−10)	9 (8−10)	8 (5−10)	8 (4.5−10)	10 (8−10)	0.154
Use of specific consent form for HIV‐positive receptors		10 (9−10)	10 (9−10)	10 (8−10)	8 (5−10)	10 (9−10)	0.058
Willingness to participate in an HIV D+/R+ trial, *n* (%)		84 (85%)	16 (94%)	26 (78%)	12 (75%)	30 (91%)	0.238

*Note*: 0 = fully disagree; 10 = fully agree. Kruskal‐Wallis test (for *p*‐values from the first to the penultimate row) and Fischer's exact test (for the *p*‐value of the last row) were used.

Abbreviations: ART, antiretroviral therapy; HIV/ID, HIV/infectious diseases; KT, kidney transplant; NEPH, nephrology; TC, transplant coordination team; URO, urology; VS, HIV virological suppression.

^a^ Median (IQR).

**Conclusions**: Most Spanish KT team specialists would use kidneys from virologically suppressed HIV‐positive deceased or living donors for HIV‐positive recipients with indications for KT. The results of this survey could lead to a change in Spain's donor laws.

**References**

1. Muller E, Kahn D, Mendelson M. Renal transplantation between HIV‐positive donors and recipients. N Engl J Med. 2010;362:2336‐7.

2. Durand CM, Zhang W, Brown DM, Yu S, Desai N, Redd AD, et al. A prospective multicenter pilot study of HIV‐positive deceased donor to HIV‐positive recipient kidney transplantation: HOPE in action. Am J Transplant. 2021;21:1754‐64.

3. Ministerio de Sanidad y Consumo. Orden de 24 de junio de 1987 sobre pruebas de detección anti‐VIH, en materia de obtención, extracción, trasplante, injerto o implantación de órganos humanos [Internet]. 1987. [cited 2024 Jul 3] Available from: https://www.boe.es/eli/es/o/1987/06/24/(2).

### PrEP‐ing for the Future

### O47 Differences in oral PrEP use patterns and intention to use long‐acting regimens among MSM between formal and informal PrEP provision pathways in 20 European countries: a latent class analysis

Haoyi Wang ^1^, Alejandro Adriaque Lozano^1^, Hanne Zimmermann^1^, Johann Kolstee^1^, Melanie Schroeder^2^, Ama Appiah^3^, Ana Milinkovic^4^, Supriya Sarkar^2^, Kai Jonas^1^

^1^Department of Work and Social Psychology, Maastricht University, Maastricht, Netherlands. ^2^Global Health Outcomes, ViiV Healthcare, Brentford, UK. ^3^Patient Engagement, ViiV Healthcare, Brentford, UK. ^4^Global Medical, ViiV Healthcare & Chelsea and Westminster Hospital, Brentford, London, UK

**Background**: Different PrEP provision pathways (PPPs) exist across Europe. Formal PPPs include sexual health clinics/centres, medical specialists or GPs. In countries where PrEP is not/less implemented, informal PPPs, such as community‐based access, are more prevalent to facilitate PrEP use. With the recent authorization of long‐acting (LA) PrEP, the complexity of current PPPs is expected to increase. Some concerns accessing oral PrEP via informal PPPs may lead to less optimal use patterns, particularly regarding adherence and discontinuation, which can significantly reduce PrEP's effectiveness. We aimed to investigate the differences in PPP access, variations in PrEP use patterns and whether MSM's intention to use LA‐PrEP is determined by PPPs.

**Methods**: A cross‐sectional survey (PROTECT) conducted in 20 European countries between October 2023 and April 2024, included 7505 MSM with current/former PrEP use. First, a latent class analysis (LCA) was performed to identify individuals’ latent socio‐economic positions (latent‐SEPs), and their likelihood of accessing different PPPs. Next, logistic regression was used to compare PrEP adherence and discontinuation patterns, and linear regression to compare their intentions to use LA‐PrEP between formal/informal PPPs, adjusting for individuals’ latent‐SEPs.

**Results**: Of the samples, 6671 (88.9%) accessed PrEP via formal PPPs, and 834 (11.1%) accessed via informal PPPs. The LCA identified two latent‐SEP groups: one group was younger, less educated, more unemployed, with lower income and more likely to be migrants. This less‐advantaged group showed a significantly higher likelihood of accessing PrEP via informal PPPs (OR 1.22, 95% CI 1.01–1.49). Compared with formal PPPs, MSM who accessed PrEP via informal PPPs showed higher likelihoods of suboptimal adherence (OR 1.29, 95% CI 1.04–1.58), and discontinuation (OR 3.57, 95% CI 3.02–4.22), with their latent‐SEP playing a significant role. They also showed a higher intention to use LA‐PrEP (B 0.18, 95% CI 0.10–0.26, Figure 1).

**O47: Figure 1:**
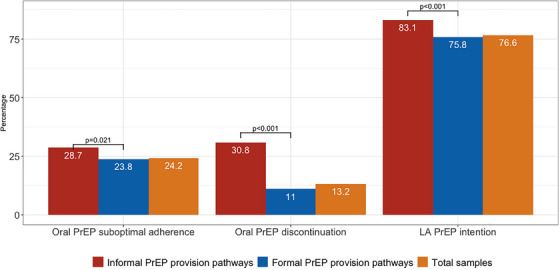
Oral PrEP use patterns and long‐acting PrEP use intention between MSM accessing PrEP via formal and informal PrEP provision pathways in 20 European countries. *Note*: Differences between different PrEP provision pathways were tested by a chi‐square test. LA PrEP intention was measured on a 1–5 Likert scale, and shown in high (extreme/somehow intention) and low (neutral/somehow/extreme non‐intention).

**Conclusions**: Differences exist in PrEP access among MSM in Europe, and the informal PPPs offer opportunities for MSM with less‐advantaged SEPs to benefit from PrEP. However, less optimal oral PrEP adherence and retention were found among these users. Tailored efforts should ensure equal accessibility and affordability across different PPPs to enhance the overall PrEP use. LA‐PrEP could reduce these PrEP use disparities if implemented equally across different PPPs.

### O48 Persistence in use of twice‐yearly lenacapavir versus daily oral PrEP in the PURPOSE 1 phase 3 trial

Linda‐Gail Bekker ^1^, Noah Kiwanuka^2^, Pearl Selepe^3^, Amy Ward^4^, Ramin Ebrahimi^5^, Yang Zhao^5^, Christoph Carter^5^, Alexander Kintu^5^, Jenna Yager^5^, Moupali Das^5^, Quarraisha Abdool Karim^6^, for the PURPOSE 1 Study Team

^1^The Desmond Tutu HIV Centre, University of Cape Town, Cape Town, South Africa. ^2^Department of Epidemiology and Biostatistics, Makerere University School of Public Health, Kampala, Uganda. ^3^Clinical Research Division, The Aurum Institute – Klerksdorp Clinical Research Site, Klerksdorp, South Africa. ^4^Department of Medicine, Vuka Research Clinic, University of Cape Town, Cape Town, South Africa. ^5^Gilead Sciences Inc., Foster City, CA, USA. ^6^Department of Epidemiology and Prevention, Centre for the AIDS Programme of Research in South Africa, Durban, South Africa, and Department of Epidemiology, Mailman School of Public Health, New York, NY, USA

**Background**: In PURPOSE 1, a phase 3, randomised, blinded study, twice‐yearly subcutaneous (SC) lenacapavir (LEN) was safe and 100% efficacious compared with background HIV incidence (bHIV), and superior to emtricitabine/tenofovir disoproxil fumarate (F/TDF) for pre‐exposure prophylaxis (PrEP), whereas HIV incidence in the emtricitabine/tenofovir alafenamide (F/TAF) and F/TDF groups was similar to bHIV. In this sub‐analysis, we characterise annual persistence, defined as consistent adherence over 1 year, to twice‐yearly SC injections or daily oral PrEP in PURPOSE 1.

**Materials and methods**: Participants were randomised 2:2:1 in a blinded fashion to receive LEN, F/TAF or F/TDF. We assessed 1‐year persistence in a random, pre‐selected 10% sample of participants, including only those who could have had ≥1 year of study follow‐up at the time of the interim analysis. Persistence to SC injections (LEN or placebo) was defined as on‐time injection at baseline and Week 26, and on‐time follow‐up visit at Week 52. Persistence to oral study drug (F/TAF or F/TDF) was defined as dried blood spot (DBS) tenofovir‐diphosphate concentrations consistent with ≥4 doses/week at Weeks 13, 26, 39 and 52.

**Results**: Of 265 participants in this analysis, 112 were randomised to receive LEN plus daily oral placebo, and 153 to receive daily F/TAF (n = 99) or F/TDF (n = 54) plus LEN placebo, and had ≥1 available DBS sample. For the LEN group, 89/112 (79.5%) participants were persistent at 1 year versus 8/153 (5.2%) participants in the oral PrEP groups. Overall, persistence to SC injections, including both LEN and placebo, was similar across study arms (79.5%, 81.8% and 75.9% for LEN, F/TAF and F/TDF arms, respectively).

**Conclusions: **Our approach to evaluating persistence in PURPOSE 1 allowed us to measure consistent adherence to PrEP over time: an important predictor of PrEP effectiveness. We found significantly higher persistence to injectable PrEP versus daily oral PrEP in PURPOSE 1, which supports the potential for LEN to have individual and public health benefits beyond currently available PrEP options. This approach may be valuable to assess the potential impact of new PrEP modalities elsewhere, as is planned in the PURPOSE 5 study of LEN and F/TDF in the UK and France.

### O49 Twice‐yearly lenacapavir PrEP in cisgender gay men, transgender women and men, and gender‐diverse people (PURPOSE 2)

Colleen F Kelley^1^, Maribel Acevedo‐Quiñones^2^, Allison L Agwu^3^, Anchalee Avihingsanon^4^, Paul Benson^5^, Jill Blumenthal^6^, Cynthia Brinson^7^, Carlos Brites^8^, Pedro Cahn^9^, Valeria D Cantos^10^, Jesse Clark^11^, Meredith Clement^12^, Cathy Creticos^13^, Gordon Crofoot^14^, Ricardo S Diaz^15^, Susanne Doblecki‐Lewis^16^, Jorge A Gallardo‐Cartagena^17^, Aditya Gaur^18^, Beatriz Grinsztejn^19^, Juan Carlos Hinojosa^20^, Theo Hodge^21^, Richard Kaplan^22^, Marcus Lacerda^23^, Anthony LaMarca^24^, Marcelo H Losso^25^, Jose Valdez Madruga^26^, Kenneth H Mayer^27^, Anthony Mills^28^, Karam Mounzer^29^, Nkosiphile Ndlovu^30^, Richard M Novak^31^, Alma Perez Rios^32^, Nittaya Phanuphak^33^, Moti Ramgopal^34^, Peter J Ruane^35^, Breno Santos^36^, Patric Schine^37^, Tanya Schreibman^38^, LaShonda Y Spencer^39^, Olivia T Van Gerwen^40^, Ricardo Vasconcelos^41^, Jose Gabriel Vasquez^42^, Zwelethu Zwane^43^, Stephanie Cox^44^, Chris Deaton^45^, Ramin Ebrahimi^44^, Pamela Wong^44^, Renu Singh^44^, Lillian B Brown^44^, Christoph C Carter^44^, Moupali Das^44^, Jared M Baeten^44^, Onyema Ogbuagu ^46^, for the PURPOSE 2 Study Team

^1^Hope Clinic of Emory University School of Medicine, Decatur, GA and Grady Health System, Atlanta, GA, USA. ^2^Centro Ararat, San Juan, Puerto Rico. ^3^Divisions of Pediatric and Adult Infectious Diseases, The Johns Hopkins University School of Medicine, Baltimore, MD, USA. ^4^The HIV Netherlands Australia Thailand Research Collaboration, Thai Red Cross AIDS Research Centre and Center of Excellence in Tuberculosis, Faculty of Medicine, Chulalongkorn University, Bangkok, Thailand. ^5^Be Well Medical Center, Berkley, MI, USA. ^6^Department of Medicine, University of California San Diego, San Diego, CA, USA. ^7^Central Texas Clinical Research, Austin, TX, USA. ^8^Complexo Hospitalar Universitário Professor Edgard Santos, Salvador, Brazil. ^9^Fundación Huésped, Buenos Aires, Argentina. ^10^Division of Infectious Diseases, Emory University – Ponce de Leon Center Clinical Research Site, HIV/AIDS Clinical Trials Unit, Atlanta, GA, USA. ^11^Department of Medicine, University of California, Los Angeles, CA, USA. ^12^Department of Infectious Diseases,Louisiana State University Health Sciences Center – New Orleans, New Orleans, LA, USA. ^13^Howard Brown Health, Chicago, IL, USA. ^14^Crofoot MD Clinic and Research Center, Houston, TX, USA. ^15^Universidade Federal de São Paulo, São Paulo, Brazil. ^16^Division of Infectious Diseases, University of Miami Miller School of Medicine, Miami, FL, USA. ^17^Centro de Investigaciones Tecnológicas, Biomédicas y Medioambientales, Universidad Nacional Mayor de San Marcos, Lima, Peru. ^18^ Department of Infectious Diseases, St. Jude Children's Research Hospital, Memphis, TN, USA. ^19^Fundação Oswaldo Cruz – Instituto Nacional de Infectologia Evandro Chagas, Rio de Janeiro, Brazil. ^20^Asociación Civil Selva Amazónica, Iquitos, Peru. ^21^Washington Health Institute, Washington, DC, USA. ^22^Desmond Tutu Health Foundation, Cape Town, South Africa. ^23^Fundação de Medicina Tropical Doutor Heitor Vieira Dourado, Manaus, Brazil. ^24^Therafirst Medical Center, Fort, FL, USA. ^25^Hospital General de Agudos José María Ramos Mejía, Buenos Aires, Argentina. ^26^Centro de Referência e Treinamento DST/AIDS‐SP, São Paulo, Brazil. ^27^The Fenway Institute, Fenway Health and Department of Medicine, Harvard Medical School, Boston, MA, USA. ^28^Mills Clinical Research, West Hollywood, CA, USA. ^29^Philadelphia FIGHT Community Health Centers, Jonathan Lax Treatment Center, Philadelphia, PA, USA. ^30^Wits RHI, Faculty of Health Sciences, School of Public Health, University of the Witwatersrand, Johannesburg, South Africa. ^31^Division of Infectious Diseases, University of Illinois Health Sciences, Chicago, IL, USA. ^32^Centro de Investigacion Farmaceutica Especializada de Occidente SC, Guadalajara, Mexico. ^33^Institute of HIV Research and Innovation, Pribta Tangerine Clinic, Bangkok, Thailand. ^34^Midway Immunology and Research Center, Fort Pierce, FL, USA. ^35^Ruane Clinical Research, Los Angeles, CA, USA. ^36^Infectious Diseases Service, Hospital Nossa Senhora da Conceição, Port Alegre, Brazil. ^37^Bios Clinical Research, LLC, Palm Springs, CA, USA. ^38^CAN Community Health, Sarasota, FL, USA. ^39^Drew Center for AIDS Research, Education, and Services, Charles R. Drew University, Los Angeles, CA, USA. ^40^Department of Medicine, Division of Infectious Diseases, University of Alabama at Birmingham, Birmingham, AL, USA. ^41^Hospital das Clínicas da Faculdade de Medicina da Universidade de São Paulo, São Paulo, Brazil. ^42^Via Libre, Lima, Peru. ^43^The Aurum Institute – Pretoria Clinical Research Site, Pretoria, South Africa. ^44^Gilead Sciences, Foster City, CA, USA. ^45^Gilead Sciences, Cambridge, UK. ^46^Section of Infectious Diseases, Yale School of Medicine, New Haven, CT, USA

**Background**: Twice‐yearly subcutaneous lenacapavir (LEN) has been shown to be safe and efficacious for HIV prevention in cisgender women. We evaluated LEN for pre‐exposure prophylaxis (PrEP) in cisgender gay, bisexual and other men who have sex with men and gender‐diverse populations who are disproportionately affected by HIV.

**Materials and methods**: PURPOSE 2 (NCT04925752) is a phase 3, double‐blind, active‐controlled, randomised trial evaluating HIV prevention efficacy of twice‐yearly subcutaneous LEN among cisgender gay, bisexual and other men, transgender women, transgender men and non‐binary people who have sex with partners assigned male at birth from the Americas, Africa and Asia. Participants were randomised 2:1 to receive subcutaneous LEN every 26 weeks or daily oral emtricitabine‐tenofovir disoproxil fumarate (F/TDF), with corresponding placebos. The primary efficacy endpoint compared HIV incidence in the LEN group with background HIV incidence in the screened cohort, and the secondary analysis compared HIV incidence between LEN and F/TDF groups. LEN adherence was defined as administration within 28 weeks of prior injection; oral F/TDF adherence was evaluated by dried blood spot (DBS) assessment of tenofovir diphosphate levels in a randomly preselected 10% of participants’ study visits.

**Results**: Among 3267 participants who were initially HIV negative, two incident HIV infections occurred in the LEN group (0.10/100 person‐years; 95% CI: 0.01‐0.37) versus nine infections in the F/TDF group (0.93/100 person‐years; 95% CI: 0.43‐1.77). HIV incidence in the screened cohort was 2.37/100 person‐years (95% CI: 1.65‐3.41; N = 4637). LEN significantly reduced HIV incidence versus background incidence (incidence rate ratio [IRR]: 0.04; 95% CI: 0.01‐0.18) and F/TDF (IRR: 0.11; 95% CI: 0.02‐0.51). LEN and placebo injection adherence was high (91% on time). No new or significant safety concerns with LEN were reported. Injection‐site reactions were more frequent in the LEN group (83.2%) versus the F/TDF group (69.5%), but discontinuations due to these reactions were low (1.2%). DBS drug levels in the 10% adherence cohort, and LEN pharmacokinetics and resistance analyses in those who acquired HIV, are pending.

**Conclusions**: Twice‐yearly subcutaneous LEN showed superior efficacy to daily oral F/TDF in preventing HIV and was safe and well tolerated among cisgender men and trans and gender‐diverse people.

## POSTER PRESENTATIONS

### ARV‐based prevention—Vertical transmission

### P001 Postnatal prophylaxis among infants born to women living with HIV in England, 2018−2022

Emily Dema ^1^, Rebecca Sconza^1^, Helen Peters^1^, Alasdair Bamford^2^, Hermione Lyall^3^, Kate Francis^1^, Claire Thorne^1^

^1^Great Ormond Street Institute of Child Health, University College London, London, UK, ^2^Department of Infectious Diseases, Great Ormond Street Hospital for Children NHS Foundation Trust, London, UK, ^3^Department of Paediatric Infectious Diseases, Imperial College Healthcare NHS Trust, London, UK

**Background**: British HIV Association (BHIVA) guidelines recommend a risk‐stratification approach for infant postnatal prophylaxis (PNP). Vertical transmission risk level (very low/low/high) is determined based on duration of maternal ART, viral load (VL) throughout pregnancy/at delivery, with consideration of gestational age, ART adherence and resistance. We describe clinical practice around PNP use and alignment with BHIVA guidelines risk levels since their introduction in 2018.

**Methods**: The Integrated Screening Outcomes Surveillance Service (ISOSS), part of the NHS Infectious Diseases in Pregnancy Screening Programme, conducts comprehensive, population‐based surveillance of pregnancies in (and infants of) women living with HIV in England. Data collection includes infant PNP type and duration. We analysed data on all mother‐infant pairs born from 01 Jan 2018 to 31 Dec 2022 (reported by 31 Mar 2024). We applied adapted risk‐strata for this analysis (Table 1), reflecting ISOSS data availability.

**P001: Table 1:** British HIV Association (BHIVA) guidelines for infant postnatal prophylaxis (PNP) and Integrated Screening Outcomes Surveillances Service (ISOSS)‐adapted risk‐strata for analysis

BHIVA risk strata	Recommended PNP	Adapted risk‐strata using available ISOSS data
**Very low risk**		
Woman on combination antiretroviral treatment (cART) for >10 weeks; AND	Two weeks of zidovudine monotherapy	Woman on cART for >10 weeks; AND
Two documented maternal viral load (VL) <50 copies/ml during pregnancy at least 4 weeks apart; AND		Maternal VL <50 copies/ml at delivery (within 30 days prior or 7 days after delivery)
Maternal VL <50 copies/ml at or after 36 weeks		
Low risk		
If the criteria for “very low risk” are not all fulfilled but maternal VL is <50 copies/ml at or after 36 weeks	Four weeks of zidovudine monotherapy	Adapted criteria for “very low risk” are not all fulfilled, but maternal VL is <50 copies/ml at delivery (defined above)
If the infant is born prematurely (<34 weeks) but most recent maternal VL is <50 copies/ml		If the infant is born prematurely (<34 weeks), but delivery VL is <50 copies/ml
**High risk**		
Maternal birth VL is known to be or likely to be >50 copies/ml on day of birth, if uncertainty about maternal adherence/VL	Three‐drug combination postnatal prophylaxis (zidovudine, lamivudine and nevirapine) for 4 weeks	Delivery VL (within 30 days prior or 7 days after delivery) is >50 HIV RNA copies/ml

Abbreviations: BHIVA, British HIV Association; cART, combination antiretroviral therapy; HIV, human immunodeficiency virus; RNA, ribonucleic acid.

**Results**: There were 2015 live‐born infants from 1966 pregnancies. According to the adapted risk strata (Table 1), 86.6% (1702) of pregnancies fulfilled “very low‐risk,” 6.1% (119) “low‐risk” and 7.5% (145) “high‐risk.” Median maternal delivery VL in the “high‐risk” group was 102 copies/ml (range 51–1,083,193). Among 2015 infants, 92.4% (1862) received zidovudine (ZDV) alone, 7.0% (142) received a three‐drug combination and 0.1% (3) received no PNP. Among infants in “very low‐risk” and “low‐risk” strata (Figure 1), 97.6% (1687/1729) and 85.1% (114/134) received ZDV alone, respectively. Among infants in the adapted “high‐risk” strata, 57.9% (88/152) received three‐drug combinations. Of 61 infants in this stratum who received ZDV alone, delivery maternal VL was >50 but <200 copies/ml in 50 (82.0%). Overall, 4/2015 infants were infected; two “very low‐risk” received ZDV (1) or a three‐drug combination (1) and of two “high‐risk” (both *in utero* infections), one received a three‐drug combination, and one ZDV/lamivudine/nevirapine/raltegravir.

**P001: Figure 1:**
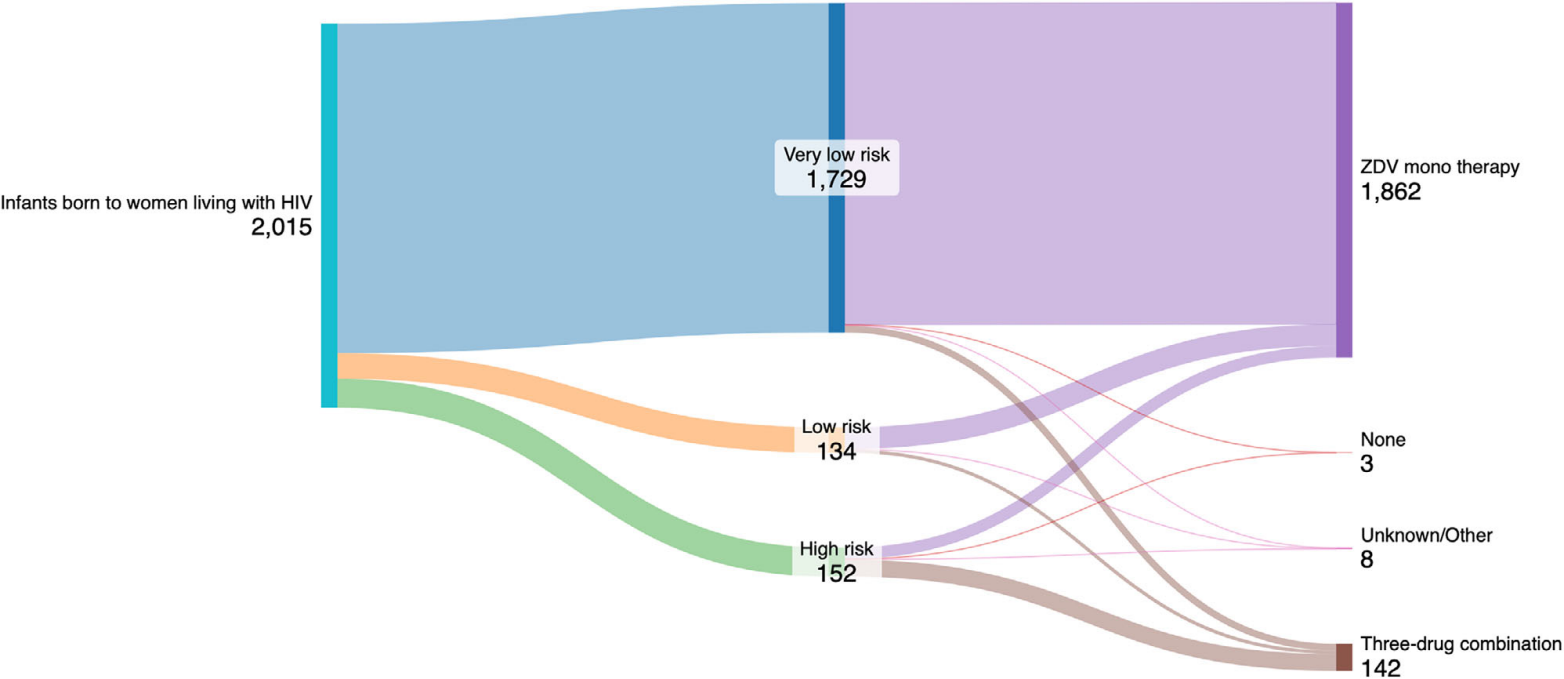
Infant postnatal prophylaxis (PNP) among infants born to women living with HIV in England from 1 January 2018 to 31 December 2022, by adapted British HIV Association (BHIVA) risk groups. ZDV, zidovudine.

**Conclusions**: Although caution is required due to limitations of currently available surveillance data in determining risk strata, these data provide the first partial evidence for good clinical adherence to current policies on risk strata and PNP, as well as the possible power to monitor practice using observational data. Potential modifications to ISOSS data collection tailored to upcoming guidelines would ensure any limitations are minimized.

### P002 Outcomes following prenatal exposure to DTG‐containing antiretroviral therapy regimens: data from the DOLOMITE‐EPPICC study

Rebecca Sconza ^1^, Georgina Fernandes^1^, Heather Bailey^2^, Karoline Aebi‐Popp^3^, Luminita Ene^4^, Marco Floridia^5^, Anna Maria Gamell^6^, Marta Illán Ramos^7^, Helen Peters^1^, Anna Samarina^8^, Leigh Ragone^9^, Vani Vannappagari^9^, Claire Thorne^1^

^1^UCL Great Ormond Street Institute of Child Health, University College London, London, UK, ^2^University College London, London, UK, ^3^Department of Infectious Diseases, Bern University Hospital, Bern, Switzerland, ^4^HIV Department, Victor Babes Hospital, Bucharest, Romania, ^5^National Center for Global Health, Istituto Superiore di Sanità, Rome, Italy, ^6^Department of Paediatrics, Hospital Sant Joan d Déu, Esplugues de Llobregat, Spain, ^7^Department of Paediatrics, Hospital Universitario Clínico San Carlos, Madrid, Spain, ^8^Department of Motherhood and Childhood, St Petersburg Centre for AIDS and Infectious Diseases, St Petersburg, Russian Federation, ^9^Epidemiology and Real World Evidence, ViiV Healthcare, Durham, NC, USA

**Background**: Dolutegravir (DTG)‐based antiretroviral therapy (ART) is widely recommended during pregnancy for maternal viral suppression and prevention of vertical HIV transmission. We aimed to describe pregnancy and neonatal outcomes (including birth defects) by earliest antenatal DTG‐containing regimen using real‐world European data.

**Materials and methods**: DOLOMITE‐EPPICC is a multi‐cohort European observational study of DTG use in pregnant individuals living with HIV and their infants. Our analysis focussed on five DTG‐containing regimens: DTG+lamivudine (3TC) +abacavir (ABC), DTG+tenofovir disoproxil fumarate (TDF) +emtricitabine (FTC), DTG+tenofovir alafenamide (TAF) +FTC, DTG+3TC and DTG+rilpivirine (RPV). Earliest antenatal DTG‐containing regimen was the first DTG‐containing regimen reported in pregnancy. Birth defects were classified using World Health Organization's International Classification of Diseases: Tenth Revision (ICD‐10); prevalence of birth defects was calculated among live‐born infants. Analysis of other neonatal outcomes (preterm delivery, low birthweight, small‐for‐gestational‐age) was restricted to singleton live‐born infants.

**Results**: There were 833 pregnancies with any DTG use included across seven cohorts. Median age at conception was 32 years (IQR 27–36). Earliest DTG use was in the first trimester for 565 (68.2%) and the second/third trimester for 264 (31.8%) (four missing timing). The earliest DTG‐containing regimen was DTG+3TC+ABC in 468 (56.2%), DTG+TDF+FTC in 132 (15.9%), DTG+TAF+FTC in 23 (2.8%), DTG+3TC/RPV in seven (0.8%) pregnancies. Of the remaining 203 (24.4%) pregnancies with other first DTG‐containing regimens, most (78.8% [160/203]) were on regimens containing a drug from an additional anchor class (e.g. darunavir/ritonavir+TDF+FTC+DTG, atazanavir/ritonavir+TDF+FTC+DTG). Pregnancy and neonatal outcomes by earliest DTG‐containing regimen are presented in Table 1. The overall prevalence of birth defects was 4.3% (95% CI 3.0–6.0%).

**P002: Table 1:** Pregnancy and neonatal outcomes by earliest prenatal DTG‐containing regimen in DOLOMITE‐EPPICC

	All DTG‐containing regimens	DTG+3TC+ABC	DTG+TDF+FTC	DTG+TAF+FTC	DTG+3TC/RPV^a^	Other^b^
Total pregnancies	833	468	132	23	7	203
Live birth	770 (92.4%)	425^c^ (90.8%)	124 (93.9%)	21 (91.3%)	6 (85.7%)	194 (95.6%)
Spontaneous abortion	37 (4.4%)	25 (5.3%)	4 (3.0%)	2 (8.7%)	1 (14.3%)	5 (2.5%)
Induced abortion	21 (2.5%)	14 (3.0%)	4 (3.0%)	0	0	3 (1.5%)
Stillbirth	5 (0.6%)	4 (0.9%)	0	0	0	1 (0.5%)
Total live‐born infants	790	438	127	21	6	198
With birth defects	34/783 (4.3%, 95% CI 3.0−6.0%)	22/434 (5.1%, 95% CI 3.2−7.6%)	4/125 (3.2%, 95% CI 0.9−8.0%)	0	0	8/197 (4.1%, 95% CI 1.8−7.8%)
Total singleton live‐born infants	750	412	121	21	6	190
Preterm (<37 weeks)	97/729 (13.3%)	52/407 (12.8%)	12/118 (10.2%)	5 (23.8%)	2 (33.3%)	26/177 (14.7%)
Very preterm (<34 weeks)	30/729 (4.1%)	16/407 (3.9%)	5/118 (4.2%)	2 (9.5%)	1 (16.7%)	6/177 (3.4%)
Low birthweight (<2500 g)	92/742 (12.4%)	48/408 (11.8%)	16/118 (13.6%)	3 (14.3%)	1 (16.7%)	24/189 (12.7%)
Very low birthweight (<1500 g)	19/742 (2.6%)	11/408 (2.7%)	3/118 (2.5%)	3 (14.3%)	0	2/189 (1.1%)
Small‐for‐gestational‐age^d^	61/706 (8.6%)	34/396 (8.6%)	12/111 (10.8%)	3 (14.3%)	0	12/172 (7.0%)

*Note*: Amended denominator indicated where data incomplete.

Abbreviations: 3TC, lamivudine; ABC, abacavir.

^a^DTG+3TC (*n* = 3) or DTG+RPV (*n* = 4).

^b^Includes DTG‐containing regimens containing drugs of additional anchor classes.

^c^Includes one twin pregnancy with discordant outcome (one miscarried twin).

^d^Classified using INTERGROWTH‐21st standards.

**Conclusions**: The birth defect prevalence reported here is consistent with rates reported for DTG‐exposed pregnancies in the Antiretroviral Pregnancy Registry (3.96%). Monitoring of use and safety of DTG‐containing regimens in pregnancy is ongoing in EPPICC, as sample size for some groups is too small to exclude association with rare outcomes.

### P003 Antiretroviral therapy in pregnancy in England in 2019−2022: common regimens and treatment modifications

Rebecca Sconza ^1^, Helen Peters^1^, Laurette L. Bukasa^1^, Laura Byrne^2^, Alasdair Bamford^3^, Hermione Lyall^4^, Graham P. Taylor^5^, Claire Thorne^1^

^1^Population, Policy and Practice Research and Teaching Department, UCL Great Ormond Street Institute of Child Health, London, UK, ^2^School of Medicine, St George's, University of London, London, UK, ^3^Department of Infectious Diseases, Great Ormond Street Hospital for Children NHS Foundation Trust, London, UK, ^4^Department of Paediatric Infectious Diseases, Imperial College Healthcare NHS Trust, London, UK, ^5^Section of Virology, Department of Infectious Disease, Imperial College London, London, UK

**Background**: Nearly all diagnosed pregnant people living with HIV in England receive antenatal antiretroviral therapy (ART). We aimed to describe commonly used ART regimens and the rate of regimen modification in pregnancy in recent years.

**Methods**: We used national surveillance data on pregnancies in people living with HIV‐1 reported to the Integrated Screening Outcomes Surveillance Service with an estimated date of delivery (EDD) 2019–2022. Pregnancies with unknown outcome or ART receipt were excluded. We described common first antenatal ART regimens (reported in ≥10 pregnancies) for those with known timing and type of ART. The first regimen was the earliest reported in pregnancy; regimen modification was defined as any change to the first pregnancy regimen (i.e. switch, intensification, simplification), excluding dosage changes.

**Results**: There were 2464 pregnancies in 2132 people. Median age at EDD was 35 years (IQR 30–39). Antenatal ART was used in 98.9% (2436/2464) of pregnancies (99.9% [2179/2180] of those ending in live/stillbirth). Most (81.4% [2001/2459]) pregnancies were known to be conceived on ART. Where antenatal ART was used, timing/type were reported for 98.3% (2397/2436). All common first ART regimens are presented in Figure 1. Among pregnancies conceived on ART, most common (reported in >5%) first regimens were efavirenz (EFV) + tenofovir disoproxil fumarate (TDF) + emtricitabine (FTC) (13.5% [267/1974]), rilpivirine (RPV) +TDF+FTC (11.6% [228/1974]), darunavir/ritonavir (DRV/r) +TDF+FTC (8.0% [158/1974]), dolutegravir (DTG) + lamivudine (3TC) + abacavir (ABC) (7.7% [152/1974]), and raltegravir (RAL) +TDF+FTC (6.4% [127/1974]). Where ART was initiated during pregnancy (55.7% [234/420] diagnosed antenatally), median gestational age at start was 15 weeks (IQR 12–19); most common first regimens were RAL+TDF+FTC (17.7% [75/423]), DRV/r+TDF+FTC (15.4% [65/423]), atazanavir/ritonavir (ATV/r) +TDF+FTC (12.5% [53/423]), DTG+3TC+ABC (9.2% [39/423]) and DTG+TDF+FTC (8.3% [35/423]). Overall, 8.3% (199/2397) of first regimens contained cobicistat (CBS) (195 started pre‐conception). Of pregnancies ending in live/stillbirth, 19.1% (413/2164) had a regimen modification, more commonly among those conceived on ART (20.2% [355/1755] vs. 14.2% [58/409] among those initiating antenatally, *p* = 0.005). Where the first regimen contained pre‐conception CBS, modification occurred in 70.9% (117/165) at median 11 gestational weeks (IQR 8–16), with 103/117 having a second regimen excluding CBS.

**P003: Figure 1:**
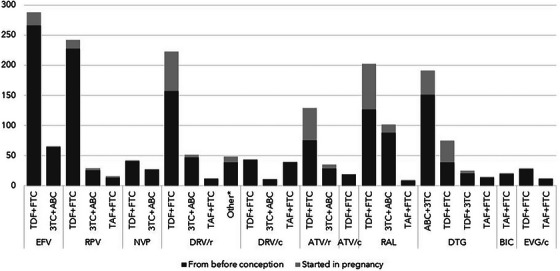
Common first ART regimens in pregnancy by timing of initiation, 2019–2022. *Includes reported regimens DRV/r+TDF (*n* = 27), DRV/r+TAF (*n* = 12) and DRV/r+3TC (*n* = 10). *Note*: Figure excludes regimens DRV/r+DTG+TDF+FTC (*n* = 11), DRV/r+RAL+TDF+FTC (*n* = 10) and TDF+FTC (no anchor reported) (*n* = 12). 3TC, lamivudine; ABC, abacavir; ATV/c, atazanavir/cobicistat; ATV/r, atazanavir/ritonavir; BIC, bictegravir; DRV/c, darunavir/cobicistat; DRV/r, darunavir/ritonavir; DTG, dolutegravir; EFV, efavirenz; EVG/c, elvitegravir/cobicistat; FTC, emtricitabine; NVP, nevirapine; RAL, raltegravir; RPV, rilpivirine; TAF, tenofovir alafenamide; TDF, tenofovir disoproxil fumarate.

**Conclusions**: Modification was frequent but may reflect varied clinical scenarios (e.g. safety concerns, treatment failure, etc.). Future investigation will consider impact on viral suppression/other outcomes.

### P004 HIV treatment cascade among pregnant women with pre‐conception diagnosis: 2017−2022

Helen Peters ^1^, Rebecca Sconza^1^, Kate Francis^1^, Laura Byrne^2^, Claire Thorne^1^

^1^Integrated Screening Outcomes Surveillance Service, UCL Great Ormond Street Institute of Child Health, London, UK, ^2^Institute for Medical & Biomedical Education, St George's University Hospitals NHS Trust, London, UK

**Background**: The Joint United Nations Programme on HIV/AIDS (UNAIDS) “treatment cascade” targets have been met in England since 2017 and the UK Health Security Agency (UKHSA) HIV Action Plan aims to end new transmissions by 2030. In England, antenatal HIV screening coverage is 99.8% and ∼90% of women living with HIV becoming pregnant are already diagnosed.

**Methods**: The Integrated Screening Outcomes Surveillance Service (ISOSS), part of the NHS Infectious Diseases in Pregnancy Screening Programme, collects data on all pregnancies in women diagnosed with HIV by delivery and their infants in England. We describe a treatment cascade for pregnancies in women diagnosed pre‐conception, restricted to livebirths with antenatal booking 2017–2022, known viral load (VL) at delivery (≤30 days pre‐delivery and <7 days post‐delivery).

**Results**: Of the 2464 women included, 88.1% (809/918) were on pre‐conception ART (pART) in 2017–18, 92.2% (780/846) 2019–20 and 93.0% (651/700) 2021–22, *p* < 0.001 (Figure 1). Age and ethnicity differed (7.6% <25 years vs. 4.3% with pART, *p* < 0.05) (16.3% white, 8.9% Black Caribbean vs. 23.8%, 4.1% with pART, *p* < 0.05). Among pregnancies conceived on ART, first pregnancy VL (fVL) was undetectable (<50 c/ml) in 89.7% (2009/2240) and delivery VL (dVL) undetectable in 95.1% (2130/2240) (no change over time). Among those with detectable fVL, 81.4% (188/231) had undetectable dVL (no change over time). Median detectable dVL was 100 c/ml (range 51–85,000). Those with detectable dVL were more likely to be non‐UK born (*p* < 0.05). There was no difference in timing of booking, ethnicity or age. Among the 224 pregnancies in women without pART: 38.7% had late antenatal booking (≥13 weeks) (vs. 24.3% with pART, *p* < 0.001); 99.1% (222/224) received ART antenatally (28.2% started treatment in the first and 12.0% in the third trimester); 81.1% (180/222) had undetectable dVL (no difference with ART start in first trimester vs. later). Median detectable dVL was 90 c/ml (range 51–1 million c/ml).

**P004: Figure 1:**
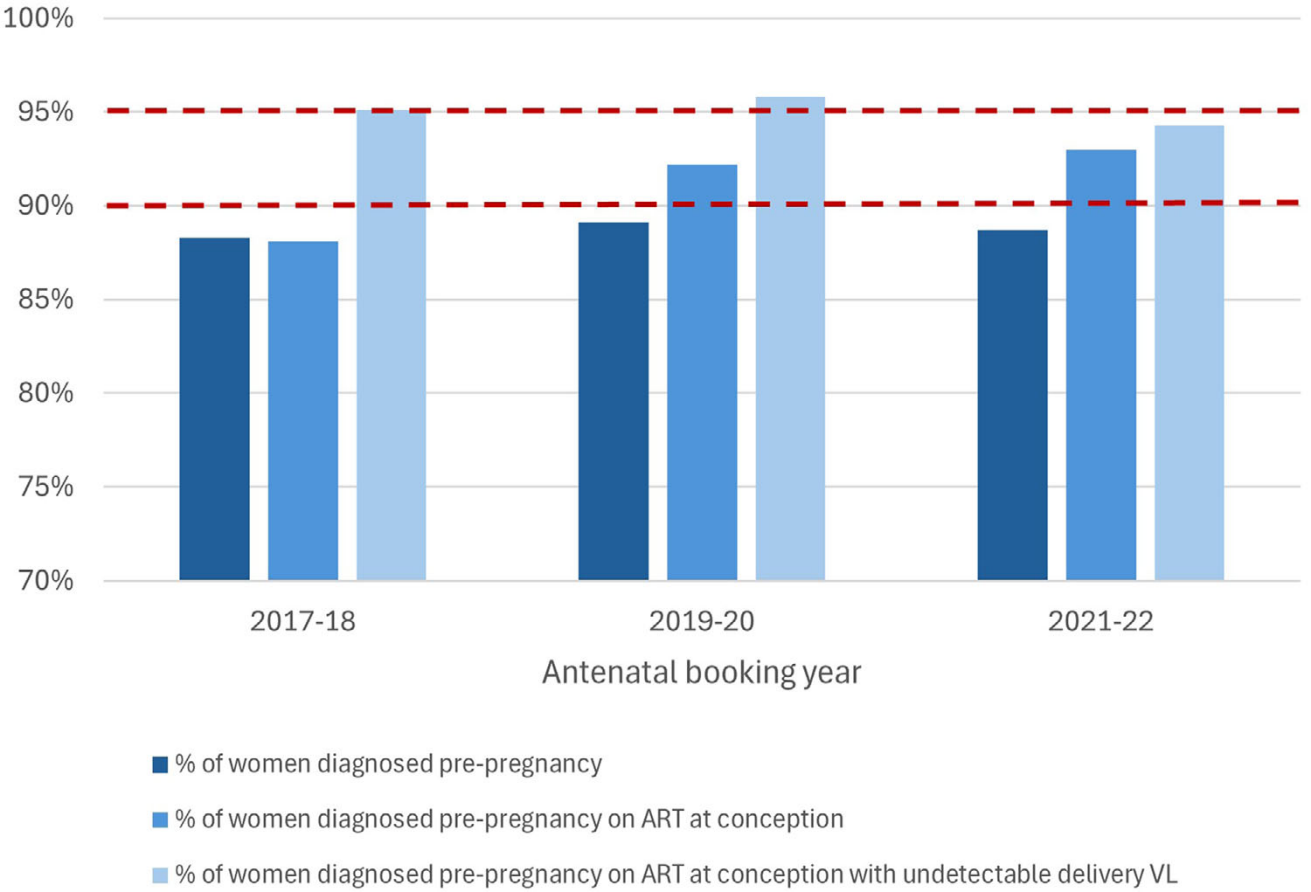
Diagnosis and treatment cascade for pregnant women seen for antenatal care in England, 2017–2022.

**Conclusions**: Most pregnant women living with HIV are on treatment before pregnancy and deliver with undetectable VL. The proportion of pregnancies in women diagnosed with HIV but not on treatment at conception is small and declining over time. Further work is needed to understand barriers to timely care for specific groups, as late booking limits opportunity to reach viral suppression by delivery.

### P005 The peripartum viral load cascade and outcomes of infants exposed to HIV in Lesotho

Kathrin Haenggi^1^, Moleboheng Mokebe^2^, Lipontso Motaboli^2^, Makobefo Chakela^2^, Mpho Kao^3^, Mathebe Kopo^2^, Irene Ayakaka^4^, Monrongoe Nyakane^5^, Tapiwa Tarumbiswa^5^, Niklaus Daniel Labhardt ^1^, Jennifer Anne Brown^1^, Nadine Tschumi^1^

^1^Department of Clinical Research, University Hospital Basel, Basel, Switzerland, ^2^SolidarMed Lesotho, Butha‐Buthe, Lesotho, ^3^SolidarMed Lesotho, Mokhotlong, Lesotho, ^4^SolidarMed Lesotho, Maseru, Lesotho, ^5^Ministry of Health, Government of Lesotho, Maseru, Lesotho

**Background**: Sustained viral suppression during pregnancy and breastfeeding and timely postpartum antiretroviral therapy (ART) are key to preventing vertical transmission. This study aims to describe the peripartum viral load monitoring cascade among pregnant and breastfeeding people, and ART‐initiation, HIV testing and outcomes of infants exposed to HIV in routine care in Lesotho, southern Africa.

**Materials and methods**: Data from a medical charts review of paper‐based registers at 20 healthcare facilities in two districts of Lesotho were combined with data from the Viral Load Cohort North East Lesotho (VICONEL). We included people with HIV who attended their first antenatal care visit at a participating healthcare facility after 31 December 2019, had a delivery date before 1 January 2022 and initiated dolutegravir‐based ART before delivery, as well as their infants.

**Results**: A total of 350 pregnancies among 345 pregnant people with HIV and 354 infants exposed to HIV were recorded (Figure 1). Characteristics of pregnant people with HIV and viral load monitoring during pregnancy and postpartum are displayed in Table 1. Among 17/350 (5%) with an initial viral load ≥1000 copies/ml, a timely follow‐up (≤4 months) viral load was provided for 2/17 (12%). Among 230/350 (66%) with an initial viral load <1000 copies/ml, a timely follow‐up (≤7 months) was provided for 117/230 (51%). For 103/350 (29%), no viral load measurement was available during pregnancy. Overall, peripartum viral load monitoring met the guidelines recommended intervals in 152/350 (43%) pregnancies. Among infants exposed to HIV, 299/354 (84%), 272/354 (77%) and 172/354 (49%) received post‐natal ART within <48 hours, had a timely first infant HIV testing (≤2 months) and 9‐month HIV testing, respectively. Vertical transmissions were documented in 2/354 (1%) infants, after ART initiation in the 34th and 21st gestational week, respectively.

**P005: Figure 1:**
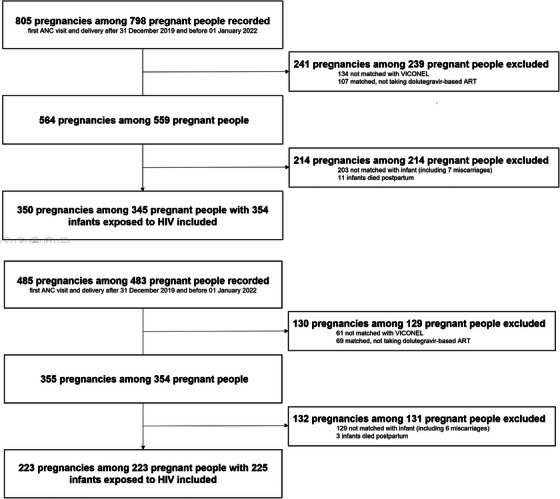
Study flow chart. ANC, antenatal care; ART, antiretroviral therapy; VICONEL, Viral Load Cohort North‐East Lesotho.

**P005: Table 1:** Characteristics of participating pregnant people with HIV and viral load monitoring during pregnancy and postpartum

	*N* = 350
**Characteristics of peripartum parent**	
Age at delivery, *n* (%)	
15−19 years	26 (7%)
20−29 years	144 (41%)
30−39 years	152 (43%)
≥40 years	28 (8%)
Parity, *n* (%)	
0	64 (18%)
1	109 (31%)
2	86 (25%)
3	44 (13%)
≥4	47 (13%)
Gestational age at first ANC visit in weeks, median (IQR)	20.7 (14.0−27.4)
Median number of ANC visits attended (IQR) [range]	3 (2−4) [1−7]
**HIV and ART history**	
History of viraemia before pregnancy, *n* (%)	
Always VL <50 copies/ml (no history of viraemia)	137 (39%)
Always VL <1000, at least one VL ≥50 copies/ml	43 (12%)
At least one VL ≥1000 copies/ml	21 (6%)
No VL before pregnancy/not documented	149 (43%)
ART initiated, *n* (%)	
Before pregnancy	260 (74%)
Newly initiated during pregnancy	90 (26%)
Median time taking ART at delivery [years], median (IQR)	3.4 (0.6−6.1)
ART regimen at delivery, *n* (%)	
TDF‐3TC‐DTG	349 (100%)
ABC‐3TC‐DTG	1 (0%)
**Viral load testing and outcomes**	
Number of VL tests per pregnancy during pregnancy and 6 months postpartum, median (IQR) [range]	
During pregnancy	1 (0−1) [0−3]
0−6 months postpartum	0 (0−1) [0−2]
Throughout pregnancy and 6 months postpartum	1 (1−2) [0−4]
Timing of first VL after pregnancy start, *n* (%)	
First trimester	79 (23%)
Second trimester	88 (25%)
Third trimester	80 (23%)
≤6 months postpartum	77 (22%)
No VL ≤6 months postpartum/not documented	26 (7%)
Timing of first VL if ART initiation during pregnancy, *n* (%)	*n* = 90
In time (≤7 months after ART initiation)	66 (73%)
Late/no VL/not documented	24 (27%)
VL in copies/ml during pregnancy and 6 months postpartum, *n* (%)	
Always VL <50 copies/ml	263 (75%)
Always VL <1000, at least one VL ≥50 copies/ml	39 (11%)
At least one VL ≥1000 copies/ml	21 (6%)
No VL during pregnancy and 6 months postpartum/not documented	27 (8%)
VL in copies/ml at first VL (pregnancy), *n* (%)	
<50 copies/ml	205 (59%)
50−999 copies/ml	25 (7%)
≥1000 copies/ml	17 (5%)
No VL during pregnancy/not documented	103 (29%)
FU when VL <1000 copies/ml at first VL, *n* (%)	*n* = 230
In time (≤7 months)	117 (51%)
Late/no FU/not documented	113 (49%)
FU when VL ≥1000 copies/ml at first VL, *n* (%)	*n* = 17
In time (≤4 months), resuppressed to <1000 copies/ml	1 (6%)
In time (≤4 months), sustained VL ≥1000 copies/ml	1 (6%)
Late/no FU/not documented	15 (88%)

Abbreviations: 3TC, lamivudine; ABC, abacavir; ART, antiretroviral therapy; DTG, dolutegravir; FU, follow‐up; IQR, interquartile range; TDF, tenofovir disoproxil fumarate; VL, viral load.

**Conclusions**: There were gaps in the implementation of viral load monitoring in the priority population of pregnant and postpartum people with HIV. In combination with the suboptimal implementation of HIV testing among infants exposed to HIV, these gaps reflect a missed opportunity to improve prevention of vertical transmission of HIV.

### P006 Late or missed HIV diagnosis during pregnancy is still occurring in a high‐income country and represents a high risk of MTCT

Carmela Pinnetti ^1^, Cristina Marelli^2^, Alice Ranzani^3^, Valentina Mazzotta^1^, Lucia Taramasso^2^, Oscar Cirioni^4^, Andrea Costantini^5^, Barbara Menzaghi^6^, Rosa Fontana Del Vecchio^7^, Enrico Girardi^8^, Annalisa Saracino^9^, Antonella Cingolani^10^, Antonio Di Biagio^2^, Antonella d'Arminio Monforte^11^, Andrea Antinori^1^

^1^Clinical Department, National Institute for Infectious Diseases “L. Spallanzani”, IRCCS, Rome, Italy, ^2^Infectious Disease Clinic, IRCCS Ospedale Policlinico San Martino, Genoa, Italy, ^3^Infectious Disease Unit, IRCCS San Gerardo dei Tintori, Monza, Italy, ^4^Department of Biomedical Sciences & Public Health, Clinic of Infectious Disease, Polytechnic University of Marche, Ancona, Italy, ^5^Clinical Immunology Unit, Polytechnic University of Marche, Ancona, Italy, ^6^Department of Infectious Diseases, ASST Valle Olona, Busto Arsizio, Italy, ^7^Department of Infectious Diseases, Umberto I Public Hospital, Siracusa, Italy, ^8^Scientific Direction, National Institute for Infectious Diseases “L. Spallanzani”, IRCCS, Rome, Italy, ^9^Clinic of Infectious Diseases, Department of Precision and Regenerative Medicine and Ionian Area, University Hospital Polyclinic, University of Bari, Bari, Italy, ^10^Department of Safety and Bioethics, Clinic of Infectious Diseases, Fondazione Policlinico Universitario Agostino Gemelli IRCCS, Università Cattolica del Sacro Cuore, Rome, Italy, ^11^ICONA Foundation, Milan, Italy

**Background**: Among pregnant women with HIV (pWWH), integrated strategies of early diagnosis and treatment during pregnancy and delivery have resulted in MTCT reduction to <2%. We aim to analyse therapy changes in planning and during pregnancy and investigate pregnancy outcomes.

**Materials and methods**: We included all pWWH enrolled in ICONA cohort in 2011–2024. Chi‐squared and Wilcoxon rank‐sum test were used to describe population characteristics. Changes in ART, maternal immunovirological status and MTCT were analysed.

**Results**: Four hundred and three pregnancies in 293 pWWH were evaluated. Outcomes were available in 333 pregnancies (95 naives, 238 ART‐experienced) of 262 pWWH: 38 miscarriages, 31 voluntary pregnancy interruptions and 264 full‐term births (two intrauterine deaths). Characteristics of pWWH are summarized (Table 1). Seventy‐one women were diagnosed with HIV while pregnant (38 first, 23 second, 10 third trimester) and started ART at median gestational (IQR) time of 15 weeks (13–21). Eleven women received diagnosis and ART post‐partum. Foreign‐born pWWH had the same chance of Italian‐borns of being diagnosed late or after pregnancy (15/171 vs. 6/91, *p* = 0.54). Among the ART‐experienced, 76/238 (32.0%) switched ART 6 months before pregnancy or in the first trimester: dolutegravir was interrupted in 3/101 (3%), cobicistat in 15/114 (13%) and tenofovir alafenamide in 26/195 (13%). Tenofovir disoproxil/emtricitabine remained the most common backbone in pregnancy (112/264, 42%). At delivery, a substantial proportion of pWWH received protease inhibitors (32%) and integrase strand transfer inhibitor (23%). The proportion of women achieving viral suppression by the third trimester was 84% in 2011–2015 and 93% in 2016–2024; *p* < 0.02. Caesarean section occurred in 57.2%, and vaginal deliveries in 32.9% (no data in 9.8%). We observed two HIV‐positive newborns (0.8%): the first from a mother diagnosed late during pregnancy and starting ART shortly before delivery (29 weeks) without obtaining HIV‐RNA undetectability, the second in a woman lost to follow‐up during pregnancy and returned to care after delivery. Both pWWH were born outside Italy.

**P006: Table 1:** Main characteristics of pWWH

Variables	Total *N* = 262 (100%)	ART‐naive *N* = 95 (36%)	ART‐experienced *N* = 167 (64%)	*p*‐value
Age (years), median [IQR]	29 [25−33]	30 [25−36]	28 [25−32]	0.021
Ethnicity, *n* (%)				0.037
Asian	5 (1.9)	1 (1.0)	4 (2.4)	
Black	97 (37.0)	45 (47.4)	52 (31.1)	
Caucasian	131 (50.0)	36 (37.9)	95 (56.9)	
Hispanic‐Latino	25 (9.5)	11 (11.6)	14 (8.4)	
Other/unknown	4 (1.5)	2 (2.1)	2 (1.2)	
Foreign‐born, *n* (%)	171 (65.3)	73 (76.8)	98 (58.7)	0.003
Mode of HIV transmission, *n* (%)				0.182
Heterosexual contact	237 (90.5)	88 (92.6)	149 (89.2)	
IDU	11 (4.2)	5 (5.3)	6 (3.6)	
Other/unknown	14 (5.3)	2 (2.11)	12 (7.2)	
Years of HIV infection, median [IQR]	0.5 [0.1−3.2]	0.4 [0.1−1.0]	0.8 [0.2−7.3]	<0.001
AIDS diagnosis, *n* (%)	21 (8.0)	0 (0.0)	21 (12.6)	<0.001
Positive HCVAb, *n* (%)	20 (7.6)	4 (4.2)	16 (9.6)	0.116
Positive HBsAg, *n* (%)	10 (3.8)	3 (3.2)	7 (4.2)	0.675
Zenith HIV RNA (log10 copies/ml), median [IQR]	4.5 [3.9−5.1]	4.2 [3.4−4.8]	4.7 [4.1−5.3]	<0.001
Nadir CD4^+^, median [IQR]	313 [170−452]	400 [270−559]	269 [132−391]	<0.001
HIV RNA undetectable at first trimester, *n* (%)	91/262 (34.7)	22/95 (23.2)	69/167 (41.3)	0.003
HIV RNA undetectable at third trimester, *n* (%)	152/202 (75.3)	57/84 (67.9)	95 /118 (80.5)	0.04

Abbreviations: HBsAg, hepatitis B surface antigen; HCVAb, hepatitis C virus antibodies; IDU, injecting drug user.

**Conclusions**: HIV diagnosis occurred late during pregnancy or was missed even after delivery in 21/264 (8.0%) of pWWH, resulting in two cases of MTCT. Interventions are still needed to improve access to antenatal care and HIV testing, especially for foreign‐born women.

### P007 The experience of pregnancy in women living with HIV (WLWH) between 2000 and 2023: a single‐centre retrospective observational analysis

Sara Giovanna De Maria ^1^, Raffaella Marocco^2^, Alessandra Grimaldi^2^, Sara Corazza^2^, Andrea Gasperin^2^, Paola Zuccalà^2^, Cosmo Del Borgo^2^, Valeria Belvisi^2^, Tiziana Tieghi^2^, Miriam Lichtner^3^

^1^Infectious Diseases, La Sapienza, University of Rome, S.M. Goretti Hospital, Rome, Latina, Italy, ^2^Infectious Diseases, S.M. Goretti Hospital, Latina, Italy, ^3^Infectious Diseases, Sapienza University of Rome, Rome, Italy

**Background**: During recent years, the increased access to antiretroviral therapy (ART), the U═U paradigm, has significantly changed the reproductive landscape of WLWH. The main aim of our study is to describe the approach on MTCT during the last 23 years at our centre.

**Materials and methods**: This is a retrospective descriptive single‐centre study including 80 pregnancies, 56 WLWH followed at S. M. Goretti Hospital between 2000 and 2023. Data were compared between 2000–2016 and after 2016, when the U═U campaign was launched. Statistical analysis was conducted by using the GraphPad Prism programme.

**Results**: Patients’ characteristics are reported in Table 1.  A constant annual birth rate was observed in our study period (median of 3 pregnancies/year); no change followed the introduction of vaginal delivery in 2016. Non‐Italian patients increased in the later study group (57.3% vs. 73.6%; *p* = 0.28). Overall, 73.2% (41) women were known HIV carriers (mean infection duration: 6.5 years), while 26.7% (15) received HIV diagnosis during pregnancy (13 before 2016). Eleven participants had an AIDS diagnosis. Eighteen women had an HIV‐positive partner. About ART, 34 out of 80 pregnancies (42.5%) were on a PI‐containing regimen, while nucleoside backbone combination was used in 17 subjects (16 on TDF/FTC vs. one on ABC/3TC). Among the 56 gestations from 2008, 21 were on INSTIs, with 81% on raltegravir, and 9.5% on dolutegravir (introduced upon the third trimester). No patient changed ART regimen during pregnancy, and 17% did at least three therapy switches from pregnancy until today. Spontaneous vaginal delivery increased after 2016 (31.5% vs. 9.8%); 78.75% (63) underwent C‐section. Forty‐seven undetectable women before 2016, would have undergone vaginal delivery by using the U═U paradigm. Preterm delivery (<37 weeks) occurred in 12.5% pregnancies and the prevalence of low birth weight (<2.5 kg) was observed in 6.2%. No MTCT occurred thanks to the implementation of ART.

**P007: Table 1:** Patients’ characteristics

Variables	Total (*n* = 80)	2000−2016 (*n* = 61)	2017−2023 (*n* = 19)	*p*‐value
Total pregnancies	80	61	19	
Age (years), median	32.3 (21−47)	32.4 (21−47)	31.6 (22−41)	0.99
Italian	31/80 (55.3%)	26/61 (42.6%)	5/19 (26.3%)	
Other countries	25/80 (44.6%)	35/61 (57.3%)	14/19 (73.6%)	0.28
Risk factor for HIV: vertical	4/80 (5%)	1/61 (1.6%)	3/19 (15.7%)	0.03
Risk factor for HIV: drugs of abuse IV	10/80 (12.5%)	9/61 (14.7%)	1/19 (5.2%)	0.43
Risk factor for HIV: sex	66/80 (82.5%)	51/61 (83.6%)	15/19 (78.9%)	0.73
Smoking	27/80 (33.7%)	24/61 (39.3%)	3/19 (15.7%)	0.09
HCV co‐infection	10/80 (12.5%)	9/61 (14.7%)	1/19 (5.2%)	0.43
Mean years of HIV infection	6.5 (0−30)	5.6 (0−19)	9.9 (0−30)	0.34
CD4 nadir (median) cells/mmc	295 (6−886)	302.9 (6−886)	263.3 (12−557)	0.76
HIV‐RNA zenith (median) cp/ml	209,453	21,231	935,452	<0.0001
CD4 I trimester (median) cells/mmc	643.3	517.87 (193−1142)	1244.8 (485−6646)	0.01
HIV‐RNA I trimester (cp/ml)	5160.5	6139 (0−113,719)	0 (0−0)	0.01
HIV‐RNA upon delivery (cp/ml)	1556	1498	1732	0.06
CD4^+^ upon delivery (cells/mmc)	608	585	694	0.64
AIDS diagnosis, CDC stage 3, *n* (%)	11 (13.7%)	7 (11.4%)	4 (21%)	0.28

**Conclusions**: Our findings demonstrate that more women (21%) are diagnosed late in the 2017–2023 period; however, modern ART conferred excellent virological control, with all WLWH achieving viral suppression by first trimester and thus achieving zero MTCT.

### P008 A multicentre observational study to determine the safety and effectiveness of dolutegravir (DTG) use during pregnancy: data from DOLOMITE‐NEAT ID Network study

Justyna Kowalska ^1^, Sergii Antoniak^2^, Lambert Assoumou^3^, Anton Pozniak^4^, Tetiana Rybak^5^, Stephane De Wit^6^, Ana Milinkovic^7^, Sherie Roedling^8^, Carl Fletcher^9^, Leigh Ragone^10^, Claire Thorne^11^, Vani Vannappagari^10^

^1^Hospital for Infectious Diseases, Medical University of Warsaw, Warsaw, Poland, ^2^Viral Hepatitis and AIDS Department, Gromashevsky Institute of Epidemiology and Infectious Diseases, Kyiv, Ukraine, ^3^INSERM, Paris, France, ^4^Chelsea & Westminster Hospital, London, UK, ^5^Odesa Regional Centre of Socially Significant Diseases, Odesa, Ukraine, ^6^CHU Saint‐Pierre, Brussels, Belgium, ^7^Global Medical, ViiV Healthcare, London, UK, ^8^Mortimer Market Centre, London, UK, ^9^Research Organisation (Kings Cross) Ltd, London, UK, ^10^Global Medical, ViiV Healthcare, Durham, NC, USA, ^11^Integrated Screening Outcomes Surveillance Service, UCL Great Ormond Street Institute of Child Health, London, UK

**Background**: Dolutegravir (DTG) is an integrase inhibitor used in combination with other antiretrovirals (ARVs) and is widely recommended during pregnancy for maternal viral suppression and prevention of vertical HIV transmission. This analysis assessed pregnancy and neonatal outcomes of pregnancies in individuals living with HIV using DTG‐based regimens (DBRs) by trimester of earliest exposure.

**Methods**: The analysis uses data from clinical sites participating in the DOLOMITE‐NEAT ID Network study from across Europe. Outcomes assessed include birth defects, live births, stillbirths, birth weight and gestational age at birth.  

**Results**: The analysis included 186 DTG exposed pregnancies resulting in 174 live neonates (including nine sets of twins and one set of triplets), two stillbirths, eight induced abortions, 11 spontaneous abortions and two with unknown pregnancy outcomes. Of 124 (66.7%) individuals exposed to DTG during their first trimester, 104 conceived while on DTG, seven within the first 6 weeks after conception and 13 after 6 weeks. Among 153 live births with gestational age available, 28 (18.3%) were preterm (<37 weeks of gestation) and six (3.9%) were severely preterm (<32 weeks of gestation). Among 152 live births with birth weight available, 28 (18.4%) had low birth weight (<2500 grams) and six (3.9%) had very low birth weight (<1500 grams). A total of 11 birth defects were seen in nine live births with a defect prevalence of 6.3% and one birth defect in a stillbirth but no neural tube defects (NTDs) were reported. A total of five drug‐related adverse events (AEs) and severe adverse events (SAEs) were reported including one serious adverse reaction (SAR). Viral load at delivery was undetectable (<50 copies/ml) among 87.3% of individuals with two reported vertical transmissions resulting in a 1.4% (0.2–5.0) transmission rate.

**Conclusions**: The number of pregnancies is too small to make definitive conclusions. No significant difference in the frequency of birth defects was observed for infants with the earliest exposure in the first compared to the second/third trimester; no NTDs were reported. The results highlight the importance of starting ARV regimen early in pregnancy in order to achieve viral suppression.

### ARV‐based prevention—PEP

### P009 Management of reported side effects to PrEP: high rates of retention on PrEP after switching to a different F/TDF generic brand

Nicolo Girometti ^1^, Frances Lander^1^, Elizabeth Ridsdill Smith^2^, Hasan Mirza^2^, Sheena McCormack^3^, Victoria Tittle^1^

^1^HIV and GU Directorate, 56 Dean Street, Chelsea and Westminster Hospital NHS Foundation Trust, London, UK, ^2^HIV and GU Directorate, Chelsea and Westminster Hospital NHS Foundation Trust, London, UK, ^3^ Clinical Trials Unit, Institute of Clinical Trials & Methodology, University College London, London, UK

**Background**: Gastro‐intestinal side effects are commonly reported among emtricitabine/tenofovir‐disoproxil (F/TDF) PrEP users though these rarely lead to drug discontinuation. Fatigue, headache and less frequently skin hypersensitivity reactions have also been attributed to PrEP intake. We describe the management of individuals reporting non‐bone or renal adverse drug reaction (ADR)‐related discontinuation of PrEP.

**Materials and methods: **A notes review of individuals accessing “PrEP review service” at 56 Dean Street sexual health clinic between January 2022 and May 2024 was performed and those reporting PrEP discontinuation due to non‐renal, non‐bone‐related side effects were included in the descriptive analysis.

**Results**: PrEP discontinuation occurred in 34 individuals: 32/34 MSM, median age 32 years (IQR 29–37 years). Reasons for discontinuation were: 22/34 ≥1 gastro‐intestinal side effects, 12/34 erythematous skin rash, 2/34 fatigue, 3/34 headache and 2/34 arthralgia, with seven individuals reporting ≥1 symptom leading to PrEP interruption. All individuals switched from their initial F/TDF PrEP formulation to an alternate generic. Additionally, 13/34 required supportive medication (i.e. antiemetics, antispasmodics or antidiarrhoeals) to mitigate symptoms and chose to take PrEP with food or crushing it into water or juice to aid administration. Many (14/34) were advised to re‐start PrEP on incremental micro dosing (starting with ¼ TDF/F PrEP dose for 3–5 days, then increasing to ½ dose for 3–5 days and then one full dose, if tolerated), further to condom use if sexually active. Lastly, 3/34 were asked to switch from event‐based to daily PrEP. Of those with available follow‐up, 21/27 (78%) reported symptoms improvement/resolution and remained on TDF/F PrEP. In six cases, switching to another generic formulation of TDF/F did not improve symptoms (four erythematous skin rashes, two gastrointestinal symptoms, one headache). Of these, four were then switched to tenofovir‐alafenamide/F (TAF/F) PrEP, with two tolerating it well and two still manifesting a skin reaction as when on TDF/F. In total, 4/27 are currently off PrEP.

**Conclusions**: Re‐challenging PrEP with an alternate generic F/TDF formulation or F/TAF may help overcome reported side effects or mild skin reactions that previously warranted PrEP interruption. A PrEP review service and an individualized approach to side effect management are valuable assets to support PrEP persistence.

### P010 Analysis of occupational accidents involving exposure to biological fluids in a Portuguese peripheral hospital: implementation and efficacy of post‐exposure prophylaxis

Clara Batista ^1^, Rita Pinto^2^, Frederico Duarte^1^, Ricardo Correia de Abreu^1^, Sofia Jordão^1^

^1^Infectology, Unidade Local de Saúde de Matosinhos, Porto, Portugal, ^2^Occupational Health, Unidade Local de Saúde do Alto Minho, Viana do Castelo, Portugal

**Background**: Occupational accidents (OAs) with biological fluids are a significant concern in Occupational Health due to their high prevalence. Exposure is defined as a “percutaneous injury and/or contact with blood, tissues or other potentially infectious bodily fluids with mucous membranes, broken skin, or extensive areas.” Post‐exposure prophylaxis (PEP) is essential to prevent the risk of infection following exposure [1−3]. This study aims to analyse the occurrence of OAs with biological risk, the implementation of PEP and the associated outcomes in a hospital context.

**Materials and methods**: A descriptive and retrospective observational study of biological risk OAs that occurred between January 2020 and March 2024 at “Unidade Local de Saúde de Matosinhos.” Data were collected from “SClínico Hospitalar” software.

**Results**: Of the 134 cases analysed, 81% were female and 19% were male. The main professional categories involved were Nurses (42%), Operational Assistants (16%), Medical Doctors (12%) and Pharmacists (4%). Regarding patients’ source serologies, 58% were unknown. Of the known serologies, 30% were positive for HIV and 4% were positive for HBV. All workers who underwent PEP started it within the first 72 hours after the accident. From the total workers, 72% started PEP for HIV (63%: tenofovir/emtricitabine and raltegravir; 37%: zidovudine/lamivudine and raltegravir), with 4% needing to change antiretroviral therapy due to intolerance to adverse effects. Of the total workers, 27% did not have protective anti‐HBs antibodies titles therefore started PEP for HBV – from these 83% received the HBV vaccine plus HBIG and 17% received only the HBV vaccine. There were zero infections of HIV, HBV or HCV after 6 months following up [4−6].

**Conclusions**: OAs occurred predominantly among healthcare professionals, probably due to a combination of factors inherent to the hospital environment and the nature of their daily tasks. When necessary, implementing PEP, monitoring adherence to therapy and its adverse effects are crucial in preventing infections. In summary, OAs can have a significant impact on the worker, society and on the economy. Therefore, investing in preventive policies and improving working conditions is essential to reduce their occurrence.

**References**

1. de Lira CRN, de Cassia Akitsu R, de Farias Costa PR, de Oliveira Leite L, Beck da Silva KB, Botelho RBA, et al. Occupational risks in hospitals, quality of life, and quality of work life: a systematic review. Int J Environ Res Public Health. 2021;18(21),11434.

2. Leite E, Galaio L, Almeida C, França D, Ramos D, Tavares J, et al. ACIDENTES DE TRABALHO COM EXPOSIÇÃO A SANGUE E A OUTROS FLUIDOS ORGÂNICOS Recomendações da Sociedade Portuguesa de Medicina do Trabalho. 2017. Available at: https://www.spmtrabalho.org/_files/ugd/a7d6ed_8272a838e7c3426cbcb12a43e4dbd02a.pdf. Accessed Jun 2024.

3. Lei n.° 98/2009 de 4 de Setembro. Diário da República n.° 172 ‐ I Série. Lisboa. Available at: 0589405920.pdf (dre.pt). Accessed Jun 2024.

4. Zachary KC. Management of health care personnel exposed to HIV, UpToDate. Edited by RM Gulick and J Mitty. 2023. Available at: https://www.uptodate.com/contents/management‐of‐health‐care‐personnel‐exposed‐to‐hiv. Accessed Jun 2024.

5. Aberg JA. Management of nonoccupational exposures to HIV and hepatitis B and C in adults, updated 3 April 2024. Available at: https://www.uptodate.com/contents/management‐of‐nonoccupational‐exposures‐to‐hiv‐and‐hepatitis‐b‐and‐c‐in‐adults?search = hepatitis+b+exposure&source = search_result&selectedTitle = 2∼150&usage_type = default&display_rank = 2#H1992516762. Accessed Jun 2024.

6. European AIDS Clinical Society Guidelines v.12.2023. Available at: https://www.eacsociety.org/media/guidelines‐12.0.pdf. Accessed Jun 2024.

### ARV‐based prevention—PrEP

### P011 Prevalence of HIV drug resistance in people newly diagnosed with HIV who have used pre‐exposure prophylaxis in Europe: the PrEPaRe study

Valentina Cambiano^1^, Tina Bruun^2^, Marie Louise Jakobsen ^2^, Ann Sullivan^3^, Andrew Phillips^1^

^1^Institute for Global Health, University College London, London, UK, ^2^Centre of Excellence for Health, Immunity and Infections (CHIP), Region H, Rigshospitalet, Copenhagen, Denmark, ^3^HIV/GUM, Chelsea and Westminster Hospital NHS Foundation Trust, London, UK

**Background**: As of 2021, 22 countries in the World Health Organization European Region had made PrEP available and fully reimbursed. However, people have also been accessing it online, via private healthcare and sourcing it from abroad. We aimed to estimate the proportion of new HIV diagnoses in Europe among people who had used PrEP, the prevalence of HIV drug resistance in those newly diagnosed and before ART initiation, and to explore the circumstances under which infections in PrEP users occurred.

**Methods**: We conducted an observational study collecting aggregated data on total number of HIV diagnoses (Oct 2020–Mar 2024) and individual data (self‐completed questionnaires and clinical referral forms) on adults newly diagnosed with HIV who reported having used PrEP prior to HIV diagnosis at 36 HIV clinics and community‐based testing across 10 European countries.

**Results**: Eighteen sites across nine countries provided aggregated data for 31.75 years of follow‐up; 1725 new HIV diagnoses were recorded. In 89% (*n* = 1529) of cases, clinics had asked about PrEP, and 5% (*n* = 70) had used it. Fifty‐five participants completed the questionnaire: all were men, 89% gay, with a median age of 35 years (range 24–55), 71% of white ethnicity and 91% with at least secondary school education. In 58% of cases, the first HIV‐positive test was at a hospital/clinic as an outpatient and in 18% at a general practitioner and the main reason (60%) was regular testing. Twenty‐three percent (*n* = 13) thought that they acquired HIV while taking PrEP, 13% (*n* = 7) possibly, 51% (*n* = 28) did not think they acquired HIV while on PrEP and seven did not know. Prevalence of major antiretroviral drug resistance mutation (ART‐DRM) to emtricitabine (FTC) or tenofovir (TDF) (specifically K65REN, M184VI, K70E) was 24% (12/50; 95% CI 12–36%) (Table 1). Five individuals thought they acquired HIV while taking PrEP and being fully adherent: three had major ART‐DRMs to FTC/TDF, one did not have any detected and for one the resistance test was not available.

**P011: Table 1:** Resistance among people living with HIV exposed to PrEP

	Prevalence (*n*/*N*)	95% confidence interval
Major antiretroviral drug resistance mutation to FTC or TDF detected with frequency above 15% (K65REN, M184VI, K70E)	24% (12/50)	(12−36%)
M184VI exclusively	10	
K103N exclusively	1	
L10I, 33I, E35D, N37S, R41K, L63T, A71T, I93L, A98S, D123E, E204A, Q207E, R211K, K277R	1	

**Conclusions**: In our study, one in 20 new HIV diagnoses occurred in people who knew about PrEP and had previously used it. The prevalence of resistance aligns with estimates from randomized controlled trials among individuals with acute HIV at enrolment.

### P012 PrEP cascade improvement through same‐day PrEP initiation

Kevin Woodward ^1^, Timothy Guimond^2^, Hommood Alrowais^3^, Victor Monroy^3^, Olivia Gemmell^3^

^1^Infectious Diseases, McMaster University, Hamilton, Canada, ^2^Psychiatry, University of Toronto, Toronto, Canada, ^3^Sexual Health, HQ Health Hub, Toronto, Canada

**Background**: This observational cohort study aims to measure the impact of same‐day PrEP initiation among gay, bisexual and other men who have sex with men (gbMSM) in Ontario. We hypothesize that same‐day initiation will increase PrEP uptake through consolidation of the offer of PrEP, acceptance, screening and initiation.

**Materials and methods**: Individuals at HQ clinic, aged 16 years and older, seeking either sexually transmitted and blood‐borne infections (STBBIs) testing or to start PrEP, and not currently on PrEP, were eligible to participate. PrEP eligibility and identification was determined using a modified HIRI score during intake. Participants underwent HIV testing at baseline by fourth‐generation serological testing after patients accepted PrEP initiation (in contrast with past studies where HIV testing occurred prior to identification) [1]. PrEP offers (TAF/FTC) were made immediately following HIV testing. The primary endpoint was the proportion of eligible PrEP users initiating treatment within 24 hours compared to historical rates within other clinics in Ontario. Secondary endpoints included demographic data analysis, time from HIV test to PrEP initiation, PrEP conversion rate, HIV test positivity and various health metrics including STBBI diagnosis rate, health‐related anxiety, renal safety and PrEP retention at 6 months (defined as receiving a 90‐day supply three or more times in the 6‐month period).

**Results**: Eight hundred and eighty‐seven individuals were offered express testing with rapid PrEP initiation in stage 1 (Figure  1). Thirteen were excluded for testing positive for HIV (1.5% excluded in stage 2). Fifty‐seven individuals did not return to start PrEP within 24 hours from initial screening (6.4% excluded in stage 2). 92.1% of individuals initiated PrEP (*n* = 817) (stages 3 and 4). By 6 months, 178 individuals had stopped PrEP (20.1%), 661 individuals were retained on PrEP (69.4%) and 23 individuals (2.6%) were inconsistently taking their PrEP leading to non‐compliant adherence (stage 5).

**P012: Figure 1:**
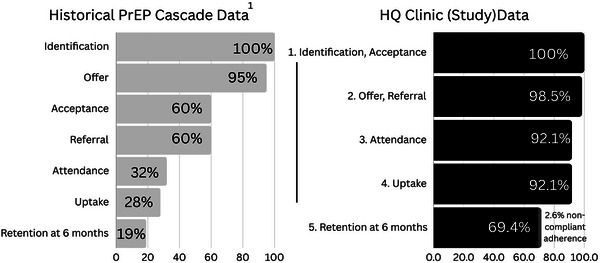
Comparative analysis of historical Ontario and HQ clinic PrEP cascade data.

**Conclusions**: Same‐day initiation of PrEP significantly streamlines the PrEP cascade for high‐risk populations, demonstrating improved uptake and retention rates among gay, bisexual and other men who have sex with men.

**Reference**

1. Pico Espinosa OJ, Hull M, Fisher K, Mohammed S, Tengra Z, Chris A, et al. PrEP cascade among HIV negative gay, bisexual and other men who have sex with men (gbMSM) in Ontario and British Columbia. Baseline data from the PrEP Implementation Project (PRIMP). 18th European AIDS Conference; 2021 Oct 27–30. Abstract PE6/14.

### P013 HIV‐1 prevalence and pre‐exposure prophylaxis uptake among key populations in high‐income economies (2017−2023): a systematic review and meta‐analysis of real‐world studies

Li Tao ^1^, Xiwen Huang^1^, Juan Yang^2^, Jesse Najarro Cermeño^1^, Soodi Navadeh^1^, Julianna Catania^3^, Kylie Scott^4^, Sophie Schoeni^4^, Dylan Mezzio^5^

^1^Real World Evidence Virology, Gilead Sciences, Inc., Foster City, CA, USA, ^2^Real World Evidence Generation, Gilead Sciences, Inc., Foster City, CA, USA, ^3^Evidence Development, Costello Medical, Boston, MA, USA, ^4^ Real‐World Evidence, Costello Medical, Boston, MA, USA, ^5^Global Value and Access, HEOR, Gilead Sciences, Inc., Foster City, CA, USA

**Background**: This systematic literature review (SLR) and meta‐analysis aimed to provide a comprehensive summary of real‐world evidence on HIV‐1 prevalence and pre‐exposure prophylaxis (PrEP) uptake and effectiveness among key populations in World Bank‐defined high‐income economies, excluding the United States and African countries.

**Methods**: A systematic search of Embase, Cochrane Library and PubMed for peer‐reviewed publications of real‐world studies, supplemented with a search of conference publications, was performed in July 2023; a grey literature search was performed in November 2023. The key populations included men who have sex with men (MSM), transgender individuals, people who inject drugs (PWID), people in prison and people with sexual risk behaviours. The review was conducted using a standard two‐reviewer approach in accordance with Preferred Reporting Items for Systematic Reviews and Meta‐Analysis (PRISMA) guidelines, with bias assessed using the modified Joanna Briggs Institute's critical appraisal tool. A third reviewer was consulted if agreements could not be reached. Meta‐analyses, using random effects models, were used to estimate pooled overall HIV‐1 prevalence, PrEP uptake and PrEP effectiveness (HIV‐1 prevalence in PrEP users).

**Results**: A total of 191 real‐world studies from 39 high‐income economies were identified. HIV‐1 prevalence was reported by 127 studies (published in or after 2019); PrEP uptake and PrEP effectiveness were reported by 83 and 25 studies, respectively (published in or after 2017). Among key populations across all included geographies, overall PrEP uptake was 17.7% and HIV‐1 prevalence was 6.7%; HIV‐1 prevalence was 0.2% in PrEP users. Denmark and Portugal had relatively low PrEP uptake (9.7% and 6.0%, respectively) and high overall HIV‐1 prevalence (∼13%) among key populations. Croatia had the lowest PrEP uptake (0.3%) and low HIV‐1 prevalence (1.3%), while the UK had the highest PrEP uptake (32.2%) and low to moderate HIV‐1 prevalence (3.8%) (Figure 1). The pooled prevalence of HIV‐1 in MSM and PWID across all included economies was 8.0% and 6.5%.

**P013: Figure 1:**
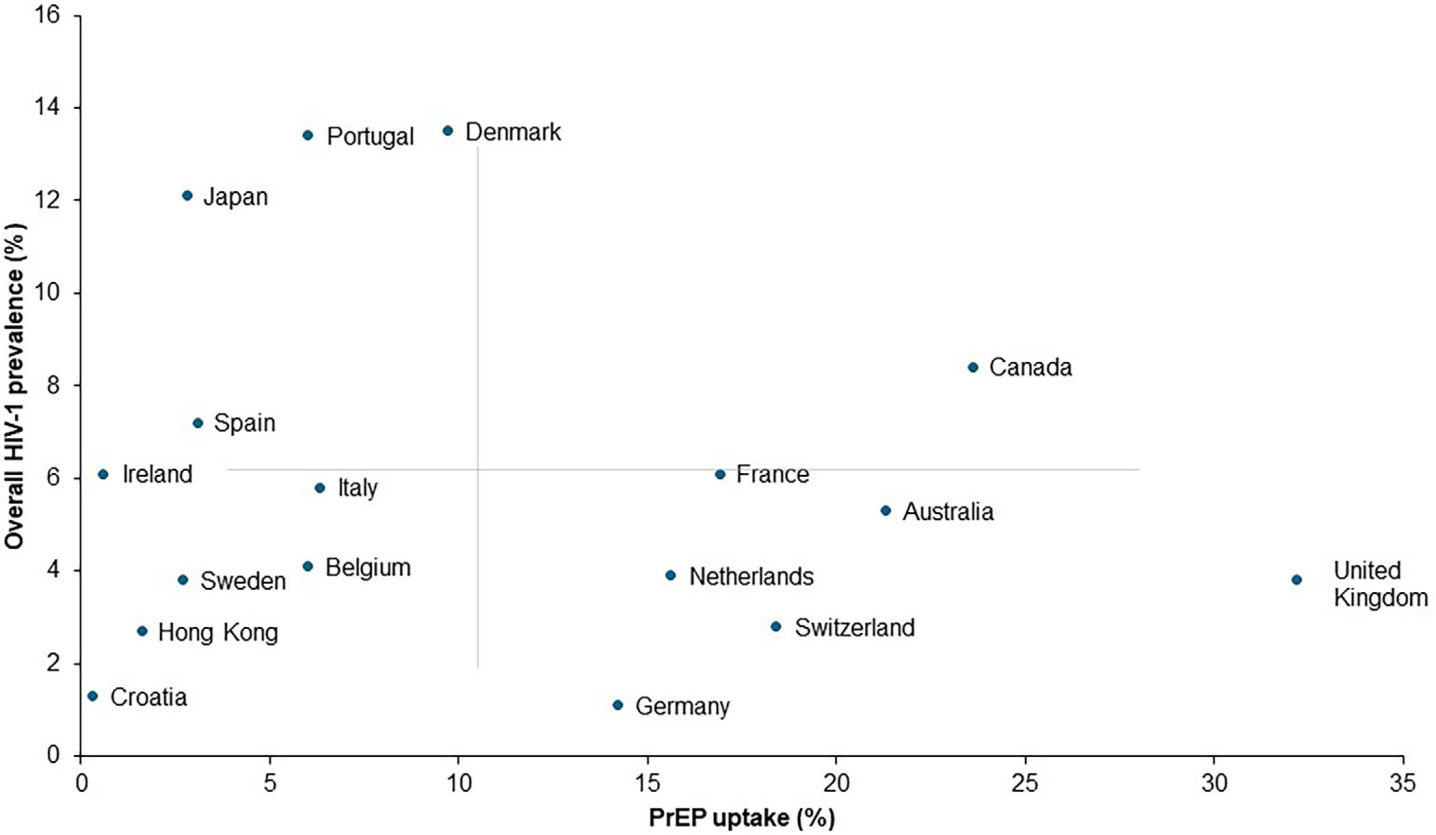
Pooled estimates of overall HIV‐1 prevalence and PrEP uptake among key populations, by economy*. *All World Bank‐defined high‐income economies were included except the United States and African countries to provide a comparable context with healthcare resources and transmission patterns. Out of a total of 39 high‐income economies eligible for meta‐analysis of the SLR, 17 economies with data available for both PrEP uptake and HIV‐1 prevalence in key populations were plotted. The position of each economy represents pooled meta‐analysis estimates for both statistics. The dashed lines represent the economy‐level pooled estimated median overall HIV‐1 prevalence (6.1% for 36 economies) and median PrEP uptake (9.6% for 17 economies). PrEP, pre‐exposure prophylaxis; SLR, systematic literature review.

**Conclusions**: This SLR demonstrates the varied HIV‐1 prevalence between economies and underscores the high effectiveness of PrEP in lowering HIV‐1 rates among key populations in high‐income economies. Disparities in PrEP uptake highlight the need for targeted strategies to provide equitable access to PrEP to strengthen local HIV‐1 prevention initiatives.

### P014 Preparing for long‐acting PrEP delivery: provider preferences for the provision of long‐acting PrEP differ between MSM and heterosexual individuals in Europe

Hanne Zimmermann ^1^, Haoyi Wang^1^, Johann Kolstee^1^, Alejandro Adriaque Lozano^1^, Melanie Schroeder^2^, Ama Appiah^3^, Ana Milinkovic^4^, Supriya Sakar^2^, Kai Jonas^1^

^1^Applied Social Psychology, Maastricht University, Maastricht, Netherlands, ^2^Global Health Outcomes, ViiV Healthcare, Brentford, UK, ^3^Patient Engagement, ViiV Healthcare, Brentford, UK, ^4^Global Medical, ViiV Healthcare & Chelsea and Westminster Hospital, Brentford, London, UK

**Background**: The approval of long‐acting (LA) PrEP in Europe brings opportunities to overcome challenges with oral PrEP, particularly among heterosexual individuals for whom LA‐PrEP showed superior efficacy. However, successful implementation of LA‐PrEP will require tailoring its delivery to the needs of those who could benefit from it. Current PrEP delivery models are predominantly geared towards men who have sex with men (MSM), while data on provider preferences among heterosexual individuals are lacking. Therefore, to support effective LA‐PrEP implementation, provider preferences were explored for the provision of LA‐PrEP among MSM and heterosexual individuals in Europe.

**Materials and methods**: PROTECT, a cross‐sectional survey, was conducted among MSM, heterosexual cis‐men (HM) and cis‐women (HW) in 20 European countries between October 2023 and April 2024. Differences were identified between all groups in socio‐demographic characteristics, LA‐PrEP awareness, interest and intention, and provider preferences for LA‐PrEP.

**Results**: Nineteen thousand, six hundred and ninety HIV‐negative participants were included; 14,730 were MSM, 2547 HM and 2413 HW, with a median age of 37 (IQR 29–47). MSM were older and more highly educated than HW/HM (*p* < 0.001). HW more often reported a migration background and to be struggling on their current income versus MSM and HM (*p* < 0.001). Previous oral PrEP use was reported in 50.9% of MSM, but only in <1% of the heterosexual groups. LA‐PrEP awareness cascades and provider preferences significantly differed between MSM and heterosexual groups. Among MSM, 28.9% were aware of LA‐PrEP, 72.9% expressed interest and 67.9% intention to use LA‐PrEP, while among the heterosexual sample, 3.2% and 2.6% were aware, 12.7% and 11.4% expressed interest and 8.8% and 7.7% high intention to use LA‐PrEP among HM/HW, respectively. LA‐PrEP provider preferences in MSM were most often sexual health clinics (SHCs) (35.1%), general practitioners (GPs) (31.5%) and medical specialists (19.7%). HM/HW predominantly preferred GPs (47.4%; 48.9%) and medical specialists (22.9%; 18.9%), but significantly less often preferred sexual health clinics (17.0%; 19.7%; *p* < 0.001).

**Conclusions**: Our results show that oral PrEP use, LA‐PrEP awareness and provider preferences differ between MSM and heterosexual individuals and suggest an important role for GPs. Simply providing LA‐PrEP through the established SHCs pathways does not alleviate existing access hurdles and may miss access possibilities for HM/HW.

### P015 Intention to use long‐acting PrEP among 4960 heterosexual women and men in 20 European countries: results from the PROTECT survey

Kai Jonas ^1^, Haoyi Wang^1^, Hanne Zimmermann^1^, Alejandro Adriaque Lozano^1^, Melanie Schroeder^2^, Ama Appiah^3^, Ana Milinkovic^4^, Supriya Sakar^2^, Johann Kolstee^1^

^1^Applied Social Psychology, Maastricht University, Maastricht, Netherlands, ^2^Global Health Outcomes, ViiV Healthcare Ltd, Brentford, UK, ^3^Patient Engagement, ViiV Healthcare Ltd, Brentford, UK, ^4^Global Medical, ViiV Healthcare Ltd, Chelsea and Westminster Hospital, Brentford/London, UK

**Background**: Long‐acting injectable pre‐exposure prophylaxis for HIV (LA‐PrEP) is approved for use in Europe and can substantially contribute to HIV prevention by fulfilling unmet needs, also in heterosexual populations that have come back into focus in recent years. To date, no large‐scale comprehensive data is available that investigates interest and intention to use this new modality among heterosexual men and women in Europe.

**Methods**: Data were collected via a cross‐sectional survey from December 2023 to April 2024 among 4960 heterosexual HIV‐negative cis‐females (*n* = 2413) and cis‐males (*n* = 2547) from 20 European countries. Interest and intention to use LA‐PrEP and determinants for intention, including socio‐demographic information, sexual behaviour, sexual health behaviour, substance use and psychosocial indicators, were explored using multivariable logistic regression.

**Results**: Overall, interest and intention to use LA‐PrEP were low (cis‐female 12.7%; 8.8% and cis‐male 11.4%; 7.8%, respectively) but compared with almost negligible rates of current oral PrEP use pointing to substantial unmet needs. Likelihood of the intention to use LA‐PrEP was associated with females reporting: being a student (aOR 1.43; 95% CI 1.01–2.03), being unemployed (aOR 1.71; 1.02–2.75), first‐generation migrant (aOR 1.45; 1.04–2.02), having 2–10 sex partners (aOR 1.71; 1.06–2.74) and perceived HIV concern (aOR 1.65; 1.36–1.98). It was lower among cis‐females reporting: living in a medium‐sized city (aOR 0.56; 0.36–0.98), or village (aOR 0.50; 0.28–0.84), and fear of needles (aOR 0.88; 0.79–0.98). Cis‐male participants reported a higher intention to use LA‐PrEP associated with: being a first‐generation migrant (aOR 2.02; 1.43–2.83), substance use (aOR 1.92; 1.13–3.13), STI testing every 6 months (aOR 13.89; 2.34–269.79), vaginal intercourse in the last 6 months (aOR 1.84; 1.27–2.73), perceived HIV concern (aOR 1.48; 1.17–1.85) and thinking to be at risk for HIV (aOR 1.42; 1.19–1.68). For cis‐men, determinants of a lower likelihood did not turn out significant in the multivariable model.

**Conclusions**: Specific subgroups of heterosexual men and women are interested and intend to use LA‐PrEP if made available. This innovation has the potential to help fulfilling unmet HIV prevention needs in this population. The results can inform tailoring of access and public health policies and messaging.

### P016 Low uptake of mpox and HPV vaccination among PrEP users in Switzerland

Benjamin Hampel ^1^, Andrea Farnham^1^, Celine Capelli^1^, Enos Bernasconi^2^, Dominique L. Braun^3^, Alexandra Calmy^4^, Julia Notter^5^, Marcel Stoeckle^6^, Bernard Surial^7^, Vanessa Christinet^8^, Katharine E. A. Darling^9^, Manuela Rasi^1^, Claudia Bernardini^10^, David Haerry^11^, Florian Vock^12^, Nicola Low^13^, Jan S. Fehr^1^

^1^Department of Public and Global Health, Epidemiology, Biostatistics and Prevention Institute, University of Zurich, Zurich, Switzerland, ^2^Division of Infectious Diseases, Ente Ospedialiero Cantonale, University of Geneva and University of Southern Switzerland, Lugano, Switzerland, ^3^Department of Infectious Diseases and Hospital Epidemiology, University Hospital Zurich, Zurich, Switzerland, ^4^HIV Unit, Division of Infectious Diseases, Geneva University Hospital, Geneva, Switzerland, ^5^Division of Infectious Diseases and Hospital Epidemiology, Cantonal Hospital St. Gallen, St. Gallen, Switzerland, ^6^Division of Infectious Diseases and Hospital Epidemiology, University Hospital Basel, Basel, Switzerland, ^7^Department of Infectious Diseases, Inselspital, Bern University Hospital, Bern, Switzerland, ^8^Checkpoint Vaud, Profa, Lausanne, Switzerland, ^9^Infectious Diseases Service, Lausanne University Hospital and University of Lausanne, Lausanne, Switzerland, ^10^Centre for Addiction Medicine, Arud, Zurich, Switzerland, ^11^Positive Council, Zurich, Switzerland, ^12^Swiss AIDS Federation, Zurich, Switzerland, ^13^Institute of Social and Preventive Medicine, University of Bern, Bern, Switzerland

**Background**: Comprehensive HIV pre‐exposure prophylaxis (PrEP) programmes offer opportunities to promote the uptake of key vaccinations in at‐risk populations. Within the SwissPrEPared online consultation tool, healthcare professionals are reminded to check for vaccination status at every PrEP visit. In this study, we evaluate vaccination uptake within the SwissPrEPared study population for hepatitis A (HAV), hepatitis B (HBV), mpox and human papillomavirus (HPV).

**Materials and methods**: By June 2024, 7292 people were enrolled in the SwissPrEPared study and had at least one visit. Visits are recommended every 3–6 months. We analysed data on vaccination status at baseline and after five and eight visits for HAV, HBV and HPV. For mpox, we calculated the vaccination uptake since the introduction of the vaccine in Switzerland in November 2022. For HPV, we included only participants under the age of 27 at baseline, as the vaccine is only recommended until this age for men in Switzerland.

**Results**: Data about HAV and HBV vaccination status were available for 6456 participants at baseline, 4065 at visit 5 and 2609 at visit 8. The proportion of participants with a complete HAV vaccination status increased from 54% (3502) at baseline to 65% (2901) at visit 5 and 83% (2092) at visit 8. Complete HBV vaccination status increased from 65% (4179) at baseline to 82% (3331) at visit 5 and 88% (2285) at visit 8. Data about HPV vaccination status was available from 918 participants under the age of 27 at baseline, 456 at visit 5 and 230 at visit 8. Complete HPV vaccination status increased from 26% (242) at baseline to 47% (214) and 57% (132) at visit 8. Of 5878 participants with at least one visit since November 2022 and no diagnosis of mpox, 2972 (51%) received at least one mpox vaccine dose.

**Conclusions**: Proportions of cohort participants with complete vaccination status increased over time within the study population. Completed vaccination was lowest for HPV and mpox. More research is needed to understand programme‐, physician‐ and participant‐related reasons for suboptimal vaccination and to develop measures to improve vaccine uptake in this population.

### P017 Recurrence of sexually transmitted infections is commonly found in a sub‐population of Austrian PrEP users

Nikolaus Urban ^1^, Thomas Neidhart^1^, Katharina Grabmeier‐Pfistershammer^1^, Kaspar Laurenz Schmidt^1^, Veronique Touzeau‐Roemer^1^, Nathalie Judith Auer^2^, Robert Strassl^3^, Wolfgang Weninger^1^, Birgit Willinger^4^, Wolfgang Michael Bauer^1^, David Chromy^1^

^1^Department of Dermatology, Medical University of Vienna, Vienna, Austria, ^2^Department of Dermatology and Venereology, University Hospital Essen, University Duisburg‐Essen, Essen, Germany, ^3^Department for Clinical Virology, Department of Laboratory Medicine, Medical University of Vienna, Vienna, Austria, ^4^Department for Clinical Microbiology, Department of Laboratory Medicine, Medical University of Vienna, Vienna, Austria

**Background**: In recent years, a rise in sexually transmitted infections (STIs) has been observed among men who have sex with men (MSM) using HIV pre‐exposure prophylaxis (PrEP). Additionally, “chemsex” (sexualized drug use) has been linked to increasing STI rates in numerous studies. In Austria, however, data on behavioural trends and STI incidence in PrEP users is scarce and hinders prevention strategies. To address this gap, we aimed to assess the incidence of bacterial STIs and associated risk factors among Austrian PrEP users.

**Materials and methods**: In June 2020, we initiated a prospective observational cohort study at the General Hospital of Vienna, comprising Viennese PrEP users. Participants underwent quarterly STI testing and completed questionnaires on sexual behaviour and substance use.

**Results**: Between 06/2020 and 12/2023, 360 individuals (99% MSM) were enrolled. Two hundred and thirty‐six participants with available follow‐up covered 379 person‐years. We detected 276 STIs in 154 individuals, with symptomatic infection in 23% (36/154). Incidence rates for gonorrhoea, chlamydia and syphilis were 29.9 (95% CI 24.3–35.3), 22.7 (95% CI 17.9–27.5) and 9.8 (95% CI –6.6 to 12.9) infections per 100 person‐years, respectively. Extra‐genital infections accounted for 95% (97/102) of gonorrhoea and 81% (65/80) of chlamydia cases. No HIV‐ or hepatitis C virus infections were detected. Notably, individuals with one or more reinfections (18%; 65/360) comprised 68% (187/276) of all STIs. “Chemsex” was reported by 44% (157/360) of participants and was associated with higher rates of gonorrhoea (38% vs. 21%, *p* < 0.001) and syphilis (17% vs. 5%, *p* < 0.001) but not chlamydia (26% vs. 19%, *p* = 0.118). A binary logistic regression model identified “chemsex” as the sole predictor of STI reinfection in multivariate analysis (adjusted odds ratio 3.31 [95% CI 1.67–6.65]; *p* < 0.001).

**Conclusions**: Throughout the study, 43% were affected by a bacterial STI, mostly with asymptomatic and extra‐genital infections. Importantly, 18% of PrEP users had multiple infections, accounting for two‐thirds of the overall number of STIs. “Chemsex” was highly prevalent in our study participants and identified as the only independent predictor for STI reinfection, highlighting benefits of harm reduction strategies in combating STIs.

### P018 Effects of bariatric surgery on intracellular tenofovir‐diphosphate levels in patients taking HIV pre‐exposure prophylaxis

Matthew McGarrity ^1^, Paul MacPherson^2^, Abby Li^1^, Mark Naccarato^3^, Peter Anderson^4^, Darrell Tan^1^

^1^Division of Infectious Diseases, St. Michael's Hospital, Toronto, Canada, ^2^Division of Infectious Diseases, The Ottawa Hospital, Ottawa, Canada, ^3^Department of Pharmacy, St. Michael's Hospital, Toronto, Canada, ^4^Department of Pharmaceutical Sciences, University of Colorado Anschutz Medical Campus, Aurora, CO, USA

**Background: **While bypassing/resecting portions of the gastrointestinal tract, bariatric surgery may influence the pharmacokinetics of orally administered medications. We measured trough concentrations (C_trough_) of tenofovir disphosphate (TFV‐DP) among individuals taking tenofovir disoproxil fumarate plus emtricitabine (TDF/FTC) or tenofovir alafenamide plus emtricitabine (TAF/FTC) as pre‐exposure prophylaxis (PrEP) who were scheduled to undergo/already underwent bariatric surgery.

**Materials and methods: **We enrolled HIV‐negative TDF/FTC or TAF/FTC PrEP users attending clinics in Toronto or Ottawa who were undergoing/underwent bariatric surgery. Dried blood spots were collected immediately before participants administered their next daily dose of PrEP, following at least 7 consecutive days of dosing. TFV‐DP C_trough_ was measured by liquid chromatography tandem mass spectrometry. Participants who had already undergone bariatric surgery provided samples at baseline only. One participant undergoing planned bariatric surgery provided prospectively collected specimens pre‐operatively and on post‐operative days 7, 28 and 84 (±3). We compared results against the expected TFV‐DP C_trough _at varying degrees of adherence (seven doses/week corresponds to ≥1450 fmol/punch for TDF/FTC and ≥2000 fmol/punch for TAF/FTC).

**Results**: Of seven eligible participants, six self‐identified as white and one as Indigenous and white. All were gay, cis‐gender men. Median age was 48 years (Q1 44, Q3 51). Six participants previously underwent bariatric surgery before enrolment; four received Roux‐en‐Y gastric bypass (RYGB) and two received sleeve gastrectomy. Of these six, four were taking TDF/FTC and two were taking TAF/FTC. All had expected TFV‐DP C_trough_ except for one TDF/FTC participant who underwent sleeve gastrectomy (Figure 1). One participant taking TAF/FTC enrolled in the study before receiving RYGB and displayed a modest decrease in TFV‐DP C_trough_ over time, although all values remained in the expected range at days 7, 28 and 84.

**P018: Figure 1:**
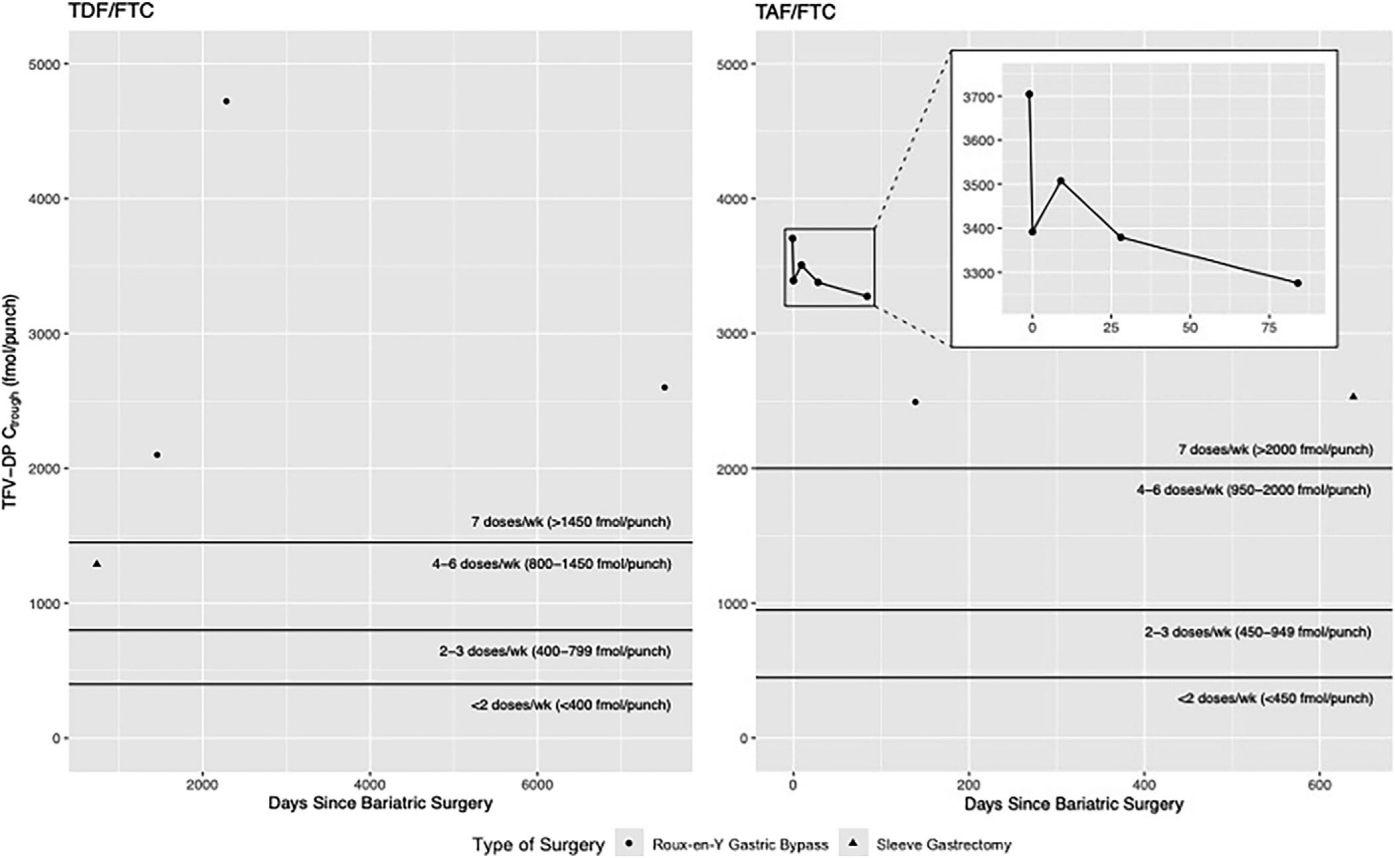
Trough concentrations (C_trough_) of tenofovir disphosphate (TFV‐DP) in dried blood spot samples from HIV pre‐exposure prophylaxis (PrEP) users taking either tenofovir disoproxil fumarate plus emtricitabine (TDF/FTC) or tenofovir alafenamide plus emtricitabine (TAF/FTC) with expected reference ranges, stratified by type of bariatric surgery.

**Conclusions**: Dried blood spots (DBS) concentrations of tenofovir diphosphate were at or near expected levels in this small sample of men using oral PrEP who underwent RYGB or sleeve gastrectomy.

### P019 Barriers and facilitators to pre‐exposure prophylaxis (PrEP) uptake in England: experiences of people newly diagnosed with recently acquired HIV

Carina Hoerst ^1^, Hannah Kitt^1^, Dolores Mullen^1^, Helen Corkin^1^, Clare Humphreys^2^, Ammi Shah^1^, Vicky Gilbert^3^, Adamma Aghaizu^1^

^1^Blood Safety, Hepatitis, STI & HIV Division (BSHSH), UK Health Security Agency, London, UK, ^2^Health Protection, UK Health Security Agency, Chiltern, UK, ^3^Blood Safety, Hepatitis, STI & HIV Division (BSHSH), Public Health England (retired), London, UK

**Background**: In 2022, 3805 people were newly diagnosed with HIV in England. While improvements have been achieved among gay and bisexual men who have sex with men and people of white ethnicity, smaller declines and even increases in diagnoses were recorded for women exposed through sex with men and individuals from black African, Asian or mixed communities.

**Methods**: Working towards the English governments target of ending HIV transmission by 2030 and reducing inequalities in HIV prevention uptake, we conducted 26 semi‐structured in‐depth interviews with people newly diagnosed with recently acquired HIV to identify barriers and facilitators to using or considering pre‐exposure prophylaxis (PrEP). Data was analysed thematically; first deductively following the COM‐B model (exploring Capability, Opportunity and Motivation to engage with the target behaviours), then inductively within each domain.

**Results**: Barriers to using or considering PrEP were knowledge gaps around HIV (including outdated knowledge), knowledge gaps around PrEP and event‐based PrEP; poor visibility of HIV prevention campaigns, low HIV risk perception, reassurance or “trust” in sexual partners and stigma (internalized, anticipated and experienced both within sexual healthcare settings by clinic staff and wider societal). Further barriers were lack of identification of PrEP need by sexual health service staff and fear of side effects. PrEP facilitators were in most instances the inverse of the barriers identified, for example perceiving HIV prevention a social norm, having heard about PrEP from others and different outlets.

**Conclusions**:  Awareness, interactions, access and beliefs about applicability seemed to explain levels of engagement with PrEP and HIV prevention more broadly. These insights can inform behavioural interventions to contribute to decreasing new HIV diagnoses.

### P020 Changing unmet HIV PrEP need among men who have sex with men in London: an analysis of community cross‐sectional surveys prior to and following routine HIV PrEP implementation in England

Flavien Coukan ^1^, Dana Ogaz^2^, Gary Murphy^3^, Hamish Mohammed^2^, John Saunders^2^, Fiona Burns^1^

^1^Institute for Global Health, University College London, London, UK, ^2^Blood Safety, Hepatitis, STI and HIV Division, UK Health Security Agency, London, UK, ^3^National Infection Service Laboratories, UK Health Security Agency, London, UK

In England, HIV pre‐exposure prophylaxis (PrEP) availability was restricted to participants of the PrEP Impact Trial Oct 2017 to Oct 2020, when PrEP was routinely implemented in sexual health services. Using data from the Gay Men's Sexual Health Survey, a serial, anonymized, cross‐sectional, self‐administered survey in London commercial venues (e.g. clubs, bars, saunas) of gay, bisexual and other men who have sex with men (MSM), we describe PrEP use and unmet need before and during the period of routine implementation. From Jun 2019 to Aug 2019 and Nov 2022 to Jan 2023, MSM aged ≥18 recruited from London commercial venues were asked to self‐complete a sexual health questionnaire. All cis‐ and transgender MSM were eligible for participation and inclusion in the subsequent analysis. The survey collected information on the participants’ socio‐demographic characteristics, service engagement and outcomes, along with sexual and prevention behaviours. We performed descriptive analyses investigating current PrEP use and unmet PrEP need in MSM who reported having negative/unknown HIV status. PrEP need was defined as condomless anal intercourse (CAI) in the last 3 months, or CAI in the last year with an HIV‐positive/unknown status partner not known to be on HIV treatment, in reflection of UK PrEP guidelines. Of the 1408 surveys included in the 2019 analysis and the 1090 surveys included in the 2022 analysis, the proportion of MSM of self‐perceived HIV‐negative/unknown status who reported current PrEP use increased from 19.7% in 2019 (242/1230) to 43.5% (350/804) post‐commissioning. The proportion of MSM with unmet PrEP need declined from pre‐commissioning levels (68.2% [431/632]) to (44.3% [212/479]) (Table 1). Those of Asian and Latin American ethnicity accounted for an increasing proportion of MSM with an unmet PrEP need in 2022 (5.2% and 10.4%, respectively) compared to 2019 (3.3% and 3.9%). Following routine commissioning of PrEP in England, the proportion of PrEP‐using MSM increased over two‐fold. However, the proportion of men with PrEP need remained high in a population that still accounts for a large proportion of new HIV diagnoses. Equitable access to PrEP in underserved sub‐populations is necessary to achieve the Government's goal to end new HIV transmission in the UK by 2030.

**P020: Table 1:** Socio‐demographic characteristics in (a) MSM of HIV‐negative/unknown status with a PrEP need and (b) those with unmet PrEP need

	(a) MSM with PrEP need		(b) MSM with unmet PrEP need	
Characteristics	2019 (*N* = 632) *n* (%)	2022 (*N* = 479) *n* (%)	2019 (*N* = 431) *n* (%)	2022 (*N* = 212) *n* (%)
Age				
18−24	80 (12.7%)	40 (8.4%)	64 (14.9%)	23 (10.9%)
25−29	141 (22.3%)	94 (19.6%)	92 (21.4%)	42 (19.8%)
30−34	137 (21.7%)	121 (25.3%)	96 (22.3%)	49 (23.1%)
35−39	79 (12.5%)	82 (17.1%)	50 (11.6%)	34 (16.0%)
40−44	89 (14.1%)	47 (9.8%)	47 (10.9%)	16 (7.6%)
45+	103 (16.3%)	87 (18.2%)	79 (18.3%)	44 (20.8%)
Ethnicity				
White	485 (76.7%)	343 (71.6%)	334 (77.5%)	145 (68.4%)
Black	21 (3.3%)	13 (2.7%)	16 (3.7%)	6 (2.8%)
South East Asian	13 (2.1%)	11 (2.3%)	8 (1.9%)	4 (1.9%)
Asian	25 (4.0%)	22 (4.6%)	14 (3.3%)	11 (5.2%)
Latin American	27 (4.3%)	48 (10.0%)	17 (3.9%)	22 (10.4%)
Mixed	61 (9.7%)	41 (8.6%)	42 (9.7%)	23 (10.9%)
Country of birth				
Born in the UK	328 (51.9%)	246 (51.4%)	230 (53.4%)	114 (53.8%)
Not UK‐born	298 (47.2%)	225 (47.0%)	197 (45.7%)	96 (45.3%)
Residence				
London	511 (80.9%)	381 (79.5%)	343 (79.6%)	158 (74.5%)
Outside London	74 (11.7%)	51 (10.7%)	57 (13.2%)	26 (12.3%)
Outside UK	33 (5.2%)	46 (9.6%)	21 (4.9%)	27 (12.7%)
Current employment				
No	67 (10.6%)	40 (8.4%)	50 (11.6%)	23 (10.9%)
Yes	564 (89.2%)	432 (90.2%)	381 (88.4%)	186 (87.7%)
Education since age 16				
0−2 years	94 (14.9%)	66 (13.8%)	73 (16.9%)	29 (13.7%)
≥2 years/still full‐time	528 (83.5%)	411 (85.8%)	350 (81.2%)	181 (85.4%)

### P021 Chlamydia trachomatis serovars involved in lymphogranuloma venereum infections of men who have sex with men (MSM) and transgender women (TGW) in Buenos Aires, Argentina

Luciana Spadaccini ^1^, Martin Vacchino^2^, Maria Ines Figueroa^1^, Gissella Mernies^1^, Agustin Nava^1^, Carolina Perez^1^, Diego Salusso^1^, Maria Victoria Iannantuono^1^, Jonathan Garcia^1^, Carina Cesar^1^, Valeria Fink^1^, Zulma Ortiz^1^, Patricia Galarza^2^, Pedro Cahn^1^

^1^Research Department, Fundación Huésped, Buenos Aires, Argentina, ^2^ITS Laboratory, ANLIS Malbran Institute, Buenos Aires, Argentina

**Background**: In Argentina, the first reports of lymphogranuloma venereum (LGV) occurred in 2018. Since then, new cases have occurred mainly in MSM living with HIV. This study aims to describe the socio‐demographic, clinical characteristics and serovars involved in LGV infections among MSM and TGW in Buenos Aires, Argentina.

**Materials and methods**: Retrospective review of the LGV cases assisted at a research site that provides HIV prevention and treatment services in Buenos Aires, Argentina (March 2019–November 2023). LGV diagnosis was made in the first void of urine, rectal and/or ulcer swabs by sequencing a fragment of the ompA gene among samples with previous positive Chlamydia trachomatis PCR. We collected data regarding age, gender, educational level, HIV pre‐exposure prophylaxis (PrEP) use, concomitant sexually transmitted infections (STIs) and substance use at the time of LGV diagnosis.

**Results**: Twenty‐eight cases of LGV were diagnosed in the study period: 25 (89%) among MSM and three (11%) in TGW, median age was 33 years (IQR 27.8–42): 76% completed tertiary education, 52% reported substance use (mainly poppers and cannabis). Regarding concomitant STIs, eight had HIV diagnosis (4/8 recent diagnosis), four had syphilis, four gonorrhoea, five resolved HBV infection and one active HCV. Among HIV negative (20, 71%), 95% were on PrEP. Anatomic location was rectal (93%) and genital (7%); 93% were symptomatic. We detected three serovars: L1 (46.4%), L2b (17.9%) and L2 (35.7%). We found a statistical association between serovars and type of population, HIV status and anatomic location (see Table 1). Serovar L1 was most frequently found in HIV‐negative MSM and serovar L2b was the only serovar present in genital LGV.

**P021: Table 1:** Associations between serovars and variables

Variable/serovar	Overall, *n* = 28	L2b, *n* = 5	L2, *n* = 10	L1, *n* = 13	*p*‐value^a^
Population					0.034
MSM	25 (89%)	3 (60%)	9 (90%)	13 (100%)	
TGW	3 (11%)	2 (40%)	1 (10%)	0 (0%)	
HIV status					0.007
Negative	20 (71%)	1 (20%)	7 (70%)	12 (92%)	
People living with HIV	8 (29%)	4 (80%)	3 (30%)	1 (8%)	
PrEP	19 (68%)	1 (20%)	7 (70%)	11 (85%)	0.036
LGV localization					0.026
Rectal	26 (93%)	3 (60%)	10 (100%)	13 (100%)	
Genital	2 (7.1%)	2 (40%)	0 (0%)	0 (0%)	

^a^Fisher's exact test.

**Conclusions**: In our population, most LGV were diagnosed in PrEP users, among these L1 was the prevalent serovar. L2b was more frequent in people living with HIV and in genital location. Rectal involvement was the most frequent location among all serovars. Clinical suspicion and regular screening among PrEP users is important to avoid delay in diagnosis, prevent complications and stop transmission.

### P022 HIV pre‐exposure prophylaxis (PrEP) failures in a large observational cohort from Poland with expansion of A6 subtype transmissions

Bartosz Szetela ^1^, Karol Serwin^2^, Aleksander Zinczuk^3^, Lukasz Lapinski^4^, Aleksandra Szymczak^1^, Kamila Zielinska^3^, Mateusz Bozejko^1^, Anna Urbanska^2^, Jacek Gasiorowski^3^, Milosz Parczewski^2^

^1^Infectious Diseases, Liver Disease and Acquired Immune Deficiencies, Wroclaw Medical University, Wroclaw, Poland, ^2^Infectious, Tropical Diseases and Acquired Immunodeficiency, Pomeranian Medical University, Szczecin, Poland, ^3^All Saints Clinic, Wroclawskie Centrum Zdrowia, Wroclaw, Poland, ^4^Veterinary Medicine, Wroclaw University of Environmental and Life Sciences, Wroclaw, Poland

**Background**: PrEP efficacy relies on adherence and appropriate dosing [1–4]. As data on real‐life PrEP rollout and efficacy from Central and Eastern Europe are scarce, we explored PrEP failures in observational cohort and analysed phylodynamic information among users infected with A6 subtype [5].

**Materials and methods**: A cohort of 887 men having sex with men (MSM) on generic emtricitabine/tenofovir disoproxil (FTC/TDF) was followed for cumulative 2587 person‐years (PY). In newly infected users, acquisition of HIV was confirmed using molecular and immunoassays, with subtype, and genotyping performed at diagnosis. For phylogenetic analysis, we used 2087 HIV‐1 sub‐subtype A6 partial pol sequences from Poland as background, with a two‐state asymmetric discrete trait analysis model to infer the geographic node locations.

**Results**: Nine (1.01%) PrEP users acquired HIV infection during the follow‐up with one user diagnosed baseline and excluded from incidence analysis. Estimated HIV incidence was 0.347/100 PY with the relative risk reduction of 90.86%. All users who acquired HIV were cis‐gendered Caucasian MSM (median age 35.5 [IQR 33–37.75] years) with infections due to inadequate PrEP dosing/exposure. Most users (8/9) dosed PrEP on demand but seven skipped doses, while one user dosed TDF/FTC on demand but only prior to receptive sexual contacts. Genotypic data were available for all cases: in five (55.6%) subtype B and in four (44.4%) A6 variant was acquired with no transmitted drug resistance mutations. Phylogenetic analysis revealed that each of the four PLWH with the A6 sub‐subtype belongs to distinct clusters, with no connections to other individuals followed up locally (Figure 1), three of which had distinct regional origins within the country, and one of a foreign ancestry, as confirmed by modelling the probability of at least 0.94. Users were started on tenofovir alafenamide/emtricitabine/bictegravir in the median of 10.5 (IQR 7–21) days from diagnosis with undetectable viral load after median of 63 (IQR 28–84) days.

**P022: Figure 1:**
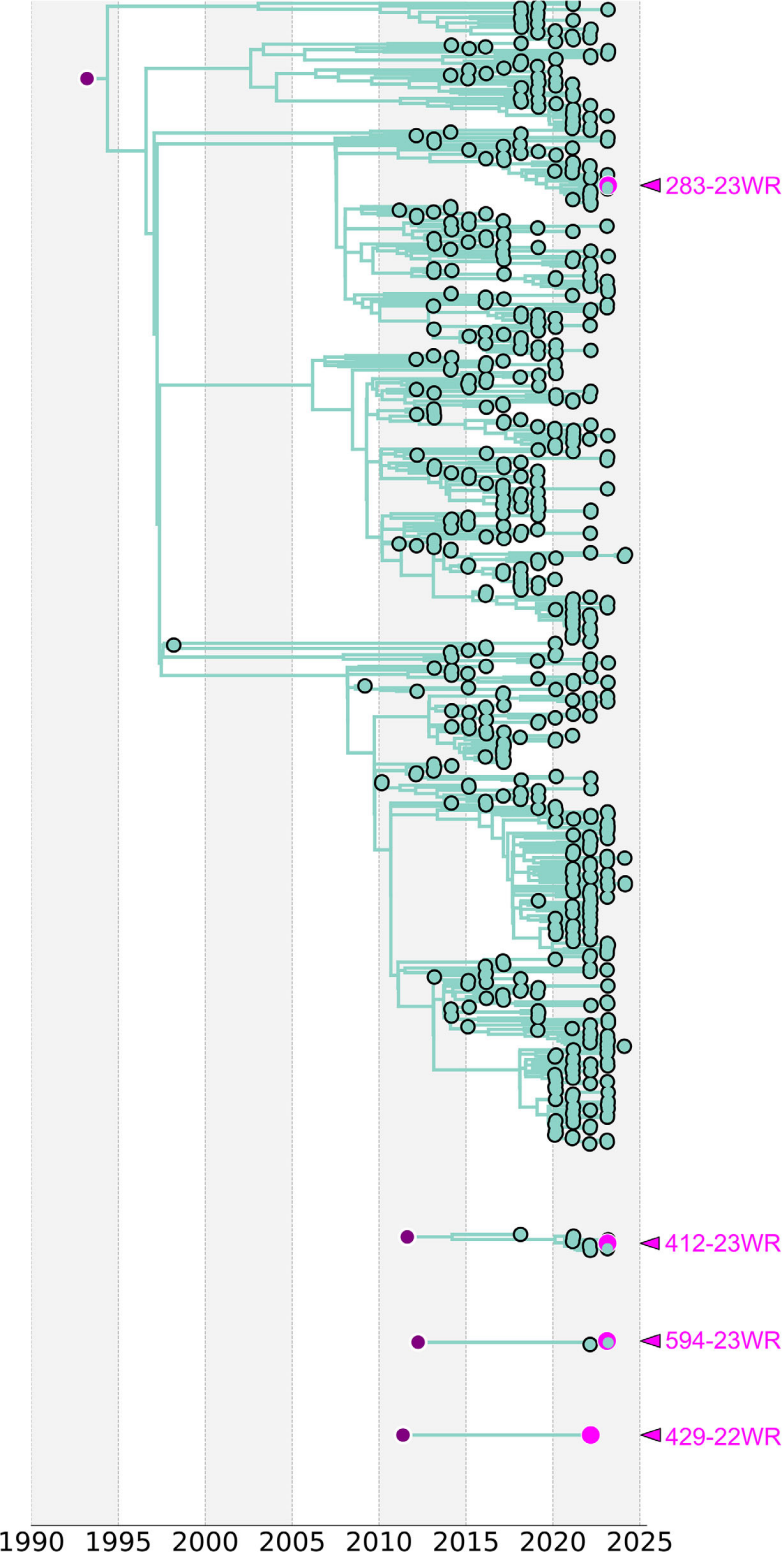
A time‐scaled phylogeographic tree with four different origins of the HIV‐1 A6 acquired by individuals from the Wroclaw All Saints cohort; based on phylogeographic analysis of 2087 Polish A6 sequences and = 11,955 partial pol sequences worldwide. One individual (283‐23WR) is part of the largest, long‐standing cluster of the Polish A6 epidemic. The following two individuals (594‐23WR and 412‐23WR) belong to smaller clusters containing three and 10 sequences, respectively. The last individual (429‐22WR) represents a new introduction to Poland. Purple circles represent non‐Polish ancestral nodes, while magenta circles denote A6 sub‐subtype individuals from the Wroclaw All Saints cohort. Green leaves indicate other A6 Polish sequences.

**Conclusions**: TDF/FTC PrEP was highly efficacious with the majority of failures related to suboptimal adherence. However, quick suppression of HIV replication was achieved with cART. Despite the lack of transmitted drug resistance, we note a higher number of infections with A6 subtype and independent lineage.

**References**

1. European AIDS Clinical Society. EACS Guidelines, version 12 [Internet]. 2023 [cited 2024 May 7]. Available from: https://www.eacsociety.org/guidelines/eacs‐guidelines/eacs‐guidelines.html.

2. Parczewski M, Witak‐Jędra M, Aksak‐Wąs B. Zasady opieki nad osobami zakażonymi HIV. Zalecenia PTN AIDS 2023. Warszawa–Szczecin: Polskie Towarzystwo Naukowe AIDS; 2023.

3. Molina J‐M, Ghosn J, Assoumou L, Delaugerre C, Algarte‐Genin M, Pialoux G, et al. Daily and on‐demand HIV pre‐exposure prophylaxis with emtricitabine and tenofovir disoproxil (ANRS PREVENIR): a prospective observational cohort study. Lancet HIV. 2022;9(8):e554‐62.

4. Girometti N, McCormack S, Tittle V, McOwan A, Whitlock G. Rising rates of recent preexposure prophylaxis exposure among men having sex with men newly diagnosed with HIV: antiviral resistance patterns and treatment outcomes. AIDS. 2022;36(4):561‐6.

5. Serwin K, Chaillon A, Scheibe K, Urbańska A, Aksak‐Wąs B, Ząbek P, et al. Circulation of human immunodeficiency virus 1 A6 variant in the Eastern border of the European union—dynamics of the virus transmissions between Poland and Ukraine. Clin Infect Dis. 2023;76(10):1716‐24.

### P023 Addressing the unmet HIV prevention needs of women who have migrated from sub‐Saharan Africa in France: an urgent call for PrEP integration in family planning centres

Victoria Manda ^1^, Julie Castaneda^2^, Jean Guilleminot^3^, Fati Abdou^4^, Samantha Devlin^5^, Eleanor Friedman^5^, Jessica P. Ridgway^5^, Amy K. Johnson^6^, Geoffroy Liegeon^5^

^1^Infectious Diseases, AP‐HP—Saint Louis Hospital, Paris, France, ^2^Family Planning Center, AP‐HP Lariboisière Hospital, Paris, France, ^3^Family Planning Center, Le Raincy‐Montfermeil Hospitals, Montfermeil, France, ^4^ URACA, BASILIADE Association, Paris, France, ^5^Infectious Diseases and Global Health, The University of Chicago Medicine, Chicago, IL, USA, ^6^Adolescent Medicine, Ann & Robert H. Lurie Children's Hospital of Chicago, Chicago, IL, USA

**Background**: A major HIV prevention gap persists among women who have migrated from sub‐Saharan Africa (WMSSA) in France, who account for ∼20% of new HIV infections but less than 2% of PrEP users. To bridge this gap, we investigated the potential of offering PrEP in WMSSA attending family planning centres (FPC) by evaluating their needs and perceptions towards PrEP.

**Methods**: Trained staff conducted computer‐assisted personal interviewing with adult cisgender WMSSA in two FPCs of the Paris region from 1 January 2023 to 30 June 2024. The 30‐minute questionnaire evaluated factors such as living conditions, sexual behaviours, PrEP awareness, perceived barriers to its use and relation with the FPCs.

**Results**: Seventy‐eight women from 14 SSA countries participated in the study with a median age of 31 years (IQR 26–38), a median time in France of 4 years (IQR 1–7), 94% French‐speaking and 36% reporting literacy difficulties. During the past year, 46% of the women had difficulty in paying for basic needs, 49% had no residence permit, 15% experienced unstable housing, 15% were uninsured, 56% were tested for HIV and 41% had unplanned pregnancies. Eleven women (14%) reported forced sex in France. Among 72 women who had sexual intercourse in the past 6 months, 63% never used condoms, 31% had unknown partner HIV status, 21% had casual partners, 10% had ≥2 partners, 7% engaged in transactional sex and 6% received a sexually transmitted infection treatment, resulting in nearly 45% meeting the standard eligibility criteria for PrEP. Of the 78 women, 88% have never heard about PrEP, and 42% reported that they definitely would like to start it within 6 months. The most commonly perceived barriers to PrEP use were fear of side effects (68%), having to take pills daily (47%), being stigmatized as living with HIV (35%) and safety concerns during pregnancy (33%). Most women were very satisfied with care in FPCs (68%) and identified them as the preferred place to get PrEP (85%).

**Conclusions**: WMSSA attending FPCs have unmet HIV prevention needs, highlighting the urgent need for tailored implementation strategies to integrate PrEP as part of FPC services.

### P024 Determinants of loss to follow‐up among pre‐exposure prophylaxis users at a Portuguese tertiary hospital

Sara Magalhães, Sara Lino, Raquel Pinto, Maria Carlos, Gonçalo Cristóvão, Ana Catarina Gonçalves, João Caria, Vasco Almeida, Claudina Cruz, Marta Leal Santos, Diogo Bento, Stepanka Betkova, Raquel Garrote, Freddy Ramirez, Diana Póvoas, Diana Seixas, Orlando Cardoso, Maria José Manata, Fernando Maltez

Infectious Diseases Department, Unidade Local de Saúde São José, Lisboa, Portugal

**Background**: Pre‐exposure prophylaxis (PrEP), approved in Portugal since June 2017, is a safe and effective tool to prevent HIV infection in high‐risk individuals [1]. Although PrEP prescription and distribution were approved outside hospital settings in December 2023, this has not yet become a reality in Portugal. This presents several barriers to its utilization. Understanding the factors associated with loss to follow‐up in PrEP users stands as fundamental to guide the development of strategies to enhance retention in PrEP care.

**Materials and methods**: In this retrospective observational study, we reviewed the electronic medical records of all referenced individuals who started PrEP between April 2018 and May 2024 at an infectious diseases department in a Portuguese tertiary hospital. Loss to follow‐up (LTFU) was defined as not having had an office/virtual visit within 6 months prior to data collection.

**Results**: A total of 1912 referenced individuals initiated PrEP. The mean age was 34.8 years, and 94.8% of the individuals were cisgender men. Most identified as men who have sex with men (86.9%). In this cohort, 188 individuals were sex workers and 411 were people who used illicit substances during sexual activity (chemsex). The mean follow‐up time was 29 months. The overall cumulative LTFU rate was 22.8%. Factors identified as potentially associated with higher risk of LTFU included: individuals of non‐Portuguese origin (LTFU rate: 25.8% vs. 14.7% in individuals of Portuguese origin); cisgender women (LTFU rate: 41.3% vs. 22.2% in cisgender men); sex workers (LTFU rate: 34% vs. 21.5% in non‐sex workers); and individuals without a sexually transmitted infection (STI) (LTFU rate: 30.1% vs. 16.6% in individuals with an STI). Among those who maintained follow‐up, nine individuals acquired HIV infection. Of those who were lost to follow‐up, 11.5% had appointments for STI screening/diagnosis, 1.4% were awaiting a new PrEP appointment, 1.1% were diagnosed with HIV infection and 0.7% required post‐exposure prophylaxis.

**Conclusions**: Our study is in line with the published evidence [2–4], indicating that PrEP retention is suboptimal, especially in migrants, sex workers and cisgender women, reinforcing the importance of adopting targeted strategies and community‐based PREP care.

**References**

1. McCormack S, Dunn DT, Desai M, Dolling DI, Gafos M, Gilson R, et al. Pre‐exposure prophylaxis to prevent the acquisition of HIV‐1 infection (PROUD): effectiveness results from the pilot phase of a pragmatic open‐label randomised trial. Lancet. 2016;387(10013):53‐60.

2. Mayer KH, Agwu A, Malebranche D. Barriers to the wider use of pre‐exposure prophylaxis in the United States: a narrative review. Adv Ther. 2020;37(5):1778‐811.

3. Herns S, Panwala R, Pfeil A, Sardinha M, Rossi V, Blumenthal J, et al. Predictors of PrEP retention in at risk patients seen at a HIV primary care clinic in San Diego. Int J STD AIDS. 2023;34(11):785‐90.

4. Chan PA, Patel RR, Mena L, Marshall BD, Rose J, Sutten Coats C, et al. Long‐term retention in pre‐exposure prophylaxis care among men who have sex with men and transgender women in the United States. J Int AIDS Soc. 2019;22(8):e25385.

### P025 Evolution from an HIV PrEP clinic to a comprehensive sexual health service in a tertiary university hospital in southeast Asia

Dariusz Olszyna, Tiane Le, Sophia Archuleta

Medicine, National University Hospital, Singapore, Singapore

**Background**: Delivery of sexual health services in some Asian countries where sex, sexual health and sexually transmitted infections are taboos, faces many challenges. In 2016, the first HIV pre‐exposure prophylaxis (PrEP) clinics started in Singapore but had poor uptake of their services.

**Materials and methods**: BePrEP was established in 2016 and initially focused exclusively on providing PrEP to men who have sex with men (MSM). In 2019, with slow uptake of PrEP in this population, we decided to broaden its scope to provide comprehensive sexual health services beyond the MSM population. In socially conservative Singapore where public healthcare institutions cannot directly advertise their services, we employed innovative interventions to increase uptake of our services and expand beyond MSM. These included: recruitment of a programme manager and navigator from key populations, training of a female doctor to cater to female clients, engaging local family physicians, provision of free HIV, syphilis and kidney function tests, implementation of accelerated referral flow from the emergency department and engagement of community groups in educational social media campaigns with linkage to our clinic.

**Results**: Between 2016 and 2018, there was a slow uptake of services in our clinic with only 11–14 new clients a year. In 2019–2022, after the interventions and rebranding of the clinic, it increased to stable 92–114 new clients a year (Figure 1). Retention in clinic increased from 26% in 2019 to 56% in 2022. Percentage of non‐MSM clients increased steadily from 12% in 2019 to 50% in 2022. Number of HIV and syphilis tests per patient per year doubled in this period.

**P025: Figure 1:**
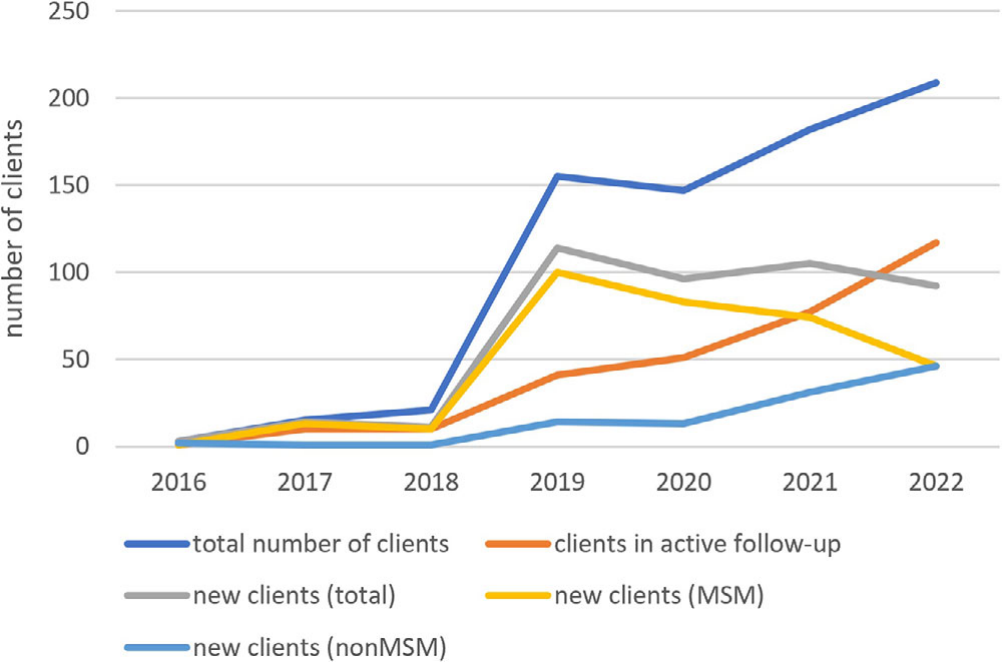
Changes in BePrEP clinic client population after introduction of innovative interventions in 2019. Multiple interventions introduced in our clinic from 2019 on were associated with a marked increase in total number of clients, new clients presenting to clinic every year and non‐MSM clients.

**Conclusions**: We demonstrated how a sexual health/PrEP service can successfully grow, transform and increase its scope through employing innovative solutions based on inclusion of key population staff, collaboration with other healthcare providers as well as community groups.

### P026 HIV incident infections in a PrEP cohort

Isabel Pastor, Guillermo Alberto Viloria, Mariana Angelica Kundro, Marina Alonso Serena, Maria Belen Zorz, Federico Cardozo, Marcelo Horacio Losso

Emergent Diseases, Hospital General de Agudos José María Ramos Mejía, Buenos Aires, Argentina

**Background**: HIV pre‐exposure prophylaxis (PrEP) is an intervention based on the use of antiretroviral drugs (ARV) intended to prevent HIV transmission. The ARV used in PrEP include tenofovir disoproxil fumarate and emtricitabine (TDF‐FTC) [1] or cabotegravir (CAB‐LA) [2]. Resistance‐associated mutations (RAMs) have been observed in people who acquire HIV in the setting of PrEP, requiring a genotype to better choose the antiretroviral therapy regimen (ART) [3]. Our aim was to describe the RAMs and virological outcomes of the HIV incident infections in our cohort of PrEP users.

**Methods**: A retrospective cohort study was performed. The data from clinical records of PrEP users during the period of analysis were reviewed.

**Results**: Between June 2018 and June 2024, 517 subjects started PrEP in ClinSex, a reference sexual health clinic for people at risk in Buenos Aires. The ARV used for PrEP was TDF/FTC in 391 (76%) and CAB in 126 subjects (24%). The median age of clients was 32 years (IQR 27–37) and 99% of them were cisgender MSM. The median days in PrEP was 545 (IQR 318–967). During that period, six (1%) HIV infections were found, four in patients receiving TDF/FTC and two with CAB. In the TDF/FTC PrEP users, 3/4 (75%) reported irregular adherence due to missing doses and intolerance. Both individuals who used CAB reported good adherence. The genotypic resistance testing revealed major RAMs in three of the incident infections: E138K and Q148K in the two CAB PrEP users, K70KE and M184I in one (25%) of the TDF/FTC PrEP users. All the individuals achieved undetectable HIV RNA after starting ART with TDF/FTC/DRV/r, DTG/DRV/r or TDF/3TC/DTG, according to the previous ARV exposure. All regimens were started before receiving the genotypic results. An unexpected genotypic finding (major RAMs NNRTI; K101E and E138K) was detected in one subject without history of NNRTI exposure.

**Conclusions**: Although PrEP is highly effective, infrequent HIV incident infections occur. We found major RAMs to the PrEP regimen used in half of the acute infections in our cohort.

**References**

1. McCormack S, Dunn DT, Desai M, Dolling DI, Gafos M, Gilson R, et al. Pre‐exposure prophylaxis to prevent the acquisition of HIV‐1 infection (PROUD): effectiveness results from the pilot phase of a pragmatic open‐label randomised trial. Lancet. 2016;387(10013):53‐60.

2. World Health Organization. Guidelines on long‐acting injectable cabotegravir for HIV prevention. Geneva: World Health Organization; 2022.

3. Gandhi RT, Bedimo R, Hoy JF, Landovitz RJ, Smith DM, Eaton EF, et al. Antiretroviral drugs for treatment and prevention of HIV infection in adults: 2022 recommendations of the International Antiviral Society‐USA Panel. JAMA. 2023;329(1):63‐84.

### P027 Prevalence of the use of sports supplements and illicit drugs for use in gyms in people included in HIV pre‐exposure prophylaxis programmes (Gym‐PrEP cohort)

Raquel Muñoz‐Andrés^1^, Sara de la Fuente Moral^2^, Natalia Vicente‐López^3^, Ilduara Pintos‐Ramos^3^, Alberto Diaz de Santiago ^2^

^1^Medicine, Universidad Autónoma de Madrid, Madrid, Spain, ^2^HIV‐AIDS Medicine (Internal Medicine), Puerta de Hierro University Hospital, Madrid, Spain, ^3^Internal Medicine, Puerta de Hierro University Hospital, Madrid, Spain

**Background**: The use of exercise‐related products in the gym is widespread in our environment and could be increased among users of HIV pre‐exposure prophylaxis (PrEP‐HIV) programmes, but there is little data in Spain. Additionally, there is a theoretical potential for liver and kidney damage from some of these products. Our primary objective is to measure the prevalence of fitness product use in patients on PrEP‐HIV.

**Materials and methods**: A cross‐sectional study conducted through a telephone survey describes the prevalence of gym product use in 94 people on PrEP‐HIV from May 2022 to August 2023. Data on their baseline creatinine and liver profile, as well as at 6 months and first year, were collected from their electronic medical records. The statistical analyses were performed using the programme Stata v. 12.0 (Texas, USA).

**Results**: One hundred percent were males. The median age was 34 years (IQR 30–42). Median follow‐up period was 16 months (IQR 12–20). Seventy‐nine percent were Spanish and 17% were born in Latin America. 81.91% attend the gym regularly. Among those who go to the gym, 33.77% do between 1 and 4 hours per week, 59.74% do between 5 and 9 hours, and 6.49% (*N* = 5) do between 10 and 15 hours. 53.2% use gym products (64% of those who attend gym regularly). Of these, 8% use illicit drugs for the gym. More hours per week at the gym correlate with a higher prevalence of product use (100% in 10–15 hours per week users). Among sports supplements, the most consumed are protein powder (78%), creatine (68%) and multivitamin complexes (38%) (Table 1). Anabolic androgenic steroids (AAS) were consumed by 8% (IM route 50%, and oral + IM routes: 50%), tamoxifen 4%, growth hormone 2%. We did not find use of thyroid hormone, selective androgen receptor modulators (SARMs), insulin or beta‐human chorionic gonadotropin (B‐HCG). In patients who consume gym products, there has been an increase of 0.047 mg/dl (95% CI 0.017–0.077) in creatinine in the first year.

**P027: Table 1:** Prevalence of consumption of different legal products related to gym exercise among PrEP users

*N* = 50	*N*	%
Protein powder	39	78
Creatine	34	68
Multivitamins	18	36
Vitamin C	18	36
Vitamin B12	17	34
Vitamin B1	13	26
Caffeine	13	26
Beta‐alanine	13	26
Branched‐chain amino acids	10	20
Energy bars	7	14
Omega‐3 fatty acids	7	14
Glutamine	7	14
Melatonin	6	12
L‐carnitine	5	10
Magnesium	4	8
Vitamin B6	4	8
Sports drinks	3	6
Iron	3	6
Ashwagandha	3	6
Vitamin D	2	4
Essential amino acids	1	2
Vitamin E	1	2
Betaine	2	2

*Note*: *N* = 50. Decreasing order.

**Conclusions**: The high prevalence of gym product use in patients on PrEP‐HIV and the possible associated renal toxicity make it necessary to continue researching this population and to promote greater awareness about their use.

### P028 Injectable PrEP is here, who wants it? Early lessons learned from injectable cabotegravir rollout in Zambia

Daniel Mwamba

HIV/Tuberculosis, USAID/Zambia, Lusaka, Zambia

**Background**: In February 2024, Zambia started to offer cabotegravir long‐acting (CAB‐LA) for HIV prevention. One of the prerequisites to the CAB‐LA rollout was to define eligibility criteria for potential clients. This abstract describes the clinical eligibility criteria to receive CAB‐LA and characterize the early CAB‐LA client cohort at United States Agency for International Development (USAID)‐supported health facilities in Zambia.

**Methods**: A CAB‐LA steering committee conducted a desk review of published evidence and developed an initial clinical eligibility criterion for potential clients in Zambia. Due to limited data on CAB‐LA efficacy, the following groups are not eligible: pregnant and breastfeeding women, clients aged <16 years or weighing <35 kg. Criteria 1. Excluding HIV infection by a negative HIV rapid test plus a retrospective negative nucleic acid test (NAT) result; absence of HIV exposure in the last 72 hours and no signs of acute HIV infection in the past 2 weeks confirmed by clinical assessment. Criteria 2. Excluding liver disease and toxicity by assessing drug‐drug interaction (DDI), excessive alcohol intake using the Cut down, Annoyed, Guilty, Eye opener (CAGE) scale; testing for HBsAg and liver enzymes. Criteria 3. Excluding depression and anxiety using the four‐item Patient Health Questionnaire (PHQ4). Clients who visit the health facilities for various health issues were offered cabotegravir and screened for eligibility.

**Results**: Six USAID‐supported sites screened 738 clients from February to May 2024; 641 (87%) started CAB‐LA. Forty‐five were found ineligible due to positive HBsAg, 15 had a positive HIV test, 13 were pregnant, four lactating women, one DDI, 14 excessive alcohol intake and two had psychiatric disorder. Among those who received cabotegravir, 215 (34%) are adolescent boys and young men, 208 (32%) adolescent girls and young women, 27 (4%) female sex workers, 10 (1.5%) men who have sex with men, five (0.7%) transgender, six male in serodifferent relationship and 170 (27%) belong to the general population.

**Conclusions**: The majority of clients screened were eligible for CAB‐LA. There is a high demand for CAB‐LA among adolescent and young people; this could be explained by the discretion of product use and ease of adherence. However, a longitudinal follow‐up of this cohort is needed to ascertain.

### P029 PrEP‐associated stigma in Europe: findings from a real‐world survey

Melanie Schroeder^1^, Fritha Hennessy^2^, Libby Turner^2^, Tim Holbrook^2^, Ama Appiah^3^, Jenny Scherzer ^4^

^1^Health Economics, ViiV Healthcare, Brentford, UK, ^2^Infectious Disease, Adelphi Real World, Bollington, UK, ^3^Europe Patient Affairs, ViiV Healthcare, Brentford, UK, ^4^Global Medical Affairs, ViiV Healthcare, Munich, Germany

**Background**: Oral pre‐exposure prophylaxis (PrEP), used to prevent HIV acquisition, is effective when taken as prescribed. However, barriers to uptake and adherence, such as stigma, remain. We aimed to assess PrEP‐associated stigma and attitudes towards future alternative options.

**Methods**: Data were collected from the Adelphi PrEP Disease Specific Programme™, a real‐world, cross‐sectional survey in four European countries (France, Germany, Italy and Spain; October 2022–July 2023). PrEP users (PU) and individuals not currently receiving PrEP (PrEP non‐users [NU]) answered questions related to PrEP‐associated stigma. Analyses were descriptive.

**Results**: In total, 221 PU and 157 NU (never received PrEP, *n* = 149; previously received PrEP, *n* = 8) completed surveys. Overall, 28% of PU and 46% of NU reported that they currently kept, or would keep, their oral PrEP use a secret (Figure 1). Both PU and NU reported that PrEP use was associated with being HIV positive (PU: 35%; NU: 42%) and “unsafe” sexual practices (PU: 34%; NU: 36%; Table 1). NU were more likely to be impacted by being deemed as at “high risk” of HIV acquisition versus PU (35% vs. 22%). Regarding future PrEP options, 56% of PU and 45% of NU would be “likely”/“very likely” to take an injection for PrEP administered every 8 weeks.

**P029: Figure 1:**
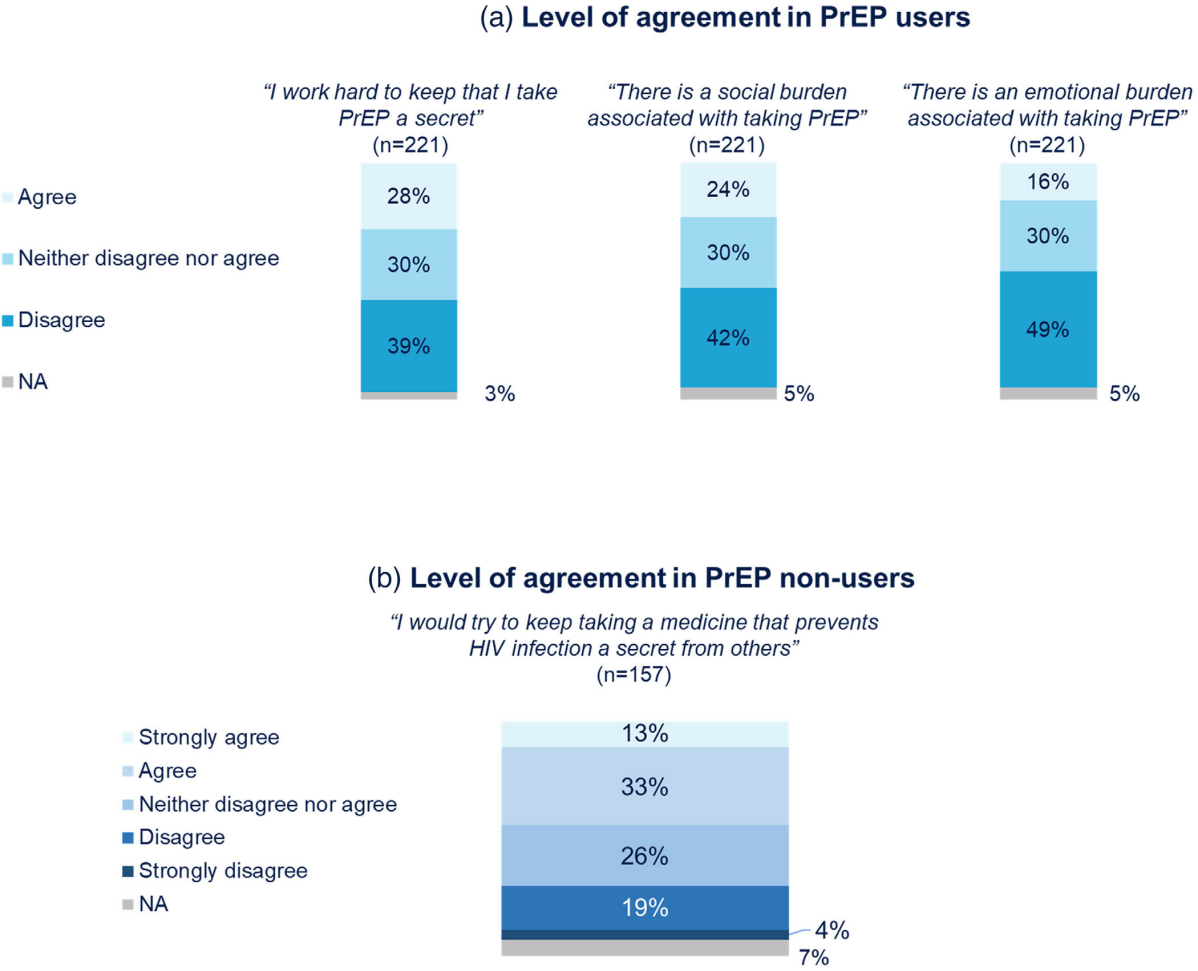
Social and emotional burden associated with PrEP among PrEP users and non‐users*. *Data collected from France, Germany, Italy and Spain. No data from the UK were available. N/A, not available; PrEP, pre‐exposure prophylaxis.

**P029: Table 1:** Factors associated with stigma and factors that affect feelings towards taking PrEP among PrEP users and non‐users^a^

	PrEP non‐users (*n* = 157)	PrEP users (*n* = 221)
“Which factors do you believe could cause people to feel like there is a stigma associated with using PrEP?”^b^		
PrEP use can often be associated with being HIV positive	42%	35%
Taking PrEP can be associated with “unsafe” sexual practices	36%	34%
PrEP is often seen as a medication only taken by gay men	33%	26%
Taking PrEP can be associated with promiscuity	27%	28%
I do not believe there is a stigma associated with using PrEP	20%	23%
PrEP can be perceived as a less “moral” HIV prevention choice	15%	14%
When talking about PrEP, doctors, nurses and other healthcare workers sometimes use words that make patients feel ashamed	9%	7%
“Have your feelings towards taking PrEP ever been impacted/affected by any of the following?”		
Being referred to as at “high risk” of HIV acquisition	35%	22%
No	31%	44%
Concern around friends/family finding out that I take PrEP	30%	16%
Having to report number of sexual partners	24%	15%
Fear of discrimination for taking PrEP	19%	12%
Having to report sexual acts to doctors/nurses	17%	11%
Negative connotations surrounding PrEP use/HIV within my culture	15%	10%
Worried that people would find out/think I was gay if they found out I was taking PrEP	13%	6%
Concerns around managing my relationships when taking PrEP (e.g. telling my partners that I take PrEP)	10%	9%

Abbreviation: PrEP, pre‐exposure prophylaxis.

^a^Only answers reported by >5% were included.

^b^PrEP users were asked “From your experience, which factors do you believe could cause people to feel like there is a stigma associated with using PrEP?” PrEP non‐users were asked “Which factors do you believe could cause people to feel like there is a stigma associated with using a medication to decrease risk of HIV infection?”

**Conclusions**: Stigma due to the association of PrEP with HIV impacts users, including misconceptions regarding sexual lifestyle choices and PU feeling they have to keep PrEP use a secret. Both PU and NU expressed interest in long‐acting alternatives, which may help reduce the burden of stigma.

### P030 Implementation of PrEP in Italy: results of PrIDE survey

Silvia Nozza ^1^, Valentina Mazzotta^2^, Thomas Masoero^3^, Alessandro Tavelli^3^, Filippo Leserri^4^, Lucia Taramasso^5^, Daniele Tesoro^6^, Enrico Tesoro^7^, Antonella d'Arminio Monforte^3^, Francesco Maria Fusco^8^, Marianna Menozzi^9^, Eugenio Milano^10^, Davide Moschese^11^, Roberto Rossotti^12^, Francesco Barbaro^13^, Salvio Cecere^14^, Maddalena Giglia^15^, Serena Venturelli^16^, Massimo Cernuschi^7^, Antonella Castagna^1^, Andrea Antinori^17^

^1^Infectious Diseases, Vita‐Salute San Raffaele University, Milano, Italy, ^2^Clinical and Research Infectious Diseases Department, National Institute for Infectious Diseases, Lazzaro Spallanzani IRCCS, Rome, Italy, ^3^ICONA Foundation, Milan, Italy, ^4^PLUS Roma, Rome, Italy, ^5^Infectious Diseases, IRCCS Ospedale Policlinico San Martino, Genoa, Italy, ^6^ Infectious Diseases, ASST Santi Paolo e Carlo, Milan, Italy, ^7^Milano Checkpoint ETS, Milan, Italy, ^8^Infectious Diseases, D. Cotugno, Naples, Italy, ^9^Infectious Diseases, Azienda Ospedaliero‐Universitaria, Policlinico of Modena, Modena, Italy, ^10^Infectious Diseases, Polyclinic of Bari, University Hospital Polyclinic, University of Bari, Bari, Italy, ^11^Infectious Diseases, Luigi Sacco Hospital, ASST Fatebenefratelli Sacco, Milan, Italy, ^12^School of Medicine and Surgery, Infectious Diseases, ASST Grande Ospedale Metropolitano Niguarda, Milan, Italy, ^13^Infectious Diseases, University Hospital of Padova, Padue, Italy, ^14^Bologna Checkpoint, Bologna, Italy, ^15^Infectious Diseases, IRCCS Policlinico di Sant'Orsola, University of Bologna, Bologna, Italy, ^16^Infectious Diseases, ASST Papa Giovanni XXIII, Bergamo, Italy, ^17^Infectious Diseases, National Institute for Infectious Diseases, Lazzaro Spallanzani IRCCS, Rome, Italy

**Background**: HIV pre‐exposure prophylaxis (PrEP) is highly effective in preventing HIV acquisition. In Italy, free of charge availability started in May 2023. We designed a protocol to implement PrEP across Italian Cohort of Patients Naive from Antiretrovirals (ICONA): PrIDE protocol, and we collected preliminary data about PrEP users in Italy by a survey. The aim of the study is to describe distribution, organization of PrEP centres and PrEP/PLWHIV ratio in centres participating to ICONA.

**Methods: **Retrospective, observational study. Fifty‐nine centres included in ICONA and three checkpoints completed a survey focused on number of individuals in PrEP, new individuals in PrEP in 2023, organization of PrEP services and doctors involved. We estimated the PLWHIV (people living with HIV)/PrEP ratio to investigate whether PrEP access has had an impact on the management of clinics.

**Results: **In all 62 centres that completed the survey, PrEP is offered in 57 (92%): 45 (78.9%) have a PrEP unit, 12 (21.1%) follow individuals in PrEP in other units (e.g. services dedicated to PLWHIV). Eleven thousand, six hundred and seventy‐five individuals started PrEP and 9221 are currently in follow‐up. The distribution of PrEP users is not equal in all Italian regions: 45.8% of individuals are in Lombardia and 16.9% in Lazio. In 2023, new individuals who started PrEP were 4276, which represents 36.6% of all individuals who started PrEP in Italy; 24.6% in Lombardia and 23.7% in Lazio. Medical doctors available in PrEP services are not equally distributed: they are 3.5 in Lombardia, 3.4 in Lazio and 5.6 in Emilia Romagna. After the free availability of PrEP, there are 13 new centres in Italy. Number of medical doctors are different in every region: in particular, they are 0.001 in Lombardia and 0.002 in Lazio. According to ICONA data, PLWHIV/PrEP ratio is 3.51 in Lombardia and 7.52 in Lazio (Figure 1).

**P030: Figure 1:**
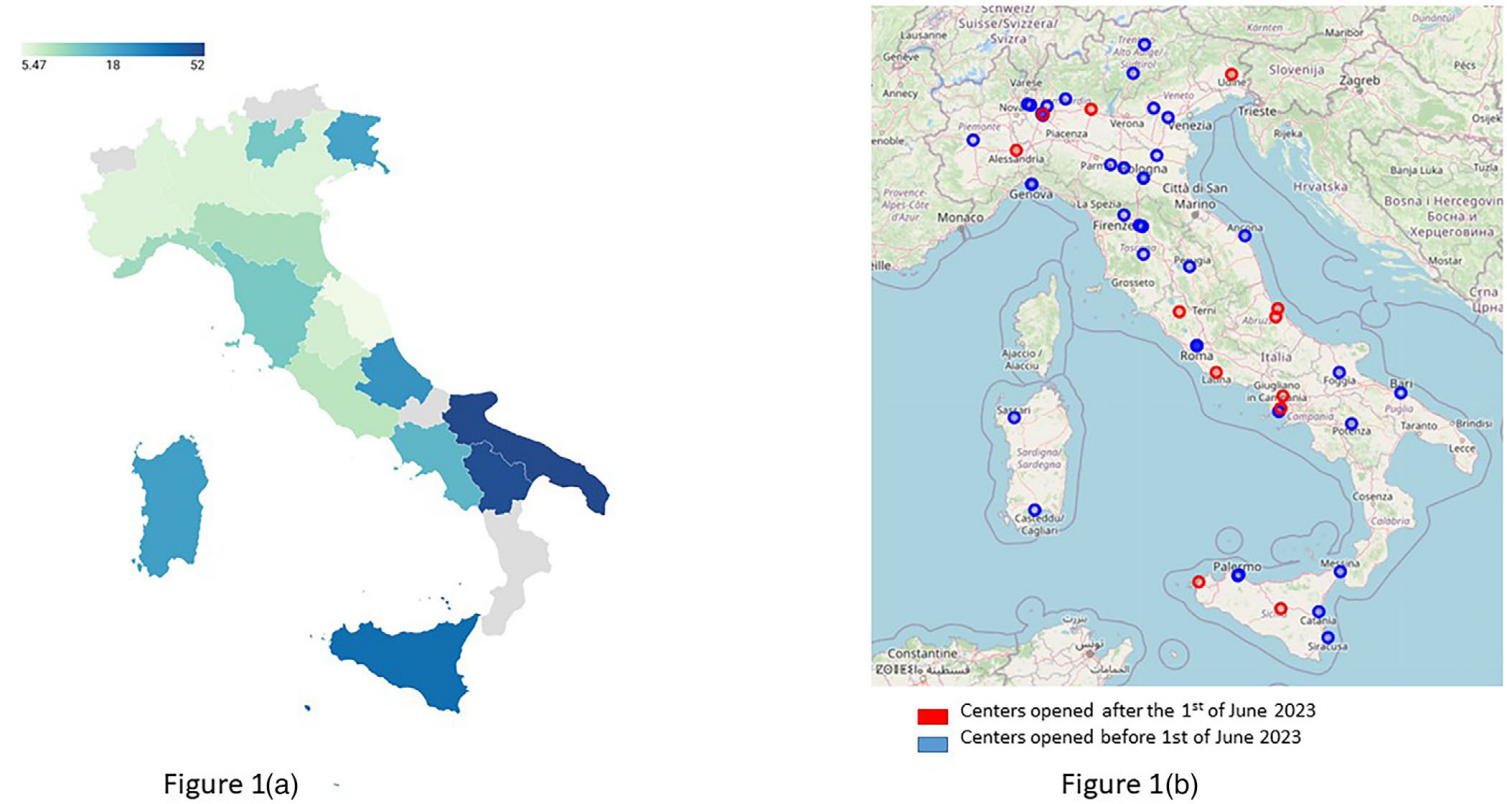
Ratio of people living with HIV (PLWHIV) monitored by ICONA centres, over the number of PrEP users undergoing follow‐up care within these centres as of 2023 (Figure 1(a)), ICONA centres that prescribe PrEP (Figure 1(b)).

**Conclusions: **Despite the overall increase in PrEP use, the organization and work pressure are not equally distributed. Implementation of PrEP is currently rapid in two regions, Lombardia and Lazio. The availability of free PrEP increased access and prescriptions.

### P031 Dynamics of STIs during and after the COVID‐19 pandemic in PrEP‐cohort at the University Medical Center Hamburg‐Eppendorf (UKE)

Nils Janis Steinhaus ^1^, Maher Almahfoud^1^, Lukas Weimann^1^, Hanna Matthews^1^, Philine Diekhoff^1^, Selina Pollich^1^, Sabine Jordan^1^, Till Koch^2^, Guido Schaefer^3^, Anja‐Dorothee Huefner^1^, Stefan Schmiedel^1^, Julian Schulze zur Wiesch^1^, Olaf Degen^1^

^1^Outpatient Center—Department for Infectious Diseases, University Medical Center Hamburg‐Eppendorf, Hamburg, Germany, ^2^First Department of Medicine, University Medical Center Hamburg‐Eppendorf, Hamburg, Germany, ^3^Study Center, Infektionsmedizinisches Centrum Hamburg (ICH), Hamburg, Germany

**Background**: The use of pre‐exposure prophylaxis (PrEP) for high‐risk groups provides reliable protection against HIV infection. Surveillance of other sexually transmitted infections (STIs) remains critical due to potential behavioural and epidemiological changes. The COVID‐19 pandemic has had a profound impact on social life, potentially altering sexual behaviour and spread of non‐HIV STIs.

**Materials and methods**: At the Outpatient Center for Infectious Diseases at the University Medical Center Hamburg‐Eppendorf (UKE), we have been following a PrEP cohort since October 2020 as part of an ongoing prospective observational study. We use a recurring online questionnaire (RedCap), standard blood tests and STI tests. We analysed the data collected in the period from Q1 2020 to Q4 2023. Positivity rates were calculated at different times during and after the pandemic using RedCap and Excel. We used AI tools (DeepL, ChatGPT) for translation and text editing. Standard descriptive statistic methods were employed.

**Results**: In total, we analysed 2521 laboratory results, leading to 322 positive diagnoses over the entire period, of which 80 in 2020, 96 in 2021, 90 in 2022 and 56 in 2023. Diagnoses included 122 gonococcal infections, 46 syphilis and 157 chlamydia. In Q4 2021, we observed a short‐term, significant increase in positivity rates for chlamydia and gonococci, followed by a decline. The positivity rate for gonococci in 2022 (5.91%) was slightly higher compared to 2021 (4.45%). The distribution of syphilis infections was uneven and showed no clear pattern.

**Conclusions**: Our study provides insights into STI dynamics among PrEP users during and after the COVID‐19 at UKE. The Q4 2021 increase in chlamydia and gonococci positivity rates may be due to catch‐up effects and eased pandemics restrictions from 15 September 2021. The lack of an increase in syphilis diagnoses could be due to a later symptom onset. Further trend analysis is crucial for adapting prevention strategies improving STI management within PrEP programmes.

### P032 Does tenofovir disoproxil fumarate/emtricitabine for HIV pre‐exposure prophylaxis induce changes in kidney function in people older than 50 years old?

Francisco Novela, Flávia Faria, Ema Pos, Soraia Almeida, Ana Cipriano, Josefina Mendez

Infectious Diseases, Unidade Local de Saúde de Santo António, Porto, Portugal

**Background**: Tenofovir disoproxil fumarate (TDF) and emtricitabine (FTC) are the staple of HIV pre‐exposure prophylaxis (PrEP) [1]. TDF causes kidney injury through toxicity to the proximal tubule, leading to a decline in renal function [2]. Concerns over TDF nephrotoxicity remain, particularly in those older than 50 years, who are at higher risk of developing chronic kidney disease (CKD) [3].

**Materials and methods**: We conducted a retrospective review of the HIV PrEP outpatient in a Portuguese tertiary hospital, between 2018 and 2024. Subjects included were older than 50 years. Demographics, PrEP management, risk factors for CKD and serum creatinine were recorded. Serum creatinine was measured before and 1 year after PrEP initiation. Kidney function was estimated using the 2021 CKD Epidemiology Collaboration equation (CKD‐EPI) based on creatinine. CKD was classified according to KDIGO guidelines.

**Results**: Sixty‐three people older than 50 years receiving PrEP were identified. Forty‐eight were included in the study, with the remainder excluded due to insufficient data. Ninety‐eight percent (*n* = 47) were cisgender men who have sex with men aged 50–75 years old (median 53). Daily PrEP was the preferred regimen (85%, *n* = 41). The main risk factors reported for CKD were hypertension (18%, *n* = 9) and diabetes mellitus (6%, *n* = 3), which coexisted in two cases. Eighty‐three percent (*n* = 40) of patients showed no changes in kidney function. Of those with declining renal function, 75% (*n* = 6) were using PrEP daily and 25% (*n* = 2) were using it on demand. Risk factors for CKD were only found in two patients with declining renal function, namely arterial hypertension.

**Conclusions**: No significant changes in renal function were observed during follow‐up in most subjects, in spite of their age and the presence of risk factors for CKD. This reinforces the safety of TDF/FTC for HIV PrEP in this age group. However, most of the patients had no previous risk factors and further studies with a longer period of monitoring are required.

**References**

1. Petruccelli KCS, Baía‐da‐Silva DC, Val F, Santos Valões M, Cubas‐Vega N, Vilhena Silva‐Neto A, et al. Kidney function and daily emtricitabine/tenofovir disoproxil fumarate pre‐exposure prophylaxis against HIV: results from the real‐life multicentric demonstrative project PrEP Brazil. AIDS Res Ther. 2022;19(1):12.

2. Schaefer R, Amparo da Costa Leite PH, Silva R, Abdool Karim Q, Akolo C, Caceres CF, et al. Kidney function in tenofovir disoproxil fumarate‐based oral pre‐exposure prophylaxis users: a systematic review and meta‐analysis of published literature and a multi‐country meta‐analysis of individual participant data. Lancet HIV. 2022;9(4):e242‐53.

3. Tittle V, Dalton R, Nugent D, Girometti N, Whitlock G, Mcowan A, et al. Complex PrEP: the factors requiring consultant‐led review of PrEP users. Sex Transm Infect. 2022;98(8):595‐8.

### P033 There are still myths and misconceptions about it!! Assessing pre‐exposure prophylaxis knowledge among adolescents in Zambia, a mixed method survey

Yvonne Kumwimba

HIV, Centre for Infectious Diseases Research in Zambia, Lusaka, Zambia

**Background**: Zambia adopted HIV oral pre‐exposure prophylaxis (PrEP) since 2017, and in 2023, over 270,000 people were initiated on PrEP in the country; of these, 41% were adolescents and young people. Despite this, Zambia still records over 28,000 new HIV infections annually with a growing incidence among adolescents. Raising awareness and addressing misconception about PrEP among adolescents is crucial to curb down HIV incidence in this population. This study assesses PrEP awareness and acceptability among adolescents in Lusaka, Zambia.

**Method**: A mixed‐method survey was conducted among 120 adolescents aged 15−24 years in Lusaka between March and September 2023. Data was collected through structured questionnaires, capturing information on PrEP knowledge, acceptability, HIV risk perceptions and key demographic characteristics. Descriptive statistics were used to analyse the data, and chi‐square tests were performed to explore associations between key demographic characteristics and PrEP acceptability.

**Results**: The findings revealed that 70% of the participants had prior PrEP knowledge, but there were misconceptions about its purpose, with some equating it with HIV antiretroviral therapy. While 57% of respondents indicated that they did not have any concerns about using PrEP, 31% of participants were unsure about its effectiveness and safety. Chi‐square tests reveal statistically significant associations between the variables age, sex and likely to use PrEP. Younger females (15−19 age group) were more likely to consider using PrEP than older adolescents (20−24 age group). Concerns raised about oral PrEP included pill burden, stigma, fear of intimate partner violence and low HIV risk perception. While only 63% of respondents had ever tested for HIV, 89% believed they were unlikely to acquire HIV.

**Conclusions**: The study revealed low awareness and inadequate knowledge about PrEP among adolescents in Zambia. Awareness campaigns that improve PrEP knowledge, dispel myths surrounding its use and address concerns about its effectiveness and safety are essential to promote PrEP acceptability among adolescents. These findings underscore the importance of comprehensive sexual education and risk reduction strategies to address HIV risk perceptions. Such public health interventions will increase PrEP uptake and reduce the burden of HIV/AIDS among adolescents in Zambia.

### P034 Clinical landscape of sexually transmitted infections among PrEP users: insights from a district hospital

Clara Bacelar, Fábio Reis, Eduarda Pena, Sara Araújo, Clara Batista, Ricardo Correia de Abreu, Frederico Duarte

Infectious Diseases, Unidade Local de Saúde de Matosinhos, Matosinhos, Portugal

**Background: **PrEP effectively prevents HIV transmission; however, these populations are at risk and have a higher prevalence of other STIs. This study aims to elucidate the clinical landscape of STIs among PrEP users in our local setting, a district hospital, also serving patients from centralized units where the waiting times for PrEP can exceed 6 months.

**Materials and methods: **Retrospective study of PrEP users with a minimum 3‐month follow‐up in 2023. Data was collected from clinical records, prescriptions and laboratory results. Microbiological swabs were primarily obtained when symptoms were present.

**Results: **A total of 105 PrEP users were identified: 97.1% cis‐male, median age of 34.8 years (21–65), 65.7% Portuguese and 23.8% from Brazil. *T. pallidum* IgG positivity prior to PrEP initiation was 39%, with a 12‐month reinfection rate of 38.1% and 7.1% incidence among those without prior history. Among participants, 44.8% reported a history of other bacterial STIs prior to PrEP. After PrEP initiation, 20 new events were documented in 15% PrEP users, including gonococcal urethritis (30%), non‐gonococcal urethritis (35%), non‐specific urethritis (5%) and extra‐genital infection (30%), with a median time of follow‐up of 10 months until the event. Other STIs diagnosed during PrEP included a case of hepatitis C, a case of monkeypox and frequent human papillomavirus (HPV) lesions. Vaccination was recommended at all visits, with vaccination rates reaching 42.9% for monkeypox and 30.5% for HPV. No HIV infections were reported during the follow‐up.

**Conclusions: **This population exhibited significant sexual risk with high STIs prevalence before PrEP. Syphilis and gonococcal infections were particularly notable among those with prior history. Patient education, regular monitoring and proactive vaccination (often challenged by high‐cost regimens) likely contributed to reduce incidence. Ongoing vigilance and targeted interventions are crucial to improve outcomes and mitigate STIs impact among PrEP users.

### Treatment strategies—Novel therapeutic targets (phase I and II)

### P035 Pharmacokinetic/pharmacodynamic and resistance analyses of GS‐1720, a once‐weekly oral integrase strand transfer inhibitor

Brie Falkard ^1^, Haeyoung Zhang^2^, Mutaz Jaber^2^, Eva Mortensen^3^, Furong Wang^4^, Christian Callebaut^1^, Dhananjay D. Marathe^2^

^1^HIV Clinical Virology, Gilead Sciences, Inc., Foster City, CA, USA, ^2^Clinical Pharmacology, Gilead Sciences, Inc., Foster City, CA, USA, ^3^Clinical Research—Virology, Gilead Sciences, Inc., Foster City, CA, USA, ^4^Biostatistics, Gilead Sciences, Inc., Foster City, CA, USA

**Background**: Long‐acting oral antiretrovirals remain an unmet medical need. GS‐1720 is an oral integrase strand transfer inhibitor (INSTI) with potent anti‐HIV‐1 activity. In a phase Ib study in people with HIV‐1, GS‐1720 was well tolerated and demonstrated antiviral activity (mean reduction in HIV‐1 RNA across four dosing cohorts [30, 150, 450 and 900 mg] of 1.74−2.44 log_10_ copies/ml from baseline to day [D] 11) [1], comparable with approved once‐daily INSTIs. We report GS‐1720 pharmacokinetics/pharmacodynamics and resistance data from this phase Ib study.

**Methods**: In this open‐label multicohort study, participants, who were treatment‐naïve or treatment‐experienced but naïve to INSTIs and off antiretroviral therapy for ≥12 weeks, were administered oral GS‐1720 on D1 and D2 and followed for 10 days. Participants were tested for genotypic and phenotypic integrase (IN) resistance at baseline and D11. Endpoints included: change in HIV‐1 RNA (log_10_ copies/ml) from baseline to D11 [1], GS‐1720 concentrations on D11, correlation between concentrations at D11 versus reduction of plasma HIV‐1 RNA, emergence of IN resistance mutations at D11.

**Results**: Participants were enrolled into 30, 150, 450 and 900 mg cohorts (*n* = 28; 7/cohort). Median (range) age was 33 (18–62) years; 89.3% were male. Mean GS‐1720 concentrations on D11 were 1.64, 5.87, 8.78 and 18.4 µg/ml in 30, 150, 450 and 900 mg cohorts, respectively; mean HIV‐1 RNA reductions on D11 were 1.74, 2.18, 2.44 and 2.37 log_10_ copies/ml, respectively. In participants with GS‐1720 concentrations at D11 above two‐fold the inhibitory quotient (IQ2; 3.876 µg/ml), robust antiviral activity (≥1.5 log_10_ copies/ml reduction in HIV‐1 RNA from baseline to D11) was observed. No participant had primary resistance‐associated mutations (RAMs) in IN at screening. No resistance to the INSTI‐class was detected at D11. One individual (lowest dose [30 mg] cohort) had increasing HIV‐1 RNA after D7 of the monotherapy period. However, resistance testing showed no INSTI RAMs at D11.

**Conclusions**: GS‐1720 showed robust antiviral activity with D11 concentrations above IQ2. There were no observed cases of treatment‐emergent IN resistance, including participants with low GS‐1720 concentrations. The robust pharmacokinetics/pharmacodynamics data and lack of resistance support further clinical development and dose selection for GS‐1720.

**Reference**

1. Fichtenbaum CJ, Berhe M, Bordon J, Lalezari JP, Oguchi G, Sinclair G, et al. Antiviral activity, safety, and pharmacokinetics of GS‐1720, a novel weekly oral INSTI [CROI Abstract 116]. Top Antivir Med. 2024;32(1):19.

### P036 Effect of acid‐reducing agents on the pharmacokinetics of oral GS‐4182

Naveed Shaik^1^, Sean Regan^1^, Deqing Xiao^1^, Furong Wang^2^, Jason Hindman^3^, Ramesh Palaparthy ^4^

^1^Clinical Pharmacology, Gilead Sciences, Inc., Foster City, CA, USA, ^2^Biostatistics, Gilead Sciences, Inc., Foster City, CA, USA, ^3^Clinical Development—Virology, Gilead Sciences, Inc., Foster City, CA, USA, ^4^Clinical Pharmacology—HIV, Gilead Sciences, Inc., Foster City, CA, USA

**Background**: Medication adherence and viral suppression in people with HIV‐1 may improve with the use of antiretrovirals that require less frequent administration. Lenacapavir (LEN), a first‐in‐class long‐acting capsid inhibitor, is rapidly absorbed following oral administration, but has low absolute bioavailability. GS‐4182 is an oral LEN prodrug with pharmacokinetics (PK) suitable for once‐weekly (QW) dosing. GS‐4182 is metabolized in the gastrointestinal tract, releasing LEN and inactive metabolites. In addition to managing polypharmacy in people with HIV, potential drug‐drug interactions (DDIs) are a reason for lower engagement with HIV pre‐exposure prophylaxis. Therefore, in a phase Ia study, we assessed the impact of an acid‐reducing agent (ARA), esomeprazole, on the PK of GS‐4182.

**Methods**: Oral GS‐4182 was administered to participants without HIV‐1, aged 18–45 years, in various single‐dose, multiple‐dose, food‐effect and DDI cohorts. Participants in the esomeprazole DDI cohort received esomeprazole 40 mg daily on days 1–5, plus a single dose of GS‐4182 400 mg on day 5 (fasting conditions). GS‐4182 PK data from this cohort were compared to week 1 PK data from the GS‐4182 400 mg multiple dose cohort, as reference. Intensive PK sampling was conducted through 168 hours post dose in both cohorts. Geometric‐least squares means (GLSM) ratios and 90% CI for LEN area under the concentration‐time curve from 0 to 168 hours (AUC_0‐168h_) and maximum concentration (C_max_) were calculated.

**Results**: In the esomeprazole (*n* = 12) and reference (*n* = 9) cohorts, respectively, median (range) age was 31 (20–42) and 25 (21–40) years and 25.0% and 44.4% were female. GS‐4182 was undetected in most participants after oral administration. Median time to maximum concentration (T_max_) was 12.0 hours in the esomeprazole and reference cohorts (Table 1). GLSM ratios (90% CI) are shown in Table 1.

**P036: Table 1:** LEN exposure with and without esomeprazole

Plasma LEN PK parameter	GS‐4182 400 mg + esomeprazole 40 mg (*n* = 12)	GS‐4182 400 mg (reference cohort) (*n* = 9)	GLSM ratio, % (90% CI)
Geometric mean AUC_0‐168h_ (%GCV), hour*ng/ml	6080 (45.1)	4810 (85.1)	126% (81.2−197)
Geometric mean C_max_ (%GCV), ng/ml	50.7 (54.4)	44.7 (141)	113% (62.5−206)
Median T_max_ (Q1, Q3), hours	12.0 (10.0, 48.0)	12.0 (4.0, 24.1)	−

Abbreviations: AUC_0‐168h_, area under the concentration versus time curve from 0 to 168 hours; C_max_, maximum observed concentration; CI, confidence interval; GCV, geometric coefficient of variation; GLSM, geometric‐least squares means; LEN, lenacapavir; PK, pharmacokinetics; T_max_, time to maximum concentration.

**Conclusions**: LEN exposure following a single oral dose of GS‐4182 400 mg was similar with and without esomeprazole coadministration. Therefore, GS‐4182 could be administered without regard to ARA use.

### P037 Correlation of baseline phenotypic sensitivity with virological response to VH3810109 (N6LS) in BANNER

Margaret Gartland ^1^, Peter Leone^2^, Judah Abberbock^3^, Kathryn Brown^4^, Paul Wannamaker^5^, Rulan Griesel^6^, Viviana Wilches^7^, Jan Losos^5^

^1^Translational Medicine, ViiV Healthcare, Durham, NC, USA, ^2^Research & Development, ViiV Healthcare, Durham, NC, USA, ^3^Statistics, GlaxoSmithKline, Collegeville, PA, USA, ^4^Clinical Pharmacology, Certara, Radnor, PA, USA, ^5^Clinical Development, ViiV Healthcare, Durham, NC, USA, ^6^Clinical Development, ViiV Healthcare, Brentford, UK, ^7^Clinical Development, GlaxoSmithKline, Collegeville, PA, USA

**Background**: VH3810109 (N6LS) is a CD4‐binding site antibody being developed for long‐acting HIV‐1 therapy. N6LS was well tolerated and demonstrated robust antiviral efficacy in people with HIV‐1 in part 1 of the proof‐of‐concept BANNER study. Pre‐treatment viral susceptibility testing may guide N6LS use. Correlation of baseline phenotypic sensitivity with demographic variables and clinical outcomes was evaluated.

**Materials and methods**: The randomized, open‐label, two‐part BANNER study assessed N6LS safety, pharmacokinetics and antiviral activity in adults naive to ART with viral load (VL) ≥5000 c/ml. N6LS was evaluated during monotherapy after single‐dose administration (IV or SC), followed by 48 weeks of standard of care (SOC) ART. Monotherapy duration was determined by virological non‐response (VL <0.5 log_10_ by day 11) or rebound (VL ≥1.0 log_10_ over nadir or <0.5 log_10_ from baseline). Plasma virus antibody sensitivity was determined retrospectively using the Monogram PhenoSense mAb RNA assay.

**Results**: Baseline N6LS phenotypic sensitivity was broad (IC_90_ 0.09 to >50 µg/ml). Using an exploratory phenotypic threshold of IC_90_ ≤2 µg/ml and maximum percent inhibition >98%, 44/54 (81%) participants successfully tested had virus sensitive to N6LS at baseline (Figure 1). No association between phenotypic sensitivity and age, sex, race, HIV‐1 subtype or CDC HIV stage was observed. Across doses, weak‐to‐moderate correlations (Pearson's *r* >0.3–0.7) were observed between increased baseline N6LS phenotypic sensitivity and greater VL decline (VLD). Additionally, weak‐to‐moderate correlations between more sensitive virus and longer time to rebound were observed for all doses except 70 mg IV. Higher baseline CD4^+^ T‐cell count showed weak‐to‐moderate correlations with maximum VLD and time to rebound with 40 mg/kg and 280 mg IV, while weak correlations (*r* >0.3–0.5) between lower baseline log_10_ VL and longer time to rebound were apparent with 280 mg and 700 mg IV.

**P037: Figure 1:**
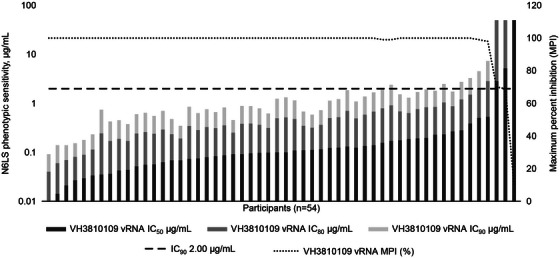
Distribution of pre‐dose viral RNA sensitivity (IC_50_, IC_80_, IC_90_ µg/ml and MPI) to N6LS. IC, inhibitory concentration; MPI, maximum percent inhibition.

**Conclusions**: Baseline N6LS viral sensitivity correlated with magnitude and duration of antiviral response, which were linked to dose and resulting N6LS serum concentration. Other factors (baseline VL, baseline CD4^+^ T‐cell count, inherent control by the individual) may impact response to N6LS. Overall, 81% of participants with successful phenotypic testing met protocol‐defined N6LS sensitivity criteria for enrolment in the ongoing phase IIb study.

### P038 Polyphenol‐rich Camu Camu capsules effect on weight and liver markers in people living with HIV on antiretroviral therapy: results of the Camu Camu pilot study (CTN PT 032)

Stephane Isnard^1^, Léna Royston^1^, Tsoarello Mabanga^1^, Carolina A. Berini^1^, Amélie Pagliuzza^2^, Talat Bessissow^3^, Peter L. Lakatos^3^, Thibault Varin^4^, Emendez Salazar^5^, Taiki Hakozaki^5^, Bertrand Lebouché^6^, Cecilia T. Costiniuk^1^, Giada Sebastiani^1^, Marina Klein^1^, Nicolas Chomont^2^, Bertrand Routy^7^, André Marette^4^, Jean‐Pierre Routy ^1^

^1^Infectious Diseases and Immunity in Global Health (IDIGH), McGill University Health Centre—Research Institute, Montreal, Canada, ^2^Immunopathology, Centre de Recherche du Centre Hospitalier de l'Université de Montréal, Montreal, Canada, ^3^Gastroenterology, McGill University Health Centre, Montreal, Canada, ^4^Medicine, Université Laval, Quebec, Canada, ^5^Cancerology, Centre de Recherche du Centre Hospitalier de l'Université de Montréal, Montreal, Canada, ^6^Center for Outcomes Research and Evaluation (CORE), McGill University Health Centre—Research Institute, Montreal, Canada, ^7^Oncology, Centre de Recherche du Centre Hospitalier de l'Université de Montréal, Montreal, Canada

**Background**: Non‐AIDS comorbidities such as liver steatosis are linked with gut microbiota dysbiosis, gut permeability and inflammation in people living with HIV (PLWH) on antiretroviral therapy (ART). Camu Camu (CC), an Amazonian superfruit, modified the gut microbiota and decreased inflammation in obese mice and in smokers. In a single‐arm pilot clinical trial, we assessed the influence of daily intake of CC capsules on gut permeability and inflammation in ART‐treated PLWH.

**Materials and methods**: We recruited 22 ART‐treated PLWH with a CD4/CD8 <1 to select those with higher levels of inflammation. Participants took 1 g of CC in capsules daily for 12 weeks while remaining on ART. Blood and stools were collected at two baseline visits, after 4 and 12 weeks of CC and 8 weeks after stopping CC. Plasma biomarkers were quantified by enzyme‐linked immunosorbent assay (ELISA). Stool microbiota was characterized by 16S rDNA sequencing. HIV DNA and RNA were quantified in sorted CD4 T cells by nested quantitative polymerase chain reaction (qPCR).

**Results**: Median age was 53, and 21/22 were male. One serious adverse event occurred and was deemed not related to CC intake. CD4, CD8 counts and viral load remained stable. Participants lost a median of 1.2 kg after 12 weeks of CC. Serum levels of liver enzymes aspartate transaminase (AST) and alanine transaminase (ALT) decreased by 11% from baseline to week 4 (*p* < 0.01) and tended to decrease at week 12. Levels of gut damage markers I‐FABP and REG3α tended to decrease at week 4. Gut microbiota composition remained stable at the genus level during the study. Plasma levels of CC‐chemokine ligand 20 (CCL20), an attractant of protective Th17 T cells in the gut, decreased at week 4 (*p* = 0.002). Plasma tumour necrosis factor alpha (TNFα) levels tended to decrease at week 4. A 1.3‐fold increase in HIV RNA levels in CD4 T cells was observed at week 20 only (*p* = 0.03).

**Conclusions**: CC intake slightly reduced weight, liver transaminases and tended to decrease inflammation in ART‐treated PLWH. The potential effect of CC or its produce on weight and liver inflammation should be validated using higher doses in larger studies.

### P039 Geno2pheno‐bNAbs: interpretable and accurate prediction of HIV‐1 bNAb resistance

Martin Pirkl ^1^, Michael Boehm^1^, Joachim Buech^1^, Philipp Schommers^2^, Florian Klein^1^, Rolf Kaiser^1^, Thomas Lengauer^1^

^1^Institute of Virology, University Hospital Cologne, Cologne, Germany, ^2^Department of Internal Medicine, University Hospital Cologne, Cologne, Germany

**Background**: HIV‐1 is still a serious issue with many deaths, especially in countries with poor access to therapeutic options. Antiretroviral therapy (ART) is a useful option for people living with HIV‐1 (PLWH) and effective against many viral strains. However, most ARTs are still a relatively large inconvenience for PLWH. The most common ARTs involve combinations of drugs targeting viral or cellular proteins. Most of these drugs have to be taken every day. An alternative to ARTs with protein inhibitors are broadly neutralizing antibodies (bNAbs). These have been shown to yield long‐term viral suppression and have to be administered only once every several weeks or even months. However, bNAbs share the problem of viral resistance with protein inhibitors. Solutions that are accurate, interpretable and easy to use are necessary to tackle the problem of predicting viral resistance to bNAbs. We developed a web‐service g2p‐bNAbs that allows users to upload viral genotypes and our service uses trained models to predict resistance to many common bNAbs [1]. The predictions consist of classification (sensitive or resistant) and the IC50 score.

**Materials and methods**: We used logistic and linear regression as our models for each task and multi‐task learning to train both models simultaneously. During training, we aim to decrease the sum of the cross‐entropy (misclassification), the mean‐squared‐error (IC50 prediction error) and the negative covariance of class‐probability and IC50‐prediction. The negative covariance penalizes models, which predict a high probability of resistance and a low IC50, and vice versa, for the same sample. In addition, this can be viewed as a regularization to prevent overfitting to the training data. The training and test data was downloaded from the CATNAP database [2].

**Results**: We compared the models of g2p‐bNAbs to other state‐of‐the‐art methods like support‐vector machines and recurrent neural nets, and found them to be competitive in regards to accuracy and have the benefit of being easily interpretable in regards to features, that is positions on the envelope.

**Conclusions**: We developed a web‐service for the prediction of antibody resistance (g2p‐bNAbs) to HIV‐1, which is free to use and can be extended to other viruses, like Sars‐Cov2, in the future.

**References**

1. Max Planck Institute for Informatics. g2p bNAbs [Internet]. 2024 [cited 2024 Aug 6]. Available from: https://bnabs.geno2pheno.org/.

2. Yoon H, Macke J, West Jr AP, Foley B, Bjorkman PJ, Korber B, et al. CATNAP: a tool to compile, analyze and tally neutralizing antibody panels. Nucleic Acid Res. 2015;43(W1):W213‐9.

### Treatment strategies—RCTs: Oral and injectable therapy in first line and suppressed switch populations

### P040 Similar virological outcomes and frequency of isolated viraemic events (blips, low‐level viraemia and suspected virological failure) between oral and long‐acting antiretroviral therapy: a pooled analysis of phase III/IIIb cabotegravir + rilpivirine long‐acting studies

John Thornhill ^1^, Louise Garside^2^, Chinyere Okoli^3^, Patricia de los Rios^4^, Kimberley Brown^5^, Lori A. Gordon^6^, Christine L. Latham^7^, William Spreen^8^

^1^Global Medical Affairs, ViiV Healthcare, Brentford, UK. ^2^Statistics, Phastar, Macclesfield, UK. ^3^Regional Medical, ViiV Healthcare, Brentford, UK. ^4^Global Medical, ViiV Healthcare, Montreal, Canada. ^5^Global Medical Affairs, ViiV Healthcare, Durham, NC, USA. ^6^Scientific Communications & Medical Education, ViiV Healthcare, Durham, NC, USA. ^7^Clinical Virology and Translation Medicine, ViiV Healthcare, Durham, NC, USA. ^8^Research and Development, ViiV Healthcare, Durham, NC, USA

**Background: **The definition and management of viral blips varies across clinical guidelines. Long‐acting (LA) antiretroviral therapy (ART) may present different management considerations for healthcare providers. In phase III/IIIb studies, viral blips (single viral load [VL] between 50 and <200 c/ml with adjacent values <50 c/ml) with cabotegravir plus rilpivirine LA (CAB+RPV LA) were not associated with confirmed virological failure (CVF; two consecutive VLs ≥200 c/ml) through up to 152 weeks of follow‐up. We present a pooled *post hoc* analysis assessing virological outcomes following viral blips, low‐level viraemia (LLV; ≥2 consecutive VLs between 50 and <200 c/ml), and isolated suspected virological failure (SVF; single plasma VL ≥200 c/ml with the subsequent value <200 c/ml), through 1 year in CAB+RPV LA phase III/IIIb studies.

**Materials and methods: **Viral blips, LLV and VLs meeting isolated SVF criterion were analysed across the phase III/IIIb FLAIR, ATLAS, ATLAS‐2M (through week 48) and SOLAR (through month 12) studies. Isolated SVF was divided into a: (1) single VL ≥200–<500 c/ml; (2) single VL ≥500–<1000 c/ml; and (3) single VL ≥1000 c/ml—all with the subsequent adjacent VL <200 c/ml. SVFs which became CVF at next VL testing were excluded (Figure 1). Plasma samples were analysed for HIV‐1 RNA VL using the Abbott RealTime HIV‐1 assay.

**P040: Figure 1:**
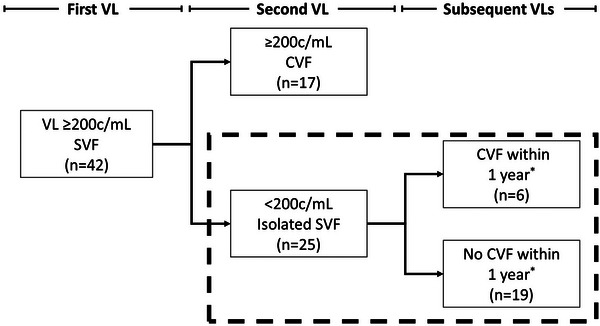
Flow diagram showing the SVF analysis population. Dashed box shows an analysis of isolated SVF data. SVFs that became CVFs at the second VL were excluded, per the definition of isolated SVF (single plasma VL ≥200 c/ml with the subsequent value <200 c/ml). *Within 1 year from the start of maintenance treatment. CVF, confirmed virological failure; SVF, suspected virological failure; VL, viral load.

**Results: **Overall, 2506 participants were included (CAB+RPV LA, *n* = 1692; comparator oral ART, *n* = 814). The proportion of participants experiencing viral blips was 6% (*n* = 97/1692) and 7% (*n* = 61/814) with LA and oral ART, respectively; <2% had LLV or isolated SVF in both arms. CVF occurred in <1% of participants (LA arm, *n* = 16/1692; oral, *n* = 7/814). In the LA arm, 25% (*n* = 3/12) of participants with isolated SVF had subsequent CVF versus 23% (*n* = 3/13) in the oral ART arm. Table 1 shows SVFs by magnitude of VL elevation.

**P040: Table 1:** Virological outcomes following blips, LLV and SVF through 1 year

*n* (%)	CAB+RPV LA (*n* = 1692)	Oral ART (*n* = 814)^a^
Participants with CVF^b^	16/1692 (<1)	7/814 (<1)
Blips^c^	97/1692 (6)	61/814 (7)
CVF with blips	0/97	1/61 (1)
LLV^d^	18/1692 (1)	10/814 (1)
CVF with LLV	1/18 (6)	0/10
Isolated SVF^e^	12/1692 (<1)	13/814 (2)
CVF with previous isolated SVF	3/12 (25)	3/13 (23)
Isolated SVF 200 to <500 c/ml	9/1692 (<1)	4/814 (<1)
CVF with previous isolated SVF 200 to <500 c/ml	2/9 (22)	1/4 (25)
Isolated SVF 500 to <1000 c/ml	3/1692 (<1)	5/814 (<1)
CVF with previous isolated SVF 500 to <1000 c/ml	1/3 (33)	1/5 (20)
Isolated SVF >1000 c/ml	0/1692	4/814 (<1)
CVF with previous isolated SVF >1000 c/ml	0/0	1/4 (25)

Abbreviations: ART, antiretroviral therapy; BIC/FTC/TAF, bictegravir/emtricitabine/tenofovir alafenamide; CAB, cabotegravir; CVF, confirmed virological failure; DTG/ABC/3TC, dolutegravir/abacavir/lamivudine; LA, long‐acting; LLV, low‐level viraemia; NRTI, nucleoside reverse‐transcriptase inhibitor; RPV, rilpivirine; SVF, suspected virological failure.

^a^Oral regimens were: DTG/ABC/3TC (*n* = 283; FLAIR [participants with side effects to this therapy, or who were positive for HLAB*5701, received DTG + two non‐ABC NRTIs]), BIC/FTC/TAF (*n* = 223; SOLAR) and various standard oral therapies (*n* = 308; ATLAS).

^b^Two consecutive HIV‐1 RNA ≥200 c/ml.

^c^A single HIV‐1 RNA 50–<200 c/ml with adjacent values <50 c/ml.

^d^≥2 consecutive viral loads 50–<200 c/ml.

^e^A single plasma HIV‐1 RNA value ≥200 c/ml with the subsequent value <200 c/ml.

**Conclusions: **In this pooled analysis through 1 year, CVF rates were low and similar between oral and LA. Viral blips and LLV were similarly infrequent with LA and oral ART and were not associated with CVF. Isolated SVF events were rare, with similar rates of subsequent CVF with LA and comparator oral ART. These data suggest similar outcomes after isolated viraemic events with both LA and oral ART.

### P041 Doravirine plus raltegravir (DOR/RAL) two‐drug regimen (2‐DR) as a maintenance antiretroviral therapy in virally suppressed people living with HIV (PLWH): results of the international randomized DORAL trial

Romain Palich ^1^, Antonella Castagna^2^, Clotilde Allavena^3^, Andrea Antinori^4^, Diana Canetti^2^, Juan Tiraboschi^5^, Claudine Duvivier^6^, Esteban Martinez^7^, Pere Domingo^8^, Salima Barkat^9^, Yasmine Dudoit^1^, Lydie Beniguel^9^, Gilles Peytavin^10^, Cathia Soulié^11^, Anne‐Geneviève Marcelin^11^, Lambert Assoumou^9^, Christine Katlama^1^

^1^Infectious Diseases, Sorbonne University, Pitié‐Salpêtrière Hospital, AP‐HP, Paris, France, ^2^Infectious Diseases, Vita‐Salute San Raffaele University, San Raffaele Scientific Institute, Milano, Italy, ^3^Infectious Diseases, Nantes University Hospital, Nantes, France, ^4^Infectious Diseases, National Institute for Infectious Diseases Lazzaro Spallanzani, IRCCS, Roma, Italy, ^5^ Infectious Diseases, Hospital Universitari de Bellvitge, Hospitalet de Llobregat, Barcelona, Spain, ^6^Necker‐Pasteur Infectiology Center, Infectious Diseases, Necker‐Enfants Malades Hospital, AP‐HP, Paris, France, ^7^Infectious Diseases, Hospital Clinic of Barcelona, Barcelona, Spain, ^8^ Infectious Diseases, Hospital de la Santa Creu i Sant Pau, Barcelona, Spain, ^9^Epidemiology, Sorbonne Université, INSERM, Institut Pierre Louis d'Épidémiologie et de Santé Publique (IPLESP), Paris, France, ^10^Toxicology—Pharmacology, Paris Cité University, Bichat‐Claude Bernard Hospital, AP‐HP, Paris, France, ^11^Virology, Sorbonne University, Pitié‐Salpêtrière Hospital, AP‐HP, Paris, France

**Background**: Taking advantage of the virological and pharmacological properties of doravirine (DOR) and raltegravir (RAL), we investigated the efficacy of the oral once daily 2‐DR DOR/RAL in virally suppressed PLWH.

**Methods**: DORAL is a prospective, international (eight centres in France, Italy and Spain), open‐label, randomized trial where PLWH with plasma viral load (pVL) <50 copies/ml for more than 12 months, no drug‐resistance mutations, no HBV co‐infection, naïve to DOR, were randomly assigned (2:1) to switch to DOR/RAL (100/1200 mg once daily) or to maintain their current regimen. The primary endpoint was virological failure ([VF], defined as two consecutive pVL ≥50 copies/ml) at week (W) 48. Secondary outcomes included treatment success rate (pVL <50 copies/ml), tolerance and changes in body weight, CD4 counts and CD4/CD8 ratio at W48.

**Results**: From October 2020 to August 2023, 114 participants were included, and started study treatment (79 in the DOR/RAL group and 35 in the continuing regimen group). Participants’ baseline characteristics are shown in Table 1. Over 48 weeks, one VF (1.3%, 95% CI 0.0–6.9) occurred at W12 in the DOR/RAL group (pVL 91 copies/ml confirmed 89 copies/ml), in a participant with adequate plasma drug concentrations and no NNRTI or INSTI baseline resistance; at failure, E138A mutation emerged; pVL was resuppressed after resumption of nevirapine‐based three‐drug previous regimen. No VF (0%, 95% CI 0.0–10.0) occurred in the continuing regimen group. The proportion of participants who maintained virological suppression at week 48 was 92.4% (95% CI 84.2–97.2) in the DOR/RAL group and 88.6% (95% CI 73.3–96.8) in the continuing regimen group. Two drug‐related grade 3–4 adverse events occurred in two participants (2.7%) in the DOR/RAL group, and none (0.0%) in the continuing regimen group at week 48 (*p* = 0.99). There were no significant differences in the changes in body weight (+0.08 vs. +0.04 kg, *p* = 0.51), CD4 counts (+37 vs. +26 cells/µl, *p* = 0.076) and CD4/CD8 ratio (+0.04 vs. +0.03, *p* = 0.912) between groups at week 48.

**P041: Table 1:** Patient characteristics at inclusion (*n* = 114)

Age, years, median (IQR)	57 (49−65)
Gender, *n* (%)	
Male	81 (71)
Female	33 (29)
Country of inclusion, *n* (%)	
France	53 (46)
Italy	37 (33)
Spain	24 (21)
Transmission group, *n* (%)	
Heterosexual	49 (43)
MSM	48 (42)
Other	17 (15)
CDC stage C, *n* (%)	21 (19)
CD4 nadir, cells/mm^3^ (IQR)	246 (125−350)
Time from HIV diagnosis, years, median (IQR)	21 (13−28)
Time from ART initiation, years, median (IQR)	18 (11−25)
Duration of viral suppression, years, median (IQR)	10 (6−15)
CD4 count, cells/mm^3^ (IQR)	700 (549−914)
CD4/CD8 ratio, median (IQR)	1.1 (0.8−1.5)
Body weight, kg, median (IQR)	75 (65−86)
Body mass index, kg/m^2^, median (IQR)	25.3 (22.6−28.3)
Antiretroviral strategy prior to inclusion, *n* (%)	
INSTI‐based 3‐drug regimen	43^a^ (38)
NNRTI‐based 3‐drug regimen	28 (24)
bPI‐based 3‐drug regimen	8 (7)
2‐drug regimen	35^b^ (31)

^a^Including 19 participants already receiving raltegravir.

^b^Including 31 participants already receiving raltegravir.

**Conclusions**: DOR/RAL combination represents a suitable, effective alternative strategy for PLWH at risk of drug‐drug interactions or needing to avoid specific toxicities of certain drugs or their negative impact on comorbidities.

### P042 Viral blips in the doravirine phase III clinical trials DRIVE‐FORWARD and DRIVE‐AHEAD

Jean‐Michel Molina ^1^, Chloe Orkin^2^, Roger Paredes^3^, Zhi Jin Xu^4^, Feng‐Hsiu Su^4^, Ernest Asante‐Appiah^5^, Rebeca M. Plank^6^, Rima Lahoulou^7^

^1^Infectious Diseases, St‐Louis and Lariboisière Hospitals, APHP, University of Paris Cité, Paris, France, ^2^HIV/AIDS Medicine, Queen Mary University of London, London, UK, ^3^Infectious Diseases, Hospital Germans Trias i Pujol, Badalona, Spain, ^4^Biostatistics, Merck & Co., Inc., Rahway, NJ, USA, ^5^Biology‐Discovery, Merck & Co., Inc., Rahway, NJ, USA, ^6^Clinical Research, Merck & Co., Inc., Rahway, NJ, USA, ^7^Clinical Research, MSD France, Puteaux, France

**Background: **Transient viraemia (“viral blip”) occurs frequently during antiretroviral therapy, but its significance is unclear. We examined associations between viral blips, baseline characteristics and virological failure (VF) in two phase III studies of doravirine (DOR), a non‐nucleoside reverse transcriptase inhibitor for HIV‐1 treatment.

**Materials and methods**: Post‐hoc analysis of viral blips (HIV‐1 RNA ≥50 copies/ml immediately preceded and followed by <50 copies/ml) in DRIVE‐FORWARD (MK‐1439‐018) and DRIVE‐AHEAD (MK‐1439A‐021). Adults with untreated HIV‐1 were randomized (1:1) to the DOR or comparator (darunavir/r or efavirenz) regimen for 96 weeks (double‐blind phase), followed by 96 weeks of the DOR regimen (open‐label extension). Cox proportional hazard models were used to analyse relationships between baseline characteristics (age, history of AIDS, HIV‐1 RNA, CD4 count, race, sex and study), blips and VF (strict definition of two consecutive HIV‐1 RNA ≥50 copies/ml after suppression).

**Results**: In the double‐blind phase, blips occurred in 11.1–11.9% of the DOR groups and 12.1–15.4% of the comparator groups, and most participants with blips had only one episode (Table 1). Baseline HIV‐1 RNA ≤100,000 copies/ml was associated with a lower hazard ratio (HR) for blips, 0.41 (95% CI 0.29–0.58). VF was more common in participants with blips (13.6%, 23/169) versus those without blips (6.1%, 71/1169), and blips were associated with an increased HR for VF, 3.79 (2.32–6.18). Treatment regimen did not appear to impact risk for blips or VF after blip. In the open‐label extension, blips occurred in 5.5–7.9% of participants (Table 1). Among participants who continued DOR, those with baseline HIV‐1 RNA ≤100,000 copies/ml had lower risk for blips: HR 0.41 (0.19–0.87). Baseline CD4 ≤200 cells/mm^3^ was not associated with increased risk for blips except in extension participants who switched to DOR: HR 3.47 (1.43–8.43). Only six participants had VF after blip in the extension (Table 1).

**P042: Table 1:** Summary of viral blips in DRIVE‐FORWARD and DRIVE‐AHEAD

	DRIVE‐FORWARD (1439‐018)		DRIVE‐AHEAD (1439A‐021)	
Double‐blind phase (day 1–week 96)	DOR + 2NRTIs, *n* (%)	DRV/r + 2NRTIs, *n* (%)	DOR/3TC/TDF, *n* (%)	EFV/FTC/TDF, *n* (%)
Participants in population	342	338	336	322
Total # of blips	42	56	54	46
Participants with blips	38/42 (11.1)	52/338 (15.4)	40/336 (11.9)	39/322 (12.1)
With 1 blip	35 (92.1)	48 (92.3)	28 (70.0)	35 (89.7)
With 2 blips	2 (5.3)	4 (7.7)	10 (25.0)	3 (7.7)
With 3 or more blips	1 (2.6)	0 (0.0)	2 (5.0)	1 (2.6)
Months to first blip, median (IQR)^a^	11.2 (5.4−16.6)	8.5 (5.5−16.7)	13.9 (6.2−18.3)	11.1 (8.3−16.6)
Virological failure after blip	8/342 (2.3)	7/338 (2.1)	5/336 (1.5)	3/322 (0.9)

**Open‐label extension (week 100–192)**	**Continued DOR + 2NRTIs**	**Switched to DOR + 2NRTIs**	**Continued DOR/3TC/TDF**	**Switched to DOR/3TC/TDF**
Participants in population	253	225	286	265
Total # of blips	14	22	20	24
Participants with blips	14/253 (5.5)	17/225 (7.6)	19/286 (6.6)	21/265 (7.9)
With 1 blip	14 (100.0)	13 (76.5)	18 (94.7)	18 (85.7)
With 2 blips	0 (0.0)	3 (17.6)	1 (5.3)	3 (14.3)
With 3 or more blips	0 (0.0)	1 (5.9)	0 (0.0)	0 (0.0)
Months to first blip, median (IQR)^a^	8.4 (1.0−15.7)	8.5 (1.0−15.6)	12.0 (4.7−15.9)	11.9 (4.6−15.5)
Virological failure after blip	1/253 (0.4)	1/225 (0.4)	1/286 (0.3)	3/265 (1.1)

Abbreviations: 3TC, lamivudine; DOR, doravirine; DRV/r, darunavir with ritonavir; EFV, efavirenz; FTC, emtricitabine; IQR, interquartile range; NRTI, nucleoside reverse transcriptase inhibitor; TDF, tenofovir disoproxil fumarate.

^a^Time to first blip was calculated from the start of treatment.

The denominator when calculating percentages is the number of participants with blips, unless otherwise specified.

**Conclusions**: In DRIVE‐FORWARD and DRIVE‐AHEAD, the DOR and comparator groups had similar rates of viral blips; in both groups, blips were more common in participants with baseline HIV‐1 RNA >100,000 copies/ml and were associated with increased risk for subsequent VF. Blips were less common after ≥2 years on DOR or switching to DOR.

### P043 Long‐term efficacy and safety of dolutegravir/lamivudine in virologically suppressed persons with HIV and history of resistance to lamivudine: week 96 results of VOLVER clinical trial—GESIDA 11820

Maria de Lagarde Sebastian ^1^, Rosa de Miguel Buckley^2^, Jose Luis Blanco‐Arevalo^3^, Adriana Pinto^4^, Rocio Montejano Sanchez^2^, Angela Gutiérrez Liarte^5^, Roser Navarro‐Soler^4^, Esperanza Cañas Ruano^6^, Alexy Inciarte^3^, Luz Martin‐Carbonero^2^, Arkaitz Imaz^7^, Cristina Hernandez Gutierrez^8^, Antonio Ocampo Hermida^9^, Marta de Miguel^10^, Rafael Delgado Vazquez^11^, Federico Pulido Ortega^1^, Jose Ramon Arribas Lopez^12^

^1^Internal Medicine/HIV Unit—imas12, University Hospital 12 de Octubre; CIBER of Infectious Diseases (CIBERINFEC); Universidad Complutense Madrid (UCM), Madrid, Spain, ^2^Internal Medicine/Infectious Diseases Unit—IdiPAZ, Hospital Universitario La Paz; CIBER of Infectious Diseases (CIBERINFEC), Madrid, Spain, ^3^Internal Medicine/Infectious Diseases Unit, Hospital Clínic, Barcelona, Spain, ^4^Internal Medicine/HIV Unit—imas12, University Hospital 12 de Octubre, Madrid, Spain, ^5^Internal Medicine/Infectious Diseases, Hospital Universitario La Princesa, Madrid, Spain, ^6^Department of Infectious Diseases, Hospital del Mar, Barcelona, Spain, ^7^HIV and STI Unit, Department of Infectious Diseases; Bellvitge University Hospital; University of Barcelona, Bellvitge Biomedical Research Institute (IDIBELL), Hospitalet de Llobregat, Barcelona, Spain, ^8^Internal Medicine/Infectious Diseases Unit, Hospital Universitario Príncipe de Asturias, Alcala de Henares, Madrid, Spain, ^9^Unidad de Enfermedades Infecciosas, Hospital Alvaro Cunqueiro, Vigo, Spain, ^10^Clinical Research Department, Fundacion SEIMC‐GESIDA, Madrid, Spain, ^11^Microbiology Department—imas12, University Hospital 12 de Octubre; CIBER of Infectious Diseases (CIBERINFEC); Universidad Complutense Madrid (UCM), Madrid, Spain, ^12^Internal Medicine/Infectious Diseases Unit—IdiPAZ, Hospital Universitario La Paz; CIBER of Infectious Diseases (CIBERINFEC); Universidad Autónoma Madrid (UAM), Madrid, Spain

**Background**: VOLVER clinical trial demonstrated at 48 weeks that dolutegravir/lamivudine effectively maintained virological suppression in people with HIV and historical confirmed or suspected lamivudine resistance after excluding lamivudine mutations in proviral DNA by population sequencing. No treatment‐emergent resistance was observed. Here, we present results through week 96.

**Materials and methods**: Open‐label, single‐arm, multicentric clinical trial. Participants were eligible if virologically suppressed at baseline and proviral DNA Sanger sequencing at screening did not detect lamivudine resistance mutations. Proviral DNA next‐generation sequencing (NGS) was retrospectively performed in baseline samples. The efficacy endpoint was the proportion of participants with HIV‐1 RNA viral load (VL) ≥50 copies/ml at 96 weeks in the intention‐to‐treat exposed (ITT‐e) population using the US Food and Drug Administration (FDA) Snapshot algorithm. Safety and tolerability outcomes were treatment‐emergent resistance, incidence of adverse events and treatment discontinuations. NCT04880785.

**Results**: One hundred and twenty‐one participants, 114 with a prior plasma genotype with M184V/I and 23 (19%) with M184V/I in baseline proviral DNA NGS (>5% threshold). At 48 weeks, 4/121 participants had VL ≥50 copies/ml, with no evidence of integrase mutations nor re‐emergence of M184V/I or K65R; eight (6.6%) participants discontinued the study for other reasons. Between week 48 and 96, there were no new virological failures among the 109 remaining participants including 21 with M184V/I (>5%) in baseline proviral DNA NGS. Overall, the proportion of participants with HIV‐1 RNA VL ≥50 copies/ml through 96 weeks was 4/121 (3.3%, 95% CI 0.9–8.2%) (Figure 1 and Table 1). No new resistance genotyping tests were performed per‐protocol. Between 48 and 96 weeks, there were four (3.7%) additional discontinuations: one treatment‐unrelated death (progression of baseline condition‐chronic obstructive pulmonary disease, COPD), one protocol deviation (HBsAg+ although HBV‐DNA undetectable), one consent withdrawal and one loss of follow‐up. There were no new discontinuations related to adverse events. At 96 weeks, the proportion of participants with VL <50 copies/ml was 86.8% (105/121; 95% CI 79.4–92.2).

**P043: Figure 1:**
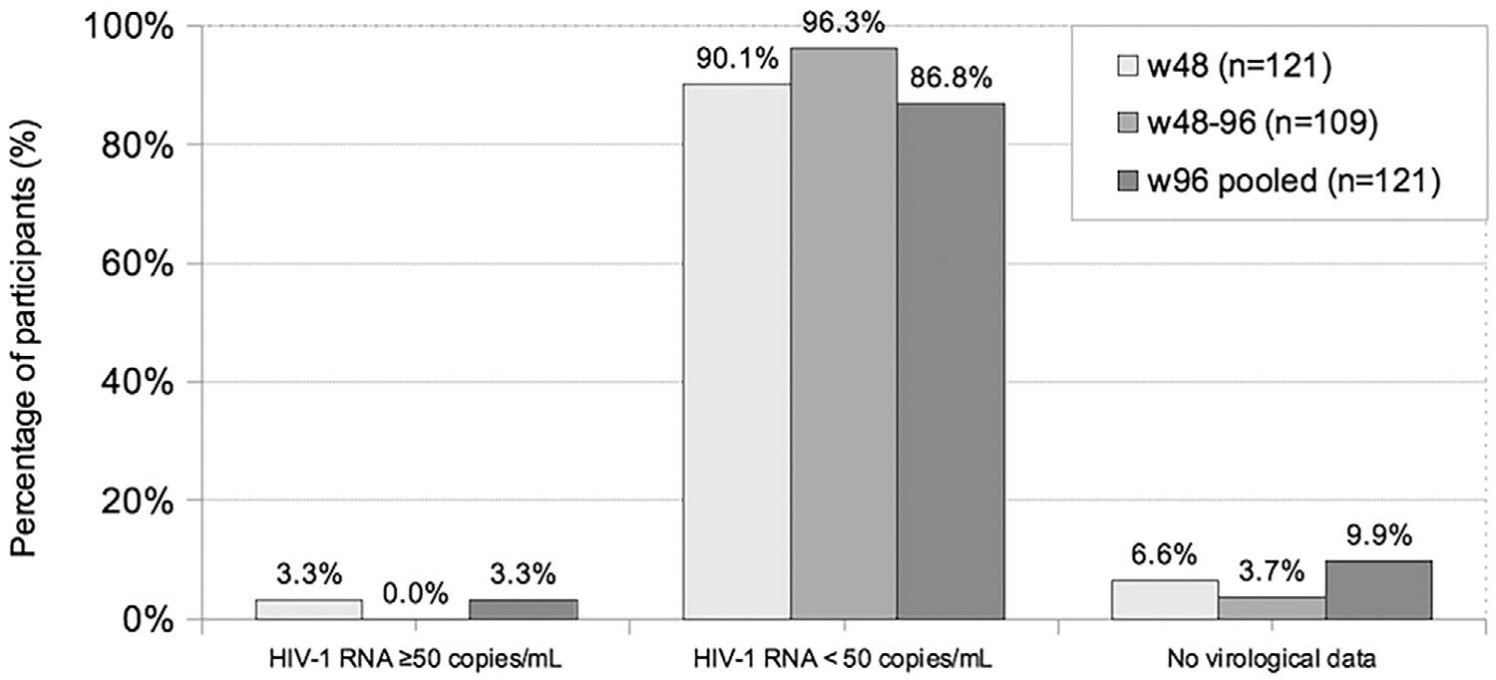
Efficacy at 96 weeks. FDA Snapshot ITTe.

**P043: Table 1:** Week 96 efficacy data (FDA Snapshot intention to treat‐exposed)

	All (*n* = 121)
**HIV‐1 RNA <50 copies/ml**	**105 (86.8%)**
**HIV‐1 RNA ≥50 copies/ml**	**4 (3.3%)**
HIV‐1 RNA ≥50 copies/ml in week 96 window	0 (0%)
Discontinuation due to lack of efficacy^a^	2 (1.7%)
Discontinuation for other reasons and last available HIV‐1 RNA ≥50 copies/ml^b^	2 (1.7%)
**No virological data at week 96**	**12 (9.9** **%)**
Discontinuation due to an adverse event	3 (2.5%)
Discontinuation for other reasons and last available HIV‐1 RNA <50 copies/ml^c^	9 (7.4%)

^a^One precautionary virological withdrawal (re‐suppressed with co‐formulated darunavir/cobicistat/tenofovir alafenamide/emtricitabine); one confirmed virological withdrawal (re‐suppressed while still on dolutegravir/lamivudine but met withdrawal criteria and was switched to dolutegravir plus darunavir/cobicistat).

^b^One lost to follow‐up; one adverse event and HIV viral load at study withdrawal of 90 copies/ml.

^c^Two lost to follow‐up; two protocol deviation (one M184V in proviral DNA at screening, chronic hepatitis B with HBAgs +); two investigator criteria (one lung cancer, one M184V detected in plasma population genotyping at transient rebound, suppressed while still on dolutegravir/lamivudine not fulfilling criteria for protocol virological withdrawal but switched to dolutegravir plus darunavir/cobicistat); one death, unrelated (COPD progression); two consent withdrawal.

**Conclusions**: After excluding lamivudine mutations in proviral DNA by population sequencing, dolutegravir/lamivudine effectively maintained virological suppression after 2 years of follow‐up in participants with history of lamivudine resistance. Notably, no treatment‐emergent resistance was observed. Virological efficacy was not affected by detection of M184V/I in proviral DNA using NGS.

### P044 Efficacy of dolutegravir plus lamivudine in treatment‐naïve people living with HIV without baseline drug‐resistance testing available: 48‐week results from the randomized D2ARLING study

Ezequiel Cordova, Jeniffer Hernandez Rendon, Veronica Mingrone, Patricio Martin, Gisela Arevalo Calderon, Soledad Seleme, Jamile Ballivian, Norma Porteiro

Clinical Research Unit, Fundación IDEAA, Buenos Aires, Argentina

**Background**: DTG+3TC two‐drug regimen is recommended as a preferred regimen for treatment‐naïve people with HIV (PHIV). However, efficacy data in PHIV without baseline HIV‐1 drug‐resistance testing is limited. As a result, some treatment guidelines do not recommend initiating DTG+3TC without baseline resistance testing information.

**Materials and methods**: D2ARLING is a randomized, non‐inferiority, open‐label, phase IV study designed to assess the efficacy and safety of DTG+3TC in treatment‐naïve PHIV with no available baseline resistance testing. Participants were randomized 1:1 (stratified by screening plasma HIV‐1 RNA and CD4^+^ T‐cell count) to DTG+3TC or DTG+TDF/XTC. Per protocol, a genotypic drug‐resistance test was performed on day 1 and remained double‐blinded throughout the study, simulating a scenario of inaccessibility of baseline resistance testing. Additionally, genotypic drug‐resistance testing was done in participants experiencing protocol‐defined virological failure (PDVF). Primary endpoint: proportion of participants with HIV‐1 RNA <50 copies/ml at week 48 (ITT‐exposed analysis, snapshot algorithm, non‐inferiority 95% CI margin 10%). Week 24 interim analysis was presented at International AIDS Society (IAS) 2023. Week 48 results are reported here (ClinicalTrials.gov: NCT04549467).

**Results**: Out of 244 subjects screened, 214 were randomized and treated with DTG+3TC (*n* = 106) or DTG+TDF/XTC (*n* = 108). Baseline characteristics were similar between arms. Median age 31 years (IQR 26–39), 23% female, 31% HIV‐1 RNA >100,000 copies/ml and 21% CD4^+^ T‐cell count <200 cells/ml. At week 48 in the ITT‐exposed snapshot, 91.5% of participants in the DTG+3TC arm and 88.9% in the DTG+TDF/XTC arm achieved HIV‐1 RNA <50 copies/ml (difference 2.6%; 95% CI −5.3%, 10.6%) (Table 1). No participants in the DTG+3TC arm and two in the DTG+TDF/XTC arm met PDVF; none developed treatment‐emergent resistance mutations. Adverse event (AE) rates were similar (DTG+3TC *n* = 61, DTG+TDF/XTC *n* = 59), and rates of withdrawals due to AEs were low (<1%) in both arms. All serious AEs were unrelated: DTG+3TC (2.8%) versus DTG+TDF/XTC (5.6%).

**P044: Table 1:** Virological outcomes at week 48

	DTG+3TC (*n* = 106)	DTG+TDF/XTC *n* = 108	Difference (95% CI)
HIV‐1 RNA <50 copies per ml	97 (91.5%)	96 (88.9%)	2.6 (−5.3, 10.6)
Virological non‐response	5	4	
No virological data	4	8	

**Conclusions**: DTG+3TC was non‐inferior to DTG+TDF/XTC at week 48 in treatment‐naïve PHIV without baseline resistance testing information. These findings suggest that genotypic testing may not be considered a specific requirement for initiating treatment with DTG+3TC. This approach could facilitate the timely initiation of this two‐drug regimen, especially in resource‐limited settings where access to baseline resistance testing may be limited.

### P045 Virological suppression, viral blip, low‐level viraemia and confirmed viraemia in virologically suppressed people with HIV‐1 switching to tenofovir DF‐containing, ainuovirine‐based antiretroviral regimen: secondary, and subgroup virological efficacy analyses of the SPRINT trial, a randomized, active‐controlled phase III study

Fujie Zhang^1^, Hao Wu^2^, Weiping Cai^3^, Ping Ma^4^, Qingxia Zhao^5^, Hongxia Wei^6^, Hongzhou Lu^7^, Hui Wang^8^, Shenghua He^9^, Zhu Chen^10^, Yaokai Chen^11^, Min Wang^12^, Wan Wan^13^, Heliang Fu^13^, Hong Qin ^13^

^1^Clinical and Research Center for Infectious Diseases, Beijing Ditan Hospital Capital Medical University, Beijing, China, ^2^Clinical and Research Center for Infectious Diseases, Beijing Youan Hospital, Capital Medical University, Beijing, China, ^3^Infectious Disease Center, Guangzhou Eighth People's Hospital, Guangzhou Medical University, Guangzhou, China, ^4^Department of Infectious Diseases, Tianjin Second People's Hospital, Tianjin, China, ^5^Department of Infectious Diseases, The Sixth People's Hospital of Zhengzhou, Zhengzhou, China, ^6^Department of Infectious Diseases, The Second Hospital of Nanjing, Nanjing, China, ^7^National Clinical Research Centre for Infectious Diseases, The Third People's Hospital of Shenzhen, The Second Affiliated Hospital of Southern University of Science and Technology, Shenzhen, China, ^8^Department of Infectious Diseases, Shenzhen Third People's Hospital, Shenzhen, China, ^9^Department of Infectious Diseases, Public Health Clinical Medical Center of Chengdu, Chengdu, China, ^10^Drug Clinical Trial Institution, Public Health Clinical Medical Center of Chengdu, Chengdu, China, ^11^Division of Infectious Diseases, Chongqing Public Health Medical Center, Chongqing, China, ^12^Institute of HIV/AIDS, The First Hospital of Changsha, Changsha, China, ^13^Clinical Research & Development, Jiangsu Aidea Pharmaceutical Co., Ltd, Yangzhou, China

**Background**: In the SPRINT trial, switch to ACC008, a novel fixed‐dose combination containing ainuovirine (ANV) plus lamivudine (3TC) and tenofovir DF (TDF) backbone, was non‐inferior to that to cobicistat‐boosted elvitegravir (EVG/c) plus emtricitabine (FTC) and tenofovir alafenamide (TAF) for virologically suppressed people with HIV (PWHs) in virological non‐suppression at week 48. We herein reported key and other secondary efficacy endpoints, and subgroup analyses at week 48 as prespecified.

**Methods**: Virologically suppressed adult PWHs (*n* = 762) were randomized to ACC008 (*n* = 381) or comparator arm (*n* = 381) for 48 weeks. Virological suppression (key secondary) was defined as <50 copies/ml (further <40 copies/ml), viral blip as transient ≥50 copies, low‐level viraemia as ≥50 but ≤200 copies/ml and viraemia as confirmed >200 copies/ml at week 48 as per the US FDA Snapshot algorithm with the intention‐to‐treat‐exposed analysis set. Subgroup analyses were performed for the key secondary efficacy endpoint with prespecified baseline characteristics as covariates.

**Results**: ACC008 arm showed a proportion of PWHs with virological suppression non‐inferior to comparator arm at week 48 (<50 copies/ml, 98.2% [374/381] vs. 98.4% [375/381], estimated treatment difference [95% CI] −0.003 [−0.021, 0.016], *p* = 0.387; <40 copies/ml, 96.9% vs. 97.1%, −0.003 [−0.023, 0.029], *p* = 0.416) (Table 1). Six (0.8%) PWHs had no virological data available (*n* = 3 for each arm), and five out of these six PWHs remained virologically suppressed at the last available visit. Four (1.0%) and three (0.8%) PWHs had HIV RNA ≥50 copies/ml in ACC008 and comparator arms, respectively; out of these seven PWHs, six had low‐level viraemia (LLV) (*n* = 3 for each arm), and one had viraemia (ACC008 arm). The seven, excepting one on comparator (confirmed viraemia, *n* = 1), PWHs achieved virological suppression at subsequent visit following continuation (*n* = 4) or switch to ACC008 (*n* = 2), namely, previously experiencing viral blip. The PWH with viraemia at week 48 became virologically suppressed at subsequent visit following continuation of ACC008 treatment. No prespecified baseline characteristics were determined to have significant effects on the key secondary efficacy endpoint (Figure 1).

**P045: Table 1:** Key and other secondary virological efficacy endpoints at week 48

ITT‐E	ANV/3TC/TDF	EVG/c/FTC/TAF	ETD [95% CI]	*p*‐value
*N*	381	381	N/A	N/A
<50 copies/ml, % (*n*)	98.2% (374)	98.4% (375)	−0.003 [−0.021, 0.016]	0.387
<40 copies/ml, % (*n*)	96.9% (369)	97.1% (370)	−0.003 [−0.023, 0.029]	0.416
≥50 copies/ml, % (*n*)	1.0% (4)	0.8% (3)	N/A	N/A
Viral blip	1.0% (4)	0.5% (2)	N/A	N/A
LLV	0.8% (3)	0.8% (3)	N/A	N/A
Confirmed viraemia	0	0.3% (1)	N/A	N/A
No virological data	0.8% (3)	0.8% (3)	N/A	N/A

*Note*: LLV, and confirmed viraemia occasionally occurred, which normally did not require alteration of ART regimen.

Abbreviations: 3TC, lamivudine; ANV, ainuovirine; CI, confidence interval; ETD, estimated treatment difference; EVG/c, elvitegravir/cobicistat; FTC, emtricitabine; ITT‐E, intention‐to‐treat‐exposed; LLV, low‐level viraemia; N/A, not applicable; TAF, tenofovir alafenamide; TDF, tenofovir disoproxil fumarate.

**P045: Figure 1:**
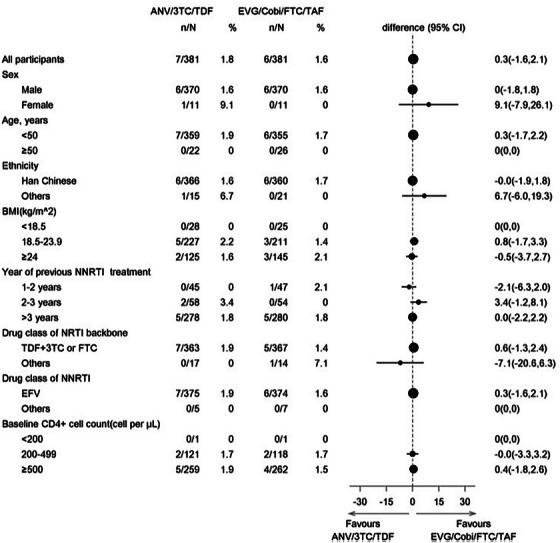
Forest plots of subgroup analyses for the primary efficacy endpoint (proportion of participants with HIV‐1 RNA ≥50 copies per ml at 48 weeks, including no virological data as per the Snapshot algorithm) in the intention‐to‐treat‐exposed population. ANV/3TC/TDF, ainuovirine/lamivudine/tenofovir disoproxil; E/C/F/TAF, elvitegravir/cobicistat/emtricitabine/tenofovir alafenamide. Data are unadjusted for treatment group differences, except for the overall data that show the adjusted treatment difference. Error bars represent 95% confidence intervals.

**Conclusions**: High proportion of virological suppression was achieved in virologically suppressed PWHs switching to ANV/3TC/TDF non‐inferior to EVG/c/FTC/TAF. Viral blip, LLV, and confirmed viraemia occasionally occurred, which normally did not require alteration of ART regimen.

### P046 High virological suppression in treatment‐naïve people with HIV‐1 on ainuovirine‐ versus efavirenz‐based antiretroviral regimen as initial therapy: effect of pretreatment NNRTI resistance from RACER trial, a multi‐centre, randomized, active‐controlled study

Fujie Zhang^1^, Qun Li^1^, Fengting Yu^1^, Hao Wu^2^, Weiping Cai^3^, Qingxia Zhao^4^, Min Wang^5^, Yaokai Chen^6^, Hongxia Wei^7^, Hong Qin ^8^

^1^Clinical and Research Center for Infectious Diseases, Beijing Ditan Hospital Capital Medical University, Beijing, China, ^2^Clinical and Research Center for Infectious Diseases, Beijing Youan Hospital, Capital Medical University, Beijing, China, ^3^Infectious Disease Center, Guangzhou Eighth People's Hospital, Guangzhou Medical University, Guangzhou, China, ^4^Department of Infectious Diseases, The Sixth People's Hospital of Zhengzhou, Zhengzhou, China, ^5^Institute of HIV/AIDS, The First Hospital of Changsha, Changsha, China, ^6^Division of Infectious Diseases, Chongqing Public Health Medical Center, Chongqing, China, ^7^Department of Infectious Diseases, The Second Hospital of Nanjing, Nanjing, China, ^8^Clinical Research & Development, Jiangsu Aidea Pharmaceutical Co., Ltd, Yangzhou, China

**Background**: Pre‐treatment drug resistance (PDR), especially to non‐nucleoside reverse transcriptase inhibitor (NNRTI), is a major therapeutic concern over antiretroviral treatment (ART). This study aimed to evaluate the effects of PDR to NNRTI on virological efficacy of ainuovirine‐ (ANV), a new‐generation NNRTI with high genetic barrier to resistance, versus efavirenz‐ (EFV) based regimen as initial therapy for treatment‐naïve people with HIV‐1 (PWH).

**Methods**: Six hundred and thirty eligible treatment‐naïve PWH aged between 18 and 65 years were equally randomized to receive ANV or EFV (*n* = 315 per group), both combined with lamivudine and tenofovir DF, for 48 weeks. The HIV‐1 pol gene fragment was sequenced for the entire protease (codons 1–99) and the first 300 codons of reverse transcriptase at baseline, and analysed against the Stanford HIV Drug Resistance Database. Viral suppression (VS) was defined as HIV‐1 RNA titre below 50 copies/ml as per US FDA Snapshot algorithm. The primary efficacy endpoint was the proportion of participants with VS at week 48 for PWH with concomitant PDR to NNRTI subgroup with the intention‐to‐treat‐exposed analysis.

**Results**: Genotyping was successful in 591 participants (93.8%), but failed or was not done in 39 participants (6.2%). PDR was detected in 81 participants (12.9%), and that to NNRTI was detected in 71 participants (11.3%), including 44 in ANV group (14.0%) and 27 in EFV group (8.6%), respectively, with V179E (*n* = 29, 4.6%) as the most frequent resistance‐associated mutation (Figure 1). For participants with concomitant PDR to NNRTI, the proportion of participants with VS in ANV group was noninferior to, and marginally higher than that with EFV group (81.8% vs. 66.7%; estimated treatment difference 0.152 [−0.050, 0.359], Newcombe‐Wilson method with missing‐as‐failure analysis) (Table 1). The proportion of VS was similar between the two treatment groups for PWH without PDR, at an overall percentage of 90.7%.

**P046: Figure 1:**
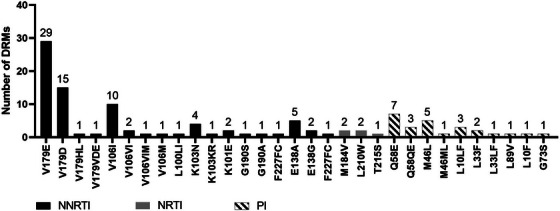
Number of detected pre‐treatment DRMs in reverse transcriptase and protease for treatment‐naïve people with HIV‐1 (*N* = 81). DRMs, drug resistance‐associated mutations; NNRTI, non‐nucleoside reverse transcriptase inhibitor; NRTI, nucleoside reverse transcriptase inhibitor; PI, protease inhibitor.

**P046: Table 1:** Virological outcomes of treatment‐naïve people with HIV‐1 on ANV‐ versus EFV‐based antiretroviral regimen as initial therapy at week 48 (PDR to NNRTI subgroup, *N* = 71)

*n* (%)	ANV+3TC+TDF (*N* = 44)	EFV+3TC+TDF (*n* = 27)
Viral suppression (HIV RNA <50 copies/ml)	36 (81.8)	18 (66.7)
Incomplete viral suppression (observed HIV RNA ≥50 copies/ml)	3 (6.8)	4 (14.8)
HIV RNA ≥50∼<1000 copies/ml	2 (4.5)	3 (11.1)
HIV RNA ≥1000 copies/ml	1 (2.3)	1 (3.7)
No virological data	5 (11.4)	5 (18.5)

Abbreviations: 3TC, lamivudine; ANV, ainuovirine; EFV, efavirenz; NNRTI, non‐nucleoside reverse transcriptase inhibitor; PDR, pretreatment drug resistance; RNA, ribonucleic acid; TDF, tenofovir disoproxil fumarate.

**Conclusions**: ANV‐based antiretroviral regimen achieved a relatively high VS in treatment‐naïve PWH although PDR to NNRTI prevailed in the study population. In consideration of additional safety benefits of ANV compared to EFV, ANV‐based regimen is recommended as a first‐line treatment of option alternative to EFV‐based regimen.

### P047 An EYEWITNESS to successful diversity in antiretroviral switch studies

Cornelius Van Dam^1^, Maria Jose Crusells‐Canales^2^, Bliss Leverette ^3^, Mounir Ait‐Khaled^4^, Emilio Letang^5^, Alex Pitchford^6^, Richard Grove^6^, Riya Moodley^4^, Karen Anderson^7^, Deanna Merrill^8^, Miguel Pascual‐Bernaldez^9^, V. Paul DiMondi^10^, Bryn Jones^11^

^1^Infectious Diseases, Cone Health Regional Center for Infectious Disease, Greensboro, NC, USA, ^2^Infectious Diseases, Hospital Clínico Universitario Lozano Blesa, C/San Juan Bosco, Zaragoza, Spain, ^3^Clinical Development, ViiV Healthcare, Durham, NC, USA, ^4^ViiV Healthcare, Clinical Development, Brentford, UK, ^5^Medical Affairs, ViiV Healthcare, Madrid, Spain, ^6^Statistics, GlaxoSmithKline, Brentford, UK, ^7^Clinical Operations, GlaxoSmithKline, Mississauga, Canada, ^8^Research & Development, ViiV Healthcare, Durham, NC, USA, ^9^Clinical Development, ViiV Healthcare, Madrid, Spain, ^10^Scientific Communications and Medical Education, ViiV Healthcare, Durham, NC, USA, ^11^Medical Affairs, ViiV Healthcare, Brentford, UK

**Background**: Achieving true diversity has long been a challenge in HIV clinical trials, preventing adequate assessment of treatments across all affected populations [1,2]. The EYEWITNESS study (NCT05911360) was designed to dismantle this obstacle to equitable research opportunities [3]. This international, phase IIIb, multicentre, prospective, open‐label, single‐arm trial describing the efficacy of switching from BIC/TAF/FTC to DTG/3TC was designed with diversity at its core. Specifically with an eye towards participants aged ≥50 years, the study enrolment targets older adults, women and individuals identifying as Black/African American or Hispanic/Latinx—groups that have historically faced systemic barriers to research participation. Here, we describe the recruitment results and strategies that facilitated achieving these goals.

**Materials and methods**: The ongoing EYEWITNESS study is conducted at 56 sites across 12 countries and enrolled people living with HIV‐1 with virological suppression (HIV‐1 viral load <50 c/ml) on BIC/TAF/FTC for >6 months with no major resistance or prior virological failure. Enrolled participants were switched to DTG/3TC and will be followed up to 96 weeks. Diversity targets were set at 30% for age ≥65 years, female sex at birth, and Black/African American race and 10% for Hispanic/Latinx ethnicity with a total planned enrolment of 200 participants. Diversity‐oriented strategies were embedded in study planning, feasibility, site selection, startup and recruitment.

**Results**: Recruitment lasted from 7 Jul 2023 to 16 Feb 2024 with 266 participants screened and 208 enrolled. All diversity targets were exceeded with 37% of participants aged ≥65 years, 42% female sex at birth, 33% Black/African American and 14% Hispanic/Latinx (Table 1). Demographics of participants recruited were generally consistent across North America and Europe. Key strategies included careful site selection, thorough diversity training, imposing screening restrictions based on targets and close communication with sites (Figure 1). Site feedback indicated that this strategic approach was well received. Diversity targets did not result in any recruitment delays.

**P047: Table 1:** Summary of demographic characteristics of enrolled participants

Characteristic	Enrolled total (*N* = 208)	Enrolled Europe (*N* = 109)	Enrolled North America (*N* = 99)
Age			
*N*	207	108	99
Median (range), year	61 (50−83)	63 (50−83)	60 (50−81)
Age ≥65 years, *n* (%)	77 (37)	48 (44)	29 (29)
Sex at birth, *n* (%)			
*N*	208	109	99
Female sex at birth	87 (42)	55 (50)	32 (32)
Race, *n* (%)			
*N*	208	109	99
Black or African American	68 (33)	22 (20)	46 (46)
Asian	3 (1)	1 (<1)	2 (2)
White	126 (61)	83 (76)	43 (43)
Unknown	7 (3)	3 (3)	4 (4)
Other races^a^	4 (2)	0	4 (4)
Ethnicity, *n* (%)			
*N*	208	109	99
Hispanic or Latinx	30 (14)	7 (6)	23 (23)
Not Hispanic or Latinx	177 (85)	101 (93)	76 (77)
Not reported	1 (<1)	1 (<1)	0

*Note*: Final data cleaning is in progress, and minor adjustments based on the final data set will be required.

^a^Includes American Indian or Alaskan Native.

**P047: Figure 1:**
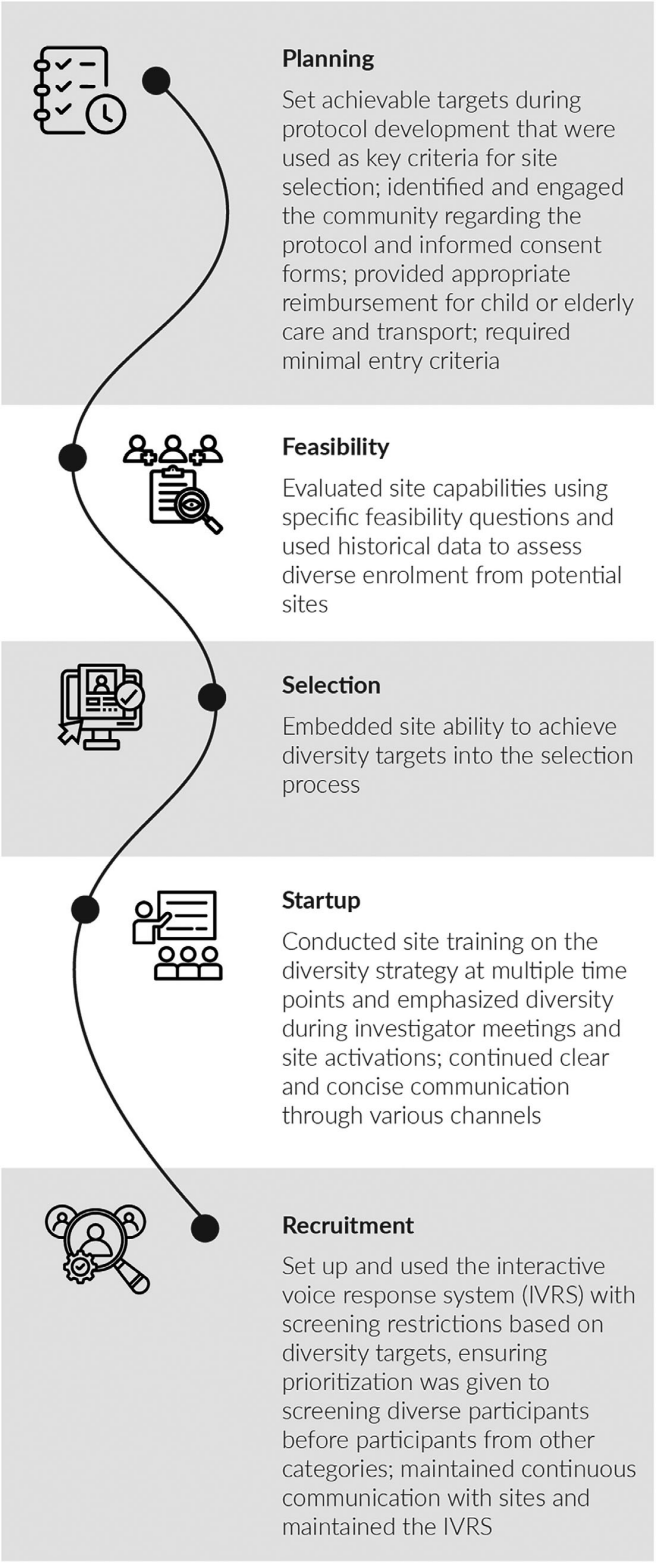
Key strategies implemented to achieve enrolment diversity targets.

**Conclusions**: The successful enrolment of a remarkably diverse study population in EYEWITNESS demonstrates the importance of proactive planning and implementation of tailored strategies to achieve diversity in clinical trials. Such efforts are vital to ensure treatments address often unmet needs of all people living with HIV‐1.

**References**

1. National Academies of Sciences, Engineering, and Medicine. Improving representation in clinical trials and research: building research equity for women and underrepresented groups. Washington, DC: National Academies Press; 2022.

2. Reid MM, Davis SP, Henry ON, Mathew AA, McCallister S, Nero TT, et al. Demographic diversity of US‐based participants in GSK‐sponsored interventional clinical trials. Clin Trials. 2023;20(2):133‐144.

3. ClinicalTrials.gov. A study to evaluate efficacy, safety and tolerability in antiretroviral therapy (ART)‐experienced participants of at least 50 years of age living with human immunodeficiency virus (HIV) with virologic suppression who switch to DTG/3TC FDC from BIC/FTC/TAF (EYEWITNESS). ClinicalTrials.gov identifier: NCT05911360 [Internet]. 2024 [cited 2024 May 30]. Available from: https://classic.clinicaltrials.gov/ct2/show/NCT05911360.

### P048 Changes in patient‐reported neuropsychological outcomes in virologically suppressed persons with HIV switching to DTG/3TC or BIC/FTC/TAF: a substudy of the PASO‐DOBLE randomized clinical trial

Lucio Garcia‐Fraile^1^, Mar Masia^2^, Maria J. Crusells‐Canales^3^, Pere Domingo^4^, Adria Curran^5^, Roberto Guerri‐Fernandez^6^, Enrique Bernal^7^, Joaquin Bravo^8^, Boris Revollo^9^, Juan Macias^10^, Juan M. Tiraboschi^11^, Rocio Montejano Sanchez^12^, Concepcion Amador^13^, Miguel Torralba^14^, Dolores Merino^15^, Vicens Diaz‐Brito^16^, Maria J. Galindo Puerto^17^, Sergio Ferra^18^, Aroa Villoslada^19^, Juan E. Losa^20^, Francisco J. Fanjul^21^, Javier Perez‐Stachowski^22^, Joaquim Peraire^23^, Joaquin Portilla^24^, Sara de la Fuente Moral^25^, Carlos Dueñas^26^, Maria J. Vazquez^27^, Silvana Di Gregorio^28^, Eduardo Manzanares^29^, Pedro Gil^29^, Marta de Miguel^29^, Jose Luis Blanco‐Arevalo^30^, Pablo Ryan^31^, Belen Alejos^32^, Esteban Martinez ^30^

^1^Infectious Diseases Unit, Hospital Universitario de la Princesa, Madrid, Spain, ^2^Infectious Diseases Unit, Hospital General Universitario, Elche, Spain, ^3^Infectious Diseases Unit, Hospital Clínico Universitario Lozano Blesa, Zaragoza, Spain, ^4^Infectious Diseases Unit, Hospital de la Santa Creu i Sant Pau, Barcelona, Spain, ^5^Infectious Diseases Unit, Hospital Universitari Vall d´Hebron, Barcelona, Spain, ^6^Infectious Diseases Unit, Hospital del Mar, Barcelona, Spain, ^7^Infectious Diseases Unit, Hospital Reina Sofía, Murcia, Spain, ^8^Infectious Diseases Unit, Hospital Morales Meseguer, Murcia, Spain, ^9^Infectious Diseases Unit, Hospital Universitari Germans Trias i Pujol, Badalona, Spain, ^10^Infectious Diseases Unit, Hospital Universitario Virgen de Valme, Sevilla, Spain, ^11^Infectious Diseases Unit, Hospital Universitari de Bellvitge, L'Hospitalet de Llobregat, Spain, ^12^Infectious Diseases Unit, Hospital Universitario La Paz, Madrid, Spain, ^13^Infectious Diseases Unit, Hospital Marina Baixa, Villajoyosa, Spain, ^14^Infectious Diseases Unit, Hospital Universitario, Guadalajara, Spain, ^15^Infectious Diseases Unit, Hospital Juan Ramon Jimenez, Huelva, Spain, ^16^Infectious Diseases Unit, Parc Sanitari Sant Joan de Deu, Sant Boi de Llobregat, Spain, ^17^Infectious Diseases Unit, Hospital Clínico Universitario, Valencia, Spain, ^18^Infectious Diseases Unit, Hospital Universitario Torrecardenas, Almeria, Spain, ^19^Infectious Diseases Unit, Hospital Universitario Son Llatzer, Palma de Mallorca, Spain, ^20^Infectious Diseases Unit, Hospital Universitario Fundacion Alcorcon, Alcorcon, Spain, ^21^Infectious Diseases Unit, Hospital Universitario Son Espases, Palma de Mallorca, Spain, ^22^Infectious Diseases Unit, Hospital Costa del Sol, Marbella, Spain, ^23^Infectious Diseases Unit, Hospital Universitari Joan XXIII, Tarragona, Spain, ^24^Infectious Diseases Unit, Hospital General Universitario Dr. Balmis, Alicante, Spain, ^25^Infectious Diseases Unit, Hospital Universitario Puerta de Hierro‐Majadahonda, Majadahonda, Spain, ^26^Infectious Diseases Unit, Hospital Clínico Universitario, Valladolid, Spain, ^27^Medical Department, ViiV Healthcare, Tres Cantos, Spain, ^28^Medical Department, CP Endocrinologia i Nutrició S.L., Barcelona, Spain, ^29^Fundación SEIMC‐GeSIDA, Madrid, Spain, ^30^Infectious Diseases Unit, Hospital Clínic & University of Barcelona, Barcelona, Spain, ^31^Infectious Diseases Unit, Hospital Universitario Infanta Leonor, Madrid, Spain, ^32^Independent Researcher, Madrid, Spain

**Background**: PASO‐DOBLE (ClinicalTrials.gov NCT04884139) demonstrated that DTG/3TC was noninferior and produced less weight gain than BIC/FTC/TAF in virologically suppressed persons with HIV (PWH). Second‐generation integrase inhibitors have been associated with adverse neuropsychological effects, which may be difficult to detect. Patient‐reported outcomes measures (PROMs) capture subjective perception of health through questionnaires. We used PROMs assessing neuropsychological symptoms in PWH from PASO‐DOBLE.

**Methods**: Clinically stable, virologically suppressed PWH on regimens containing ≥1 pill/day, boosters, or drugs with cumulative toxicity such as efavirenz or TDF were randomized (1:1) to switch stratifying by TAF in the regimen discontinued and sex. Pittsburgh Sleep Quality Index (PSQI), Hospital Anxiety and Depression Scale (HADS), and HIV Symptoms Index (HSI) were assessed at baseline, 6, 24 and 48 weeks. Increasing scores in the evaluated PROMs represent a subjective worsening of health status. Clinically relevant thresholds were: PSQI >5 (poor sleep), and HADS‐Anxiety/HADS‐Depression >8 (mild) or >11 (moderate). Differences from baseline within each arm and between arms were assessed.

**Results**: Between 14 July 2021 and 24 March 2023, 553 PWH initiated DTG/3TC (*n* = 277) or BIC/FTC/TAF (*n* = 276), including 155 (28%) with TAF in previous regimen and 147 (27%) women. At baseline, >50% had poor sleep, nearly 25% anxiety and approximately 10% depression (Table 1). Adjusted (presence of TAF in previous regimen, sex, age, race and baseline PROMs values) mean changes in PROMs from baseline are shown in Figure 1. Within each arm, there were significant decreases from baseline in global PSQI, HADS‐Anxiety and total HSI scores at 6 weeks, but differences disappeared at 48 weeks. Between arms, there were no differences in changes from baseline in PROMs or in the proportions of participants above clinically relevant thresholds at the different time points evaluated.

**P048: Table 1:** Patient‐reported outcomes (PROMs) at baseline

	DTG/3TC (*n* = 277)	BIC/FTC/TAF (*n* = 276)	*p*‐value
**Global Pittsburgh Sleep Quality** Index (PSQI)			
Mean (standard deviation)	6.3 (3.5)	5.5 (3.5)	0.011
Proportions (95% CI) of scores >5 (poor sleep)	61.9 (55.3−68.2)	55.1 (48.4−61.7)	0.141
HADS‐Anxiety			
Mean (standard deviation)	5.4 (3.7)	5.3 (3.9)	0.695
Proportions (95% CI) of scores >8 (mild anxiety)	24.2 (19.2−29.7)	24.4 (19.4−29.9)	0.959
Proportions (95% CI) of scores >11 (moderate anxiety)	9.9 (6.6−14.1)	9.1 (6.0−13.1)	0.750
HADS‐Depression			
Mean (standard deviation)	3.2 (3.1)	3.6 (3.6)	0.173
Proportions (95% CI) of scores >8 (mild depression)	9.6 (6.4−13.7)	12.4 (8.7−16.8)	0.301
Proportions (95% CI) of scores >11 (moderate depression)	3.0 (1.3−5.7)	6.9 (4.2−10.6)	0.033
HIV Symptoms Index (HSI)			
Mean total scores (standard deviation)	13.9 (11.5)	12.5 (11.5)	0.158

^a^Adjusted for presence of TAF in previous regimen, sex, age, race and baseline PROMs values.

**P048: Figure 1:**
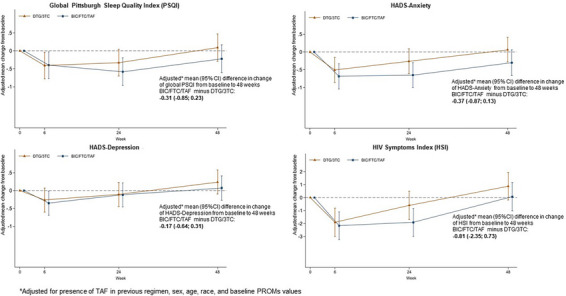
Mean adjusted^*^ changes from baseline in Pittsburgh Sleep Quality Index (PSQI), Hospital Anxiety and Depression Scale (HADS), and HIV Symptoms Index (HSI) validated tools.

**Conclusions**: Sleep quality and anxiety were very common in this clinically stable cohort. PROMs initially improved after switching to either arm, therefore, diluting the neuropsychological symptoms relative to the pre‐switch status. There were no differences between arms in changes in neuropsychological PROMs from baseline.

### P049 Pharmacokinetic (PK) analysis of oral once‐daily bictegravir (BIC) plus lenacapavir (LEN) administered separately (BIC 75 mg + LEN 25 mg; BIC 75 mg + LEN 50 mg) and as BIC/LEN 75/50 mg single‐tablet regimen to support phase III dose selection

Priyanka Arora ^1^, Elise Oh^1^, Jairo M. Montezuma‐Rusca^2^, Peter Sklar^2^, Deqing Xiao^1^, Nieves Velez de Mendizabal^1^, Ramesh Palaparthy^1^, Dhananjay D. Marathe^1^

^1^Clinical Pharmacology, Gilead Sciences, Inc., Foster City, CA, USA, ^2^Clinical Development, Gilead Sciences, Inc., Foster City, CA, USA

**Background**: A new FDC antiretroviral regimen, comprising BIC and LEN, is being developed. BIC+LEN co‐administration was investigated in the phase II portion of ARTISTRY‐1, which demonstrated efficacy and safety in virologically suppressed people with HIV. We report the PK of BIC and LEN, either as single agents co‐administered (SAC), or as BIC/LEN FDC.

**Materials and methods**: The phase II portion of ARTISTRY‐1 (NCT05502341), an ongoing, randomized, open‐label, multicentre phase II/III study, included PK evaluation of two dose combinations of BIC+LEN co‐administered once daily for 24 weeks: BIC 75 mg + LEN 25 mg or LEN 50 mg. Additionally, a relative bioavailability (rBA) study (GS‐US‐621‐6292) compared exposures of single doses of BIC/LEN 75/50 mg FDC versus BIC 75 mg + LEN 25 mg. Intensive PK samples were collected at weeks 16 or 24 (steady state) following once‐daily dosing in the phase II study, and until days 8 (BIC) and 50 (LEN) following single dose in the rBA study. PK parameters derived by non‐compartmental analysis were compared using descriptive statistics. For LEN, a population‐PK (popPK) model was developed with pooled LEN studies to further enable LEN dose selection.

**Results**: PK data included 47 and 120 participants in the phase II and rBA study, respectively. BIC exposures were comparable between BIC+LEN doses in phase II and were within pre‐defined bounds for FDC versus SAC in the rBA study (Table 1). Dose linear increases in steady‐state LEN exposures for co‐administered BIC 75 mg + LEN 50 mg versus BIC 75 mg + LEN 25 mg in the phase II, and for single doses of BIC/LEN 75/50 mg FDC versus co‐administered BIC 75 mg + LEN 25 mg in the rBA study, were observed (Table 1). The popPK model‐based simulations showed better efficacy coverage with LEN 50 mg (vs. 25 mg) to account for any potential missed doses in a daily regimen.

**P049: Table 1:** Plasma PK parameters (derived by non‐compartmental analysis) of co‐administered BIC + LEN and BIC/LEN FDC tablets

Parameter		Phase II Study	Phase II Study	Relative Bioavailability Study	Relative Bioavailability Study	Relative Bioavailability Study
		(*n* = 47; steady‐state data from weeks 16 or 24 [daily dosing])^a^	(*n* = 47; steady‐state data from weeks 16 or 24 [daily dosing])^a^	(*n* = 120; collected until days 8 [BIC] and 50 [LEN] after a single dose)	(*n* = 120; collected until days 8 [BIC] and 50 [LEN] after a single dose)	(*n* = 120; collected until days 8 [BIC] and 50 [LEN] after a single dose)
		BIC 75 mg + LEN 25 mg (*n* = 26)	BIC 75 mg + LEN 50 mg (*n* = 21)	BIC 75 mg + LEN 25 mg (*n* = 60)	BIC/LEN 75/50 mg (*n* = 60)	%GLSM ratio (90% CI)^b^
C_max_, ng/ml, mean (% CV)	BIC	9740 (31)	9460 (37)	5790 (29)	6580 (22)	117 (107−128)
	LEN	82 (100)	134 (74)	3.7 (58)	7.4 (61)	200 (169−236)
C_trough_, ng/ml, mean (% CV)	BIC	4330 (47)	4540 (64)	−	−	−
	LEN	58 (77)	108 (80)	−	−	−
AUC_tau_, hour*ng/ml, mean (% CV)	BIC	150,000 (31)	137,000 (44)	−	−	−
	LEN	1460 (77)	2690 (79)	−	−	−
AUC_inf_, hour*ng/ml, mean (% CV)	BIC	−	−	129,000 (34)	160,000 (28)	127 (114−142)
	LEN	−	−	1120 (57)	2570 (50)	237 (202−278)

Abbreviations: AUC_inf_, area under the curve to infinity; AUC_tau_, area under the plasma concentration–time curve over the dosing interval; BIC, bictegravir; CI, confidence interval; C_max_, maximum observed plasma drug concentration; C_trough_, trough serum concentration; CV, coefficient of variation; FDC, fixed‐dose combination; GLSM, geometric least‐squares mean; LEN, lenacapavir; PK, pharmacokinetic.

^a^These treatments were administered in conjunction with loading doses of oral LEN 600 mg on days 1 and 2.

^b^%GLSM was calculated from BIC/LEN 75/50 mg FDC tablet versus co‐administered BIC 75 mg + LEN 25 mg for the respective analytes.

**Conclusions**: Our analyses support the use of BIC/LEN 75/50 mg FDC for phase III, which will match the exposure experience of BIC 75 mg + LEN 50 mg in phase II. This dose combination was chosen based on efficacy, safety, cumulative PK in phase II and rBA, and additional consideration of the exposure coverage for potential missed doses, projected by popPK analysis.

### P050 Metabolic changes at 48 weeks in virologically suppressed people with HIV switching from complex antiretroviral regimens to bictegravir plus lenacapavir: ARTISTRY‐1 trial

David Prelutsky^1^, Karam Mounzer^2^, Moti Ramgopal^3^, Jihad Slim^4^, Malcolm Hedgcock^5^, Sorana Segal‐Maurer^6^, Mark Bloch^7^, Xu Zhang^8^, Jairo M. Montezuma‐Rusca ^9^, Peter Sklar^9^, Peter Ruane^10^

^1^Southampton Healthcare, Saint Louis, MO, USA, ^2^Jonathan Lax Treatment Center, Philadelphia FIGHT, Philadelphia, PA, USA, ^3^Midway Immunology and Research Center, Fort Pierce, FL, USA, ^4^Saint Michael's Medical Center, New York Medical College, Valhalla, NY, USA, ^5^Spectrum Health, Vancouver, Canada, ^6^The Dr James J Rahal Jr Division of Infectious Diseases, New York‐Presbyterian Queens, Flushing, NY, USA, ^7^Holdsworth House Medical Practice, Darlinghurst, Sydney, Australia, ^8^Biostatistics, Gilead Sciences, Inc., Foster City, CA, USA, ^9^Clinical Development, Gilead Sciences, Inc., Foster City, CA, USA, ^10^Men's Health Foundation, Los Angeles, CA, USA

**Background**: The combination of bictegravir (BIC) and lenacapavir (LEN) is in development as an alternative to complex antiretroviral regimens for people with HIV (PWH). BIC+LEN has demonstrated efficacy in virologically suppressed PWH and could help optimize treatment and reduce the risk of adverse events, including metabolic complications. Here, we report the week 48 metabolic outcomes for BIC+LEN versus complex antiretroviral regimens in virologically suppressed PWH participating in phase II of the ARTISTRY‐1 study.

**Materials and methods**: ARTISTRY‐1 (NCT05502341) is an ongoing, randomized, open‐label, multicentre phase II/III study. In phase II, participants on complex antiretroviral regimens (≥6 months before screening) were randomized to receive once‐daily oral BIC 75 mg + LEN 25 mg (BIC+LEN 75+25), oral BIC 75 mg + LEN 50 mg (BIC+LEN 75+50) or continue on complex antiretroviral regimens. We evaluated changes from baseline in lipid parameters and fasting glucose up to week 48.

**Results**: Of 128 participants, 51 received BIC+LEN 75+25, 52 received BIC+LEN 75+50 and 25 continued on complex antiretroviral regimens. At baseline, 81% of participants were male, 30% were Black and 16% were Hispanic/Latine; median age was 60 years. Participants were taking a median (range) of 3 (2–9) tablets/day. At week 48, fasting lipid parameters generally improved from baseline in the BIC+LEN 75+25 and BIC+LEN 75+50 groups versus the complex antiretroviral regimen group (Figure 1); median change (mg/dl) in: total cholesterol, −21 (−12.3%) and −14 (−8.1%) versus +4 (+1.8%); low‐density lipoprotein cholesterol, −10 (−11.0%) and −11 (−9.9%) versus +5 (+8.7%); triglycerides, −18 (−14.6%) and −9 (−10.2%) versus +6 (+5.1%), respectively. High‐density lipoprotein cholesterol remained stable: −2 (−2.9%) and 0 (0.0%) versus −3 (−6.0%), respectively. Median absolute change (quartile [Q] 1, Q3) in fasting glucose level was +3 (−7, 7) mg/dl in the BIC+LEN 75+25 group, +2 (−6, 14) mg/dl in the BIC+LEN 75+50 group and −6 (−13, 1) in the complex antiretroviral regimen group.

**P050: Figure 1:**
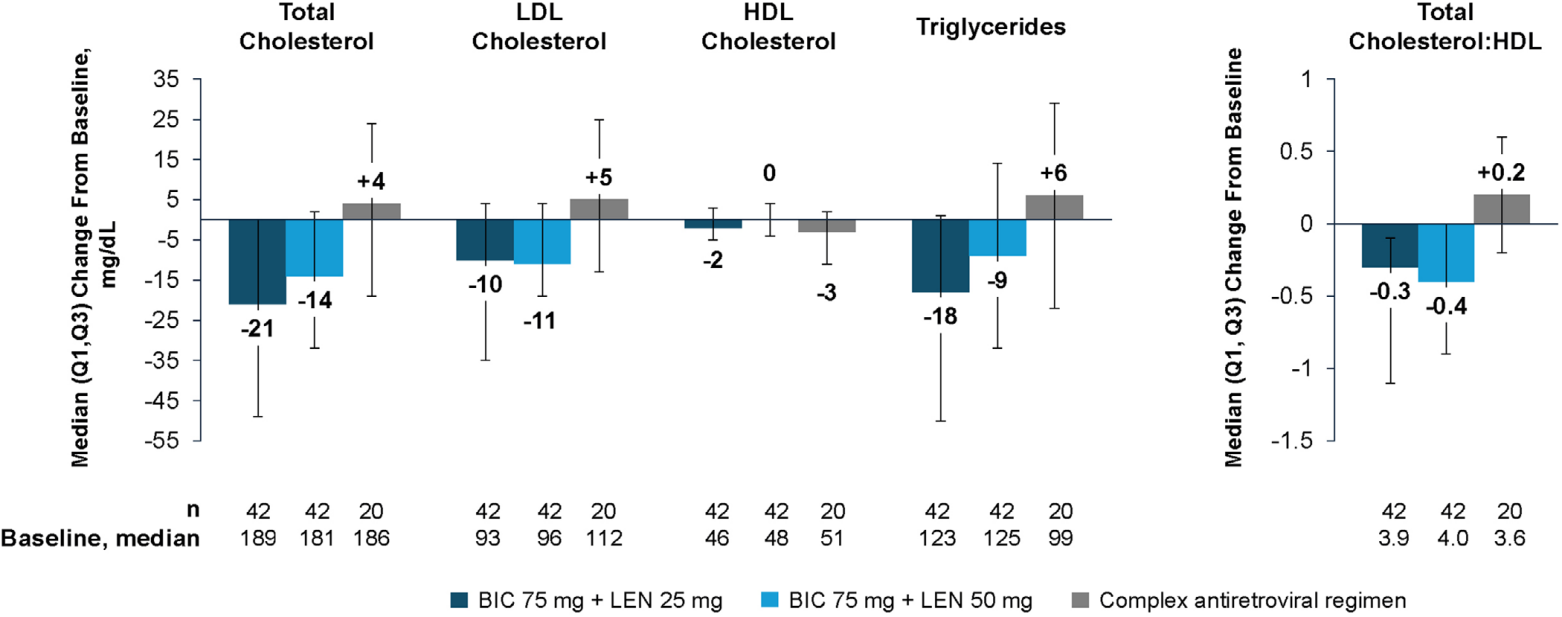
Absolute change from baseline in fasting lipid parameters at week 48. HDL, high‐density lipoprotein; LDL, low‐density lipoprotein.

**Conclusions**: After 48 weeks of follow‐up, metabolic parameters generally improved after switching from baseline complex antiretroviral regimens to BIC+LEN. Phase III studies are underway to confirm the efficacy and safety of BIC+LEN combination therapy in PWH.

### P051 Comparison of treatment‐emergent resistance associated mutations among single tablet regimens and cabotegravir+rilpivirine for the treatment of virologically suppressed people with HIV: a systematic literature review and network meta‐analysis

Ishfaq Rashid^1^, Teerapon Dhippayom^2^, Connor Willis^1^, Howard Weston Schmutz^1^, Moti Ramgopal ^3^, Elizabeth Sherman^4^, Rachel Safran^5^, Nicholas Yared^6^, Amy R. Weinberg^7^, Soodi Navadeh^8^, Nathan R. Unger^7^, Nathorn Chaiyakunapruk^1^

^1^Department of Pharmacotherapy, University of Utah College of Pharmacy, Salt Lake City, UT, USA, ^2^Faculty of Pharmaceutical Sciences, Naresuan University, Phitsanulok, Thailand, ^3^Midway Specialty Care, Fort Pierce, FL, USA, ^4^Department of Pharmacy Practice, Nova Southeastern University College of Pharmacy, Davie, FL, USA, ^5^Internal Medicine, MultiCare Health System INW, Spokane, WA, USA, ^6^Division of Infectious Diseases, Department of Medicine, Henry Ford Health System, Detroit, MI, USA, ^7^US Medical Affairs HIV Treatment, Gilead Sciences, Foster City, CA, USA, ^8^Real‐World Evidence‐Virology, Gilead Sciences, Foster City, CA, USA

**Background**: Treatment‐emergent resistance associated mutations (TE‐RAMs), including dual‐class resistance, are developing in people with HIV (PWH) adherent to the injection schedule for cabotegravir+rilpivirine (CAB+RPV). TE‐RAMs have not been observed in people adherent to HIV guideline recommended single tablet regimens (STRs) with a high barrier to resistance. This study compared the risk of TE‐RAMs among STRs and CAB+RPV in virologically suppressed (VS) PWH.

**Methods**: Randomized controlled trials (RCTs) investigating switching to any STR or CAB+RPV in VS PWH with ≥48 weeks of follow‐up in both arms, and published 2003–March 2024, were retrieved from PubMed, Embase, Cochrane CENTRAL and EBSCO Open Dissertation. Arms comprised of multi‐tablet regimens were included only if the intervention arm was an STR. For studies with multiple regimens in the comparison arm, the regimen with the most participants was used. Risk ratios (RR) with 95% confidence intervals were estimated using a random‐effects model. Surface under the cumulative ranking curves (SUCRA) were used to rank interventions to prevent TE‐RAMs. SUCRA scores signal the probability a treatment has of being among the best options in the network. Higher scores represent better ranking.

**Results**: Nineteen RCTs (10,760 participants) were included. At 48 weeks, risk of TE‐RAMs with B/F/TAF and DTG/ABC/3TC is potentially 80% lower than CAB+RPV Q8W (RR 0.20 [0.02–1.83] and 0.20 [0.002–16.67], respectively), and tended to be lower than CAB+RPV Q4W and all two‐ and three‐drug STRs (Table 1). Risk of TE‐RAMs with CAB+RPV Q4W appears 56% lower than Q8W (RR 0.44 [0.16–1.22]). CAB+RPV Q8W showed a trend towards a higher risk of TE‐RAMs and a lower probability of preventing TE‐RAMs than all INSTI‐ and PI‐based STRs (Figure 1). B/F/TAF (74.3%) ranked highest and EFV/FTC/TDF (22.7%) ranked lowest for probability of preventing TE‐RAMs.

**P051: Table 1:** Pooled estimates for risk of TE‐RAMs at 48 weeks (RR [95% CI])

B/F/TAF												
0.45 (0.05−4.34)	CAB+RPV Q4W											
0.20 (0.02−1.83)	0.44 (0.16−1.22)	CAB+RPV Q8W										
0.34 (0.00−30.94)	0.76 (0.01−79.65)	1.74 (0.02−186.99)	D/C/F/TAF									
0.99 (0.02−49.69)	2.21 (0.02−205.13)	5.04 (0.06−458.15)	2.90 (0.01−1134.89)	DTG+2NRTIs								
0.35 (0.01−12.32)	0.78 (0.02−28.74)	1.78 (0.05−69.14)	1.03 (0.01−163.78)	0.35 (0.00−70.41)	DTG/3TC							
1.00 (0.02−50.04)	2.23 (0.02−206.57)	5.08 (0.06−461.38)	2.92 (0.01−1142.90)	1.01 (0.00−256.15)	2.85 (0.01−566.10)	DTG/ABC/3TC						
0.35 (0.00−31.65)	0.79 (0.01−81.51)	1.80 (0.02−191.35)	1.04 (0.00−261.33)	0.36 (0.00−138.70)	1.01 (0.01−159.53)	0.35 (0.00−137.72)	DTG/RPV					
0.26 (0.03−2.01)	0.58 (0.08−4.41)	1.31 (0.15−11.17)	0.75 (0.01−54.12)	0.26 (0.00−21.66)	0.73 (0.04−14.89)	0.26 (0.00−21.51)	0.73 (0.01−51.62)	E/C/F/TXF				
0.10 (0.01−1.81)	0.22 (0.01−4.52)	0.49 (0.02−10.90)	0.28 (0.00−26.61)	0.10 (0.00−12.98)	0.28 (0.01−5.57)	0.10 (0.00−12.89)	0.27 (0.00−25.40)	0.37 (0.04−3.95)	EFV/FTC/TDF			
0.82 (0.02−38.72)	1.83 (0.04−94.30)	4.17 (0.08−224.63)	2.39 (0.01−430.53)	0.83 (0.00−201.66)	2.34 (0.05−117.23)	0.82 (0.00−200.24)	2.31 (0.01−411.53)	3.18 (0.10−99.17)	8.48 (0.69−104.17)	RPV/FTC/TAF		
0.11 (0.00−6.49)	0.24 (0.00−15.74)	0.56 (0.01−37.39)	0.32 (0.00−68.18)	0.11 (0.00−31.64)	0.31 (0.00−19.59)	0.11 (0.00−31.42)	0.31 (0.00−65.19)	0.42 (0.01−17.06)	1.13 (0.07−19.52)	0.13 (0.01−1.67)	RPV/FTC/TDF	
0.17 (0.02−1.57)	0.38 (0.03−4.61)	0.86 (0.07−11.10)	0.50 (0.01−24.95)	0.17 (0.00−15.49)	0.48 (0.02−12.15)	0.17 (0.00−15.38)	0.48 (0.01−23.77)	0.66 (0.12−3.63)	1.76 (0.18−17.60)	0.21 (0.01−6.25)	1.56 (0.04−60.76)	bPI+2NRTIs

Abbreviations: B/F/TAF, bictegravir/emtricitabine/tenofovir alafenamide; bPI, boosted protease inhibitor; CAB+RPV, cabotegravir+rilpivirine; CI, confidence interval; D/C/F/TAF, darunavir/cobicistat/emtricitabine; DTG, dolutegravir; DTG/3TC, dolutegravir/lamivudine; DTG/ABC/3TC, dolutegravir/abacavir/lamivudine; DTG/RPV, dolutegravir/rilpivirine; E/C/F/TXF, elvitegravir/cobicistat/emtricitabine/(TDF or TAF); EFV/FTC/TDF, efavirenz/emtricitabine/tenofovir disoproxil fumarate; NRTI, nucleoside reverse transcriptase inhibitor; Q4W, every 4 weeks; Q8W, every 8 weeks; RR, risk ratio.

**P051: Figure 1:**
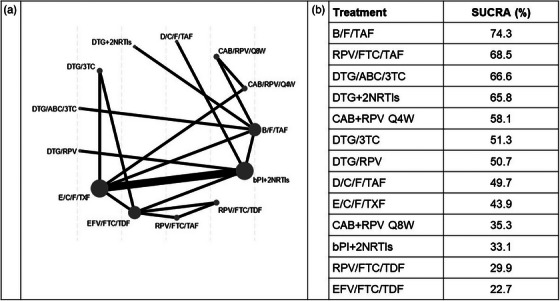
(a) Network map and (b) SUCRA ranking for TE‐RAMS at 48 weeks. B/F/TAF, bictegravir/emtricitabine/tenofovir alafenamide; bPI, boosted protease inhibitor; CAB+RPV, cabotegravir+rilpivirine; D/C/F/TAF, darunavir/cobicistat/emtricitabine; DTG, dolutegravir; DTG/3TC, dolutegravir/lamivudine; DTG/ABC/3TC, dolutegravir/abacavir/lamivudine; DTG/RPV, dolutegravir/rilpivirine; E/C/F/TXF, elvitegravir/cobicistat/emtricitabine/(TDF or TAF); EFV/FTC/TDF, efavirenz/emtricitabine/tenofovir disoproxil fumarate; NRTI, nucleoside reverse transcriptase inhibitor; Q4W, every 4 weeks; Q8W, every 8 weeks.

**Conclusions**: In VS PWH, B/F/TAF has the highest probability of preventing TE‐RAMs and tended to have the lowest risk of TE‐RAMs, whereas CAB+RPV Q8W performed similar to STRs with lower barriers to resistance. Since treatment is lifelong, and resistance impacts current and future treatment options, clinicians should include the differential risk of TE‐RAMs in shared‐decision making discussions when switching ART in stable, suppressed individuals.

### P052 Virological outcomes and resistance profile in people with HIV‐1 switching to ainuovirine‐ from NNRTI‐based antiretroviral regimen containing two‐NRTI backbone: the integrated efficacy analysis of the RACER‐EXT and SPRINT studies, two multi‐centre, randomized, active‐controlled phase III trials

Yaokai Chen^1^, Min Wang^2^, Fujie Zhang^3^, Hao Wu^4^, Weiping Cai^5^, Ping Ma^6^, Qingxia Zhao^7^, Hongxia Wei^8^, Hongzhou Lu^9^, Hui Wang^10^, Shenghua He^11^, Zhu Chen^12^, Wan Wan^13^, Heliang Fu^13^, Hong Qin ^13^

^1^Division of Infectious Diseases, Chongqing Public Health Medical Center, Chongqing, China, ^2^Institute of HIV/AIDS, The First Hospital of Changsha, Changsha, China, ^3^Clinical and Research Center for Infectious Diseases, Beijing Ditan Hospital Capital Medical University, Beijing, China, ^4^Clinical and Research Center for Infectious Diseases, Beijing Youan Hospital, Capital Medical University, Beijing, China, ^5^Infectious Disease Center, Guangzhou Eighth People's Hospital, Guangzhou Medical University, Guangzhou, China, ^6^Department of Infectious Diseases, Tianjin Second People's Hospital, Tianjin, China, ^7^Department of Infectious Diseases, The Sixth People's Hospital of Zhengzhou, Zhengzhou, China, ^8^Department of Infectious Diseases, The Second Hospital of Nanjing, Nanjing, China, ^9^The Second Affiliated Hospital of Southern University of Science and Technology, The Third People's Hospital of Shenzhen, National Clinical Research Centre for Infectious Diseases, Shenzhen, China, ^10^Department of Infectious Diseases, Shenzhen Third People's Hospital, Shenzhen, China, ^11^Department of Infectious Diseases, Public Health Clinical Medical Center of Chengdu, Chengdu, China, ^12^Drug Clinical Trial Institution, Public Health Clinical Medical Center of Chengdu, Chengdu, China, ^13^Clinical Research & Development, Jiangsu Aidea Pharmaceutical Co., Ltd, Yangzhou, China

**Background**: Ainuovirine (ANV) is a new‐generation non‐nucleoside reverse transcriptase inhibitor (NNRTI). Switch to ANV plus lamivudine and tenofovir DF (ANV/3TC/TDF) showed durable virological suppression in people with HIV‐1 (PWH) in the RACER‐EXT and SPRINT studies, two multi‐centre, randomized, active‐controlled phase III trials. This integrated efficacy analysis aimed to evaluate virological outcomes and resistance profile at post‐switch week 48.

**Methods**: In the RACER‐EXT study, out of 296 PWH completing the preceding 48‐week treatment with EFV plus 3TC and TDF, 287 participants (*N* = 287) switched to ANV/3TC/TDF in the 48‐week open‐label extensional period. In the SPRINT study, 381 participants (*N* = 381) previously on NNRTI‐based ARV regimen were randomized to ANV/3TC/TDF for 48 weeks. The two study populations were pooled for analyses with the intention‐to‐treat‐exposed principle (*N* = 668). Virological outcomes were classified at switch, and post‐switch week 48, as per US FDA Snapshot algorithm. Genotypic resistance testing was performed for resistance‐associated mutations (RAMs) when a participant had a confirmed HIV RNA above 400 copies/ml, or discontinued study treatment and/or prematurely withdrew from the study.

**Results**: High proportion of virological suppression remained durable over 48‐week switch therapy in the pooled analysis (at switch vs. post‐switch, 97.6% vs. 96.9%; estimated treatment difference [95% CI] −0.008 [−0.026, 0.011], Newcombe‐Wilson method; *p* = 0.202, one‐tailed), and similar between the two studies (Figure 1). Proportion of incomplete virological suppression remained the same between at switch and post‐switch week 48 (2.4% vs. 2.4%). The difference in virological suppression was primarily driven by occurrence of no virological data between the two time points (0% vs. 0.7%). Four participants with virological failure were eligible for genotypic resistance testing while on switch therapy: amplification failure in three participants, and persistence of baseline pre‐existing V179E, and newly emerging Y181C and M184I in one (Table 1). Five participants had virological data missing at post‐switch week 48, all of whom remained virologically suppressed at last available visits.

**P052: Figure 1:**
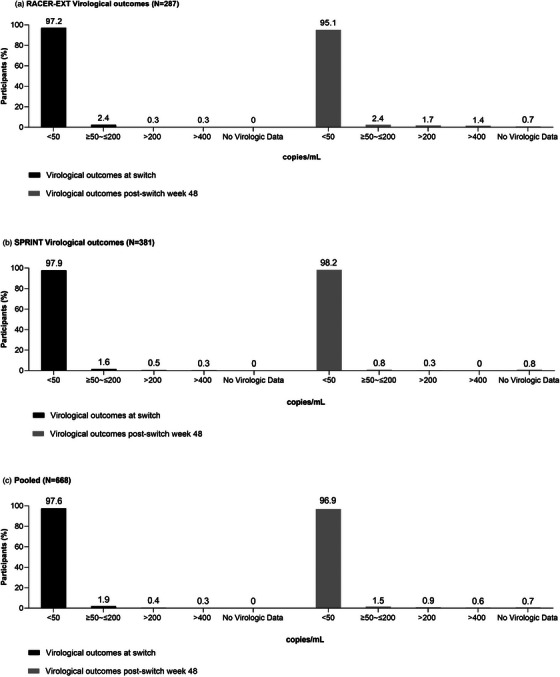
Virological outcomes of people with HIV‐1 switching to ANV/3TC/TDF (*N* = 668). ANV/3TC/TDF, ainuovirine/lamivudine/tenofovir disoproxil fumarate; ITT‐E, intention‐to‐treat‐exposed; RACER‐EXT, Research of Ainuovirine‐ Controlled by Efavirenz‐based antiretroviral Regimen, extensional; RNA, ribonucleic acid; SPRINT, Switch People with HIV‐1 to Receive Innovative Non‐nucleoside reverse transcriptase inhibitor‐based Therapy.

**P052: Table 1:** Resistance profile of people with HIV‐1 switching to ANV/3TC/TDF (*N* = 9)

					NNRTI RAMs		NRTI RAMs	
Study	Random. no.	HIV RNA titre at switch, copies/ml	HIV RNA titre at post‐switch week 48, copies/ml	HIV RNA titre at the last available visit, copies/ml	BSL^a^	Post‐treatment^b^	BSL^a^	Post‐treatment^b^
RACER‐EXT	#246	TND	929	N/A	WT	Amplification failure	WT	Amplification failure
RACER‐EXT	#529	TND	770	N/A	WT	Amplification failure	WT	Amplification failure
RACER‐EXT	#063	6192	Premature withdrawal^c^	6540	WT	Amplification failure	WT	Amplification failure
RACER‐EXT	#518	TND	Premature withdrawal^c^	1294	V179E	V179E, **Y181C**	WT	**M184I**
RACER‐EXT	#317	TND	No data available^d^	<40	N/A	N/A	N/A	N/A
RACER‐EXT	#582	<40	No data available^d^	TND	N/A	N/A	N/A	N/A
SPRINT	#029	TND	No data available^d^	TND	N/A	N/A	N/A	N/A
SPRINT	#520	TND	No data available^d^	TND	N/A	N/A	N/A	N/A
SPRINT	#641	<40	No data available^d^	TND	N/A	N/A	N/A	N/A

Abbreviations: ANV/3TC/TDF, ainuovirine/lamivudine/tenofovir disoproxil fumarate; BSL, baseline; N/A, not applicable; NNRTI, non‐nucleoside reverse transcriptase; NRTI, nucleoside reverse transcriptase; RACER‐EXT, **R** esearch of **A** inuovirine‐ **C** ontrolled by **E** favirenz‐based antiretroviral **R** egimen, extensional; RAMs, resistance‐associated mutations; Random., randomization; RNA, ribonucleic acid; SPRINT, **S** witch **P** eople with HIV‐1 to **R** eceive **I** nnovative **N** on‐nucleoside reverse transcriptase inhibitor‐based **T** herapy; TND, target not detected; WT, wild type.

^a^Baseline was defined as the time of initiating efavirenz‐based antiretroviral treatment for the RACER‐EXT study.

^b^Post‐treatment was defined as the time of initiating efavirenz‐ and/or switching to ainuovirine‐based antiretroviral treatment.

^c^Premature withdrawal due to lack of efficacy.

^d^Premature withdrawal due to non‐efficacy reasons and/or with missing data within the time window while still on treatment. The underlined fonts indicate persistence of pre‐existing mutations, and the italic bold fonts indicate newly emerging mutations.

**Conclusions**: High proportion of virological suppression was achieved in PWH switching to ANV/3TC/TDF from previous efavirenz‐ or other NNRTI‐based ARV regimens. Virological failure, including premature withdrawal due to lack of efficacy, and documented resistance‐associated mutations were very occasionally (<1%) reported in PWH switch to ANV/3TC/TDF.

### P053 Predictive efficacy of dual therapy combining integrase strand transfer inhibitors with second‐generation non‐nucleoside reverse transcriptase inhibitors following HIV‐1 treatment failure in Cameroon: potential implications for the use of long‐acting therapeutic strategy in low‐ and middle‐income countries

Davy Hyacinthe Gouissi Anguechia ^1^, Yagai Bouba^1^, Ezechiel Ngoufack Jagni Semengue^1^, Desire Takou^1^, Aude Ka'e^1^, Collins Ambe Chenwi^1^, Grace Beloumou^1^, Alexis Ndjolo^2^, Nicaise Ndembi^3^, Vittorio Colizzi^4^, Carlo‐Federico Perno^5^, Joseph Fokam^1^

^1^Virology, Chantal Biya International Reference Centre for Research on HIV/AIDS Prevention and Management (CIRCB), Yaounde, Cameroon, ^2^General Directorate, Chantal Biya International Reference Centre for Research on HIV/AIDS Prevention and Management (CIRCB), Yaounde, Cameroon, ^3^Research Strategy, Africa Centres for Disease Control and Prevention, Addis Ababa, Ethiopia, ^4^Immunology, University of Rome Tor Vergata, Rome, Italy, ^5^Microbiology, University of Rome Tor Vergata, Rome, Italy

**Background**: Lifetime antiretroviral therapy (ART) for HIV has created interest in dual cART regimens to minimize cumulative drug exposure and toxicities. The use of dual therapy (DT) combining integrase strand transfer inhibitors (INSTIs) with second‐generation non‐nucleoside inhibitors (2gen‐NNRTIs) offers new possibilities for HIV treatment after long exposure to nucleoside analogues. However, the high rate of HIV resistance to NNRTIs in our context may compromise the effectiveness of this therapeutic strategy. Our objective was to describe the genotypic susceptibilities of HIV‐1 to dual therapy combining NNRTIs/INSTIs in HIV‐infected individuals in virological failure.

**Materials and methods**: A laboratory‐based study with 130 patients experiencing virological failure was carried out at the Chantal Biya International Reference Centre (CIRCB), Yaoundé‐Cameroon. We genotyped HIV‐1 Reverse transcriptase (RT) and integrase (INT) gene by Sanger sequencing and assessed acquired HIV‐1 drug resistance (ADR) mutations. We characterized the effect of ADR mutations on the predicted susceptibility to dual therapy combining second‐generation NNRTIs/INSTIs using Stanford HIVdb algorithm. Statistical comparison was performed using the chi2 and Fisher test.

**Results**: Among the 130 patients included, 50.8% failing 2NRTI+PI/r, 40.0% 2NRTI+NNRTI and 9.2% 2NRTI+INSTIs regimen. Median (IQR) ART‐duration and viraemia were 84 (42–144) months and 35,078 (5746–178,550) copies/ml, respectively. 56.2% were infected with CRF02_AG, followed by A1 (13.1%). The presence of at least one NNRTIs or INSTIs RAMs was 92.3% and 1.5%, respectively. The prevalence major RAM was Y181C (32.3%) in 2gen‐NNRTI and R263K (0.7%) in INSTIs. Among 2gen‐NNRTI, 43.85%, 41.54% and 38.46% conserved efficacy of ETR, DOR and RPV, respectively. For INSTIs, 97.69% efficacy was observed for BIC and DTG, 96.15% for CAB and 92.31% for EVG and RAL. Regarding the predictive efficacy of dual therapy, ETR plus DTG or BIC showed the highest scoring (43.8%). Efficacy was significantly lower among patients who failed first‐generation NNRTI‐based ART (*p* < 0.01). Susceptibility to 2gen‐NNRTIs+INSTIs was independently associated with age, ART‐duration and viral subtype (*p* > 0.05).

**Conclusions**: There are high levels of NNRTIs‐RAMs and low level of INSTI‐RAMs among patients failing ART in Cameroon. Consequently, the efficacy of dual therapy combining INSTIs and 2gen‐NNRTIs might be suboptimal for most patients with history of ART failure.

### P054 RUMBA's week 144 results confirm reassuring metabolic outcomes in both DTG/3TC and B/FTC/TAF

Sophie Degroote ^1^, Evy Blomme^2^, Liesbeth Delesie^1^, Linos Vandekerckhove^1^, Marie‐Angélique De Scheerder^1^

^1^General Internal Medicine, Ghent University Hospital, Ghent, Belgium, ^2^Internal Medicine and Paediatrics, Ghent University, Ghent, Belgium

**Background**: Three‐drug antiretroviral regimens (3DR) have been the golden standard in HIV treatment for decades. However, with the introduction of new potent and well‐tolerated antivirals, two‐drug regimens (2DR) have become a valid alternative. Modern ART, predominantly containing integrase inhibitors and/or tenofovir alafenamide (TAF), can be associated with weight gain and metabolic changes. Long‐term ART should, therefore, be investigated beyond viral load. The RUMBA trial secondary and exploratory outcomes investigated the metabolic effect of switching to 2DR DTG/3TC or switching to/staying on 3DR B/FTC/TAF.

**Materials and methods**: RUMBA is a phase IV, longitudinal single‐centre study. PLHIV were randomized 2:1 to switch to DTG/3TC or to switch or stay on B/FTC/TAF. Clinical parameters (weight, waist circumference) as well as lipids and insulin resistance (HOMA‐IR) were measured at baseline and every 6 months up to week (W) 144. Dual‐energy X‐ray absorptiometry (DXA) scans and fibro scans were performed at baseline, W48 and 144 to assess body composition and liver fibrosis, respectively. We report the differences between W144 and baseline, adjusted for baseline ART regimen, BMI and response value, and for baseline statin use for lipid outcomes.

**Results**: A total of 134 PLHIV were randomized at T0 and data were available for 103 participants at W144 (73 DTG/3TC; 30 B/FTC/TAF). Complete case analyses revealed no significant differences between the groups with regard to weight, waist circumference, lipids (total and LDL cholesterol, triglycerides), HOMA‐IR, body composition and liver fibrosis. In both groups, lipids decreased over time. Total cholesterol decreased by 11% in both DTG/3TC [CI 7–15] and B/FTC/TAF [5–17]. LDL‐cholesterol decreased by 14% [8–20] in DTG/3TC and by 12% in B/FTC/TAF [2–20]. None of the groups showed a significant weight increase over time.

**Conclusions**: Staying on or switching to B/FTC/TAF or switching to DTG/3TC did not appear to have a negative impact on metabolic outcomes in the RUMBA trial after 144 weeks follow‐up. Moreover, we show stable and for lipids even beneficial evolutions over time, which could potentially be attributed to an increased focus on lifestyle and statin use. These results confirm long‐term metabolic safety of DTG/3TC and B/FTC/TAF in a real‐world setting.

### P055 Immunological efficacy and early response in virologically suppressed people with HIV‐1 switching to tenofovir DF‐containing, ainuovirine‐based compared to tenofovir alafenamide‐containing, boosted elvitegravir‐based antiretroviral regimen: secondary immunological efficacy analyses of the SPRINT trial, a randomized, active‐controlled phase III study

Hao Wu^1^, Weiping Cai^2^, Ping Ma^3^, Qingxia Zhao^4^, Hongxia Wei^5^, Hongzhou Lu^6^, Hui Wang^7^, Shenghua He^8^, Zhu Chen^9^, Yaokai Chen^10^, Min Wang^11^, Fujie Zhang^12^, Wan Wan^13^, Heliang Fu^13^, Hong Qin ^13^

^1^Clinical and Research Center for Infectious Diseases, Beijing Youan Hospital, Capital Medical University, Beijing, China, ^2^Infectious Disease Center, Guangzhou Eighth People's Hospital, Guangzhou Medical University, Guangzhou, China, ^3^Department of Infectious Diseases, Tianjin Second People's Hospital, Tianjin, China, ^4^Department of Infectious Diseases, The Sixth People's Hospital of Zhengzhou, Zhengzhou, China, ^5^Department of Infectious Diseases, The Second Hospital of Nanjing, Nanjing, China, ^6^National Clinical Research Centre for Infectious Diseases, The Third People's Hospital of Shenzhen, The Second Affiliated Hospital of Southern University of Science and Technology, Shenzhen, China, ^7^Department of Infectious Diseases, Shenzhen Third People's Hospital, Shenzhen, China, ^8^Department of Infectious Diseases, Public Health Clinical Medical Center of Chengdu, Chengdu, China, ^9^Drug Clinical Trial Institution, Public Health Clinical Medical Center of Chengdu, Chengdu, China, ^10^Division of Infectious Diseases, Chongqing Public Health Medical Center, Chongqing, China, ^11^Institute of HIV/AIDS, The First Hospital of Changsha, Changsha, China, ^12^Clinical and Research Center for Infectious Diseases, Beijing Ditan Hospital Capital Medical University, Beijing, China, ^13^Clinical Research & Development, Jiangsu Aidea Pharmaceutical Co., Ltd, Yangzhou, China

**Background**: ACC008, a novel fixed‐dose combination (FDC), contains ainuovirine (ANV), a new‐generation NNRTI, plus lamivudine (3TC) and tenofovir DF (TDF) backbone as complete antiretroviral treatment (ART) regimen. In the SPRINT trial, switch to ACC008 showed a virological efficacy at week 48 non‐inferior to that to cobicistat‐boosted elvitegravir (EVG/c) plus emtricitabine (FTC) and tenofovir alafenamide (TAF) (comparator) in people with HIV‐1 (PWHs) virologically suppressed on previous NNRTI‐based regimen. We herein reported secondary immunological efficacy endpoints at week 48 as prespecified.

**Methods**: Virologically suppressed adult PWHs (*n* = 762) were randomized to ACC008 (*n* = 381) or comparator arm (*n* = 381) for 48 weeks. Primary immunological efficacy endpoint was absolute change from baseline (CFB) in CD4^+^ cell count at week 48. Other supportive immunological efficacy endpoints were absolute CFBs in CD4^+^ cell count at weeks 4 and 12, and proportions of PWHs with CD4^+^ cell count absolute increase ≥100 cells/µl or percentage increase ≥30% at weeks 4 and 12 from baseline (early immunological response).

**Results**: The two arms had a comparable CD4^+^ cell count (617.3±11.9 vs. 626.4±10.6 cells/mm^3^) at baseline, with few PWHs with baseline count <200 cells/mm^3^ (*n* = 1 for each arm). At week 48, absolute CFBs in CD4^+^ cell count were similar between the two arms (least square mean, 0.24 vs. 5.10 cells/mm^3^; mixed model for repeated measures with data‐as‐observed, estimated treatment difference [95% CI] −4.86 [−25.4, 15.7], *p* = 0.630) (Figure 1a). Both arms showed a small‐magnitude decrease at week 4 (−14.9±6.9 vs. −29.3±6.9 cells/mm^3^, analysis of covariance, *p* = 0.144) but continued to increase through 12 weeks (6.4±7.4 vs. −12.4±7.4 cells/mm^3^, *p* = 0.073) and onwards. Early immunological response rates were similar at week 4 between the two arms (14.8% vs. 13.5%, *p* = 0.592), but significantly higher in ACC008 arm compared to that in comparator arm at week 12 (22.4% vs. 15.5%, *p* = 0.015) (Figure 1b).

**P055: Figure 1:**
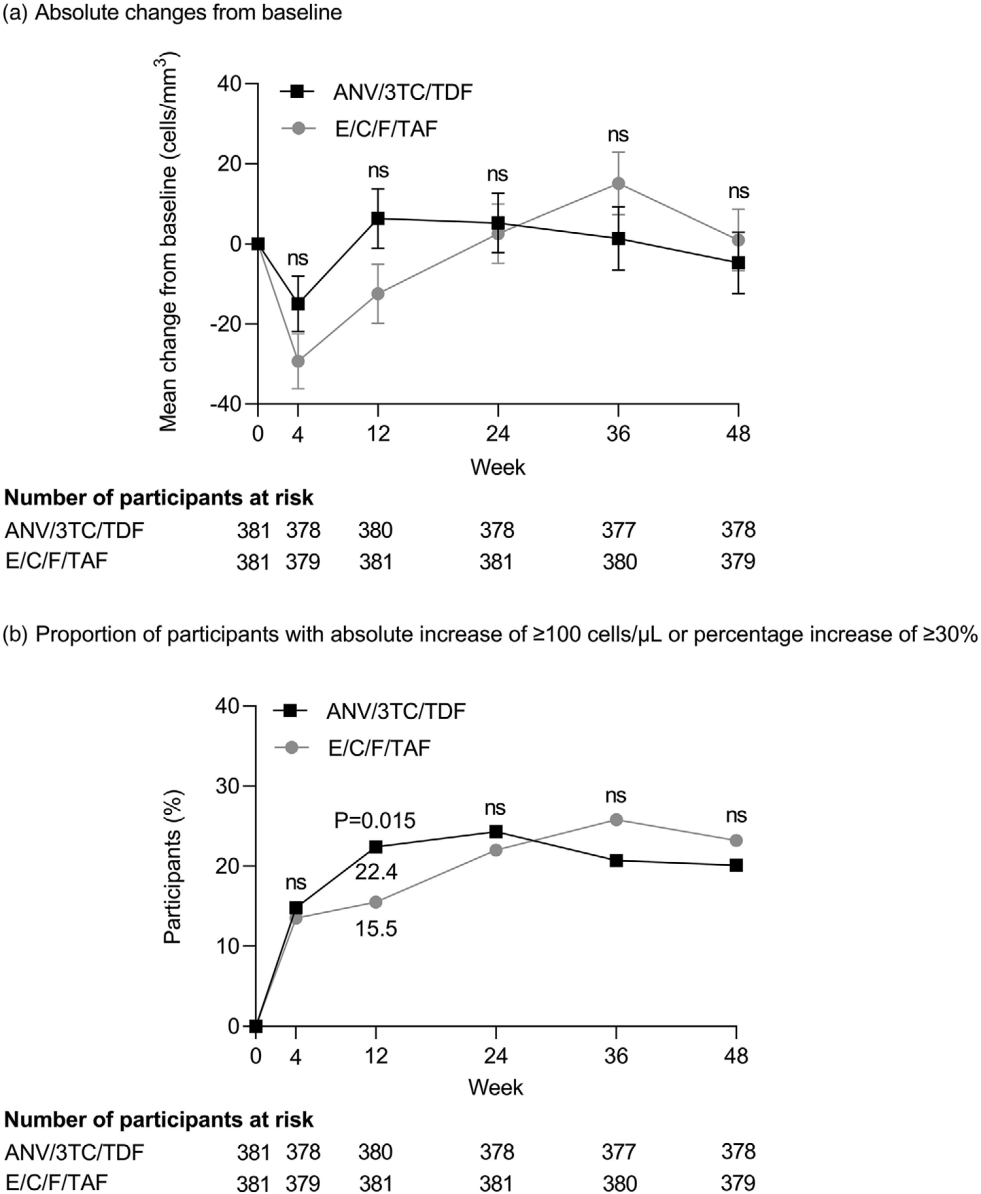
Changes from baseline in CD4^+^ cell count over 48 weeks. (a) Absolute changes from baseline; (b) proportion of participants with absolute increase of ≥100 cells/µl or percentage increase of ≥30%. ANV/3TC/TDF, ainuovirine/lamivudine/tenofovir disoproxil; E/C/F/TAF, elvitegravir/cobicistat/emtricitabine/tenofovir alafenamide. Data in (a) are presented in least square mean ± standard error.

**Conclusions**: Immunological efficacy was comparable between virologically suppressed PWHs, switching to ANV/3TC/TDF and those to EVG/c/FTC/TAF, few of whom were severely immunocompromised at baseline. However, switch to ANV/3TC/TDF resulted in a favourable early immunological response within the first 12 weeks of switch, compared to that to EVG/c/FTC/TAF.

### Treatment strategies—Real world and implementation science studies oral and injectable therapy

### P056 Real‐world outcomes of cabotegravir and rilpivirine for treating PLHIV in Spain: a multicentre, ambispective and nationwide study (the RELATIVITY cohort)

Luis Buzon Martin ^1^, María Luisa Montes^2^, Maria J. Galindo Puerto^3^, Miguel Torralba González^4^, Guillermo Pousada^5^, Mireia Santacreu^6^, Ignacio de los Santos^7^, Alfonso Cabello Úbeda^8^, Noemi Cabello Clotet^9^, María José Crusells‐Canales^10^, Luis Enrique Morano^5^, Patricia Martin Rico^11^, Carmen Montero Hernández^12^, Alberto Diaz de Santiago^13^, Álvaro Cecilio^14^, Miguel Alberto de Zárraga Fernández^15^, Enrique Bernal^16^, María Antonia Sepúlveda^17^, María Jesús Vivancos Gallego^18^, Roberto Pedrero Tomé^19^, Mar Masiá Canuto^20^, Ruth Calderón Hernáiz^21^, Cristina Diez Romero^22^, Juan Emilio Losa García^23^, Manuel Gutiérrez Cuadra^24^, Jara Llenas‐García^25^, Ana Cerezales Calviño^26^, Antonio Sánchez Guirao^27^, Josefa Soler González^28^, Miriam Estébanez^29^, Beatriz de la Calle Riaguas^30^, María Ángeles Garcinuño Jiménez^31^, Maria del M. García Navarro^32^, Noemi Ramos Vicente^33^, Marta Clavero Olmos^34^, Miguel Egido Murciano^35^, Eva Ferreira Pasos^36^, Jesús Troya García^19^

^1^Infectious Diseases, Hospital Universitario de Burgos, Burgos, Spain, ^2^Infectious Diseases, Hospital Universitario La Paz, Madrid, Spain, ^3^Infectious Diseases, Hospital Clínico Universitario de Valencia, Valencia, Spain, ^4^Infectious Diseases, Hospital Universitario de Guadalajara, Guadalajara, Spain, ^5^Infectious Diseases, Hospital Universitario Álvaro Cunqueiro, Vigo, Spain, ^6^Infectious Diseases, Hospital Universitario 12 de Octubre, Madrid, Spain, ^7^Infectious Diseases, Hospital Universitario de la Princesa, Madrid, Spain, ^8^Infectious Diseases, Hospital Universitario Fundación Jiménez Diaz, Madrid, Spain, ^9^Internal Medicine, Hospital Clínico San Carlos, Madrid, Spain, ^10^Infectious Diseases, Hospital Clínico Universitario Lozano Blesa, Zaragoza, Spain, ^11^Internal Medicine, Hospital de Denia, Denia, Spain, ^12^Internal Medicine, Hospital Universitario de Torrejón, Torrejón, Spain, ^13^Infectious Diseases, Hospital Universitario Puerta de Hierro, Madrid, Spain, ^14^Infectious Diseases, Hospital Universitario Miguel Servet, Zaragoza, Spain, ^15^Internal Medicine, Hospital Universitario San Agustín, Avilés, Spain, ^16^Infectious Diseases, Hospital General Universitario Reina Sofia, Murcia, Spain, ^17^Internal Medicine, Hospital Universitario de Toledo, Toledo, Spain, ^18^Infectious Diseases, Hospital Universitario Ramón y Cajal, Madrid, Spain, ^19^Infectious Diseases, Hospital Universitario Infanta Leonor, Madrid, Spain, ^20^Infectious Diseases, Hospital General Universitario de Elche, Elche, Spain, ^21^Internal Medicine, Hospital Universitario de Fuenlabrada, Fuenlabrada, Spain, ^22^Infectious Diseases, Hospital Universitario Gregorio Marañón, Madrid, Spain, ^23^Infectious Diseases, Hospital Universitario Fundación de Alcorcón, Alcorcón, Spain, ^24^Infectious Diseases, Hospital Universitario Marqués de Valdecilla, Santander, Spain, ^25^Infectious Diseases, Hospital Comarcal de la Vega Baja, Alicante, Spain, ^26^Infectious Diseases, Hospital Universitario Doctor José Molina Orosa, Las Palmas, Spain, ^27^Internal Medicine, Hospital General Universitario Morales Meseguer, Murcia, Spain, ^28^Infectious Diseases, Hospital Universitario de Cabueñes, Gijón, Spain, ^29^Infectious Diseases, Hospital Central de la Defensa Gómez Hulla, Madrid, Spain, ^30^Clinical Pharmacy, Hospital General Nuestra Señora de Prado, Talavera de la Reina, Spain, ^31^Internal Medicine, Complejo Asistencial de Ávila, Ávila, Spain, ^32^Internal Medicine, Hospital Universitario de Vinalopo, Alicante, Spain, ^33^Internal Medicine, Hospital Obispo Polanco, Teruel, Spain, ^34^Internal Medicine, Hospital Universitario Infanta Elena, Madrid, Spain, ^35^Internal Medicine, Hospital Universitario San Jorge, Huesca, Spain, ^36^Internal Medicine, Complejo Asistencial de Segovia, Segovia, Spain

**Background**: Randomized clinical trials and several real‐life cohorts have provided evidence regarding non‐inferiority of long‐acting intramuscular cabotegravir (CAB) and rilpivirine (RPV) compared to standard oral ART. In this context, the RELATIVITY cohort aims to evaluate the efficacy, safety and durability of CAB and RPV in Spain where drugs are available since December 2022.

**Methods**: The RELATIVITY cohort is an ambispective cohort evaluating PLHIV treated at 37 Spanish hospitals. All PLHIV older than 18 years who received the first dose of treatment outside a clinical trial context before 1 January 2024, were retrospectively included after signing an informed consent, and prospectively followed up.

**Results**: As of today, 1418 PLHIV have been recruited, and 1204 analysed (84.91%). 85.0% were male, median age was 45 (37–55) years and 71.1% were Spanish. 5.6% underwent oral leading. 79.1% switched from INSTI‐based regimens (DTG [57.8%] and BIC [21.3%]), with backbones consisting primarily of FTC/TAF (42.5%), 3TC (42.4%) and RPV (32.6%). At baseline, 1.7% of patients had a viral load >50 copies/ml. The CD4 nadir was 336.0 [195.0–484.0]. Thirteen percent were C3 stage. Baseline CD4^+^ count was 774.0 [591.5–999.5]. The median follow‐up duration was 6.6 [3.2–9.1] months. Undetectable viral load ranged between 95.6% and 100% throughout the entire follow‐up period (Figure 1). Only three patients have experienced virological failure as of today (two at 7 months and one at 9 months), with RAMs affecting INSTI and NNRTI detected in one case. Other reasons for discontinuation included local injection site reactions (17) and systemic adverse effects (six).

**P056: Figure 1:**
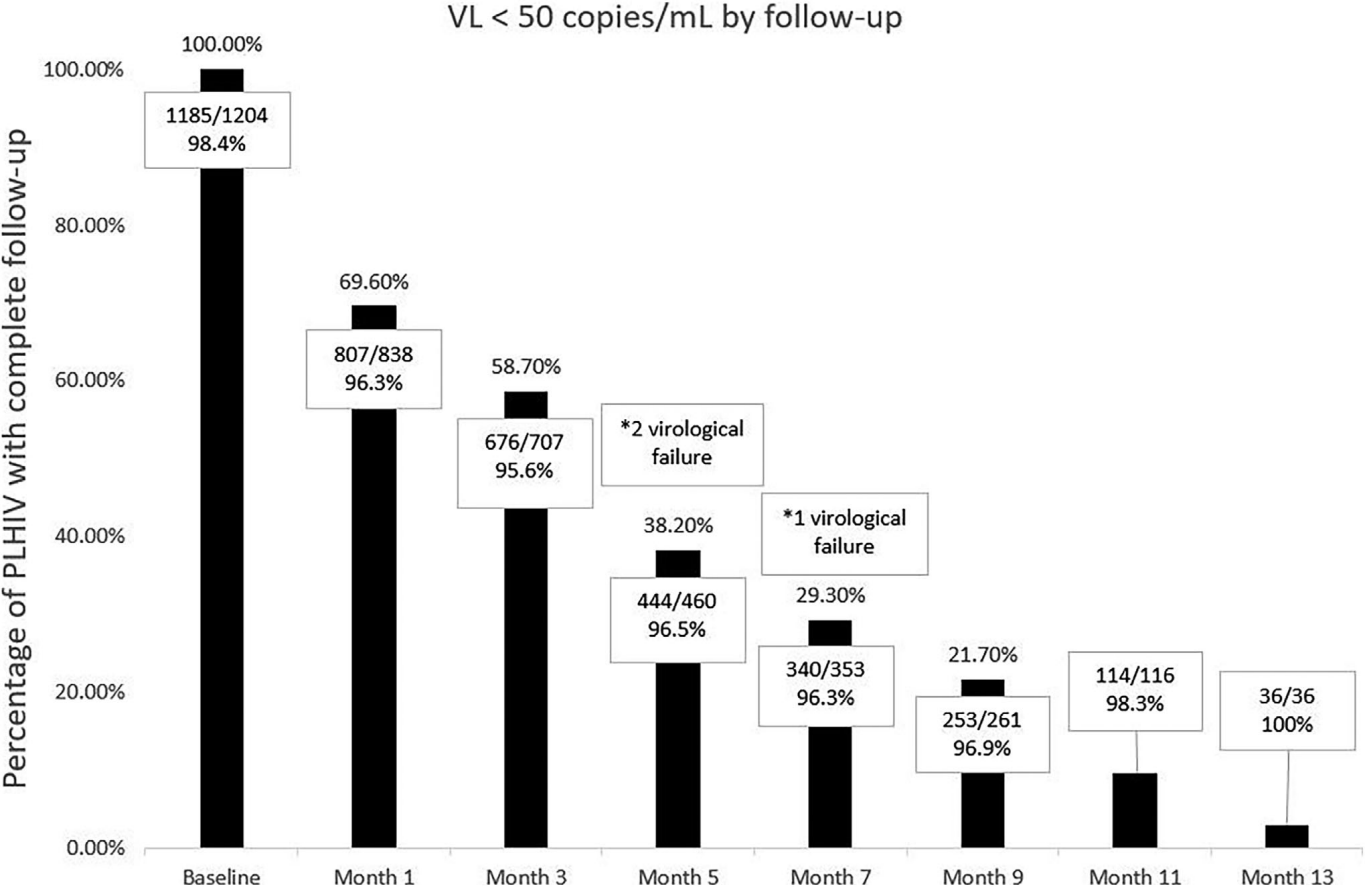
Percentage of PLHIV with complete follow‐up and VL <50 copies/ml at different months.

**Conclusions**: The RELATIVITY cohort lines up with the results provided by randomized clinical trials and other real‐life cohorts (ATHENA, OPERA, CARLOS) in terms of efficacy, tolerability, durability and virological failures. Longer follow‐up is required to improve our knowledge regarding CAB/RPV.

### P057 Antiretroviral treatment in PLWH with late diagnosis initiating ART with DTG/3TC or BIC/TAF/FTC: a real‐world cohort analysis

Gundolf Schuettfort ^1^, Alfonso Cabello^2^, Maria Crusells‐Canales^3^, Miguel Górgolas Hernández‐Mora^2^, Carmen Hidalgo Tenorio^4^, Juan Lopez^5^, Rafael Mican^6^, Roser Navarro‐Soler^7^, Eugenia Negredo^8^, Sebastian Noe^9^, Jordi Puig^8^, Jürgen Rockstroh^10^, Sergio Rodriguez^11^, Rosario Serrao^12^, Christoph Stephan^1^, Miguel Torralba^13^, Diva Trigo^14^, Maria Vivancos^15^, Annette Haberl^1^

^1^Infectious Diseases, University Hospital Frankfurt, Frankfurt, Germany, ^2^Infectious Diseases, Hospital Universitario Fundacion Jimenez Diaz, Madrid, Spain, ^3^Infectious Diseases, Hospital Universitario Lozano Blesa, Zaragoza, Spain, ^4^Infectious Diseases, Hospital Universitario Virgen de las Nieves, Granada, Spain, ^5^Infectious Diseases, Hospital Universitario Gregorio Maranon, Madrid, Spain, ^6^Infectious Diseases, Hospital Universitario La Paz, Madrid, Spain, ^7^Infectious Diseases, Hospital Universitario 12 de Octubre, imas12, Madrid, Spain, ^8^Infectious Diseases, Hospital Universitario Germans Trias i Pujol, Barcelona, Spain, ^9^Infectious Diseases, MVZ München am Goetheplatz, München, Germany, ^10^Infectious Diseases, University Hospital Bonn, Bonn, Germany, ^11^Infectious Diseases, Hospital Universitario Getafe, Madrid, Spain, ^12^Infectious Diseases, Centro Hospitalar de Sao Joao, Sao Joao, Portugal, ^13^Infectious Diseases, Hospital Universitario Guadalajara, Guadalajara, Spain, ^14^Infectious Diseases, Hospital Professor Doutor Fernando Fonseca, Lisbon, Portugal, ^15^Infectious Diseases, Hospital Universitario Ramon y Cajal, Madrid, Spain

**Background**: Active opportunistic infections and/or low CD4^+^ T‐cell (CD4^+^) counts are exclusion criteria in most clinical trials. People living with HIV (PLWH) and late diagnosis are, therefore, inadequately represented in studies assessing the efficacy of antiretroviral treatment (ART) regimens. There are currently no data from European studies solely focusing on virological outcomes in treatment naïve PLWH with baseline CD4 count ≤200/µl comparing dolutegravir/lamivudine (DTG/3TC) and bictegravir/tenofovir alafenamide/emtricitabine (BIC/TAF/FTC). Aim of the presented study was to obtain information on the effectiveness and tolerability/safety of DTG/3TC in treatment naïve PLWH with a CD4 cell count ≤200/µl. Results were compared with data from treatment naïve PLWH with a CD4 cell count <200/µl treated with BIC/TAF/FTC.

**Methods**: Retrospective, multicentre, multinational study with 15 investigational sites in Germany, Spain and Portugal. Primary objective was effectiveness of initial ART with DTG/3TC compared to BIC/TAF/FTC in patients with low CD4 counts at baseline (<200/µl). Primary endpoint was proportion of PLWH with HIV‐1 RNA <50 copies/ml 48 weeks after treatment initiation treated with either DTG/3TC or BIC/TAF/FTC. All PLWH with CD4 <200/µl and/or an AIDS‐defining disease starting first‐line ART with DTG/3TC or BIC/TAF/FTC between July 2019 and September 2022 were included in this study. Statistical analysis was performed after matching the study participants 1 to 1 regarding age, gender, CDC status, baseline CD4‐count and HIV viral load. Virological response was analysed using FDA Snapshot analysis (HIV‐1 RNA <50 copies/ml at week 48).

**Results**: A total of 259 PLWH were included in the study. Sixty‐nine PLWH were started on two‐drug regimen (2DR) DTG/3TC and 190 on three‐drug regimen (3DR) BIC/TAF/FTC. After matching 1 to 1, 138 PLWH were considered for analysis (*n* = 69 for each regimen). Mean baseline CD4 count was 98/µl (SD 43) and 24.6% presented with CDC C3 status. 97.1% and 94.2% of PLWH on 2DR and 3DR, respectively, had a viral load <50 copies/ml at week 48. Discontinuation rates were 5.8% in the 2DR and 7.2% in the 3DR group.

**Conclusions**: In a European cohort of PLWH and late diagnosis starting first‐line ART with DTG/3TC or BIC/TAF/FTC, there were no significant differences in discontinuation rates or virological response rates at week 48.

### P058 Long‐acting cabotegravir plus lenacapavir as a fully injectable maintenance antiretroviral regimen in people with HIV with adherence issues

Romain Palich ^1^, Romain Manchon^2^, Jérémy Zeggagh^2^, Elisabete Gomes‐Pires^2^, Sophie Seang^1^, Marc‐Antoine Valantin^1^, Marc Wirden^3^, Marianne Burgard^4^, Gilles Peytavin^5^, Claudine Duvivier^2^

^1^Infectious Diseases, Sorbonne University, Pitié‐Salpêtrière Hospital, AP‐HP, Paris, France, ^2^Infectious Diseases, Paris Cité University, Necker Hospital, AP‐HP, Paris, France, ^3^Virology, Sorbonne University, Pitié‐Salpêtrière Hospital, AP‐HP, Paris, France, ^4^Virology, Paris Cité University, Necker Hospital, AP‐HP, Paris, France, ^5^Toxicology‐Pharmacology, Paris Cité University, Bichat‐Claude Bernard Hospital, AP‐HP, Paris, France

**Background**: Long‐acting injectable (LAI) antiretroviral therapy (ART) represents a breakthrough in managing HIV, providing an alternative to daily oral ART, especially for PLWH with adherence challenges. However, the use of LAI‐cabotegravir (CAB) in association with LAI‐rilpivirine (RPV) is contraindicated in PLWH with previous RPV‐associated resistance mutations. LAI‐lenacapavir (LEN) may help address barriers to treatment adherence among PLWH with RPV‐resistant virus.

**Methods**: In this series, we report on eight pretreated virally suppressed (plasma viral load [pVL] <50 copies/ml) adult PLWH with RPV‐resistant virus, who started LAI‐ART with CAB plus LEN between January 2021 and August 2023, after approval by a multidisciplinary committee in two French hospitals. CAB and LEN were started on the same day: oral loading dose of LEN 600 mg on day 1 and day 2, and subcutaneous LEN 927 mg on day 1 and then every 6 months, in combination with intramuscular CAB 600 mg on day 1, week 4, and then every 8 weeks. Antiretroviral plasma concentrations (C_pl_) were routinely determined by UPLC‐MS/MS at each visit.

**Results**: Patients were four women and four men; median age (IQR 25–75) 56 years (44–58); duration from ART initiation 25 years (18–32); duration of viral suppression 32 months (7–59); four had CD4 counts below 200/mm^3^ (Table 1). All had difficulty accepting their illness and had adherence problems. All patients were monitored for at least 6 months, and three for 12 months, with a median of 3 pVL measurements per patient (range 1–4). No virological failures were observed during follow‐up, as all pVL remained below 50 copies/ml. No serious adverse events or discontinuations were reported. All LEN trough C_pl_ were >15.5 ng/ml (4xPA‐IC95 in MT‐4 cells) and median (IQR 25–75) CAB C_pl_ was 1829 ng/ml (1483–2166) approximately 58 days after the last intramuscular injection. Despite the expected moderate injection site reactions, all patients expressed a preference for this treatment over oral ART.

**P058: Table 1:** Clinical, immunological and virological characteristics of the eight patients who switched to long‐acting injectable cabotegravir plus lenacapavir

Patient	#1	#2	#3	#4	#5	#6	#7	#8
Gender	Female	Female	Female	Male	Male	Male	Male	Female
Age	39 years	41 years	66 years	36 years	58 years	57 years	63 years	54 years
Body mass index (kg/m^2^)	24	23	22	18	23	30	28	29
Transmission mode	Mother to child	Mother to child	Intravenous drug use	Sex with men	Intravenous drug use	Sex with men	Sex with women	Sex with men
CD4 T‐cell nadir	1 cell/l	1 cell/l	5 cells/l	537 cells/l	25 cells/l	109 cells/l	5 cells/l	128 cells/l
Time from ART initiation	35 years	33 years	31 years	5 years	28 years	21 years	20 years	13 years
Cumulated duration of HIV plasma viral load 200 copies/ml from ART initiation	144 months	32 months	76 months	2 months	18 months	43 months	204 months	29 months
HIV subtype	B	A1	B	B	B	B	CRF02AG	CRF02‐AG
RPV‐associated resistance mutations	E138K	L100I; K103N; V179I; G190E; P225H; P236L	A98S; K103N; V106A; V179I; Y181C; G190A	M230I	K101E; G190A	K101E; G190A	E138A	A98S; K101E; G190A; M230I
Prior ART before switch	BIC/FTC/TAF	BIC/FTC/TAF+DTG	bDRV+RPV	RPV/FTC/TAF	bEVG/FTC/TAF	bDRV+RPV	BIC/FTC/TAF	DTG/3TC
HIV plasma viral load at time to switch	20 copies/ml	20 copies/ml	20 copies/ml	51 copies/ml	20 copies/ml	20 copies/ml	54 copies/ml	20 copies/ml
Duration of viral suppression (HIV plasma viral load 50 copies/ml) prior switch	60 months	8 months	58 months	3 months	182 months	20 months	1 month	44 months
CD4 T‐cell count	88 cells/l	54 cells/l	383 cells/l	886 cells/l	406 cells/l	714 cells/l	124 cells/l	128 cells/l
Reasons for adherence difficulties with oral ART	Feeling of injustice about having been infected with HIV at birth; disgust and nausea when taking ART	Non‐acceptance of illness; fear of stigmatization; dysphagia and difficulty swallowing pills	Major depressive disorders, social loneliness (and drug interactions with breast cancer therapy)	Non‐acceptance of illness; fear of stigmatization	Unweaned IV drug addiction	Major depressive disorders	Non‐acceptance of illness; stigmatization; socio‐economic precarity	Non‐acceptance of illness; stigmatization; socio‐economic precarity

Abbreviations: PA‐IC95, protein‐adjusted IC95; UPLC‐MS/MS, ultra‐performance liquid chromatography and tandem mass spectrometry.

**Conclusions**: CAB plus LEN maintained effective viral suppression with good tolerability. It holds great promise for vulnerable PLWH struggling with oral ART adherence, particularly when RPV is not an option anymore, and merits prospective evaluation in a large, randomized trial.

### P059 Real‐world experience of DTG+3TC regimen: results from the French Dat'AIDS cohort (2015−2022)

Clotilde Allavena ^1^, Romain Palich^2^, Alain Makinson^3^, Amélie Ménard^4^, David Rey^5^, Laetitia Roustand^6^, Corinne Vouillot^7^, Laurent Hocqueloux^8^

^1^CHU de Nantes, Nantes, France, ^2^Hôpital Pitié Salpétrière, Paris, France, ^3^CHU Montpellier, Montpellier, France, ^4^IHU Marseille, Marseille, France, ^5^CHRU Strasbourg, Strasbourg, France, ^6^GlaxoSmithKline, Rueil Malmaison, France, ^7^ViiV Healthcare, Rueil Malmaison, France, ^8^CHU d'Orléans, Orléans, France

**Background: **Phase III clinical studies (GEMINI 1 and 2; TANGO and SALSA) have shown a high efficacy and tolerability of the dual therapy DTG+3TC both in naïve and maintenance strategies. Few real‐world data of DTG+3TC efficacy and tolerance are available.

**Materials and methods: **Dat'AIDS cohort includes 33 French HIV centres. Adult patients with HIV (PWH) starting DTG+3TC (separate or fixed combination from March 2020) either in first line in ART‐naïve PWH (naive group) or in maintenance therapy in ART‐experienced suppressed PWH (MT group) were retrospectively included in the study between 01/01/2015 and 31/12/2022. Main objectives were to evaluate virological failure (VF) defined by two consecutive viral load (VL) >50 copies/ml or one VL >200 copies/ml and reasons for treatment interruptions.

**Results: **Among the 88,619 PWH followed in the cohort, DTG+3TC was initiated in 252 naïve PWH (3.5%) and 6770 PWH (96.5%) in MT. Baseline patient characteristics are presented in Table 1. On treatment, 96.1% and 98.6% of PWH were virologically suppressed at last control in naïve and MT group, respectively. Ninety‐eight PWH (1.4%) stopped DTG+3TC with a VL >50 copies/ml but only 23 (0.3%) with a confirmed VF. After a median follow‐up of 1.4 year (IQR 0.8–2.1), DTG+3TC was discontinued in 964 PWH (13.7%) mostly for adverse events (7.9% in naïve group, 5.2% in MT), with neuropsychological disorders in 4.4% and 2% of naïve and MT group, respectively. The median weight gain was +3.0 kg (IQR 0.0–5.2) in naïve group and +1.0 kg (IQR −1.0, 3.0) in MT. At VF, 26 genotypes were available and among them four harboured resistance‐associated mutations, one in the naïve group and three in the MT group; M184V on reverse transcriptase was observed in all cases, associated with N155H on integrase in one PWH of the naive group (VL at 5162 copies/ml, 11.2 months after DTG+3TC initiation).

**P059: Table 1:** Baseline patient characteristics

*N* (%) or median (IQR)	Naive PWH (*n* = 252)	ART‐experienced PWH in maintenance (MT) (*n* = 6770)
Male	186 (73.8)	4800 (70.9)
Age (year)	37.0 (28.2−48.6)	52.8 (44−60.4)
CDC stage C	5 (2)	1182 (17.5)
Known HIV infection (year)	0.1 (0.0−1.6)	15.3 (7.7−24.1)
Baseline CD4/mm^3^	492 (357−658)	712 (536−924)
Median HIV RNA (copies/ml)	7250 (991−28,475)	<50
Duration of VL <50 copies/ml (year)	−	8.5 (4.5−13.5)
Duration of ART therapy	−	12.3 (6.6−20.4)
Nb of previous ART line	−	4.0 (2.0−7.0)
Historical genotype with M184V mutation	0 (0)	521 (12.9)
BMI	24.2 (21.8−26.0)	22.9 (21.0−25.1)

**Conclusions: **DTG+3TC combination used in real life in France confirms the results of clinical studies in naïve and virologically controlled PWH. The combination was mainly prescribed in ART‐experienced PWH with prolonged undetectability. Virological failures were infrequent with rare emerging resistance mutations, confirming the robustness of the dual therapy.

### P060 Feasibility, acceptability and effectiveness of long‐acting injectable antiretrovirals in the “Acceptability and Feasibility of long‐acting INjectable ART in Adolescents and Young Adults (AFINAty)” study in South Africa

Lauren Jennings, Millicent Atujuna, Chantel Schreuder, Nyiko Mashele, Metsekae Madimabe, Linda‐Gail Bekker, Catherine Orrell

University of Cape Town, Desmond Tutu HIV Centre, Cape Town, South Africa

**Background**: Young people living with HIV (YLHIV) face many barriers to achieving optimal antiretroviral therapy (ART) adherence, particularly with oral tablets. Long‐acting injectable (LAI) antiretrovirals, like cabotegravir and rilpivirine, are an alternative method of ART delivery that may help to overcome some of these barriers. We assessed the feasibility, acceptability and effectiveness of LAIs delivered in a primary‐care community clinic setting in Cape Town, South Africa.

**Methods**: We conducted a mixed method study in YLHIV aged 12–24. All participants were on first‐line oral ART, enrolled in three cohorts: (1) YLHIV who are virally suppressed and adherent; (2) YLHIV with viraemia or documented adherence challenges; and (3) YLHIV who were ART‐naïve. The 6‐month screening phase allowed for intensive adherence counselling to achieve viral suppression (<50 copies/ml). Once virally suppressed, YLHIV were offered a switch to cabotegravir and rilpivirine LAIs (every 8 weeks). Viral load was measured at weeks 24 and 48 after switching. We conducted in‐depth interviews in a sub‐set of participants to understand the acceptability of LAIs.

**Results**: We screened 179 YLHIV. One hundred and thirty‐four were virally suppressed and switched to LAIs. The median age was 20 years (IQR 18–22) and 85 (63.4%) identified as female. As of 31 May 2024, 97 participants had reached week 24. Of these, 95 (97.9%) were virally suppressed, with one lost to follow‐up and one with low‐level viraemia (<100 c/ml). Of the 24 participants who reached week 48, 21 (87.5%) were virally suppressed and three had low‐level viraemia (<300 c/ml). There was no confirmed virological failure on retest within 4 weeks. There have been no severe drug‐related adverse events or injection site reactions. Data from interviews indicated that LAIs reduced the burden of pill‐taking and daily schedules, provided YLHIV an increased sense of freedom, alleviated anxiety associated with unplanned disclosure and fear of missing doses, and reduced pill‐taking fatigue. All YLHIV interviewed expressed preference for LAI over oral ART.

**Conclusions**: Delivery of long‐acting injectable ART is feasible, acceptable and well‐tolerated in YLHIV in a South African community setting. LAIs address several pill‐taking related barriers to adherence and their use in high‐risk, adherence‐challenged populations should be further explored.

### P061 Durability of doravirine containing regimens in people with HIV in real‐life settings in the ANRS CO3 cohort: AQUIVIH‐NA

Olivier Leleux^1^, Alaric Peyrouny‐Mazeau^1^, Adélaïde Perrier^1^, Mojgan Hessamfar^2^, Gwenaël Le Moal^3^, Didier Neau^4^, Laure Alleman^5^, Charles Cazanave^4^, Estibaliz Lazaro^6^, Pierre Duffau^2^, Agnés Riché^7^, Yann Gérard^8^, Marie‐Anne Vandenhende^9^, Fabrice Bonnet ^2^

^1^Bordeaux Population Health Research Center, INSERM U1219, CIC‐EC 1401, Univ. Bordeaux—ISPED, Bordeaux, France, ^2^Service de Médecine Interne et Maladies Infectieuses, Hôpital Saint‐André, Centre Hospitalier Universitaire (CHU) de Bordeaux, Bordeaux, France, ^3^Service de Maladies Infectieuses et Tropicales, Centre Hospitalier Universitaire (CHU) de Poitiers, Poitiers, France, ^4^Service de Maladies Infectieuses et Tropicales, Hôpital Pellegrin, Centre Hospitalier Universitaire (CHU) de Bordeaux, Bordeaux, France, ^5^Service de Maladies Infectieuses, Centre Hospitalier de la Côte Basque, Bayonne, France, ^6^Service de Médecine Interne et Maladies Infectieuses, Hôpital Haut‐Lévèque, Centre Hospitalier Universitaire (CHU) de Bordeaux, Pessac, France, ^7^Service de Medecine Interne, Centre Hospitalier d'Angoulême, Angoulême, France, ^8^Service de Médecine Interne et Maladies Infectieuses, Centre Hospitalier de Dax, Dax, France, ^9^Service de Médecine Interne, Hôpital Pellegrin, Centre Hospitalier Universitaire (CHU) de Bordeaux, Bordeaux, France

**Background**: Since 2019, people living with HIV (PWH) in France have had access to doravirine (DOR), a recent non‐nucleoside reverse transcriptase inhibitor. The long‐term durability of DOR treatment and the reasons for switching treatments in PWH, whether they have a suppressed viral load or not, is underreported in clinical routine care.

**Methods**: PWH followed in the French regional prospective cohort ANRS‐CO3‐AquiHIV‐NA and switching to a treatment including DOR between 2019/04/01 and 2022/12/31 were included if they had CD4 count and HIV RNA available at that time. Virological failure (VF) was defined as one HIV RNA >1000 cp/ml or two consecutive HIV RNA >50 cp/ml.

**Results**: During the period, 541 PWH had received at least one DOR containing regimen (DORcr) including 466 with suppressed HIV RNA (sRNA), median age was 52.8 years, 34.5% were women, 23% at AIDS stage, median BMI was 25.8 and median CD4 count was 720/mm^3^ [IQR 530–949] at switch time. Among the 75 unsuppressed HIV RNA (usRNA), including six naive; median age was 53.1 years, 30.7% were women, 16% were at AIDS stage, median BMI was 24.8 and median CD4 count was 525/mm^3^ [IQR 277–947], median HIV viral load was 205 cp/ml [IQR 84–5774]. The most frequent treatment prior switch were regimens containing 42.2% TAF and 19.6% DTG. After 12 months of follow‐up, the cumulative probability of discontinuation of DORcr was 22.3% overall [IQR 18.8–25.9%], including 21.3% [IQR 17.7–25.2%] for sRNA and 28.2% [IQR 18.3–39.0%] for usRNA (Figure 1a). The main reasons for DOR discontinuation in sRNA patients and usRNA patients were: side‐effects (34% including one quarter for neurological side‐effects in sRNA and 9.5% in usRNA), person's choice (18.6%; 23.8%), physician's choice (17.5%; 33.3%) and VF (3.1%; 19%), respectively. At month 12, 3.6% [IQR 2.1–5.6%] of PWH had a cumulative probability of VF on DORcr, of which 2.8% [IQR 1.5–4.8%] for sRNA and 8.6% [IQR 3.1–17.7%] for usRNA (Figure 1b).

**P061: Figure 1:**
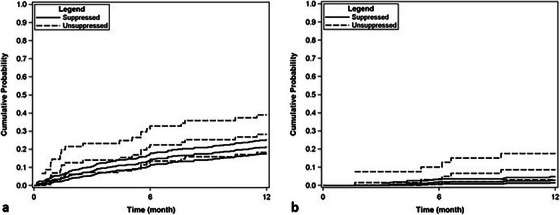
(a) Cumulative probability (Aalen Johansen method) of discontinuation of DOR during the first 12 months according to the unsuppressed/suppressed status at baseline; (b) cumulative probability (Aalen Johansen method) of virological failure under DOR during the first 12 months (censored at discontinuation and last follow‐up) according to the unsuppressed/suppressed status at baseline.

**Conclusions**: In this real‐life cohort, the probability of DORcr discontinuation at month 12 was 22.3%, mainly related to side‐effects. The risk of VF was 2.8% in sRNA patients and 8.6% in usRNA patients.

### P062 Re‐suppression regimens and outcomes after virological failure in randomized controlled trials and real‐world evidence studies evaluating cabotegravir and rilpivirine (CAB+RPV)

Alexa Elias, Melanie Smuk, Kyle Ring, Chloe Orkin

Sexual Health, HIV All East Research (SHARE) Collaborative, Queen Mary University of London, Blizard Institute, London, UK

**Background**: Virological failure (VF) on CAB+RPV treatment is infrequent. However, VF is commonly accompanied by emergent resistance making post‐VF ART regimen choice important. We present the first summary of post‐VF regimen use and re‐suppression outcomes from randomized controlled trials (RCTs) and real‐world evidence (RWE).

**Materials and methods**: We performed a systematic review from 01/2020 to 03/2024 on CAB+RPV use in RWE. We included six RCTs and 27 RWE studies with VF events where CAB+RPV was prescribed according to the FDA label. We synthesized data from a sub‐set of studies summarizing post‐VF regimens and re‐suppression outcomes. We categorized post VF‐regimens as: integrase inhibitor (INI), protease inhibitor (PI), non‐nucleoside reverse transcriptase inhibitor (NNRTI), multi‐core agent, re‐suppression on CAB+RPV, not reported (NR) or no follow‐up data. We report proportion with re‐suppression where known.

**Results**: VF definitions varied widely (Table 1). RCTs: VF events occurred in 30 participants across six trials (ATLAS, ATLAS‐2M, FLAIR, SOLAR, CARES, CARISEL). Post‐VF regimen was described in 28/30 participants (no follow‐up data for 2/30). 78.6% (22/28) of regimens used were PI‐based, 17.9% (5/28) INI‐based and 3.6% (1/28) NNRTI‐based (Figure 1). 92.9% (26/28) re‐suppressed on post‐VF regimens (INI‐based 100% [5/5], NNRTI‐based 100% [1/1] and PI‐based 90.9% [20/22]). RWE: of 27 RWE studies in virally suppressed individuals which reported on VF outcomes, 11 studies also included data on post‐VF regimens used. In those studies, 34/40 individuals had a known post‐VF suppression regimen (1/40 lost‐to‐follow‐up, 5/40 NR). 38.2% (13/34) of post‐VF regimens were INI‐based, 14.7% (5/34) PI‐based, 11.8% (4/34) multi‐core agents and 35.3% (12/34) continued CAB+RPV post‐VF (Figure 1). Of the 28/34 people for whom post‐VF regimen and re‐suppression outcome were known, 96.4% (27/28) achieved re‐suppression. Re‐suppression was correlated to drug class in 8/27 (29.6%): (INI‐based [*n* = 2], PI‐based [*n* = 5], CAB+RPV [*n* = 1]). No correlation with a regimen could be deduced in 19/27 (70.3%) individuals who re‐suppressed post‐VF.

**P062: Table 1:** VF definitions used in RWE studies (*n* = 11)

Definition	Number of studies with each definition
No definition reported	6
HIV VL ≥50 c/ml	2
Two consecutive measurements of VL ≥50 c/ml	1
2 VL ≥200 c/ml or 1 VL ≥200 c/ml followed by discontinuation	1
VL >200 c/ml	1

**P062: Figure 1:**
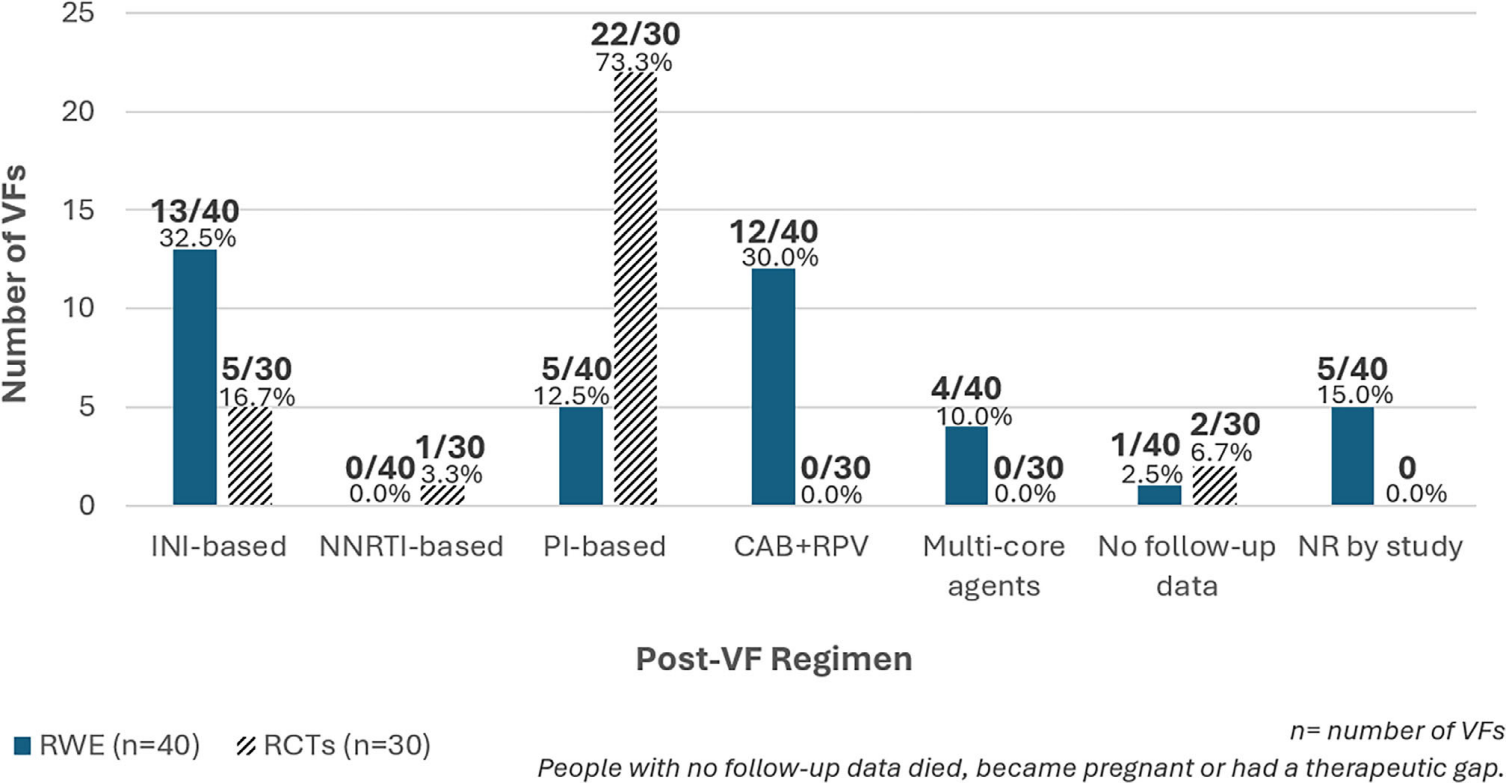
Post‐VF regimens used in RWE and RCTs by class.

**Conclusions**: We summarized post‐VF regimen use and re‐suppression outcomes (where known) in RWE and RCTs evaluating CAB+RPV. In RCTs, PI‐based post‐VF regimens were used most frequently, whereas in RWE, continuation of CAB+RPV or INI‐based regimens were most common. Re‐suppression occurred in >90% of individuals in RCTs. Evidence on re‐suppression outcomes is scant in RWE so far.

### P063 Four‐year outcomes from the BICSTaR study: observational analysis of bictegravir/emtricitabine/tenofovir alafenamide (B/F/TAF) in treatment‐naïve (TN) and treatment‐experienced (TE) people with HIV in Canada, France and Germany

Alex Wong ^1^, Daniel Beer^2^, Claudine Duvivier^3^, Hugues Cordel^4^, Anja Meurer^5^, David Thorpe^6^, Marion Heinzkill^7^, Andrea Marongiu^6^, Johanna Ramroth^6^, Benoit Trottier^8^

^1^Division of Infectious Diseases, University of Saskatchewan, Saskatchewan, Canada, ^2^Praxis/Labor Dr. med. Heribert Knechten, PZB Aachen, Aachen, Germany, ^3^Infectious Diseases Department, AP‐HP‐Necker Hospital, Paris, France, ^4^Maladies Infectieuses et Tropicales, Hopital Avicenne, Bobigny, France, ^5^Internal Medicine and General Medicine, Zentrum für Innere Medizin und Infektiologie, Munich, Germany, ^6^Real World Evidence, Gilead Sciences Europe Ltd, Uxbridge, UK, ^7^Medical Affairs, Gilead Sciences GmbH, Martinsried, Germany, ^8^Clinique de Médecine Urbaine du Quartier Latin, Québec, Canada

**Background**: BICSTaR is an ongoing, multi‐country, prospective, observational cohort study evaluating the effectiveness and safety of B/F/TAF in clinical practice. After 2 years in the main study, eligible participants from Canada, France and Germany could enter a 3‐year extension phase.

**Materials and methods**: Participants were enrolled between Jun 2018 and Aug 2019 (data cut‐off: 01 Sep 2023). Data analysed included HIV‐1 RNA <50 copies/ml (missing = excluded [M = E]/discontinuation = failure [D = F]), safety and patient‐reported outcomes (PROs; SF‐36 and HIV‐SI).

**Results**: Eight hundred participants were included in this analysis (Table 1); 178, 231 and 391 from Canada, France and Germany, respectively; TN 125 (16%), TE 675 (84%). Of these, 465 (58%) completed the main study, were eligible and reconsented for the extension phase (TN 70 [15%], TE 395 [85%]). At 4 years, viral load remained undetectable (HIV‐1 RNA <50 copies/ml) in 98% and 97% (M = E) and 67% and 74% (D = F) of TN and TE participants, respectively (Figure 1). Median changes in CD4 count (cells/µl) and CD4/CD8 ratio were +350.0 and +0.54 (TN) and +95.5 and +0.10 (TE), respectively. Overall, 143 (18%) discontinued B/F/TAF through 4 years. No treatment‐emergent resistance to B/F/TAF was reported. Drug‐related adverse events (DRAEs) and serious DRAEs were reported in 17%/0% (TN) and 14%/0.3% (TE), respectively. Discontinuation of B/F/TAF due to DRAEs occurred in 7% overall, with weight gain being the most commonly reported reason (3%). Median change in body weight at 4 years was +4.4 kg (*p* = 0.019) and +1.6 kg (*p* < 0.001) for TN and TE participants, respectively. At 4 years, overall HIV‐SI scores improved in TN participants (median change [Q1, Q3]: −3 [−6, 1], *p* = 0.053) and remained stable in TE participants (0 [−2, 2], *p* = 0.798). Similarly, SF‐36 mental component summary scores showed improvement in TN but no change in TE participants (median change: +3.3, *p* = 0.030, and +0.7, *p* = 0.225, respectively); SF‐36 physical component summary scores remained stable in both TN and TE participants (−0.1, *p* = 1.000, and −0.2, *p* = 0.569, respectively).

**P063: Table 1:** Participant characteristics at baseline

	TN (*n* = 125)	TE (*n* = 675)
Age, years	40 (31−51)	49 (39−56)
Age ≥50 years	34 (27)	326 (48)
Male sex	112 (90)	585 (87)
Weight, kg^a^	70.0 (65.0−79.8) [*n* = 29]	77.0 (66.5−86.5) [*n* = 269]
BMI, kg/m^2a^	23.0 (21.6−25.2) [*n* = 29]	24.9 (22.3−27.7) [*n* = 269]
Any medical history or comorbidity	76 (61)	552 (82)
Concomitant medication	59 (50) [*n* = 119]	420 (64) [*n* = 659]
HIV‐1 RNA, log_10_ copies/ml	4.83 (4.02−5.36) [*n* = 123]	1.28 (1.28−1.28) [*n* = 608]
CD4 count, cells/µl^a^	436 (240−598) [*n* = 45]	650 (455−884) [*n* = 274]
CD4/CD8 ratio^a^	0.36 (0.19−0.58) [*n* = 44]	0.89 (0.60−1.24) [*n* = 243]
SF‐36: MCS score	45.3 (35.3−52.7) [*n* = 115]	49.8 (38.8−56.2) [*n* = 592]
SF‐36: PCS score	53.4 (48.2−58.4) [*n* = 115]	55.7 (49.8−59.0) [*n* = 592]
HIV‐SI overall bothersome count^a^	6.5 (2.0−9.0) [*n* = 44]	4.0 (1.0−7.0) [*n* = 292]

*Note*: Data shown as *n* (%) or median (Q1, Q3).

Abbreviations: BMI, body mass index; HIV‐SI, HIV Symptom Index; MCS, mental component summary; PCS, physical component summary; Q, quartile; SF‐36, 36‐Item Short Form Health Survey.

^a^Participants with values at baseline and 48 months.

**P063: Figure 1:**
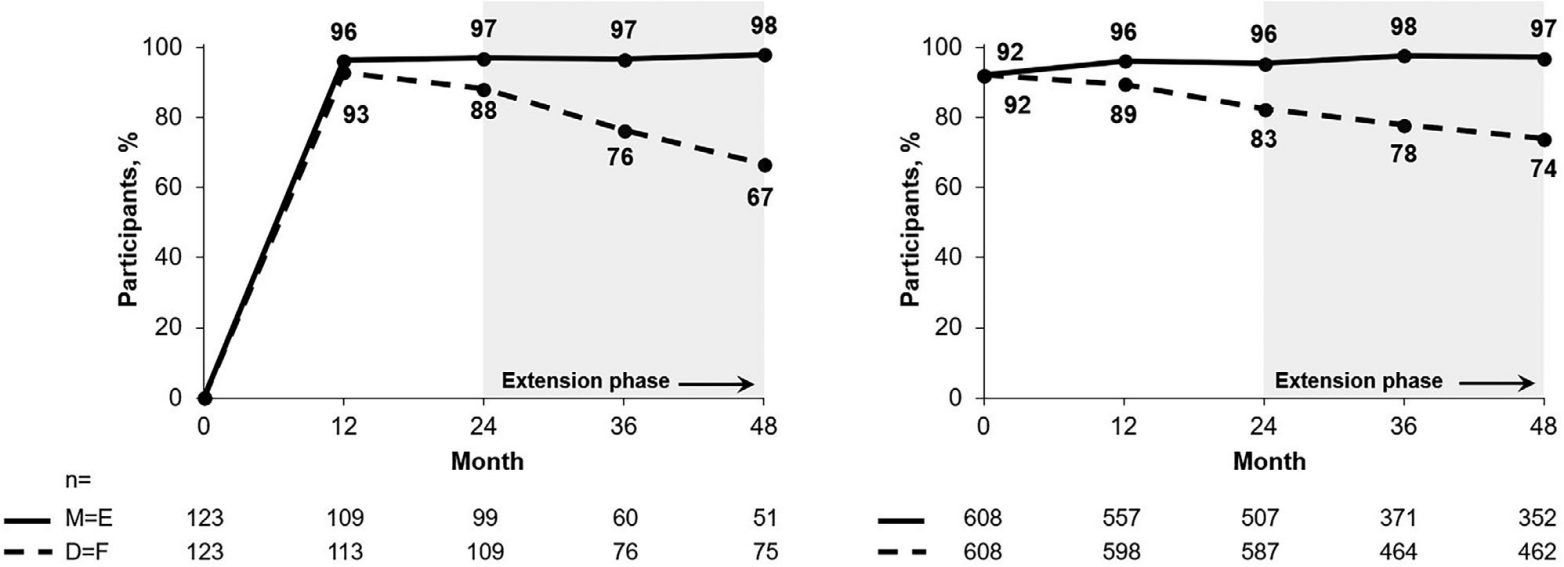
Proportion of participants achieving HIV‐1 RNA <50 copies/ml over 4 years (M = E population and D = F analyses). *n*, number of participants with available viral load data; D = F, discontinuation = failure (whereby discontinuation of B/F/TAF is imputed as HIV‐1 RNA ≥50 copies/ml); M = E, missing = excluded.

**Conclusions**: In a group of BICSTaR study participants with 4 years of follow‐up, B/F/TAF continued to demonstrate high levels of effectiveness and tolerability in clinical practice, with some improvements in PROs observed in TN participants.

### P064 Long‐acting injectable cabotegravir and rilpivirine outcomes in HIV‐positive migrants in Spain: do they have worse outcomes?

Jara Llenas‐García ^1^, Roberto Pedrero Tome^2^, Luis Ramos Ruperto^3^, María José Galindo Puerto^4^, Mariano Matarranz del Amo^5^, Carolina Navarro^6^, Miguel Torralba^7^, Gutiérrez Lara^8^, María Aguilera García^9^, Alfonso Cabello Úbeda^10^, Isabel San Joaquín Conde^11^, Luis Enrique Morano^12^, Noemí Cabello‐Clotet^13^, Patricia Martín Rico^14^, Carmen Montero Hernández^15^, Elisa Pino^16^, Alberto Díaz de Santiago^17^, Ruth Calderón Hernaiz^18^, Enrique Bernal^19^, María Jesús Vivancos Gallego^20^, María Antonia Sepúlveda^21^, Chiara Fanciulli^22^, Álvaro Cecilio^23^, Josefa Soler González^24^, Sergio Padilla^25^, Juan Emilio Losa García^26^, Carlos Armiñanzas Castillo^27^, Antonio Jesús Sánchez Guirao^28^, María del Mar García Navarro^29^, Ana Cerezales Calviño^30^, María Ángeles Garcinuño Jiménez^31^, Eva Ferreira Pasos^32^, Miriam Estébanez^33^, Beatriz de la Calle Riaguas^34^, Teresa Omiste Sanvicente^35^, Noemí Ramos Vicente^36^, Marta Clavero Olmos^37^, Juan Manuel Tiraboschi^38^, Ana Lucas Dato^39^, on behalf of the RELATIVITY Multicentre Cohort^40^

^1^Internal Medicine, Hospital Vega Baja, FISABIO.UMH.CIBERINFEC, Orihuela, Spain, ^2^Science Investigation, Fundación para la Investigación e Innovación Biomédica del Hospital Infanta Leonor, Madrid, Spain, ^3^Infectious Diseases, Hospital Universitario La Paz, Madrid, Spain, ^4^Infectious Diseases, Hospital Clínico Universitario de Valencia, Valencia, Spain, ^5^Internal Medicine, Hospital Universitario Infanta Leonor, Madrid, Spain, ^6^Infectious Diseases, Hospital de Burgos, Burgos, Spain, ^7^Internal Medicine, Hospital Universitario de Guadalajara, Guadalajara, Spain, ^8^HIV Unit, Hospital Universitario 12 de Octubre, Madrid, Spain, ^9^Infectious Diseases, Hospital Universitario La Princesa, Madrid, Spain, ^10^Infectious Diseases, Hospital Universitario Fundación Jiménez Díaz, Madrid, Spain, ^11^Infectious Diseases, Hospital Clínico Universitario Lozano Blesa, Zaragoza, Spain, ^12^Infectious Diseases, Hospital Universitario Álvaro Cunqueiro, Vigo, Spain, ^13^Internal Medicine, Hospital Clínico San Carlos, Madrid, Spain, ^14^Infectious Diseases, Hospital de Denia, Denia, Spain, ^15^Internal Medicine, Hospital Universitario de Torrejón, Torrejón de Ardoz, Spain, ^16^Internal Medicine, Hospital Universitario San Agustín, Avilés, Spain, ^17^Internal Medicine, Hospital Universitario Puerta de Hierro, Majadahonda, Spain, ^18^Internal Medicine, Hospital Universitario de Fuenlabrada, Fuenlabrada, Spain, ^19^Infectious Diseases, Hospital Reina Sofía, Murcia, Spain, ^20^Infectious Diseases, Hospital Universitario Ramón y Cajal, Madrid, Spain, ^21^Internal Medicine, Hospital Universitario de Toledo, Toledo, Spain, ^22^ Infectious Diseases, Hospital General Universitario Gregorio Marañón, Madrid, Spain, ^23^Internal Medicine, Hospital Universitario Miguel Servet, Zaragoza, Spain, ^24^Internal Medicine, Hospital Universitario de Cabueñes, Cabueñes, Spain, ^25^Infectious Diseases, Hospital General Universitario de Elche, Elche, Spain, ^26^Infectious Diseases, Hospital Universitario de Alcorcón, Alcorcón, Spain, ^27^Internal Medicine, Hospital Universitario Marqués de Valdecilla—IDIVAL CIBERINFEC, ISCIII, Santander, Spain, ^28^Internal Medicine, Hospital General Universitario Morales Meseguer, Murcia, Spain, ^29^Internal Medicine, Hospital Universitario de Vinalopo, Elche, Spain, ^30^Internal Medicine, Hospital Universitario Doctor José Molina Orosa, Las Palmas, Spain, ^31^Internal Medicine, Complejo Asistencial de Ávila, Ávila, Spain, ^32^Internal Medicine, Complejo Asistencial de Segovia, Segovia, Spain, ^33^Infectious Diseases, Hospital Central de la Defensa Gómez Ulla, Madrid, Spain, ^34^Internal Medicine, Hospital General Nuestra Señora del Prado, Talavera de la Reina, Spain, ^35^Internal Medicine, Hospital Universitario San Jorge, Huesca, Spain, ^36^Internal Medicine, Hospital Obispo Polanco, Teruel, Spain, ^37^Internal Medicine, Hospital Universitario Infanta Elena, Valdemoro, Spain, ^38^Internal Medicine, Hospital Universitario de Bellvitge, Hospitalet de Llobregat, Spain, ^39^Internal Medicine, Hospital Vega Baja, Orihuela, Spain, ^40^Infectious Diseases, Multicenter Cohort, Spain, Spain

**Background**: Cabotegravir and rilpivirine (CAB+RPV) is the first long‐acting injectable (LAI) treatment approved in Europe. Nevertheless, there is a scarcity of data regarding its efficacy in migrants, a highly mobile and vulnerable population often with incomplete information on basal HIV‐1 genotype, subtype or prior ART history.

**Materials and methods**: A multicentre non‐controlled retrospective study was conducted, including HIV‐1‐positive virally suppressed patients switching to CAB+RPV LAI from 37 hospitals in Spain. Basal characteristics and outcomes of migrant patients were compared with those of Spanish‐born patients. Quantitative variables were contrasted using U‐Mann‐Whitney test; categorical variables using chi‐square and Fisher's exact tests.

**Results**: Of 1350 HIV‐positive patients switching to LAI CAB+RPV, 396 (29.3%) were migrants. The majority of migrants were from Latin America (303/396), followed by Western Europe (26/396) and Africa (25/394). Migrants were generally younger (median age: 41 vs. 47 years, *p* < 0.001); a higher percentage were women (17.6% vs. 13.6%, *p* = 0.062). Modes of HIV acquisition differed, with migrants more likely to be GBMSM (71% vs. 62.1%, *p* = 0.003) or heterosexual (21.4% vs. 17.9%, *p* = 0.154) and less likely to be people who inject drugs (1.4% vs. 9.6%, *p* < 0.001) (Table 1). Migrants had a shorter median time with undetectable viral load before switching (60 vs. 96 months, *p* < 0.001). Prior genotype tests and HIV‐1 subtypes also varied, with migrants showing higher rates of certain non‐B subtypes. Prior virological failure did not differ statistically between both groups. After a median follow‐up of 7.5 months, 7.1% of migrants discontinued LAI CAB+RPV compared to 3.8% of Spaniards (OR 2.1, 95% CI 1.29–3.44, *p* = 0.014). Side effects led to discontinuation in 3.5% of migrants and 1.4% of Spaniards (OR 2.65, 95% CI 1.14–6.19, *p* = 0.017); local side effects were predominant. There were six virological failures (0.8% in migrants vs. 0.3% in Spaniards, *p* = 0.364), with integrase mutations emerging in two migrants and one Spanish‐born patient.

**P064: Table 1:** Comparative analysis of basal characteristics and outcomes between migrants and Spanish‐born patients switching to LAI CAB+RPV in the RELATIVITY cohort

	Migrants (*n* = 396)	Spanish‐born (*n* = 954)	OR (95% CI)	*p*‐value
Age (years); median [IQR]	41.0 [33.0−49.0]	47.0 [40.0−57.0]	−	<0.001
Sex: women	17.6%	13.6%	1.37 (0.98−1.90)	0.062
Mode of HIV acquisition: GBMSM	71.0%	62.1%	1.49 (1.13−1.97)	0.003
Mode of HIV acquisition: heterosexual	21.4%	17.9%	1.25 (0.91−1.70)	0.154
Mode of HIV acquisition: people who inject drugs	1.4%	9.6%	0.13 (0.04−0.32)	<0.001
Mode of HIV acquisition: other/unknown	6.2%	10.4%	0.63 (0.40−1.01)	0.052
Months from diagnosis to first ART regimen; median [IQR]	2.0 [0.0−6.0]	3.0 [1.0−20.0]	−	<0.001
Years on ART when starting CAB+RPV	7.0 [4.0−11.0]	10.0 [6.0−16.0]	−	<0.001
Months of undetectability prior to CAB+RPV	60.0 [22.0−108.0]	96.0 [48.2−140.0]	−	<0.001
Prior genotype test non‐available	52.4%	44.9%	1.35 (1.05−1.74)	0.017
Prior genotype test: wild type	64.6%	67.7%	0.87 (0.60−1.28)	0.514
Prior genotype test: INSTI mutations	1.7%	0.4%	4.30 (0.49−51.93)	0.114
Prior genotype test: NNRTI mutations	9.1%	6.2%	1.51 (0.75−2.94)	0.227
Prior genotype test: NRTI mutations	8.0%	9.9%	0.80 (0.39−1.51)	0.548
HIV‐1 subtype B	47.4%	45.5%	1.08 (0.75−1.55)	0.660
HIV‐1 subtype A	2.9%	3.2%	0.88 (0.25−2.58)	1.000
HIV‐1 subtype F/CRF	6.3%	2.0%	3.26 (1.23−8.73)	0.010
Prior virological failure	3.0%	5.5%	0.54 (0.25−1.06)	0.079
Prior virological failure to NNRTI	0.6%	1.3%	0.45 (0.10−2.06)	0.306
Body mass index (kg/m^2^); median [IQR]	25.1 [22.2−27.7]	24.5 [22.1−27.2]	−	0.298
LA CAB+RPV discontinuation	7.1%	3.8%	2.10 (1.29−3.44)	0.014
LA CAB+RPV discontinuation due to side effects	3.5%	1.4%	2.65 (1.14−6.19)	0.017
LA CAB+RPV discontinuation due to virological failure	0.8%	0.3%	2.42 (0.49−12.04)	0.280

Abbreviations: ART, antiretroviral treatment; CAB, cabotegravir; GBMSM, gays, bisexuals and other men who have sex with men; INSTI, integrase strand transfer inhibitor; IQR, interquartile range; LAI, long‐acting injectable; NNRTI, non‐nucleoside reverse transcriptase inhibitor; NRTI, nucleoside reverse transcriptase inhibitor; OR, odds ratio; RPV, rilpivirine.

**Conclusions**: Almost a third of patients switching to LAI CAB+RPV in this large Spanish cohort were migrants, mainly from Latin America. Migrant HIV‐positive patients had twice the risk of discontinuing LAI CAB+RPV than Spanish‐born patients and a higher risk of discontinuation due to side effects.

### P065 Efficacy and safety of long‐acting intramuscular cabotegravir and rilpivirine in women: a substudy of the RELATIVITY cohort

Maria Jose Galindo Puerto ^1^, Noemí Cabello Clotet^2^, Teresa Aldamiz‐Echevarría Lois^3^, Jara Llenas‐García^4^, Mari Mar Arcos^5^, Jesús Troya^6^, Luis Buzón Martín^7^, Miguel Torralba^8^, Mireia Santacreu^9^, Maria Aguilera García^10^, Alfonso Cabello Úbeda^11^, Maria Jose Crusells‐Canales^12^, Luis Morano^13^, Roberto Pedrero Tomé^14^, Patricia Martin Rico^15^, Carmen Montero Hernández^16^, Desiree Perez Martinez^17^, Sara de la Fuente Moral^18^, Ruth Calderón Hernáiz^19^, Enrique Bernal^20^, María Jesus Vivancos Gallego^21^, Maria Antonia Sepúlveda^22^, Alvaro Cecilio^23^, Víctor Arenas García^24^, Sergio Padilla^25^, Juan Emilio Losa García^26^, Francisco Arnaiz de las Revillas Almajano^27^, Antonio Jesús Sánchez Guirao^28^, María del Mar García Navarro^29^, Ana Cerezales Calviño^30^, Maria Angeles Garcinuño Jiménez^31^, Eva Maria Ferreira Pasos^32^, Miriam Estebanez^33^, Beatriz De la Calle Riaguas^34^, Miguel Egido Murciano^35^, Noemí Ramos Vicente^36^, Marta Clavero Olmos^37^, Juan Manuel Tiraboschi^38^, on behalf of the RELATIVITY Multicentre Cohort^39^

^1^Internal Medicine, Hospital Clínico Universitario Valencia, Unit of Infectious Diseases, Valencia, Spain, ^2^Internal Medicine, Hospital Clínico San Carlos, IdISS, CiberInfec, Universidad Complutense, Madrid, Spain, ^3^Infectious Diseases, Hospital General Universitario Gregorio Marañón, Madrid, Spain, ^4^Internal Medicine, Hospital Vega Baja de Orihuela, Universidad Miguel Hernández de Elche, CIBERINFEC, Instituto de Salud Carlos III, Orihuela, Spain, ^5^Infectious Diseases, Hospital Universitario La Paz, Madrid, Spain, ^6^Internal Medicine, Hospital Universitario Infanta Leonor, Madrid, Spain, ^7^Internal Medicine, Hospital de Burgos, Burgos, Spain, ^8^Internal Medicine, Hospital Universitario de Guadalajara, IDISCAM, Guadalajara, Spain, ^9^Internal Medicine, Hospital Universitario 12 de Octubre, Madrid, Spain, ^10^Infectious Diseases, Hospital Universitario La Princesa, Madrid, Spain, ^11^Infectious Diseases, Hospital Universitario Fundación Jiménez Díaz, Madrid, Spain, ^12^Infectious Diseases, Hospital Clínico Universitario Lozano Blesa, Zaragoza, Spain, ^13^Infectious Diseases, Hospital Universitario Alvaro Cunqueiro, Vigo, Spain, ^14^Science Investigation, Fundación para la Investigación e Innovación Biomédica del Hospital Infanta Leonor, Madrid, Spain, ^15^Internal Medicine, Hospital de Denia Marina Salud, Denia, Spain, ^16^Internal Medicine, Hospital Universitario de Torrejón, Madrid, Spain, ^17^Internal Medicine, Hospital Universitario San Agustín, Avilés, Spain, ^18^Servicio de Medicina Interna, Hospital Universitario Puerta de Hierro, Unidad de VIH, ITS y PrEP, Majadahonda, Spain, ^19^Internal Medicine, Hospital Universitario de Fuenlabrada, Madrid, Spain, ^20^Unidad Enfermedades Infecciosas, Hospital Universitario Reina Sofía, Murcia, Spain, ^21^Infectious Diseases, Hospital Universitario Ramón y Cajal, Madrid, Spain, ^22^Internal Medicine, Hospital Universitario, Toledo, Spain, ^23^Infectious Diseases, Hospital Universitario Miguel Servet, Zaragoza, Spain, ^24^Infectious Diseases, Hospital Universitario de Cabueñes, Gijón, Spain, ^25^Infectious Diseases, Hospital General Universitario, Elche, Spain, ^26^Infectious Diseases, Hospital Universitario de Alcorcón, Madrid, Spain, ^27^Infectious Diseases, Hospital Universitario Marqués de Valdecilla—IDIVAL CIBERINFEC, ISCIII, Santander, Spain, ^28^Infectious Diseases, Hospital General Universitario Morales Meseguer, Murcia, Spain, ^29^Internal Medicine, Hospital Universitario de Vinalopo, Alicante, Spain, ^30^Internal Medicine, Hospital Universitario Doctor José Molina Orosa, Las Palmas, Spain, ^31^Internal Medicine, Complejo Asistencial de Avila, Avila, Spain, ^32^Infectious Diseases, Complejo Asistencial de Segovia, Segovia, Spain, ^33^Infectious Diseases, Hospital Central de la Defensa Gómez Ulla, Madrid, Spain, ^34^Internal Medicine, Hospital General Nuestra Señora del Prado, Talavera de la Reina, Spain, ^35^Internal Medicine, Hospital Universitario San Jorge, Huesca, Spain, ^36^Internal Medicine, Hospital Obispo Polanco, Teruel, Spain, ^37^Internal Medicine, Hospital Universitario Infanta Elena, Madrid, Spain, ^38^Infectious Diseases, Hospital Universitario de Bellvitge, Barcelona, Spain, ^39^Infectious Diseases, Multicentre Cohort, Spain

**Background**: Intramuscular cabotegravir (CAB) and rilpivirine (RPV), administered every 2 months, can be used as a switching strategy in virologically suppressed people who live with HIV (PLWH). Women are underrepresented in clinical trials [1–3]. Real‐life data regarding efficacy and safety in this population are scarce. The aim of this substudy is to determine efficacy, tolerability and safety of this strategy when used to treat women who live with HIV (WLWH) in real life, out of a clinical trial context.

**Materials and methods**: The RELATIVITY cohort is a multicentre, non‐controlled, ambispective study, which evaluates virologically suppressed PLWH who switched to long‐acting CAB+RPV from 37 hospitals in Spain (RELATIVITY cohort). Patients were compared based on gender. Quantitative variables were contrasted using T‐Student and U‐Mann‐Whitney tests; categorical variables were compared using chi‐square and Fisher's exact tests.

**Results**: Of 1358 HIV‐positive patients on CAB+RPV, 201 (14.8%) were women. Baseline characteristics compared to men are depicted in Table 1. Time on ART (13.0 [8.0–20.0] vs. 9.0 [5.0–13.5] years; *p* < 0.001), and length of undetectability before switching to CAB+RPV (96.0 [45.0–147.0] months vs. 80.0 [40.0–124.0] months; *p* = 0.058) were longer in women. Additionally, rate of virological failure (VF) prior to switching was higher compared to men (11.7% vs. 3.5%; *p* < 0.001). Current follow‐up period is shorter for women (7.2 [4.6–9.6] months vs. 7.7 [5.1–11.1] months; *p* = 0.051) and discontinuation rate (8.5% vs. 4.1%; *p* = 0.014) and rate of local adverse injection reactions are higher compared to men (3.5% vs. 1.1%; *p* < 0.001). There were no differences in systemic side effects or VF development compared to men.

**P065: Table 1:** Comparative analysis of women and men living with HIV who switched to long‐acting CAB+RPV in the RELATIVITY cohort in Spain

	Women (*N* = 201)	Men (*N* = 1157)	*p*‐value
Age (years), median [IQR]	51.0 [42.0−58.0]	44.0 [37.0−53.4]	<0.001
Country of origin, *n* (%) Spain	129 (62.5)	822 (71.9)	0.067
Country of origin, *n* (%) Foreign	69 (34.8)	322 (28.1)	0.072
Country of origin, *n* (%) Foreign Latin America	37 (18.4)	262 (22.6)	0.213
Country of origin, *n* (%) Foreign Africa	12 (6.0)	13 (1.1)	<0.001
Transmission route, *n* (%) GBMSM	0 (0.0)	814 (75.2)	−
Transmission route, *n* (%) HTX	133 (72.7)	108 (10.0)	<0.001
Transmission route, *n* (%) PID	23 (12.6)	68 (6.3)	<0.001
Comorbidities, *n* (%) Hypertension	28 (13.9)	104 (9.0)	0.040
Comorbidities, *n* (%) Peripheral vascular disease	4 (2.0)	5 (0.4)	0.041
Comorbidities, *n* (%) Osteopenia/osteoporosis	29 (14.4)	51 (4.4)	<0.001
Comorbidities, *n* (%) Psychiatric disorders	25 (12.4)	94 (8.1)	0.063
CD4 nadir (cells/mm^3^), median [IQR]	249.5 [115.8−380.8]	350.0 [210.0−500.0]	<0.001
CD4/CD8, median [IQR] Baseline	1.1 [0.7−1.5]	0.9 [0.7−1.3]	0.001
CD4/CD8, median [IQR] End of period	1.1 [0.8−1.6]	0.9 [0.7−1.3]	<0.001
AIDS, *n* (%)	35 (19.2)	128 (11.7)	0.007

Abbreviations: GBMSM, gays, bisexuals and other men who have sex with men; HTX, heterosexual; IQR, interquartile range; PID, people who inject drugs.

**Conclusions**: Although WLWH who switched to CAB+RPV had a worse profile regarding comorbidities and prevalence of AIDS, they do not seem to have a higher risk of VF compared to men, but discontinuation rate might be higher. A longer follow up is necessary to understand outcomes in this underrepresented and critical sub‐population of PLWH treated with CAB+RPV.

**References**

1. Curno MJ, Rossi S, Hodges‐Mameletzis I, Johnston R, Price MA, Heidari S. A systematic review of the inclusion (or exclusion) of women in HIV research: from clinical studies of antiretrovirals and vaccines to cure strategies. J Acquir Immune Defic Syndr. 2016;71(2):181‐8.

2. Ramgopal MN, Castagna A, Cazanave C, Diaz‐Brito V, Dretler R, Oka S, et al. Efficacy, safety, and tolerability of switching to long‐acting cabotegravir plus rilpivirine versus continuing fixed‐dose bictegravir, emtricitabine, and tenofovir alafenamide in virologically suppressed adults with HIV, 12‐month results (SOLAR): a randomised, open‐label, phase 3b, non‐inferiority trial. Lancet HIV. 2023;10(9):e566‐77.

3. Overton ET, Richmond G, Rizzardini G, Thalme A, Girard P‐M, Wong A, et al. Long‐acting cabotegravir and rilpivirine dosed every 2 months in adults with human immunodeficiency virus 1 type 1 infection: 152‐week results from ATLAS‐2M, a randomized, open‐label, phase 3b, noninferiority study. Clin Infect Dis. 2023;76(9):1646‐54.

### P066 Causes of discontinuation of long‐acting cabotegravir and rilpivirine in clinical practice: results from the prospective multicentre SCOLTA cohort

Lucia Taramasso^1^, Nicola Squillace ^2^, Elena Ricci^3^, Sergio Ferrara^4^, Giancarlo Orofino^5^, Eleonora Sarchi^6^, Emanuele Pontali^7^, Giovanni Cenderello^8^, Giovanni Francesco Pellicanò^9^, Filippo Lagi^10^, Elena Salomoni^11^, Olivia Bargiacchi^12^, Maria Aurora Carleo^13^, Luigi Pusterla^14^, Salvatore Martini^15^, Rita Bellagamba^16^, Giordano Madeddu^17^, Giuseppe Vittorio De Socio^18^, Barbara Menzaghi^19^, Goffredo Angioni^20^, Katia Falasca^21^, Antonio Cascio^22^, Antonio Di Biagio^23^, Paolo Bonfanti^24^

^1^Infectious Diseases, IRCCS Ospedale Policlinico San Martino, Genova, Italy, ^2^Infectious Diseases, Fondazione IRCCS San Gerardo dei Tintori, Monza, Italy, ^3^Fondazione ASIA, Milano, Italy, ^4^Infectious Diseases, Department of Clinical and Experimental Medicine, A.O.U. “Policlinico Riuniti”, Foggia, Italy, ^5^Infectious Diseases, Amedeo di Savoia Hospital, Torino, Italy, ^6^Infectious Diseases, Ss. Antonio e Biagio e Cesare Arrigo Hospital, Alessandria, Italy, ^7^Infectious Diseases, Galliera Hospital, Genova, Italy, ^8^Infectious Diseases, Sanremo Hospital, Sanremo, Italy, ^9^Department of Clinical and Experimental Medicine, University of Messina, Messina, Italy, ^10^Infectious and Tropical Diseases, Careggi Hospital, Firenze, Italy, ^11^Infectious Diseases, Santa Maria Annunziata Hospital, Firenze, Italy, ^12^Infectious Diseases, Ospedale Maggiore della Carità, Novara, Italy, ^13^Infectious Diseases and Gender Medicine, Cotugno Hospital, Napoli, Italy, ^14^Infectious Diseases, Ospedale Sant'Anna, Como, Italy, ^15^Infectious Diseases, University of Campania Luigi Vanvitelli, Napoli, Italy, ^16^Clinical and Research Infectious Diseases, National Institute for Infectious Diseases Lazzaro Spallanzani IRCCS, Roma, Italy, ^17^Medicine, Surgery and Pharmacy, University of Sassari, Sassari, Italy, ^18^Infectious Diseases, Santa Maria Hospital, Perugia, Italy, ^19^Infectious Diseases, ASST della Valle Olona, Busto Arsizio, Italy, ^20^Infectious Diseases, Ss. Trinità Hospital, Cagliari, Italy, ^21^Medicine and Science of Aging, G. D'Annunzio University, Chieti, Italy, ^22^Health Promotion, Mother and Child Care, Internal Medicine and Medical Specialties, University of Palermo, Palermo, Italy, ^23^DISSAL—Department of Health Science, University of Genova, Genova, Italy, ^24^Infectious Diseases, University of Milano‐Bicocca, Monza, Italy

**Background**: The aim of this study is to describe the causes of long‐acting (LA) cabotegravir (CAB) + rilpivirine (RPV) discontinuation in clinical practice.

**Materials and methods**: Observational multicentre prospective SCOLTA (Surveillance Cohort Long‐Term Toxicity Antiretrovirals) cohort. PWH who started CAB+RPV LA from July 2022 to February 2024 were included if they had at least one follow‐up visit. Probability of discontinuation for adverse events (AE) was assessed through log‐rank test, while factors associated with AE were estimated using a Cox proportional‐hazards model.

**Results**: Three hundred and seventy‐seven PWH were included and observed for median 10 months (IQR 6–13), 90 (23.9%) were women, 362 Caucasian (96.0%), most were men who have sex with men (180, 47.8%) and heterosexuals (132, 35.0%), mainly in CDC stage A (224, 60%). Mean age was 48.5 (±11.2) years and median time on ART was 10.0 (IQR 6.3–16.3) years. Previous ART contained an INSTI in 188 (49.9%) people, an NNRTI in 89 (23.6%), both INSTI+NNRTI in 100 (26.5%). One hundred and sixty‐five participants (43.8%) had at least one treatment in addition to ART (145 were taking 1–2 other drugs; 67 ≥3 other drugs). Thirty‐four PWH (9%) discontinued after median 3 months (IQR 1–5), of which 24 for AE (Table 1), four (1%) for virological failure, six (1.5%) for other reasons (lost to follow up [*N* = 1], pregnancy [*N* = 1], resistance to RPV [*N* = 1], residence change [*n* = 1] and inconvenience with injection schedule [*N* = 2]). The probability of AE leading to discontinuation was not influenced by previous ART, sex, BMI, age, risk factor for HIV, oral lead‐in or concomitant treatments (*p* > 0.1 for all). PWH in CDC stage C were more likely to discontinue (*p* = 0.0476), and this result was confirmed at the multivariate analysis including all these factors (adjusted hazard ratio 3.00, 95% CI 1.11–8.12, *p* = 0.030).

**P066: Table 1:** Adverse events leading to discontinuation of long‐acting therapy with cabotegravir/rilpivirine in SCOLTA cohort

	Sex at birth, age	Previous regimen	Pain/local reaction	Fever	Other	Causal correlation with therapy	Days since first injection
1	F, 59	3TC/DTG	Yes, G 3	No	No	Certain	132
2	M, 67	3TC/DTG	Yes, G 2	Yes, G 2	No	Certain	56
3	M, 55	RPV/DTG	Yes, G 2	No	No	Certain	29
4	M, 40	RPV/DTG	Yes, G 2	No	No	Certain	28
5	M, 61	RPV/DTG	Yes, G 2	No	No	Certain	56
6	M, 54	FTC/TAF/BIC	Yes, G 2	No	No	Certain	445
7	M, 55	FTC/TAF/BIC	Yes, G 1	No	No	Certain	64
8	M, 35	FTC/TAF/BIC	No	Yes, G 3	No	Certain	112
9	M, 44	RPV/DTG	No	Yes, G 2	Arthromyalgia, G 2	Possible	87
10	M, 49	3TC/DTG	No	Yes, G 2	No	Probable	1
11	M, 32	3TC/DTG	No	Yes, G 1	No	Unlikely	94
12	F, 65	RPV/DTG	No	Yes, G 1	No	Possible	181
13	M, 56	3TC/ABC/DTG	No	No	Difficulty walking, G 3; weight gain, G 1	Unlikely for both	87
14	M, 46	RPV/DTG	No	No	Arthromyalgia, G NA	Certain	30
15	M, 59	FTC/TAF/BIC	No	No	Arthromyalgia, G 1	Probable	19
16	M, 51	RPV/DTG	No	No	Rash, G 2	Possible	140
17	M, 31	FTC/TAF/BIC	No	No	Arthromyalgia, G 4	Certain	63
18	M, 54	FTC/TAF/RPV	No	No	Acute pancreatitis, G 4	Probable	67
19	M, 66	FTC/TAF/RPV	No	No	Glycaemic decompensation, G 3	Possible	98
20	F, 41	FTC/TAF/BIC	No	No	Migraine, G 2	Certain	31
21	M, 62	FTC/TAF/BIC	No	No	Hepatitis, G 3	Probable	92
22	F, 62	3TC/DTG	No	No	Rash, G 3	Possible	143
23	M, 50	FTC/TAF/BIC	No	No	Altered emotionality, G 3	Probable	339
24	F, 58	RPV/DTG	NA	NA	NA	NA	283

Abbreviations: 3TC, lamivudine; ABC, abacavir; BIC, bictegravir; DTG, dolutegravir; F, female; FTC, emtricitabine; G, grade of adverse event; M, male; NA, not available; RPV, rilpivirine; TAF, tenofovir alafenamide.

**Conclusions**: The frequency of LA CAB+RPV interruptions for AE was higher in this real‐life cohort than in registrational trials, while high virological efficacy was seen, with 1% virological failure. To continue the active surveillance of AE in cohort studies will be critical to understanding the key to LA persistence.

### P067 Treatment patterns in virologically suppressed, treatment‐experienced people with HIV: a US real‐world database study

Samir Gupta^1^, Katherine Cappell^2^, Kwanza Price ^3^, Machaon Bonafede^4^, Joshua Gruber^5^, JeanPierre Coaquira Castro^6^, Soodi Navadeh^7^, Robert Sedgley^8^, Sorana Segal‐Maurer^9^

^1^Medicine/Division of Infectious Diseases, Indiana University School of Medicine, Indianapolis, IN, USA, ^2^Real World Evidence, Veradigm, Life Sciences, Chicago, IL, USA, ^3^Health Economics and Outcomes Research, Gilead Sciences, Inc., Foster City, CA, USA, ^4^Research Consulting, Real World Evidence, Veradigm, Chicago, IL, USA, ^5^Global Medical Affairs Research, Gilead Sciences, Inc., Foster City, CA, USA, ^6^Global HEOR, Global Value & Access, Gilead Sciences, Inc., Foster City, CA, USA, ^7^Real‐World Evidence—Virology, Gilead Sciences, Inc., Foster City, CA, UK, ^8^Research Services, Real World Evidence, Veradigm, Chicago, IL, USA, ^9^Division of Infectious Diseases, New York‐Presbyterian Queens, New York, NY, USA

**Background**: Virologically suppressed, treatment‐experienced people with HIV (VSTE‐PWH) often receive complex antiretroviral (ARV) regimens. Better understanding and characterization of this subset of PWH may help optimize treatment strategies. We recently described defining criteria for VSTE‐PWH, and their characteristics, using a large US database [1]; here, we report treatment patterns in VSTE‐PWH using this database.

**Methods**: In this retrospective, observational analysis, treatment‐experienced PWH with ≥2 ARV lines of therapy (LOT) were identified from the Veradigm Network electronic health record (EHR)‐linked claims database (study period: 01/2015–12/2022). All VSTE‐PWH met ≥1 VSTE‐defining treatment criteria (Figure 1), had virological suppression (≤2.3 log copies/ml) ≤90 days from index date (most recent date on which VSTE criteria met) and remained virologically suppressed throughout the index ARV LOT. Participants were aged ≥18 years, diagnosed with HIV, had continuous claims enrolment and EHR activity. LOT periods ended at discontinuation or change to any regimen component. We report treatment patterns for the 6‐month period after index.

**P067: Figure 1:**
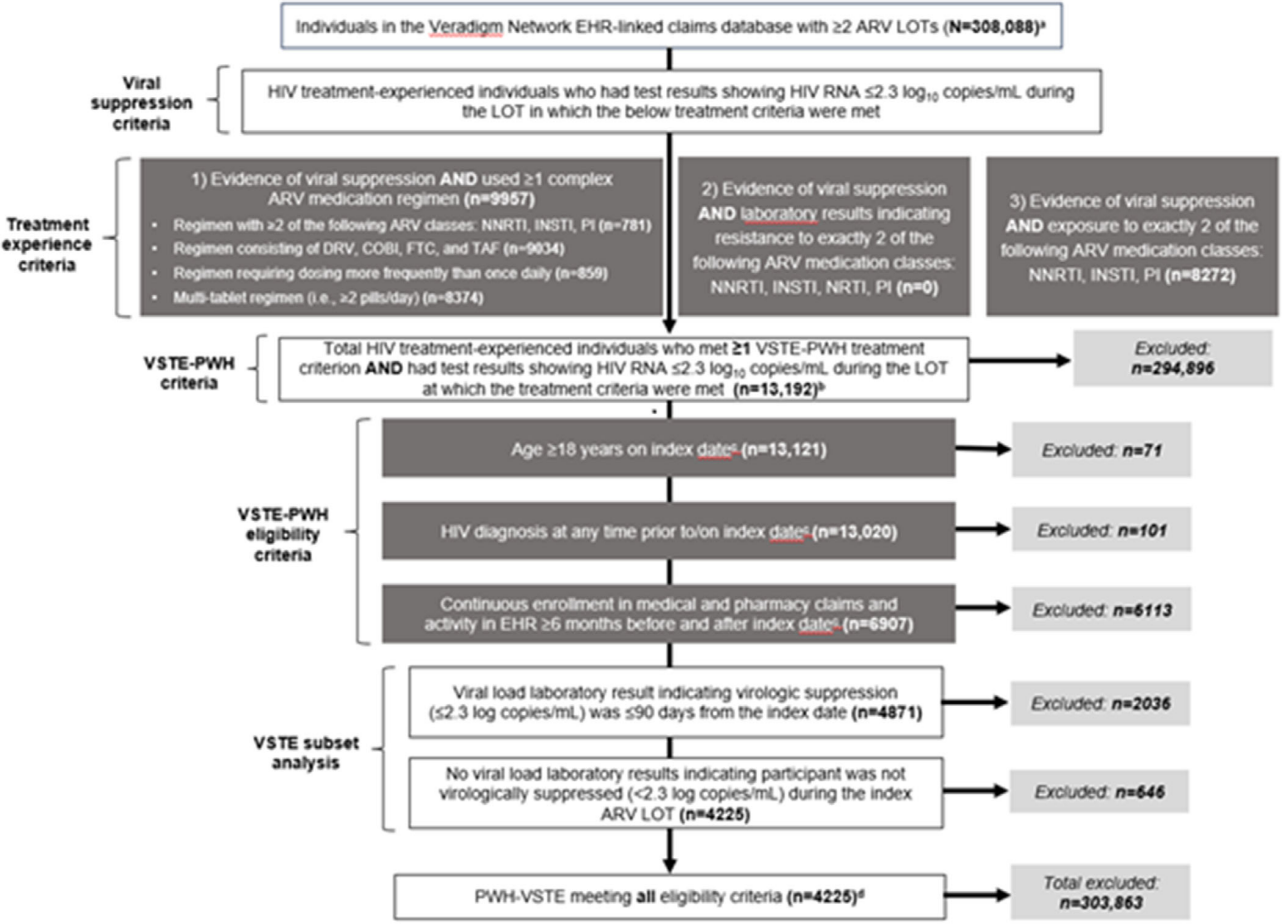
VSTE‐PWH study attrition diagram. ^a^Identified within the study period of 1 January 2015−31 December 2022; ^b^5037 PWH met ≥2 treatment experience criteria; ^c^index date was defined as the most recent date on which one of the VSTE‐defining treatment criteria was met; ^d^PWH‐VSTE were mostly male (71%) and using Medicaid (54%), and a large proportion were Black (41%), aged 50–59 years (37%), and from Southern US states (38%). ARV, antiretroviral therapy; COBI, cobicistat; DRV, darunavir; EHR, electronic health record; FTC, emtricitabine; INSTI, integrase strand transfer inhibitor; LOT, line of therapy; NNRTI, non‐nucleoside reverse transcriptase inhibitor; NRTI, nucleoside/nucleotide reverse transcriptase inhibitor; PI, protease inhibitor; VSTE‐PWH, virologically suppressed, treatment‐experienced people with HIV; TAF, tenofovir alafenamide.

**Results**: Of 308,088 treatment‐experienced PWH, 13,192 (4%) met ≥1 VSTE‐defining criteria, with 4225 eligible for analysis (Figure 1). During the 6‐month follow‐up, 875 (20.7%) VSTE‐PWH experienced one regimen change, 230 (5.4%) experienced two changes and 103 (2.4%) experienced ≥3 changes. Overall, 1555 (37%) participants did not persist with their original index LOT (LOT1); of these, 347 (8%) discontinued treatment. Median (IQR) treatment duration was 265 (90–658) days during LOT1, decreasing to a median of 54–136 days for LOT2–5. Mean (SD) percent of days covered was 93% (11%) during LOT1, and 87–91% in LOT2–5. Mean time from end of one LOT to start of the next was 0–14.5 days across LOT1–4. During LOT1, mean (SD) pills/day was 1.6 (0.9), increasing to 2.5 (1.0) by LOT5. Table 1 shows the most common ARV combinations for LOT1–5.

**P067: Table 1:** Most common medication regimens (≥5% in any LOT) for LOTs 1–5

*n* (%)	LOT1, *n* = 4225	LOT2, *n* = 1208	LOT3, *n* = 333	LOT4, *n* = 103	LOT5, *n* = 22
NRTI‐NRTI‐INSTI	1163 (28)	257 (21)	37 (11)	6 (6)	1 (5)
NRTI‐NRTI‐INSTI‐COBI	768 (18)	147 (12)	19 (6)	7 (7)	0
INSTI, NRTI‐NRTI	409 (10)	103 (9)	36 (11)	13 (13)	2 (9)
NRTI‐NRTI‐NNRTI	289 (7)	69 (6)	9 (3)	3 (3)	1 (5)
NRTI‐NRTI, PI	170 (4)	65 (5)	19 (6)	11 (11)	2 (9)
INSTI	17 (0)	42 (4)	11 (3)	6 (6)	0

*Note*: Hyphenated terms represent a single tablet regimen. Terms separated by commas represent a multiple tablet regimen.

Abbreviations: COBI, cobicistat; INSTI, integrase strand transfer inhibitor; LOT, line of therapy; NNRTI, non‐nucleoside reverse transcriptase inhibitor; NRTI, nucleoside/nucleotide reverse transcriptase inhibitor; PI, protease inhibitor.

**Conclusions**: Among VSTE‐PWH, treatment changes were common and accompanied by increased pill burden, poor adherence and progressively decreasing treatment duration. Treatment changes can impact health‐related quality of life due to physical and psychological impacts of new regimens. Optimized treatment options could help improve adherence and LOT duration challenges observed in this population [2].

**References**

1. Gupta S, Cappell K, Price K, Bonafede M, Gruber J, Mezzio D, et al. Characterization of people with HIV who are virologically suppressed with treatment experience using a US real‐world database. IDWeek 2024; 2024 Oct 16–19; Los Angeles, CA, USA.

2. Altice F, Evuarherhe O, Shina S, Carter G, Beaubrun AC. Adherence to HIV treatment regimens: systematic literature review and meta‐analysis. Patient Prefer Adherence. 2019;13:475‐90.

### P068 Real‐world effectiveness in treatment‐experienced (TE) people with HIV (PWH) switching to bictegravir/emtricitabine/tenofovir alafenamide (B/F/TAF) with distinct patterns of self‐reported adherence

Marta Boffito ^1^, Jason Brunetta^2^, Itzchak Levy^3^, Chia‐Jui Yang^4^, Joaquín Portilla^5^, Eoghan De Barra^6^, Roger Vogelmann^7^, Tomoyuki Endo^8^, Olivier Robineau^9^, Loredana Sarmati^10^, David Thorpe^11^, Andrea Marongiu^12^, Tali Cassidy^12^, Berend van Welzen^13^

^1^HIV, Sexual and Gender Health, Dermatology, Chelsea and Westminster Hospital, London, UK, ^2^Maple Leaf Medical Clinic, Ontario, Canada, ^3^Infectious Disease Unit, Sheba Medical Center, Ramat Gan, Israel, ^4^Department of Internal Medicine, Far Eastern Memorial Hospital, New Taipei City, Taiwan, ^5^Department of Internal Medicine, General University Hospital of Alicante, Alicante, Spain, ^6^Department of International Health and Tropical Medicine, Royal College of Surgeons in Ireland (RCSI) University of Medicine and Health Sciences, Beaumont Hospital, Dublin, Ireland, ^7^Mannheimer Onkologie Praxis, Mannheim, Germany, ^8^Department of Hematology, Hokkaido University Hospital, Sapporo, Japan, ^9^Centre Hospitalier de Tourcoing, EA2694, Univ Lille, Tourcoing, France, ^10^Infectious Diseases Clinic, University Hospital of Rome Tor Vergata, Rome, Italy, ^11^Global HIV Medical Affairs, Gilead Sciences Ltd, Uxbridge, UK, ^12^Real World Evidence, Gilead Sciences Ltd, Uxbridge, UK, ^13^Department of Infectious Diseases, University Medical Center Utrecht, Utrecht, Netherlands

**Background**: Adherence to antiretroviral therapy (ART) is important for maintaining virological suppression in PWH. BICSTaR is a large, multi‐country, observational study evaluating the effectiveness, safety and patient‐reported outcomes of B/F/TAF in treatment‐naïve and TE PWH in routine clinical practice. We present an analysis of self‐reported treatment adherence through 24 months (M) in TE participants switching to B/F/TAF.

**Materials and methods**: Self‐reported adherence at baseline, 6M, 12M and 24M was measured using visual analogue scale (VAS) adherence questionnaires (% ART doses taken in the last 30 days) and number of missed doses in the last 4 and 30 days. A joint group‐based trajectory modelling approach used these measures to identify distinct adherence patterns among TE participants. Multinomial regression identified significant associations between baseline characteristics and adherence trajectory group. Effectiveness at 24M was analysed by adherence group (HIV‐1 RNA <50 copies/ml; missing = excluded and B/F/TAF discontinuation = failure).

**Results**: The analysis included 1496 TE participants who switched to B/F/TAF. At both baseline and 24M, median (IQR) VAS adherence score was 100% (97–100%) [baseline, *n* = 1298; 24M, *n* = 941] and median (IQR) doses missed over the last 4 and 30 days were 0 (0–0) [baseline, *n* = 1250; 24M, *n* = 904] and 0 (0–1) [baseline, *n* = 1252; 24M, *n* = 905], respectively. The model identified five distinct adherence groups (Figure 1). Most participants had “near‐perfect” to moderately high adherence (Group 1 [reference], Group 2 and Group 3), which was stable over time. In two smaller groups, adherence was initially high but then declined (Group 4), or was initially low but increased over time (Group 5) following switch to B/F/TAF. Baseline characteristics associated with each adherence group are presented in Table 1. Compared with Group 1, Group 4 participants were younger, with higher baseline HIV symptom counts. Group 5 participants were more likely to be Black and have low CD4 counts, higher viral loads and higher baseline HIV symptom counts. Regardless of adherence trajectory, all groups showed high effectiveness with B/F/TAF at 24M (96% overall [missing = excluded]; Figure 1), with no treatment‐emergent resistance.

**P068: Figure 1:**
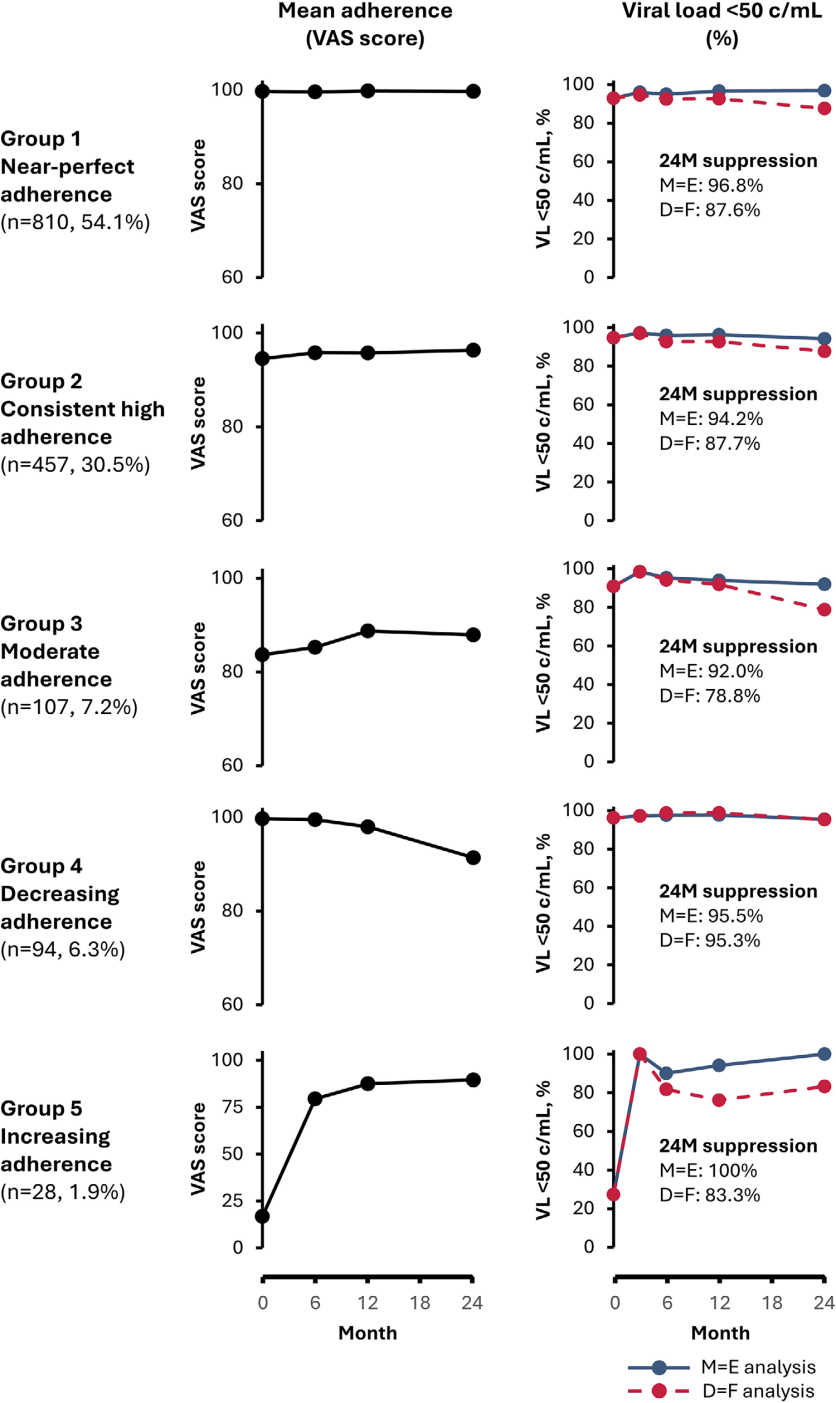
Self‐reported adherence measured using the VAS adherence score and effectiveness over time, by group. The best‐fit model was selected using the Bayesian information criterion. c, copies; D = F, discontinuation = failure; M, month; M = E, missing = excluded; VAS, visual analogue scale; VL, viral load.

**P068: Table 1:** Baseline clinical and demographic characteristics by adherence group

	Group 1: near‐perfect adherence (*n* = 810, 54.1%)	Group 2: consistent high adherence (*n* = 457, 30.5%)	Group 3: moderate adherence (*n* = 107, 7.2%)	Group 4: decreasing adherence (*n* = 94, 6.3%)	Group 5: increasing adherence (*n* = 28, 1.9%)
Male sex, *n* (%)	677 (83.6)	387 (84.7)	88 (82.2)	82 (87.2)	23 (82.1)
Black race, *n* (%)	79 (9.8)	52 (11.4)	13 (12.1)	7 (7.4)	7 (25.0)^a^
Age at B/F/TAF initiation, years, median (IQR)	49 (40−56)	47 (37−54)^a^	45 (35−53)^a^	44.5 (35−55)^a^	45.5 (38−51.5)
Baseline CD4/CD8 ratio, median (IQR)	0.8 (0.6−1.2)	0.9 (0.6−1.3)	0.8 (0.6−1.3)	0.9 (0.7−1.3)	0.5 (0.3−0.7)^a^
Baseline CD4 count, cells/µl, median (IQR)	669.0 (420.0−874.0)	659.0 (492.0−902.0)	664.5 (453.5−835.0)	680.0 (520.0−834.0)	442.0 (309.6−946.0)
HIV‐1 RNA viral load <50 copies/ml at baseline, *n* (%)	652 (92.9)	375 (94.7)	80 (90.9)	76 (96.2)	6 (27.3)^a^
History of or ongoing neuropsychiatric disorder, *n* (%)	189 (23.3)	131 (28.7)^a^	28 (26.2)	21 (22.3)	6 (21.4)
Baseline MCS score,^b^ median (IQR)	51.1 (42.5−56.4)	47.7 (38.7−54.3)^a^	46.3 (38.0−52.3)^a^	47.6 (40.3−52.9)	44.9 (38.8−55.0)
Baseline HIV‐SI overall bothersome count, median (IQR)	3.0 (1.0−6.0)	4.0 (1.0−7.0)^a^	5.0 (2.0−8.0)^a^	4.0 (1.5−7.0)^a^	3.5 (1.0−10.5)^a^

*Note*: Data in participants with data available at baseline.

Abbreviations: B/F/TAF, bictegravir/emtricitabine/tenofovir alafenamide; HIV‐SI, HIV Symptom Index; IQR, interquartile range; MCS, mental component summary.

^a^ *p* < 0.05; statistically significant associations with adherence group compared with reference group (Group 1) in univariate and multivariate multinomial regression models.

^b^MCS score is standardized to a mean of 50 (range 1–100), scores >50 and <50 represent better‐than‐average and poorer‐than‐average function, respectively.

**Conclusions**: High levels of effectiveness (>90%) were observed across all groups following switch to B/F/TAF, including in those participants with decreasing adherence over 24M.

### P069 Long‐acting cabotegravir/rilpivirine as a safe antiretroviral therapy in solid organ transplanted HIV patients

Ana Moreno ^1^, Santos Del Campo^1^, Maria Jesus Perez‐Elias^1^, Jose Luis Casado^1^, Miguel Garcia^2^, Manuel Velez^3^, Maria Jesus Vivancos^1^, Santiago Moreno^1^

^1^Infectious Diseases, Hospital Ramon y Cajal, Madrid, Spain, ^2^Gastroenterology (Liver Transplant Unit), Hospital Ramon y Cajal, Madrid, Spain, ^3^Pharmacy, Hospital Ramon y Cajal, Madrid, Spain

**Background**: Long‐acting (LA) cabotegravir/rilpivirine (C/R) has emerged as the first new paradigm in antiretroviral therapy (ARV). To date, there is scant information on its usefulness in patients who have undergone solid organ transplantation (SOT).

**Materials and methods**: Descriptive analysis on the viral efficacy and safety of C/R started between September 2023 and May 2024 (administered baseline—week 4—and each 8 weeks thereafter, without oral leading) in five HIV‐infected patients with SOT attended at an HIV outpatient clinic from a tertiary hospital in Madrid, Spain.

**Results**: There were four liver transplants and one kidney transplant. Only one subject actively asked for LA, in the remaining therapy was prescribed after medical proposal. All subjects were white, 60% male, median age 60 years (49–61), prior AIDS in 60%. The median time on ARV before LA was 28 years (14–29), with a median of 8 lines of therapy (6–22), whereas the median time between SOT and C/R was 6 years (1–12). HIV subtype was lacking in all subjects, HBs antibodies (HBsAb) were present in all, in two cases due to vaccination. Median baseline CD4 count 583 cells/ml (347–856), 100% with undetectable HIV RNA. Median baseline BMI and GFR were 25 (21–38) and 76 ml/minute (24–91), respectively. In the two obese patients, larger needles were used for the injection. Prior ARV were BIC/TAF/FTC (*N* = 3), and DTG/3TC and DTG/3TC/ABC one each. Prior NNRTI use was reported in four (80%), with RPV experience in three, but no cases of viral failure. Concomitant immunosuppressants were: cyclosporin plus mycophenolate (*N* = 1), everolimus monotherapy (*N* = 1), everolimus plus prednisolone (*N* = 1), tacrolimus plus mycophenolate (*N* = 1); only the kidney recipient was on triple therapy with cyclosporin plus mycophenolate plus prednisone. All patients presented at least one comorbidity: hypertension 60%, dyslipidaemia 60% and diabetes 40%. Liver cirrhosis, oropharyngeal cancer, pulmonary obstructive disease, ischaemic myocardial disease and depressive symptoms in one case each. To date, after a median time of follow‐up of 34 weeks (6–40), all patients remain on LA with no viral failure or safety issues.

**Conclusions**: LA with C/R may be considered in SOT patients as a safe and effective ARV.

### P070 Early virological and immunological effectiveness of ainuovirine‐ compared to efavirenz‐based antiretroviral regimen as initial therapy in treatment‐naive people with HIV‐1: the pooled analysis of prospective or retrospective real‐world studies

Hai Long^1^, Linghua Li^2^, Min Wang^3^, Shangjie Wu^4^, Quan Zhang^4^, Zhong Chen^3^, Quanying He^5^, Shujing Ma^6^, Lei Guo^1^, Yufang Zheng^7^, Chunyun Zhang^7^, Hong Qin ^7^

^1^Department of Infectious Diseases, GuiYang Public Health Clinical Center, Guiyang, China, ^2^Infectious Disease Center, Guangzhou Eighth People's Hospital, Guangzhou Medical University, Guangzhou, China, ^3^Institute of HIV/AIDS, The First Hospital of Changsha, Changsha, China, ^4^Department of Pulmonary and Critical Care Medicine, The Second Xiangya Hospital, Central South University, Changsha, China, ^5^Department of Outpatient, Yunnan Provincial Infectious Disease Hospital, Kunming, China, ^6^School of Public Health, the Key Laboratory of Environmental Pollution Monitoring and Disease Control, Guizhou Medical University, Ministry of Education, Guiyang, China, ^7^Clinical Research & Development, Jiangsu Aidea Pharmaceutical Co., Ltd, Yangzhou, China

**Background**: Virological and immunological efficacy of ainuovirine (ANV), a new‐generation NNRTI, has been validated in the RACER study, a multi‐centre, randomized, active‐controlled, non‐inferiority phase III trial. This pooled analysis aimed to evaluate 24‐week virological and immunological effectiveness of ANV compared to efavirenz (EFV), both combined with lamivudine (3TC) and tenofovir DF (TDF), as initial therapy for treatment‐naïve people with HIV‐1 (PWH) in real‐world clinical practice.

**Methods**: Four study populations involving treatment‐naïve PWH exposed to ANV‐ compared to EFV‐based antiretroviral (ARV) regimen were pooled for further analyses (*N* = 1655), including two prospective (*N* = 391) and two retrospective ones (*N* = 1264). The primary outcome measure was proportion of PWH with HIV RNA below 50 copies/ml or lower limit of quantitation, where applicable, at week 24. Subgroup analysis was performed for the primary outcome measure in PWH with a baseline HIV RNA titre ≥100,000 copies/ml. The key secondary outcome measure was absolute change from baseline (CFB) in CD4^+^ cell count at week 24.

**Results**: The two arms were well balanced in baseline demographics (ANV arm, *N* = 664; EFV, *N* = 991), at a median age of 40.9 versus 41.5 years, with 73.6% versus 79.4% being male, and with a mean body mass index of 21.9 versus 22.1 kg/m^2^. The primary outcome measures at week 24 were 86.4% with ANV arm and 80.0% with EFV arm (estimated treatment difference, 0.064 [95% CI 0.028–0.100] (Figure 1a), Newcombe‐Wilson method; *p*‐value <0.001, one‐tail), respectively. Subgroup analysis also established superiority of ANV arm to EFV arm in virological suppression for PWH with baseline high viral load (86.4% vs. 57.5%, 0.289 [0.193–0.372], *p* < 0.001) (Figure 1b). Absolute CFB in median CD4^+^ cell count was significantly higher in ANV arm than that in EFV arm (159 vs. 102 cell/mm^3^) at week 24.

**P070: Figure 1:**
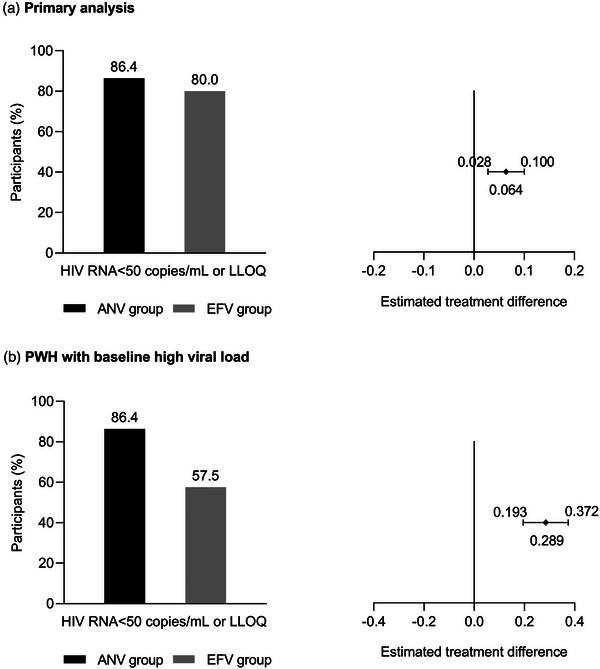
Primary analysis and subgroup analysis in PWH with baseline high viral load of 24‐week virological outcomes. ANV, ainuovirine; EFV, efavirenz; PWH, people with HIV‐1.

**Conclusions**: ANV‐based ARV regimen was superior to EFV‐based regimen in early virological suppression at week 24 in real‐world studies. This superiority was also observed in PWH with baseline high viral load. ANV‐based regimen was also beneficial for CD4^+^ cell restoration compared to EFV‐based regimen in treatment‐naïve PWH.

### P071 Switching from integrase strand transfer inhibitors (INSTIs) based triple therapy to dual therapy in people with HIV‐1 infection (PWH): the French hospital database on HIV (ANRS CO4 FHDH) real‐life experience

Emilie Lanoy^1^, Claudine Duvivier^2^, Nathalie De Castro^3^, Sophie Abgrall^4^, Sylvie Bregigeon‐Ronot^5^, Fabienne Caby^6^, Colin Deschanvres^7^, Jade Ghosn^8^, Marina Karmochkine^9^, Christine Katlama^10^, Odile Launay^11^, Christian Pradier^12^, Jacques Reynes^13^, Dominique Salmon^9^, Dominique Costagliola^1^, Sophie Grabar ^14^

^1^Institut National de la Santé et de la Recherche Médicale (INSERM), Sorbonne Université, Institut Pierre‐Louis d'Épidémiologie et de Santé Publique, Paris, France, ^2^Institut Pasteur Medical Centre, Assistance Publique des Hôpitaux de Paris (AP‐HP), Necker Hospital, Paris Cité University, Institut Hospitalo‐Universitaire (IHU) Imagine, Paris, France, ^3^Maladies Infectieuses, AP‐HP, Hôpitaux Saint Louis‐Lariboisière, Paris, France, ^4^Médecine Interne, AP‐HP, Hôpital Antoine‐Béclère, Clamart, France, ^5^Centre d'Hémato‐Immunologie Clinique (CHIC), Assistance Publique des Hôpitaux de Marseille (AP‐HM), Centre Hospitalo‐Universitaire (CHU) Sainte‐Marguerite, Marseille, France, ^6^HIV and STI, AP‐HP, Hôpital Victor‐Dupouy, Argenteuil, France, ^7^Maladies Infectieuses, CHU Nantes, Nantes, France, ^8^Maladies Infectieuses, AP‐HP, Hôpital Bichat, Paris, France, ^9^Maladies Infectieuses, AP‐HP, Hôtel Dieu, Paris, France, ^10^Maladies Infectieuses, AP‐HP, Pitié‐Salpêtrière, Paris, France, ^11^Centre d'Investigation Clinique (CIC), AP‐HP, Hôpital Cochin, Paris, France, ^12^Maladies Infectieuses, CHU Nice, Nice, France, ^13^Maladies Infectieuses, CHU Montpellier, Montpellier, France, ^14^INSERM, Département de Santé Publique, Sorbonne Université, AP‐HP, Hôpital Saint‐Antoine, Institut Pierre‐Louis d'Épidémiologie et de Santé Publique, Paris, France

**Background**: Dolutegravir‐based dual therapy (2‐DR) has demonstrated its efficacy as switching strategies in randomized clinical trials. Using real‐world data from the French ANRS CO4‐FHDH cohort, we investigated the impact of switching from an INSTIs‐based triple therapy (3‐DR) to dual therapy on virological and clinical outcomes.

**Materials and methods**: PWH who initiated a first INSTI‐based 3‐DR containing bictegravir, dolutegravir, elvitegravir or raltegravir between 2008 and 2018 for at least 1 year and had a controlled viral load (HIV‐RNA <50 cp/ml) before switching to an INSTI‐2‐DR were included. Switchers with at least 1 year of follow‐up were matched with up to four individuals who did not switch (non‐switchers) at the index date, defined as the date of the switch. Matching criteria included being eligible to the switch within 1 month of the switcher's eligibility [SG1] [EL2] date and being followed in a hospital with a similar proportion of switchers. To control for indication of switching bias, a pseudo‐population was emulated by inverse probability of switch weighting (IPW) to identify the hazard ratio (HR) between switchers and non‐switchers for virological failure, defined as the first of two consecutive HIV‐RNA >50 copies/ml, AIDS and death.

**Results**: Of the 78,145 participants who initiated an INSTI‐based 3‐DR, 41,619 met the eligibility criteria and 7643 (18%) have switched to INSTI‐dual therapy. In the IPW pseudo‐population, the total weighted number of participants was 8368 switchers and 19,452 non‐switchers. Median age at the index date was 51 years (interquartile range [IQR] 43–57), with no difference between switchers and non‐switchers, and median CD4 cell counts were 666 (476–888) and 683 (508–896)/mm^3^, respectively. Median follow‐up from the index date was 2.2 years in both groups. There were no differences in time to virological failure, AIDS or deaths between groups (Figure 1). In the IPW‐population, the HRs in switchers versus non‐switchers were 0.93 (95% CI 0.82–1.04) for virological failure (2072 events), 0.78 (0.51–1.19) for AIDS (162 events) and 1.05 (0.84–1.32) for death (501 events).

**P071: Figure 1:**
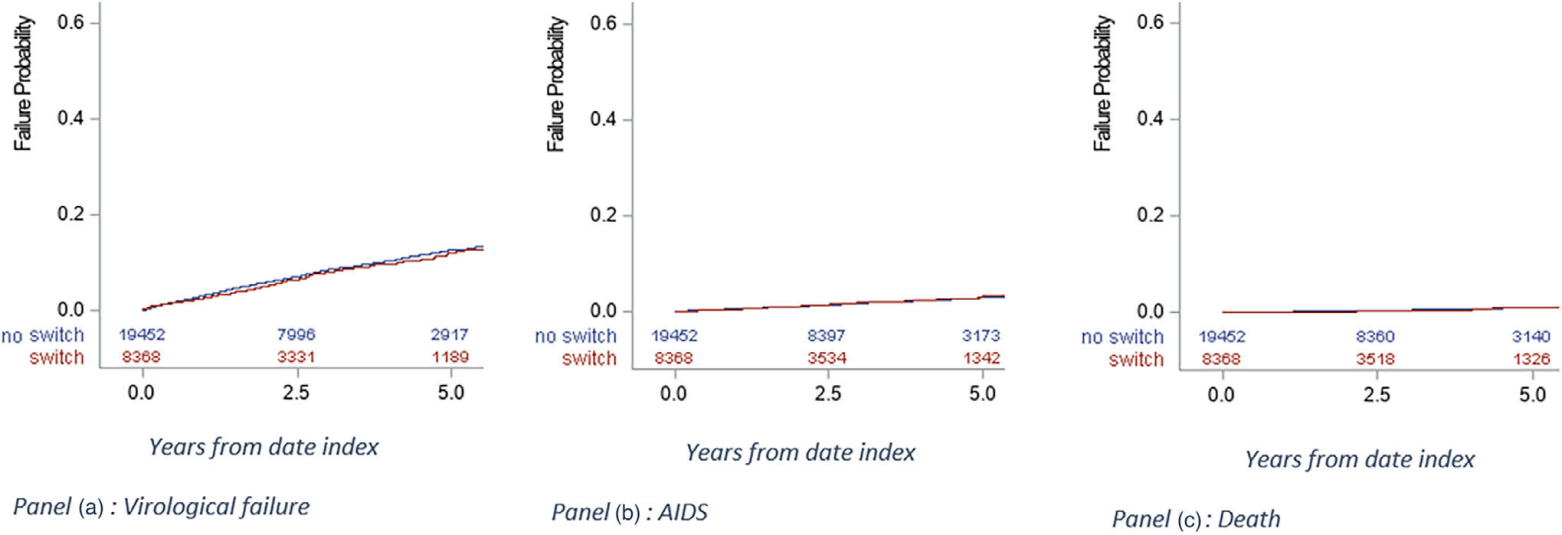
Time to virological failure, AIDS and death in switchers and non‐switchers.

**Conclusions**: Using IPW models on real‐world data, no difference was observed in virological and clinical outcomes between PWH on INSTI 3‐DR switching to INSTI 2‐DR or remaining on 3‐DR.

### P072 Central nervous system safety of long‐acting cabotegravir/rilpivirine in patients with previous oral INSTI‐related CNS toxicity

Ana Moreno ^1^, Santos Del Campo^1^, Maria Jesus Vivancos^1^, Manuel Velez^2^, Jose Luis Casado^1^, Javier Martinez^1^, Fernando Dronda^1^, Santiago Moreno^1^, Maria Jesus Perez‐Elias^1^

^1^Infectious Diseases, Hospital Ramón y Cajal, Madrid, Spain, ^2^Pharmacy, Hospital Ramón y Cajal, Madrid, Spain

**Background**: Central nervous system (CNS) toxicity is the main reason for treatment withdrawal in patients receiving oral integrase inhibitors (INSTI), and there is not information on the role of long‐acting (LA) cabotegravir/rilpivirine (C/R) in this setting.

**Materials and methods**: Evaluation of the CNS safety of C/R in patients with prior intolerance to oral INSTI in a clinical cohort of 321 patients starting C/R between January 2023 and June 2024 at an HIV outpatient clinic from a tertiary hospital in Madrid, Spain. C/R was administered baseline‐week 4 and each 8 weeks thereafter, without oral leading.

**Results**: Overall, 29 patients (9.03%) reported previous intolerance to oral INSTI, mostly dolutegravir *n* = 15 (52%) or bictegravir *N* = 12 (42%), being insomnia (*n* = 14, 48%), headache (*n* = 10, 35%) and nightmares (*n* = 5, 17%) the most common reason for withdrawal. Prior to C/R, 45% (*N* = 13) were on NNRTI‐based therapies: TAF/FTC/RPV in 11 and TAF/FTC plus doravirine in two. Table 1 shows the comparison in baseline features and evolutive safety in patients with and without prior oral INSTI‐related CNS toxicity. After a median follow‐up time of 10 months (1–24), the overall rate of C/R withdrawal was 5% (*N* = 16); the rate of CNS‐related withdrawal was 1.5% (*N* = 5, 1.5%), higher among patients with previous oral INSTI‐related toxicity (10% vs. 0.7%, *p* = 0.0002).

**P072: Table 1:** Comparison of baseline and evolutive features between patients with and without prior oral INSTI‐related CNS toxicity

Baseline features	Prior oral INSTI CNS toxicity (*n* = 29)	No prior oral INSTI toxicity (*n* = 292)	*p*‐value
Female at birth (*n*, %)	6 (21%)	35 (12%)	NS
Age (years, mean±SD)	52.13±11	44.7±12	0.002
BMI (mean±SD)	24±3	26±4	0.017
Years on ARV (mean±SD)	15±7	11±8	0.016
Lines of prior ARV (mean±SD)	7±4	4±4	0.005
Prior AIDS (%)	17	15	NS
CD4 cells/µl (mean±SD)	781±225	765±332	NS
Comorbidities (%)	83	52	0.001
Evolutive CNS safety			
CNS toxicity on C/R (*n*, %)	6 (21)	2 (0.7)	0.0001
Withdrawal due to CNS toxicity	3/29 (10%)	2/292 (0.7%)	0.002

**Conclusions**: In this cohort of heavily pretreated patients, the rate of CNS‐related toxicity withdrawal was very low (1.5%), but the incidence of CNS toxicity and CNS‐related withdrawals were significantly higher in patients with prior oral INSTI‐related CNS adverse events.

### P073 DTG/3TC as initial therapy in people living with HIV and viral load >500,000 copies/mL

Juan Martin^1^, Maria Lagarde ^1^, Judit Iglesias^2^, David Rial^1^, Otilia Bisbal‐Pardo^1^, Adriana Pinto^1^, Roser Navarro^1^, Mireia Santacreu^1^, Maria Teresa Lopez^1^, Laura Bermejo Plaza^3^, Maria Asuncion Hernando^3^, Rafael Rubio^4^, Federico Pulido Ortega^1^

^1^Unit HIV (Internal Medicine), Hospital Universitario 12 de Octubre, Madrid, Spain, ^2^Intensive Care Unit, Hospital Universitario La Princesa, Madrid, Spain, ^3^Unit HIV (Fundacion de Investigacion), Hospital Universitario 12 de Octubre, Madrid, Spain, ^4^Unit HIV (Internal Medicine), Hospital Universitario 12 de Octubre, Facultad Medicina Universidad Complutense de Madrid, Madrid, Spain

**Background**: The effectiveness of initial antiretroviral therapy (ART) with DTG/3TC in people living with HIV (PLWH) with HIV viral load (HIV‐VL) ≥500,000 copies/ml is not well established. This study aims to compare the effectiveness of DTG/3TC versus BIC/F/TAF at 24 weeks in ART‐naïve PLWH with HIV‐VL ≥500,000. Additionally, the study assesses immune recovery and safety profiles.

**Materials and methods**: This single‐centre retrospective cohort study included all PLWH with HIV‐VL ≥500,000 copies/ml initiating ART with either DTG/3TC or BIC/F/TAF in a monographic HIV unit. We conducted descriptive and bivariate comparative analyses to evaluate treatment effectiveness (HIV‐VL <50 copies/ml 24±2 weeks, an intention to treat efficacy analysis was performed, missing = failure [ITT M = F]), immune recovery (CD4 count) and safety profiles (weight, lipid profile and creatinine levels). A time‐to‐event analysis was performed using Kaplan‐Meier curves and log‐rank tests.

**Results**: A total of 73 patients (29% on DTG/3TC and 71% on BIC/F/TAF) were included. No baseline differences were observed between groups, except for CDC stage and immunological status. Detailed participant characteristics are provided in Table 1. The median time to reach undetectability was 91 days (IQR 64–116) for DTG/3TC and 88 days (IQR 63–130) for BIC/F/TAF. At 24 weeks, 90.5% (19/21) of participants in the DTG/3TC group and 88.5% (46/52) of those in the BIC/F/TAF group reached HIV‐VL <50 copies/ml (*p* = 1; Dif: 2%, CI 95% −18.3, 15.3). No ART discontinuations were observed in any of the therapeutic strategies. No significant differences in effectiveness were observed (Figure 1). In subgroup analyses (primary infection, HIV‐VL ≥1,000,000 copies/ml, CD4 >200 cells/mm^3^), median time to HIV‐VL <50 copies/ml showed no differences between regimens. Other sub‐analysis including only patients who initiated ART with CD4 >200 cells/mm^3^, immune recovery was independent of the ART regimen recovery (median increase of CD4 were 285 cells/mm^3^ [IQR 188–527] and 187 cells/mm^3^ [IQR 52–320] for DTG/3TC and BIC/F/TAF, respectively; *p* = 0.4). Both therapies demonstrated a good safety profile, with no significant differences in weight gain, lipid profile or creatinine levels.

**P073: Table 1:** Characteristics of the participants at baseline

		BIC/F/TAF (*n* = 52)	DTG/3TC (*n* = 21)	
		*n*; % (CI 95%)	*n*; % (CI 95%)	*p*‐value
Age at diagnosis (years)	median (IQR)	34 (27−46)	31 (26−35)	0.3
Sex at birth				1
Male		48; 92% (82−97)	20; 95% (77−99)	
Female		4; 8% (3−18)	1; 5% (1−23)	
Gender identity				1
Cisgender		51; 98% (90−100)	21; 100% (85−100)	
Transgender		1; 2% (0−10)	0 (0−15)	
Risk group				0.07
Heterosexual		18; 35% (24−49)	3; 14% (5−35)	
Homosexual		33; 65% (51−76)	18; 86% (65−95)	
Native‐born Spaniards		20; 38% (26−52)	9; 43% (24−63)	0.5
Weight (kg)	median (IQR)	67.5 (57−80)	71 (64−77)	0.3
Comorbidities				
Arterial hypertension		5; 10% (4−21)	2; 9% (3−29)	1
Diabetes mellitus		3; 6% (2−16)	0 (0−15)	0.5
Dyslipidaemia		9; 17% (9−30)	0 (0−15)	0.06
Smoking		20; 38% (26−52)	8; 38% (21−59)	0.3
CDC stages A1 and A2		16; 31% (20−44)	19; 90% (71−97)	<0.001
AIDS		34; 65% (52−77)	0 (0−15)	<0.001
Immunovirological status				
HIV‐VL baseline (cp/ml)	median (IQR)	1,575,916 (719,010−4,353,577)	1,156,084 (812,707−7,687,241)	0.9
HIV‐VL baseline ≥1,000,000 cp/ml		34; 65% (52−77)	14; 67% (45−83)	0.9
CD4 nadir (cells/mm^3^)	median (IQR)	122 (41−303)	320 (273−424)	<0.001
Time to ART initiation (days)	median (IQR)	7 (3−18)	10 (4−16)	0.5

**P073: Figure 1:**
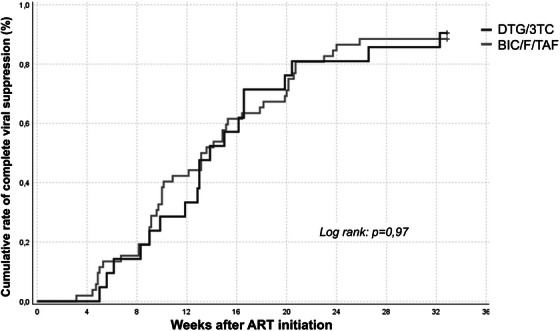
Kaplan‐Meier survival analysis of DTG/3TC and BIC/F/TAF groups from baseline to VL <50 copies/ml.

**Conclusions**: In PLWH initiating ART with HIV‐VL ≥500,000 copies/ml, DTG/3TC and BIC/F/TAF demonstrated similar effectiveness and safety profiles at 24 weeks.

### P074 Cabotegravir‐rilpivirine long‐acting injectable regimen: an analysis of the causes of interruption and impact of genotypic drug resistance in a multicentric cohort

Giada Canavesi ^1^, Maurizio Mena^1^, Elena Zaninetta^2^, Lidia Gazzola^2^, Teresa Bini^2^, Giancarlo Orofino^3^, Andrea De Vito^4^, Giordano Madeddu^4^, Chiara Grillo^5^, Claudia Bartalucci^6^, Federica Centorrino^6^, Nicola Squillace^7^, Paolo Bonfanti^7^, Giorgio Tiecco^8^, Emanuele Focà^8^, Marianna Menozzi^9^, Giovanni Guaraldi^9^, Nicolas Brian Bana^10^, Giovanni Cavazza^10^, Roberto Rossotti^10^, Sergio Lo Caputo^5^, Antonio Di Biagio^6^, Stefano Rusconi^1^

^1^Infectious Diseases Unit, Ospedale Nuovo di Legnano, ASST Ovest Milanese, Legnano, Italy, ^2^Infectious Diseases Unit, ASST Santi Paolo e Carlo, San Paolo Hospital and Dipartimento di Scienze della Salute, Milan, Italy, ^3^Infectious Diseases Unit, Amedeo di Savoia Hospital and University of Turin, Turin, Italy, ^4^Infectious Diseases Unit, Azienda Ospedaliera Universitaria di Sassari and University of Sassari, Sassari, Italy, ^5^Infectious Diseases Unit, Azienda Ospedaliero‐Universitaria “Ospedali Riuniti” di Foggia and University of Foggia, Foggia, Italy, ^6^Infectious Diseases Unit, Ospedale Policlinico San Martino and University of Genoa, Genova, Italy, ^7^Infectious Diseases Unit, IRCCS San Gerardo dei Tintori and University of Milan‐Bicocca, Monza, Italy, ^8^Infectious Diseases Unit, Spedali Civili di Brescia and University of Brescia, Brescia, Italy, ^9^Infectious Diseases Unit, Azienda Ospedaliera‐Universitaria di Modena and University of Modena and Reggio Emilia, Modena, Italy, ^10^Infectious Diseases Unit, ASST Grande Ospedale Metropolitano Niguarda, Milan, Italy

**Background**: The use of combination regimens is paramount in the treatment of HIV infection. Virologically suppressed patients may benefit to change their treatment in a two‐drug regimen (2DR). Long‐acting (L‐A) injectable 2DR may be a good option in selected patients.

**Methods**: We analysed the data supplied by 10 infectious diseases units, where CAB‐RPV regimen is available and administered, to make a descriptive analysis of the causes of interruption and the impact of genotyping drug resistance, when available.

**Results**: The total of patients receiving CAB‐RPV was 758; 66 interrupted the treatment. The average age of the patients of this cohort was 53 years and average BMI was 25.26 kg/m^2^ (BMI max 44.28 kg/m^2^). Before starting the injectables, 32 patients took a triple oral therapy (regimen mostly used: TAF/FTC/RPV), while 33 assumed a 2DR regimen (regimens mostly used: DTG/3TC‐DTG/RPV); as a whole, 42 took an INSTI‐based regimen. Only one PWH received a mono therapy. Sixteen had an oral lead in. As far as the genotypic resistance pattern, 46 patients had a genotypic resistance test (GRT) before starting CAB‐RPV; five patients had documented resistance to NNRTI (138A, which is an important risk factor of viral failure of RPV and others), while none had INSTI resistance. We collected 14 documented viral failures. All of them carried out a GRT post VF; the results are shown in Table 1. The other 52 patients interrupted L‐A therapy for other reasons (local pain, adverse events, toxicity, patient's choice, drugs interactions). The mean time of duration of L‐A regimen was 5.25 months: 6 (± 4.23 SD) months for patients with VF and 5.06 (± 4.69 SD) for patients who interrupted for other reasons, without statistical significance.

**P074: Table 1:** Mutations detected in patients experiencing VF (before and after VF)

	NRTI mutations (basal GRT)	NRTI mutations (after VF GRT)	NNRTI mutations (basal GRT)	NNRTI mutations (after VF GRT)	PI mutations (basal GRT)	PI mutations (after VF GRT)	INSTI mutations (basal GRT)	INSTI mutations (after VF GRT)
Patient 1	0	0	0	0	0	0	0	0
Patient 2	NA	**M41L; D67N; L210V; T215Y**	0	0	0	0	0	0
Patient 3	0	0	0	0	10I	0	0	0
Patient 4	0	0	0	0	0	0	0	0
Patient 5	0	**151M; 70R; 65R**	138A	**181I; 190A**	10F	**73S; 90M**	0	**140S; 148H**
Patient 6	0	0	0	**138K; 179I**	0	**10V**	0	**148R**
Patient 7	0	**D67N; K70R; M184V**	0	**K103N; V108I; P225H**	0	**K70R**	0	**G140S; Q148K**
Patient 8	T69A; S68G	S68G	0	0	0	0	0	**E138EK; G140S; G163R**
Patient 9	0	**V21V/I; V35V/I; V60/VI; K122E; D123E**	0	**V245E/K; A272P; K281R**	0	**A71T; V77I**	0	0
Patient 10		**M41L; D67N; L210V; T215Y**	NA	0	NA	0	NA	0
Patient 11	0	GRT in progress	0	GRT in progress	M36I; L63P; L89M	GRT in progress	0	GRT in progress
Patient 12	0	0	0	**S68GV; E138A; Y188H**	0	0	0	**E138EK; Q148R**
Patient 13	0	0	0	**E138K**	0	0	0	**N155NH; H51Y**
Patient 14	39A; 41L; 67N; 210W; 211K; 215Y; 218E; 219Q; M41L; D67N; K70R; Q151L; T215Y; K219Q; M41L; L74V; M184V; K219Q	M41L; D67N; K70R; L74V; M184V; T215Y; K219Q	101P; 103N; K101P; K103S; Y181C	**V106VI; N348I**	13V; 30N; 35D; 36V; 63P; 71V; 77I; 88DR; D30N; L33F; I50L; A71V; V82A; N88D; D30N	N88D	0	**G140S; Q148H; D232DN**

*Note*: Newly emerged mutations are in bold.

**Conclusions**: 1.8% of our cohort experienced a VF; this result is coherent with the main studies evaluating a low failure rate of CAB‐RPV. CAB‐RPV L‐A regimen was well‐tolerated. Respect of eligibility criteria and awareness of risk factors for VF plus strict monitoring of viro‐immunological parameters are fundamental in reducing the risk of VF and the possible onset of new NNRTI/INSTI resistance mutations.

### P075 Real‐life effectiveness, safety and acceptance of long‐acting cabotegravir‐rilpivirine: results from a large single‐centre cohort

Ana González‐Cordón, Montserrat Laguno, Leire Berrocal, Maria Martínez‐Rebollar, Berta Torres, Ivan Chivite, Alexy Inciarte, Paula Arreba, Juan Ambrosioni, Alberto Foncillas, Lorena de la Mora, José Luis Blanco‐Arevalo, Júlia Calvo, Esteban Martinez, Abiu Sempere, Jose M. Miró, Roger Llobet, Elisa de Lazzari, Josep Mallolas

Infectious Diseases, HIV Unit, Hospital Clínic, University of Barcelona, Institut d'Investigacions Biomèdiques August Pi i Sunyer (IDIBAPS), Barcelona, Spain

**Background**: Long‐acting intramuscular cabotegravir plus rilpivirine (LA‐CAB/RPV) is available in Spain since January 2023. We aim to analyse effectiveness, tolerability and acceptance, including patient‐reported outcomes (PROs), in a real‐world scenario in the first year of follow‐up.

**Materials and methods**: People starting LA‐CAB/RPV in Hospital Clínic Barcelona were invited to participate in an observational, prospective single‐centre study approved by the local Ethical Committee. Clinical information was routinely collected, and PROs were self‐administered through electronic questionnaires. We evaluated effectiveness, defined as HIV‐RNA <50 copies/ml (on treatment [OT], modified intention‐to‐treat [mITT] and intention to‐treat [ITT]), tolerability and safety in patients who reached week (w)28 and w52.

**Results**: From 2 Feb 2023 to 2 May 2024, 554 people with HIV started LA‐CAB/RPV (9% of total cohort), and 533 accepted to participate. Of these, 318 (60%) and 99 (19%) participants reached w28 and w52 of follow‐up, respectively. Baseline characteristics of the 318 participants who reached w28 are shown in Table 1. Eight (3%) participants underwent an oral lead‐in (OLI) phase, 96% of injections were administered within the window period and 2% of administrations were delayed. By OT, mITT and ITT, effectiveness was 98%, 92% and 91% at w28 and 97%, 93% and 92% at w52. There were four (1%) confirmed virological failures (defined as two consecutive viral loads [VLs] >50 cp/ml) all with low‐level viraemia (VL <200 cp/ml), two achieved VL <50 cp/ml without changing ART and two did so after switching to oral ART. By w28, 21 (7%) participants switched to oral ART: two (10%) virological failure, seven (33%) potential treatment‐related adverse events (TRAEs), seven (33%) patient preference, two (10%) medical decision and three (14%) changed location. From w28 to w52, one additional participant switched to oral ART due to medical decision. Besides injection site reactions (ISRs), 12 (4%) participants reported TRAE by w28, all grade 1–2. Acceptance of ISR and pain significantly improved during follow‐up (Figure 1). At w28, 87% of participants considered injection site reactions to be very or totally acceptable.

**P075: Table 1:** Baseline characteristics of participants who reach week 28 (*n* = 318)

Age, median (IQR)	45 (37−54)
Sex, male, *n* (%)	293 (92%)
Origin, *n* (%)	
Spanish	142 (45%)
Other European countries	43 (14%)
Latin America	104 (33%)
Other	29 (9%)
Mode of HIV acquisition, *n* (%)	
MSM	258 (82%)
Heterosexual	29 (9%)
IDU	21 (7%)
BMI >30 kg/m^2^, *n* (%)	28 (11%)
Years since diagnostic, median (IQR)	12 (8−19)
Undetectable VL (<50 cp/ml), *n* (%)	313 (98%)
Time with VL <50 cp/ml, median years (IQR)	8 (4−13)
Last CD4, cells/𝜇l, median (IQR)	724 (563−938)
Nadir CD4, cells/𝜇l, median (IQR)	335 (214−452)
Historic genotype available, *n* (%)	166 (52%)
Any non‐polymorphic mutation present	33 (10%)
Subtype A	4 (1%)
Years in ART, median (IQR)	9 (4−15)

Abbreviations: IDU, injection drug user; IQR, interquartile range; MSM, men who have sex with men; VL, viral load.

**P075: Figure 1:**
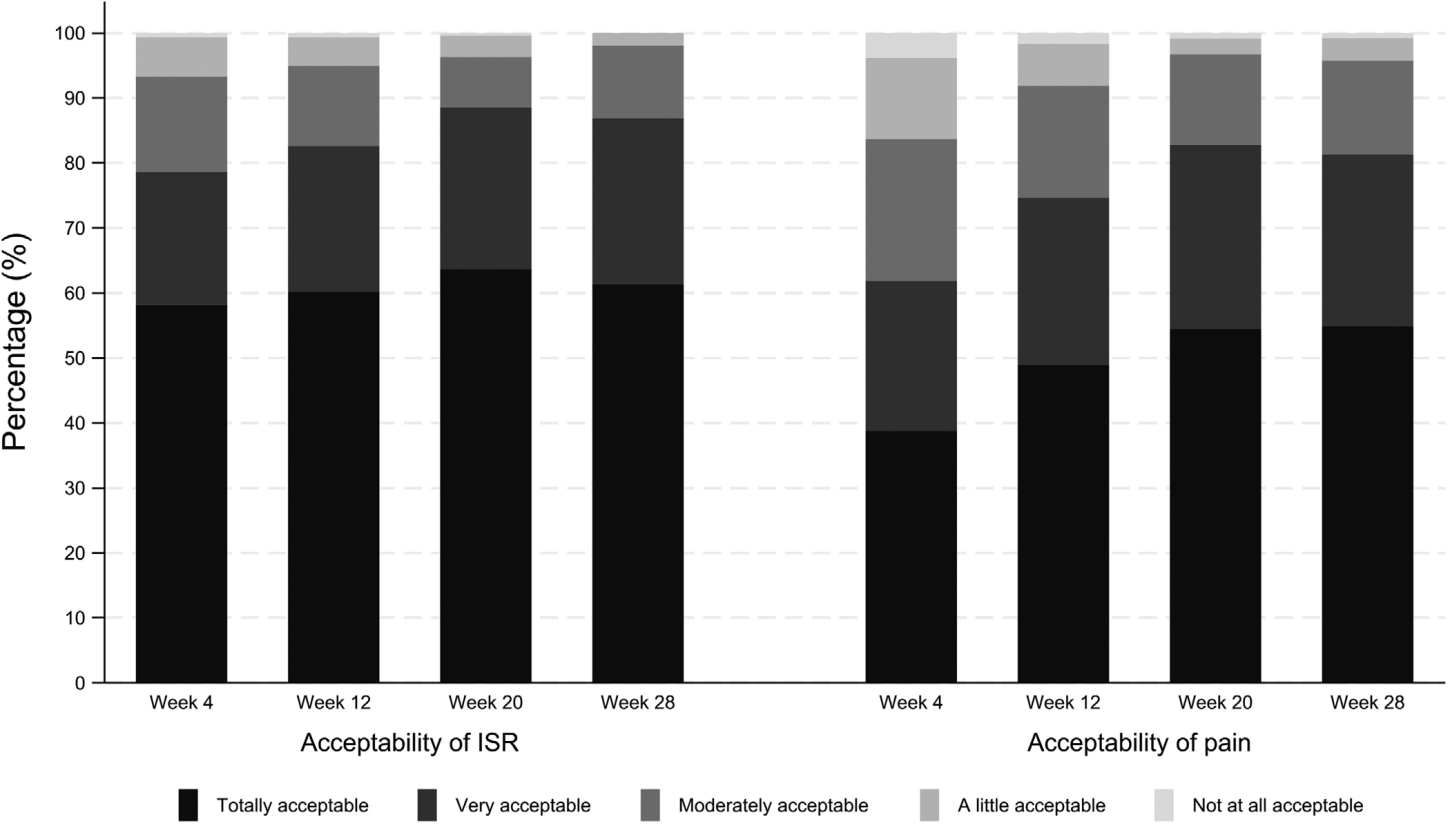
Acceptance of injection site reactions and pain.

**Conclusions**: LA‐CAB/RPV shows high effectiveness, tolerability and acceptance in a real‐life setting. Additional analysis on patient‐reported outcomes is ongoing.

### P076 Persistence, safety and virological outcomes of B/F/TAF as a baseline or switch regimen in HIV‐infected people living with advanced HIV disease in the real world: the BIC‐CD4 study

Diego Cecchini ^1^, Carla Serrano^2^, Martín Brizuela^1^, María Magdalena Puchulu^2^, Gastón Copertari^1^, Brenda Bacelar^1^, Juan Manuel Nuñez^3^, Juan Gonzalo Tomás^3^, Romina Mauas^1^, Macarena Roel^1^, Edgardo Bottaro^1^, Isabel Cassetti^1^

^1^Medical Area, Helios Salud, Buenos Aires, Argentina, ^2^Medical Area, Centro de Enfermedades Infecciosas y Parasitarias (CEIP), Tucumán, Argentina, ^3^Medical Area, Clínica Mayo UMCB, Tucumán, Argentina

**Background**: Real‐world data on B/F/TAF showed high levels of virological suppression (VS) in treatment‐naïve (TN) and experienced (TE) people living with HIV (PLWH). The World Health Organization defines advanced HIV disease (AHD) as a CD4 cell count <200 cells/mm^3^ or clinical stage 3 or 4 in adults. This population is underrepresented in clinical trials and real‐world studies. The BIC‐CD4 study aims to describe safety, persistence and VS (<50 c/ml) in adult PLWH who started B/F/TAF with CD4 <200/mm^3^.

**Materials and methods**: Retrospective multisite observational open cohort study. TN and TE PLWH who started B/F/TAF from October 2019 to January 2024 were registered in three HIV clinics in Argentina.

**Results**: Registries of 12,768 PLWH were screened, of which 3823 started B/F/TAF. Of them, 250 (6.5%) had CD4 T‐cell count <200/mm^3^: 132 TN (52.8%) and 118 TE (47.2%). Baseline characteristics: 74% male; median (IQR) age, 43 (35–51) years; >99% of Latin ethnicity. TN group had a median baseline viral load and CD4 count of 169,500 c/ml (37,250–473,000) and 94/mm^3^ (45–149); 18% had comorbidities. For TN, 24‐ and 48‐week persistence rates were 100% and 99%, VS was 81% and 83%, and CD4 significantly increased to a median of 236 (146–326) and 284 (195–416), respectively. Adverse event rate (AER) was zero. Of TE group, median CD4 count was 141/mm^3^ (92–177); 40% had comorbidities. Only 50% had VS when started B/F/TAF (TE‐undetectable: TEU); the rest had no VS (TENU). The TENU subgroup had lower CD4 (119 vs. 161, *p* < 0.001) and higher frequency of exposure to EFV and ATV/r, ongoing toxicity (31% vs. 14%) and virological failure (14% vs. 0%). Among TEU, predominant reasons for switching were simplification (58%) and toxicity prevention (28%). VS rates at 24 and 48 weeks for TEU and TENU were 98% versus 73% (*p* < 0.001) and 98% versus 89% (*p* = 0.2), respectively. Overall persistence was >99%, and AER was zero. Median CD4 increased to 190 (157–235) and 212 (178–281) at 24 and 48 weeks, respectively, without differences between subgroups.

**Conclusions**: B/F/TAF provided excellent levels of persistence, safety profile and adequate virologic suppression rates (VSRs) in PLWH with AHD.

### P077 Effectiveness of long‐acting ART with cabotegravir/rilpivirine in the ICONA cohort

Roberta Gagliardini^1^, Sara De Benedittis^2^, Alessandro Tavelli ^2^, Giuseppe Lapadula^3^, Valentina Mazzotta^1^, Elena Bruzzesi^4^, Adriana Cervo^5^, Giorgia Carrozzo^6^, Annalisa Sarracino^7^, Stefano Rusconi^8^, Giulia Marchetti^9^, Francesca Ceccherini‐Silberstein^10^, Andrea Antinori^1^, Antonella d'Arminio Monforte^2^, Camilla Muccini^4^

^1^UOC Immunodeficienze Virali, INMI Lazzaro Spallanzani, Rome, Italy, ^2^Icona Foundation, Milan, Italy, ^3^University of Milano Bicocca, Fondazione IRCCS San Gerardo dei Tintori, Monza, Italy, ^4^IRCCS Ospedale San Raffaele, Vita‐Salute San Raffaele University, Milan, Italy, ^5^Infectious Diseases Clinic, University Hospital of Modena, Modena, Italy, ^6^Luigi Sacco Department of Biomedical and Clinical Sciences, ASST Fatebenefratelli Sacco, Department of Infectious Diseases, Milan, Italy, ^7^Clinic of Infectious Diseases, Department of Precision and Regenerative Medicine and Ionian Area, Polyclinic of Bari, University of Bari, Bari, Italy, ^8^Infectious Diseases Unit, ASST Ovest Milanese, and Dipartimento di Scienze Biomediche e Cliniche, Università degli Studi di Milano, Ospedale Civile di Legnano, Milan, Italy, ^9^Clinic of Infectious Diseases, Department of Health Sciences, University of Milan, ASST Santi Paolo e Carlo, Milan, Italy, ^10^Department of Experimental Medicine, University of Rome, Rome, Italy

**Background**: Cabotegravir (CAB) + rilpivirine (RPV) long acting (LA) been effective and tolerable in phase III studies. Real‐life data on the effectiveness and discontinuation of CAB+RPV LA are limited.

**Methods**: All PWH enrolled in the ICONA cohort who started CAB+RPV LA as maintenance therapy with viral load (VL) under 50 cp/ml and with at least one follow‐up (FU) were included. Time to treatment discontinuation (TD) and to virological failure (VF, two consecutive VLs >50 cp/ml or one VL >1000 cp/ml followed by ART‐change) were estimated using the Kaplan‐Meier method. Cox regression models, adjusted for age, sex and mode of HIV transmission and stratified for centre were employed.

**Results**: Overall, 506 PWH started CAB+RPV LA, with a median FU of 8.7 months (interquartile range [IQR] 4.8–10.8). Main characteristics are in Table 1. Oral lead‐in was prescribed in 15% of cases. Forty‐seven treatment discontinuations were observed. One‐year estimated cumulative probability of TD was 13.3% (95% CI 9.7–18.1%). Causes of TD were toxicity/adverse events (6.5%), PWH's choice (2.2%), virological failure (0.2%), pregnancy (0.2%), drug‐drug interactions (0.2%). One‐year cumulative probability of TD for toxicity/adverse events was 10.1% (95% CI 6.7–14.3%). Multivariable analysis identified male sex, heterosexual intercourse and intravenous drug use (IDU) as risk factors for TD (Table 2), confirmed also in the analysis of TD for toxicity/adverse events. Two VFs were observed, with 1‐year cumulative probability of VF of 0.57% (95% CI 0.14–2.38%). One VF was in a subject with HIV subtype B/F1, no previous resistance‐associated mutations (RAMs) to NNRTI or INSTI, BMI 29.7 kg/m^2^, who failed with VL of 55 cp/ml and then 69 cp/ml and resuppressed without ART change. The other was in a subject with subtype B, no previous RAM to NNRTI, BMI 24.9 kg/m^2^, who failed with 636 and 66,500 cp/ml; resistance test showed K101K/E, E138E/A and E157Q at failure and ART was changed.

**P077: Table 1:** Main characteristics of PWH switching to CAB+RPV LA

Female sex, *n* (%)	57 (11.3%)
Italian nationality, *n* (%)	452 (88.7%)
Mode of HIV transmission, *n* (%)	
Heterosexual	129 (25.5%)
IDU	9 (1.8%)
MSM	344 (68%)
Other/unknown	24 (4.7%)
BMI, median (IQR)	24.4 (22.7−26.8)
BMI >30, *n* (%)	39 (7.7%)
CD4 at BL, median (IQR)	766 (590−959)
CD4 at nadir, median (IQR)	387 (243−532)
CD4 <200 at nadir, *n* (%)	98 (19.4%)
Age, median (IQR)	46 (37−54)
Age >50 years	165 (32.6%)
Years of viral suppression, median (IQR)	7.0 (3.7−9.6)
ART exposure, years, median (IQR)	7.3 (4.4−10.2)
Previous AIDS event, *n* (%)	42 (8.3%)
HBcAb positive, *n* (%)	79 (19.8%)
HCVAb positive, *n* (%)	35 (7.2%)
ART line, median (IQR)	4 (3−5)
GRT RT pre‐CAB/RPV, *n* (%)	415 (82.0%)
RPV fully susceptible, *n* (%)	405 (97.6%)
GRT INSTI pre‐CAB/RPV, *n* (%)	231 (45.7%)
CAB fully susceptible, *n* (%)	231 (100%)
HIV subtype, *n* (%)	
A1	7 (1.4%)
B	280 (55.3%)
Others	71 (14.0%)
Missing	148 (29.2%)

Abbreviations: GRT, genotype resistance test; INSTI, integrase strand transfer inhibitor; RT, retrotranscriptase.

**P077: Table 2:** Factors associated with treatment discontinuation at Cox regression analysis

	Unadjusted model			Adjusted model		
	HR	95% CI	*p*‐value	aHR	95% CI	*p*‐value
Female (vs. male)^a^	0.78	0.27−2.25	0.649	0.34	0.10−1.12	0.075
Age, per 10 years older^b^	1.00	0.76−1.31	0.997	0.93	0.70−1.23	0.605
Mode HIV transmission^c^						
MSM	1.00			1.00		
Hetero	2.26	1.10−4.68	0.027	3.60	1.53−8.47	0.003
IDU	4.21	0.95−18.63	0.058	4.80	1.07−21.55	0.041
Other/unknown	0.43	0.06−3.23	0.408	0.47	0.06−3.59	0.465
Italian (vs. non‐Italian born)^d^	1.17	0.41−3.32	0.771	0.95	0.33−2.75	0.92
BMI ≥25 (vs. <25 kg/m^2^)^d^	0.93	0.50−1.73	0.823	0.82	0.43−1.57	0.553
Oral lead‐in (vs. non‐oral lead‐in)^d^	2.35	0.88−6.27	0.088	2.11	0.75−5.90	0.156
HCV‐Ab pos (vs. HCV‐Ab neg)^d^	1.02	0.31−3.35	0.978	0.87	0.24−3.09	0.826
Previous AIDS event (vs. non‐AIDS)^d^	1.45	0.59−3.53	0.418	1.55	0.60−4.01	0.362
Previous NNRTI use (vs. no NNRTI use)^d^	0.87	0.47−1.63	0.668	0.92	0.48−1.75	0.76

^a^Adjusted for age and mode of HIV transmission.

^b^Adjusted for sex and mode of HIV transmission.

^c^Adjusted for age and sex.

^d^Adjusted for age, sex and mode of HIV transmission.

**Conclusions**: This analysis shows good short‐term effectiveness of CAB‐RPV LA, with a low rate of virological failure. A 13% probability of discontinuation overall and 10% for toxicity/adverse events emerged, higher than in phase III studies but similar to other real‐life data.

### P078 Use of long‐acting cabotegravir and rilpivirine in a real‐life setting: 12‐month results of virological outcome, adherence, safety, durability, in the ANRS CO3 AquiVIH‐NA cohort‐France

Mojgan Hessamfar^1^, Olivier Leleux^2^, Alaric Peyrouny‐Mazeau^2^, Camille Krzyzanowsky^1^, Gwenaël Le Moal^3^, Didier Neau^4^, Hélène Ferrand^5^, Arnaud Desclaux^4^, Estibaliz Lazaro^6^, Pierre Duffau^7^, Yann Gérard^8^, Marie‐Anne Vandenhende^9^, Fabrice Bonnet ^1^

^1^Service de Médecine Interne et Maladies Infectieuses, Hôpital Saint‐André, Centre Hospitalier Universitaire (CHU) de Bordeaux, Bordeaux, France, ^2^Bordeaux Population Health Research Center, INSERM U1219, CIC‐EC 1401, University of Bordeaux—ISPED, Bordeaux, France, ^3^Service de Maladies Infectieuses et Tropicales, Centre Hospitalier Universitaire (CHU) de Poitiers, Poitiers, France, ^4^Service de Maladies Infectieuses et Tropicales, Hôpital Pellegrin, Centre Hospitalier Universitaire (CHU) de Bordeaux, Bordeaux, France, ^5^Service de Maladies Infectieuses et Médecine Vasculaire, Centre Hospitalier de Libourne, Libourne, France, ^6^Service de Médecine Interne et Maladies Infectieuses, Hôpital Haut‐Lévèque, Centre Hospitalier Universitaire (CHU) de Bordeaux, Pessac, France, ^7^Service de Médecine Interne et Immunologie Clinique, Hôpital Saint‐André, Centre Hospitalier Universitaire (CHU) de Bordeaux, Bordeaux, France.  ^8^Service des Maladies Infectieuses, Centre Hospitalier de Dax, Dax, France, ^9^Service de Médecine Interne, Hôpital Pellegrin, Centre Hospitalier Universitaire (CHU) de Bordeaux, Bordeaux, France

**Background: **The first complete long‐acting antiretroviral therapy (ART) regimen, cabotegravir + rilpivirine long‐acting (CAB+RPV LA) injectable, was approved in France in December 2021, for HIV‐1 treatment in individuals on a stable antiretroviral regimen virologically suppressed (VS), with no virological failure (VF) history under NNRTIs or InSTIs, and no resistance to either cabotegravir or rilpivirine. We describe patient characteristics, safety and virological effectiveness of CAB+RPV LA in routine clinical care in South‐Western France.

**Methods**: From the ANRS CO3‐AQUIVIH‐NA cohort, all adults with HIV who received their first CAB+RPV LA injection from 1st January 2022 were included. VF was defined as 1 HIV RNA >1000 cp/ml or 2 consecutive HIV RNA >50 cp/ml; or no HIV RNA <50 cp/ml at 6 months in patients not virologically suppressed at baseline.

**Results**: Among 374 individuals who received at least one injection of CAB+RPV LA, 362 (97%) were VS at baseline. Five had a VL between 50 and 200 cp/ml and seven >200. One hundred and seventy‐nine (48%) received an oral lead‐in. At baseline, median age was 47 years (range 20–81), 98 (26%) were female sex at birth and two transgender women. Seventy‐four percent were born in France and 15% in sub‐Saharan Africa. Median BMI was 25.9 kg/m^2^ (IQR 22.7–27.8) and median LDL‐cholesterol (LDL‐C) 3 mmol/l (IQR 2.5–3.7). The median number of previous lines of ART was 4. At month 12, 12 patients experienced VF (3.2%) among which three were not VS at baseline and six had low‐level viraemia. Thirty‐eight patients (10.2%) had discontinued the treatment, seven (1.9%) for virological failure, 13 (3.5%) for adverse events (five [1.3%] for injection site reactions). No changes in BMI nor LDL‐C were observed.

**Conclusions: **In this large French cohort, 90% of patients continued CAB+RPV LA injections at month 12 and virological control was maintained. These results suggest that CAB+RPB LA injectable can be administered effectively during routine clinical care.

### P079 Switching to LA CAB/RPV in virologically suppressed PLHIV: does knowing previous genotyping really matter? A substudy from the RELATIVITY cohort

Luis Buzon Martin ^1^, María Luisa Montes^2^, María José Galindo Puerto^3^, Miguel Torralba^4^, Victoria Mancheño De Real^5^, María Aguilera García^6^, Alfonso Cabello Úbeda^7^, Isabel San Joaquín Conde^8^, Luis Morano^9^, Noemi Cabello Clotet^10^, Patricia Martín Rico^11^, Carmen Montero Hernández^12^, Miguel Alberto de Zárraga Fernández^13^, Sara de la Fuente Moral^14^, Ruth Calderón Hernaiz^14^, Enrique Bernal^15^, María Jesús Vivancos Gallego^16^, María Antonia Sepúlveda^17^, Roberto Pedrero Tomé^18^, Cristina Diez Romero^19^, Álvaro Cecilio^20^, Jara Llenas‐García^21^, Victor Arenas García^22^, Mar Masiá Canuto^23^, Juan Emilio Losa García^24^, Carlos Armiñanzas Castillo^25^, Antonio Jesús Sánchez Guirao^26^, María del Mar García Navarro^27^, Ana Cerezales Calviño^28^, María Ángeles Garcinuño Jiménez^29^, Eva Ferreira Pasos^30^, Miriam Estébanez^31^, Beatriz de la Calle Riaguas^32^, Miguel Egido Murciano^33^, Noemi Ramos Vicente^34^, Marta Clavero Olmos^35^, Juan Manuel Tiraboschi^36^, Jesús Troya^37^

^1^Infectious Diseases, Hospital Universitario de Burgos, Burgos, Spain, ^2^Infectious Diseases, Hospital Universitario La Paz, Madrid, Spain, ^3^Infectious Diseases, Hospital Clínico Universitario de Valencia, Valencia, Spain, ^4^Infectious Diseases, Hospital Universitario de Guadalajara, Guadalajara, Spain, ^5^HIV Unit, Infectious Diseases, Hospital Universitario 12 de Octubre, Madrid, Spain, ^6^Infectious Diseases, Hospital Universitario de la Princesa, Madrid, Spain, ^7^Infectious Diseases, Hospital Universitario Fundación Jiménez Diaz, Madrid, Spain, ^8^Infectious Diseases, Hospital Clínico Universitario Lozano Blesa, Zaragoza, Spain, ^9^Infectious Diseases, Hospital Universitario Álvaro Cunqueiro, Vigo, Spain, ^10^Infectious Diseases, Hospital Universitario Clínico San Carlos, Madrid, Spain, ^11^Infectious Diseases, Hospital de Denia Marina Salud, Alicante, Spain, ^12^Infectious Diseases, Hospital de Torrejón, Madrid, Spain, ^13^Infectious Diseases, Hospital Universitario San Agustín, Avilés, Spain, ^14^Infectious Diseases, Hospital Universitario Puerta de Hierro, Madrid, Spain, ^15^Infectious Diseases, Hospital Universitario Reina Sofía, Murcia, Spain, ^16^Infectious Diseases, Hospital Universitario Ramón y Cajal, Madrid, Spain, ^17^Infectious Diseases, Hospital Universitario de Toledo, Toledo, Spain, ^18^Fundación para la Investigación e Innovación Biomédica, Hospital Universitario Infanta Leonor, Madrid, Spain, ^19^Infectious Diseases, Hospital Universitario Gregorio Marañón, Madrid, Spain, ^20^Infectious Diseases, Hospital Universitario Miguel Servet, Zaragoza, Spain, ^21^Infectious Diseases, Hospital Comarcal de la Vega Baja, Alicante, Spain, ^22^Infectious Diseases, Hospital Universitario de Cabueñes, Gijon, Spain, ^23^Infectious Diseases, Hospital General Universitario de Elche, Elche, Spain, ^24^Infectious Diseases, Hospital Universitario de Alcorcón, Alcorcón, Spain, ^25^Infectious Diseases, Hospital Universitario Marques de Valdecilla, Santander, Spain, ^26^Infectious Diseases, Hospital General Universitario Morales Meseguer, Murcia, Spain, ^27^Infectious Diseases, Hospital Universitario de Vinalopo, Alicante, Spain, ^28^Infectious Diseases, Hospital Universitario Doctor José Molina Orosa, Las Palmas, Spain, ^29^Infectious Diseases, Complejo Asistencial de Ávila, Ávila, Spain, ^30^Infectious Diseases, Complejo Asistencial de Segovia, Segovia, Spain, ^31^Infectious Diseases, Hospital Central de la Defensa Gómez Hulla, Madrid, Spain, ^32^Pharmacy, Hospital General Nuestra Señora del Prado, Talavera de la Reina, Spain, ^33^Infectious Diseases, Hospital Universitario San Jorge, Huesca, Spain, ^34^Infectious Diseases, Hospital Obispo Polanco, Teruel, Spain, ^35^Infectious Diseases, Hospital Universitario Infanta Elena, Madrid, Spain, ^36^Infectious Diseases, Hospital Universitario de Bellvitge, Barcelona, Spain, ^37^Infectious Diseases, Hospital Universitario Infanta Leonor, Madrid, Spain

**Background**: Resistance‐associated mutations (RAMs) to RPV and/or INSTI, along with BMI >30 and HIV subtype A1/A6, increase the risk of CAB/RPV‐associated virological failure. The aim of the current substudy is to compare efficacy outcomes in real life in virologically suppressed PLHIV who switched to CAB/RPV according to whether previous genotyping was available or not at the time of switching.

**Methods**: The RELATIVITY cohort is a nationwide Spanish cohort of PLHIV older than 18 years old treated with CAB/RPV. Patients coming from CAB/RPV clinical trials are not included. One thousand, two hundred and eighty‐five cases from 37 institutions are described. Quantitative variables are compared using the U‐Mann‐Whitney test and qualitative variables using chi‐square and/or Fisher's exact test.

**Results**: The group in which previous genotyping results were available comprised 675/1285 cases (52.5%). Within this group, Spanish nationality was more common (74% vs. 67.7%; *p* = 0.012), people were older (46.0 [37.0–56.0] vs. 44.0 [37.0–52.0]; *p* = 0.012) and had more psychiatric disorders (11.1% vs. 7.0%; *p* = 0.015). Besides, blips (33.0% vs. 15.0%; *p* = 0.005) and virological failures before switching (5.9% vs. 3.2%; *p* < 0.001) were more frequent in it. Time on ART until switch to LA CAB/RPV was significantly shorter in this group (9.0 [5.0–12.0] vs. 10.0 [6.0–18.0] years; *p* < 0.001), as was the length of the period with undetectable viral load in plasma before switching (73.0 [37.2–117.0] vs. 96.0 [45.0–156.0] months; *p* < 0.001). Switching from DTG/RPV (27.5% vs. 19.6%; *p* < 0.001) or DRV/b‐based regimens (8.2% vs. 3.6%; *p* < 0.001) was more frequent when genotyping was unavailable. There were no differences in time of follow up, CD4 T‐cell count at the time of switch, abandon rate or the development of virological failure.

**Conclusions**: Our results suggest that unavailability of previous genotyping does not seem to increase the risk of virological failure in virologically suppressed PLHIV who switch to LA CAB/RPV. These seem to line up with the results of the CARES study [1]. Nevertheless, a longer follow up is required to reach solid conclusions.

**Reference**

1. Kityo C, Mambule IK, Musaazi J, Sokhela S, Mugerwa H, Ategeka G, et al. Switch to long‐acting cabotegravir and rilpivirine in virologically suppressed adults with HIV in Africa (CARES): week 48 results from a randomised, multicentre, open‐label, non‐inferiority trial. Lancet Infect Dis. 2024:S1473‐3099(24)00289‐5. Epub ahead of print.

### P080 To describe the prevalence of virological failure and resistance patterns to long‐acting cabotegravir‐rilpivirine: a real‐life single‐centre cohort study

Ana González‐Cordón ^1^, Alexy Inciarte^1^, Mar Mosquera^2^, Leire Berrocal^1^, Maria Martinez‐Rebollar^1^, Berta Torres^1^, Ivan Chivite^1^, Paula Arreba^1^, Juan Ambrosioni^1^, Alberto Foncillas^1^, Lorena De La Mora^1^, Julia Calvo^1^, Abiu Sempere^1^, Jose M. Miró^1^, Roger Llobet^1^, Elisa de Lazzari^1^, Josep Mallolas^1^, Esteban Martinez^1^, Montserrat Laguno^1^, Jose Luis Blanco‐Arevalo^1^

^1^Infectious Diseases, Hospital Clinic, Barcelona, Spain.^2^Microbiology, Hospital Clinic, Barcelona, Spain

**Background**: Long‐acting intramuscular cabotegravir plus rilpivirine (LA‐CAB/RPV) is currently used as maintenance treatment for HIV‐1. There is scarce information about virological failures (VFs) and resistance selection after the VF to LA‐CAB/RPV. Our objective is to perform an in‐depth analysis of subjects who presented a VF in a real‐world setting in the 28 weeks of follow‐up.

**Materials and methods: **Patients starting LA‐CAB/RPV at Hospital Clínic Barcelona were invited to participate in an observational, prospective single‐centre study. We performed an in‐depth clinical and virological analysis in cases with VF (two consecutive VL >50 cp/ml) during LA‐CAB/RPV therapy. Genotypic resistance testing by ultra deep sequencing (UDS) of RNA and/or DNA was conducted post‐VF.

**Results: **From 2 Feb 2023 to 2 May 2024, 554 people with HIV started LA‐CAB/RPV and 318 (60%) participants reached week 28 of follow‐up. Four subjects (1.25%) presented VF at week 28. All were male; three had BMI <30; all but one had HIV‐1 subtype B; baseline VL was >50 cp/ml in two subjects (88 and 154 cp/ml); no patient had a previous VF or selection of NNRTI resistance mutations prior to the initiation of LA‐CAB/RPV. Adherence to treatment was good in all subjects except for one who delayed one dose by 2 weeks. VL at the time of VF was less than 200 copies/ml in all individuals. In three of the four participants, it was possible to sequence the virus at the time of failure by UDS on proviral DNA showing genotypic resistance mutations in all of them (three to NNRTI and two to INSTI). However, all detected mutations may reflect apolipoprotein B mRNA editing enzyme, catalytic polypeptide (APOBEC) activity which makes it difficult to interpret their impact on the CAB/RPV resistance. Two individuals achieved VL <50 cp/ml without ART switch, and two did so after switching the ART (Table 1).

**P080: Table 1:** Clinical and virological characteristics of the four subjects on VF

ID	Age	HIV‐subtype	BMI	Prior Tx	Baseline VL	Needles	Adh	VF week	First VL at VF	UDS‐DNA GRT in first VL at VF	Second VL at VF	UDS‐DNA GRT in second VL at VF	Tx after VF	Last VL
37	50	B	27	ABC/3TC/DTG	154	23G	OK	20	154	RT: 138K (20%), 184I (57%), 230I (60%); PR: 30N (6%), 46I (5%); IN: NA	137	RT: 138K (11%), 184I (57%), 230I (68%); IN: 163R (4%)	DRVc/FTC/TAF	60
142	41	B	24	DTG/RPV	88	23G	OK	4	88	RT: 184I (19%), 138K (10%), 230I (21%); PR: 90M (97%); IN: NA	89	R: 184I (19%), 230I (40%); PR: 90M (90%); IN: 140S (8%)	DRVc/FTC/TAF	74
297	32	UKN	24	B/F/TAF	<50	23G	2 weeks delay	20	210	ND	53	NA	No change	<50
309	63	B	46	B/F/TAF	<50	21G	OK	20	53	WT	59	RT: 190E (22%); IN: NA since 260 position	No change	<50

Abbreviations: Adh, adherence; BMI, body mass index; IN, integrase gene; NA, not amplified; ND, not done; PR, protease gene; RT, retrotranscriptase gene; Tx, treatment; UDS‐DNA GRT, genotypic resistance test on DNA by ultra deep sequencing; VF, virological failure; VL, viral load; WT, wild type.

**Conclusions: **In our series, at week 28, the rate of VF (two consecutive VL >50 cp/ml) was low (1.25%), all of them were with low‐level viraemias (<200 copies/ml) and the resistance mutations detected may reflect APOBEC activity and should be carefully interpreted by a resistance specialist.

### P081 Long‐acting cabotegravir and rilpivirine in HIV individuals with a BMI over 30: a real‐world study (RELATIVITY cohort)

Jesús Troya ^1^, Luis Morano^2^, Jose Ignacio Bernardino^3^, Luis Buzon Martin^4^, Roberto Pedrero Tomé^5^, María José Galindo Puerto^6^, Miguel Torralba^7^, Noemí Cabello Clotet^8^, María García López^9^, Silvia Lope^10^, Miguel Alberto de Zárraga Fernández^11^, Alfonso Cabello^12^, María Aguilera García^13^, Álvaro Cecilio^14^, Alberto Díaz de Santiago^15^, María Ángeles Garcinuño Jiménez^16^, Enrique Bernal^17^, María Teresa López Caballero^18^, Ruth Calderón Hernaiz^19^, María Jesús Vivancos Gallego^20^, Teresa Omiste Sanvicente^21^, Eva Ferreira Pasos^22^, Juan Emilio Losa García^23^, Josefa Francisca Soler González^24^, María Antonia Sepulveda^25^, María del Mar García Navarro^26^, on behalf of the RELATIVITY Multicentre Cohort^27^

^1^Internal Medicine, Hospital Universitario Infanta Leonor, Madrid, Spain, ^2^Internal Medicine, Hospial Álvaro Cunqueiro, Vigo, Spain, ^3^Infectious Diseases, Hospital Universitario La Paz, Madrid, Spain, ^4^Internal Medicine, Hospital de Burgos, Burgos, Spain, ^5^Science Investigation, Fundación para la Investigación e Innovación Biomédica del Hospital Infanta Leonor, Madrid, Spain, ^6^Internal Medicine, Hospital Clínico Universitario de Valencia, Valencia, Spain, ^7^Internal Medicine, Hospital Universitario de Guadalajara, Guadalajara, Spain, ^8^Internal Medicine, Hospital Universitario Clínico San Carlos, Madrid, Spain, ^9^Internal Medicine, Hospital Comarcal la Vega Baja, Alicante, Spain, ^10^Internal Medicine, Hospital de Denia Marina Salud, Alicante, Spain, ^11^Internal Medicine, Hospital Universitario San Agustín, Avilés, Spain, ^12^Infectious Diseases, Hospital Universitario Fundación Jiménez Díaz, Madrid, Spain, ^13^Infectious Diseases, Hospital Universitario La Princesa, Madrid, Spain, ^14^Internal Medicine, Hospital Universitario Miguel Servet, Zaragoza, Spain, ^15^Internal Medicine, Hospital Universitario Puerta de Hierro, Madrid, Spain, ^16^Internal Medicine, Complejo Asistencial de Ávila, Ávila, Spain, ^17^Internal Medicine, Hospital General Universitario Reina Sofía, Murcia, Spain, ^18^Internal Medicine, Hospital Universitario 12 de Octubre, Madrid, Spain, ^19^Internal Medicine, Hospital Universitario de Fuenlabrada, Madrid, Spain, ^20^Infectious Diseases, Universitario Ramón y Cajal, Madrid, Spain, ^21^Internal Medicine, Hospital Universitario San Jorge, Huesca, Spain, ^22^Internal Medicine, Complejo Asitencial de Segovia, Segovia, Spain, ^23^Infectious Diseases, Hospital Universitario de Alcorcón, Alcorcón, Spain, ^24^Internal Medicine, Hospital Universitario de Cabueñes, Cabueñes, Spain, ^25^Internal Medicine, Hospital Universitario de Toledo, Toledo, Spain, ^26^Intrnal Medicine, Hospital Universitario de Vinalopo, Alicante, Spain, ^27^Infectious Diseases, Multicenter Cohort, Spain

**Background**: Switching to long‐acting cabotegravir and rilpivirine (CAB+RPV) has emerged as a standard approach for people living with HIV, offering high efficacy, safety and convenience. Nevertheless, there is a scarcity of data regarding people with a body mass index (BMI) over 30, a factor potentially related to virological failure in studies.

**Materials and methods**: We conducted a multicentre, non‐controlled, retrospective study on HIV virologically suppressed individuals who switched to long‐acting CAB+RPV (RELATIVITY cohort). We evaluated demographic and clinical factors in individuals with a BMI >30.

**Results**: The study included 113 individuals from 25 hospitals in Spain, 8.3% of the RELATIVITY cohort with 1366 individuals. The median age was 48 years (41–54), with 78.8% being men. 68.5% were Spaniards, followed by 20.3% South Americans. HIV transmission comprised 50.5% MSM and 34.6% heterosexual transmission. The median duration of previous oral antiretroviral therapy was 10.7 years (6.0–17.0), and viral suppression was 8.0 years (4.6–12.0). Individuals were in 19.1% in CDC stage C3. The nadir CD4^+^ cell count was 289 cells/mm^3^ (105–455), and 4.5% had a previous virological failure with resistance mutations (four to PIs and one to INSTI). The most frequent antiretrovirals strategies before switching included DTG/3TC (27.4%), DTG/RPV (23%), BIC/FTC/TAF (20.3%) and DRV/c/FTC/TAF (14.2%). The main reasons for switching were improvement in quality of life (50.4%), patient request (44.2%) and simplification (29.2%). The median BMI at switching was 32.3 [30.9–34.1]. A 38‐mm needle was used in 56.7% of cases. Intramuscular injections were applied to the dorsogluteal location in 79.3% of individuals. At week 24, one patient had an isolated detectable viral load of 57 copies/ml but continued treatment. At week 39, there were two virological failures, both with previous resistance mutations (one to INSTIs, also using a short needle and the other to NNRTIs). Three discontinuations due to injection site side effects occurred at weeks 21, 29 and 30.

**Conclusions**: In a real‐life setting, switching to long‐acting CAB+RPV seems to be a viable option for individuals with a BMI over 30, lining up with other cohorts. Further investigation is needed.

### P082 CARAVEL: evaluation of real‐world effectiveness and sustainability of the two‐drug regimen dolutegravir/lamivudine fixed‐dose combination in treatment‐naive adults and pre‐treated adults who are virologically suppressed, in routine clinical care, in France: 2‐year interim analysis results

Patrick Philibert ^1^, Charlotte Charpentier^2^, Nilakshani Naguleswaran^3^, Caroline Philippe^3^, Laetitia Roustand^4^, Corinne Vouillot^5^, Laurent Hocqueloux^6^

^1^Hôpital Européen Marseille, Bouches‐du‐Rhône, Marseille, France, ^2^Hôpital Bichat‐Claude Bernard, Paris, France, ^3^QUALEES, Paris, France, ^4^GlaxoSmithKline, Hauts‐de‐Seine, Rueil‐Malmaison, France, ^5^ViiV Healthcare, Hauts‐de‐Seine, Rueil‐Malmaison, France, ^6^Centre Hospitalo‐Universitaire Orléans, Loiret, Orléans, France

**Background**: This study is conducted to supplement data gathered from clinical trials with real‐world evidence. The primary objective is to describe virological outcome of DTG/3TC for the initial suppression of HIV replication in treatment‐naïve people living with HIV (TN‐PLWH), as well as maintaining virological suppression in pre‐treated PLWH (PT‐PLWH) who are virologically suppressed.

**Materials and methods**: CARAVEL is a French, prospective, non‐interventional, single‐arm, multi‐centre, 3‐year cohort study in TN‐PLWH and PT‐PLWH receiving DTG/3TC for the first time in accordance with the Licence. Presented here are the 2‐year interim analysis results.

**Results**: Forty‐nine centres included 304 patients: 56 TN‐PLWH (mean age at baseline 37 years, 84% men) and 248 PT‐PLWH (mean age at baseline 51 years, 75% men). Forty‐nine TN‐PLWH and 234 PT‐PLWH had at least one viral load (VL) measurement within 6 months of DTG/3TC initiation. Initial virological suppression (VL <50 c/ml) was attained for 44 (89.8%) TN‐PLWH after 6 months of initiating DTG/3TC, with a median time to virological suppression of 1.1 (±1.0) month, and no virological failure during the 2‐year follow‐up. Two years after switch to DTG/3TC, 222 (94.8%) PT‐PLWH maintained a VL <50 c/ml. During the follow‐up, 12 PT‐PLWH did not maintain a VL <50 c/ml: six due to intermittent viraemia and six due to virological failure (VF). Among the six patients with a VF, a genotypic resistance test was available for two patients of whom one developed 3TC resistance [M184V/I] due to non‐adherence; four out of six discontinued DTG/3TC. Overall, DTG/3TC was discontinued for 56 PLWH: seven TN‐PLWH and 49 PT‐PLWH, mainly due to patient's request (*N* = 36). More than half of these patients (*N* = 19; 54.3%) were switched to long‐acting injectable cabotegravir + rilpivirine (CAB/RPV‐LA). Median change in body weight over the first 2 years was 0.5 kg in TN‐PLWH (IQR −2.0, +2.0; *n* = 33) and 0.0 kg in PT‐PLWH (IQR −3.0, +2.0; *n* = 170).

**Conclusions: **These real‐world data demonstrated high virological success after 2 years on DTG/3TC in both TN‐PLWH and PT‐PLWH populations, without significant weight change. The main reason for DTG/3TC discontinuation remains the patient's request to switch to CAB/RPV‐LA.

### P083 Incidence of comorbidities over 18 months with BIC/FTC/TAF, DTG/ABC/3TC or DTG/3TC in real‐life settings in the ANRS‐CO3‐AquiVIH‐NA cohort

Olivier Leleux ^1^, Alaric Peyrouny‐Mazeau^1^, Adélaïde Perrier^1^, Mojgan Hessamfar^2^, Gwenaël Le Moal^3^, Didier Neau^4^, Laure Alleman^5^, Charles Cazanave^4^, Estibaliz Lazaro^6^, Pierre Duffau^2^, Agnés Riché^7^, Yann Gérard^8^, Marie‐Anne Vandenhende^9^, Fabrice Bonnet^2^

^1^Bordeaux Population Health Research Center, INSERM U1219, CIC‐EC 1401, Univ. Bordeaux—ISPED, 33076, Bordeaux, France, ^2^Service de Médecine Interne et Maladies Infectieuses, Hôpital Saint‐André, Centre Hospitalier Universitaire (CHU) de Bordeaux, Bordeaux, France, ^3^Service de Maladies Infectieuses et Tropicales, Centre Hospitalier Universitaire (CHU) de Poitiers, Poitiers, France, ^4^Service de Maladies Infectieuses et Tropicales, Hôpital Pellegrin, Centre Hospitalier Universitaire (CHU) de Bordeaux, Bordeaux, France, ^5^Service de Maladies Infectieuses, Centre Hospitalier de la Côte Basque, Bayonne, France, ^6^Service de Médecine Interne et Maladies Infectieuses, Hôpital Haut‐Lévèque, Centre Hospitalier Universitaire (CHU) de Bordeaux, Pessac, France, ^7^Service de Medecine Interne, Centre Hospitalier d'Angoulême, Angoulême, France, ^8^Service de Médecine Interne et Maladies Infectieuses, Centre Hospitalier de Dax, Dax, France, ^9^Service de Médecine Interne, Hôpital Pellegrin, Centre Hospitalier Universitaire (CHU) de Bordeaux, Bordeaux, France

**Background**: InSTI‐based regimen with bictegravir (BIC) and dolutegravir (DTG) are widely used in switch settings and have shown high rates of efficacy and good safety profiles in trials. However, the incidence of comorbidities with these regimens is underreported in real‐life settings.

**Methods**: We performed a retrospective analysis on subjects from the French regional prospective cohort ANRS‐CO3‐AquiHIV‐NA. We included subjects switching to BIC/FTC/TAF, DTG/ABC/3TC or DTG/3TC between 2018/01/01 and 2021/12/31 with a suppressed HIV plasma RNA <50 cp/ml, an available CD4 count at baseline and who completed 18 months of follow‐up.

**Results**: One thousand, eight hundred and sixty‐seven PLWH were included in the analysis: 1179 on BIC/FTC/TAF, 192 on DTG/ABC/3TC and 491 on DTG/3TC, respectively. At inclusion, median age was 53.1, 54.6 and 54.5; women were 26.4%, 33.3% and 25.7%; median BMI was 24.5, 24.3 and 24.2 kg/m^2^. Subjects in the BIC/FTC/TAF group had more HBV co‐infection, more previous exposition to TDF, less comorbidities, less frequently estimated glomerular filtration rate (eGFR) <60 ml/minute and less co‐prescription of statins, antidiabetics, and antihypertensive treatments. After 18 months of follow‐up, the comorbidity incidence density per 1000 person‐years (PY) rate (CI 95%) were: eGFR <60 ml/minute 10.7 (6.4–17.7), 34.0 (15.3–75.7), 30.5 (18.7–49.8); diabetes 18.4 (12.5–27.0), 16.3 (5.3–50.6), 19.1 (10.6–34.5); osteoporosis 7.4 (4.1–13.4), 10.0 (2.5–39.9), 6.5 (2.5–17.4); cancer 9.4 (5.4–16.1), 5.5 (0.8–38.9), 10.8 (4.8–23.9) cardiovascular event 10.0 (5.9–16.9), 16.1 (5.2–49.8), 21.1 (12.0–37.1) including: myocardial infarction 4.0 (1.8–8.9), 14.6 (4.7–45.1), 13.0 (6.5–26.0), CNS vascular event 4.5 (2.2–9.5), 0, 6.2 (2.3–16.6), peripheral vascular event 4.6 (2.2–9.7), 5.1 (0.7–35.9), 8.1 (3.4–19.5), hypertension 64.0 (49.0–83.6), 151.7 (91.5–251.7), 81.2 (56.5–116.9).

**Conclusions**: Baseline characteristics of patients treated with these strategies are different with more patients at high cardiovascular profile in the DTG groups. At month 18, we observed similar incidence rates of new diagnosis of diabetes, osteoporosis and cancer between the three strategies. We observed a trend for higher incidence of hypertension in the DTG/ABC/3TC group and of eGFR <60 ml/minute in the DTG groups than in the BIC/FTC/TAF group.

### P084 Real‐world utilization of cabotegravir + rilpivirine in Southern Spain: data from the CARIPLA study

Rosario Palacios ^1^, Cristina Gómez‐Ayerbe^1^, Marisa Mayorga^2^, Nuria Espinosa^3^, Alicia Hidalgo^4^, Francisco Téllez^5^, Ana Belén Lozano^6^, Mónica Loring^7^, Jesús Santos^1^

^1^Infectious Diseases Unit, Hospital Universitario Virgen de la Victoria, Málaga, Spain, ^2^Infectious Diseases Unit, Hospital Regional Carlos Haya, Málaga, Spain, ^3^Infectious Diseases Unit, Hospital Virgen del Rocío, Sevilla, Spain, ^4^Infectious Diseases Unit, Hospital Juan Ramón Jiménez, Huelva, Spain, ^5^Infectious Diseases, Hospital Puerto Real, Cádiz, Spain, ^6^Infectious Diseases Unit, Hospital El Ejido, Almería, Spain, ^7^Internal Medicine, Hospital de La Axarquía, Málaga, Spain

**Background: **Cabotegravir+rilpirivine (CAB+RPV) is the first complete long‐acting (LA) antiretroviral therapy (ART) approved for the treatment of ART‐experienced people with undetectable viral load (VL). Our objective was to analyse the efficacy and safety of CAB+RPV in HIV‐infected patients who switched from any other ART in real‐world settings.

**Patients and methods: **Open‐label, multicentre study including patients who switched to CAB+RPV between 23 Jan and 24 May. Epidemiological, clinical and immunovirological characteristics were recorded. Primary endpoint: percentage of patients with HIV‐VL <50 cp/ml at 24 and 48 weeks. Secondary endpoints: percentage of patients who discontinued CAB+RPV and their reasons. Statistic programme: SPSS 24.0.

**Results: **A total of 281 participants were included: 245 (87.2%) men, median age 44.2 years, three (13.2%) AIDS cases; median time on ART 133.4 months, with a median of 3 prior ART regimens; median baseline CD4 cells count was 774 cells/mm^3^; all but one had VL <200 copies/ml (90.4% <20 copies/ml, 4.2% 20–50 copies/ml, 2.1% 50–100 copies/ml, 1.4% 100–200 copies/ml). Two patients had NNRTI resistance mutations (V108I one, and 103N the other). Reasons for switching were convenience (52%), simplification (39.8%) and others (7.9%). Prior regimens: DTG/3TC (30.6%), TAF/FTC/BIC (26.6%), DTG/RPV (12.0%), TAF/FTC/RPV (9.6%), TAF/FTC/DRVc (3.2%) and others (17.7%). Median follow‐up time after first injection was 7.8 months with median of 7 injections. There were 264 (93.9%) CAB+RPV initiators who remained on the regimen at end of follow‐up; eight (2.8%) discontinued due to transfer to another city, four (1.4%) due to patient's preference, three (1.0%) due to injection pain, one gestational desire and one lost to follow‐up. Among all initiators, 120 (42.7%) had recorded VL at 24 weeks and 40 (14.2%) at 48 weeks, being <200 copies/ml in 97.5%. Among all individuals, 246 (87.5%) had at least one VL after switching, with all follow‐up VL <200 copies/ml.

**Conclusions**: CAB+RPV was effective and safe in long‐term and highly ART‐experienced HIV‐infected patients under any prior treatment, suggesting high effectiveness of this regimen in real‐world settings. Convenience and simplification were the main reasons for changing, and DTG‐based dual therapies were the most common prior regimens. Few patients discontinued CAB+RPV, exceptionally due to adverse event.

### P085 HIV‐2 chronic infection and BIC/FTC/TAF experience in a Portuguese tertiary hospital in Lisbon

Margarida Almeida, Constança Antunes, Mariana Pereira, Marta Almeida, Ana Cláudia Miranda, Kamal Mansinho

Doenças Infeciosas, Hospital de Egas Moniz, Unidade Local de Saúde de Lisboa Ocidental, Lisboa, Portugal

**Background**: HIV‐2 infection is endemic in West Africa, and Portugal has the highest prevalence within Europe. The first line of recommended treatments includes a combination of two NRTIs plus an INSTI or a boosted PI—accumulated evidence on second‐generation INSTI results from in vitro or cohort studies [1,2].

**Materials and methods**: Real‐life, retrospective, observational cohort study. Analysis of a cohort of adult patients with chronic HIV‐2 infection, under medical follow‐up at a tertiary hospital in Lisbon between 1985 and 2023, who completed at least 6 months of ART with BIC/FTC/TAF. Demographic, epidemiological, clinical, laboratory and therapeutic evaluation.

**Results**: Nineteen HIV‐2 patients on BIC/TAF/FTC were included: four (21%) treatment naïve and 15 (79%) treatment‐experienced. The naïve patients: 75% females; mean age of 53.0 years; 75% assumed as heterosexual acquisition; all HIV stage A (CDC Atlanta); mean baseline TCD4+ 521/mm^3^. The ART initiation was due to HIV‐2 RNA detection at baseline (three), mean 7833 copies/ml and one diagnosed 23 years earlier due to chemotherapy. The mean treatment time is 16 months, with a mean increase of 219 cells/mm^3^ from the baseline and a current TCD4+ 620/mm^3^. All patients are virologically suppressed, without ART intolerance. The treatment‐experienced patients: 63% females; mean age of 63.0 years; 80% assumed as heterosexual acquisition; 67% HIV stage A and 13% stage C (CDC Atlanta); mean baseline TCD4+ 402 cells/mm^3^ and 20% with undetectable HIV‐2 RNA; mean of 19.4 years since diagnosis and 14.1 years on ART. Until the initiation of BIC/FTC/TAF, patients showed a mean increase of 35 cells/mm^3^/year, and 80% had undetectable HIV‐2 RNA, comparing to 13% when ART started. The reason for switching to BIC/FTC/TAF: 80% for simplification, 13% to prevent drug interactions and 7% due to ART resistance. Currently, the median treatment time with BIC/FTC/TAF is 20 months, no intolerances recorded, with a mean TCD4+ of 637 cells/mm^3^ and an increase of 20 cells/mm^3^/year. All have undetectable HIV‐2 RNA.

**Conclusions**: Our results support the use of BIC/FTC/TAF as a viable option in HIV‐2 infection. The majority of the patients had long‐term HIV‐2 infection and were treatment‐experienced, yet the regimen demonstrated durability and efficacy.

**References**

1. Kapoor AK, Padival S. HIV‐2 infection. In: StatPearls [Internet]. 2022 [cited 2024 Aug 14]. Available from: https://www.ncbi.nlm.nih.gov/books/NBK572083/.

2. Berzow D, Descamps D, Obermeier M, Charpentier C, Kaiser R, Guertler L, et al. Human immunodeficiency virus‐2 (HIV‐2): a summary of the present standard of care and treatment options for individuals living with HIV‐2 in Western Europe. Clin Infect Dis. 2021;72(3):503‐9.

### P086 Discontinuation of rilpivirine and cabotegravir in HIV‐1 virologically suppressed adults: a multicentre observational study in Tuscany (LAHIV study)

Filippo Lagi ^1^, Giuseppe Formica^2^, Massimiliano Fabbiani^3^, Barbara Rossetti^4^, Matteo Piccica^5^, Susanna Giachè^6^, Daniela Messeri^7^, Silvia Costarelli^8^, Erika Riguccini^9^, Giovanni Sarteschi^10^, Michele De Gennaro^11^, Emanuela Francalanci^2^, Giuseppe Gasparro^2^, Marco Fognani^2^, Riccardo Paggi^2^, Paola Corsi^1^, Marco Pozzi^1^, Gaetana Sterrantino^2^, Mario Tumbarello^3^, Pierluigi Blanc^7^, Filippo Bartalesi^5^, Donatella Aquilini^6^, Cesira Nencioni^4^, Danilo Tacconi^9^, Spartaco Sani^8^, Antonella Vincenti^10^, Alessandro Bartoloni^1^

^1^Infectious and Tropical Diseases Unit, Careggi University Hospital, Florence, Italy, ^2^Department of Experimental and Clinical Medicine, University of Florence, Florence, Italy, ^3^Infectious and Tropical Diseases Unit, Santa Maria alle Scotte University Hospital, Siena, Italy, ^4^Infectious Diseases Unit, Misericordia Hospital, Azienda Unità Sanitaria Locale (AUSL) Toscana Sud Est, Grosseto, Italy, ^5^Infectious Diseases Unit, Santa Maria Annunziata Hospital, Azienda Unità Sanitaria Locale (AUSL) Toscana Centro, Bagno a Ripoli, Italy, ^6^Infectious Diseases Unit, Santo Stefano Hospital, Azienda Unità Sanitaria Locale (AUSL) Toscana Centro, Prato, Italy, ^7^Infectious Diseases Unit, San Jacopo Hospital, Azienda Unità Sanitaria Locale (AUSL) Toscana Centro, Pistoia, Italy, ^8^Infectious Diseases Unit, Livorno Hospital, Azienda Unità Sanitaria Locale (AUSL) Toscana Nord‐Ovest, Livorno, Italy, ^9^Infectious Diseases Unit, San Donato Hospital, Azienda Unità Sanitaria Locale (AUSL) Toscana Sud Est, Arezzo, Italy, ^10^Infectious Diseases Unit, Nuovo Ospedale Apuane, Azienda Unità Sanitaria Locale (AUSL) Toscana Nord Ovest, Massa, Italy, ^11^Infectious Diseases Unit, San Luca Hospital, Azienda Unità Sanitaria Locale (AUSL) Toscana Nord Ovest, Lucca, Italy

**Background**: Cabotegravir (CAB) + rilpivirine (RPV), available in Italy from June 2022, dosed intramuscularly every 2 months, is the first long‐acting (LA) regimen used to maintain HIV‐1 virological suppression. We evaluated the durability of this regimen.

**Materials and methods**: This multicentre observational study enrolled virologically suppressed adults living with HIV (HIV‐RNA <50 cp/ml) from 10 Tuscan units who initiated CAB+RPV. Participants were monitored from their first injection until regimen discontinuation, death or last visit. Discontinuation criteria included regimen switch or two consecutive missed injections. Virological failure (VF) was defined as two consecutive HIV‐RNA >50 copies/ml detections or >1000 copies/ml followed by ART modification. Statistical analyses included chi‐square, non‐parametric tests for categorical and continuous variables, and Kaplan‐Meier survival analysis for discontinuation probability estimation.

**Results**: The cohort included 129 PLWH and a combined at‐risk period of 75.5 years, with a median follow‐up of 28 weeks (interquartile range [IQR] 12–48). Most participants (81.4%) were male, with a median age of 51 years (IQR 42–57), and a median ART duration of 13 years (IQR 8–20). Participants discontinuing LA showed no clinical/demographic differences from those continuing, except a shorter time from the last detectable HIV‐RNA and CAB+RPV introduction (0.6 [IQR 0.2–4] vs. 5 years [IQR 1–9], *p* = 0.012). Ten participants discontinued CAB+RPV: six (4.6%) due to adverse events, one by patient choice, one lost to follow‐up and two (1.5%) due to VF (Table 1). Notably, one participant experiencing VF had pre‐existing high‐level mutations for RPV, but no mutations for CAB, and viral suppression was restored with T/F/DRVc (Table 1). The overall discontinuation rate was 13.2 per 100 person‐years (95% CI 7.1–24.5), higher than reported in randomized controlled trials (RCTs), possibly due to our shorter follow‐up (Figure 1).

**P086: Table 1:** Reasons for discontinuation of a population of adults with HIV‐1, ART experienced with HIV‐RNA level <50 copies/ml switching to RPV+CAB in 10 out 11 infectious diseases units in Tuscany, Italy

Previous regimen	Viraemia (>50 cp/ml) less than 6 months ago	Subtype	BMI at BL	OLI	NNRTI or INSTI mutations on the last genotype	Cause	Week	Post‐regimen
3TC/DTG	No	N/A	N/A	No	N/A	High fever with widespread joint pain and gluteal pain. Walking impairment	4	3TC/DTG
RPV/DTG	Yes	B	N/A	No	Not detected	Rash	28	RPV/DTG
3TC/DTG	Yes	B	29.5	No	98G 106I 108I 181V	Virological failure (4930 cp/ml)	4	T/F/DRVc
RPV/DTG	Yes	N/A	20.2	No	N/A	General malaise	4	RPV/DTG
3TC/DTG	No	N/A	25.3	No	N/A	Depression	4	3TC/DTG
RPV/DTG	Yes	N/A	22	No	N/A	Lost‐to follow‐up	12	//
T/F/RPV	Yes	N/A	23	No	Not detected	Panic attacks	12	T/F/RPV
T/F/RPV	No	N/A	23.4	No	N/A	Excessive clinic visits, local reaction	28	T/F/RPV
T/F/DOR	No	N/A	26.6	No	Not detected	Virological failure (200 cp/ml)	28	3TC/DTG
3TC/DTG	No	N/A	21	No	N/A	Weight gain of 10 kg, worsening of depressive symptoms, insomnia, asthenia	44	3TC/DTG

Abbreviations: 3TC, lamivudine; BL, baseline; BMI, body mass index; DTG, dolutegravir; F, emtricitabine; INSTI, integrase strand transfer inhibitor; N/A, not available; NNRTI, non‐nucleoside reverse transcriptase inhibitors; OLI, oral lead‐in; RPV, rilpivirine; T, TAF, tenofovir alafenamide.

**P086: Figure 1:**
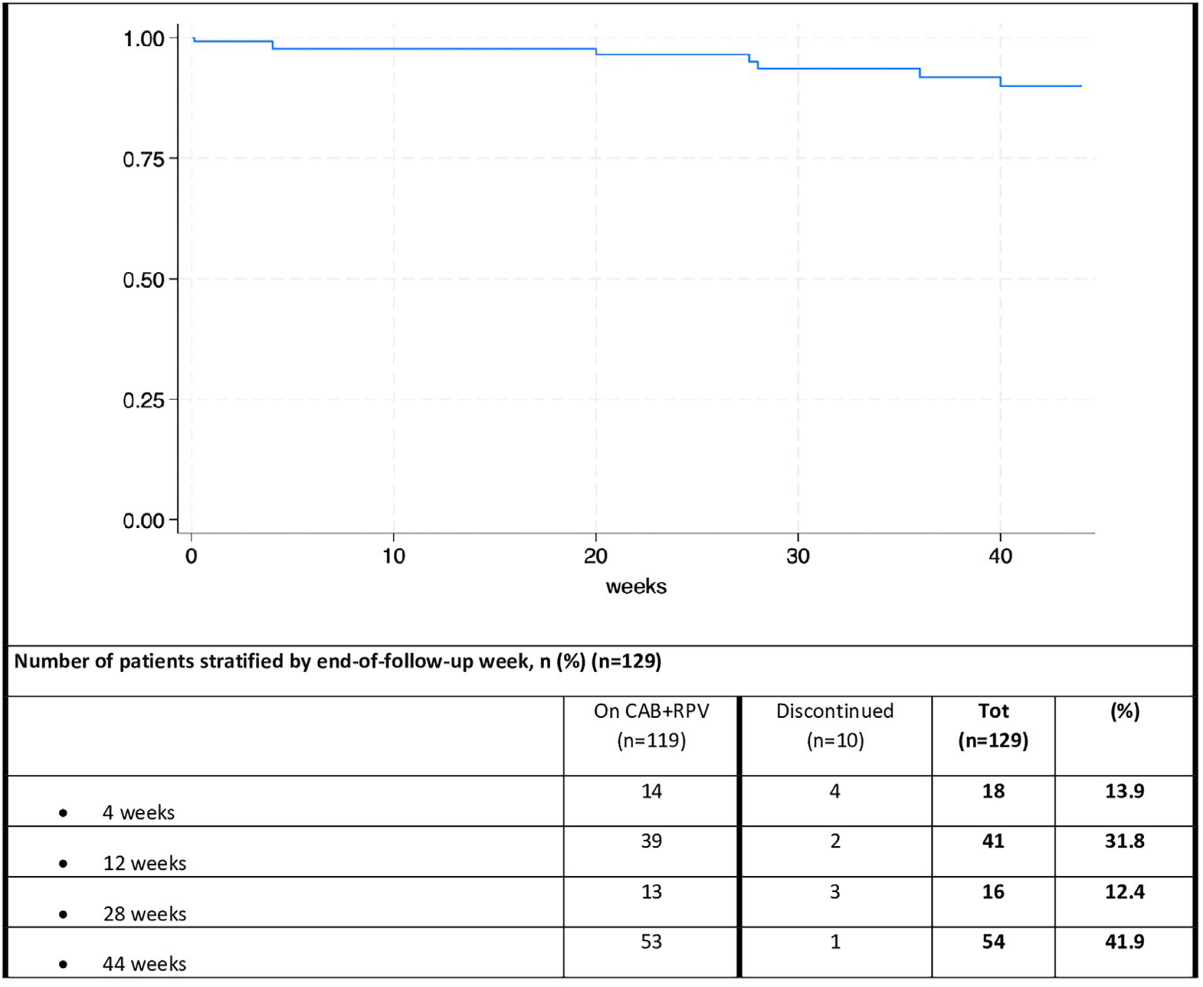
Probability of remaining free from treatment discontinuation for all causes in adults with HIV‐1 ART experienced with HIV‐RNA level <50 copies/ml switching to RPV+CAB in 10 out of 11 infectious diseases units in Tuscany, Italy.

**Conclusions**: Early findings indicate that CAB+RPV appears to be a well‐tolerated regimen. However, the overall discontinuation of CAB+RPV in real life seems to be slightly higher than those reported in trials. Discontinuation due to virological failure remains a rare event.

### P087 Effectiveness and safety of BIC/FTC/TAF for late‐presenting people with HIV‐1 infection

Hai Long, Xiaoxin Xie, Xiaoyan Yang, Yanhua Fu, Lin Gan, Shujing Ma

Infectious Disease Department, Guiyang Public Health Clinical Center, Guiyang, China

**Background**: The efficacy and safety of bictegravir/emtricitabine/tenofovir alafenamide (BIC/FTC/TAF) has been demonstrated in clinical trials of treatment‐naïve therapy. However, real‐world evidence for this regimen in late‐presenting people with HIV‐1 (PWH) is lacking. We investigated the virological and safety outcomes of BIC/FTC/TAF for late‐presenting PWH.

**Methods**: We conducted a retrospective cohort analysis of PWH who were late‐presenting and initiated the antiretroviral regimen of BIC/FTC/TAF between June 2021 and February 2023. Treatment effectiveness, defined as HIV‐1 RNA <50 copies/ml, was analysed. Changes in immunological, metabolic, liver and renal parameters after 48 weeks of treatment were evaluated. Late‐presenting PWH was defined as surviving PWH with CD4^+^ T‐cell count <200 cells/µl or surviving AIDS patients with CD4^+^ T‐cell count ranging from 200 to 499 cells/µl.

**Results**: A total of 102 participants were included. Predominantly men (75.7%), with an age was 46.3±14.8 years. At baseline, the median log10 HIV‐1 RNA is 4.98 (4.61–5.78) which contained 27.5% (28/102) cases of HIV‐1 RNA ≥500,000 copies/ml. The median CD4^+^ T‐cell count was 82.5 (28.0–141.3) cells/µl, and 63.7% of cases were complicated by opportunistic infections. At week 48, 94.1% (96/102) achieved HIV‐1 RNA <50 copies/ml. CD4^+^ T‐cell count increased by 133 cells/µl, and CD4/CD8 increased by 0.17 (*p* < 0.001). Thirteen (12.7%) participants experienced adverse events (AEs), with five (4.9%) drug‐related AEs. No participants discontinued treatment due to lack of efficacy or AEs.

**Conclusions**: BIC/FTC/TAF demonstrated robust virological suppression and tolerability in late‐presenting PWH.

### P088 Virological failure rate and emergent resistance in real‐world studies evaluating long‐acting cabotegravir and rilpivirine in people with baseline viral suppression

Melanie Smuk, Alexa Elias, Kyle Ring, Chloe Orkin

SHARE Collaborative, Queen Mary University of London, Blizard Institute, London, UK

**Background**: The virological failure rate in RCTs (SOLAR, ATLAS‐2M, CARES) evaluating 2‐monthly injectable cabotegravir and rilpivirine (CAB+RPV) for treatment of HIV‐1 is <2%. No synthesis of virological failure (VF) outcomes in real‐world evidence (RWE) has been presented/published.

**Materials and methods**: We performed evidence synthesis on a systematic literature review conducted from January 2020 to March 2024 on RWE use of CAB+RPV for treatment. We included all studies reporting information on VF events in individuals who were virally suppressed at baseline. Total frequency of VF was ascertained across all studies. Studies used nine different definitions of VF. In studies which reported on emergent resistance, the frequency and percentage of studies with and without emergence of resistance was calculated. The number and proportion of individuals with emergent resistance to non‐nucleoside reverse transcriptase inhibitors (NNRTIs) and integrase inhibitors (INIs) was summarized by class (Table 1).

**P088: Table 1:** Emergent resistance data available on people who experienced VF in RWE^a^

Participants with VF	Emergent resistance? (No/NNRTI/INI)	Mutations
1	NNRTI + INI	(Y181C) + (L74I, T97A, E138K, Q148R and N155H)
2	NNRTI	K103N, L100I
3	No	NA
4	No	NA
5	No	NA
6	NNRTI + INI	(L100I, K103NS) + (E138KA, G140SAC, Q148HRK)
7	NNRTI	M230L, V179E
8	NNRTI	E138A, V179I
9	No	NA
10	No	NA
11	No	NA
12	NNRTI + INI	(101E+138K+230L) + (155H)
13	NNRTI + INI	(101E+138K) + (138K+148R)
14	NNRTI	E138A
15	NNRTI + INI	(L74L/M, T97T/A) + (G140S, Q148H, K101P, E138K, I178L, Q207E)
16	INI	L74I, T97T/A, S147S/G, N155H
17	INI	G140G/S, Q148Q/R
18	NNRTI + INI	(K101E, N348I) + (E138A, G140S, Q148S)
19	NNRTI + INI	(Y188L) + (Q148R)
20	NNRTI + INI	(K101E, I178M) + (N155H, R263K)
21	No	NA
22	NNRTI	K103N, E138Q
23	NNRTI	K103K/R, E138G/R
24	NNRTI + INI	(M230M/L) + (E138E/K, Q148Q/K)
25	No	NA
26	No	NA
27	No	NA

^a^We included studies where relationship between drug class and emergent mutations was specified.

**Results**: We identified 27 studies with available data on VF, including 5048 individuals who were virally suppressed at switch. The median number of people per study was 76.0 (IQR 28.0–164.0, range 1–1293), with a total of 73 VF events recorded across all studies. The duration of follow‐up varied greatly (range 1–24 months) and thus estimating a pooled VF rate across studies would be uninterpretable due to the heterogeneity between studies. Fifteen studies provided information about emergent resistance. Eleven (73.3%) of these studies reported emergent resistance, while four (26.7%) reported no emergent resistance. Resistance emerged in 18 individuals, 15/18 had NNRTI resistance (83.3%) and 11/18 had INSTI mutations (61%). Eight of 18 had both NNRTI and INSTI resistance.

**Conclusions**: Calculating an accurate VF rate using RWE is difficult due to heterogeneity of VF definition and study design. Resistance emerged commonly where VF occurred.

### P089 Profile of people with HIV (PWH) switching prior antiretroviral treatment (ART) to a doravirine (DOR)‐based regimen in the real‐world clinical setting in Greece: the DORAVITO study

Antonios Papadopoulos^1^, George Adamis^2^, Panagiota Lourida^3^, Vassileios Paparizos^4^, Helen Sambatakou^5^, Symeon Metallidis^6^, Charalampos Moschopoulos^1^, Myrto Astriti^2^, Charisis Totsikas^3^, Varvara Vasalou^4^, Eleni Boutselakou^7^, Dimitris Tsokos ^7^, Georgios Trimis^7^, Lazaros Poughias^7^

^1^4th Department of Internal Medicine, University Hospital Attikon, Athens, Greece, ^2^1st Department of Internal Medicine and Infectious Diseases, G. Gennimatas General Hospital of Athens, Athens, Greece, ^3^5th Department of Internal Medicine—Division of Infectious Diseases, Evangelismos General Hospital of Athens, Athens, Greece, ^4^AIDS Unit, Clinic of Venereologic and Dermatologic Diseases, Athens Medical School, Syngros Hospital, Athens, Greece, ^5^HIV Unit, Second Department of Internal Medicine, Athens Medical School, Hippokration University General Hospital, Athens, Greece, ^6^Aristotle University HIV Unit, AHEPA University Hospital, Thessaloniki, Greece, ^7^Global Medical & Scientific Affairs, Merck Sharp & Dohme, Athens, Greece

**Background**: DOR is a new‐generation non‐nucleoside reverse transcriptase inhibitor, which has emerged as an option for PWH owing to its favourable safety/efficacy profile, unique resistance pathway and limited drug‐drug interactions. This study aimed at better understanding DOR‐based treatment use in Greece, focusing on characteristics of PWH, and drivers of treatment switch.

**Materials and methods**: DORAVITO was a cross‐sectional retrospective chart review study, with a planned consecutive enrolment of 100 PWH. Eligible individuals were adults with HIV‐1 infection who were switched to a DOR‐based regimen based on physician's decision; the switch should have occurred in the post‐marketing setting and ≥30 days before site initiation. Individuals exposed to DOR at any time in the past before switching to the DOR‐based regimen were excluded. The observation period extended from initial HIV‐1 diagnosis to the date of first DOR prescription.

**Results**: From 12 Jul 2023 to 31 Oct 2023, 110 PWH were enrolled across six public hospital clinics. At baseline (closest prior to/on date of first DOR prescription), mean age of PWH was 49.3 years, most were males (90.9%), 88.2% were asymptomatic and 86.5% were virologically suppressed (Table 1). The median Charlson Comorbidity Index score was 1.0 (interquartile range 0.0–2.0), with 33.6% of PWH suffering from multimorbidity (excluding infections/infestations). Most common comorbidities were dyslipidaemia (30.9%) and hypertension (12.7%), while 45.5% of PWH were receiving comedications for the treatment of comorbidities (most common: lipid‐lowering drugs [24.5%], antihypertensives/heart failure agents [10.9%], antidepressants/anxiolytics and antithrombotics [7.3%, each]). Hepatitis C virus co‐infection was reported in 5.5% of PWH. Most PWH (87.3%) were prescribed DOR/lamivudine/tenofovir disoproxil fumarate fixed‐dose combination. PWH started DOR a median of 11.7 years after first‐ever ART initiation, corresponding to 2nd/3rd/≥4th ART line in 33.6%/33.6%/32.7%; 60.9% of them were proactively switched to a DOR‐based regimen (Figure 1a). Most common reasons for switching were “regimen simplification” (42.7%), “tolerability” (26.4%) and “prevention of toxicities” (18.2%) (Figure 1b).

**P089: Table 1:** Key demographic, disease and treatment characteristics

		Overall (*N* = 110)	DOR/3TC/TDF fixed dose combination (*N* = 96)	DOR single agent combined with other ARV(s) (*N* = 14)	Switch to DOR in the second line (*N* = 37)	Switch to DOR in the >second line (*N* = 71)^a^
**Demographic characteristics at baseline**						
Age, mean (SD), years		49.3 (10.8)	48.6 (10.5)	54.6 (11.5)	43.1 (11.2)	52.0 (8.2)
Male sex assigned at birth, %		90.9	91.7	85.7	86.5	93.0
Greek ethnic origin, %		75.5	75.0	78.6	59.5	83.1
**Clinical, virological and immunological characteristics at baseline**						
HIV clinical stage, %	Asymptomatic	88.2	87.5	92.9	91.9	85.9
HIV clinical stage, %	Symptomatic (without AIDS‐defining conditions)	8.2	8.3	7.1	5.4	9.9
HIV clinical stage, %	AIDS	3.6	4.2	−	2.7	4.2
Available virological suppression status (HIV‐load <50 copies/ml)^b^, *n*		*n* = 89	*n* = 75	*n* = 14	*n* = 33	*n* = 54
Virologically suppressed among evaluable pts, %		86.5	86.7	85.7	84.8	90.7
Available immunological testing in the last 6 months, *n*		*n* = 95	*n* = 82	*n* = 14	*n* = 35	*n* = 59
CD4^+^ cell absolute count (cells/mm^3^), mean (SD)		697.2 (304.6)	689.1 (307.4)	744.2 (294.2)	628.6 (275.7)	738.7 (317.5)
**Prior treatment history**						
Time from start of first ART to DOR initiation, median (IQR), years		11.7 (6.6−14.3)	10.4 (5.9−13.9)	14.1 (11.7−20.3)	4.2 (1.8−12.2)	12.8 (8.9−15.8)
Number of prior ART regimens, median (IQR)		2.0 (1.0−3.0)	2.0 (1.0−3.0)	3.0 (2.0−5.0)	1.0 (1.0−1.0)	2.0 (2.0−4.0)
≥2 prior ART regimens, %		66.4	64.6	78.6	−	100.0
**Clinically significant comorbidities at baseline**						
HCV, %		5.5	5.2	7.1	8.1	4.2
HBV, %		3.6	3.1	7.1	−	5.6
Presence of multimorbidity^c^ (excluding infections/infestations), %		33.6	30.2	57.1	16.2	40.8
CCI score ≥3 (moderate to high comorbidity burden), %		15.5	14.6	21.4	8.1	18.3
**Characteristics of the prescribed DOR‐based regimen**						
DOR prescribed in combination with other ARVs, %		100.0	100.0^d^	100.0	100.0	100.0
Dual NRTI only		94.5	96.9^d^	78.6	100.0	94.4
Dual NRTI + INSTI or entry inhibitor		3.6	3.1^d^	7.1	−	5.6
PI + INSTI + boosting agent		0.9	−	7.1	−	−
INSTI only		0.9	−	7.1	−	−

Abbreviations: 3TC, lamivudine; ART, antiretroviral therapy; ARV, antiretroviral; CCI, Charlson Comorbidity Index; DOR, doravirine; HBV, hepatitis B; HCV, hepatitis C; INSTI, integrase strand transfer inhibitor; IQR, interquartile range; NRTI, nucleoside reverse transcriptase inhibitor; PI, protease inhibitor; SD, standard deviation; TDF, tenofovir disoproxil fumarate.

^a^Excluding two patients who were switched to DOR combined with ARVs other than NRTIs, who received this regimen in the ≥4th line setting.

^b^For at least 6 months prior to first DOR prescription.

^c^≥2 clinically significant medical conditions.

^d^Dual NRTI is part of the multi‐tablet regimen.

**P089: Figure 1:**
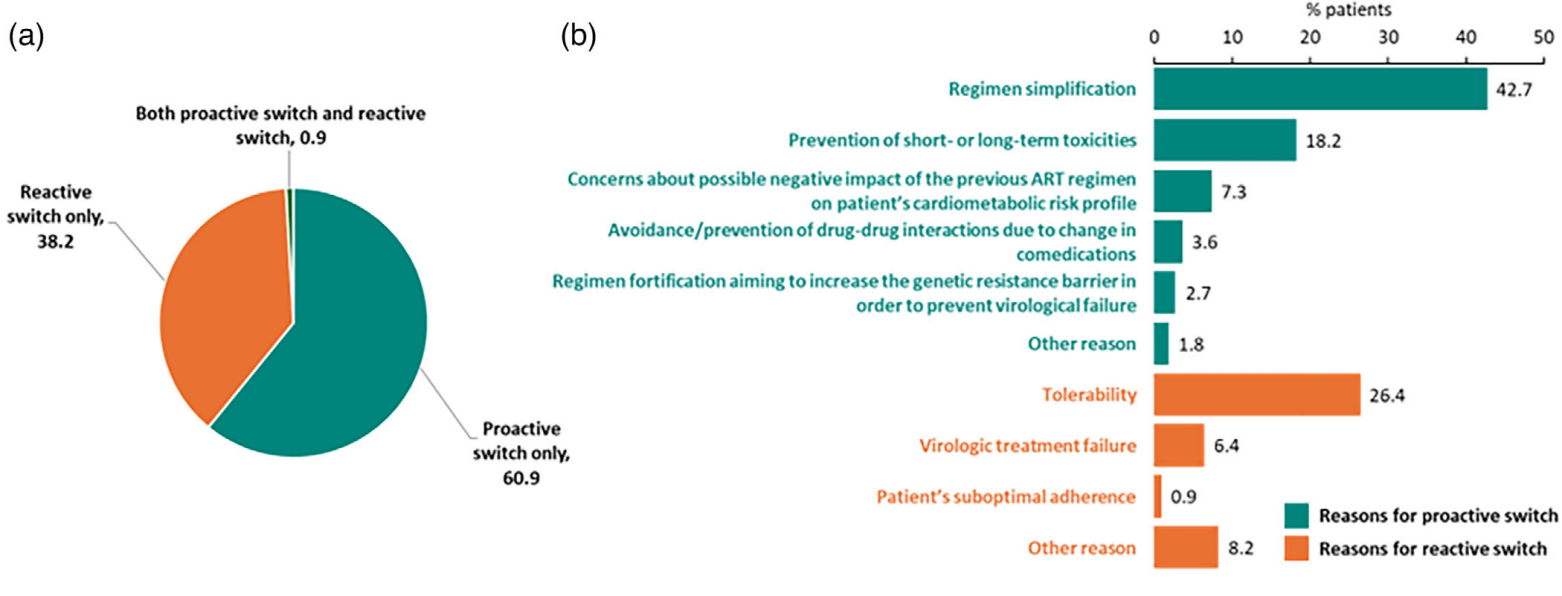
(a) Types of switch and (b) reasons for switch from a prior antiretroviral therapy to a doravirine‐based regimen, in the overall study population (*N* = 110).

**Conclusions**: Real‐world data from the DORAVITO study provide insight into the current prescription landscape of DOR in Greece, revealing that switching to a DOR‐based regimen is a valuable option for PWH who require ART simplification, increased tolerability and avoidance of drug toxicities.

### P090 PLWH treated with cabotegravir‐rilpivirine have increased CD4/CD8 ratio and decreased CD8 T cells, as expression of lower inflammation, while maintaining virological suppression: a real‐life 48 weeks analysis

Giuseppe Nunnari ^1^, Serena Spampinato^1^, Giuseppe Nicolo Conti^2^, Carmen Giarratana^2^, Teresa Cirelli^1^, Viviana Coco^1^, Michele Salvatore Paterno Raddusa^3^, Viviana Fisicaro^3^, Chiara Gullotta^3^, Roberto Bruno^1^, Eugenia Pistarà^1^, Nunziatina Villari^1^, Bruno Cacopardo^1^, Emmanuele Venanzi Rullo^3^, Andrea Marino^1^, Benedetto Maurizio Celesia^1^, Giovanni Pellicanò^3^, Alessia Mirabile^1^

^1^Clinical and Experimental Medicine, University of Catania, Catania, Italy, ^2^Clinical and Experimental Medicine, University of Catania, University of Messina, Catania, Messina, Italy, ^3^Clinical and Experimental Medicine, University of Messina, Messina, Italy

**Background**: Switching from oral antiretroviral therapy (ART) to intramuscular administration of cabotegravir‐rilpivirine (CAB+RPV) eliminates the challenges related to patient adherence and compliance with daily oral regimens. Clinical trials have reported not only the virological and immunological effectiveness, but also assessed safety and patient satisfaction. The present study evaluates, real life, the safety and effectiveness of CAB+RPV.

**Methods**: Our retrospective‐observational study included 51 out of 85 PLWH who were on CAB+RPV that reached 48 weeks of treatment, followed at the Infectious Diseases Unit of ARNAS Garibaldi Hospital, University of Catania, Italy. These individuals were virologically suppressed and had been adherent to ART for at least 6 months. Importantly, they did not have documented or suspected resistance mutations to CAB/RPV. Data were collected at baseline and 48 weeks after the initiation of CAB+RPV, with or without the lead‐in phase with oral CAB+RPV. We measured CD4^+^, CD8^+^, CD4^+^/CD8^+^ ratio, HIV‐RNA plasma viral load and clinical chemistry parameters. Friedman multiple comparison test was used for non‐normally distributed variables, one‐way ANOVA for normally distributed variables. The study was approved by the Provincial Ethics Committee of Messina (SHICohort‐protocol approved 22/05/2017).

**Results**: We enrolled 51 PLWH, predominantly males (79%), with a median age of 44 years (IQR 38–55). Of these participants, 66% had transitioned from an integrase strand transfer inhibitor (INSTI)‐based regimen, 31% from a TAF/FTC‐based regimen. Two patients out of 85 returned to their previous oral regimen due to the emergence of anxiety disorders, but virologically suppressed. All 51 patients remained virally suppressed at 48 weeks, except one who had a viral load of 55. Of importance, a statistically significant augmentation of CD4/CD8 ratio has been observed, along with a reduction of CD8 T cells and creatinine levels (Figure 1). Furthermore, HDL levels significantly increased (Figure 1). Pain, as reported in clinical trials, emerged as the primary side effect (Table 1).

**P090: Figure 1:**
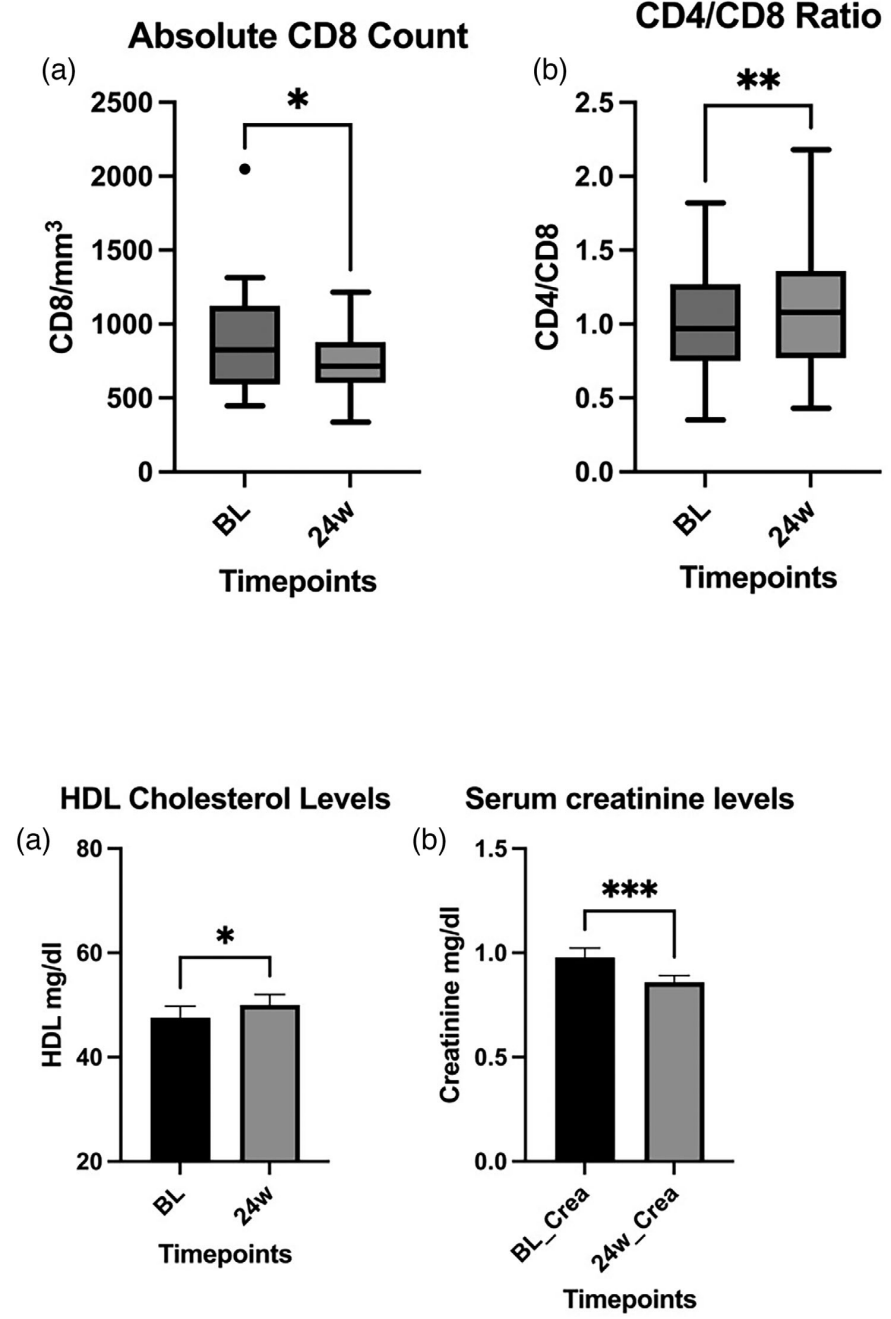
(Top) Comparison of (a) absolute CD8 count and (b) CD4/CD8 ratio between baseline and 24‐weeks time point and (bottom) comparison of (a) HDL levels and (b) serum creatinine levels between baseline and 24‐weeks time point.

**P090: Table 1:** Resume of parameters affected by statistically significant variations

Parameter	Value at baseline	Value at 24 weeks	*p*‐value
Serum creatinine (mg/dl, mean±SD)	0.98 (0.22)	0.86 (0.16)	0.0001
HDL cholesterol (mg/dl, median±IQR)	47 (13.5)	48 (13)	0.049
CD4/mm^3^ (mean±SD)	845 (287.6)	813 (255.5)	0.3395
CD8/mm^3^ (mean±SD)	897 (361)	752 (217)	0.0118
CD4/CD8 ratio3 (mean±SD)	1 (0.38)	1.12 (0.42)	0.0038
Virological suppression	31	29	−

*Note*: Mean±SD was used to describe normally distributed variables, while median±IQR was used to describe variables that did not follow a normal distribution.

**Conclusions**: In our real‐life experience, CAB+RPV not only maintained virological suppression, but also induced the reduction of CD8 T cells and an increase in CD4/CD8 ratio, as the expression of reduced inflammation. Our real‐life experience confirmed the virological, immunological effectiveness and safety of CAB+RPV.

### P091 Discontinuation rates of doravirine/lamivudine/tenofovir‐DF due to neuropsychiatric adverse effects

Iresh Jayaweera, Rory Grier‐Gavin, Rosy Weston, Nicola Mackie, Lucy Garvey, John Walsh, Alan Winston, Borja Mora‐Peris

Department of HIV and Sexual Health, Imperial College NHS Trust, London, UK

**Background**: Neuropsychiatric adverse effects (NPAEs) leading to discontinuation of doravirine in randomized clinical trials are rare with slightly increased rates reported in a recent cohort study [1]. Due to high costs, NHS England antiretroviral commissioning policies [2] encouraged to consider switching away from rilpivirine/emtricitabine/tenofovir‐DF (RPV/F/TDF) in people living with HIV offering generic formulation switches. When individuals declined switch to separate generic components, an in‐class alternative single tablet regimen option of doravirine/lamivudine/tenofovir‐DF (DOR/L/TDF) was offered after local multi‐disciplinary team (MDT) approval due to perceived advantages including lack of DDIs and food requirement. We assessed the frequency and reasons for discontinuation after such a switch.

**Materials and methods**: We reviewed all RPV/F/TDF switches to DOR/L/TDF at a single UK centre from February 2022 to April 2023 with follow‐up until July 2024. The percentage of NPAE experienced and numbers switching back were assessed. Descriptive statistics were calculated for individual characteristics, follow‐up length and time of switch.

**Results**: Of 140 switches from RPV/F/TDF, 78 switched to DOR/L/TDF. The majority were male, median age was 49 years and the median follow‐up was 1.8 years. Sixteen of 78 (20.51%) individuals experienced at least one NPAE, 10/78 (12.8%) switched back to RPV/F/TDF due to NPAE and 3/78 (3.84%) switched due to drug‐drug interactions. NPAEs included insomnia (9/78, 11%) and other sleep‐related complaints. Six of 78 (7.7%) experienced two or more NPAEs. The average time to switching off DOR/L/TDF was 91 days. No virological failures were observed. Clear documentation of patient involvement in the rationale and decision to switch treatment was present in all cases. See further characteristics in Table 1.

**P091: Table 1:** Patient characteristics and types of neuropsychiatric adverse effects (NPAEs) explained

	Total *N* = 78	Continued on DOR/L/TDF *N* = 65	Switched due to NPAE *N* = 10	Switched due to other causes *N* = 3
Gender male	65 (83%)	55 (85%)	8 (80%)	2 (67%)
Age (years), median (range)	49 (27−80)	50 (27−80)	50 (32−59)	50 (43−55)
Previous NPAE on EFV	4 (5.1%)	3 (4.61%)	0	1 (33.3%)
NPAE on DOR/L/TDF	16 (20.5%)	6 (9.2%)	10 (100%)	N/A
Insomnia	9 (11.5%)	2 (3%)	7 (70%)	N/A
Nightmares	5 (6.4%)	1 (1.5%)	4 (40%)	N/A
Headache	4 (5.1%)	2 (3%)	2 (20%)	N/A
Other sleep complaints	6 (7.69%)	0	6 (60%)	N/A
Dizziness	1 (1.2%)	0	1 (10%)	N/A
Altered sensorium	1 (1.2%)	0	1 (10%)	N/A

**Conclusions**: Rates of NPAE leading to discontinuation of DOR/L/TDF when switching from RPV/F/TDF in this small cohort are higher than described in large randomized studies and ongoing vigilance is justified.  Importantly, all patients were involved in their ART decision‐making and regular screening of adverse effects is required.

**References**

1. Lanting VR, Oosterhof P, Ait Moha D, van Heerde R, Kleene MJT, Stalenhoef JE, et al.; HIV‐team OLVG. Switching to doravirine in cART‐experienced patients: an effective and highly tolerated option with substantial cost savings. J Acquir Immune Defic Syndr. 2024;95(2):190‐6.

2. NHS England and NHS Improvement, HIV CRG Drugs Subgroup. National antiretroviral treatment prescribing toolkit [Internet]. 2022 [cited 2023 Jul 4]. Available from: https://www.bhiva.org/file/637cada4d2395/National‐antiretroviral‐treatment‐prescribing‐toolkit.pdf.

### P092 Switch to dolutegravir/lamivudine in virologically suppressed people living with HIV: a real‐world experience in a low‐middle income country

Julian Vega^1^, Micaela Sogga Alfano^2^, María Clara Villaverde^2^, Alejandra Mariaca^2^, Diego Sanchez Thomas^2^, José Ángel Enrique Barletta ^1^, María José Rolón^1^, Gustavo Lopardo^2^

^1^Infectious Diseases, Hospital General de Agudos, Buenos Aires, Argentina, ^2^Infectious Diseases, Centro de Estudios Infectología Dr. Stamboulian, Buenos Aires, Argentina

**Background**: On the path of antiretroviral therapy (ART) optimization which people living with HIV (PLHIV) receive, switch to dolutegravir/lamivudine (DTG/3TC) in those receiving ART with three drugs (3D) has shown to be safe and effective in multiple randomized clinical trials (RCTs). Instead, data from real‐world experience (RWE) studies in low‐middle income settings is limited. Our aim was to evaluate virological and metabolic outcomes in PLHIV who switched from 3D to DTG/3TC in a non‐controlled scenario.

**Materials and methods**: Retrospective cohort study in two HIV clinics in Buenos Aires, Argentina. We included PLHIV who switched from 3D to DTG/3TC between 2/2016 and 11/2023 with at least 3 months of follow‐up. Clinical data before and after switch was collected. Descriptive statistics and Wilcoxon signed‐rank test were utilized for data analysis. A *p*‐value of <0.05 was considered statistically significant.

**Results**: We included 268 participants; median age was 51 years (Q1–Q3 39–57) and 211 (78.7%) were male at birth. Only 17 (6.3%) had drug resistance test data available and none presented an M184V/I mutation. Cohort ART history is shown in Figure 1. Median time of follow up after switch was 19 months (Q1–Q3 10–26). Laboratory results are shown in Table 1. Statistically significant decreases in triglycerides, total cholesterol and estimated glomerular filtration rate (eGFR) and increases in CD4 cell count and creatinine (Cr) were observed. At the end of follow‐up, 263 (98.1%) people presented an undetectable viral load (<40 cop/ml), while the rest had low‐level viraemias <100 cop/ml. Only three (1.1%) participants were switched back to 3D, none due to virological failure or adverse effects but rather due to 3TC unavailability.

**P092: Figure 1:**
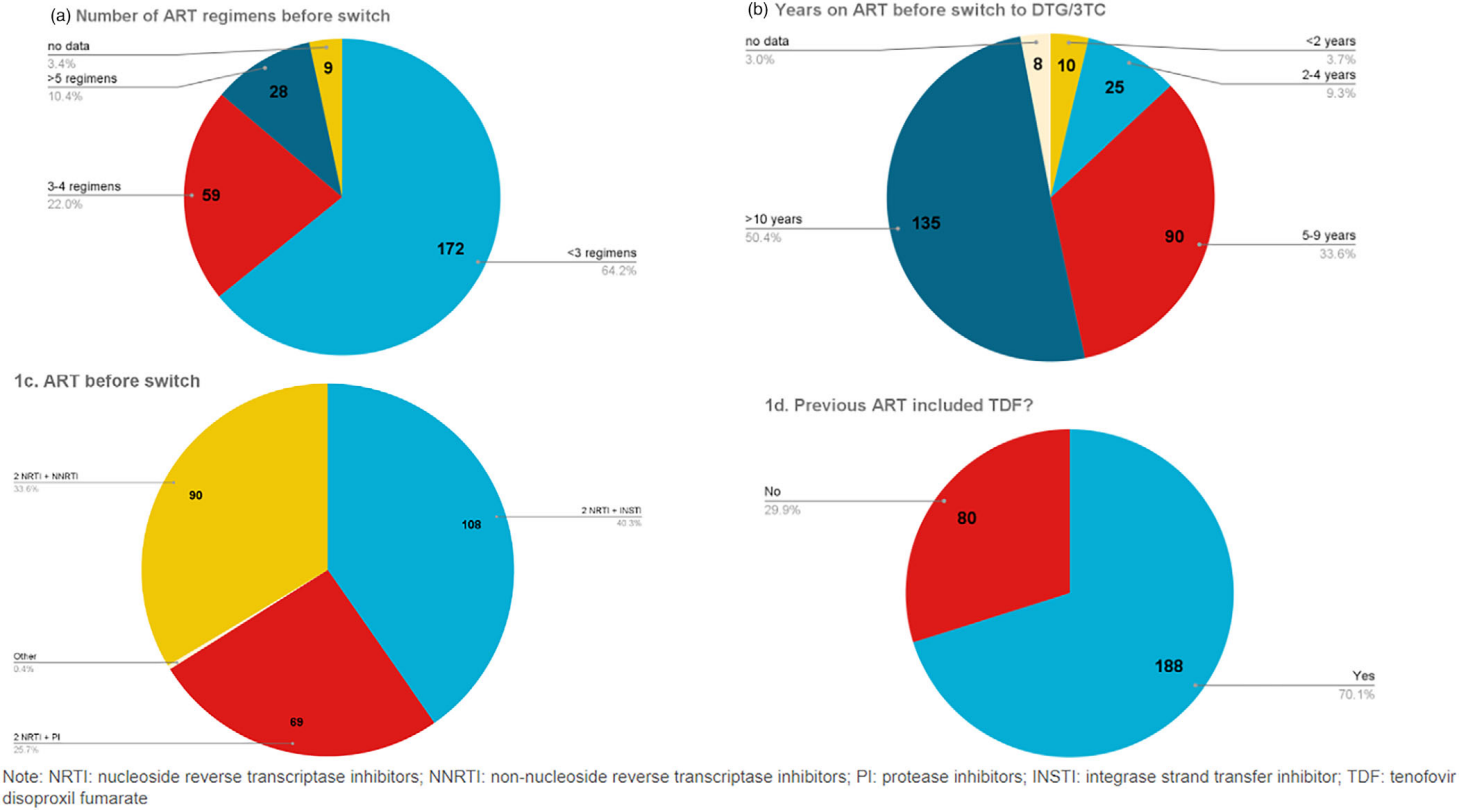
ART history before switch to DTG/3TC.

**P092: Table 1:** Laboratory results

	Median before switch (Q1−Q3) *n* = 268	Median after switch (Q1−Q3) *n* = 268	*p*‐value for Wilcoxon signed‐rank test
Absolute CD4 count (cells/µl)	725 (534−963)	787 (613−1002)	<0.05
Total cholesterol (mg/dl)^a^	193 (166−227)	187 (160−216)	<0.05
HDL cholesterol (mg/dl)^b^	45 (38−55)	45 (38−55)	0.5965
LDL cholesterol (mg/dl)^c^	119 (99−146)	118 (93−144)	0.3054
Triglycerides (mg/dl)^d^	130 (93−186)	113 (78−153)	<0.05
Glycaemia (mg/dl)^e^	95 (90−103)	96 (90−103)	0.3229
Creatinine (mg/dl)^f^	1 (0.8−1.2)	1.09 (0.88−1.22)	<0.05
Estimated glomerular filtration rate by CKD‐EPI^f^	92.78 (71.4−104.7)	82.7 (68.8−98.44)	<0.05

Abbreviation: CKD‐EPI, Chronic Kidney Disease Epidemiology Collaboration equation.

^a^Data available for 249 and 256 participants, respectively.

^b^Data available for 239 and 243 participants, respectively.

^c^Data available for 238 and 243 participants, respectively.

^d^Data available for 243 and 250 participants, respectively.

^e^Data available for 243 and 249 participants, respectively.

^f^Data available for 253 and 260 participants, respectively.

**Conclusions**: DTG/3TC was effective at maintaining virological suppression in our cohort, without a clinically significant impact in metabolic variables. Although an increase in Cr and decrease in the GFR values was observed, this finding may be linked to DTG activity on OCT 2 receptors and/or prescription bias in real‐world settings. More RWE studies are needed in order to broaden our understanding of these findings.

### P093 Effectiveness and safety of dual therapy with co‐packed DTG and 3TC compared to triple therapy in clinical practice in Argentina

Edgardo Bottaro^1^, Diego Cecchini ^1^, Brenda Bacelar^1^, Angeles Tisné^1^, Claudia Migazzi^1^, Macarena Roel^1^, Maximiliano Bergman^2^, Isabel Cassetti^1^

^1^Medical Area, Helios Salud, Buenos Aires, Argentina, ^2^Medical Affairs, Laboratorio Richmond, Buenos Aires, Argentina

**Background**: To improve access to antiretroviral therapy (ART), Argentina approved a generic presentation of non‐coformulated DTG + 3TC as a once‐daily 2‐in‐1 medication pack (co‐pack). There is no real‐world data on its effectiveness and safety. We describe persistence, safety and virological suppression rates (VSRs) at 6, 12 and 18 months of co‐packed DTG + 3TC versus DTG‐based triple therapy (with either XTC/TDF or 3TC/ABC) as switching strategies in clinical practice.

**Materials and methods**: Retrospective observational cohort study, period 10/2019–11/2023 in a reference HIV centre in Argentina. We included experienced people living with HIV (PLWH) with virological suppression (viral load <50 copies/ml) who switched to DTG regimens.

**Results**: Of 601 PLWH, 245 (40.8%) were switched to dual therapy (DT) and 356 (59.2%) to triple therapy (TT): 139 to 3TC/ABC and 198 to XTC/TDF. Baseline characteristics: 68.3% were male; those in TT were younger (median 47 vs. 50 years, *p* = 0.03) and had a higher prevalence of previous virological failure (7.5% vs. 0.8%, *p* < 0.01). Previous ARTs were based mainly on first‐generation NNRTIs (40.6%) and boosted PIs (36.3%). The main reasons for switching were different among groups, with a higher frequency of prevention of toxicity in DT (24.9% vs. 14%) and of ongoing toxicity in TT (35.5% vs. 27%). Persistence rates at 6, 12 and 18 months for DT were: 100%, 97.6% and 100%; for TT: 97.4%, 96.8% and 96.4%, respectively (XTC/TDF: 95.3%, 95.7%, 94.8%; 3TC/ABC: 100%, 100%, 98.5%). VSR for DT at 6, 12 and 18 months were: 98.1%, 99% and 93.5%; for TT: 96.7%, 98.3% and 94.5%, respectively (XTC/TDF: 95.2, 98.9, 94.4; 3TC/ABC: 98.1%, 98.7%, 96.1%). Rates of attributable adverse events at 6, 12 and 18 months were 0%, 0.8% and 0% for DT and 0.7%, 0.4% and 0% for TT, respectively (XTC/TDF: 1.2%, 0.7% and 0%; 3TC/ABC: 0% at all time points).

**Conclusions**: DTG + 3TC in a co‐pack presentation provided high levels of persistence and VSR in clinical practice with a low rate of adverse events. Its effectiveness and safety were comparable to those of the available DTG‐based triple regimens in Argentina.

### P094 Two‐year long‐term data on the efficacy and tolerability of dolutegravir‐based regimens from the prospective multi‐centre TESLA cohort study in ART‐naïve and pre‐treated people living with HIV in Russia

Anna Basova ^1^, Stella Minaeva^2^, Natalia Sizova^3^, Firaya Nagimova^4^, Elena Orlova‐Morozova^5^, Margarita Radzikhovskaya^6^, Valeriy Shevchenko^7^, Andrés Maldonado‐Doblado^8^, Bryn Jones^9^

^1^Medical Affairs, GlaxoSmithKline, Moscow, Russian Federation, ^2^Infectious Diseases, Nizhny Novgorod Regional Centre for AIDS and Infectious Diseases Prophylaxis and Control, Nizhny Novgorod, Russian Federation, ^3^Infectious Diseases, Centre for AIDS and Infectious Diseases Prophylaxis and Control, St Petersburg, Russian Federation, ^4^Infectious Diseases, Tatarstan Republican Centre for AIDS and Infectious Diseases Prophylaxis and Control, Kazan, Russian Federation, ^5^Infectious Diseases, Moscow Regional Centre for AIDS and Infectious Diseases Prophylaxis and Control, Moscow, Russian Federation, ^6^Infectious Diseases, Regional Centre for AIDS and Infectious Diseases Prophylaxis and Control, Chelyabinsk, Russian Federation, ^7^Infectious Diseases, Altai Regional Centre for AIDS and Infectious Diseases Prophylaxis and Control, Barnaul, Russian Federation, ^8^ViiV Healthcare, Medical Affairs, Wavre, Belgium, ^9^Medical Affairs, ViiV Healthcare, Brentford, UK

**Background**: TESLA is a prospective real‐world data study on dolutegravir‐based regimens (DBRs) long‐term safety and efficacy in both ART‐naïve and experienced people living with HIV (PLHIV) in Russia with a 3‐year follow‐up. Here, we present the 24‐month tolerability and efficacy outcomes for two‐drug regimen (2DR; dolutegravir [DTG] + lamivudine) and three‐drug regimen (3DR; DTG + 2 other drugs) sub‐populations.

**Materials and methods**: TESLA included 1000 adult PLHIV from 14 centres in Russia initiating DTG as part of ART for HIV‐1 infection treatment. Participants were evaluated at routine clinical care visits until DTG discontinuation, death, loss of follow‐up or the end of data collection. Reasons for DTG discontinuation were recorded, and virological response was assessed at 24 months; viral load and CD4 cell count change were estimated.

**Results**: Overall, 959 PLHIV were included in the analysis for 24 months (82% ART‐experienced, 58% male, median age 40 years, median body mass index 24 kg/m^2^). Of these, 258 (27%) participants received 2DR, and 621 (65%) received 3DR. Proportions of participants with different reasons for starting/discontinuing DTG are presented in Figure 1. At 24 months in the overall 3DR sub‐population, the proportion of participants with viral suppression with VL <250 copies/ml increased from 58.0% (360/621) to 83% (456/549), and the proportion with VL <50 copies/ml increased from 48.1% (299/621) to 77% (423/549). In the 2DR, the proportion of participants was 86.7% (217/250) with VL <250 copies/ml and 83% (209/250) with VL <50 copies/ml. Meanwhile, CD4 cell count increased significantly in both subgroups (Table 1). Overall, at 24‐month, 147 adverse drug reactions deemed related to DTG (ADRs)/serious adverse events (SAEs) were documented in 118 PLHIV (12.3%), of which 85 ADRs in 80 PLHIV (8.3%) were DTG‐related, 19 ADRs, including two SAEs, resulted in discontinuation of DTG in 1.7% (*n* = 16/959). The rate of DTG‐related ADRs was 6.4% (40 persons) in the 3DR and 13.2% (34 persons) in the 2DR sub‐populations.

**P094: Figure 1:**
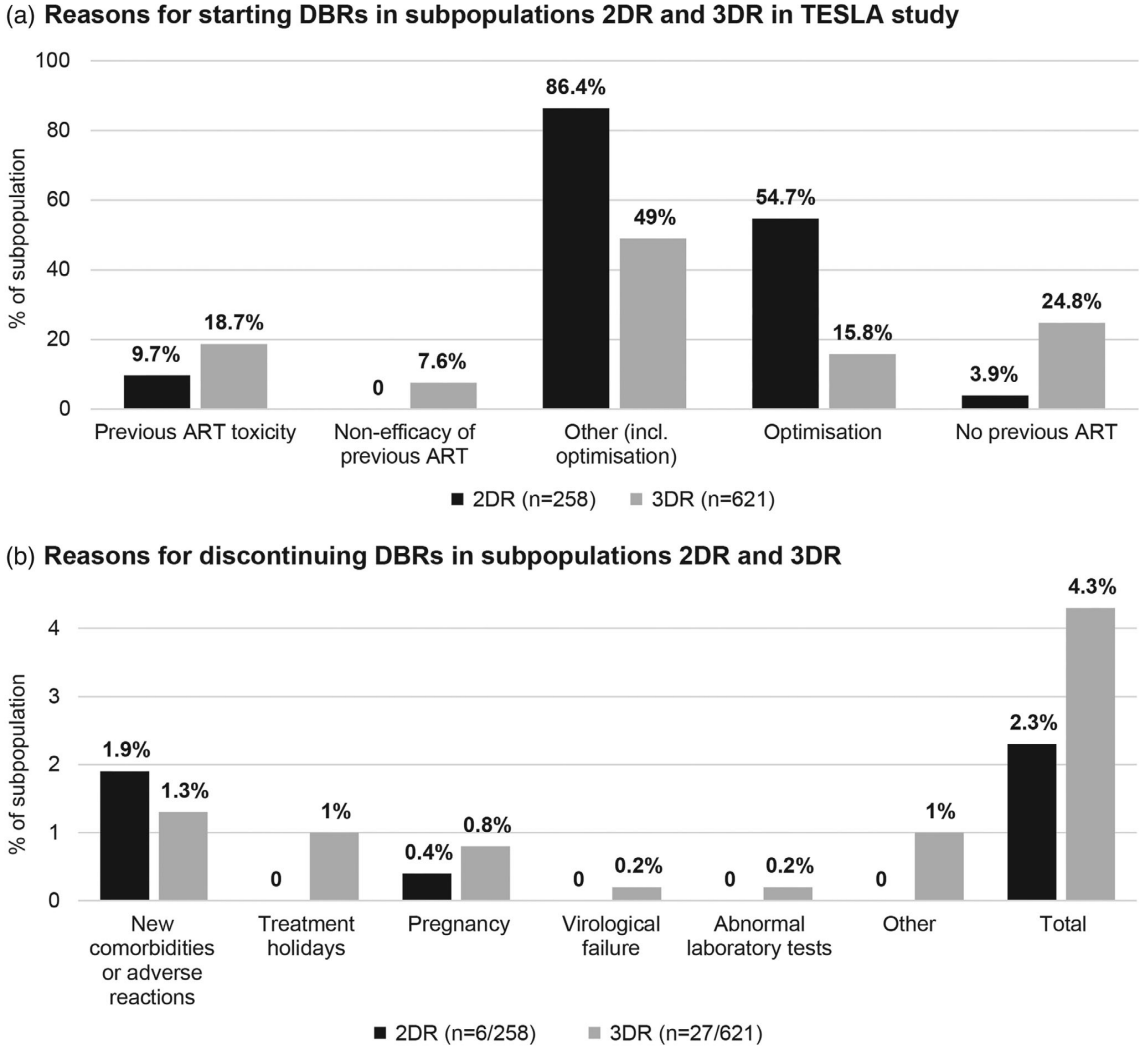
Reasons for starting DTG (a) or discontinuing DTG (b) in sub‐populations 2DR and 3DR in the TESLA study by 24 weeks of observation. 2DR, two‐drug regimen; 3DR, three‐drug regimen.

**P094: Table 1:** Virological outcomes at 24 months in real‐life settings in the Russian TESLA cohort

	Viral suppression (VL <250 copies/ml), *n* (%)^a^		Low‐level viraemia (250 VL 500 copies/ml), *n* (%)^a^		Viral suppression (VL <50 copies/ml), *n* (%)^a^		Virological rebound, *n* (%)^a^		Non‐response, *n* (%)^a^		CD4 cell count change compared to baseline, cells/mm^3^ mean±SD/median	
	ART‐naive	ART‐experienced	ART‐naive	ART‐experienced	ART‐naive	ART‐experienced	ART‐naive	ART‐experienced	ART‐naive	ART‐experienced	ART‐naive	ART‐experienced
Two‐drug regimen (2DR) sub‐population (ART‐naïve *n* = 10/258 [3.9%]; ART‐experienced *n* = 248/258 [96.1%])												
*N*	10	248	10	248	10	248	10	248	10	248	10	248
Baseline	n/a	233 (94.0%)	n/a	0 (0.0%)	n/a	224 (90.3%)	−**^c^**	−**^c^**	n/a	n/a	n/a	n/a
*n* ^b^	10	240	10	240	10	240	10	240	10	240	10	240
24 months	9 (90.0%)	208 (86.7%)	0 (0.0%)	3 (1.2%)	9 (90.0%)	200 (83.3%)	−^c^	−^c^	0 (0.0%)	2 (0.8%)	238.9±173.4 /249.0	69.1±200.7/58.5^d^
Three‐drug regimen (3DR) sub‐population (ART‐naïve *n* = 154/621 [24.8%]; ART‐experienced *n* = 467/621 [75.2%])												
*n*	154	467	154	467	154	467	154	467	154	467	154	467
Baseline	n/a	360 (77.1%)	n/a	11 (2.4%)	n/a	299 (64.0%)	n/a	n/a	n/a	n/a	n/a	n/a
*n* ^b^	126	423	126	423	126	423	126	423	126	423	126	423
24 months	106 (84.1%)	350 (82.7%)	1 (0.8%)	5 (1.2%)	100 (79.4%)	323 (76.4%)	1 (0.8%)	3 (0.7%)	3 (2.3%)	5 (1.2%)	237.8±220.4 /236.0^d^	60.1±200.7 /64.0^d^

Abbreviations: 3DR, three‐drug regimen; *n*, number of persons; n/a, not applicable; SD, standard deviation; VL, viral load.

^a^Percentages are calculated in the corresponding sub‐population.

^b^Number excluding persons lost from contact and change of place of residence/removed from dispensary registration.

^c^No data available.

^d^ *p* < 0.001.

**Conclusions**: The use of DBRs in real‐life settings in the Russian TESLA cohort was associated with high effectiveness and safety for 24 months. The rate of DTG‐related ADRs was 8.3%; no new safety signals were identified.

### P095 Demographic and clinical characteristics of treatment naïve people with HIV, and their healthcare professionals' reasons for treatment choice: findings from a real‐world survey in five European countries

Andrea Marongiu ^1^, Tali Cassidy^1^, Joshua Gruber^2^, Hannah Jones^3^, Katherine Li^3^, Will Ambler^3^, Fritha Hennessy^3^, Tim Holbrook^3^

^1^Real‐World Evidence, Gilead Sciences Ltd, Uxbridge, UK, ^2^Global Medical Affairs, Gilead Sciences, Inc., Foster City, CA, USA, ^3^Real‐World Evidence, Adelphi Real World, Bollington, UK

**Background**: We aim to compare demographics and clinical characteristics for treatment naïve (TN) people with HIV (PWH) receiving different classes of antiretroviral therapy (ART), and the reasons for healthcare professional (HCP) treatment choice.

**Materials and methods**: Data were drawn from the Adelphi HIV Disease Specific Programme™, a real‐world, cross‐sectional survey including retrospective clinical data run between September 2022 and June 2023 in France, Germany, Italy, Spain and the United Kingdom. HCPs provided data for 10 sequential consulting PWH. Demographics and clinical characteristics (where available) were compared using ANOVA or chi‐square tests. HCP‐reported reasons for treatment choice were grouped using principal component analysis (PCA). Inverse probability weighted regression adjustment (IPWRA) was used to predict the percentage of PWH for whom their HCP would select a response within a given factor by the most frequent ARTs.

**Results**: HCPs (*n* = 181) provided data for 1099 TN‐PWH. The median TN‐PWH age was 36 (IQR 30–45) years. Most (72%) were cisgender male and 81% (*n* = 756/929) were White. The median viral load (VL) prior to current treatment was 31,750 (IQR 4500–110,000; *n* = 711) copies/ml and CD4 count was 384 (IQR 240–561, *n* = 737) cells/mm^3^. At the time of data collection, 64% had no comorbidities, the median time on current treatment was 1.03 years (IQR 0.4–2.4). Most TN‐PWH received a single‐tablet regimen (STR; 88%), containing an integrase inhibitor (INSTI) (69%, *n* = 668/963). The most prescribed regimens were BIC/TAF/FTC (37%) and DTG/3TC (10%), with other regimens grouped by third agent due to lower frequency. Across the treatment regimens, statistically significant differences (*p* < 0.05) were observed for most baseline characteristics (Table 1). The most common HCP‐stated reason for selecting current ART was virological potency (65%), followed by tolerability (52%) and clear and simple dosing instructions (45%). Further reasons for treatment selection varied across regimens (Figure 1). IPWRA indicated several PCA‐derived factors were associated with BIC/TAF/FTC or DTG/3TC.

**P095: Table 1:** Baseline demographics and clinical characteristics of treatment‐naïve people with HIV by most frequent regimens/drug classes

	**BIC/TAF/FTC, *n* = 404**	**DTG/3TC, *n* = 112**	**Other DTG‐based STRs, *n* = 88**	**Other INSTI‐based STRs, *n* = 64**	**NNRTi‐based STRs, *n* = 197**	**PI‐based STRs, *n* = 98**	**MTRs, *n* = 136**	***p*‐value**
**Age**, *n*; median (IQR)	404; 36.0 (29.00−44.00)	112; 35 (30.00−43.00)	88; 38.5 (31.00−49.00)	64; 40.5 (34.00−48.50)	197; 36 (29.00−47.00)	98; 34 (29.00−42.00)	136; 38.5 (30.00−49.00)	0.0034
**Gender**, *n* (%)								
Cisgender man	308 (76.24)	89 (79.46)	60 (68.18)	46 (71.88)	125 (63.78)	73 (75.26)	92 (68.66)	0.0030
Cisgender woman	69 (17.08)	23 (20.54)	17 (19.32)	14 (21.88)	61 (31.12)	16 (16.49)	33 (24.63)	
Transgender woman	14 (3.47)	0 (0.00)	8 (9.09)	3 (4.69)	3 (1.53)	5 (5.15)	3 (2.24)	
Transgender man	1 (0.25)	0 (0.00)	1 (1.14)	1 (1.56)	4 (2.04)	1 (1.03)	2 (1.49)	
Other	12 (2.97)	0 (0.00)	2 (2.27)	0 (0.00)	3 (1.53)	2 (2.06)	4 (2.99)	
**Sexual orientation**, *n* (%)								
Homosexual	226 (55.94)	62 (55.36)	50 (56.82)	29 (45.31)	73 (37.06)	38 (38.78)	55 (40.44)	0.0002
Heterosexual	148 (36.63)	41 (36.61)	34 (38.64)	30 (46.88)	111 (56.35)	48 (48.98)	63 (46.32)	
Bisexual/pansexual	30 (7.43)	8 (7.14)	4 (4.55)	5 (7.81)	13 (6.60)	11 (11.22)	18 (13.24)	
Other (specify)	0 (0.00)	1 (0.89)	0 (0.00)	0 (0.00)	0 (0.00)	1 (1.02)	0 (0.00)	
**BMI, grouped categories**, *n* (%)	352	103	71	56	179	91	102	<0.0001
Underweight	12 (3.41)	2 (1.94)	1 (1.41)	0 (0.00)	3 (1.68)	1 (1.10)	7 (6.86)	
Healthy weight	207 (58.81)	48 (46.60)	46 (64.79)	43 (76.79)	100 (55.87)	50 (54.95)	54 (52.94)	
Overweight	120 (34.09)	49 (47.57)	24 (33.80)	11 (19.64)	56 (31.28)	38 (41.76)	31 (30.39)	
Obese	13 (3.69)	4 (3.88)	0 (0.00)	2 (3.57)	20 (11.17)	2 (2.20)	10 (9.80)	
**Ethnicity**, *n* (%)	354	95	65	47	165	97	106	0.2121
White/Caucasian	297 (83.90)	85 (89.47)	52 (80.00)	37 (78.72)	130 (78.79)	80 (82.47)	75 (70.75)	
Hispanic/Latino/Latina	31 (8.76)	7 (7.37)	8 (12.31)	4 (8.51)	11 (6.67)	9 (9.28)	9 (8.49)	
Afro‐Caribbean	12 (3.39)	1 (1.05)	3 (4.62)	5 (10.64)	17 (10.30)	6 (6.19)	16 (15.09)	
Middle Eastern	6 (1.69)	1 (1.05)	1 (1.54)	0 (0.00)	2 (1.21)	1 (1.03)	1 (0.94)	
Asian (other)	3 (0.85)	0 (0.00)	1 (1.54)	0 (0.00)	2 (1.21)	0 (0.00)	3 (2.83)	
South‐East Asian	2 (0.56)	1 (1.05)	0 (0.00)	0 (0.00)	2 (1.21)	0 (0.00)	1 (0.94)	
Asian (Indian subcontinent)	1 (0.28)	0 (0.00)	0 (0.00)	0 (0.00)	1 (0.61)	0 (0.00)	0 (0.00)	
Other	2 (0.56)	0 (0.00)	0 (0.00)	1 (2.13)	0 (0.00)	1 (1.03)	1 (0.94)	
**Viral load prior to initiation of current ART, copies/ml**, *n*; median (IQR)	289; 47,000 (9870.00−1.6e+05)	87; 7814 (1280.00−38,600.00)	56; 73,781.5 (22,082.50−1.8e+05)	36; 57,595.5 (15,000.00−2.3e+05)	103; 18,800 (3444.00−50,000.00)	74; 12,000 (1240.00−59,000.00)	66; 52,450 (10,000.00−1.5e+05)	0.0191
**CD4T** **levels prior to initiation of current ART, cells/mm^3^**, *n*; median (IQR)	287; 420 (223.00−580.00)	89; 533 (418.00−660.00)	58; 306.5 (240.00−390.00)	36; 289 (200.00−461.00)	113; 340 (260.00−500.00)	81; 415 (222.00−620.00)	73; 327 (200.00−453.00)	<0.0001
**CDC stage**, *n* (%)								<0.0001
1 (CD4 count ≥500 cells/mm^3^)	293 (72.52)	96 (85.71)	51 (57.95)	46 (71.88)	126 (63.96)	77 (78.57)	77 (56.62)	
2 (CD4 count ≥200 cells/mm^3^)	43 (10.64)	7 (6.25)	23 (26.14)	9 (14.06)	41 (20.81)	10 (10.20)	21 (15.44)	
3 (CD4 count <200 cells/mm^3^)	46 (11.39)	1 (0.89)	7 (7.95)	1 (1.56)	15 (7.61)	8 (8.16)	15 (11.03)	
Unknown	22 (5.45)	8 (7.14)	7 (7.95)	8 (12.50)	15 (7.61)	3 (3.06)	23 (16.91)	
**Presence of any resistance mutations, *n* (%)**	319	90	65	42	133	52	95	<0.0001
No treatment resistance mutations	306 (95.92)	84 (93.33)	57 (87.69)	40 (95.24)	107 (80.45)	46 (88.46)	62 (65.26)	
Treatment resistance mutations (any)	13 (4.08)	6 (6.67)	8 (12.31)	2 (4.76)	26 (19.55)	6 (11.54)	33 (34.74)	

Abbreviations: 3TC, lamivudine; ABC, abacavir; ART, anti‐retroviral therapy; BIC, bictegravir; BMI, body mass index; c, cobicistat; CDC, Centers for Disease Control and Prevention; DOR, doravirine; DRV, darunavir; DTG, dolutegravir; EFV, efavirenz; EVG, elvitegravir; FTC, emtricitabine; INSTI, integrase inhibitor; IQR, interquartile range; MTR, multi‐tablet regimen; NNRTI, non‐nucleoside reverse transcriptase inhibitor; PI, protease inhibitor; PWH, people with HIV; RPV, rilpivirine; STR, single tablet regimen; TAF, tenofovir alafenamide; TDF, tenofovir disoproxil.

Regimen groups: Other DTG‐based STRs (DTG/ABC/3TC, DTG/RPV); Other INSTI‐based STRs (EVG/c/TAF/FTC, EVG/c/TDF/FTC), NNRTI‐based STRs (DOR/TDF/3TC, EFV/FTC/TDF, RPV/TDF/FTC, RPV/TAF/FTC); PI‐based STRs (DRV/c/TAF/FTC). Responses to “Please indicate your reasons for choosing the patient's current HIV ART therapy,” allowing for multiple response multi‐choice categoric question, 45 options. The groupings are mutually exclusive based on the PWH current treatment regimen; the following statistical tests were performed: ANOVA tests for continuous and chi‐square tests for categorical outcomes.

**P095: Figure 1:**
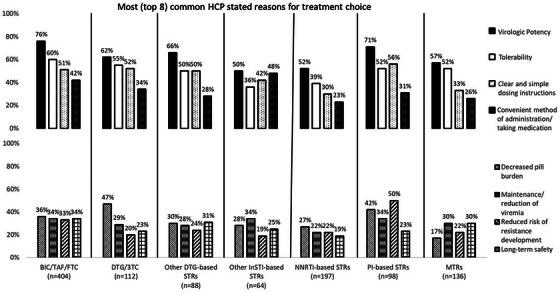
Most common (top eight) HCP‐reported reasons for treatment choice by most frequent regimens/drug classes. Treatment groups: Other DTG‐based STRs (DTG/ABC/3TC, DTG/RPV); Other INSTI‐based STRs (EVG/c/TAF/FTC, EVG/c/TDF/FTC); NNRTI‐based STRs (DOR/TDF/3TC, EFV/FTC/TDF, RPV/TDF/FTC, RPV/TAF/FTC); PI‐based STRs (DRV/c/TAF/FTC). Responses to “Please indicate your reasons for choosing the patient's current HIV ART therapy,” allowing for multiple response (multi‐choice categoric question). The groupings are mutually exclusive based on the PWH current treatment regimen. 3TC, lamivudine; ABC, abacavir; ART, anti‐retroviral therapy; BIC, bictegravir; BMI, body mass index; BP, blood pressure; c, cobicistat; CDC, Centers for Disease Control and Prevention; DOR, doravirine; DTG, dolutegravir; EFV, efavirenz; EVG, elvitegravir; FTC, emtricitabine; INSTI, integrase strand transfer inhibitor; IQR, interquartile range; MTR, multi‐tablet regimen; NNRTI, non‐nucleoside reverse transcriptase inhibitor; PI, protease inhibitor; RPV, rilpivirine; STR, single tablet regimen; TAF, tenofovir alafenamide; TDF, tenofovir disoproxil.

**Conclusions**: Although first‐line ART prescribing generally followed European guidelines [1], regimen preferences varied depending on patients’ baseline characteristics. Following balancing on population differences, HCPs’ reasons for choice varied when comparing B/F/TAF and DTG/3TC. These results warrant additional studies to understand HCP perceptions of ART and PWH key groups’ needs.

**Reference**

1. European AIDS Clinical Society. EACS Guidelines, version 11.0 [Internet]. 2021 [cited 2024 Jun 24]. Available from: https://www.eacsociety.org/media/final2021eacsguidelinesv11.0_oct2021.pdf.

### P096 Results at month 7 of CABO‐CHANCE study: real‐world evidence (RWE) on the use of intramuscular cabotegravir plus rilpivirine long‐acting (CAB+RPV LA) dosed every 2 months in viral suppressed people with HIV (PWH)

Carmen Hidalgo Tenorio ^1^, María Aguilera‐García^2^, Ignacio De los Santos^2^, David Vinuesa^3^, Mohamed Omar^4^, Ana Lopez Lirola^5^, Enrique Bernal^6^, Onofre Juan Martinez^7^, Alberto Romero^8^, Patricia Sorni^9^, Teresa Lopez^10^, Salvador López Cárdenas^11^, Santiago Moreno^12^, Isabel San Joaquín Conde^13^, José Ramón Blanco^14^, Coral Garcia‐Vallecillos^1^

^1^Infectious Diseases, Hospital Universitario Virgen de las Nieves, Granada, Spain, ^2^Infectious Diseases, Hospital Universitario de la Princesa, Madrid, Spain, ^3^Infectious Diseases, Hospital Universitario San Cecilio, Granada, Spain, ^4^Infectious Diseases, Complejo Hospitalario de Jaen, Jaen, Spain, ^5^Infectious Diseases, Hospital Universitario de Canarias, La Laguna, Spain, ^6^Infectious Diseases, Hospital Universitario Reina Sofia, Murcia, Spain, ^7^Hospital Santa Lucia, Cartajena, Spain, ^8^Infectious Diseases, Hospital Universitario Puerto Real, Cádiz, Spain, ^9^Infectious Diseases, Hospital Universitario Son Llatzer, Palma de Mallorca, Spain, ^10^Internal Medicine, Hospital Santa Ana, Motril, Spain, ^11^Infectious Diseases, Hospital Universitario de Jerez, Cádiz, Spain, ^12^Infectious Diseases, Hospital Universitario Ramón y Cajal, Madrid, Spain, ^13^Infectious Diseases, Hospital Universitario Lozano Blesa, Zaragoza, Spain, ^14^Infectious Diseases, Hospital Universitario San Pedro, Logroño, Spain

**Background**: PWH have experienced a large increase in survival due to antiretroviral treatment (ART). There is currently a double paradigm shift in ART, from triple therapy to dual therapy (DT), and from oral to injectable DT with CAB+RPV LA. The aim of this study was to describe the PWH receiving CAB+RPV LA in Spain, its effectiveness, safety and the changes it may cause in inflammatory markers, HRQoL and sleep quality, health satisfaction and stigma at month 7.

**Methods**: Prospective (inclusion period June 2023–January 2024), longitudinal, Spanish multicentre (14 hospitals) study, including pre‐treated PWH with HIV‐1 RNA (VL) <50 c/ml at least 6 months that switching from any oral‐ART to CAB+RPV LA. At baseline (BL) and 7 months (7M) thereafter, CD4, CD8, VL, anthropometric parameters, creatinine clearance (CrCl), lipids and inflammatory markers were assessed; and PROs using as instruments WHOQOL‐HIV‐BREF, HIV‐Stigma Scale in Spaniards (HSSS), Pittsburgh Sleep Quality Index (PSQI) and also health satisfaction and adverse effects questionnaires.

**Results**: Two hundred and twenty‐four PWH were included in this analysis, with a mean age of 45.4 (±11.2), 90.2% male, 78.5% MSM, 28.6% had AIDS history, 4 (IQR 2–5) lines of previous ART and were diagnosed for 12 years (IQR 7–17). In 83.5% of cases, the switch to CAB+RPV LA was proposed by the physician. One hundred and forty‐nine out of 224 participants completed the first 7 months of follow‐up and 3.6% had VL ≥50 copies/ml, all were blips (min 52–max 176 copies/ml). There was no discontinuation. Between BL and 7 months, there was statistically increase in BMI (25.7±3.6 vs. 25.8±4.1; *p* = 0.009), improvement in CrCl (90.9 ml/minute [IQR 78.2–101.6] vs. 99.1 [IQR 85.1–110.3]; *p* = 0.0001), and reduction in interleukin‐6 levels (2.62 pg/ml [IQR 1.4–6.1] vs. 1.76 [IQR 1.4–3.1]; *p* = 0.002). There were no changes in CD4/CD8 ratio, lipids, reactive C‐protein, fibrinogen or D‐dimer. Participants reported statistically improvements in adverse events (reducing pain intensity 3 [IQR 3–3] vs. 2 [IQR 2–2]; *p* = 0.0001, and dysthermic sensation [35% vs. 0.7%; *p* = 0.0001]), in health satisfaction (none: 0.5% vs. 0.7%; little: 7.8% vs. 0.7%; some: 18.8% vs. 12.8%; quite a lot: 54.1% vs. 64.5%; a lot: 18.8% vs. 21.3%; *p* = 0.021), sleep quality (quite good: 24.3% vs. 30.5%; good: 44.0% vs. 47.5%; bad: 26.1% vs. 17%; quite bad: 5.5% vs. 5.0%); but no change reported in quality of life (*p* = 0.661) or stigma (*p* = 0.81).

**Conclusions**: This RWE study of CAB+RPV LA in PWH with extensive prior oral‐ART and more than a decade of diagnosis, demonstrated that in the first 7 months of follow‐up the LA regimen maintains viral suppression, with adverse effects decreasing in intensity, improved creatinine clearance, decreased IL‐6 and positive influences health satisfaction and sleep quality.

### P097 Efficacy and safety of long‐acting cabotegravir + rilpivirine at All Saints Clinic in Wroclaw (Poland): real‐life single‐centre 2‐year experience

Marzena Dawiec ^1^, Jacek Gasiorowski^1^, Aleksandra Szymczak^2^, Aleksander Zinczuk^1^, Malgorzata Inglot^2^, Brygida Knysz^2^, Kamila Zielinska^1^, Joanna Bortkiewicz^1^, Michal Furdal^1^, Mateusz Bozejko^2^, Bartosz Szetela^1,2^

^1^All Saints Clinic, Wroclawskie Centrum Zdrowia, Wroclaw, Poland, ^2^Infectious Diseases, Liver Disease and Acquired Immune Deficiencies, Wroclaw Medical University, Wroclaw, Poland

**Background**: Long‐acting injectable cabotegravir + rilpivirine (CAB+RPV LAI) was made available to Polish patients in June 2022 and covered by the National Health System [1–4]. The All Saints Clinic was the first centre to offer this regimen in Poland and represents now almost 50% of all Polish LAI patients.

**Materials and methods**: We analysed medical records of patients who received at least one dose of CAB+RPV LAI between June 2022 and May 2024. They included socio‐demographic, clinical, metabolic and immunological variables including HIV genotyping and cART history as well as follow‐up.

**Results**: One hundred and ninety‐two patients had started CAB+RPV LAI in the analysed period. One hundred and eighty‐eight patients had data available for analysis. One hundred and seventy patients commenced LAI virally suppressed (suppressed switch—SS), and 18 patients without suppression (non‐SS). In the SS group, nine patients completed only LAI initiation phase (two visits), and 161 patients completed at least three. One hundred and sixty‐nine patients (99.41%) maintained suppression up to the data cut‐off point or at last observation on LAI treatment. Most patients reported temporary stage G1/G2 AEs (pain, pyrexia). Ten patients discontinued LAI due to non‐virological reasons, nine for injection‐related AEs interfering with daily routine and one for time constraints. One VF was observed (overlooked interaction with rifampicin; re‐suppression achieved on switched therapy TDF/FTC QD + DLG BID). In summary, 159 out of 170 (93.52%) successfully continued LAI with virological and immune control. In the non‐SS group at baseline, 12 patients had HIV‐1 VL 50–1000 copies/ml and six had VL >1000 copies/ml, mostly due to non‐adherence. Sixteen out of 18 patients (88.88%) suppressed after the fourth injection. The two non‐responders were heavily experienced and had VL at switch above 1000 c/ml, were pill non‐adherent and one developed rilpivirine and all‐INSTI resistance mutations. No discontinuations due to non‐virological reasons were seen.

**Conclusions**: CAB+RPV LAI has been shown to be highly efficacious in real‐life setting with low VF rate of 1.59% (3/188) in a mixed cohort. The few failures were due to unsuppressed viraemia at baseline or DDIs. Tolerance profile was acceptable by most patients with low overall drop‐out rate of 5.31% (10/188).

**References**

1. European AIDS Clinical Society. EACS Guidelines, version 12.0 [Internet]. 2023 [cited 2024 July 4]. Available from: https://www.eacsociety.org/media/guidelines‐12.0.pdf.

2. Parczewski M, Witak‐Jędra M, Aksak‐Wąs B, Ciechanowski P. Zasady opieki nad osobami zakażonymi HIV. Zalecenia PTN AIDS 2024 [Internet]. 2024 [cited 2024 July 4]. Available from: http://ptnaids.pl/images/pliki/zalecenie_2024‐caloscZAKLADKI.pdf.

3. Christopoulos K, Grochowski J, Mayorga‐Munoz F, Hickey MD, Imbert E, Szumowski JD, et al. First demonstration project of long‐acting injectable antiretroviral therapy for persons with and without detectable human immunodeficiency virus (HIV) viremia in an urban HIV clinic. Clin Infect Dis. 2023;76(3):e645‐51.

4. Sax PE, Thompson MA, Saag MS; IAS‐USA Treatment Guidelines Panel. Updated treatment recommendation on use of cabotegravir and rilpivirine for people with HIV from the IAS‐USA guidelines panel. JAMA. 2024;331(12):1060‐1.

### P098 Real‐world persistence of bictegravir‐ versus dolutegravir‐based single tablet regimens in a large urban Canadian HIV centre

David Knox ^1^, Jennifer McCully^2^, Graham S. Smith^1^, Negin Mousadifar^3^, Angela Underhill^2^, Taban Saifi^4^, Dylana Mumm^4^, Colin Kovacs^5^, Jason Brunetta^1^, William Tsang^1^, Megan Acsai^1^, Barry Merkley^1^, Kevin Giolma^1^, David Fletcher^1^, Tali Cassidy^6^, Soodi Navadeh^7^, Mona Loutfy^8^

^1^Medicine, Maple Leaf Medical Clinic, Toronto, Canada, ^2^Research, Maple Leaf Medical Clinic, Toronto, Canada, ^3^Department of Medicine, University of Toronto, Toronto, Canada, ^4^Medical Affairs, Gilead Sciences Canada, Inc., Mississauga, Canada, ^5^Medicine, University of Toronto, Maple Leaf Medical Clinic and Department of Medicine, Toronto, Canada, ^6^Epidemiology, Gilead Sciences Europe, Ltd., Stockley Park, UK, ^7^Epidemiology, Gilead Sciences, Inc., Foster City, CA, USA, ^8^Research and Innovation, Women's College Hospital, and Medicine, Maple Leaf Medical Clinic, and Department of Medicine, University of Toronto, Toronto, Canada

**Background**: Current guidelines for first‐line ART and subsequent switches have increasingly recommended the use of single‐tablet regimens (STRs) that incorporate integrase strand transfer inhibitors (INSTIs) such as bictegravir (BIC) and dolutegravir (DTG). Clinically, it is important to determine which STR regimen offers better tolerability and results in fewer discontinuations.

**Materials and methods**: We compared the persistence (i.e. the lack of regimen discontinuation) between BIC/FTC/TAF and DTG‐containing STRs, combined and individually (ABC/3TC/DTG, 3TC/DTG, RPV/DTG). Retrospective analyses from adults with HIV at the Maple Leaf Medical Clinic in Toronto, Canada, who started or switched to ABC/3TC/DTG, 3TC/DTG, DTG/RPV and BIC/FTC/TAF between 1 January 2016 and 31 December 2022 were conducted. The primary outcome, being persistence, was defined as the number of days from STR baseline start date until STR discontinuation (defined as an STR prescription switch or delay in electronic medical record [EMR] STR prescription refill >90 days) by 31 December 2023. The discontinuation could include a switch to another regimen or complete ART discontinuation. Switches of ABC/3TC/DTG to 3TC/DTG were not considered as clinically significant discontinuations. Time‐to‐event analyses comparing BIC/FTC/TAF to all DTG‐containing STRs were conducted using Kaplan‐Meier curves and Cox proportional hazards models to assess regimen persistence.

**Results**: There were 1732 adults with HIV (1187‐BIC/FTC/TAF, 359‐3TC/ABC/DTG, 113‐3TC/DTG, 73‐DTG/RPV) included. By 31 December 2023, 387 (22.3%) had discontinued their STR by a median of 402 days (IQR 151–871). For the BIC/FTC/TAF group, 224 (18.9%) discontinued by 383 (IQR 140–774) median days. In the overall DTG‐containing STR group, 163 (29.9%) discontinued by 479 (IQR 156–1042) median days. Compared to BIC/FTC/TAF, the DTG‐containing STRs overall and ABC/3TC/DTG subgroups had significantly higher risks of discontinuations with unadjusted hazard ratios of 1.35 (95% CI 1.09–1.66) and 1.43 (95% CI 1.13–1.80), respectively. Compared to BIC/FTC/TAF, discontinuations for the 3TC/DTG and DTG/RPV subgroups did not show statistically significant differences.

**Conclusions**: STRs containing INSTIs proved to have robust persistence with discontinuations less than 30% with a long median time to discontinuation of 402 days. BIC/FTC/TAF had a preferable portfolio of persistence over DTG‐containing STR regimens overall and ABC/3TC/DTG, making it a continued important choice as an ART in first‐line therapy and for switch.

### P099 Feasibility and satisfaction of interventions measures (FIM and HIVTISQ) of implementation long‐acting (LA) CAB+RPV administration out of HIV units: the IMADART study

Alfonso Cabello ^1^, Irene Carrillo Acosta^1^, Aws Al‐Hayani^1^, Marta López De Las Heras^1^, Cristina Algar^2^, Inmaculada Burillo^2^, Beatriz Álvarez‐Álvarez^1^, Laura Prieto‐Pérez^1^, Ignacio Mahíllo‐Fernández^3^, Macarena Bonilla^4^, Laura García^4^, José Manuel Caraballo^5^, Mónica Esparis^5^, Beatriz Gallego^6^, Javier Bécares^4^, Miguel Górgolas Hernández‐Mora^1^

^1^Infectious Diseases, Fundación Jiménez Díaz University Hospital (FJD) Health Research Institute, Autónoma University of Madrid (UAM), Madrid, Spain, ^2^Infectious Diseases, Fundación Jiménez Díaz Health Research Institute, Madrid, Spain, ^3^Fundación Jiménez Díaz Health Research Institute, Madrid, Spain, ^4^Pharmacy, Fundación Jiménez Díaz University Hospital (FJD) Health Research Institute, Madrid, Spain, ^5^Specialist Care Centres, Madrid, Spain, ^6^Polyvalent Day Hospital, Madrid, Spain

**Background**: Long‐acting CAB and RPV has shown to be acceptable and feasible for the maintenance of HIV suppression for PWH. The real‐world implementation in different health settings outside the hospital HIV units and closer to patients’ neighbourhoods still have some challenges and lack of knowledge.

**Materials and methods**: Phase IV, open‐label, randomized, double‐arm, implementation‐effectiveness multicentre clinical trial, assessing the feasibility of intervention measure of (FIM) and satisfaction (HIVTISQ) of CAB LA+RPV LA administering in different healthcare settings (1:2): polyvalent day hospital (PDH) units and specialist care centres (SCCs).

**Results**: Ninety participants were included. Baseline characteristics and reasons to prefer LA treatment are in Table 1. Participants reported having ever concerns about their HIV status disclosure (65.6%), about forgetting to take oral ART (82.2%) or reminders of HIV status related to daily oral treatment (73.3%). The proportion of participants with an average composite FIM score greater than or equal to 4 at month 3 was 85.5%; satisfaction improved from 58.9 to 60 in HIVTSQs score. No differences between PDH and SCC was observed. In the intention‐to‐treat (ITT) analysis (missing = failure), effectiveness at month 3 was 94.4% (85/90) [95% CI 87.5–98.2%], and in the per‐protocol analysis (discontinuations not related with therapy excluded) was 98.8% (85/86) [95% CI 93.7–100%]. One patient discontinued due to injection site reactions (ISRs) (1.1%), three due to withdrawal of consent (change of city address or patient decision) and one due to researcher discretion (lack of adherence to visit schedules).

**P099: Table 1:** Participants’ baseline characteristics

Baseline characteristics		Participants (p) (*p* = 90)
Age (years), median (IQR)		44 (36−51)
Gender	Male	85 (94.4%)
	Female	5 (5.6%)
	Transgender woman	0 (0%)
HIV transmission	MSM	81 (91.0%)
	MSW	4 (4.5%)
	IDU	3 (3.4%)
	Unknown	1 (1.1%)
Race	Caucasian	72 (80.0%)
	Latin American	17 (18.9%)
	Others	1 (1.1%)
Chemsex practice	Yes	28 (31.1%)
Hepatitis B co‐infection	HBsAg+	0%
	Anti‐HBc+	16 (18.0%)
	Anti‐HBs+	56 (62.2%)
CD4 cells/mm^3^ (mean ± SD)	Basal CD4^+^	843±325
	CD4 <200	1 (1.1%)
Median time with HIV (years)		9.3 (IQR 6.1−14.1)
Median time with ART (years)		8.6 (IQR 5.9−12.7)
Previous drug resistance testing not available		47 (52.2%)
Reasons to prefer switch to long‐acting treatment	Tired of taking pills every day	49 (54.4%)
	More convenient to receive injections	74 (82.2%)
	Not having to carry the treatment	64 (71.1%)
	Worry about other people seeing or finding pills	42 (46.7%)
	To feel more in control of managing their HIV	36 (40%)
	Having more frequent interactions with HIV team	26 (28.9%)

Abbreviations: Anti‐HBc, hepatitis B core antibody; Anti‐HBs, hepatitis B surface antibody; ART, antiretroviral treatment; HBsAg, hepatitis B surface antigen; IDU, injecting drug user; MSW, men who have sex with women.

**Conclusions**: Treatment with CAB LA+RPV LA administered in different healthcare settings, outside of HIV units, is feasible and well tolerated for PWH, without compromising effectiveness and improving treatment satisfaction.

### P100 Long‐term efficacy, safety and metabolic impact of generic dolutegravir in treatment‐naïve and experienced people living with HIV: a real‐world study in Argentina

Edgardo Bottaro^1^, Angeles Tisne^1^, Brenda Bacelar^1^, Claudia Migazzi^1^, Macarena Roel^1^, Maximiliano Bergman^2^, Diego Cecchini ^1^, Isabel Cassetti^1^

^1^ Medical Area, Helios Salud, Buenos Aires, Argentina, ^2^Medical Affairs, Laboratorio Richmond, Buenos Aires, Argentina

**Background**: Dolutegravir (DTG) is a recommended integrase inhibitor for treatment‐naïve (TN) and treatment‐experienced (TE) people with HIV (PWHIV). Argentina has access to generic DTG, but long‐term follow‐up data are lacking. We aim to describe for generic DTG combined with NRTIs: (1) persistence, efficacy (viral load <50 c/ml) and safety; (2) metabolic impact; (3) body weight changes in both TN and TE PWHIV.

**Methods**: Real‐world descriptive study of PWHIV initiating DTG+NRTIs in Argentina with two components: a retrospective analysis (48‐month follow‐up) from 10/2019 to 05/2024, and a prospective cohort (24‐month follow‐up) from 10/2021 to 10/2023.

**Results**: Retrospective cohort (*n* = 884): 84% TE (*n* = 740), 71% were male. Median (IQR) age was higher in TE: 47 (40–55) versus 36 (29–44) years in TN, as was comorbidity prevalence (64% vs. 30%); 89% of TE were undetectable at DTG initiation. Median viral load in TN was 35,400 c/ml (9972–134,471). TE were mostly switching from PI‐based (39%) or NNRTI‐based (38%) regimens. Accompanying NRTIs were predominantly XTC/TDF (74% in TN, 39% in TE) and 3TC (15% in TN, 37% in TE). Persistence at 24, 36 and 48 months was 97%, 98% and 100%, respectively; efficacy was 92%, 92% and 96%; adverse event prevalence was 1.3%, 0% and 0%. TE showed sustained decreases in median total cholesterol, HDL, LDL and triglycerides (*p* < 0.01 compared to baseline) without significant weight changes. TN showed no changes in metabolic profile. Prospective cohort (*n* = 123): 73% TE, 69% male, 71% on triple therapy. Median age was higher in TE than TN (53 vs. 39 years), as was baseline weight: 81 (71–92) versus 63 (55–70) kg. Persistence at 12 and 24 months was 99% and 100%; 93% were undetectable at 24 months. TE maintained stable weight: median of 81 (70–92) kg throughout follow‐up. TN showed significant weight gain: baseline median 63 (55–70) kg versus 73 (57–82) kg at 12 and 76 (63–83) kg at 24 months.

**Conclusions**: DTG had no long‐term impact on weight in TE. Generic DTG+NRTIs demonstrated high efficacy, safety and a favourable long‐term metabolic profile in TN and TE PWHIV.  These findings support the use of generic DTG‐based regimens in resource‐limited settings.

### P101 PAIRED—PAtIent Reported Experiences and perceiveD benefit of treatment with dolutegravir/lamivudine (DTG/3TC): a sub‐analysis of people with HIV (PWH) switching from bictegravir/emtricitabine/tenofovir alafenamide (BIC/FTC/TAF) in the United States

Jihad Slim^1^, Andrew P. Brogan^2^, Gavin Harper^3^, Katie Mycock^3^, Abigail McMillan^3^, Deanna Merrill^4^, Gustavo Verdier ^5^

^1^Infectious Diseases, New York Medical College, Valhalla, NY, USA, ^2^Health Outcomes, ViiV Healthcare, Durham, NC, USA, ^3^Health Outcomes, Adelphi Real World, Bollington, UK, ^4^Research & Development, ViiV Healthcare, Durham, NC, USA, ^5^Medical Affairs, ViiV Healthcare, Montreal, Canada

**Background**: PAIRED was a cross‐sectional survey and qualitative study of stable‐switch PWH receiving DTG/3TC in the United States. This sub‐analysis focused on PWH who switched from BIC/FTC/TAF to DTG/3TC to explore their treatment experiences and outcomes.

**Materials and methods**: PWH ≥18 years, receiving DTG/3TC for ≥3 months, were recruited through site‐led and community outreach methods. The survey included validated instruments (HIV‐Treatment Satisfaction Questionnaire [HIV‐TSQs], Adelphi Adherence Questionnaire™ [ADAQ], PoZQoL). Descriptive statistics were used to analyse survey results.

**Results**: Of the 474 PWH included in PAIRED, 132 (28%) switched from BIC/FTC/TAF to DTG/3TC. Median age of this subgroup was 49 years, 33% were female assigned sex at birth, 76% identified as non‐White and 33% were of Hispanic, Latinx or Spanish origin (Table 1). Majority of participants (60%) had been taking BIC/FTC/TAF for >12 months before switching to DTG/3TC, and for 70% of them, BIC/FTC/TAF was not their first regimen. Most common factors influencing the switch to DTG/3TC were to avoid side effects (66%), to minimize long‐term impact (53%) and to reduce the number of medicines (32%). PWH switching from BIC/FTC/TAF reported high satisfaction with DTG/3TC using the HIV‐TSQs (median total score 57.0 [IQR 52.0–60.0] out of 60). PWH also reported improved treatment satisfaction with DTG/3TC compared with BIC/FTC/TAF with 75% judging that DTG/3TC was somewhat to significantly better than BIC/FTC/TAF; 30% reported being very satisfied (6/6) before switching and 70% reported being very satisfied after switching to DTG/3TC (Figure 1). Good adherence was observed using the ADAQ (median score 0.3 [IQR 0.1–0.5] out of 4), with 92% of PWH reporting never or rarely missing a DTG/3TC dose. PWH reported moderate to high quality of life using the PoZQoL (median score 45.0 [IQR 36.2–54.0] out of 65).

**P101: Table 1:** PWH switching from BIC/TAF/FTC demographics (*n* = 132)

Age, median years (IQR)	49.0 (37.0−58.8)
<50 years	68 (51.5%)
≥50 years	64 (48.5%)
Gender identity (%)	
Cisgender woman	41 (31.1%)
Cisgender man	87 (65.9%)
Transgender woman	1 (0.8%)
Transgender man	1 (0.8%)
Non‐binary/gender queer	0 (0%)
A gender identity not listed here	2 (1.5%)
Race	
Asian	3 (2.3%)
Black or African American	45 (34.1%)
American Indian or Alaska Native	1 (0.8%)
Native Hawaiian or Pacific Islander	0 (0%)
White/Caucasian	63 (47.7%)
Multiracial	7 (5.3%)
Another race not mentioned	13 (9.8%)
Hispanic, Latinx and/or Spanish origin	
Yes	44 (33.3%)
No	88 (66.7%)
Sexual orientation	
Homosexual (gay/lesbian)	58 (43.9%)
Heterosexual (straight)	56 (42.4%)
Bisexual/pansexual	12 (9.1%)
Prefer not to say	6 (4.5%)
Insurance coverage (*n* = 124)	
Medicare	27 (21.8%)
Medicaid (or equivalent in your state)	42 (33.9%)
AIDS Drug Assistance Program (ADAP)	35 (28.2%)
Employer provided/sponsored insurance	50 (40.3%)
Privately arranged insurance	9 (7.3%)
Health insurance exchange plan	3 (2.4%)
Tricare/veterans healthcare	0 (0%)
Other	6 (4.8%)
Don't know	3 (2.4%)
Length of time person has been diagnosed with HIV‐1, median years (IQR)	11.5 (4.2−22.0)
Total number of different, prior ART regimens	
1	39 (29.5%)
2	31 (23.5%)
3	20 (15.2%)
4	8 (6.1%)
More than 4	34 (25.8%)

**P101: Figure 1:**
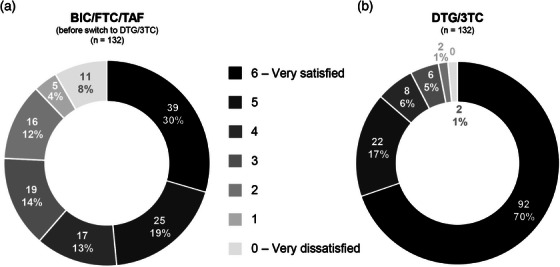
Overall satisfaction with BIC/FTC/TAF before switch to DTG/3TC (a) and satisfaction with DTG/3TC after switch (b).

**Conclusions**: PWH who switched from BIC/FTC/TAF to DTG/3TC reported high treatment satisfaction, good adherence and moderate to high quality of life. Avoiding side effects, minimizing long‐term impact and reducing the number of medicines were key factors influencing the switch. These findings suggest that DTG/3TC may offer a valuable treatment option for PWH who seek a simplified regimen with life‐time exposure to fewer drugs.

### P102 Factors influencing the prescription of long‐acting injectable dual therapy cabotegravir/rilpivirine in a Northwest Paris region cohort

Jeremie Leporrier ^1^, Vincent Daneluzzi^2^, Souhayla Amari^2^, Ghania Bouteira^3^, Séverine Dubois^2^, Feng Zeng^3^, Martine Bloch^1^, Ghislaine Nguisseu^2^, Elisabeth Aslangul^4^

^1^Medecine Interne et Maladies Infectieuses, Hopital Louis Mourier (APHP)/Hopital de Nanterre, Colombes, France, ^2^Medecine Interne et Maladies Infectieuses, Hopital de Nanterre, Nanterre, France, ^3^Medecine Interne et Maladies Infectieuses, COREVIH Ile de France Ouest, Nanterre, France, ^4^Medecine Interne et Maladies Infectieuses, Université Paris Cité, Paris, France

**Background**: In 2021, 50% of newly treated HIV patients in France were migrants, predominantly from sub‐Saharan Africa, facing gender imbalances, social difficulties and therapeutic inequalities: less integrase inhibitor (II), more protease inhibitor (PI) and fewer simplification strategies.

**Methods**: Description of socio‐demographic and immunovirological characteristics of patients, primarily migrants from sub‐Saharan Africa, who received at least one cabotegravir/rilpivirine injection at our centre.

**Results**: Of the 1237 HIV‐positive patients in our cohort, 97 (7.8%) received injectable dual therapy cabotegravir/rilpivirine between February 2022 and December 2023. The mean age was 48 years; 53% were male, 46% female and one transgender. Fifty‐eight percent were born in sub‐Saharan Africa, 30% in France and 27% identify as MSM. Half lived alone in their own accommodation. Of the 58 patients analysed, 64% were employed, 25% unemployed, two on disability and two retired. The average HIV follow‐up was 16.3 years, with a CD4 nadir of 70/mm^3^ and initial viral load of 96,000 copies/ml; 15% had an AIDS‐defining event. HIV subtypes included 27% CRF02, 20% subtype B and 6% subtype A/A6. Before treatment, the average CD4 count was 267/mm^3^, with 93 patients having undetectable viral loads. They had received an average of 3.8 treatment lines. Eighty‐three percent of patients were managed by three of nine physicians, with 57% starting with an oral phase. The average duration of injectable ARV exposure was 13 months, with 97% of injections administered in the clinic. Treatment interruptions occurred in 10% of cases: five due to intolerance, two for pregnancy planning, two at patient request and one due to inefficacy. Four patients had detectable viral loads during follow‐up, which became undetectable without treatment change in three cases. One patient discontinued treatment after two consecutive detectable viral loads, which became undetectable after switching therapy.

**Conclusions**: In our single‐centre cohort of PLHIV primarily born in sub‐Saharan Africa and facing social disadvantages, the initiation of long‐acting injectable cabotegravir/rilpivirine treatment is practitioner‐driven. One‐year tolerance and efficacy are favourable, demonstrating the feasibility of ensuring equal therapeutic opportunities for all.

### P103 T‐cell homeostasis parameters following switch to injectable cabotegravir plus rilpivirine in virally suppressed people living with HIV (PLWH)

Camilla Tincati ^1^, Leonardo Francesco Rezzonico^2^, Andrea Santoro^2^, Emanuela Ferrante^2^, Teresa Bini^2^, Lidia Gazzola^2^, Giulia Marchetti^2^

^1^Department of Health Sciences, Clinic of Infectious Diseases, San Paolo Hospital, University of Milan, Milan, Italy, ^2^Health Sciences, Clinic of Infectious Diseases, University of Milan, San Paolo Hospital, Milan, Italy

**Background**: Injectable cabotegravir (CAB) plus rilpivirine (RPV) is an ART regimen showing solid results in terms of virological suppression; in contrast, its effect on T‐lymphocyte homeostasis and activation is less established. We assessed peripheral T‐cell homeostasis parameters in virally suppressed PLWH switching to long‐acting, injectable (LAI) CAB+RPV.

**Materials and methods**: Retrospective single‐centre study. Individuals who switched to CAB+RPV and followed for 1 year were included. Lymphocyte sub‐populations were evaluated at four different time points (0, 3, 6 and 12 months). The following surface expression markers were assessed on whole blood by flow cytometry: CD4^+^/CD8^+^CD127^+^, CD4^+^/CD8^+^CD45RA^+^, CD8^+^CD45R0^+^, CD8^+^CD38^+^, CD8^+^CD38^+^R0^+^. Data were analysed by fitting a mixed model (GraphPad Prism Software).

**Results**: Fifty‐seven subjects were included in the analysis; 36 switching from two‐day regimen (2‐DR) and 21 from three‐day regimen (3‐DR) (Table 1). No virological failures were observed. Overall, the CD4/CD8 ratio and T‐cell parameters remained stable except for a trend to decreases in CD8^+^CD127^+^ (central memory) cells (T0: 703 cells/mmc, IQR [521–975]; T12: 647 cells/mmc, IQR [461–870], *p* = 0.04). In line with these findings, subjects switching from 3‐DR to CAB/RPV showed no major changes were observed in T‐cell parameters. Conversely, in the 2‐DR subgroup, we detected a decline in CD127‐expressing CD4^+^ (T0: 703 IQR [567–966], T12: 647 cells/mmc IQR [524–786], *p* = 0.01; Figure 1a) and CD8^+^ cells (T0: 819 IQR [609–1045]; T12: 673 cells/mmc IQR [490–885], *p* = 0.004; Figure 1b) as well as naïve CD8^+^CD45RA^+^ cells (T0: 354 IQR [267–562]; T12: 281 cells/mmc IQR [187–486], *p* = 0.01; Figure 1c). Of note, individuals switching from dolutegravir (DTG)/lamivudine (3TC), yet not those from DTG/RPV, showed a tendency to significant decreases CD8^+^ central memory cells (T0: 819 cells/mmc IQR [645–1125]; T12: 687 cells/mmc IQR [496–854], *p* = 0.05).

**P103: Table 1:** Demographic and HIV‐related parameters of the study population

Characteristic	*N* **= 57**
Male gender, *n* (%)	51 (90)
Age at switch, years, median [IQR]	47 (40−53)
Previous AIDS, *n* (%)	7 (12)
Previous ART regimens, median [IQR]	3 (2−4)
CD4^+^ at nadir, cells/mmc, median [IQR]	300 (235−505)
CD4^+^ at switch, cells/mmc, median [IQR]	745 (586−943)
CD4/CD8 ratio at switch, median [IQR]	0.95 (0.72−1.21)
Regimen before switch, *n* (%)	
DTG/RPV	19 (33)
DTG/3TC	17 (30)
TAF/FTC/RPV	12 (21)
BIC/TAF/FTC	7 (12)

**P103: Figure 1:**
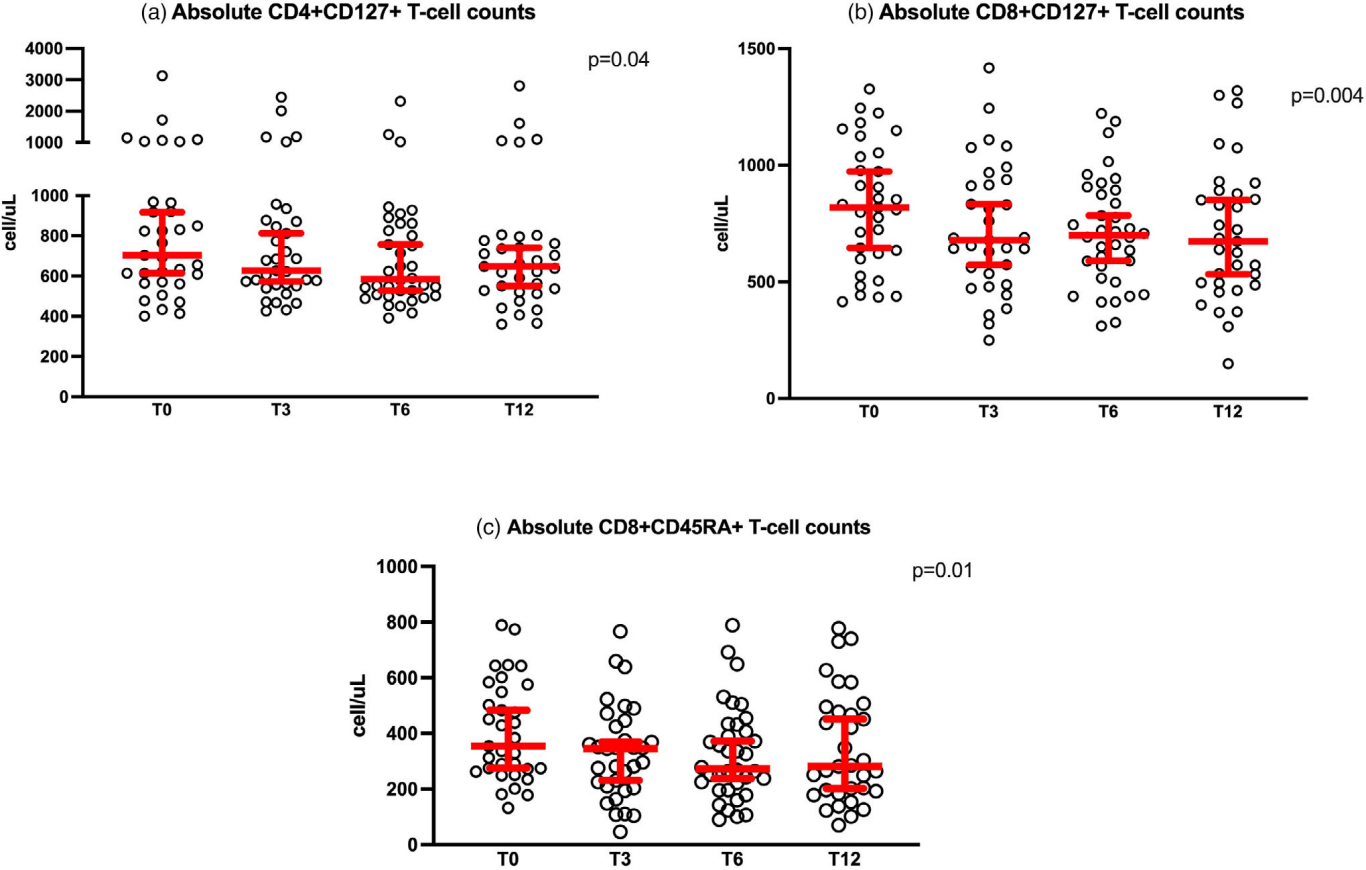
T‐cell homeostasis in PLWH switching to LAI from an oral 2‐DR regimen.

**Conclusions**: Switching to injectable CAB/RPV did not have major effects on T‐cell homeostasis. While central memory CD4^+^ and CD8^+^ T cells as well as naïve CD8^+^ cells show a decreasing trend in individuals switching from oral 2‐DR, the mechanism(s) by which long‐acting injectables may influence T‐cell homeostasis in the long‐term needs careful investigation.

### P104 Switching to long‐acting intramuscular cabotegravir and rilpivirine in virologically suppressed PLHIV treated with dolutegravir/rilpivirine: a substudy from the RELATIVITY cohort

María José Galindo Puerto ^1^, Luis Buzón Martín^2^, Jesús Troya^3^, Luz Martín‐Carbonero^4^, Laura Bermejo Plaza^5^, Miguel Torralba^6^, Carmen Montero Hernández^7^, Miguel Alberto de Zárraga Fernández^8^, Roberto Pedrero Tomé^9^, Noemí Cabello Clotet^10^, Víctor Arenas García^11^, Javier García Abellán^12^, Alfonso Cabello Úbeda^13^, Juan Emilio Losa García^14^, María José Crusells‐Canales^15^, Jara Llenas‐García^16^, Beatriz De la Calle Riaguas^17^, Luis Morano^18^, Maria Aguilera García^19^, Patricia Martin Rico^20^, Enrique Bernal^21^, Sara de la Fuente Moral^22^, Ruth Calderón Hernáiz^23^, María Antonia Sepúlveda^24^, Marouane Menchi^25^, Marta Clavero Olmos^26^, Manuel Gutiérrez Cuadra^27^, Miriam Estebanez^28^, Barbara Alonso Moreno^29^, Álvaro Cecilio^30^, Miguel Egido Murciano^31^, Guillermo Cuevas Tascón^3^, on behalf of the RELATIVITY Multicentre Cohort^32^

^1^Unit of Infectious Diseases, Internal Medicine, Hospital Clínico Universitario Valencia, Valencia, Spain, ^2^Internal Medicine, Hospital de Burgos, Burgos, Spain, ^3^Internal Medicine, Hospital Universitario Infanta Leonor, Madrid, Spain, ^4^Infectious Diseases, Hospital Universitario La Paz, Madrid, Spain, ^5^HIV Unit, University Hospital 12 de Octubre—imas12, Madrid, Spain, ^6^Internal Medicine, Instituto de Investigación en Salud de Castilla‐La Mancha (IDISCAM), Hospital Universitario de Guadalajara, Guadalajara, Spain, ^7^Internal Medicine, Hospital Universitario de Torrejón, Torrejón, Spain, ^8^Internal Medicine, Hospital Universitario San Agustín, Avilés, Spain, ^9^Science Investigation, Fundación para la Investigación e Innovación Biomédica del Hospital Infanta Leonor, Madrid, Spain, ^10^Internal Medicine, Hospital Clínico San Carlos, IdISS, CiberInfec, Universidad Complutense, Madrid, Spain, ^11^Internal Medicine, Hospital de Cabueñes, Unidad de Enfermedades Infecciosas, Gijón, Spain, ^12^Unit of Infectious Diseases, Internal Medicine, Hospital General Universitario, Elche, Spain, ^13^Infectious Diseases, Fundación Jiménez Díaz University Hospital, IIS‐FJD, Universidad Autónoma de Madrid, Madrid, Spain, ^14^Infectious Diseases, Hospital Universitario de Alcorcón, Alcorcón, Spain, ^15^Infectious Diseases, Hospital Clínico Universitario Lozano Blesa, Zaragoza, Spain, ^16^Internal Medicine, Hospital Vega Baja de Orihuela, Universidad Miguel Hernández de Elche, CiberInfec, Instituto de Salud Carlos III, Orihuela, Spain, ^17^Internal Medicine, Hospital General Nuestra Señora del Prado, Talavera de la Reina, Spain, ^18^Infectious Diseases, Hospital Universitario Alvaro Cunqueiro, Vigo, Spain, ^19^Infectious Diseases, Hospital Universitario La Princesa, Madrid, Spain, ^20^Internal Medicine, Hospital de Denia Marina Salud, Denia, Spain, ^21^Infectious Diseases, Hospital Universitario Reina Sofía, Murcia, Spain, ^22^Servicio de Medicina Interna, Hospital Universitario Puerta de Hierro, Unidad de VIH, ITS y PrEP, Majadahonda, Spain, ^23^Internal Medicine, Hospital Universitario de Fuenlabrada, Madrid, Spain, ^24^Internal Medicine, Hospital Universitario de Toledo, Toledo, Spain, ^25^Internal Medicine, Hospital Universitario de Vinalopo, Alicante, Spain, ^26^Internal Medicine, Hospital Universitario Infanta Elena, Madrid, Spain, ^27^Infectious Diseases, Hospital Universitario Marqués de Valdecilla, Santander, Spain, ^28^Infectious Diseases, Hospital Central de la Defensa Gómez Ulla, Madrid, Spain, ^29^Infectious Medicine, Hospital Universitario Doctor José Molina Orosa, Las Palmas, Spain, ^30^Infectious Diseases, Hospital Universitario Miguel Servet, Zaragoza, Spain, ^31^Internal Medicine, Hospital Universitario San Jorge, Huesca, Spain, ^32^Infectious Diseases, Multicentre Cohort, Spain, Spain

**Background**: Cabotegravir and rilpivirine (CAB+RPV) is the first long‐acting injectable (LAI) treatment approved for people living with HIV (PLHIV). It is indicated in patients with undetectable viral load, without evidence or suspected resistance to CAB+RPV. Dolutegravir/rilpivirine (DTG+RPV) is similar to CAB+RPV and has been used in real life as oral lead‐in before starting LAI.

**Materials and methods**: The RELATIVITY cohort is a multicentre, non‐controlled, ambispective study on HIV virologically suppressed individuals who switched to LAI CAB+RPV. We analysed the characteristics of the patients who were on treatment with DTG+RPV prior to the switch. Additionally, patients were compared based on prior knowledge of their genotype. Quantitative variables were contrasted using T‐Student and U‐Mann‐Whitney tests; categorical variables were compared using chi‐square and Fisher's exact tests.

**Results**: A total of 313 individuals from 30 hospitals in Spain were analysed, representing 22.9% of the RELATIVITY cohort, which comprised 1366 individuals. Median follow‐up was 7.9 [IQR 5.2–11.5] months. 83.3% were male. The most common transmission route was GBMSM (57.8%), followed by HTX (24.9%). AIDS prevalence was 11.4%. The most frequent comorbidities were dyslipidaemia (24.3%), hypertension (9.3%) and psychiatric disorders (8.3%). Previous genotyping was available in 44.4% (134/313) of cases: 13/134 presented NNTRI mutations. Patients with known genotype tended to be younger (46.2 [40.0–54.0] vs. 49.8 [40.0–58.0]; *p* = 0.080) and Spanish instead of foreigners (84.3%. vs. 70.7%, *p* = 0.009). Prior virological failure (VF) was more common when previous genotype was available (8.5% vs. 5.5%; *p* = 0.029). Time on ART was similar on both groups (11.0 [8.0–17.0] years on ART). Patients decided to switch regimens for convenience or improve quality of life (52.1%), personal request (28.4%), simplification (25.9%) and malabsorption (8.0%) (more frequent reason [12.7%] among patients with known genotype [*p* = 0.023]). Eleven patients discontinued treatment: two due to systemic adverse effects and only one due to VF (known genotype group).

**Conclusions**: In real‐life settings, switching from DTG+RPV to CAB+RPV is safe and well tolerated. Our results suggest that in virologically suppressed PLHIV under treatment with DTG/RPV, previous genotyping results might not be necessary in order to switch to CAB+RPV.

### P105 Real‐world evidence of B/F/TAF in Chile: effectiveness and safety from the BIKUCH cohort

Alejandro Afani ^1^, Alba Rivas^1^, Camila Maulen^1^, Pedro Zitko^2^

^1^HIV Center, Clinical Hospital University of Chile, Santiago, Chile, ^2^School of Public Health, University of Chile, Santiago, Chile

**Background**: Chile is one of the countries with the highest incidence of HIV in Latin America. Access to ART is guaranteed to all patients living with HIV (PLHIV). B/F/TAF is available in the country since 2020. Our aim was to study the effectiveness, safety, tolerability and adherence of B/F/TAF in a real‐world evidence (RWE) cohort of PLHIV in Chile.

**Methods**: The cohort was conformed with PLHIV in care at the Clinical Hospital, University of Chile (BIKUCH). All PLHIV aged >18 years with B/F/TAF were invited during 2023. Both, treatment naïve (TN) and treatment experienced (TE) PLHIV, were included and followed at 12, 36 and 60 weeks. Viral load (VL), CD4 cell count, other laboratory tests, and safety, adherence and tolerability outcomes were evaluated. Institutional Ethical Committee approved the protocol. Multilevel modelling was implemented.

**Results**: This cohort included 464 PLHIV (TN: 37.5% and TE: 62.5%). Four hundred and forty‐one (98%) male, mean age 37.2 years (18–70.8). In TN, before starting B/F/TAF, the mean of VL was 347,635 (0–4,630,000), 35.9% presented VL >100,000 copies/µl and CD4 cell count was 328.9 (4.3–1383). Likewise in TE, the mean of VL was 3299 (0–502,000), and CD4 cell count was 570.4 (35–1731). Simplification was the main reason of switch in TE (96.8%) from other INSTI use (EVG: 63.8%; RAL: 14.3%; DTG: 10.4%). The most frequent comorbidities were dyslipidaemia (11.4%), hypertension (6.2%) and mental health disorders (4.7%). At week 60, 90% (95% CI 85.5–94.5%) of TN and 92.7% (95% CI 89.7–95.7%) of TE maintained virological suppression (VL <50 copies/µl). CD4 cell count improved in 187.2 cells/µl (95% CI 135.7–239.6) in TN and 70.1 cells/µl (95% CI 24.6–115.3) in TE. No discontinuations due to serious adverse events were registered. Good tolerability was reported by 87.2% (TN: 96.5%; TE: 81.7%). Adherence was 83.3% (TN: 86%; TE: 81.7%). No significant changes were observed in laboratory profiles.

**Conclusions**: BIKUCH is the first RWE of B/F/TAF in Chile. Our results show the high effectiveness and immune recovery over time, both in TN and TE PLHIV. This cohort reinforces a B/F/TAF as an effective and safe option for ART. Our results are comparable to other RWE studies of B/F/TAF.

### P106 Antiretroviral treatment with BIC/FTC/TAF: where we come from and where we are going

Adrian Rodriguez ^1^, Aroa Villoslada^2^, Patricia Sorni^2^, Marta Molero^2^, Antoni Adrover^2^, Ana Andreu^2^, Ana Liebana^2^, Joaquin Serrano^3^, Francisco Homar^2^, Antoni Payeras^2^

^1^Infectious Disease, IdISBa, Hospital Universitari Son Llatzer, Palma, Spain, ^2^Internal Medicine, Infectious Disease, Hospital Universitari Son Llatzer, Palma, Spain, ^3^Pharmacy, Hospital Universitari Son Llatzer, Palma, Spain

**Background**: Since its commercialization in Spain in 2019, bictegravir/emtricitabine/tenofovir alafenamide (BIC/FTC/TAF) has been one of the preferred antiretroviral treatments (ART) in people living with HIV (PLWH). Its advantages include being a single tablet regimen, having a high genetic barrier and being active against hepatitis B virus (HBV). The objective of this study is to analyse the changes in treatment from or to BIC/FTC/TAF, as well as the reasons for these changes.

**Materials and methods**: Data were analysed from all PLWH in University Hospital Son Llatzer, in Mallorca, Spain, who at some point had been treated with BIC/FTC/TAF. We studied the previous ART as well as the subsequent treatment (if any), and the reasons for these changes.

**Results**: Since 2019, 641 PLWH have been treated with BIC/FTC/TAF. There were 21.5% women, and 40% of patients with positive total core antibody for HBV. The majority of patients who started BIC/FTC/TAF were naive patients or transferred from other countries without previous treatment in our hospital (248; 38.7%). There were 181 (28.2%) patients who came from regimens with another integrase inhibitor with cobicistat (EVGc/TAF/FTC) and 98 (15.3%) patients who did not have INSTI in their ART (RPV/TAF/FTC, DRVc/TAF/FTC or EFV/TAF/FTC). There were also changes from two‐pill regimens (77, 12%), and some changes from dual therapies with DTG/3TC or DTG/RPV (18, 2.8%). Of the 641 PLWH treated with BIC/FTC/TAF, 575 (89.7%) maintain this treatment until now. Of the remaining 66 PLWH who switched to other treatments, most of them switched to intramuscular CAB/RPV (24; 36.4%), followed by oral dual therapies such as DTG/3TC (17; 25.8%) or DTG/RPV (4; 6%). The reasons for all changes are summarized in Table 1, with the discontinuation of a booster or the introduction of an integrase inhibitor being the main reasons for initiating BIC/FTC/TAF, and the change in route of administration or simplification being the main reasons for discontinuing BIC/FTC/TAF.

**P106: Table 1:** Reasons of change to or from BIC/FTC/TAF

Changes to BIC/FTC/TAF		
Reason of administration/change	*N*	Percentage
Naive/change of hospital	248	38.7
Eliminate cobicistat	182	28.4
Initiate INSTI	100	15.6
Simplification	77	12.0
Secondary effects	14	2.2
Prevent interaction	8	1.2
Virological failure/blips	5	0.8
Adherence problems/loss follow up	4	0.6
Other	3	0.5
Total	641	

Changes from BIC/FTC/TAF		
Reason of change	*N*	Percentage
Change administration route	24	36.4
Simplification	15	22.7
Secondary effects	12	18.2
Adherence problems/loss follow up	7	10.6
Prevent interaction	2	3.0
Virological failure/blips	2	3.0
Other	4	6.1
Total	66	

*Note*: Results are expressed as number and percentages.

**Conclusions**: Antiretroviral therapy with BIC/FTC/TAF is the most commonly used ART in our hospital, being a safe therapy that is generally maintained over time and suitable for PLWH co‐infected with HBV. The main reason for discontinuation is the participant's desire to switch to intramuscular therapies.

### P107 Patient‐reported outcomes in switching to long‐acting cabotegravir/rilpivirine: a real‐life experience

Anna Carraro ^1^, Raffaella Marocco^1^, Giulia Mancarella^1^, Paola Zuccalà^1^, Lorenzo Ansaldo^1^, Sara De Maria^1^, Sara Corazza^1^, Alessandra Grimaldi^1^, Andrea Gasperin^1^, Cosmo Del Borgo^1^, Miriam Lichtner^2^

^1^Infectious Diseases Unit, SM Goretti Hospital, Sapienza University of Rome, Department of Public Health and Infectious Diseases, Latina, Italy, ^2^Università Sapienza Roma, Dipartimento di Neuroscienze Salute Mentale e Organi di Senso (NESMOS), Roma, Italy

**Background**: There is an increasing focus on the patient's overall health which includes sexual health, mental health and social wellbeing. PROMs (Patient‐reported outcome measures) are tools that help to assess the overall health of the person living with HIV (PLWH).

**Materials and methods**: This is a real‐life experience where we offered several PROMs to PLWH at the time of long‐acting cabotegravir/rilpivirine (LA‐CAB/RPV) starting and 6 months after, in Outpatient HIV Clinic‐S.M. Goretti Hospital in Latina (Italy); here, we will analyse some of them: HIV Dependent Quality of Life (HIVDQoL), HIV Stigma Scale (HSS), HIV Treatment Satisfaction Questionnaire status version (HIVTSQs), HIV Treatment Satisfaction Questionnaire change version (HIVTSQc), Perception of Injection (PIN).

**Results**: Thirty‐six patients have joined it, via own mobile phone; 11 assigned female at birth (30.5%), with a median age of 47 years (range 29–68), 11% with a AIDS history; pre‐switch treatment were: DTG/3TC (27.8%), RPV/TAF/FTC (25%), BIC/TAF/FTC (22.2%), DTG/RPV (11%), DRV/cobi/TAF/FTC (5.6%), TDF/FTC + RAL (2.8%), TDF/FTC/ABC (2.8%), DRV+RTV+3TC+ETV (2.8%). After switching to LA CAB/RPV, the feeling towards own life changes: more people feel to have a great or very good quality of life (T6 88% vs. T0 33%, *p* = 0.01); there is an increase in the number of people who feel own quality of life would be the same if they did not have HIV (T6 44% vs. T0 22%, *p* = 0.38) (Figure 1). Switching to LA CAB/RPV reduces efforts to keep own serostatus hidden (T0 87.5% vs. T6 33%, *p* = 0.009) and reduces the self‐perception that PLWH are more marginalized (T0 73% vs. T6 50%). Comparing the treatment satisfaction (status version vs. change version), 83% expressed the highest satisfaction to intramuscular therapy (73% before switch). Concerning the commitment involved intaking therapy, the highest satisfaction score was expressed by 91% at T6 (before switch only 27%, *p* = 0.008). Sixty‐six percent described CAB/RPV as convenient (vs. 31% at T0). Injection pain is perceived by most patients, but this decreases over time (at the first injection 78%; at the fifth injection 60% with only mild‐to‐moderate pain, *p* = 0.39); pre‐dose anxiety and worry also decrease (T1 35% vs. T6 10%, *p* = 0.34). Everyone decides to continue therapy despite pain.

**P107: Figure 1:**
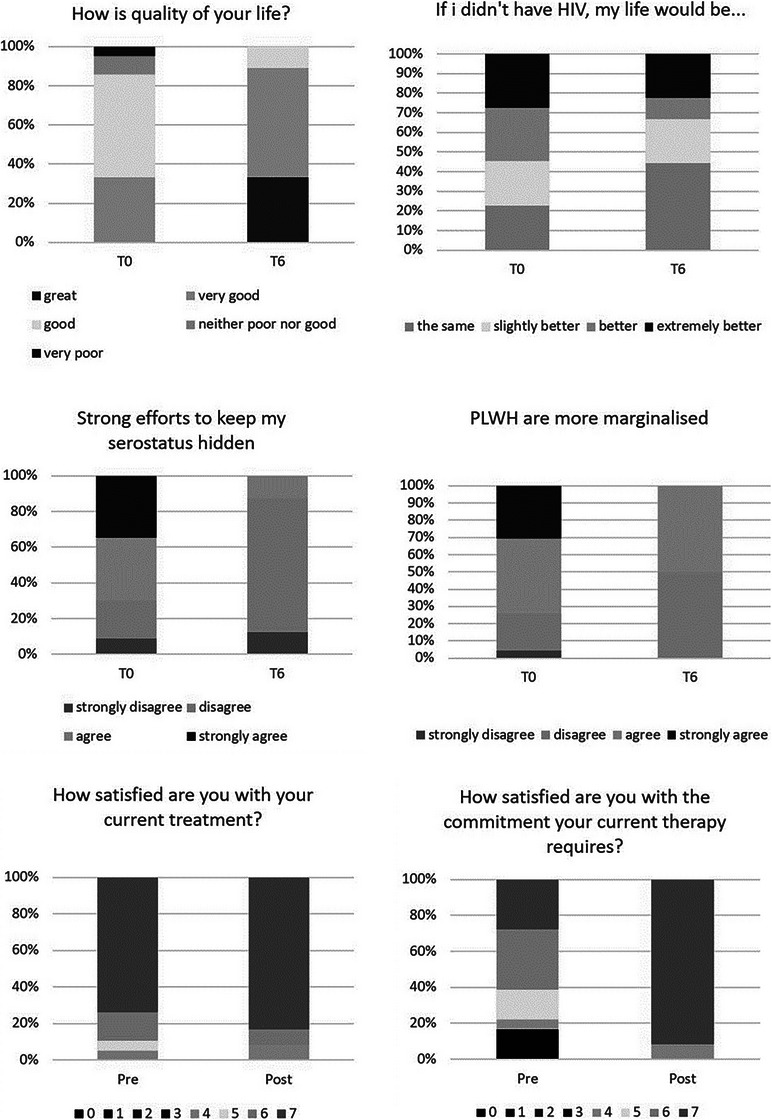
Graphs relating to some specific issues.

**Conclusions**: For patients who agree to the therapeutic switch, CAB/RPV LA is a well‐tolerated therapy that improves quality of life and own relationship with the infection.

### P108 The analysis of metabolic parameters in people living with HIV using dolutegravir‐based regimens in routine clinical practice in Russia

Anna Basova ^1^, Stella Minaeva^2^, Natalia Sizova^3^, Firaya Nagimova^4^, Elena Orlova‐Morozova^5^, Margarita Radzikhovskaya^6^, Valeriy Shevchenko^7^, Andrés Maldonado‐Doblado^8^, Bryn Jones^9^

^1^Medical Affairs, GlaxoSmithKline, Moscow, Russian Federation, ^2^Infectious Diseases, Nizhny Novgorod Regional Centre for AIDS and Infectious Diseases Prophylaxis and Control, Nizhny Novgorod, Russian Federation, ^3^Infectious Diseases, Centre for AIDS and Infectious Diseases Prophylaxis and Control, St Petersburg, Russian Federation, ^4^Infectious Diseases, Tatarstan Republican Centre for AIDS and Infectious Diseases Prophylaxis and Control, Kazan, Russian Federation, ^5^Infectious Diseases, Moscow Regional Centre for AIDS and Infectious Diseases Prophylaxis and Control, Moscow, Russian Federation, ^6^Infectious Diseases, Regional Centre for AIDS and Infectious Diseases Prophylaxis and Control, Chelyabinsk, Russian Federation, ^7^Infectious Diseases, Altai Regional Centre for AIDS and Infectious Diseases Prophylaxis and Control, Barnaul, Russian Federation, ^8^Medical Affairs, ViiV Healthcare, Wavre, Belgium, ^9^Medical Affairs, ViiV Healthcare, Brentford, UK

**Background**: Long‐term effects of antiretroviral therapy (ART) on metabolic health of people living with HIV (PLHIV) require further assessment through real‐world evidence. Here, we present the results of a 24‐month observation of body weight, metabolic parameters and cardiometabolic events in PLHIV receiving dolutegravir‐based regimens (DBRs), as part of an interim analysis of the prospective, real‐world data, 3‐year TESLA study in Russia.

**Materials and methods**: Overall 959 adult PLHIV were included. By 24 months, 258 participants received the two‐drug regimen (dolutegravir+lamivudine; sub‐population 2DR): 3.9% ART‐naïve, 96.1% ART‐experienced; 621 received a three‐drug regimen (DTG+2 other drugs; sub‐population 3DR): 24.8% ART‐naïve, 75.2% ART‐experienced. Metabolic parameters included blood glucose, ALT, AST, TC, TG, LDL and HDL as well as body weight and BMI.

**Results**: At 24 months in the 3DR sub‐population, 79.4% (*n* = 100) of ART‐naïve and 76.4% (*n* = 323) of ART‐experienced had HIV‐RNA <50 copies/ml. In receiving 2DR, this parameter was reached in 90% (*n* = 9) and 83.3% (*n* = 200), respectively. Overall, at 24 months, body weight was assessed for 85.3%; mean body weight and BMI values slightly increased in both sub‐populations (Table 1). Body weight increase was reported as adverse drug reaction (ADR) for 30 (11.6%) participants in sub‐population 2DR, for 33 (5.3%) participants in 3DR. For blood biochemistry parameters, data for 24‐month observation were available for 75.9% of participants for glucose, 82.2% and 82.0% of population had data on ALT and AST, respectively, 61.6% had TC data, 34.1% had TG data, 20.7% and 20.0% had data on LDL and HDL, respectively. In both 2DR and 3DR sub‐populations, the proportions of participants with clinically significant abnormalities in the above blood parameters did not exceed baseline values, with exception for LDL and HDL in sub‐population 3DR (Table 1). Cardiovascular or metabolic serious adverse events (SAEs)/ADRs reported were myocarditis in sub‐population 2DR in one (0.4%) participant; acute left ventricular failure, cardiopulmonary failure, diabetes mellitus, diabetic ketoacidosis, deep vein thrombosis, shock, hypertensive crisis, one participant (0.2%) each, in sub‐population 3DR.

**P108: Table 1:** Metabolic parameters in sub‐populations 3DR and 2DR of PLHIV receiving DBRs at 24‐month observation in TESLA study

Sub‐population	2DR (*N* = 258)		3DR (*N* = 621)	
Study visit	Baseline	24 months	Baseline	24 months
Body weight, kg (M±SD)	73.1±14.6	75.6±15.3	71.8±14.2	74.1±14.7
BMI, kg/m^2^ (M±SD)	24.5±4.2	25.2±4.2	24.2±4.1	24.9±4.4
Participants with clinically significant abnormalities, *n* (%)^a^				
Blood glucose	0	0	3 (0.5%)	1 (0.2%)
TC	20 (7.8%)	6 (2.3%)	34 (5.5%)	15 (2.4%)
TG	5 (1.9%)	3 (1.2%)	19 (3.1%)	8 (1.3%)
LDL	12 (4.7%)	1 (0.4%)	4 (0.6%)	6 (1.0%)
HDL	5 (1.9%)	1 (0.4%)	1 (0.2%)	8 (1.2%)
ALT	5 (1.9%)	0	34 (5.5%)	6 (1.0%)
AST	2 (0.8%)	0	30 (4.8%)	7 (1.1%)

Abbreviations: 2DR, two‐drug regimen; 3DR, three‐drug regimen; ALT, alanine aminotransferase; AST, aspartate aminotransferase; DBR, dolutegravir‐based regimen; DTG, dolutegravir; HDL, high‐density lipoprotein; LDL, low‐density lipoprotein; M±SD, mean ± standard deviation; *n* (%), number of participants (% of sub‐population); TC, total cholesterol; TG, triglycerides.

^a^Subjective assessment was used based on the experience of expert doctors.

**Conclusions**: Interim 24‐month observation data do not suggest negative effect of DBRs on metabolic health of PLHIV; body weight changes should be discussed in the context of obesogenic environment.

### P109 Switching from triple therapy to DTG/3TC in HIV‐1 infected migrants without previous resistance test results

Frederico Duarte, Clara Bacelar, Eduarda Pena, Fábio Reis, Clara Batista, Susana Oliveira, Sofia Jordão, Maria João Gonçalves, Sónia Rocha, Cristina Soeiro, Ricardo Correia de Abreu

Infectious Diseases, Unidade Local de Saúde de Matosinhos, Matosinhos, Portugal

**Background**: Some clinicians still express concerns about DTG/3TC combination's robustness, particularly in patients on triple therapy without prior resistance test, due to the possibility of monotherapy in the presence of nucleoside mutations.

**Methods**: To retrospectively evaluate the immunovirological response of switching from triple therapy to DTG/3TC in migrants living with HIV‐1, who began regular follow‐up between 2022 and 2023.

**Results**: From a total of 161 new patients with HIV‐1, 74 were treatment‐naive and 87 were migrants (69 Brazil, seven Argentina, five USA, four Venezuela, two Spain) already on treatment. From these, 81 did not have resistance test results, 52 were on TDF/XTC+DTG (three hepatitis B co‐infected, one hepatitis C). Forty‐seven monoinfected patients switched to DTG/3TC after confirming virological suppression under the previous triple regimen. Forty‐two were men, average age of 36 years (21–67) and infection duration around 9 years (2–26). At switch, their average weight was 76 kg (55–120 kg), their BMI was 25 (18–37), absolute CD4 count of 705 cells/µl (177–1518), a %CD4 of 35 (14–43) and CD4/CD8 ratio of 0.96 (0.26–2.09). After an average of 56 weeks (6–97) of follow‐up on DTG/3TC, their average weight was 78 kg (58–119 kg), BMI of 25.6 (17–37), absolute CD4 count of 792 cells/µl (356–1518), %CD4 of 34 (16–50%), CD4/CD8 ratio of 1.02 (0.29–2.06), 100% had an HIV‐1 RNA <20 copies/ml.

**Conclusions**: After switching from triple therapy to DTG/3TC, and more than a year of follow‐up, this diverse migrant population, with a long duration of infection and potentially significant previous therapeutic variability, showed no treatment discontinuations; minimal impact on average weight and BMI; immunological status was maintained, with an increase in absolute CD4 count and CD4/CD8 ratio; and a sustained virological suppression in all patients. The switch to DTG/3TC, a regimen with potentially lower long‐term toxicity and few drug interactions, proved to be a solid option.

### P110 Real‐world experience with 3TC+DTG in low‐resource setting in Latin America

Jimena Lopez Pineiro^1^, Karla Bendezu Mejía^1^, Juan Pablo Stagnaro ^2^

^1^Consultorios Externos Vespertino, Hospital Muñiz, Buenos Aires, Argentina, ^2^Centro Universitario de Microbiología y Parasitología, Universidad Nacional de La Plata, La Plata, Argentina

**Background**: Dual therapy with DTG+3TC is an option for switching ART in PLWH with undetectable viral load (uVL) and without known mutations associated with resistance to 3TC or DTG. Access to resistance testing is limited or unavailable in low‐resource settings.

**Objective**: To analyse the time to virological failure after switching to DTG+3TC without prior resistance testing (RT) in a cohort of suppressed PLWH.

**Materials and methods**: Data from a cohort of suppressed PLWH, who switched to DTG+3TC without baselined RT in a public hospital in Argentina, was analysed. Data was collected from the national HIV database and anonymized before analysis. Sex, age at diagnosis, previous ART, CD4 previous and post‐switch, VL during follow‐up and time between diagnosis and switch were evaluated. Pre‐ and post‐switch VL (3 and 6 months) were compared with the McNemar test; categoric data resumed by percentage; numeric data by mean and SD or median and IQR, and Spearman rho; *p* = 0.05. Time to virologic failure was analysed using Kaplan‐Meier (KM) survival analysis and compared with log rank test according to previous antiretrovirals.

**Results**: *N* = 98 PLWH uVL, 100% with previous exposure to 3TC or FTC. Females 41 (42.3%), males 56 (57.7%).  Table 1 shows the baseline characteristics of the PLWH. Previous ART: TDF xTC DTG: 48 (51%), TDF xTC EFV: 16 (17.2%), TDF xTC DRVr: 10 (10.6%). Figure 1 shows KM survival analyses. Ninety‐two PLWH were followed up 2415 months. Incidence density: 0.00083 detectable viral load (dVL)/month. Undetectable VL pre‐switch 100% and uVL post‐switch 96.7%, McNemar test = 3, *p* = 0.083, indicating that there is no difference between the proportion of PLWH with uVL before and after bitherapy (BT). Of the 3/93 dVL postBT, two PLWH VL 51 and 43 copies and one PLWH with 373 copies/ml with and INI resistance study in progress. CD4 pre‐switch 650 cells/ml (IQR 462–852), CD4 post‐switch 731 cells/ml (IQR 584–1042), Spearman rho = 0.78, *p* = 0.000.

**P110: Table 1:** Baseline characteristics of the PLWH under bitherapy

	*N* (%)
Age (mean and SD)	36.6 (10.6)
Years between diagnostic and BT, median (IQR)	9.9 (IQR 4.8−14.8)
Prior INI	63 (63.6)
Prior NNRTI	19 (19.1)
Prior boosted PI	15 (15.1)
Undetectable viral load preBT	94 (94.5)
CD4, median (IQR) cells/ml	650 (IQR 462−852)

**P110: Figure 1:**
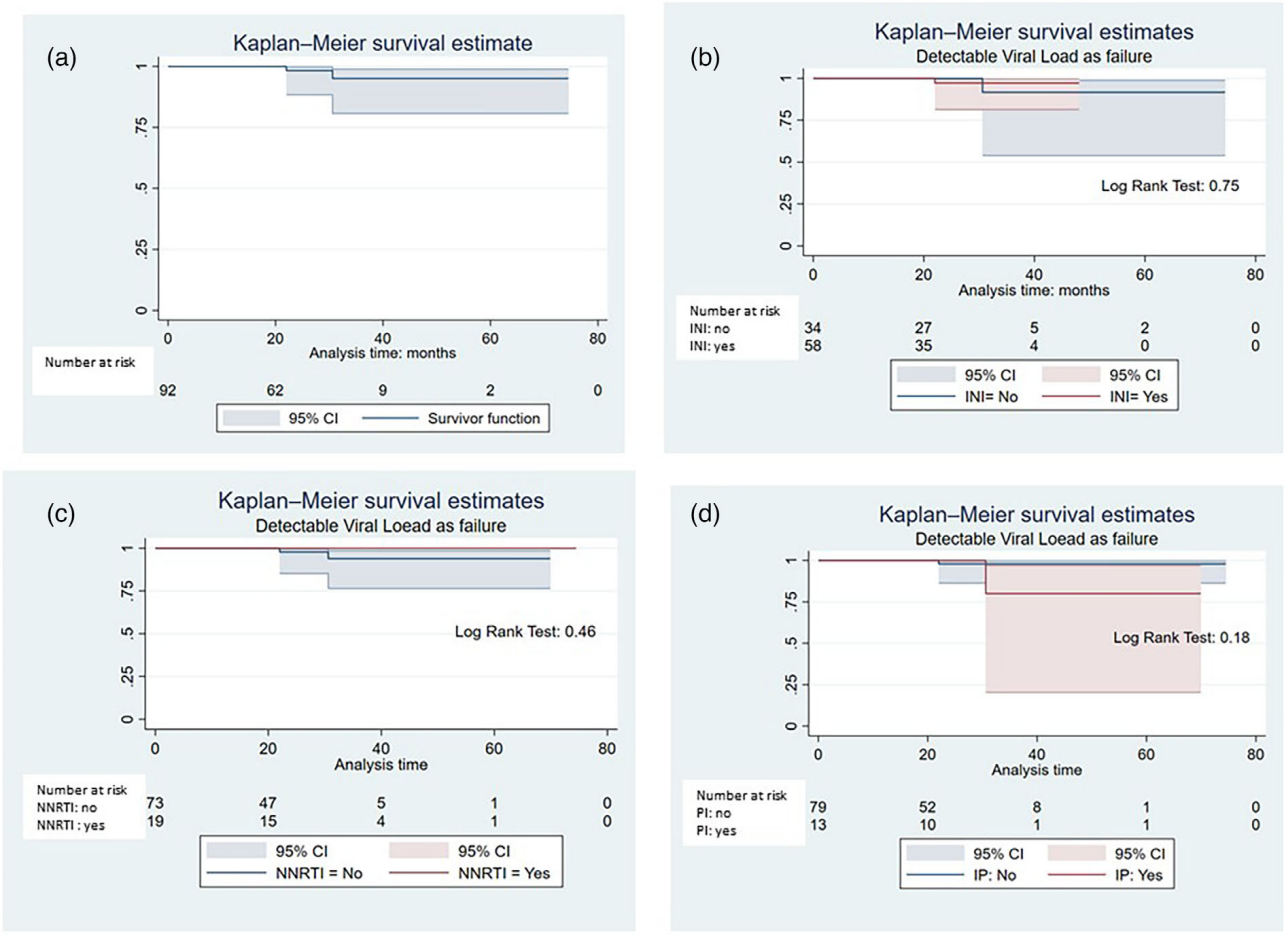
Survival analysis. Model failure: detectable viral load; follow up in months. Overall (a) and previous ART: integrase inhibitors (INIs) (b), non‐nucleoside inhibitors (NNRTIs) (c) and protease inhibitors (PIs) (d).

**Conclusions**: Dual therapy without prior resistance testing may be an alternative in suppressed PLWH without previous history of treatment failure. Data from controlled clinical trials is needed to further support this strategy.

### P111 Treatment adherence and virological suppression in an outpatient cohort of people living with HIV (PLWH) on long‐acting injectable HIV therapy

Catharina Dannenberg ^1^, Maher Almahfoud^1^, Lukas Weimann^1^, Hanna Matthews^1^, Olaf Degen^1^, Julian Schulze zur Wiesch^2^

^1^Outpatient Center—Department of Infectious Diseases, University Medical Center Hamburg‐Eppendorf, Hamburg, Germany, ^2^Division of Infectious Diseases, First Department of Medicine, University Medical Center Hamburg‐Eppendorf, Hamburg, Germany

**Background**: The HIV epidemic remains a significant global challenge, with long‐term viral suppression in people living with HIV (PLWH) crucial to reducing transmission rates. The introduction of long‐acting injectables cabotegravir/rilpivirine (LA‐CAB/RPV) offers potential solutions to adherence issues and stigmatization‐related non‐compliance. However, the impact of LA‐CAB/RPV on adherence and virological suppression in PLWH outside clinical trials has been little studied.

**Materials and methods**: Retrospective observation of patients who received LA‐CAB/RPV at our Infectious Diseases Outpatient Center in Hamburg‐Eppendorf, Germany between 05/2021 and 04/2024. We collected data on demographics, treatment adherence and treatment success. All adult patients who consented and received at least one dose of LA‐CAB/RPV were included, regardless of virological suppression.

**Results**: One hundred and six participants were included: 76 (71.7%) received in‐label treatment, nine (8.5%) off‐label and 21 (19.8%) started LA‐CAB/RPV as part of a phase IIIb drug trial before clinical routine. Median age: 49.2 (23–78) years; 81% male patients, most born in Germany (68.9%) and sub‐Saharan Africa (9.4%). HIV diagnosis was median 9.9 (0–33) years prior to the first injection, with 8.7 (0–27) years on antiretroviral therapy beforehand. 80.2% received integrase‐containing regimes before switching. During the observation period, 1051 injections were administered to the in‐label group, averaging 13.8 (1–19) injections per patient over a therapy duration of 26.1 (1–35) months. Nine hundred and seventeen (87.3%) injections were administered on time, while 134 (12.7%) were delayed with a median delay of 3.0 (1–53) days, 88.8% delayed within 7 days. All patients with a delay >7 days showed viral load <50 cop/ml. Virological suppression rates were similar between on‐time and delayed injections: 2.0% and 2.9% showed viral loads >50 cop/ml, respectively, and 0.4% and 1.0% had viral loads >200 cop/ml. 94.7% of all patients maintained virological suppression (<200 cop/ml) during observation. 18.4% switched back to oral therapy.

**Conclusions**: In this diverse cohort of 106 PLWH on LA‐CAB/RPV, we observed high rates of virological suppression. Minor delays in injection schedules did not significantly impact virological outcomes, suggesting flexibility that could benefit adherence. These findings highlight the potential of LA‐CAB/RPV to address adherence challenges and reduce stigmatization, supporting long‐term viral suppression in real‐world settings.

### P112 Therapy satisfaction and preference for injectable long‐term options of Portuguese HIV patients: a multicentric case‐study

Cristina Valente ^1^, Ruben Carvalho^1^, Catia Caldas^2^, Carmela Pineiro^2^, Daniel Coutinho^3^, Sofia Nunes^3^, Sara Lino^4^, Fernando Maltez^4^, Raquel Pinho^5^, Josefina Mendez^6^, João Matos^6^, Helder Pinheiro^7^, Inês Vaz Pinto^7^, Frederico Duarte^8^, Ricardo Correia de Abreu^8^, Ricardo Racha‐Pacheco^9^

^1^Infectious Diseases, Unidade Local de Saúde (ULS) Coimbra, Coimbra, Portugal, ^2^Infectious Diseases, Unidade Local de Saúde (ULS) São João, Porto, Portugal, ^3^Infectious Diseases, Unidade Local de Saúde (ULS) Gaia e Espinho, Gaia, Portugal, ^4^Infectious Diseases, Unidade Local de Saúde (ULS) São José, Lisboa, Portugal, ^5^Internal Medicine, Unidade Local de Saúde (ULS) Algarve‐ H.Portimão, Coimbra, Portugal, ^6^Infectious Diseases, Unidade Local de Saúde (ULS) Santo António, Porto, Portugal, ^7^Infectious Diseases, Hospital de Cascais, Lisboa, Portugal, ^8^Infectious Diseases, Unidade Local de Saúde (ULS) Matosinhos, Matosinhos, Portugal, ^9^Q2Science, Lisboa, Portugal

**Background**: Recent innovations in the field of antiretroviral therapy (ART) have positively impacted the health outcomes and quality of life (QoL) of people living with HIV (PLHIV), but the latter is not yet equivalent to that of the normal population. Cabotegravir‐rilpivirine (CAB/RPV) became the first approved long‐acting injectable ART in 2021. This innovation could improve the experience of PLHIV through greater convenience, privacy and medication management. Our interventional multicentric study aimed to generate national evidence on the level of satisfaction of Portuguese PLHIV with their current ART regimens, and explore their demographics, psychosocial challenges and preference for changing to injectable long‐term ART.

**Methods**: PLHIV from eight Portuguese centres were enrolled in this study. Data collection was performed through consultation of clinical files and a self‐completion questionnaire (both on paper or digital format). The questionnaire was created specifically for this study and contemplated demographic and HIV infection characterization components as well as a section ascertaining the participants satisfaction, challenges and preferences regarding ART. Informed consent was obtained.

**Results**: A total of 442 PLHIV participated in this study. The average age was 48.1 years and 75% of participants had been receiving ART for less than or exactly 5 years. Most individuals were Portuguese (74%) and 82% were infected through sexual transmission. A high level of satisfaction (4.4/5) with daily oral therapy was found, but 94% of participants stated to face at least one challenge. Older age and longer time on ART associated with higher satisfaction and less reporting of challenges (i.e. ART being an uncomfortable reminder of their HIV infection, and concerns about forgetting the medication). Overall, 59% of the enrolled PLHIV expressed a preference for changing to the injectable long‐acting regimen. This percentage was 73% for those with lower average satisfaction score (1–3/5). The preference for changing was associated with younger age and less time on ART, as well as with lower levels of satisfaction.

**Conclusions**: PLHIV from Portugal have a high degree of satisfaction with their daily oral therapy but still face some challenges, with a majority expressing a preference for changing to an injectable long‐acting ART regimen.

### P113 T‐cell homeostasis and microbial translocation in PLWH switching from triple to dual INSTI‐based combination antiretroviral therapy (cART)

Valeria Bono, Camilla Tincati, Matteo Augello, Roberta Rovito, Sabrina Marozin, Andrea Santoro, Giulia Marchetti

Clinic of Infectious Diseases, Department of Health Sciences, ASST Santi Paolo e Carlo, University of Milan, Milan, Italy

**Background**: Dual cART regimens containing a second‐generation INSTI are used in both first‐line and switch strategies. While effective in viro‐immunological control, their impact on immune dysregulation and microbial translocation remains unclear.

**Materials and methods**: PLWH switching from a three drug (3DR), viro‐suppressive strategy to a dual drug (2DR) regimen with pre/post‐switch plasma were studied. We measured T‐cell maturation (CD127/CD45RA) and activation (CD38/CD45R0) by flow cytometry, gut barrier dysfunction (E‐cadherin, I‐FABP) and microbial translocation (sCD14, LBP) by ELISA. Statistics: Wilcoxon test.

**Results**: We enrolled 60 PLWH on virally effective long‐term cART (Table 1). Immune parameters were measured at a median of 11 (8–14.7) months from switch to 2DR. Switching to 2DR led to significant increases in CD4 counts (CD4: 32% [27–39] vs. 35% [30–40], *p* = 0.0001; CD4: 701 cells/µl [543–937] vs. 747 [607–1026], *p* = 0.03), reduction in CD8 lymphocytes (CD8%: 40% [34–45] vs. 37% [30–42], *p* < 0.0001; CD8: 868 cells/µl [681–1141] vs. 748 [607–1029], *p* = 0.01) and rise in CD4/CD8 (0.83 [0.65–0.91] vs. 0.96 [0.76–1.3], *p* = 0.0007). Additionally, switch to 2DR resulted in a decrease of percentage and absolute naïve T cells (CD4/CD45RA: 11% [8–17] vs. 10% [6–13], *p* < 0.0001; 241 cells/µl [152–328] vs. 185 [122–338], *p* = 0.001; CD8/CD45RA: 18% [15–22] vs. 17% [12–20], *p* = 0.002; 409 cells/µl [282–511] vs. 327 [245–524], *p* = 0.03) as well as a significant expansion of central memory phenotypes (CD4/CD127: 28% [24–33] vs. 32% [26–37], *p* < 0.0001; 608 cells/µl [477–835] vs. 687 [552–861], *p* = 0.01; CD8/CD127: 30% [24–34] vs. 32% [26–36], *p* = 0.01). Furthermore, we also observed a marked reduction in activation phenotypes (CD8/CD38: 5% [4.8] vs. 3% [2.5], *p* < 0.0001; 122 cells/µl [90–194] vs. 72 [39–109], *p* < 0.0001; CD8/CD45R0: 7% [4–11] vs. 6 [3–8], *p* = 0.03); (CD8/CD38/CD45R0: 1% [0–1] vs. 0% [0–1], *p* < 0.0001: 17 cells/µl [11–26] vs. 9 [5–14], *p* < 0.0001). No changes in markers of intestinal damage and microbial translocation were detected prior to and after the switch.

**P113: Table 1:** Demographic and clinical features of the study population (*n* = 60) on suppressive 3DR (pre‐switch)

Age, years, median (IQR)	48 (42−58)
Sex male, *n* (%)	50 (83.33%)
Risk factors for HIV infection MSM, *n* (%)	40 (66.66%)
Risk factors for HIV infection heterosexual, *n* (%)	17 (28.33%)
Time since HIV diagnosis (years), median (IQR)	14 (10−18)
Time on cART (years), median (IQR)	13 (10−18)
Time from switch (months), median (IQR)	11 (8−14.7)
CD4 cell count nadir (cells/mm^3^), median (IQR)	275 (182.5−407.5)
HIV RNA (copies/ml) pre‐switch, median (IQR)	0 (0−0)
Total time with viral load <50 copies/ml (years), median (IQR)	9 (7−9)
Past AIDS‐defining events (CDC C), *n* (%)	9 (15%)
Number of cART lines prior to switch 0−3, *n* (%)	47 (78.33%)
Number of cART lines prior to switch 4−8, *n* (%)	11 (18.33%)
Pre‐switch 3DR cART INSTI‐based, *n* (%)	41 (68.33%)
Pre‐switch 3DR cART NNRTI‐based, *n* (%)	14 (23.33%)
Post‐switch 2DR cART 3TC/DTG, *n* (%)	53 (88.33%)
Post‐switch 2DR cART DTG/RPV, *n* (%)	7 (11.66%)

Abbreviations: 2DR, two‐drug regimen; 3DR, three‐drug regimen.

**Conclusions**: Switching from 3DR to 2DR appears to improve T‐cell homeostasis through the increase of central memory cells and reduction of T‐cell activation, suggesting a possible effect also on peripheral T‐cell function. In contrast, the stable gut barrier/function and microbial translocation markers suggest no effect of dual cART on gastrointestinal permeability.

### P114 HIV‐1 viral decay in blood and semen in antiretroviral‐naive adults initiating dolutegravir/lamivudine versus bictegravir/emtricitabine/tenofovir alafenamide

Ran Wang ^1^, Yongjian Liu^2^, Lijun Sun^1^, Aixin Li^1^, Shiyun Lv^1^, Yuanyi Zhai^1^, Rui Li^1^, Wei Hua^1^, Xi Wang^1^, Lin Li^2^, Lilli Dai^1^

^1^Center for Infectious Diseases, Beijing Youan Hospital, Capital Medical University, Beijing, China, ^2^State Key Laboratory of Pathogen and Biosecurity, Academy of Military Medical Sciences, Beijing, China

**Background: **Despite the dolutegravir/lamivudine (DTG/3TC) regimen has been recommended as first‐line antiretroviral therapy (ART) regimen for ART‐naïve people with HIV (PWH) by guidelines, the discussion on its efficacy, particularly regarding the viral decay and inflammatory changes in body fluids, compared with three‐drug regimens is still ongoing. This study aimed to compare the decline in HIV‐1 RNA in blood and semen, and differences in inflammatory biomarkers changes among ART‐naïve PWH treated with DTG/3TC versus bictegravir/emtricitabine/tenofovir alafenamide (BIC/FTC/TAF), within a real‐world clinical setting.

**Materials and methods: **This real‐world study conducted in HIV/AIDS Clinic of Capital Medical University‐affiliated Beijing Youan Hospital. Eligible participants were antiretroviral‐naïve men who have sex with men (MSM), aged 18 years or older, who had a baseline plasma HIV‐1 RNA >1000 copies/ml. Participants initiated once‐daily ART with either BIC/FTC/TAF or DTG/3TC between May 2021 and September 2022. The study visits were scheduled at baseline, and weeks 4, 12, 24 and 48. HIV‐1 RNA was measured (Abbott Diagnostics Inc, USA) in both blood plasma (BP) and seminal plasma (SP). The levels of eight soluble factors were determined using the ELLA microfluidic analyser. Generalized estimating equations (GEEs) were applied to assess longitudinal trends.

**Results: **Among the 100 patients in this study, 96 participants completed the 48‐week follow‐up (BIC/FTC/TAF, *n* = 56; DTG/3TC, *n* = 40) (Table 1). Viral suppression rates in both BP and SP were comparable between the BIC/FTC/TAF and DTG/3TC groups in per‐protocol analyses at week 48 (BP, 96.4% vs. 100%, *p* = 0.519; SP, 100% vs. 100%, *p* > 0.999). Both regimens demonstrated similar effectiveness in reducing HIV‐1 RNA levels in blood (3.0 vs. 3.1 log10 copies/ml) and seminal plasma (0.9 vs. 1.2 log10 copies/ml). There were no statistically significant differences in the changes of inflammatory biomarkers over the 48‐week follow‐up.

**P114: Table 1:** Baseline characteristics of participants who completed the 48‐week follow‐up in both groups

Characteristic	BIC/FTC/TAF (*n* = 56)	DTG/3TC (*n* = 40)	*p*‐value
Age, median (IQR), year	32 (28−39)	32 (29−35)	0.963
Race (Han), *n* (%)	49 (87.5)	38 (95.0)	0.375
RPR‐positive, *n* (%)	22 (39.3)	15 (37.5)	0.859
BP HIV‐1 RNA, median (IQR), log10 copies/ml	4.3 (3.8−5.0)	4.3 (3.7−4.7)	0.869
>5 log10 copies/ml, *n* (%)	13 (23.2)	10 (25.0)	0.840
SP HIV‐1 RNA, median (IQR), log10 copies/ml	2.5 (1.6−4.4)	1.8 (1.6−3.2)	0.415
CD4 count, median (IQR), cells/µl	318 (218−423)	330 (237−434)	0.638
CD4/CD8 ratio, median (IQR)	0.3 (0.2−0.4)	0.3 (0.2−0.4)	0.734

*Note*: Data are presented as median [IQR] unless otherwise specified.

Abbreviations: 3TC, lamivudine; BIC, bictegravir; BP, blood plasma; DTG, dolutegravir; FTC, emtricitabine; IQR, interquartile range; PBMCs, peripheral blood mononuclear cells; RPR, rapid plasma reagin; SP, seminal plasma; TAF, tenofovir alafenamide.

**Conclusions: **These findings suggest comparable effectiveness of DTG/3TC versus BIC/FTC/TAF in achieving viral suppression in both BP and SP and similar changes in inflammatory biomarkers in blood plasma.

### Treatment strategies—Treatment experienced adults (second‐line and multi‐drug resistance studies)

### P115 Simplification of complex antiretroviral treatment regimens to two‐class therapies in people with HIV

Christine Baumgartner ^1^, Dominique Laurent Braun^2^, Huldrych Günthard^3^, Roger Kouyos^3^, Alexandra Calmy^4^, Catia Marzolini^5^, Matthias Cavassini^6^, Enos Bernasconi^7^, Patrick Schmid^8^, Gilles Wandeler^9^, Andri Rauch^1^, Bernard Surial^1^, Anna Hachfeld^1^

^1^Department of Infectious Diseases, Inselspital, Bern University Hospital, University of Bern, Bern, Switzerland. ^2^Department of Infectious Diseases and Hospital Epidemiology, University Hospital Zurich, University of Zurich, Zurich, Switzerland. ^3^Department of Infectious Diseases and Hospital Epidemiology, University Hospital Zurich, University of Zurich, and Institute of Medical Virology, Zurich, Switzerland. ^4^Division of Infectious Diseases, University Hospital Geneva, University of Geneva, Geneva, Switzerland. ^5^Division of Infectious Diseases and Hospital Epidemiology, University Hospital Basel and University of Basel, Service of Clinical Pharmacology, Department of Laboratory Medicine and Pathology, University of Lausanne, Basel, Switzerland. ^6^Department of Infectious Diseases, University Hospital of Lausanne, Lausanne, Switzerland. ^7^Division of Infectious Diseases, Ente Ospedaliero Cantonale Lugano, University of Geneva and University of Southern Switzerland, Lugano, Switzerland. ^8^Division of Infectious Diseases, Cantonal Hospital of St Gallen, St. Gallen, Switzerland. ^9^Department of Infectious Diseases Bern University Hospital, University of Bern and Institute of Social and Preventive Medicine, Bern, Switzerland

**Background**: Complex antiretroviral treatment (ART) regimens with ≥3 drug classes compared to two‐class regimens increase the risk for toxicity and drug‐drug interactions in people with HIV. We aimed to describe the population receiving ≥3 drug classes after the introduction of integrase strand transfer inhibitor (INSTI)‐based single tablets, assess the proportion who switched to any two‐class regimen and explore predictors of switching.

**Materials and methods**: Participants from the Swiss HIV Cohort Study on ART containing ≥3 drug classes and with follow‐up from 11/2013 to 11/2023 were included. Drug classes included nucleoside/nucleotide reverse transcriptase inhibitors, non‐nucleoside reverse transcriptase inhibitors, protease inhibitors, INSTIs or entry inhibitors. Switchers were participants who had their ≥3‐class regimen replaced by a regimen containing any two drug classes. Non‐switchers were individuals who continued ART containing ≥3 drug classes. We compared characteristics of switchers and non‐switchers at the index date. The index date was the switching date for individuals who newly adopted a two‐class regimen, and a random sample of these switching dates was selected and assigned to individuals who continued the ≥3‐class regimen. Multivariable logistic regression was used to identify factors associated with switching.

**Results**: Among 1736 participants with a regimen containing ≥3 drug classes, 55.5% switched to a two‐class regimen over the study period. Mean age was 53 years, and 67% had a history of virological failure, which was less common in switchers compared to non‐switchers (58% vs. 78%; Table 1). Factors associated with a lower likelihood of switching included time since ART start (adjusted odds ratio [aOR] 0.54, 95% CI 0.39–0.78 for 10–20 years vs. <10 years), a history of virological failure (aOR 0.63, 95% CI 0.46–0.84), a prior AIDS‐defining event (aOR 0.63, 95% CI 0.51–0.79) and availability of resistance testing (aOR 0.55, 95% CI 0.43–0.71). There was no association between low adherence to ART, recreational drug use or hazardous drinking with switching (Figure 1).

**P115: Table 1:** Characteristics of people with HIV receiving ≥3 drug classes at the index date, stratified by switch to a two‐class regimen during follow‐up

Characteristics	Total (*n* = 1736)	Non‐switchers (*n* = 773)	Switchers (*n* = 963)	*p*‐value
Age in years, mean (SD)	52.8 (11.8)	53.9 (11.4)	51.8 (12.0)	0.003
Female sex	480 (27.7)	205 (26.5)	275 (28.6)	0.35
Current CD4 count in cells/µl, median (IQR)	575 (403−773)	572 (405−753)	587 (401−792)	0.47
Nadir CD4 count in cells/µl, median (IQR)	132 (50−250)	109 (40−216)	159 (57−288)	<0.001
Current viral suppression	1357 (78.5)	634 (82.3)	723 (75.4)	<0.001
Treatment history				
Time since ART start in years, median (IQR)	18.2 (8.9−22.0)	19.5 (13.6−22.8)	16.4 (6.0−21.3)	<0.001
Ever received NRTI monotherapy	886 (51.0)	465 (60.2)	421 (43.7)	<0.001
History of virological failure^a^	1167 (67.2)	606 (78.4)	561 (58.3)	<0.001
Low adherence^b^	87 (5.0)	36 (4.6)	51 (5.3)	0.54
Comorbidities				
Prior AIDS‐defining event	594 (34.2)	323 (41.8)	271 (28.1)	<0.001
HBs‐Ag ever positive	94 (5.4)	38 (4.9)	56 (5.8)	0.41
Depression or psychiatric treatment	349 (20.1)	154 (19.9)	195 (20.2)	0.87
Recent recreational drug use	295 (17.0)	128 (16.6)	167 (17.3)	0.67
Current hazardous drinking^c^	957 (55.1)	426 (55.1)	531 (55.1)	0.99
Resistance				
Prior resistance test performed	1287 (74.1)	606 (78.4)	681 (70.7)	<0.001

*Note*: Numbers are presented as *n* (%), unless indicated otherwise.

Abbreviations: ART, antiretroviral therapy; IQR, interquartile range; NRTI, nucleoside/nucleotide reverse transcriptase inhibitor; SD, standard deviation.

^a^Defined as two consecutive viral loads >200 cp/ml or one viral load >200 cp/ml followed by a treatment change if the patient had experienced ≥180 days of continuous ART.

^b^Defined as a missed ART more than once every 2 weeks, or more than one dose in a row.

^c^Defined by an AUDIT‐C (Alcohol Use Disorders Identification Test‐Concise) score ≥3 in women and ≥4 in men.

**P115: Figure 1:**
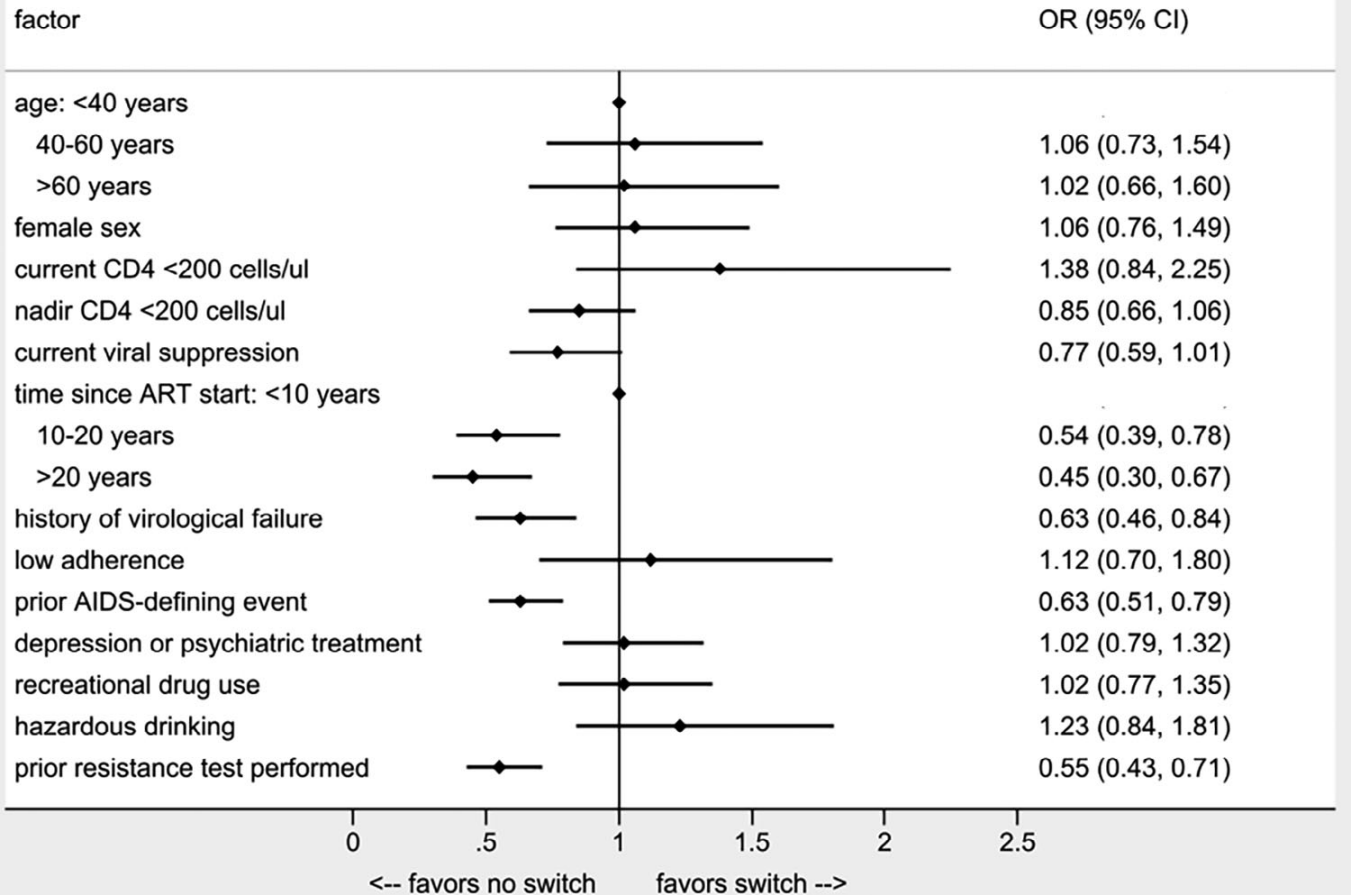
Factors associated with switching from an antiretroviral treatment (ART) regimen with ≥3 drug classes to a two‐class regimen in multivariable analysis*. *In addition to the variables shown, following predictors were included in the model: ethnicity, mode of HIV acquisition, history of treatment interruption >30 d, prior nucleoside/nucleotide reverse transcriptase monotherapy, current boosted regimen, HBs‐Ag ever positive, anti‐HBc‐alone positive, cardiovascular disease, liver disease, diabetes mellitus, osteoporosis, estimated glomerular filtration rate <60 ml/minute.

**Conclusions**: More than half of participants with complex treatment regimens were switched to a two‐class regimen within the last decade. Prior virological failure, AIDS‐defining events and a long treatment history were barriers to switching, while low ART adherence and substance use were not.

### P116 CD4 T‐cell, CD4/CD8 ratio improvement and a general reduction in inflammatory biomarkers with low‐level viraemia (LLV) up to week 192 with fostemsavir (FTR)‐based regimens in individuals with multidrug‐resistant (MDR) HIV‐1

Vincenzo Spagnuolo^1^, Natalia Gregori^2^, Iacopo Marcon^2^, Fangfang Du^3^, Bo Li^3^, Marcia Wang^3^, Alftan Dyson^4^, Manyu Prakash ^5^, Andrew Clark^5^

^1^Infectious Diseases, IRCCS San Raffaele Scientific Institute, Milan, Italy. ^2^Medical Affairs, ViiV Healthcare, Verona, Italy. ^3^Statistics, GlaxoSmithKline, Collegeville, PA, USA. ^4^Medical Affairs, ViiV Healthcare, Durham, NC, USA. ^5^Clinical Development, ViiV Healthcare, Brentford, UK

**Background**: Persistent LLV (40−1000 c/ml) is associated with virological failure, drug resistance, and increased risk of inflammation and may impact morbidity and mortality. FTR (prodrug of the first‐in‐class attachment inhibitor temsavir) is indicated with other antiretrovirals (ARVs) for heavily treatment‐experienced individuals with MDR HIV‐1 unable to construct suppressive regimens. BRIGHTE participants did not have to stop FTR due to LLV. We describe outcomes and inflammatory biomarkers through week 192 in participants with LLV (<40, 40–400, 400–1000 and >1000 c/ml) on FTR‐based regimens from the randomized cohort (RC) in the BRIGHTE study.

**Materials and methods**: BRIGHTE was a phase III study (*N* = 371; RC, *n* = 272; non‐randomized cohort, *n* = 99) in adults failing their current ARV regimen (HIV‐1 RNA >400 c/ml) with ≤2 fully active approved ARVs. Participants with one or two active ARVs entered the RC and received open‐label FTR + optimized background therapy after an 8‐day blinded placebo‐controlled period. Virological and immunological responses were analysed by baseline (BL) demographics and disease characteristics.

**Results**: At BL in the RC, 89% of participants had CD4 T‐cell count <350 cells/mm^3^, with 5% between 350 and <500 cells/mm^3^ and 6% ≥500 cells/mm^3^. Mean CD4 T‐cell increase observed in the <40 c/ml (*n* = 142) group was 331 cells/mm^3^, 40–400 c/ml (*n* = 25) was 263 cells/mm^3^, 400–1000 c/ml (*n* = 2) was 218 cells/mm^3^, and in participants with >1000 c/ml (*n* = 10) was 107 cells/mm^3^. Similar mean increase in CD4/CD8 ratio was observed in participants with LLV; ratio in the <40 c/ml (*n* = 142) group was 0.38, 40–400 c/ml (*n* = 25) was 0.31, 400–1000 c/ml (*n* = 2) was 0.32, and in participants with >1000 c/ml (*n* = 10) was 0.09. Measured biomarkers (sCD14, D‐dimer, sCD163) showed a mean general reduction from BL across all groups (<40 c/ml, 40–400 c/ml, 400–1000 c/ml, >1000 c/ml).

**Conclusions**: In participants with LLV, there is a persistent increase in CD4 T‐cell count and improvement in CD4/CD8 ratio, with a general reduction in inflammatory markers up to week 192. Results highlight the value of FTR‐based regimens for sustained improvement where there is incomplete virological suppression.

### P117 Genotypic assessment of the viability of second‐generation NNRTIs as an alternative therapy for ART‐experienced individuals with multi‐class HIV drug resistance in Botswana

Nokuthula Ndlovu ^1^, Ontlametse Choga^1^, Wonderful Choga^1^, Natasha Moraka^1^, Dorcas Maruapula^1^, Patrick Mokgethi^1^, Kaelo Seatla^1^, David Nkwe^2^, Vlad Novitsky^3^, Sikhulile Moyo^1^, Simani Gaseitsiwe^1^

^1^Research Laboratory, Botswana Harvard Health Partnership, Gaborone, Botswana. ^2^Department of Biological Sciences and Biotechnology, Botswana International University of Science and Technology, Palapye, Botswana. ^3^Department of Immunology and Infectious Diseases, Harvard T.H. Chan School of Public Health, Boston, MA, USA

**Background**: The emergence of multi‐class HIV‐1 drug resistance (MDR) significantly limits antiretroviral therapy (ART) options. This study investigated the prevalence of MDR HIV‐1 strains in Botswana, and evaluated the susceptibility of these strains to second‐generation non‐nucleoside reverse transcriptase inhibitors (NNRTIs), doravirine (DOR), etravirine (ETR) and rilpivirine (RPV).

**Materials and methods**: HIV‐1C proviral pol sequences from 4769 ART‐experienced individuals were included in this study. Major HIV drug‐resistance mutations (DRMs) were analysed according to the Stanford HIV drug‐resistance database. Sequences with hypermutations were excluded from the analysis. Participants harbouring resistance to two or more ARV classes (MDR), with at‐least NNRTI, were further evaluated for resistance to DOR, ETR and RPV. The Stanford “DRM penalty scores” were utilized to predict resistance levels and interpretation (individuals with low‐level, intermediate‐level and high‐level resistance were considered to have resistance).

**Results**: A total of 1549 (32.5%; 95% Cl 31.1–33.8) had resistance to at least one ARV class, with 1122/1549 (72.4%) resistant to NNRTIs. Six hundred and eleven out of 1122 (54.5%) had resistance to one drug class and 511 (45.5%) had MDR. Of the 511, 495 (96.9%; 95% Cl 95.1–98.2) had resistance to at least one second‐generation NNRTI, of which 366 (71.6%) were resistant to all three ARVs; DOR, ETR and RPV. The majority of participants had low‐level DOR (78.1%) and ETR (80.4%) resistance. Sixty‐five percent, 15.4% and 9% had intermediate RPV, ETR and DOR resistance, respectively. The study observed high‐level resistance (20.1%) to RPV compared to 12.8% and 4.2% in DOR and ETR. Mutations M230I, E138K and G190E were the most predominant (Table 1). MDR individuals failing second‐generation NNRTIs had a high prevalence of resistance to nucleoside reverse transcriptase inhibitors (399/495; 80.6%) and protease inhibitors (255/495; 51.5%). A prevalence of 5.9% (29/495) integrase strand transfer inhibitor (INSTI) resistance was observed within these individuals.

**P117: Table 1:** Prevalence of DOR, ETR and RPV resistance and associated mutations within treatment‐experienced individual with MDR

	DOR (%) *n* = 398	ETR (%) *n* = 382	RPV (%) *n* = 477
Overall (*n* = 495)	80.4	77.2	96.4
Low level	78.1	80.4	14.9
Intermediate	9	15.4	65
High level	12.8	4.2	20.1
Specific mutations			
A98G	^d^	^d^	3.6^a^
L100I	0.8^a^	0.8^b^	0.6^c^
K101E	3.5^a^	3.7^a^	2.9^b^
K101P	^d^	0.3^c^	0.2^c^
V106A	0.5^c^	^d^	^d^
V106M	4.0^b^	^d^	^d^
E138K	^d^	^d^	12.2^b^
E138Q	^d^	^d^	0.8^a^
Y181C	^d^	6.8^b^	5.5^b^
Y181I	^d^	0.3^c^	0.2^c^
Y188F	0.3^b^	^d^	0.2^b^
Y188L	1.5^c^	^d^	1.3^c^
G190E	7.8^c^	8.1^b^	6.5^c^
G190S	0.5^b^	^d^	^d^
H221Y	^d^	^d^	1.0^a^
P225H	2.3^b^	^d^	^d^
F227C	0.3^c^	0.3^b^	0.2^b^
F227L	0.8^c^	^d^	^d^
M230I	^d^	^d^	64.4^b^
M230L	0.5^c^	0.5^b^	0.4^c^
Y318F	0.3^c^	^d^	^d^

^a^Mutation associated with low‐level resistance.

^b^Mutation associated with intermediate resistance.

^c^Mutation associated with high‐level resistance.

^d^Mutation associated with susceptibility.

**Conclusions**: We report a high proportion of resistance to second‐generation NNRTIs among treatment‐experienced individuals with MDR who had no prior exposure to second‐generation NNRTIs. Our results strongly suggest genotypic‐resistance testing prior to ART use among treatment‐experienced individuals.

### P118 High prevalence of HIV drug resistance among people living with HIV on dolutegravir‐based antiretroviral therapy with viral load above 200 copies/mL in Francistown, Botswana

Ontlametse T. Choga ^1^, Goitseone M. Lemogang^1^, Wonderful T. Choga^1^, Gaonyadiwe Muzanywa^1^, Thembinkosi M. Shadreck^1^, Charity Ralegoreng^1^, Dorcas Maruapula^1^, Natasha O. Moraka^1^, Catherine K. Koofhethile^1^, Patrick T. Mokgethi^1^, Kedumetse Seru^1^, Boitumelo Zuze^1^, Patience Montshosi^1^, Irene Gobe^2^, Modisa S. Motswaledi^2^, Rosemary Musonda^1^, Tony Chebani^3^, Judith Nawa^3^, Lindani Bochena^4^, Sikhulile Moyo^1^, Simani Gaseitsiwe^1^

^1^Research Lab, Botswana Harvard Health Partnership, Gaborone, Botswana. ^2^Department of Medical Sciences, University of Botswana, Gaborone, Botswana. ^3^HIV National Treatment Data Warehouse, Ministry of Health, Gaborone, Botswana. ^4^Nyangabgwe HIV Reference Laboratory, Ministry of Health, Francistown, Botswana

**Background: **HIV drug‐resistance monitoring among people living with HIV (PLWH) with detectable viral load (VL) in the era of dolutegravir (DTG)‐based antiretroviral therapy (ART) is a necessity to preserve future ART options. To date, no study has characterized HIV‐1 drug‐resistance mutations (DRMs) in PLWH on DTG‐based ART in the Botswana National Treatment Program (BNAP). Therefore, this study intends to characterize HIV DRMs among PLWH who are predominantly on DTG‐based ART with detectable VL >200 copies/ml in the central HIV VL testing laboratory in Francistown, Botswana.

**Materials and methods: **The study utilized 100 residual HIV‐1 VL samples of PLWH who are enrolled in BNAP residing in Francistown, Botswana. Samples were categorized into low‐level viraemia (LLV) (VL 200–999 copies/ml) and virological failure (VF) (VL ≥1000 copies/ml). HIV‐1 *protease, reverse transcriptase* and *integrase* genes were sequenced using an in‐house next‐generation sequencing protocol. Known HIV‐1 DRMs were identified using the Stanford HIV resistance database.

**Results: **Among 100 PLWH, 30.0% (30) had LLV, while 70.0% (70) had VF. ART regimen data was available for 90 PLWH whereby 89.0% were on DTG‐based ART. Among 58 PLWH with sequences, 32.8% had HIV DRMs. By VL groups, the prevalence of HIV DRMs was 33.3% (4/12) at LLV and 32.6% (15/46) at VF (*p* = 0.99). The most three predominant DRMs with a prevalence >5% were E138A (10.2%), K103N (8.2%) and M184V (8.2%) (Figure 1) and mostly were resistant to rilpivirine‐containing regimen (Table 1). Overall, 31.8% (14/44) of PLWH on DTG‐based ART had at least one DRM. The overall prevalence of protease inhibitors (PIs)‐, non‐nucleoside reverse transcriptase inhibitors (NNRTIs)‐, nucleoside reverse transcriptase inhibitors (NRTIs)‐ and integrase strand transfer inhibitors (INSTIs)‐associated resistance mutations was 5.3%, 23.7%, 13.2% and 5.1%, respectively, among PLWH on DTG‐based ART. Mutations N155H, G118R, E138K, R263K, L94M and Q95K were the INSTI‐resistance‐associated mutations reported among PLWH on DTG‐based ART. 

**P118: Table 1:** Characteristics of PLWH with HIV DRMs

**Characteristics**	**ART initiation date**	**Previous ART regimen**	**Current ARV regimen**	**Previous VL results (copies/ml)**	**HIV DRMs**	**ARV‐resistance levels**
Age: 26 years old Sex: Male Citizen: Yes	Sept 2003	ND	TLD	1300, 430, 3200, 4575, 8900, 3000, 5500, 5700, 5700, 13,000, 1900, <400*(6), 30, 30, 2406	INSTI: N155H, Q95K NRTI: M184V, T215Y NNRTI: K103N, V108I PI: None	Intermediate: ABC, AZT, D4T, DDI, CAB. High‐level: FTC, 3TC, EFV, NVP, EVG, RAL.
Age: 46 years old Sex: Male Citizen: No	Aug 2023	ND	ND	1326	INSTI: R263K, E157Q NRTI: M41L, E44A, D67N, M184V, L210W, T215Y, K219E NNRTI: K103N, E138G, Y181C PI: None	Intermediate: ETR, DTG, BIC, EVG, RAL. High‐level: ABC, AZT, D4T, DDI, FTC, TDF, EFV, NVP, RPV, CAB.
Age: 57 years old Sex: Female Citizen: Yes	June 2008	AZT/3TC/EFV	TLD	<400*(7), 30, 30, 0, 30, 30, 30, 790	INSTI: G118R, E138K, R263K, L94M NRTI: None NNRTI: None PI: None	Intermediate: None. High‐level: BIC, CAB, DTG, EVG, RAL.
Age: 35 years old Sex: Female Citizen: Yes	Sept 2008	ND	TLD	499, <400, 55, 2989	INSTI: None NNRTI: None NNRTI: K103N PI: None	Intermediate: None. High‐level: EFV, NVP.
Age: 62 years old Sex: Male Citizen: Yes	Sept 2015	ND	DTG	4000, 43,507, 22,350, 1290, 76,091, 643, 3475, 500, 211, 1452, 131, 1452	INSTI: FS NRTI: D67N, T69D, L74I, Y115F, M184V, K219Q NNRTI: None PI: V82L, L90M, L33F, Q58E, G73T	Intermediate: LPVr, AZT, D4T, TDF. High‐level: ATVr, FPVr, DRVr, IDVr, NFV, SQVr, TPVr, ABC, DDI, FTC, 3TC.
Age: 65 years old Sex: Male Citizen: Yes	June 2016	ND	TLD	<400*(7), 30, 85,067, 30, 383, 1028, 685, 30, 30, 59,343	INSTI: None NRTI: None NNRTI: None PI: Q58E	Intermediate: TPVr. High‐level: None.
Age: 36 years old Sex: Male Citizen: Yes	Nov 2014	ND	TDF/FTC/EFV	<400, <400, <400, 1118, <400, <400, 30, 89,154	INSTI: None NRTI: None NNRTI: E138A PI: None	Intermediate: RPV. High‐level: None.
Age: 24 years old Sex: Male Citizen: Yes	Jun 2016	ND	ND	<400*(5), 30, 45,697	INSTI: FS NRTI: None NNRTI: E138A PI: None	Intermediate: RPV. High‐level: None.
Age: 55 years old Sex: Male Citizen: Yes	Dec 2014	3TC	TLD	<400*(7), 40,082	INSTI: None NRTI: M184V NNRTI: None PI: None	Intermediate: ABC. High‐level: FTC, 3TC.
Age: 25 years old Sex: Female Citizen: Yes	Jan 2015	ND	TLD	<400*(7), 627, <400*(5), 318	INSTI: None NRTI: None NNRTI: E138A PI: None	Intermediate: RPV. High‐level: None.
Age: 48 years old Sex: Female Citizen: Yes	Aug 2008	ND	TLD	59,000, 11,000, <400, <400*(13), 964,822, 20,134, 30, 30, 30, 30, 30, 1,953,806, 476	INSTI: None NRTI: A62V, K65R, S68G NNRTI: K101E, Y181C, G190A PI: None	Intermediate: FTC, 3TC, DOR. High‐level: D4T, DDI, TDF, EFV, ETR, NVP, RPV.
Age: 29 years old Sex: Female Citizen: No	Feb 2022	ND	TLD	7292, 181, 3523	INSTI: FS NRTI: None NNRTI: None PI: V82L	Intermediate: FPVr, TPVr. High‐level: None.
Age: 16 years old Sex: Female Citizen: Yes	Feb 2022	AZT/3TC/NVP	AZT/3TC/NVP	543, <400(35), 8448, 94, 30, 30, 1525, 311, 30, 30, 33190	INSTI: None NRTI: None NNRTI: E138A PI: None	Intermediate: RPV. High‐level: None.
Age: 39 years old Sex: Female Citizen: Yes	Feb 2016	TDF/FTC/EFV	TLD	<400*(10), 997, 30, 20, 57,1850	INSTI: None NRTI: None NNRTI: V108I PI: None	Intermediate: DOR. High‐level: EFV, NVP.
Age: 43 years old Sex: Male Citizen: Yes	Nov 2011	TDF/FTC/NVP	TDF/FTC/NVP	<400*(10), 20, 30, 30, 7,500,000	INSTI: None NRTI: None NNRTI: None PI: V82L	Intermediate: TPVr. High‐level: None.
Age: 27 years old Sex: Female Citizen: Yes	Aug 2023	ND	TLD	133,674, 1574	INSTI: None NRTI: M184I NNRTI: V108I, Y181V, Y188F, G190S PI: None	Intermediate: ABC. High‐level: FTC, 3TC, DOR, EFV, ETR, RPV.
Age: 41 years old Sex: Female Citizen: Yes	Mar 2007	ND	DTG	670,000, <400*(8), 1000, 670, 750,000, 417,512, 367,265, 166,121, 16,000, 280,000, 470,000, <400, 29,643, 2994, 3384, <400, 2905, 991, 14	INSTI: None NRTI: None NNRTI: V179D PI: None	Intermediate: None. High‐level: None.
Age: 28 years old Sex: Female Citizen: Yes	Apr 2016	ND	TLD	5300, 28,575, 15,000, 11,000, <400, <400, <400, 19,000, 10,240, 13,541, <400, 5518, 38,247, 50,084, 56,940, 30, 30, 44,530, 1527, 44,339	INSTI: None NRTI: None NNRTI: K101P, K103N PI: None	Intermediate: None. High‐level: EFV, ETR, NVP, RPV.
Age: 44 years old Sex: Male Citizen: Yes	Apr 2009	ND	TLD	<400(13), 30, 30, 30, 206	INSTI: FS NRTI: None NNRTI: E138A PI: None	Intermediate: RPV. High‐level: None.

Abbreviations: 3TC, lamivudine; ABC, abacavir; ATV/r, atazanavir/ritonavir; AZT, zidovudine; BIC, bictegravir; CAB, cabotegravir; D4T, stavudine; DDI, didanosine; DOR, doravirine; DRMs, drug‐resistance mutations; DTG, dolutegravir; EFV, efavirenz; ETR, etravirine; EVG, elvitegravir; FPV/r, fosamprenvir/ritonavir; FS, failed sequencing; FTC, emtricitabine; FVL, first VL measurement was genotyped; IDV/r, indinavir/ritonavir; INSTI, integrase strand transfer inhibitor; LLV, low‐level viraemia; LPV/r, lopinavir/ritonavir; *N*, number of sequences available; ND, not documented; NFV, nelfinavir; NNRTI, non‐nucleoside reverse transcriptase inhibitor; NRTI, nucleoside reverse transcriptase inhibitor; NVP, nevirapine; PI, protease inhibitor; RAL, raltegravir; RPV, rilpivirine; TDF, tenofovir disoproxil fumarate; TLD, tenofovir disoproxil fumarate/lamivudine/dolutegravir; TPV, tipranavir.

* The consecutive VL measurement of the same value; () the times that the same VL measurement value was recorded.

**P118: Figure 1:**
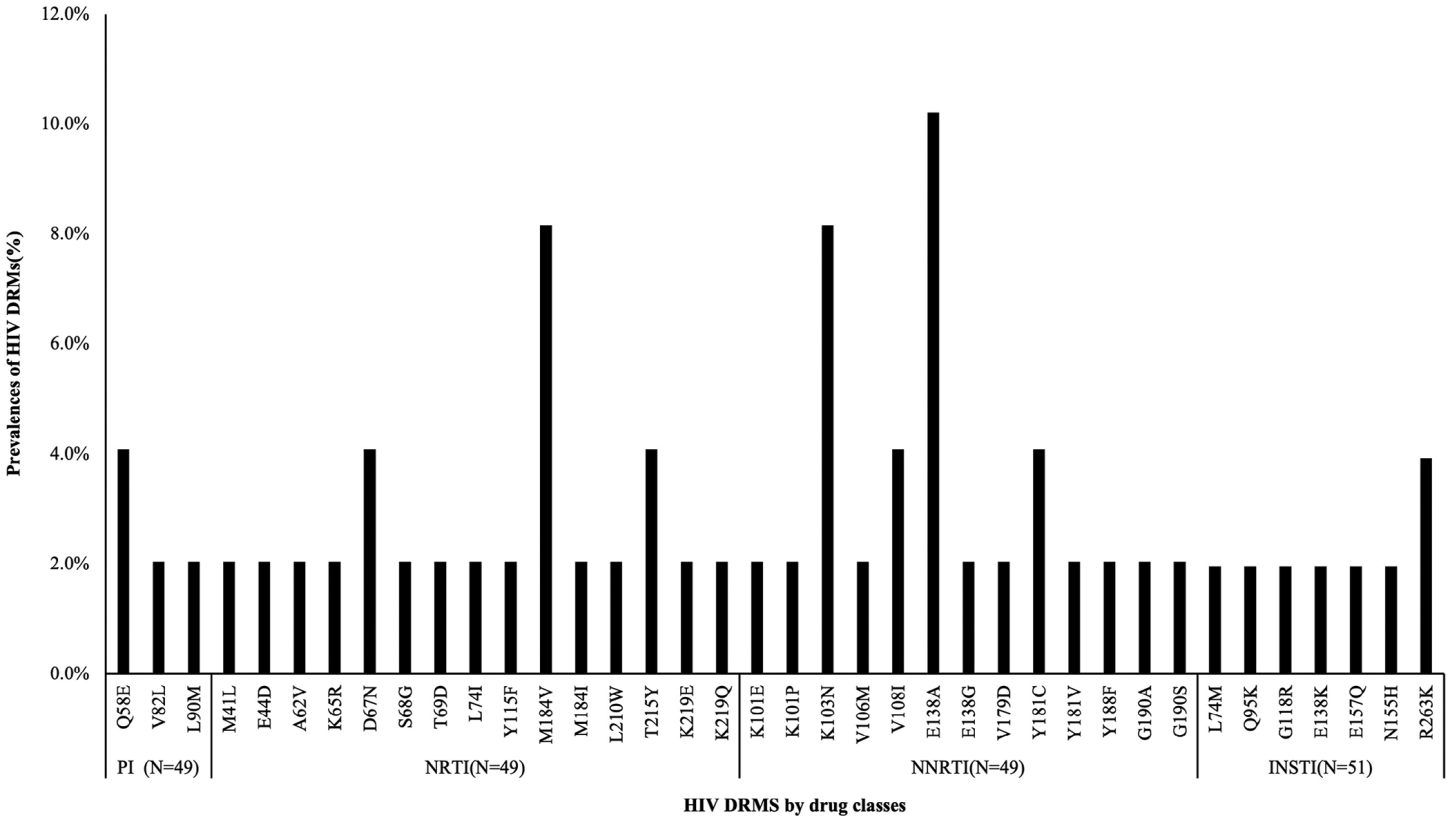
HIV DRMs by drug classes. INSTI, integrase strand transfer inhibitor; NNRTI, non‐nucleoside reverse transcriptase inhibitor; NRTI, nucleoside reverse transcriptase inhibitor; PI, protease inhibitor.

**Conclusions**: The overall prevalence of HIV DRMs in PLWH who are primarily on DTG‐based ART with detectable VL >200 copies/ml was high. However, a low prevalence of DTG‐resistance‐associated mutations was reported in this cohort supporting the continual use of DTG‐based ART with HIV drug‐resistance monitoring.

### P119 Replication‐competent HIV‐1 harbouring resistance‐associated mutations is present in the viral reservoir

Michelle L. D'Antoni, Silvia Chang, Laurie A. VanderVeen, Christian Callebaut

Clinical Virology, Gilead Sciences, Inc., Foster City, CA, USA

**Background**: Detecting resistance‐associated mutations (RAMs) in proviral HIV‐1 DNA can be useful for regimen selection. However, this method is insensitive due to sampling limited numbers of latently infected peripheral CD4^+^ T cells. Interpretation of proviral genotyping in terms of clinical relevance of archived resistance is also confounded by not knowing whether identified RAMs are in replication‐competent virus. We examined whether RAMs identified by proviral genotyping were also present in replication‐competent virus.

**Materials and methods**: Genotyping of proviral HIV‐1 protease (PR)/reverse transcriptase (RT)/integrase (IN) was conducted on peripheral blood mononuclear cells (PBMCs) from virologically suppressed people with HIV, using GenoSure Archive (1–3 replicates; Monogram Biosciences). Quantitative virus outgrowth assays (QVOA; Accelevir), performed on matching PBMC aliquots, were used to recover and sequence replication‐competent virus (Illumina MiSeq; Seq‐IT). Primary (PR/RT/IN) or secondary (IN only) RAMs identified by proviral and outgrowth virus genotyping were compared. RAM detection (%) was calculated as (no. of times an RAM was detected/no. of GenoSure Archive assays run or no. of outgrowth wells in QVOA) × 100.

**Results**: From 12 PBMC samples, 87 RAMs were identified across both assays: 17 PR inhibitor, 45 nucleos(t)ide RT inhibitor (NRTI), 13 non‐NRTI, four primary IN strand transfer inhibitor (INSTI) and eight secondary INSTI RAMs. Of the 70 RAMs identified by GenoSure Archive, 67.1% (*n* = 47) were also detected by QVOA. When RAMs were detected in all GenoSure Archive replicates (*n* = 55), concordant detection by QVOA increased to 76.4% (*n* = 42). Seventeen of 87 (19.5%) RAMs were identified by QVOA only (2–54% detection). For M184V specifically (*n* = 8), when detection across GenoSure Archive replicates was reproducible (100%) versus variable (50–67%), detection by QVOA was *n* = 3/4 versus *n* = 0/3; *n* = 1 was detected by QVOA only.

**Conclusions**: RAMs reported by proviral genotyping may also be found in replication‐competent virus, indicating that potentially infectious, antiretroviral‐resistant viruses can persist in the reservoir. Reproducible RAM detection by proviral genotyping was linked to increased RAM detection by QVOA. Lastly, proviral genotyping may miss RAMs that are found in replication‐competent virus. These data reinforce the need to consider all treatment history and prior resistance data for regimen selection as proviral genotyping might miss previous RAMs.

### P120 Virological efficacy of dolutegravir plus darunavir in multi‐drug‐resistant HIV patients: a real‐world cohort study with data from the PRESTIGIO registry

Filippo Lagi ^1^, Michele Bellomo^2^, Riccardo Lolatto^2^, Filippo Ducci^3^, Seble Tekle Kiros^3^, Rebecka Papaioannu^4^, Tommaso Celemente^4^, Leonardo Calza^5^, Marcello Feasi^6^, Emanuele Focà^7^, Andrea Giacomelli^8^, Roberto Gulminetti^9^, Barbara Menzaghi^10^, Antonella Castagna^4^

^1^Infectious and Tropical Diseases Unit, Careggi University Hospital, Florence, Italy. ^2^Infectious Diseases, IRCCS San Raffaele Scientific Institute, Milan, Italy. ^3^Department of Experimental and Clinical Medicine, University of Florence, Florence, Italy. ^4^Vita‐Salute San Raffaele University, IRCCS San Raffaele Hospital, Milan, Italy. ^5^Unit of Infectious Diseases, Department of Medical and Surgical Sciences, S. Orsola Hospital, Bologna, Italy. ^6^Ospedali Galliera, Department of Infectious Diseases, Genoa, Italy. ^7^Unit of Infectious and Tropical Diseases, ASST Spedali di Brescia, Brescia, Italy. ^8^Division of Infectious Diseases, ASST Fatebenefratelli Sacco, Luigi Sacco Hospital, Milan, Italy. ^9^University of Pavia, Fondazione IRCCS Policlinico San Matteo, Pavia, Italy. ^10^Unit of Infectious Diseases, ASST della Valle Olona, Busto Arsizio, Italy

**Background**: This study evaluated the virological efficacy of DTG plus DRV/b in people with four‐class drug‐resistant HIV (4DR‐PWH) in a real‐world setting.

**Materials and methods**: Retrospective study analysing antiretroviral treatments (ARTs) used by adults with 4DR HIV from the PRESTIGIO registry [1]. The ART regimes were categorized into three groups: (i) DTG and DRV/b only (DTG+DRV/b); (ii) DTG and DRV/b plus ≥1 additional antiretroviral drug (ARV) (DTG+DRV/b+other); (iii) regimens not including the combination of DTG and DRV/b (Other). Follow‐up started from the first evidence of 4DR (baseline) until death, loss to follow‐up or 30 May 2024. A person can change groups multiple times during follow‐up. The relationship between DTG+DRV/b and virological failure (VF) was analysed using mixed‐effects logistic regression. VF was defined as ≥2 HIV‐RNA determinations >50 copies/ml or one ≥1000 copies/ml. Regimens were classified as 0 (not failed) or 1 (failed) at each HIV‐RNA measurement. Regression was adjusted for age, ART duration, number of fully active ARV, sex at birth and nadir CD4^+^. Individual failure predisposition was estimated with a random intercept.

**Results**: We evaluated 844 regimens from 249 4DR‐PWH with a median follow‐up of 8.7 years (5.9–11.5). Specifically, the 844 regimens were distributed as follows: 72 (8.5%) DTG+DRV/b, 508 (60.2%) DTG+DRV/b+other and 264 (31.3%) Other. In the DTG+DRV/b, the median number of fully active ARV included and the percentage of full activity of DTG and DRV/b were higher than the other groups (Table 1). Logistic analysis indicated the odds of VF is 77% and 35.9% lower with DTG+DRV/b and DTG+DRV/b+other, respectively, compared to “Other.” Each fully active ARV in the regimen decreases VF odds by 40%. Older age and longer ART duration lessen the odds of VF, likely due to better HIV control over time (Figure 1). DTG+DRV/b remains virologically effective despite the partial activity of its components in the group (DRV/b fully active in 47.2%, DTG fully active in 63.9%).

**P120: Table 1:** Number, duration and activity of antiretrovirals included in the ART regimes analysed

	**DRV+DTG (*n* = 72)**	**DTG+DRV+other (*n* = 508)**	**Other (*n* = 254)**
Duration of the regimen in years; median (IQR)	4.2 (1.7−5.7)	1.8 (0.6−3.5)	1.3 (0.5−2.8)
Cumulative duration of the regimen in years	280.1	656.9	1010.1
Number of individuals exposed to the indicated regimen	60 (24.1%)	136 (54.6%)	181 (72.7%)
Number of fully active drugs included at each regimen initiation; median (IQR)	1 (1−2)	1 (0−2)	1 (0−1)
Percentage of regimen with fully activity of DRV/b at the time of its inclusion in the regimen	47.2%	26.5%	23.7%^a^
Percentage of regimen with fully activity of DTG at the time of its inclusion in the regimen	63.9%	46.2%	49.7%^b^

Abbreviations: DRV/b, boosted darunavir; DTG, dolutegravir.

^a^Regimen with DRV in other *N* = 207 (40.7%).

^b^Regimen with DTG in other *N* = 179 (35.2%).

**P120: Figure 1:**
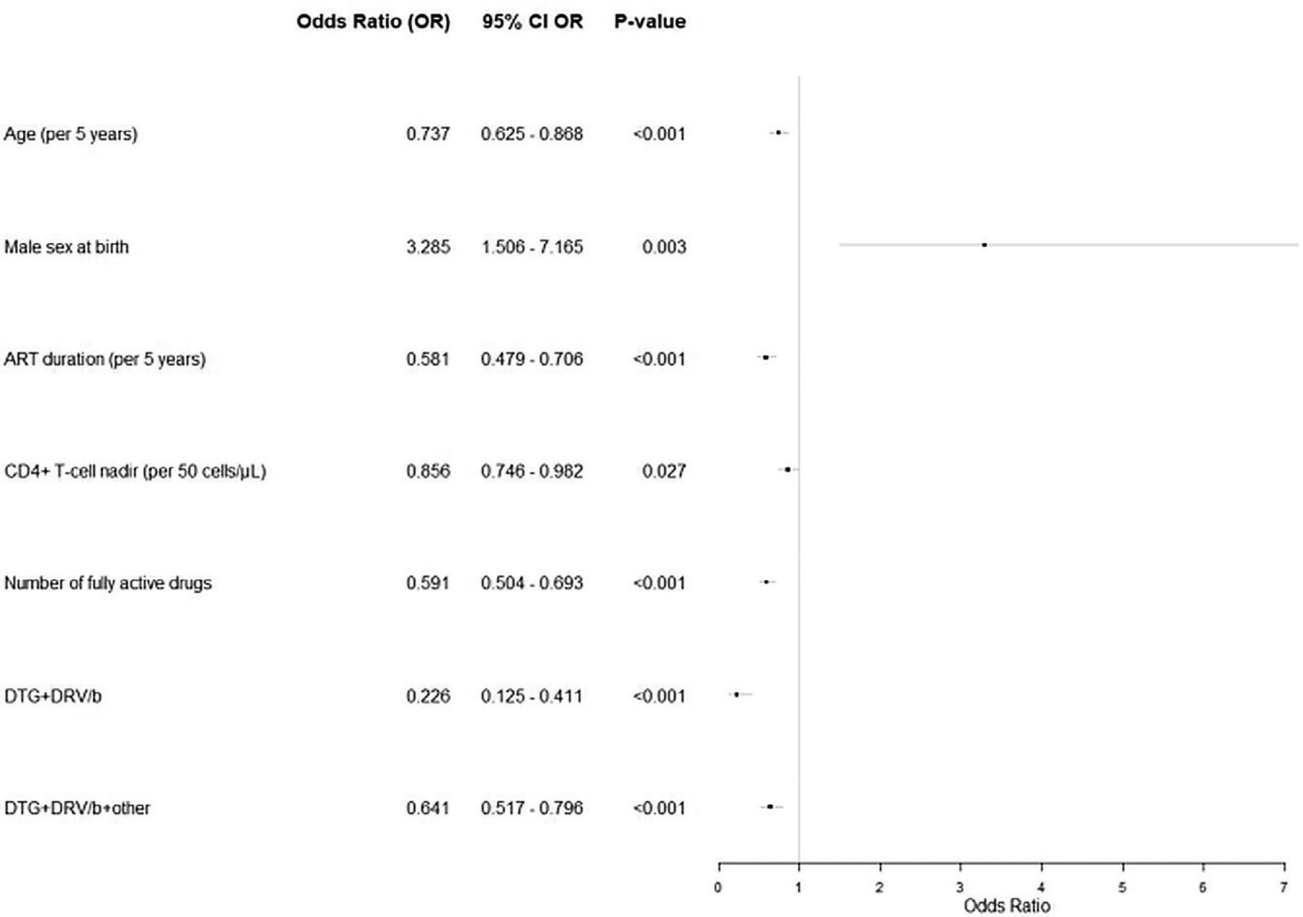
Forest plot of multivariate logistic regression analysing the relationship between DTG+DRV/b and virological failure in a cohort of people with four‐class drug‐resistant HIV.

**Conclusions**: Among 4DR‐PWH, DTG+DRV/b is associated with a lower odd of VF even after adjusting for the number of fully active drugs. This combination is also effective when not fully active or combined with other ARVs.

**Reference**

1. Clemente T, Galli L, Lolatto R, Gagliardini R, Lagi F, Ferrara M, et al. Cohort profile: PRESTIGIO, an Italian prospective registry‐based cohort of people with HIV‐1 resistant to reverse transcriptase, protease and integrase inhibitors. BMJ Open. 2024;14(2):e080606.

### P121 Effectiveness of switching to B/F/TAF in virologically suppressed people with HIV and with pre‐existing resistance‐associated mutations in Italy: the BIC‐BARRIER study

Federico Conti ^1^, Laura Pezzati^2^, Alessandro Cozzi‐Lepri^3^, William Gennari^4^, Cristina Mussini^5^, Emanuele Pontali^6^, Anna Volpe^7^, Ilaria Vicenti^8^, Annalisa Saracino^7^, Barbara Rossetti^9^, Bianca Bruzzone^10^, Adrian Shallvari^11^, Laura Albini^12^, Dario Corsini^11^, Maurizio Zazzi^8^, Stefano Rusconi^13^

^1^Infectious Diseases Unit, ASST Lecco, Lecco, Italy. ^2^Infectious Diseases Unit, ASST Ovest Milanese, Legnano, Italy. ^3^Centre for Clinical Research, Epidemiology, Modelling and Evaluation (CREME), Institute for Global Health, UCL, London, UK. ^4^SDD Virologia‐Microbiologia Molecolare, Azienda Ospedaliero‐Universitaria di Modena, Modena, Italy. ^5^Infectious Diseases Unit, Università degli Studi di Modena‐Reggio Emilia, Modena, Italy. ^6^Infectious Diseases Unit, Ente Ospedaliero Ospedali Galliera, Genova, Italy. ^7^Infectious Diseases Unit, Università degli Studi di Bari “Aldo Moro”, Bari, Italy. ^8^Dipartimento di Biotecnologie Mediche, Università degli Studi di Siena, Siena, Italy. ^9^Infectious Diseases Unit, USL Sud‐Est Toscana, Ospedale della Misericordia, Grosseto, Italy. ^10^Hygiene Unit, Azienda Ospedaliero‐Universitaria San Martino, Genova, Italy. ^11^InformaPRO S.r.l., EuResist Network GEIE, Rome, Italy. ^12^Medical Affairs, Gilead Sciences, Milan, Italy. ^13^University of Milan, Dipartimento di Scienze Biomediche e Cliniche (DIBIC), Legnano, Italy

Bictegravir/emtricitabine/tenofovir alafenamide (B/F/TAF) is a widespread switching option in people with HIV (PWH). We investigated the prevalence and impact of pre‐existing resistance‐associated mutations (RAMs) and Stanford genotypic susceptibility scores (GSS) in adult virologically suppressed PWH enrolled in the ARCA cohort, when first switching to B/F/TAF with HIV‐RNA ≤50 copies/ml (baseline/BL). We conducted a survival analysis of the time to confirmed virological rebound (VR, i.e. two consecutive HIV‐RNA values >50 copies/ml) using Kaplan‐Meier curves, and evaluated the association between exposure factors linked to resistance or history of previous virological failure (VF) and risk of VR by standard Cox regression analysis after controlling for confounding factors. A sensitivity analysis was conducted using the 200 copies/ml threshold for VR. We included 739 PWH and 617 were included in the survival analysis. Median age was 53 years [IQR 43–59], 25% were female. Major NRTI RAMs were found in 26.7% (23.5–30.1) PWH, the most common being M184V in 20.9% (18.0–24.1). Major INSTI RAMs were detected in 3.4% (1.8–5.9) of the 350 PWH with an available INSTI GRT, the most common being N155H in 2% (0.8–4.1) of PWH; the 0.6% (0.1–2.1) harboured Q148H. In the adjusted analysis, there was no evidence for a difference in risk of VR according to NRTI RAMs detection (*p* = 0.61, Figure 1 (top)). By 36 months from BL, the risk of VR was 18.1% (95% CI 2.9–33.2) in PWH with prior INSTI VF versus 4.8% (95% CI 2.8–6.8) in those without (*p* < 0.0001, Figure 1 (bottom)). After controlling for confounding factors, the detection of major INSTI RAMs at BL, a history of INSTI VF and the number of GRT performed before BL were all strongly associated with a higher risk of VR (Table 1). Results were similar for VR >200 copies/ml (not shown). The estimated prevalence of NRTI RAMs in our study was similar to that shown in the literature for the suppressed switched setting while INSTI‐resistance was infrequent (<5%). B/F/TAF appears to remain effective in the presence of NRTI RAMs; however, evidence of past INSTI failures and of major INSTI RAMs pose a risk for VR.

**P121: Table 1:** Unadjusted and adjusted relative hazards of VR >50 copies/ml from fitting a Cox regression model

	Unadjusted RH (95% CI)	*p*‐value	Adjusted RH (95% CI)^a^	*p*‐value	Adjusted RH (95% CI)^b^	*p*‐value
Any NRTI DRM						
No	1.00		1.00		1.00	
Yes	1.22 (0.57−2.61)	0.609	0.73 (0.27−2.00)	0.540	1.44 (0.65−3.21)	0.367
M184I or M184V						
No	1.00		1.00		1.00	
Yes	1.20 (0.51−2.79)	0.680	0.77 (0.27−2.17)	0.617	1.31 (0.55−3.13)	0.542
Any major NRTI DRM						
No	1.00		1.00		1.00	
Yes	1.12 (0.51−2.46)	0.768	0.61 (0.21−1.76)	0.358	1.35 (0.59−3.05)	0.477
Any major INSTI DRM						
No	1.00		1.00		1.00	
Yes	6.98 (2.11−23.11)	0.001	5.83 (1.36−24.91)	0.017	6.22 (1.63−23.73)	0.007
GSS of BIC regimen						
3	1.00		1.00		1.00	
0−2.75	1.29 (0.59−2.81)	0.527	1.10 (0.44−2.74)	0.840	1.49 (0.63−3.51)	0.367
GSS of BIC drug						
Sensitive	1.00		1.00		1.00	
Resistant	0.18 (0.02−1.36)	0.097	0.15 (0.02−1.20)	0.073	0.20 (0.03−1.57)	0.127
Previous history of VF						
No	1.00		1.00		1.00	
Yes	2.14 (1.03−4.44)	0.042	2.11 (0.72−6.18)	0.174	2.40 (1.12−5.16)	0.024
Previous history of INSTI VF						
No	1.00		1.00		1.00	
Yes	3.08 (1.37−6.93)	0.006	2.52 (0.93−6.82)	0.068	3.05 (1.32−7.03)	0.009
Previous number of VF						
Per 1 additional	1.15 (0.96−1.38)	0.131	1.17 (0.96−1.43)	0.130	1.21 (1.00−1.46)	0.055
Previous number of GRT						
Per 1 additional	1.19 (1.08−1.31)	<.001	1.18 (1.04−1.33)	0.012	1.20 (1.07−1.34)	0.001

Abbreviations: DRM, drug‐related mutation; GRT, genotypic‐resistance testing; RH, relative hazard.

^a^For age, gender, ethnicity, HIV subtype, year of BIC initiation, number of previous regimens failure, HIV‐RNA at switch, time from last available GRT and previous gap in care.

^b^For ethnicity, HIV subtype, year of BIC initiation, HIV‐RNA at switch, time from last available GRT and previous gap in care.

**P121: Figure 1:**
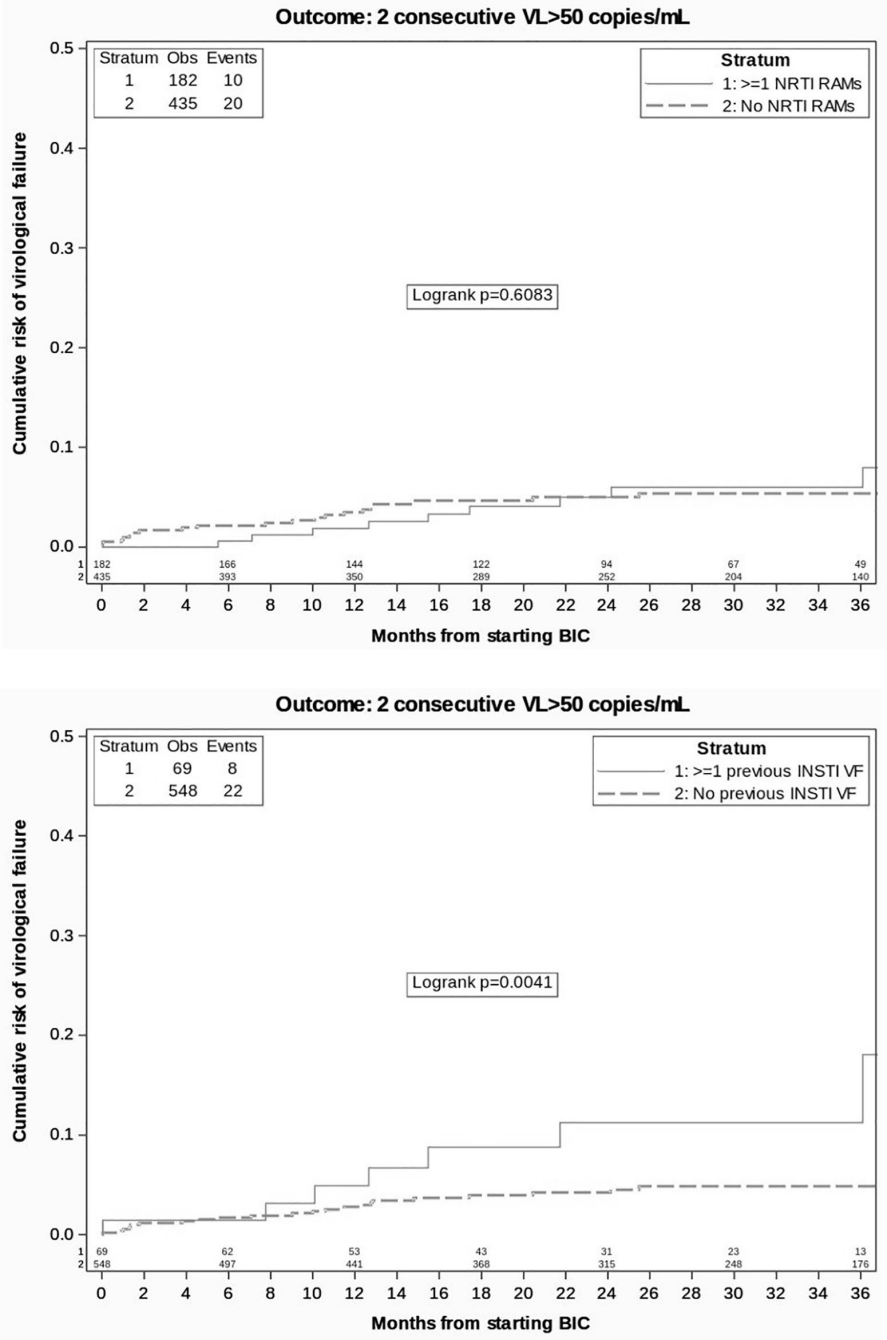
(top) Kaplan‐Meier curve of the time to VR >50 copies/ml according to previous detection of NRTI RAMs; (bottom) Kaplan‐Meier curve of the time to VR >50 copies/ml according to previous history of INSTI‐VF.

### P122 Switch from etravirine to doravirine as a part of antiretroviral combination in virally suppressed people living with HIV (PLWH): results of the French multicentre, observational SWEED study

Romain Palich ^1^, Jade Ghosn^2^, Karine Lacombe^3^, Christia Palacios^4^, Pierre Delobel^5^, Pascale Puglièse^6^, Clotilde Allavena^7^, Claudine Duvivier^8^, Firouze Bani‐Sadr^9^, Olivier Robineau^10^, Yasmine Dudoit^1^, Duc Hoan^11^, Aissata Kaba^11^, Cathia Soulie^12^, Christine Katlama^1^, Lambert Assoumou^11^

^1^Infectious Diseases, Sorbonne University, Pitié‐Salpêtrière Hospital, APHP, Paris, France. ^2^Infectious Diseases, Paris‐Cité University, Bichat Hospital, APHP, Paris, France. ^3^Infectious Diseases, Sorbonne University, Saint Antoine Hospital, APHP, Paris, France. ^4^Infectious Diseases, Sorbonne University, Tenon Hospital, APHP, Paris, France. ^5^Infectious Diseases, Toulouse Purpan University Hospital, Toulouse, France. ^6^Infectious Diseases, Nice University Hospital, Nice, France. ^7^Infectious Diseases, Nantes University Hospital, Nantes, France. ^8^Infectious Diseases, Paris‐Cité University, Necker Hospital, APHP, Paris, France. ^9^Infectious Diseases, Reims University Hospital, Reims, France. ^10^Infectious Diseases, Tourcoing University Hospital, Tourcoing, France. ^11^Pierre Louis Epidemiology and Public Health Institute, Sorbonne University, INSERM 1136, Epidemiology, Paris, France. ^12^Virology, Sorbonne University, Pitié‐Salpêtrière Hospital, APHP, Paris, France

**Background**: Doravirine is the most recent NNRTI, which may advantageously replace etravirine. We investigated the tolerance and efficacy of doravirine‐based treatments (DOR‐ART), as switch regimens in PLWH virally suppressed under etravirine‐based treatments (ETR‐ART).

**Methods**: SWEED is a national, multicentre, observational study enrolling adults PLWH with plasma viral load (pVL) <50 copies/ml under an ETR‐ART, who switched to a DOR‐ART (with no changes in number of associated drugs), from 01/09/2019, in 10 French hospitals. The primary outcome was the rate of virological failure (VF: confirmed pVL ≥50 copies/ml or single pVL ≥50 copies/ml with ART change) at week (W) 48. Secondary outcomes included: rate of strategy success rates (pVL <50 copies/ml with no ART change) at W48, occurrence of discontinuations due to side effects, changes in body weight and CD4 counts over the entire follow‐up.

**Results**: One hundred and nine patients were included, with 86 (79%) men, median (IQR) age: 59 years (53–65), ART duration: 25 years (20–27), duration of virological suppression: 13 years (9–17), CD4 count: 653/mm^3^ (463–858) (Table 1). ETR‐ART were two‐drug regimens in 58/109 (53%) patients, three‐drug regimen in 46/109 (42%), four‐ or more drug regimens in 5/109 (5%). Forty‐five of 109 (41%) patients had past VF on NNRTI; 42/91 (46%) had past documented viral mutations leading to resistance to at least one NNRTI. Median follow‐up after switch to DOR‐ART was 30 months (IQR 19–47). Only one VF occurred at W23 (pVL = 186 confirmed 382 copies/ml) under DOR+DTG+ATV/r; no genotype was performed at failure; pVL was resuppressed without ART change; virological failure rate was 0.9% (95% CI 0.0–5.0) at W48. There were four treatment discontinuations before W48 due to adverse events, leading to a strategy success rate of 91.7% (95% CI 84.9–96.2) at W48. No additional VF and 13 additional treatment discontinuations occurred between W48 and the end of follow‐up. There were no significant changes in body weight (+0.0 kg, *p* = 0.58) and CD4 counts (−14 cells/mm^3^, *p* = 0.88) over follow‐up.

**P122: Table 1:** Patient characteristics at inclusion (*n* = 109)

**Age**, years, median (IQR)	59 (53−65)
**Gender**, *n* (%)	
Male	86 (79)
Female	23 (21)
**Country of birth**, *n* (%)	
France	85 (78)
Other	24 (22)
**Transmission group**, *n* (%)	
Heterosexual	35 (32)
MSM	53 (49)
Other	21 (19)
**CDC stage C**, *n* (%)	33 (30)
**CD4 nadir**, cells/mm^3^ (IQR)	157 (83−243)
**Time from HIV diagnosis**, years, median (IQR)	29 (22−32)
**Time from ART initiation**, years, median (IQR)	25 (20−27)
**Previous virological failure**, *n* (%)	
Under NRTI	69 (63)
Under NNRTI	45 (41)
Under PI	43 (39)
Under INSTI	7 (6)
**Duration of viral suppression**, years, median (IQR)	13 (9−17)
**CD4 count**, cells/mm^3^ (IQR)	653 (463−858)
**CD4/CD8 ratio**, median (IQR)	0.8 (0.6−1.3)
**Body weight**, kg, median (IQR)	75 (68−88)
**Body mass index**, kg/m^2^, median (IQR)	24.4 (21.9−27.3)
**Kind of antiretroviral strategy including ETR and then DOR**, *n* (%)	
Two‐drug regimen	58^a^ (53)
Three‐drug regimen	46^b^ (42)
Four‐drug regimen	3 (3)
Five‐drug regimen	2 (2)

^a^Including 48 participants receiving ETR+RAL before switch.

^b^Including 31 participants receiving 2 INTIs+ETR before switch.

**Conclusions**: These findings suggest that doravirine can replace etravirine in virally suppressed PLWH, including those with prior VF with potential resistance to other NNRTIs. Its once‐daily dosing, good safety profile and absence of drug interactions are an advantage for patients.

### P123 Kinetics of non‐nucleosidic reverse transcriptase (NNRT) resistance‐associated mutations in HIV‐1 blood reservoir in NNRTI‐experienced people with HIV: the KINNDeR study

Thomas Drumel ^1^, Justine Sourice^1^, Colin Deschanvres^2^, Audrey Rodallec^1^, Clotilde Allavena^2^, François Raffi^2^, Elisabeth Garnier^1^

^1^Virology, Nantes University Hospital, Nantes, France. ^2^Infectiology, Nantes University Hospital, Nantes, France

**Background**: In blood mononuclear cells of people living with HIV (PLWH) with a history of virological failure (VF), some viral strains archived as proviral DNA can harbour mutations conferring resistance to antiretrovirals (ARVs). The objective of our study is to describe the temporal evolution of archived resistance‐associated mutations (RAMs) to non‐nucleosides (NNRTI) using next‐generation sequencing (NGS).

**Materials and methods**: This French single‐centre retrospective study included (i) PLWH ≥18 years old with a history of VF on an NNRTI‐containing regimen, (ii) a plasma genotype with one or more major NNRTI‐RAM detected by Sanger sequencing (RNA) at time of VF and (iii) one or two blood sample after virological suppression (VS) on subsequent ARV regimens (VS1, VS2, if available, at least 12 months apart). NGS target specific of the reverse transcriptase gene (ABL) and quantification of proviral DNA load (Biocentric) were performed on DNA extracts. Variables associated with persistence of NNRTI‐RAM in peripheral blood mononuclear cells (PBMCs) were assessed by Mann‐Whitney test.

**Results**: Eighty‐four patients were included: 62 with two NGS (VS1 and VS2) and 22 with one NGS (VS1). Sequencing was successful in 79 patients (94%) (Table 1). Among those, VS1 and VS2 were sampled after a median of 16 [IQR 11–18] and 20 years [IQR 15–22] post‐VF and after a median duration of VS of 10 [IQR 5–13] and 14 years [IQR 10–17], respectively. At VS1, no NNRTI‐RAM was detected in 40/79 (50.6%) of the sequences (threshold 2%). In univariate analysis, patients with persistent NNRTI‐RAM had a higher zenith viral load (*p* = 0.014), a higher current DNA viral load (*p* = 0.075) and a longer duration of replication under NNRTI (*p* = 0.091). At VS2, the detection of all NNRTI‐RAM by NGS decreased from a median of 28% to 8% (*p* < 0.001) and the median mutational DNA viral load decreased from 193 to 43 copies/106 PBMC (*p* < 0.001).

**P123: Table 1:** NNRTI‐RAM sequencing (NGS) and mutational viral load in PBMC during follow‐up of PLWH with prior NNRTI failure

		% (median % in NGS)	% (median % in NGS)	Mutational viral load^c^	Mutational viral load^c^
Mutation	VF (%) *N* = 84	VS1**^a^** *N* = 79	VS2**^b^** *N* = 62	VS1	VS2
K103N	47.6	22.8 (28)	22.6 (31)	189	119
G190A	29.8	11.4 (38)	14.5 (22)	159	108
Y181C	27.4	10.1 (40)	16.1 (18)	332	127
K101E	15.5	5.1 (18)	6.5 (36)	175	270
V106A	10.7	3.8 (74)	1.6 (20)	341	69
L100I	8.3	3.8 (23)	3.2 (53)	252	93
A98G	4.8	2.5 (6)	3.2 (17)	38	137
Y181I	4.8	1.3 (52)	0	700	/
H221Y	4.8	1.3 (96)	0	734	/

^a^NGS failure in five patients at VS1.

^b^Ten patients without follow up (VS2).

^c^Median copies/10^6^ PBMC.

**Conclusions**: Clearance of NNRTI archived resistance mutations is frequently achieved in long‐term virologically controlled patients. Further studies need to assess whether NGS and proviral mutational viral load can guide the prescription of a long‐acting NNRTI or a new‐generation NNRTI in virologically suppressed patients.

### P124 Evidence of resistance to integrase strand transfer inhibitors (INSTIS) among antiretroviral therapy‐naïve PLHIV in Greece during 2021−2024

Dimitrios Paraskevis^1^, Evangelia Georgia Kostaki ^1^, Eleni Papachristou^1^, Alexandros Vasilakis^1^, Chrysa Rokka^1^, Maria Chini^2^, Georgios Tsekes^2^, Anastasia Antoniadou^3^, Antonios Papadopoulos^3^, Magda Bletsa^1^, Triantafyllia Sotiriadou^1^, Mina Psichogiou^4^, Elpida Mastrogianni^4^, Vasileios Papastamopoulos^5^, Charisis Totsikas^5^, Georgios Adamis^6^, Myrto Astriti^6^, Dimitra Paraskeva^7^, Chrysa Tsiara^7^, Aikaterini Isari^7^, Foteini Giannou^7^, Magdalini Pylli^7^, Nikolaos Sipsas^8^, Olga Kosmopoulou^9^, Georgios Chrysos^10^, Helen Sambatakou^11^, Malvina Lada^12^, Periklis Panagopoulos^13^, Pagona Lagiou^1^, Gkikas Magiorkinis^1^

^1^Department of Hygiene, Epidemiology and Medical Statistics, Medical School, National and Kapodistrian University of Athens, Athens, Greece. ^2^3rd Department of Internal Medicine‐Infectious Diseases Unit, ‘Korgialeneio‐Benakeio’ Red Cross GH, Athens, Greece. ^3^4th Department of Medicine, Attikon GH, Medical School, National and Kapodistrian University of Athens, Athens, Greece. ^4^1st Department of Internal Medicine, Laiko GH, Medical School, National and Kapodistrian University of Athens, Athens, Greece. ^5^5th Department of Internal Medicine and Infectious Diseases, Evaggelismos GH, Athens, Greece. ^6^1st Department of Internal Medicine, G. Genimatas GH, Athens, Greece. ^7^Directorate for HIV/AIDS, STDs and Hepatitis Prevention and Epidemiological Surveillance, Hellenic National Public Health Organization, Marousi, Greece. ^8^Department of Pathophysiology, Laiko GH, Medical School, National and Kapodistrian University of Athens, Athens, Greece. ^9^1st Department of Internal Medicine, Nikaia GH, Piraeus, Greece. ^10^Department of Internal Medicine, Tzaneio GH, Piraeus, Greece. ^11^HIV Unit, 2nd Department of Internal Medicine, Hippokration GH, Medical School, National and Kapodistrian University of Athens, Athens, Greece. ^12^2nd Department of Internal Medicine, Sismanogleion GH, Marousi, Greece. ^13^Department of Internal Medicine, University GH, Democritus University of Thrace, Alexandroupolis, Greece

**Background: ** Integrase strand transfer inhibitors (INSTIs) are antiretroviral agents that are recommended for the first‐line treatment of HIV‐1. HIV‐1 drug resistance in the era of INSTIs is a global concern. We aimed to estimate the prevalence of drug resistance and specifically the major and accessory resistance mutations associated with INSTIs, in newly diagnosed people living with HIV (PLWH) naive to antiretroviral therapy (ART) in Greece.

**Materials and methods**: Our study sample consisted of 865 sequences from Southern and Central Greece, obtained using Illumina next‐generation sequencing or Sanger sequencing. We analysed integrase gene sequences from ART‐naïve PLHIV who were diagnosed between 2021 and mid‐2024. During this period, resistance testing for INSTIs was performed for all newly diagnosed PLHIV. The prevalence of major and accessory resistance mutations and levels of associated resistance to INSTIs were estimated on the study sequences using the HIVdb programme available on the Stanford University HIV drug resistance database.

**Results**: The overall prevalence of resistance to INSTIs was 5.78% (50/865). Major INSTIs resistance mutations were detected at a prevalence of 0.81% (7/865). Major INSTIs mutations included N155H (2/865, 0.23%), E138K (2/865, 0.23%), E138A (1/865, 0.12%), Y143C (1/865, 0.12%) and S147G (1/865, 0.12%). Accessory INSTIs resistance mutations were detected at a higher prevalence equal to 5.32% (46/865) and included T97A (22/865, 2.54%), L74M (19/865, 2.20%) and S153F (10/865, 1.16%). According to the HIVdb programme, the estimated levels of resistance were as follows: 17 of 865 (2.0%) for bictegravir (BIC), 32 of 865 (3.7%) for cabotegravir (CAB), 17 of 865 (2.0%) for dolutegravir (DTG), 38 of 865 (4.4%) for elvitegravir (EVG) and 38 of 865 (4.4%) for raltegravir (RTG).

**Conclusions**: We present the most recent data on drug resistance to integrase‐based first‐line regimens of HIV‐1 in Greece. Our study provides evidence that major or accessory resistance mutations are detectable among ART‐naïve PLWH in Greece. Although the prevalence of resistance to INSTIs is low, our findings highlight the necessity of resistance testing for INSTIs among newly diagnosed PLHIV.

### P125 Efficacy and safety of dolutegravir (DTG)‐based antiretroviral treatment (ART) in patients with HIV and solid organ transplantation (SOT): a single‐arm clinical trial (DTG‐SOT)

Jose M. Miró ^1^, Daniela Malano Barletta^2^, Leire Berrocal^2^, Christian Manzardo^2^, Anna Castelli^2^, Merce Brunet^3^, Octavi Roman^2^, Juan Ambrosioni^1^, Frederic Cofan^4^, Angela Gonzalez^4^, Pablo Ruiz^5^, Gonzalo Crespo^5^, Alejandro Forner^5^, Maria Angeles Castel^6^, Montserrat Laguno^1^, Montserrat Tuset^7^, Elisa de Lazzari^2^, Antonio Rimola^5^, Asuncion Moreno^2^

^1^Infectious Diseases Service—HIV/AIDS Unit, Hospital Clinic‐IDIBAPS‐University of Barcelona‐CIBERINFEC, Barcelona, Spain. ^2^Infectious Diseases Service—HIV/AIDS Unit, Hospital Clinic‐IDIBAPS‐University of Barcelona, Barcelona, Spain. ^3^Toxicology Service, Hospital Clinic‐IDIBAPS‐University of Barcelona, Barcelona, Spain. ^4^Nephrology Service—Kidney Transplant Unit, Hospital Clinic‐IDIBAPS‐University of Barcelona, Barcelona, Spain. ^5^Hepatology Service—Liver Transplant Unit, Hospital Clinic‐IDIBAPS‐University of Barcelona, Barcelona, Spain. ^6^Cardiology Service—Heart Transplant Unit, Hospital Clinic‐IDIBAPS‐University of Barcelona, Barcelona, Spain. ^7^Pharmacy Department, Hospital Clinic‐IDIBAPS‐University of Barcelona, Barcelona, Spain

**Background**: We demonstrated that DTG‐based ART did not change tacrolimus and mycophenolic acid pharmacokinetic profiles in patients with HIV (PWH) and SOT [1]. However, there is limited clinical information on DTG use in the transplantation setting since raltegravir (RAL)‐based ART is commonly used. The hypothesis for switching from RAL to DTG was to move to a more convenient ART‐regimen while maintaining/improving efficacy and tolerance. However, in the only published study, five (50%) of the 10 HIV‐positive liver transplant (LT) recipients in whom ART was switched to DTG for simplification returned 1 year later to the previous ART regimens due to adverse events (AEs) [2]. The aim of this trial was to know the efficacy and safety of DTG (plus two NRTIs) in PWH and SOT.

**Methods**: Single‐arm trial including consecutive HIV‐infected SOT adult recipients on stable and effective RAL‐based ART who were switched to DTG‐based ART and were followed‐up for 48 weeks. Patients had plasma HIV viral load (VL) <50 copies/ml during ≥12 months. DTG was combined with tenofovir disoproxil fumarate (TDF)/emtricitabine (FTC) or lamivudine (3TC)/abacavir (ABC). Primary end point: plasma HIV VL <50 copies/ml at 48 weeks. Secondary end points: CD4 counts evolution and treatment discontinuation rates. ClinicalTrials.gov Identifier: NCT03360682.

**Results**: We included 19 PWH (median [IQR] 57 years [51–60]), 58% were males, SOT type: liver (*n* = 12); kidney (*n* = 6); heart (*n* = 1). ART was 3TC/ABC/DTG in 63% and FTC/TDF/DTG in 37%. All patients (100%) remained suppressed (VL <50 copies/ml) at 48 weeks. There were no changes in CD4 counts (*p* = 0.4193) (Figure 1‐top) or percentages (*p* = 0.5155), total cholesterol (*p* = 0.0686), LDL‐cholesterol (*p* = 0.7384), HDL‐cholesterol (*p* = 0.1373) or triglycerides (*p* = 0.7476) during follow‐up. Although the estimated glomerular filtration (eGF) rate slightly decreased (*p* = 0.0015) (Figure 1‐bottom) and creatinine slightly increased (*p* = 0.0001), these changes were not clinically relevant. Protein/creatinine ratios remained unchanged (*p* = 0.6379), with no significant changes in liver enzymes or glucose. Three (16%) patients discontinued DTG‐treatment due to AEs (neuropsychological alterations [*n* = 2], worsening diabetes [*n* = 1]). No patients experienced organ rejection during the study.

**Conclusions**: Switching to DTG‐based ART was effective in PWH and SOT. More studies are needed to evaluate DTG safety in this setting.

**P125: Figure 1:**
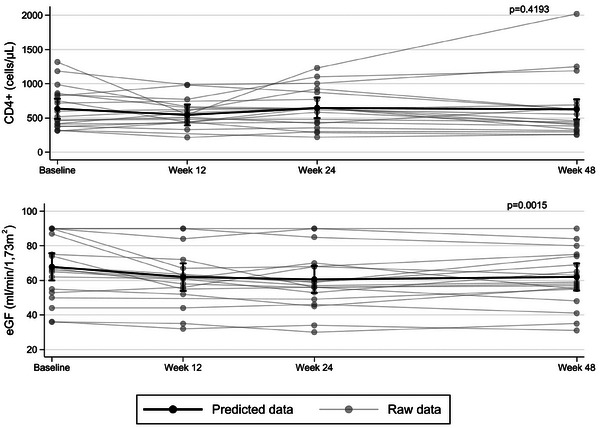
Evolution of CD4^+^ cell counts (top) and the estimated glomerular filtration (eGF) rate (bottom) in PWH and SOT receiving DTG‐based ART throughout the study. The grey lines represent individual values and the black lines the predicted values with the corresponding 95% confidence intervals (CI).

**References**

1. Manzardo C, Castelli A, Brunet M, Roman O, Ambrosioni J, Cofan F et al. Drug‐drug interactions between dolutegravir (DTG) and immunosuppressant drugs (IS) in HIV‐infected patients with solid organ transplantation (SOT): a single‐arm clinical trial (DTG‐SOT). 17th European AIDS Conference (EACS). November 6–9, 2019. Basel, Switzerland. Poster PE27/7.

2. Cattaneo D, Sollima S, Meraviglia P, Milazzo L, Minisci D, Fusi M, et al. Dolutegravir‐based antiretroviral regimens for HIV liver transplant patients in real‐life settings. Drugs R D. 2020;20:155‐160.

### P126 Differentiating APOBEC‐ and drug‐resistant populations by clonal analysis of near full‐length proviral HIV‐1 genome in highly treatment‐experienced people living with HIV

Nina Engel^1^, Axelander Thielen^1^, Martin Däumer^1^, Jens Verheyen^1^, Roger Vogelmann ^2^

^1^Institute of Immunology and Genetics, Labor Thiele, Kaiserslautern, Germany. ^2^Universitätsmedizin Mannheim, II. Medizinische Klinik, Mannheim, Germany

**Background**: HIV‐1 genotypic‐resistance testing from proviral DNA can be beneficial in switch settings where therapy history is incomplete and/or viral RNA is undetectable. However, the interpretation of a proviral‐resistance profile can be challenging, since apolipoprotein B mRNA editing enzyme, catalytic polypeptide (APOBEC)‐induced mutations may also appear at drug‐resistance‐associated positions and the majority of proviral variants is defective (>90–95%). Here, we describe a near‐full‐length amplification and long‐read sequencing approach to discriminate populations with APOBEC mutations from replication‐competent populations by covariation analysis in highly treatment‐experienced PLWH.

**Materials and methods**: Seventeen heavily treatment experienced and virologically suppressed (median 10 years) PLWH were included in this study. A single round, near full‐length PCR was done from HIV‐1 proviral DNA followed by long‐read sequencing using nanopore technology. Covariation analysis was used to extract linkage information of mutations. Potential APOBEC mutations in protease, reverse transcriptase and integrase were determined using Stanford HIVdb. Replication incompetence of individual variants was defined as the presence of premature stop codons found anywhere in the genome.

**Results**: No APOBEC‐induced premature stop codons were observed in 7/17 samples, indicating a fully replication‐competent population. One sample showed a population with stop codons in >99% of the genomes, indicating almost complete replication incompetence. Mixed populations with and without premature stop codons were detected in 9/17 samples. These showed a total of 89 mutations at resistance‐associated positions with frequencies between 1% and 99%. In 8/9 samples, drug‐resistant populations were replication‐competent with frequencies of replicative viruses between 0.2% and 89%. By using mutation linkage analysis within individual viruses, mutations of ambiguous origin such as M184I or M230I in the reverse transcriptase could clearly be classified as resistance‐ or APOBEC‐associated.

**Conclusions**: By using near‐full‐length amplification of the HIV‐1 genome in combination with long‐read sequencing, we were able to discriminate APOBEC from potential replication competent populations in the proviral DNA. All 17 PLWH were virologically suppressed despite critical resistance profiles in some cases, indicating that APOBEC‐related stop codons may have an impact on viral replication competence of drug‐resistant variants. The characterization of a proviral population in terms of replication competence might be helpful in ART switch settings.

### P127 Bictegravir/emtricitabine/tenofovir alafenamide (Biktarvy^®^/BFTAF) in patients with baseline nucleoside reverse transcriptase inhibitor (NRTI) mutations

Jane Akodu ^1^, Zoe Agyemang^1^, Gavin Marshall^1^, Alan Hunter^1^, Pedro Simoes^1^, Jennifer Hart^2^, Fiona Burns^1^, Tristan J. Barber^1^

^1^Ian Charleson Day Centre, Royal Free London Hospitals, London, UK. ^2^Virology Department, Royal Free London Hospitals, London, UK

**Background**: BFTAF is licensed for antiretroviral therapy in adults with HIV‐1 without present/past evidence of resistance to its component agents. Despite this, there is evidence that BFTAF may be effective in those with NRTI mutations. We reviewed viral outcomes in people with NRTI‐resistance mutations in our clinic who received BFTAF between 2019 and 2023 after multidisciplinary team (MDT) approval.

**Methods**: Adults prescribed BFTAF 22/11/2019–30/11/2023 with baseline NRTI resistance were included. Demographic data, treatment naïve versus switch, mutations and viral loads were recorded.

**Results**: One hundred and sixty‐four people were identified (78% male, 58% White). One individual with transmitted M184V only was naïve to ART and initiated BFTAF, became virally suppressed by week 3 and has remained so on BFTAF. One hundred and sixty‐three of 164 were switched to BFTAF, 38/163 (23%) had a detectable viral load (>40 c/ml) at time of switch. Follow‐up since switch was 22–1424 days. Sixteen discontinued BFTAF (9.8%); 13/16 (81.3%) due to side effects, 1/16 (6.3%) due to low‐level viraemia, one for drug interactions, one due to poor adherence. Those who discontinued BFTAF were excluded from the final virological analysis. One hundred and forty‐seven remain on BFTAF. One hundred and seventeen of 147 have follow up of ≥24 weeks; 108/117 (92%) suppressed (<40 c/ml). Nine with detectable viral load had long standing issues with adherence. Four have had resistance tests (three nil new, one evolved two minor INSTI mutations—S230R and E157Q, with planned switch off BFTAF following MDT discussion); five had viral blips 49–155 copies/ml. Follow‐up data on 30 people does not yet extend to 6 months; however, of those who had post switch safety bloods, 22/30 (73%) had <40 c/ml (Table 1 and Figure 1).

**P127: Table 1:** Prevalence of NRTI mutations

	Common NRTI RAM	Selected ART	Prevalence, *n* = 163
Discriminatory mutations	M184VI	XTC	114
Discriminatory mutations	K65R	XTC + TDF	7
Discriminatory mutations	K70E	TDF	2
Discriminatory mutations	L74VI	ABC	7
Discriminatory mutations	Y115F	TDF	1
Thymidine analogue mutations (TAMs)	M41L	TDF	35
Thymidine analogue mutations (TAMs)	D67N	AZT	37
Thymidine analogue mutations (TAMs)	K70R	TDF	30
Thymidine analogue mutations (TAMs)	L210W	TDF	14
Thymidine analogue mutations (TAMs)	T215Y	TDF	45
Thymidine analogue mutations (TAMs)	K219QE	AZT	28
MDR mutations	T69ins	XTC +TDF	14
MDR mutations	Q151M	XTC +TDF	2
Minor mutations	D67G	AZT	3
Minor mutations	K219NR	AZT	5
Minor mutations	V75MA	AZT	4
Minor mutations	T215DEISV	AZT	5

Abbreviation: MDR, multi‐drug (nucleotide) resistance.

**Conclusions: **BFTAF has been used in people with a broad range of NRTI mutations in our clinic of which the most common was M184I/V in isolation; smaller numbers had multiple mutations including seven with K65R. Viral suppression was maintained in 108/117 (92%) of those with NRTI mutations remaining on BFTAF and with follow up of at least 24 weeks.

**P127: Figure 1:**
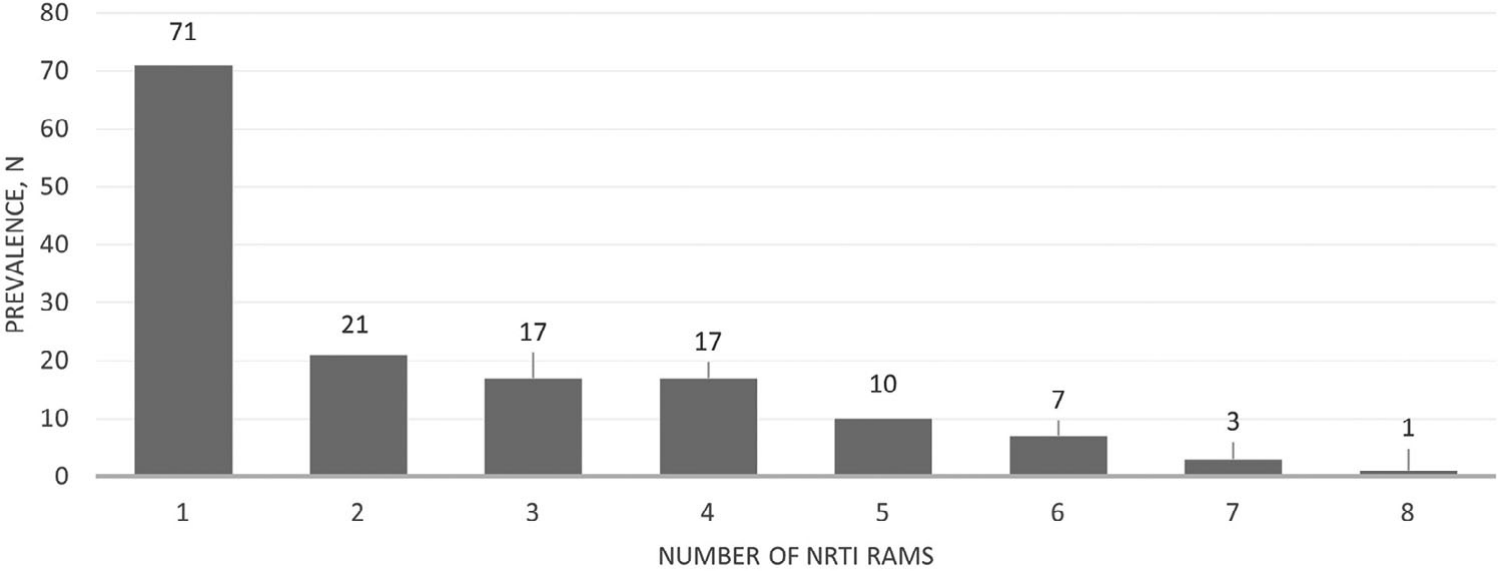
Prevalence according to the number of resistance‐associated mutations per patient.

### P128 Long‐term durability of rilpivirine + darunavir/cobicistat dual regimen in antiretroviral‐experienced people living with HIV (RilDaco study)

Diego Ripamonti ^1^, Laura Comi^1^, Federica Borghi^1^, Serena Venturelli^1^, Daniela Valenti^2^, Andrea Francavilla^2^

^1^Infectious Diseases, ASST Papa Giovanni XXIII, Bergamo, Italy. ^2^FROM, Fondazione per la Ricerca Ospedale di Bergamo—ETS, Bergamo, Italy

**Background**: Two‐drug regimens based on a high genetic barrier agent (as dolutegravir or boosted protease inhibitor) are currently used in naïve and switch strategies. Rilpivirine (RPV) plus darunavir‐cobicistat (DRV‐c) may be useful in people living with HIV (PLWH) when the integrase inhibitor (INSTI) is contraindicated for intolerance, toxicity or resistance issues. We report on durability of this combination assessed by treatment failure (TF) over time.

**Materials and methods**: This is a retrospective, observational, single‐centre study in virologically suppressed PLWH switched to RPV+DRV‐c for any reasons. The primary composite end point was TF defined as any reason of discontinuation, including virological failure (VF) (HIV RNA above 50 copies/ml or any values followed by a switch). Survival analysis with Kaplan‐Meier estimator was used to assess the probability of treatment discontinuation over time. Multiple logistic regression was used to estimate the probability of TF at 1 year following the initiation of RPV+DRV‐c.

**Results**: A total of 97 individuals were included (80% males, median age 57 years, median nadir CD4^+^ T cell 260 cells/ml, 15% with AIDS, 32% had 1–2 previous treatment lines). At study entry, 87% and 99% of individuals had CD4^+^ T‐cell count >500 cells/ml and HIV RNA <50 c/ml, respectively. Among those whose genotype tests were available, only 9% and 5% had NRTI‐ and NNRTI‐associated mutations, respectively. Median follow‐up was 69 (IQR 66–73) months since RPV+DRV‐c initiation. The probability of non‐TF was 96%, 87%, 77%, 67%, 62% and 52% after 6, 12, 24, 36, 48 and 60 months of treatment, respectively (Figure 1). Among the 54 (55%) subjects who discontinued treatment during follow‐up, 37%, 19%, 17% and 9% switched therapy for dyslipidaemia, drug‐interaction, simplification and side effects, respectively. Only one participant discontinued for a viral blip. At multiple logistic regression age, sex and baseline CD4 count were not predictors of TF at 1 year (*p* > 0.05 for all).

**Conclusions**: RPV+DRV‐c is an effective and durable combination in switch strategy. Most reasons of discontinuations were other than VF (mainly metabolic issues or drug‐interactions), confirming the virological efficacy for those not eligible to INSTI‐based options.

**P128: Figure 1:**
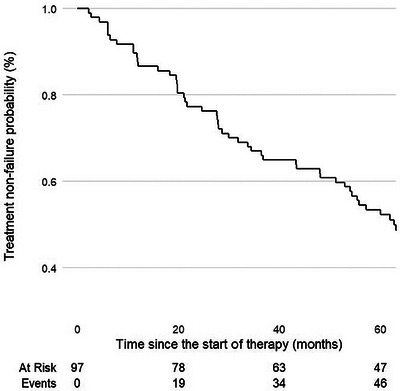
Probability of treatment discontinuation over time.

### P129 Long‐term efficacy and safety of a maintenance 3‐day‐per‐week schedule with the single tablet regimen efavirenz/emtricitabine/tenofovir disoproxil fumarate

Beatriz Borjabad ^1^, Alexy Inciarte^2^, Ivan Chivite^2^, Anna González‐Cordon^2^, Maria del Mar Mosquera^3^, Berta Torres^2^, Lorena De La Mora^2^, Maria Martinez‐Rebollar^2^, Montserrat Laguno^2^, Alberto Foncillas^2^, Juan Ambrosioni^2^, Josep Mallolas^2^, Cristina Rovira^2^, Carmen Hurtado^2^, Abiu Sempere^2^, Julia Calvo^2^, Leire Berrocal^2^, Jose M. Miró^2^, Jose Alcamí^4^, Jose Luis Blanco‐Arevalo^2^, Sonsoles Sanchez‐Palomino^2^, Elisa de Lazzari^2^, Esteban Martinez^2^

^1^Internal Medicine, Hospital Moisès Broggi, Sant Joan Despí, Spain. ^2^Infectious Diseases Unit, Hospital Clínic, Barcelona, Spain. ^3^Microbiology Department, Hospital Clínic—Instituto de Salud Global de Barcelona (ISGLOBAL), Barcelona, Spain. ^4^Infectious Diseases, Institute of Health Carlos III, Consorcio Centro de Investigación Biomédica en Red MP (CIBER), Madrid, Spain

**Background**: Due to the long half‐life of its components, we hypothesized that a maintenance schedule of 3‐day‐per‐week efavirenz/emtricitabine/tenofovir‐disoproxil‐fumarate would maintain virological efficacy while reduce toxicity in the long‐term.

**Materials and methods**: After an initial 24‐week randomized phase [1], all A‐TRI‐WEEK trial (ClinicalTrials.gov, NCT01778413) participants were offered to switch to the 3‐day‐per‐week strategy. HIV‐RNA, CD4/CD8 cells, and blood and urine chemistries were collected every 6 months. Lumbar/hip bone mineral densities (BMDs) were screened once a year. Treatment failure was defined as virological failure (confirmed HIV RNA ≥50 copies/ml), therapy discontinuation for any reason or lost to follow‐up. Secondary outcomes were changes in CD4/CD8 cells, plasma lipids, estimated glomerular filtration rate (eGFR, Chronic Kidney Disease Epidemiology Collaboration equation [CKD‐EPI]), urine protein/creatinine and lumbar/femur BMD.

**Results**: Of 61 persons initially enrolled, 59 (97%) accepted to participate in the extension phase. Most of participants were men (*n* = 53, 90%). Median (IQR) baseline values were: age 56 years (IQR 46–60); CD4 563 (457–697) and CD8 569 (447–703) cells/mm^3^; total, LDL and HDL cholesterol 194 (168–218), 124 (117–131) and 47 (40–55) mg/dl; eGFR 94 (92–97) ml/minute/1.73 m^2^ and urine protein/creatinine 80 (59–100) mg/g; lumbar and hip BMD were 0.93 (0.90–0.96) and 0.96 (0.93–0.99) g/cm^2^, respectively. After a median of 7 years of follow‐up, 37 persons (62%) remained free of treatment failure. Twenty‐two (37%) persons discontinued antiretroviral regimen due to: persistent CNS symptoms (*n* = 7), decreasing bone mineral density (*n* = 7), risk of interactions (*n* = 2), preference for other regimens (*n* = 2) and neoplasia, virological failure, non‐availability of medication due to travel, and death (end‐stage liver disease, unrelated to antiretroviral therapy). Median (IQR) CD4 and CD8 changes at 7 years were +91 (31–151) and +125 (53–197) cells/mm^3^. Total, LDL and HDL cholesterol remained stable over time. Triglycerides, eGFR, urine protein/creatinine, and lumbar and hip BMD showed a bimodal curve with stable values in the first half of follow‐up, and increases (triglycerides, urine protein/creatinine) or decreases (eGFR and BMD) in the second half of follow‐up. Seventy‐five percent of discontinuations occurred in the second half of follow‐up.

**Conclusions**: Three‐day‐per‐week efavirenz/emtricitabine/tenofovir‐disoproxil‐fumarate as a maintenance therapeutic strategy was effective in the long‐term and deferred toxicity.

**Reference**

1. Rojas J, Blanco JL, Sanchez‐Palomino S, Marcos MA, Guardo AC, Gonzalez‐Cordon A, et al. A maintenance 3‐day‐per‐week schedule with the single tablet regimen efavirenz/emtricitabine/tenofovir disoproxil fumarate is effective and decreases sub‐clinical toxicity. AIDS. 2018;32(12):1633–41.

### P130 Evaluation of HIV‐DNA resistance evolution in highly treatment‐experienced and multi‐resistant individuals under virological control: a longitudinal study from the PRESTIGIO registry

Daniele Armenia^1^, Vincenzo Spagnuolo^2^, Maria Concetta Bellocchi^3^, Laura Galli^2^, Greta Marchegiani^3^, Tommaso Clemente^2^, Luca Carioti^3^, Riccardo Lolatto^2^, Micol Ferrara^4^, Roberta Gagliardini^5^, Giulia Carla Marchetti^6^, Carlo Torti^7^, Giuseppe De Socio^8^, Chiara Fornabaio^9^, Maurizio Zazzi^10^, Antonella Castagna^2^, Maria Mercedes Santoro ^3^

^1^Departmental Faculty, Saint Camillus International University of Health Sciences, Rome, Italy. ^2^Clinic of Infectious Diseases, Istituto Scientifico San Raffaele, Milan, Italy. ^3^Department of Experimental Medicine, University of Rome, Rome, Italy. ^4^Department of Medical Sciences, Unit of Infectious Diseases, University of Turin, Turin, Italy. ^5^Clinical Department, National Institute for Infectious Diseases, Rome, Italy. ^6^Clinic of Infectious Diseases, Department of Health Sciences, San Paolo Hospital, Azienda Socio Sanitaria Territoriale (ASST) Santi Paolo e Carlo, University of Milan, Milan, Italy. ^7^Dipartimento di Scienze di Laboratorio e Infettivologiche, Fondazione Policlinico Universitario, Rome, Italy. ^8^Unit of Infectious Diseases, Santa Maria Hospital, Perugia, Italy. ^9^Infectious Diseases Unit, Cremona Azienda Socio Sanitaria Territoriale (ASST) Hospital, Cremona, Italy. ^10^Department of Medical Biotechnologies, University of Siena, Siena, Italy

**Background**: This study aimed to clarify whether resistance detected in HIV‐DNA might evolve in virologically suppressed highly treatment‐experienced (HTE) individuals with multidrug resistance (MDR).

**Methods**: Twenty‐three HTE MDR individuals from the PRESTIGIO registry (https://registroprestigio.org/project) with two available peripheral blood mononuclear cell (PBMC) longitudinal samples (T0–T1) were analysed. T1 was collected after 12 months (median [IQR]: 12.8 [11.8–13.6]) from T0 under a suppressive regimen. HIV‐DNA levels were quantified through droplet digital PCR and HIV‐DNA resistance was assessed through next‐generation sequencing (NGS, Illumina‐MiSeq) set at 5%. Major‐resistance mutations (MRMs) were evaluated according to HIVdb ver 9.5.1.

**Results**: At T0, individuals had been exposed to ART for 22 (21–24) years and virologically suppressed for 42 (31–61) months under a salvage regimen mostly containing dolutegravir (95.7%) and/or darunavir (69.6%). The median (IQR) HIV‐DNA was 2588 (929–5122) copies/10^6^ CD4^+^ cells at T0 and did not significantly change at T1 (2322 [1521–4138] copies/10^6^ CD4^+^ cells, *p* = 0.855). In HIV‐DNA, the prevalence of ≥3‐class resistance was 87.0% and slightly decreased at T1 (78.2%, *p* = 0.607). Also, the number of any MRM and class‐specific MRM did not significantly change over time (Table 1). Concerning specific MRM, the prevalence of several reverse‐transcriptase inhibitor (RTI) mutations decreased over time (Figure 1, panel 1), but a significant decrease (T1–T0) was observed only for M184V (T0: 91.3%, T1: 60.9%, *p* = 0.016) and M41L (T0: 87.0, T1: 56.5%; *p* = 0.016). Individuals who lost M184V had a significantly lower mutational load at T0 compared to those who had the mutation persistently detectable (Figure 1, panel 2). M184V was more likely to be cleared in individuals with mutational load <1000 versus ≥1000 copies/10^6^ CD4^+^ cells at T0 (7 out of 13, 53.8% vs. 0 out of 8, 0%; *p* = 0.018). This phenomenon was not observed for M41L (Figure 1, panel 3).

**P130: Table 1:** Number of major‐resistance mutations (MRMs) in longitudinal PBMC samples of virologically suppressed HTE‐MDR PWH

MRM detected with NGS‐GRT set at 5%, median (IQR)	T0	T1	*p*‐value (Wilcoxon test for matched pairs)
Any MRM	12 (10−16)	13 (8−14)	0.384
PI	6 (2−7)	6 (2−6)	0.617
NRTI	6 (4−6)	4 (2−7)	0.170
NNRTI	2 (1−3)	1 (0−2)	0.153
INSTI	1 (0−1)	1 (0−1)	0.822

Abbreviation: NGS‐GRT, next‐generation sequencing genotype resistance testing.

**Conclusions**: Within 1 year of observation in stably suppressed HTE‐MDR PWH, there is minimal evolution of MRM. M184V uniquely declined over time and the decline was associated with low mutational load at the first time point evaluated. Therefore, assessing the M184V burden might help to identify individuals more prone to lose it.

**P130: Figure 1:**
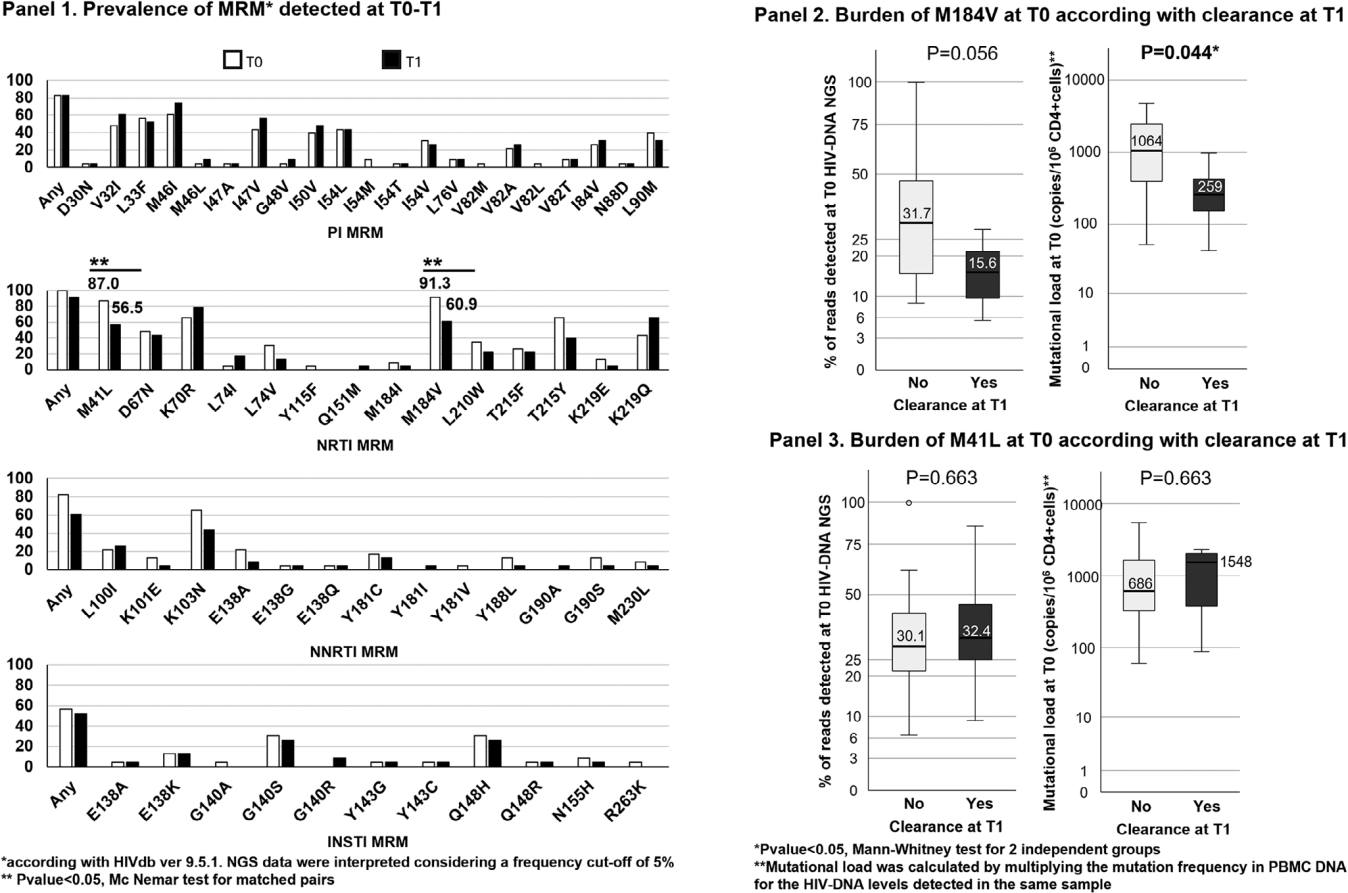
Overview of major‐resistance mutations (MRMs) prevalence detected through HIV‐DNA NGS from longitudinal PBMC samples of HTE‐MDR PWH.

### P131 Low prevalence of protease inhibitor mutations among people living with HIV in Botswana: insights from the Botswana Combination Prevention Project

Marea Pema, Dorcas Maruapula, Doreen Ditshwanelo, Natasha Moraka, Nokuthula Ndlovu, Ontlametse Bareng, Patrick Mokgethi, Wonderful Choga, Boitumelo Zuze, Catherine Koofhethile, Sikhulile Moyo, Simani Gaseitsiwe

Research Lab, Botswana Harvard Health Partnership, Gaborone, Botswana

**Background**: The use of protease inhibitors (PIs) as part of second‐line antiretroviral therapy (ART) in Botswana has contributed to the management of HIV; however, the presence of PI resistance‐associated mutations (RAMs) may limit the ART treatment options. There is limited data on the prevalence of PI RAMs in Botswana. We investigated the baseline prevalence of PI RAMs in ART‐naïve and ART‐experienced participants from the Botswana Combination Prevention Project (BCPP) which recruited people living with HIV (PLWH) from 2013 to 2018.

**Methods**: A total of 6075 proviral HIV‐1 *pol* available sequences were used to screen for PI RAMs. All sequences were screened for hypermutations using the Los Alamos database‐Hypermut tool. The Stanford University HIV Drug Resistance Database was used to interpret PI RAMs for both ART naïve and experienced populations.

**Results**: Out of 6075 PLWH, 1281 (21.1%) were from ART naïve, 4779 (78.7%) were ART‐experienced and 15 (0.25%) had no ART status. The overall prevalence of PI RAMs for those with ART status was 0.68% (41/6060). Among the ART‐naïve participants, the PI RAMs prevalence was 0.39% (5/1281), while 0.75% (36/4779) was observed in ART‐experienced participants. The PI RAMs observed among the ART‐naïve group were M46I (0.16%, *n* = 2), L90M (0.16%, *n* = 2), D30N (0.08%, *n* = 1) and M46L (0.08%, *n* = 1). Among the ART‐experienced participants, the PI RAMs observed were M46I (0.36%, *n* = 17), D30N (0.33%, *n* = 16), I54V (0.04%, *n* = 2), V82A (0.04%, *n* = 2), L76V (0.02%, *n* = 1) and M46L (0.19%, *n* = 9). Two hundred and sixty‐eight participants were on PI regimen of which 22 were experiencing virological failure (VF) (HIV viral load [VL] >400 copies/ml) after being on PI‐based ART for at least 3 months and these participants did not have PI RAMs (Table 1). When stratified by HIV VL, three participants had a VL ranging from 401 to 1000 copies/ml, while 19 participants had a viral load >1000 copies/ml.

**P131: Table 1:** HIV DRMs detected in individuals who were experiencing virological failure while on PIs

		Frequency of DRMs in participants experiencing virological failure
Drug classes	DRMs	*n* (%)
NRTI	V75I	1 (0.37%)
NRTI	M184V	9 (3.36%)
NRTI	D67N	2 (0.75)
NRTI	K70R	2 (0.75)
NRTI	T215I	1 (0.37%)
NRTI	K219E	2 (0.75)
NRTI	T215F	1 (0.37%)
NNRTI	A98G	1 (0.37%)
NNRTI	K103N	5 (1.87%)
NNRTI	Y188L	1 (0.37%)
NNRTI	E138A	4 (1.49)
NNRTI	K103S	1 (0.37%)
NNRTI	P225H	1 (0.37%)
NNRTI	V108I	1 (0.37%)
NNRTI	M230I	1 (0.37%)
NNRTI	V106A	1 (0.37%)
NNRTI	F227L	1 (0.37%)

Abbreviations: DRMs, drug‐resistance mutations; *n*, participants experiencing virological failure; *n*, the total number of participants on PIs; NNRTI, non‐nucleoside reverse transcriptase inhibitor; NRTI, nucleoside reverse transcriptase inhibitor; PI, protease inhibitor.

**Conclusions**: The overall PI prevalence was relatively low at 0.72% in the BCPP cohort. Future studies are warranted to determine RAMs in the HIV *gag* region for individuals who are experiencing VF on PI‐based ART without PI RAMs.

### P132 Three‐ versus two‐drug doravirine‐based regimens: a multicentre observational study

Adriana Cervo ^1^, Andrea Giacomelli^2^, Roberto Rossotti^3^, Marianna Menozzi^1^, Beatrice Fontana^1^, Elena Martini^1^, Giacomo Menegotto^1^, Maddalena Albertini^1^, Andrea Rabbione^4^, Maria Vittoria Cossu^4^, Davide Moschese^4^, Cristina Gervasoni^4^, Giovanni Guaraldi^1^

^1^Infectious Diseases Department, University Hospital of Modena, Modena, Italy. ^2^Department of Biomedical and Clinical Sciences, University of Milan, Milan, Italy. ^3^Infectious Diseases Department, ASST Grande Ospedale Metropolitano Niguarda, Milan, Italy. ^4^Infectious Diseases Department, ASST Fatebenefratelli Luigi Sacco Hospital, Milan, Italy

**Background**: The aim of this study was to compare the effectiveness of three‐drug (3DR) versus two‐drug (2DR) doravirine (DOR)‐based regimens.

**Methods**: Retrospective multicentric study including treatment‐experienced people with HIV (PWH) who started regimen with DOR/lamivudine (3TC)/tenofovir dysoproxil (TDF) (single tablet regimen) or DOR+3TC. Demographics, HIV‐related characteristics and metabolic parameters were compared. Reasons for treatment discontinuation and virological failure (VF), defined as HIV‐RNA >200 copies/ml for two consecutive measurements or HIV‐RNA >1000 copies/ml, were analysed.

**Results**: Two hundred and eighteen individuals were included: 171 (78.8%) in 3DR and 47 (21.6%) in 2DR. Clinical characteristics are shown in Table 1. PWH on DOR+3TC were older and with longer history of HIV infection and antiretrovirals exposure (*p* = 0.01). Reasons for 2DR switch were mainly driven by metabolic issues and psychiatric symptoms. Lipids and creatinine values at baseline were similar in the two groups, while hypertension, diabetes, osteopenia/osteoporosis and chronic kidney disease were more prevalent in the DOR+3TC group. Discontinuations were 59 (35%) in DOR/3TC/TDF and 12 (25.5%) in DOR+3TC (logrank = 0.834) (Figure 1), with median time to discontinuation of 18.2 months (2.1–40.8) versus 5.8 months (0.13–32.1) (*p* = 0.017). Main reasons for discontinuation were toxicity/intolerance (24/59 [40.6%] in 3DR vs. 5/12 [41.6%] in 2DR) (*p* = 0.597), reduction in pill/drug burden (18/59 [30.5%] vs. 2/12 [16.6%]) (*p* = 0.276) and virological failure/increase of genetic barrier (9/59 [15.3%] vs. 5/12 [41.6%]) (*p* = 0.051). During 25 months (2.8–44.3) follow‐up period, three VF (1.7%) occurred in the 3DR group leading to discontinuation and emergence of resistance mutations in two. During a follow‐up of 23.8 months (2.8–37.7), one VF (2.1%) was reported in the 2DR group with emergence of N348I (with no impact on DOR susceptibility), while two blips led to treatment intensification. Genotypic susceptibility score <2 was reported in seven out of 39 individuals with available genotypic‐resistance test on 2DR mainly for the presence of archived M184V but three individuals showed also DOR mutations: of these, four maintained virological suppression until last available follow‐up (median 19.2 months).

**P132: Table 1:** Characteristics of PWH on DOR/3TC/TDF versus DOR/3TC

	DOR/3TC/TDF (*n* = 171)	DOR/3TC (*n* = 47)	*p*‐value
Age (years), median (IQR)	50.9 (31.2−68.6)	55.9 (32.4−74.5)	0.0199
Males, *n* (%)	117 (68.0)	35 (74.5)	0.271
Ethnicity, *n* (%)			0.001
Caucasian	132 (77.2)	43 (91.5)	
African	28 (16.4)	0 (0)	
Asiatic	7 (4.1)	0 (0)	
Other	4 (2.33)	4 (8.5)	
Time since HIV diagnosis (years), median (IQR)	15.1 (2.4−34.2)	18.2 (5.4−37.1)	0.011
HIV mode of acquisition, *n* (%)			0.037
Heterosexual	73 (42.7)	19 (40.4)	
MSM	76 (44.4)	14 (29.8)	
Other	22 (12.9)	13 (27.7)	
CD4 nadir, median (IQR)	287 (25−773)	240 (31−501)	0.223
HIV RNA zenith (copies/ml), median (IQR)	63,150 (2800−1,699,328)	30,200 (5100−500,000)	0.914
AIDS, *n* (%)	46 (29.3)	11 (26.8)	0.460
Time since ART initiation (years), median (IQR)	12.0 (1.8−26.4)	17.5 (3.5−29.6)	0.009
Duration VS pre‐switch (months), median (IQR)	104 (1−228)	105 (21−180)	0.549
Previous regimen, *n* (%)			
3DR	151 (88.3)	24 (51.0)	0.000
TXF	139 (81.3)	22 (46.8)	0.000
DOR	3 (1.8)	5 (10.6)	0.013
Other NNRTI	101 (59.1)	18 (38.3)	0.007
PI	64 (37.4)	13 (27.6)	0.131
INSTI	63 (36.8)	19 (40.4)	0.409
Reasons for switch, *n* (%)			
Pill/drug reduction	53 (31.0)	21 (44.7)	0.092
Metabolic	67 (39.2)	25 (53.2)	0.102
Toxicity/intolerance	34 (19.9)	20 (42.5)	0.003
Bone	0 (0)	12 (25.5)	0.000
Psychiatric	14 (8.2)	13 (27.7)	0.001
Resistance/intensification	9 (5.3)	2 (4.2)	0.533
HBsAg pos, *n* (%)	8 (4.7)	−	−
CD4 cell count, median (IQR)	698 (348−1317)	778 (415−1231)	0.340
CD4/CD8 ratio, median (IQR)	0.94 (0.23−1.76)	0.96 (0.39−2.05)	0.966
HIV RNA <50 copies/ml	153 (94.4)	47 (100)	0.229
Full GSS^a^, *n* (%)	110 (91.6)	32 (82.1)	0.086
Previous acquired mutations to DOR, *n* (%)	2 (1.7)	3 (7.7)	0.090
Previous acquired mutations to 3TC, *n* (%)	8 (6.7)	7 (17.9)	0.006
Hypertension, *n* (%)	43 (25.1)	23 (48.9)	0.002
Diabetes, *n* (%)	15 (8.8)	9 (19.1)	0.045
Dyslipidaemia, *n* (%)	105 (61.4)	29 (61.7)	0.555
Cardiovascular disease, *n* (%)	14 (8.2)	8 (17.0)	0.071
Lipodistrophy, *n* (%)	25 (25.3)	16 (34.0)	0.182
Chronic kidney disease, *n* (%)	2 (1.7)	3 (6.4)	0.066
Psychiatric disease, *n* (%)	36 (21.1)	15 (31.9)	0.092
Osteopenia/osteoporosis, *n* (%)	12 (7.0)	21 (44.7)	0.000
Statin, *n* (%)	59 (34.5)	17 (36.2)	0.491
ASCVD risk score, median (IQR)	7.0 (1.0−25.3)	7.1 (1.1−47.7)	0.303
BMI (kg/m^2^), median (IQR)	25.7 (20.7−33.0)	25.3 (17.8−31.4)	0.482

Abbreviations: 3TC, lamivudine; 3DR, three‐drug regimen; ART, antiretroviral therapy; ASCVD, atherosclerotic cardiovascular disease; BMI, body mass index; DOR, doravirine; GSS, genotypic susceptibility score; INSTI, integrase strand transfer inhibitor; IQR, interquartile range; MSM, men who have sex with men; NNRTI, non‐nucleoside reverse transcriptase inhibitor; PI, protease inhibitor; TXF, tenofovir alafenamide or tenofovir disoproxil fumarate.

^a^Genotypic‐resistance test available for 120 individuals on DOR/3TC/TDF and 39 individuals on DOR/3TC.

**Conclusions**: DOR/3TC/TDF and DOR+3TC were comparable in terms of effectiveness and durability. DOR+3TC might be an option in case of complex interplay of metabolic disorders and history of intolerance, exclusively if full known susceptibility to the regimen and reinforced adherence.

**P132: Figure 1:**
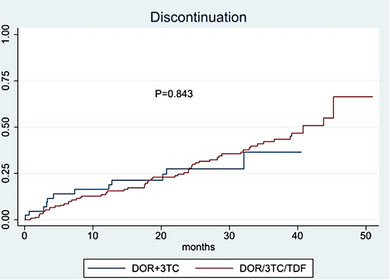
Discontinuation according to three‐drug (3DR) versus two‐drug (2DR) doravirine (DOR)‐based regimens.

### P133 No impact of the M184I/V mutation on the efficacy of tenofovir or abacavir+lamivudine+doravirine in HIV treatment‐experienced people

Cathia Soulie^1^, Aliou Baldé^2^, Fofana Djeneba^3^, Charlotte Charpentier^4^, Pascale Bonnafous^1^, Justine Sourice^5^, Anne De Monte^6^, Veronique Avettand‐Fenoel^7^, Helene Le Guillou‐Guillemette^8^, Laurence Bocket^9^, Stephanie Raymond^10^, Stephanie Marque Juillet^11^, Mary‐Anne Trabaud^12^, Brigitte Montes^13^, Anne Maillard^14^, Cedric Hartard^15^, Elodie Alessandri‐Gradt^16^, Etienne Brochot^17^, Anne Signori‐Schmuck^18^, Lambert Assoumou^2^, Anne‐Genevieve Marcelin ^1^

^1^Virology, AP‐HP Pitié Salpêtrière, Sorbonne Université, INSERM, Paris, France. ^2^Institut Pierre Louis d'Epidémiologie et de Santé Publique, Sorbonne Université, INSERM, Paris, France. ^3^Virology, AP‐HP Saint Antoine, Paris, France. ^4^Virology, AP‐HP Bichat, Paris, France. ^5^Virology, CHU Nantes, Nantes, France. ^6^Virology, CHU Nice, Nice, France. ^7^Virology, Hôpital Cochin, APHP GHU Centre—Université Paris Cité, Paris, France. ^8^Virology, CHU Angers and HIFIH Laboratory EA 3859, LUNAM, Angers, France. ^9^Virology, CHU Lille, Lille, France. ^10^Virology, CHU Toulouse Purpan, Toulouse, France. ^11^Service Biologie, Unité de Microbiologie, CH de Versailles, Versailles, France. ^12^Virology, Institut des Agents Infectieux, Hospices Civils de Lyon, Centre de Biologie Nord, Hôpital de la Croix Rousse, Lyon, France. ^13^Virology, CHU Montpellier, University of Montpellier, Montpellier, France. ^14^Virology, CHU Rennes, Rennes, France. ^15^Virology, CHRU Nancy, Nancy, France. ^16^Virology, CHU de Rouen, Université de Rouen Normandie UNIRouen, Rouen, France. ^17^Virology, CHU Amiens, Faculté de Pharmacie Amiens, Amiens, France. ^18^Virology, CHU Grenoble‐Alpes, Grenoble, France

**Background**: The impact of the M184I/V mutation on the rate of virological failure (VF) in people living with HIV (PLWHIV) with HIV RNA plasma viral load (VL) <50 copies/ml who switch to a tri‐therapy regimen has not been evaluated.

**Materials and methods**: A retrospective national study of antiretroviral‐experienced PLWHIV receiving an abacavir or tenofovir+lamivudine+doravirine regimen in a context of switch (VL <50 copies/ml) was conducted. VF was defined as two consecutive VL ≥50 copies/ml or one VL ≥200 copies/ml and virological blip (VB) as one isolated VL ≥50 copies/ml at month 6 (M6). Genotypic‐resistance tests were interpreted using the Stanford (v9.4.1) and ANRS (v33) algorithms. The M184I/V mutation was examined as a potential factor associated with VF or VB. The adjustment variables were sex, viral subtype, nadir CD4 count, CD4 count at baseline, log zenith plasma HIV‐1 RNA, presence of NNRTI‐resistance mutations at baseline and genotypic susceptibility scores (GSS).

**Results**: Among the 338 PLWHIV analysed, doravirine was mainly associated with tenofovir+lamivudine (311/338, 92.0%) (Table 1). Globally, 45 had a genotypically documented M184I/V mutation before switching (29 M184V and 14 M184I on tenofovir+lamivudine; two M184V on abacavir+lamivudine). PLWHIV with documented M184I/V had lower nadir CD4 (*p* < 0.0001), higher zenith VL (*p* = 0.0285), lower GSS (*p* < 0.0001) and more NNRTI mutations (*p* < 0.0001) at baseline compared to those without M184I/V. Virological failure at M6 was 14.0% and 17.8% in the absence and presence of M184I/V, respectively. The presence of M184I/V was not associated with VF (*p* = 0.2121, aOR 2.07, 95% CI 0.66–6.43). The only factor associated with VF at M6 in this study was a higher log zenith plasma HIV VL (*p* = 0.0029, aOR 1.65, 95% CI 1.19–2.30). The proportion of VB at M6 was 2.4% and 6.7% in PLWHIV in the absence and presence of M184I/V, respectively. The incidence of the VB at M6 was not found to be influenced by the presence or absence of the M184I/V mutation (*p* = 0.3965, aOR 1.97, 95% CI 0.41–9.47) or by the other factors studied.

**P133: Table 1:** Baseline characteristics of the participants on tri‐therapy according to the presence or not of M184V/I at baseline among switch participants (10 imputed datasets)

	M184V/I at baseline	M184V/I at baseline	
Characteristic	No (*n* = 293) *n*/*N* (%) or median (IQR)	Yes (*n* = 45) *n*/*N* (%) or median (IQR)	*p*‐value
Gender			0.3138
Male	191/293 (65.2)	33/45 (73.3)	
Female	102/293 (34.8)	12/45 (26.7)	
Viral subtype			0.5998
B	158/293 (53.9)	28/45 (62.2)	
CRF02	59/293 (20.2)	8/45 (17.8)	
Other non‐B	76/293 (25.9)	9/45 (20.0)	
Nadir CD4 count (cells/mm^3^)	257 (130−409)	157 (42−318)	0.0246
CD4 count at baseline (cells/mm^3^)	620 (467−846)	616 (421−910)	0.9044
Doravirine co‐treatment			0.5539
3TC+TDF	268/293 (91.5)	43/45 (95.6)	
3TC+ABC	25/293 (8.5)	2/45 (4.4)	
GSS with doravirine (Stanford)	3.0 (3.0−3.0)	1.5 (1.0−2.0)	<.0001
GSS with doravirine (ANRS)	3.0 (3.0−3.0)	2.0 (1.5−2.0)	<.0001
Number of NNRTI mutations at baseline	0 (0−1)	1 (0−2)	<.0001
Log zenith plasma HIV‐1 RNA (log10 copies/ml)	4.9 (3.9−5.5)	4.9 (4.3−5.7)	0.5038

*Note: p*‐values were obtained from Fisher's Exact test for categorical variables and Wilcoxon test for numeric variables. Number of missing values: nadir CD4 count (*n* = 5), CD4 count (*n* = 10), zenith plasma HIV‐1 RNA (*n* = 34), plasma HIV‐1 RNA (*n* = 2), GSS with doravirine (*n* = 20), number of NNRTI mutations at baseline (*n* = 61).

**Conclusions**: In antiretroviral‐experienced PLWHIV switching to abacavir or tenofovir+lamivudine+doravirine in clinical practice, we found no evidence of an impact of the previously acquired M184I/V mutation on treatment response.

### P134 Salvage ART regimens based on a combination of an integrase inhibitor and doravirine in heavily antiretroviral‐treated people living with HIV

Irina Ianache ^1^, Cristiana Costescu^2^, Miruna Dragne^2^, Gratiela Tardei^3^, Roxana Radoi^2^, Cristiana Oprea^1^

^1^Infectious Diseases, HIV, Carol Davila University of Medicine and Pharmacy, Bucharest, Romania. ^2^Infectious Diseases, HIV, Victor Babes Hospital for Infectious and Tropical Diseases, Bucharest, Romania. ^3^Laboratory of Virology and Immunology, Victor Babes Hospital for Infectious and Tropical Diseases, Bucharest, Romania

**Background**: Due to particular epidemiological aspects and poor adherence to antiretroviral treatment (ART) in Romanian people living with HIV (PLWH), salvage ART regimens were more frequently used. The aim of this study was to assess epidemiological aspects and outcomes in PLWH under salvage ART regimens based on a combination of a non‐nucleoside reverse transcriptase inhibitor (DOR) and an integrase inhibitor—dolutegravir (DTG) or bictegravir (BIC).

**Methods**: Prospective study on PLWH in active care between January and December 2023 at a tertiary care centre, Bucharest, with ART containing a combination of DOR with either DTG or BIC. Statistical comparisons were performed using Mann‐Whitney test for quantitative variables and chi‐square test for qualitative ones.

**Results**: Out of 69 PLWH on salvage ART regimens, 40 (57.9%) males, with a median age 36 years (IQR 35–49), 50.0% were on BIC/FTC/TAF + DOR, 35.2% 3TC/TDF/DOR + DTG and 14.7% 3TC/DTG + DOR. ART was changed to a salvage regimen after a median time on ART of 21.5 years (IQR 15.75–24). Modes of HIV acquisition were parenteral mode during childhood (PM) 63.7%, heterosexual contact (HSX) 26.0%, injecting drug use 2.5%, men having sex with men 1.4% and by vertical transmission 4.3%. The median nadir CD4/µl and HIV viral load (log_10_ copies/ml) at HIV diagnosis were 37 (IQR 15–124) and 5.24 (IQR 4.71–5.69), respectively. The most common reasons for change were simplification of heavy ART regimen (32), and failure (37). Forty‐nine PLWH had comorbidities, dyslipidaemia (28), neuro‐psychiatric (13) and cardio‐vascular (10) being most frequent. Compared to HSX, PM were younger (*p* = 0.011), spent more time on ART before switch (*p* < 0.0001) and had a higher number of prior ART regimens (*p* < 0.0001) (Table 1). More than half of PLWH 55.0% (38/69) had detectable HIV viral load at change. Viral suppression was achieved in 68.3% (41/60) after 6 months and in 75.0% (36/48) after 12 months on salvage ART (Figure 1).

**P134: Table 1:** Comparison of immuno‐virological characteristics and ART history based on modes of HIV acquisition

Characteristics		PM *N* = 44	HSX *N* = 18	*p*‐value
Gender (male)	*n* (%)	24 (54.5)	9 (50.0)	0.481
Age (years)	Median (IQR)	35.5 (35−36)	49 (36.75−59.5)	0.011
Time on ART before switch	Median (IQR)	23.5 (21−25)	11 (6.75−16.5)	<0.0001
Nadir CD4 cell count/µl	Median (IQR)	80 (12.5−113)	49.5 (18.5−135.75)	0.569
HIV‐RNA at diagnosis	Median (IQR)	5.23 (4.90−5.69)	5.27 (4.70−5.56)	0.964
CD4 cell count/µl at switch	Median (IQR)	309.5 (175−684.25)	331.5 (106.75−572.5)	0.424
HIV‐RNA <50 copies/ml at switch	*n* (%)	21 (47.7)	9 (50.0)	0.546
N of previous ART regimens	*n* (%)	7 (5.75−9)	5 (4−6)	<0.0001
Time spent on INSTI (years)	Median (IQR)	7 (4−12)	4.25 (2.25−6)	0.001
Time spent on PIs (years)	Median (IQR)	17 (10−21.5)	6 (2−10)	0.024
Time spent on NNRTIs (years)	Median (IQR)	6 (3−10)	3 (1−8.75)	0.024

**Conclusions**: The number of PLWH changed to salvage ART regimens significantly increased during last year, especially among PM with heavy ART history. Efficacy of salvage ART regimens was high in PLWH adherent to ART.

**P134: Figure 1:**
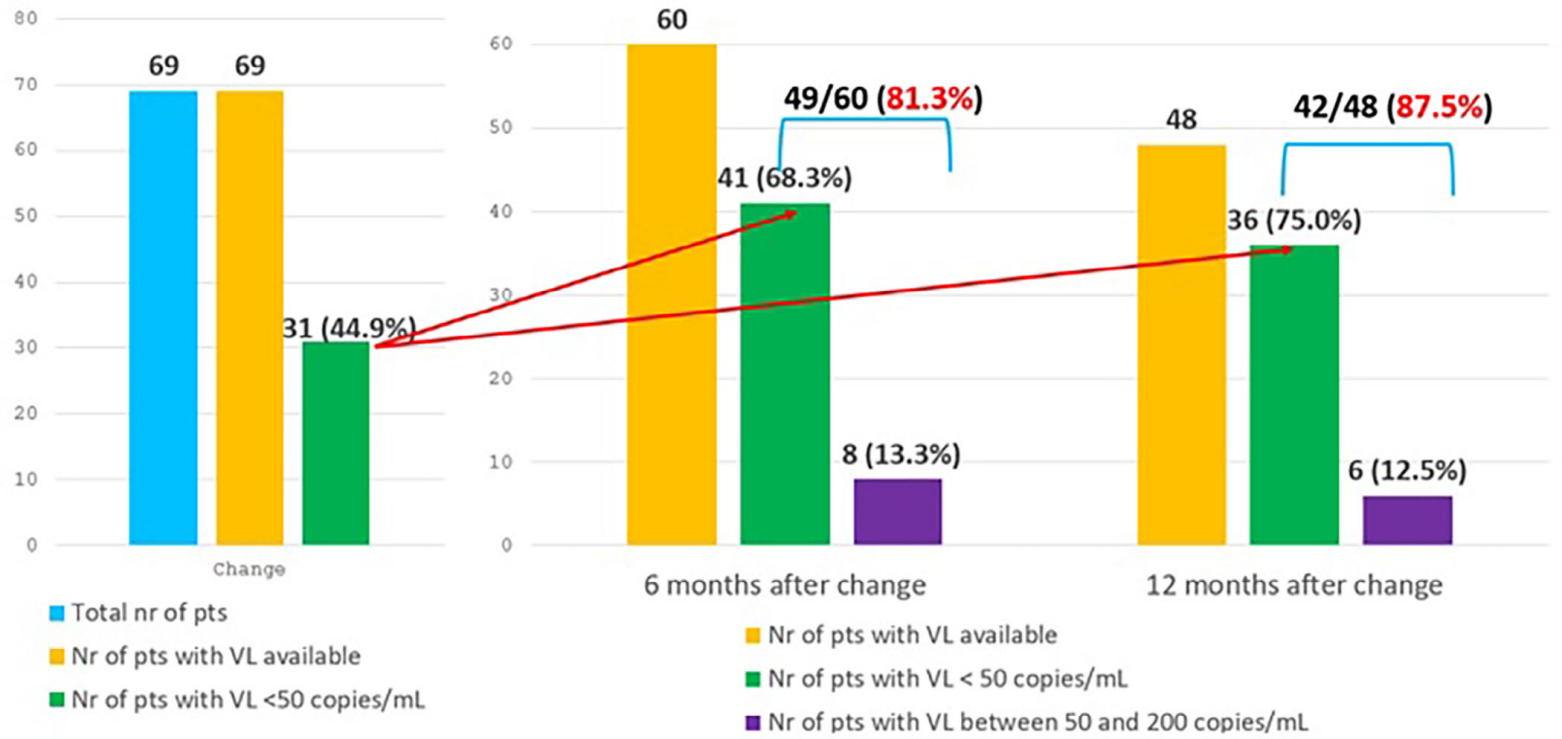
Viral suppression rates after initiation of ART salvage regimen.

### P135 Similar efficacy, safety and CD4 T‐cell increase up to week 96 observed with fostemsavir (FTR)‐based regimens in the BRIGHTE study and dolutegravir (DTG)‐based regimens in the VIKING‐3 study in individuals with multidrug‐resistant (MDR) HIV‐1

Antonella Castagna ^1^, Natalia Gregori^2^, Iacopo Marcon^2^, Fangfang Du^3^, Bo Li^3^, Marcia Wang^3^, Alftan Dyson^4^, Bryn Jones^5^, Manyu Prakash^6^, Andrew Clark^6^

^1^Infectious Diseases, Vita‐Salute San Raffaele University and IRCCS San Raffaele Scientific Institute, Milan, Italy. ^2^Medical Affairs, ViiV Healthcare, Verona, Italy. ^3^Statistics, GlaxoSmithKline, Collegeville, PA, USA. ^4^Medical Affairs, ViiV Healthcare, Durham, NC, USA. ^5^Medical Affairs, ViiV Healthcare, Brentford, UK. ^6^Clinical Development, ViiV Healthcare, Brentford, UK

**Background**: Constructing suppressive regimens in individuals with MDR HIV‐1 can be challenging. We assessed populations with limited antiretroviral (ARV) therapy (ART) options using FTR‐based (BRIGHTE) and DTG‐based regimens (VIKING‐3).

**Materials and methods**: BRIGHTE was a phase III study (*N* = 371; randomized cohort [RC], *n* = 272; non‐randomized cohort, *n* = 99) in adults failing current ART (HIV‐1 RNA >400 c/ml) with ≤2 fully active approved ARVs. Participants with one or two active ARVs entered the RC and received open‐label FTR + optimized background therapy (OBT) after an 8‐day blinded placebo‐controlled period. In the RC, the most common agent in initial OBT was DTG (84%), with 64% taking it twice daily (BID). VIKING‐3 (*n* = 183) was a single‐arm open‐label phase III study in adults with INI‐resistant virus receiving DTG 50 mg BID + failing regimen (without raltegravir or elvitegravir) through day 7, then the regimen was optimized with ≥1 fully active ARV and DTG continued. Virological and immunological response was analysed by baseline demographics and characteristics.

**Results**: BRIGHTE participants were male (*n* = 290, 78%), median age 48 years and White (*n* = 259, 70%). Observed antiviral response in the RC was 81% (<40 c/ml, *n* = 128) and 88% (<400 c/ml, *n* = 140). Mean CD4 increase from baseline was 204.7 cells/mm^3^, with baseline mean CD4 count of 152.5 cells/mm^3^. VIKING‐3 participants were male (*n* = 141, 77%), median age 48 years and 71% (*n* = 130) White. Antiviral response observed in VIKING‐3 was 84% (<50 c/ml, *n* = 101) and 93% (<400 c/ml, *n* = 111). Mean CD4 increase was 192 cells/mm^3^, with baseline mean CD4 count of 202 cells/mm^3^. Safety across study populations was as follows: BRIGHTE (RC), *n* = 92 (34%) serious adverse events (AEs), *n* = 57 (21%) drug‐related AEs and *n* = 7 (3%) AEs leading to withdrawal; VIKING‐3, *n* = 60 (33%) serious AEs, *n* = 52 (28%) drug‐related AEs and *n* = 8 (4%) AEs leading to withdrawal.

**Conclusions**: Despite limited ARV options in individuals with MDR HIV‐1, data demonstrate that both DTG (BID) and FTR‐based regimens provide robust viral suppression and CD4 T‐cell improvement over 96 weeks. FTR and DTG were commonly used together in BRIGHTE, engaging different mechanisms of action. These data provide further support for effective ARVs for individuals living with MDR HIV‐1.

### P136 What is the current place for protease inhibitors in people living with HIV? A retrospective single‐centre study

Ava Diarra ^1^, Agnes Meybeck^1^, Vincent Derdour^1^, Maxime Degrendel^1^, Macha Tetart^1^, Emmanuelle Aissi^1^, Nathalie Viget^1^, Laurence Bocket^2^, Enagnon Kazali^2^

^1^Infectious Disease, CH Dron, Tourcoing, France. ^2^Virology Laboratory, CHU Lille, Lille, France

**Background**: Protease inhibitors (PIs) have been at the origin of highly active antiretroviral therapy. Their metabolic side effects and the development of new drugs relegated them to second‐line treatments. Our study aims to describe the characteristics of people living with HIV (PLWHIV) treated with PI and identify factors associated with their maintenance.

**Materials and methods**: We conducted a retrospective monocentric study in a regional reference centre. All PLWHIV who received PIs for at least 1 year between January 2013 and December 2023 were included. Patients who did not benefit from a follow‐up from 2022 (except for death) were excluded.

**Results**: Among 3607 PLWHIV, 641 (18%) received PIs for at least 1 year and were still under regular follow‐up. The majority were men (63%) with a mean age of 44 years. The main road of contamination was heterosexual in 50% of cases. Almost half of the patients (48%) were born abroad, 31% in sub‐Saharan Africa. Thirty‐two patients (5%) had HBV co‐infection. Main comorbidities were hypertension (11%), diabetes (7%) and dyslipidaemia (6.4%). A median of 5 regimens (IQR 1.3–16.8) were prescribed before PIs initiation. PIs were mostly prescribed in combination with reverse transcriptase nucleoside inhibitors in 77% of cases, for a median duration of 3.2 years (IQR 2–5). At the end of the follow‐up period (median 8 years, IQR 6–9.2), 172 PLWHIV (27%) were still receiving PI. Twenty‐six patients had died. The mortality rate was higher among PLWHIV who were still on PI (8.7% vs. 2.3%, *p* < 0.001). The reason for stopping PIs was most often a simplification (70%). Genotypic‐resistance mutations before the introduction of PI were significantly higher in PLWHIV with PI (83% vs. 74%, *p* = 0.01). The presence of at least one viral load >200 copies/ml during follow‐up was more common in PLWHIV who continued PI (17% vs. 2.8%, *p* < 0.001).

**Conclusions**: Our study reveals that the long‐term retention of PI currently affects a small proportion of PLWHIV. Factors associated with the maintenance of PIs were the presence of mutations at the time of PI initiation and a higher viral load during follow‐up.

### P137 Real‐life use of bictegravir/TAF/emtricitabine in a cohort of people with HIV with a high burden of comorbidities and a history of advanced HIV disease

Andrea De Vito ^1^, Giulia Moi^1^, Giuseppe Conti^2^, Benedetto Maurizio Celesia^2^, Serena Spampinato^2^, Andrea Marino^2^, Claudia Calì^3^, Maria Antonietta Di Rosolini^3^, Laura Corda^1^, Vincenzo Raimondo^2^, Giovanna Sanna^4^, Goffredo Angioni^4^, Giuseppe Nunnari^2^, Giordano Madeddu^1^

^1^Unit of Infectious Diseases, Department of Medicine, Surgery, and Pharmacy, University of Sassari, Sassari, Italy. ^2^Department of Clinical and Experimental Medicine, University of Catania, Catania, Italy. ^3^Infectious and Tropical Diseases Unit, Modica Hospital, Ragusa, Italy. ^4^Infectious Diseases Unit, SS Trinità Hospital, Cagliari, Italy

**Background**: Few long‐term follow‐up studies on bictegravir/TAF/emtricitabine (B/F/TAF) are available in a real‐life setting. This study investigates the real‐life effectiveness and safety of B/F/TAF as a switch strategy in experience people with HIV (PWH).

**Methods**: We conducted a multicentre retrospective analysis through the SHiNe‐SHiC cohort, with data from four centres in Sardinia and Sicily, Italy. We included PWH who started B/F/TAF with an HIV‐RNA <50 copies/ml. Demographical, clinical, viro‐immunological and biochemical data were collected at baseline, 12 and 24 months. We evaluated the incidence of treatment discontinuation (TD) and virological failure (VF).

**Results**: We included 215 PWH, with a median age of 50.7 (IQR 39.3–57.5) years. The duration of HIV infection at switch was 12.5 (IQR 8.0–24.0) years. Nearly 50% had a nadir of CD4 count <200 cells/ml, and 67% had <350 cells/ml. Seventy‐five had AIDS‐defining conditions. We observed a high comorbidity burden: 121 (56.3%) dyslipidaemia, 58 (27%) hypertension and 32 (14.9%) psychiatric disorders. More than 50% were receiving at least one non‐ART treatment, and 11 (5.1%) were treated with five or more. Regarding previous ART, most (198, 92.1%) were treated with triple‐drugs regimens: 28 (13%) NNRTI‐based, 19 (8.8) PI/b and 151 (70.2%) INSTI. Only six (2.8%) received a dual regimen, one 3TC/DTG and five 3TC+PI/b. During 4680.6 person‐months of follow‐up (PMFU), we observed 14 TD, with a rate of 2.9 TD per 1000 PMFU. In seven cases, TD was due to adverse events (three CNS, two gastrointestinal, one asthenia and one neutropenia). Four people chose to discontinue, and three simplified to long‐acting. Additionally, we observed an incidence of HIV‐RNA >50 copies/ml of 4.06 per 1000 PMFU without discontinuation. We found a significant improvement in CD4 cells and CD4/CD8 ratio. A significant increase in creatinine and a decrease in total cholesterol and LDL were also observed (Figure 1).

**Conclusions**: Our study demonstrates sustained virological suppression and immunological improvement over 24 months on B/F/TAF, with low rates of treatment discontinuation primarily due to adverse events. Additionally, significant reductions in total cholesterol and LDL indicate potential cardiovascular benefits. These findings support B/F/TAF as an effective and safe switch strategy for PWH in clinical practice, including those with advanced HIV disease and a high comorbidity burden, including psychiatric disorders.

**P137: Figure 1:**
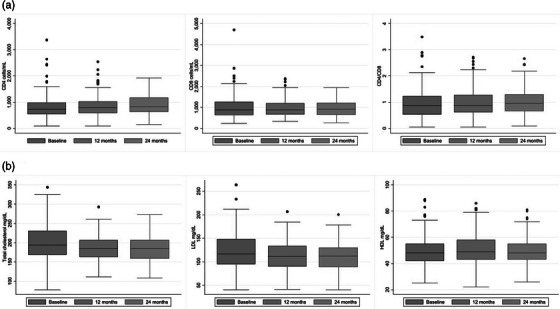
(A) CD4, CD8 and CD4/CD8 in people with HIV starting B/F/TAF at baseline, 48 and 96 weeks. (B) Total cholesterol, LDL and HDL in people with HIV starting B/F/TAF at baseline, 48 and 96 weeks.

### P138 Bictegravir's effect on persistent low‐level viraemia and immunological response. BELAIR study: a pilot prospective observational study

Vasileios Petrakis ^1^, Periklis Panagopoulos^1^, Petros Rafailidis^1^, Grigorios Trypsianis^2^, Stavroula Zisaki^3^, Dimitrios Papazoglou^4^

^1^Department of Infectious Diseases, 2nd University Department of Internal Medicine, University General Hospital Alexandroupolis, Alexandroupolis, Greece. ^2^Laboratory of Medical Statistics, Medical School, Democritus University of Thrace, Alexandroupolis, Greece. ^3^Blood Transfusion Center, University General Hospital of Alexandroupolis, Alexandroupolis, Greece. ^4^Department of Infectious Diseases, 2nd University Department of Internal Medicine, Democritus University of Thrace, University General Hospital of Alexandroupolis, Alexandroupolis, Greece

**Background**: Real‐world experience with low‐level viraemia (LLV) remains not well‐reported and clinical studies evaluating the clinical management and impact of persistent LLV between 50 and 200 copies HIV‐RNA/ml are limited. Co‐formulated bictegravir, emtricitabine and tenofovir alafenamide (BIC/FTC/TAF) is recommended as first‐line treatment with high genetic barrier for people with HIV (PWH). The aim of the present study is to assess the effect of bictegravir on experienced PWH with persistent LLV.

**Patients and methods**: This is an observational prospective study conducted in the Department of Infectious Diseases during the period June 2022–June 2024. PWH at least 5 years on HAART were included in the study after switch to BIC/TAF/FTC. Demographic (age, gender) and clinical data (transmission mode, CDC stage, years with HIV diagnosis, years on HAART, treatment before switch, CD4 cell count and HIV‐RNA at diagnosis) were reported from the patients’ medical records. CD4 cell count, CD4/CD8 ratio and HIV‐RNA were evaluated 2, 6, 12, 18 and 24 months after the BIC/TAF/FTC initiation. Statistical analysis of the data was performed using IBM Statistical Package for Social Sciences (SPSS), version 19.0.

**Results**: A total number of 25 PWH, 16 males with median age 47.08±7.48 years, were included in the study. Current HAART before switch was based on integrase strand transfer inhibitors (INSTIs) in 20 PWH (80%) for a median period of 4.62±0.87 years. CD4 values after BIC (619.45±181.71, *p* < 0.001) were significantly elevated compared to CD4 value before BIC initiation (485.85±166.92). Repeated measures ANOVA revealed significant changes of HIV‐RNA viral load over time (*p* < 0.001). Two months after BIC initiation, 56% (*n* = 14) were virally suppressed and at 12 months 84% (*n* = 21). No blips or virological failure was documented. A significant gradual elevation of CD4/CD8 ratio was also observed (0.51±0.30 before switch vs. 0.69±0.35 at 12 months, *p* < 0.001).

**Conclusions**: The results of the present study indicate that BIC/TAF/FTC could improve the immunological response of PWH and limit the dimensions of persistent LLV reducing the risk of VF and drug‐resistance issues. Further studies in larger patient series are needed in order to determine this beneficial role of BIC.

### P139 HIV treatment with maraviroc: forgotten, not needed or still useful? Results from the MIRROR study

Andreas Müller ^1^, Markus Bickel^2^, Eva Herrmann^3^, Pavel Khaykin^4^, Axel Müller^5^, Gundolf Schuettfort^1^, Christoph Stephan^1^, Annette Haberl^1^

^1^Infectiology, Universitätsklinikum Frankfurt, Frankfurt am Main, Germany. ^2^Infectiology, Infektiologikum Frankfurt, Frankfurt am Main, Germany. ^3^Institute of Biostatistics and Mathematical Modelling, Goethe University Frankfurt, Frankfurt am Main, Germany. ^4^Infectiology, MainFachArzt, Frankfurt am Main, Germany. ^5^Infectiology, Praxis im Nordend, Frankfurt am Main, Germany

**Background**: The CCR5 antagonist maraviroc was approved in 2007 for pre‐treated persons with HIV. Since then, the drug has never been clinically established to a large extend, mainly due to the required tropism test before starting treatment. However, maraviroc is still available and could still be an option for pre‐treated people with HIV in terms of their resistance profile, tolerability issues or comorbidities. We investigated to what extent, in which persons and how successfully maraviroc has been used to date.

**Methods**: MIRROR is a retrospective, multicentre study. Primary objective: Virological response at week 48 in persons who switched to maraviroc between October 2007 and December 2021. Secondary objectives: Reasons for treatment modification, ART prior to switch, maraviroc treatment interruptions, CD4 at baseline and week 48. Chi^2^‐test and Wilcoxon‐Mann‐Whitney U‐test were used to analyse variables influencing the virological outcome.

**Results**: During the observational period, 339 persons were treated with maraviroc: 236 male, 103 female. Mean age at baseline 47.0 years; mean time on ART 11.3 years; history of AIDS 29.2%; mean CD4 515/µl. Viral load (VL) <50 copies 151/339 (44.5%) at baseline and 225/339 (66.4%) at week 48 (ITT analysis). As‐treated analysis: VL <50 130/274 (47.5%) at baseline and 225/274 (82.1%) at week 48. Main reasons for the switch to maraviroc: Virological failure (34.1%) and adverse events (22.4%). ART prior to switch was predominantly protease inhibitor based (50.6%). Fifty‐two (15.3%) persons stopped maraviroc before week 48. Main reasons were virological failure (15.4%) and adverse events (13.5%). Variables influencing the virological response: VL at baseline (*p* = 0.001), CD4 at baseline (*p* = 0.012), history of virological failure (*p* = 0.03) and number of drugs combined with maraviroc (*p* = 0.04).

**Conclusions**: In our study, we analysed data of 339 persons on maraviroc treatment. Main reasons for switching to maraviroc were virological failure and tolerability issues. In the as‐treated analysis, the virological response improved from 47.5% <50 copies at baseline to 82.1% at week 48. Our results demonstrate that the CCR5 antagonist maraviroc can still be considered as a switch option for pre‐treated persons with HIV.

### P140 The virological efficacy of B/F/TAF in people living with HIV who experienced treatment failure: a real‐world cohort

Min Yin, Ziqing Yuan

HIV Department, No. 2 People's Hospital of Fuyang City, Fuyang, China

**Background: **Bictegravir/emtricitabine/tenofovir alafenamide (B/F/TAF) is a recommended therapy for people living with HIV (PLWH) who are antiretroviral‐naive or on stable antiretroviral therapy (ART) with viral suppression (VS). A study from Taiwan shows that B/F/TAF is effective in re‐achieving VS among PLWH who experienced virological failure (VF), and pre‐existing nucleoside reverse transcriptase inhibitor (NRTI)‐related resistance‐associated mutations (RAMs) did not have adverse impact on the effectiveness of B/F/TAF [1]. We aimed to assess the virological effectiveness of B/F/TAF for PLWH who experienced VF from NNRTI or protease inhibitor (PI)+ NRTIs.

**Materials and methods**: We retrospectively enrolled PLWH who had VF with plasma HIV RNA more than 200 copies/ml, genotypic resistances were detected in PLWH with viral load more than 1000 copies/ml. The primary end point was the proportion of PLWH who re‐achieving VS within the first 48 weeks after switch, defined as VL <50 copies/ml.

**Results: **Sixty‐eight PLWH were included. Of whom 42 (61.8%) PLWH were males, the average age was 54 years old. Ninety percent of PLWH were under treatment of NNRTI+ NRTIs before switch and 10% PLWH were under treatment of PI+NRTIs. Median duration of treatment was 7.7 years (IQR 5.1–13.1). Median viral load was 5255 (IQR 678–102,175) copies/ml. The re‐achieving VS rate was 100% for B/F/TAF within the first 48 weeks. The CD4 counts continuously increased from baseline 231 (IQR 167–295) to 460 (IQR 395–525) counts/mm^3^ at first year (WALD square 204.987, *p* < 0.001). Forty‐four PLWH with viral load more than 1000 copies/ml performed genotypic‐resistance testing and 43.2% of whom had major NRTI‐related RAMs (Table 1). All of them successfully achieved VS. We also set a post‐hoc interview questionnaire of satisfaction and adherence. The score of satisfaction increased significantly from 6 (IQR 5–7) to 10 (IQR 9–10) as ART switched (*p* < 0.001). There were more complains about the way of taking medicine was not simple enough on previous regimens. The proportion with adherence rate more than 95% increased from 68.2% at baseline to 97.1% after switch (*p* = 0.045).

**Conclusions: **Switching to B/F/TAF could re‐achieve VS in PLWH who experienced VF. Pre‐existing NRTI‐related RAMs did not have adverse impact on the effectiveness of B/F/TAF. In addition, the satisfaction and adherence both improve.

**P140: Table 1:** Detailed information of 44 PLWH who had results of RAMs to NRTIs

No.	NRTI‐related RAMs	ART before switch	VL before switch, copies/ml
1	None	EFV+3TC+TDF	7100
4	K65R, T69del	EFV+3TC+TDF	292,000
5	None	EFV+3TC+TDF	2870
6	None	EFV+3TC+TDF	1040
7	None	EFV+3TC+TDF	1820
8	None	EFV+3TC+TDF	6810
11	None	EFV+3TC+TDF	1080
12	K70KN, L74V, M184V	EFV+3TC+TDF	3560
13	M184V	EFV+3TC+TDF	291,000
15	M41ML, K65R, S68G, K70T, M184V	EFV+3TC+TDF	564,000
17	None	EFV+3TC+TDF	185,000
22	None	LPV/r+3TC+TDF	7550
23	K65R, S68G, Y115YF, M184V	EFV+3TC+TDF	39,200
24	None	LPV/r+3TC+TDF	197,000
25	None	EFV+3TC+TDF	87,700
27	D67DN, K70E	NVP+3TC+AZT	6940
29	M184V	NVP+3TC+AZT	195,000
31	M184V	EFV+3TC+TDF	29,400
34	M41ML	NVP+3TC+AZT	3900
35	None	NVP+3TC+AZT	4000
38	None	NVP+3TC+AZT	3700
42	M41L, D67N, K70R, V75VILM, M184V, T215Y, K219E	NVP+3TC+D4T	1,380,000
44	None	NVP+3TC+AZT	1,400,000
45	None	EFV+3TC+AZT	9470
46	None	NVP+3TC+D4T	1100
47	None	NVP+3TC+AZT	1260
48	M184V	NVP+3TC+AZT	34,000
49	None	NVP+3TC+D4T	1800
50	None	ATV+3TC+D4T	1630
51	M184V	NVP+DDI+D4T	272,000
52	G190Q	NVP+3TC+AZT	231,000
53	M184V	NVP+DDI+D4T	2800
54	K65R, T69del	NVP+3TC+AZT	7900
55	T69D, K70KQ, Y115F, M184V	NVP+3TC+AZT	48,300
56	None	NVP+DDI+D4T	5400
57	T69AD, K70EQ, Y115F, M184V	LPV/r+3TC+TDF	5110
58	None	NVP+3TC+AZT	24,600
59	None	EFV+3TC+TDF	107,000
61	T69AD, K70EQ, Y115F, M184V	EFV+3TC+TDF	995,000
62	None	EFV+3TC+TDF	520,000
63	K70KN, L74V, M184V	LPV/r+3TC+TDF	2200
64	K65R, S68SR, V75VILM, Y115YF, F116FY, M184MV	EFV+3TC+TDF	110,000
67	None	LPV/r+3TC+TDF	294,000
68	None	EFV+3TC+TDF	6900

**Reference**

1. Chen GJ, Sun HY, Chang SY, Hsieh SM, Sheng WH, Chuang YC, et al. Effectiveness of second‐generation integrase strand‐transfer inhibitor‐based regimens for antiretroviral‐experienced people with HIV who had viral rebound. J Microbiol Immunol Infect. 2023;56:988‐95.

### P141 Early treatment failure with lenacapavir in HIV‐2 infection

Martin Obermeier ^1^, Özlem Yildirim^1^, Robert Ehret^1^, Maher Almahfoud^2^, Dirk Berzow^3^, Bernhard Krüdewagen^4^, Olaf Degen^2^

^1^Molecular Diagnostics, Medizinisches Infektiologiezentrum Berlin, Berlin, Germany. ^2^Infectiology, University Medical Center Hamburg‐Eppendorf, Hamburg, Germany. ^3^Infectiology, Private Practice, Hamburg, Germany. ^4^Zentrallabor, Deutsches Herzzentrum der Charité, Berlin, Germany

**Background**: In vitro data suggest the efficacy of lenacapavir (LEN) against HIV‐2, although mean IC50 is 11‐fold higher as compared to HIV‐1. We describe an early treatment failure after LEN initiation in an individual infected with HIV‐2 with so far unobserved mutations.

**Materials and methods**: A 65‐year‐old male was diagnosed with HIV‐2 in 1991 (WHO/CDC C2). ART was started in 2005 and adapted due to the development of resistance. Previous (Feb 2018) genotypic‐resistance analysis showed mutations against NRTIs (K65R, M184V) and PIs (I50V, I54M, I64V) without INI mutations. Last ART was dolutegravir (DTG, BID), 3TC, AZT and lopinavir/r. In June 2023, HIV‐2 VL was 10,280 IU/ml, CD4 cells were 32/µl (2%). Salvage‐ART was initiated with LEN, DTG (BID), 3TC and AZT. HIV‐2 VL was measured with the Altona RealStar^®^ HIV‐2 RT‐PCR Kit 1.0, mutations were identified by amplicon‐based nanopore sequencing of the gag, pol and env region. Plasma drug levels were determined with liquid chromatography with tandem mass spectrometry (LC‐MS/MS). HIV‐2 drug resistance was interpreted with the HIV2EU tool.

**Results**: After 21 days, a rapid decrease in HIV‐2 VL from 10,280 to 1400 IU/ml was documented, followed by an increase at day 35 to 1,200,000 IU/ml (CD4^+^ 84 cells/µl). LEN plasma levels were between 20 and 62 ng/ml (reference C(tau) CI 19.8–52.6 ng/ml) and DTG 9810–11,400 ng/ml (reference C(tau) CI 790–4266 ng/ml). Treatment was well tolerated with small lumps at the injection sites. In comparison to the baseline sequence, a single amino acid change N73D (capsid position numbering HIV‐2 BEN) at day 35 was observed. Re‐analysis of the pol region at baseline confirmed mutations in the protease and reverse transcriptase with additional mutations D67N and V111I. Integrase mutations T97A, Y143A and N155S were observed, of which only the N155S was detected.

**Conclusions**: Despite the initial drop in HIV‐2 VL and LEN plasma levels in the expected range, rapid resistance development was observed after 35 days of treatment. The found integrase mutations are most probably associated with resistance and were leading to subsequent LEN mono therapy. Further studies are needed to investigate the effectiveness and the use of LEN in salvage settings in HIV‐2 patients.

### P142 HIV‐GRADE drug‐resistance interpretation web tool update for capsid and post‐attachment inhibitors

Martin Obermeier ^1^, Robert Ehret^1^, Alexander Thielen^2^, Martin Däumer^3^, Björn Jensen^4^, Carina Elsner^5^, Christopher Dächert^6^, Elena Knops^7^, Eva Geringer^8^, Eva Heger^7^, Eva Wolf^9^, Frank Wiesmann^10^, Hauke Walter^11^, Andrea Hauser^12^, Christian Hoffmann^13^, Irene Görzer^8^, Jens Verheyen^14^, Josef Eberle^15^, Karin Metzner^16^, Klaus Korn^17^, Thomas Lengauer^18^, Karolin Meixenberger^19^, Martin Stürmer^20^, Max Münchhoff^6^, Patrick Braun^10^, Stefan Esser^21^, Thomas Klimkait^22^, Rolf Kaiser^23^, Nadine Lübke^24^

^1^Molecular Diagnostics, Medizinisches Infektiologiezentrum Berlin, Berlin, Germany. ^2^Bioinfomatics, Seq‐IT, Kaiserslautern, Germany. ^3^Molecular Diagnostics, Institut für Immunologie und Genetik, Kaiserslautern, Germany. ^4^Klinik für Gastroenterologie, Hepatologie und Infektiologie, Uniklinikum Düsseldorf, Düsseldorf, Germany. ^5^Institut für Virologie, Universitätsklinikum Essen, Essen, Germany. ^6^Max von Pettenkofer‐Institut, Ludwig‐Maximilians‐Universität München, Munich, Germany. ^7^Virologie, Uniklinikum Köln, Cologne, Germany. ^8^Zentrum f. Virologie, Medizinische Universität Wien, Wien, Austria. ^9^Clinical Research, MUC Research, Munich, Germany. ^10^Labor, PZB Aachen, Aachen, Germany. ^11^Labor, Medizinisches Labor Stendal, Stendal, Germany. ^12^Private Practice, Berlin, Germany. ^13^Private Practice, ICH Hamburg, Hamburg, Germany. ^14^Labor, Institut für Immunologie und Genetik, Kaiserslautern, Germany. ^15^Private Practice, Freising, Germany. ^16^Virologie, Universitäts Spital Zürich, Zürich, Switzerland. ^17^Virologie, Universität Erlangen, Erlangen, Germany. ^18^Bioinformatics, Max Planck Institute, Saarbrücken, Germany. ^19^HIV‐Studienlabor, Robert‐Koch Institut, Berlin, Germany. ^20^Labor, IMD Labor Frankfurt, Frankfurt, Germany. ^21^Zentrum für HIV, AIDS, Proktologie und Geschlechtskrankheiten, Universitätsmedizin Essen, Essen, Germany. ^22^Biomedicine, University of Basel, Basel, Germany. ^23^Institut für Virologie, Uniklinikum Köln, Cologne, Germany. ^24^Institut für Virologie, Universitätsklinikum Düsseldorf, Düsseldorf, Germany

**Background**: With the approval of new drug classes with different modes of action and new drug‐resistance mutations in targets that are currently not covered by the existing analysis tools, an update for these regions was necessary and implemented. Mutations for capsid inhibitors can be found in the gene region of gag coding for the capsid protein, while mutations against post‐attachment inhibitors can be found in the env gene gp120 region.

**Materials and methods**: The existing framework of the HIV‐GRADE (Genotypic Resistance‐Algorithm DEutschland) tool, a web‐based tool to identify and interpret viral drug resistance (https://www.hiv‐grade.de/cms/grade/), was extended to recognize and correctly highlight the amino acid positions of the capsid region and the gp120 region. Subtype‐specific consensus sequences were gathered from Los Alamos National Laboratories (LANL) HIV sequence database. These sequences were then realigned and patterns for recognition of the gene regions extracted (92 each for capsid and gp120). Test sequences were also retrieved from LANL HIV sequence database (459 each). Interpretation rules were generated by the HIV‐GRADE expert team based on published data and translated into ASI‐XML files.

**Results**: All of the 459 test sequences for each gene region could be correctly identified and interpreted. Known drug‐resistance mutations were identified and drug resistance was predicted based on the ruleset defined by the HIV‐GRADE team. This includes subtype‐specific mutations that can lead to pre‐existing drug resistance.

**Conclusions**: With the upgrade of the HIV‐GRADE tool v.07/24, drug‐resistance interpretation of new drug classes can now be substantially simplified. This allows for more accessible drug‐resistance interpretation in individuals with limited treatment options.

### P143 Safety and efficacy of switch to bictegravir/emtricitabine/tenofovir alafenamide fumarate following dual regimen therapy in HIV: insights from the ICONA cohort

Andrea De Vito ^1^, Alessandro Tavelli^2^, Alessandro Cozzi‐Lepri^3^, Andrea Giacomelli^4^, Roberto Rossotti^5^, Giacomo Ponta^6^, Nicoletta Bobbio^7^, Alice Ianniello^8^, Antonella Cingolani^9^, Giordano Madeddu^1^, Andrea Antinori^10^, Antonella d'Arminio Monforte ^2^

^1^Unit of Infectious Diseases, Department of Medicine, Surgery, and Pharmacy, University of Sassari, Sassari, Italy. ^2^ICONA Foundation, Milan, Italy. ^3^Centre for Clinical Research, Epidemiology, Modelling and Evaluation, Institute for Global Health, London, UK. ^4^Infectious Diseases Unit, ASST Fatebenefratelli‐Sacco, DIBIC Luigi Sacco, University of Milan, Milan, Italy. ^5^Department of Infectious Diseases, ASST Grande Ospedale Metropolitano Niguarda, Milan, Italy. ^6^Infectious Diseases Unit, IRCCS San Raffaele Scientific Institute, Vita Salute San Raffaele University, Milan, Italy. ^7^Department of Infectious Diseases, Galliera Hospital, Genoa, Italy. ^8^Division I of Infectious and Tropical Diseases, ASL Città di Torino, Turin, Italy. ^9^Clinic of Infectious Diseases, Department of Safety and Bioethics, Università Cattolica del Sacro Cuore, Rome, Italy. ^10^National Institute for Infectious Diseases, Lazzaro Spallanzani IRCCS, Rome, Italy

**Background**: Most treatment switches are for simplification from three‐drug regimens (3DR) to dual regimens (2DR). However, there is a small proportion of people with HIV (PWH) who may switch to bictegravir/emtricitabine/tenofovir alafenamide fumarate (B/F/TAF) after a period of time spent on 2DR.

**Methods**: We included all PWH enrolled in the ICONA cohort who started B/F/TAF after a period of treatment with 2DR INSTI‐based (3TC/DTG, RPV/DTG, RPV/CAB or DOR+DTG). Virological failure (VF), virological suppression and toxic dose (TD) for toxicity or failure of B/F/TAF were evaluated using Kaplan‐Meier curves. Linear mixed models for small sample (Kenward and Roger method), adjusted for age and sex, with random intercept and slopes with a change in slope before and after switch, were used to evaluate the trajectories of triglycerides, cholesterol, CD4 and CD4/CD8 2 years before and after B/F/TAF switch. Viro‐immunological analyses were stratified according to HIV‐RNA strata at B/F/TAF at switch.

**Results**: Among the 3662 PWH who started a 2DR INSTI‐based regimen, 71 (1.9%) switched to B/F/TAF; 60 had follow‐up data after switch, for a median of 10.9 months (IQR 3.6–24.7). PWH characteristics are summarized in Figure 1, left. Forty PWH switched with undetectable HIV‐RNA (uVL, <50 copies/ml), 20 with detectable HIV‐RNA (dVL, ≥50 copies/ml): 13/20 with HIV‐RNA 50–200 copies/ml. Reasons for switching to B/F/TAF were: 28.3% failure, 18.3% pill reduction, 16.3% patients’ choice, 21.7% toxicities and 15% other/unknown. Among the uVL group, one VF was reported (HIV‐RNA: 99, 71 and then 29 copies/ml without switching). Among the dVL group, the 1‐year cumulative probability of reaching undetectable VL was 80% (95% CI 57.6–95.1). Fourteen PWH interrupted B/F/TAF: seven for simplification (50.0%), four for toxicity (28.6%), two for VF (14.2%) and one for patient's choice (7.1); the 1‐year cumulative probability of TD for toxicity/failure was 10.7% (95% CI 14.5–24.5). We observed an increase in the CD4/CD8 (+0.023 CD4/CD8 per month, *p* = 0.026) only in the dVL group. Changes in immunological and lipids are shown in Figure 1, right.

**Conclusions**: Switching from 2DR‐INSTI to B/F/TAF is infrequent; this switch results in a low rate of toxicity/failure and a good immuno‐virological and lipid profile. CD4/CD8 gain is observed as a result of achieving undetectable HIV‐RNA.

**P143: Figure 1:**
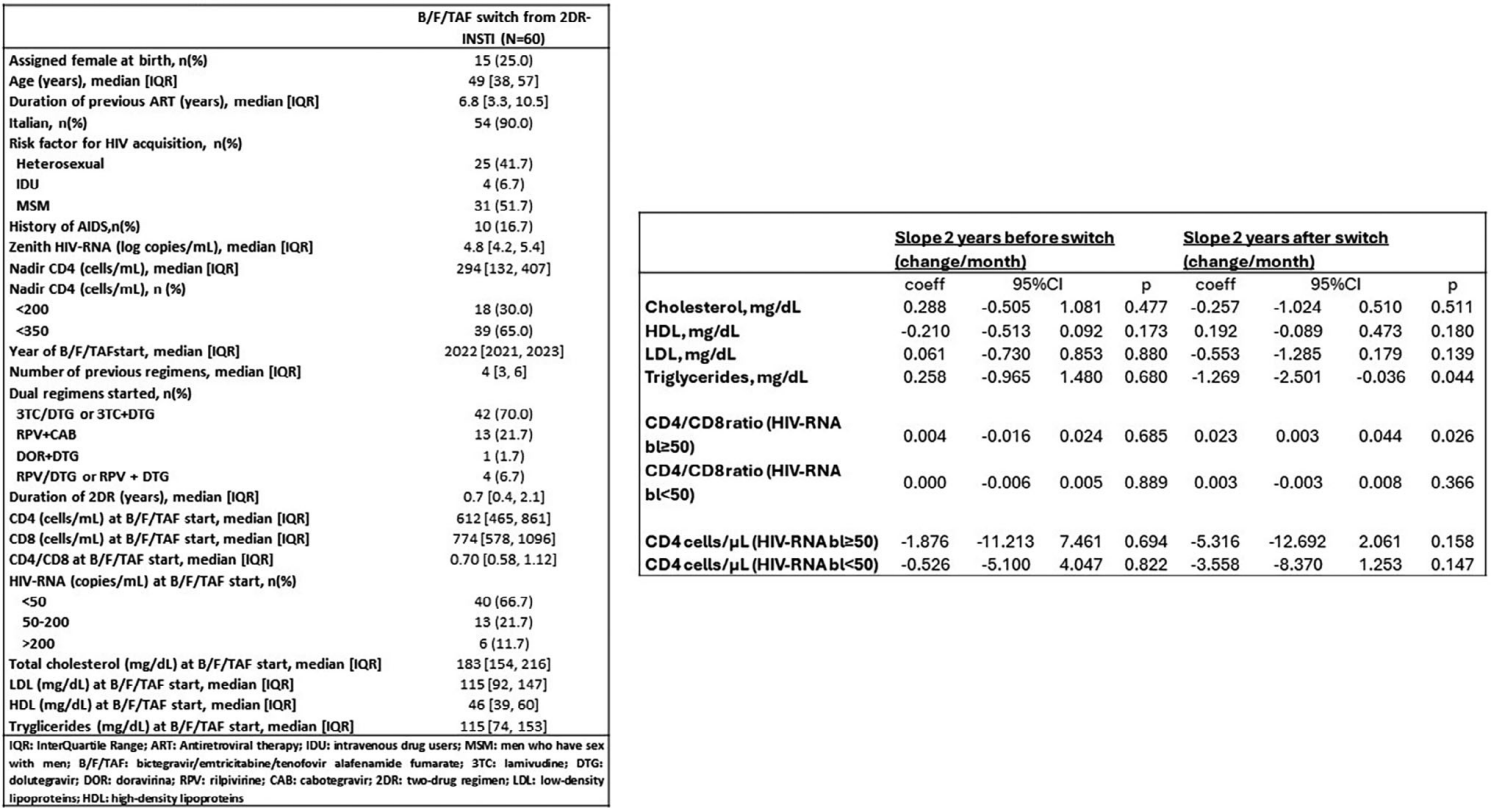
(left) Characteristics of 60 people with HIV treated with 2DR INSTI‐based who started treatment with B/F/TAF; (right) slopes from fitting linear mixed models adjusted for age and sex with change at B/F/TAF switch.

### Treatment strategies—Models of care for ageing/frail populations including virological failure and switching

### P144 Safety and efficacy of doravirine plus tenofovir alafenamide/emtricitabine/bictegravir (TAF/FTC/BIC) in heavily pre‐treated patients

Ana Moreno ^1^, Jose Luis Casado^1^, Maria Jesus Vivancos^1^, Isabel Izuzquiza^1^, Manuel Velez^2^, Fernando Dronda^1^, Javier Martinez^1^, Raquel Ron^1^, Santos Del Campo^1^, Santiago Moreno^1^, Maria Jesus Perez‐Elias^1^

^1^Infectious Diseases, Hospital Ramon y Cajal, Madrid, Spain. ^2^Pharmacy, Hospital Ramon y Cajal, Madrid, Spain

**Background**: Doravirine (DOR) is a versatile antiretroviral drug that may be used in combination beyond standard triple therapy.

**Materials and methods**: Ongoing, retrospective‐prospective analyses to evaluate the safety and efficacy of DOR pus TAF/FTC/BIC started between November 2022 and March 2024 in 23 HIV patients attended at an HIV outpatient clinic from a tertiary hospital in Madrid, Spain, due to viral failure, drug interactions or optimization.

**Results**: White 74% (*N* = 17), male 65% (*N* = 15, 73% MSM), median age 59 years (35–77), prior AIDS 43% (*N* = 10). Median time on antiretroviral therapy was 25 years (1–31), with a median of 11 prior ARV lines (1–40). Baseline CD4 count 632 cells/ml (119–1398). Prior ARV was INSTI based in 12 cases (52%), PI based in one (4%), and both INSTI and PI in 10 (43%), with additional maraviroc in three cases (13%). Prior NNRTI experience was present in 18 (78%), with more than one prior NNRTI in 10 (55%), failure in eight (44%) and mutations in six (33%). DOR plus TAF/FTC/BIC was prescribed due to drug interactions in two cases (9%), optimization in four (17%) and low‐grade viraemia (less than 1000 copies/ml) in 17 cases (74%): of these, 76% (*N* = 13) reached undetectable HIV RNA, with a median time on the combination of 54w (14–76). Among the four subjects with persistent low‐grade viraemia, two changed to long‐acting cabotegravir/rilpivirine with virological success, and two maintained the combination with last available HIV RNA of 1.82 and 1.90 logs, respectively. There were no withdrawals due to toxicity.

**Conclusions**: The use of DOR plus TAF/FTC/BIC in heavily pre‐treated patients was safe—no withdrawals due to toxicity—and led to undetectable HIV RNA in 76% of patients with prior viral failure.

### P145 Safety and efficacy of dual doravirine plus lamivudine as a switch strategy in HIV patients with metabolic or renal issues

Ana Moreno ^1^, Jose Luis Casado^1^, Manuel Velez^2^, Maria Jesus Vivancos^1^, Isabel Izuzquiza^1^, Javier Martinez^1^, Santos Del Campo^1^, Santiago Moreno^1^, Maria Jesus Perez‐Elias^1^

^1^Infectious Diseases, Hospital Ramon y Cajal, Madrid, Spain. ^2^Hospital Ramon y Cajal, Pharmacy, Madrid, Spain

**Background**: Dual doravirine (DOR) plus lamivudine (3TC) (DOR/3TC) regimens can maintain high levels of viral suppression in experienced patients [1]. This relies on the high genetic barrier and versatility of DOR.

**Materials and methods**: Ongoing, retrospective‐prospective analyses to evaluate the safety and efficacy of dual DOR/3TC started between March 2021 and June 2024 as a switch strategy in 36 HIV patients with metabolic (obesity, diabetes/prediabetes, dyslipidaemia) or renal issues attended at an HIV outpatient clinic from a tertiary hospital in Madrid, Spain.

**Results**: White 72% (*N* = 26), male 75%, median age 54 years (28–71), prior AIDS 28% (*N* = 10). Median time on antiretroviral therapy was 16 years (2–32), with a median of 6 prior ARV lines (2–27). Baseline CD4 count 588 cells/ml (90–1292), all with undetectable HIV RNA. Prior ARV was INSTI based in 32 cases (89%, including three cases on long‐acting cabotegravir/rilpivirine). Thirty‐one patients (86%) reported prior NNRTI experience, with the absence of viral failure in all. DOR plus 3TC was prescribed due to metabolic issues in 18 cases (50%), mainly in Hispanic versus White subjects (90% vs. 35%, *p* = 0.03): obesity—BMI >30—in 12 (67%), diabetes (*N* = 5, 28%), prediabetes (*N* = 2, 11%), dyslipidaemia (*N* = 5, 28%). Renal deterioration was the main reason of prescription in 17 cases (47%), with adjusted 3TC dosing in seven (41%). Baseline median glomerular filtration rate (GFR) and tubular phosphate resorption were 43.22 ml/minute (7.57–59.26) and 63.63% (12.58–93.34), respectively, with GFR <30 ml/minute in seven (41%), haemodialysis in two (12%) and peritoneal dialysis in one. Hypertension was present in 14 cases (39%). Overall, after a median time on DOR/3TC of 15w (1–147), there were no cases of viral failure. Only three subjects stopped therapy due to CNS DOR‐related adverse events. Costs per day were reduced from 14.87€ (INSTI‐based oral therapies) to 5.86€.

**Conclusions**: DOR/3TC was safe and maintained viral suppression in heavily pre‐treated patients with metabolic issues or renal deterioration on prior INSTI‐based therapies, with reduced daily costs. These results may lead to larger comparative maintenance trials.

**Reference**

1. Perfezou P, Hall N, Duthe JC, Abdi B, Seang S, Arvieux C, et al. Doravirine plus lamivudine two‐drug regimen as maintenance antiretroviral therapy in people living with HIV: a French observational study. J Antimicrob Chemother. 2023;78(8):1929‐1933.

### P146 Switching to dual therapy in elderly and multi‐experienced patients: profile of a reference service in Brazil

Luisa de Oliveira Pereira, Jorge Casseb, Mariana Monteiro, Ana Paula Rocha Veiga, Najara Ataide de Lima Nascimento, Luisa Caracik de Camargo

Department of Dermatology, HCFMUSP, São Paulo, Brazil

**Background**: With the current availability of drugs with greater potency, tolerability and genetic barrier, interest in antiretroviral‐sparing strategies to reduce toxicity, regimen complexity and costs has resurfaced [1]. Current studies have already shown that this appears to be a safe option [2−4], but little has been studied to date in long‐lived and multi‐experienced populations.

**Materials and methods**: To evaluate the profile of patients who switched to dual regimens (DTG + 3TC or DTG + DRV/r or DRV/r + 3TC) at the ADEE3002 outpatient clinic. Retrospective analysis with data collected from April 2021 to December 2023 from PLWHA followed at the ADEE3002 (Grupo do Ambulatório de Imunodeficiências Secundárias)/HCFMUSP (Hospital das Clínicas da Faculdade de Medicina da Universidade de São Paulo) outpatient clinic, São Paulo. The patients evaluated were switched to the dual regimen having been undetectable for at least 6 months and with no reported resistance. Data were retrieved from medical records.

**Results**: The ADEE3002 outpatient clinic currently has 430 active patients, of which 34 are eligible for our analysis. The main characteristics of this population analysed are: men 29/34 (85.29%), mean age 55.6 years, mean time of HIV infection 18.5 years, mean CD4 nadir 327.44, previous diagnosis of advanced HIV in 10/34 (29.41%) and previous opportunistic infection in 7/34 (20.6%). The average time of exposure to ARV was around 16 years, the average number of previous regimens was 4.12, exposure to integrase inhibitors 20/34 (58.8%), exposure to protease inhibitors 21/34 (61.76%). Only 8/34 (23.5%) of patients did not have any comorbidity. Among the main comorbidities were dyslipidaemia 19/34 (55.9%), renal dysfunction 16/34 (47%), systemic arterial hypertension 14/34 (41.2%), type II diabetes 7/34 (20.6%), psychiatric comorbidities 6/34 (17.6%), lipodystrophy 6/34 (17.6%), osteopenia or osteoporosis 4/34 (11.8%), neurological sequelae 4/34 (11.8%). After 12 months of exchange, 32/34 (94.11%) remained undetectable. No virological failure or need to change the regimen was detected in the patients analysed. CD4 T lymphocyte values remained without significant changes.

**Conclusions**: Even in long‐lived and multi‐experienced populations, dual therapy regimens with DTG + 3TC or DRV/r + 3TC or DTG + DRV/r appear to be safe options in the management of comorbidities and adverse effects in PLWHA undergoing viral suppression without prior resistance.

**References**

1. Baril J‐G, Angel JB, Gill MJ, Gathe J, Cahn P, van Wyk J, et al. Dual therapy treatment strategies for the management of patients infected with HIV: a systematic review of current evidence in ARV‐naive or ARV‐experienced, virologically suppressed patients. PLoS One. 2016;11:e0148231.

2. Cahn P, Madero JS, Arribas JR, Antinori A, Ortiz R, Clarke AE, et al. Durable efficacy of dolutegravir plus lamivudine in antiretroviral treatment‐naive adults with HIV‐1 infection: 96‐week results from the GEMINI‐1 and GEMINI‐2 randomized clinical trials. J Acquir Immune Defic Syndr. 2020;83(3):310‐8. Erratum in: J Acquir Immune Defic Syndr. 2020;84(3):e21.

3. van Wyk J, Ajana F, Bisshop F, De Wit S, Osiyemi O, Portilla Sogorb J, et al. Efficacy and safety of switching to dolutegravir/lamivudine fixed‐dose 2‐drug regimen vs continuing a tenofovir alafenamide‐based 3‐ or 4‐drug regimen for maintenance of virologic suppression in adults living with human immunodeficiency virus type 1: phase 3, randomized, noninferiority TANGO study. Clin Infect Dis. 2020;71(8):1920‐9.

4. Pulido F, Ribera E, Lagarde M, Pérez‐Valero I, Palacios R, Iribarren JA, et al.; DUAL‐GESIDA‐8014‐RIS‐EST45 Study Group. Dual therapy with darunavir and ritonavir plus lamivudine vs triple therapy with darunavir and ritonavir plus tenofovir disoproxil fumarate and emtricitabine or abacavir and lamivudine for maintenance of human immunodeficiency virus type 1 viral suppression: randomized, open‐label, noninferiority DUAL‐GESIDA 8014‐RIS‐EST45 trial. Clin Infect Dis. 2017;65(12):2112‐8.

## Treatment strategies—Rapid ART initiation

### P147 Impact of NGS‐detected viral mutations on HIV viral decay in patients initiating a BIC/TAF/FTC regimen

Marcello Trizzino^1^, Roberta Gaudiano^2^, Luca Pipitò^2^, Celestino Bonura^2^, Claudia Gioè^1^, Benedetta Romanin ^1^, Irene Ganci^2^, Dalila Mimì Arena^2^, Antonio Cascio^2^

^1^Infectious and Tropical Diseases Unit, University Hospital Paolo Giaccone, Palermo, Italy. ^2^Department of Health Promotion, Mother and Child Care, Internal Medicine and Medical Specialties, University Hospital Paolo Giaccone, Palermo, Italy

**Background**: Integrase inhibitors are recommended as a component of initial HIV treatment in most guidelines. Next‐generation sequencing (NGS) has the capability to identify low‐abundance drug‐resistant HIV‐1 variants within the viral quasi‐species at levels lower than 20%. The objective of our study is to evaluate the impact of minor transmitted resistance mutations in viral decay in a group of people newly diagnosed with HIV and starting a regimen with BIC/F/TAF.

**Materials and methods**: In our study, we enrolled 48 patients newly diagnosed with HIV and who started a regimen containing BIC/TAF/FTC. A resistance test was performed on all patients using the NGS method, together with the determination of HIV viraemia and CD4^+^ lymphocyte count and the CD4/CD8 ratio. HIV‐RNA viraemia and CD4^+^ determination were repeated 4 and 12 weeks after antiretroviral start.

**Results**: Table 1 shows the characteristics of the population under analysis. Thirty‐six PLWH showed no resistance to NGS testing, whereas 12 PLWH showed ≥1 mutation on the reverse transcriptase/integrase class. The analysis of the nucleotide sequences was carried out by positioning the resistance threshold at 5%, 10% and 20%. After 4 and 12 weeks, the determination of HIV‐1 viraemia and lymphocyte typing was repeated in both analysed groups. The analysis in Table 2 did not highlight any statistically significant difference regarding viraemia and CD4^+^ values between the two groups during the entire observation period. The wild‐type group took 12.73 weeks to achieve HIV 1‐RNA viraemia below 50 copies/ml, whereas the transmitted resistance group took 19.92 weeks to achieve virosuppression (*p* = 0.037).

**P147: Table 1:** Characteristics of the groups analysed

	Population (*n* = 48)	Wild type (*n* = 36)	Non‐wild type (*n* = 12)
Male	42 (87.5%)	30 (83.3%)	12 (100%)
Female	5 (10.4%)	5 (13.9%)	–
MtF	1 (2.1%)	1 (2.8%)	–
Age (min−max)	46.40 (23−78)	44.17 (23−78)	53.08 (23−73)
Weeks of HIV (min−max)	96.46 (22−168)	96.89 (22−168)	95.15 (55−125)
CDC stage C at diagnosis (%)	20 (41.7%)	16 (44.5%)	4 (33.3%)
HIV‐RNA at diagnosis, copies/ml	1,103,614	914,231	1,655,983
AIDS event	18 (37.5%)	15 (41.7%)	3 (25%)
% of HIV‐RNA >100,000 copies/ml at diagnosis	33 (68.8%)	24 (66.7%)	9 (75.5%)
% of HIV‐RNA >500,000 copies/ml at diagnosis	19 (39.6%)	13 (36.1%)	6 (50%)
CD4^+^ at diagnosis	243.83 (4−654)	250 (9−598)	225 (4−654)
CD4/CD8 at diagnosis	0.3077 (0.00−1.40)	0.3186 (0.00−1.40)	0.2750 (0.00−0.90)
HCV Ig positive	1 (2.1%)	1 (2.8%)	0
CMV Ig positive	38 (79.2%)	28 (77.8%)	10 (83.3%)
HIV‐1 subtypes			
A1	2 (4.2%)	2 (5.6%)	–
B	22 (45.8%)	15 (41.7%)	7 (58.3%)
C	1 (2.1%)	1 (2.8%)	–
CRF	19 (39.6%)	15 (41.7%)	4 (33.3%)
F1	3 (6.3%)	3 (8.3%)	–
G	1 (2.1%)	–	1 (83.3%)

**P147: Table 2:** Results

	Wild type (*n* = 36)	Not wild type (*n* = 12)	*p*‐value
HIV‐RNA at diagnosis, copies/ml	914,231	1,655,983	0.913
HIV‐RNA 4 weeks (±2 ww), copies/ml	382.2	374.6	0.973
HIV‐RNA 12 weeks (±2 ww), copies/ml	42.9	267.7	0.212
CD4^+^ at diagnosis, cells/mmc	250	225	0.520
CD4^+^ 4 weeks (±2 ww), cells/mmc	341	301	0.505
CD4^+^ 12 weeks (±2 ww), cells/mmc	398	345	0.572
CD4/CD8 at diagnosis	0.28	0.32	0.856
CD4/CD8 4 weeks (±2 ww)	0.43	0.43	0.675
CD4/CD8 12 weeks (±2 ww)	0.31	0.52	0.354
Weeks to obtain HIV 1‐RNA <50 cp/ml	12.73	19.92	0.037

**Conclusions**: The availability of high genetic barrier regimens such as BIC/F/TAF allows the clinician to obtain successful virological results even in scenarios in which the resistance test conducted with the NGS method detects minor resistance to classes such as NRTIs or even INIs. The data presented in this real‐life study aims to strengthen the safety of a regimen such as BIC/F/TAF even in conditions in which it is not possible to obtain the results of the resistance test before starting antiretroviral therapy or in the context of rapid ART initiation.

### P148 Synthesizing HIV clinical guidelines on characteristics, challenges and facilitators of rapid initiation of antiretroviral therapy: a rapid scoping review

Moustafa Laymouna^1^, Kim Engler^2^, Dominic Chu^1^, Nicholas Hickens^1^, Tibor Schuster^1^, Joel Ishak^2^, Alexandra De Pokomandy ^1^, Bertrand Lebouché^3^

^1^Family Medicine, Faculty of Medicine and Health Sciences, McGill University, Montreal, Canada. ^2^Centre for Outcomes Research and Evaluation, Research Institute of the McGill University Health Centre, Montreal, Canada. ^3^Chronic Viral Illness Service, Division of Infectious Diseases, Department of Medicine, McGill University Health Centre, Montreal, Canada

**Background**: Rapid initiation of antiretroviral therapy (ART) after HIV diagnosis improves patient outcomes and reduces virus transmission earlier. However, variations in guidelines can hinder effective ART initiation. This review aims to synthesize guidelines to identify recommendations, characteristics, challenges and facilitators of rapid ART initiation, providing a clear framework for consistent implementation across diverse healthcare settings.

**Materials and methods**: A rapid scoping review of HIV guidelines from Organisation for Economic Co‐operation and Development (OECD) countries from 2017 to 2024 was conducted. Three databases were searched, with dual reviews of records, followed by independent reviews. Extracted elements included definitions, timelines, treatment regimens, population criteria, co‐infection management, drug interactions and evidence strength. Challenges, facilitators and emerging data on rapid ART initiation were also analysed.

**Results**: Twenty‐four guidelines from seven OECD countries (France, Korea, Sweden, Canada, United States, United Kingdom and Belgium) and two international health organizations (World Health Organization [WHO] and International AIDS Society‐USA [IAS‐USA]) were identified. Characteristics of rapid ART initiation included recommendations for initiation within 7 days of diagnosis in 83% of guidelines, with 62% advising same‐day initiation. Preferred regimens, mainly dolutegravir‐based therapies, were specified in 88% of guidelines. B/F/TAF (bictegravir/emtricitabine/tenofovir alafenamide) was included in 29% of guidelines. Special considerations were given for pregnant women and children in 91% and 45% of guidelines, respectively. Management of co‐infections like tuberculosis was emphasized in 88% of guidelines, with specific timing adjustments in 79%. Drug interaction management instructions were present in 83% of guidelines. Challenges included logistical issues (83%), missed clinical conditions (75%) and patient readiness issues (71%). Facilitators included streamlined care processes (79%), patient education and counselling (88%), and robust healthcare infrastructure (83%).

**Conclusions**: Rapid ART initiation is widely supported in HIV guidelines, with strong evidence for health benefits. Significant challenges require targeted interventions and further research, while streamlined processes, patient support and robust healthcare systems are crucial. Variations in recommendations and emerging data highlight the need for consistent guidelines. Future guidelines should integrate mental health support, comprehensive patient education, community engagement, telemedicine, enhanced drug interaction management and tailored recommendations for specific populations to better support rapid ART initiation.

### P149 Temporal trends from HIV diagnosis to ART initiation among adults living with HIV in the Asia‐Pacific (2013−2023)

Thinh Vu ^1^, Dhanushi Rupasinghe^2^, Vohith Khol^3^, Romanee Chaiwarith^4^, Junko Tanuma^5^, Nagalingeswaran Kumarasamy^6^, Suwimon Khusuwan^7^, I. Ketut Agus Somia^8^, Sanjay Pujari^9^, Man Po Lee^10^, Rohidas T. Borse^11^, Sasisopin Kiertiburanakul^12^, Evy Yunihastuti^13^, Iskandar Azwa^14^, Jun Yong Choi^15^, Hsin‐Pai Chen^16^, Rossana Ditangco^17^, Anchalee Avihingsanon^18^, Yasmin Gani^19^, Jeremy Ross^20^, Awachana Jiamsakul^2^

^1^Community Health and Social Sciences, CUNY School of Public Health and Health Policy, New York, NY, USA. ^2^The Kirby Institute, University of New South Wales (UNSW) Sydney, Sydney, Australia. ^3^Social Health Clinic, National Center for HIV/AIDS, Dermatology and STDs (NCHADS), Phnom Penh, Cambodia. ^4^Department of Medicine, Faculty of Medicine and Research Institute for Health Sciences, Chiang Mai University, Chiang Mai, Thailand. ^5^National Center for Global Health and Medicine, Tokyo, Japan. ^6^Chennai Antiviral Research and Treatment Clinical Research Site (CART CRS), Voluntary Health Services, Chennai, India. ^7^Chiangrai Prachanukroh Hospital, Chiang Rai, Thailand. ^8^Faculty of Medicine, Udayana University—Prof. Dr. I.G.N.G. Ngoerah Hospital, Bali, Indonesia. ^9^Institute of Infectious Diseases, Pune, India. ^10^Queen Elizabeth Hospital, Yau Ma Tei, Hong Kong. ^11^Byramjee Jeejeebhoy Government Medical College and Sassoon General Hospitals, Pune, India. ^12^Faculty of Medicine, Ramathibodi Hospital, Mahidol University, Bangkok, Thailand. ^13^Faculty of Medicine, Universitas Indonesia, Dr. Cipto Mangunkusumo General Hospital, Jakarta, Indonesia. ^14^Infectious Diseases Unit, Department of Medicine, University of Malaya, Kuala Lumpur, Malaysia. ^15^Division of Infectious Diseases, Department of Internal Medicine, Yonsei University College of Medicine, Seoul, South Korea. ^16^Taipei Veterans General Hospital, Taipei, Taiwan. ^17^Research Institute for Tropical Medicine, Muntinlupa City, Philippines. ^18^Faculty of Medicine, Chulalongkorn University, Bangkok, Thailand. ^19^Hospital Sungai Buloh, Sungai Buloh, Malaysia. ^20^TREAT Asia—amfAR, The Foundation for AIDS Research, Bangkok, Thailand

**Background**: Data on the impact of World Health Organization (WHO) guideline changes and COVID‐19 on ART initiation in the Asia‐Pacific remain scarce. This study described temporal trends from HIV diagnosis to ART initiation from 2013 to 2023.

**Materials and methods**: Adults (≥18 years) diagnosed with HIV after 2013 in the TREAT Asia HIV Observational Database Continuum of Care (TAHOD‐CC) cohort were included. Fine and Gray competing risk regression examined predictors of ART initiation (≥3 antiretroviral medications), with those who died or lost to follow‐up prior to ART initiation considered as competing risks.

**Results**: Among 14,968 participants, the majority were male (70.1%), with a median age of 36 years (interquartile range [IQR] 28–44). At HIV diagnosis, median CD4 count was 208 cells/µl (IQR 69–395), and median viral load was 86,296 copies/ml (IQR 13,186–392,000; Table 1). Over 85% of participants had initiated ART during the study period. Median time from HIV diagnosis to ART initiation differed across years of HIV diagnosis: 2.12 years (2013–2015), 0.19 years (2016–2019) and 0.15 years (2020–2023; Figure 1). Factors associated with shorter time to ART initiation were higher country income‐level (upper‐middle: sub‐distribution hazard ratio [SHR] 1.34, 95% CI 1.28–1.40; high: SHR 1.35, 95% CI 1.28–1.43; vs. lower‐middle); HIV transmission modes via male‐to‐male sex (SHR 1.06, 95% CI 1.02–1.11) or injection drug use (SHR 1.23, 95% CI 1.09–1.38; vs. heterosexual contact); and later years of HIV diagnosis (2016–2019: SHR 1.33, 95% CI 1.28–1.38; ≥2020: SHR 1.40, 95% CI 1.33–1.48; vs. 2013–2015). Those with higher CD4 counts had longer time to ART start (350–499 cells/µl: SHR 0.76, 95% CI 0.67–0.86; >500 cells/µl: SHR 0.55, 95% CI 0.49–0.61; vs. CD4 <200 cells/µl).

**Conclusions**: Time to ART initiation from HIV diagnosis decreased post‐2016, aligning with evolving WHO guidelines, regardless of COVID‐19‐related impacts. Ensuring optimal treatment initiation during the Treat All era is crucial, especially among those with higher CD4 counts.

**P149: Table 1:** Patient characteristics at HIV diagnosis in TAHOD‐CC

	Total (*N* = 14,968)	PLHIV who initiated ART (*N* = 12,749)
	*n* (%)	*n* (%)
Age at diagnosis (years)		
Median (IQR)	36.0 (28.0−44.0)	35.0 (28.0−44.0)
<30	4165 (27.8)	3741 (29.3)
30−39	4843 (32.4)	4116 (32.3)
40−49	3674 (24.5)	3024 (23.7)
≥50	2286 (15.3)	1868 (14.7)
Sex		
Male	10,497 (70.1)	9100 (71.4)
Female	4471 (29.9)	3649 (28.6)
Country income level		
Lower‐middle	6364 (42.5)	4852 (38.1)
Upper‐middle	6407 (42.8)	5814 (45.6)
High	2197 (14.7)	2083 (16.3)
Mode of HIV transmission		
Heterosexual contact	9386 (62.7)	7733 (60.6)
Men who have sex with men	4240 (28.3)	3926 (30.8)
Injecting drug use	172 (1.2)	164 (1.3)
Other	1170 (7.8)	926 (7.3)
CD4 at diagnosis (cells/µl)		
Median (IQR)	208.0 (69.0−395.0)	198.0 (66.0−372.0)
<200	1893 (12.6)	1589 (12.5)
200−349	824 (5.5)	693 (5.4)
350−499	526 (3.5)	418 (3.3)
≥500	641 (4.3)	460 (3.6)
Not reported	11,084 (74.1)	9589 (75.2)
Viral load at diagnosis (copies/ml)		
Median (IQR)	86,296.0 (13,186.0−392,000.0)	99,150.0 (17,510.0−421,683.5)
≤1000	145 (1.0)	107 (0.8)
>1000	972 (6.5)	865 (6.8)
Not reported	13,851 (92.5)	11,777 (92.4)
HCV co‐infection		
Negative	9143 (61.1)	8105 (63.6)
Positive	460 (3.1)	422 (3.3)
Not reported	5365 (35.8)	4222 (33.1)
HBV co‐infection		
Negative	10,045 (67.1)	8782 (68.9)
Positive	772 (5.2)	696 (5.5)
Not reported	4151 (27.7)	3271 (25.6)
Year of HIV diagnosis		
2013−2015	4811 (32.1)	4027 (31.6)
2016−2019	7223 (48.3)	6305 (49.4)
≥2020	2934 (19.6)	2417 (19.0)

**P149: Figure 1:**
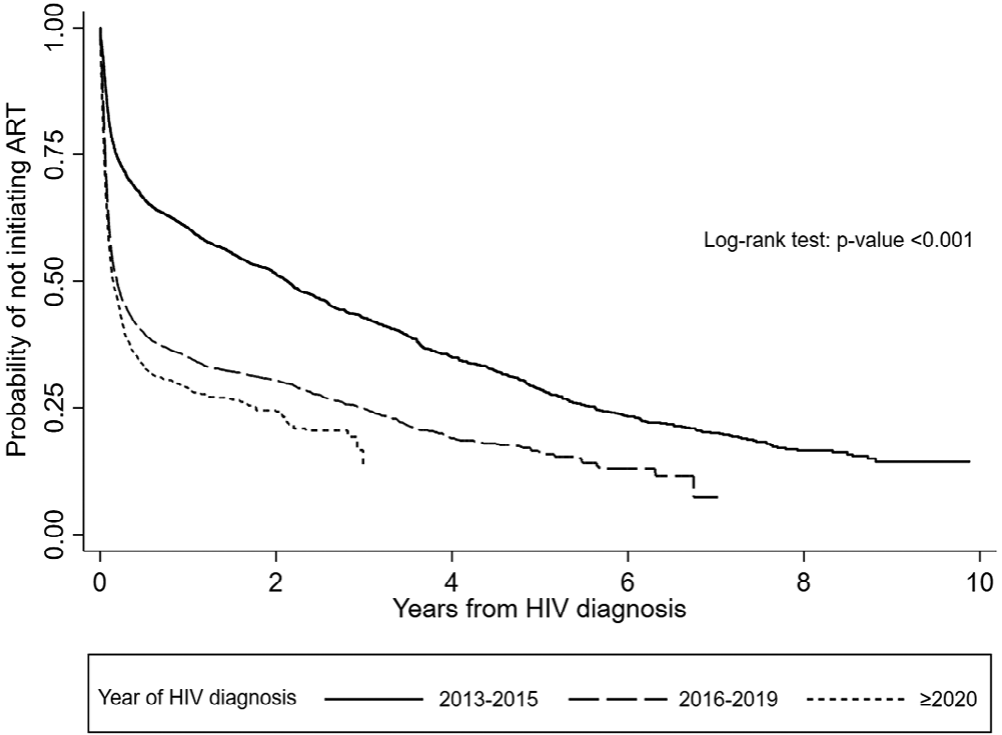
Time to ART initiation from HIV diagnosis among people living with HIV enrolled in TAHOD‐CC.

### P150 HIV‐1 infection recency and pre‐treatment drug resistance in recently diagnosed individuals in Greater Gaborone, Botswana: a cross‐sectional insight to the “Treat All” strategy

Natasha Onalenna Moraka ^1^, Marea Neo Pema^1^, Goitseone Martha Lemogang^1^, Charity Ralegoreng^2^, Thembinkosi Milili Shadreck^1^, Patrick Mokgethi^1^, Doreen Ditshwanelo^1^, Segomotso Maphorisa^3^, Modiegi Bridget Mothudi^3^, Wonderful Tatenda Choga^1^, Ontlametse Thato Bareng^1^, Dorcas Maruapula^1^, Terence Mohammed^4^, Sikhulile Moyo^4^, Simani Gaseitsiwe^4^

^1^Research Laboratory, Botswana Harvard Health Partnership, Gaborone, Botswana. ^2^Serology/Chemistry, Botswana Harvard Health Partnership, Gaborone, Botswana. ^3^HIV Drug Resistance, Botswana Ministry of Health, Gaborone, Botswana. ^4^Laboratory, Botswana Harvard Health Partnership, Gaborone, Botswana

**Background**: We aimed to identify HIV‐1 recent infection and pre‐treatment HIV‐1 drug resistance (PDR) using a two‐step recent infection testing algorithm (RITA) and population‐based sequencing in recently diagnosed, ART‐naïve individuals within the Greater Gaborone (Botswana).

**Materials and methods**: Plasma samples were collected from an ongoing prospective longitudinal cohort study recruiting individuals who are recently HIV diagnosed from government clinics within the Greater Gaborone (2023–2024), the *Tekodiso* study. All samples were collected through a census; where all participants diagnosed with HIV by double rapid HIV testing were included in the study on the same day of diagnosis before ART initiation. Recent infection classification was determined using limiting antigen (LAg) avidity and HIV viral load (VL) >1000 copies/ml. LAg‐normalized optical density (ODn) <1.5 represented recency window of within 130 days post‐infection. HIV VL in plasma was quantified by Abbott m2000sp/rt assay. Sanger sequencing of HIV *pol* was performed using the TaqPath Seq HIV‐1 Genotyping Kit (ThermoFisher Scientific, USA). Stanford HIV Drug Resistance Database was used for the identification of associated drug‐resistance mutations (DRMs).

**Results**: A total of 77/90 plasma samples were included in this analysis. Median age at enrolment was 34 years and majority 55 (61.1%) were female. The median log_10_ HIV VL was 4.7 copies/ml. A total of six (7.8%) individuals were classified as recent infections using LAg‐based RITA. Twenty‐eight (36%) samples were successfully sequenced for HIV protease and reverse transcriptase. Two (7.1%) ART‐naïve individuals had E138A major non‐nucleoside reverse transcriptase inhibitor mutation. No integrase strand transfer inhibitor (INSTI) mutations were found in 30 (39%) successfully sequenced HIV integrase samples.

**Conclusions**: We report low rates of PDR and HIV recent infection by LAg‐based RITA in recently diagnosed ART‐naïve individuals in Botswana. Our results show no INSTI‐associated HIV DRMs, supporting dolutegravir‐based first‐line treatment for recent diagnoses in Botswana.

### P151 Impact of bictegravir/emtricitabine/tenofovir alafenamide on health‐related quality of life and economic outcomes in HIV care: a sub‐study of the BIC‐NOW clinical trial

Sergio Sequera‐Arquelladas^1^, Maria J. Vivancos^2^, David Vinuesa^3^, Antonio Collado^4^, Ignacio De los Santos^5^, Patricia Sorni^6^, Noemi Cabello‐Clotet^7^, Marta Montero^8^, Carlos Ramos‐Font^9^, Alberto Terrón^10^, Maria J Galindo Puerto^11^, Onofre Juan Martínez^12^, Pablo Ryan^13^, Mohamed Omar^14^, Helena Albendin‐Iglesias^15^, Rosario Javier^1^, Miguel A. López‐Ruz^1^, Alberto Romero^16^, Coral García‐Vallecillos^1^, Miguel A. Calleja^17^, Carmen Hidalgo Tenorio ^1^

^1^Infectious Diseases, Hospital Universitario Virgen de las Nieves, Granada, Spain. ^2^Infectious Diseases Service, Hospital Ramón y Cajal, Madrid, Spain. ^3^Unit of Infectious Diseases, Hospital Universitario San Cecilio, Granada, Spain. ^4^Unit of Infectious Diseases, Hospital Universitario Torrecardenas, Almería, Spain. ^5^Infectious Diseases Service, Hospital Universitario de la Princesa, Madrid, Spain. ^6^Infectious Diseases, Hospital Universitario Son Llatzer, Palma de Mallorca, Spain. ^7^Infectious Diseases Unit, Hospital Clínico San Carlos, Madrid, Spain. ^8^Infectious Diseases Service, Hospital Universitario La Fé, Valencia, Spain. ^9^Nuclear Medicine Service, Hospital Universitario Virgen de las Nieves, Granada, Spain. ^10^Unit of Infectious Diseases, Hospital Universitario de Jerez, Cádiz, Spain. ^11^Infectious Diseases Service, Hospital Universitario Clínico de Valencia, Valencia, Spain. ^12^Unit of Infectious Diseases, Hospital Universitario Santa Lucia, Cartagena, Spain. ^13^Internal Medicine Service, Hospital Universitario Infanta Leonor, Madrid, Spain. ^14^Infectious Diseases, Complejo Hospitalario de Jaén, Jaén, Spain. ^15^HIV and STI Unit, Department of Internal Medicine, Hospital Universitario Virgen de la Arrixaca, Murcia, Spain. ^16^Infectious Diseases, Hospital Universitario de Puerto Real, Cádiz, Spain. ^17^Pharmacy, Hospital Universitario Virgen Macarena, Sevilla, Spain

**Background**: In order to assess the burden of HIV infection on people living with HIV and healthcare systems, a number of techniques have been employed, including the use of participant questionnaires and pharmacoeconomic evaluations of antiretroviral therapy. The BIC‐NOW clinical trial evaluated the efficacy, safety, treatment satisfaction, adherence and retention of a bictegravir/emtricitabine/tenofovir alafenamide rapid initiation model of care in naïve participants. This sub‐study aimed to assess changes in health‐related quality of life (HRQoL) and the economic impact of the BIC‐NOW trial.

**Materials and methods: **This phase IV, multicentre, open‐label, single‐arm, 48‐week follow‐up trial recruited participants between January 2020 and June 2022. HRQoL data were collected using the European Quality of Life 5 Dimensions 3 Level Version (EQ‐5D‐3L) and dichotomized HIV Symptom Index (HIV‐SI) questionnaires. A pharmacoeconomic cost‐utility analysis was conducted using literature data for comparators. The Moses Extreme test was used in cases where the variability between groups (AIDS and non‐AIDS) was of particular interest.

**Results: **A total of 208 participants were recruited with a mean age of 34 (IQR 27–44) years, 87.5% were male, 42.9% had completed higher education and 67.1% were employed. Mean scores on the EQ‐5D quality of life questionnaire increased significantly at 48 weeks (0.940±0.117 vs. 0.959±0.083, *p* = 0.012), with a calculated benefit of 0.877±0.093 quality‐adjusted life years (QALYs). The improvement in the usual activities dimension was significant (10.8% vs. 4.1%, *p* = 0.036), and overall, all dimensions assessed improved. Moses’ test for extremes indicated significant variability between AIDS stage and non‐AIDS participants (*p* < 0.001 for all). There was a significant reduction in reported bothersome symptoms in HIV‐SI (75.4% vs. 62.2%, *p* = 0.035) and the quantitative analysis showed a reduction in the median score for the number of bothersome symptoms (2 [IQR 1–5] vs. 1 [IQR 0–4], *p* = 0.039) at 48 weeks. The pharmacoeconomic study showed the financial feasibility of this ART with a value of 6550.21 Euro/QALY gained.

**Conclusions: **BIC/FTC/TAF is an appropriate, quality‐of‐life enhancing and pharmacoeconomically feasible option for a rapid initiation model of care in naive people living with HIV.

### P152 Multicentre, prospective cohort study of same‐day initiation of antiretroviral therapy with BIC/FTC/TAF among antiretroviral‐naïve people with HIV

Yi‐Chia Huang ^1^, Hsin‐Yun Sun^2^, Po‐Liang Lu^3^, Po‐Lin Chen^4^, Nan‐Yao Lee^4^, Sung‐Hsi Huang^1^, Chien‐Yu Cheng^5^, Shu‐Hsing Cheng^5^, Chia‐Jui Yang^6^, Hung‐Jen Tang^7^, Shih‐Ping Lin^8^, Bo‐Huang Liou^9^, Yuan‐Ti Lee^10^, Chien‐Ching Hung^1^

^1^Internal Medicine, National Taiwan University Hospital Hsin‐Chu Branch, Hsinchu County, Taiwan. ^2^Internal Medicine, Department of Internal Medicine, National Taiwan University Hospital and National Taiwan University College of Medicine, Taipei, Taiwan. ^3^Internal Medicine, Kaohsiung Medical University Hospital, Kaohsiung, Taiwan. ^4^Internal Medicine, National Cheng Kung University Hospital, College of Medicine, National Cheng Kung University, Tainan, Taiwan. ^5^Internal Medicine, Taoyuan General Hospital, Taoyuan, Taiwan. ^6^Internal Medicine, Far Eastern Memorial Hospital, New Taipei City, Taiwan. ^7^Internal Medicine, Chi Mei Medical Center, Tainan, Taiwan. ^8^Internal Medicine, Taichung Veterans General Hospital, Taichung, Taiwan. ^9^Internal Medicine, Hsinchu Mackay Memorial Hospital, Hsinchu City, Taiwan. ^10^Internal Medicine, Chung Shan Medical University Hospital, Taichung, Taiwan

**Background**: Taiwan has implemented same‐day ART initiation after the introduction of immunochromatography (ICT) to expedite the confirmation of HIV diagnosis since 2021. This ongoing multicentre, prospective cohort study aimed to investigate the clinical outcomes of people with HIV (PWH) who initiated coformulated bictegravir (BIC), emtricitabine (FTC) and tenofovir alafenamide (TAF) (BIC/FTC/TAF) within 24 hours of confirmed HIV diagnosis.

**Materials and methods**: We enrolled people who were diagnosed with HIV by ICT from January 2021 to December 2023. Baseline evaluations were conducted, and treatment with BIC/FTC/TAF was initiated within 24 hours of confirmed HIV diagnosis. The primary end points were the rates of retention in care and PWH achieving plasma HIV RNA load (PVL) <50 copies/ml per FDA Snapshot algorithm at week 48. The secondary end points were the proportions of PWH achieving PVL <50 and <200 copies/ml at weeks 1, 4 and 24, and occurrences of adverse effects (AEs).

**Results**: During the 3‐year study period, 225 newly diagnosed PWH (mean age, 34.1 years) were enrolled; 96.7% were male, and 94.2% were GBMSM. The mean CD4 count was 282 cells/mm^3^; 35.1% presented with CD4 counts <200 cells/mm^3^ or any AIDS‐defining illness; 64.9% had PVL >100,000 copies/ml and 24.9% >1,000,000 copies/ml. As of 30 June 2024, 192 participants (85.3%) had completed 48 weeks of observation. Per FDA Snapshot analysis at week 48, 67.7% of the participants achieved PVL <50 copies/ml, 18.1% ≥50 copies/ml and 14.1% no data (Figure 1). The reasons for drop‐out (*n* = 17 [7.6%], time to drop‐out, 31 days [IQR 24–139]) were loss to follow‐up (*n* = 6), participant's preference (6), skin rash (2), protocol violation (2) and transfer (1). The rates of participants with PVL <50 copies/ml at weeks 1, 4 and 24 were 5.1%, 41.6% and 68.2%, respectively, while the rates of PWH with PVL <200 copies/ml were 10.2%, 70.1% and 85.9%, respectively. Forty severe AEs were documented, but none were deemed to be related to BIC/FTC/TAF.

**Conclusions**: Among PWH who initiated same‐day ART with BIC/FTC/TAF, including a substantial proportion of PWH with high baseline PVL, two‐thirds were able to achieve PVL <50 copies/ml at week 48. No BIC/FTC/TAF‐related severe AEs were observed.

**P152: Figure 1:**
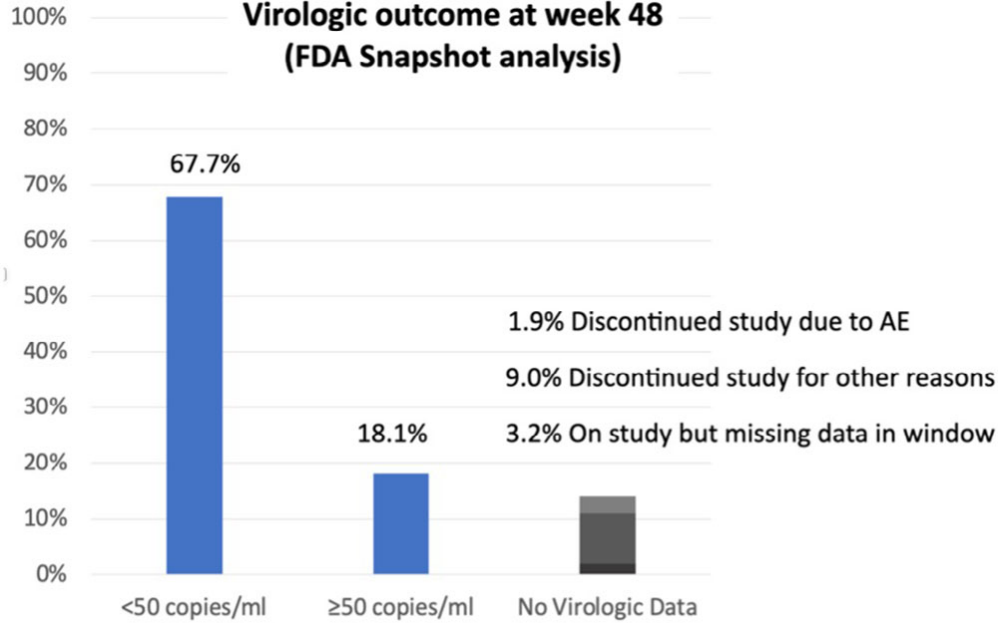
Virological outcome at week 48 (FDA Snapshot analysis).

### P153 Examining the rapid initiation of B/F/TAF strategy through a Canadian lens: an HIV transmission modelling approach

Kimberly Guinan^1^, Karine Mathurin^1^, Bertrand Lebouché ^2^, Jean Lachaine^3^

^1^Health Economics and Outcomes Research, PeriPharm Inc., Montreal, Canada. ^2^Department of Medicine, McGill University Health Centre, Montreal, Canada. ^3^Health Economics and Outcomes Research, PeriPharm Inc./University of Montreal, Montreal, Canada

**Background**: Antiretroviral therapy (ART) is the standard of care to treat HIV. While it is recommended to begin ART promptly following an HIV diagnosis to lower viral load, clinical practice often faces delays, which can increase the risk of virus transmission. The objective of this study was to estimate, from a Canadian healthcare system perspective, the epidemiological and economic impact of the rapid initiation of bictegravir/emtricitabine/tenofovir alafenamide (B/F/TAF) compared to a standard initiation.

**Materials and methods**: A dynamic transmission model for HIV was adapted to assess the impact of rapid B/F/TAF initiation (7 days from diagnosis) compared to standard initiation (45 days from diagnosis to treatment) in Canada. A Markov tree was developed over a 20‐year projection period (2020–2040). Three key subgroups were considered: heterosexual men and women, men who have sex with men (MSM) and people who inject drugs (PWID). The prevalent HIV population was divided by health state, each with a different transmission risk, as per subgroup. Infectious individuals contributed to the incidence of new infections in that year. A lifetime direct healthcare cost of HIV was applied to HIV patients. Productivity costs were added in a scenario analysis.

**Results**: The model predicts an average annual decline in HIV incidence in both strategies, estimated at 0.93% and 0.81%, in rapid B/F/TAF initiation and standard initiation, respectively. Rapid B/F/TAF initiation is expected to prevent 415 HIV infections in Canada compared to the standard initiation (Figure 1), resulting in savings of $139 million over 20 years. Nearly half (42%) of new infections avoided would be from MSM, while 33% would be from heterosexuals and 25% from PWID. When considering productivity costs, expected savings increased to $510 million. Varying the time to standard ART initiation by ±7 days results in savings ranging from $115 to $162 million over the 20‐year projection period.

**Conclusions**: This study suggests that rapid B/F/TAF initiation represents an advantageous therapeutic strategy to reduce HIV incidence and provide cost savings for the Canadian healthcare system. Consequently, efforts should be made country‐wide to adopt this strategy as the standard of care.

**P153: Figure 1:**
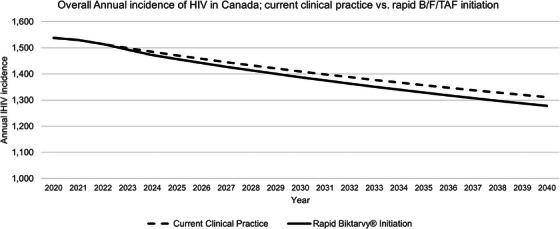
Predicted overall annual HIV incidence by strategy.

### P154 Rapid start with bictegravir/emtricitabine/tenofovir alafenamide (B/F/TAF) as initial treatment in people with HIV‐1 (PWH): a systematic literature review (SLR) of clinical and patient‐reported outcomes (PROs)

Jade Ghosn ^1^, Jeremy Chow^2^, Monica Gandhi^3^, Miguel Górgolas Hernández‐Mora^4^, Aws Al‐Hayani^5^, Hansel Tookes^6^, Max Lee^7^, Emily Kaiser^7^, David Malebranche^8^, Fernando Bognar^8^, Bhumi Gandhi‐Patel^8^, Lilli Dai^9^

^1^Department of Infectious Diseases, Hôpital Bichat, Paris, France. ^2^Department of Internal Medicine, University of Texas Southwestern Medical Center, Dallas, TX, USA. ^3^School of Medicine, University of California, San Francisco, San Francisco, CA, USA. ^4^Internal Medicine, Fundación Jiménez Díaz, Madrid, Spain. ^5^Internal Medicine, Fundación Jiménez Díaz, University Hospital, Madrid, Spain. ^6^Infectious Diseases, University of Miami—Miller School of Medicine, Miami, FL, USA. ^7^Literature Reviews and Synthesis, Costello Medical, Boston, MA, USA. ^8^Global HIV Medical Affairs, Gilead Sciences, Foster City, CA, USA. ^9^Department of Infectious Diseases, Beijing Youan Hospital, Capital Medical University, Beijing, China

**Background**: HIV treatment guidelines recommend rapid antiretroviral therapy (ART) initiation in eligible PWH to increase ART uptake, improve engagement in care and reduce time to viral suppression, thereby improving individual health and reducing HIV transmission [1−3]. B/F/TAF, an integrase strand transfer inhibitor‐based single‐tablet regimen [4], has demonstrated efficacy and safety, and is guideline‐recommended for rapid start [1,2]. However, a comprehensive analysis of all published research on rapid start with B/F/TAF is lacking. The objective of this SLR is to synthesize evidence on the efficacy, safety and effect on PROs of B/F/TAF rapid start among ART‐naïve PWH.

**Materials and methods**: MEDLINE, Embase, Cochrane Database of Systematic Reviews and Cochrane Central Register of Controlled Trials databases were searched in January 2024, supplemented by searches of conference proceedings and the International Clinical Trials Registry Platform. Interventional studies of B/F/TAF rapid start, in ART‐naïve‐adult‐PWH, reporting efficacy and safety outcomes, and PROs were eligible. Study quality was assessed using York Centre for Reviews and Dissemination or Risk Of Bias In Non‐randomised Studies‐of Interventions (ROBINS‐I) checklists.

**Results**: Eight rapid start studies were included (Table 1 and Table 2). Virological suppression (HIV‐1 RNA <50 copies/ml) in B/F/TAF rapid start groups was observed in ≥80% PWH at week (W) 24 across four single‐arm studies. In two studies comparing B/F/TAF rapid start versus any non‐rapid ART, virological suppression and engagement in care favoured rapid start. Of three studies reporting number of patients with adverse events (AEs), <2% of B/F/TAF rapid start patients experienced grade 3/4 AEs. Four studies reported low study discontinuation rates due to AEs (≤3%) for B/F/TAF rapid start. Four studies reported PROs such as anxiety reduction, health‐related quality of life (HRQoL) and treatment satisfaction. On B/F/TAF rapid start, anxiety decreased through W48 in two studies. HRQoL (measured by EuroQoL‐5‐Dimensions EQ‐5D) significantly improved in one study through W48. Two studies noted high patient satisfaction and favourable experience on B/F/TAF rapid start.

**P154: Table 1:** Efficacy and safety outcomes at key timepoints reported by included rapid start (RS) studies

Characteristic/Endpoints	BIC‐NOW (Spain) [5]	BIFAST (Spain) [6]	FAST (France) [7]	Rainbow (Italy) [8]	Test&Treat (Spain) [9]	Benidir 2022 (USA) [10]	
Study design	Single arm	Single arm	Single arm	Single arm	Single arm	Non‐RCT (RS vs non‐RS)	
Phase	IV	IV	IV	IV	III	NR	
Treatment arms	B/F/TAF RS (n=208)	B/F/TAF RS (n=59)	B/F/TAF RS (n=112)	B/F/TAF RS (n=30)	B/F/TAF RS (n=100)	B/F/TAF RS (n=65)	B/F/TAF non‐RS (n=42)
Efficacy Outcomes							
Virologic suppression (Viral load <50 copies/mL)	W24 (n=160), 88.8%	W24 (n=59), 84.4%	W24 (n=112), 80.4%	W24 (n=30), 80%, p < 0.001	W4 (n=100), 52%	Visit 5 (timepoint NR) (n=65), 100%, p ≥ 0.05	Visit 5 (timepoint NR) (n=42), 100%
Engagement in care (Attendance at visits)				W48 (n=30), 100%	W4 (n=100), 100%	Visit 4 (median 192.0 [IQR 185.0,238.0] days) (n = 16), 56.3%, p = 0.003	Visit 4 (median 243.5 [IQR 180.2, 306.8] days) (n = 22), 9.1%
Safety Outcomes							
Grade 3 or 4 AEs	W48, (n = 208), 0% of patients						
TRAEs			W48, (n = 112), 0% of events [Grade 3/4/SAE]	W48, (n = 209), 7% of events [Any grade]			
Study discontinuation due to AE	W48 (n = 208), 0%		W48 (n = 112), 2.7%	W48 (n = 30), 0%			

Characteristic/Endpoints	BIC T&T (UK) [11]		Lv 2023 (China) [12]				
Study design	RCT (RS vs RS)		RCT (RS vs non‐RS, B/F/TAF and EFV+3TC+TDF)				
Phase	III		NR				
Treatment arms	B/F/TAF RS (n = 19)	DRV/COBI/FTC/TAF RS (n = 17)	B/F/TAF RS (n = 132)	B/F/TAF non‐RS (n = 91)	EFV+3TC+TDF RS (n = 126)	EFV+3TC+TDF non‐RS (n = 122)	
Efficacy Outcomes							
Virologic suppression (Viral load <50 copies/mL)			W24 (n = 108), 93.5%		W24 (n = 91), 74.7%, p<0.001 vs. B/F/TAF RS	W24 (n = 88), 76.1%, p = 0.16 vs. EFV+3TC+RDF RS	
Engagement in care (Attendance at visits)			W24, combined RS (n = 258), 92.6%, p = 0.053	W24, combined non‐RS (n = 213), 86.9%	Data reported in B/F/TAF RS column	Data reported in B/F/TAF non‐RS column	
Safety Outcomes							
Grade 3 or 4 AEs	W48, (n = 19), 0% of patients	W48, (n = 17), 0% of patients	Timepoint NR, (n = NR), 1.9% of patients		Timepoint NR, (n = NR), 1.1% of patients		
TRAEs			Timepoint NR, (n = 108), 13.9% of patients [Grade NR]		Timepoint NR, (n = 91), 31.9% of patients [Grade NR]		
Study discontinuation due to AE			W24 (n = 108), 0%		W24 (n = 121), 4.9%	W24 (n = 122), 4.1%	

**P154: Table 2:** Patient reported outcomes at key timepoints reported by included rapid start (RS) studies

Characteristic/Endpoints	BIC‐NOW (Spain) [5]	BIFAST (Spain) [6]	FAST (France) [7]	Rainbow (Italy) [8]	Test&Treat (Spain) [9]
Anxiety			STAI‐Y mean score (n = 448): BL: 52; W48: 37	BAI (n = NR): BL, score <16: 23 patients; score ≥16: 4 patients; W48, score <16 : 20 patients; score ≥16 : 2 patients p = 0.678 [all]	
Patient satisfaction	W48 (n = 178), mean score 90 (assessment method and scale NR)	Numerical value not presented	Timepoint NR HIVTSQc mean score (general) (n = NR), 25.9‐26.8 out of 30 (p<0.05) Opinion of same‐day treatment initiation and continuing B/F/TAF average score (n = 112) ∼8‐9 out of 10		
HRQoL				EQ‐5D mean score (n = NR): BL score: 57; W48: 75 p = 0.001; SF‐12 mean score (n = NR): BL score 48; W48: 51 Not significant, p = NR	
Depression				BDI II mean score (n = NR): BL, score <20: 25 patients; ≥20 : 2 patients; W48, score <20 : 21 patients ; ≥20 : 1 patient p = 1.000 [all]	
Sleep				Pittsburgh Sleep Quality Index (n = NR) BL, ≤5: 20 patients; >5: 5 patients; W48, ≤5: 19 patients; >5: 3 patients p = 0.706 [all]	
Stress			Timepoint NR, level of stress with same‐day initiation mean score (n = NR) 6.3 out of 10		

**Conclusions**: Results of this SLR demonstrate favourable efficacy and safety for B/F/TAF rapid start, supporting current HIV treatment guideline recommendations. B/F/TAF rapid start was also associated with positive PROs including high treatment satisfaction, improved health‐related quality of life and reduced anxiety.

**References**

1. Clinicalinfo.HIV.gov. DHHS Guidelines for the Use of Antiretroviral Agents in Adults and Adolescents with HIV, Dec 2023; B2, E2‐E4 [Internet]. 2023 [cited 2024 Feb 7]. Available from: https://clinicalinfo.hiv.gov/sites/default/files/guidelines/documents/adult‐adolescent‐arv/guidelines‐adult‐adolescent‐arv.pdf.
2. Hidalgo‐Tenorio C, Sequera S, Vivancos MJ, Vinuesa D, Collado A, De Los Santos I, et al. Bictegravir/emtricitavine/tenofovir alafenamide as first‐line treatment in naïve HIV patients in a rapid‐initiation model of care: BIC‐NOW clinical trial. HIV Med. 2023;24(Suppl. 5):212.
3. Al‐Hayani AWM, Carrillo I, Cabello A, Algar C, Prieto‐Pérez L, Ãlvarez B, et al. Starting antiretroviral therapy (ART) at the first HIV‐specialist appointment with or without baseline laboratory data with BIC/FTC/TAF (The BIFAST study). J Int AIDS Soc. 2022;25(Suppl. 3):e25935.
4. Camici M, Gagliardini R, Lanini S, Del Duca G, Mondi A, Ottou S, et al. Rapid ART initiation with bictegravir/emtricitabine/tenofovir alafenamide in individuals presenting with advanced HIV disease (Rainbow study). Int J Antimicrob Agents. 2024;63:107049.
5. Ugarte A, De La Mora L, Chivite I, de Lazzari E, Fernández E, Solbes E, et al. Rapid initiation of antiretroviral therapy (ART) with bictegravir/emtricitabine/tenofovir alafenamide (BIC/FTC/TAF) in a tertiary hospital in Barcelona, Spain: a prospective clinical trial. J Int AIDS Soc. 2022;25(Suppl. 6):e26009.
6. Benidir B, Ali T, Puga Sanchez L, Salunkhe V, Gootee S, Arnold FW. Impact of rapid initiation of antiretroviral therapy on retention in care in the Southern United States. Open Forum Infect Dis. 2022;9(Suppl. 2):S545.
7. Whitlock G, Fidler S, Clarke A, Xhikola. A randomised control trial of BIC/F/TAF vs DRV/c/F/TAF in context of HIV test‐and‐treat. HIV Med. 2023;24(Suppl. 5):212‐3.
8. Lv S, Sun L, Ma P, Wang L, Zhou Y, Wu C, et al. Rapid ART initiation using BIC/FTC/TAF and TDF+3TC+EFV in people with HIV in China. Top Antivir Med. 2023;31(2):205.
9. Ugarte A, De La Mora L, Chivite I, de Lazzari E, Fernández E, Solbes E, et al. Rapid initiation of antiretroviral therapy (ART) with bictegravir/emtricitabine/tenofovir alafenamide (BIC/FTC/TAF) in a tertiary hospital in Barcelona, Spain: a prospective clinical trial. J Int AIDS Soc. 2022;25(Suppl. 6):e26009.
10. Benidir B, Ali T, Puga Sanchez L, Salunkhe V, Gootee S, Arnold FW. Impact of rapid initiation of antiretroviral therapy on retention in care in the Southern United States. Open Forum Infect Dis. 2022;9(Suppl. 2):S545.
11. Whitlock G, Fidler S, Clarke A, Xhikola. A randomised control trial of BIC/F/TAF vs DRV/c/F/TAF in context of HIV test‐and‐treat. HIV Med. 2023;24(Suppl. 5):212‐3.
12. Lv S, Sun L, Ma P, Wang L, Zhou Y, Wu C, et al. Rapid ART initiation using BIC/FTC/TAF and TDF+3TC+EFV in people with HIV in China. Top Antivir Med. 2023;31(2):205.

### P155 Same‐day initiation with bictegravir/emtricitabine/tenofovir alafenamide: real‐life experience from a centralized single‐centre model of care for people living with HIV in Croatia

Josip Begovac, Iva Lisicar, Vanja Romih Pintar, Sime Zekan

HIV/AIDS Department, University Hospital for Infectious Diseases, Zagreb, Croatia

**Background**: Bictegravir/emtricitabine/tenofovir alafenamide (B/F/TAF) is a recommended first‐line antiretroviral (ART) regimen [1]. Croatia has centralized care for people living with HIV (PLWH), with all persons treated in a single centre, preferably in a same‐day initiation model whenever suitable [2,3]. This retrospective cohort study evaluated ART‐naïve PLWH who initiated B/F/TAF in a same‐day model.

**Methods**: We collected information from the electronic database of the Croatian HIV Reference Centre and identified 108 PLWH who started B/F/TAF at their first clinical visit from May 2019 until December 2022 and were followed up until March 2024. B/F/TAF was initiated in all subjects within 24 hours. We present our efficacy results on the whole population (intent‐to‐treat, ITT) and on those evaluated (on‐treatment, OT).

**Results**: A total of 108 PLWH were included; the mean age was 38.6 years, and 103 (95.4%) were males. The mean CD4 count was 340.7 cells/µl (26.9% had a CD4 count <200 cells/µl), clinical AIDS was present in eight (7.4%) and the mean HIV‐1 RNA was 4.9 log_10_ copies/ml (43.5% had >100,000 copies/ml). Acute/recent infection was diagnosed in 32 (29.6%), four (3.9%) were HBsAg positive and one had anti‐HCV antibodies. At 6 months (range 4–8), the efficacy (HIV‐1 RNA <50 copies/ml) on ITT was 83.3%. The OT population included 98 PLWH, 90 (91.8%) had <50 copies/ml, and eight (8.2%) had between 50 and 200 copies/ml. Of the 10 PLWH who did not have HIV‐1 RNA measurements, five subsequently had an undetectable viral load (VL), three were lost to follow‐up, one moved and one died. At 12 months (range 9–15), the efficacy (HIV‐1 RNA <50 copies/ml) on ITT was 78.7%. The OT efficacy (HIV‐1 RNA <50 copies/ml) was 91.4% (85/93), and eight (8.6%) had between 50 and 200 copies/ml. Of the 15 PLWH who did not have VL measurements, nine subsequently had an undetectable VL, three were lost to follow‐up, two moved and one died.

**Conclusions**: In our real‐life setting, same‐day treatment with B/F/TAF was virologically successful, and no documented discontinuations of B/F/TAF were observed. B/F/TAF was an efficacious option for treatment‐naïve PLWH in a same‐day start ART model.

**References**

1. European AIDS Clinical Society (EACS). EACS guidelines, version 12.0 October 2023 [Internet]. 2023 [cited 2024 Jun 25]. Available from: https://www.eacsociety.org/guidelines/eacs‐guidelines.

2. Bogdanić N, Bendig L, Lukas D, Zekan Š, Begovac J. Timeliness of antiretroviral therapy initiation in the era before universal treatment. Sci Rep. 2021;11:10508.

3. Begovac J, Bogdanić N, Močibob L, Lukas D, Zekan Š. Antiretroviral therapy initiation at entry into HIV care, Croatia, 2015–2020. AIDS 2022: 24th International AIDS Conference; 2022 Jul 29‐Aug 2; Montreal, Quebec, Canada. Poster EPC184.

### P156 Effectiveness and safety of rapid initiating with BIC/FTC/TAF regimen in advanced people with HIV: a real‐world study from southwest of China (EVIDENCE study)

Yingchun Zhu, Lin Cai, Yin Wang, Buyun Wang

Infectious Disease Department, Public Health Clinical Center of Chengdu, Chengdu, China

**Background**: This study is aimed to evaluate the effectiveness and safety of rapid initiating antiretroviral therapy (ART) of BIC/FTC/TAF within 7 days of confirmed diagnosis in advanced HIV‐1 treatment‐naive patients.

**Methods**: A single‐centre retrospective study was conducted analysing advanced HIV‐1 treatment‐naive patients (CD4^+^ T‐cell count ≤200 cells/ml and/or with AIDS defined diseases) who initiated BIC/FTC/TAF regimen within 7 days of diagnosis between June 2021 and October 2023. Primary end point was the proportion of achieving HIV‐1 RNA <50 copies/ml after 24 weeks of treatment. Secondary end points were changes in CD4 T‐cell count, CD4/CD8 ratio, renal function, blood lipids and liver function.

**Results**: A total of 54 patients were included, 46 males (85.2%) and eight females (14.8%), with a median age of 42 years (22–86 years). Eighteen cases (33%) had opportunistic infections, including four cases of pneumocyte pneumonia, eight cases of bacterial pneumonia, one case of fungal pneumonia, one case of Penicillium marneffei, five cases of other disease. Baseline median HIV‐1 RNA was 5.18 log_10_ copies/ml, and median CD4^+^ T‐cell count was 119.00 cells/µl. The median time to ART initiation was 4 days. At 4 weeks, the median decrease in HIV‐1 RNA was 3.29 log_10_ copies/ml. At 24 weeks, viral suppression (HIV‐1 RNA <50 copies/ml) rate was achieved in 86.27% (*n* = 44) of patients. While 11.76% (*n* = 6) had 50< HIV‐1 RNA <200 copies/ml, and 1.96% (*n* = 1) had HIV‐1 RNA >200 copies/ml (691 copies/ml, a decrease of 3 log_10_ copies/ml from baseline). CD4^+^ T‐cell count increased from 121.5 cells/µl at baseline to 240.5 cells/µl at 24 weeks (*p* < 0.01), and CD4/CD8 ratio increased from 0.15 to 0.27 (*p* < 0.01). At 48 weeks, viral suppression rate was observed in 92.59% (*n* = 50) of patients, with all four patients not achieving virological suppression having HIV RNA <100 copies/ml. No treatment discontinuations due to adverse reactions or incidences of immune reconstitution inflammatory syndrome (IRIS) were observed.

**Conclusions**: This real‐world study demonstrates that rapid initiation of ART with the BIC/FTC/TAF regimen in advanced treatment‐naive HIV patients achieves favourable virological suppression and immunological responses, with well safety profile.

### P157 **Rapid initiation of antiretroviral therapy under the Treat‐All policy reduces loss to follow‐up and virological failure in routine HIV care settings in China: a retrospective cohort study (2016**−**2022)**

Huan Xia, Ping Ma

Department of Infectious Diseases, Tianjin Second People's Hospital, Tianjin, China

**Background**: Following the World Health Organization's guidelines for rapid ART initiation (≤7 days after HIV diagnosis), China implemented Treat‐All in 2016 and has made significant efforts to provide timely ART since 2017. The purpose of this study was to evaluate the effects of rapid ART on loss to follow‐up (LTFU) and virological failure compared to those of delayed ART.

**Methods**: This research included newly diagnosed HIV‐positive adults from Tianjin, China between June 2016 and December 2022. Our primary outcome was LTFU (more than 90 days after the prior clinical visit) at 12 months following enrolment. The secondary outcome was 12‐month virological failure after treatment initiation. Furthermore, Kaplan‐Meier estimators were utilized to examine the LTFU time for rapid and delayed ART initiation. Moreover, the correlation between rapid ART initiation and LTFU was evaluated using univariate and multivariable Cox regression, whereas the relationship between rapid ART and 12‐month virological failure was investigated via logistic regression.

**Results**: A total of 896 (19.1%) of 4688 participants received ART ≤7 days post‐HIV diagnosis. The rate of rapid ART initiation has increased from 7.5% in 2016 to 33.3% by 2022. Rapid ART was less common in persons with baseline CD4 counts of 350–499 and ≥500 compared to those <200. Furthermore, it was also less prevalent in individuals who were diagnosed with tuberculosis or had an unknown route of HIV infection, while was more common in people who used Medicare/self‐paid medications. The rapid ART group had an LTFU rate of 3.3%, as opposed to 5.0% in the delayed initiation group. Moreover, the rapid ART group had a much‐reduced virological failure rate (0.6% vs. 1.8%). Rapid ART individuals had a reduced likelihood of LTFU (adjusted hazard ratio 0.65, 95% CI 0.44–0.96) and virological failure (adjusted odds ratio 0.35, 95% CI 0.12–0.80).

**Conclusions**: Under China's Treat‐All policy, rapid ART initiation reduced the risk of LTFU and virological failure. The real‐world data indicated that rapid ART initiation is practicable and beneficial for Chinese people with HIV, providing evidence and guide for its widespread implementation and scaling up.

### P158 Rapid start: evaluating the time to HIV therapy initiation in a tertiary care hospital in Portugal (2020−2023)

Tiago Neves, Mariana Lopes, Helena Alves, Cristina Valente

Infectious Diseases, Unidade Local de Saúde de Coimbra, Coimbra, Portugal

**Background**: The rapid initiation of antiretroviral therapy (ART) within 7–14 days of diagnosis has become standard practice in many parts of the world and has been shown to reduce HIV‐related morbidity and mortality, as well as decrease the risk of transmission. Portugal, which has one of the highest rates of new HIV diagnoses in the European Union, faces challenges in adhering to national guidelines for timely ART initiation. This retrospective study assessed the period from referral to the first hospital consultation and consultation to ART initiation among newly diagnosed individuals.

**Materials and methods**: All first HIV consultations at Unidade Local de Saúde de Coimbra from 2020 to 2023 were included, with the exception of individuals with a previous HIV diagnosis and ongoing ART. Baseline demographic characteristics, origin of referral, date of referral, date of first appointment, date of ART initiation and the stage of HIV infection according to the CDC classification system (1993) were recorded.

**Results**: Of a sample size of 815 individuals, 228 were included in this study, 78.9% were male, the median age was 44 years and the majority (81.1%) had Portuguese nationality. The number of new diagnoses was higher in 2023 (67 cases). Concerning the origin of referral, 43.4% were from primary healthcare units, 41.6% from hospital care and 15% from other sites. The median number of days from initial referral to the first HIV consultation and also from consultation to ART initiation was 7 days. The median number of days from referral to start of therapy was 22 days in 2020, 18 days in 2021, 21 days in 2022 and 15 days in 2023. At diagnosis, 126 were asymptomatic (categories A1, A2 and A3), accounting for 45.8% of cases in 2020 and 67.1% in 2023.

**Conclusions**: This study evidenced an improvement in waiting time between referral and the start of ART, and an increase in the number of asymptomatic cases, which could highlight the role of both healthcare and non‐profit organizations in early referral. Continued monitoring of these timelines is essential to optimize health outcomes for individuals living with HIV.

### P159 Effectiveness and safety analysis of B/F/TAF for rapid initiation of therapy in people with HIV

Yuanhong He^1^, Lin Liu^2^, Tongtong Yang ^1^, Jinghong Li^3^, Dianxia Cheng^1^, Ruifeng Zhou^1^

^1^Communicable Diseases Department 1, Chengdu Public Health Clinical Medical Center, Chengdu, China. ^2^Department of Public Health, Chengdu Medical College, Chengdu, China. ^3^HIV Care Center, Chengdu Public Health Clinical Medical Center, Chengdu, China

**Background**: Guidelines recommend rapid initiation of antiretroviral therapy (ART) for patients newly diagnosed with HIV‐1 infection, but data in China is limited. This study aimed to evaluate the effectiveness, safety and patient satisfaction of B/F/TAF for rapid initiation therapy in people with HIV (PWH).

**Methods**: This is a single‐centre, retrospective cohort study which analysed patients who initiate B/F/TAF therapy within 7 days of diagnosis at Chengdu Public Health Clinical Medical Center from May 2020 to January 2024. Baseline characteristics, HIV‐1 RNA virological suppression rate, CD4^+^ T‐cell count changes, safety and patient satisfaction surveys were evaluated.

**Results**: One hundred and eleven patients were included with a median age of 39 (30–55) years. Median baseline HIV‐1 RNA was 8.91 (2.18–23.1) log_10_ copies/ml, the rate of HIV‐1 RNA >100,000 copies/ml was 46.8%. Median CD4^+^ T‐cell count was 255 (141,376) cells/µl, with 36.0% having CD4 <200 cells/µl. 8.1% of cases had HBV co‐infection. Median time from diagnosis to initiation of B/F/TAF was 4 (1–6) days and 25.2% of cases initiated therapy on the same day. The virological suppression rate, HIV‐1 RNA <50 copies/ml, at 4 and 24 weeks were 64% (57/89) and 86.7% (91/105), respectively. At week 4, median decline of HIV‐1 RNA was 4.84 log_10_ copies/ml. CD4^+^ T‐cell counts significantly increased at 4 and 24 weeks (*p* < 0.001), with median increases of 61 and 165 cells/µl from baseline, respectively. Discontinuation rate due to adverse events was 0%. Ninety‐five percent of patients were satisfied or extremely satisfied with B/F/TAF taking.

**Conclusions**: B/F/TAF has good virological suppression rate, immunological reconstitution effect, safety and high patient treatment satisfaction. B/F/TAF could be a preferred regimen for rapid initiation of ART in PWH.

## Treatment strategies—Adherence

### P160 Real‐world utilization of cabotegravir/rilpivirine: an observational analysis of adherence and persistence using a patient support programme in Canada, preliminary results

Callahan LaForty^1^, Adenike Adelakun ^2^, Ryan Ng^3^, Bo Chen^4^, Maria Esther Perez Trejo^5^, Lidija Latifovic^4^, Simbarashe Mhishi^2^, Joann Ban^2^

^1^IQVIA Solutions Inc., Mississauga, Canada. ^2^Health Economics and Outcomes Research, GlaxoSmithKline Inc., Mississauga, Canada. ^3^Health Access and Outcomes, IQVIA Solutions Inc., Mississauga, Canada. ^4^Real World Solutions, IQVIA Solutions Inc., Mississauga, Canada. ^5^Real World Evidence, IQVIA Solutions Inc., Mississauga, Canada

**Background**: To achieve viral suppression and prevent HIV transmission, optimal adherence to antiretroviral therapy (ART) is required. However, persons living with HIV (PWH) continue to have suboptimal adherence to oral ARTs leading to treatment failure, increased risk of morbidity/mortality, decreased quality of life and substantial economic burden. Cabotegravir/rilpivirine long‐acting (CAB+RPV LA) injectable is the only complete long‐acting regimen for virologically suppressed PWH, reducing dosing from 365 days (daily single or multi‐tablet regimens) to 6 days (every 2 months dosing) or 12 days (monthly dosing) per year. This study evaluates real‐world adherence and persistence to CAB+RPV LA among PWH who are enrolled in the Canadian CAB+RPV LA patient support programme (PSP). The PSP navigates reimbursement pathways for patients with PSP nurses administering CAB+RPV LA.

**Materials and methods**: Retrospective interim analysis of the CAB+RPV LA PSP data was conducted (18/10/2022−30/05/2024). Participants in the CAB+RPV LA PSP were followed from their first injection (index date) with the PSP to the interim data cut‐off date (30 May 2024). Adherence, defined as proportion of days covered (PDC) ≥90%, and persistence (duration of time from index date to discontinuation or end of analysis period) were evaluated for those with 365 days follow‐up from index date. PDC was calculated as the ratio of days covered by medication to the total days in the period.

**Results**: Six hundred and twenty‐nine eligible PWH were identified for this interim analysis (mean age: 46.5±12.3 years; male: 75.4%; oral lead‐in: 24.2%; ≥365 days of follow‐up: 37%/233 PWH). For the 233 PWH with at least 365 days of follow‐up, 211 PWH (90.6%) followed a 2‐month CAB+RPV LA dosing schedule, while 3.9% switched from monthly to every 2 months. 98.7% of the 233 PWH receiving CAB+RPV LA in the PSP were adherent (PDC ≥90%) over a 365‐day follow‐up period. The median (IQR) injections per participant through follow‐up was 7 (7–7). 95.3% of patients remained persistent through the 365‐day analysis period.

**Conclusions**: High adherence and persistence to CAB+RPV LA were observed among Canadian PWH receiving their injections through the PSP. Long‐acting injectables may improve adherence and persistence, potentially ensuring the long‐term treatment success.

### P161 Effectiveness of enhanced adherence counselling among adolescents receiving dolutegravir‐containing regimens with detectable viraemia in Cameroon: optimizing virological outcomes in paediatric settings

Suzie Moyo Tetang^1^, Yagai Bouba^2^, Alex Durand Nka ^2^, Aude Christelle Ka'e^2^, Rachel Kamgaing^3^, Davy Hyacinthe Gouissi Anguechia^2^, Nelly Kamgaing^4^, Michel Carlos Tommo Tchouaket^2^, Nadine Nguendjoung Fainguem^2^, Annie Nga Motaze^1^, Francis Ateba^5^, Desire Takou^2^, Lum Forgwei^6^, Dominik Guebiapsi Tameza^7^, Cynthia Ayafor^8^, Efakika Gabisa Jeremiah^2^, Michelle A. Abate^9^, Felicite Noukayo^10^, Suzanne Essamba^11^, Alice Ketchaji^12^, Liman Yakouba^11^, Ezechiel Ngoufack Jagni Semengue^2^, Basseck Roland Wome^13^, Agabus Wiadamong^8^, Abdou Gnambi^6^, Etame Naomi‐Karell^2^, Aurelie Minelle Kengni Ngueko^2^, Larissa Moko^2^, Junie Flore Yimga^2^, Collins Ambe Chenwi^2^, Grace Beloumou^2^, Sandrine Djupsa^2^, Rita Ekwoge Mejane^2^, Samuel Martin Sosso^2^, Rogers A. Ajeh^14^, Abdelkader Bacha^15^, Patrice Tchendjou^16^, Anne Esther Njom Nlend^17^, Paul Koki Ndombo^18^, Daniele Armenia^19^, Vittorio Colizzi^20^, Francesca Ceccherini‐Silberstein^21^, Maria Mercedes Santoro^21^, Alexis Ndjolo^2^, Carlo‐Federico Perno^22^, Joseph Fokam^2^

^1^Pediatrics, Essos Hospital Centre, Yaounde, Cameroon. ^2^Virology, Chantal Biya International Reference Centre for Research on the Prevention and Management of HIV/AIDS (CIRCB), Yaounde, Cameroon. ^3^Medical Laboratory Analysis, Chantal Biya International Reference Centre for Research on the Prevention and Management of HIV/AIDS (CIRCB), Yaounde, Cameroon. ^4^Technical Medical Unit, Chantal Biya International Reference Centre for Research on the Prevention and Management of HIV/AIDS (CIRCB), Yaounde, Cameroon. ^5^Pediatrics, Central African Protestant University, Yaounde, Cameroon. ^6^Virology, Central African Catholic University, Yaounde, Cameroon. ^7^Virology, Central Technical Group, National AIDS Control Committee, Yaounde, Cameroon. ^8^Virology, Central African Protestant University, Yaounde, Cameroon. ^9^Virology, Integrated Research Group, Yaounde, Cameroon. ^10^Virology, Mbalmayo District Hospital, Mbalmayo, Cameroon. ^11^Virology, Cite‐Verte District Hospital, Yaounde, Cameroon. ^12^Virology, Department of Disease, Epidemics and Pandemic Control, Ministry of Public Health, Yaounde, Cameroon. ^13^Virology, University of Yaounde I, Yaounde, Cameroon. ^14^Virology, HIV, Tuberculosis and Malaria Global Funds Subvention Coordination Unit, Ministry of Public Health, Yaounde, Cameroon. ^15^Virology, UNICEF‐HIV/AIDS and Adolescents, Country Office, Dakar, Senegal. ^16^Virology, Elizabeth Glaser Pediatric AIDS Foundation, Country Office, Douala, Cameroon. ^17^Pediatrics, Health Ebene Consulting, Yaounde, Cameroon. ^18^Pediatrics, Mother and Child Centre, Chantal BIYA Foundation, Yaounde, Cameroon. ^19^Virology, UniCamillus—Saint Camillus International University of Health Sciences, Rome, Italy. ^20^Immunology, Chantal Biya International Reference Centre for Research on the Prevention and Management of HIV/AIDS (CIRCB), Yaounde, Cameroon. ^21^Virology, University of Rome Tor Vergata, Rome, Italy. ^22^Virology, Bambino Gesu Pediatric Hospital, Rome, Italy

**Background**: In low‐ and middle‐income countries (LMICs), mortality among adolescents living with HIV (ADLHIV) is still concerning, driven by poor viral suppression (VS) in the frame of limited adherence monitoring approaches. We herein evaluated the virological outcomes after enhanced adherence counselling (EAC) among ADLHIV with non‐VS and low‐level viraemia (LLV) in the era of tenofovir‐lamivudine‐dolutegravir (TLD) in Cameroon.

**Materials and methods**: In the frame of the CIPHER‐ADOLA project, we conducted a multicentre facility‐based cohort‐study among ADLHIV (10–19 years) in Cameroon. Three EAC sessions were provided to ADLHIV with a viral load (VL) ≥50 copies/ml for a period of 3 months. VS, LLV and virological failure (VF) were defined as VL <1000, 50–999 and ≥1000 copies/ml, respectively. The outcome after EAC among ADLHIV with LLV and VF was, respectively, to achieve undetectability and VS.

**Results**: Of 252 ADLHIV enrolled for VL testing, 70 (27.8%) had a VL ≥50 copies/ml (female: 58.6%; median [IQR] age 15 [13–17]). Pre‐TLD backbones included ABC+3TC (50.0%), TDF+3TC (41.4%) and AZT+3TC (8.6%); median TLD duration was 23.5 [±11.6] months. Before EAC, 72.9% (51/70) had poor ART adherence; 44.6% and 55.4% had VLs 50–999 and ≥1000, respectively. At the end of follow‐up, 60.7% had good adherence; with higher rates of adherence associated with being female (22/37 [59.4%]) versus male (15/26 [57.7%]; *p* = 0.889) and attending all three EAC sessions (57.1% [32/56] vs. 42.9% [24/56]; *p* = 0.001). Among the 56 ADLHIV with available VLs post‐EAC, 58.9%, 19.6% and 21.4% had VLs <50, 50–999 and ≥1000, respectively, indicating an overall 33% reduction in poor VS. Interestingly, among ADLHIV with VL ≥1000 at baseline, 70% achieved VS and 30% experienced VF after EAC. In LLV group at baseline, 74% had a VL <50 and 26% remain in LLV. Despite a non‐significant decline in median VL (126 [52–1871] post‐EAC vs. 135 [95–8519] at baseline; *p* = 0.945), viral non‐suppression and LLV significantly declined from 55.4% to 21.4% (*p* = 0.0002) and 44.6% to 19.6% (*p* = 0.0046), respectively, after EAC. Overall, 73.2% significant decline in VLs (−0.5 log_10_ RNA) was observed between pre‐ and post‐EAC.

**Conclusions**: In LMICs transitioning to TLD, an effective EAC would substantially improve ART outcomes among ADLHIV experiencing non‐VS. In context, LMICs are encouraged to accompany the transition to paediatric dolutegravir‐containing regimens with a robust adherence support strategy for ADLHIV with detectable VL to achieve elimination of paediatric AIDS by 2030.

### P162 Factors associated with lost to follow‐up (LTFU) on TB treatment at a large volume clinic in Kampala, Uganda

Lyness Bitira, Mark Muhumuza, Prisca Mwanga, Patrick Kazooba

Medical, Reach Out Mbuya Community Initiative, Kampala, Uganda

**Background: **TB remains a leading cause of global mortality, especially in an endemic country like Uganda. While the use of anti‐TBs has been key in reducing TB‐related mortality, LTFU while on TB treatment remains a major obstacle in this fight. It is not only associated with clinical complications but also with drug resistance and the potential spread of resistant strains to communities.

**Methods**: We conducted a retrospective review of TB treatment records at Kawaala Health Centre IV from January 2021 to December 2022. Demographic data, HIV status, diagnosis methods, TB type and presence of phone or treatment support were extracted from unit TB facility registers. Data were entered into an Excel data abstraction tool and analysed using Stata 14. Descriptive analysis was done. We assessed factors associated with LTFU and adjusted for a priori confounders such as age, gender and HIV status to determine independent predictors for TB patient attrition.

**Results**: There were 746 TB entries, more than half, 468 (63.87%) were male, mean age was 31.6 years (SD±13.62), 66% were pulmonary bacteriologically confirmed (PBCs), treatment success rate (TSR) was (656/741) 88.5%. There was 78% reduction of LTFU among the newly diagnosed clients compared to those who had been restarted on anti‐TBs after being LTFU (aOR 0.22, 95% CI 0.08–0.60, *p* = 0.001) and association with facility referrals compared to community referrals (aOR 2.86, 95% CI 0.63–5.08, *p* = 0.005), as per Table 1. No association with age, sex, HIV status, distance from the health facility or presence of a treatment supporter.

**Conclusions**: There is a need to customize interventions for previously LTFU patients being reinitiated on anti‐TBs to mitigate the recurrence of LFTU outcome and the high mortality rates observed in TB cases.

**P162: Table 1:** Factors associated with lost to follow‐up among TB patients—multivariate regression model

	Categories	Adjusted odds ratios	*p*‐value
Age in years	0−10 years	1	
	11−20 years	1.78 (0.43−7.4)	0.429
	21−30 years	1.07 (0.41−2.82)	0.886
	31−40 years	0.99 (0.38−2.59)	0.987
	Greater than or equal to 40 years	1.70 (0.58−4.96)	0.333
Gender	Female	1	
	Male	0.77 (0.43−1.40)	0.390
HIV status	HIV negative	1	
	Person with >3 months HIV diagnosis at anti‐TB initiation	0.91 (0.48−1.72)	0.770
	Person with <3 months HIV diagnosis at the time of anti‐TB initiation	0.84 (0.42−1.73)	0.647
Referral	Community referral	1	
	Facility referral	2.86 (0.63−5.08)	0.012
Presence of at least one phone number		1	
Presence of no phone number		2.97 (0.97−9.02)	0.044

### P163 Global survey to evaluate engagement in care and treatment experiences of people with HIV

Xavier Guillaume^1^, Robin Barkins^2^, Marcel Dams^3^, Nomfundo Eland^4^, Maureen Owino^5^, Carlos Saucedo^6^, Yun‐Chung Lu^7^, Amina Omri^1^, Alissar Moussallem^1^, Larkin Callaghan^8^, Michael Bogart^8^, Connie Kim^8^, Megan Dunbar ^8^

^1^Oracle Life Sciences, Paris, France. ^2^To Restore, Unite, Support, and Transform, Los Angeles, CA, USA. ^3^Aidshilfe NRW e.V, Cologne, Germany. ^4^Emthonjeni Counselling & Training, Cape Town, South Africa. ^5^York University, Toronto, Canada. ^6^Agenda LGBT A.C., Mexico City, Mexico. ^7^Harmony Home & We As One Association, Taipei, Taiwan. ^8^Gilead Sciences, Inc., Foster City, CA, USA

Understanding the experiences of people with HIV (PWH) in the current era is vital to enhance engagement in care to achieve long‐term treatment success. A 45‐minute, cross‐sectional, online survey was co‐developed by investigators and community advocates representing survey countries (Canada, France, Germany, Italy, Japan, Mexico, South Africa, Spain, Taiwan, United Kingdom and United States) and translated into local languages. Survey questions assessed the experiences of PWH across the HIV care cascade. Participants (target *N* = 2500) were ≥18 years old with a self‐reported diagnosis of HIV, recruited through patient databases, consumer panels, patient advocacy groups and physician's referral, to ensure diversity and inclusion of communities most impacted by HIV. Preliminary descriptive analyses reported here include HIV treatment experience of participants who completed the survey in English across Canada, South Africa, the United Kingdom and the United States (*n* = 347); the survey is ongoing. Demographics and treatment characteristics are shown in Table 1. Of 338 participants who had started antiretroviral therapy (ART), similar proportions initiated treatment within 7 days of diagnosis (*n* = 102/338, 30%), 8–30 days from diagnosis (*n* = 105/338, 31%) and >30 days from diagnosis (*n* = 131/338, 39%). Common reasons for starting treatment >30 days after diagnosis included: physician recommendation based on CD4 count (*n* = 45/131, 34%), fear of potential side effects (*n* = 42/131, 32%) and time needed to accept diagnosis (*n* = 37/131, 28%). Most participants did not report adherence challenges with oral (*n* = 265/315, 84%) or injectable (*n* = 11/13, 85%) ART. When asked which treatment features would be most important for staying on treatment long term and for switching, participants identified the same top three reasons: achieving/maintaining undetectable viral load, reducing side effects and long‐term safety/effectiveness; however, these reasons differed in order of importance (Figure 1). The overall median 10‐item HIV Treatment Satisfaction Questionnaire status version score was 54/60 (*n* = 328); for daily oral ART, median scores were highest among those on bictegravir/emtricitabine/tenofovir alafenamide, 57/60 (*n* = 51). Results showed that 39% of participants delayed initiating treatment, highlighting opportunities to support/improve rapid initiation. Participants receiving ART reported high levels of adherence and satisfaction, with effectiveness, side effects and long‐term safety being top considerations for both switching and remaining on treatment long term.

**P163: Table 1:** Participant demographics and treatment characteristics

Demographic/characteristic	Participants (*N* = 347)
Gender, *n* (%)	
Male	202 (58)
Female	139 (40)
Other gender category	3 (0.9)
Nonbinary or gender nonconforming	2 (0.6)
Prefer not to answer	1 (0.3)
Age, mean (SD), year	43.0 (13)
Country, *n* (%)	
United States	154 (44)
South Africa	109 (31)
United Kingdom	58 (17)
Canada	26 (7.5)
Participant sub‐population, *n* (%)	
MSM	143 (41)
Cis women	136 (39)
Older people with HIV (≥50 years)	111 (32)
People who use drugs	89 (26)
People with HIV diagnosed since the COVID‐19 pandemic	72 (21)
Un/underinsured	72 (21)
BIPOC MSM	53 (15)
Migrants	24 (6.9)
Trans or nonbinary people	10 (2.9)
Young adult people with HIV (18−24 years)	9 (2.6)
Time since initiation of ART, mean (SD) [median], year^a^	12.5 (9.6) [9.0]
Currently treated, *n* (%)	328 (95)
Treated with 1 oral daily pill, *n* (%)^b^	248 (76)

Abbreviations: ART, antiretroviral therapy; BIPOC, Black, Indigenous and other people of colour; MSM, men who have sex with men; SD, standard deviation.

^a^ *N* = 338.

^b^ *N* = 328.

**P163: Figure 1:**
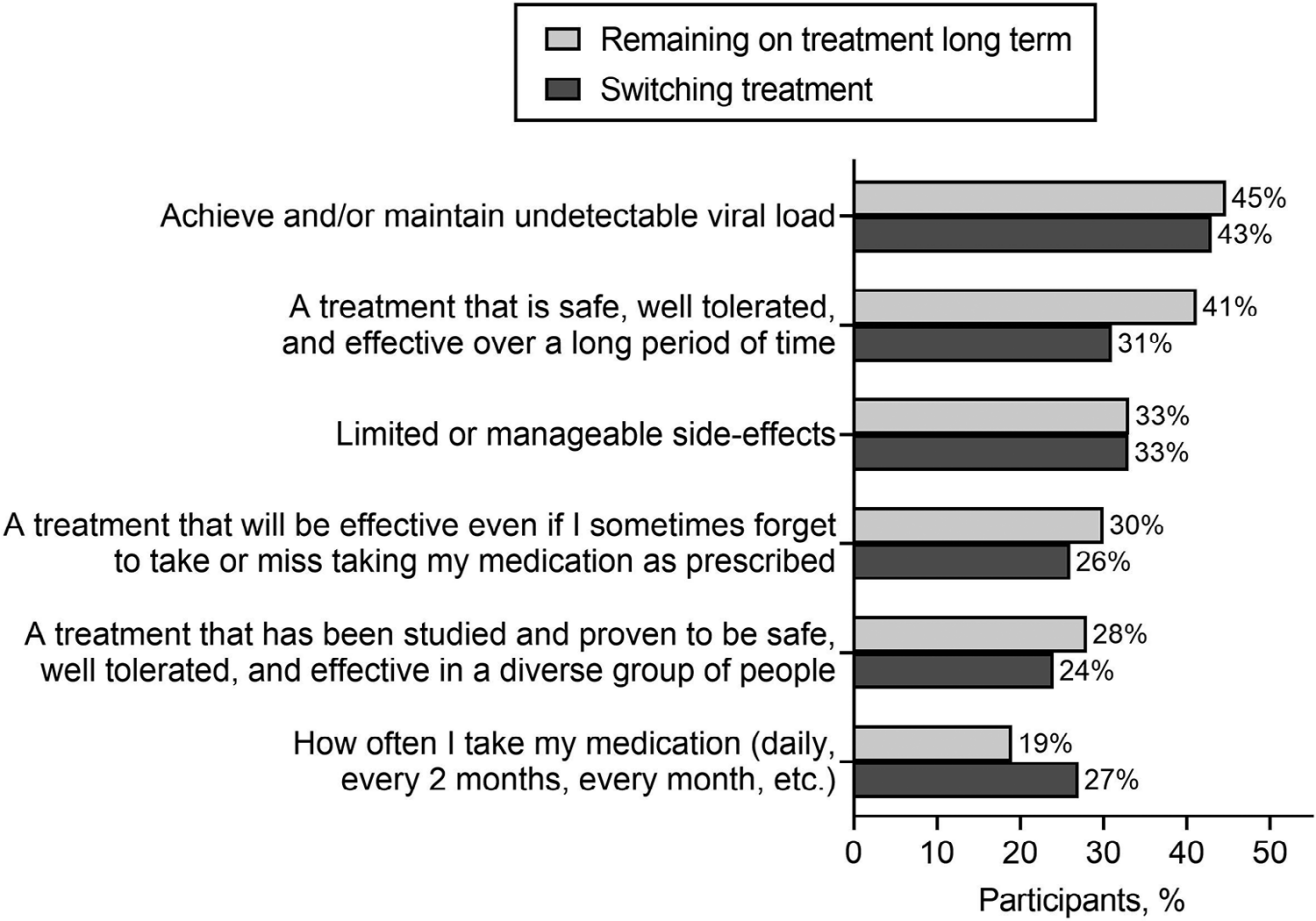
Participants were asked to rank their top three treatment features, among 13 possible responses, that would be most important in remaining on HIV treatment over the long term and their top three reasons that would be most important in switching HIV treatment. Treatment features selected by >25% of participants are shown.

### P164 Exploring the minimal proportion of days covered (PDC) threshold required for achieving 90% viral suppression in people with HIV in the era of single‐tablet regimens

Chun‐Yuan Lee, Tien‐Heng Ku, Yi‐Pei Lin, Chia‐Yu Tsai, Po‐Liang Lu

Department of Internal Medicine, Kaohsiung Medical University Hospital, Kaohsiung City, Taiwan

**Background**: The required adherence to the multiple‐tablet regimen is high. We investigate the minimal proportion of days covered (PDC) threshold needed to achieve 90% viral suppression (VS) (<200 copies/ml) in people with HIV (PWH) treated with single‐tablet regimens (STRs). We also examine the predictive power of this PDC threshold for VS and identify factors relevant to the PDC threshold.

**Materials and methods**: This cross‐sectional study was conducted at a Taiwan medical centre from December 2023 to July 2024. We included PWH who had been on HAART >365 days and had complete antiretroviral medication dispensing records for the preceding 365 days. We defined PDC as the proportion of days within the past 365 days in which HAART was dispensed. Data of HIV plasma viral load (PVL) was the nearest PVL within ±90 days of the questionnaire date. We defined VS as PVL <200 copies/ml. We first identified the PDC threshold for achieving 90% VS (PDC categories: <40%, 40–50%, 50–60%, 60–70%, 70–80%, 80–90%, >90%). We then used binary logistic regression to explore the association of the PDC threshold with failing to achieve VS and finally identify factors relevant to failing to reach this PDC threshold.

**Results**: We included 515 participants (83.69% MSM; 69.71% aged 30–50; 97.28% male; 74.79% diagnosed with HIV for over 5 years). The PDC distribution frequency was as follows: <40% (2.34%), 40–50% (0.97%), 50–60% (1.36%), 60–70% (0.78%), 70–80% (3.12%), 80–90% (6.82%) and >90% (84.60%). We first identified 60% as the PDC threshold for achieving 90% VS (Figure 1). The PDC <60% (vs. ≥60%) was a substantial factor associated with failing to achieve VS (AOR 20.31, 95% CI 7.76–52.51, *p* < 0.001). We finally identified having a sexually transmitted infection in the past 3 months (AOR 3.55, 95% CI 1.25–10.10, *p* = 0.02) and being aged 30–50 years (vs. ≤30 years) (AOR 0.25, 95% CI 0.09–0.67, *p* = 0.006) as factors associated with failing to reach this PDC threshold.

**Conclusions**: Although the PDC threshold (60%) for achieving VS is lower in the era of STR than before, it is crucial to note that a significant proportion of patients, 4.67%, still fall below this threshold, profoundly impacting their treatment outcomes.

**P164: Figure 1:**
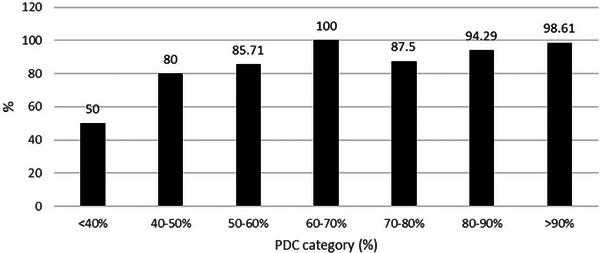
Percentage of viral suppression across PDC categories.

### P165 Physician attitudes towards anti‐retroviral therapy adherence and unmet treatment needs in people with HIV: a real‐world survey in Europe and the United States

Rebecca Charlton, Will Ambler, Mark Small, Tim Holbrook, Fritha Hennessy

Real‐World Evidence, Adelphi Real World, Bollington, UK

**Background**: Although previous studies have identified factors affecting adherence to anti‐retroviral therapy (ART) in people with HIV (PWH), there is a paucity of data surrounding physicians’ attitudes towards ART and factors affecting equitable outcomes. We aimed to describe physicians’ perceptions of treatment adherence and unmet treatment needs in PWH prescribed ART.

**Materials and methods**: Data were drawn from the Adelphi Real World HIV Disease Specific Programme™, a cross‐sectional survey of physicians in France, Germany, Italy, Spain, the United Kingdom (UK) and the United States (US) from June 2021 to June 2023. Physicians completed an attitudinal survey reporting perceptions on ART adherence, treatment challenges and areas for treatment improvement. Analyses were descriptive.

**Results**: Overall, 270 physicians (France *n* = 49, Germany *n* = 50, Italy *n* = 40, Spain *n* = 42, UK *n* = 29, US *n* = 60) reported data. A total of 85% physicians tended to agree, or strongly agreed with the statement “poor adherence is the main driver of multi‐drug resistant HIV strain development.” Stigma was a key barrier to adherence among high‐risk individuals (58% of physicians tended to or strongly agreed), and ART pill burden was reported as a problem (58%), with 51% of physicians tending to, or strongly agreeing that younger PWH were less worried about skipping daily doses. Three‐quarters of physicians tended to agree, or strongly agreed, that social, psychological and treatment‐related challenges were greater for older individuals, and that novel ARTs are needed for the ageing population who are prescribed other treatments for concomitant conditions (70% of cases). Physicians tended to agree, or strongly agreed, that for all PWH, long‐acting therapeutics could improve adherence (81% of physicians) and address challenges including pill burden, stigma and drug/food interactions (76% of physicians).

**Conclusions**: Physicians highlighted stigma and pill burden as factors affecting treatment adherence, and identified ageing PWH as having particular unmet needs. These data highlight the need for improved treatment regimens to ensure equitable outcomes for all PWH. Increased adoption of longer‐acting therapeutics, that can be taken in combination with other medications, may help address this.

### P166 Exploring latent adherence profiles in South African people living with HIV using group‐based trajectory modelling

Campbell McDuling ^1^, Lauren Jennings^1^, Christopher Ferraris^2^, Paul D'Avanzo^2^, Robert H. Remien^2^, Catherine Orrell^1^, Francesca Little^3^

^1^Centre for Adherence and Therapeutics, Desmond Tutu Health Foundation, Cape Town, South Africa. ^2^HIV Center for Clinical and Behavioral Studies, Psychiatric Institute, Columbia University, New York, NY, USA. ^3^Department of Statistics, University of Cape Town, Cape Town, South Africa

**Background**: Monitoring adherence to antiretroviral therapy (ART) is critical in the management of the HIV/AIDS epidemic, particularly in high‐burden settings like South Africa. Group‐based trajectory models (GBTMs) offer a promising approach to identify latent classes of adherence behaviour from longitudinal data.

**Materials and methods**: We applied GBTM to 12 months of electronic monitoring (EM) device data from a 24‐month prospective observational study (ADD‐ART) of 250 virally suppressed, people living with HIV in the Western Cape region of South Africa. We then applied a Kaplan‐Meier survival analysis on the sample, after stratifying by adherence profile.

**Results**: The GBTM analysis revealed five distinct adherence trajectories, as illustrated in Figure 1: (1) stable and excellent adherence (19% of participants); (2) stable and acceptable adherence (26%); (3) stable and poor adherence (17%); (4) slowly deteriorating adherence (18%); and (5) rapidly deteriorating adherence (20%).

These subgroups exhibited markedly different viral suppression outcomes, with the two deteriorating adherence groups exhibiting a much faster decline in the probability of viral suppression over time.

**Conclusions**: This analysis approach provides a valuable tool for identifying heterogeneous patterns of longitudinal ART adherence that have important implications for clinical management and viral outcomes. This approach could be extended to other adherence monitoring methods and settings to better target adherence interventions.

**P166: Figure 1:**
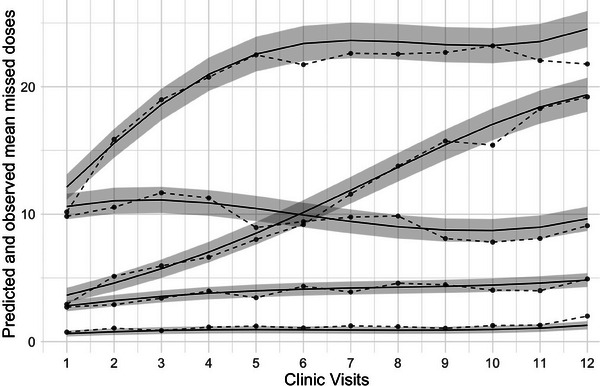
Observed and predicted mean number of EM missed doses for each latent adherence group.

### P167 Effectiveness of mobile health technology in improving HIV care continuity and service delivery among sex workers in Sex Workers Outreach Project (SWOP) clinics, Kenya

Idah Mirera, Maureen Akolo

Department of Health, Partners for Health and Development in Africa, Nairobi, Kenya

**Background**: The delivery and results of healthcare can be enhanced by using mobile health technology, or m‐health, which is the provision of health services and information via mobile phones through phone calls or messaging by addressing problems like accessibility to medical facilities, particularly in remote locations, Kenya's healthcare system's digitization holds the potential role to improve health outcomes. This m‐health innovation not only increases access to healthcare but also eases traffic and improves system performance overall. This study's primary goal was to evaluate the effectiveness of mobile health interventions in enhancing HIV continuity.

**Materials and methods**: Weekly appointments were conducted using a longitudinal data gathering strategy by making pre‐visit calls, such as those for the seven, three, one and actual dates of the anticipated visit, tracking forms and putting in place a case management team to supervise the appointment scheduling process were how this was accomplished.

**Results**: Before the introduction of m‐health appointment keeping was around 54% and a viral load uptake of 75%, but after the implementation of m‐health, appointment keeping improved by more than 86% and viral load uptake at 89%, enhancing health efficiency. Care clients were also free to disclose to the health provider any health issues that might have prevented them from visiting the facility, such as being preoccupied at work, and receive assistance with medication at their convenience without having to visit the facility. These clients were also able to disclose obstacles to keeping appointments, and they would reschedule the appointment visit dates appropriately with the health providers improving HIV retention. Reminders about viral load sample collection led to a rise in viral load uptake and an increased appointment‐keeping rate, and the use of mobile health technology in conjunction with other retention strategies produced a noticeable improvement in HIV/care continuity and treatment outcomes in all SWOP clinics in Kenya.

**Conclusions**: To enhance the quality of care provided, it is necessary to increase the capacity of the health workers and provide clients with accurate health information on the need to provide accurate phone numbers for effective service delivery.

### P168 Health‐related quality of life among the people living with HIV in Armenia

Tatevik Balayan

Epidemiology, National Center for Disease Control and Prevention in Armenia, Yerevan, Armenia

**Background**: The availability of ART has increased the average life expectancy of PLWHIV and given the longevity achievable with ART preserving quality of life of these people has become a focus of interventions and programmes. This is the first study evaluating HRQoL among PLWHIV in Armenia. We aimed to identify modifiable risk factors for poor HRQoL in Armenia. These results are intended to inform the design and potential effectiveness of HRQoL interventions. The study was conducted in 2018. The institutional review boards at the Harvard T. H. Chan School of Public Health (18‐1378) and the American University of Armenia (2017‐007) approved the study.

**Methods**: Study design: Cross‐sectional survey with self‐administered questionnaire. Target population: PLWHIV ≥18 receiving ART. Setting: NGO Positive People Armenian Network (PPAN), *N* = 180. Convenience non‐probability sampling. The questionnaire consisted of seven sections: (1) Socio‐demographic data; (2) Care provided at the HIV Clinic; (3) Knowledge on HIV/AIDS and treatment; (4) Adherence to ART (Morisky Medication Adherence Scale, MMAS); (5) Existence of side effects (AIDS Clinical Trials Group, ACTG); (6) Social support (Medical Outcomes Study Social Support Scale, MOS‐SS); and (7) Health‐related quality of life (36‐Item Short Form Health Survey, SF‐36). Descriptive statistics. General linear regression models. Stepwise variable selection with an entry *p*‐value of 0.20 and retained variables with a *p*‐value <0.20. All *p*‐values were two‐sided with a *p*‐value  < 0.05 considered statistically significant. Statistical analyses were performed using SPSS 16.

**Results**: The highest HRQoL domain score was 85.3 (SD 24.7) for physical functioning, followed by 82.1 (SD 25.0) for pain, 77.9 (SD 24.2) for social functioning, 76.4 (SD 39.6) for emotional role‐functioning, 71.1 (SD 39.7) for physical role‐functioning, and 64.0 (SD 20.3) for energy/fatigue, 63.7 (SD 22.7) for emotional wellbeing and 63.4 (SD21.2) for general health. In the physical domain, chronic comorbidities and low emotional support were associated with worse physical functioning, physical role‐functioning, general health and pain scores (*p* < 0.05). Unemployment and hepatitis C coinfection were associated with worse physical role‐functioning and pain scores (*p* < 0.01). As for mental HRQoL, we found that unemployment, chronic comorbidities and lower emotional support were associated with poorer emotional wellbeing, energy and emotional role‐functioning scores (*p* < 0.05).

**Conclusions**: These findings suggest that improved social support employment opportunities, mental health services and integrated care for non‐communicable comorbidities may improve HRQoL in Armenia and similar settings.

### P169 Determinants of treatment interruptions in tuberculosis patients at Reach Out Mbuya: insights from a 3‐year retrospective study

Damalie Nakyomu ^1^, Patrick Kazooba^2^, Gertrude Namale^1^, Emmanuel Sendaula^3^, Josephine Kaleebi^4^

^1^Medical, Reach Out Mbuya Community Health Initiative, Kampala, Uganda. ^2^Research & Innovation, Reach Out Mbuya Community Health Initiative, Kampala, Uganda. ^3^Monitoring & Evaluation, Reach Out Mbuya Community Health Initiative, Kampala, Uganda. ^4^Programmes, Reach Out Mbuya Community Health Initiative, Kampala, Uganda

**Background**: Tuberculosis (TB) remains critical globally. Uganda struggles with TB control and interruptions in treatment undermine management, risking drug‐resistant TB, increased morbidity, mortality and disease transmission. This study identified predictors of treatment interruptions among Reach Out Mbuya (ROM) patients from January 2020 to January 2023.

**Methods**: This retrospective study reviewed medical records of TB patients treated at Reach Out Mbuya (ROM) Clinic between January 2020 and January 2023. Data on demographics, clinical parameters, TB diagnosis, treatment initiation and completion dates, HIV status, diagnostic methods, TB classification, and referrals were extracted and recorded in an Excel database and analysed in Stata. TB treatment interruption was defined as exceeding 168 days (about 6 months) between treatment start and completion. We determined crude associations with treatment interruption and used multivariable logistic regression to determine independent predictors of interruptions.

**Results**: A total of 268 TB patients’ records were reviewed, with 55% male. Median age was 35.5 years (IQR 29–45), mean weight 53.75 kg (SD 15.27) and mean mid‐upper arm circumference (MUAC) 25 cm (SD 9.7). Most were new TB cases (88.81%), mainly pulmonary bacteriologically confirmed TB (61.57%) diagnosed by lipoarabinomannan assay (LAM) (35.82%). The cure rate was approximately 57.30%. Treatment interruptions affected 51.87% of patients. All deaths (13) and lost follow‐ups (four) had interruptions. Logistic regression found higher odds in age >50 years (aOR 3.73, 95% CI 1.20–11.66, *p* = 0.017) and pulmonary bacteriologically confirmed (PBC) TB (aOR 10.19, 95% CI 1.17–88.36, *p* = 0.035). Relapsed TB had lower odds (aOR 0.27, 95% CI 0.10–0.70, *p* = 0.007) than new cases.

**Conclusions**: Treatment interruptions significantly impact TB management at our facility, with over half of patients affected. Older age and pulmonary bacteriologically confirmed TB were major risk factors for interruptions, while relapsed TB cases had a reduced risk compared to new TB cases. Targeted interventions are needed to improve adherence, especially for high‐risk groups, to enhance treatment outcomes and control TB in resource‐limited settings like Uganda.

## Clinical management considerations—Women

### P170 HIV infection in migrant women from sub‐Saharan Africa in North East of Italy: a snapshot of a vulnerable population

Anna Ferrari^1^, Lolita Sasset ^1^, Marco Cola^1^, Claudia Cozzolino^2^, Maria Cristina Rossi^3^, Fabio Rigo^4^, Veronica Del Punta^5^, Angela Londero^6^, Massimiliano Lanzafame^7^, Sandro Panese^8^, Marina Malena^9^, Vinicio Manfrin^4^, Annamaria Cattelan^1^

^1^Tropical and Infectious Diseases Clinic, Padua University Hospital, Padua, Italy. ^2^Department of Cardiac, Thoracic, Vascular Sciences, and Public Health, University of Padua, Padua, Italy. ^3^Infectious Diseases Unit, Treviso Hospital, Treviso, Italy. ^4^Infectious Diseases Unit, Vicenza Hospital, Vicenza, Italy. ^5^Infectious Diseases Outpatient Clinic, Azienda Ulss 9 Scaligera (AULSS9), Verona, Italy. ^6^Infectious Diseases Unit, Azienda Sanitaria Universitaria Friuli Centrale, Udine, Italy. ^7^Infectious Diseases Unit, Trento Hospital, Trento, Italy. ^8^Infectious Diseases Unit, Mestre‐Venice Hospital, Venice, Italy. ^9^Infectious Diseases Unit, Rovigo Hospital, Rovigo, Italy

**Background**: In 2023, over 40% of new HIV diagnoses in the EU/EEA were among migrants, with 58% of sub‐Saharan Africa (SSA) migrants diagnosed late (CD4^+^ less than 350 cells/ml). In our region, there is a large presence of SSA immigrants, with a majority being women. However, available data regarding this specific population are scarce.

**Methods**: A multicentre cross‐sectional observational study was conducted to describe the demographic, social, economic, viral, immunological characteristics, co‐infections and comorbidities of women living with HIV (WLWH) from SSA who were receiving care at 10 infectious diseases (ID) centres in the North‐East of Italy during 2020.

**Results**: We included 454 WLWH, coming from Nigeria (212), Ghana (64), Ivory Coast (41), Cameroon (38) and from 30 other SSA states. Demographics, clinical and social characteristics are reported in Table 1. The median age at diagnosis was 29 years old [IQR 25–34.5]; 33 (7.3%) were diagnosed with HIV before arriving in Italy. Most reported infection through heterosexual contact (86.3%). At diagnosis, median CD4^+^ cell count was 244 cells/ml [IQR 133.2–372.2]; late diagnosis was recorded in 277 (71%) women. Four hundred and twenty‐eight (99.1%) women were receiving antiretroviral therapy (ART); 78.9% and 15.7% of them were on triple therapy (34% INI, 16.8% PI, 28.3% NNRTI), and on dual therapy (DTG+RPV, XTC+DTG or XTC+PI/b), respectively. Two hundred and thirty‐six women (78.2%) were virologically suppressed, 27 (9%) had low‐level viraemia (<200 copies/µl) and 38 (12.6%) had virological failure/viral blip. No significant associations were identified between virological failure and social, clinical or viro‐immunological variables.

**P170: Table 1:** Characteristics, co‐infections and comorbidities of migrant WLWH in care at our ID centres

Age at HIV diagnosis	Med [IQR]	29 [25−34.5]
Education, *n* (%)	Yes	240 (52.9)
	No	34 (7.5)
	Unknown	180 (39.6)
Bureaucratic civil status, *n* (%)	Italian citizenship	19 (4.2)
	Long‐term residence permit	231 (50.9)
	Short‐term residence permit	27 (5.9)
	Refugee	8 (1.8)
	Undocumented	16 (3.5)
	Unknown	153 (33.7)
Marital status, *n* (%)	Pluripartner	7 (1.5)
	Stable relationship	224 (49.3)
	Single	71 (15.6)
	Unknown	152 (33.5)
Employment, *n* (%)	Occasionally employed	35 (7.7)
	Fully employed	104 (22.9)
	Unemployed	76 (16.7)
	Unknown	239 (52.6)
HIV risk factor, *n* (%)	Heterosexual contact	392 (86.3)
	Mother to child transmission	3 (0.7)
	Blood transfusion	3 (0.7)
	Intravenous drug use	1 (0.2)
	Unknown	55 (12.1)
Time of diagnosis, *n* (%)	Before arrival in Italy	33 (7.3)
CD4 nadir, cells/mmc	Med [IQR]	244 [133.2−372.2]
CD4 nadir, cells/mmc	0−199	156 (40)
	200−349	121 (31)
	350−499	73 (18.7)
	500	40 (10.3)
HCV coinfection	Yes	7 (1.5)
HBV coinfection	HbcAb isolated	54 (11.9)
	HbsAg positive	29 (6.4)
	Natural immunity	136 (30)
	Negative	203 (44.7)
	Unknown	32 (7)
Mycobacterium tuberculosis infection, *n* (%)	Latent	24 (5.3)
	Active, treated	30 (6.6)
	Negative	237 (52.2)
	Unknown	163 (35.9)
Syphilis, *n* (%)	Yes	20 (4.4)
Diabetes, *n* (%)	Yes	49 (10.8)
Hypertension, *n* (%)	Yes	142 (31.3)
Dyslipidaemia, *n* (%)	Yes	101 (22.2)
Oncological diseases, *n* (%)	Yes	36 (7.9)

**Conclusions**: Over 99% of SSA WLWH in care at our ID centres received ART, but only 78.2% were virologically suppressed. Unstable socio‐economic conditions, language barriers and lack of education likely contributed to poor ART adherence. In addition, a significant number of women were diagnosed with HIV after arriving in Italy, underscoring the need for targeted HIV prevention and testing programmes to prevent late HIV diagnoses, which are significantly higher among migrant women in our cohort compared to those in other European countries.

### P171 Women living with HIV: how far to fulfil the gap?

Adriana Cervo ^1^, Mariachiara Pellegrino^2^, Federica Casari^2^, Cinzia Puzzolante^1^, Giovanni Guaraldi^2^, Cristina Mussini^2^

^1^Infectious Diseases Unit, University Hospital Policlinico of Modena, Modena, Italy. ^2^Infectious Diseases Unit, University of Modena and Reggio Emilia, Modena, Italy

**Background**: The aim of this study was to define the clinical profile of women living with HIV (WLWH) to find potential gaps in care and to implement strategies for retention and improving quality of life.

**Materials and methods**: Retrospective study including cisgender WLWH attending HIV Clinic in Modena since 1996. Active follow‐up defined as presence of at least one visit after 01/01/2023. Demographic, metabolic, HIV‐related and ART‐related characteristics compared according to ethnicity in WLWH in active follow‐up.

**Results**: Nine‐hundred and sixty‐four women had at least one access in HIV Clinic: 67.6% Caucasian, 28% African, 1% Asiatic and 3% Central/South‐American. Among those, 29.5% were lost to follow‐up (40.5% and 55% of them were African and Caucasian, respectively), 5.1% moved to other centre, 12.2% deceased. Among 491 women in active follow‐up, 368 (72%) and 123 (24%) were Caucasian and African, respectively, as shown in Table 1. Caucasian WLWH were more frequently smokers and dyslipidaemic (*p* < 0.01). African women were more frequently obese and diabetic (*p* < 0.01); they experienced more frequently virological failure (defined as HIV RNA >200 copies/ml on ART and/or no visit for more than 18 months) (*p* = 0.021) and had lower CD4 cell count (*p* = 0.05) and CD4/CD8 ratio (*p* = 0.001) at the last follow‐up. Regarding current ART (Figure 1), African WLWH were more frequently on three‐drug regimen (3DR) (*p* < 0.001), often TAF‐based (*p* < 0.001); main use of NNRTI/TXF/FTC in single‐tablet regimen was prevalent in African women than Caucasian ones (50% vs. 38%) (*p* = 0.012), while second‐generation INSTIs were less used (*p* < 0.01). Among 221 women on dual therapy (45%), 22 (10%) were African; only nine were in long‐acting regimen, mainly Caucasian.

**P171: Table 1:** Characteristics of African women living with HIV in Modena compared to Caucasian women

	Total (*n* = 491)	Caucasian (*n* = 368)	African (*n* = 123)	*p*‐value
Age at HIV diagnosis (years), median (IQR)	29.9 (18.5−53.2)	28.8 (18.2−54.7)	31.2 (18.7−48.4)	0.05
Age at last FU (years), median (IQR)	55 (34−70)	57 (37−71)	46 (30−59)	0.000
Risk factors, *n* (%)				0.000
Sex	347 (70.7)	237 (64.4)	110 (89.4)	
Vertical	4 (0.8)	4 (1.1)	0 (0)	
IVDU	104 (21.2)	103 (28.0)	1 (0.8)	
Transfusions	9 (1.8)	6 (1.6)	3 (2.4)	
Unknown	27 (5.5)	18 (4.9)	9 (7.3)	
AIDS, *n* (%)	83 (16.9)	67 (18.2)	16 (13.0)	0.115
HCV, *n* (%)	141 (28.7)	139 (37.8)	2 (1.6)	0.000
HbsAg, *n* (%)	22 (4.5)	9 (2.4)	13 (10.6)	0.001
Viro‐immunological characteristics				
CD4 cell count at diagnosis (cells/mmc), median (IQR)	427 (42−1167)	458 (39−1232)	326 (42−715)	0.000
CD4/CD8 ratio at diagnosis, median (IQR)	0.53 (0.08−1.30)	0.58 (0.11−1.95)	0.39 (0.05−0.98)	0.000
HIV RNA >50 copies/ml at last FU, *n* (%)	0.53 (0.08−1.30)	16 (4.3)	11 (8.9)	0.044
Number of VF, median (IQR)	0 (0−3)	0 (0−2)	0 (0−4)	0.021
Last value of CD4 (cells/mmc), median (IQR)	773 (282−1433)	789 (288−1512)	702.9 (251.5−1290)	0.046
Last ratio CD4/CD8, median (IQR)	1.09 (0.30−2.30)	1.13 (0.36−2.37)	0.94 (0.19−2.26)	0.001
Antiretroviral regimen at last follow up				
Three drug‐based regimen, *n* (%)	270 (55)	171 (46.5)	99 (80.5)	0.000
Two drug‐based regimen, *n* (%)	221 (45)	199 (54)	22 (17.9)	0.000
PI, *n* (%)	52 (10.6)	38 (10.3)	14 (11.4)	0.439
INSTI, *n* (%)				0.000
RAL	18 (3.7)	12 (3.3)	6 (4.9)	
DTG/BIC/CAB	319 (65.0)	262 (71.2)	57 (46.3)	
NNRTI, *n* (%)				0.113
RPV/NEV/EFV	178 (36.3)	124 (33.7)	54 (43.9)	
DOR	24 (4.9)	18 (4.9)	6 (4.9)	
TAF‐based regimen, *n* (%)	220 (44.8)	142 (38.6)	78 (63.4)	0.000
Biochemistry at last follow‐up visit				
Hb (g/dl), median (IQR)	13.6 (11.1−15.3)	13.8 (11.6−15.3)	12.7 (10.7−14.6)	0.000
LDL cholesterol >130 mg/dl, *n* (%)	108 (22.0)	88 (23.9)	20 (16.3)	0.130
Triglycerides >150 mg/dl, *n* (%)	57 (11.6)	52 (14.1)	5 (4.1)	0.002
Comorbidities				
Smoking, *n* (%)	154 (31.4)	147 (39.9)	7 (5.7)	0.000
Hypertension, *n* (%)	179 (36.5)	130 (35.3)	49 (39.8)	0.174
Diabetes, *n* (%)	45 (9.2)	26 (7.1)	19 (15.4)	0.005
Dyslipidaemia, *n* (%)	310 (63.1)	250 (67.9)	60 (48.8)	0.000
Cardiovascular disease, *n* (%)	22 (4.5)	16 (4.3)	6 (4.9)	0.471
eGFR <50 ml/minute, *n* (%)	36 (7.3)	26 (7.1)	10 (8.1)	0.396
BMI >30, *n* (%)	81 (16.5)	45 (12.2)	36 (29.3)	0.000
Osteoporosis^a^, *n* (%)	98 (20.0)	90 (24.5)	8 (6.5)	0.015
Menopause^b^, *n* (%)	214 (43.6	196 (53.2)	18 (14.6)	0.000
Cancer^c^, *n* (%)	108 (22.2)	92 (25.3)	16 (13.0)	0.005
Gynaecological	53 (49.1)	45 (48.9)	8 (50)	
Breast	13 (12.0)	11 (12.0)	2 (12.5)	
Anal	10 (9.3)	6 (6.5)	4 (25)	
Skin	10 (9.3)	9 (9.8)	1 (6.3)	
Haematological	7 (6.5)	6 (6.5)	1 (6.3)	
Other cancer	15 (13.9)	15 (16.3)	0 (0.0)	

Abbreviations: ART, antiretroviral therapy; BIC, bictegravir; BMI, body mass index; CAB, cabotegravir; DOR, doravirine; DTG, dolutegravir; eGFR, estimated glomerular filtration rate; FU, follow‐up; HCV, hepatitis C virus; INSTI, integrase strand transfer inhibitor; IVDU, intravenous drug user; NNRTI, non‐nucleoside reverse transcriptase inhibitor; PI, protease inhibitor; RAL, raltegravir; TAF, tenofovir diproxil alafenamide; VF, virological failure.

^a^Data on osteoporosis were available for 237 and 41 Caucasian and African women, respectively.

^b^Data on menopause were available for 259 and 58 Caucasian and African women, respectively.

^c^Data on cancer were available for 366 and 117 Caucasian and African women, respectively.

**Conclusions**: African WLWH were generally less adherent, with higher rate of loss to follow‐up and virological failure than Caucasian women. Moreover, they were characterized by higher burden of comorbidities, in particular obesity and diabetes. That led to multiple vicious circles, enhanced by different cultural, social and economic determinants: treatment choice of 3DR versus dual regimen, difficulties in screening and management of comorbidities, lower rate of cancer screening. More efforts are needed to fulfil the gap among African WLWH, adopting different retention‐in‐care, screening and preventive strategies in order to improve their quality of life.

**P171: Figure 1:**
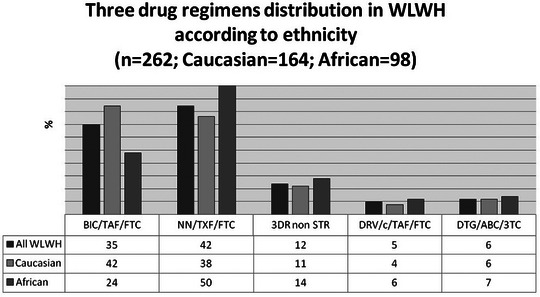
Three‐drug regimen distribution in WLWH according to ethnicity in Modena.

### P172 Postpartum care pathway of women living with HIV in the North of France from 2011 to 2021

Noémie Lemee ^1^, Agnès Meybeck^1^, Roxane Vanspranghels Gibert^2^, Maxime Degrendel^3^, Pauline Coulon^4^, Olivier Robineau^1^, Pauline Thill^5^

^1^Infectious Diseases, Hospital Dron, Tourcoing, France. ^2^Gynecology Obstetrics, University Hospital of Lille, Lille, France. ^3^Health Research Unit, Hospital Dron, Tourcoing, France. ^4^Virology, University Hospital of Lille, Lille, France. ^5^Infectious Diseases, University Hospital of Lille, Lille, France

**Background**: In France, 1500 women living with HIV (WLWH) give birth each year, with a mother‐to‐child transmission (MTCT) rate of 0.2%. French recommendations address obstetric and infectious prenatal care but less the postpartum period, although it is known to be at risk of loss to follow‐up and virological failure. Our main objective was to address the adequacy of the WLWH care pathway to French recommendations during the postpartum period. The secondary objective was to study the follow‐up of WLWH at 1 and 2 years after delivery.

**Materials and methods**: We conducted an observational retrospective study in two hospitals in the North of France. The main criterion was a composite score of adequacy of postpartum medical follow‐up. An adequate care pathway was defined by a consultation with the infectious disease (ID) specialist within 3 months, with the gynaecologist within 2 months and HIV viral load (VL) test within 1 month.

**Results**: A total of 185 WLWH and 258 pregnancies were studied. The mean age was 33 years and 79.8% of the population were of foreign origin. During pregnancy, 99.6% received antiretroviral therapy and 85.3% delivered with undetectable VL. No MTCT was detected in the 265 children. Our score for postpartum care found 9.3% adequacy. Only 26.7% of women saw their gynaecologist within 2 months and 19.8% had an HIV VL test within 1 month but 76.7% saw their ID specialist within 3 months. After 1 year, 5.8% of WLWH stopped their follow‐up with the ID specialist and 34.9% with the obstetrician (Figure 1). After 2 years, only 75% of WLWH maintained viral suppression and patients with an inadequate postpartum score appeared to be at higher risk of loss to follow‐up (22.6% vs. 12.5%, *p* = 0.065). Follow‐up of the children appeared to be better, with 88.4% of them having had three HIV VL tests, all negative.

**Conclusions**: These data showed a severe lack of adherence to the postpartum care pathway. Simplification of the pathway with a joint postpartum consultation with both ID specialist and obstetrician could improve adherence. These data suggest the need for very close management of women who are going to breastfeed.

**P172: Figure 1:**
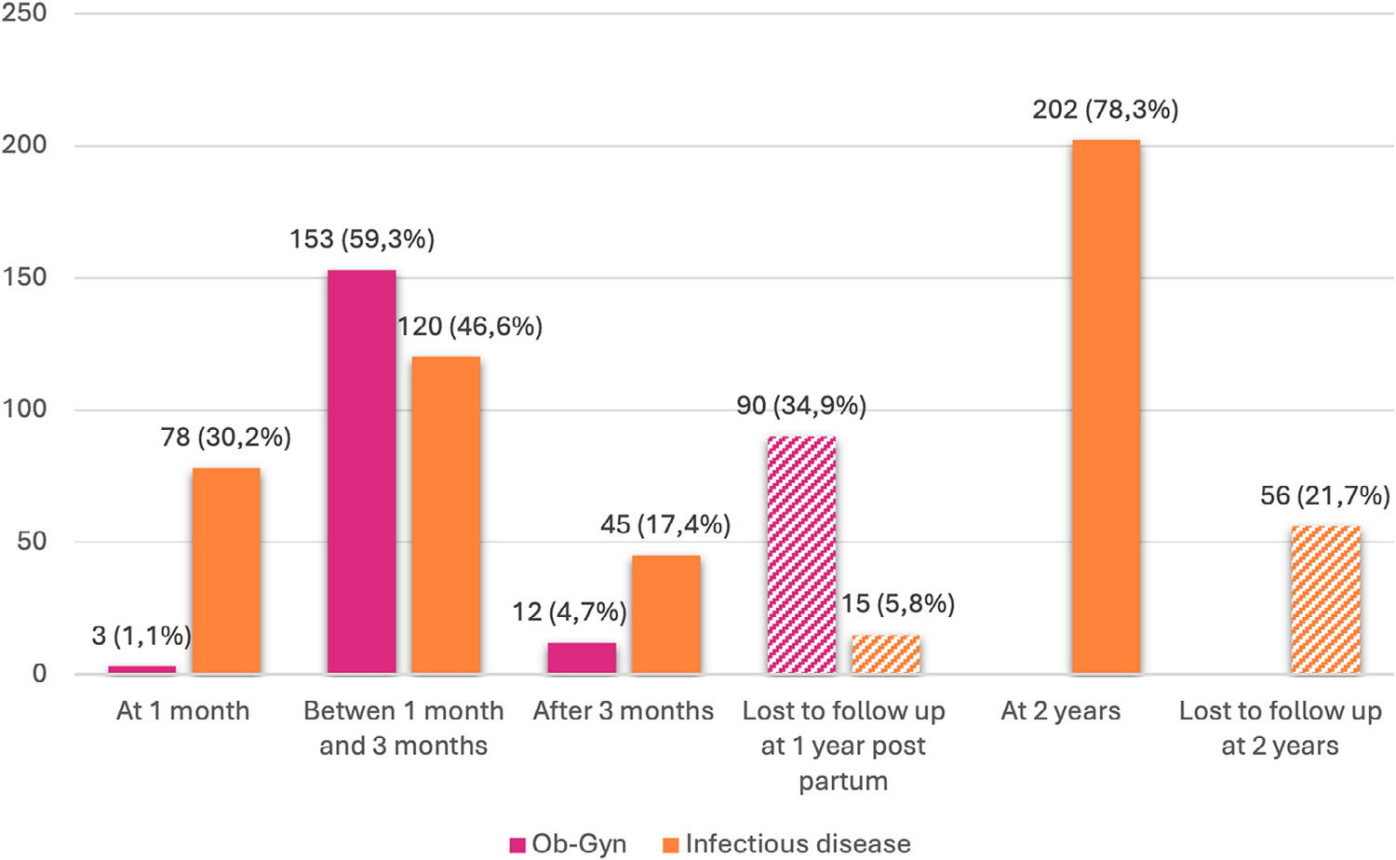
Women living with HIV (WLWH) postpartum follow‐up in Lille and Tourcoing hospitals between 2011 and 2021 (*N* = 258).

### P173 CONception to Child‐Rearing in the Early Treatment Era (CONCRETE): a qualitative study of the experiences of women living with HIV during pregnancy and motherhood

Clara Merlin ^1^, Clara Le Saux^2^, Nelly Courvoisier^2^, Jose Damas^1^, Katharine Darling^1^

^1^Infectious Diseases Service, University Hospital Lausanne, Lausanne, Switzerland. ^2^Social Sciences Department, University Centre of General Medicine and Public Health, Lausanne, Switzerland

**Background**: Women living with HIV (WLWH) represent an increasing proportion of people with HIV, yet their unique needs, especially around childbirth, remain underexplored or unvoiced. This study aimed to document the experience of WLWH who delivered during the early antiretroviral era, when long‐term safety data were unavailable, and to determine how these insights might benefit today's generation of WLWH.

**Methods**: Semi‐structured interviews were conducted with 12 mothers living with HIV in an urban area of French‐speaking Switzerland. The interview guide covered topics including pregnancy and birth plans, health, relationships with health professionals, social support, stigma and motherhood. Content analysis using IRaMuTeQ software was performed to identify key themes in the women's narratives.

**Results**: Mean age of the women at interview was 51.6 years. Eight women were White, four were Black or Hispanic; six had completed education beyond mandatory schooling; two reported former injecting drug use. Eight women had delivered between 2001 and 2004 and four between 2005 and 2008, with an average of 9.2 years between HIV diagnosis and delivery. Concerning the narratives, a prominent theme was the challenge of discussing HIV status with family and friends, and societal perceptions and attitudes towards HIV. Medical concerns were another prominent theme, emphasizing the importance of a trust‐based relationship with their HIV physician during pregnancy and delivery. The women expressed difficulties in accepting medical interventions imposed on them, notably the need for Caesarean section and the advice not to breastfeed. A final prominent theme was the adjustment to life at home with the newborn. Themes relating to the role of the father/partner, the impact on the couple or physical and emotional changes were barely expressed or absent.

**Conclusions**: In this sample of WLWH, medical aspects of pregnancy were a central theme, overshadowing the broader implications of pregnancy and child‐rearing such as the role of the father/partner. While the women's narratives underscored the central role of the HIV physician, they also highlighted the need for a more holistic approach in supporting WLWH during pregnancy, notably, the phase beyond childbirth at home with the newborn and broader psychosocial aspects such as HIV status sharing.

### P174 Managing menopause in women living with HIV: impact of online medical education on physician knowledge and confidence

Shanthi Voorn ^1^, Alessia Piazza^1^, Gill Adair^1^, Elizabeth Bukusi^2^, Susan Cole‐Haley^3^, Shema Tariq^4^

^1^Global Education, Medscape LLC, London, UK. ^2^Department of Obstetrics and Gynecology, University of Washington, Seattle, WA, USA. ^3^Global Network of People Living with HIV (GNP+), London, UK. ^4^Institute for Global Health, University College London, London, UK

**Background**: Women with HIV are now reaching older age and experiencing menopause. Severe menopausal symptoms in these women are linked to greater psychological distress, lower quality of life, reduced adherence to antiretroviral therapy and decreased clinic attendance. We investigated if online medical education could enhance HIV and infectious disease (ID) physicians’ knowledge and confidence in managing menopause in women with HIV. As part of a broader curriculum, we created an online Continuing Medical Education (CME) activity titled “Managing Menopause in Women Living with HIV” to increase understanding of the impact and data on menopause in these women.

**Methods**: ID/HIV specialists participated in an online CME activity (https://www.medscape.org/viewarticle/997643) consisting of a 30‐minute discussion with three experts with accompanying slides. Educational effect was assessed using a four‐question repeated pairs, pre‐/post‐assessment. A paired samples *t*‐test was conducted for significance testing on overall average number of correct responses and for confidence rating, and a McNemar's test was conducted at the question level (5% significance level). Cohen's *d* for paired samples estimated the effect size of the education (<0.20 modest, 0.20–0.49 small, 0.59–0.79 moderate, ≥0.80 large). The CME activity launched on 2/7/2024, and the data were collected through 5/20/2024.

**Results**: A total of 978 ID/HIV specialists participated in the activity, of whom 31 completed the pre‐ and post‐activity questions. Overall, 69% of ID/HIV specialists improved their knowledge managing menopause in women living with HIV (*p* < 0.001) indicating a considerable effect of the education (Cohen's *d* = 1.02). The average percentage of correct responses rose from 6% to 45% for ID/HIV specialists’ pre‐activity to post‐activity. Seventy‐one percent of ID/HIV specialists had a measurable improvement in confidence in their ability to support patients coping with menopause optimally.

**Conclusions**: This online CME activity significantly improved ID/HIV specialists’ knowledge regarding the management of menopause in women living with HIV. There were significant confidence gains in physician ability to optimally support their patients with menopause. However, there is room for further improvement, up to 55% of physicians provided incorrect answers post‐education. By further addressing these educational gaps, physicians will be better equipped to care for patients living with HIV. 

### P175 Contraceptive methods in women living with HIV after a pregnancy: Condesa's Clinics, Mexico City

Teresita Cabrera ^1^, Marla Toiber^2^, Edgar Pérez^3^, Carlos Nava^4^, Ubaldo Ramos^4^, Ana Karen Soto^5^, Elena Langarica^6^, Adrian Cruz^7^, Paula Viveros^8^, Andrea Gónzalez^8^

^1^Ginecoobstetricia y Colposcopia, Clínica Especializada Condesa Iztapalapa, Ciudad de México, Mexico. ^2^Salud Mental, Clínica Especializada Condesa Iztapalapa, Mexico City, Mexico. ^3^Infectología, Clínica Especializada Condesa Iztapalapa, Mexico City, Mexico. ^4^Ginecoobstetricia, Clínica Especializada Condesa, Mexico City, Mexico. ^5^Trabajo Social, Clínica Especializada Condesa Iztapalapa, Mexico City, Mexico. ^6^Trabajo Social, Clínica Especializada Condesa, Mexico City, Mexico. ^7^Clínica Especializada Condesa Iztapalapa, Mexico City, Mexico. ^8^Center for HIV/AIDS, Clínica Especializada Condesa, Mexico City, Mexico

**Background**: Eighty‐two percent of pregnant and breastfeeding women living with HIV globally accessed antiretroviral treatment in 2022, this has led to a 58% reduction in new HIV infections among children from 2010 to 2022 [1]. In Mexico, 37 cases of vertical transmission of HIV were reported in 2023, compared to 133 cases in 2018 [2]. Contraceptive methods are a strategy to prevent vertical transmission of HIV. This study describes the contraceptive methods provided to women living with HIV in two public Specialized Clinics in Mexico City.

**Methods**: Cross‐sectional study, of a cohort of pregnant women living with HIV in Condesa Cuauhtemoc and Condesa Iztapalapa Specialized Clinic, Mexico City. From August 2013 to March 2024.

**Results**: Two hundred and eighty‐two WLHIV, 47.8% (135) chronic HIV women who knew their HIV diagnosis previous to their pregnancy and 52.1% (147) with HIV diagnosis during pregnancy because of screening. Age at HIV diagnosis 23.5 years old (7.5), years living with HIV 7.5 years (SD 5.5), current age 31 years old (SD 6.4). Partner HIV status: serodiscordant 50% (143), concordant 39.3% (113), unknown 3.5% (26). Age of onset of sexual activity 16.7 years old (SD 2.7), number of sexual partners 1–3: 59.5% (168), 4–6: 27.3% (77) more than 6 2.1% (37). Number of pregnancies 2.4 (SD 1.5). Resolution of pregnancy: C‐section birth 87.9% (248), vaginal delivery 6.3% (18), voluntary abortion 2.4% (7), spontaneous abortion 3.1% (9). Virological suppression 66.6% (188), virological failure 6.3% (18), lost to follow‐up 25.1% (71), deaths 1.7% (5). Viral load at the end of pregnancy <1000 c/ml 96% (271) and CD4 418 c/µl (SD 236). 87.2% (246) of women with some contraceptive method: definitive contraceptive method 57% (161), intradermal implants 19.1% (54) and hormonal intrauterine device 10.9% (31). No family planning method 12% (34), unknown 0.7% (2). Caesarean section was associated with acceptance of definitive contraceptive method (*p* < 0.05). Mean age of women with definitive contraceptive method was 32.3 years old (SD 5.96), mean age for hormonal intrauterine device was 29.3 years old (SD 6.84), hormonal implants 27.4 years old (SD 5.93). Compared to women who declined contraceptive methods 31.8 years old (SD 7.14).

**Conclusions**: The acceptance of any contraceptive method in our population was high; however, we will continue to work in achieving 100% acceptance of a contraceptive method. Through this strategy, we aim to reduce vertical transmission.

**References**

1. UNAIDS. The path that ends AIDS: UNAIDS Global AIDS Update 2023. Geneva: Joint United Nations Programme on HIV/AIDS; 2023.

2. CENSID, Centro Nacional para la Prevención y el Control del VIH y el Sida. Día Mundial del Sida 1 de diciembre 2023 [Internet]. 2023 [cited 2024 Jul 11]. Available from: www.gob.mx/cms/uploads/attachment/file/873833/BOLETIN_DAI_ESPECIAL_2023_30112023_1.pdf.

### P176 Prevalence and determinants of HIV sero‐positivity among vulnerable older women in rural Uganda: a call for targeted policy interventions

Gertrude Ddamulira Namale, Emmanuel Sendaula, Hilda Achayo, Silas Masari, Patrick Kazooba, Josephine Kaleebi

Reach Out Mbuya, Mbuya Community Health Initiative, Kampala, Uganda

**Background**: In sub‐Saharan Africa (SSA), there is increasing evidence that challenges in terms of unemployment, illiteracy levels and poverty place older women in rural areas in a uniquely vulnerable situation for acquiring HIV. However, despite the global attention being paid to the HIV epidemic, HIV sero‐positivity rates among older women in SSA have been a neglected area of research. We assessed the prevalence and determinants of HIV sero‐positivity among vulnerable older women attending a rural clinic in Central Uganda.

**Materials and methods**: Between January 2019 and December 2022, we conducted a mixed methods cross‐sectional study among older women aged ≥50 years. Vulnerability was defined as the risk of falling into poverty (inability to meet the basic necessities of life, poor access and quality of social services and inadequate infrastructure). Eligible and interested women were consented and offered HIV counselling and testing. Data on socio‐demographic and clinical characteristics were collected (Figure 1). Multivariable logistic regression was used to identify factors associated with HIV prevalence. In‐depth interviews were conducted to elicit information on participants’ knowledge of HIV and its prevention, and the challenges they go through. Qualitative data were analysed using a thematic content approach.

**Results**: Seven hundred and seventy‐nine women were included in the analysis; mean age was 68 (SD±10) years old. More than a third 279 (35%) of women were aged between 60 and 69 years and more than half 406 (52%) attained primary education. Almost two‐thirds 436 (56%) reported moderate vulnerability. Overall HIV sero‐positivity was 74 of 779 (9.5%), and of these, almost half 34 (46%) of the women had hypertension. In adjusted multivariable analysis, HIV sero‐positivity was more likely among women aged 50−59 years old (aOR = 15.28; 95% CI: 5.0−47.18) and those critically vulnerable (aOR = 6.97; 95% CI: 3.08−15.77). Qualitative data showed that most participants had good knowledge of HIV and its prevention. However, a significant proportion reported economic constraints and scarcity of resources including nutritional, medical and social support needs.

**Conclusions**: The prevalence of HIV sero‐positivity among older women was higher than the national average of 5.5%. Women aged 50−59 years and those critically vulnerable are at a higher risk of acquiring HIV. These findings highlight the critical need for targeted HIV prevention interventions that address multiple socio‐economic and behavioural factors contributing to the vulnerability in this population.

**P176: Figure 1:**
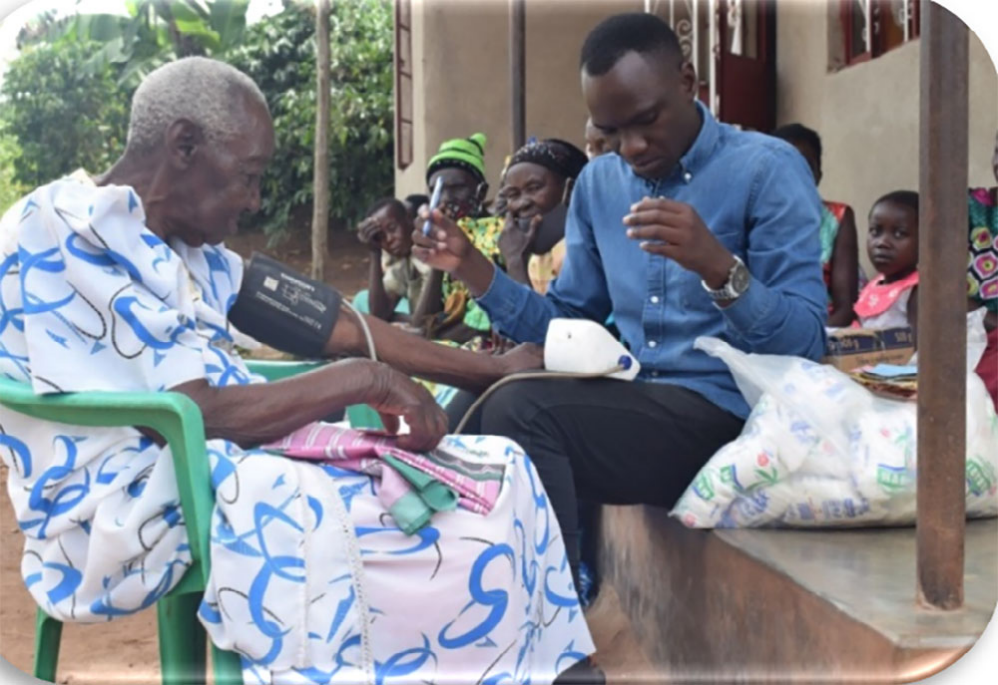
The staff assesses the vital status of an elderly woman during a community outreach.

## Clinical management considerations—Late presenters

### P177 Effectiveness of dolutegravir and boosted‐darunavir three‐drug regimens in advanced ART naïve: a trial emulation

Roberta Gagliardini^1^, Andrea Giacomelli^2^, Cristina Mussini^3^, Stephen R. Cole^4^, Jessie K. Edwards^4^, Carmela Pinnetti^1^, Alessandro Raimondi^5^, Spinello Antinori^6^, Silvia Nozza^7^, Valentina Mazzotta^1^, Giulia Marchetti^8^, Sergio Lo Caputo^9^, Andrea Antinori ^1^, Alessandro Cozzi‐Lepri^10^

^1^UOC Immunodeficienze Virali, INMI L. Spallanzani, Rome, Italy. ^2^III Infectious Diseases Unit, ASST Fatebenefratelli‐Sacco, Department of Biomedical and Clinical Sciences (DIBIC) Luigi Sacco, University of Milan, Milan, Italy. ^3^Department of Surgical, Medical, Dental and Morphological Sciences, University of Modena, Department of Infectious Diseases, Azienda Ospedaliero‐Universitaria Policlinico of Modena, Modena, Italy. ^4^Department of Epidemiology, Gillings School of Global Public Health, University of North Carolina, Chapel Hill, NC, USA. ^5^Department of Infectious Diseases, ASST Grande Ospedale Metropolitano Niguarda, School of Medicine and Surgery, Milan, Italy. ^6^III Infectious Diseases Unit, ASST Fatebenefratelli‐Sacco, DIBIC Luigi Sacco, University of Milan, Milan, Italy. ^7^Infectious Diseases Unit, IRCCS San Raffaele Scientific Institute, Vita Salute San Raffaele University, Milan, Italy. ^8^Clinic of Infectious Diseases, ASST Santi Paolo e Carlo, Department of Health Sciences, University of Milan, Milan, Italy. ^9^Clinic of Infectious Diseases, Department of Clinical and Surgical Sciences, University of Foggia, Foggia, Italy. ^10^Centre for Clinical Research, Epidemiology, Modelling and Evaluation, Institute for Global Health, London, UK

**Background**: There are no available randomized comparisons between dolutegravir (DTG) and boosted‐darunavir (DRV/b [ritonavir or cobicistat])‐based regimens in persons with HIV (PWH) presenting with advanced disease.

**Methods**: ART‐naïve PWH in the Icona Foundation Cohort with CD4 count <200 cells/mm^3^ and/or AIDS diagnosis (excluding cancers, cryptococcosis, tuberculosis and other mycobacterial infection), who started a first‐line three‐drug regimen with DTG or DRV/b over the period 2014–2023 were included. The primary outcome was a composite end point defined as a newly developed AIDS, serious non‐AIDS events (CVD, ESLD, ESRD, cancer), death, virological failure >200 copies/ml and treatment discontinuation of the anchor drug due to failure or toxicity. The secondary outcome counted only clinical progression events. We used a marginal structural Cox regression model to estimate the causal effect of starting DTG instead of DRV/b‐based regimens.

**Results**: Characteristics of the 1307 ART‐naïve PWH were (DTG = 888; DRV/b = 419): females 21%; heterosexual contacts 49%, MSM 37%; born outside Italy 30%; AIDS presenting 33%; median age 45 years (IQR 36–53), median CD4 count at nadir 70 cells/mm^3^ (IQR 26–130); median HIV‐RNA 5.30 log_10_ copies/ml (IQR 4.77–5.80). The unweighted risk of developing the composite end point by 48 months were 39.9% (95% CI 33.9–46.1%) for DRV/b versus 21.9% (18.5–25.3%) for DTG (*p* < 0.0001, Figure 1, top). Overall, PWH who initiated DTG showed a lower risk of experiencing the composite end point compared to those who started DRV/b (weighted [w] RH 0.49 [95% CI 0.36–0.67]), even in recent years (in 2019–2023 wRH 0.42 [95% CI 0.26–0.68]) (Table 1). The unweighted risk of developing the clinical end point by 48 months was 17.5% (95% CI 12.8–22.3%) for DRV/b versus 14.3% (11.4–17.2%) for DTG (*p* = 0.12, Figure 1, bottom). Also, in this secondary analysis, a trend towards a lower risk of clinical progression for DTG versus DRV/b was observed (wRH 0.66 [95% CI 0.44–1.01]), regardless of calendar year of starting ART.

**Conclusions**: Under our strong assumptions, our analysis suggests that in ART‐naïve PWH with CD4 count <200 or AIDS initiating ART with DTG versus DRV/b‐based regimens leads to a 50% reduction in the risk of treatment failure. The difference was mainly but not fully explained by anchor drug discontinuation.

**P177: Table 1:** RH of the estimated causal effect of treatment on the risk of developing the composite end point^a^ and the clinical end point from fitting a weighted Cox regression model (overall and stratified by calendar periods of starting ART)

Unweighted and weighted relative hazards (RHs) of treatment failure^a^				
	Unweighted RH (95% CI)	*p*‐value	Weighted^b^ RH (95% CI)	*p*‐value
All years				
DRV/b	1.00		1.00	
DTG	0.52 (0.42−0.66)	<0.001	0.49 (0.36−0.67)	<0.001
2014−2018				
DRV/b	1.00		1.00	
DTG	0.53 (0.40−0.70)	<0.001	0.51 (0.33−0.80)	0.003
2019−2023				
DRV/b	1.00		1.00	
DTG	0.51 (0.33−0.78)	0.002	0.42 (0.26−0.68)	<0.001

Unweighted and weighted relative hazards (RHs) of AIDS/SNAE or death				
	Unweighted RH (95% CI)	*p*‐value	Weighted^b^ RH (95% CI)	*p*‐value
All years				
DRV/b	1.00		1.00	
DTG	0.78 (0.56−1.07)	0.127	0.66 (0.44−1.01)	0.055
2014−2018				
DRV/b	1.00		1.00	
DTG	0.67 (0.47−0.98)	0.038	0.64 (0.36−1.14)	0.131
2019−2023				
DRV/b	1.00		1.00	
DTG	1.22 (0.62−2.41)	0.561	0.54 (0.28−1.06)	0.076

Abbreviations: CVD, cardiovascular disease; ESLD, end‐stage liver disease; ESRD, end‐stage renal disease; RH, relative hazard.

^a^Newly developed AIDS and SNAE events, death, viral failure >200 copies/ml and of discontinuation of DRV or DTG due to failure or toxicity.

^b^Weighted for age, gender, mode of HIV transmission, nationality, calendar year of starting ART, AIDS/SNAE at baseline, time from HIV diagnosis, NRTI used and current alanine aminotransferase (ALT), CD4 and HIV‐RNA values.

**P177: Figure 1:**
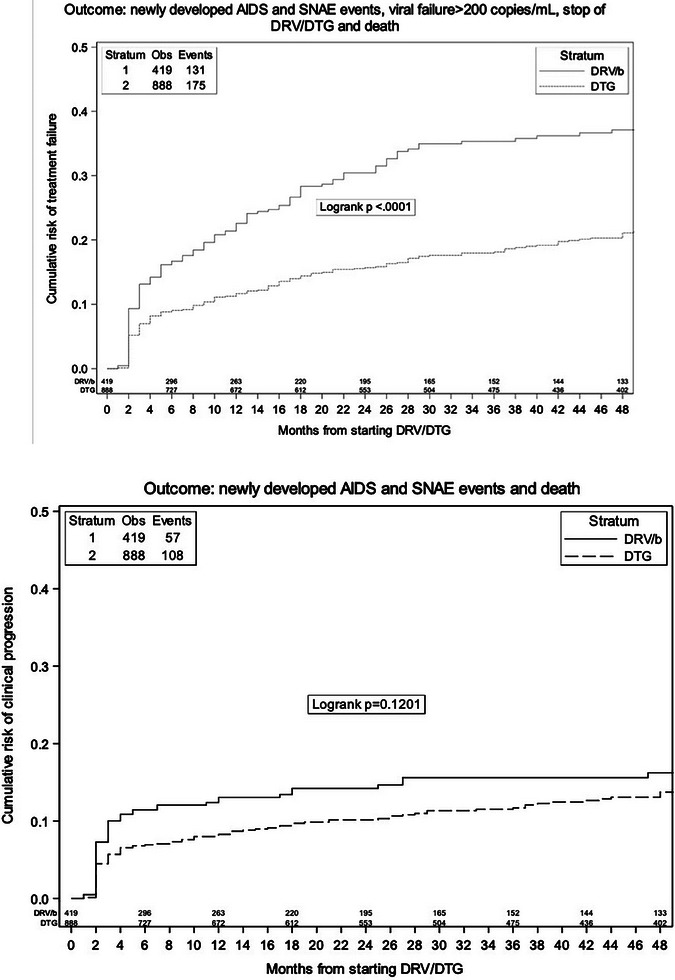
(top) Unweighted Kaplan‐Meier curves by anchor drug (DTG vs. DRV/b) for the composite end point; (bottom) unweighted Kaplan‐Meier curves by anchor drug (DTG vs. DRV/b) for the clinical end point. DRV/b, darunavir boosted; DTG, dolutegravir; SNAE, serious non‐AIDS event.

### P178 Risk of long‐term clinical progression in PWH initiating a first‐line ART with advanced HIV disease and failing to have a robust CD4 count response despite viral suppression

Giulia Marchetti^1^, Camilla Tincati ^1^, Alessandro Cozzi‐Lepri^2^, Valeria Bono^1^, Alessandro Tavelli^3^, Eugenio Milano^4^, Stefania Piconi^5^, Cecilia Costa^6^, Annalisa Mondi^7^, Andrea Gori^8^, Miriam Lichtner^9^, Andrea Antinori^7^, Francesca Ceccherini‐Silberstein^10^, Antonella d'Arminio Monforte^3^, Carlo‐Federico Perno^11^

^1^Department of Health Sciences, University of Milan, San Paolo Hospital, Milan, Italy. ^2^Institute for Global Health, Centre for Clinical Research, Epidemiology, Modelling and Evaluation, UCL, London, UK. ^3^Fondazione ICONA, Milan, Italy. ^4^Clinic of Infectious Diseases, University Hospital Policlinic of Bari, Bari, Italy. ^5^Clinic of Infectious Diseases, Alessandro Manzoni Hospital‐ASST Lecco, Lecco, Italy. ^6^Infectious Disease‐SOC1, USL Centro Firenze, Santa Maria Annunziata Hospital, Florence, Italy. ^7^Clinical and Research Infectious Diseases, National Institute for Infectious Diseases Lazzaro Spallanzani IRCCS, Rome, Italy. ^8^Biomedical and Clinical Sciences Luigi Sacco, University of Milan, Sacco Hospital, Milan, Italy. ^9^Neuroscience and Mental Health and Sense Organs, Sapienza University, Sant'Andrea Hospital, Rome, Italy. ^10^Experimental Medicine, University of Rome, Tor Vergata, Rome, Italy. ^11^Unit of Diagnostic Microbiology and Immunology and Multimodal Medicine Area, Bambino Gesù Children's Hospital, Rome, Italy

**Background**: A proportion of persons with HIV (PWH) do not achieve robust CD4 count response upon initiation of ART despite sustained viral suppression. Whether impaired early CD4 count recovery may influence the long‐term risk of clinical progression has been seldom evaluated.

**Materials and methods**: We included PWH of the Icona Foundation Study who started a first ART over 1997–2021, with a CD4 nadir <200/mm^3^, achieved HIV‐RNA ≤50 copies/ml over the time window 6–12 months of ART and maintained viral suppression for ≥24 months. CD4 count non‐response (CD4‐NR) was defined as having never achieved an absolute CD4 T‐cell count >350/mm^3^ and displaying an average slope of CD4^+^ increase <8.3 cells/month over the 24 months with HIV‐RNA ≤50 copies/ml. At month 24 (baseline), PWH were classified as CD4‐NR versus responders (CD4‐R). Kaplan‐Meier curves and standard Cox proportional‐hazard regression models with baseline time‐fixed covariates were used to compare the time to developing a new AIDS or serious non‐AIDS events (SNAEs) diagnosis (cardiovascular disease, non‐AIDS cancer, end‐stage liver disease and end‐stage renal disease) or death after baseline according to CD4 count response groups.

**Results**: A total of 1885 PWH were included; 78% were male, 51% acquired HIV through heterosexual contacts, 36% had a previous AIDS diagnosis and 10% HCVAb+. At baseline, 510 (27%) were classified as CD4‐NR, the proportion CD4‐NR in INSTI era (after 2014) was 29% (Figure 1a). CD4‐NR and CD4‐R differed significantly in terms of median age (46 vs. 41 years, *p* < 0.001), nadir CD4 (71 vs. 94 cells/mm^3^, *p* < 0.001) and baseline CD4 count (237 vs. 469 cells/mm^3^, *p* < 0.001). By 10 years from baseline, 21.4% (95% CI 15.9–26.9%) CD4‐NR and 14.5% (95% CI 11.7–17.3%) CD4‐R developed AIDS, SNAE or death (log‐rank test *p* = 0.0001, Figure 1b). The relative hazard of AIDS, SNAE/death associated with CD4‐NR was 1.48 (1.09–2.00; *p* < 0.01) after controlling for key confounders. In contrast, age appeared to explain most of the excess risk of SNAE/death associated with CD4‐NR (Table 1).

**P178: Table 1:** Relative hazards (RHs) from fitting a Cox regression model

	Unadjusted RH (95% CI)	*p*‐value	Adjusted^a^ RH (95% CI)	*p*‐value	Adjusted^b^ RH (95% CI)	*p*‐value
Outcome: new SNAE/death						
CD4‐R	1		1		1	
CD4‐R	1.67 (1.24−2.27)	<0.001	1.30 (0.95−1.77)	0.096	1.43 (0.99−2.05)	0.056
Outcome: new AIDS, SNAE/death						
CD4‐R	1		1		1	
CD4‐R	1.83 (1.38−2.43)	<0.001	1.46 (1.09−1.95)	0.011	1.60 (1.15−2.24)	0.006

^a^For age.

^b^For age, gender, nationality, mode of HIV transmission, year of baseline, CD4 nadir, baseline CD8 and at ART initiation, AIDS, INSTI use in first‐line, hepatitis co‐infection and VL blip before baseline.

**Conclusions**: CD4‐NR shortly after initiation of therapy remains frequent in the era of INSTI. CD4 non‐response appears to be a strong predictor of long‐term clinical progression, while the association with the risk of SNAE/death was substantially confounded by age.

**P178: Figure 1:**
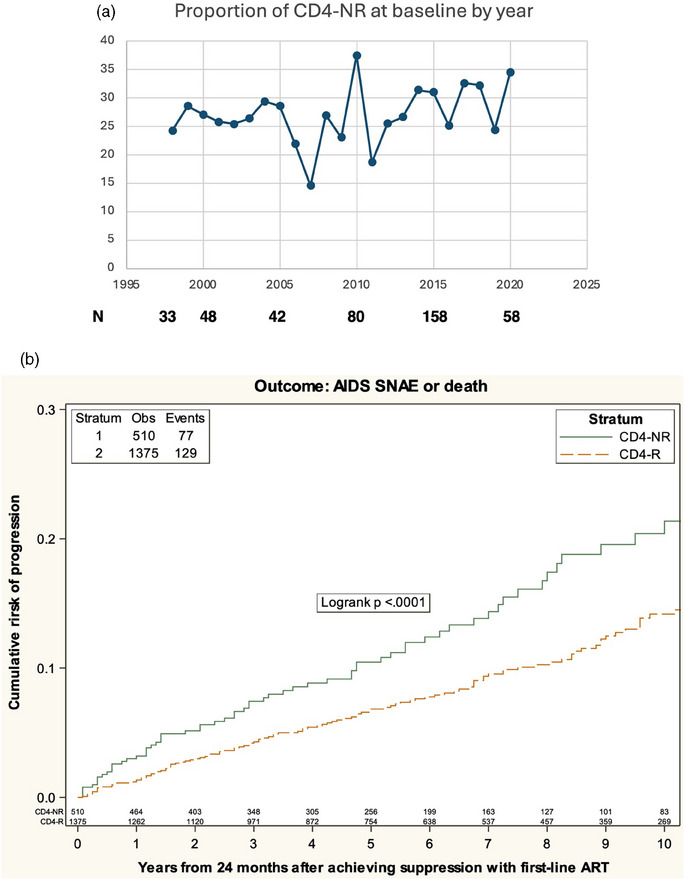
(a) Proportion of CD4‐NR according to calendar year of baseline; (b) Kaplan‐Meier estimates of the time to AIDS/SNAE/death according to CD4 response.

### P179 Screening characteristics of participants in an open label, multi‐centre, randomized controlled trial investigating integrase inhibitor versus boosted protease inhibitor antiretroviral therapy for late presenters with advanced HIV disease (LAPTOP)

Georg Behrens ^1^, Lambert Assoumou^2^, Jose Arribas^3^, Christine Katlama^4^, Frank Post^5^, Jean Michel Molina^6^, Andrea Antinori^7^, Rafael Micán^3^, Stephane De Wit^8^, Jürgen Rockstroh^9^, Lisa Hamzah^10^, Pere Domingo^11^, Adrian Curran^12^, Montserrat Laguno^13^, Carl Fletcher^14^, Debbie Roberts^14^, Jack Moody^14^, Anton Pozniak^15^, Study Group LAPTOP^16^

^1^Department of Rheumatology and Immunology, Hannover Medical School, Hannover, Germany. ^2^Centre de Méthodologie et de Gestion (CMG), Institut Pierre Louis d'Epidémiologie et de Santé Publique, Paris, France. ^3^Unidad de Enfermedades Infecciosas, Hospital Universitario La Paz, Madrid, Spain. ^4^Service de Maladies Infectieuses, Hopital Universitaire Pitié‐Salpetrière, Paris, France. ^5^Department of Inflammation Biology, Kings College Hospital NHS Foundation Trust, London, UK. ^6^Service des Maladies Infectieuses et Tropicales, Hospital Saint‐Louis and Lariboisière, Paris, France. ^7^Unità Operativa Complessa (UOC) Immunodeficienze Virali, National Institute for Infectious Diseases Lazzaro Spallanzani IRCCS, Roma, Italy. ^8^Department of Internal Medicine, University Hospital of Saint‐Pierre, Brussels, Belgium. ^9^Department of Internal Medicine, University Hospital Bonn, Bonn, Germany. ^10^Department of Clinical Infection, St George's Hospital, London, UK. ^11^Infectious Diseases Unit, Hospital de la Santa Creu I Sant Pau, Barcelona, Spain. ^12^Department of Infectious Diseases, Hospital Universitario Vall d'Hebron, Barcelona, Spain. ^13^Infectious Diseases Service, Hospital Clinic de Barcelona, Barcelona, Spain. ^14^Project Management, Research Organisation (Kings Cross), London, UK. ^15^Department of Infectious Diseases, Chelsea and Westminster NHS Foundation Trust, London, UK. ^16^Research Organisation (Kings Cross), London, UK

**Background**: Over half of new HIV diagnoses in Europe present late (CD4 cell count <350/µl), yet most first‐line HIV treatment randomized controlled trials recruit individuals who have low baseline viral loads, high CD4 counts, fewer co‐morbidities, drug‐drug interactions and other treatment failure risks than those presenting with advanced disease. We conducted the LAPTOP trial in people with advanced disease; here, we present participants’ clinical characteristics at screening.

**Methods**: LAPTOP is a 48‐week, open‐label, European, multi‐centre, non‐inferiority, controlled trial comparing outcomes for people with advanced HIV disease randomized 1:1 to receive bictegravir (BIC) or darunavir (DRV)/cobicistat (c) co‐formulated with emtricitabine (FTC)/tenofovir alafenamide (TAF). Inclusion criteria include untreated HIV‐1, HIV‐RNA >1000 copies/ml, and at least one of the following: (1) AIDS‐defining condition/any CD4 count; (2) severe bacterial infection/CD4 <200/µl; (3) any or no symptoms/CD4 <100/µl; and (4) serious opportunistic infection currently under treatment. Resistance data were collected at screening.

**Results**: Four hundred and forty‐seven screened individuals (80.8% male, 66% White or White mixed ethnicity, median age: 43 years, IQR 35–53 years) were analysed. The median (IQR) HIV RNA was 5.6 (5.1–6.0) log copies/ml with 81.4% of patients with late diagnosis had HIV‐RNA >100,000 copies/ml. CD4 count median (IQR) was 41 (17–79) cells/ml, and CD4:CD8 ratio median (IQR) was 0.08 (0.04–0.14). Two hundred and twenty of 447 (49%) were included due to an AIDS‐defining illness. Twenty‐seven of 425 participants had intermediate‐ or high‐level resistance to at least one ARV class, mostly NNRTI. 96.5% and 97.9% were fully susceptible to BIC/FTC/TAF or DRV/c/FTC/TAF, respectively (Table 1).

**P179: Table 1:** Resistance data at screening

Resistance testing available, *n* (%)	425 (95.1)
Among those with resistance tests, number of days between resistance test and screening visit (median [IQR])	4 (0−9)
No high‐level or intermediate resistance to ARV	398/425 (93.6)
NRTI resistance, *n* (%) high‐level; intermediate resistance	3 (0.7); 2 (0.5)
NNRTI resistance, *n* (%) high‐level; intermediate resistance	16 (3.8); 3 (0.7)
PI resistance, *n* (%) high‐level; intermediate resistance	0 (0); 1 (0.2)
INSTI resistance, *n* (%) high‐level; intermediate resistance	3 (0.7); 1 (0.2)
Resistance to more than one class	2 (0.5)

*Note*: Please note resistance interpretation was made using the Stanford algorithm (last updated on 2023‐11‐05).

**Conclusions**: Almost half of LAPTOP trial participants were diagnosed after an AIDS‐defining condition was present, and over half had CD4 counts less than 50 cells/µl. The trial will generate important safety and efficacy data for current ART in this population.

### P180 Late presentation and HIV‐1 subtype diversity among Ukrainian migrants newly diagnosed with HIV in Poland

Milosz Parczewski ^1^, Iwona Cielniak^2^, Ewa Siwak^3^, Elżbieta Mularska^4^, Adam Witor^4^, Błażej Rozpłochowski^5^, Bogusz Aksak‐Was^1^, Magdalena Witak‐Jedra^6^, Aleksandra Szymczak^7^, Bartosz Szetela^7^, Monika Bociąga Jasik^8^, Anna Kalinowska‐Nowak^8^, Paweł Jakubowski^9^, Maria Hlebowicz^10^, Elzbieta Jablonowska^11^, Wladysław Łojewski^12^, Anita Olczak^13^, Kaja Mielczak^1^, Piotr Zabek^14^

^1^Department of Infectious, Tropical Diseases and Immune Deficiency, Pomeranian Medical University in Szczecin, Szczecin, Poland. ^2^Faculty of Medical Science, Collegium Medicum Cardinal Stefan Wyszynski University in Warsaw, Warsaw, Poland. ^3^Department of Infectious and Tropical Diseases and Hepatology, Medical University of Warsaw, Warsaw, Poland. ^4^Department of Infectious Diseases, Regional Hospital Chorzów, Chorzow, Poland. ^5^Department of Infectious Diseases, Hepatology and Acquired Immunodeficiencies, Karol Marcinkowski University of Medical Sciences, Poznań, Poland. ^6^Department of Infectious, Tropical Diseases and Immune Deficiency, Regional Hospital, Szczecin, Poland. ^7^Department of Infectious Diseases, Liver Diseases and Acquired Immune Deficiencies, Wroclaw Medical University, Wroclaw, Poland. ^8^Department of Infectious and Tropical Diseases, Jagiellonian University Medical College, Kraków, Poland. ^9^Infectious Diseases, Pomeranian Hospitals, Gdańsk, Poland. ^10^Department of Family Medicine and Infectious Diseases, University of Warma and Mazury in Olsztyn, Olsztyn, Poland. ^11^Department of Infectious Diseases and Hepatology, Medical University of Lódz, Lódz, Poland. ^12^Department of Infectious Diseases, Regional Hospital in Zielona Gora, Zielona Góra, Poland. ^13^Department of Infectious Diseases and Hepatology, Faculty of Medicine, Nicolaus Copernicus University Ludwik Rydygier Collegium, Bydgoszcz, Poland. ^14^Molecular Diagnostics Laboratory, Hospital for Infectious Diseases, Warsaw, Poland

**Background**: Migration is a well‐established factor associated with late HIV diagnosis. War in Ukraine forced displacement of ∼6 million people, with large proportion taking refuge in Poland, which is now hosting >3500 Ukrainian migrants living with HIV [1]. Majority were diagnosed and initiated antiretroviral therapy still in the home country; however, approximately 25% of people with HIV (PWH) in Ukraine were estimated to be undiagnosed. Here, we present the data on the national survey on new HIV diagnoses in Ukrainian migrants since the beginning of war.

**Materials and methods**: Clinical data from 439 Ukrainian migrants newly diagnosed with HIV in Poland since February 2022 were collected from 11 Polish HIV treatment centres. Where available, baseline HIV‐1 sequence was analysed for subtype [2] and drug resistance [3]. Late diagnosis was defined using updated definition [4] and associated with clinical and virological variables. Time trends in late diagnosis were calculated using logistic regression.

**Results**: Gender distribution in the group was similar (49.2% female, 50.8% male), with median age at HIV diagnosis of 37 (IQR 31–44) years and lymphocyte CD4 count of 198 (IQR 50–428) cells/µl (Table 1). Transmission route was mostly heterosexual (72.4%). AIDS was diagnosed in 153 patients (34.9%), 32.9% of these had tuberculosis. At diagnosis, hepatitis B surface antigen was detectable in 3.8%, while 19.4% of PWH presented with anti‐hepatitis C virus (HCV) antibodies (72.5% of them with replicative HCV virus). A6 variant was predominant (88.5%) with drug‐resistance mutations to non‐nucleoside reverse transcriptase inhibitors (NNRTIs) observed in 13.7% of sequences, mutations to other drug classes being infrequent. Late presentation was common (68.6%) and associated with older age, heterosexual and injection drug use transmission routes, HCV coinfection and presence of subtype A6, while timely diagnoses more common among men who have sex with men, people with diagnosed syphilis and subtype B. Proportion of late diagnosed migrants was stable across the 2 years since outbreak of war (Figure 1).

**P180: Table 1:** Clinical characteristics of Ukrainian migrants with newly diagnosed HIV infection

	Total	Late diagnosed (*n* = 301, 68.6%)	Timely diagnosed (*n* = 138, 31.4%)	*p*‐value
Age, median (IQR)	37 (31−44)	40 (34−45)	33 (27−40)	<0.0001
Gender, *n* (%)				
Male	223 (50.8)	153 (68.6)	70 (31.4)	0.98
Female	216 (49.2)	148 (68.5)	68 (31.5)	
Transmission route, *n* (%)				
Heterosexual	317 (72.4)	219 (69.1)	98 (30.9)	<0.0001
Men who have sex with men	76 (17.3)	41 (52.6)	36 (47.4)	
People who inject drugs	45 (10.3)	41 (91.1)	4 (8.9)	
Lymphocyte CD4 count at care entry, cells/µl, median (IQR)	198 (50−428)	106 (20−210)	508 (428−672)	<0.0001
HIV‐RNA levels at baseline, log copies/ml, median (IQR)	5.1 (4.28−5.7)	5.4 (4.77−5.9)	4.22 (3.68−4.98)	<0.0001
HBV surface antigen positive (HBsAg), *n* (%)	16 (3.8)	14 (4.9)	2 (1.5)	0.08
HBV core antibody positive (HBcAb), *n* (%)	110 (30.2)	84 (33.5)	26 (23.0)	0.04
HBV surface antibody (HBsAb) >10 IU/ml (positive), *n* (%)	73 (20.6)	50 (20.5)	23 (20.9)	0.92
Anti‐HCV IgG antibody positive, *n* (%)	82 (19.4)	64 (22.2)	18 (13.5)	0.037
HCV‐RNA detectable (only analysed in anti‐HCV positive), *n* (%)	58 (72.5)	43 (71.7)	15 (75.0)	0.2
Syphilis non‐treponemal serology—Venereal Research Disease Laboratory (VDRL) or equivalent positive, *n* (%)	50 (12.8)	28 (10.45)	22 (18.2)	0.035
HIV variant (available for 227 cases), *n* (%)				
A6	201 (88.5)	151 (75.1)	50 (24.9)	0.002
B	22 (9.7)	8 (38.1)	14 (63.6)	
Other	4 (1.7)	3 (1.3)	1 (0.4)	

**Conclusions**: Late HIV diagnoses remain frequent among Ukrainian migrants with tuberculosis as the most common AIDS‐defining condition. Transmissions of A6 variants continue to predominate in the group with common NNRTI drug resistance.

**P180: Figure 1:**
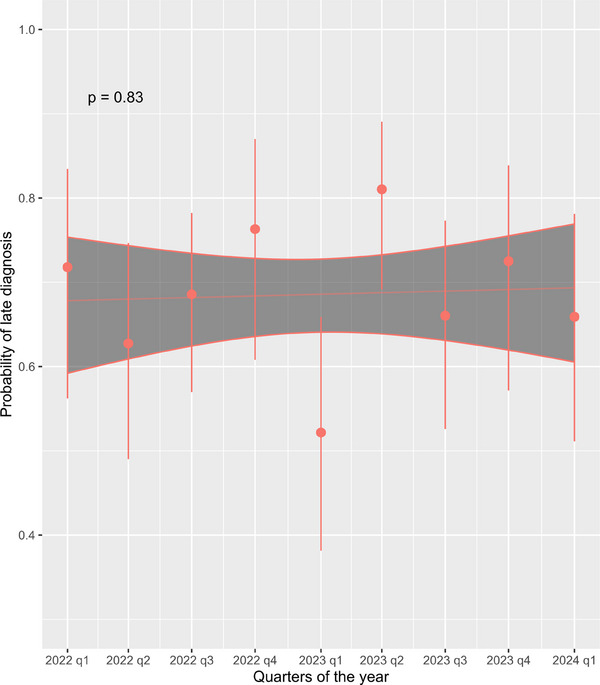
Time trends in late HIV diagnosis since the beginning of war in Ukraine among newly diagnosed migrants and refugees.

**References**

1. Parczewski M, Jabłonowska E, Wójcik‐Cichy K, Zhyvytsia D, Witak‐Jędra M, Leszczyszyn‐Pynka M, et al. Clinical perspective on human immunodeficiency virus care of Ukrainian war refugees in Poland. Clin Infect Dis. 2023;76(10):1708‐15.

2. Struck D, Lawyer G, Ternes AM, Schmit JC, Bercoff DP. COMET: adaptive context‐based modeling for ultrafast HIV‐1 subtype identification. Nucleic Acids Res. 2014;42(18):12.

3. Stanford University. HIV Drug Resistance Database [Internet]. 2024 [cited 2024 Jul 25]. Available from: https://hivdb.stanford.edu/.

4. Croxford S, Stengaard AR, Brännström J, Combs L, Dedes N, Girardi E, et al. Late diagnosis of HIV: an updated consensus definition. HIV Med. 2022;23(11):1202‐8.

### P181 HIV late presentation and advanced HIV disease increased in Latin America during second year of the COVID‐19 pandemic

María Marta Greco ^1^, Pedro Zitko^2^, Carlos Beltrán^3^, Ernesto Martínez Buitrago^4^, Alejandro Afani^5^, Jesús Elías Dawaher^6^, Andrea González^7^, Isabel Cassetti^8^, Mónica Mantilla^9^, Ana Lucía Tobias^10^, Elena Obieta^11^

^1^Infectious Diseases, Spanish Hospital, La Plata, Argentina. ^2^Unit for Healthcare Research, Barros Luco Trudeau Hospital, Santiago, Chile. ^3^Infectology, Hospital Barros Luco Trudeau, Santiago, Chile. ^4^Infectology, Sociedad Integral de Especialistas en Salud, Institución Prestadora de Salud, Cali, Colombia. ^5^Infectology, Hospital Clínico Universidad Nacional de Chile, Santiago, Chile. ^6^Infectology, Pablo Suarez Hospital, Quito, Ecuador. ^7^Clínica Condesa, Mexico City, Mexico. ^8^Helios Salud, Buenos Aires, Argentina. ^9^HIV Program Vivir Plus Virrey Solis, Institución Prestadora de Salud, Bogotá, Colombia. ^10^Infectious Diseases, Instituto de Seguridad Social, Guatemala, Guatemala. ^11^Infectious Diseases, Hospital Municipal, Boulogne City, Argentina

**Background**: HIV late presentation (LP) to care (CD4^+^ count <350 cells/µl) and advanced HIV disease (AD) (CD4^+^ count <200 cells/µl or an AIDS‐defining event) remain prevalent worldwide. Our group, in a sample of 43,911 new HIV cases entering to care between 2013 and 2017, reported a decrease in AD and LP: 40.5% to 31.9% and 63.7% to 54.1%, respectively. Data are limited regarding of the impact of the SARS‐CoV2 pandemic in HIV testing in Latin America. We aim to review the landscape of HIV late presentation in the region during the second year of COVID‐19 pandemic.

**Materials and methods**: We conducted a cross‐sectional, multicentre and multi‐country study using data from HIV centres part of the Latin‐American HIV Workshop Study Group (LAHWSG), including 101 centres from 14 countries. Data of people entering to HIV care during 2021 were stratified by age, sex and CD4^+^ count were collected. Three‐level random effect meta‐analysis was implemented, using weighted data according to type of HIV centre (public or private) and the expected number of patients in care.

**Results**: Eight thousand, three hundred and fifteen new HIV infected patients, from 59 centres from 12 Latin‐American countries provided data for the analysis; 85.1% were men and 10.6% older than 50 years old. LP accounted 56.7% [95% CI 49.1–64.2], while AD achieved 33.4% [95% CI 25.8–41.1]. Approximately, 40% of variance of LP and AD was accounted by country‐level factors. LP and AD was slightly higher in women: 60.6% [95% CI 48.7–72.4] versus 55.6% [95% CI 47.4–63.8], and 36.0% [95% CI 23.9–48.1] versus 33.1% [95% CI 24.9–41.3], respectively. LP and AD showed higher rates according to age increase (Figure 1).

**Conclusions**: During second year of COVID‐19 pandemic, the rate of LP and AD of people entering to HIV care increased in Latin America, rising along with age. HIV testing policies should be urgently intensified and tailored considering characteristics of population arriving late to care.

**P181: Figure 1:**
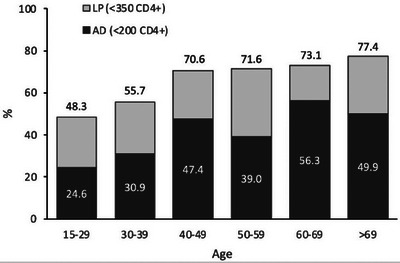
Percentage of people with HIV late presentation and advanced HIV disease according to age.

### P182 Implementation of HIV teams sustainably improves HIV indicator condition testing rates in hospitals in the Netherlands: the #aware.hiv study

Carlijn Jordans ^1^, Klaske Vliegenthart‐Jongbloed^1^, Kara Osbak^2^, Jaap Hanssen^3^, Marion Vriesde^1^, Jan van Beek^1^, Natasja van Holten^3^, Willemien Dorama^3^, Dorien van der Sluis^3^, Annelies Verbon^4^, Anna Roukens^3^, Casper Rokx^1^

^1^Infectious Diseases, Erasmus Medical Center, Rotterdam, Netherlands. ^2^Medical Microbiology, Maasstad Hospital, Rotterdam, Netherlands. ^3^Infectious Diseases, Leiden University Medical Center, Leiden, Netherlands. ^4^Infectious Diseases, University Medical Center Utrecht, Utrecht, Netherlands

**Background**: Missed testing opportunities contribute to late HIV diagnoses impacting many people worldwide. HIV indicator condition (IC)‐guided testing helps to identify undiagnosed HIV infections. The aim of this study was to evaluate the effect of implementing HIV teams on HIV IC‐guided testing in hospitals in the Netherlands.

**Materials and methods**: The #aware.hiv project is an ongoing prospective implementation study. Here, we present data from January 2020 to July 2023. Patients ≥18 years newly diagnosed with HIV ICs in Erasmus Medical Center (EMC) were included. The intervention consisted of proactive peer‐to‐peer HIV testing recommendations and education from the HIV team to the treating physician. The primary outcome was the overall HIV testing rate in patients diagnosed with HIV ICs. Secondary outcomes were HIV testing rates over time, per specialty and the HIV prevalence. Additionally, we evaluated reasons for physicians to withhold HIV testing. For external validation, we implemented this strategy in a second hospital in Leiden University Medical Center (LUMC).

**Results**: At EMC, the HIV testing rate increased significantly from 50.1% (222/443) pre‐implementation of HIV teams to 80.7% (1576/1952) post‐implementation (*p* < 0.001). The overall HIV testing rate showed a sustained increase with variability over time (range 72.4–89.9%). This sustained increase was observed across all medical specialties, except for dermatology (Figure 1). HIV prevalence among those tested was 0.6% (95% CI 0.3–1.1%). The HIV team intervened in 411 HIV ICs, resulting in 69 (16.3%) extra HIV ICs being tested for HIV. Reasons for not testing were provided in 49.4% of cases. Most frequent reasons were patient not returning to the hospital after HIV test advice was given (18.4%), HIV test in diagnostic plan but not performed (10.2%) and physicians assuming there was no clinical indication to test for HIV (8.5%) (Table 1). When HIV teams were implemented in LUMC, the testing rate increased significantly from 51.8% (87/168) pre‐implementation to 66.3% (114/172) post‐implementation (*p* < 0.01) with an HIV positivity rate of 0.9% (95% CI 0.02–4.8%).

**P182: Table 1:** Reasons provided by treating physicians within the #aware.hiv project for not testing for HIV at Erasmus Medical Center

Reasons not to test for HIV	*n* (%)
No reason provided	173 (50.6)
Patient did not return to hospital after HIV test advice was given	63 (18.4)
HIV test in diagnostic plan, but not performed	35 (10.2)
Physician assumed there was no clinical indication to test for HIV	29 (8.5)
Physician advised to perform HIV test somewhere else	12 (3.5)
Patient was in palliative stage	7 (2.0)
HIV test was ordered by physician, but not performed	7 (2.0)
Shared decision‐making physician and patient to not perform HIV test	5 (1.5)
HIV test offered by physician, but not accepted by patient	4 (1.2)
Patient died soon after HIV test advice was given	1 (0.3)
Physician forgot to test for HIV	1 (0.3)
HIV test in diagnostic plan, patient refused to come back to the department	1 (0.3)
Patient wants no further appointments	1 (0.3)
Physician assumed that HIV testing was done elsewhere	1 (0.3)
Physician thinks that other specialty should test for HIV	1 (0.3)
Physician reports that patient has been recently tested elsewhere, but no documentation on HIV test result has been documented	1 (0.3)
Total	342

**Conclusions**: Implementing HIV teams in hospitals significantly and sustainably increased HIV IC‐guided testing and can be successfully implemented in another hospital. These results encourage the broader adoption of HIV teams in diverse hospital settings.

**P182: Figure 1:**
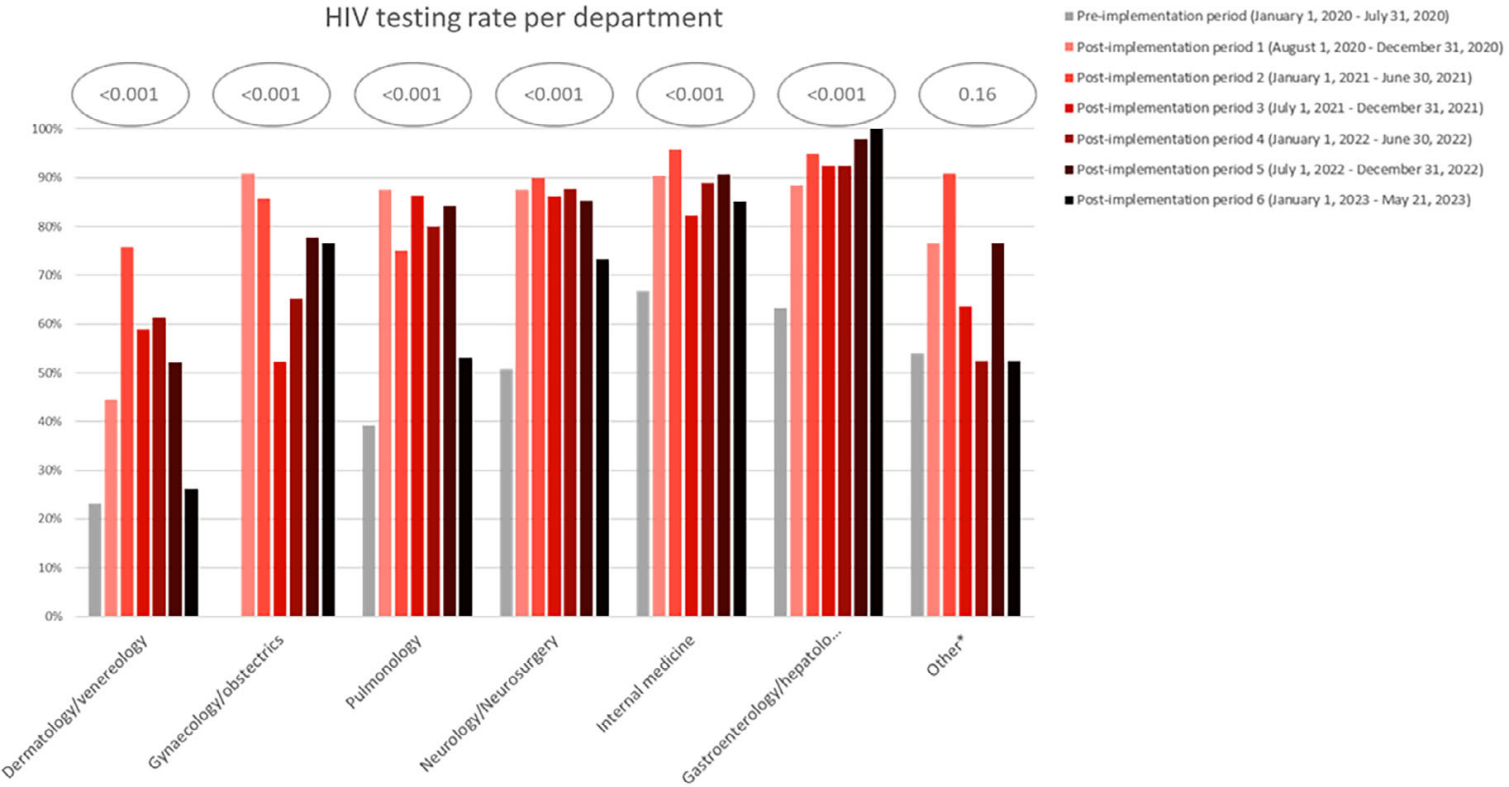
HIV testing rates over time per department, pre‐ and post‐implementation of HIV teams at Erasmus Medical Center. *Included the following departments: cardiothoracic surgery, ophthalmology, orthopaedic surgery, otorhinolaryngology, psychiatrics, rheumatology, surgery and urology; **p‐value calculated using chi‐square tests.

### P183 Late HIV diagnosis in a cohort from Buenos Aires: high mortality and burden of HIV‐related disease during the first year

Ana Rodriguez, Victoria Pinto, Julián Vega, Violeta Zulema Ortiz, José Ángel Enrique Barletta, María José Rolón

Infectious Disease, Hospital General de Agudos, Ciudad Autónoma de Buenos Aires, Argentina

**Background**: Late diagnosis of HIV (LD) represents a major challenge worldwide, and is associated with a high burden of HIV‐related disease. In Argentina, 43.8% of new HIV cases are LD, and 27.2% have advanced HIV disease (AHD) with a 10% first‐year mortality.

**Materials and methods**: This retrospective study was aimed to describe clinical profile and first‐year mortality among PLHIV with LD in a cohort from a referral centre in Buenos Aires, Argentina. Adults with new diagnoses between 2016 and 2022 were included and followed during the first year after cohort entry. Data was collected from medical records and surveillance systems. LD was defined as CD4 <350 cells/µl and/or World Health Organization (WHO) 3–4 events; and AHD as CD4 <200 cells/µl and/or WHO events 3–4.

**Results**: A total of 920 new HIV diagnoses were made during the study period, 489 (53.1%) were LD and 354 (72.4%) of them had AHD accounting for 38.5% of all new HIV cases. Table 1 shows baseline characteristics of the cohort of people with LD. Nearly half (49.5%) of people with LD and 68.4% of those with AHD had at least one WHO 3–4 event during the first year of follow‐up. Of the 242 participants who had WHO 3–4 events, 65.3% (158) had one event, 26% (63) had two and 8.7% (21) had three or more. Figure 1 shows the frequency of individual events. During the first year of follow‐up, 35.8% of people with LD and 49.2% of the AHD subgroup had at least one HIV‐related hospitalization. First year overall mortality and HIV‐related mortality were 9.2% (45) and 6.7% (33) for all the LD cohort, and 12.7% (45) and 9.3% (33) for the AHD subgroup.

**P183: Table 1:** Baseline characteristics of the cohort of people with LD (*n* = 489)

	Total LD	AHD (*n* = 354)	LD without AHD (*n* = 135)
Gender			
Cis men, *n* (%)	354 (72.4)	252 (71.2)	102 (75.5)
Cis women, *n* (%)	105 (21.5)	76 (21.5)	29 (21.5)
Trans men, *n* (%)	1 (0.2)	1 (0.3)	0 (0)
Trans women, *n* (%)	29 (5.9)	25 (7)	4 (3)
Age (years), median (Q1−Q3)	35 (28−45)	37 (29−46.75)	31 (25−40)
Plasma HIV RNA viral load (log10), median (Q1−Q3)	5.03 (4.41−5.63)	5.21 (4.67−5.68)	4.61 (4.03−5.13)
CD4 count (cell/µl), median (Q1−Q3)	138 (52.25−253.5)	94 (32.87−161.5)	293 (255.25−320)

**Conclusions**: In our cohort, the majority of new HIV cases are LD and most of them present with AHD, exceeding the proportions in national reports. Excess HIV‐related and overall mortality is observed among those PLHIV with AHD during the first year of follow‐up. These results highlight the importance of reinforcing strategies for improving timely access to HIV diagnosis and for the appropriate management of AHD and its complications.

**P183: Figure 1:**
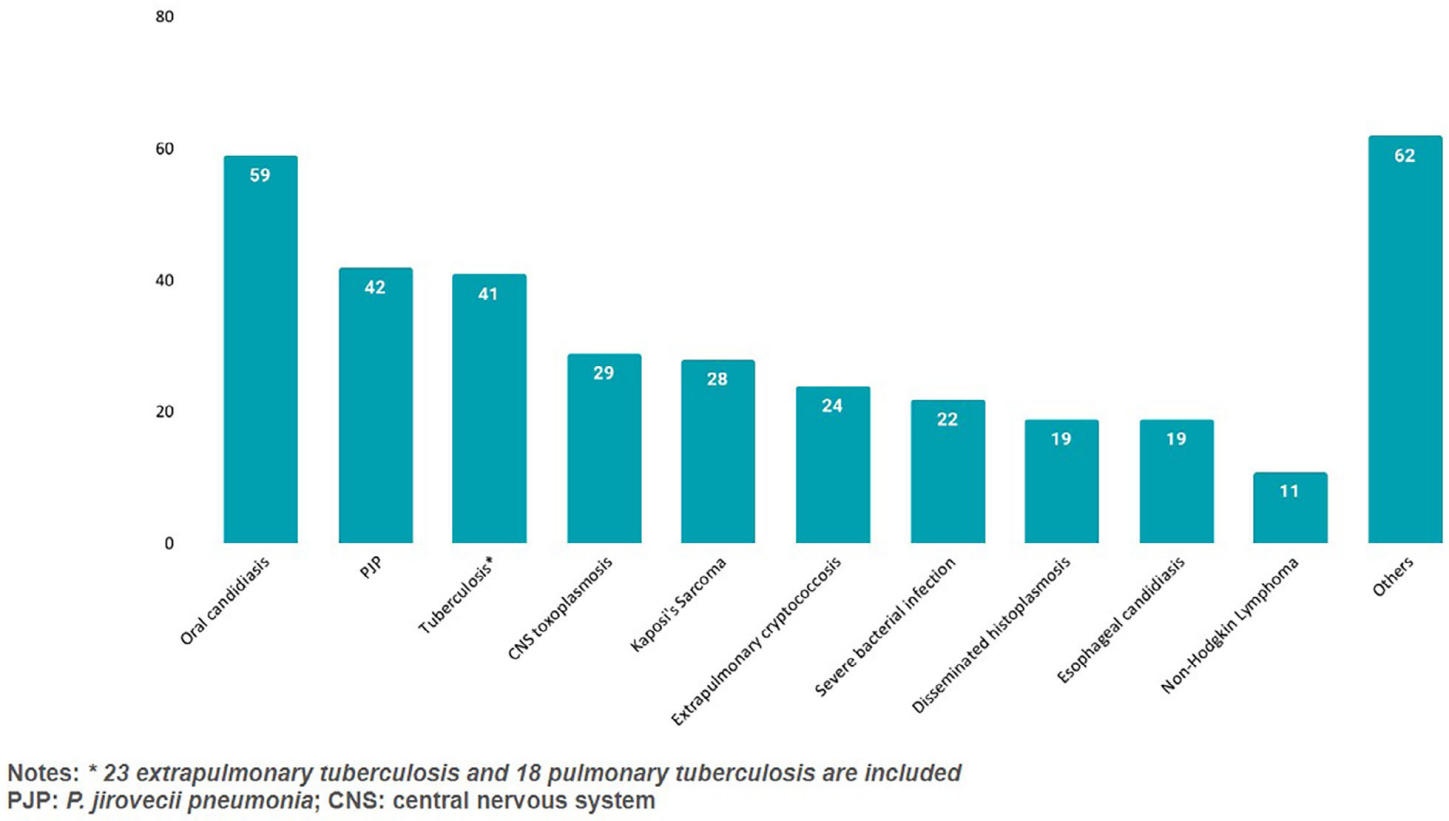
Frequency of WHO 3–4 clinical events (*n* = 356).

### P184 Assessing the burden of advanced HIV disease among newly diagnosed individuals in three high‐volume facilities in Kampala, Uganda: a retrospective study (2021−2023)

Julia Linda Ainemukama ^1^, Wycliff Sekayi^1^, Patrick Kazooba^1^, Gertrude Namale^1^, Emmanuel Sendaula^2^, Josephine Kaleebi^3^

^1^Medical, Reach Out Mbuya Community Health Initiative, Kampala, Uganda. ^2^Monitoring and Evaluation, Reach Out Mbuya Community Health Initiative, Kampala, Uganda. ^3^Programs, Reach Out Mbuya Community Health Initiative, Kampala, Uganda

**Background**: Despite global strategies for early detection and treatment of HIV, a significant proportion of newly identified people living with HIV (PLHIV) present with advanced HIV disease (AHD). In Uganda, approximately 30% of newly identified PLHIV present with AHD. This study examines the burden of AHD and its correlates among newly diagnosed PLHIV at three health facilities in Uganda.

**Methods**: We abstracted data from electronic medical records and registers on patients aged 15 years and older, newly diagnosed with HIV from January 2021 to December 2023 in three health centres in Kampala City. AHD was defined as CD4 cell count of <200 cells/ml and/or a World Health Organization (WHO) stage 3 or 4 event. Age, gender, marital status, WHO clinical stage, tuberculosis (TB) status, CD4 count, risk categories, cryptococcal antigen (CRAG) status, nutritional status and weight categories were described and analysed for crude association with AHD. A multivariate logistic regression model was built to identify independent predictors of AHD.

**Results**: Among the 826 clients (median age 31.5 years, IQR 26–39), majority (61.38%) were female. Most (66.22%) were in WHO clinical stage 1, 12.83% had TB and 32.83% had CD4 cells <200 cells/ml. The overall prevalence of AHD was 36.68% (95% CI 33.46–40.03). In multivariate analyses, male gender was associated with AHD (aOR 1.73, 95% CI 1.22–2.45, *p* = 0.002). Compared to age group 15–25 years, the odds of AHD increased with age groups; 26–35 years (aOR 1.91, 95% CI 1.18–3.07, *p* = 0.008), 36–45 years (aOR 2.57, 95% CI 1.48–4.46, *p* = 0.001) and 46–55 years (aOR 3.01, 95% CI 1.47–6.16, *p* = 0.003). In comparison to never‐married clients, higher odds of AHD were noted among divorced/separated (aOR 1.69, 95% CI 1.15–2.50, *p* = 0.008) and widowed individuals (aOR 2.74, 95% CI 1.16–6.51, *p* = 0.022). Moderate malnutrition (aOR 4.11, 95% CI 2.37–7.13, *p* < 0.001) and severe malnutrition (aOR 14.73, 95% CI 3.19–68.01, *p* = 0.001) significantly increased the odds of AHD compared to a normal nutritional status.

**Conclusions**: The prevalence of AHD among newly diagnosed HIV patients in Kampala was higher than the national average. This highlights the need for interventions targeting categories of gender, age, marital status and nutritional status among late presenters.

### P185 Late HIV diagnosis in low‐ and middle‐income countries (LMIC): opportunities and challenges ahead

Victoria Pinto, Ana Rodriguez, José Ángel Enrique Barletta, Julián Vega, Violeta Zulema Ortiz, Maria José Rolón

Infectious Diseases, Hospital General de Agudos, Ciudad Autónoma de Buenos Aires, Argentina

**Background**: Despite the worldwide decrease in new HIV diagnoses in the last decade, the proportion of people first presenting with late HIV diagnosis (LD) and advanced HIV‐related disease (AHD) remains stable. In Argentina, 43.8% of the new HIV cases in 2021–2022 were LD; and by 2017, 67.8% of PLHIV on ART were virally suppressed.

**Materials and methods**: This retrospective, single‐centre cohort study was aimed to characterize a cohort of PLHIV with LD; as well as to describe trends in the frequency of LD and AHD, and to analyse the HIV continuum of care during the first year of follow‐up. Adults with new HIV diagnoses between 2016 and 2022 were included, and followed for 1 year. Data was collected from medical records and surveillance systems. LD was defined as CD4 <350 and/or World Health Organization (WHO) 3–4 events; and AHD as CD4 <200 and/or WHO 3–4 events. Continuum of care outcomes were analysed as per Pan American Health Organization (PAHO) Monitoring Framework (2014).

**Results**: A total of 920 new HIV diagnoses were made during the study period; 489 (53.1%) were LD and 354 (72.4%) of them had AHD accounting for 38.5% of all new HIV cases. Among those with LD, median age was 35 years (Q1–Q3 28–45), 354 (72.4%) were cis men, 105 (21.5%) cis women, 29 (5.9%) trans women and one (0.2%) trans man. Median CD4 count was 138 cells/µl (Q1–Q3 52.25–253.5). For the continuum of care analysis, 66 patients transferred to other centres and 45 deceased were excluded. Figure 1 shows cascade analysis and trends of LD and AHD during the study period. The ART initiation analysis included 423 persons, 95% initiated ART during the first year and 61% of those eligible for immediate initiation did so within the first 7 days.

**Conclusions**: In our cohort, the proportion of LD and AHD remained stable during the study period. These results are similar to other published series. Proportion of viral suppression among those on ART exceeded the national level for PLHIV. These results highlight the importance of reinforcing strategies to promote timely HIV diagnosis, and the implementation of differentiated services to improve cascade outcomes among PLHIV with LD.

**P185: Figure 1:**
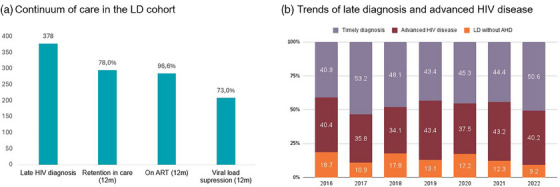
HIV‐related disease.

## Clinical management considerations—People who inject drugs (PWID)

### P186 Exploring potential interactions between intramuscular cabotegravir (CAB)/rilpivirine (RPV) and methadone: a case report

Pierluigi Francesco Salvo ^1^, Gianmaria Baldin^2^, Valentina Iannone^1^, Elena Visconti^2^, Simona Di Giambenedetto^1^

^1^Dipartimento di Sicurezza e Bioetica, Università Cattolica del Sacro Cuore, Rome, Italy. ^2^Dipartimento di Scienze Mediche e Chirurgiche, Fondazione Policlinico Universitario A. Gemelli IRCCS, Rome, Italy

ART may impact the plasmatic levels of methadone in PWH who are receiving methadone treatment. Methadone is metabolized by enzymes CYP2B6 and CYP3A4. Existing literature indicates that the concurrent use of oral RPV and methadone does not affect the maximum concentration, area under the curve (AUC) or minimum concentration of RPV, but reduces the AUC of methadone, even though no alterations in dosage are necessary when initiating the concurrent use of methadone and RPV. Clinical monitoring may be advisable, as methadone maintenance therapy might require adjustments. This is a case of a 64‐year‐old female living with HIV for 39 years, with history of HCV infection and drug addiction under methadone treatment. She has consistently shown low levels of adherence to ART, leading us physicians to propose to the patient a switch from 3TC + DRV/c to long‐acting CAB/RPV, which the patient accepted. At the latest follow‐up visit prior to the switch, she had 930 CD4/mmc and non‐detectable HIV‐RNA. No genotypic‐resistance testing was available at the time of switch. The first two doses were injected in November 2023. Ten days after, the patient began to exhibit withdrawal symptoms, prompting her to seek assistance from Addiction Services (AS). She reported absence of such symptoms in recent years, indicating that they had been completely controlled with 60 mg/day of methadone. This crisis was managed by increasing the dosage to 80 mg/day. Suspecting a potential interaction, a switch to oral therapy was performed to TAF/FTC/BIC. Over the following weeks, in accordance with her AS, the patient managed to reduce the daily dosage of methadone to 60 mg/day. After 4 weeks, she again reported adherence issues, expressing a desire to return to intramuscular therapy. A new administration of CAB/RPV was performed in March 2024, resulting in the recurrence of withdrawal symptoms, necessitating the patient to seek assistance from AS. Currently, the patient is again tapering off the methadone dosage, in anticipation of transitioning back to oral therapy. PWID are generally individuals with adherence issues to antiretroviral therapy, where long‐acting therapy can certainly be helpful. However, further studies are needed regarding the potential interaction between methadone and ART.

### P187 The community pop‐up clinic (CPC): HIV treatment in vulnerable population of people who use drugs

David Truong, Shana Yi, Christina Wiesmann, Brian Conway

Infectious Diseases, Vancouver Infectious Diseases Center, Vancouver, Canada

**Background**: To address the HIV pandemic, a concerted effort is needed to include vulnerable inner‐city residents, many of whom are actively using drugs, disengaged from care, and facing issues such as housing insecurity and active untreated addiction. We have evaluated a new model of HIV care using our community pop‐up clinic (CPC) as a strategy to identify HIV‐infected individuals to engage them in care and successfully engage and maintain them on antiretroviral therapy.

**Methods**: Weekly events are conducted at places of residence in Vancouver's inner city. Point‐of‐care testing for HCV and HIV are completed (with phlebotomy performed on site), along with ascertainment of prior HCV or HIV infection status. All individuals in whom this is indicated are then offered access to antiretroviral therapy delivered within a multidisciplinary programme with adherence support.

**Results**: From 01/21 to 11/23, we conducted 125 CPCs (3.5 events/month) evaluating 2111 individuals, 68 (3.2%) of whom tested positive for HIV antibodies, all previously diagnosed with HIV infection and lost to follow‐up. Of the 68, 45 (66.2%) showed active HCV co‐infection. Among HIV‐infected subjects, we note median age 50 (25–66) years, 24 (35.3%) female, 11 (16.2%) indigenous, and 26 (38.3%) with unstable housing, 28 (41.2%) experiencing recent incarceration, and 26 (38.2%) were active drug users, 25 of whom had a significant opioid overdose in the previous 6 months. Of 68, 45 (66.2%) engaged in long‐term care at our centre and continued with antiretroviral therapy.

**Conclusions**: Although we have made significant progress in the control of the HIV pandemic in British Columbia and across Canada, many inner‐city residents have disengaged from care and discontinued antiretroviral therapy. To control disease progression and transmission in this priority population, there is an urgent need to develop and evaluate interventions such as our CPC programme to optimize our approach to the diagnosis and treatment of HIV infection in Canada.

### P188 Switch to bictegravir/emtricitabine/tenofovir alafenamide (B/F/TAF) among vulnerable HIV‐infected individuals: evidence for long‐term efficacy

David Truong, Shana Yi, Christina Wiesmann, Brian Conway

Infectious Diseases, Vancouver Infectious Diseases Center, Vancouver, Canada

**Background**: Single tablet regimens (STRs) are associated with higher rates of sustained virological suppression and patient satisfaction. This is true of STRs including unboosted integrase strand transfer inhibitors with an increased barrier to resistance, tolerability and fewer drug interactions. This is beneficial for marginalized populations, with challenges of adherence and lower tolerance for side effects. We have previously demonstrated sustained virological suppression over 18 months among 41/43 living with HIV, active injection drug users following a switch of prior ARV therapy to the STR B/F/TAF. We sought to evaluate whether this benefit would be maintained over an additional 24 months of follow‐up.

**Methods: **The inception cohort consisted of 43 individuals who were followed up after having received B/F/TAF for 18 months. They remained enrolled in a multi‐disciplinary programme, with B/F/TAF provided with enhanced adherence support, allowing daily observed therapy. The end point of analysis was the rate of virological suppression after an additional 24 months of follow‐up, for a total of 42 months after initiating B/F/TAF therapy.

**Results**: Forty‐three subjects were included in this analysis: median age 54 (34–66) years, 11.1% female, 20% indigenous, 37.8% men who have sex with men and all were active drug users, with 91.1% being fentanyl users. At 18 months of follow‐up, we noted median CD4 count 612 cells/mm^3^. All 43 remained on B/F/TAF for the 24 months of follow‐up, with no long‐term disengagement. Forty‐one of 43 had maximal virological suppression, including both participants with detectable HIV RNA at month 18. Two cases of detectable HIV RNA (1520 and 3000 copies/ml) were documented at month 42. In both cases, virological suppression was achieved after the resumption of B/F/TAF.

**Conclusions**: Among a group living with HIV injection drug users experiencing transient viraemia, switching to B/F/TAF remains effective in the long term. Its efficacy and tolerability make it a particularly useful therapeutic option in this vulnerable population.

## Clinical management considerations—Transgender people

### P189 Transforming HIV care: intramuscular bimonthly cabotegravir and rilpivirine for transgender people with HIV in Spain (RELATIVITY cohort)

Alberto Díaz de Santiago ^1^, Pablo Ryan‐Murua^2^, María José Crusells‐Canales^3^, Luis Buzon Martin^4^, Patricia Martín‐Rico^5^, Otilia Bisbal‐Pardo^6^, Víctor Arenas‐García^7^, Alfonso Cabello‐Úbeda^8^, Miguel Egido‐Murciano^9^, Roberto Pedrero‐Tomé^10^

^1^HIV‐AIDS Medicine (Internal Medicine), Puerta de Hierro University Hospital, Madrid, Spain. ^2^Internal Medicine, Hospital Universitario Infanta Leonor, Madrid, Spain. ^3^Internal Medicine, Hospital Clínico Universitario Lozano Blesa, Zaragoza, Spain. ^4^Internal Medicine, Hospital de Burgos, Burgos, Spain. ^5^Internal Medicine, Hospital de Denia Marina Salud, Alicante, Spain. ^6^Internal Medicine, Hospital Universitario 12 de Octubre, Madrid, Spain. ^7^Internal Medicine, Hospital Universitario de Cabueñes, Asturias, Spain. ^8^Internal Medicine, Fundación Jiménez Díaz, Madrid, Spain. ^9^Internal Medicine, Hospital Universitario San Jorge, Huesca, Spain. ^10^Internal Medicine, Fundación para la Investigación e Innovación Biomédica del Hospital Infanta Leonor, Madrid, Spain

**Background**: The management of HIV in transgender individuals presents unique challenges, influenced by demographic characteristics, social disadvantages and less accessibility. This study forms part of the RELATIVITY cohort (with more than 1300 PHIV receiving intramuscular cabotegravir and rilpivirine), focusing exclusively on transgender individuals to descriptively analyse their clinical outcomes in Spain.

**Materials and methods**: A multicentre non‐controlled ambispective study was conducted, including HIV‐1 positive virally suppressed patients switching to long acting (LA) intramuscular (IM) CAB+RPV from 37 hospitals in Spain. This cohort comprised eight transgender individuals (0.6%) treated across seven hospitals. Data collected included demographic details (sex, nationality), clinical parameters (baseline BMI, CD4/CD8 ratios, viral load, liver and kidney function tests, lipid profile, glucose) and treatment specifics (type of antiretroviral therapy [ART], previous virological failures and blips, and treatment switches).

**Results**: The cohort predominantly consisted of transgender females (87.5%), with a greater number of Latin American (62.5%). Median age was 49.5 years, with a baseline BMI of 22.5 kg/m^2^. Median viral load at HIV diagnosis was 3125 copies/ml, with a median nadir CD4T of 525 cells/mm^3^. Patients had a median time of 13 months from diagnosis to the initiation of first ART, 10 years from first ART to CAB+RPV regimen, and 66 months of sustained undetectable viral loads before switching to study regimen. 37.5% were vaccinated against HBV, 25% showed past infection (with no occult infection) and 37.5% had no available data. Dorso‐gluteal route was the preferred (62.5%), and standard intramuscular needle was used in 100%. One (12.5%) had history of previous virological failure, and 75% showed blips before switching. DTG+RPV was previous oral ART regimen in 50%. All participants maintained undetectable viral loads throughout follow‐up (5.8 months), with no discontinuation of treatment or switches to oral regimens. The baseline and follow‐up CD4/CD8 ratios revealed a median increase from 1.2 to 1.3 (an absolute CD4 T cells from 798 to 881/mm^3^). No hormonal treatment data was available.

**Conclusions**: This descriptive analysis highlights successful ART management of transgender people with HIV using CAB+RPV LA IM regimen. Further research is needed to understand broader treatment dynamics and outcomes in diverse transgender populations.

## Clinical management considerations—Adolescents

### P190 Low burden of clinically relevant anaemia and thrombocytopenia among adolescents living with HIV receiving tenofovir/lamivudine plus dolutegravir: the CIPHER‐ADOLA study in Cameroon

Nadine Fainguem Nguendjoung ^1^, Yagai Bouba^2^, Rachel Kamgaing^1^, Davy Hyacinthe Gouissi Anguechia^3^, Aude Christelle Ka'e^3^, Alex Durand Nka^3^, Annie Nga Motaze^4^, Francis Ateba^5^, Alice Ketchaji^6^, Nelly Kamgaing^7^, Michel Carlos Tommo Tchouaket^1^, Rogers Ajeh^8^, Calixte Ida Penda^9^, Vittorio Colizzi^10^, Paul Koki Ndombo^11^, Alexis Ndjolo^3^, Carlo‐Federico Perno^12^, Samuel Martin Sosso^1^, Joseph Fokam^13^

^1^Medical Analysis Laboratory, Chantal Biya International Reference Centre for Research on the Prevention and Management of HIV/AIDS (CIRCB), Yaounde, Cameroon. ^2^Health Sciences, Saint Camillus International University of Health Sciences, Rome, Italy. ^3^Virology, Chantal Biya International Reference Centre for Research on the Prevention and Management of HIV/AIDS (CIRCB), Yaounde, Cameroon. ^4^Paediatrics, Essos Hospital Centre, Yaounde, Cameroon. ^5^HIV/AIDS/TB Unit, Mother and Child Centre, Chantal Biya Foundation, Yaounde, Cameroon. ^6^HIV/AIDS, Department of Disease, Epidemics and Pandemic Control, Ministry of Public Health, Yaounde, Cameroon. ^7^Paediatrics, Faculty of Medicine and Health Sciences, University of Yaounde I, Yaounde, Cameroon. ^8^HIV/AIDS, TB and Malaria UNIT, HIV, Tuberculosis and Malaria Global Funds Subvention Coordination Unit, Ministry of Public Health, Yaounde, Cameroon. ^9^Paediatrics, Douala General Hospital, Douala, Cameroon. ^10^Virology, Evangelic University of Bandjoun, Bandjoun, Cameroon. ^11^Paediatrics, Mother and Child Centre, Chantal Biya Foundation, Yaounde, Cameroon. ^12^Virology, Bambino Gesu Pediatric Hospital, Rome, Italy. ^13^Virology, Central Technical Group, National AIDS Control Committee, Yaounde, Cameroon

**Background**: An efficient transition to tenofovir/lamivudine+dolutegravir (TLD) requires pharmacovigilance related to haematological disorders among adolescents living with HIV (ADLHIV). We evaluated the rate of anaemia and thrombocytopenia among ADLHIV receiving TLD in the Cameroonian context.

**Methods**:  A cross‐sectional and multicentre study was conducted among ADLHIV aged 10–19 years receiving TLD in the CIPHER‐ADOLA cohort in Cameroon. Socio‐demographic and clinical data were collected; whole‐blood was collected to perform full blood count, viral load (VL [copies/ml]) and CD4‐count [cells/mm^3^]. Predictors of anaemia (haemoglobin [HB] level ≤11.5 g/dl) and thrombocytopenia (platelets count <150,000/µl) were assessed using multivariate logistic regression.

**Results**: Of the 252 ADLHIV (males: 50.8%; median ⦋IQR⦌ age, time on ART and TLD duration: 15 ⦋13–17⦌, 10 [6–13] years and 26 [12–33] months, respectively), 7.2% were underweight, 15.5% lived in rural settings; 71.4%, 13.1% and 15.5% had a VL <50, 50–999 and ≥1000, respectively. Overall, median [IQR⦌ HB level was 12.2 ⦋11.3–13.1⦌ g/dl; 79 (31.3%) were anaemic (severe: 0.4% [*N* = 1]; moderate: 7.1% [*N* = 18]; mild: 23.8% [*N* = 60]). Of these, 47 (59.5%) had microcytic anaemia, with 61.7% (*N* = 29) microcytic hypochromic and 38.3% (*N* = 18) microcytic normochromic anaemia. Anaemia rate was higher in females (43.5%) versus males (19.5%), *p* < 0.001; rural (47.7%) versus urban settings (28.2%), *p* = 0.011; primary (42.6%) versus secondary school (26.6%), *p* = 0.033. Compared to non‐anaemic, anaemic group had a significantly lower median ART‐duration (9 [9–12] vs. 11 [7–14] years, *p* = 0.008) and CD4 count (578 [391–814] vs. 698 [523–924], *p* = 0.006). In multivariate analysis, female sex (aOR [95% CI] 3.165 [1.743–5.748]), living in rural settings (2.178 [0.995–4.769]) and VL ≥1000 (3.135 [1.275–7.710]) independently predicted anaemia. Globally, 6.7% (*n* = 17) were thrombocytopenic (<50/µl: *N* = 2), with higher proportion in males (9.4%) versus females (4.0%), *p* = 0.091; CD4 <500 (12.7%) versus CD4 ≥500 (4.4%), *p* = 0.019; and increased with increasing VL levels (<50 [3.3%], 50–999 [15.2%] and ≥1000 [15.4%], *p* = 0.003). In multivariate analysis, VL 50–999 (aOR [95% CI] 5.069 [1.439–17.859]) versus VL <50 independently predicted thrombocytopenia.

**Conclusions**: ADLHIV receiving TLD experience a low burden of moderate/severe anaemia and thrombocytopenia, with vulnerability driven by female, living in rural settings and poor ART response; coupled to thrombocytopenia, driven by poor ART response. Thus, while scaling‐up transition to TLD among ADLHIV, monitoring those at risk of haematological disorders would secure long‐term benefits and survival towards the elimination of paediatric AIDS.

### P191 Baseline HIV genotyping in Saudi Arabian population, a multicentre, cross‐sectional study

Roaa Alosaimi ^1^, Batool Ali^1^, Moayad Alqurashi^2^, Reem Almutairi^3^, Ali Alsaeed^4^, Meqbel Alshelawi^5^, Abdullah Alkhalaf^6^, Abdullah Alsubaie^5^, Layla Faqih^7^

^1^Department of Adult Infectious Diseases, East Jeddah Hospital, Jeddah, Saudi Arabia. ^2^Division of Adult Infectious Diseases, Department of Medicine, King Fahad Armed Forces Hospital, Jeddah, Saudi Arabia. ^3^Pharmaceutical Care Department, King Fahad Armed Forces Hospital, Jeddah, Saudi Arabia. ^4^Department of Infectious Diseases, Dammam Medical Complex of the Eastern Health Cluster, Dammam, Saudi Arabia. ^5^Department of Medicine, East Jeddah Hospital, Jeddah, Saudi Arabia. ^6^Department of Medicine, Dammam Medical Complex of the Eastern Health Cluster, Dammam, Saudi Arabia. ^7^Department of Clinical Laboratory Sciences, King Saud University, Riyadh, Saudi Arabia

**Background**: Estimated number of people living with HIV (PLWH) in Saudi Arabia is around 20,539 persons. Local baseline genotyping studies are limited to a single centre with low sample size, limited age range and geographical distribution warranting a large multi‐centre study to further understand the situation.

**Methods**: A multi‐centre, retrospective cross‐sectional study was conducted over three centres from two distinct regions of Saudi Arabia. Adults (≥18 years old) who were antiretroviral treatment‐naïve were included regardless of gender, ethnicity, co‐morbidities or pregnancy status, while those who were paediatrics, treatment‐experienced, PrEP users or elite controllers were excluded. Although estimated significant sample size needed was around 385, all possible PLWH were included. Data were collected through a unified electronic data sheet form separately for each centre and combined later for data analysis using Microsoft Excel.

**Results**: We included 614 PLWH, majority (72.3%) from the western region with male predominance. Although the majority had no subtype mentioned (21.3%) in their reports, the most common detected subtype was C at 19.7% (121/614) followed by CRF02_AG at 15.6% (96/614) then G at 12.7% (78/614). The most common drug‐resistance mutations (DRMs) among the NRTI were S68G/N/S at 9.6% followed by M184I/M/V at 1.6%, while in the NNRTI class, V179A/D/E/I/T/V at 13.8% followed by A98A/G/R/S at 9.6%. Among the PI class, V82F/I/L/V (23.9%) and L89I/L/M/V (80.3%) were the most common major and accessory DRM, respectively. Finally, in the INSTI class, E138D/E/K followed by G140A were the major DRMs detected at 10.6% and 0.3% respectively, while L74I/L/M/V and G163A/D/E/G/M/N/Q/R/S/V were the most common accessory DRMs at 10.6% and 8.1%, respectively.

**Conclusions**: This study concurs with the previous reports of the most common subtype in Saudi Arabia but differs in the distribution of other subtypes. While the majority of detected DRMs are known to cause resistance to some antiretrovirals, none did significantly affect the first‐line regimens used currently (INSTI‐based regimen). Some mutations are selected by the prior use of certain antiretroviral, which indicate possible local transmission among patients who were either not compliant to their medications or experiencing virological failure of therapy.

### P192 Increasing understanding in transitioning into adult care for adolescents living with HIV: effect of online education on physician knowledge and confidence

Shanthi Voorn ^1^, Alessia Piazza^1^, Gill Adair^1^, Ann Chahroudi^2^, Caroline J. Foster^3^

^1^Global Education, Medscape LLC, London, UK. ^2^Department of Pediatrics, Emory University School of Medicine, Atlanta, GA, USA. ^3^Imperial College Healthcare NHS Trust, St Mary's Hospital, London, UK

**Background**: People living with HIV experience different clinical challenges at various life stages due to factors such as age. Physicians need to identify and tackle the challenges adolescents face when transitioning to adult HIV care. We assessed if online medical education could increase HIV and infectious disease (ID) physicians’ knowledge and confidence regarding these challenges and the tools for a smooth transition. As part of a larger curriculum, we developed an online Continuing Medical Education (CME) activity titled: “Transitioning Into Adult Care for Adolescents Living With HIV” to improve the knowledge of these challenges and the tools to overcome them.

**Methods**: ID/HIV specialists participated in an online CME activity (https://www.medscape.org/viewarticle/999385) consisting of a 30‐minute video discussion between two experts. Educational effect was assessed using a four‐question repeated pairs, pre‐/post‐assessment. A paired samples *t*‐test was conducted for significance testing on overall average number of correct responses and for confidence rating, and a McNemar's test was conducted at the question level (5% significance level). Cohen's *d* for paired samples estimated the effect size of the education (<0.20 modest, 0.20–0.49 small, 0.59–0.79 moderate, ≥0.80 large). The CME activity launched on 1/10/2024, and the data were collected through 3/23/2024.

**Results**: A total of 397 ID/HIV specialists participated in the activity, of whom 32 completed the pre‐ and post‐activity questions. Overall, 51% of ID/HIV specialists improved their knowledge of challenges that adolescents with HIV experience (*p* < 0.001) indicating a considerable effect of the education (Cohen's *d* = 0.91). The average percentage of correct responses rose from 24% to 51% for ID/HIV specialists’ pre‐activity to post‐activity. Fifty percent of ID/HIV specialists had a measurable improvement in confidence in their ability to optimally support people living with HIV when transitioning from paediatric to adult care.

**Conclusions**: This online CME activity significantly improved ID/HIV specialists’ knowledge of challenges faced by adolescents with HIV, such as ART adherence, stigma and additional morbidity burden. There were also significant gains in physicians’ confidence in supporting adolescents transitioning to adult care.

## Clinical management considerations—Paediatrics

### P193 HIV drug resistance among children and adolescents with viraemia in Lesotho and Tanzania: a secondary analysis of the GIVE MOVE trial

Christof Manuel Schönenberger ^1^, Kathrin Haenggi^1^, Isaac Kaumbuthu Ringera^1^, Ezekiel Luoga^2^, Moniek Bresser^3^, Buoang Mothobi^4^, Kuena Mokhele^4^, David Sando^5^, Brenda Simba^5^, Mamello Molatelle^6^, Lineo Thahane^7^, Dorcas Mnzava^8^, Robert Ndege^8^, Mosa Molapo Hlasoa^7^, Buntshi Paulin Kayembe^7^, Josephine Muhairwe^4^, Tracy Renée Glass^3^, Thomas Klimkait^9^, Maja Weisser^10^, Niklaus Daniel Labhardt^1^, Nadine Tschumi^1^, Jennifer Anne Brown^1^

^1^Division of Clinical Epidemiology, Department of Clinical Research, University Hospital Basel, Basel, Switzerland. ^2^Ifakara Health Institute, Ifakara, Tanzania. ^3^Department of Medicine, Swiss Tropical and Public Health Institute and University of Basel, Allschwil, Switzerland. ^4^Partnerships for Health, SolidarMed, Maseru, Lesotho. ^5^Management and Development for Health, Dar es Salaam, Tanzania. ^6^Seboche Mission Hospital, Seboche, Lesotho. ^7^Baylor College of Medicine Children's Foundation Lesotho, Maseru, Lesotho. ^8^Ifakara Health Institute, Ifakara, Tanzania. ^9^Molecular Virology, Department of Biomedicine, University of Basel, Basel, Switzerland. ^10^Division of Infectious Diseases, University Hospital Basel, Basel, Switzerland

**Background**: The GIVE MOVE trial [1] compared genotypic‐resistance testing (GRT) informed management of viraemia with usual care (no GRT) in children and adolescents with HIV in Lesotho and Tanzania. It found no significant difference in viral suppression at 9 months follow‐up between arms. Here, we report a pre‐planned secondary analysis on GRT data among viraemic participants.

**Materials and methods**: We included participants with available GRT at baseline, at 9 months or both. We report the number of drugs predicted to be active per participants’ current three‐drug ART regimen (defined as at most “potential low‐level resistance” according to Stanford database).

**Results**: Overall, 150 participants had 171 GRT available at baseline, at 9 months or both (Table 1). Among 120/150 (80%) with GRT data at baseline (see resistance mutations in Figure 1), 11/120 (9%), 32/120 (27%), 7/120 (6%) and 70/120 (58%) had zero, one, two and three drugs predicted to be active in their baseline ART regimen, respectively. At 9 months, 51/150 (34%) had successful GRT. Among them, 4/51 (8%), 9/51 (18%), 6/51 (14%) and 32/51 (63%) had zero, one, two and three drugs predicted to be active in their 9‐month ART regimen, respectively. Of the 50 participants with <3 active drugs in their regimen at baseline, at 9 months, 28/50 (56%) achieved viral resuppression to <400 copies/ml at 9 months (17/27 [63%] with a changed and 11/20 [55%] with an unchanged regimen). Nineteen of 50 (38%) had ongoing viraemia ≥400 copies/ml (10/27 [37%] with a changed, 9/20 [45%] with an unchanged regimen). Three (6%) were lost‐to‐follow‐up. At 9 months, 4/19 (21%) had viraemia without and 11/19 (58%) with relevant mutations. Four of 19 (21%) had no successful GRT.

**P193: Table 1:** Socio‐demographic and clinical participant characteristics

Number of included participants	150	
Age (median [IQR])	12.0 [5.4−16.1]	
Sex—female	88 (58.7%)	
Sex—male	62 (41.3%)	
Country—Lesotho	115 (76.7%)	
Country—Tanzania	35 (23.3%)	
	Baseline visit (*n* = 150)	9‐month visit (*n* = 150)
Current regimen—INSTI‐based^a^	72 (48.0%)	102 (70.3%)
Current regimen—PI‐based^a^	69 (46.0%)	42 (29.0%)
Current regimen—NNRTI‐based^a^	9 (6.0%)	1 (0.7%)
Viral load; <400 copies/ml	22 (14.7%)	74 (49.3%)
Viral load; 400−999 copies/ml	15 (10.0%)	10 (6.7%)
Viral load; 1000−99,999 copies/ml	72 (48.0%)	49 (32.7%)
Viral load; >100,000 copies/ml	15 (10.0%)	10 (6.7%)
Viral load; missing VL	26 (17.3%)	7 (4.7%)
GRT result; no viral load, no GRT	12 (8.0%)	2 (1.3%)
GRT result; VL <400 copies/ml, no GRT	12 (8.0%)	73 (48.7%)
GRT result; VL ≥400 copies/ml, no GRT	6 (4.0%)	19 (12.7%)
GRT result; lost to follow up	0 (0%)	5 (3.3%)
GRT result; GRT successful	120 (80.0%)	51(43.0%)
GRT result; GRT successful—three active drugs^b^	70 (58.3%)	32 (62.7%)
GRT result; GRT successful—two active drugs^b^	7 (5.8%)	6 (11.8%)
GRT result; GRT successful—one active drug^b^	32 (26.7%)	9 (17.6)
GRT result; GRT successful—zero active drug^b^	11 (9.2%)	4 (7.8%)
Susceptibility score (median [IQR])^c^, %	3.0 [1.5−3.0]	3.0 [1.75−3.0]

Abbreviations: GRT, genotypic‐resistance test; INSTI, integrase strand transfer inhibitor; IQR, interquartile range; NNRTI, non‐nucleoside reverse transcriptase inhibitor; PI, protease inhibitor; VL, viral load.

^a^Five lost‐to‐follow up without information on regimen at 9‐month.

^b^Out of people with successful GRT.

^c^We obtained resistance levels (high‐level resistance, intermediate resistance, low‐level resistance, potential low‐level resistance and susceptible) for each of the three drugs of the participant's ART regimen through the Stanford HIV drug resistance database. We translated these resistance levels into a numeric score (possible range 0.00–1.00). Lower values indicate lower susceptibility and, therefore, higher resistance. We calculated an overall score per regimen (possible range 0.00–3.00).

**Conclusions**: In most children and adolescents with viraemia while taking ART, treatment failure could not be explained by the presence of major drug‐resistance mutations. Resuppression with regimens that were not fully active was frequent, as was ongoing viraemia in regimens predicted to be fully active.

**P193: Figure 1:**
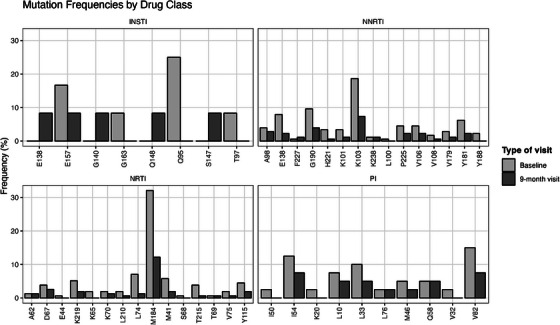
Mutation frequencies by drug class; stratified by type of visti. INSTI, integrase strand transfer inhibitor; NNRTI, non‐nucleoside reverse transcriptase inhibitor; PI, protease inhibitor.

**Reference**

1. Brown JA, Ringera IK, Luoga E, Bresser M, Mothobi B, Kabundi L, et al. Resistance‐informed versus empirical management of viraemia in children and adolescents with HIV in Lesotho and Tanzania (GIVE MOVE trial): a multisite, open‐label randomised controlled trial. Lancet Glob Health. 2024;12(8):e1312‐22.

### P194 Tolerability of lopinavir versus dolutegravir for children and adolescents living with HIV (LoDoCA): a prospective cohort study in Lesotho, Southern Africa

Jacob Blankenberger ^1^, Akash Devendra^2^, Meenakshi Bakaya^2^, Tristan Lee^1^, Teresa Steffy^2^, Thithili Makhesi^2^, Jennifer Belus^1^, Nadine Tschumi^1^, Nthuseng Marake‐Raleie^3^, Reto Huber^4^, Frédérique Chammartin^1^, Niklaus Labhardt^1^, Jennifer Brown^1^

^1^Division of Clinical Epidemiology, Department of Clinical Research, University Hospital Basel and University of Basel, Basel, Switzerland. ^2^Children's Foundation Lesotho, Baylor College of Medicine, Maseru, Lesotho. ^3^Ministry of Health, Government of Lesotho, Maseru, Lesotho. ^4^Child Development Center, University Children's Hospital Zurich and University of Zurich, Zurich, Switzerland

**Background**: Tolerability of antiretroviral therapy (ART) is essential for adherence and viral suppression. Dolutegravir (DTG)‐based ART has recently been rolled out as the preferred regimen across Africa. In Lesotho in southern Africa, children and adolescents previously taking ART containing ritonavir‐boosted lopinavir (LPV/r) were transitioned to DTG‐based ART in 2022–2023. Here, we investigate associated changes in treatment satisfaction and known side effects of LPV/r (notably gastrointestinal) and DTG (notably neuropsychiatric, including insomnia).

**Materials and methods**: This single‐centre prospective cohort study enrolled children and adolescents <18 years changing from LPV/r‐ to DTG‐based ART in the context of the programmatic DTG rollout in Lesotho. Participants fulfilling additional criteria (including age ≥6 years and last viral load <50 copies/ml) were eligible for sleep monitoring through actigraphy. Participants were enrolled 2 weeks before (with actigraphy) or at (without actigraphy) their regimen change and followed up for 4 weeks post regimen change. The co‐primary end points were: (i) self‐ or caregiver‐reported change in treatment satisfaction at 4 weeks, measured by the HIV Treatment Satisfaction Questionnaire change version (HIVTSQ‐c), and (ii) mean sleep duration in the 2 weeks before versus 2–4 weeks after the regimen change (among those with actigraphy). HIVTSQ‐c results were compared with “no change” by two‐sided Wilcoxon signed‐rank test. Sleep duration before versus after regimen change was compared by paired *t*‐test. Secondary end points included further questionnaires and sleep parameters at and 4 weeks after change to DTG. ClinicalTrials.gov: NCT05426421.

**Results**: Among 245 participants with transition and post‐4‐week data, 115 (46.9%) were female and median age was 11.1 [interquartile range 8.9–13.6] years. Sixty‐eight (27.7%) participants were included in the actigraphy analysis. Mean caregiver‐ and self‐reported HIVTSQ‐c outcomes were significantly greater than 0 (“no change”), indicating improved treatment satisfaction (Table 1). No significant changes were observed for the estimated length of the sleep window from sleep onset to waking up, or the duration of sleep within the sleep window (Table 1). Other tolerability and sleep parameters did not change meaningfully (Table 1).

**P194: Table 1:** Primary and selected secondary endpoints

Measure	Range	At transition	At 4 weeks	Difference	*p*‐value
Questionnaires		Median (IQR)	Median (IQR)	Mean (95% CI)	
Co‐primary					
HIVTSQ‐c, caregiver‐reported (participants <12 years) (*n* = 149)	−30, 30^a^	N/A	30.00 [30.00−30.00]	N/A	<0.001
HIVTSQ‐c, self‐reported (participants ≥12 years) (*n* = 92)	−16, 16^a^	N/A	16.00 [16.00−16.00]	N/A	<0.001
Secondary					
HIVTSQ‐s, caregiver‐reported (participants <12 years) (*n* = 144)	0−60^b^	60.00 [58.00−60.00]	60.00 [59.00−60.00]	0.10 (−0.51, 0.70)	−
HIVTSQ‐s, self‐reported (participants ≥12 years) (*n* = 89)	0−32^b^	31.00 [29.00−32.00]	32.00 [31.00−32.00]	0.75 (0.16−1.34)	−
CES‐DC self‐reported (participants ≥6 years) (*n* = 221)	0−60^c^	6.00 [3.00−11.00]	6.00 [3.00−9.00]	−1.37 (−2.24, −0.49)	−
GSRS‐PI, caregiver‐reported (participants <12 years) (*n* = 150)	13−78^c^	13.00 [13.00−14.75]	13.00 [13.00−13.00]	−0.59 (−1.26, 0.07)	−
GSRS‐PI, self‐reported (participants ≥12 years) (*n* = 92)	13−78^c^	13.00 [13.00−16.00]	13.00 [13.00−16.25]	0.42 (−0.96, 1.81)	−
Actigraphy (*n* = 68)		Mean (SD)	Mean (SD)	Mean (95% CI)	0.050
Co‐primary					
Estimated length of sleep window, in hours	N/A	8.96 (0.94)	9.13 (0.92)	0.17 (−0.02, 0.34)	−
Secondary					
Estimated duration of sleep in sleep window, in hours	N/A	7.39 (1.08)	7.38 (0.95)	−0.01 (−0.18, 0.17)	−
Estimated number of awakenings during sleep window	N/A	21.09 (4.51)	22.31 (4.21)	1.23 (0.25−2.2)	−

Abbreviations: CES‐DC, Center for Epidemiological Studies Depression Scale for Children [1]; CI, confidence interval; GSRS‐PI, Gastrointestinal Symptom Rating Scale modified to the characteristics of the protease inhibitors [2]; HIVTSQ‐c, HIV Treatment Satisfaction Questionnaire change version [3]; HIVTSQ‐s, HIV Treatment Satisfaction Questionnaire status version [3]; IQR, interquartile range.

^a^Positive scores indicate increased, negative scores indicate decreased treatment satisfaction.

^b^Higher scores indicate greater treatment satisfaction.

^c^Higher scores indicate higher number of severity of symptoms.

**Conclusions**: Treatment satisfaction improved after changing to DTG, with no other major changes in tolerability. These findings support the rollout of DTG‐based ART for paediatric HIV care.

**References**

1. Woodcock A, Bradley C. Validation of the revised 10‐item HIV Treatment Satisfaction Questionnaire status version and new change version. Value Health. 2006;9(5):320‐33.

2. Sáez de la Fuente J, Granja V, Escobar I, Collada de la Fuente E, Moreno V, Rubio R. Study of the gastrointestinal tolerance of a new tablet formulation of the lopinavir/ritonavir antiretroviral in HIV‐infected patients. J Acquir Immune Defic Syndr. 2009;50(3):294‐8.

3. Faulstich ME, Carey MP, Ruggiero L, Enyart P, Gresham F. Assessment of depression in childhood and adolescence: an evaluation of the Center for Epidemiological Studies Depression Scale for Children (CES‐DC). Am J Psychiatry. 1986;143(8):1024‐7.

### P195 Structured caregiver literacy sessions: a path to 100% viral load suppression among children living with HIV, Matata Hospital experience, Homa Bay County

Brian Boit

Comprehensive Care Clinic, Matata Hospital, Homabay, Kenya

**Background**: CLHIV lag behind in viral load suppression because they solemnly depend on their caregivers to give them antiretroviral. Caregivers have demonstrated significant capability to help in medication adherence for paediatrics once empowered. Viral load (VL) suppression is essential in reducing child morbidity and mortality and this requires collaboration between healthcare providers and caregivers. The viral load suppression among CLHIV has been at 63% which is below the Joint United Nations Programme on HIV/AIDS (UNAIDS) target of 95%.

**Methods**: Data was abstracted for CLHIV (<10 years old) with valid VL from the National AIDS and STIs Control Programme (NASCOP) website between October 2021 and April 2022. Out of the 31 CLHIV, 11 were low detectable level (LDL), six had between 50 and 199 copies/ml, four had between 200 and 999 copies/ml, six were above 1000 copies/ml and four were new to care hence not eligible for VL. We assessed the caregivers’ characteristics in terms of age, sex, HIV status, VL suppression, education level, change of caregivers and alcohol uptake. The caregivers were engaged in biweekly focused group discussion and peer to peer sessions targeting on disclosure, adherence and benefits of antiretroviral therapy (ART). Descriptive analysis was performed to determine the age, sex, HIV status and education levels. A linear regression analysis was performed to determine degree of association between the caregiver characteristics and VL suppression of the CLHIV (Figure 1).

**Results**: There was 100% VL suppression rate among CLHIV with 23 LDL and eight (50–199 copies). A linear regression analysis between the CLHIV VL, sex of caregiver, caregiver's HIV status, caregiver's VL and change of caregiver indicates a weak positive correlation between the child's low VL and the change of caregiver (0.187) and caregiver's VL (0.167), respectively.

**Conclusions**: There is a weak correlation between caregiver's education levels, sex, alcoholism and child's low VL. Change of caregiver and caregiver's low VL significantly contribute to a child's low VL. The differences in the means from a *t*‐test indicate that structured caregiver literacy session contributes to CLHIV VL suppression despite their dynamic characteristics.

**P195: Figure 1:**
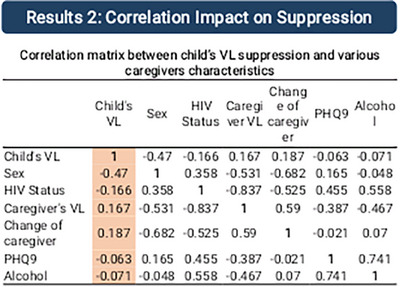
Correlation matrix between child's viral load suppression and caregiver characteristics.

## Clinical management considerations—Drug‐drug interactions

### P196 Dolutegravir‐based antiretroviral therapy does not reduce plasma levonorgestrel or medroxyprogesterone acetate concentrations among contraceptive users living with HIV compared with HIV‐negative controls

Rebecca Ryan ^1^, Aamirah Mussa^1^, Bame Bame^1^, Mbapi Bapabi^1^, Neo Moshashane^1^, Imogen Mechie^1^, Samuel Ensor^1^, Neo Ndlovu^1^, Shelby Chakona^1^, Selebaleng Simon^1^, Maipelo Tsuaneng^1^, Laura Else^2^, Alieu Amara^2^, Vinay Tatipamula^2^, Laura Dickinson^2^, Saye Khoo^2^, Chelsea Morroni^3^

^1^Botswana Sexual and Reproductive Health Initiative, Botswana Harvard Health Partnership, Gaborone, Botswana. ^2^Department of Pharmacology and Therapeutics, University of Liverpool, Liverpool, UK. ^3^Centre for Reproductive Health, University of Edinburgh, Edinburgh, UK

**Background**: Drug‐drug interactions between contraceptive methods and antiretroviral therapy (ART) increase the risk of unintended pregnancy [1,2]. Pharmacokinetic data evaluating drug‐drug interactions between dolutegravir and the levonorgestrel (LNG)‐implant or depot medroxyprogesterone acetate (DMPA)‐injection, are lacking. We evaluate drug‐drug interactions between dolutegravir‐based ART and the LNG‐implant or DMPA‐injection; investigating pharmacokinetic parameters, safety and tolerability.

**Materials and methods**: We conducted a phase IV parallel arm, non‐randomized pharmacokinetic study. Non‐pregnant women living with HIV or HIV‐negative, ART‐naïve women aged 18–45 years and living in Gaborone, Botswana, who chose to initiate either the LNG‐implant or DMPA‐injection, were included. Blood was collected for pharmacokinetic analysis before contraceptive initiation and then at defined 2‐ or 4‐weekly intervals up to 12 weeks (DMPA‐injection arm) or 24 weeks (LNG‐implant arm). Data was collected on safety, tolerability and method continuation, alongside pregnancy testing, at each study visit. The primary outcome was comparison of the area under the concentration‐time curve (AUC) for LNG over 24 weeks (LNG AUC_24weeks_, LNG‐implant arm) or MPA over 12 weeks (MPA AUC_12weeks_, DMPA‐injection arm) between women living with HIV on DTG‐based ART versus HIV‐negative, ART‐naïve controls.

**Results**: One hundred and forty women were enrolled; 70 initiated the LNG‐implant and 70 the DMPA‐injection (35 on DTG‐based ART and 35 HIV‐negative, ART‐naive in each arm). Enrolment characteristics were similar in all study groups. In the LNG‐implant arm, the median (range) LNG AUC_24weeks_ was 13,732 (6252–46,797) pg.week/ml and 10,157 (3922–36,311) pg.week/ml in women on dolutegravir‐based ART and HIV‐negative women, respectively; geometric mean ratio (GMR) 1.295, 95% confidence interval (95% CI) 0.998–1.681, *p* = 0.52. In the DMPA‐injection group, the median (range) MPA AUC_12weeks_ was 10,770 (4665–20,005) pg.week/ml and 10,212 (3018–18,122) pg.week/ml in women on DTG‐based ART and HIV‐negative women, respectively; GMR 1.094, 95% CI 0.909–1.315, *p* = 0.336 (Figure 1). Both contraceptive methods were well‐tolerated, with no unintended pregnancies nor serious adverse events in any arm.

**P196: Figure 1:**
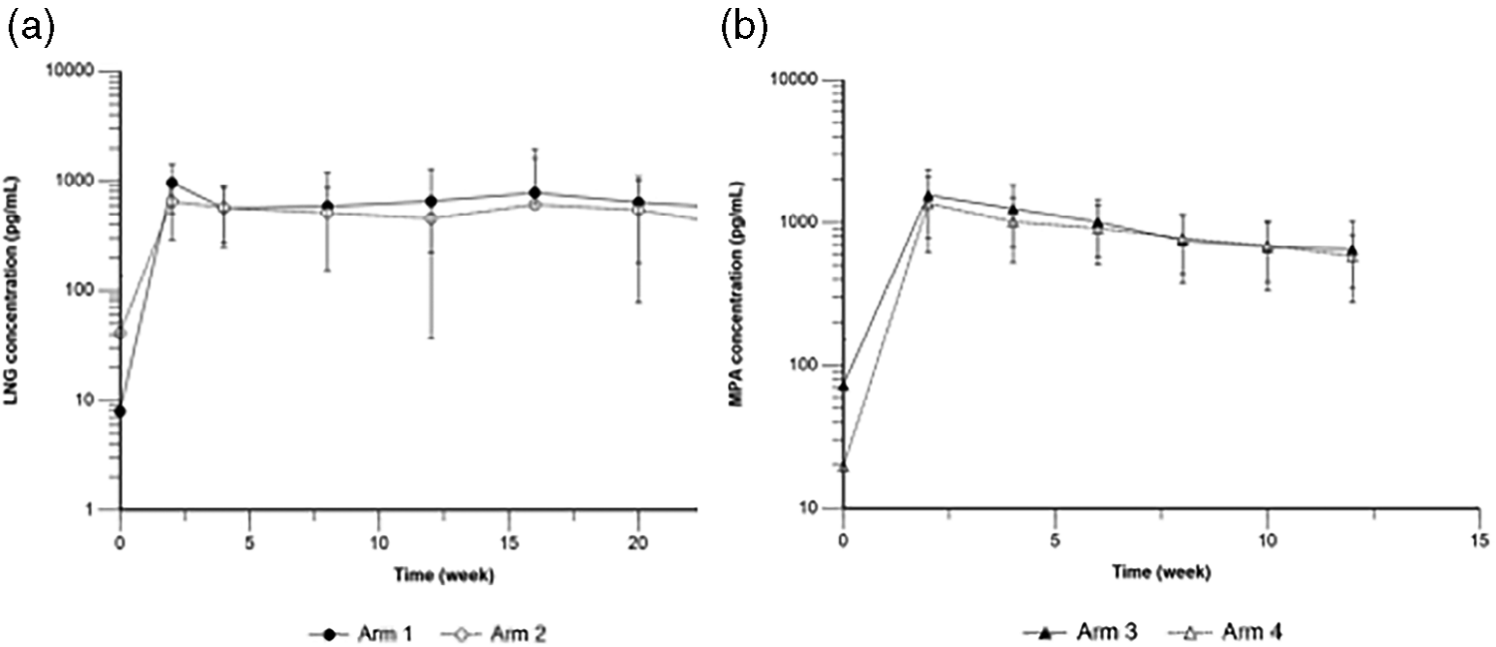
Concentration‐time profiles for levonorgestrel and medroxyprogesterone acetate on a log‐linear scale for women living with HIV on dolutegravir‐based antiretroviral therapy and HIV‐negative, ART‐naive women. (a) Mean ± SD levonorgestrel (LNG) concentration‐time profiles on a log‐linear scale in women who have initiated the LNG sub‐dermal implant (Arm 1: women living with HIV on dolutegravir [DTG]‐based therapy; Arm 2: HIV‐negative, ART‐naïve women); (b) mean ± SD medroxyprogesterone acetate (MPA) concentration‐time profiles on a log‐linear scale in women who have initiated the depot MPA injection (Arm 3: women living with HIV on dolutegravir [DTG]‐based therapy; Arm 4: HIV‐negative, ART‐naïve women).

**Conclusions**: Dolutegravir‐based ART does not reduce plasma LNG or MPA concentrations in women living with HIV compared to HIV‐negative, ART‐naïve controls. Both the LNG‐implant and DMPA injection are highly effective, safe and well‐tolerated contraceptive options for women living with HIV on dolutegravir‐based ART.

**References**

1. Patel RC, Amorim G, Jakait B, Shepherd BE, Mocello AR, Musick B, et al. Pregnancies among women living with HIV using contraceptives and antiretroviral therapy in western Kenya: a retrospective, cohort study. BMC Med. 2021;19(1):178.

2. Okoboi S, Eunice A, Oceng R, Etukoit B. Correlation between co‐therapy of efavirenz‐based ART among HIV‐positive women on hormonal contraceptive implants at TASO Tororo‐Uganda: a retrospective review. J AIDS Clin Res. 2018;9(2):1000759.

### P197 Effect of fluconazole on the pharmacokinetics of ainuovirine in healthy adults

Jianfei Huang^1^, Yan Lei^2^, Weiping Cai^1^, Yu Meng^1^, Lei Xiao^2^, Yi Zhao^2^, Weitong Lin^2^, Yaozu He^1^, Hong Qin ^3^, Yufang Zheng^3^, Chunyun Zhang^3^, Kaipeng Huang^2^, Linghua Li^1^

^1^Guangzhou Medical Research Institute of Infectious Diseases, Guangzhou Eighth People's Hospital, Guangzhou Medical University, Guangzhou, China. ^2^Phase I Clinical Trial Center, Guangzhou Eighth People's Hospital, Guangzhou Medical University, Guangzhou, China. ^3^Clinical Research & Development, Jiangsu Aidea Pharmaceutical Co., Ltd, Yangzhou, China

**Background**: Ainuovirine (ACC007) is a next‐generation non‐nucleoside reverse transcriptase inhibitor (NNRTI) for combinational therapy for people living with HIV, which is primarily metabolized by CYP2C19. The aim of this phase I study was to assess the drug‐drug interactions (DDIs) and safety of ainuovirine when co‐administered with fluconazole, a strong CYP2C19 inhibitor in healthy adults. A physiologically based pharmacokinetics (PBPK) model was also developed for dose adjustment prediction of ainuovirine when combined with fluconazole.

**Methods**: This was a single‐centre, open‐label, parallel‐group, sequential design, two‐period study in healthy adults (aged 20–45 years). A total of 36 healthy adults were randomly allocated into two groups. In group A, 18 healthy adults received oral ainuovirine (150 mg) once daily in Period 1 (days 1–7), followed by co‐administration with oral fluconazole (200 mg) once daily in Period 2 (days 8–14). In group B, 18 healthy adults received oral fluconazole (200 mg) once daily in Period 1 (days 1–7), followed by co‐administration with oral ainuovirine (150 mg) once daily in Period 2 (days 8–14). Blood samples were collected before and after dosing. A PBPK model (PK‐SIM^®^ version 11.2, Open System Pharmacology, USA) of ainuovirine and fluconazole was developed and validated to predict their DDIs. C_min,ss_ was defined as minimum plasma concentration at steady state; C_max,ss_ as maximum plasma concentration at steady state; AUC_0‐24,ss_ as area under the plasma concentration‐time curve during 24 hours at steady state; T_max,ss_ as time to maximum plasma concentration at steady state.

**Results**: All participants (*N* = 36) completed the study. In group A, when co‐administered with fluconazole, geometric means of ainuovirine pharmacokinetics parameters C_min,ss_, C_max,ss_ and AUC_0‐24,ss_ increased to 532.9%, 233.0% and 349.6%, respectively, compared to those of ainuovirine alone, whereas median T_max,ss_ was unaffected (Figure 1). In group B, there were no apparent effects of ainuovirine on C_max,ss_, AUC_0‐24,ss_ and T_max,ss_ for fluconazole. Possible treatment‐related adverse events (AEs) assessed by investigators were fewer in group A (83.3%) versus those in group B (94.4%); no death or grade ≥3 serious AE was reported. The PBPK modelling supported a dose reduction by half for co‐administration of ainuovirine with fluconazole.

**Conclusions**: Co‐administration of ainuovirine with fluconazole significantly increased ainuovirine systemic exposure, whereas ainuovirine did not appear to affect fluconazole exposure. Therefore, it is recommended that the ainuovirine dose be halved (i.e. 75 mg) when co‐administered with strong CYP2C19 inhibitors, including fluconazole.

**P197: Figure 1:**
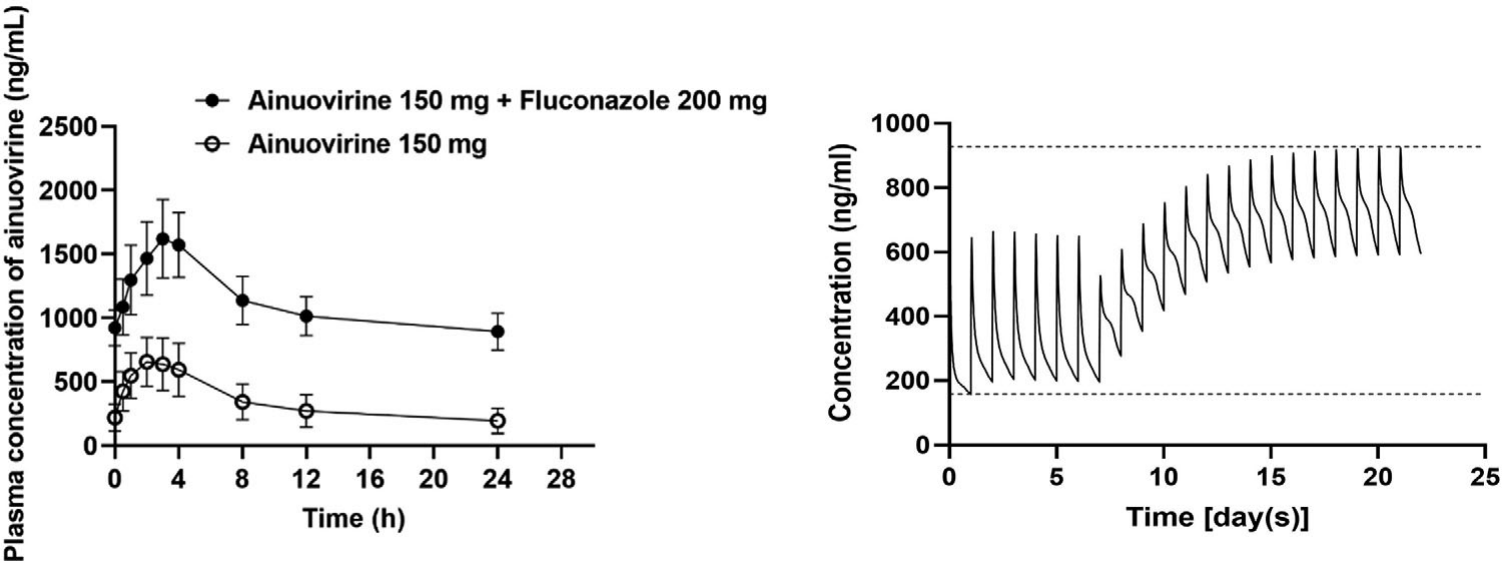
Comparison of clinically obtained arithmetic mean (SD) plasma concentration–time profiles of ainuovirine 150 mg with or without co‐administration of fluconazole 200 mg in healthy adults (left panel) with stimulation‐based PBPK model of ainuovirine 150 mg for 7 days, followed by ainuovirine 75 mg co‐administered with fluconazole 200 mg (right panel). SD, standard deviation.

### P198 The geriatric assessment and frequency of polypharmacy and severe drug‐drug interactions in older adults with HIV in a tertiary care centre in Mexico City

Su Jung Choi^1^, Alvaro López‐Íñiguez^1^, Yanink Caro‐Vega^1^, Virgilio Hernández‐Ruiz^2^, José Alberto Ávila‐Funes^2^, Rocio Angelica Sánchez‐Morales^1^, Melissa Guerrero‐Tobias^1^, Karen Aranza Marañón‐Solorio^1^, Juan Carlos Franco‐Rodríguez^1^, Brenda Eloisa Crabtree‐Ramírez ^1^, Juan Sierra‐Madero^1^

^1^Infectious Disease, The National Institute of Medical Sciences and Nutrition Salvador Zubirán, Mexico City, Mexico. ^2^Geriatrics, The National Institute of Medical Sciences and Nutrition Salvador Zubirán, Mexico City, Mexico

**Background**: The life expectancy of people living with HIV (PWH) has improved due to the efficacy of antiretroviral treatment (ART) [1−3]. As a consequence, age‐related comorbidities and, therefore, polypharmacy and drug‐drug interactions (DDIs) are common [4−6]. Most HIV clinics have limited access to a geriatric evaluation (GE) [7]. Thus, the performance of routine HIV care (RHC) compared to RHC plus a geriatric assessment is relevant.

**Methods**: A retrospective cohort analysis of PWH aged ≥50 who attended a tertiary care centre in Mexico City from 2022 to 2023 was done. We estimated the frequency of polypharmacy (≥5 drugs including ARV) and unfavourable DDI (according to Liverpool and www.drugs.com checker platforms) between two groups: (1) PWH who received at least one GE by geriatricians additionally to the RHC by ID specialists; and (2) PWH who received only RHC without a geriatrician evaluation. We described the most common comorbidities and comedication. Also, the frequency of severe DDIs between platforms was compared.

**Results**: Among the 243 PWH included, 121 (49%) received GE and 123 (51%) RHC. The median age was 58 years (IQR 55–60), with 88% being men and a median of 18 years of HIV diagnosis. Fifty‐three percent (129) of the population had polypharmacy without differences between groups. One hundred and ninety‐two (79%) participants had at least one comorbidity (76.2% in GE and 82% in RHC, *p* = 0.36); the three most common comorbidities were dyslipidaemia (46%), hypertension (20.1%) and osteoporosis (12.7%) without differences between groups. The most common drugs prescribed were lipid‐lowering (34.5%), cardiovascular (25.5%), psychotropic (13.5%) and antidiabetic drugs (13.1%), without differences in groups. The frequency of any DDI was similar in the groups (*p* = 0.067), but severe DDIs were different across the checker platforms (1.6% with Liverpool vs. 17% with www.drugs.com [*p*≤0.00001]).

**Conclusions**: The high burden of comorbidities in the population could explain the lack of differences in the frequency of polypharmacy and DDIs between GE and RHC, and highlights the complexity of HIV care. The frequency of severe DDIs using the Liverpool platform was similar to other studies (1.6%); however, it was higher with www.drugs.com. These discrepancies between platforms may have clinical implications and warrant further research.

**References**

1. Sarma P, Cassidy R, Corlett S, Katusiime B. Ageing with HIV: medicine optimisation challenges and support needs for older people living with HIV: a systematic review. Drugs Aging. 2023;40(3):179‐240.

2. da Silva RPN, Marins LMS, Guaraldo L, Luz PM, Cardoso SW, Moreira RI, et al. Pharmacotherapeutic profile, polypharmacy and its associated factors in a cohort of people living with HIV in Brazil. AIDS Res Ther. 2023;20(1):57.

3. Smit M, Brinkman K, Geerlings S, Smit C, Thyagarajan K, Sighem A, et al. Future challenges for clinical care of an aging population infected with HIV: a modeling study. Lancet Infect Dis. 2015;15(7):810‐8.

4. Zamudio D, Funes A. Guía para la Atención de las Personas Adultas Mayores que viven con VIH. CENSIDA. 2018;1(1).

5. Hernandez‐Ruiz V, Antonio‐Villa NE, Crabtree‐Ramírez BE, Belaunzarán‐Zamudio PF, Caro‐Vega Y, Brañas F, et al. Characterization of data‐driven geriatric syndrome clusters in older people with HIV: a Mexican multicenter cross‐sectional study. Lancet Reg Health Am. 2023;22:100502.

6. Guaraldi G, Falutz J. Multidimensional geriatric assessment in older patients with HIV. In: Guaraldi G, Falutz J, Mussi C, Silva AR, editors. Managing the older adult patient with HIV. Cham: Springer International Publishing; 2016. p. 123–8.

7. Sangarlangkarn A, Appelbaum JS. Comprehensive geriatric assessment in older persons with HIV. Open Forum Infect Dis. 2020;7(11):ofaa485.

### P199 Safety and efficacy of pharmacotherapy containing the second‐generation integrase inhibitors (INSTIs) and chemotherapy drugs in AIDS‐related diffuse large B‐cell lymphoma: a single‐centre retrospective analysis

Jing Yang

Department of General Surgery and Oncology Surgery, Public Health Clinical Center of Chengdu, Chengdu, China

**Background**: Previous studies have shown that the combination of antiviral and anti‐tumour drugs may have a superposition of toxic side effects and drug‐drug interactions (DDIs) risk, our research aims to explore the safety and efficacy of pharmacotherapy containing the second‐generation integrase inhibitors (INSTIs) and chemotherapy drugs in AIDS‐related diffuse large B‐cell lymphoma (AR‐DLBCL).

**Methods**: We conducted a retrospective cohort study of patients with AR‐DLBCL newly diagnosed at the Public Health Clinical Center of Chengdu from February 2020 to May 2023. All included patients were treated with the second‐generation INSTI‐based regimen during chemotherapy. The primary end points were frequency and severity of adverse effects (AEs). The secondary end point included CD4 count, CD4/CD8 ratio, HIV viral load and complete response (CR) rate at the end of treatment (EOT). An evaluation was conducted every chemotherapy cycle. AEs were assessed according to the Common Terminology Criteria for Adverse Events, version 4.02.

**Results**: We enrolled a total of 96 AR‐DLBCL with a median follow‐up of 12.2 months (range 5–33). Sixty out of 96 patients were treated with BIC/TAF/FTC, and 36 patients treated with DTG/3TC/ABT. The most common AEs of grade 3 or higher during treatment were neutrophils (32.29%) and thrombocytopenia (20.83%) attributed to anticancer agents. The additional serious complications during treatment were reported in seven patients (two of pulmonary tuberculosis, one of multiple organ dysfunction, one of intracranial infection, one of renal failure and two of severe COVID‐19), with three deaths. CD4 and CD4/CD8 decreased slightly from baseline (251.76+188.53 and 0.71+0.69) at the sixth month (233.44+140.528 and 0.66+0.55) and there was no statistical difference (*p* = 0.375 and *p* = 0.608). Viral load rebound was not observed among patients during chemotherapy. The objective response rate was 85.41%, and the complete response rate was 51.04%. As of June 2024, 15 died from severe infections or tumour progression.

**Conclusions**: The second‐generation INSTIs might be a safe, effective first‐line therapy option for AR‐DLBCL during chemotherapy.

### P200 Predicting drug‐drug interactions between ainuovirine and rifampicin plus isoniazid using PBPK modelling

Xianmin Meng^1^, Hong Qin ^2^, Yufang Zheng^2^, Chunyun Zhang^2^, Hongzhou Lu^1^

^1^National Clinical Research Centre for Infectious Diseases, The Third People's Hospital of Shenzhen, The Second Affiliated Hospital of Southern University of Science and Technology, Shenzhen, China. ^2^Clinical Research & Development, Jiangsu Aidea Pharmaceutical Co., Ltd, Yangzhou, China

**Background**: Ainuovirine (ACC007) is a next‐generation non‐nucleoside reverse transcriptase inhibitor (NNRTI) primarily metabolized by CYP2C19. The primary objective of this study was to simulate and predict drug‐drug interaction (DDI) between ainuovirine (ANV) and rifampicin (RFP) combined with isoniazid (INH) using the physiologically based pharmacokinetic (PBPK) modelling in people living with HIV coinfected with tuberculosis.

**Methods**: A comprehensive whole‐body PBPK model for DDI was developed using PK‐SIM^®^ (version 11.2, Open System Pharmacology, USA). This model was validated against phase I DDI study of ANV with RFP, and reported clinical data for all drugs administered alone, and ANV‐RFP/ANV‐INH dual‐ and ANV‐RFP‐INH triple‐drug concomitantly. This model contained the potent induction and concentration‐dependent inhibition mechanisms of RFP and INH on CYP2C19. This model was considered valid if the predicted pharmacokinetic values were within 0.5‐ to 2‐fold versus the observed. Alternative dosing regimens for ANV were simulated to ensure that the C_trough_ (concentration immediately prior to administration of the next dose) exceeded the lower limit of clinical reference interval 153.04±59.22 ng/ml, while the C_max_ (peak concentration) remained below the upper limit of 740.0±248.1 ng/ml (target safety margin).

**Results**: The PBPK model was successfully validated according to the aforementioned criteria. Simulation of different dose adjustments predicted that a change in regimen to ANV 225 mg (75+150 mg regimen, i.e. ANV 75 mg co‐administered with RFP plus INH, and an additional dose of 150 mg at an interval of 12 hours) offsetted the inhibitive effect of INH on ANV C_max_ and inductive effect of RFP on ANV C_trough_ at the steady state, both of which were within the clinical reference intervals.

**Conclusions**: The developed PBPK model characterized the opposite effect‐mediated DDIs between RFP and INH on metabolisms of ANV, accurately predicting a narrowed therapeutic window when ANV 150 mg daily was co‐administered with RFP plus INH. A change in ANV dosing regimen from 150 to 225 mg was predicted to mitigate the effect of the DDIs on the C_max_ and C_trough_ of ANV, maintaining plasma concentration levels above the therapeutic threshold but well below the safety margin.

### P201 Tracing the evolution of polypharmacy and drug‐drug interactions in people living with HIV

Victoria Lopez Delhoulle ^1^, Li‐Cécile Destordeur^1^, Nathalie Maes^2^, Karine Fombellida^1^, Majdouline El Moussaoui^1^, Gilles Darcis^1^

^1^Infectious Diseases Department, Liège University Hospital, Liège, Belgium. ^2^Biostatistics and Research Method Center, Liège University Hospital, Liège, Belgium

**Background**: The advent of antiretroviral therapy has significantly increased the life expectancy of people living with HIV (PLWH), resulting in an ageing HIV population. Consequently, polypharmacy has become common, leading to frequent drug‐drug interactions (DDIs), which continue to evolve with the introduction of new antiretroviral drugs. Our objective was to analyse this evolution over time.

**Materials and methods**: This retrospective cohort study was conducted at the University Hospital of Liège, Belgium, from January 2017 to December 2022. We analysed ARV and non‐ARV treatments of 812 PLWH, aged over 18, each of them with at least one annual consultation. The University of Liverpool HIV drug interactions database was used to identify DDIs, only focusing on contraindicated interactions.

**Results**: The number of comedications significantly increased during the study period. Participants with at least one comedication increased from 81.7% in 2017 to 88.6% in 2022 (*p* < 0.0001), while those with over five comedications increased from 20.7% to 28.9% (*p* < 0.0001) (Table 1). The number of contraindicated DDIs remained fairly stable over time (3.9% in 2017 to 2.8% in 2022; *p* = 0.18) but decreased when adjusting for the number of non‐ARV comedications (*p* = 0.0030). The number of non‐ARV comedications significantly increased the risk of having a DDI (*p* < 0.0001). NNRTI and booster were mostly associated with a higher risk of contraindicated DDIs (*p* = 0.0011 and *p* < 0.0001, respectively).

**P201: Table 1:** Number of non‐ARV comedications and drug‐drug interactions in participants with at least one consultation per year between 2017 and 2022, in the University Hospital of Liège, Belgium

	2017	2018	2019	2020	2021	2022	*p*‐value
Number of participants	812	812	812	812	812	812	
Number of participants with no comedication (%)	149 (18.3)	124 (15.3)	110 (13.6)	121 (14.9)	101 (12.4)	93 (11.4)	<0.0001^a^
Number of participants with one or more comedication (%)	663 (81.7)	688 (84.7)	702 (86.4)	691 (85.1)	711 (87.6)	719 (88.6)	<0.0001^a^
1−4	495	509	516	480	481	484	
≥5	168	179	186	211	230	235	<0.0001^a^
Mean ± SD	2.7±2.8	2.9±2.9	3.1±3.1	3.2±3.1	3.4±3.2	3.5±3.2	<0.0001^b^
Median (Q1−Q3)	2 (1−4)	2 (1−4)	2 (1−4)	2 (1−5)	3 (1−5)	3 (1−5)	
Min−max	0−17	0−18	0−23	0−21	0−23	0−20	
Number of participants with DDIs (%)	32 (3.9)	31 (3.8)	23 (2.8)	22 (2.7)	26 (3.2)	23 (2.8)	0.18^a^
Total number of DDIs	46	42	33	29	37	28	0.051^c^

^a^Generalized estimating equations model (GEE).

^b^Repeated measures ANOVA.

^c^Linear model.

**Conclusions**: Despite progress in ARV treatments leading to more favourable DDI profiles, our study shows the persistence of a significant number of contraindicated DDIs. The average age of PLWH uprises while polypharmacy is becoming common, leading to more DDIs. Clinicians should be aware of this remaining issue and tools should be implemented to alert healthcare providers and shrink the number of DDIs.

### P202 Concomitant administration of long‐acting cabotegravir/rilpivirine and intramuscular antibiotics for the treatment of sexually transmitted infections: no evidence of pharmacodynamic interference

Roberto Rossotti, Nicholas Brian Bana, Francesco Peracchi, Gabriele Cavazza, Elisa Di Gennaro, Chiara Baiguera, Alessandro Raimondi, Marco Merli, Carlotta Rogati, Federico D'Amico, Maria Cristina Moioli, Massimo Puoti

Infectious Diseases, Azienda Socio Sanitaria Territoriale (ASST) Grande Ospedale Metropolitano Niguarda, Milan, Italy

**Background**: People living with HIV (PLWH) receiving cabotegravir/rilpivirine long‐acting (CARLA) treatment are often at high risk of sexually transmitted infections (STIs) acquisition requiring intramuscular antibiotic injections. No pharmacokinetics interactions are expected, but both CARLA and benzathine penicillin generate drug depot formation. Thus, concurrent intramuscular administrations might result in perturbations in drug dissolution and absorption. Aim of this study is to assess whether concomitant intramuscular CARLA and antibiotic injections might lead to inadequate antimicrobial response.

**Materials and methods**: A double comparative study was designed. Study population was represented by PLWH receiving CARLA and benzathine penicillin, ceftriaxone or gentamycin to treat an STI. To assess antiretroviral efficacy, they were compared to PLWH who received CARLA the same day but were not exposed to antibiotics; HIV RNA values before and after injections were compared. To assess antibacterial efficacy, study population was compared to individuals who received benzathine penicillin but were not exposed to CARLA; four‐fold decrease in rapid plasma reagin (non‐treponemal) test (RPR) titre was evaluated. Descriptive statistics, non‐parametric tests and standard survival analyses were used.

**Results**: The study enrolled 319 individuals: 25 receiving CARLA and antibiotics, 98 receiving only CARLA and 209 receiving only benzathine penicillin. They were mainly males (310, 94.2%), born in Italy (258, 78.4%) and with a median age of 39 (IQR 32–49) years. Immunological and virological parameters were excellent, but PLWH belonging to syphilis control group were frequently treatment naïve thus HIV RNA was more commonly detectable. Table 1 shows demographic and clinical features of enrolled individuals. Median time between CARLA and antibiotic injections was 8 (IQR 6–24) days. There were no differences in HIV RNA values between groups before (*p* = 0.525) and after (*p* = 0.788) the injections nor within the same group before and after the administration (*p* = 0.791, Figure 1, left). Time to RPR four‐fold decrease was comparable between study population and the control group (Figure 1, right). Adjusted Cox regression analysis failed to detect an effect of CARLA administration on serological response (aHR 1.29, 95% CI 0.60–2.78, *p* = 0.518).

**P202: Table 1:** Demographic and clinical features of study population

		PLWH receiving LA and antibiotics (*N* = 25)	Controls for HIV RNA (*N* = 98)	Controls for syphilis (*N* = 206)	*p*‐value
Age, years, median (IQR)		39 (36−48)	48 (39−56)	36 (30−44)	0.034
Born in Italy, *n* (%)		21 (84.0)	82 (83.7)	155 (75.2)	0.217
Gender, *n* (%)	Males	25 (100)	87 (88.8)	198 (96.1)	0.011
	Females	–	11 (11.2)	5 (2.4)	
	TGW	–	–	3 (1.5)	
CD4 cell count, cell/mmc, median (IQR)		844 (596−1006)	805 (588−1026)	716 (473−934)	0.298
HIV RNA class, *n* (%)	TND	18 (72.0)	76 (77.6)	24 (68.5)^a^	0.010
	BLQ	3 (12.0)	15 (15.3)	3 (8.6)^a^	
	20−200	4 (16.0)	7 (7.1)	3 (8.6)^a^	
	>1000	–	–	5 (14.3)^a^	
HIV RNA, log10 cp/ml, median (IQR)		1.53 (1.51−1.62)	1.60 (1.34−1.89)	4.27 (1.78−4.83)	<0.001
RPR value, median (IQR)		16 (8−64)		8 (2−32)	0.177
Days between CARLA and antibiotic injection, median (IQR)		8 (6−24)			
PLWH, *n* (%)				37 (18.0)	
Syphilis, *n* (%)		15 (60.0)			
Gonorrhoea, *n* (%)		6 (24.0)			
Partner notification (not tested/no diagnosis)^b^, *n* (%)		4 (16.0)			

Abbreviations: BLQ, below the limit of quantification; CARLA, cabotegravir + rilpivirine long acting; PLWH, people living with HIV; RPR, rapid plasma reagin (non‐treponemal) test; TGW, transgender women; TND, target not detected.

^a^Data available for 35 individuals.

^b^Individuals who received an antibiotic injection upon a syphilis/gonorrhoea partner notification but were not tested or laboratory result was negative.

**P202: Figure 1:**
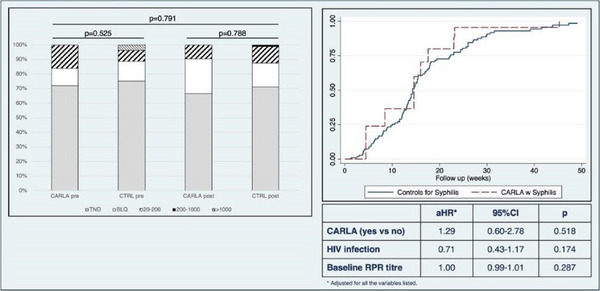
(left) HIV RNA class distribution before and after intramuscular CARLA/antibiotic injection; (right) Kaplan‐Meier estimates for 4‐fold decrease in RPR titre.

**Conclusions**: Concomitant intramuscular CARLA and antibiotic injections 8 days apart did not lead to inadequate antiretroviral and antibacterial responses.

### P203 A study on pharmacist antiretroviral stewardship in relation to drug interaction checking within the Genitourinary Medicine and Infectious Diseases (GUIDe) outpatient clinic in St James's Hospital

Arlene Heekin ^1^, Teresa M. Barbosa^2^, Miriam Moriarty^1^, Miriam Coghlan^1^, Colm Bergin^3^

^1^Pharmacy, St James's Hospital, Dublin, Ireland. ^2^Pharmacy, University College Cork, Cork, Ireland. ^3^The GUIDE Clinic, St James's Hospital, Dublin, Ireland

**Background**: Antiretrovirals (ARVs) are highly susceptible to potential drug‐drug interactions (PDDIs) illustrating the requirement for antiretroviral stewardship programmes (ARVSPs). In an ageing HIV cohort with increasing polypharmacy, pharmacists through their role in ARVSPs can play a key part in mitigating medication errors with ARVs especially in identifying PDDIs.

**Methods**: Prospective cohort study conducted in an outpatient clinic in St James's Hospital. Patients who attended HIV and HIV/hepatitis co‐infection clinics between April and June 2022 were included unless exclusion criteria were met. Co‐medications were assessed for PDDIs with ARVs. Types of interactions and interaction management strategy were analysed. Moderate‐severe interactions were assessed by a peer review panel using a validated scoring tool to grade potential harm had these not been identified by a pharmacist.

**Results**: In total, data was gathered from 398 patients. Median age of the study population was 43 and 77.9% were males. Polypharmacy was reported in 19%. PDDIs were identified in 37.9%, with patients ≥50 years having a greater risk of PDDIs (46.9%, *n* = 60/128). Increased polypharmacy in those ≥50 years (37.5%, *n* = 48/128) was associated with increased PDDI risk. Integrase strand transfer inhibitors (INSTIs) were the ARV class most commonly implicated in PDDIs (49.4%, *n* = 85/172). Co‐medications associated with the highest prevalence of PDDIs included vitamins, mineral supplements and drugs for acid‐related disorders. Administration advice was the most frequent management strategy to overcome PDDI risk (61%, *n* = 105/172). The peer review panel determined patients had a 4% risk of severe harm, 93% risk of moderate harm and 3% risk of minor harm had interactions not been identified and managed by the pharmacist (Table 1 and Figure 1).

**Conclusions**: Polypharmacy and older age are associated with an increased risk of PDDIs. Increased INSTI use has seen their association with PDDIs also increases, implicating mainly non‐prescribed medication. This demonstrates the importance of complete medication histories including over‐the‐counter medicines and herbal supplements. Medication history taking and DDI checking are well‐established roles of pharmacists, further supporting the value of pharmacists in ARVSPs.

**P203: Table 1:** Frequency of polypharmacy and type of potential drug‐drug interactions

	Age <50 years (*n* = 270)	Age ≥50 years (*n* = 128)	Total study population (*n* = 398)
Number of patients with polypharmacy (≥5 total medications), *n* (%)	29 (10.7)	48 (37.5)	77 (19)
Number of patients with ≥2 PDDI, *n* (%)	20 (7.4)	22 (17.2)	42 (10.6)
Type of PDDI			
Severe, *n* (%)	3 (1.1)	3 (2.3)	6 (1.5)
Moderate, *n* (%)	87 (32.2)	79 (61.7)	166 (41.7)
Weak, *n* (%)	32 (11.9)	13 (10.2)	45 (11.3)

**P203: Figure 1:**
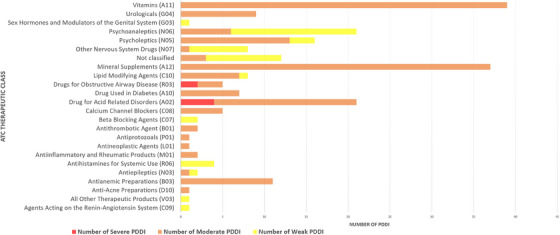
Non‐ARVs associated with severe, moderate and weak potential drug‐drug interactions.

### P204 Cabotegravir and rilpivirine injectable therapy with concomitant rifampicin treatment for a multidrug‐resistant surgical site infection: management of antiretroviral treatment in real world using therapeutic drug monitoring

Lorenzo Ciullini ^1^, Paul Thoueille^2^, Emilie Piet^3^, Laurent Arthur Decosterd^2^, Catia Marzolini^4^, Alexandra Calmy^1^

^1^HIV/AIDS Unit, Division of Infectious Diseases, Geneva University Hospital (HUG), Geneva, Switzerland. ^2^Service of Clinical Pharmacology, Department of Laboratory Medicine and Pathology, Lausanne University Hospital and University of Lausanne, Lausanne, Switzerland. ^3^Division of Infectious Diseases, Annecy‐Genevois Hospital Centre, Annecy, France. ^4^Service of Clinical Pharmacology, Division of Infectious Diseases & Hospital Epidemiology, Dept Molecular Clinical Pharmacology, Lausanne University Hospital and University of Lausanne, Basel University Hospital, University of Liverpool, Lausanne, Switzerland

**Background**: The potent inducer rifampicin reduces the area under the curve (AUC) and the trough concentration (C_min_) of oral cabotegravir and rilpivirine (CAB/RPV) [1]. Its effect on the long‐acting injectable (LAI) formulation has not been evaluated in real life, although modelling studies predict a significant reduction in AUC and C_min_ [2,3]. Management of inaugural rifampicin treatment while on LAI has not been described. We describe a case of a subject on LAI requiring rifampicin for a multidrug‐resistant surgical site infection.

**Materials and methods**: A 45‐year‐old male subject living with HIV suppressed on LAI CAB/RPV (initiation: 17 February 2024; last injection: 29 January 2022) had a traumatic tibia fracture in December 2023 requiring internal fixation on 2 January 2024. He developed an early‐onset deep infection at the surgical site by methicillin‐resistant *S. aureus* and *P. aeruginosa*. After several antibiotics leading to improvement, daily (QD) oral rifampicin (600 mg) was initiated for its anti‐biofilm activity, lasting 12 weeks. To prevent sub‐therapeutic plasma concentrations of CAB/RPV and related resistances, a three‐drug oral antiretroviral regimen (tenofovir disoproxil fumarate/emtricitabine 245/200 mg QD and dolutegravir 50 mg twice daily, BID) was initiated on 26 February. Dolutegravir BID was maintained for 3 weeks after rifampicin discontinuation due to the persisting inducing effect, then reduced to 50 mg QD. Five weeks after rifampicin discontinuation, LAI CAB/RPV was re‐initiated and oral antiretrovirals were discontinued. Tolerability, HIV‐1 viral load and therapeutic drug monitoring (TDM) of CAB/RPV were assessed during and after rifampicin treatment.

**Results**: CAB/RPV plasma concentrations substantially decreased by day 8 after rifampicin initiation falling below target thresholds (664 ng/ml for CAB and 32 ng/ml for RPV) from day 10 (Figure 1). Tenofovir‐emtricitabine and dolutegravir were well tolerated with target range concentrations during rifampicin treatment. Rifampicin's inducing effect resolved 3 weeks after discontinuation, allowing dolutegravir to be reduced to 50 mg QD.

**Conclusions**: LAI CAB/RPV concentrations significantly decrease during concomitant rifampicin. Rapid switch to oral QD tenofovir/emtricitabine plus BID dolutegravir maintained effective antiretroviral levels and viral suppression during rifampicin co‐administration. TDM is useful for managing complex drug interactions. Further real‐life studies are needed for LAI CAB/RPV.

**P204: Figure 1:**
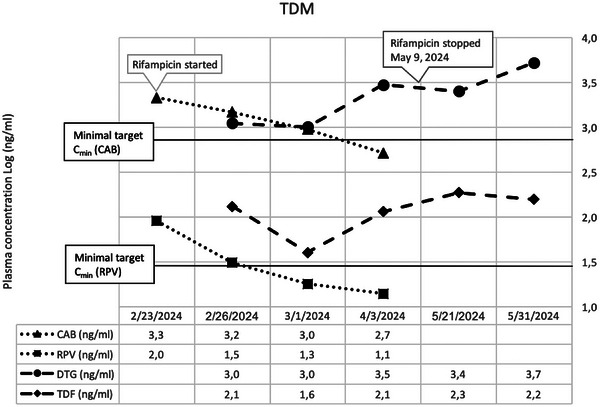
Plasmatic trough concentrations (C_min_) expressed in log scale of ng/ml by date of sampling. CAB, cabotegravir intramuscular; DTG, dolutegravir; RPV, rilpivirine intramuscular; TDF, tenofovir disoproxil fumarate.

**References**

1. Ford SL, Sutton K, Lou Y, Zhang Z, Tenorio A, Trezza C, et al. Effect of rifampin on the single‐dose pharmacokinetics of oral cabotegravir in healthy subjects. Antimicrob Agents Chemother. 2017;61(10):e00487‐17.

2. Rajoli RKR, Curley P, Chiong J, Back D, Flexner C, Owen A, et al. Predicting drug‐drug interactions between rifampicin and long‐acting cabotegravir and rilpivirine using physiologically based pharmacokinetic modeling. J Infect Dis. 2019;219(11):1735‐42.

3. Bettonte S, Berton M, Stader F, Battegay M, Marzolini C. Management of drug‐drug interactions between long‐acting cabotegravir and rilpivirine and comedications with inducing properties: a modeling study. Clin Infect Dis. 2023;76(7):1225‐36.

## Cure/post‐treatment control

### P205 Characterizing HIV‐1 elite controllers within the Italian ICONA cohort: implications for HIV functional cure

Francesca Ceccherini‐Silberstein^1^, Luna Colagrossi ^2^, Alessandro Tavelli^3^, Omar El Khalili^1^, Stefano Rusconi^4^, Giuseppe Lapadula^5^, Antonella Cingolani^6^, Alessandra Vergori^7^, Andrea Calcagno^8^, Sergio Lo Caputo^9^, Antonella Castagna^10^, Alessandro Cozzi‐Lepri^11^, Giulia Carla Marchetti^12^, Carlo‐Federico Perno^13^, Antonella d'Arminio Monforte^3^

^1^Department of Experimental Medicine, University of Rome Tor Vergata, Rome, Italy. ^2^Unit of Diagnostic Microbiology and Immunology, Bambino Gesù Children's Hospital, Rome, Italy. ^3^ICONA Foundation, Milan, Italy. ^4^Infectious Diseases Unit, Ospedale Civile di Legnano, ASST Ovest Milanese, DIBIC Luigi Sacco, University of Milan, Milan, Italy. ^5^Department of Infectious Diseases, IRCCS San Gerardo dei Tintori, University of Milano‐Bicocca, Monza, Italy. ^6^Department of Safety and Bioethics, Clinic of Infectious Diseases, Università Cattolica del Sacro Cuore, Fondazione Policlinico Universitario Agostino Gemelli IRCCS, Rome, Italy. ^7^Clinical and Research Infectious Diseases Department, National Institute for Infectious Diseases Lazzaro Spallanzani, IRCCS, Rome, Italy. ^8^Unit of Infectious Diseases, Department of Medical Sciences, University of Turin, Amedeo di Savoia Hospital, Turin, Italy. ^9^Department of Clinical and Surgical Sciences, University of Foggia, Foggia, Italy. ^10^Infectious Diseases Unit, IRCCS San Raffaele Scientific Institute, Vita Salute San Raffaele University, Milan, Italy. ^11^Centre for Clinical Research, Epidemiology, Modelling and Evaluation, Institute for Global Health, London, UK. ^12^Clinic of Infectious Diseases, ASST Santi Paolo e Carlo, University of Milan, Milan, Italy. ^13^Unit of Diagnostic Microbiology and Immunology and Multimodal Medicine Area, Bambino Gesù Children's Hospital, Rome, Italy

**Background**: A very small percentage of people with HIV‐1 (PWH), defined as elite controllers (ECs), control viral replication without antiretroviral therapy (ART). How long can this status be maintained over time remains to be fully studied.

**Materials and methods**: EC‐200 were defined as PWH with ≥3 consecutive HIV‐RNA <200 copies/ml for ≥12 months without ART, after enrolment in the ICONA Foundation Study (baseline). We estimated cumulative probability of maintaining EC‐200 after baseline. Loss of EC‐200 was defined as two consecutive HIV‐RNA >200 copies/ml or initiation of ART with one HIV‐RNA >200 copies/ml or with CD4^+^ cell count <500. A competing risk survival analysis (Fine‐Gray, with competitive event defined as ART start with CD4 >500 cells/mmc and/or HIV‐RNA <200 copies/ml) was performed. The role of HIV‐RNA <50 copies/ml (three consecutive values defined EC‐50) for predicting maintenance of EC‐200 was evaluated in PWH enrolled after 2003. Sensitivity survival analysis was done in EC‐50 with a threshold of 50 copies.

**Results**: We identified 123 EC‐200, mostly Italian (75.6%), male (62.6%), with a median age of 37 (IQR 33–48) years; 57 (46.3%) were EC‐50. Median time from HIV diagnosis to enrolment was 2.0 (IQR 0.1–10.3) years, median CD4 count was 765 (IQR 578–965) cells/mmc, CD4/CD8 ratio 1.0 (IQR 0.7–1.5). The probability of maintaining the EC‐200 status was 70.9 (95% CI 61.7–79.5) by 5 years, 54.5 (95% CI 43.6–66.2) by 10 years (40 events by end of follow‐up: 28 with two HIV‐RNA >200 copies/ml, eight start ART with CD4 <500/mmc and four with one HIV‐RNA >200 copies/ml; Figure 1a). This probability was higher in EC‐50: by 5 years maintenance of EC‐200 87.8% (95% CI 74.0–96.3, *p* = 0.017; Figure 1b). Among EC‐50, the 5 years probability of maintaining EC‐50 was 83.6% (95% CI 70.9–92.9; Figure 1c). After controlling for confounding factors (age, sex, year of enrolment, years from diagnosis to enrolment) EC‐50, higher CD4 and CD4/CD8 were associated with longer time with EC‐200 status (Table 1).

**P205: Table 1:** Fine‐Gray regression models estimating the sub‐hazard (SHR) ratio of losing the EC‐200 status

	SHR	95% CI	*p*‐value	aSHR^a^	95% CI	*p*‐value
EC‐50						
No	1			1		
Yes	0.33	0.14−0.75	0.008	0.29	0.11−0.76	0.012
Baseline CD4, per 100 cells/mmc higher	0.85	0.77−0.99	0.035	0.85	0.73−0.99	0.038
Baseline CD4/CD8 ratio, per 0.1 higher	0.87	0.80−0.94	0.001	0.87	0.80−0.94	0.001

^a^Adjusted for age, sex, calendar year of enrolment, years from HIV diagnosis to enrolment.

**P205: Figure 1:**
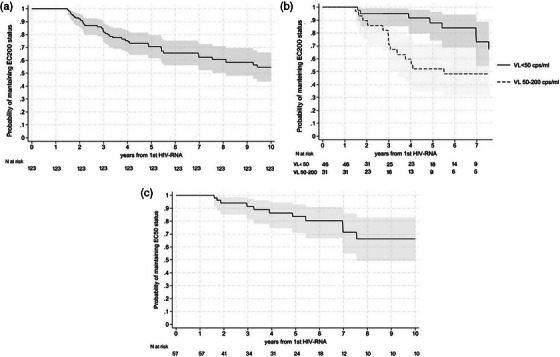
Competing‐risk curves estimating the probability of maintaining the EC‐200 status overall (a) and stratified by baseline HIV‐RNA (b); and maintaining the EC‐50 status in the EC‐50 group (c).

**Conclusions**: Approximately 50% of our population lost their EC‐200 status by 10 years; levels of baseline HIV‐RNA, CD4 and CD8 are critical in controlling viral replication in absence of treatment. Deeper viro‐immunological characterization of these ECs can serve as models of a functional cure for HIV.

### P206 Absence of viral rebound without antiretrovirals after CCR5Δ32/Δ32 allogeneic hematopoietic stem cell transplantation: a new case of a potential cure of HIV?

Olivia Zaegel‐Faucher ^1^, Céline Boschi^2^, Samir Benkouiten^1^, Hélène Laroche^1^, Maëva Dos Santos^1^, Anne Motte^2^, Faezeh Izadifar‐Legrand^3^, Daniel Olive^4^, Philippe Colson^2^, Sylvie Bregigeon‐Ronot^1^

^1^Centre d'Informations et de Soins de l'Immunodéficience Humaine et des Hépatites Virales (CISIH), Assistance Publique‐Hôpitaux de Marseille (AP‐HM), Marseille, France. ^2^IHU Méditerranée Infection, Assistance Publique‐Hôpitaux de Marseille (AP‐HM), Marseille, France. ^3^Département d'Hématologie, Institut Paoli‐Calmettes, Marseille, France. ^4^Centre de Recherche en Cancérologie de Marseille (CRCM), Institut Paoli‐Calmettes, Marseille, France

**Background**: Six cases of HIV‐1 remission have been reported to date, five of them after CCR5Δ32/Δ32 allogeneic haematopoietic stem cell transplantation (aHSCT). We report a woman in her 50s diagnosed with HIV‐1 in 1999, who initiated early antiretroviral therapy (ART) and exhibited undetectable HIV loads in 2010. She developed acute myeloid leukaemia (AML) and received CCR5Δ32/Δ32 aHSCT from an HLA‐mismatched (HLA‐A) donor in July 2020 after a Baltimore‐based conditioning and graft versus host disease (GVHD) prophylaxis. Full donor chimerism was obtained after three donor lymphocyte infusions. Pre‐aHSCT, CD4 count was 250 cells/mm^3^, HIV‐1 DNA was 32 copies/10^6^ peripheral blood mononuclear cells (PBMCs) and HIV‐1 RNA was undetectable (<20 copies/ml). Post‐aHSCT, no HIV‐1 DNA or RNA were detected in circulating CD4^+^ cells or plasma. Treatment interruption (TI) occurred 39 months post‐aHSCT in October 2023.

**Materials and methods**: Post‐TI, samples were collected every week for 2 months, then once a month. HIV‐1 RNA/DNA were measured using NeuMoDx (Qiagen)/Xpert (Cepheid) and generic (Biocentric) assays, respectively, and by ultrasensitive (US) assays for some samples. HIV‐1 RNA/DNA sequencing was performed by Sanger or next‐generation sequencing (NGS) (Illumina) technologies with quasispecies analysis. HIV‐1‐specific antibodies were detected by Western blot analysis (MP Diagnostics) on sequential samples. Standard HIV‐1 co‐culture and cell permissiveness to R5 HIV‐1 strains were assayed using in‐house protocols.

**Results**: Samples were collected at 13 post‐TI time points. Eight months post‐TI, CD4 count and percent were 1103 cells/mm^3^ and 25.3%, respectively. No HIV‐1 DNA or RNA were detected in circulating CD4^+^ cells or plasma, even using US assays. HIV‐1 antibodies slightly declined over time. HIV‐1 co‐culture was negative and host cells were non‐permissive for R5‐tropic HIV‐1 strains, while pre‐aHSCT HIV genotypic tropism tests using NGS showed the presence of an R5‐tropic virus population without minority X4‐tropic variants. HIV remission has been sustained to date, with no significant clinical events, except pneumococcal meningitis in November 2023, in a context of splenectomy and patient's refusal of prophylaxis.

**Conclusions**: We report a new case of remission and possible HIV‐1 cure in a woman with no evidence of HIV‐1 RNA rebound and no detectable HIV‐1 DNA at 8 months post‐TI and 47 months post‐CCR5Δ32/Δ32 aHSCT for AML.

### P207 Characteristics and management of patients with low‐level viraemia (LLV) in Greece: a multicentre retrospective study

Lydia Leonidou ^1^, Giota Lourida^2^, Konstantinos Protopapas^3^, Chralambos Moschopoulos^3^, Dimitrios Zoulas^4^, Charisis Totsikas^2^, Dimitrios Basoulis^5^, Elpida Mastrogianni^5^, Apostolos Beloukas^6^, Maria Lagadinou^1^, Markos Marangos^1^, Vasileios Papastamopoulos^2^, Antonios Papadopoulos^3^, Mina Psichogiou^5^

^1^Infectious Diseases Department, General University Hospital of Patras, Patras, Greece. ^2^Infectious Diseases Department, Evangelismos General Hospital, Athens, Greece. ^3^4th Department of Internal Medicine, School of Medicine, Attikon University Hospital, National and Kapodistrian University of Athens, Athens, Greece. ^4^Blood Bank Department, Evangelismos General Hospital, Athens, Greece. ^5^Internal Medicine Department, National and Kapodistrian University of Athens, Laiko General Hospital, Athens, Greece. ^6^Department of Biomedical Sciences, School of Health Sciences, University of West Attica, Athens, Greece

**Background**: Despite effective ART, a subset of people with HIV (PWH) still experience low‐level viraemia (LLV). The study aim was to assess LLV prevalence and management in a setting where next‐generation sequencing (NGS) genotyping, therapeutic drug monitoring (TDM) and electronic drug adherence records are non‐available.

**Materials and methods**: This is a 2‐year retrospective analysis (1/2022–12/2023) from four HIV clinics. LLV was defined as ≥2 consecutive HIV‐RNA measurements between 51 and 200 copies/ml on PWH virologically suppressed for ≥6 months. We assessed three consecutive VL measurements after the first LLV and epidemiological/medical records data. Adherence was self‐reported but also assessed by the treating physician.

**Results**: Among 3800 PWH with available VL measurements, 35 patients had LLV. They were mostly male (69%), Greek (74%), with mean age 49 years, MSM/IVDU (45/11%). Coinfection with HBV/HCV was rare (2.9%/8.6%, respectively), and 9/35 had substance use records [alcohol/IVDU/cannabis]. At baseline, 23 (63.8%) were CDC stage A and four (11%) stage C and had median CD4 count 328 cells/µl (IQR 6–847), and median VL 821,622 copies/ml (IQR 346–1 × 10^6^). At the time of the first LLV measurement, they had received 3.5 ART lines (IQR 1–14), were virologically suppressed for 7 years and were all on three‐drug ART: 20 (57%) on high genetic barrier ART (second‐generation INSTI or PI). All had previous genotype‐resistance testing (GRT) with clinically significant resistance mutations in three (8.5%), while six (17%) had a history of virological failure (VF). LLV resolved in 26/35 (74%) within the study follow‐up period, persisted in eight and recurred in one case. The reason for LLV was unknown for 18/35 (51.5%), low adherence for 14/35 (40%) and DDIs for 3/35 (8.5%). Strategies that lead to LVV resolution were: ART switch (eight patients, 30%), improved adherence (seven patients, 26.9%), DDIs management (three patients, 11.5%), combination of the three (eight patients, 30%). GRT was not available for VL <200 copies. Interestingly, only 10/17 (58.8%) who switched ART achieved viral suppression.

**Conclusions**: Adherence and switch strategy could lead to lower LLV. In a resource‐limited setting, empiric ART switch was found to be frequently implemented in PWH with LLV but was efficient in less than ⅔ of cases.

### P208 VH3810109 (N6LS) administration dose‐responsively enhances anti‐HIV antibody‐dependent cellular cytotoxicity (ADCC) and antibody‐dependent cellular phagocytosis (ADCP) activity in ex vivo models

Michael Keegan ^1^, Margaret Gartland^2^, Saikat Chakraborty^3^, Judah Abberbock^4^, Wilson Chen^5^, Paul Wannamaker^6^, Peter Leone^6^, Jan Losos^6^, Richard Dunham^5^

^1^Translational Research & Cure, ViiV Healthcare Ltd., London, UK. ^2^Virology, ViiV Healthcare Ltd., Durham, NC, USA. ^3^Statistics, Bangalore, GlaxoSmithKline, India. ^4^Statistics, GlaxoSmithKline, Collegeville, PA, USA. ^5^Translational Research & Cure, ViiV Healthcare Ltd., Durham, NC, USA. ^6^Clinical Development, ViiV Healthcare Ltd., Durham, NC, USA

**Background: **HIV‐specific broadly neutralizing antibodies (bNAbs) are being investigated for their antiviral activities through neutralization of the HIV Env protein, preventing interaction with cellular receptors and infection of new cells. Antibodies also mediate important immunological effects, including directing effector cells of the innate immune system to clear infected cells producing viral proteins through ADCC or ADCP. This may contribute to reducing the viral reservoir. VH3810109 is a bNAb in phase IIb clinical trials for maintenance of HIV suppression. In this exploratory analysis in participants naive to ART in the phase IIa BANNER study, we measured the capacity of VH3810109 to change ADCC and ADCP activity in serum during single‐dose monotherapy and related this to virological response.

**Materials and methods**: ADCC was measured as depletion of BG505.SOSIP.664‐coated CEM.NKR targets by primary natural killer (NK) cells isolated from a healthy donor in the presence of a serial dilution of study serum. ADCP was assessed through uptake of BG505.SOSIP.664‐coated polystyrene beads by THP1 cells in the presence of a serial dilution of study serum. ADCC and ADCP areas under the curve (AUCs) were determined and compared across study time points.

**Results**: We observed a range of baseline ADCC and ADCP activities with some individuals having nearly no activity and others having near maximum activity even with dilutions >1:100,000 and >1:1000, respectively. Following VH3810109 administration, we observed an increase in ADCC and ADCP activity in most participants. We observed a dose‐response increase in ADCP activity that corresponded to VH3810109 exposure. A weaker trend was observed with ADCC. The observed decline in viral load across treatment arms in the BANNER study corresponded to both increasing VH3810109 exposure and ADCP AUC. This was not observed for ADCC.

**Conclusions**: We demonstrate that VH3810109 has immunological activities ex vivo that correlate with clinically relevant virological outcomes. These data underscore the importance of the immunological activities of bNAbs and support continued development of these agents as direct‐acting antiviral agents as well as components of remission/cure regimens.

### P209 Perceptions of healthcare professionals, civil society, pharmaceutical industry and decision‐makers around the role of Latin America and the Caribbean in seeking HIV cure

Natalia Laufer^1^, Gaston Devisich^2^, Gabriela Turk^1^, Fernando Valiente‐Echeverria^3^, María Figueroa ^2^, Pedro Cahn^2^, Omar Sued^4^, Claudia Cortes^3^, Brenda Crabtree‐Ramírez^5^, Isabel Cassetti^6^

^1^Instituto de Investigaciones Biomédicas en Retrovirus y SIDA (INBIRS), Universidad de Buenos Aires, Buenos Aires, Argentina. ^2^Fundación Huésped, Buenos Aires, Argentina. ^3^Faculty of Medicine, Universidad de Chile & Fundación Arriarán, Santiago, Chile. ^4^Pan American Health Organization, Washington, DC, USA. ^5^Departamento de Infectología, Instituto Nacional de Ciencias Medicas y Nutricion Salvador Zubiran, Mexico City, Mexico. ^6^Helios Salud, Buenos Aires, Argentina

**Background**: Multiple initiatives and collaborative projects are working towards achieving a cure for HIV with significant progress. However, HIV cure‐related research efforts (both biomedical and social) in Latin America and the Caribbean (LAC) are fragmented, scattered and poorly integrated into the global agenda. Thus, a consultation on research and advocacy needs was conducted to delineate actions for an HIV Cure Consortium in the region.

**Methods**: An online survey performed between 25 March and 26 June 2024, available in Spanish, Portuguese, English and French assessing perceptions of LAC healthcare professionals and researchers (HP/R), pharmaceutical industry (PI), civil society (CS) and decision‐makers (DM) on HIV cure. This was distributed via email, social media and instant messaging apps. Answers were collected in RedCap and analysed.

**Results**: A total of 661 answers were received from individuals from 20/33 LAC countries: predominantly from Argentina (16.25%), Mexico (16%), Brazil (11.8%), Ecuador (9.2%), Colombia (6%), Bolivia (4.9%), among others. Most of the individuals (50.3%) were between 35 and 54 years old and had long‐term experience in the HIV field (>10 years, 50%). HP/R and CS reported discordant levels of knowledge regarding strategies to attempt HIV elimination, new cases of cure and funding for research. All groups agreed that there is a low level of priority given to HIV cure in the region; with low financial investment, both national and international, being identified as the main barrier for HIV cure. Multisectorial dialogue spaces, the inclusion of HIV cure research in health policies and training opportunities were considered as critical needs. Promotion of research and advocacy campaigns and specific funding opportunities were selected as the top priorities of an HIV Cure Consortium in LAC. CS indicated that having to discontinue treatment is the main factor that discourages PLWH from participating in a clinical study.

**Conclusions**: The survey results highlight the importance of creating a regional HIV Cure Consortium to enhance collaborations resulting in synergistic research and advocacy agendas, educational programmes and financial opportunities to implement local research and position the region at the global movement.

### P210 Insights to a cure: unique controller phenotypes in the Rotterdam HIV‐2 cohort

Kathryn Hensley ^1^, Rob Gruters^2^, Els van Nood^1^, Mariana de Mendonça Melo^1^, Ronald Overmars^2^, Alicja Gorska^3^, Casper Rokx^1^, Cynthia Lungu^2^, David van de Vijver^2^, Thibault Mesplède^2^, Jeroen van Kampen^2^

^1^Internal Medicine, Section Infectious Diseases, Medical Microbiology and Infectious Diseases, Erasmus University Medical Centre, Rotterdam, Netherlands. ^2^Viroscience, Erasmus University Medical Centre, Rotterdam, Netherlands. ^3^Viroscience, Medical Microbiology and Infectious Diseases, Erasmus University Medical Centre, Rotterdam, Netherlands

**Background**: HIV‐2, although less common than HIV‐1, exhibits a higher proportion of elite controllers (ECs). These ECs can naturally suppress the virus without antiretroviral therapy (ART), a phenomenon rarely observed in HIV‐1. Studying these ECs in HIV‐2 could yield valuable insights into viral control mechanisms and potentially lead to a cure for HIV.

**Materials and methods**: We retrospectively characterized a cohort of people living with HIV‐2 at the Erasmus University Medical Center, Rotterdam, the Netherlands, using data from 1989 to 2023. The primary goal was to identify ECs and other distinct categories of control in people with HIV‐2 based on HIV‐2 plasma viral loads, CD4^+^ T‐cell count trajectories and responses to ART.

**Results**: The Rotterdam Erasmus MC HIV‐2 cohort compromised 52 people living with HIV‐2, primarily of West African origin (80.8%). Follow‐up duration ranged from less than 1 to 32 years. Median CD4^+^ T‐cell count at diagnosis was 240 cells/mm^3^ (IQR 80–740). Eight participants were lost to follow‐up (15.4%), and 17 participants passed away (32.7%), with seven deaths occurring before combination ART became available. The cohort included 13 ECs with CD4^+^ T‐cell counts above 350 cells/mm^3^ and viral loads under 200 copies/ml without ART. Four participants had CD4^+^ T‐cell counts under 350 cells/mm^3^ despite an undetectable viral load (<200 copies/ml), necessitating ART initiation (categorized as non‐viraemic progressors). Nineteen participants exhibited a classical phenotype of viraemic progression—low CD4^+^ T‐cell counts with high HIV‐2 plasma viral loads leading to ART initiation. Notably, three individuals initially demonstrated EC status for at least 5 years but eventually lost viral and immunological control, without apparent clinical trigger, and suppressed upon starting ART. Additionally, five participants had HIV‐1 and HIV‐2 dual infections, and one participant defied categorization by experiencing loss of control with an unexplained rebound in viraemia up to 89,000 copies/ml followed by EC status for more than 10 years without any ART use (re‐controller).

**Conclusions**: These data highlight relevant trajectories among ECs, and understanding the underlying mechanisms can inform decisions on pre‐emptive treatment and contribute to finding a cure for all people living with HIV.

### P211 Safety and immunogenicity of GS‐1966+GS‐1144 vaccines in virally suppressed adults living with HIV‐1: a phase Ib, randomized, placebo‐controlled study

Constance A. Benson^1^, Cynthia Brinson^2^, Moti N. Ramgopal^3^, Anthony M. Mills^4^, Edwin DeJesus^5^, Karin Jooss^6^, Pamela M. Odorizzi^7^, Susie S. Y. Huang^7^, Keith Flower^7^, Yongwu Shao^7^, Christiaan R. de Vries ^7^, Devi SenGupta^7^

^1^Division of Infectious Diseases and Global Public Health, University of California San Diego, San Diego, CA, USA. ^2^Central Texas Clinical Research, Austin, TX, USA. ^3^Infectious Disease, Internal Medicine, Midway Immunology and Research Center, Fort Pierce, FL, USA. ^4^Men's Health Foundation, Orlando, FL, USA. ^5^Orlando Immunology Center, Orlando, FL, USA. ^6^Gritstone Bio Inc, Emeryville, CA, USA. ^7^Virology Clinical, Gilead Sciences Inc, Foster City, CA, USA

**Background**: Therapeutic vaccination to enhance HIV‐specific T‐cell immunity may be a crucial component of a future combination HIV cure or long‐term remission strategy. GS‐1966+GS‐1144 is a novel heterologous vaccine regimen containing GS‐1966, a chimpanzee adenovirus, and GS‐1144, a self‐amplifying mRNA‐lipid nanoparticle, which both encode a novel conserved element HIV‐1 immunogen spanning Gag, Pol and Nef.

**Materials and methods**: This phase Ib, randomized, single‐blind, placebo‐controlled study evaluated the safety, tolerability and immunogenicity of GS‐1966+GS‐1144 in virologically suppressed people with HIV (PWH) on antiretroviral therapy (ART). The study included three cohorts with participants randomized 2:1 to receive GS‐1966+GS‐1144 or placebo. Cohorts 1 and 2 received a monovalent HIV‐1 immunogen; cohort 3 received a bivalent version (Figure 1). Participants maintained ART. Immunogenicity was evaluated longitudinally by interferon‐γ (IFN‐γ) ELISpot assay.

**Results**: The 49 participants enrolled included 45 males and four females with median (Q1, Q3) age 40 (33, 51) years and 10 (6, 17) years since HIV diagnosis. No serious adverse events occurred and the most common treatment‐emergent adverse events related to study drug were transient flu‐like symptoms. Treatment was discontinued in one participant who experienced Bell's palsy, which resolved in 14 days. Transient decreases in lymphocyte counts were observed 1 day after administration of either vaccine and resolved within 2 weeks. Pre‐existing vaccine‐specific T‐cell responses were detected in all cohorts. Median peak vaccine‐specific T‐cell responses were not significantly different between cohorts or compared with placebo, but cohort 3 had the numerically largest change from baseline to peak T‐cell response (Table 1).

**P211: Table 1:** GS‐1966+GS‐1144 immunogenicity

Total vaccine‐specific response (SFU/10(6) PBMCs)	Cohort 1 (*N* = 10)	Cohort 2 (*N* = 8)	Cohort 3 (*N* = 11)	Placebo (*N* = 14)	*p*‐value
Peak, median (Q1, Q3)	1169 (538, 2476)	1254 (725, 3251)	1860 (838, 3400)	1846 (1204, 2309)	0.53
Change from baseline to peak, median (Q1, Q3)	647 (0, 948)	526 (252, 1253)	986 (834, 1388)	740 (493, 1361)	0.50

Abbreviations: PBMC, peripheral blood mononuclear cell; Q, quartile; SFU, spot‐forming unit.

**Conclusions**: GS‐1966+GS‐1144 was safe and well‐tolerated in this first‐in‐human study of a novel therapeutic HIV‐1 T‐cell vaccine in chronic PWH on ART. There was no significant difference in immunogenicity by IFN‐γ ELISpot between vaccine recipients and placebo, which may be due, in part, to high levels of pre‐existing T‐cell responses to the vaccine insert and variability within cohorts. Cohort 3 recipients of the bivalent vaccine had the largest change from baseline to peak T‐cell responses. Further investigations are underway into additional metrics of T‐cell functionality. Future studies are needed to optimize immunogen design and therapeutic vaccine strategies for HIV cure.

**P211: Figure 1:**
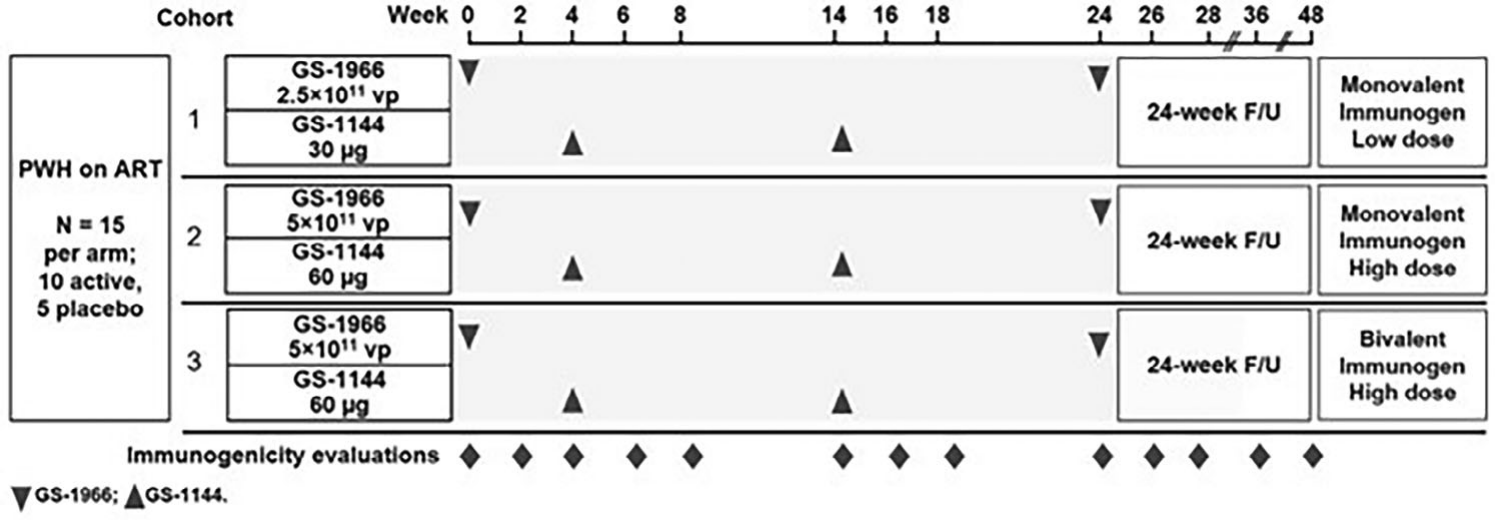
Study design. ART, antiretroviral; F/U, follow‐up; PWH, people with HIV; vp, viral particle.

### P212 Peptide‐induced apoptosis of latently infected cells and reduction of the HIV reservoir in people living with HIV: preliminary results of a clinical trial

Ricardo Sobhie Diaz ^1^, Mauro Schechter^2^, Daniel Elbirt^3^, Esmira Naftali^4^, Juliana Maricato^5^, Marcella Vassao de Almeida Baptista^5^, Juliana Galinskas^5^, Danilo Dias^5^, Alessandra Bassini^5^, Nadya Lisovoder^3^, James Hunter^5^, Eynat Finkelshtein^4^

^1^Infectious Diseases, Universidade Federal de Sao Paulo, Sao Paulo, Brazil. ^2^Infectious Diseases, Universidade Federal do Rio de Janeiro, Rio de Janeiro, Brazil. ^3^Clinical Immunology, Allergy and AIDS Center, Kaplan Medical Center, Rehovot, Israel. ^4^Code Pharma, Rehovot, Israel. ^5^Laboratório de Retrovirologia, Universidade Federal de Sao Paulo, Sao Paulo, Brazil

**Background**: Gammora^®^, an HIV‐1 integrase‐derived peptide, in association with a protease inhibitor (PI), has been shown to increase in vitro apoptosis of HIV‐infected cells selectively [1,2]. We investigated whether in vivo Gammora^®^ plus a PI‐based antiretroviral therapy (ART) can lead to a reduction of the HIV reservoir.

**Materials and methods**: This is a pilot open‐label randomized clinical trial. Six ART naïve individuals were randomized to receive 20 mg of Gammora^®^ SC plus darunavir‐based ART (*n* = 7) or ART only (*n* = 6). In the Gammora^®^ arm, participants had a 2‐week lead‐in period with Gammora^®^ given daily before ART was started, followed by 12 weeks of daily ART + Gammora^®^ every other day. The first 2 weeks of the trial were labelled w‐2 and w‐1 for both groups. ART was started for both arms at w‐0.

**Results**: Herein, we present the first 8 weeks of ART results. Viral load declined in all participants. In four of the seven Gammora^®^ arm participants, there was a one log_10_ decrease in total DNA in the first 8 weeks of combined therapy, accompanied by a highly significant increase in early and late apoptosis markers, which started only after ART was introduced (Figure 1). In these two participants, CD4 counts zigzagged up and down. CD4 counts steadily increased in all others, and total DNA remained unchanged.

**Conclusions**: Highly significant increases in apoptosis markers were accompanied by transient drops in CD4 counts and a one log_10_ decline in total HIV DNA in the Gammora^®^ arm participants, a magnitude of decline that would take many years to occur under normal circumstances. These results suggest that latently HIV‐infected cells were being rapidly eliminated since ART and Gammora^®^ suppressed HIV replication induces apoptosis exclusively of HIV‐infected cells. If confirmed by an ongoing, larger trial, Gammora^®^ may forward the path to cure HIV infection.

**P212: Figure 1:**
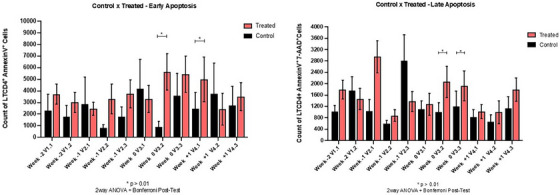
There was a higher elevation of early and late apoptosis markers once Gammora^®^ was associated with a PI‐based ART. W‐2 and W‐1 refer to the lead‐in period, and W‐0 and W‐1 to the ART period. Samples were collected thrice weekly (W‐2 1, W‐2 2, W‐2 3, etc).

**References**

1. Levin A, Hayouka Z, Friedler A, Loyter A. Specific eradication of HIV‐1 from infected cultured cells. AIDS Res Ther. 2010;7:31.

2. Rizza SA, Badley AD. HIV protease inhibitors impact on apoptosis. Med Chem. 2008;4(1):75‐9.

### P213 Perceptions of analytical treatment interruption among people with HIV using antiretroviral therapy in the United States

Karine Dube^1^, Sorana Segal‐Maurer^2^, Martha Gauthier^3^, Martin Duracinsky^4^, Kathy Vong^3^, Harlow Sharp^5^, Blaise Cureg^3^, Imani Weeks^3^, Kwanza Price ^6^, James Jarrett^6^, Caroline Burk^7^

^1^Division of Infectious Diseases and Global Public Health, University of California San Diego, San Diego, CA, USA. ^2^The Dr James J Rahal Jr Division of Infectious Diseases, New York‐Presbyterian Queens, Flushing, NY, USA. ^3^Patient Centered Outcomes, Lumanity, Boston, MA, USA. ^4^Health Economics Clinical Trial Unit, Patient‐Reported Outcomes Quality of Life (PROQOL), Paris, France. ^5^Patient Centered Outcomes, Lumanity, Long Beach, CA, USA. ^6^Global Value & Access, Gilead Sciences Inc, Foster City, CA, USA. ^7^Health Outcomes & Clinical Research, Gilead Sciences, Foster City, CA, USA

**Background**: Analytical treatment interruption (ATI) is often included in HIV cure‐related trials. Understanding the perceptions of ATI from people with HIV (PWH) provides insight that may be incorporated into clinical trial designs and considered in decision‐making as it relates to cure‐related research participation.

**Materials and methods**: All participants were sourced from the Rare Patient Voice recruitment agency. Qualitative interviews were conducted via Health Insurance Portability and Accountability Act (HIPAA)‐compliant video‐conferencing with PWH in the United States currently receiving antiretroviral therapy. Recruitment aimed to achieve diversity in gender, race, ethnicity and treatment experience. Participants were given a hypothetical scenario describing ATI and asked to explain the expected impact of being off standard‐of‐care HIV treatment, including a timeline for when the impact of being off treatment may be meaningful. Interview transcripts were analysed to identify the most common expected impacts of ATI.

**Results**: Fifteen participants representing various demographic backgrounds were interviewed: nine males (60%) and six females (40%) [at birth]; Black/African American (*n* = 8, 53.3%), White (*n* = 7, 46.7%), Asian (*n* = 1, 6.7%); average age (SD) 43.7 (13.7) years. Participants most commonly reported anticipated impacts on emotional wellbeing and relationships with intimate partners. Among the 30 anticipated impacts of ATI reported, 63.3% were coded as positive and 36.7% as negative. The most common expected impacts are presented with example quotes (Table 1). Anticipated positive impacts were more often associated with impacts on daily activities, social life and work/school, whereas negative impacts more often were associated with emotions, specifically with regard to concern about efficacy, and partner relationships regarding communication and sexual activity. Expected meaningful time off all treatments ranged from immediate to 1–2 years, with 33.3% of participants reporting that they would need to be off treatment for at least 3 months for it to be meaningful.

**P213: Table 1:** Most common expected impacts of ATI reported by PWH (*N* = 15)

Impact, *n* (%)	Example quotes from individual participants
Feel concerned about not maintaining viral suppression/efficacy concerns 9 (60.0%)	“I'd be happy to not have to take anything. But… in the back of my head, I would probably be worried, like, what's going to happen? Is it going to progress? Is it going to be dormant and then come eat me alive? And then come full force, 20 times full?”
Have more of a social life 4 (26.7%)	“I would just be happy to have any type of normalcy or closeness to… what I once had. And that would be me being able to work out—regularly work out, be fitness‐prone… [*to*] go out, maybe go out dancing or go hang out with friends. I miss hanging out with my friends.”
Less focused on pills 3 (20.0%)	“I was just going to say just even over to the aspect of not having to chug around or look for a bunch of pills or just when you hear pills, you only think about… you won't think about your pills as much because I don't take them like that. You now suppose you hear a pill jar shaking, you automatically think about your pills.”

**Conclusions**: Although PWH expressed concerns regarding the impacts of ATI on their emotional wellbeing and partner relationships, many positive outcomes were also anticipated from being off standard‐of‐care HIV‐related treatment. Concerns related to ATI can be used to facilitate discussions between PWH and their clinicians regarding participation in clinical research. These results may also inform future trial design including recruitment strategies and incorporating additional support during ATI.

### P214 Relationship between the functionality of HIV‐specific T‐cell response and HIV persistence: relevance in the context of cure strategies

Lucia Baquero, Leonel Cruces, Sofia Stover, Alejandra Urioste, Alejandro Czernikier, Tomás Langer, Yanina Ghiglione, Gabriela Turk, Natalia Laufer

INBIRS, Universidad de Buenos Aires‐CONICET, Buenos Aires, Argentina

**Background: **A functional HIV‐specific T‐cell response is key to controlling the infection. Current cure strategies under study point to enhance HIV‐specific T‐cell response, reduce HIV reservoirs and improve cell functionality through different strategies, including programmed cell death protein 1 (PD‐1) blockers. Here, we aim to evaluate the magnitude and functionality of the ex vivo HIV‐specific response and its relation with viral persistence and immune markers associated with activation and exhaustion, and HIV reservoir.

**Methods: **Peripheral blood mononuclear cells from nine individuals with HIV on antiretroviral treatment, with undetectable viral load for more than 2 years, were stimulated with HIV‐peptides for 14 days. The expression and co‐expression (polyfunctionality) of CD107A/B, tumour necrosis factor (TNF), interleukin‐2 (IL‐2), macrophage inflammatory protein 1β (MIP1β), interferon gamma (IFNγ) and granzyme B were evaluated by flow cytometry. Results were correlated with T‐cell immune phenotype (memory/effector sub‐populations), activation (cluster of differentiation 38 [CD38], human leukocyte antigen‐DR [HLA‐DR]) and exhaustion (PD‐1), and with the composition of the viral reservoir evaluated by intact proviral DNA assay (IPDA). Data were analysed using non‐parametric methods.

**Results: **Clinical characteristics are depicted in Table 1. No association was observed between the magnitude of the HIV‐specific T‐cell response and the total provirus per million CD4 T cells. However, lower polyfunctionality of the HIV‐specific CD8 response was correlated with higher percentages of PD‐1^+^ within effector memory (*p* = 0.031; *r* = −0.73), central memory (*p* = 0.017; *r* = −0.78), terminal effector (*p* = 0.003; *r* = −0.88) and stem‐cell memory (*p* = 0.0045; *r* = −0.87) CD8 T cells. Additionally, higher polyfunctionality of the HIV‐specific CD4 T cells correlated with higher CD4/CD8 ratios (*p* = 0.025; *r* = 0.75), lower levels of CD38^+^HLA‐DR^+^ CD4 T cells (*p* = 0.025; *r* = −0.75), higher levels of PD‐1^−^CD38^−^HLA‐DR^−^ CD8 T cells (*p* = 0.037; *r* = 0.72) and lower percentages of intact provirus (*p* = 0.049; *r* = −0.68).

**P214: Table 1:** Clinical characteristics

Women, *n* (%)	Age (years)^a^	CD4 count, cells/µl^a^	CD4/CD8 ratio^a^	Known time of HIV infection, years^a^	Time with undetectable viral load, years^a^
4 (44.4%)	30 (23−48.5)	772 (656.5−1128)	0.96 (0.64−1.489)	22 (12−25.5)	14 (6.5−18.75)

^a^Median (interquartile range; R1–R3).

**Conclusions: **These preliminary results indicate that lower PD‐1 expression is associated with improved HIV‐specific T‐cell polyfunctionality. Association between higher CD4 T‐cell polyfunctionality and lower intact provirus indicates that effective CD4‐CD8 collaboration could contribute to reduce the intact viral reservoir which is relevant for viral rebound. These results add evidence to the negative impact of immune activation on T‐cell response functionality and the potential benefit of PD‐1 blocking strategies to enhance the HIV‐specific response.

## Opportunistic infections and AIDS‐defining cancers

### P215 Comparative effectiveness of BIC/FTC/TAF versus other ART regimens in patients with AIDS‐defining conditions: virological suppression and immunological recovery at 24 and 48 weeks in the ACTUAS II study

Ignacio Perez Valero ^1^, Diana Corona Mata^1^, Angela Camacho Espejo^1^, Marina Gallo^1^, Inmaculada Jarrin^2^, Antonio Rivero Juarez^1^, Alejandro G. García‐Ruiz de Morales^3^, Chiara Fanciulli^4^, Luz Martín‐Carbonero^5^, Sonia Calzado Isbert^6^, Cristina Hernández Gutiérrez^7^, Víctor Asensi^8^, Antonio Rivero Roman^1^

^1^Enfermedades Infecciosas, Hospital Reina Sofia, IMIBIC, CIBERINFEC, Cordoba, Spain. ^2^Centro Nacional de Epidemiología, Instituto de Salud Carlos III, Madrid, Spain. ^3^Servicio Enfermedades Infecciosas, Hospital Ramon y Cajal, CIBERINFEC, Carlos III Health Institute, Madrid, Spain. ^4^Servicio de Microbiología Clínica y Enfermedades Infecciosas, Hospital General Universitario Gregorio Marañón, Instituto de Investigación Sanitaria Gregorio Marañón (IiSGM), CIBERINFEC, Carlos III Health Institute, Madrid, Spain. ^5^Unidad de VIH, Servicio de Medicina Interna, Hospital Universitario La Paz, IdiPAZ, CIBERINFEC, Carlos III Health Institute, Madrid, Spain. ^6^Servei de Malalties Infeccioses, Parc Taulí Hospital Universitari, Institut d'Investigació i Innovació Parc Taulí (I3PT‐CERCA), Departament Medicina, Universitat Autònoma de Barcelona, Sabadell, Spain. ^7^Unidad de Enfermedades Infecciosas, Hospital Universitario Príncipe de Asturias, Madrid, Spain. ^8^Hospital Universitario Central de Asturias, Oviedo, Spain

**Background**: There is a lack of comparative data on the effectiveness of bictegravir/emtricitabine/tenofovir alafenamide (BIC/FTC/TAF) versus other ART regimens in patients starting ART with AIDS‐defining conditions. This study aims to address this gap.

**Materials and methods**: The ACTUAS II study compared the effectiveness and safety of BIC/FTC/TAF versus other ART regimens in participants with AIDS‐defining conditions who started ART in the CORIS cohort between 2019 and 2021. The primary objective was to compare virological suppression (VS: HIV‐RNA <50 copies/ml) at 24 and 48 weeks. Secondary objectives included time to viral suppression, immunological recovery (IR: CD4 >200 cells/mm^3^) and ART discontinuations. Data were analysed using chi‐square tests, Kaplan‐Meier estimates and adjusted odds ratios (ORs), accounting for demographic and clinical variables.

**Results**: Between 2019 and 2021, 184 participants with AIDS‐defining conditions started ART with BIC/FTC/TAF (*n* = 90) or other regimens (*n* = 94). Baseline characteristics were similar between groups (Table 1). At 24 weeks, the VS rate for BIC/FTC/TAF was 75.6% compared to 56.5% for other regimens (*p* = 0.023; adjusted OR 0.36, 95% CI 0.16–0.78). At 48 weeks, VS rates were 87.2% for BIC/FTC/TAF and 81.6% for other regimens (*p* = 0.283). For IR, at 24 weeks, 47.7% on BIC/FTC/TAF achieved IR compared to 61.9% on other regimens (*p* = 0.087). At 48 weeks, 70.1% on BIC/FTC/TAF achieved IR compared to 82.7% on other regimens (*p* = 0.046; adjusted OR 2.25, 95% CI 1.01–4.05). Median time to VS was shorter for BIC/FTC/TAF at 17 weeks compared to 28 weeks for other regimens (*p* = 0.040) (Figure 1). Discontinuation rates at 48 weeks were lower for BIC/FTC/TAF (10.0%) compared to other regimens (36.2%), with significant differences (*p* < 0.001). Discontinuations due to adverse events were 3.3% for BIC/FTC/TAF and 8.5% for other regimens (*p* = 0.136).

**Conclusions**: BIC/FTC/TAF demonstrates superior efficacy in achieving virological suppression at 24 weeks compared to other ART regimens in individuals with previous AIDS‐defining conditions. By 48 weeks, the effectiveness of the regimens becomes comparable. BIC/FTC/TAF also achieved faster virological suppression and had lower discontinuation rates. These findings support the preferential use of BIC/FTC/TAF for initial ART in patients with previous AIDS‐defining conditions for better early virological outcomes and reduced treatment discontinuations.

**P215: Table 1:** Baseline characteristics

	With previous AIDS‐defining conditions, *N* = 184		
	BIC/FTC/TAF, *N* = 90	Other regimens, *N* = 94	*p*‐value
Sex (*N* [%])			
Male	77 (85.6)	80 (85.1)	0.931
Female	13 (14.4)	14 (14.9)	
Age, years (*N* [%])			
Median (IQR)	42 (35−54)	43 (35−53)	0.633
Transmission category (*N* [%])			
Men who have sex with men	53 (58.9)	43 (45.7)	0.083
Heterosexual	30 (33.3)	35 (37.2)	
Intravenous drug users	3 (2.2)	1 (1.1)	
Other/unknown	5 (5.6)	15 (16.0)	
Country of origin (*N* [%])			
Spain	55 (61.1)	48 (51.1)	0.203
No Spain	34 (37.8)	46 (48.9)	
Unknown	1 (1.1)	0	
CD4 count, cells/l (*N* [%])			
Median (IQR)	58 (26−153)	78 (29−207)	0.230
50	33 (36.7)	28 (29.8)	0.553
50	41 (45.6)	45 (47.9)	
Unknown	16 (17.8)	21 (22.3)	
Viral load, copies/ml (*N* [%])			
100,000	17 (18.9)	17 (18.1)	0.785
100,000	61 (67.8)	61 (64.9)	
Unknown	12 (13.3)	16 (17.0)	

**P215: Figure 1:**
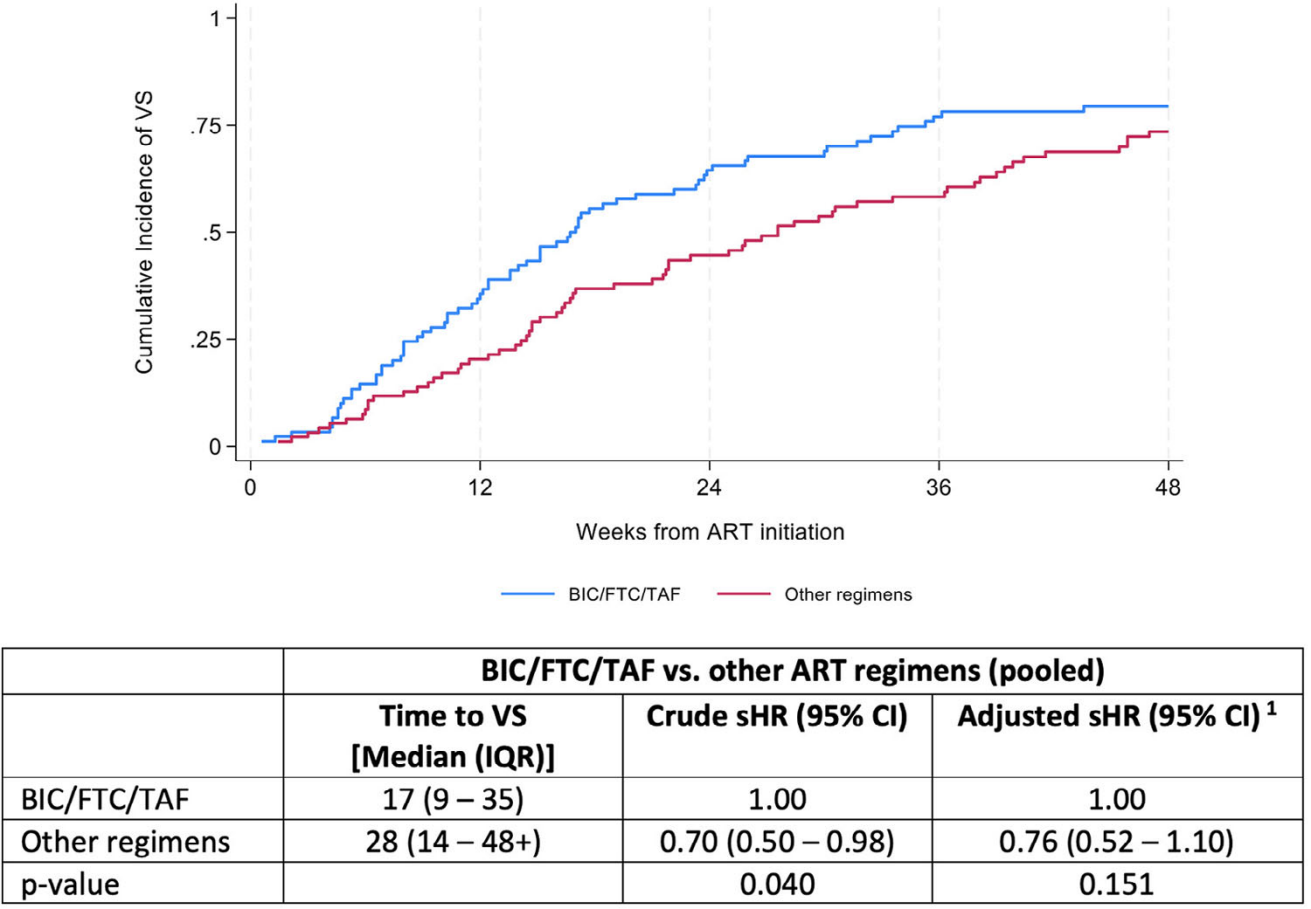
Time to viral suppression.

### P216 Trends in HIV diagnoses and AIDS‐defining conditions, Croatia 2009−2023

Josip Begovac, Ivana Benković, Sime Zekan, Nina Vrsaljko, Marija Santini

HIV/AIDS Department, University Hospital for Infectious Diseases, Zagreb, Croatia

**Background**: All persons living with HIV (PLWH) in Croatia (population: 3.9 million) are treated at one centre, the University Hospital of Infectious Diseases (UHID) in Zagreb. We aimed to assess the trend in presentation to care with an HIV diagnosis and AIDS‐defining conditions (ADCs) and to describe changes for the period 2009–2023.

**Methods**: The data were extracted from the electronic database at UHID. We included all PLWH who were treated at UHID in the period 2009–2023. Trends were examined by Joinpoint Trend Analysis Software and by the chi‐square test for trend.

**Results**: Of 1401 PLWH who entered care, 1205 have never been in care previously, and 196 were previously in care elsewhere (“in‐migration”). There was a decline in previously HIV‐undiagnosed PLWH entering care after 2015 (Figure 1). The number of PLWH who in‐migrated increased over the period reaching 41% (41 of 99) in 2023 (*p* < 0.001). Three hundred and fifty‐five PLWH had 456 episodes of an ADC (Table 1), with an insignificant trend over time (Figure 1). The most frequent ADC was *Pneumocystis jirovecii* pneumonia (*n* = 153, 33.6%) followed by oesophageal candidiasis and non‐Hodgkins's lymphoma (NHL) (both 42, 9.2%) and tuberculosis (*n* = 33, 7.2%). The proportion of tuberculosis cases among ADC ranged from 20% (5/24) in 2009 to no cases in 2021 and 2022 (*p* = 0.018). Of 456 ADC, 326 (71.5%) occurred within 3 months of the HIV diagnosis with a non‐significant trend over time (*p* = 0.111). The median time from HIV diagnosis to a non‐concurrent HIV/AIDS diagnosis was 6.6 (Q1–Q3, 3.3–11.3) years. PLWH with NHL had more frequently a non‐concurrent HIV/AIDS diagnosis (23/42) compared to all other ADC (107/414, *p* < 0.001), and had HIV‐1 RNA less than 200 copies/ml in 42.9% (18/42) at diagnosis. Fifty‐five (15.5%) persons died within 6 months and 64 (18.0%) within 12 months from the first AIDS diagnosis (*p* = 0.438 and 0.502, respectively).

**P216: Table 1:** Main characteristics of 355 persons living with HIV with an AIDS‐defining condition, Croatia, 2009−2023

Characteristics	Total (*N* = 355)
Gender, males	322 (90.7)
Age at clinical AIDS event, years, median (Q1−Q3)	43.0 (36.0−50.7)
Mode of acquisition	
MSM	278 (78.3)
Heterosexual	53 (14.9)
PWID	7 (2.0)
Other/unknown	17 (4.8)
CD4 cells, per mm^3^, median (Q1−Q3)	37.0 (16.0−94.0)
CD4 cell categories, mm^3^	
<50	193 (59.4)
50−200	94 (8.9)
>200	38 (11.7)
HIV‐1 RNA copies/ml	
≥1000	288 (87.8)
<1000	40 (12.2)
Calendar years of clinical AIDS diagnosis	
2009−2011	70 (19.7)
2012−2014	76 (21.4)
2015−2017	76 (21.4)
2018−2020	72 (20.3)
2021−2023	61 (17.2)
Calendar years of inclusion into care	
<2009	44 (12.4)
2009−2013	111 (31.3)
2014−2018	119 (33.5)
2019−2023	81 (22.8)
Concurrent HIV and AIDS diagnosis, yes	257 (72.4)
Living in Zagreb, yes	121 (34.1)
Died within 6 months of first clinical AIDS, yes	55 (15.5)
Died within 12 months of first clinical AIDS, yes	64 (18.0)
Number of clinical AIDS events per person	
One	275 (77.5)
Two	61 (17.2)
Three	17 (4.8)
Four	2 (0.6)

*Note*: Values are frequencies with percentages or medians with Q1 to Q3. Some frequencies do not add up to the total because of missing data. Data on AIDS, CD4 cell counts and calendar year of diagnosis refer to the first clinical event.

Abbreviations: MSM, men who have sex with men; PWID, persons who inject drugs; Q1, first quartile; Q3, third quartile.

**Conclusions**: Since 2015, there has been a significant decline in the rate of previously HIV‐undiagnosed PLWH entering care at UHID. Presentation with tuberculosis declined, whereas NHL compared to other ADC occurred more frequently. Interventions to sustain the decrease of new HIV infections and to decrease AIDS presentations are needed.

**P216: Figure 1:**
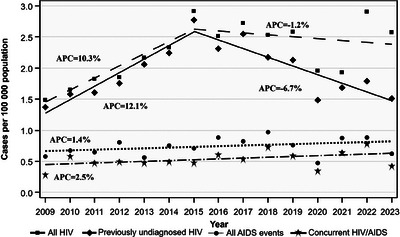
The trend in presentation to care with HIV and AIDS‐defining diseases, Croatia, 2009−2023. Lines represent modelled crude rates using joinpoint regression and symbols are observed rates. “All HIV” includes previously diagnosed and newly diagnosed persons. APC, annual percent change.

### P217 Residual disease challenge efforts to achieving successful cervical dysplasia treatment in women living with HIV in Botswana

Patricia Rantshabeng, Nambumba Loide Amundaba, Moses Rugemalila, Alemayehu Eshetu, Lynnette Kyokunda

Department of Pathology, University of Botswana, Gaborone, Botswana

**Background**: Cervical cancer is the fourth most common malignancy in women globally, and the most common in women in Botswana, especially those living with HIV. Cervical cancer is preceded by the CIN3 lesion and commonly treated with loop electrosurgical excision procedure (LEEP), which is widely accepted as a safe and effective method for the treatment of cervical intraepithelial lesions globally. Women living with HIV have been reported to experience residual disease and recurrence after treatment. The aim of this study was to determine the proportion of women with residual disease and predictive factors for recurrence after treatment with LEEP in WLWH.

**Materials and methods**: Ethical approval was sought from the University of Botswana and Princess Marina Hospital Institutional Boards. A study permit and access to specimens and patient records was sought from the Ministry of Health. Archived formalin‐fixed paraffin‐embedded (FFPE) cervical tissues from women diagnosed with CIN2/3 were enrolled into the study. Clinical data associated with samples was extracted from electronic medical records. Tissue sections were cut and stained with p16 and haematoxylin and eosin (H&E) before review with a consultant anatomic pathologist.

**Results**: Eighty FFPE tissues were enrolled from women aged 29–71 years (median 41 years). Eighty‐six percent were WLWH and on HAART; 97% were virally suppressed and 81% had CD4 cell counts >350/µl. Sixty‐one percent had positive surgical margins; 58% CIN3 with glandular involvement and 75% were p16 positive. Furthermore, 46.3% of the women had recurrent CIN3 after 6–12 months of follow‐up.

**Conclusions**: This study reports an increased failure rate of CIN3 excisional treatment as indicated by positive margins. This further confirms ongoing challenges with achieving successful cervical dysplasia treatment in WLWH, adding to the rising burden of cervical cancer cases in Botswana despite viral suppression and calls for increased efforts towards improved therapies for WLWH to achieve cervical cancer elimination target.

### P218 Evaluation of the new AltoStar^®^ HHV‐7/‐8 PCR Kit 1.5 RUO for the quantitative detection of HHV‐8 DNA in plasma, whole blood and PBMCs

Martin Obermeier ^1^, Özlem Yildirim^1^, David Mwangi^1^, Malik Prentice^1^, Stefan Breuer^1^, Pascal Migaud^2^, Robert Ehret^1^

^1^Molecular Diagnostics, Medizinisches Infektiologiezentrum Berlin, Berlin, Germany. ^2^Infectiology, St. Joseph Krankenhaus, Berlin, Germany

**Background**: In persons whose immune system is compromised, human gammaherpesvirus 8 (HHV‐8) is the causative agent for Kaposi's sarcoma, multicentric Castleman's disease and primary effusion lymphoma. Most of the assays used to detect HHV‐8 DNA are qualitative laboratory developed assay. We evaluated the research use only quantitative AltoStar^®^ HHV‐7/‐8 PCR Kit 1.5 RUO (Altostar).

**Materials and methods**: Thirty‐four anonymized residual sample material from persons with known HHV‐8 infection was either tested as whole blood sample, plasma or separated peripheral blood mononuclear cells (PBMCs) and viral load was compared between those compartments. Dilution series of three different cell lines infected with HHV‐8 were generated and the quantity of HHV‐8 DNA in these samples was determined by digital PCR before testing with the Altostar. Limit of detection was calculated using probit 95% hit rate.

**Results**: Limit of detection of the assay was calculated at 83 copies/ml (95% probit hit rate). The assay showed good linearity between 100 and 1,000,000 copies/ml. In 24 (70%) of the plasma samples, HHV‐8 DNA could be detected between 83 and 1,900,000 copies/ml. Detection rates were lower in whole blood (40%) and PBMCs (51%). If HHV‐8 DNA could be detected in whole blood, the viral load was in 75% of the cases higher than in plasma.

**Conclusions**: The new Altostar assay is a substantial addition to the repertoire of HHV‐8 testing and allows with a high flexibility to detect HHV‐8 DNA in different blood compartments. Although the limit of detection in whole blood is decreased due to the lower possible input volume in the used nucleic acid extraction system, additional information can be gained, especially from the perspective of clonal expansion of infected cells due to lymphoproliferation by testing this compartment.

### P219 **High proportion of circulating CD8^+^CD28** ^−^ **T cells predicts inferior prognosis in AIDS‐related non‐Hodgkin lymphoma**

Juanjuan Chen, Jie Peng, Zixin Kang, Xin Tao, Shuting Wu

Department of Infectious Diseases, Nanfang Hospital, Southern Medical University, Guangzhou, China

**Background**: Tumour cells and the immune system interact in dynamic and intricate ways, but the precise immune cell types influencing AIDS‐related non‐Hodgkin lymphoma (AR‐NHL) prognosis remain elusive. Furthermore, the disparities in circulating lymphocyte subsets between AR‐NHL and HIV‐negative NHL patients are poorly understood. This study aimed to characterize the immunophenotype distribution in AR‐NHL compared to the HIV‐seronegative NHL setting and correlate these findings with prognosis, HIV status and lymphoma burden. Such investigations are crucial for the development of tailored and effective personalized immunotherapy strategies.

**Materials and methods**: This observational, longitudinal cohort study enrolled 31 newly diagnosed adult AR‐NHL patients and 52 matched HIV‐negative NHL populations. Peripheral lymphocyte immunophenotyping was dynamically assessed during immunochemotherapy using flow cytometry. Multivariate survival analysis was performed with the Cox regression model.

**Results**: In our study, AR‐NHL patients exhibited comparable overall survival and progression‐free survival to those observed in HIV‐negative NHL individuals. Compared to HIV‐seronegative NHL cohorts, AR‐NHL patients tended to be younger with elevated levels of β2‐microglobulin (β2‐MG), erythrocyte sedimentation rate and EBV‐encoded RNA (EBER). At diagnosis, AR‐NHL populations demonstrated decreased counts of CD4^+^ T cells and CD4/CD8 ratio, along with reduced percentages of Treg cells, naive CD45RA^+^ and memory CD45RO^+^ CD4^+^ T cells. Conversely, they displayed increased proportions of Tregs/CD4, CD8^+^, CD8^+^CD28^+^ and CD8^+^CD28^−^ T cells. Additionally, these alterations in Treg cells, CD4^+^, memory CD45RO^+^ CD4^+^, CD8^+^, CD8^+^CD28^+^ and CD8^+^CD28^−^ T cells persisted throughout immunochemotherapy. Notably, multivariate analysis revealed that a heightened presence of initial CD8^+^CD28^−^ T cells independently predicted an unfavourable prognosis in AR‐NHL patients (Table 1). This subset of T cells was strongly correlated with aggressive clinical indicators, including elevated β2‐MG, decreased albumin levels, diminished CD4/CD8 ratio, high International Prognostic Index score and positivity for EBER.

**Conclusions**: The prognosis of AR‐NHL patients significantly improved with high‐intensity chemotherapeutic regimens in the combined antiretroviral therapy era, becoming comparable to that of the general NHL population. AR‐NHL individuals exhibited distinct premature immunosenescence phenotypes compared to HIV‐negative patients. A high proportion of circulating CD8^+^CD28^−^ T cells served as an independent biomarker for predicting outcomes in AR‐NHL, potentially associated with heightened viral exposure and chronic immune activation.

**P219: Table 1:** Baseline demographics and clinical characteristics of patients with AR NHL and HIV‐negative NHL

Characteristics, no. (%)	NHL (*n* = 52)	AR‐NHL (*n* = 31)	*p*‐value
Male	41 (78.8)	25 (80.6)	0.844
Age, years [median, IQR]	56 [51 60]	41 [34–54]	**0.002**
>60	11 (21.2)	5 (16.1)	0.575
Lymphoma subtype			0.965
DLBCL	42 (80.8)	25 (80.6)	
BL	6 (11.5)	4 (12.9)	
NKTL	4 (7.7)	2 (6.5)	
Ann Arbor stage III/IV	48 (92.3)	27 (87.1)	0.464
Extra‐nodal involvement ≥2	36 (69.2)	21 (67.7)	0.888
LDH, U/l	254 [168–462]	358 [232–1069]	0.068
>ULN	30 (57.7)	20 (64.5)	0.539
ECOG PS ≥2	20 (38.5)	11 (35.5)	0.786
IPI			0.711
Low risk (0–1)	10 (19.2)	6 (19.4)	
Low‐middle risk (2)	12 (23.1)	9 (29.0)	
Middle‐high risk (3)	6 (11.5)	3 (9.7)	
High risk (4–5)	24 (46.2)	13 (41.9)	
B symptoms (Yes)	20 (38.5)	10 (32.3)	0.569
Bulky tumour (≥7.5 cm)	10 (19.2)	9 (29.0)	0.304
Bone marrow involvement	7 (13.5)	8 (25.8)	0.157
CNS involvement	9 (17.3)	8 (25.8)	0.353
β2‐MG, mg/l [median, IQR]	2.80 [2.24–4.21]	3.91 [3.16–6.05]	**0.002**
ESR, mm/hour [median, IQR]	21 [11–50]	52 [15–85]	**0.032**
EBER (Positive)	6 (11.5)	12 (38.7)	**0.004**
LMR [median, IQR]	2.22 [1.50–3.79]	2.21 [0.91–3.72]	0.572
<2.12	24 (46.2)	15 (48.4)	0.844
PLR [median, IQR]	169.34 [110.52–272.83]	204.82 [132.45–459.46]	0.051
≥204.82	21 (40.1)	16 (51.6)	0.319
ALB, g/l [median, IQR]	36.55 [32.85–39.90]	35.70 [30.20–39.40]	0.164
<35.70	19 (36.5)	15 (48.4)	0.288
CD4 count, cells/µl *[median, IQR]	467 [282–693]	172 [123–247]	**<0.001**
<200	8 (18.2)	21 (67.8)	**<0.001**
CD4/CD8 [median, IQR]	1.24 [0.85–2.05]	0.31 [0.22–0.38]	**<0.001**
≥0.31	45 (86.5)	16 (51.6)	**<0.001**
cART	–	29 (93.5)	–
HIV‐1 RNA, copies/ml [median, IQR]	–	36,500 [1416–505,000]	–
≥100,000	–	11 (35.5)	–
20,100,000		14 (45.1)	
Undetectable	–	6 (19.4)	–

Abbreviations: ALB, albumin; β2‐MG, β2 microglobulin; BL, Burkitt lymphoma; cART, combined antiretroviral therapy; CNS, central nervous system; DLBCL, diffuse large B‐cell lymphoma; EBER, Epstein Barr virus (EBV)‐encoded RNA; ECOG PS, Eastern Cooperative Oncology Group performance status; ESR, erythrocyte sedimentation rate; IPI, International Prognostic Index; LDH, lactate dehydrogenase; LMR, lymphocyte to monocyte ratio; NKTL, natural killer/T‐cell lymphoma; PLR, platelet to lymphocyte ratio.

### P220 Pneumocystis pneumonia cases in HIV and non‐HIV individuals in a peripheral hospital in Portugal: an 11‐year experience

Gustavo Lemos Correia ^1^, Francisco Soares Laranjeira^2^, Raquel Tavares^1^, Filipa de Oliveira Nunes^1^, Liliana Alves^1^, Rita Sérvio^1^, Paulo Rodrigues^1^

^1^Infectious Diseases, Hospital Beatriz Ângelo, Loures, Portugal. ^2^Internal Medicine, Hospital da Luz, Lisboa, Portugal

**Background**: Pneumocystis pneumonia (PCP) continues to be one of the most common fungal diseases in immunosuppressed people, particularly in people living with HIV (PLWHIV). However, with effective dual combination antiretroviral therapy (ART) and recommendations for primary prophylaxis for individuals with HIV who have a CD4 T lymphocyte count of less than 200/µl, the prevalence of this respiratory infection has decreased in this population, in contrast to non‐HIV immunocompromised individuals, whose prevalence has increased due to new biological therapies.

**Materials and methods**: We conducted a retrospective review of all cases of PCP diagnosed at tertiary hospital in Portugal over an 11‐year period, from March 2012 to December 2023. The study included both HIV‐positive and HIV‐negative individuals who were diagnosed with PCP based on clinical presentation, radiological findings and microbiological tests, including direct fluorescent antibody staining or polymerase chain reaction assays from induced sputum or bronchoalveolar lavage samples. For each case, we collected demographic, clinical, radiological, microbiological and therapeutic data.

**Results**: There were reported 47 patients diagnosed with PCP. The ratio of non‐HIV to HIV PCP cases showed an increase over the years from 0 (2012–2015) to peaks of 0.5 in 2016, 1 in 2022, and then 0.5 in 2023, indicating a growing proportion of non‐HIV cases. In the HIV‐positive population, 55.3% were naïve individuals and 44.7% were treatment‐experienced individuals that either were on ART or had previous ART regimens taken. The mean CD4^+^ count at the time of the diagnosis was 45.8 cells/µl and the mean viral load was 476,144.1 copies/ml. From the treatment‐experienced individuals with PCP diagnosis, 88.2% were people with poor adherence. Out of the 47 patients with PCP, four patients (8.5%) died. The mortality rate was 5.26% (two out of 38) among HIV‐positive and 22.22% (two out of nine) among HIV‐negative individuals, with no statistically significant difference in mortality rates between the two groups (*p* = 0.329).

**Conclusions: **The study displays the importance of implementation of prophylaxis of PCP in non‐HIV immunosuppressed patients as well as in PLWHIV with CD4 T‐lymphocytes count less than 200/µl to prevent PCP and, therefore, reduce burden and mortality of this fungal disease.

### P221 Breaking the age and immunological limits: enhancing cancer treatment in people living with HIV with anti‐PD‐(L)1 therapies

Sibo Li ^1^, Wenjing Wang^1^, Yue Yuan^2^, Xiaojie Huang^1^, Hao Wu^1^

^1^Center for Infectious Disease, Beijing Youan Hospital, Capital Medical University, Beijing, China. ^2^Dermatology, The Affiliated Hospital of Qingdao University, Qingdao, China

**Background**: Many studies have confirmed the good efficacy of anti‐PD‐(L)1 therapy. However, because people living with HIV (PLWH) have impaired autoimmunity and may experience more adverse events after receiving therapy, they are often excluded from studies. To systematically determine the safety and efficacy of anti‐PD‐(L)1 therapy for PLWH.

**Methods**: PubMed, the Web of Science and the Cochrane Library were systematically searched from inception to 7 May 2023, for studies reporting relevant data, including the objective response rate (ORR) and the incidence of immune‐related adverse events (irAEs). Either the fixed‐effect model or the random‐effect model was selected based on the heterogeneity. StataMP 14.0 was utilized to compute the 95% confidence intervals (CIs) for the effect sizes.

**Results**: There were seven prospective clinical trials and 11 retrospective case series involving 254 PLWH with cancer. The ORR of patients receiving anti‐PD‐(L)1 therapy was 0.25 ([95% CI 0.19–0.31]; I^2^ = 0%). PLWH ≤50 years old (0.58 [95% CI 0.38–0.78]) responded better than elderly PLWH (0.21 [95% CI 0.11–0.31]). AIDS‐defining cancers (ADCs), such as NHL (0.63 [95% CI 0.40–0.86]; I^2^ = 36.7%) and KS (0.46 [95% CI 0.27–0.65]; I2 = 64.4%), demonstrated a relatively high level of efficacy when treated with anti‐PD‐(L)1 therapy. PLWH with cancer seemed to respond better to pembrolizumab (0.25 [95% CI 0.13–0.36]; I^2^ = 34.6%) as monotherapy. Pembrolizumab+pomalidomide and nivolumab+ipilimumab have also demonstrated positive outcomes. The incidence of irAEs was 0.38 (95% CI 0.29–0.48; I^2^ = 0%), and the incidence of grade 3 or higher irAEs was 0.08 (95% CI 0.03–0.13; I^2^ = 0%) in PLWH, similar to that in general patients. No immune reactivation of the inflammatory syndrome was observed during anti‐PD‐(L)1 therapy. The baseline CD4^+^ T‐cell count and CD4^+^/CD8^+^ T‐cell ratio did not affect treatment efficacy or safety. The majority of treated patients exhibited stable CD4^+^ T‐cell counts (Figure 1).

**Conclusions**: Anti‐PD‐(L)1 therapy can be a safe and effective treatment for PLWH with cancer, regardless of the CD4^+^ T‐cell count and CD4/CD8 ratio. Pembrolizumab may be a promising treatment for PLWH with cancer. ADCs show a better response to anti‐PD‐(L)1 therapy. More suitable immunosuppressive treatment strategies are needed for elderly PLWH to enhance efficacy.

**P221: Figure 1:**
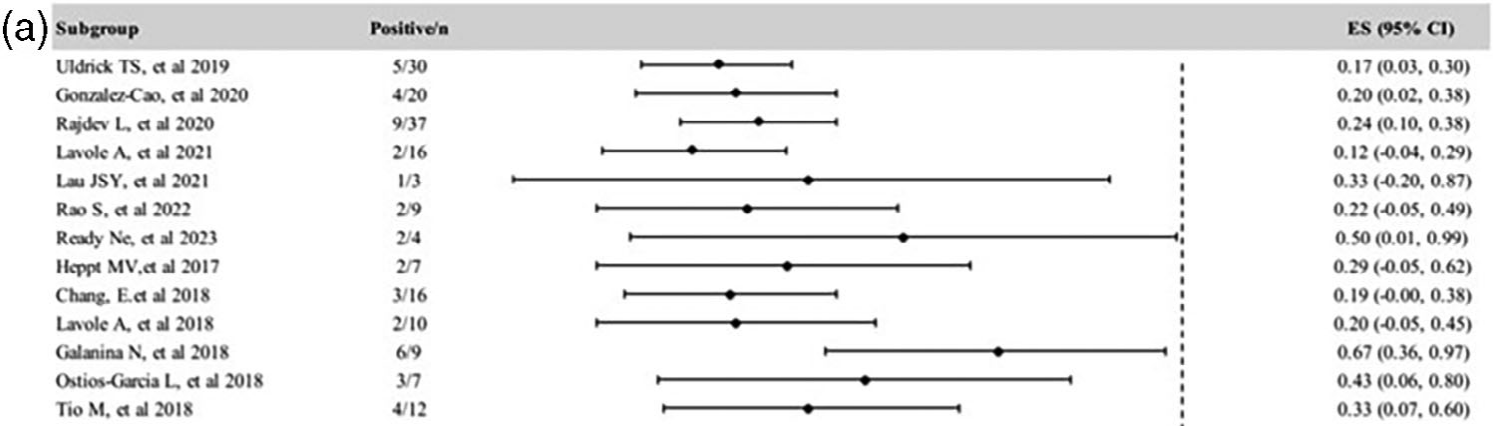
Forest plot of the efficacy data of anti‐PD‐(L)1 therapy. (a) The pooled ORR of patients receiving anti‐PD‐(L)1 therapy; (b) the ORR of patients receiving anti‐PD‐(L)1 therapy based on different ICI types.

### P222 Cryptosporidiosis in HIV/AIDS patients in Belgrade, Serbia: a single‐centre study

Ivana Gmizic^1^, Zorica Dakic^2^, Ivan Rajkovic ^1^, Jovana Ranin^1^, Borko Pavlica^3^, Snezana Jovanovic^2^, Jovan Ranin^1^

^1^Clinic for Infectious and Tropical Diseases, University Clinical Center of Serbia, Belgrade, Serbia. ^2^Department of Medical Microbiology, University Clinical Center of Serbia, Belgrade, Serbia. ^3^University Clinical Center of Republika Srpska, Banja Luka, Bosnia and Herzegovina

**Background**: *Cryptosporidium* species can cause chronic diarrhoea in people living with HIV (PLWH) and be associated with advanced immunodeficiency [1]. The prevalence of cryptosporidiosis among PLWH is decreasing in the era of antiretroviral treatment (ART), with the estimated global prevalence of 7.6% [2]. The main objective of our study is to determine the prevalence and risk factors for cryptosporidiosis among PLWH in Belgrade, Serbia.

**Materials and methods**: Our cross‐sectional study included all cases of cryptosporidiosis among PLWH in our centre between January 2011 and April 2024 (Figure 1). Diagnose was made by microscopically identifying cryptosporidial oocysts in faecal smears stained with a modified Ziehl‐Neelsen stain. Depending on the availability, faecal samples were also tested with a rapid diagnostic test (RDT) for the qualitative detection of Cryptosporidium‐specific antigens (RIDA^®^QUICK or CerTest). Molecular detection of Cryptosporidium genus‐specific nucleic acid using rapid multiplexed nested RT‐PCR via the BioFire^®^ Gastrointestinal Panel was additionally recently introduced.

**Results**: Multiple faecal samples from 511 PLWH with a diarrhoeal syndrome were microscopically examined, and cryptosporidial oocysts were found in 18 cases (3.5%). The RDT test was performed in 11 cases, four of which were negative. Multiplex PCR was used in four cases, and the results were consistent with microscopy. Included patients were predominantly male (77.8%), 43±11.9 years old, half were newly diagnosed with average 67±62 cells/µl CD4^+^ T cells, while the other half were PLWH with known HIV status for approximately 106±70 months; the majority of them (55.5%) were not on ART ≥6 months at the time of cryptosporidiosis; symptoms lasted longer in newly diagnosed PLWH (4.4 vs. 2.4 months, *p* = 0.039), including diarrhoea (94.4%), weight loss (83.3%) and fever (44.4%); the majority of patients were undisclosed on the mode of HIV acquisition or on the epidemiological risks for cryptosporidiosis. Two patients with newly discovered HIV infections died from a septic condition, while the others fully recovered after receiving symptomatic treatment and initiating ART.

**Conclusions**: To our knowledge, this is the first study to estimate the prevalence of cryptosporidiosis among Serbian PLWH. Our results show relatively high prevalence of cryptosporidiosis, given that the 50–60% of newly diagnosed PLWH are late presenters.

**P222: Figure 1:**
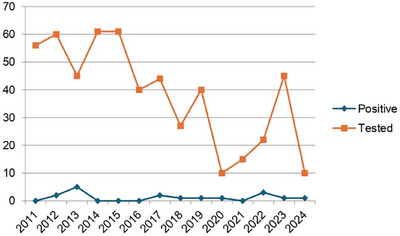
Timeline of microbiology testing and cryptosporidiosis cases.

**References**

1. Ahmadpour E, Safarpour H, Xiao L, Zarean M, Hatam‐Nahavandi K, Barac A, et al. Cryptosporidiosis in HIV‐positive patients and related risk factors: a systematic review and meta‐analysis. Parasite. 2020;27:27.

2. Dong S, Yang Y, Wang Y, Yang D, Yang Y, Shi Y, et al. Prevalence of Cryptosporidium infection in the global population: a systematic review and meta‐analysis. Acta Parasitologica. 2020;65(4):882‐9.

## Clinical pharmacology

### P223 Effectiveness of a dual therapy based on DTG/3TC on reduction of the viral reservoir, immune recovery and immune activation compared with a triple antiretroviral therapy based on DTG/TAF/FTC in patients with HIV infection without prior treatment

Abraham Saborido‐Alconchel ^1^, María Trujillo‐Rodríguez^1^, Ana Serna‐Gallego^1^, Esperanza Muñoz‐Muela^1^, Inmaculada Rivas‐Jeremias^1^, Ana Álvarez‐Ríos^2^, Nuria Espinosa^1^, Cristina Roca^1^, Marta Herrero^1^, César Sotomayor^1^, Alicia Gutierrez‐Valencia^1^, Luis Fernando López‐Cortés^1^

^1^Institute of Biomedicine of Seville, Virgen del Rocio University Hospital, CSIC, University of Seville, Clinical Unit of Infectious Diseases, Clinical Microbiology and Parasitology, Seville, Spain. ^2^Department of Clinical Biochemistry, Virgen del Rocío University Hospital, Seville, Spain

**Background**: In clinical trials such as GEMINI 1 and 2 [1], the virological success of DTG/3TC in treatment‐naïve patients has been demonstrated, defined as achieving or maintaining undetectable plasma HIV‐1 RNA. However, whether the initial treatment influences viral reservoir dynamics, inflammatory profile and immune recovery remains.

**Methods**: The TRIDUNA (TRIple versus Dual therapy in treatment‐NAïve patients) was a randomized, open‐label, multicentre, phase IV clinical trial enrolling adult treatment‐naïve PWH (NCT04295460) in which participants started treatment with DTG/3TC (2DR) or DTG/TAF/FTC (3DR). CD4^+^ and CD8^+^ activation (CD38^+^/HLA‐DR+), apoptosis (Annexin V), proliferation (Ki67), senescence (CD57), and exhaustion (PD‐1/TIGIT/TIM‐3/LAG‐3), monocyte activation (sCD14/sCD16), β2‐microglobulin, inflammatory plasma markers: hsPCR, IL‐1β, IL‐6, IL‐8, TNF‐α, IFN‐γ, IP‐10, D‐dimer (different immunoassays), quality and quantity of viral reservoir (IPDA and IVRA), and immune reconstitution (ΔCD4^+^/CD8^+^) were assessed after 12 months of treatment. Statistical analysis: χ2, Mann‐Whitney U‐test and general linear model for repeated measures. Results were expressed as median (M), *p*‐value.

**Results**: Seventy‐four treatment‐naïve PWH were enrolled. Sixty‐six PWH were evaluable, of whom 33 started ART with 2DR and 33 with 3DR. Baseline characteristics: 97% were male in each group. 2DR: CD4^+^ count of 394 cells/ml, a CD4^+^/CD8^+^ ratio of 0.57 and a viral load of 57,300 copies/ml. 3DR: CD4^+^ count of 421 cells/ml, a CD4^+^/CD8^+^ ratio of 0.47 and a viral load of 53,900 copies/ml. There were no differences in other basal characteristics. After 12 months of follow‐up, there were no differences between both treatment arms in immune activation, apoptosis, proliferation, senescence, exhaustion of CD4^+^ and CD8^+^ T cells, inflammatory profile, quality and quantity of viral reservoir, and immune recovery.

**Conclusions**: After this comprehensive assessment, we did not find differences between 2DR and 3DR in viral reservoir dynamics, CD4^+^ and CD8^+^ phenotypic changes, inflammatory profile and immune recovery.

Abbreviations: 2DR, two‐drug regimen; 3DR, three‐drug regimen; HLA‐DR, human leukocyte antigen‐DR; hsCRP, high sensitivity C reactive protein; IFN‐γ, interferon‐γ; IL‐1β, interleukin‐1β; IL‐6, interleukin‐6; IL‐8, interleukin‐8; IP‐10, interferon gamma‐induced protein 10; IPDA, intact provirus DNA assay; IVRA, intact viral RNA assay; LAG‐3, lymphocyte‐activation gene 3; PD‐1, programmed death‐1; sCD14, soluble cluster of differentiation 14; sCD16, soluble cluster of differentiation 16; TIGIT, T‐cell immunoreceptor with Ig and ITIM domains; TIM‐3, T‐cell immunoglobulin and mucin containing protein‐3; TNF‐α, tumour necrosis factor‐α.

**Reference**

1. Cahn P, Madero JS, Arribas JR, Antinori A, Ortiz R, Clarke AE, et al.; GEMINI Study Team. Dolutegravir plus lamivudine versus dolutegravir plus tenofovir disoproxil fumarate and emtricitabine in antiretroviral‐naive adults with HIV‐1 infection (GEMINI‐1 and GEMINI‐2): week 48 results from two multicentre, double‐blind, randomised, non‐inferiority, phase 3 trials. Lancet. 2019;393(10167):143‐55.

### P224 Pharmacokinetics of oral islatravir (ISL) plus lenacapavir (LEN) given once weekly in an open‐label, active‐controlled, phase II study of virologically suppressed people with HIV‐1

Diane Longo^1^, Gillian Gillespie ^2^, Michelle Pham^1^, Stephanie Klopfer^3^, Haeyoung Zhang^4^, Ramesh Palaparthy^4^, Angela S. Y. Liu^5^, Randolph P. Matthews^2^, Cyril Llamoso^2^, Elizabeth G. Rhee^2^, Aubrey Stoch^2^, Dhananjay D. Marathe^4^, Ryan Vargo^1^

^1^Data Science, Merck & Co., Inc., Rahway, NJ, USA. ^2^Clinical Research, Merck & Co., Inc., Rahway, NJ, USA. ^3^Biostatistics, Merck & Co., Inc., Rahway, NJ, USA. ^4^Clinical Pharmacology, Gilead Sciences, Foster City, CA, USA. ^5^Biostatistics, Gilead Sciences, Foster City, CA, USA

**Background**: Once‐weekly (QW) oral ISL, a nucleoside reverse transcriptase translocation inhibitor, plus LEN, an HIV‐1 capsid inhibitor, is being evaluated in a phase II study of participants switching from once‐daily oral bictegravir/emtricitabine/tenofovir alafenamide (BIC/FTC/TAF). QW ISL+LEN maintained high rates of virological suppression and was generally well tolerated through week 24. Pharmacokinetic (PK) and pharmacodynamic (PD) analyses from this study are presented here.

**Materials and methods**: GS‐US‐563‐6041 (NCT05052996) is an open‐label, active‐controlled study in people with HIV‐1 virologically suppressed on BIC/FTC/TAF for ≥24 weeks. Participants (*N* = 104) were randomized (1:1) to switch to QW oral ISL (2 mg) plus LEN (300 mg), with LEN 600‐mg loading doses on days 1 and 2, or to continue once‐daily oral BIC/FTC/TAF for 48 weeks. Sparse PK samples were collected from all participants on day 1 (∼1‐hour post‐dose) and at any time on weeks 4, 8, 12, 18 and 24. An intensive PK sub‐study was conducted in 14 participants in the ISL+LEN group, with sample collection for plasma ISL and LEN on day 1, day 2 and week 12. A population PK (popPK) model developed from ISL phase I–III studies was used to predict ISL‐triphosphate (TP) exposures from plasma ISL concentrations. PK/PD models developed from ISL phase II–III studies were used to simulate CD4^+^ T‐cell and lymphocyte counts using popPK‐predicted ISL‐TP exposures.

**Results**: Based on popPK model projections, steady‐state mean ISL‐TP C_trough_ (5.60 µM) remained well above the inhibitory quotient (IQ)5 (1.25 µM) for M184V/I variants and IQ5 (0.25 µM) for wild‐type HIV‐1 (Figure 1). Steady‐state mean LEN C_trough_ (35.9 ng/ml; Table 1) remained well above IQ4 (15.5 ng/ml) and IQ1 (3.87 ng/ml). PK/PD simulations demonstrated a lack of ISL‐TP exposure‐related changes in CD4^+^ T‐cell and lymphocyte counts in ISL+LEN‐treated participants.

**P224: Table 1:** Pharmacokinetic parameters of ISL and LEN (intensive PK sub‐study)

	Day 1	Day 2	Steady state
	ISL 2 mg + LEN 600 mg (*N* = 13)	LEN 600 mg (*N* = 13)	ISL 2 mg + LEN 300 mg (*N* = 14)
ISL PK parameter			
C_max_ (ng/ml)	18.4 (42.3)	−	17.7 (42.4)
T_max_ (hour)	0.583 (0.50−1.00)	−	0.783 (0.50−1.00)
C_8h_ (ng/ml)	1.80 (58.7)^a^	−	1.38 (28.8)
C_trough_ (ng/ml)	−	−	0.169 (55.5)
AUC_0‐8h_ (h.ng/ml)	52.0 (25.6)^a^	−	42.2 (18.7)
AUC_tau_ (h.ng/ml)	−	−	131 (74.7)
LEN PK parameter			
C_max_ (ng/ml)	46.4 (62.4)	183 (103)	99.2 (72.6)
T_max_ (hour)	7.97 (4.00−24.0)	6.18 (6.00−7.93)	6.00 (4.00−6.17)
C_8h_ (ng/ml)	39.0 (64.2)	151 (84.2)	82.2 (78.4)
C_trough_ (ng/ml)	−		35.9 (60.5)^b^
AUC_0‐8h_ (h.ng/ml)	235 (64.5)	1040 (104)	625 (76.6)
AUC_tau_ (h.ng/ml)	−	−	9730 (73.9)^b^

*Note*: PK parameters are presented as mean (%CV) except T_max_, which is median (Q1, Q3).

Abbreviations: AUC_0‐8h_, area under the curve from 0 to 8 hours; AUC_tau_, area under the curve from time 0 to time tau (the dosing interval); C_8h_, concentration at 8 hours; C_max_, peak concentration; C_trough_, concentration immediately prior to administration of the next dose; T_max_, time to peak concentration.

^a^ *N* = 11. ^b^ *N* = 13.

**Conclusions**: ISL 2 mg QW is predicted to produce ISL‐TP exposures sufficient to cover both wild‐type and M184V/I variants with no negative impact on CD4^+^ T‐cell or lymphocyte counts. LEN 300 mg QW resulted in efficacious LEN exposures, consistent with approved subcutaneous LEN. These results are consistent with previous model‐based predictions and support ISL/LEN 2/300 mg QW dosing in phase III.

**P224: Figure 1:**
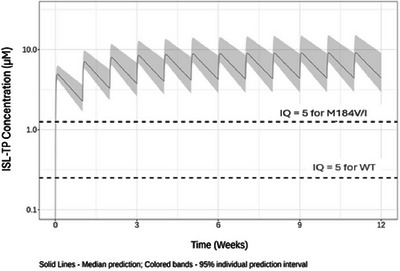
Simulations of ISL‐TP concentration time series following ISL 2 mg QW dosing (*n* = 52).

### P225 Assessing the effects of age and polypharmacy on dolutegravir exposure in plasma and the central nervous system

Brian Ho ^1^, Miao Zhang^1^, Scott Letendre^2^, Ronald Ellis^3^, Donald Franklin^4^, Leah Rubin^5^, Anne Bang^6^, Gene Morse^1^, Donald Mager^1^, Nicholas Smith^1^, Qing Ma^1^

^1^School of Pharmacy and Pharmaceutical Sciences, University at Buffalo, Buffalo, NY, USA. ^2^Department of Medicine, University of California San Diego, San Diego, CA, USA. ^3^Department of Neurosciences, University of California San Diego, San Diego, CA, USA. ^4^CHARTER Center, University of California San Diego, San Diego, CA, USA. ^5^Department of Neurology, John Hopkins University, Baltimore, MD, USA. ^6^Conrad Prebys Center for Chemical Genomics, Sanford Burnham Prebys Medical Discovery Institute, San Diego, CA, USA

**Background**: Neuropsychiatric adverse events (NP‐AEs) associated with dolutegravir (DTG) administration have emerged as a critical factor necessitating DTG discontinuation in affected patients. The study aim was to develop a population pharmacokinetic (PopPK) model to simultaneously capture the distribution characteristics of DTG in both plasma and cerebrospinal fluid (CSF).

**Materials and methods**: Plasma and CSF samples obtained from 45 HIV‐infected individuals were utilized for analysis. Additional plasma concentration‐time profiles of healthy volunteers from a previously published study were included to augment the dataset for PopPK analysis. Structural models were tested sequentially using NONMEM (Icon, v7.5) to simultaneously characterize the distribution of DTG in the plasma and CSF. The impact of age and total concomitant medications on DTG pharmacokinetics in plasma and CSF was also evaluated. The finalized model was utilized to simulate steady‐state DTG plasma and CSF concentrations in individuals receiving 50 mg DTG once daily to evaluate target‐attainment in the plasma and CSF based on in vitro IC_50_ (0.0002 µg/ml) and protein‐adjusted IC_90_ (0.064 µg/ml) targets.

**Results**: A total of 61 individuals, contributing 243 DTG plasma (165 from literature) and 35 CSF concentrations were included in the PopPK analysis. The observed peak DTG plasma concentration was 6.5 µg/ml but failed to exceed 0.02 µg/ml in the CSF. The data were best described by a two‐compartment model, utilizing a biophase compartment to describe the CSF DTG concentration, with parameter estimates in Table 1. Testing age or concomitant medication use as either a continuous or categorical covariate failed to produce a statistically significant impact. Steady‐state DTG simulations (Figure 1) demonstrated that effective DTG concentrations were achieved in plasma with steady‐state plasma trough concentrations surpassing the IC_90_ target, whereas the steady‐state DTG concentration in CSF only achieved IC_50_ targets.

**P225: Table 1:** Final population parameter estimates for dolutegravir model

Parameter (unit)	Description	Estimate (% RSE) [shrinkage]	Bootstrap median (95% CI)
CL/F (l/hour)	Central compartment clearance rate	0.845 (6%)	0.845 (0.767−0.906)
VC/F (l)	Central compartment volume of distribution	16.8 (8%)	16.8 (13.9−16.8)
VP/F (l)	Peripheral compartment volume of distribution	2.75 (19%)	2.75 (2.45−2.98)
Q/F (l/hour)	Inter‐compartment clearance rate	0.375 (25%)	0.375 (0.327−0.407)
ka (hour‐1)	Absorption rate constant	1.42 (20%)	1.42 (1.19−1.42)
ALAG1 (hour)	Absorption lag time	0.473 (6%)	0.473 (0.405−0.495)
Kin (l/hour)	Central compartment to biophase clearance rate	0.467 (13%)	0.467 (0.386−0.484)
Kout (l/hour)	Biophase to central compartment clearance rate	150 (18%)	150 (135−155)
Inter‐individual variability (IIV)			
ω^2CL_Literature	IIV of CL using literature data	0.0641 (41%) [54%]	0.0641 (0.0550−0.0652)
ω^2CL_Clinical	IIV of CL using patient data	2.15 (56%) [39%]	2.15 (1.90−2.58)
ω^2Vc	IIV of central compartment volume	0.192 (24%) [42%]	0.192 (0.146−0.223)
ω^2ka	IIV of absorption rate constant	0.264 (23%) [29%]	0.264 (0.194−0.271)
ω^2Kin	IIV of central compartment to biophase clearance rate	0.479 (57%) [56%]	0.479 (0.443−0.556)
Residual variability			
σ^2Prop_Lit	Variance of proportional residual error for literature data	0.162 (8%) [11%]	0.162 (0.157−0.168)
σ^2Prop_Clin_Plasma	Variance of proportional residual error for clinic plasma data	0.192 (18%) [34%]	0.192 (0.178−0.217)
σ^2Add_Clin_Plasma	Variance of additive residual error for clinic plasma data	0.457 (16%) [34%]	0.457 (0.437−0.465)
σ^2Prop_Clin_CSF	Variance of proportional residual error for clinic CSF data	0.194 (35%) [37%]	0.194 (0.174−0.197)
σ^2Add_Clin_CSF	Variance of additive residual error for clinic CSF data	0.00315 (23%) [37%]	0.00315 (0.00271−0.00324)

Abbreviation: %RSE, percent relative standard error.

**Conclusions**: Our PopPK model provides a foundational framework for assessing DTG exposure simultaneously in plasma and CSF, as well as assessing potential covariates and HIV inhibition target attainment. Overall, further exploring DTG pharmacodynamics in the CNS and associations with NP‐AEs will be pivotal in assessing the impact of low DTG CNS concentrations on long‐term HIV management.

**P225: Figure 1:**
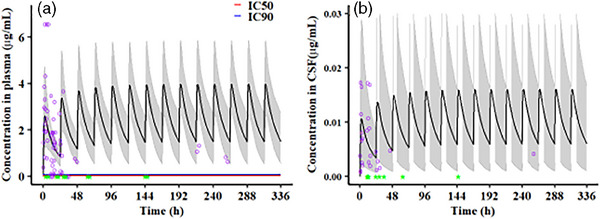
Simulated results of DTG administered 50 mg once daily for 14 consecutive days in plasma (a) and CSF (b). Purple circles represent observed clinical data and green asterisks represent observed data below limit of quantification (BLQ). The black curve represents the predicted median value and grey area represents the predicted 90% confidence interval.

### P226 Association of adenosine triphosphate‐binding cassette transporter G2 (ABCG2) genetic polymorphisms with subjective symptoms and weight gain by bictegravir administration in Japanese HIV‐1‐infected patients

Hiroki Yagura ^1^, Dai Watanabe^1^, Takao Nakauchi^2^, Kazuyuki Hirota^3^, Takuro Matsumura^3^, Takashi Ueji^3^, Yasuharu Nishida^3^, Munehiro Yoshino^2^, Tomoko Uehira^3^, Takuma Shirasaka^3^

^1^Department of Advanced Medicine for HIV Infection, Institute for Clinical Research, NHO Osaka National Hospital, Osaka, Japan. ^2^Department of Pharmacy, NHO Osaka National Hospital, Osaka, Japan. ^3^AIDS Medical Center, NHO Osaka National Hospital, Osaka, Japan

**Background**: Adenosine triphosphate‐binding cassette transporter G2 (ABCG2) is known to be expressed in the liver, the apical membrane of the small intestine and the blood‐brain barrier, and bictegravir (BIC) and other integrase inhibitors have been shown to be substrates of ABCG2. The principal aim of the study was to investigate the association between subjective symptoms induced by BIC administration and genetic polymorphisms of ABCG2.

**Materials and methods**: The study subjects were 77 Japanese patients with HIV‐1 infections who were receiving BIC at National Hospital Organization (NHO) Osaka National Hospital from April 2019 to December 2023. ABCG2 was genotyped using the sequence method. We compared the frequency of subjective symptoms among three groups: A (with homozygous mutations in ABCG2 (421C>A)); B (patients with heterozygous mutations); and C (wild‐type). In addition, weight gain during the first 24 weeks of treatment was also compared in treatment‐naïve patients.

**Results**: The median age of the 77 patients (75 were male) was 38 years (interquartile range 33–47 years); 56 (73%) patients introduced BIC in the first antiretroviral treatment. Subjective symptoms developed in 34 of 77 patients (44%), and of these: increased appetite in 20 (26%); insomnia in six (8%); vivid dreams in four (5%); forgetfulness in two (3%); headache in two (3%); and drowsiness in two (3%).The frequencies of subjective symptoms in the three groups were: A, 80%; B, 60%; and C, 18%. Significant difference in the frequency of subjective symptoms was evident in terms of ABCG2 genetic polymorphisms (*p* < 0.001). The mean increase in body weight during the first 24 weeks of treatment was 2.9 kg for A+B and 1.2 kg for C, and was significantly higher in the cases with the genetic mutation (*p* = 0.018).

**Conclusions**: ABCG2 genetic polymorphism may be a predictor of subjective symptoms and weight gain with BIC administration.

## Community‐based treatment and prevention initiatives, including primary care screening

### P227 Is undiagnosed HIV prevalence among MSM in London changing in the era of combination prevention? Data from the Gay Men's Sexual Health Survey, a serial cross‐sectional community sample

Flavien Coukan ^1^, Dana Ogaz^2^, Gary Murphy^3^, Hamish Mohammed^2^, John Saunders^2^, Fiona Burns^1^

^1^Institute for Global Health, University College London, London, UK. ^2^Blood Safety, Hepatitis, STI and HIV Division, UK Health Security Agency, London, UK. ^3^National Infection Service Laboratories, UK Health Security Agency, London, UK

**Background**: New HIV diagnoses are falling in gay, bisexual and other men who have sex with men (MSM) in England, in part thanks to routine implementation of HIV pre‐exposure prophylaxis (PrEP) in sexual health services since 2020. We report diagnosed and undiagnosed HIV prevalence before and during the period of implementation using data from the Gay Men's Sexual Health Survey: a serial, anonymous, cross‐sectional, self‐administered survey in London commercial venues (e.g. clubs, pubs, bars, saunas).

**Methods**: From Jun 2019 to Aug 2019 and Nov 2022 to Jan 2023, MSM aged ≥18 recruited from London commercial venues self‐completed a sexual health questionnaire and provided an optional oral fluid (OF) sample for HIV antibody (Ab) testing. We conducted descriptive analyses examining HIV prevalence; self‐reported HIV status; demographics; sexual risk; HIV testing history and PEP or PrEP use in those who were HIV Ab serology positive but self‐reported negative. Samples were excluded from the analysis for those who did not report their HIV status or those with total IgG <0.2mg/l.

**Results**: Of the 1408 surveys included in the 2019 analysis and the 1090 surveys in the 2022 analysis, the proportion of MSM who provided an eligible OF sample increased from 60.7% in 2019 (*n* = 855) to 68.9% (*n* = 751). In 2019, 8.3% (*n* = 71) were HIV Ab positive, of whom 8.5% self‐reported as HIV negative (*n* = 6). In 2022, 7.5% (*n* = 56) were HIV Ab positive, of whom 12.5% self‐reported as HIV negative (*n* = 7). Undiagnosed HIV prevalence was 0.8% (6/784) in 2019 and 1.0% (7/696) in 2022. In 2019, of the six self‐reporting as HIV negative but HIV Ab positive, 33.3% (*n* = 2) reported using PEP in the last year, similar to 2022 levels (28.6%, *n* = 2). Of those self‐reporting as HIV negative but HIV Ab positive, half (50%, *n* = 5) reported current PrEP use in 2019, which dropped to 14.3% (*n* = 1) in 2022.

**Conclusions**: Despite the reduction of new diagnoses nationally, HIV prevalence and rate of undiagnosed HIV infection appear constant. Increased community‐based efforts are needed to ensure prevention measures reach all those who need it and diagnose those who erroneously assume they are HIV negative to meet the UK Government's goal to end new HIV transmissions by 2030.

### P228 **Immunovirological outcomes in people living with HIV between immigrant and non‐immigrant at the Hospital Clinic of Barcelona (2010**−**2023)**

Alexy Inciarte, Elizabeth Zamora‐Clemente, Leire Berrocal, Berta Torres, Lorena De La Mora, Elisa de Lazzari, Montserrat Laguno, Maria Martinez‐Rebollar, Ana González‐Cordón, Ivan Chivite, Alberto Foncillas, Julia Calvo, Abiu Sempere, Juan Ambrosioni, Jose Luis Blanco‐Arevalo, Jose M. Miró, Esteban Martinez‐Chamorro, Josep Mallolas

HIV‐Unit, Hospital Clinic, Barcelona, Spain

**Background**: HIV‐positive immigrants face unique healthcare challenges, necessitating inclusive health strategies. This study aims to identify areas needing focused healthcare strategies by comparing immunovirological outcomes between immigrant and non‐immigrant populations.

**Materials and methods**: This retrospective‐longitudinal study of initial consultations at the Hospital Clinic of Barcelona between 2010 and 2023 compared immigrant and non‐immigrant PWH. We assessed demographic and clinical characteristics, viral load (VL), immunological status, rates of late diagnosis, and outcomes for both treatment‐naïve (TN) and treatment‐experienced (TE) individuals. Mixed‐effects logistic and linear regression models were employed to analyse the VL and CD4 data.

**Results**: The study included 5344 individuals (Table 1), of which 91% (*n* = 4861) were male with a median age of 34 (29–40). Men who had sex with men (MSM) was 77% (*n* = 3887), and 46% (*n* = 2217) were treatment‐naive (TN). Overall, there were 13% (*n* = 390) trans/non‐binary individuals. Immigrants comprised 65% (*n* = 3494) of the population, had higher rates of treatment‐experienced individuals (59% vs. 41%, *p* < 0.001), had more MSM (74% vs. 68%), were more frequently identified as female (9% vs. 8%, *p* = 0.306) and had lower rates of injecting drug users (2% vs. 7%) compared to non‐immigrants. At baseline, TN immigrants presented a lower zenith VL than non‐immigrants (53,945 vs. 67,700), *p* = 0.001. TE immigrants had lower undetectable VL than non‐immigrants (95% vs. 97%, *p* = 0.029; 34,360 vs. 45,015, *p* = 0.001, respectively). The median CD4 nadir was also lower in immigrants (314 vs. 332, *p* = 0.032). There was no significant difference in late diagnosis between immigrants and non‐immigrants (49% vs. 46%, *p* = 0.196) (Table 1). At yearly follow‐up, TN undetectability rates were similar for immigrants and non‐immigrants. OR 0.82 [95% CI 0.67–1.01], *p* = 0.063. TN immigrants had significantly lower CD4 counts than non‐immigrants during the follow‐up (coef: −38 [95% CI −60, −16], *p* = 0.001). TE immigrants also had significantly lower CD4 counts (−39.33 [95% CI −64.4, −14.3], *p* = 0.002) (Table 1).

**P228: Table 1:** Demographic and clinical characteristics of people with HIV: comparison between immigrant and non‐immigrant populations

	Overall (*n* = 5344)	Immigrants	Non‐immigrants	*p*‐value
Male	91% (*n* = 4861)	91% (3168/3494)	91% (1693/1850)	0.306
Median age (IQR)	34 (29−40)	34 (29−40)	37 (30−45)	0.001
MSM	77% (3887/5046)	80% (2628/3289)	72% (1259/1259)	0.001
Treatment‐naive (TN)	46% (2217/4834)	41% (1402/3455)	59% (815/1379)	0.001
Trans/non‐binary gender identity	13% (390/3102)	13% (372/2796)	6% (18/306)	0.001
Immigrants	65% (3494/5344)			
IDU mode of infection	3% (174/5243)	1% (51/3415)	7% (123/1828)	0.001
Zenith VL (TN)	58,770 (14,100−235,000), *n* = 2217	53,945 (13,000−206,000), *n* = 1402	67,700 (15,000−308,000), *n* = 815	0.001
Detectable VL (TN)	96% (2118/2207)	95% (1329/1395)	97% (789/812)	0.029
Median VL (TN)	38,050 (8070−155,000), *n* = 2207	34,360 (6880−129,800), *n* = 1395	45,015 (9476.5−212,700), *n* = 812	0.001
Undetectable VL (TE)	77% (2003/2597)	76% (1554/2043)	81% (449/554)	0.013
Median CD4 nadir (TN)	320 (201−448), *n* = 2206	314 (195−439), *n* = 1393	332 (211−471), *n* = 813	0.032
Late diagnosis (TN)	48% (1060/2206)	49% (684/1393)	46% (376/813)	0.196
CD4 count (TN)	358.5 (220−505), *n* = 2206	353 (216−492), *n* = 1393	372 (227−530), *n* = 813	0.063
CD4 count (TE)	526 (364−714), *n* = 2577	524 (356−710), *n* = 2027	544 (400−738), *n* = 550	0.012

Abbreviation: IDU, intravenous drug user.

**Conclusions**: Immigrants had lower CD4 counts throughout follow‐up than non‐immigrants, yet late diagnosis was similar.  At baseline, TE immigrants had a lower percentage of undetectable VL than non‐immigrants; follow‐up showed similar rates. (Figure 1)

**P228: Figure 1:**
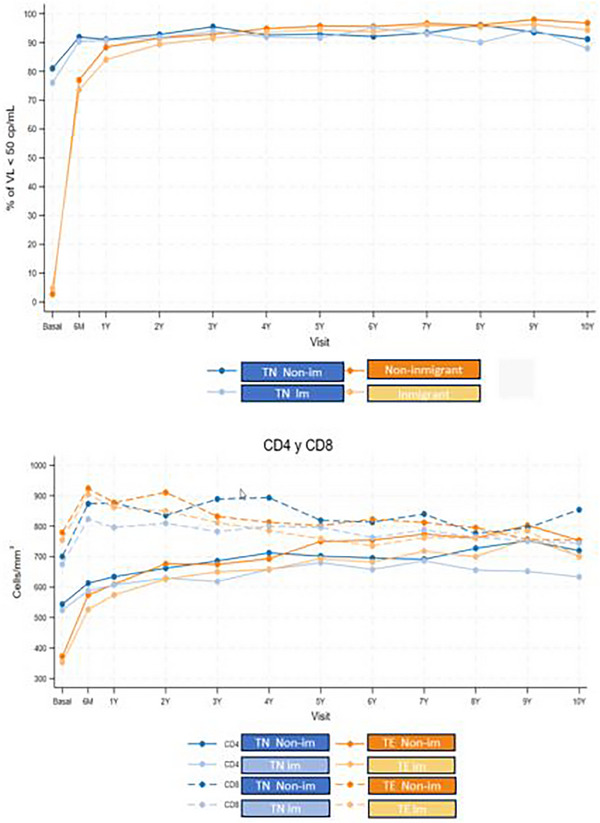
Longitudinal analysis of CD4/CD8 counts and viral load suppression in naive and treatment‐experienced patients by immigrant status. Im, immigrants; Non‐im, non‐immigrants; TE, treatment‐experienced; TN, treatment‐naive.

### P229 The war in Ukraine exacerbates the problem of late HIV detection

Tetiana Koval, Liliia Buria, Olena Marchenko, Olga Stetsenko, Tetiana Bajda

Infectious Diseases, Poltava State Medical University, Poltava, Ukraine

**Background: **The HIV epidemic in Ukraine is estimated to be the second largest in Eastern Europe and Central Asia. Among all HIV‐infected people in the European region, 14% are registered in Ukraine. Poltava region has consistently had a lower incidence HIV rate than Eastern regions of Ukraine. However, since the start of the war, 168,000 people from the Eastern regions of Ukraine (Kharkiv, Donetsk, Lugansk) have been officially displaced to Poltava region. Massive displacement of people, separation of families, constant anxiety and fear lead to the destruction of social contacts and late seeking medical care. The purpose of the study is to analyse the impact of the war in Ukraine to the effectiveness of HIV detection.

**Materials and methods: **We analysed the statistical data of the Poltava Regional HIV/AIDS Center and medical records of 1090 new HIV‐infected patients registered in Poltava region from January 2018 to December 2023. Nadir CD4 count <350 cells/ml and/or AIDS‐defining illness were defined as late presenters.

**Results: **In Ukraine, in 2023, a significant increase in the number of people tested for HIV was observed due to the screening of those mobilized for the army. Thus, the total number of people tested in Poltava region in 2023 compared to 2022 increased from 46,710 to 65,887 people, which almost corresponds to the level of 2018. The number of positive HIV tests in Poltava region in 2018 was 499, in 2022—343, in 2023—348. Thus, despite a 30% increase in the total number of people tested in 2023, the part of positive results decreased from 0.73% in 2022 to 0.53% in 2023. However, despite the growth in testing coverage, there was a significant rise in the proportion of late HIV presenters. Thus, the proportion of newly diagnosed HIV‐positive people with a CD4 count below 200 cells/µl increased from 35.7% in 2018 to 44.1% in 2023, and those with a CD4 count below 350 cells/µl from 53.4% to 60.6%, respectively.

**Conclusions**: The war in Ukraine is leading to a drop in the detection of HIV‐infected patients, which may contribute to the spread of the epidemic.

### P230 HIV in a cohort of female sex workers: far to meet 95:95:95 goals

María Macarena Sandoval^1^, Mona Loutfy^2^, Virginia Zalazar^1^, Agustín Nava^1^, Nadir F. Cardozo^1^, Mariela Ceschel^1^, Marcela Romero^3^, Georgina Orellano^4^, Carina Cesar^1^, Carolina Pérez^1^, Ana Gun^1^, María Inés Figueroa ^1^, Adriana Durán^5^, Inés Arístegui^1^, Zulma Ortiz^1^, Pedro Cahn^1^, Sharon Walmsley^6^, Valeria Fink^1^

^1^Research Department, Fundación Huésped, Buenos Aires, Argentina. ^2^Women's College Hospital, University of Toronto, Toronto, Canada. ^3^Asociación de Travestis, Transexuales y Transgénero de Argentina, Buenos Aires, Argentina. ^4^Sindicato de Trabajadorxs Sexuales de Argentina, Buenos Aires, Argentina. ^5^Coordinación Salud Sexual, VIH e Infecciones de Transmisión Sexual del Ministerio de Salud de la Ciudad de Buenos Aires, Buenos Aires, Argentina. ^6^University Health Network, University of Toronto, Toronto, Canada

**Background**: Key populations including female sex workers (FSWs) are at high risk of HIV. Barriers including stigma and discrimination limit access to sexual and reproductive health services, HIV testing, prevention and treatment among FSWs.

**Materials and methods**: “MAS por Nosotras” is a prospective cohort of cisgender (CGW) and transgender (TGW) FSW in Buenos Aires, Argentina built through a collaboration between a non‐governmental research organization, the local Ministry of Health and a Canadian research team. At cohort visits, medical assessment for HIV and STI and psychosocial interviews are conducted. We describe the baseline sexual activity, PrEP use and knowledge, HIV status and the frequency of prior HIV testing.

**Results**: Between June 2023 and March 2024, 200 participants were enrolled: 99 TGW and 101 CGW. Median time in sex work was (years): TGW 12 [IQR 6.5–19], CGW 10 [IQR 5–19]; 44.8% TGW and 22.8% CGW reported >20 sexual partners in the previous month (*p* = 0.001), and 56.6% and 52.5%, respectively, reported at least one condomless anal or vaginal intercourse. Baseline HIV prevalence was 8.5% (TGW: 34.3%; CGW: 3%), including five new diagnoses (four TGW and one CGW). Among those with HIV, 61% had VL <50 copies/ml and a median CD4 cell count of 848 cells/µl (IQR 646–1075), while those with detectable VL had median CD4 305 cells/µl (IQR 238–716). Among those without HIV, 18.4% CGW and 94.3% of TGW reported never having tested for HIV. The median time since the last HIV test was 5 months (IQR 2–22) for TGW and 20 months (IQR 8–59) for CGW (*p* < 0.001). 16.7% TGW and 3.1% CGW were on PrEP. Among those not receiving PrEP, TGW had previously used PrEP (14.2% vs. 1%) and reported more knowledge about PrEP (50% vs. 18.8%, *p* < 0.001) compared to CGW. When offered PrEP, acceptance was higher in TGW than CGW (55.6% vs. 30.5%).

**Conclusions**: Knowledge of HIV status and PrEP was low, especially in CGW. For those with known HIV, viral control was sub‐optimal. Our findings highlight the need to enhance strategies to improve HIV prevention and treatment in FSW with a gender‐specific focus in order to reach the 95:95:95 targets.

### P231 Pilot implementation of HIV self‐testing in Ghana: analysis of the community‐based demand generation approach

Ernest Amoabeng Ortsin ^1^, Jonathan Tetteh‐Kwao Teye^2^, Richard Socrate Adzesi^3^, Kwadwo Koduah Owusu^4^, Anthony Ashinyo^4^, Stephen Ayisi Addo^4^

^1^Ghana HIV and AIDS Network, Accra, Ghana. ^2^Dreamweaver Organization, Accra, Ghana. ^3^Ghana National TB Voice Network, Accra, Ghana. ^4^National AIDS/STI Control Programme, Accra, Ghana

**Background**: Under the auspices of the Ministry of Health (MoH) and National AIDS/STI Control Programme (NACP), Ghana piloted an HIV Self‐Testing (HIVST) project in 2023. The implementation took place in 50 HIV high‐burden districts and it was spearheaded by Ghana HIV and AIDS Network (GHANET), a grassroots civil society organization (CSO) with membership across the country. This paper examines the strategies and outcomes of the project which was sponsored by the Global Fund and currently being expanded as part of Grant Cycle Seven (GC7) implementation from 2024 to 2026.

**Methods**: One hundred and fifty volunteers from 50 community‐based organizations (CBOs) were trained to distribute ORAQUICK HIVST kits to local residents in the selected districts. Video instructions on the effective use of the test kits (produced in English and six different Ghanaian dialects) were made available to persons who voluntarily received the kits. Through an online portal (GHANET Information Management System [GIMS]), data about the distributions were recorded. Follow‐ups were conducted to ascertain, among others, how the kits were used. The data was quantitatively analysed and descriptive statistics was used to summarize the data using Statistical Package for Social Science (SPSS v.28).

**Results**: A total of 123,088 ORAQUICK HIVST kits were distributed (with follow‐ups) to 73,853 (60%) females and 49,235 (40%) males. Young people aged 20–24 received the highest number of kits totalling 28,790 which is about 23%, followed by those aged 25–29 who received 25,006 which is about 20% and those aged 30–34 who received 17,460 which is about 14%. A total of 422 persons comprising 304 (72%) females and 118 (28%) males self‐reported their results as reactive. Of this number, 329 persons comprising 72% of females and 28% of males were confirmed positive. And, out of that number, 202 persons comprising 73% females and 27% males were successfully initiated on ART in health facilities.

**Conclusions**: Male participation of 40% in the testing exercise was encouraging as it is above the national average of 20%. It is, therefore, recommended that in Ghana, HIVST should, particularly, be targeted at men.

### P232 Factors contributing to pneumococcal, COVID‐19 and influenza vaccine uptake among people living with HIV in Belgium: a retrospective study

Li‐Cécile Destordeur ^1^, Victoria Lopez Delhoulle^1^, Iraklis Papadopoulos^2^, Karine Fombellida^1^, Majdouline El Moussaoui^1^, Gilles Darcis^1^

^1^Infectious Diseases Department, Liège University Hospital, Liège, Belgium. ^2^Biostatistics and Research Method Center, Liège University Hospital, Liège, Belgium

**Background**: People living with HIV remain at risk of complications from vaccine‐preventable diseases despite antiretroviral therapy. There is limited research about vaccination rates and factors associated with adherence to vaccination among HIV‐positive individuals. This study aims to investigate pneumococcal, COVID‐19 and influenza vaccine coverage in people living with HIV in Belgium and identify factors associated with vaccine uptake.

**Materials and methods**: This is a single‐centre retrospective observational study conducted at the Liege University Hospital, Belgium. The data were extracted from the Liege University Hospital HIV database from 2017 to 2022. The data were input into the database by practitioners during outpatient encounters. Participants were HIV‐positive individuals of 18 years and older, receiving care at the Liege University Hospital. Patients who had less than 2 years of follow‐up were excluded from the study. We evaluated vaccination coverage for influenza, COVID‐19 and pneumococcal disease. We also collected demographic, clinical and biological data to assess factors associated with vaccine uptake.

**Results**: The study included 791 participants. 89.1% of participants received at least one dose of COVID‐19 vaccine. Five hundred and thirty‐eight (68%) people received at least one dose of influenza vaccine during the study period, but only 80 of them (10.1%) were annually vaccinated. Pneumococcal vaccination rates were the lowest, with only 37.8% of participants having received at least one dose of pneumococcal vaccine. Complete vaccine adherence (pneumococcal, Covid‐19 and annual influenza vaccination) was correlated with age (aOR 1.02, *p* = 0.024) and nadir CD4 (OR 0.99, *p* = 0.037). Partial vaccine adherence was associated with age (aOR 2.66, *p* = 0.026) and number of consultations during the study period (aOR 1.23, *p* = 0.0002). It was also inversely associated with intravenous drug use (aOR 0.15, *p* = 0.015) (Table 1).

**P232: Table 1:** Factors associated with partial vaccine uptake (at least one dose of pneumococcal or COVID‐19 or influenza vaccine) in univariable and multivariable logistic regression among people living with HIV

Variable	*N*	Categories	Reference	OR (95% CI)	*p*‐value	aOR (95% CI) (*N* = 791)	*p*‐value
Gender	791	Male	Female	1.1 (0.53−2.1)	0.88		
Age (< or ≥50 years old)	791	<50	≥50	2.6 (1.2−6.4)	0.027	2.66 (1.19−6.77)	0.026
BMI (kg/m^2^)	790			1 (0.96−1.1)	0.46		
Number of consultations between 2017 and 2022	791			1.2 (1.1−1.4)	0.0004	1.23 (1.11−1.38)	0.0002
Mode of transmission	791	IV drugs	Homosexual/bisexual	0.12 (0.028−0.59)	0.004	0.15 (0.03−0.79)	0.015
		Heterosexual	Homosexual/bisexual	0.93 (0.39−2)	0.85	0.98 (0.41−2.17)	0.961
		Mother to child	Homosexual/bisexual	0.23 (0.052−1.6)	0.081	0.27 (0.06−2.01)	0.136
		Others	Homosexual/bisexual	0.62 (0.18−2.9)	0.49	0.73 (0.2−3.43)	0.651
Ethnicity	791	Caucasian	African	1.3 (0.63−2.7)	0.5		
		Others	African	0.32 (0.11−1.1)	0.05		
Smoking	766	Yes	Never/stopped	1.19 (0.54−3.01)	0.682		
Alcohol consumption	657	Yes	Occasional/never/stopped	4.6 (0.96−81.7)	0.14		
Nadir CD4	791	(Cells/mm^3^)		1.0 (0.998−1.001)	0.33		
Hepatitis A	619	Immunized	Not immunized	1.1 (0.44−2.6)	0.76		
Hepatitis B	789	Isolated anti‐HBcs	Not infected/not immunized	2.5 (0.68−15.8)	0.24		
		Immunized	Not infected/not immunized	1.9 (0.95−4.0)	0.068		
		Chronic infection	Not infected/not immunized	3.5 (0.69−63.3)	0.23		
Hepatitis C	791	Cured	Not infected	0.34 (0.09−2.2)	0.17		
		Acute infection	Not infected	N/A	N/A		
		Infection	Not infected	0.64 (0.12−11.7)	0.67		

Abbreviation: N/A, not applicable.

**Conclusions**: Our study revealed that, while COVID‐19 vaccine uptake is high, vaccination coverage for influenza and pneumococcal disease remains insufficient. Vaccine uptake was primarily associated with age, nadir CD4 and number of consultations. It should be noted that vaccine adherence was particularly low among intravenous drug users. Targeted interventions and vaccine reminders should be conducted to increase vaccination rates.

### P233 Reporting standard compliance in publications of mobile application interventions for antiretroviral therapy adherence among people living with HIV

Abdulhammed Babatunde ^1^, Dimeji Olawuyi^1^, Folashade Olajuwon^1^, Shamsudeen Olatokun^1^, Olutola Awosiku^2^, Isaac Ekundayo^3^

^1^Medicine and Surgery, University of Ibadan, Ibadan, Nigeria. ^2^Digital Health, Africa Centre for Disease Prevention and Control, Addis Ababa, Ethiopia. ^3^Medicine and Surgery, University of Ilorin, Ilorin, Nigeria

**Background**: In recent decades, there has been a proliferation of mobile health (mHealth) interventions to address public health challenges such as HIV/AIDS. Hence, the need for standardization of reporting of mHealth interventions and frameworks to enable effective knowledge sharing and promote developments emerged. In 2016, the World Health Organization (WHO) mHealth Technical Evidence Review Group published a 16‐item checklist for reporting health interventions using mobile phones. This study aims to review publications on mobile applications used for antiretroviral therapy (ART) adherence among people living with HIV (PLHIV) to evaluate their compliance with the standard reporting guideline by the WHO [1].

**Methods**: A comprehensive search of literatures was conducted on PubMed, PubMed Central and MEDLINE databases. We selected randomized controlled trials reporting mobile applications used in improving adherence to ART among PLHIV. Only studies published in the last 10 years and in the English language were included. Each selected study was reviewed by two independent reviewers against the standard 16‐item checklist developed by the WHO.

**Results**: A total of 19 studies were included in the review. Most of the studies were conducted in the United States of America (*n* = 8). Only four (21%) of the studies reported more than 70% (11/16) of the items on the standard reporting checklist by WHO. More than 80% of the studies reported the intervention delivery (*n* = 17) and intervention content (*n* = 17). The least reported items were: interoperability/health information systems (HIS) context (*n* = 2), infrastructure (population level such as electricity, internet connectivity, etc.) (*n* = 3) and cost assessment (*n* = 4), see Figure 1. However, these are important factors that ensure the sustainability and usability of mHealth intervention, especially in low‐ and middle‐income countries.

**Conclusions**: Our review showed that most mHealth interventions promoting ART adherence did not comply with the standard reporting guideline. The lack of standardization of mHealth interventions may be responsible for increased siloed mobile applications. Hence, there is a need for global adoption of the checklist by Ministries of Health, international organizations, journals and relevant authorities.

**P233: Figure 1:**
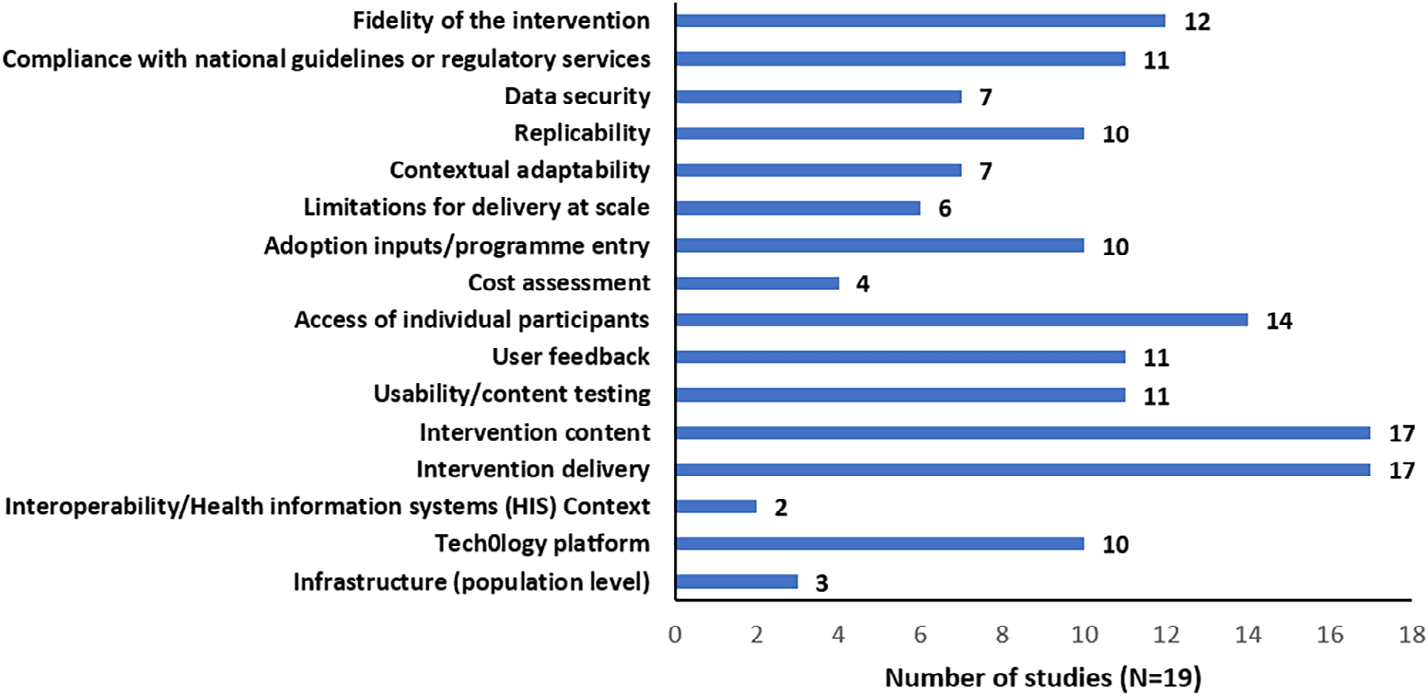
Number of studies that comply to each of the 16 items in the guideline for reporting mHealth interventions.

**Reference**

1. Agarwal S, LeFevre AE, Lee J, L'Engle K, Mehl G, Sinha C, et al. Guidelines for reporting of health interventions using mobile phones: mobile health (mHealth) evidence reporting and assessment (mERA) checklist. BMJ. 2016;352:i1174.

### P234 Implementing effective infectious disease screening in syringe service programmes

Heather Henderson, Jason Wilson, Bernice McCoy

Emergency Medicine, Tampa General Hospital/University of South Florida, Tampa, FL, USA

**Background**: Infectious disease screening is a critical element in harm reduction strategies at syringe services programmes (SSPs) due to the high risk of HIV and HCV transmission associated with injection drug use. Traditional opt‐in testing models have shown low participation rates, which can hinder early detection and treatment efforts. We demonstrate how the shift to an opt‐out screening model at our SSP vastly improved screening rates and public health outcomes.

**Materials and methods**: Our SSP implemented a clinical opt‐out model for HIV and HCV screening, integrating routine anonymous screenings within a low‐barrier approach, informing participants that testing will occur unless they explicitly decline, thereby simplifying the consent process and reducing stigma. Screenings are conducted at enrolment and repeated quarterly, with options to switch to confidential to start treatment. We emphasize person‐centred care and maintain a non‐stigmatizing environment, ensuring high retention rates.

**Results**: The implementation of the opt‐out model increased screening rates over 1000%; from 214 HIV screens in year 1, to 2402 by year 3, and 230 HCV screens in year 1 to 2859 screens by year 3, with only seven out of 1761 participants opting out since the programme's inception. The approach has facilitated early detection and treatment, leading to better health outcomes and reduced transmission of HIV and HCV. Key factors contributing to this success include routine screening, voluntary anonymity, person‐centred care, separation of research and clinical operations, and a low‐barrier model. Participants have responded positively, highlighting the programme's non‐stigmatizing approach and flexibility in screening options.

**Conclusions**: SSPs should consider an opt‐out low barrier‐approach screening model as an emerging best practice to enhance HIV/HCV screening. It is essential to maintain a non‐stigmatizing environment and offer flexible screening options to accommodate diverse participant needs. Continuous evaluation and adaptation of the programme based on participant feedback will further improve its effectiveness.

### P235 Influence of community‐based oral “PrEP” refill model on adherence and retention among adolescents in Homa Bay County, Kenya

Electrine Osewe ^1^, Seth Kagia^2^

^1^Tom Mboya Level 4 Hospital, Ministry of Health, Homa Bay County, Nairobi, Kenya. ^2^Ministry of Health, Homa Bay County, Nairobi, Kenya

**Background**: HIV incidence rate in Kenya among adolescents is four times higher than the other age groups. HIV oral PrEP has proved to have significant efficacy and efficiency in reducing new HIV infections. However, stigma remains a barrier to access PrEP among adolescents, hence suboptimal uptake, adherence and retention. We examined the role of community PrEP refill model in improving adherence and retention among adolescents in Tom Mboya hospital in Homa Bay County, Kenya.

**Methods**: Adolescents with potential risk of HIV infection were referred to the health facilities for PrEP initiation. Community PrEP refill model was created by grouping between 4 and 6 adolescents on PrEP from the same geographical area and community PrEP refill sites established. The groups’ subsequent follow ups done at the community refill sites 90 days post‐initiation for follow‐up HIV testing, 90 days refill and literacy sessions through the PrEP champions. With interventions aiming to improve adherence and retention.

**Results**: Pre‐ and post‐intervention evaluation on PrEP by analysing data on adolescents initiated, continuity and adherence. At baseline, 125 were initiated on PrEP across all ages of which 17 (13.6%) were adolescent (15–24 years), 55 (44%) remained active, while six (35%) were adolescents. After interventions, 157 were initiated on PrEP, 72 (45.8%) were adolescents depicting 32% increased access to PrEP. Sixty‐four of the 72 (88.9%) adolescents were active with good adherence, marking 42% increased retention. Fifty‐two of 64 (81.2%) were on 90 days drug refill while 18% are still having treatment concerns, no adolescent has seroconverted.

**Conclusions**: Community PrEP refill model improved adherence, retention and enabled referrals that improved PrEP uptake among adolescents. The support groups improved knowledge on PrEP reducing myths and misconceptions within the community. As the Kenya ART guidelines 2022 address ART community model, this can also be upscaled to HIV‐negative persons on PrEP.

### P236 The impact of migratory patterns in an HIV care clinic of a tertiary hospital in Portugal

Joana Marinho Silva, Gonçalo Cruz, Cristina Valente

Infectious Diseases, ULS Coimbra, Coimbra, Portugal

**Background**: The number of new diagnoses of HIV infection in the EU/EEA has increased significantly in the period of 2021–2022 by about 7.8% [1]. One of the factors that contributed was the increased migration of people living with HIV (PLHIV) throughout Europe. In Portugal, the number of migrants nearly doubled in 10 years and today represents a total of 7.6% of the Portuguese population [2].

**Materials and methods**: The scope of data under analysis refers to the new migrant PLHIV followed in a tertiary hospital in the period of 3 years (2021–2023).

**Results**: We evaluated 336 migrants (48.9% of new referrals), where 66.8% were cis men, 32.6% cis women and 0.6% trans woman. Regarding the geographical origin, 61.3% was South American, 29.8% African and 7.4% were from other countries in Europe. In fact, 69% of the diagnosis were made outside of Portugal and, of those, 78.4% were living in Portugal for less than a year. The main transmission route was sexual (86.9%), 45% being men who have sex with men. Considering coinfection status, 12 were previously infected with hepatitis B, one with hepatitis B and D and 12 with hepatitis C. From the total, 22% were naïve patients and 262 (78%) patients were on antiretroviral therapy (ART); from these, 233 (69.3%) patients had HIV viral load <200 cp/ml, 57.4% were undetectable and 50.3% had TCD4+ counts >500 cells/mm^3^. The most frequent ART regimens were based on INSTI: DTG/3TC/TDF (27.5%) followed by DTG/FTC/TDF (23.6%) and DTG/3TC (9.6%). Only eight patients were diagnosed with opportunistic infections on their arrival. At the last evaluation, 88 patients (17.3%) were either lost to follow‐up referred to another institution (5.6%) or had died (1.2%).

**Conclusions**: Poor socio‐economic conditions and high prevalence of HIV in some regions increased migration burden. At admission, 42.2% of migrants had detectable viraemia.  This highlights an urgent need to ensure adequate follow‐up of treatment and proper monitoring. Furthermore, the dropout rate from follow‐up is concerning and suggests the need for more effective strategies to retain patients in treatment programmes.

**References**

1. European Centre for Disease Prevention and Control. HIV/AIDS surveillance in Europe 2023; page 7 [Internet]. 2023 [cited 2024 Aug 12]. Available from: https://www.ecdc.europa.eu/sites/default/files/documents/HIV‐AIDS_surveillance_in_Europe_2023_%28_2022_data_%29.pdf.

2. Fundação Francisco Manuel dos Santos. Pordata. Lisboa: Fundação Francisco Manuel dos Santos; 2023.

### P237 Use of human‐centred design to co‐develop a digital platform and evaluate its use to drive uptake of HIV pre‐exposure prophylaxis (PrEP) among Nigerian youths in Lagos, Nigeria through a pilot intervention study

Yusuf Babatunde ^1^, Oluwakorede Adedeji^2^, Ibrahim Abdulmumin^2^

^1^YLabs, Youth Development Labs, Kigali, Nigeria. ^2^Pharmaceutical Sciences, University of Ilorin, Ilorin, Nigeria

**Background**: Nigerian youths are at the heart of a growing HIV crisis, with the second‐highest rate of new infections globally. HIV pre‐exposure prophylaxis (PrEP) offers great opportunities to reduce the risk among youths. This study assessed the delivery of HIV PrEP through a co‐developed web platform for youths in Lagos, Nigeria.

**Materials and methods**: This study employed five phases of human‐centred design (HCD): empathy, define, ideate, prototype and test. The empathy and define phases involved qualitative interviews with 21 youths (15–24 years old) aimed at understanding needs and barriers to accessing HIV PrEP. Expert interviews with 12 providers provided additional insights. An additional 106 youths filled out the self‐administered questionnaires. Content extraction and thematic analysis of the interviews and questionnaires informed the ideation and prototyping phases, leading to a web‐based intervention design called Binta. Binta was evaluated qualitatively for feasibility and acceptability during a 6‐month pilot using structured interviews with 42 youths at risk of HIV.

**Results**: Among the youths we co‐designed with, 65% were female, and 70% were at least 20 years old. Four key factors emerged as barriers to PrEP access and uptake among Nigerian youth: long distances to HIV clinics, confidentiality issues, low knowledge of PrEP and high costs. Features of the web platform, co‐developed with youths and providers, include: a reddit‐like community to foster safe, anonymous, and open conversations around PrEP and HIV/AIDS; a robust FAQ section with visual media; a PrEP eligibility section; and a pharmacy locator. The pilot study revealed widespread desirability of the web platform among youth at risk of HIV/AIDS, including female sex workers. Overall, 88% (*n* = 37) of youths interviewed in the pilot phase were comfortable with the platform's user interface and design. One hundred percent (*n* = 42) of the youths used the PrEP eligibility feature and more than 80% (*n* = 34) used the pharmacy locator to pick up their PrEP bottles.

**Conclusions**: Digital health technologies have the potential to expand PrEP services and show great promise in delivering health services to meet the needs of Nigerian youths. Engaging youths as partners in the design of interventions can help facilitate uptake.

### P238 Missed opportunities of HIV diagnosis and its clinical repercussions on the Portuguese population: a cohort study

João Lourinho ^1^, Maria João Miguel^1^, Frederico Gonçalves^1^, Francisco Vale^1^, Cláudia Franco^1^, Nuno Marques^2^

^1^Infectious Diseases, Hospital Garcia de Orta, Local Health Unit of Almada‐Seixal, Almada, Portugal. ^2^Infectious Diseases, Local Health Unit of Arrábida, Setúbal, Portugal

**Background**: Late HIV diagnosis has been associated with missed opportunities for earlier HIV diagnosis [1–8]. We propose a study to identify these missed opportunities, the most susceptible population subgroups and to evaluate their clinical repercussions.

**Materials and methods**: We conducted a retrospective, longitudinal, single‐centre cohort study evaluating missed opportunities to HIV diagnosis and its clinical repercussions in adult individuals with an HIV new diagnosis or who were drug naïve, who had a first consultation in our Infectious Diseases Department between 2018 and 2023. We evaluated the presence of missed opportunities in the 2 years prior to the diagnosis, or after the last negative HIV test. We compared clinical and laboratorial data from individuals with and without missed opportunities. The primary outcome considered was AIDS‐defining conditions at the time of HIV diagnosis. As a secondary outcome, the CD4 count at the time of diagnosis was compared between groups.

**Results**: We included 436 individuals among 1115 referred to consultation in our department. From our sample, 27.1% (118/436) of individuals experienced at least one missed opportunity. In individuals who experienced missed opportunities, the feminine sex was more prevalent (*p* = 0.007), the age at the first consultation was higher (*p* < 0.001), being born in Africa and in countries with high HIV prevalence occurred more frequently (*p* < 0.001), and heterosexual transmission was more frequent (*p* < 0.001). After adjustment to potential confounding factors, having missed opportunities predicted the occurrence of AIDS‐defining conditions at the time of HIV diagnosis (OR 3.84, CI 95% [1.67–8.79], *p* = 0.001). As for our secondary outcomes, we found that having a CD4 count ≤200 cells/mm^3^ at the time of diagnosis was more prevalent in individuals with missed opportunities (*p* = 0.027), but we found no significant differences in the occurrence of CD4 count ≤350 cells/mm^3^ between the two groups.

**Conclusions**: We conclude that missed opportunities translate in higher odds of developing an AIDS‐defining condition at HIV diagnosis, reflecting their clinical severity. A thorough characterization of missed opportunities and the affected population subgroups can help address the HIV epidemic by serving as a foundation for more targeted interventions.

**References**

1. Horsley Downie J, Pegler M, Widdrington J, Price D, Premchand N, Chadwick D. Late HIV diagnosis and missed opportunities for testing: piloting a standardised, multi‐source review process. Int J STD AIDS. 2020;31:208‐13.

2. Nanditha NGA, St‐Jean M, Tafessu H, Guillemi SA, Hull MW, Lu M, et al. Missed opportunities for earlier diagnosis of HIV in British Columbia, Canada: a retrospective cohort study. PLoS One. 2019;14:e0214012.

3. Downing A, Garcia‐Diaz JB. Missed opportunities for HIV diagnosis. J Int Assoc Provid AIDS Care. 2017;16:14‐17.

4. Brawley D, MacConnachie A, Nandwani R, Bell D, Fargie F, Fox R, et al. Missed opportunities for HIV diagnosis: a three‐year audit in the West of Scotland. Scott Med J. 2013;58:173‐7.

5. Tominski D, Katchanov J, Driesch D, Daley M, Liedtke A, Schneider A, et al. The late‐presenting HIV‐infected patient 30 years after the introduction of HIV testing: spectrum of opportunistic diseases and missed opportunities for early diagnosis. HIV Med. 2017;18:125‐32.

6. Mahmoud M, Ballouz T, Lahoud C, Adnan J, Habib PA, Saab R, et al. Late presentations and missed opportunities among newly diagnosed HIV patients presenting to a specialty clinic in Lebanon. Sci Rep. 2024;14:8296.

7. Rüütel K, Lemsalu L, Lätt S, Epštein J. Missed opportunities for HIV testing in people diagnosed with HIV, Estonia, 2014 to 2015. Eurosurveillance. 2019;24:1‐9.

8. Levy I, Maor Y, Mahroum N, Olmer L, Wieder A, Litchevski V, et al. Missed opportunities for earlier diagnosis of HIV in patients who presented with advanced HIV disease: a retrospective cohort study. BMJ Open. 2016;6:e012721.

### P239 Characterization of chemsex in PrEP users in four hospitals of Buenos Aires, Argentina

Maria Belen Zorz^1^, Isabel Pastor ^1^, Mariana Angelica Kundro^1^, Guillermo Alberto Viloria^1^, Marina Alonso Serena^1^, Federico Cardozo^1^, Diego Cecchini^2^, Jose Barletta^3^, Iael Altclas^4^, Marcelo Horacio Losso^1^

^1^Emergent Diseases, Hospital General de Agudos José María Ramos Mejía, Ciudad Autonoma de Buenos Aires, Argentina. ^2^Infectious Diseases, Hospital General de Agudos Dr. Cosme Argerich, Ciudad Autonoma de Buenos Aires, Argentina. ^3^Infectious Diseases, Hospital General de Agudos Dr. Juan A. Fernández, Ciudad Autonoma de Buenos Aires, Argentina. ^4^Infectious Diseases, Hospital Donación Francisco Santojanni, Ciudad Autonoma de Buenos Aires, Argentina

**Background**: The practice of chemsex consists of the intentional use of substances to increase activity or sexual pleasure. This practice could be associated with high‐risk sexual behaviours that can increase the transmission of STIs as well as expose users to potentially severe adverse events related to the drugs used [1,2]. PrEP for HIV consists of the use of antiretroviral drugs to reduce the risk of infection. A history of having practised chemsex was associated with a greater probability of having a diagnosis of STIs in PrEP users [3]. In Argentina, there is limited information regarding this problem [4]. This research aims to measure the prevalence and characterize the practice of chemsex and STIs in PrEP users in four hospitals in the City of Buenos Aires, Argentina.

**Materials and methods**: This is a descriptive, cross‐sectional study. The medical records and a self‐administer questionnaire were used to obtain the information.

**Results**: From February to June 2024, 108 PrEP users were included, the study is still enrolling and we report here preliminary results. The median age was 34 years (IQR 30–40). Ninety‐four percent identified as cisgender men. The median days in PrEP and the prevalence of STIs in the last year were, for chemsex users and not users, respectively: 628 (IQR 525–813) versus 486 days (IQR 224–715) and 52 versus 37%. In the last 12 months, 53% practiced chemsex, the drugs used were: cocaine, 35%; MDMA (ecstasy), 33%; ketamine, 23%; γ‐hydroxybutyrate (GHB), 21%; methamphetamine, 10%; γ‐butyrolactone (GBL), 3% and mephedrone, 3%. Forty percent reported the use of two or more drugs at the same time, 21% recognized chemsex as a problem in their life.

**Conclusions**: We have identified a high prevalence of chemsex in PrEP users in Buenos Aires, Argentina. This highlights the need to take into consideration this topic in the development of PrEP and sexual health strategies.

**References**

1. Strong C, Huang P, Li CW, Ku SW, Wu HJ, Bourne A. HIV, chemsex, and the need for harm‐reduction interventions to support gay, bisexual, and other men who have sex with men. Lancet HIV. 2022;9(10):e717‐25.

2. McCall H, Adams N, Mason D, Willis J. What is chemsex and why does it matter? BMJ. 2015;351:h5790.

3. MacGregor L, Kohli M, Looker KJ, Hickson F, Weatherburn P, Schmidt AJ, et al. Chemsex and diagnoses of syphilis, gonorrhoea and chlamydia among men who have sex with men in the UK: a multivariable prediction model using causal inference methodology. Sex Transm Infect. 2021;97:282‐9.

4. Salusso D, Nuñez S, Cabrini M, Rolón MJ, Cahn P. Chemsex y uso de sustancias durante las relaciones sexuales: resultados de una encuesta realizada en Argentina. Actualizaciones En Sida E Infectología. 2021;28(103):40‐50.

### P240 Long‐acting therapy (LA): what Italian PLWHIV think about it

Sandro Mattioli ^1^, Leonardo Del Negro^2^, Salvio Cecere^1^

^1^Plus aps, Bologna, Italy. ^2^Plus Roma, Roma, Italy

**Background**: In May 2022, the long‐acting injectable antiretroviral drugs against HIV were approved in Italy. For PLWH, the expectation was very high. But relatively few patients have had access to long acting since then. This is a first analysis of patients’ point of view. We also tried to understand what problems limit the switch to LA.

**Materials and methods**: From December 2023 to January 2024, we conducted an online survey, mostly promoted on the main social media.

**Results**: We collected 134 valid questionnaires. 94.7% (127) men; median age: 46 years (21/77). Eighty‐five percent (114) of the sample are gay, 7.5% (10) bisexuals, 6.7% (9) heterosexuals. 63.4% (85) live in North of Italy, 23.13% (31) in the Centre, 13.4% (18) in the South/Isl. Diagnosis year: before 1996 7.4% (10); 1997–2006 14% (19); 2007–2016 44.8% (60); 2017–2023 33.6% (45). In 63.4% (85) of cases, the doctor asked the patient to switch to the new regimen, in 36.6% (49) of cases, the patient asked. 49.4% (84) of the sample switched for the duration of long‐acting (2 months), 36.5% (62) swallowing pills every day reminds them they have HIV, 2% (14) due to toxicity of previous therapy, 5.9% (19) due to adherence problems. 73.7% (98) feel good with LA therapy, 19% (25) are still evaluating, 7.5% (10) feel bad. Side effects: 49% (92) of the sample reported pain in the injection area, 17% (32) no side effects, 13% (25) asthenia, 8% (15) weight gain, 7% (13) pyrexia, 5.8% (11) headache. 81.7% of respondents report having no problems with the clinical management of LA, 10.22% report the unavailability of the clinical centre to reschedule appointments, 1.46% (2) report difficulties in keeping appointments.

**Conclusions: **The majority of the sample confirms the positive evaluation of a switch that seems to have more psychological than clinical motivations. The constant presence of HIV in patients’ daily life appears to be the reason that pushed the majority of the sample to switch to the LA. It is also interesting to note that only 5.9% switched to LAs due to adherence problems with the previous therapy while, at an international level, the main researchers present LAs as a possible way to combat adherence difficulties. We note that 10.22% of patients complain about the clinical centre's lack of willingness to reschedule appointments despite just 1% admit they cannot be punctual. Follow‐up management could have room for improvement. More studies are needed.

### P241 HIV and STI checkpoint screening in Kiel: 4 years of screening in a medium‐sized regional centre

Lutz Ohrtmann

Checkpoint Kiel, Aidshilfe Kiel e.V., Kiel, Germany

**Background**: HIV and other STIs are still not on the scene in vulnerable populations but general population also. Reports by the Rober Koch Institute show that and are widespread in countryside areas [1]. Outside of metropolises, long distances and difficult screening and supply situations arise. The willingness to test decreases because of hard access to clients.

**Materials and methods**: Aidshilfe Kiel as a non‐profit organization offers counselling and screening for HIV and hepatitis C and other STIs based on checkpoint model and a streetwork programme. In addition to rapid tests, Checkpoint Kiel also offers laboratory tests and PCR confirmation tests. Blood, urine samples and swabs are collected at checkpoint and sent to a laboratory. Social workers offer pre‐ and post‐test counselling. If it is needed, doctors take blood samples and initiate smear tests and examine genitalia for the signs of the diseases such as HPV and genital herpes. People with tight budget are supported by discount or tested free of charge, others are covering the costs by themselves.

**Results**: Details of checkpoint activity in the period 2020–2024 are reflected in Table 1. The data collected by the checkpoint shows that heterosexual people in high demand of the screening programme (∼64%). There are other free screenings places for MSM. Most infections belong to the urogenital infections (*n* = 76 chlamydia) and (*n* = 43 Neisseria gonorrhoeae) and hepatitis C in drug‐using people (*n* = 51 HCV). HIV and syphilis are still widespread among MSM in Germany. Due to the additional free services in Kiel, MSM are an underrepresented group at Checkpoint Kiel. This could explain the round number of HIV (*n* = 5) and syphilis (*n* = 10) (Figure 1). People who use drugs in particular had to be reached through streetwork programmes. Even access to the NGOs that offer them services seems to be too difficult. The legal organization of the lack of billing options for streetwork doctors and the doctor's reservation for point‐of‐care PCR tests must be discussed further in view of the case numbers.

**P241: Table 1:** Details of checkpoint activity in the period 2020–2024

Year	Men	Women	Non‐binary	Risky sexual behaviour	Drug users	Total
2020	168	190	12	348	22	370
2021	190	200	15	377	28	405
2022	253	253	18	498	26	524
2023	211	193	1	356	49	405
June 2024	92	71	5	148	20	168

**Conclusions: **Even in industrialized countries, access to low‐threshold testing is barrier‐free. Symptomless, anamnestic risks are rarely covered by the German health insurance system. HCV is almost never treated by substituting doctors. Community‐based testing seems to fill the gap here. Due to the healthcare system in Germany, treatment in the test projects is almost impossible.

**P241: Figure 1:**
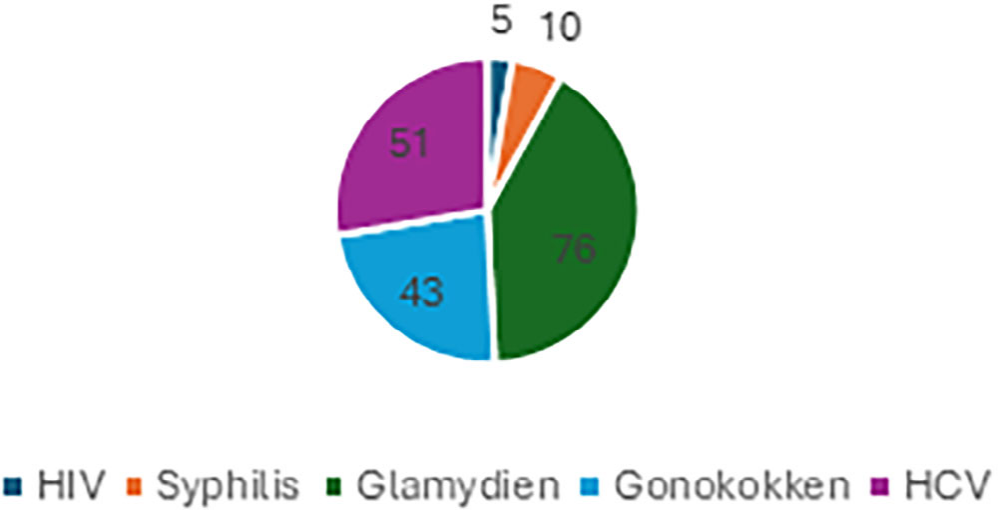
Positive laboratory tests at Checkpoint Kiel.

**Reference**

1. Bremer V, Dudareva‐Vizule S, Buder S, An der Heiden M, Jansen K. Sexuell übertragbare Infektionen in Deutschland. Bundesgesundheitsblatt Gesundheitsforschung Gesundheitsschutz. 2017;60(9):948‐57.

### P242 Check‐Mobil: counselling, testing and case management for vulnerable groups in Schleswig‐Holstein, Germany

Konstantin Kandlen ^1^, Louisa Glaum^1^, Sylvia Brillat^1^, Angela Stelling^2^, Ursula Hansen^2^, Hildegard Welbers^2^, Sarah Salvador^2^

^1^Landesverband, Aidshilfe Schleswig‐Holstein, Kiel, Germany. ^2^Vorstand, Aidshilfe Schleswig‐Holstein, Kiel, Germany

**Background**: HIV and HCV are still the ongoing challenges in northern regions of Germany. Estimated number of new HIV cases in Schleswig‐Holstein in the year 2021 was 45 and estimated number of people living with HIV without diagnose is more than 200. New cases of HCV were in the year 2021—202, in the year 2022—313. Statistics reflects ongoing processes of increasing numbers of new HIV and HCV cases, that can be emerging.

**Materials and methods**: On the way to eliminate HIV and HCV in the Federal State of Schleswig‐Holstein and improve treatment coverage, Aidshilfe Schleswig‐Holstein is improving access to low threshold testing to increase coverage in HIV and HCV testing, especially, in key groups in a huge northern territory of 15.800 sq.kms and population of 2.9 million, also as access to HIV and HCV treatment via linkage and case management. We present data for the Check‐Mobil low threshold testing in the period of 12/2023–05/2024 in the Federal State of Schleswig‐Holstein. Examination was done by antibodies express‐tests against HIV and HCV followed by pre‐ and post‐testing counselling, printed materials on HIV, STIs, HCV and condom distribution. In case of reactive result testing followed by linkage to care or case management. Every testing is followed by infomaterials, coffee, snacks and nice talk (Figure 1).

**Results**: In the time frame December 2023 till May 2024, we tested both for HIV and HCV 267 clients in 19 locations all over the Federal State. 70.4% of clients were men, 43.4% of all tested were people who use drugs. Testing locations are always connected to places relevant for key groups. In the past 6 months of active testing, we detected 10 reactive tests for HCV and no reactive for HIV. One hundred percent reactive HCV were people who use drugs. Positivity rate for HCV is 3.74%, four patients were treated for HCV successfully, others are in the process of linkage. Check‐Mobil shows its efficiency and productivity, because it goes to key groups and those people, who will never come for medical help without support and mentoring. Project is planned to be further developed.

**Conclusions**: Check‐Mobil project shows its high efficiency and demand, and planned to be developed to other parts of Federal State with improvement of services.

**P242: Figure 1:**
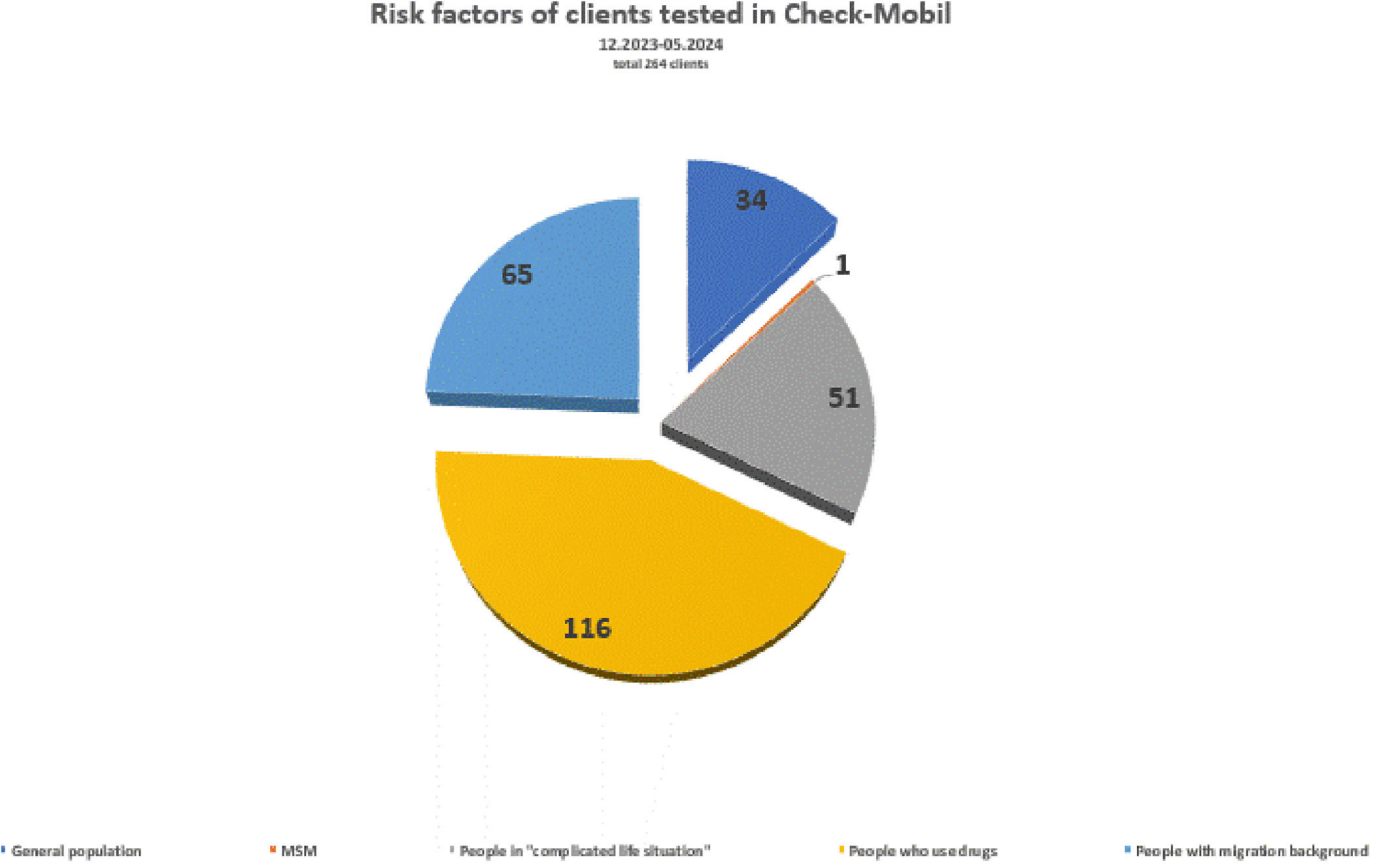
Characteristics of tested groups.

### P243 Retaining adolescents with HIV/AIDS in care through targeted youth camps in Kisumu, Kenya

Annabel Kokeyo ^1^, Geri DelaRosa Brooks^2^, Nancy Yienya^3^, Benard Samba^4^, Margret Mburu^5^, Mary A. Guze^6^, Jayne Lewis Kulzer^6^, Michelle Moghadassi^6^

^1^Social Science, Sunburst Projects Kenya, Kisumu, Kenya. ^2^Social Science, Sunburst Projects Kenya, Sacramento, CA, USA. ^3^Social Science, Ciheb, Migori, Kenya. ^4^Social Science, Family AIDS Care and Education Services, Kisumu, Kenya. ^5^Social Science, School of Public Health, University of Washington, Seattle, WA, USA. ^6^Obstetrics, Gynecology and Reproductive Sciences, University of California San Francisco (UCSF), San Francisco, CA, USA

**Background**: In Kenya, 29% of new HIV infections are among adolescents and youth and HIV remains their leading cause of mortality and morbidity. Kenyan adolescent HIV/AIDS programmes are developing comprehensive psychosocial interventions to address this vulnerable population. Camp Sunburst‐Kenya, a residential camp, is focused on supporting HIV prevention, disclosure and treatment adherence. This evaluation examined the effectiveness of Camp Sunburst‐Kenya on retention in care among adolescents in Kisumu, Kenya.

**Methods**: HIV‐positive adolescents ages 10–19 years from Family AIDS Care and Education Services (FACES) supported facilities attended a 5‐day intensive camp consisting of peer engagement, art therapy, narrative writing, HIV/AIDS knowledge games, role plays and personal experiences sharing. Staff were trained in team building, leadership, and addressing stigma, discrimination and diversity. Two independent camps were held, one in August 2013 and August 2014. Non‐camper controls were randomly selected and matched 2:1 to campers from both sessions based on age, gender and ART regimen. Demographic and clinical factors were abstracted from medical records of both campers and non‐campers. Participants active in care 6 months following the camp interval were considered retained. *p*‐Value from a mixed‐effect generalized likelihood model was used to compare campers to non‐campers both before and after the camp session controlling for repeated measures.

**Results**: In total, 71 campers and 142 non‐campers were analysed. The median age was 13.8 years (IQR 10.7–16.2) and 108 (51%) were female. From multivariate analysis, campers were four times more likely to be retained in care (aOR 4.03, 95% CI 1.46–11.14, *p* < 0.01) compared to non‐campers. The odds of retention in care may be lower per increasing year of age, (aOR 0.88, 95% CI 0.78–1.00, *p* = 0.06). Patients on first‐line regimen were seven times more likely to be retained in care than those not on any ART (aOR 7.15, 95% CI 3.06–16.7, *p* < 0.01).

**Conclusions**: Intensive adolescent resident programmes, such as Camp Sunburst‐Kenya, may be effective in improving retention in care particularly for youth experiencing stigma and other psychosocial challenges. Our results also indicate that the current test and start policies will also improve retention to care and likely provide additional opportunities for intense psychosocial support.

## Public health strategies including of policy options

### P244 Nationality and referral sources of people with HIV seeking first consultations: a 13‐year retrospective cohort study at the Hospital Clinic of Barcelona

Elizabeth Zamora‐Clemente^1^, Alexy Inciarte ^2^, Leire Berrocal^2^, Berta Torres^2^, Lorena De La Mora^2^, Elisa de Lazzari^2^, Montserrat Laguno^2^, Maria Martinez‐Rebollar^2^, Ana González‐Cordón^2^, Ivan Chivite^2^, Alberto Foncillas^2^, Julia Calvo^2^, Abiu Sempere^2^, Juan Ambrosioni^2^, Jose Luis Blanco‐Arevalo^2^, Jose M. Miró^2^, Esteban Martinez‐Chamorro^2^, Josep Mallolas^2^

^1^Medicine, University of Barcelona, Barcelona, Spain. ^2^HIV‐Unit, Hospital Clinic, Barcelona, Spain

**Background**: The increasing immigration in Europe has brought healthcare challenges, particularly among people with HIV (PWH). This demographic shift requires focused analysis to improve integration and care for immigrant‐PWH.

**Materials and methods**: A retrospective, longitudinal study was conducted at the Hospital Clinic of Barcelona (HCB). We included PWH with their first consultation between 2010 and 2023. The primary objective was to evaluate demographic changes in immigrants versus Spanish PWH. Secondary objectives were: PWH demographics, continental and country‐specific shifts, referral sources and factors influencing referral. The sources of referrals were primary care (PC), non‐governmental organizations (NGOs), sexually transmitted infections screening programmes (STISP) and hospitals.

**Results**: The study included 5344 individuals, of which immigrant PWH represented 3494 (65%) of the first consultations. There is a rise in immigrant PWH from 47% (164/347) in 2010 to 87% (412/474) in 2023. Conversely, Spanish‐PWH declined from 53% (183/347) to 13% (62/474), respectively (Figure 1). The proportion of first consultations from America rose from 31% (109/347) to 72% (343/4HY74). European (excluding Spain), African and Asian/Oceanian remained at 9–19%, 0–5% and 3%, respectively. From 2010 to 2015, Spain had the highest median PWH at 192 (44–57%). Colombia followed with a median of 14 (3–5%), then Argentina with 14 (3–5%). Brazil had a median of 26.5 (5–11%), Venezuela 7 (2–6%) and Peru had 8 (1–4%). From 2018 to 2023, Spain had a median number of 80 PWH (13–26%), making it the highest. Colombia followed with a median of 62 (6–24%), then Venezuela with 48 (11–13%). Brazil, Argentina and Peru had 30 (6–13%), 26 (6–11%), 25 (5–10%) PWH, respectively. Median percentage values are shown in 4‐year intervals in Table 1. Referrals from other hospitals were the most common, with a higher percentage for non‐immigrants (69%, *n* = 474) compared to immigrants (51%, *n* = 1442), *p* < 0.001, NGO referrals were higher for immigrants than non‐immigrants 24% (*n* = 678) versus 4% (*n* = 28), *p* = 0.001. PC referrals were 15% in both groups. HCB diagnoses accounted for 8% (*n* = 53) for non‐immigrants versus 7% (*n* = 190) for immigrants.

**Conclusions**: The study shows an increase in first consultations for immigrant PWH and a decline for Spanish PWH, with rising cases from the Americas. Referral sources, including hospitals and NGOs, display distinct patterns between immigrant and non‐immigrant populations.

**P244: Table 1:** Median and proportion of PWH consultations by country across different time periods (2010–2023)

Country	2010−2014 (*n*, %)	Median (IQR) 2010−2014	2015−2018 (*n*, %)	Median (IQR) 2015−2018	2019−2023 (*n*, %)	Median (IQR) 2019−2023
Spain	191 (54%)	192 (191−225)	127 (34%)	124 (114−159)	77 (22%)	77 (62−89)
Colombia	15 (4%)	14 (11−20)	21 (5%)	18 (11−27)	71 (17%)	71 (56−98)
Venezuela	8 (2%)	7 (7−8)	23 (6%)	24 (21−48)	48 (12%)	48 (39−60)
Brazil	25 (7%)	25 (17−28)	45 (11%)	38 (22−45)	30 (9%)	30 (30−36)
Argentina	14 (4%)	14 (10−14)	17 (5%)	18 (14−31)	31 (8%)	22 (22−48)
Perú	11 (3%)	10 (11−14)	21 (5%)	11 (9−21)	24 (6%)	25 (24−37)

**P244: Figure 1:**
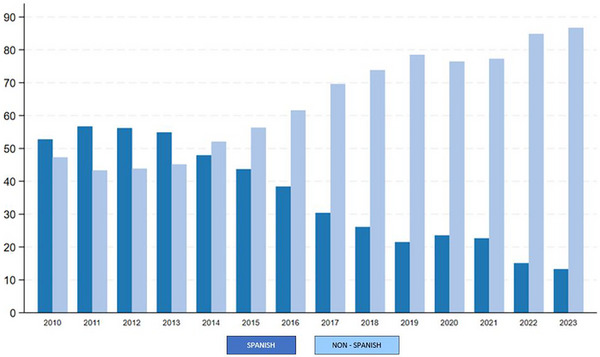
First HIV consultations at Hospital Clinic of Barcelona: immigrants versus non‐immigrants (2010–2023).

### P245 Modena and Emilia‐Romagna HIV surveillance: the application of ECDC HIV Modelling Tool

Marianna Menozzi ^1^, Adriana Cervo^1^, Gianluca Cuomo^1^, Margherita Digaetano^1^, Beatrice Fontana^1^, Alessandra Soffritti^1^, Erika Massimiliani^2^, Vanni Borghi^1^, Giovanni Guaraldi^1^, Cristina Mussini^1^

^1^Infectious Disease Clinic, University Hospital of Modena—Azienda Ospedaliero Universitaria Policlinico di Modena, Modena, Italy. ^2^Public Health Department, Emilia‐Romagna Region, Bologna, Italy

**Background**: The HIV Modelling Platform is an online instrument offered by European Centre for Disease Prevention and Control (ECDC) for the HIV surveillance data analysis, to obtain estimates of HIV epidemiological indices in a given population. The aim of this study was to interpret the available HIV surveillance data from Modena and Emilia‐Romagna Observatories using the HIV Modelling Platform tool.

**Materials and methods**: The London Method of the Tool was applied to HIV surveillance data from Modena and Emilia‐Romagna local Observatories since 1985 and 2006, respectively. Populations were defined according to ECDC guidelines for aggregated data: annual total number of HIV and/or AIDS diagnosis, deaths and stratification according to CD4 cell count at diagnosis (≥500, 350–499, 200–349 and <200 cells/mmc). Data on AIDS incidence and mortality were available only in Modena Observatory.

**Results**: Figure 1 depicts trends and estimates of HIV epidemic in the province of Modena: estimates for the HIV and AIDS diagnosis over time were aligned with those observed (Figure 1a, b); the number of HIV diagnoses and undiagnosed people decreased with time (Figure 1c, d); data and estimates regarding CD4 cell count stratification were aligned to the estimates (Figure 1e‐h). In particular, HIV diagnoses with CD4 >350 cells/mmc decreased over time, while in the last 2 years, an increase in the proportion of diagnoses with CD4 count 200–350 cells/mmc was observed. These trends in the number of new HIV and AIDS diagnoses and in the proportion of advanced diagnoses (CD4 <350 cells/mmc—data not shown) were confirmed at regional level with a powerful sample size, although with shortened follow‐up duration.

**Conclusions**: The incidence of new HIV infection and AIDS diagnoses decreased during the follow‐up time both in Modena and Emilia‐Romagna, with estimates confirmed by the ECDC HIV Modelling Platform Tool. Improvements over time were observed in the estimates of undiagnosed individuals and the average time of diagnosis, but there is still a significant proportion advanced/late diagnosis. This trend could reflect lower HIV transmission levels due to prevention campaigns, changes in the transmission route over the years, effective antiretroviral therapy and pre‐exposure prophylaxis introduction in the last years, but more effort is necessary to reduce time to diagnosis.

**P245: Figure 1:**
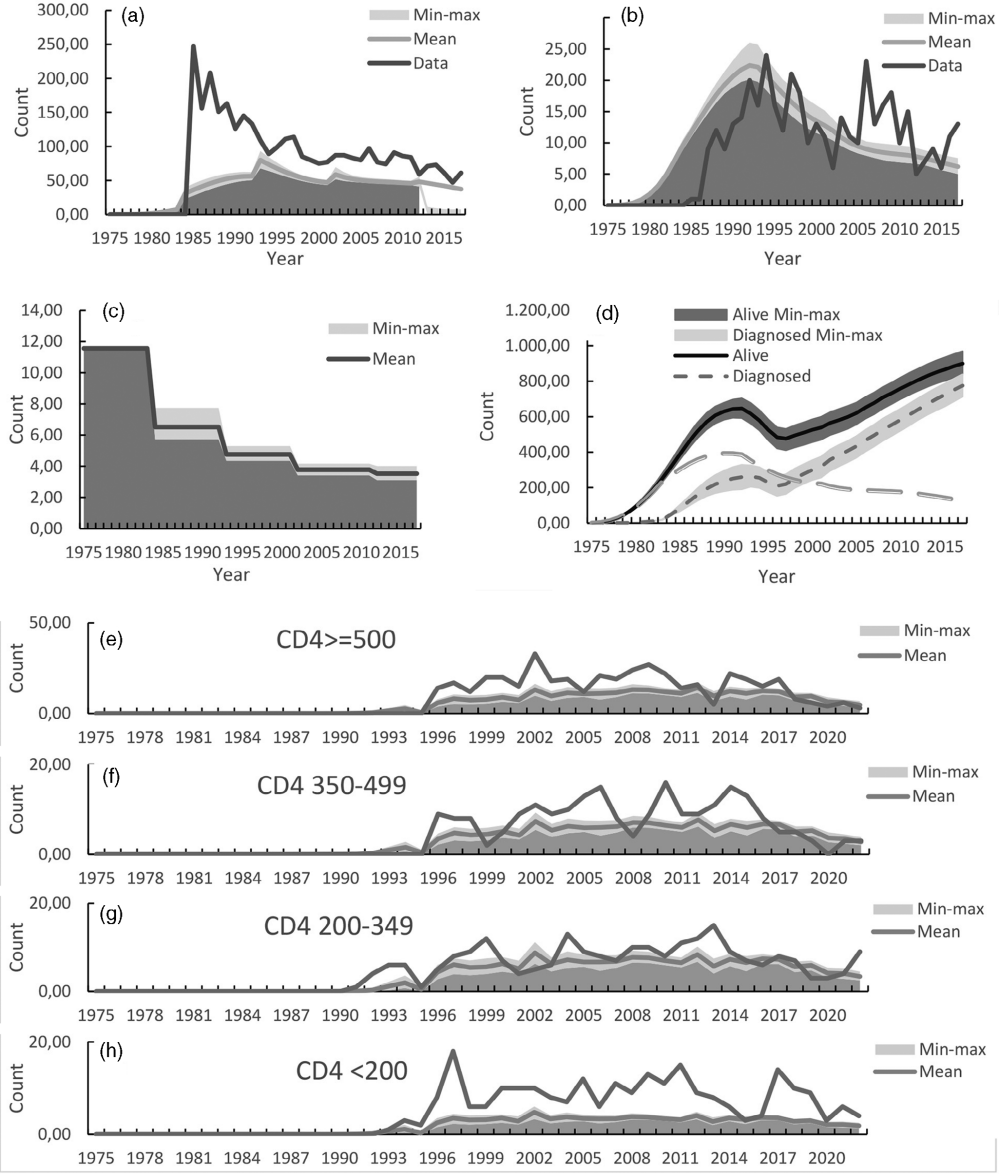
(a) Number of HIV diagnoses per year. The dotted grey line represents the expected trend of HIV diagnoses, that is aligned with the green one representing the observed data on HIV diagnosis in Modena provincial Observatory; (b) number of HIV/AIDS diagnoses per year; (c) average time from infection to diagnosis; (d) trend of HIV infection number (blue line) together with the estimate of undiagnosed infection (green line). Number of HIV infection over time according to CD4 cell count strata at diagnosis; (e) ≥500 cells/mmc; (f) 350–499 cells/mmc; (g) 200–349 cells/mmc; (h) <200 cells/mmc. The bold line represents the observed data from Modena Observatory collected since 1990. The light grey line represents the estimate according to the ECDC HIV Modelling Platform Tool.

### P246 CHildren Of the Cohort study (CHOC): the unseen population behind people living with HIV

Clara Merlin ^1^, José Damas^2^, Olivier Nawej Tshikung^3^, Noémie Wagner^4^, Anna Hachfeld^5^, Andrea Duppenthaler^6^, Marcel Stoeckle^7^, Malte Kohns^8^, Irene A. Abela^9^, Paolo Paioni^10^, Luigia Elzi^11^, Lisa Kottanattu^12^, Patrick Schmid^13^, Christian R. Kahlert^14^, Katharina Kusejko^15^, Pierre Alex Crisinel^16^, Pascal Pellegrino^17^, Matthias Cavassini^1^, Katharine Darling^1^

^1^Infectious Diseases Service, University Hospital Lausanne, Lausanne, Switzerland. ^2^Infectious Disease Service, University Hospital Lausanne, Lausanne, Switzerland. ^3^Division of Infectious Diseases, University Hospital Geneva, Geneva, Switzerland. ^4^Hôpital des Enfants, University Hospital Geneva, Geneva, Switzerland. ^5^Department of Infectious Diseases, Bern University Hospital, University of Bern, Bern, Switzerland. ^6^Department of Pediatrics, Unit of Pediatric Infectious Diseases, Bern University Hospital, University of Bern, Bern, Switzerland. ^7^Department of Infectious Diseases, University Hospital Basel, University of Basel, Basel, Switzerland. ^8^University Children's Hospital, University of Basel, Basel, Switzerland. ^9^Department of Infectious Diseases and Hospital Epidemiology, Institute of Medical Virology, University Hospital Zurich, University of Zurich, Zurich, Switzerland. ^10^Division of Infectious Diseases and Hospital Epidemiology, University Children's Hospital, Zurich, Switzerland. ^11^Division of Infectious Diseases, Regional Hospital Lugano, Lugano, Switzerland. ^12^Institute of Pediatrics of Southern Switzerland, Regional Hospital Bellinzona, Bellinzona, Switzerland. ^13^Division of Infectious Diseases, Infection Prevention and Travel Medicine, Cantonal Hospital St. Gallen, St. Gallen, Switzerland. ^14^Infectious Diseases and Hospital Epidemiology, Children's Hospital of Eastern Switzerland, St. Gallen, Switzerland. ^15^Division of Infectious Diseases and Hospital Epidemiology, University Hospital Zurich, Zurich, Switzerland. ^16^Unit of Pediatric Infectious Diseases and Vaccinology, Service of Pediatrics, Department Woman‐Mother‐Child, Lausanne University Hospital and University of Lausanne, Lausanne, Switzerland. ^17^Author, Papa Gay, Lausanne, Switzerland

**Background**: Antiretroviral therapy enables an excellent life expectancy for people with HIV (PWH) in Switzerland, and has reduced to virtually zero the vertical and horizontal transmission rate, resulting in an increase in children with ≥1 parent living with HIV. While children born to women with HIV are followed up by HIV physicians during their first 2 years of life, children of men with HIV, children born prior to a parent's diagnosis, those aged >18 years old or not living with the parent may be undocumented. This study aimed to quantify and describe such “unseen” children in Switzerland.

**Methods**: From September 2022 until October 2023, adult PWH (participants) enrolled in the Swiss HIV Cohort Study (SHCS) were invited to complete a structured questionnaire. The questionnaire was co‐designed with patient and public involvement and quantified participants’ children and the socio‐demographic parameters of each child. There were no exclusion criteria.

**Results**: Of 2565 participants, median age 54 years (IQR 35–61), 1052 (41%) were parents, having mean 1.3 children. Men represented 51.9% of parents (546/1052), of whom 26.2% were men who have sex with men (MSM) (143/546). We documented 1978 children, median age 24.9 years. Although 38.1% lived with the parent (753/1978), the parent was the source of financial support in 52.3% of cases (1034/1978). Living with the parent and receiving financial support decreased as the child aged. Most of the children (1709, 86.4%) were HIV negative; HIV status of 237 children (12%) was unknown to their parents. Parental HIV status was shared with 35.2% of children, the proportion increasing as the children aged.

**Conclusions**: In this sample of PWH, 41% were parents, of whom 13.6% were MSM. Over half of parents still supported their children financially, even if the children lived elsewhere. Questions on parenthood are not routinely asked at SHCS follow‐up visits. Omitting this social parameter potentially underestimates financial and psychosocial responsibilities among PWH. Further, parents may benefit from healthcare professional support when considering sharing their HIV status with children. Finally, “unseen” children also means “unseen” parents and reversing this could help to normalize HIV and counteract HIV‐related stigma.

### P247 Assisted partner notification for HIV: a qualitative study in Cape Town, South Africa

Shehani Perera ^3^, Alison Swartz^1^, Jennifer Nyawira Githaiga^2^

^1^Institute of Biomedical and Medical Education, University of London, London, UK. ^2^Division of Social and Behavioural Science, University of Cape Town, Cape Town, South Africa. ^3^Centre for Social Science Research, University of Cape Town, Cape Town, South Africa

**Background**: Assisted partner notification (APN) is a partner notification approach where trained providers assist individuals newly diagnosed with HIV to notify their partners and then link these partners to testing and treatment services. APN has been found to be more effective at increasing HIV testing and linkage to care rates than passive referral, where HIV‐positive individuals notify partners themselves. However, various factors have influenced the implementation of APN such as human rights concerns and ethical dilemmas, fear of social harm and social and cultural factors such as gender. In South Africa, there are no specific guidelines or policies informing the implementation of APN and we are unsure about how implementation unfolds. This study sought to better understand APN by exploring patients’ and providers’ experiences and perceptions of APN, taking into consideration different factors that shape partner notification.

**Methods: **This qualitative study involved 34 individual, semi‐structured interviews with providers (*n* = 10), female patients (*n* = 12) and key informants (*n* = 12) conducted between March 2021 and February 2022. A diary study using the WhatsApp social media platform and fieldwork journals triangulated the interview data. Data were analysed using thematic analysis with an intersectional lens. Ethics approval was obtained from the Human Research Ethics Council, University of Cape Town (HREC Ref: 840/2020) and the City of Cape Town (Ref: 28185).

**Results: **The study found that despite the absence of official APN guidelines, an unofficial APN process emerged which was shaped by communication, power and gender dynamics in the provider‐patient‐partner triad. Patients and providers used various strategies to navigate the challenges and complexities they encountered during the process, but ultimately, they felt unsafe and uncertain about how best to navigate without any formal APN guidance.

**Conclusions: **This study highlights the need for clear, context‐specific guidelines for implementing APN and a need to move towards a new framework for APN implementation. To this end, the study's insights were pooled to develop the “Six Pillars of APN” framework integrating key principles such as a basis in trust and relationship‐building, safe and ethical procedures, provider training, inclusive HIV services, community engagement and monitoring and auditing.

### P248 The benefits of free counselling and testing for HIV and STIs in Zurich, Switzerland: a cross‐sectional study

Leonie Arns‐Glaser, Andrea Farnham, Jan S. Fehr, Benjamin Hampel

Department of Public and Global Health, University of Zurich, Zurich, Switzerland

**Background**: In Switzerland, costs for HIV and sexually transmitted infection (STI) testing for people without clinical signs of an infection are generally borne by the tester. To improve access to testing for people with low income, the city of Zurich began offering free voluntary counselling and testing (VCT) in June 2023 for HIV, syphilis, chlamydia, gonorrhoea for people living in the city and either under the age of 26 or experiencing poverty. We aimed to assess the intervention's benefits to participants.

**Materials and methods**: We evaluate this intervention with a mixed methods study, collecting data from pre‐visit questionnaires that include socio‐behavioural data, clinical testing results, an anonymous digital feedback questionnaire (FBQ) for participants after their visit and qualitative interviews with the healthcare professionals administering the tests.

**Results**: In the first year of the intervention, 3475 people came for free testing. Eighty‐three percent (*N* = 2866) agreed to share their data. Twenty‐one percent (*N* = 719) completed the FBQ. Median (IQR) age of participants was 24 (23–26) years. Fifty‐four percent were assigned male at birth and 46% female. The biggest demographic groups were people identifying as women having sex with men (*N* = 1231, 43%), people identifying as heterosexual men (*N* = 830, 29%) and men having sex with men (MSM) (*N* = 683, 24%). Four HIV diagnoses were confirmed through the intervention, all of them in the group of MSM. The infection with the highest positivity rate was chlamydia (4.5%), followed by gonorrhoea (2.5%). MSM also showed the highest positivity rate in all tests. Eighty‐three percent (*N* = 587) of FBQ responders indicated that without the free testing they would have gotten tested less frequently or not at all. Eighty percent (*N* = 568) of FBQ responders indicated that the counselling increased their sexual health knowledge. After knowing one's test results (*N* = 637, 89%), the most frequently reported benefit (*N* = 400, 56%) was a non‐judgemental conversation about their sexuality. Similarly, healthcare professionals identified money and insufficient sexual education as major hurdles to obtaining adequate testing in this population.

**Conclusions**: The intervention increased the testing behaviour in this population. MSM showed the highest burden of all infections. The study shows a high benefit of linking HIV and STI testing to counselling.

### P249 Understanding knowledge and attitudes regarding HIV among secondary care healthcare professionals in the United Kingdom: a national survey

Moses Shongwe^1^, Michael Brady^2^, Daniel Clutterbuck^3^, Alyscia Curtis^4^, Hilary Curtis^5^, Nadi Gupta^6^, Claire Hutton^4^, Neal Marshall ^7^, Jane Nicholls^8^, Hajra Okhai^9^, Joel Paparello^10^, Melissa Perry^11^, Laura Waters^12^, John White^11^

^1^Grahame Hayton Unit, Bart's Health NHS Trust, London, UK. ^2^Sexual Health and HIV, King's College Hospital NHS Foundation Trust, London, UK. ^3^Chalmers Centre, NHS Lothian, Edinburgh, UK. ^4^Business Analytics & Insights, Gilead Sciences Ltd, London, UK. ^5^London, UK. ^6^Rotherham Sexual Health Services, The Rotherham NHS Foundation Trust, London, UK. ^7^Medical Affairs, Gilead Sciences Ltd, London, UK. ^8^Department of Sexual Health, Cardiff Royal Infirmary, Cardiff, Wales, UK. ^9^Institute for Global Health, UCL, London, UK. ^10^HIV Standards Support Team, Gilead Sciences Ltd, London, UK. ^11^Western and Northern Health & Social Care Trusts, Northern Ireland, UK. ^12^British HIV Association (BHIVA) and Department of HIV and Sexual Health, Mortimer Market Centre, London, UK

**Introduction**: Recent data demonstrate that stigma and misconceptions about HIV remain common among healthcare professionals (HCPs). We sought to explore knowledge and attitudes about HIV among UK HCPs working in non‐HIV or sexual health settings to identify areas for education and training.

**Methods**: A national, online survey was deployed from 14th November 2023 until 8th April 2024. This unvalidated 17‐item survey was developed in collaboration with UK HIV clinicians, researchers and a pharmaceutical company. Questions focused on HIV knowledge, occupational practices and acceptability of training. Email, social media and in person meetings were used to engage HCPs. Descriptive analysis of outcomes is presented here.

**Results**: Responses were received from 328 HCPs: 15% were doctors, 53% nurses and 43% reported over 20 years of experience. Outcomes are shown in Table 1. In response to “*How confident, if at all, are you in your knowledge and understanding of the medical conditions that should always prompt an HIV test*?” 45% reported they were confident (13% very confident), and 49% reported they were not confident (14% not at all confident). Almost all (98%) agreed that “*All healthcare workers should receive training so they know up‐to‐date information on HIV in the UK today.”*

**P249: Table 1:** Outcomes of survey

Question:	Agree/support/true	Disagree/oppose/false
There is zero risk of someone who is taking effective HIV treatment passing on HIV through sex	54%	32%
A person living with HIV can have children who are HIV negative	85%	3%
People established on effective HIV medication can expect a normal life expectancy	95%	2%
**Which of the following additional precautions, if any, would you take to protect yourself or other staff?**	**I would**	**I wouldn't**
Treat a person living with HIV in a side room	12%	82%
Make other healthcare workers aware via patient notes	63%	26%
Wear additional personal protective equipment	25%	69%
Arrange additional cleaning of clinical rooms following procedures	29%	63%
**How much, if at all, do you agree or disagree with the following statements about HIV?**	**Agree**	**Disagree**
When caring for a person with HIV, I am comfortable discussing their HIV status	72%	7%
I feel at risk of acquiring HIV when looking after people living with HIV	10%	79%
I feel confident offering an HIV test to people I care for	59%	14%
People with HIV should have the right to withhold their HIV diagnosis from medical professionals if they choose to	27%	50%

*Note*: % may not add to 100% where responses include “don't know”/“neither”/“prefer not to say.”

**Conclusions**: Although questions yielded largely correct responses, there are clear areas for education and training, for example a third did not agree with the well‐established zero risk of sexual transmission for people on suppressive treatment. Responses related to infection control indicate a need for clear, evidence‐based occupational guidance. These results suggest that a national response to educate all HCPs is needed and would be accepted in order to better support people living with HIV.

### P250 Migration as a driver of change in characteristics of new referrals of people living with HIV in a tertiary hospital in Lisbon, Portugal

Pedro Leal, Constança Antunes, Margarida Almeida, João Borralho, Sofia Rocha, Ana Cláudia Miranda, Teresa Baptista, Kamal Mansinho

Serviço de Doenças Infeciosas e Medicina Tropical, Unidade Local de Saúde Lisboa Ocidental—Hospital Egas Moniz, Lisboa, Portugal

**Background**: Migrations have become a central talking point of Portuguese and European politics in recent years. However, the role of these population shifts in reshaping the landscape of people living with HIV and their impact on healthcare systems is not often discussed. In Portugal, antiretroviral medication is freely accessible for migrants. However, initial admission into the healthcare system is still a challenge. Despite only representing 11.5% of registered residents, people born outside Portugal represented 50% of new notifications for HIV infection in 2022 [1,2].

**Materials and methods**: In this retrospective observational study, we gathered information regarding new referrals of people living with HIV from 2019 to 2023 to the Infectious Disease Department of a tertiary hospital in Lisbon, including both new and previous diagnoses, and analysed how migrations are changing population characteristics, and how it may impact the healthcare system.

**Results**: One thousand and fifty‐nine HIV‐related referrals were analysed in the 5‐year span. Almost all patients were infected with HIV‐1. Of these, 446 (42%) were new diagnoses and 613 (58%) had a previously established diagnosis and were taking medication before coming to Portugal. Approximately 73% of referrals were attributed to patients born outside of Portugal. The most common place of birth was Brazil (*n* = 431; 40.7%); followed by Portugal (*n* = 287; 27.1%). Regarding new diagnoses, the most common place of birth was Portugal (*n* = 172; 38.6%), followed by Brazil (*n* = 130; 29.1%), whereas in previously diagnosed patients, the most common place of birth was Brazil (*n* = 301; 49%), followed by Portugal (*n* = 115; 18.8%). Migrant population included a higher percentage of female (25.6% vs. 20.9%), mostly from African countries. 15.5% of the previously diagnosed migrant patients had been under treatment previously but presented with positive viral load, which suggested delays in linkage to care.

**Conclusions**: Our study reveals a growing number of migrants among people living with HIV in Lisbon, Portugal. The different demographic and cultural characteristics of this population present a challenge for health systems, which have to be able to adapt in order to provide access to care and retain all people living with HIV in the country.

**References**

1. Direção‐Geral da Saúde, Instituto Nacional de Saúde Doutor Ricardo Jorge (DGS, INSA). Infeção por VIH em Portugal – 2023. Lisbon: DGS, INSA; 2023.

2. Duarte AL, Brito J, Assunção J, Matos Águas P. Acesso à saúde por imigrantes com infeção VIH em Portugal. Lisbon: SER+ Associação Portuguesa para a Prevenção e Desafio à Sida; 2024.

### P251 Transmitted drug resistance to antiretroviral therapy: interim analysis from a cross‐sectional study including 11 healthcare centres in Chile

Maria Elena Ceballos ^1^, Cinthya Ruiz‐Tagle^1^, Felipe Castañeda^2^, Marcela Ferrés^3^, Angélica Domínguez de Landa^4^, Manuel Espinoza^4^, María Elvira Balcells^1^

^1^Enfermedades Infecciosas del Adulto, Pontificia Universidad Católica de Chile, Santiago, Chile. ^2^School of Medicine, Pontificia Universidad Católica de Chile, Santiago, Chile. ^3^Enfermedades Infecciosas e Inmunología Pediátricas, Pontificia Universidad Católica de Chile, Santiago, Chile. ^4^Salud Pública, Pontificia Universidad Católica de Chile, Santiago, Chile

**Background**: HIV morbi‐mortality and their transmission have been reduced with antiretroviral therapy (ART). Nonetheless, treatment failure can occur by acquiring a mutated strain conferring resistance to antiretrovirals. National transmitted resistance to antiretroviral drugs (TDR) was 10.45% in 2018 and is increasing worldwide. We aimed to determine the prevalence of global TDR and the relevance of incorporating the genotyping study in naïve people living with HIV in Chile.

**Materials and methods**: Observational, cross‐sectional study conducted in 11 healthcare centres located in seven regions of Chile. The sample was selected to be representative of the national population. Individuals ≥18 years old with HIV diagnosis in the last 12 months and without prior ART were invited to participate. Individuals with HIV viral load <1000 RNA copies/ml were excluded. RNA genotyping of the reverse transcriptase, protease and integrase genes was done by nested PCR with subsequent Sanger sequencing. The percentage of individuals with TDR was determined and TDR‐associated mutations were identified according to the HIV Drug Resistance Database, Stanford University.

**Results**: Between February 2023 and July 2024, 151 participants have been sequenced (final sample size 164). The average age was 35.8 years (18–78), with 88.7% belonging to the Chilean population. 85.4% were male, of which 72.2% had sex with men. The most common viral strain was subtype B (67.1%). Overall TDR (to any ART family) was 15.2% (*n* = 23). TDR for each family and mutations are shown in Table 1 and Figure 1. In addition, other mutations were found, but they were not included in the TDR percentage because they are not in the World Health Organization TDR list. Five participants presented resistance to more than one ART family; one to NRTI and NNRTI, one to NRTI and INSTI and three to NNRTI and INSTI.

**P251: Table 1:** Transmitted resistance to antiretroviral drugs (only mutations included in the WHO list)

	Total (*N* = 151)
HIV subtype	
B	100 (66.2%)
BF	48 (31.8%)
AG	2 (1.3%)
C	1 (0.7%)
Overall TDR	23 (15.2%)
Presence of TDR to NRTI	3 (2.0%)
Mutations that confer resistance to NRTI	
D67N	2 (50%)
L210W	1 (25%)
K219Q	1 (25%)
Presence of TDR to NNRTI	10 (6.6%)
Mutations that confer resistance to NNRTI	
K103N	8 (80%)
Combinations of mutations that together confer resistance	2 (20%)
Presence of TDR to PI	1 (0.7%)
Presence of TDR to INSTI	14 (9.3%)
Mutations that confer resistance to INSTI	
E92G	1 (5.88%)
T97A	1 (5.88%)
Q148K	1 (5.88%)
E157Q	2 (11.77%)
G163K	2 (11.77%)
G163R	10 (58.82%)

Abbreviations: INSTI, integrase strand transfer inhibitor; NNRTI, non‐nucleoside reverse transcriptase inhibitor; NRTI, nucleoside reverse transcriptase inhibitor; PI, protease inhibitor; TDR, transmitted resistance to antiretroviral drugs; WHO, World Health Organization.

**Conclusions**: In Chile, an increase is observed in the percentage of TDR to ART compared to what has been historically reported. The main families affected are the NNRTIs and the INSTIs (mostly first generation). Considering these preliminary results, it is considered pertinent to incorporate the baseline genotyping study in patients starting ART with NNRTI efavirenz or rilpivirine and first‐generation INSTI.

**P251: Figure 1:**
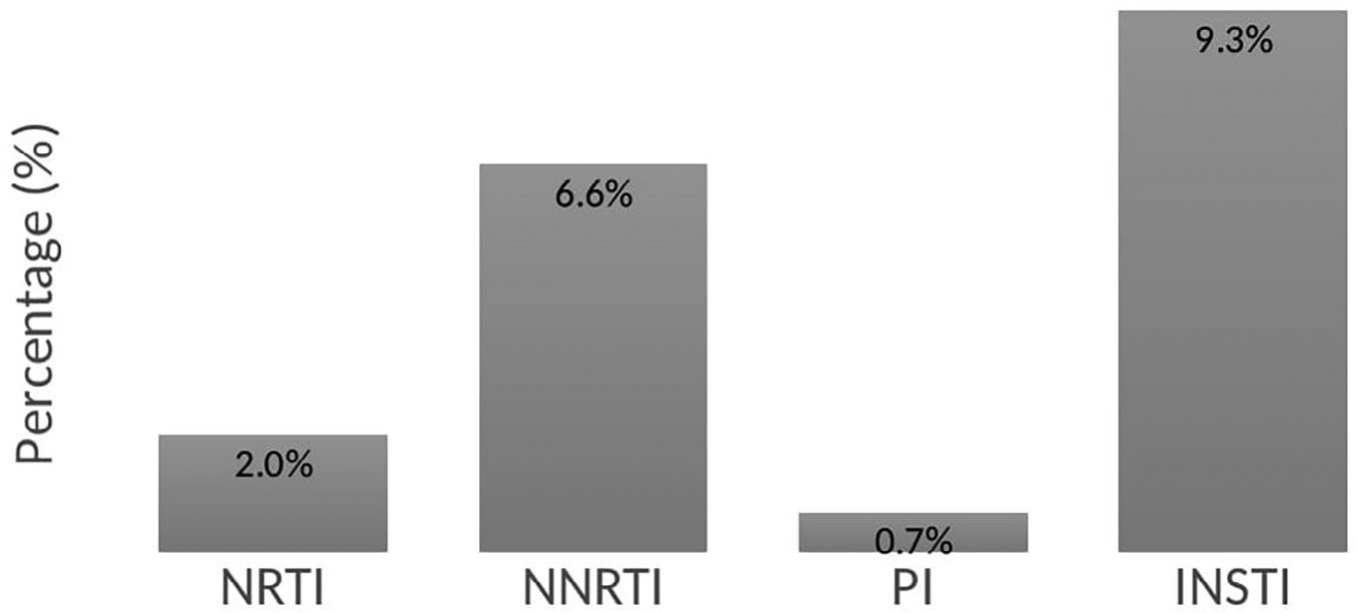
Transmitted drug resistance by ART family. ART, antiretroviral therapy; INSTI, integrase strand transfer inhibitor; NNRTI, non‐nucleoside reverse transcriptase inhibitor; NRTI, nucleoside reverse transcriptase inhibitor; PI, protease inhibitor.

### P252 Abstract withdrawn

### P253 CHildren Of the Cohort study (CHOC): exploring parenting desire among people living with HIV in Switzerland

Clara Merlin ^1^, José Damas^1^, Olivier Nawej Tshikung^2^, Noémie Wagner^3^, Anna Hachfeld^4^, Andrea Duppenthaler^5^, Marcel Stoeckle^6^, Malte Kohns^7^, Irene A. Abela^8^, Paolo Paioni^9^, Luigia Elzi^10^, Lisa Kottanattu^11^, Patrick Schmid^12^, Christian R. Kahlert^13^, Katharina Kusejko^14^, Pierre Alex Crisinel^15^, Pascal Pellegrino^16^, Matthias Cavassini^1^, Katharine Darling^1^

^1^Infectious Diseases Service, University Hospital Lausanne, Lausanne, Switzerland. ^2^Division of Infectious Diseases, University Hospital Geneva, Geneva, Switzerland. ^3^Hôpital des Enfants, University Hospital Geneva, Geneva, Switzerland. ^4^Department of Infectious Diseases, Bern University Hospital, University of Bern, Bern, Switzerland. ^5^Department of Pediatrics, Unit of Pediatric Infectious Diseases, Bern University Hospital, University of Bern, Bern, Switzerland. ^6^Department of Infectious Diseases, University Hospital Basel, University of Basel, Basel, Switzerland. ^7^University Children's Hospital, University of Basel, Basel, Switzerland. ^8^Department of Infectious Diseases and Hospital Epidemiology, Institute of Medical Virology, University Hospital Zurich, University of Zurich, Zurich, Switzerland. ^9^Division of Infectious Diseases and Hospital Epidemiology, University Children's Hospital, Zurich, Switzerland. ^10^Division of Infectious Diseases, Regional Hospital Lugano, Lugano, Switzerland. ^11^Institute of Pediatrics of Southern Switzerland, Regional Hospital Bellinzona, Bellinzona, Switzerland. ^12^Division of Infectious Diseases, Infection Prevention and Travel Medicine, Cantonal Hospital St. Gallen, St. Gallen, Switzerland. ^13^Infectious Diseases and Hospital Epidemiology, Children's Hospital of Eastern Switzerland, St. Gallen, Switzerland. ^14^Division of Infectious Diseases and Hospital Epidemiology, University Hospital Zurich, Zurich, Switzerland. ^15^Unit of Pediatric Infectious Diseases and Vaccinology, Service of Pediatrics, Department Woman‐Mother‐Child, Lausanne University Hospital and University of Lausanne, Lausanne, Switzerland. ^16^Papa Gay, Author, Lausanne, Switzerland

**Background**: Although HIV is now a chronic condition with an excellent life expectancy, some personal aspects affecting people with HIV (PWH), such as parenting desire, are relatively understudied. This study aimed to explore parenting desire among PWH in Switzerland.

**Methods**: From September 2022 until October 2023, PWH (participants) enrolled in the Swiss HIV Cohort Study (SHCS) were invited to complete a structured questionnaire co‐designed with patient and public involvement. There were no exclusion criteria. The questionnaire asked participants about parenting desire, the perceived impact of HIV on parenting desire and whether parenting desire had been discussed with their HIV physicians.

**Results**: Of 8417 PWH attending SHCS clinic visits, 2685 (31.9%) completed the questionnaire; 2565 (95.5%) complete datasets were analysed. Participants’ median age was 54 years (IQR 35–61), 27.2% were female, 45.1% were men who have sex with men (MSM), 16.5% were of African origin and 32.9% had completed tertiary‐level education. Most participants (95.6%) had HIV‐RNA plasma viral loads <50 copies/mm^3^ and 41% already had ≥1 child. Overall, 423 (16.5%) expressed parenting desire for the future, a desire associated with being aged <45 years and of African origin, and 433 (16.9%) stated that HIV impacted on family planning decisions. Only 21.3% of participants had discussed parenting desire with their HIV physicians, discussion being associated with being female, having tertiary‐level education and having parenting desire. Examining only participants who expressed parenting desire, 72% of heterosexual women had had discussions with their HIV physician against 46% of heterosexual men and only 13% of MSM.

**Conclusions**: In this sample of PWH, a minority expressed parenting desire, possibly due to the high median age of participants. Of participants who did express parenting desire, we observed a marked disparity between men and women in terms of who had discussed parenting with their HIV physicians. This disparity was further marked between heterosexual men and MSM. While surrogacy is prohibited in Switzerland, MSM have access to parenthood in other ways. Our findings suggest room for improvement whereby HIV physicians proactively invite parenting desire discussion among all PWH.

### P254 Public knowledge, views, perceptions and attitudes towards HIV and people living with HIV in Switzerland: results of a national survey

Thomas Grabinger ^1^, Marlon Gattiker^2^, David Haerry^3^, Corinna Oberle^1^, Dominique Braun^4^, Katharine Darling^5^

^1^Medical Affairs, Gilead Sciences Switzerland Sàrl, Zug, Switzerland. ^2^Expert Commission PLWH (People Living With HIV), Swiss AIDS Federation, Zurich, Switzerland. ^3^Positivrat, Positive Council Switzerland, Bern, Switzerland. ^4^Division of Infectious Diseases and Hospital Epidemiology, University Hospital of Zurich, University of Zurich, Zurich, Switzerland. ^5^Infectious Diseases Service, Lausanne University Hospital, Lausanne, Switzerland

**Background**: Limited public knowledge and awareness of HIV and perceptions and attitudes towards people living with HIV (PLWH) may result in barriers to healthcare, impacting early diagnosis, access to specialist services, retention in care and quality of life. A multinational European survey was performed to gather insights into public opinion and knowledge of HIV, HIV transmission, PLWH and the U═U (undetectable = untransmittable) message, to evaluate where focus is needed in future education campaigns. We report the results from Switzerland.

**Materials and methods**: A semi‐structured HIV public opinion survey was conducted in November 2023 using a questionnaire with closed‐ended questions to capture public stances on knowledge of HIV and PLWH. Participants aged ≥18 years were recruited by a panel institute providing population surveys by random selection and representative quota sampling across demographic variables such as age, gender, educational level and geographic location. Survey respondents were economically incentivized.

**Results**: A total of 1015 individuals (respondents) took part in the survey (Table 1). Approximately 25% of respondents knew one or more PLWH, and 45% had ever been HIV‐tested. The majority of respondents rated themselves as very (13%) or quite well (70%) informed, most were aware of advances in HIV research in the past decade, and 75% agreed that HIV can be managed with antiretroviral therapy (ART). Despite this, 60% believed that HIV can be acquired through sex with PLWH on effective treatment, and 22% believed that HIV can be acquired through kissing. Only 22% of respondents agreed with the U═U message (Figure 1a). Knowledge of the effect of modern ART on horizontal or vertical transmission was low (56% and 45%, respectively, responding “I don't know”). U═U awareness was higher among respondents who were younger, more highly educated and living in urban areas (Figure 1b–e).

**P254: Table 1:** Demographics of survey respondents

Respondents	
Total	1015 (100%)
Gender	
Male	498 (49.1%)
Female	515 (50.7%)
Diverse	2 (0.2%)
Age group	
≥18–30 years	168 (16.6%)
31–40 years	173 (17.1%)
41–50 years	175 (17.3%)
51–60 years	188 (18.6%)
61–70 years	183 (18.1%)
>70 years	128 (12.6%)
Living area	
Urban (in a city or close to a city)	824 (81.2%)
Rural (far away from a city)	191 (18.8%)
Region	
Lake Geneva Region	202 (19.9%)
Espace Mittelland	238 (23.4%)
Northwestern Switzerland	145 (14.3%)
Zurich Region	182 (17.9%)
Eastern Switzerland	142 (14.0%)
Central Switzerland	102 (10.0%)
Ticino	4 (0.4%)
Educational level	
School/completed compulsory schooling	158 (15.6%)
Apprenticeship/secondary school without leaving exam	470 (46.3%)
Secondary school with leaving exam/university degree	358 (35.3%)
Employment status	
Full‐time employed	387 (38.1%)
Part‐time employed	215 (21.2%)
Self‐employed	40 (3.9%)
Pupil/student	52 (5.1%)
Retired	228 (22.5%)
Unemployed	22 (2.2%)
Stay‐at home parent/house wife/house man	32 (3.2%)
Disabled, unable to work	28 (2.8%)
Don't want to specify	10 (1.0%)

**P254: Figure 1:**
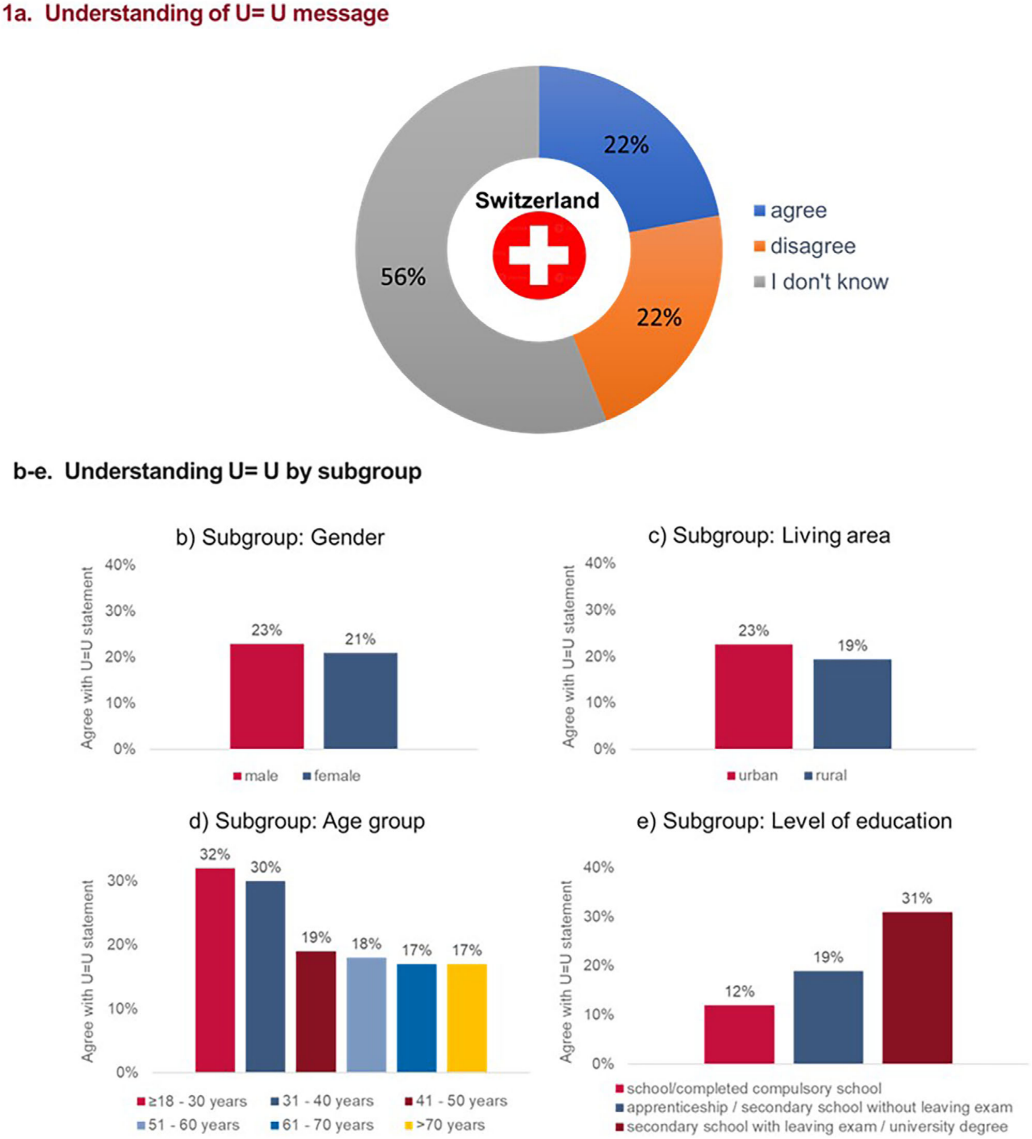
Understanding the U═U message. All respondents (a), subgroup analyses by gender (b), living area (c), age group (d) and level of education (e).

**Conclusions**: In this representative national survey, lack of knowledge regarding HIV and PLWH appeared across different gender, age and education‐level groups, which could propagate HIV‐related stigma. Effective and continued awareness campaigns are needed to spread the U═U message, help foster a more informed public stance regarding PLWH and promote an evidence‐based understanding of sexual health.

### P255 Once HIV knowledge is addressed: HIV‐stigma from the perspective of healthcare professionals working in HIV facilities in French‐speaking Switzerland

Clara Le Saux^1^, Ingrid Gilles^2^, David Jackson‐Perry^3^, Ellen Cart‐Richter^4^, Olivier Nawej Tshikung^5^, Katharine Darling ^6^

^1^Department of Epidemiology and Health Systems, Centre for Primary Care and Public Health (Unisanté), Lausanne, Switzerland. ^2^Department of General Management, Lausanne University Hospital, Lausanne, Switzerland. ^3^Infectious Diseases Service, Lausanne University Hospital, Lausanne, Switzerland. ^4^Conseil Consultatif, Lausanne University Hospital, Lausanne, Switzerland. ^5^Infectious Diseases Department, Geneva University Hospital, Geneva, Switzerland. ^6^Infectious Diseases Service, Lausanne University Hospital (CHUV), Lausanne, Switzerland

**Background**: Stigmatizing behaviour towards people with HIV (PWH) by healthcare professionals (HCPs) has been observed when HCP HIV knowledge is lacking. We conducted a qualitative study among HCPs working in HIV healthcare facilities to determine the impact of HIV‐stigma on daily practice when HIV knowledge is good.

**Methods**: HCPs working in urban HIV healthcare facilities in French‐speaking Switzerland were recruited from three professional groups (administrative staff, nurses and physicians) by judgement sampling. An interview guide adopting a thematic approach was developed, based on available literature and through patient and public involvement. Semi‐structured interviews were conducted by a researcher trained in qualitative methods. Interview transcripts were analysed using IRaMuTeQ lexicographic computer‐assisted software.

**Results**: Ten HCPs were interviewed: two administrative staff, four nurses and four physicians, working in university hospitals or private practice. Computer‐assisted analysis identified three main themes: (1) clinic reception, (2) care provision for PWH and (3) HIV knowledge. Administrative staff described a complex workload generated by maintaining different approaches to patient confidentiality, such as enabling patients to wait outside the clinic instead of the waiting room and maintaining different clinic dates for PWH who know each other socially but without sharing their HIV status. Nurses and physicians emphasized the importance of a holistic approach to care, including HIV care provision by the same team long‐term. Physicians described underestimating stigma experienced by PWH but also insufficient time during medical consultations to fulfil their medical role and discuss stigma. Lack of HIV knowledge in the general population and among non‐HIV‐specialist HCPs was described by all HCP groups as perpetuating HIV‐stigma.

**Conclusions**: HIV‐stigma can impact daily practice and care provision, even when lack of knowledge, experience or resources is removed from the equation. Reviewing well‐intentioned efforts to encourage PWH to avoid HIV status sharing may help to address rather than reinforce HIV‐stigma. Ensuring continuity of care in HIV facilities requires prioritization for HIV as a chronic condition. Finally, while improving public and non‐HIV‐specialist HCP knowledge could help to tackle HIV‐stigma, collaborative projects acting at different levels (individuals, professional groups, institutions) would be effective in adapting HCP practices.

### P256 Undetectable = Untransmittable, are we transmitting the message correctly?

Natalia Laufer^1^, Lorena Abusamra^1^, Leonel Cruces ^2^, María José Rolón^1^, Alicia Sisto^1^

^1^Infectious Diseases Unit, Hospital Juan A. Fernández, Buenos Aires, Argentina. ^2^Instituto INBIRS, CONICET ‐ Universidad de Buenos Aires, Buenos Aires, Argentina

**Background**: “Undetectable = Untransmittable” (U═U) in the context of HIV has revolutionized the paradigm of transmission. We aim to determine the level of information about U═U in the Argentinean population and its association with other parameters such as age, gender, residence, educational level and source of information.

**Materials and methods**: A national survey was conducted through the Google Forms platform between 05/10 and 05/29/2024. It was disseminated via social media, emails and WhatsApp. A descriptive analysis was performed, and associations were evaluated with Chi^2^ (Stata 14).

**Results**: A total of 4678 surveys (Table 1) were obtained from residents of Argentina, 77% of which were from the Buenos Aires region (AMBA). The percentage of cis men who correctly understood U═U was significantly higher than that of cis women (*p *= 0.0001). Regarding age, the percentage of individuals between 35 and 50 years who correctly knew the meaning of U═U was significantly higher than those <18 or >65 years, *p* = 0.0061. No significant differences were observed between other age groups nor in relation to educational level and residence. Although only 42% of healthcare workers knew the correct concept of U═U, it was significantly higher than the observed in the general population (*p* = 0.001). Regarding sources of information, those who demonstrated the best knowledge were those who searched the concept on the internet on their own motivation (50%) versus those who learned about U═U through campaigns (32%, *p* = 0.01).

**P256: Table 1:** Surveys responses according to participant characteristics

	Individuals who reported that they know what U═U mean	They had the right concept: It is not transmitted through sexual relations	Wrong concept: They considered that it means that it is not transmitted as long as a condom is used	Wrong concept: They considered that it is not transmitted through sexual relations or through blood
Health personnel *n* = 1287 (27.5%)	1125 (87.4%)	472 (42%)	161 (14.3%)	492 (38.2%)
Tertiary/university/higher *n* = 3487 (74.5%)	2665 (76.4%)	962 (30.1%)	442 (16.6%)	1261 (47.3%)
Primary/secondary *n* = 1191 (25.4%)	851 (71.4%)	291 (34.2%)	204 (24%)	356 (41.8%)
Cis women *n* = 3712 (79.3%)	2813 (75.8%)	954 (34%)	544 (19.3%)	1315 (46.7%)
Cis men *n* = 883 (18.8%)	633 (71.7%)	276 (43.6%)	92 (14.5%)	265 (41.8%)
Trans women *n* = 13 (0.27%)	6 (100%)	1 (16.6%)	3 (50%)	2 (33.3%)
Trans men *n* = 7 (0.14%)	6 (85.7%)	2 (33.3%)	0 (0%)	4 (66.6%)
Reported to learn about U═U on social networks *n *= 1022 (21.8%)	Not applicable	390 (38%)	137 (13.4%)	495 (48.4%)
Reported to learn about U═U through health campaigns *n* = 474 (10%)	Not applicable	156 (33%)	108 (22.7%)	210 (44%)
Reported to found out about U═U through internet searches *n* = 85 (1.8%)	Not applicable	40 (47%)	13 (15.3%)	32 (37.6%)
Age between 35 and 50 years *n *= 2146 (46%)	1593 (74%)	573 (35.9%)	275 (17.2%)	745 (46.7%)
Age <18 years *n* = 112 (2.4%)	65 (58%)	14 (21%)	24 (37%)	27 (41%)
Age >65 years *n* = 154 (3.2%)	101 (65%)	28 (27.7%)	45 (44.5%)	28 (27.7%)

**Conclusions**: This study presents biases such as the high percentage of residents in the AMBA region, aged 35–50 years, highly educated and cisgender women, which does not allow these results to be generalized to the entire Argentine population. However, from these data, it is clear that there is a misunderstanding of the U═U message, especially when it is also assume as the lack of transmission through blood, which can pose a serious risk. It is also very concerning that less than 50% of healthcare workers knew the concept adequately. This highlights a failure in how the message has been conveyed in our environment and underscores the importance of designing campaigns that reach the population and correctly incorporate U═U.

### P257 Opt‐out blood borne virus (BBV) testing in emergency medicine (EM) in a low prevalence setting

Daniela Brawley ^1^, Pauline Dundas^2^, Lisa Allerton^3^, Noha El Sakka^4^, Gareth Patton^5^

^1^Sexual Health/Public Health, NHS Grampian, Aberdeen, UK. ^2^Hepatology, NHS Grampian, Aberdeen, UK. ^3^Public Health, NHS Grampian, Aberdeen, UK. ^4^Virology, NHS Grampian, Aberdeen, UK. ^5^Emergency Medicine, NHS Grampian, Aberdeen, UK

**Background**: Grampian has a low prevalence of HIV; however, it has challenges with late diagnosis and missed opportunities for testing, in addition to an increase in diagnoses since 2022. With the success of NHS England's opt‐out BBV testing programme, a Scottish Government‐funded pilot was performed to assess this intervention in a low‐prevalence setting [1,2]. This assessed operational feasibility, testing uptake, new diagnoses and/or cases relinked to care and number needed to test, in addition to staff acceptability and system impact.

**Methods**: The pilot was performed from 15/1/24 to 15/5/24, funded for 4500 test bundles. IT processes were updated to allow EM opt test bundles for HIV, hepatitis B (HBV) and C (HCV). All adult patients attending EM having phlebotomy were offered testing. Public and staff communications were circulated prior to and during the pilot. Positive results were actioned by BBV teams. Testing uptake was measured against haematology results as a surrogate marker via laboratory IT systems. New diagnoses, relinked to care cases and clinical outcomes were reported by BBV teams. This was compared to a ready reckoner of predicted case numbers in low‐prevalence areas [3]. Staff qualitative interviews were performed, followed by thematic analysis.

**Results**: Four thousand, six hundred and two test bundles were performed. Mean testing uptake was 40% (Figure 1). BBV test results, clinical outcome and actual versus expected number needed to test to diagnose a new case or relink to care are shown in Table 1. Themes from staff qualitative interviews assessing acceptability and system impact include: high acceptance from EM staff who felt “proud”; minimal impact to EM workload; opportunities to test people who may not access testing elsewhere; offer/uptake impacted by EM activity and timing of pilot (winter); extended pilot may increase offer/uptake; costs higher than predicted, especially for laboratory processes; more time for public and staff communications and EM training.

**P257: Figure 1:**
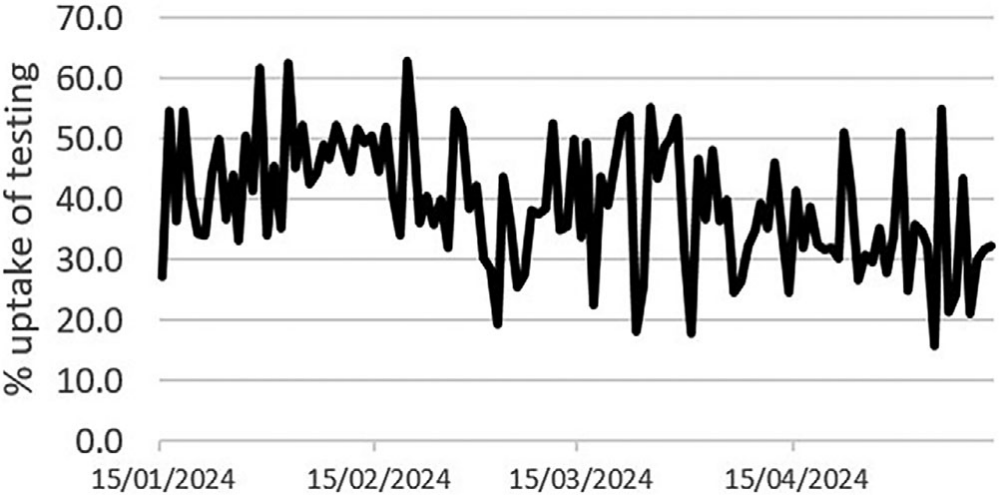
% Uptake of BBV testing.

**P257: Table 1:** BBV test results, clinical outcome and number need to test

	New diagnosis (*<5)	Relinked to care (*<5)	Clinical outcome (*<5)	Number need to test per case undiagnosed/not in care	Expected number need to test from ready reckoner
HIV	0	*	Relinked to care‐*	4602	6000
HBV (surface antibody positive)	9	0	Linked to care‐8, not engaged‐*, treatment commenced‐5	511	625
HCV (antibody and PCR positive)	14	0	Linked to care‐7, not engaged‐6, treated‐5	329	417

**Conclusions**: Despite the short timeframe, this pilot shows EM opt‐out BBV testing is feasible in low‐prevalence areas with lower numbers needing to test than expected. Further pilots would increase the evidence base and determine cost effectiveness, especially with continued late diagnoses and missed opportunities, with associated healthcare costs, morbidity and mortality.

**References**

1. NHS England. Emergency department opt out testing for HIV, hepatitis B and hepatitis C: the first 100 days [Internet]. 2022 [cited 2024 Aug 28]. Available from: https://www.england.nhs.uk/long‐read/emergency‐department‐opt‐out‐testing‐for‐hiv‐hepatitis‐b‐and‐hepatitis‐c‐the‐first‐100‐days/.

2. Hill‐Tout R. ED testing—challenges and lessons of a combined BBV approach. BHIVA Spring Conference; 2024 Apr 29‐May 1; Birmingham, UK.

3. Hutchison S. Scottish BBV seroprevalence ready reckoner. Personal communication; 2024.

### P258 U═U: awareness and impact on the quality of life in PWH; experience of a Greek HIV unit

Konstantinos Protopapas, Onisiphoros Neophytou, Charalampos D. Moschopoulos, Georgios Zampetas, Konstantinos Thomas, Dimitra Kavatha, Antonios Papadopoulos

4th Department of Internal Medicine, Medical School of Athens, National and Kapodistrian University of Athens, ATTIKON University Hospital, Athens, Greece

**Background**: “Undetectable equals untransmittable” (U═U) is a message designed to communicate the proven fact that people living with HIV (PWH) cannot transmit HIV to their sexual partners if their viral load is undetectable. This empowering message could lead to better health outcomes, amelioration of quality of life and reduction of HIV‐related stigma. The aim of this study was to investigate the awareness of the U═U message among a cohort of PWH.

**Methods**: Single‐centre, cross‐sectional study conducted in December 2023. Patients followed in the HIV‐outpatient clinic of ATTIKON University Hospital in Athens, Greece, were asked to fill out a questionnaire with 14 closed‐ended questions on their awareness regarding the U═U message and a questionnaire regarding health status (Medical Outcome Study‐HIV Health Survey, MOS‐HIV). Demographics and data regarding HIV were collected.

**Results**: Five hundred and twenty‐six PWH in this preliminary analysis (male 94%, mean age: 45 ± 10.4 years). Four hundred and twenty‐six of 526 (81%) have heard of the U═U message, but 24.1% of them were not aware of its meaning. 54.9% have heard of it from their healthcare professional (HCP), 29.88% through the social media, 18% through the media and 15.9% were informed by a friend or their partner. Four hundred and seventy‐eight of 526 (92.3%) were aware that PWH cannot transmit HIV to their sexual partners if their viral load is undetectable, but only 58.7% have discussed it in detail with their HCP. 81.2% and 76.8% reported amelioration of their quality of life and their sex life, respectively, knowing the U═U message. PWH aware of U═U were more likely to be MSM, younger and of a higher education level. No significant differences were seen in terms of age and gender. No significant association of the U═U message with health‐related quality of life (HRQOL) was observed, except for the domains of general health, physical functioning and emotional functioning. Finally, no correlation was seen between knowledge of U═U and adherence to medication.

**Conclusions**: Despite the relatively high awareness rates of the U═U message, it is obvious that the message has not been delivered to everyone. It is primarily the duty of both HCP and the community to spread this knowledge that will improve the quality of life of PWH.

### P259 Persistent HIV‐related stigma in Germany across different origins: findings from university hospital study

Anna Koval ^1^, Maher Almahfoud^1^, Hanna Matthews^1^, Angela Buchholz^2^, Sanna Higgen^2^, Anja‐Dorothee Huefner^1^, Friederike Theresia Marie Hunstig^1^, Luise Roggelin^1^, Sabine Jordan^1^, Stefan Schmiedel^1^, Olaf Degen^1^

^1^Outpatient Department for Infectious Diseases, University Medical Center Hamburg‐Eppendorf, Hamburg, Germany. ^2^Special Outpatient Department for Psychological Stress in HIV/AIDS, University Medical Center Hamburg‐Eppendorf, Hamburg, Germany

**Background**: The HIV/AIDS pandemic remains a global public health challenge, with millions affected. Despite progress, HIV‐related stigma and discrimination remain a widespread challenge [1]. In Germany, there are around 91,000 people living with HIV, with 1800 new cases in 2021 [2]. The 2021 Stigma Index project in Berlin revealed ongoing fear of discrimination and stigmatization among those with HIV in Germany [3].

**Materials and methods**: In April 2024, we launched a stigma study at the University Hospital Hamburg‐Eppendorf's outpatient infectious diseases department. This study aimed to examine stigma and discrimination among patients in HIV‐specific outpatient care and the differences between patients of various cultural backgrounds. We used the ECDC Stigma questionnaire [4] and the German adaptation of the HIV Berger Scale [5], translating both into six languages (German, English, Ukrainian, Russian, Spanish and French) to encourage participation from our diverse HIV cohort.

**Results**: By May 2024, 328 participants were enrolled, with nine declining. The main age groups were 40–49 years (24%) and 50–59 years (26%). Participants self‐identified themselves as heterosexual (40.5%), homosexual (37.5%), bisexual (7.9%) or did not disclose (13.7%) (Table 1). The ECDC questionnaire revealed that 15.8% (52) of participants were excluded from family activities due to their HIV status and 9% (30) experienced threats from family or partners. Thirty‐three percent (105) avoided health or dental care services out of fear of discrimination, and 19.8% (65) faced medical procedure rejection. The HIV Berger Scale results revealed that 70% (232) of respondents hide their HIV status, and 66% (217) find openness about it risky. Additionally, 76% (250) are selective about whom they inform. About 22% (72) have stopped contacting with those who discovered their status. A comparison of the largest cohorts—Germany (191) and Ukraine (43)—showed higher personalized stigma among Ukrainians with HIV (18 points vs. 13 points) (Figure 1).

**P259: Table 1:** Gender, age and self‐identification of stigma study participants

**Gender**	
Male	244 (74%)
Female	81 (26%)
**Age group**	
18–29	14 (4%)
30–39	48 (15%)
40–49	80 (24%)
50–59	84 (26%)
60–69	63 (19%)
70–79	28 (9%)
80–89	8 (2%)
**Self‐identification**	
Straight/heterosexual	127 (38%)
Gay or lesbian/homosexual	136 (41%)
Bisexual	28 (8.5%)
Other	9 (2.7%)
Prefer not to say	19 (9.8%)

**P259: Figure 1:**
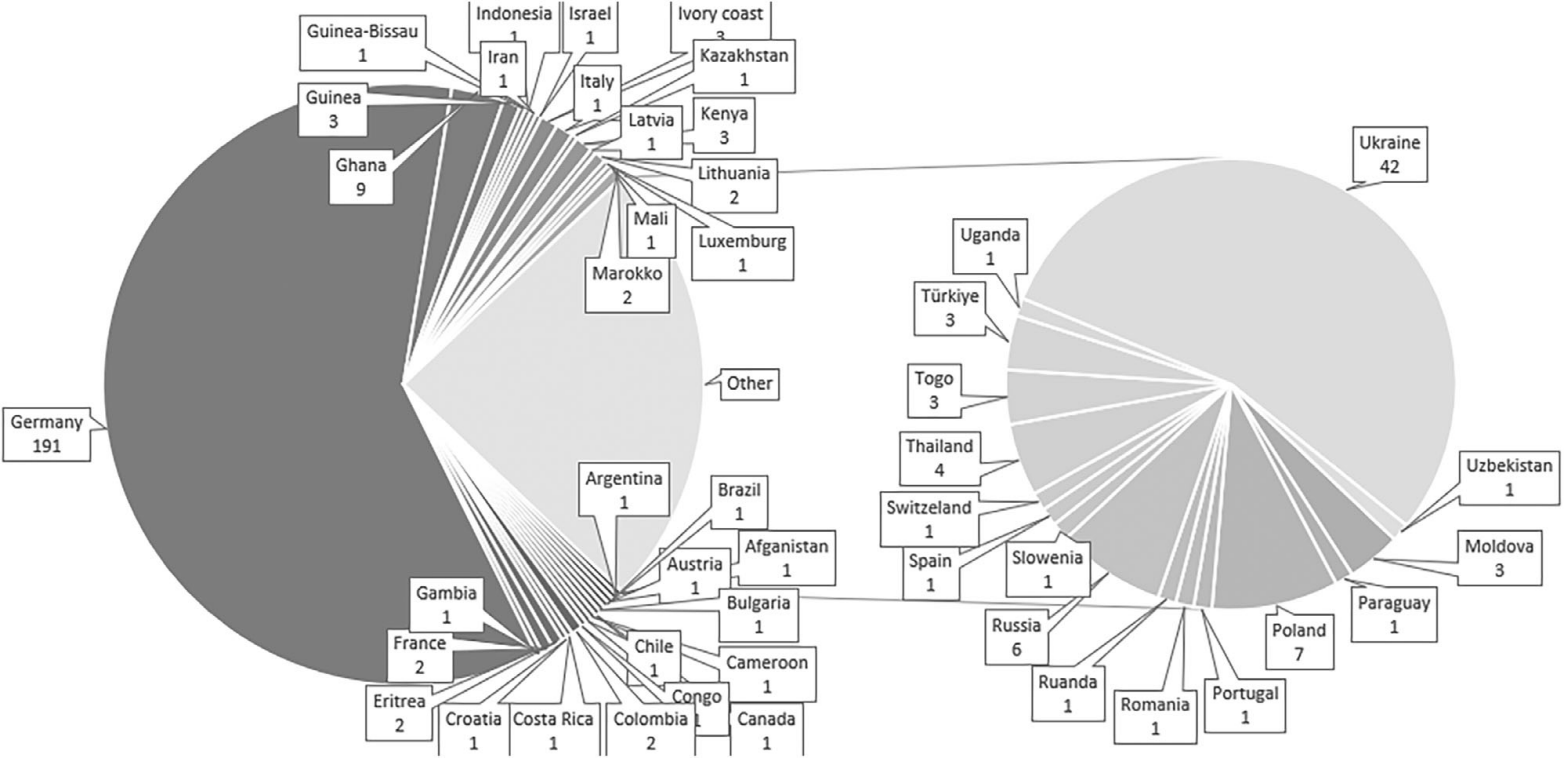
Countries of origin of stigma study participants.

**Conclusions**: Stigmatization and discrimination remain significant problems. The data indicates that discrimination against people with HIV in healthcare settings is alarmingly high. Despite the long history of HIV in society and various efforts to reduce discrimination, it persists not only within family contexts but also in healthcare settings, which is highly concerning.

**References**

1. UNAIDS. HIV‐Related Stigma, Discrimination and Human Rights Violations [Internet]. 2005 [cited 2024 Aug 12]. Available from: https://www.unaids.org/en/resources/documents/2005/20051005_jc999‐humrightsviol_en.pdf.

2. Robert Koch Institut. STIKO‐Empfehlung zur RSV‐Impfung für Personen ab 75 Jahren sowie Personen ab 60 Jahren mit Risikofaktoren [Internet]. 2024 [cited 2024 Aug 12]. Available from: https://www.rki.de/DE.

3. Deutsche Aidshilfe. Positive stimmen 2.0 [Internet]. 2021 [cited 2024 Aug 12]. Available from: https://www.aidshilfe.de/shop/archiv/positive‐stimmen‐20‐englisch.

4. European Centre for Disease Prevention and Control. Stigma: survey of people living with HIV—Monitoring implementation of the Dublin Declaration on partnership to fight HIV/AIDS in Europe and Central Asia: 2022 progress report [Internet]. 2023 [cited 2024 Aug 12]. Available from: https://www.ecdc.europa.eu/en/publications‐data/hiv‐stigma‐survey.

5. Dinkel A, Nather C, Jaeger H, Jaegel‐Guedes E, Lahmann C, Steinke C, et al. Stigmatization in HIV/AIDS: first German adaptation of the HIV‐stigma scale (HSS‐D). Psychother Psychosom Med Psychol. 2014;64(1):20‐7.

### P260 Use of participatory approach in developing interventions for HIV prevention in Africa

Abdulhammed Babatunde ^1^, Yusuf Babatunde^2^, Oluwakorede Adedeji^3^

^1^Medicine and Surgery, University of Ibadan, Ibadan, Nigeria. ^2^Drug Control, National Agency for Food and Drug Administration and Control, Lagos, Nigeria. ^3^Pharmacy, University of Ilorin, Ilorin, Nigeria

**Background**: Participatory approach such as co‐creation, crowdsourcing and designathon is a modern implementation science approach that engages multiple stakeholders, including the end‐users in the development of public health interventions. This approach fosters community engagement and empowerment and ensures the sustainability of interventions. This approach has been employed in creating human‐centred innovations in public health areas, including HIV prevention. This is a review of published articles describing the usage of crowdsourcing, designathon and co‐creation in advancing HIV prevention.

**Materials and methods**: We reviewed published literatures from online databases such as PubMed, PubMed Central and Google Scholar. Keywords used in the search strategy include designathon, co‐creation and crowdsourcing + HIV prevention + African countries. Primary studies published before March 2024 describing a participatory approach to promote HIV prevention in Africa were selected. We synthesized data on scope of the participatory activities, participants, the method of HIV prevention, facilitators and barriers of the participatory activities.

**Results**: A total of six studies were included in the review describing either crowdsourcing, designathon or co‐creation of innovations for HIV prevention in Africa. Most of the participatory activities focus on adolescents and young adults. They have been organized to promote different HIV prevention strategies such as promotion of HIV self‐testing, development of educational materials, male engagement in HIV services, promote uptake of pre‐exposure prophylaxis (PrEP), improvement of STI services, and enhancing participation of adolescents and young adults (AYA) in research. The designathon/co‐creation lasts for 3–4 days. Designathon and co‐creation have been organized in Nigeria, Mozambique, Kenya, Rwanda and South Africa. One major facilitator reported was interdisciplinary collaboration and training or workshop on human‐centred design (HCD). Major barriers include funding and expertise.

**Conclusions**: Participatory approach in HIV prevention is a potential strategy in implementation science to enhance community engagement and sustainability of HIV prevention interventions in Africa. There is a need for evaluation of designathon or co‐creation activities and their outcomes.

### P261 The attitudes towards providing care for people living with HIV among healthcare workers in Poland

Filip Fijołek ^1^, Martyna Cholewik^2^, Carlo Bienkowski^3^, Justyna D. Kowalska^3^, Agata Skrzat‐Klapaczynska^3^

^1^Ward VII, Hospital for Infectious Diseases, Warsaw, Poland. ^2^Faculty of Medicine, Medical University, Warsaw, Poland. ^3^Department of Adults' Infectious Diseases, Medical University & Ward VII, Hospital for Infectious Diseases, Warsaw, Poland

**Background**: People living with HIV constantly face stigma and discrimination. The role of healthcare workers (HCWs) in fighting stigmatization, especially self‐stigmatization, remains a key component.

**Materials and methods**: A cross‐sectional survey study was designed by the European Centre for Disease Prevention and Control and the European AIDS Clinical Society to measure HIV knowledge and attitudes in various healthcare settings in Europe and Central Asia. Responses from Poland were analysed separately and stratified by profession and time of work. Data were collected anonymously between 15 September 2023 and 30 November 2023.

**Results**: A total of 134 questionnaires were collected. The median age of participants was 41. Most of the participants were female (70.90%), physicians (57.89%), working for more than 10 years (73.85%) and worked in a hospital (66.17%). Most of respondents did not take care of people living with HIV in the year before implementing the survey (56.65%). At the same time, the majority presented up‐to‐date knowledge and assessment of the risk of HIV transmission, along with acceptance and willingness to provide services (Table 1). HCWs working for over 10 years were less worried about dressing wounds (*p* = 0.005) and drawing blood (*p* = 0.005) from people living with HIV. Physicians were more likely to agree that post‐exposure prophylaxis (*p *= 0.004) and pre‐exposure prophylaxis (*p* < 0.001) prevent the spread of the virus, that people living with HIV who have an undetectable viral load cannot transmit the virus through sexual intercourse (*p* < 0.001), and that women living with HIV can have children (*p* = 0.021). Physicians were less likely to be worried if they drew blood from a person living with HIV (*p* = 0.007). Other HCWs were more likely to avoid physical contact (*p* = 0.028) and wear double gloves (*p* < 0.001) when interacting with a person living with HIV. Other HCWs were more likely to observe a reluctance (*p* = 0.023) to take care of people living with HIV in their workplace (Table 1).

**P261: Table 1:** Distribution of responses stratified by profession and work experience

Characteristic	All, *n/N* (%)	Physicians, *n/N* (%)	Other HCWs, *n/N* (%)	Years in healthcare <10, *n/N* (%)	Years in healthcare >10, *n/N* (%)
Agreed that people living with HIV who have an undetectable viral load cannot transmit the virus sexually	89/133 (66.92)	61/77 (79.22)	28/56 (50.00)	25/34 (73.53)	62/96 (64.58)
		*p *< 0.001		*p* = 0.341	
Agreed that using post‐exposure prophylaxis prevents the virus from spreading	106/133 (79.70)	68/77 (88.31)	38/56 (67.86)	29/34 (85.29)	75/96 (78.13)
		*p *= 0.004		*p* = 0.369	
Agreed that using pre‐exposure prophylaxis prevents the virus from spreading	81/130 (62.31)	59/75 (78.67)	22/55 (40.00)	22/33 (66.67)	56/94 (59.57)
		*p *< 0.001		*p* = 0.471	
Not worried about touching the clothing of a person living with HIV	127/128 (99.22)	74/74 (100.00)	53/54 (98.15)	33/33 (100.00)	91/92 (98.91)
		*p* = 0.422		*p* = 1	
Not worried about dressing the wounds of a person living with HIV	82/114 (71.93)	55/71 (77.46)	27/43 (62.79)	14/27 (51.85)	67/84 (79.76)
		*p* = 0.091		*p* = 0.005	
Not worried about drawing blood from a person living with HIV	84/117 (71.79)	56/69 (81.16)	28/48 (58.33)	14/28 (50.00)	67/86 (77.91)
		*p* = 0.007		*p *= 0.005	
Not worried about taking the temperature of a person living with HIV	122/123 (99.19)	74/74 (100.00)	48/49 (97.96)	29/29 (100.00)	90/91 (98.90)
		*p =* 0.398		*p =* 1	
Avoided physical contact when providing care or services for a person living with HIV	8/109 (7.34)	2/67 (2.99)	6/42 (14.29)	2/25 (8.00)	7/81 (8.64)
		*p =* 0.028		*p =* 0.920	
Used double gloves when providing care or services for a person living with HIV	37/113 (32.74)	14/71 (19.72)	23/42 (54.76)	7/25 (28.00)	28/85 (32.94)
		*p*<0.001		*p =* 0.641	
Wore gloves during all aspects of the patient's care when providing care or services for a person living with HIV	59/110 (53.64)	33/69 (47.83)	26/41 (63.41)	10/24 (41.67)	48/83 (57.83)
		*p =* 0.112		*p =* 0.162	
Used any special infection‐control measures with people living with HIV that are not used with other patients	86/113 (76.11)	51/69 (73.91)	35/44 (79.55)	22/25 (88.00)	62/85 (72.94)
		*p =* 0.494		*p =* 0.119	
Observed no unwillingness to take care of people living with HIV in their workplace in the past 12 months	70/110 (63.64)	47/65 (72.31)	23/45 (51.11)	14/27 (51.85)	56/80 (70.00)
		*p =* 0.023		*p =* 0.086	
Observed no poorer quality of care provided to a person living with HIV in their workplace in the past 12 months	84/111 (75.66)	55/66 (83.33)	29/45 (64.44)	22/27 (81.48)	60/81 (74.07)
		*p =* 0.023		*p =* 0.436	
Observed no discriminatory remarks or talking badly about people living with HIV in their workplace in the past 12 months	69/118 (58.47)	45/69 (65.22)	24/49 (49.98)	15/29 (51.72)	51/86 (59.30)
		*p =* 0.078		*p =* 0.475	
Observed no disclosure of a person's HIV status without their consent in their workplace in the past 12 months	97/117 (82.91)	58/69 (84.06)	39/48 (81.25)	21/29 (72.41)	73/85 (85.88)
		*p =* 0.691		*p =* 0.100	
Agreed it is not acceptable in their facility to test a patient for HIV without their knowledge	75/131 (57.25)	45/76 (59.21)	30/55 (54.55)	16/34 (47.06)	59/94 (62.77)
		*p =* 0.594		*p =* 0.111	
Agreed that they will get in trouble at work if they discriminate against people living with HIV	75/133 (56.39)	39/77 (50.65)	36/56 (64.29)	20/34 (58.82)	53/95 (55.79)
		*p =* 0.117		*p =* 0.715	
Agreed that their facility has guidelines to protect people living with HIV from discrimination	16/133 (12.03)	9/77 (11.69)	7/56 (12.50)	3/34 (8.82)	13/96 (13.54)
		*p =* 0.887		*p =* 0.472	
Agreed that there are standardized procedures in their health facility that reduce their risk of acquiring HIV	119/133 (89.47)	69/77 (89.61)	50/56 (89.29)	31/34 (91.18)	85/96 (88.54)
		*p =* 0.951		*p =* 0.670	
Agreed that their health facility has a PEP protocol in case of needlestick injury	122/133 (91.73)	72/77 (93.51)	50/56 (89.29)	32/34 (94.12)	87/96 (90.63)
		*p =* 0.382		*p =* 0.529	
Agreed that their facility has a policy for scheduling people living with HIV on the end of an operating/procedure list	12/133 (9.02)	8/77 (10.39)	4/56 (7.14)	2/34 (5.88)	8/96 (8.33)
		*p =* 0.519		*p =* 0.645	
Agreed that in their facility there are guidelines recommending wearing double gloves when caring for people living with HIV	17/133 (12.78)	6/77 (7.79)	11/56 (19.64)	5/34 (14.71)	11/96 (11.46)
		*p =* 0.043		*p =* 0.620	
Believed that people living with HIV should be allowed to have a fulfilling sexual life	118/132 (89.39)	71/77 (92.21)	47/55 (85.45)	30/34 (88.24)	85/95 (89.47)
		*p =* 0.214		*p =* 0.842	
Believed that women living with HIV should be allowed to have babies if they wish	114/132 (86.36)	71/77 (92.21)	43/55 (78.18)	30/34 (88.24)	81/95 (85.26)
		*p =* 0.021		*p =* 0.668	
Believed that most people living with HIV have had too many sexual partners	16/132 (12.12)	9/77 (11.69)	7/55 (12.73)	1/34 (2.94)	15/95 (15.79)
		*p =* 0.856		*p =* 0.051	
Believed that people acquire HIV because they engage in irresponsible behaviours	45/132 (34.09)	26/77 (33.77)	19/55 (34.55)	8/34 (23.53)	35/95 (36.84)
		*p =* 0.925		*p =* 0.158	
Believed that HIV is punishment for bad behaviour	0/132 (0.00)	0/77 (0.00)	0/55 (0.00)	0/34 (0.00)	0/95 (0.00)
		*p =* 1		*p =* 1	
Believed that people living with HIV should feel ashamed of themselves	0/132 (0.00)	0/77 (0.00)	0/55 (0.00)	0/34 (0.00)	0/95 (0.00)
		*p =* 1		*p =* 1	
Believed that most people living with HIV do not care if they infect other people	7/132 (5.30)	3/77 (3.90)	4/55 (7.27)	1/34 (2.94)	6/95 (6.32)
		*p =* 0.393		*p =* 0.456	
Believed that people living with HIV with detectable viral loads should not be participating in sexual activity	33/132 (25.00)	16/77 (20.78)	17/55 (30.91)	8/34 (25.53)	25/95 (26.32)
		*p =* 0.185		*p =* 0.749	
Preferred not to provide care or services to people who inject prohibited drugs	19/133 (14.29)	11/77 (14.29)	8/56 (14.29)	6/34 (17.65)	12/96 (12.50)
		*p =* 1		*p =* 0.455	
Preferred not to provide care or services to men who have sex with men	3/133 (2.26)	3/77 (3.90)	0/56 (0.00)	0/34 (0.00)	3/96 (3.13)
		*p =* 0.263		*p =* 0.567	
Preferred not to provide care or services to sex workers	5/133 (3.76)	3/77 (3.90)	2/56 (3.57)	0/34 (0.00)	4/96 (4.17)
		*p =* 0.922		*p =* 0.572	
Preferred not to provide care or services to transgender men and women	4/102 (3.92)	3/57 (5.26)	1/45 (2.22)	0/23 (0.00)	4/75 (5.33)
		*p =* 0.432		*p =* 0.570	

*Note*: Chi‐square test and Fisher's exact test were used in statistical analysis.

Abbreviations: HCWs, healthcare workers; PEP, post‐exposure prophylaxis.

**Conclusions**: Majority of Polish HCWs who responded the survey presented willingness to support and provide services for people living with HIV and/or at risk of HIV. However, there is still space for improvement in education, especially among non‐doctors working in healthcare settings.

### P262 Outcomes, public health responses, lessons learned and current status of the HIV outbreak (10 year overview)

Becky Metcalfe^1^, Sharon Hutchinson^2^, Andrew McAuley^2^, Daniel Carter^3^, Julie Craik^3^, S. Erica Peters ^4^

^1^Sandyford Sexual Health Service, NHS Greater Glasgow & Clyde, Glasgow, UK. ^2^School of Health & Life Sciences, Glasgow Caledonian University, Glasgow, UK. ^3^Public Health Directorate, NHS Greater Glasgow and Clyde, Glasgow, UK. ^4^Infectious Diseases Department, NHS Greater Glasgow and Clyde, Glasgow, UK

An HIV outbreak among people who inject drugs in Glasgow city centre was declared in 2015 after an increase in cases was noted at the end of 2014. The outbreak was associated with homelessness and cocaine injecting, and in a setting of high harm reduction service coverage and free access to clinical care, medication, testing and injecting equipment. Nearly 10 years on, the IMT (incident management team) led by NHS Greater Glasgow and Clyde (NHS GGC) continues to regularly review the status of the outbreak with a wide variety of partners. Research and evaluation of the epidemiology, virology and public health response has been wide, with input from various partners from academia, HIV clinicians, addictions services and third sector organizations. This has been previously presented and published and has attracted international interest. There have been approx. 190 cases identified overall, with a high mortality rate in the cohort. A variety of combination public health interventions have been implemented and subsequently evaluated. An oral presentation with input from the various involved partners will cover an overview of the following areas, which will be of international interest to delegates: overview of the setting in Glasgow and epidemiology of the HIV outbreak; response to the outbreak: combination prevention interventions including a variety of testing interventions, HIV PrEP outreach model, active care engagement models for HIV treatment, partnership working between HIV and addiction services and novel response to opiate substitution therapy; monitoring the outbreak using incentivized survey based research—the Needle Exchange Surveillance Initiative (NESI); update on current outbreak situation and outcomes; virological phylogenetic evaluations and their use to inform interventions; modelling evaluation of interventions, including community care model—GECHO (Glasgow Enhanced Care HIV Outreach) clinical service; mortality amongst the cohort; next steps and ongoing risks in this vulnerable group; reflections and lessons learned. This will be delivered with complimentary presentations by the co‐authors, with representation from academic, clinical and public health services, who will present on behalf of the wider NHS GGC IMT group.

## Cost and cost‐effectiveness

### P263 Estimating return on investment (ROI) with increased utilization of HIV pre‐exposure prophylaxis (PrEP) among key populations in France

Turgay Ayer^1,2^, Emir Gursel^1^, Mert Edali^1,3^, Claire Idelovici‐Marchal^4^, Elias Benabadji^5^, James Jarrett^6^, Dylan Mezzio ^6^

^1^Value Analytics Labs, Boston, MA, USA; ^2^Health Systems Engineering, Georgia Institute of Technology, Atlanta, GA, USA. ^3^Department of Industrial Engineering, Yildiz Technical University, Istanbul, Turkey. ^4^Value and Access, Gilead Sciences, Paris, France. ^5^Medical Affairs, Gilead Sciences, Paris, France. ^6^Global Value and Access, HEOR, Gilead Sciences, Foster City, CA, USA.

**Background**: Pre‐exposure prophylaxis (PrEP) can help significantly reduce HIV incidence and national spending on lifelong HIV treatment. Despite the availability of oral PrEP in France since 2016, its uptake remains insufficient to impact HIV incidence. This study assessed the return on investment (ROI) of different PrEP utilization scenarios in France.

**Methods**: An HIV microsimulation model was developed to capture the clinical and net economic impact of increased oral PrEP utilization in France. The model assessed scenarios where PrEP uptake and adherence were higher among different populations with varied HIV incidence. Model inputs for disease progression and costs were derived from published real‐world data to estimate costs associated with oral PrEP (emtricitabine and tenofovir disoproxil fumarate) and HIV (Figure 1). ROI was defined as the amount in Euros of downstream HIV‐related costs averted for every additional Euro spent upfront on PrEP. A ROI greater than 1 implied a net economic savings. Model populations reflect the entire subgroup and were not restricted to only those who need or want PrEP; therefore, ROI estimates are conservative.

**P263: Figure 1:**
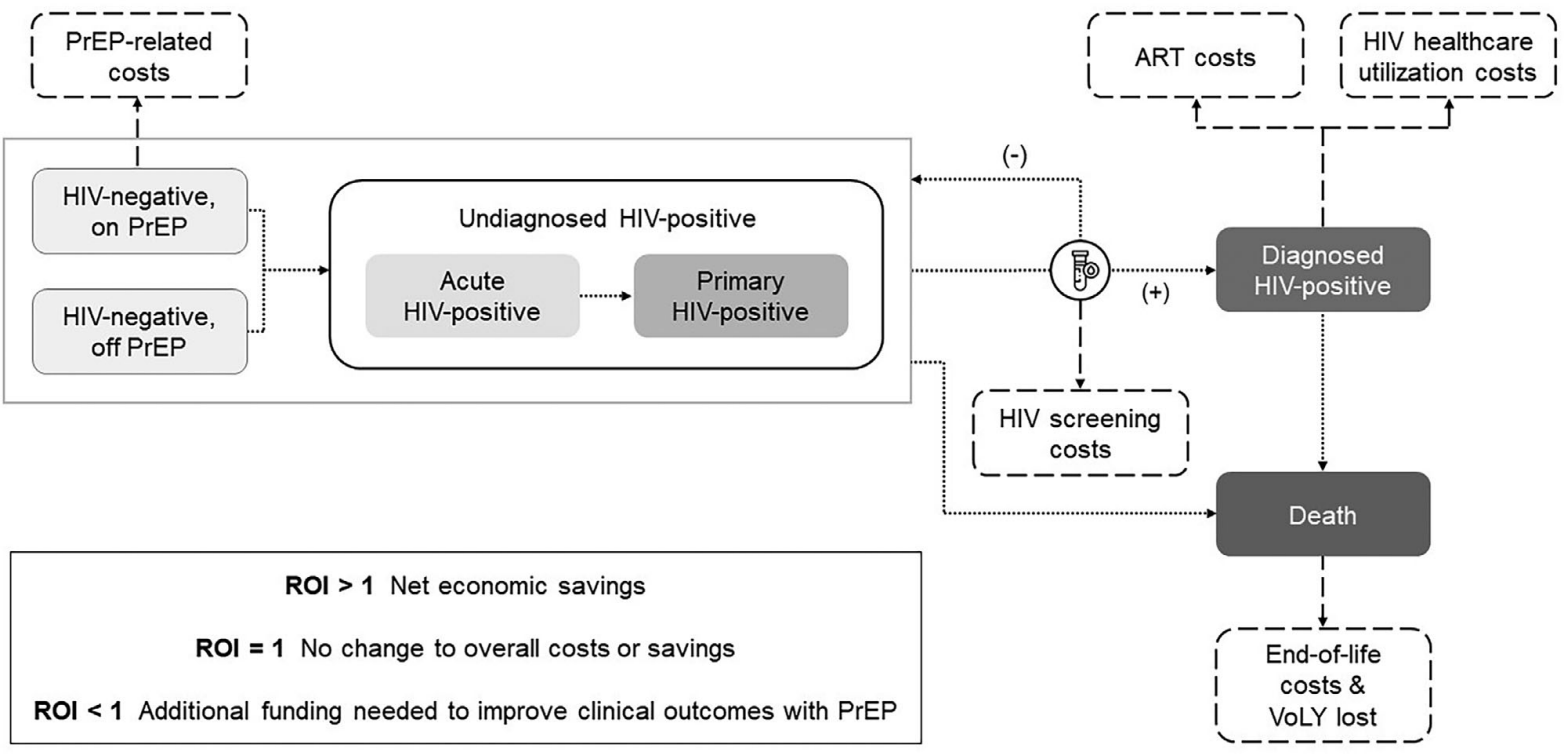
Simplified model schematic. The model used monthly probabilities to simulate the progression through various health events, which included HIV screening, PrEP discontinuation, HIV acquisition, opportunistic infections and death. Dashed lines indicate the types of costs captured in the overall model. PrEP uptake and adherence were varied and associated costs were assessed relative to base‐case levels; adherence was defined as % of days covered, with a distribution of use categorized as low (<50% use), medium (50–74% use), and high (≥75% use). ART, antiretroviral therapy; PrEP, pre‐exposure prophylaxis; ROI, return on investment; VoLY, value of life‐years lost.

**Results**: Net economic savings were observed in several hypothetical scenarios where PrEP uptake was higher than current real‐world estimates, with additional savings when PrEP adherence for all was optimized (≥75% use). For example, among all men who have sex with men, a 10% increase in uptake resulted in a ROI of 10.71, which increased to 12.08 when adherence was optimized. A net saving was also observed for transgender women with 10% higher PrEP uptake (ROI = 3.11). While the ROI was <1 for heterosexual females overall, a net saving was estimated when examining specific female sub‐populations, including sex workers (ROI = 1.56) and heterosexual women born in sub‐Saharan Africa (ROI = 3.01) (Table 1).

**P263: Table 1:** ROI across select populations with increased PrEP uptake and adherence

Sub‐population (in France)^a^	Annual HIV‐1 incidence (per 10,000 population)	ROI with 10% higher uptake^b^	ROI with 10% higher uptake^b^ and optimized adherence^c^
**MSM**	115.1	10.71	12.08
Born abroad	181.8	15.22	16.68
Born in sub‐Saharan Africa^d^	N/A	N/A	N/A
**Heterosexual men**	0.9	0.09	0.10
Born abroad	4.7	0.53	0.61
Born in sub‐Saharan Africa	19.2	2.28	2.43
**TGW**	27.1	3.01	3.11
**Sex workers**	12.4	1.43	1.56
**Heterosexual women**	0.9	0.11	0.12
Born abroad	5.0	0.56	0.64
Born in sub‐Saharan Africa	25.1	2.71	3.01

Abbreviations: MSM, men who have sex with men; N/A, not available; PrEP, pre‐exposure prophylaxis; ROI, return on investment; TGW, transgender women.

^a^ Model estimated ROI based on the entire sub‐population without filtering for PrEP eligibility; as HIV acquisition risk is higher for those considered eligible for PrEP, the presented ROI results represent a conservative estimate.

^b^ Based on available published data, the estimated current PrEP uptake in France was 12% for MSM and 0% for other categories; however, as not all individuals in these sub‐populations may need PrEP, a 10% increase in uptake represents a relatively higher impact than if the model included only individuals who need PrEP.

^c^ Optimized adherence was defined as ≥75% of days covered on oral PrEP for all individuals, based on literature that examined adherence levels and real‐world effectiveness with emtricitabine and tenofovir disoproxil fumarate.

^d^ Data not available at the time of analysis.

**Conclusions**: Our findings imply that shifting to a world where PrEP utilization is higher would lead to net economic savings (ROI > 1) while preventing HIV cases in key populations in France. The model highlights the importance of PrEP; however, without immediate investment in new advances in PrEP and increased outreach, challenges to achieve higher uptake and adherence remain.

### P264 Cost‐effectiveness and public‐health impact of cabotegravir long‐acting injectable for HIV pre‐exposure prophylaxis in Canada

Adenike Adelakun ^1^, Michael Dolph^2^, Emily Matthews^2^, Alan Oglesby^3^, Kelly Campbell^4^, Ashley Davis^5^, Janine Xu^6^, Isabelle Hardy^7^, Natalya Danchenko^8^, Sarah‐Jane Anderson^9^

^1^Health Economics and Outcomes Research (HEOR), GlaxoSmithKline, Mississauga, Canada. ^2^North American Health Economics and Outcomes Research (HEOR), Real‐world Analytics Division, Cytel, Montreal, Canada. ^3^North American Medical Affairs, ViiV Healthcare, Durham, NC, USA. ^4^Health Economics, RTI Health Solutions, Manchester, UK. ^5^Health Economics, RTI Health Solutions, Research Triangle Park, NC, USA. ^6^Health Economics and Outcomes Research, GlaxoSmithKline, Mississauga, Canada. ^7^North America Medical Affairs, ViiV Healthcare, Montreal, Canada. ^8^Global Health Outcomes, ViiV Healthcare, Brentford, UK. ^9^Value, Evidence and Outcomes, ViiV Healthcare, Brentford, UK

**Background**: In Canada, the incidence of HIV increased by 24.9% between 2021 and 2022. Cabotegravir long‐acting (CAB‐LA) injectable, the first long‐acting injectable, administered every 2 months, was approved in Canada (May 2024) as pre‐exposure prophylaxis (PrEP) to reduce the risk of sexually acquired HIV infection in at‐risk adults and adolescents, including men who have sex with men (MSM), transgender women (TGW), and cisgender women (CGW). The HIV Prevention Trials Network (HPTN) 083 (MSM and TGW) and HPTN 084 (CGW) studies demonstrated the superiority of every 2‐month CAB‐LA versus daily oral tenofovir disoproxil fumarate/emtricitabine (TDF/FTC) for PrEP.

**Materials and methods**: A decision‐analytic Markov model was used to estimate the lifetime clinical and economic impact of CAB‐LA compared with oral TDF/FTC and no PrEP from a Canadian public payer perspective. Modelled individuals initiated a PrEP option (CAB‐LA, TDF/FTC, or no PrEP) upon model entry and continued to receive their initially assigned PrEP option until discontinuation, HIV acquisition, or death. Secondary HIV seroconversions related to onwards transmission were also estimated in the model. An indirect treatment comparison including the HPTN‐083 and ‐084 studies provided an estimate of the effectiveness of CAB‐LA versus no PrEP based on the observed effectiveness of CAB‐LA versus TDF/FTC and the predicted effectiveness of TDF/FTC versus no PrEP.

**Results**: The number needed to treat (NNT) to prevent one primary HIV infection over the modelled lifetime was 13 for CAB‐LA and 19 for TDF/FTC compared with no PrEP; compared with TDF/FTC, the NNT was 37 with CAB‐LA. CAB‐LA was less costly ($174,847) and more effective (36.86 quality‐adjusted life years [QALYs]) than TDF/FTC ($192,328; 36.67 QALYs) and no PrEP ($261,682; 36.29 QALYs). These values resulted in incremental cost savings of $17,481 and QALY gains of 0.20 versus TDF/FTC, and $86,835 and 0.57 versus no PrEP (Table 1). CAB‐LA would be the dominant PrEP option based on the $50,000 willingness‐to‐pay threshold in Canada.

**P264: Table 1:** Base‐case results

	Costs	QALYs	Life years	ICER versus lowest cost intervention ($/QALY)	Primary HIV‐1 infection	Secondary HIV‐1 infections
CAB‐LA	$174,847	36.86	43.24	−	0.10	0.08
TDF/FTC	$192,328	36.67	43.23	CAB‐LA is dominant	0.13	0.10
No PrEP	$261,682	36.29	43.22	CAB‐LA is dominant	0.18	0.15

Abbreviations: CAB‐LA, cabotegravir long‐acting injectable; FTC, emtricitabine; ICER, Institute for Clinical and Economic Review; PrEP, pre‐exposure prophylaxis; QALY, quality‐adjusted life year; TDF, tenofovir disoproxil fumarate.

**Conclusions**: Overall, compared to TDF/FTC and no PrEP, the results indicate the introduction of CAB‐LA as PrEP in Canada would result in substantial public health and monetary benefits by preventing additional HIV infections and reducing the clinical and economic burden of HIV.

### P265 The cost‐effectiveness of HIV pre‐exposure prophylaxis in men who have sex with men at high risk of HIV acquisition in eight Latin American countries

Jamile Ballivian ^1^, Leandro Pastori^2^, Elena Lazo^2^, Carolina Moreno^2^, Camila Volij^3^, Velenn Penini^3^, Adrian Santoro^3^, Santiago Esteban^3^, Adolfo Rubinstein^2^, Federico Augustovski^2^, Andres Pichon‐Riviere^2^, Sebastian Garcia‐Marti^2^

^1^ETS, Institute for Clinical Effectiveness and Health Policy & Hospital Muñiz, Buenos Aires, Argentina. ^2^ETS, Institute for Clinical Effectiveness and Health Policy, Buenos Aires, Argentina. ^3^CIIPS, Institute for Clinical Effectiveness and Health Policy, Buenos Aires, Argentina

**Background**: Men who have sex with men (MSM) in Latin America experience high rates of HIV transmission [1]. Although many countries in Latin America have made progress in improving access to pre‐exposure prophylaxis (PrEP), actions to reduce the new HIV infections need to continue to be implemented [2]. We examined the epidemiological and economic outcomes of implementing pre‐exposure prophylaxis programmes in Argentina, Brazil, Chile, Colombia, Costa Rica, Ecuador, Mexico, and Peru.

**Methods**: We developed a cost‐effectiveness model of HIV prevention to evaluate the nationwide implementation of a 5‐year intervention of oral PrEP. HIV prevalence, incidence, and treatment coverage, among other parameters for each country, were from national‐based sources. PrEP effectiveness (86% HIV incidence reduction) was from literature, and PrEP drug costs ($38.93 per month) were from the Revolving Fund of the Pan‐American Health Organization. The outcomes were dollars per disability‐adjusted life year ($/DALY) expressed in 2023 USD from a health system perspective.

**Results**: With a 70% level of adherence in a 100,000 MSM cohort, we estimated that PrEP implementation averted a range from 1613 to 5799 HIV diagnosis cases (for Ecuador and Mexico, respectively) see Figure 1. In turn, the intervention resulted in 502–1433 life years saved and between 77 and 231 deaths averted for Ecuador and Chile, respectively. The incremental cost‐effectiveness ratio (ICER) ranged from $‐1407.90/DALY for Mexico to $2068.39/DALY for Ecuador. The return on investment (ROI) was positive for Chile, Costa Rica, Mexico, and Colombia, showing a higher return on the investment. However, the ROI was negative for Argentina, Brazil, Peru, and Ecuador.

**P265: Figure 1:**
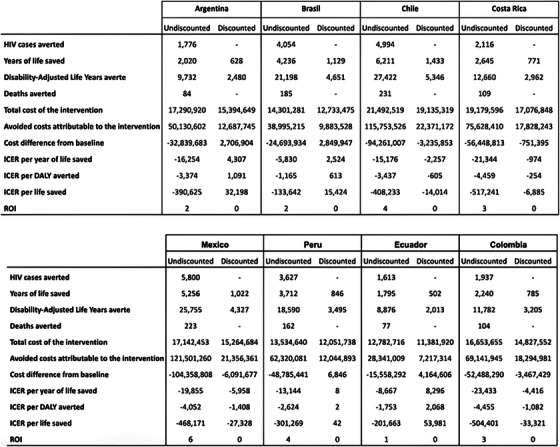
Epidemiological and economic outcomes of implementing HIV pre‐exposure prophylaxis in men who have sex with men in Argentina, Chile, Brazil, Costa Rica, México, Ecuador, Perú and Colombia.

**Conclusions**: PrEP remained cost‐saving under plausible variations in PrEP adherence for Chile, Colombia, Costa Rica, and Mexico. Daily tenofovir/emtricitabine PrEP among MSM at high risk of HIV acquisition would be cost‐effective (<1 × gross domestic product) in eight countries of Latin America.

**References**

1. United Nations. Joint United Nations Programme on HIV/AIDS (UNAIDS). In Danger: UNAIDS Global AIDS Update 2022. Geneva: Joint United Nations Programme on HIV/AIDS; 2022.

2. Guerrero‐Torres L, Sierra‐Madero JG. Implementation of PrEP in Latin America. Lancet HIV. 2023;10(2):e70‐1.

### P266 Economic estimation of the management of neuropsychiatric events in people with HIV in real life

Juan Tiraboschi ^1^, Alvaro Mena‐de‐Cea^2^, Jesus Troya Garcia^3^, Antonio Castro^4^, Pilar Cabrera^5^, Neus Vidal‐Vilar^5^

^1^HIV Unit, Infectious Diseases Service, University Hospital of Bellvitge, Hospitalet de Llobregat, Barcelona, Spain. ^2^Infectious Diseases Unit, Internal Medicine Service, University Hospital of A Coruña (CHUAC), Sergas; Clinical Virology Group, Biomedical Research Institute of A Coruña (INIBIC), A Coruña, Spain. ^3^Department of Internal Medicine, Infanta Leonor‐Virgen de la Torre University Hospital, Madrid, Spain. ^4^Market Access, Gilead Sciences, Madrid, Spain. ^5^Market Access, Outcomes'10 S.L., Castellón de la Plana, Spain

**Background**: For people with HIV (PHIV), dolutegravir (DTG) or bictegravir (BIC)‐based combinations are the standard of care for initiating therapy. However, higher rates of neuropsychiatric adverse events (NPAEs) have been reported in real‐world studies than those described in clinical trials. We aim to estimate the resource use and costs associated with managing NPAEs leading to discontinuation in PHIV treated with DTG and BIC from the Spanish National Health System perspective.

**Materials and methods**: Discontinuation rates by NPAE in real‐world patients treated with DTG and BIC were identified from a previously published systematic literature review according to their design (prospective or retrospective). The most common NPAEs leading to discontinuation (headache, sleep disorders, depression, anxiety and dizziness) and their weight (calculated as the weighted mean percentage of patients who experienced them) were used to calculate the total NPAE cost. A panel of expert clinicians estimated the resource use associated with the management of each NPAE, considering visits, tests, hospitalizations and pharmacological treatment. Costs were obtained from the Spanish databases Botplus and eSalud.

**Results**: Based on real‐world data from retrospective studies (discontinuation rate: DTG: 2.99%; BIC: 0.66%) and considering a cost per NPAE of €449.62, the cost of managing NPAEs in a cohort of 1000 patients would be €13,437 with DTG and €2947 with BIC, representing a 78.1% savings in management when patients are treated with BIC. It is estimated to save 86.08 primary care (PC) visits, 16.74 specialist visits, 4.81 emergency visits, 0.03 nursing visits, 3.44 tests and 1.08 days of hospitalization per year. In prospective studies (discontinuation rate: DTG: 2.03%; BIC: 1.98%), the cost of NPAE management would be €9118 with DTG and €8903 with BIC, representing a 2.4% savings in managing NPAE in BIC patients. It is estimated to save 1.76 PC visits, 0.34 specialist visits, 0.10 emergency visits, 0.07 tests and 0.02 days of hospitalization per year.

**Conclusions**: Real‐world discontinuation rates due to NPAEs with BIC are lower than with DTG, leading to savings in cost and resource use for their management. Findings from this study could help treatment decision‐making in PHIV.

### P267 Cost‐effectiveness analysis of ainuovirine versus efavirenz in HIV population initiating first‐line combination antiretroviral therapy

Chenlu Yang^1^, Yuanqi Mi^2^, Yuhong Zeng^3^, Feng Cheng^4^, Yufang Zheng^5^, Chunyun Zhang^5^, Hong Qin ^5^, Huatang Zeng^6^, Mengge Zhou^1^

^1^Department of Epidemiology and Biostatistics, Institute of Basic Medical Sciences Chinese Academy of Medical Sciences; School of Basic Medicine, Peking Union Medical College, Beijing, China. ^2^Department of Epidemiology, Bloomberg School of Public Health, Johns Hopkins University, Baltimore, MD, USA. ^3^Department of Epidemiology, College of Preventive Medicine, Army Medical University (Third Military Medical University), Chongqing, China. ^4^Vanke School of Public Health, Tsinghua University, Beijing, China. ^5^Clinical Research & Development, Jiangsu Aidea Pharmaceutical Co., Ltd, Yangzhou, China. ^6^Shenzhen Health Development Research and Data Management Center, Shenzhen, China

**Background**: Efavirenz (EFV) based combinational regimen is the preferred first‐line antiretroviral therapy for people living with HIV (PLWH) in China. However, EFV‐associated adverse reactions are major safety concerns for both PLWH and healthcare providers. Ainuovirine (ANV), a new‐generation non‐nucleoside reverse transcriptase inhibitor, is a promising therapeutic option alternative to EFV, with comparable effectiveness but less adverse reactions. However, previous studies remained limited for comparing cost‐effectiveness.

**Methods**: A Markov model for medical decision‐making was used to assess the cost‐effectiveness of EFV‐ and ANV‐based regimens, with most probability parameters derived from multi‐centre cohorts in China, and cost parameters obtained from multi‐centre cross‐sectional survey data in China. The remaining parameters, which could not be acquired through surveys, and effectiveness data were obtained from literature reviews. Outcomes were measured in quality‐adjusted life years (QALYs). A 15‐year (30 cycles) cost‐effectiveness analysis was conducted from a societal perspective. The stability of the model was evaluated based on univariate sensitivity analysis and probabilistic sensitivity analysis.

**Results**: Compared to EFV‐based regimen, use of ANV‐based regimen resulted in a significant reduction in cumulative adverse events. From a societal perspective, the incremental cost‐effectiveness ratio of ANV‐based regimen was USD 3844.88 per QALY, significantly lower than three times the gross domestic product (GDP) per capita (USD 30,793.15/QALY). The model remained robust in the sensitivity analysis, with the tornado diagrams indicating costs of ANV and EFV as the most influential factors. Further analysis suggested that if the cost of ANV fell below USD 393.86 per 0.5 year, ANV‐based regimen would absolutely become the dominant strategy with lower costs and greater effectiveness.

**Conclusions**: From a societal perspective, ANV‐based regimen proves to be more economically advantageous for PLWH in China compared to EFV‐based regimen, as the preferred‐over‐EFV therapy option in China.

## Models of care: Evaluation of ARV delivery and coverage

### P268 Is continuum of care for HIV and hepatitis C possible during war migration: Five Times Ninety project

Justyna Kowalska ^1^, Olena Samsonova^1^, Sergii Antoniak^2^, Bartlomiej Matlosz^3^

^1^Department of Adults Infectious Diseases, Medical University of Warsaw, Warsaw, Poland. ^2^Viral Hepatitis and AIDS Department, Gromashevsky Institute of Epidemiology and Infectious Diseases, Kyiv, Ukraine. ^3^HIV‐Outpatients Clinic, Hospital for Infectious Diseases in Warsaw, Warsaw, Poland

**Background**: Five Times Ninety project investigates linkage to care among war refugees living with HIV displaced from Ukraine to Poland.

**Materials and methods**: Ukrainian refugees living with HIV who registered to HIV care in Warsaw were followed according to “Standardized protocol for clinical management and medical data sharing for PLHIV among refugees from Ukraine” developed by the World Health Organization/Centre of Excellence for Health, Immunity and Infections (WHO/CHIP), the European AIDS Clinical Society (EACS), the Euroguidelines in Central and Eastern Europe Network Group (ECEE) and European Centre for Disease Prevention and Control (ECDC) (https://iris.who.int/handle/10665/353083). For each patient who signed an informed request, data were linked to database at the Public Health Centre (PHC) Ministry of Health Ukraine, and sent back on a standardized form.

**Results**: Among 205 refugees who registered to HIV care in Warsaw (5 Mar 2022–31 Aug 2023), 202 (98.7%) were linked with the PHC database, 121 (59.9%) were female, median age 40.0 (range 18 69, IQR 36.0 46.0), migrating from Central (67; 33.2%), South (65; 32.2%), East (39; 19.3%), West (27; 13.4%) and North (4; 2.0%) Ukraine. Mode of HIV infection was heterosexual in 113 (55.9%), other/unknown in 51 (25.2%), injecting drug use in 28 (13.9%) and MSM in 10 (4.9%) persons. Women and persons who acquired HIV through heterosexual contacts were more likely to migrate earlier, but age structure did not significantly change over time. On 31 December 2023, in total, 128 (63.4%) patients remained in care in Warsaw. One hundred and eighty‐nine patients (93.1%) had HIV viral load measured both in Ukraine and Poland, 173 (91.5%) and 155 (82.0%) were undetectable, respectively. This translates into 9.5% loss of ART efficacy during a transition period. These improved over time (Figure 1). Switching of antiretroviral therapy occurred often, yet the majority remained on integrase inhibitors (194; 98.0%) and tenofovir (176; 88.9%), 148 persons (73.6%) were tested with HCV serology in Ukraine and 187 (93.0%) in Poland. Of 59 (31.5%) tested positive in Poland, 24 were post‐DAA therapy in Ukraine. Of 35 who were tested with PCR in Poland, 10 had undetectable HCV RNA, 25 had detectable HCV RNA, of whom 10 already went through DAA therapy.

**P268: Figure 1:**
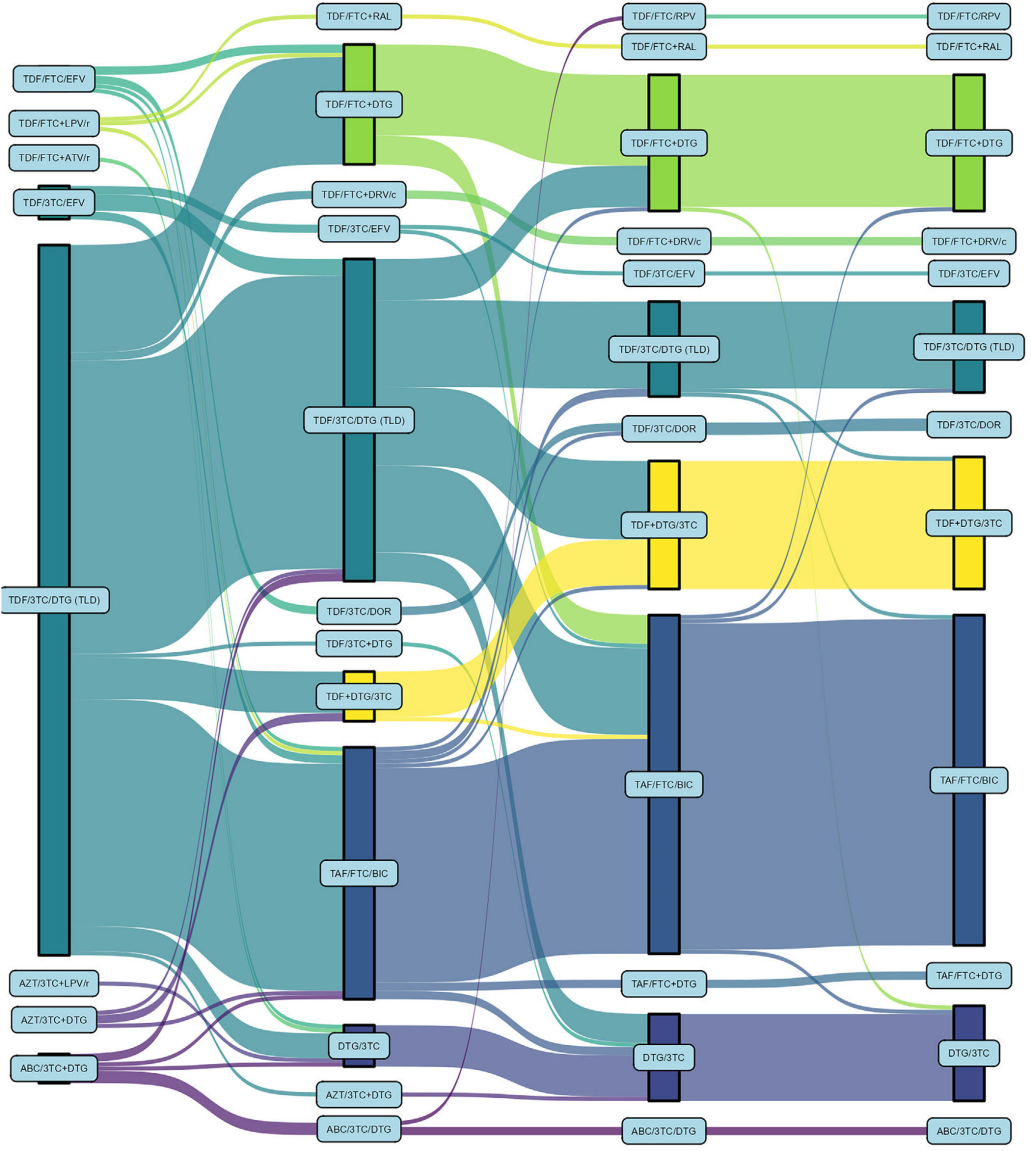
Changes in antiretroviral therapy regimens.

**Conclusions**: Viral suppression among Ukrainian refugees on ART decreased slightly, but in general remained high and improved with time. Routine HCV screening of refugees from Ukraine is a necessity, and there are no restraints on DAA treatment in Poland.

### P269 Sociodemographic factors influencing retention in care among people with HIV immigrants at Hospital Clinic of Barcelona: a 12‐year retrospective cohort study

Alexy Inciarte ^1^, Elizabeth Zamora‐Clemente^2^, Leire Berrocal^1^, Berta Torres^1^, Lorena De La Mora^1^, Elisa de Lazzari^1^, Montserrat Laguno^1^, Maria Martinez‐Rebollar^1^, Ana González‐Cordón^1^, Alberto Foncillas^1^, Ivan Chivite^1^, Julia Calvo^1^, Abiu Sempere^1^, Juan Ambrosioni^1^, Jose Luis Blanco‐Arevalo^1^, Jose M. Miró^1^, Esteban Martinez‐Chamorro^1^, Josep Mallolas^1^

^1^HIV‐Unit, Hospital Clinic, Barcelona, Spain. ^2^Medicine, University of Barcelona, Barcelona, Spain

**Background**: Immigration profoundly impacts Europe, stressing the healthcare challenges of people with HIV (PWH), necessitating focused analysis for improved integration [1,2]. This study aimed to investigate sociodemographic factors influencing retention in care among immigrant PWH in Spain.

**Materials and methods**: This retrospective longitudinal study was conducted at the Hospital Clinic of Barcelona, including immigrant PWH who first consulted between 2010 and 2022 and with at least 1 year of follow‐up. Kaplan‐Meier survival curves estimated retention in care (RIC) and the log‐rank test was used to compare rates across variables. The variables analysed included treatment‐naïve versus treatment‐experienced, time in Spain at the initial visit, educational level, employment status, gender, and origin.

**Results**: Among 3494 migrants, 91% (*n *= 3168) were male. The median age was 34 (29–40) years; 59% (2053) were non‐naïve; 46% (*n =* 1183) moved to Spain during the previous year. Regarding employment, 29% (*n =* 982) were unemployed, and 26% (*n =* 877) were temporarily employed. Forty‐eight percent (*n =* 1572) had a university education (Table 1). RIC was 87% at 1 year (95% CI 86–88%), 66% at 5 years (95% CI 64–68%), and 51% at 12 years (95% CI 48–53%). Significant differences were found in RIC for treatment‐naïve versus treatment‐experienced (χ^2^ = 34.24, *p* < 0.0001), time in Spain (χ^2^ = 24.97, *p* < 0.0001), educational level (χ^2^ = 21.29, *p *< 0.0001), and employment status (χ^2^ = 35.20, *p *< 0.0001). Working individuals had the highest RIC (90% at 1 year, 70% at 5 years, and 55% at 12 years), while the unemployed had the lowest (85%, 60%, and 40%, respectively). University‐educated PWH (90% at 1 year, 75% at 5 years, 55% at 12 years) managed better than those with less education (80%, 58%, 40%, respectively). PWH in Spain for shorter periods had better RIC (85% at 1 year, 75% at 5 years, and 60% at 12 years) than those with longer periods (80%, 60%, and 45%, respectively). No significant differences were observed for gender (χ^2^ = 0.17, *p* = 0.6776) or region of origin (χ^2^ = 6.93, *p* = 0.0741) (Figure 1).

**P269: Table 1:** Socio‐demographic characteristics of the study population

Variable	Value
Sex	
Male	3168 (91%)
Female	326 (9%)
Total	3494 (100%)
Age	
Age at first visit, median (IQR)	34 (29‐40) [3494]
Treatment status	
Not naïve	2053 (59%)
Naive	1402 (41%)
Total	3455 (100%)
Years at Spain at first visit	
Less than 1 year	1183 (46%)
1–3 years	588 (23%)
More than 3 years	820 (32%)
Total	2591 (100%)
Employment status	
Working (permanent)	953 (29%)
Working (temporary)	877 (26%)
Unemployed	982 (29%)
Student	228 (7%)
Retired	20 (0%)
Housewife	9 (0%)
Working (unspecified)	261 (8%)
Total	3338 (100%)
Educational level	
University degree	1572 (48%)
High school degree	1408 (43%)
Primary education	279 (9%)
Total	3259 (100%)

**P269: Figure 1:**
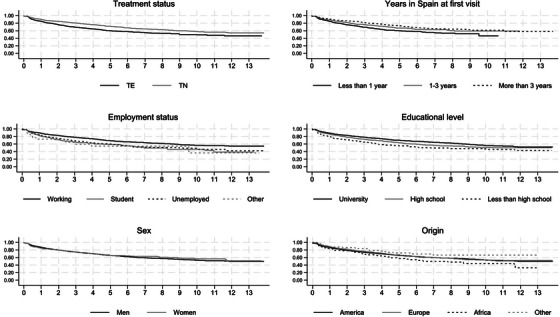
Kaplan‐Meier survival curves by socio‐demographic factors among immigrant persons with HIV at Hospital Clinic of Barcelona.

**Conclusions**: Retention rates dropped significantly, particularly within the first 5 years, with only 50% remaining after 12 years. Among immigrants, those with treatment experience had lower retention rates. However, immigrants with more extended residency, higher education, and employment demonstrated better retention in care. Gender and origin did not impact follow up

**References**

1. Fakoya I, Álvarez‐del Arco D, Woode‐Owusu M, Monge S, Rivero‐Montesdeoca Y, Delpech V, et al. A systematic review of post‐migration acquisition of HIV among migrants from countries with generalised HIV epidemics living in Europe: implications for effectively managing HIV prevention programmes and policy. BMC Public Health. 2015;15:561.

2. Carballo M, Hargreaves S, Gudumac I, Maclean EC. Evolving migrant crisis in Europe: implications for health systems. Lancet Glob Health. 2017;5(3):e252‐3.

### P270 Model of remote HIV linkage to care at the AIDS centre: utilising the EECA region as an example

Dmitrii Korenev ^1^, Kirill Barskiy^2^

^1^Steps, Moscow, Russian Federation. ^2^Regional Expert Group on Migration and Health, Moscow, Russian Federation

**Background**: According to the Russian's Law on Preventing the Spread of HIV, migrants who have been diagnosed with HIV should be deported. Many migrants are unable to return to their nation of origin due to socio‐economic factors. Furthermore, the majority of them stay in the host nation illegally after the disease is discovered. In Russia, as of 2021, there were 42,642 foreigners with HIV who were officially registered, the real figure could be much higher. Lack of access to healthcare services, including those funded under the Global Fund programme, will instigate the health deterioration of migrants living with HIV and contributes as a catalyst for the HIV epidemic in the Eastern Europe and Central Asia (EECA).

**Methods**: A novel approach of remote registration at the dispensary in the AIDS centres of the migrant countries of origin was introduced in the EECA area in 2021 by the Steps Foundation in collaboration with the Regional Expert Group on Migration and Health. The basic idea of the strategy is that migrants can get in touch with recognized NGOs in their country of destination, who can assist them in preparing the required paperwork for submission through the internet and connect them with physicians at the AIDS centre for ART receiving in their country of origin. Migrants are not required to travel back to their country to establish this mechanism.

**Results**: Moldovan migrants have been able to utilize this mechanism from 2021. Similar work protocols have been implemented since 2022 by the health ministries of Kyrgyzstan and Tajikistan. For 2023, Steps Foundation remotely registered 39 migrants from Tajikistan, Kyrgyzstan and Moldova. In 2024, this model of assistance was launched in Kazakhstan and Uzbekistan. The Fund has served as a reliable source for the point of ART delivery for Tajikistan and Armenia migrants since 2023.

**Conclusions**: This model is unique and shows high efficiency. Receiving ART from the country of origin is a sustainable healthcare delivery model for countries with restrictive migrant laws. Migrants with HIV show high adherence and reach undetectable loads, as shown in Figure 1. Additionally, it prevents epidemiological consequences for the entire region. Cross‐border collaboration is essential in preventing the spread of HIV.

**P270: Figure 1:**
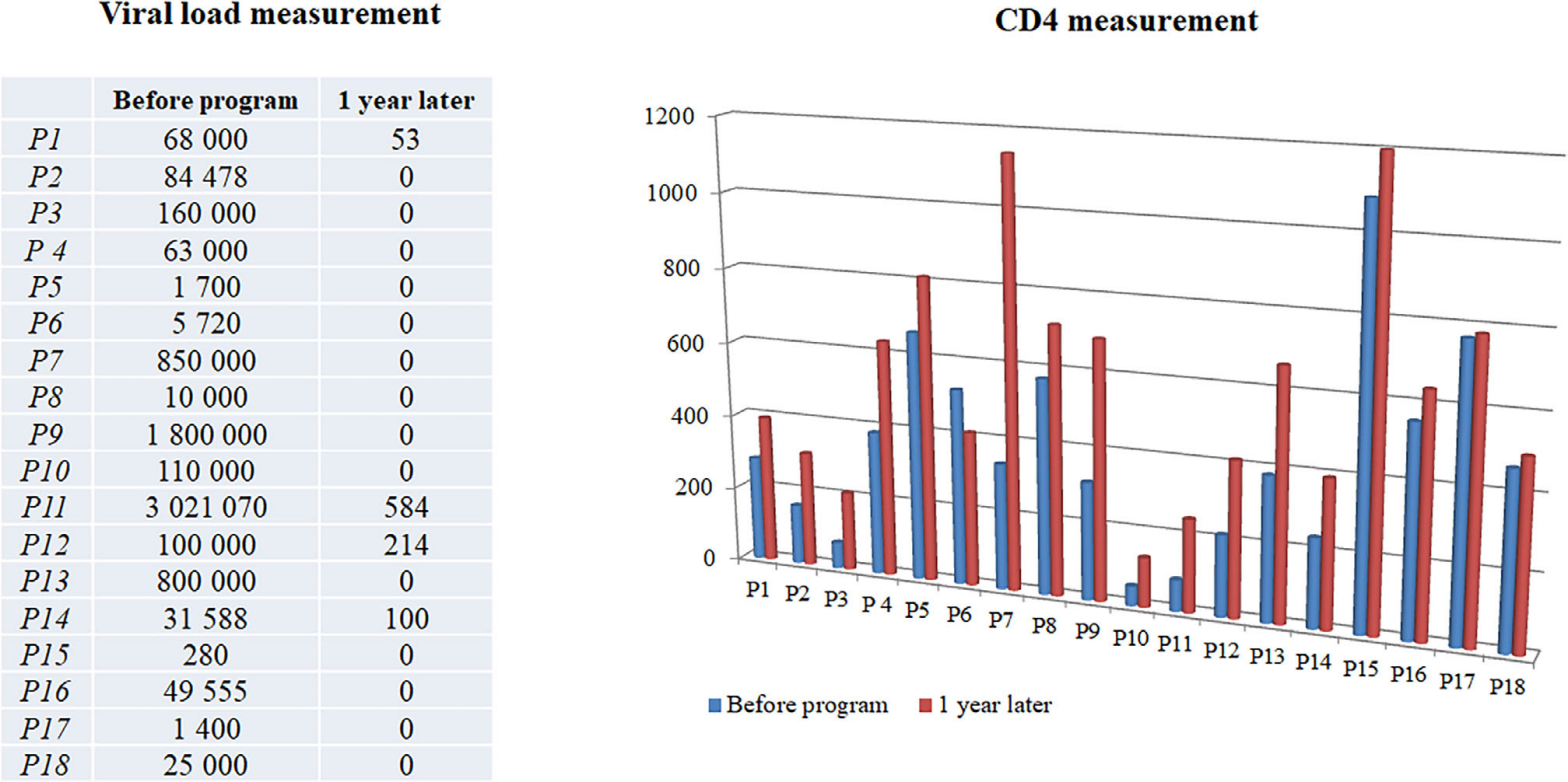
Measurement of viral load and CD4 indicators among migrants who were registered remotely in their countries of origin.

### P271 Free HIV drugs as a key tool against the HIV pandemic: a systematic review

Benoît Lemire ^1^, Melissa Doutre^1^, Marie‐Pier Godin^1^, Iliya Dmitriev^1^, Amy Bergeron^2^, Lucie Blais^3^, Ana Paula Bruno Pena Gralle^3^

^1^Pharmacy, University of Montreal & McGill University Health Centre, Montreal, Canada. ^2^Medicine & Immunology, University of Montreal, Montreal, Canada. ^3^Pharmacy, University of Montreal, Montreal, Canada

**Background**: Many studies have demonstrated the effectiveness of HIV treatment and prevention. Nevertheless, access to medication appears to be one of the major barriers to achieving the World Health Organziation (WHO)/Joint United Nations Programme on HIV/AIDS (UNAIDS) 95‐95‐95 strategy. No systematic review has evaluated the impact of providing free HIV medication. This review aims to assess the impact of free antiretroviral (ARV) drugs on aspects of the HIV cascade of care (being on therapy, viral suppression) and on pre‐exposure prophylaxis (PrEP) use.

**Materials and methods**: The following electronic databases were searched: Medline ALL via Ovid, Embase via Ovid, APA PsycInfo via Ovid, the Cumulated Index of Nursing and Allied Health Literature (CINAHL), Complete via EBSCOhost, the Web of Science Core Collection via Clarivate and grey literature. Two independent reviewers screened abstracts and full texts published between 1 January 1996 and 30 June 2024, for studies focusing on the impact of free access to antiretroviral drugs compared to those who paid a fee among people living with HIV or at high risk of acquiring HIV. The National Heart, Lung, and Blood Institute (NHLBI) Quality Assessment Tool was used to assess study quality (Prospero ID: CRD42024527274).

**Results**: We identified 19,532 documents and reviewed 374 full‐text manuscripts. Of the 24 included studies, seven studies were classified as “being on therapy” according to the outcome they measured; four studies were classified as “being virally suppressed” and two for “being on PrEP.” The remainder were classified based on their outcomes, with 29% assessed as “mortality” and 21% as “HIV transmission.” Among the studies included, most were conducted in the United States, followed by Australia and Mexico. Almost half of the studies included (48%) were conducted in high‐income countries. The absence of out‐of‐pocket costs for HIV drugs, compared to having out‐of‐pocket costs, has a positive impact on different aspects of the HIV care cascade and PrEP use.

**Conclusions**: Providing HIV drugs without any out‐of‐pocket costs has a positive impact on access to ARVs. Our results serve as a valuable reference for parts of the world that remain undecided about implementing a free ARV drug programme.

### P272 Changes in the ART coverage and viral suppression from 2020 to 2022 in Kyiv, Ukraine

Olga Fursa ^1^, Joanne Reekie^1^, Wendy Bannister^1^, Larysa Hetman^2^, Alina Kryshchuk^2^, Olena Starychenko^3^, Nana Hrytsaiuk^3^, Inna Khodus^4^, Alla Nyzhnyk^4^, Viktoriia Rakhuba^4^, Maryna Kovalevska^4^, Olena Valdenmaiier^1^, Jens Lundgren^1^, Lars Peters^1^

^1^Centre of Excellence for Health, Immunity and Infections, Rigshospitalet, Copenhagen, Denmark. ^2^Department for Coordination of HIV Diagnostic and Treatment Programmemes, Public Health Center of the Ministry of Health of Ukraine, Kyiv, Ukraine. ^3^Kyiv City AIDS Prevention and Control Center, Kyiv City Clinical Hospital No.5, Kyiv, Ukraine. ^4^Kyiv Regional Center for Public Health, Kyiv, Ukraine

**Background**: Ukraine has made vast progress in HIV management in recent years, but both the COVID pandemic and then the Russian invasion may have substantially impacted this.

**Methods**: We constructed a right‐hand side HIV cascade of care (CoC) for people living with HIV in the observational CARE cohort in Kyiv City and Kyiv Regional AIDS centres in Ukraine by calendar year (2020 to 2022). Among those under follow‐up (FU), we investigated the percentage who visited a clinic, were on ART, had CD4 cell count or HIV‐RNA measured, and who died each year. We further assessed the percentage on ART with viral suppression (HIV‐RNA ≤200 copies/ml) and the factors associated with being virally suppressed. We also investigated the proportion of treatment switches and post‐switch HIV‐RNA measurements.

**Results**: Among 1808 people under FU in 2020, the median age was 40 years, 63% were male, and 49% had injection drug use as HIV transmission mode. While the number of persons under FU decreased by 17% to 1504 in 2022, the distribution of gender and transmission modes did not change substantially, and the proportion of deaths was consistent across years (between 1% and 2%). The percentage of people on ART was >98% for all 3 years (Figure 1a). The proportion of ART patients who were virally suppressed increased from 75.9% in 2020 to 80.9% in 2021, then dropped to 64.8% in 2022, largely due to an increase in missing HIV‐RNA from 20.5% in 2020 to 31.7% in 2022 (Figure 1b). Injecting drug use, calendar year 2022, and enrolment in Kyiv Region were associated with higher odds of being unsuppressed. A large proportion of individuals switched ART in 2020 (46.8%) and 2021 (41.9%), the majority to tenofovir/lamivudine/dolutegravir, compared with 9.8% switching in 2022. Of those with treatment switch in 2020 and 2021, 34.9% and 35.6%, respectively, had missing HIV‐RNA measurements within the next 12 months.

**P272: Figure 1:**
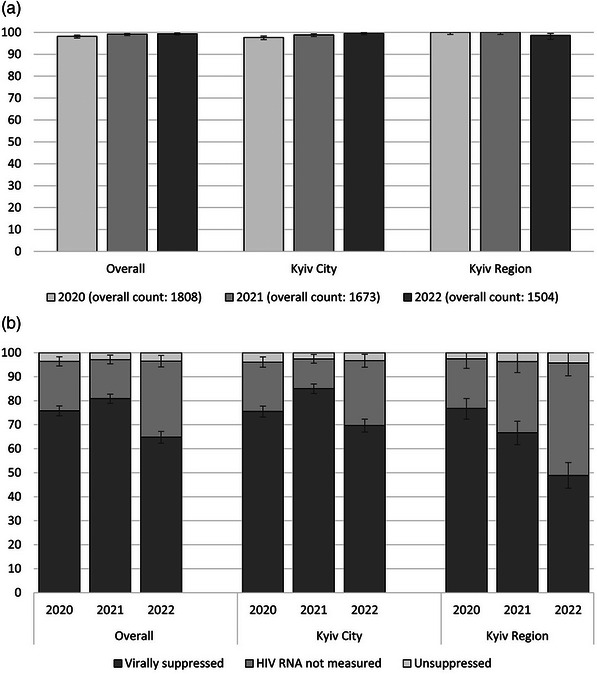
Right‐hand side of the HIV cascade of care from 2020 to 2022 in the CARE cohort in Ukraine overall and by clinical site. (a) Percentage of people on ART among those under follow‐up; (b) percentage of virologically suppressed and with missing viral load measurements among people on ART.

**Conclusions**: While the proportion of visits dropped during the first year of the war, ART coverage remained very high among those under follow‐up, and the decrease in the fraction of virally suppressed in 2022 was mostly due to HIV‐RNA tests not being performed.

### P273 The impact of migratory patterns in HIV profile: an analysis of genotype and resistance mutations of the migrant population in a tertiary hospital

Gonçalo Pereira Cruz, Joana Marinho Silva, Cristina Valente

Infectious Diseases, Unidade Local de Saúde de Coimbra, E.P.E., Coimbra, Portugal

**Background**: The number of new diagnoses of HIV infection in the EU/EEA has increased significantly in the past years, due to war, the pandemic and natural disasters. The patterns of migration of people living with HIV throughout Europe accounted for 16.6% of the diagnosis in 2022. Depending on the country of origin, the HIV genotyping and resistance mutations may vary and limit the therapeutical options in this population.

**Methods**: We conducted a retrospective study of all the migrants that were followed in our HIV clinic between 2021 and 2023. We retrieved data from their files, namely country of origin, current ART, TCD4+ count, HIV viral load, genotyping and resistance test.

**Results**: We evaluated 336 migrants in our HIV clinic, of which 142 had detectable viral load (>20 cp/ml); genotyping and resistance testing were performed only in 92 of these patients. The most common genotypes were unique recombinant forms (*n =* 28), genotype C (*n =* 20) and B (*N =* 19); genotype F1 (*n =* 9) and genotype A1 (*n =* 7) were also documented. Regarding resistance mutations, 2.68% had resistance to NRTI, 6.85% to NNRTI, 1.49% to PI and 3.5% to INSTI. Most of the patients did not have major mutations, 24 had only one class mutation, nine had two class mutations and three had more than three class mutations. The majority of the mutations were found in patients from Latin America and sub‐Saharan Africa (Table 1). Among the migrants with mutations, only one did not have an INSTI based regimen. At the last consultation, 18 patients still had detectable viral load. However, from these 18, four were lost to follow‐up and one returned to his country of origin.

**P273: Table 1:** Resistance mutation pattern according to zone of origin

Zone of origin	NRTI	NNRTI	PI	INSTI
Latin America	M184MV, S68N, M41ML, K65R, S68G, M184I, S68SG, T215S	E138A, K103N, A106VI, V106VIM, E138G, V179E, V106VEIKL,V108VI, V118I, M230MI, E138EA, M230ML, L234LI	F53FL, Q58QEK, Q58E, N88ND, M46L, V82T, L90M, V82F	Q95K, Q146QK, D232DN, L74LIM, G18GRS, T66TS, S147SR, Q148QK, N155NK
Africa	K70N, M184V, M41L, E44ED, L74I, L210W, T215Y, Del67, T69G, K219E	K103N, V106I, V108VI, V106VI, E138G, G190AS, Y318YF, E138K, V79VD, H221Y, L100I, K103N, V106VI, E138A, M230L		D232DN, Y143YC, S147SG, Q146QK, G140GD, Q146QE
Asia		K103N		

**Conclusions**: The prevalence of transmitted drug resistance in newly diagnosed HIV cases globally ranges from 10% to 17%, but in migrants the resistance rates are slightly higher, ranging between 15% and 20%, with some studies reporting values as high as 44%. In our study, the rate of resistance is closer to the global prevalence rather than the migrant. It is important to understand the continuous evolution of this population to adjust our prescription according to resistance patterns.

### P274 BIC/TAF/FTC in treatment‐naive PLWH: the experience of a centre

Constança Antunes ^1^, Sofia Rocha^1^, Andreia Meseiro^2^, Margarida Almeida^1^, Marta Almeida^1^, Dina Mendes^3^, Ana Cláudia Miranda^1^, Teresa Baptista^1^, Kamal Mansinho^1^

^1^Serviço de Doenças Infeciosas e Medicina Tropical, Unidade Local de Saúde Lisboa Ocidental, Hospital Egas Moniz, Lisbon, Portugal. ^2^Serviço de Medicina Interna, Unidade Local de Saúde do Arco Ribeirinho, Centro Hospitalar Barreiro Montijo, Barreiro, Portugal. ^3^Serviços Farmacêuticos, Unidade Local de Saúde Lisboa Ocidental, Hospital Egas Moniz, Lisbon, Portugal

**Background**: BIC/TAF/FTC is a first‐line antiretroviral therapy (ARV) recommended in European and American guidelines, available for use in Portugal since July 2019. This report aims to characterize the population of treatment‐naive PLWH that started BIC/TAF/FTC in our centre, from its introduction to December 2023, to include at least 6 months of follow‐up.

**Materials and methods**: Demographic, epidemiological, clinical, immunological, and virological data was compiled and analysed, from a total of 858 PLWH on BIC/TAF/FTC with regular follow‐up in Egas Moniz Hospital: 708 were treatment‐experienced and 150 were treatment‐naive, and the following results relate to the second group.

**Results**: This population of 150 treatment‐naive PLWH had a predominance of males (76%), with a median age of 38 years old, and an important component of migrant patients, with 38% of Portuguese PLWH and 52% from Portuguese‐speaking countries around the world. The most common form of HIV transmission reported was sexual (97.3%), with a low prevalence of coinfection, HBV in 6% of patients, and HCV in 3.3%. The most common comorbidities were cardiovascular risk factors in 19.3% of patients. BIC/TAF/FTC was started mainly in patients in stage A of the CDC classification (80%), with 11 patients in stage C (7.3%). Thirty‐one percent of patients started ARV in a *test and treat* regimen, meaning in the first appointment. As for immunological status, there was an improvement in CD4+ cell counts with an average of 426 cells/ml at diagnosis and 725 cells/ml at the most recent evaluation, and a rise in percentage from 21 to 28%, with an average of 21 months of treatment. As for virological suppression, despite 75 patients with viral load above 100,000 copies/ml (50%) at diagnosis, 98.7% achieved virological control. Of the 150 PLWH in this group, 5.3% were lost to follow‐up, and 6% discontinued therapy with BIC/TAF/FTC mostly due to intolerance.

**Conclusions**: BIC/TAF/FTC is a highly effective therapy in immunological improvement and virological suppression, recommended for treatment‐naive PLWH. The data presented above is aligned with the current available literature, reinforcing the use of BIC/TAF/FTC as a reliable treatment option, and supporting its application in clinical practice.

### P275 Successful and practical model of care targeting disengaged people living with HIV: the Oak Clinic experience at Russells Hall Hospital in Dudley, United Kingdom

Adel Shoukry

Oak Clinic, Russells Hall Hospital, Dudley, UK

**Background**: The BHIVA National Audit 2023 “Engagement in HIV Care” [1] describes patient characteristics, with key conclusions identifying that most patients who disengaged had missed appointments in the year before their last attendance, with 35% missing two or more appointments. In November 2023, we continued our services as a stand‐alone HIV service at Oak Clinic, Russells Hall Hospital, Dudley. This was an opportunity to implement new developments and improvements aimed at better, safer and more up‐to‐date patient care. BHIVA audit recommendations were taken on board, and changes in the way of service delivery, introducing new pathways and tools for assessments, a Did Not Attend (DNA) pathway and personalized interactions were introduced.

**Methods**: The clinic template was updated to include afternoon and evening HIV clinics. Clinical interactions were increased by implementing more holistic, patient centred, personalized care by introducing more clinics such as results clinics, lifestyle clinics, medications and adherence clinics supported by trained HIV CNS, and phlebotomy and observation and screening clinics supported by clinical support workers. These clinics incorporated the use of screening tools such as cognitive assessment, depression and anxiety, smoking and alcohol assessment, QRISK3, FRAX and Frailty assessments together with pathways and signposting. A new DNA pathway was introduced in November 2023 (Table 1).

**P275: Table 1:** Oak Clinic policy for DNA and engagement in HIV care

Event	Action
Patient does not attend (DNA) or does not answer call	Receptionist to text (agreed text)
Non respondent to text in 7–14 days	Booked for telephone call
If not answering telephone call	Voice mail message (if consented)
Non respondent in 14 days	Third contact by phone or text, over 1 month from DNA appointment
Non respondent to third contact	Case to be discussed at the local MDT and a case‐by‐case decision for further contact is made
Non respondent to MDT's agreed action	Notes to be filed and patient booked for local MDT in 14 months
If the patient contacts the clinic at any time	A discussion exploring reasons for earlier disengagement, offering support and agreeing a mutual action plan
If a patient does not contact the clinic for 14 months	Notes discussed at local MDT followed by a disengagement letter to the GP

**Results**: From Figure 1, the HIV attendances average for months April–October 2023 is 78 (range 63–102) compared to an average of 400 attendances for months November 2023–April 2024 (range 267–603). This five‐fold increase in attendances is mostly attributed to an increase in clinical interactions. The average DNA % for the months April–October 2023 is 8.5%. Despite an increase in attendances in November and December 2023, the DNA % average was reduced to 6.8%. The benefit of implementing the agreed changes was more apparent in the following months, January–April 2024, when attendances were increasing, yet the DNA % was decreasing with an average of 3.4%.

**P275: Figure 1:**
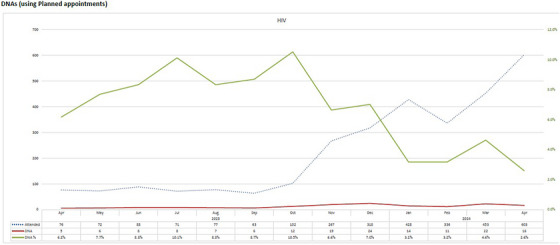
Clinic attendances in hundreds (left axis) and Did Not Attend (DNA) % (right axis) against months April 2023–April 2024. A solid line represents DNA % and a dotted line represents clinic attendances.

**Conclusions**: While most studies have monitored and looked at causes of disengagement, this abstract demonstrates a practical way forward to improve disengagement by reducing DNA %, while increasing clinical interactions and improving patient care and satisfaction.

**Reference**

1. Windebank F. BHIVA Audit 2023: Engagement in HIV care and impact on HIV inpatient admissions. BHIVA Autumn Conference; 2023 Nov 24; London, UK.

### P276 A patient survey to explore why patients ‘Did Not Attend’ in a large inner‐city HIV service

Wai Lin Htun, Anna Garner

The Northern Integrated Contraception, Sexual Health & HIV Service, Manchester University NHS Foundation Trust, Manchester, UK

**Background**: “Did Not Attends” (DNAs) are multifaceted and impact both patient care and healthcare resources. Recent data from the clinic has shown a high DNA rate among our clinic cohort ∼20%. We conducted this survey to explore the reasons for having DNAs and factors to improve patient attendance.

**Methods**: An anonymized survey was texted to the patients who DNA their appointments between 01/03/24 and 31/05/24 across five HIV clinics. The results were collected via Microsoft Form and reviewed.

**Results**: During the review period, 2938 appointments were booked with an overall DNA rate of 27.8% (818/2938). The survey was sent to 594 patients after applying exclusion criteria, and 93% (553/594) texts were delivered. Ninety patients responded to the survey, resulting in a 16% response rate. Twenty‐nine percent (26/90) were signed up to the Personal Health Record (PHR) where they can check their appointments, however, 62% (56/90) were not aware of PHR. Fifty‐two percent (47/90) contacted the clinic to cancel/amend their appointment. The most common reasons for not cancelling the appointment were forgetting the appointment 40% (17/43) and failing to contact the clinic 20% (9/43). The most common reasons for missing appointments were forgetting the appointment, family or work commitments, feeling unwell and the appointment time being inconvenient. Twenty‐seven percent (24/90) were feeling unwell due to different medical and mental health conditions on their appointment date. Eighty percent (72/90) confirmed they received a reminder text before the appointment. To avoid missing appointments in the future, 27% (39/146) preferred to use reminder text, but 26% (38/146) preferred to cancel appointments via online and 21% (31/146) preferred to cancel their appointment via text message. Thirty‐seven percent (34/90) mentioned that they preferred to self‐manage their appointment time to avoid missing it.

**Conclusions**: The survey demonstrates that patients prefer to manage their appointments according to their work/life/health conditions. A user‐friendly digital platform for appointment management is imperative to improve DNA rates in the future, while ensuring we continue to meet the needs of more vulnerable patients, and in whom English is not their first language. A further review of DNAs is planned once improvements in PHR have been deployed.

## Co‐morbidities and complications of disease and/or treatment—Ageing and frailty

### P277 Comparison of four frailty scores to predict adverse health outcomes and mortality in people living with HIV aged 70 years and more (ANRS EP66 SEPTAVIH study)

Clotilde Allavena ^1^, Diane Abulizi^2^, Hubert Blain^3^, Valérie Adriantsoanirina^2^, Sophie Abgrall^4^, Marina Karmochkine^5^, Christine Katlama^6^, Miresta Previlon^7^, André Cabie^8^, Fabrice Bonnet^9^, Laurence Meyer^10^, Alain Makinson^11^

^1^Infectious Diseases Department, INSERM EA1413, Nantes University Hospital, Nantes, France. ^2^INSERM CESP U1018, Paris‐Saclay University, Le Kremlin‐Bicêtre, France. ^3^Geriatrics Department, Montpellier University Hospital, Montpellier, France. ^4^Internal Medicine Department, Hop Antoine Béclère AP‐HP, Clamart, France. ^5^Infectiology, Hôpital Hôtel Dieu AP‐HP, Paris, France. ^6^Infectious Diseases Department, Hôpital Pitié‐Salpétrière AP‐HP, Paris, France. ^7^Infectious Diseases Department, Hôpital Saint Louis AP‐HP, Paris, France. ^8^Infectious Diseases Department, Martinique University Hospital, Fort de France, France. ^9^Infectious Diseases Department, Bordeaux University Hospital, Bordeaux, France. ^10^Public Health Department, INSERM CESP U1018, Bicêtre University Hospital AP‐HP, Paris‐Saclay University, Le Kremlin‐Bicêtre, France. ^11^Infectious Diseases, Montpellier University Hospital, Montpellier, France

**Background**: Frailty is associated with adverse health outcomes in older persons. Studies have evaluated frailty in middle‐aged PLWH. Phenotypic frailty standard evaluation uses the five‐item FRIED criteria (exhaustion, weakness, slow walking speed, weight loss and low physical activity), which has been correlated in the general and HIV population with adverse health outcomes. Other screening tools of frailty exist: the FRAIL scale (recommended in the last EACS guidelines), the Study of Osteoporotic Fractures frailty index (SOF) and the French Authority of Health questionnaire (HAS). We evaluated the association of these scores with adverse health outcomes or mortality over 36 months in geriatric PLWH on ART.

**Materials and methods**: Our study was nested in the French multicentre prospective ANRS EP66 SEPTAVIH study, aimed to assess frailty prevalence and factors in PLWH 70+ taking ART for at least 12 months. At baseline, frailty was assessed using the FRIED index score and FRAIL, SOF and HAS screening tools. Adverse health outcomes of falls, hospitalization, emergency room visit, nursing home placement, disability, and mortality were collected at 12, 24 and 36 months. We used log‐binomial regression to analyse the association between frailty and adverse health outcomes or death and the Cox proportional hazard model for association between frailty and mortality.

**Results**: Five hundred and ten PLWH, mostly male (81.4%), with a median age of 73 years, a median HIV infection duration of 22.7 years were included; 13% were classified as frail using FRIED, and 9% with FRAIL, 7% with SOF and 26% with HAS. During the 36‐month follow‐up, 40 participants (7.9%) died and 254 participants (50%) had at least one adverse health outcome. The risk of adverse health outcomes or death over 36 months was higher when PLWH were classified as frail using FRIED, FRAIL and HAS but not SOF (Table 1). Frailty was strongly associated with mortality when assessed with FRIED (hazard ratio (HR) 5.37 [95% CI 1.65–17.47], *p =* 0.005), FRAIL (HR 4.54 [95% CI 1.33–15.53], *p =* 0.02), and HAS (HR 5.0 [95% CI 2.65–9.41], *p* < 0.001) but not SOF (HR 2.84 [95% CI 0.83–9.72], *p =* 0.10).

**P277: Table 1:** Association of frailty status assessed with FRIED phenotype, FRAIL score, SOF index and HAS questionnaire, and adverse health outcomes or mortality over 36 months

	Log‐binomial regression	
Frailty score at baseline	Relative risk [95% CI]	*p*‐value
FRIED		
Robustness (*n =* 84)	1.0 [reference]	
Prefrailty (*n =* 211)	1.01 [0.85*–*1.20]	0.89
Frailty (*n =* 39)	1.25 [1.02*–*1.52]	0.03
FRAIL		
Robustness (*n =* 166)	1.0 [reference]	
Prefrailty (*n =* 116)	1.30 [1.11*–*1.52]	0.001
Frailty (*n =* 25)	1.66 [1.47*–*1.88]	<0.001
SOF		
Robustness (*n =* 97)	1.0 [reference]	
Prefrailty (*n =* 182)	1.10 [0.93*–*1.31]	0.28
Frailty (*n =* 20)	1.23 [0.95*–*1.60]	0.12
HAS		
No frailty (*n =* 261)	1.0 [reference]	
Frailty (*n =* 94)	1.41 [1.26*–*1.59]	<0.001

**Conclusion**: FRIED, FRAIL and HAS tools, but not SOF strongly predicted adverse health outcomes or mortality in a geriatric population with HIV.

### P278 Acceptability and feasibility of digital assessment of falls risk, frailty and mobility impairment using wearable sensors in PLWH as part of HIV care

Claire Norcross ^1^, Inayat Khan^2^, Tom Levett^3^, Birgit Barbini^4^, Lucy Campbell^5^, Leigh McQueen^4^, Elizabeth Hamlyn^4^, Jaime Vera^1^

^1^Global Health & Infection, Brighton & Sussex Medical School, Brighton, UK. ^2^Sexual Health & HIV, University Hospitals Sussex NHS Foundation Trust, Brighton, UK. ^3^Department of Clinical and Experimental Medicine, Brighton & Sussex Medical School, Brighton, UK. ^4^Sexual Health & HIV, King's College Hospital NHS Foundation Trust, London, UK. ^5^Department of Inflammation Biology, King's College London, London, UK

**Background**: The European AIDS Clinical Society guidelines recommend screening for frailty and falls in people living with HIV (PLWH) over 50 to enable early identification and intervention. Digital assessment tools (DAT) such as the Quantitative Timed Up and Go (QTUG™) assessment tool, allow wearable sensors and questionnaires to generate standardized mobility impairment, frailty estimate (FE), and falls risk estimate (FRE) scores, offering significant potential for enhancing frailty and falls risk identification and monitoring in both clinical and community settings for PLWH. This study aimed to assess the acceptability and feasibility of incorporating DAT into routine HIV outpatient care.

**Materials and methods**: PLWH ≥60 attending routine outpatient care from two HIV clinics in Southern England between December 2022 and July 2023 were invited to participate. Baseline demographic data and DAT output data were collected, and individuals identified as frail and/or at risk of falls were offered intervention accordingly. Acceptability was assessed using semi‐structured interviews with participants, while healthcare professionals involved in screening and care of older PLWH were invited to take part in a focus group, to explore attitudes towards frailty screening, and the use of DAT. Interviews and focus groups were analysed using thematic analysis.

**Results**: Fifty participants were recruited across two sites, with baseline demographics and frailty scores illustrated in Table 1. Participants found the DAT acceptable and appreciated its objectivity and thoroughness. Healthcare professionals agreed frailty screening could be valuable where clearly defined pathways for the management of pre‐frailty and frailty exist, but felt the DAT may be better suited to ongoing monitoring of falls risk and mobility impairment, citing test duration and technical challenges as barriers to implementation.

**P278: Table 1:** Participant demographic data and frailty scores

Factor	Total
	50 (100%)
Demographic factors	
Age	64.5 [62–68]
Gender (self‐identified)	
Male	32 (64%)
Female	17 (34%)
Non‐binary	1 (2%)
Country of birth^a^	
UK	26 (52%)
Africa	18 (36%)
Europe	2 (4%)
Other	3 (6%)
Sexual orientation	
Gay/lesbian	23 (46%)
Heterosexual	25 (50%)
Other	2 (4%)
HIV‐associated factors	
Time since HIV diagnosis	21 [16–31]
Time on ART	17.5 [13–24]
VL <50 c/ml	47 (94%)
Frailty‐associated factors	
≥5 comorbidities	12 (24%)
Polypharmacy (5 concurrent medications)	19 (38%)
≥1 falls in the past 12 months^b^	15 (31%)
Frailty and falls risk scores	
FRAIL scale score	
Robust	23 (46%)
Pre‐frail	15 (30%)
Frail	12 (24%)
DAT falls risk estimate^b^	
Low risk	30 (67%)
Medium risk	6 (13%)
High risk	9 (20%)
DAT frailty estimate^b^	
Non‐frail	23 (51%)
Transitional	11 (24%)
Frail	11 (24%)

*Note*: Categorical variables are shown as *N* (%), continuous variables as median [IQR].

^a^ Country of birth was missing for one participant.

^b^ It was not possible to obtain FRE and FE scores on five participants due to technical challenges.

**Conclusions**: Using the DAT to screen for falls and frailty as part of routine HIV care is highly acceptable to PLWH but there are perceived barriers to feasibility among healthcare professionals. The DAT identified a high number of individuals aged 60 or over as being at increased risk of falls and pre‐frail or frail, despite the sample's relatively young age, reinforcing the concept that incorporating frailty and mobility impairment screening into routine HIV care is important for the provision of comprehensive HIV care.

### P279 Antiretroviral therapy, comorbidities and concomitant medication in people with HIV aged over 75 years in one centre in London, UK

Maithili Varadarajan, Kareemah Kannike, Ankita Sahni, Yaser Al‐ Shakarchi, Nadia Naous, Modest Nwanbila, Nicolo Girometti, Timothy Tong, Marta Boffito

Genitourinary Medicine, Chelsea and Westminster Hospital, London, UK

**Background**: People with HIV are at risk of developing premature multimorbidity and ageing associated complications. De‐prescribing where polypharmacy is problematic, and antiretroviral therapy (ART) optimization taking into consideration health status, comorbidities, polypharmacy, pill burden and swallowing difficulties are key in this population.

**Materials and methods**: All prescriptions of people with HIV≥75 years old attending Chelsea and Westminster Hospital were reviewed by the HIV/geriatric multidisciplinary team (MDT). Clinical and demographic characteristics, including comorbidities, current and previous ART (2 years), documented Rockwood clinical frailty scores (CFS) were collected and descriptive statistics calculated.

**Results**: Two hundred and twenty‐four individuals were reviewed with a median age of 77 years (IQR 76–80), 195 (87%) were male and 166 (74%) were white, nine (43%) had a detectable viral load but <200 copies/ml. The median duration of time living with HIV was 24 years (IQR 16–31). Fifty‐one different ART regimens were prescribed in the 2 years preceding the review. Sixty‐one (27%) individuals underwent ART modernization between June 2022 and June 2024 with a reduction in median number of pills from 2 to 1. Thirteen percent switched to an integrase inhibitor (INSTI) containing regimen (Table 1), with the percentage of individuals on TAF/FTC/BIC doubling and on 3TC/DTG tripling. One hundred and nine individuals had a documented frailty score with the median of 3 (IQR 2–4) and a median number of 3 comorbidities (IQR 2–4). Detailed medication histories were available for 144 individuals of which 96 (66%) were on statins, 18 (13%) on other lipid lowering agents, 46 (32%) on antiplatelets and 23 (16%) on anticoagulants. Pharmacist input was essential to identify potentially inappropriate prescriptions and potential prescription omissions.

**P279: Table 1:** ART modernization in people with HIV older than 75 years between 2022 and 2024

ART regimen	Number of individuals on this regimen in 2024 (%)	Number of individuals on this regimen in 2023 (%)	Number of individuals on this regimen in 2022 (%)
Protease inhibitor (PI) containing	15 (7)	18 (8)	28 (13)
PI sparing	209 (93)	206 (93)	196 (90)
TDF sparing regimen	147 (66)	140 (63)	132 (55)
INSTI based	146 (65)	127 (57)	113 (52)
Newer NNRTI	38 (17)	39 (18)	28 (13)
Older NNRTI	29 (13)	37 (17)	57 (26)
TAF FTC BIC	84 (38)	57 (26)	51 (19)
3TC DTG	34 (15)	23 (10)	10 (5)

**Conclusions**: The majority of people with HIV over 75 years of age remain active (CFS ≤3) with a median of 3 comorbidities. However, as polypharmacy is common, active ART modernization and de‐prescribing is essential to reduce pill burden/improve adherence, drug interactions and unwanted effects. Therefore, the frailty/ageing clinic MDT should include pharmacists and focus on active drug optimization to improve the outcomes for people ageing with HIV.

### P280 The virtual care experience for persons living with HIV during the COVID pandemic

Sharon Walmsley ^1^, Marina Klein^2^, Majid Nabipoor^3^, Valerie Martel‐Laferriere^4^, Mona Loutfy^5^, Curtis Cooper^6^, Marie‐Louise Vachon^7^, Bryan Boyachuk^1^, Pamela Aldebes^8^

^1^Infectious Diseases, University Health Network, Toronto, Canada. ^2^Infectious Diseases and Immunity in Global Health, McGill University Health Centre, Montreal, Canada. ^3^Biostatistics Research Unit (BRU), University Health Network, Toronto, Canada. ^4^Department of Microbiology, Infectiology and Immunology, Centre Hospitalier de l'Université de Montréal, Montreal, Canada. ^5^Women and HIV Research Program, Women's College Hospital Research and Innovation Institute, Toronto, Canada. ^6^Infectious Diseases, Ottawa Hospital, Ottawa, Canada. ^7^Department of Microbiology‐Infectiology and Immunology, CHU de Quebec Université Laval, Quebec, Canada. ^8^Chronic Viral Illness Service, The Research Institute of the McGill University Health Centre, Montreal, Canada

**Background**: The COVID pandemic required a shift to virtual care. We hypothesized that there may be challenges for persons living with HIV with comorbidity, co‐infection or mental health concerns.

**Objective**: To describe the virtual care experience of a subset of participants in two established Canadian Trials Network (CTN) cohorts: CTN 314: CHANGEHIV‐persons >65 years of age, CTN 222 (HIV/HCV co‐infection).

**Methods**: Consenting participants invited at their cohort visit completed questionnaires reflecting their experience and satisfaction with virtual care.

**Results**: Four hundred and fifty‐four participants completed the baseline questionnaire between February 2021 and March 2023, 133 from CTN 314 and 321 from CTN 222. Overall, 55.3% had participated in virtual care; more in the older cohort 86.5% vs. 42% in the HCV cohort, *p* < 0.001, and most commonly with their primary care provider. The main reasons for not participating were: not offered 58%, did not have a phone or computer 17%, didn't know how to access 5% and only wanted to see their care provider in person 20%. Overall, 9% were satisfied, 36% somewhat and 55% were not satisfied with the virtual care. Twenty‐one percent felt virtual care was better than in person visits. Sixty‐four percent and 46% in the older and HCV cohorts, respectively (*p* = 0.004) missed the human contact of in person visits. Despite these concerns, 80% and 85% of the older and HCV cohorts felt that most/all of their concerns were addressed at their virtual visit. Three hundred and sixty‐nine (81%) participants had their HIV blood work done as often as prior to the pandemic. Of those who did not, the main reasons included: not feeling safe leaving their house (17%), doctor told to hold off on blood tests (27%), were not sure if it was still possible (48%) and did not want to do them (21%). Ninety‐eight percent were able to refill their HIV medications as usual.

**Conclusion**: There were a number of difficulties reported in older persons with HIV and those co‐infected with HCV accessing virtual care during the COVID pandemic. Although their needs were addressed, the majority missed human contact and were not satisfied with virtual care.

### P281 Association between low skeletal muscle mass and lung function decline in people with HIV

Katrine Munk ^1^, Moisés Alberto Suarez‐Zdunek^1^, Rikke Krabek^2^, Louise Bering^1^, Casper Simonsen^2^, Klaus Fuglsang Kofoed^3^, Andreas Fuchs^3^, Lars Valeur Køber^3^, Thomas Benfield^4^, Sisse Rye Ostrowski^5^, Andreas Dehlbæk Knudsen^1^, Susanne Dam Nielsen^1^

^1^Department of Infectious Diseases, Copenhagen University Hospital—Rigshospitalet, Copenhagen, Denmark. ^2^Centre for Physical Activity Research, Copenhagen University Hospital—Rigshospitalet, Copenhagen, Denmark. ^3^Department of Cardiology, Copenhagen University Hospital—Rigshospitalet, Copenhagen, Denmark. ^4^Department of Infectious Diseases, Copenhagen University Hospital—Amager and Hvidovre, Copenhagen, Denmark. ^5^Department of Clinical Immunology, Copenhagen University Hospital—Rigshospitalet, Copenhagen, Denmark

**Background**: Low muscle mass and chronic lung disease are prevalent among people with HIV (PWH). In the general population, lower muscle mass is associated with a faster decline in lung function, but this association has not been investigated in PWH. We aimed to determine the prevalence of low muscle mass, factors associated with low muscle mass and the association between low muscle mass and lung function decline in PWH.

**Materials and methods**: We included participants from the prospective Copenhagen Comorbidity in HIV infection (COCOMO) study. Low muscle mass was assessed with CT at baseline and defined as skeletal muscle mass index below the lowest 5% of a healthy population. Lung function was measured as forced expiratory volume in 1 second (FEV_1_) at baseline and 2‐year follow‐up. We used linear and logistic regression to investigate potential associations. Using linear mixed models, we investigated if low muscle mass was associated with a faster FEV_1_ decline.

**Results**: We included 509 PWH, and 82 (16%) had low muscle mass (Table 1). Older age, female sex and lower BMI were associated with lower muscle mass, while being very active was associated with increased muscle mass. High concentrations of interleukin 6 and tumour necrosis factor‐α were associated with higher odds of low muscle mass (Figure 1). The rate of lung function decline was not significantly different between PWH with low muscle mass and PWH without low muscle mass (35.9 ml/year vs. 34.0 ml/year, *p* = 0.69).

**P281: Table 1:** Baseline characteristics of PWH with and without low muscle mass at baseline

	PWH with low muscle mass (*N* = 82)	PWH without low muscle mass (*N* = 427)
Age (years), mean (SD)	53.4 (14.0)	51.1 (10.1)
Male sex, *n* (%)	80 (97.6)	392 (91.8)
BMI (kg/m^2^), mean (SD)	22.2 (3.2)	25 (2.9)
Cumulative smoking (pack‐years), median (IQR)	22 (7.5–35.3)	18 (6.0–30.0)
Physical activity		
Inactive‐slightly, *n* (%)	40 (48.9)	157 (36.8)
Moderately‐very active, *n* (%)	39 (47.6)	260 (60.9)
Airflow limitation, *n* (%)	17 (20.7)	50 (11.7)
IL‐6 (pg/ml), median (IQR)	3.8 (2.7–5.3)	3.1 (2.0–4.2)
TNF‐α (pg/ml), median (IQR)	7.7 (5.9–9.8)	7.1 (5.8–8.7)
Use of ART at baseline, *n* (%)	82 (100)	426 (99.8)
Nadir CD4+ count <200 cells/µl, *n* (%)	32 (39.0)	177 (41.5)
Current CD4+ count <350 cells/µl, *n* (%)	9 (11.0)	25 (5.9)
History of AIDS, *n* (%)	11 (13.4)	67 (15.7)

Inactive‐slightly active: complete inactivity or light activity ≤4 hours per week. Moderately‐very active: light activity >4 hours per week or strenuous activity ≥2 hours per week or regular intense training. Airflow limitation: FEV_1_/FVC ≤0.70.

Abbreviations: ART, antiretroviral therapy; BMI, body mass index; FEV_1,_ forced expiratory volume in 1s; FVC, forced vital capacity; IL‐6, interleukin 6; IQR, interquartile range; PWH, people with HIV; SD, standard deviation; TNF‐α, tumour necrosis factor alpha.

**P281: Figure 1:**
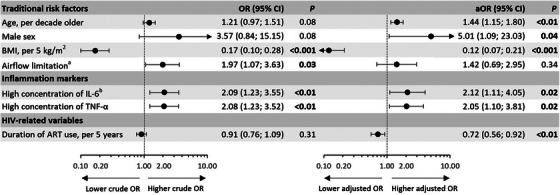
Variables significantly associated with low muscle mass. ^a^FEV_1_/FVC ≤0.70; ^b^high concentrations of inflammatory markers were defined as values above the 75th percentile. *P*‐values <0.05 are shown in bold. aOR, odds ratio adjusted for age, sex, smoking status, BMI, and physical activity level; ART, antiretroviral therapy; BMI, body mass index; FEV_1_, forced expiratory volume in 1s; FVC, forced vital capacity; IL‐6, interleukin 6; OR, odds ratio; TNF‐α, tumour necrosis factor alpha.

**Conclusions**: We found a high prevalence of low muscle mass in PWH. Traditional risk factors and inflammatory markers were associated with low muscle mass, whereas being very active was associated with greater muscle mass, emphasising the importance of physical activity in PWH. We did not find evidence of an association between low muscle mass and a faster decline in FEV_1_ among PWH.

### P282 Evaluation of T‐cell immunosenescence markers in virologically suppressed people living with HIV aged over 60 years on BIC/FTC/TAF or DTG/3TC: the Collateral study

Matteo Vassallo ^1^, Michel Ticchioni^2^, David Chirio^3^, Ami Sadou^2^, Eric Cua^3^, Carole Ceppi^3^, Sara Ferrando^3^, Alissya Naqvi^3^, Bernard Prouvost‐Keller^3^, Sommani Mangin‐Ratana^3^, Leslie Ameil^3^, Maeva Godemert^4^, Michel Carles^3^, Jacques Durant^3^

^1^Infectious Diseases, Centre Hospitalier de Cannes, Cannes, France. ^2^Immunology, Centre Hospitalier de Nice, Nice, France. ^3^Infectious Diseases, Centre Hospitalier de Nice, Nice, France. ^4^Clinical Research, Centre Hospitalier de Nice, Nice, France

**Background**: Despite successful antiretroviral therapies (ART), people living with HIV (PLWH) continue to experience higher comorbidities than uninfected subjects, suggesting premature ageing of their immune system, often referred to as immunosenescence. A critical unanswered question is whether two‐drug regimens (2DR) and three‐drug regimens (3DR) have different effects on immunosenescence, especially in older subjects with a low CD4 nadir. Collateral is an ongoing cohort study evaluating cellular and plasma inflammatory markers and the onset of comorbidities in PLWH on BIC/FTC/TAF or DTG/3TC. We present an interim analysis evaluating markers of T cell immunosenescence in participants over 60 years old.

**Materials and methods**: Collateral is a multicentre ongoing French prospective cohort study. Inclusion criteria were participants over 40 years of age, >10 years on ART, having HIV‐1 RNA <50 c/ml and taking either BIC/FTC/TAF or DTG/3TC QD for at least 6 months. HBV or HCV co‐infections and archived resistance to DTG, BIC or TAF were excluded. We analyzed inflammatory markers in CD4+ and CD8+ naïve (N), central memory (CM), effector memory (EM), terminally differentiated effector memory (TEMRA) and senescent (SEN‐KLRG1+ 57+) T‐cells in participants aged over 60 years. HIV‐negative participants were included as healthy controls.

**Results**: We included 49 PLWH (BIC/FTC/TAF = 23; DTG/3TC = 26) and five HIV‐negative participants. Forty‐nine percent (24/49) had a CD4 nadir <200/mm^3^. PLWH had higher levels of immunosenescence markers than HIV‐negative participants. Among PLWH with a CD4 nadir count <200 cells/mm^3^, despite similar demographic and background parameters, we found significantly lower levels of senescent and TEMRA T cells in the BIC/FTC/TAF group (Table 1).

**P282: Table 1:** Characteristics of patients included and immunosenescence markers

	HIV‐negative (mean ± SD)	DTG/3TC (mean ± SD)	BIC/FTC/TAF (mean ± SD)	*p*‐value 2DR vs 3DR
Characteristics				
Age	66.4 ± 2.07	70 ± 6.7	69 ± 6.7	0.84
Years from HIV diagnosis	N/A	28.03 ± 9.1	29.7 ± 7.1	0.47
CD4/CD8 ratio	2.65 ± 1.36	0.56 ± 0.17	0.72 ± 0.35	0.055^a^
Number of comorbidities	N/A	2.50 ± 1.22	2.0 ± 1.27	0.12
Inflammatory markers at inclusion				
SEN‐CD4+	15.6 ± 22.7	61.1 ± 46.3	24.7 ± 35.1	0.05
SEN‐CD8+	147 ± 224	401 ± 182	255 ± 128	0.039^a^
SEN‐CD4‐38+	19 ± 23	54 ± 42	17 ± 18	0.01^a^
SEN‐TEMRA‐CD4+	10 ± 9	28 ± 35	5.6 ± 6.3	0.04^a^
SEN‐EM‐CD4+	6 ± 10	28 ± 24	16 ± 27	0.07
SEN‐CD8‐38+	133 ± 208	297 ± 138	173 ± 120	0.03^a^
SEN‐TEMRA‐CD8+	81.2 ± 155	237 ± 119	98.1 ± 98.0	0.02^a^
SEN‐EM‐CD8+	38 ± 46	108 ± 170	36 ± 26	0.06
CD8‐TEMRA‐RA‐	109 ± 207	299 ± 168	124 ± 109	0.007^a^
CD4‐TEMRA‐RA‐	28.2 ± 30.8	36.2 ± 39.8	11.9 ± 11.2	0.056^a^

^a^ PLWH with nadir CD4 count <200/mm^3^.

**Conclusions**: In PLWH aged over 60 years with CD4 nadir <200 cells/mm^3^, we found lower levels of immunosenescence markers on BIC/FTC/TAF compared to DTG/3TC. Our results suggest that past CD4 count should be considered as criteria for treatment decision choice between 2DR and 3DR in order to reduce risks of age‐associated complications.

## Co‐morbidities and complications of disease and/or treatment—Cardiovascular/metabolic including weight gain

### P283 Risk of hypertension in treatment‐naïve people with HIV (PWH) in the US receiving integrase strand inhibitors (INSTIs) versus non‐nucleoside reverse transcriptase inhibitors (NNRTIs) or tenofovir alafenamide (TAF) versus non‐TAF‐based regimens: pooled analysis of blood pressure data from five clinical trials

Priscilla Y. Hsue ^1^, Laura Waters^2^, Chloe Orkin^3^, Juan Manuel Tiraboschi^4^, Anchalee Avihingsanon^5^, Andrea Marongiu^6^, Andrew Whiteman^7^, Yuan Tian^7^, Carrie M. Nielson^8^, Keith Aizen^9^, Cal Cohen^10^, Jason T. Hindman^11^, Jürgen K. Rockstroh^12^

^1^Division of Cardiology, University of California, San Francisco, CA, USA. ^2^Mortimer Market Centre, Central and North West London NHS Foundation Trust, London, UK. ^3^Blizard Institute, Queen Mary University, London, UK. ^4^Department of Infectious Diseases, Bellvitge University Hospital, Barcelona, Spain. ^5^HIV Netherlands Australia Thailand Research Collaboration (HIV‐NAT), Thai Red Cross AIDS Research Center, Bangkok, Thailand. ^6^Real World Evidence, Gilead Sciences Ltd, Uxbridge, UK. ^7^Clinical Bioinformatics and Exploratory Analytics, Gilead Sciences, Inc., Foster City, CA, USA. ^8^Real World Evidence, Gilead Sciences, Inc., Foster City, CA, USA. ^9^Patient Safety, Gilead Sciences, Inc., Foster City, CA, USA. ^10^Medical Affairs, Gilead Sciences, Inc., Foster City, CA, USA. ^11^Clinical, Gilead Sciences, Inc., Foster City, CA, USA. ^12^Department of Medicine I, University of Bonn, Bonn, Germany

**Background**: PWH experience an increased risk of cardiovascular disease, contributing to morbidity and mortality. Traditional risk factors and certain antiretrovirals (ARVs) may exacerbate this risk. Using pooled data from completed first‐line therapy studies, we assessed whether ARV regimens containing INSTIs and/or TAF carry greater hypertensive risks than those without.

**Materials and methods**: US participant data from five randomized, double‐blind, phase III first‐line therapy studies including adult PWH receiving NNRTI/non‐TAF, INSTI/non‐TAF or INSTI/TAF regimens were pooled [1–5]. In the primary analysis, hypertension risk was assessed using American College of Cardiology/American Heart Association‐graded [6] longitudinal blood pressure (BP) measurements and modelled with proportional odds mixed‐effect regression through 108 weeks post‐treatment initiation. In the secondary analysis, time‐to‐incident composite hypertension event through 96 weeks post‐initiation was modelled using Cox proportional hazards regression. Events were defined as the first occurrence of a hypertension‐related adverse event (AE), the initiation of anti‐hypertensive medication, or consecutive BP records indicating stage ≥2 hypertension. Analyses were controlled for selected baseline characteristics.

**Results**: Baseline characteristics (*N* = 2411) are shown (Table 1). The secondary analysis included 528, 749 and 1134 participants from the NNRTI/non‐TAF, INSTI/non‐TAF and INSTI/TAF groups, respectively. At baseline, 34.0–34.5% and 13.3–15.2% per group had stage 1 and 2 hypertension, respectively, although only 6.7–7.4% were receiving anti‐hypertensives. Estimated marginal risks of hypertension were similar for INSTI/non‐TAF and INSTI/TAF versus NNRTI/non‐TAF (Figure 1a; *p* = 0.24). As noted in Figure 1b, 425 (19%) participants experienced composite events; of these, 40% were due to initiation of BP medications, 20% had hypertension‐related AEs, and 40% had incident stage 2 hypertension. Estimated hazard ratios (95% CI) for incident hypertension were 0.83 (0.59–1.16) and 0.94 (0.69–1.29) for INSTI/non‐TAF and INSTI/TAF, respectively, compared with NNRTI/non‐TAF.

**P283: Table 1:** Baseline demographics and clinical characteristics

	NNRTI/non‐TAF^a^ (*n* = 528)	INSTI/non‐TAF^a^ (*n* = 749)	INSTI/TAF^a^ (*n* = 1134)	𝛘^2b^
Age^c^ (years), mean (SD)	36.3 (10.7)	35.2 (10.8)	34.2 (11.1)	26.9
Male sex at birth^c^, *n* (%)	493 (93.4)	671 (89.6)	1024 (90.3)	19.0
Race^c^, *n* (%)				38.1
White	318 (60.3)	404 (53.9)	612 (54.0)	
Black	171 (32.4)	294 (39.3)	466 (41.1)	
Other	38 (7.2)	51 (6.8)	56 (4.9)	
BMI^c,d^, kg/m^2^, mean (SD)	26.3 (4.9)	26.4 (5.6)	26.5 (5.8)	2.8
Estimated GFR^c^, ml/min/1.73 m^2^, mean (SD)	117.9 (29.5)	124.8 (34.2)	128.8 (37.7)	71.3
Diastolic BP^c^, mmHg, mean (SD)	77.2 (9.7)	77.1 (9.7)	77.4 (9.7)	5.3
Systolic BP^c^, mmHg, mean (SD)	120.8 (13.5)	122.8 (13.8)	122.6 (13.0)	9.2
Stage 1 hypertension^e^, *n* (%)	182 (34.5)	255 (34.0)	390 (34.4)	8.8
Stage 2 hypertension^f^, *n* (%)	70 (13.3)	114 (15.2)	165 (14.6)	8.8
Use of anti‐hypertensives^g^, *n* (%)	39 (7.4)	52 (6.9)	76 (6.7)	8.6

Abbreviations: BMI, body mass index; BP, blood pressure; GFR, glomerular filtration rate; INSTI, integrase strand transfer inhibitor; NNRTI, non‐nucleoside reverse transcriptase inhibitor; TAF, tenofovir alafenamide.

^a^Includes trial participants from the US only; NNRTI/non‐TAF: EFV/FTC/TDF (272, 52%), RPV/FTC/TDF (256, 48%); INSTI/non‐TAF: E/C/F/TDF (518, 69%), DTG/ABC/3TC (231, 31%); INSTI/TAF: E/C/F/TAF (524, 46%), B/F/TAF (418, 37%), DTG+F/TAF (192, 17%); ^b^measure of cross‐study discrepancy (higher values = more discrepancy): degree of freedom parameter = 4; ^c^model covariate; ^d^data was missing for one participant; ^e^stage 1 hypertension defined by the American Heart Association (heart.org) as 130–139 mmHg systolic or 80–89 mmHg diastolic BP; ^f^stage 2 hypertension defined by the American Heart Association (heart.org) as 140–180 mmHg systolic or 90–120 mmHg diastolic BP; ^g^participants using anti‐hypertensives at or before baseline.

**P283: Figure 1:**
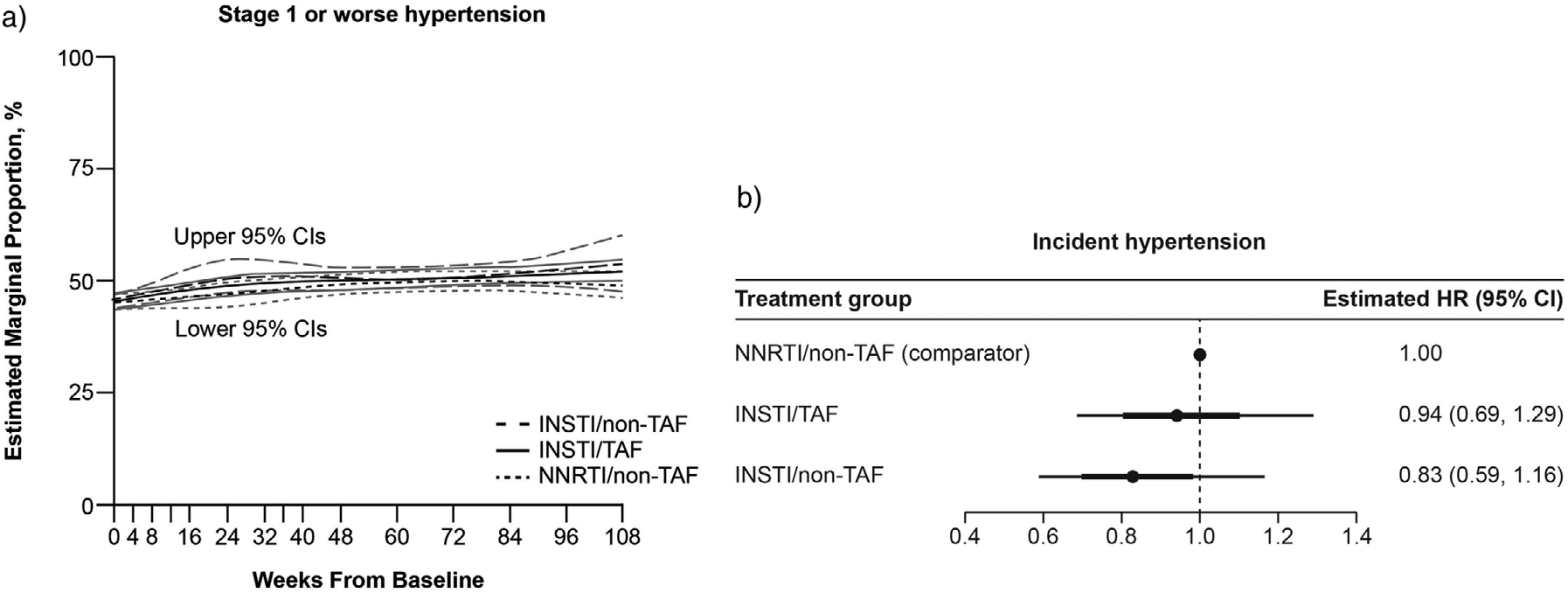
(a) Marginal proportions of hypertension (inter‐participant) and (b) incident hypertension in participants with no evidence of hypertension at baseline (*N* = 2238). Marginal proportions/HRs were adjusted for baseline covariates, including age, alanine aminotransferase, BMI, estimated GFR, sex at birth, race and systolic and diastolic blood pressure. (a) Primary analysis: all participants included regardless of indication of hypertension at baseline. Stage 1 hypertension is defined by the American Heart Association as 130–139 mmHg systolic or 80–89 mmHg diastolic blood pressure. (b) Thin lines indicate 95% CIs, thick lines indicate standard errors. Participants with hypertension at baseline (existing diagnoses and/or receiving anti‐hypertensive medications) were excluded (*n* = 173). Hypertension events: total events, 425 (19%); anti‐hypertensive medication initiated, 169 (7.6%); adverse event, 85 (3.8%); two blood pressure measurements of stage 2 or worse hypertension, 171 (7.6%). BMI, body mass index; GFR, glomerular filtration rate; HR, hazard ratio; INSTI, integrase strand transfer inhibitor; NNRTI, non‐nucleoside reverse transcriptase inhibitor; TAF, tenofovir alafenamide.

**Conclusions**: Baseline hypertension (stage ≥1) was present in ∼50% of this relatively young ARV‐naïve population, yet few were taking anti‐hypertensive medication. BP‐related events (initiation of medication, AEs and stage 2 hypertension) occurred in 19% of participants over a relatively short time. Hypertension risk for INSTI/non‐TAF and INSTI/TAF treatments was similar to NNRTI/non‐TAF treatments. This study highlights the need for careful monitoring and appropriate treatment of hypertension in this population, regardless of ARV choice.

**References**

1. ClinicalTrials.gov. Study to Evaluate the Safety and Efficacy of a Single Tablet Regimen of Emtricitabine/Rilpivirine/Tenofovir Disoproxil Fumarate Compared With a Single Tablet Regimen of Efavirenz/Emtricitabine/Tenofovir Disoproxil Fumarate in HIV‐1 Infected, Antiretroviral Treatment‐Naive Adults. ClinicalTrials.gov identifier: NCT01309243[Internet]. 2015 [cited 2024 Jul 1]. Available from: https://clinicaltrials.gov/study/NCT01309243.

2. ClinicalTrials.gov. Study to Evaluate the Safety and Efficacy of E/C/F/TAF (Genvoya®) Versus E/C/F/TDF (Stribild®) in HIV‐1 Positive, Antiretroviral Treatment‐Naive Adults. ClinicalTrials.gov identifier: NCT01780506[Internet]. 2018 [cited 2024 Jul 1]. Available from: https://clinicaltrials.gov/study/NCT01780506.

3. ClinicalTrials.gov. Study to Evaluate the Safety and Efficacy of E/C/F/TAF Versus E/C/F/TDF in HIV‐1 Positive, Antiretroviral Treatment‐Naive Adults. ClinicalTrials.gov identifier: NCT01797445[Internet]. 2020 [cited 2024 Jul 1]. Available from: https://clinicaltrials.gov/study/NCT01797445.

4. ClinicalTrials.gov. Study to Evaluate the Safety and Efficacy of Bictegravir/Emtricitabine/Tenofovir Alafenamide Versus Abacavir/Dolutegravir/Lamivudine in Human Immunodeficiency Virus‐1 (HIV‐1) Infected, Antiretroviral Treatment‐Naive Adults. ClinicalTrials.gov identifier: NCT02607930[Internet]. 2022 [cited 2024 Jul 1]. Available from: https://clinicaltrials.gov/study/NCT02607930.

5. ClinicalTrials.gov. Study to Evaluate the Safety and Efficacy of Bictegravir/Emtricitabine/Tenofovir Alafenamide Versus Dolutegravir + Emtricitabine/Tenofovir Alafenamide in Human Immunodeficiency Virus (HIV‐1) Infected, Antiretroviral Treatment‐Naive Adults. ClinicalTrials.gov identifier: NCT02607956[Internet]. 2022 [cited 2024 Jul 1]. Available from: https://clinicaltrials.gov/study/NCT02607956.

6. Whelton PK, Carey RM, Aronow WS, Casey Jr DE, Collins KJ, Himmelfarb CD, et al. ACC/AHA/AAPA/ABC/ACPM/AGS/APhA/ASH/ASPC/NMA/PCNA Guideline for the Prevention, Detection, Evaluation, and Management of High Blood Pressure in Adults: Executive Summary: A Report of the American College of Cardiology/American Heart Association Task Force on Clinical Practice Guidelines. Hypertension. 2018;71:1269‐324.

### P284 Two‐fold increased risk of cardiovascular events in people with multidrug‐resistant HIV: data from the PRESTIGIO registry

Tommaso Clemente ^1^, Sara Diotallevi^2^, Davide Minisci^3^, Antonio Di Biagio^4^, Riccardo Lolatto^2^, Letizia Attala^5^, Giovanni Cenderello^6^, Alessia Siribelli^1^, Camilla Muccini^1^, Sergio Lo Caputo^7^, Marcello Tavio^8^, Rebecka Papaioannu Borjesson^1^, Andrea Giacomelli^9^, Antonella Castagna^1^, Vincenzo Spagnuolo^2^

^1^Infectious Diseases, Vita‐Salute San Raffaele University, Milan, Italy. ^2^Infectious Diseases, IRCCS San Raffaele Scientific Institute, Milan, Italy. ^3^University Department of Infectious and Tropical Diseases, University of Brescia and ASST Spedali Civili, Brescia, Italy. ^4^Unit of Infectious Diseases, IRCCS Ospedale Policlinico San Martino, Genoa, Italy. ^5^Infectious Diseases Unit, Santa Maria Annunziata Hospital, Bagno a Ripoli, Italy. ^6^Infectious Disease Unit, Sanremo Hospital, Sanremo, Italy. ^7^Clinic of Infectious Diseases, Department of Clinical and Surgical Sciences, University of Foggia, Foggia, Italy. ^8^Infective Diseases, AOU Ospedali Riuniti, Ancona, Italy. ^9^Third Division of Infectious Diseases, ASST Fatebenefratelli Sacco, Luigi Sacco Hospital, Milan, Italy

**Background**: Aim of this study was to explore the probability of incident major adverse cardiovascular events (MACEs) in people with four‐class drug‐resistant HIV (4DR‐PWH).

**Methods**: Retrospective, propensity score‐matched cohort study on PWH without prior MACEs, classified as follows: (i) 4DR, from the PRESTIGIO registry [1]; (ii) non‐4DR. Groups matched by age (±3 years), sex‐assigned‐at‐birth and antiretroviral treatment (ART) duration (±3 years), with a 1:4 ratio. Baseline defined as the date of first 4DR evidence for 4DR‐PWH, the same date (index date) for matched non‐4DR controls. The primary outcome was the cumulative probability of first incident MACE (cardiovascular death, myocardial infarction, unstable angina, stroke, transient ischaemic attack, peripheral arterial ischaemia or revascularisation). Poisson regression modelled incidence rates (IRs), 95% confidence intervals (95% CIs) and incidence rate ratios (IRRs); follow‐up accrued from baseline until last visit (date of censoring: 12 April 2024). Kaplan‐Meier curves estimated cumulative probabilities of first incident MACE, compared using log‐rank test. Predictors of first MACE assessed by multivariable stepwise Cox model, including fixed (at baseline) and time‐dependent covariates (with univariable *p* < 0.100). Follow‐up accrued from baseline until first event or last visit.

**Results**: Overall, 223 4DR‐ and 797 non‐4DR‐PWH included characteristics reported in Table 1. During a median follow‐up of 8.2 (interquartile range 5.4–11.1) years (1833 person‐years‐of‐follow‐up [PY]), 23/223 (10.3%) 4DR‐PWH developed 29 incident MACEs: IR 1.6 (95% CI 1.1–2.3)/100 PY. During a median follow‐up of 8.4 (5.2–11.0) years (6450 PY), 42/797 (5.3%) non‐4DR controls developed 45 incident MACEs: IR 0.7 (95% CI 0.5–0.9)/100 PY; IRR (4DR/non‐4DR) 2.3 (95% CI 1.4–3.6); *p* < 0.001. Cumulative probabilities of first incident MACE were higher in 4DR‐ compared to non‐4DR‐PWH (Figure 1; *p* = 0.006). After adjusting for age (time‐dependent; per 5‐year‐higher: aHR 1.2 [95% CI 1.0–1.4]), sex‐assigned‐at‐birth (male versus female: aHR 2.2 [95% CI 0.9–5.0]), HIV load (time‐dependent; ≥50 vs. <50 copies/ml: aHR 2.2 [95% CI 1.2–3.9]), CD4+ nadir (per 100‐cell/mm^3^‐higher: aHR 1.0 [95% CI 0.9–1.1]), baseline smoking habit (aHR 1.7 [95% CI 1.0–3.0]), diabetes (aHR 2.1 [95% CI 1.2–4.0]), dyslipidaemia (aHR 2.0 [95% CI 1.0–3.9]), chronic kidney disease (aHR 2.5 [95% CI 0.8–7.8]) and HCV serostatus (positive versus negative: aHR 1.8 [95% CI 1.1–3.1]), a higher risk of MACEs was associated with 4DR status (aHR 1.8 [95% CI 1.0–3.3]).

**P284: Table 1:** Characteristics of people with HIV included in the analysis

Characteristics	Overall (*n* = 1020)	4DR (*n* = 223)	Non‐4DR (*n* = 797)	*p*‐value
Age at baseline (years)	50.1 (45.4–54.5)	50.0 (44.4–54.9)	50.3 (45.6–54.5)	0.590
Male sex‐assigned‐at‐birth	754 (73.9%)	163 (73.1%)	591 (74.2%)	0.816
Caucasian ethnicity	974 (95.5%)	209 (93.7%)	765 (96.0%)	0.148
Years since HIV diagnosis at baseline	21.5 (17.3–26.4)	21.4 (17.3–26.4)	21.6 (17.3–26.4)	0.946
ART duration at baseline (years)	17.8 (14.5–21.3)	18.2 (14.5–21.2)	17.7 (14.5–21.3)	0.517
Baseline HIV load (copies/mL)^a^	<20 (<1–85)	1512 (133–19,802)	<1 (<1–39)	<0.001
Baseline CD4+ T cell count (cells/mm^3^)	611 (401–829)	402 (210–601)	662 (466–874)	<0.001
Baseline CD4+/CD8+ ratio	0.64 (0.39–0.97)	0.37 (0.21–0.62)	0.71 (0.46–1.04)	<0.001
CD4+ T cell nadir (cells/mm^3^)	188 (74–307)	96 (23–187)	216 (102–333)	<0.001
Current or former smoking at baseline	653 (64.0%)	136 (61.0%)	517 (64.9%)	0.323
Pre‐baseline diagnosis of diabetes mellitus	101 (9.9%)	17 (7.6%)	84 (10.5%)	0.245
Pre‐baseline diagnosis of arterial hypertension	228 (22.4%)	44 (19.7%)	184 (23.1%)	0.331
Pre‐baseline diagnosis of dyslipidaemia	694 (68.0%)	148 (66.4%)	546 (68.5%)	0.600
Pre‐baseline diagnosis of chronic kidney disease	51 (5.0%)	10 (4.5%)	41 (5.1%)	0.816
Positive HCV serostatus at baseline	391 (38.3%)	71 (31.8%)	320 (40.2%)	0.029
Positive HBsAg at baseline	73 (7.2%)	15 (6.7%)	58 (7.3%)	0.871
Number of drugs in the ART regimen ongoing at baseline	3 (3–3)	3 (2–4)	3 (3–3)	<0.001
Resistance to no ART classes at last visit	689 (67.5%)	0 (0.0%)	689 (86.4%)	<0.001

Results described by median (interquartile range) or frequency (percentage); *p*‐values estimated by Mann–Whitney U‐test (continuous variables), chi‐square test or Fisher's exact test (categorical variables), as appropriate.

^a^ An undetectable HIV load was defined as <1 copies/ml, whereas a detectable but unquantifiable HIV load was defined as <20 copies/ml, as 20 copies/ml was the lower limit of HIV load quantification.

**P284: Figure 1:**
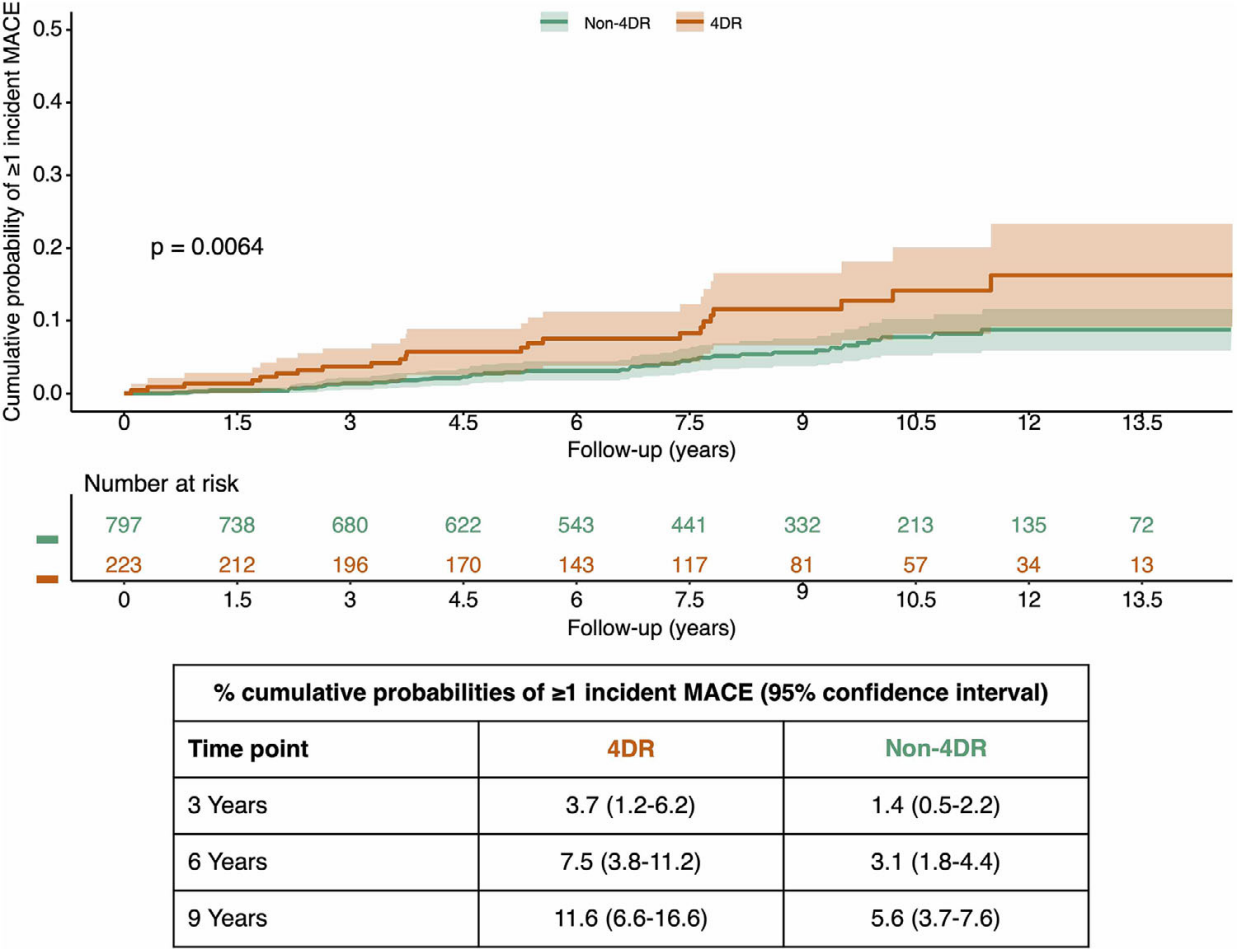
Kaplan–Meier curves for probabilities of the first incident major adverse cardiovascular event (MACE) in people with 4DR (orange line) and non‐4DR (green line) HIV.

**Conclusions**: In PWH, multidrug resistance is significantly associated with a higher incidence and risk of cardiovascular events. Early and prompt adoption of prevention strategies is mandatory in this fragile population.

**Reference**

1. Clemente T, Galli L, Lolatto R, Gagliardini R, Lagi F, Ferrara M, et al. Cohort profile: PRESTIGIO, an Italian prospective registry‐based cohort of people with HIV‐1 resistant to reverse transcriptase, protease and integrase inhibitors. BMJ Open. 2024;14(2):e080606.

### P285 Incident hypertension with antiretroviral therapy: real‐world evidence from the OPERA cohort

Philip C Lackey^1^, Laurence Brunet^2^, Jennifer S Fusco^2^, Gerald Pierone Jr^3^, Michael B Wohlfeiler^4^, Douglas T Dieterich^5^, Cassidy E Henegar^6^, Vani Vannappagari ^6^, Bryn Jones^7^, Annemiek de Ruiter^8^, Gregory Fusco^2^

^1^Internal Medicine/Section on Infectious Diseases, Wake Forest University School of Medicine, Winston‐Salem, NC, USA. ^2^Analysis, Epividian, Raleigh, NC, USA. ^3^Adult Primary Care, Whole Family Health Center, Vero Beach, FL, USA. ^4^AIDS Healthcare Foundation (AHF), Miami, FL, USA. ^5^Institute for Liver Medicine, Icahn School of Medicine at Mount Sinai, New York, NY, USA. ^6^Epidemiology and Real World Evidence, ViiV Healthcare, Durham, NC, USA. ^7^Franchise Medical, ViiV Healthcare, London, UK. ^8^Global Medical Sciences, ViiV Healthcare, London, UK

**Background**: Some studies indicate an increased risk of hypertension (HTN) with integrase strand transfer inhibitors (INSTI) and tenofovir alafenamide (TAF) use, but results are inconsistent. We assessed incident HTN with or without INSTI or TAF use in routine clinical care in the US.

**Materials and methods**: Adults with HIV with no evidence of HTN and normal blood pressure (BP) (nBP; systolic BP [SBP] <120 mmHg, diastolic BP [DBP] <80 mmHg) or normal/elevated BP (n/eBP; SBP <140 mmHg; DBP <90 mmHg) were included if they started a new two‐ or three‐drug ART regimen between 01 Jan 2016–31 Dec 2022 in the OPERA cohort. Incident HTN was defined as (a) two consecutive SBP ≥140 mmHg or DBP ≥90 mmHg, (b) new HTN diagnosis or (c) new antihypertensive prescription. Adjusted incidence rate ratios (IRR; multivariate Poisson regression) compared regimens containing INSTI, non‐nucleoside reverse transcriptase inhibitors (NNRTI) or boosted protease inhibitors (bPI) with/without TAF. Analysis were conducted among all individuals with n/eBP and in the subset with nBP, stratified by ART experience at baseline.

**Results**: A total of 7572 individuals with n/eBP were included, of whom 3220 had nBP; baseline characteristics are detailed in Table 1. In the nBP population, the HTN IR per 100 person‐years was 4.16 (95% CI 3.75–4.61) in ART‐naïve and 4.60 (3.99–5.30) in ART‐experienced individuals. No statistically significant association was observed between regimen and incident HTN in these groups (Figure 1). In the n/eBP population, the HTN IR was doubled (ART‐naïve: 7.89 [7.50–8.30]; ART‐experienced: 8.76 [8.17–9.39]). Among ART‐naïve and ART‐experienced individuals with n/eBP, no statistically significant associations were observed between any regimen and incident HTN (Figure 1).

**P285: Table 1:** Baseline population characteristics by ART experience, among individuals with normal or normal/elevated blood pressure and no evidence of HTN^a^, ^b^

	Normal BP^c^, ART‐naïve (*N* = 3220)	Normal BP^c^, ART‐experienced (*N* = 1442)	Normal/elevated BP^d^, ART‐naïve (*N* = 7572)	Normal/elevated BP^d^, ART‐experienced (*N* = 3428)
Median age (IQR)	30 (25–38)	41 (32–51)	30 (25–38)	42 (32–51)
Female sex, *n* (%)	519 (16)	311 (22)	953 (13)	605 (18)
Black race, *n* (%)	1710 (53)	576 (40)	4031 (53)	1343 (39)
Median HIV viral load (IQR)	64,835 (16,088–234,500)	20 (<20–528)	57,345 (15,400–193,000)	<20 (<20–320)
Diabetes, *n* (%)	35 (1)	63 (4)	100 (1)	160 (5)
Median eGFR (IQR)	117 (104–130)	102 (87–116)	116 (102–129)	101 (85–115)
Median BMI (IQR)	23 (21–26)	24 (22–28)	24 (21–28)	25 (22–29)

^a^ No current prescription for antihypertensive medication and no diagnosis of HTN;

^b^ 3242/10,814 (30%) ART‐naïve and 3115/6543 (48%) ART‐experienced individuals excluded due to prevalent HTN;

^c^ SBP <120 mmHG and DBP <80 mmHG at last reading within 12 months before/at ART initiation;

^d^ SBP <140 mmHG and DBP <90 mmHG at last reading within 12 months before/at ART initiation.

**P285: Figure 1:**
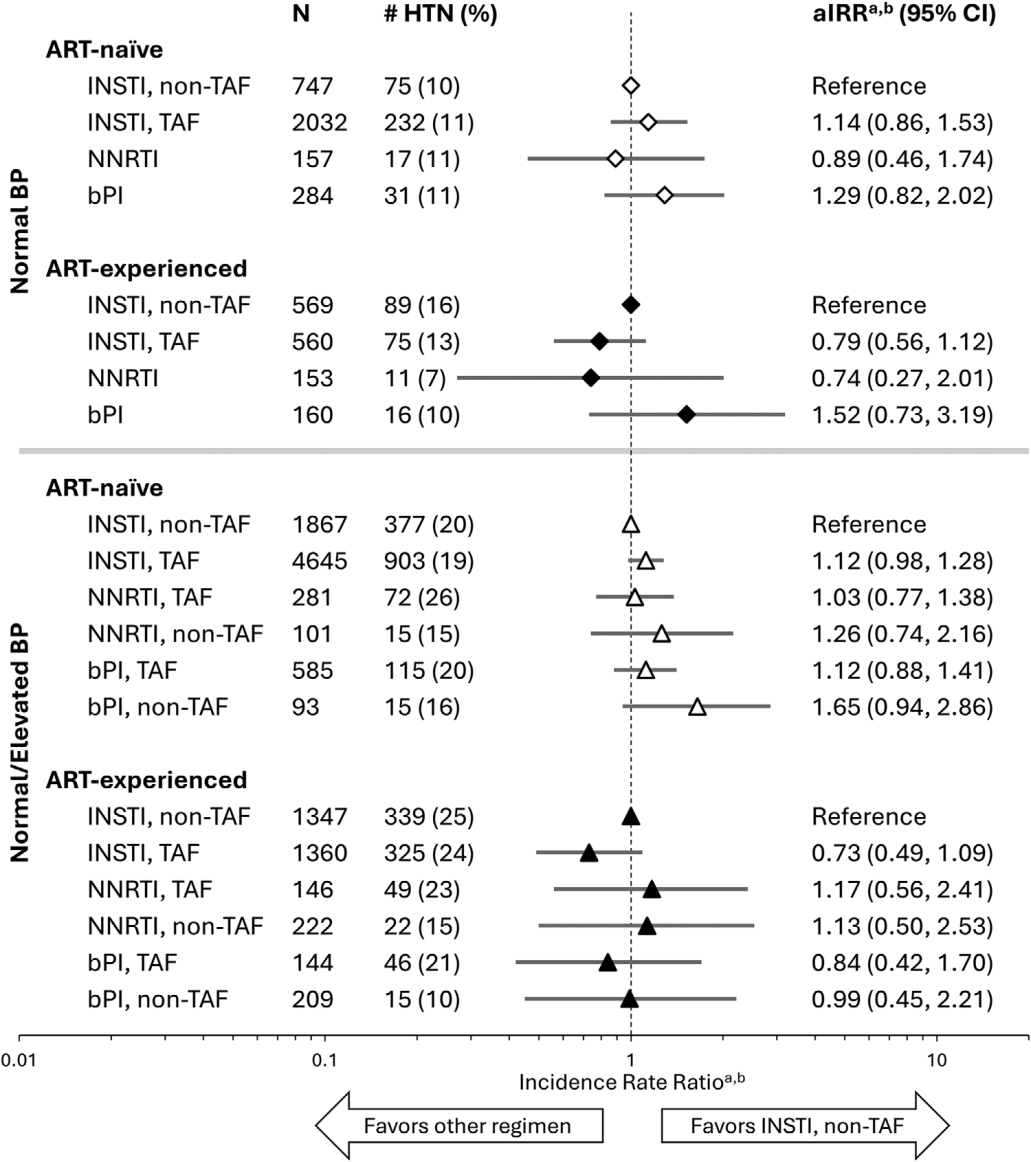
Association between ART regimen and incident hypertension by ART experience among individuals with normal or normal/elevated blood pressure. ^a^Multivariate Poisson regression adjusted for baseline age, female sex, black race, VL, diabetes, eGFR and SBP, as well as time‐updated BMI; ^b^individuals missing BMI, race and/or eGFR data were excluded from the multivariate Poisson modelling (Normal BP—ART‐naïve: *n* = 288; normal BP—ART‐experienced: *n* = 198; normal/elevated BP—ART‐naïve: *n* = 703; normal/elevated BP—ART‐experienced: *n* = 472); ^c^TAF and non‐TAF use was combined among NNRTI and bPI users due to the small number of events.

**Conclusions**: In this large real‐world US cohort, no statistically significant association was detected between ART regimens and incident HTN among the ART‐naïve population, although among those with n/eBP, a modest, non‐statistically significant elevated risk was observed for INSTI with TAF and bPI without TAF, compared to INSTI without TAF. No difference was detected across regimens in ART‐experienced individuals who were more likely to have HTN identified prior to our observation period.

### P286 Evaluation of cardiovascular disease amongst adults with perinatally acquired HIV

Merle Henderson ^1^, Vibeke Klastrup^2^, Salwa Ahmad^3^, Jessica Glenn^3^, Sara Ayres^4^, Hana Jadayel^5^, Paula Seery^5^, Caroline Foster^5^, Sarah Fidler^1^

^1^Infectious Disease, Imperial College Healthcare NHS Trust, Imperial College London, London, UK. ^2^Infectious Disease, Aarhus University, Aarhus, Denmark. ^3^Infectious Disease, Imperial College London, London, UK. ^4^Infectious Disease, Imperial College Healthcare NHS Trust, London, UK. ^5^Paediatric Infectious Disease, Imperial College Healthcare NHS Trust, London, UK

**Background**: Since 1990, an estimated 11 million children have been born with perinatally acquired HIV (PaHIV). In resourced settings, adults with PaHIV are approaching the fifth decade of life. Background rates of co‐morbidities associated with cardiovascular disease (CVD), including hypertension (blood pressure (BP) ≥140/90 mmHg) and hypercholesterolaemia (total cholesterol ≥5 mmol/l), amongst the UK general population aged 16–44 years are ∼10% and 50%, respectively [1]. Lifelong HIV‐associated inflammation and antiretroviral therapy (ART) exposure may increase this risk. We aimed to investigate cardiovascular and metabolic risk in a unique PaHIV UK cohort.

**Methods**: Case‐note review of an adult perinatal HIV service. Data captured included age, gender, ethnicity, ART, last CD4+ count, HIV‐1 viral load, lipids, HbA1c, body mass index (BMI), smoking history and in‐clinic BP across the last three attendances. Hypertension was defined as ≥140/90 mmHg by the World Health Organization (WHO) and ≥130/80 mmHg by American Heart Association (AHA) guidelines. Metabolic syndrome was defined as triglycerides ≥1.7 mmol/L, HDL <1.04 mmol/L (men) and <1.29 mmol/L (women) and BP ≥130/85 mmHg. Descriptive analysis identified those with hypertension and metabolic abnormalities.

**Results**: Data were collected on 225 individuals ≥18 years with PaHIV. Median age 27 years (IQR 23–30), 123 (55%) female and 189 (85%) of black ethnicity. One hundred and eighty‐four of 223 (83%) had an HIV‐1 viral load <50 copies/mL and 199 (89%) <200 copies/mL. Median CD4+ count was 634 cells/ml (IQR 438–815) and years on ART were 19 (IQR 13–22). Twenty‐one of 225 (9%) had WHO‐defined hypertension and 48 (21%) had AHA‐defined hypertension. Eighty‐four of 212 (39%) had abnormal triglycerides and/or HDL‐C and 120/219 (55%) were overweight (BMI 25–29) or obese (BMI ≥ 30). Six (3%) fulfilled the criteria for metabolic syndrome. Fifty‐two of 220 (24%) were smokers. For those with AHA‐defined hypertension, 41 (85%) had suppressed HIV with 39 (81%) on integrase inhibitor‐ART, and nine (19%) received anti‐hypertensive treatment.

**Conclusion**: Most individuals were virally suppressed on ART with restored immune function. Compared to the age‐matched UK population, there was no increased frequency of WHO‐defined hypertension or co‐morbidities associated with CVD. However, using AHA‐definitions one in five young people were hypertensive. Linkage to interventions including weight reduction and cardiology prevention services could enhance long‐term survival.

**Reference**

1. NHS England. Health Survey for England 2021: Data Tables [Internet]. 2021 [cited 2024 Jun 26]. Available from: digital.nhs.uk/data‐and‐information/publications/statistical/health‐survey‐for‐england/2021‐part‐2#data‐sets.

### P287 Evaluation of changes in Systematic Coronary Risk Evaluation 2 (SCORE2) in experienced people with HIV switching to DOR/3TC/TDF: real‐world data from DOROTEA multicentre cohort

Valentina Iannone ^1^, Alberto Borghetti^2^, Andrea Giacomelli^3^, Davide Moschese^3^, Filippo Lagi^4^, Massimiliano Fabbiani^5^, Andrea Rabbione^3^, Giuseppe Formica^4^, Francesca Lombardi^6^, Chiara Papalini^7^, Andrea Tommasi^7^, Daniela Francisci^7^, Andrea De Vito^8^, Giordano Madeddu^8^, Maria Vittoria Cossu^3^, Simona Di Giambenedetto^6^, Arturo Ciccullo^9^

^1^Medical and Surgical Science, Catholic University of Sacred Heart, Rome, Italy. ^2^Clinical and Experimental Medicine, Infectious Diseases Unit, University of Pisa, Pisa, Italy. ^3^Infectious Diseases, Azienda Socio Sanitaria Territoriale (ASST) Fatebenefratelli Sacco, Luigi Sacco Hospital, Milan, Italy. ^4^Infectious and Tropical Diseases Unit, Careggi University Hospital, Florence, Italy. ^5^Medical Sciences, Infectious and Tropical Diseases Unit, University Hospital of Siena, Siena, Italy. ^6^Department of Infectious Diseases, Fondazione Policlinico Universitario A. Gemelli Istituto di Ricovero e Cura a Carattere Scientifico (IRCCS), Rome, Italy. ^7^Department of Medicine, Clinic of Infectious Diseases, Santa Maria della Misericordia Hospital, Perugia, Italy. ^8^Department of Medicine, Surgery and Pharmacy, University of Sassari, Sassari, Italy. ^9^Infectious Diseases Unit, San Salvatore Hospital, L'Aquila, Italy

**Background**: In the landscape of antiretroviral treatments, doravirine (DOR) based regimen demonstrated long‐term tolerability and a favourable lipid profile [1–3], with the potential to reduce cardiovascular disease (CVD) risk in people with HIV (PWH). We aimed to assess the impact of switching to DOR/3TC/TDF on cardiovascular risk estimated by means of Systematic Coronary Risk Evaluation 2 (SCORE2).

**Materials and methods**: This was a multicentric retrospective study in which PWH switched to DOR/3TC/TDF (baseline, BL) and were enrolled in the study from six outpatient clinics in Italy. Viroimmunological parameters and lipid profiles were collected at BL and after 48 weeks (48W) of follow‐up and CVD risk was estimated at BL and 48W by means of SCORE2 in 172 PWH who met eligibility criteria. Treatment discontinuations (TD) were recorded. To assess changes in the lipid profile, SCORE2 and predictors of those changes, multivariable linear regression was employed.

**Results**: A total of 312 PWH were included, predominantly male (194, 62.4%), with a median age of 51 years (IQR 43–57). Full population characteristics are summarised in Table 1. After 48W, we observed a significant reduction in median SCORE2 (−0.7, *p* < 0.001). Baseline SCORE2 was inversely associated with improvement after 48W (B −0.3, T −6.5, *p* < 0.001), whereas a lower age was positively associated with improvement (B 0.6, T 2.7, *p* = 0.014) at a multivariate regression. A significant median change reduction in total cholesterol (TC) (median change −18 mg/dl, *p* < 0.001), LDL (‐10 mg/dl, *p* < 0.001) and triglycerides (−14 mg/dl, *p* < 0.001) were observed. Baseline cholesterol levels predicted a decrease in TC (B −0.6, T −9.6, *p* < 0.001). Similarly, changes in LDL and triglycerides were predicted solely by their baseline levels (LDL: B −0.7, T −8.5, *p* < 0.001; triglycerides: B −0.7, T −11.5, *p* < 0.001). No significant changes were observed in BMI. Seventy‐six TD were observed throughout 868.36 patient‐years of follow‐up (a rate of 8.7 per 100 patient‐year follow‐up [PYFU]); main reasons were toxicity (23, 7.4%), subject decision (19, 6.1%) and pill dimension (10, 3.2%).

**P287: Table 1:** Full population characteristics at baseline (BL)

Variables	*N* = 312
Age, (years) median (IQR)	51 (43−57)
Assigned male at birth, *n* (%)	194 (62.4)
HIV mode of acquisition, heterosexual, *n* (%)	128 (41.0)
HIV mode of acquisition, MSM, *n* (%)	136 (43.6)
HIV mode of acquisition, PWID, *n* (%)	31 (9.9)
HIV mode of acquisition, unknown, *n* (%)	17 (5.5)
Years of HIV infection, median (IQR)	14 (8−22)
Years of ART exposure, median (IQR)	21 (9−29)
CDC stage C, *n* (%)	83 (26.6)
Ab anti‐HCV, *n* (%)	16 (5.1)
CD4+ cell count nadir, median (IQR)	684 (479−898)
Zenith HIV‐RNA, log10 cps/ml, median (IQR)	5.43 (4.85−5.70)
Years of virological suppression, median (IQR)	10 (3−20)
Pre‐switch ART regimen, 2NRTI + INI, *n* (%)	98 (31.4)
Pre‐switch ART regimen, 2NRTI + NNRTI, *n* (%)	142 (45.5)
Pre‐switch ART regimen, 2NRTI + PI, *n* (%)	44 (14.1)
Pre‐switch ART regimen, 3TC‐based 2DR, *n* (%)	16 (5.1)
Pre‐switch ART regimen, other, *n* (%)	12 (3.8)
Active smokers, *n* (%)	106 (34.0)
Diabetes on treatment, *n* (%)	27 (8.6)
Hypertension on treatment, *n* (%)	68 (21.8)
Cardiovascular event pre‐BL, *n* (%)	15 (4.8)
Body mass index (BMI), median (IQR)	25.2 (23.8−27.0)
Eligible PWH for SCORE2 calculation, BL, *n* (%)	172 (55,1)
SCORE2 at BL, median (IQR)	5.0 (3.0−8.5)

Abbreviations: 2DR, two‐drug‐regimen; ART, antiretroviral therapy; B, unstandardized beta coefficient; CDC, Centers for Disease Control and Prevention; INI, integrase inhibitor; MSM, men who have sex with men; NNRTI, non‐nucleoside reverse transcriptase inhibitors; NRTI, nucleoside reverse transcriptase inhibitor; PI, protease inhibitor; PWID, people who inject drugs; SCORE2, Systematic Coronary Risk Evaluation 2; T, t‐ratio is equal to the slope divided by its standard error.

**Conclusions**: The amelioration observed in the lipid profile and in SCORE2 risk estimation at 48 weeks provide further evidence that DOR/3TC/TDF is a suitable option for dyslipidaemic PWH with a high risk of CVD.

**References**

1. Mussini C, Guaraldi G. The best place for doravirine. Lancet HIV. 2024;11(2):e64‐5. Erratum in: Lancet HIV. 2024 Mar 28:S2352‐3018(24)00091‐2.

2. Kumar P, Johnson M, Molina JM, Rizzardini G, Cahn P, Bickel M, et al. Brief report: switching to DOR/3TC/TDF maintains HIV‐1 virologic suppression through week 144 in the DRIVE‐SHIFT trial. J Acquir Immune Defic Syndr. 2021;87(2):801‐5.

3. Calza L, Colangeli V, Pensalfine G, Appolloni L, Vitale S, Bon I, et al. Doravirine/lamivudine/tenofovir disoproxil fumarate in virologically suppressed people living with HIV: a real‐life experience. Int J STD AIDS. 2023;34(14):1018‐23.

### P288 Cabotegravir and rilpivirine reduce the risk of developing metabolic syndrome

Martina Ranzenigo, Sara Diotallevi, Riccardo Lolatto, Hamid Hasson, Silvia Nozza, Flavia Badalucco Ciotta, Maria del Carmen Garcia Martearena, Ilaria Mainardi, Gaia Catalano, Antonella Castagna, Camilla Muccini

Infectious Diseases, San Raffaele Hospital, Milano, Italy

**Background**: The aims of this study were to describe the prevalence and incidence of metabolic syndrome (MS) in people with HIV (PWH) before and after switching to long‐acting cabotegravir (CAB) and rilpivirine (RPV) and to define risk factors associated with the development of MS.

**Materials and methods**: SCohoLART (NCT05663580) is a single‐centre, prospective cohort study enrolling PWH on virological suppression who switched to bimonthly long‐acting CAB+RPV. Participants with a previous stable oral regimen for ≥1 year and who have ≥1 determination of MS before (PRE‐CAB+RPV) and after switching to CAB+RPV were included. MS diagnosis was based on the NCEP ATP III 2005 criteria. A univariable Poisson regression model was used to estimate and compare crude incidence rates of MS. A multivariable Cox regression model with time‐dependent covariates was used to assess factors associated with the risk of developing MS.

**Results**: We included 393 PWH: 91.6% were male, with a median age of 50.0 years (41.0–56.9). Participants’ characteristics at the switch to CAB+RPV are described in Table 1. At the beginning of PRE‐CAB+RPV and CAB+RPV periods, the overall prevalence of MS was 46.6% and 50.6% (95% CI 45.58–55.69), respectively. After switching to CAB+RPV, MS prevalence significantly increased (McNemar's test: *p* = 0.002) to 53.2% (95% CI 48.11–58.20). The incidence rate of MS decreased from 2.37/100 person‐years of follow‐up (PYFU; 95% CI 1.36–3.86) in the 646PRE‐CAB+RPV period to 1.24/100‐PYFU (95% CI 0.60–2.29) after the switch to CAB+RPV (*p* = 0.10). After adjusting for sex, older age (adjusted hazard ratio [aHR] per 5 years older: 1.71 [95% CI 1.38–2.12]; *p* < 0.0001) was associated with an increased risk of MS; receiving CAB+RPV appeared to be a protective factor in the development of MS (CAB+RPV versus PRE‐CAB+RPV period: aHR 0.03 [0.009–0.10]; *p* < 0.0001), as well as greater years of virological suppression (aHR per 5 years older: 0.58 [0.34–0.98]; *p* = 0.04).

**P288: Table 1:** Participants’ characteristics at the switch to cabotegravir and rilpivirine

	*N* = 393
Age, median (IQR)	50.0 (41.0−56.9)
Male, *n* (%)	360 (91.6%)
Years from HIV diagnosis, median (IQR)	15.0 (9.2−21.4)
Years of antiretroviral therapy, median (IQR)	12.1 (8.2−17.9)
Years of virological suppression, median (IQR)	9.4 (5.7−13.1)
Previous AIDS diagnosis, *n* (%)	42 (10.7%)
Nadir CD4+ ≤200 cells/µl, *n* (%)	103 (26.3%)
Nadir CD4+ (cells/µl), median (IQR)	318 (194−490)
CD4+ (cells/µl), median (IQR)	780 (593−988)
CD4+/CD8+, median (IQR)	0.97 (0.69−1.27)
Positive HBcAb, *n* (%)	93 (29.8%)
Body mass index (kg/m^2^), median (IQR)	25 (23−27)
Waist circumference (cm), median (IQR)	91 (85−98)
Hypertension, yes	72 (18.3%)
Diabetes mellitus, yes	14 (3.56%)
HDL cholesterol (mg/dl)	48 (40−56)
LDL cholesterol (mg/dl)	112 (96−135)
Total cholesterol (mg/dl)	179 (159−200)
Glucose (mg/dl)	89 (83−97)
Triglycerides (mg/dl)	100 (76−138)
Type of ART regimen in use at CAB+RPV start	
2NRTI‐1PI	9 (2.29%)
2NRTI‐1NNRTI	114 (29.0%)
2NRTI‐1INI	142 (36.1%)
Dual therapy	124 (31.6%)
Other regimen	4 (1.02%)
Months to MS diagnosis in participants with MS diagnosis during CAB+RPV	5.23 (3.60−8.82)
Months of follow‐up of CAB+RPV	13.5 (10.1−15.6)

Abbreviations: CAB, cabotegravir; HBcAb, hepatitis B core antibody; HDL, high‐density lipoprotein; INI, integrase inhibitor; IQR, interquartile range; LDL, low‐density lipoprotein; MS, metabolic syndrome; NCEP ATP‐III, National Cholesterol Education Program Adult Treatment Panel‐III; NNRTI, non nucleoside reverse transcriptase inhibitor; NRTI, nucleoside reverse transcriptase inhibitor; PI, protease inhibitor; RPV, rilpivirine.

**Conclusions**: In our study, MS incidence decreased in PWH while receiving CAB+RPV. The use of CAB+RPV seemed to reduce the risk of developing MS after 1 year of follow up.

### P289 Incidence of metabolic syndrome in people with HIV in Italy who started ART since 2008: data from the ICONA cohort

Elena Bruzzesi ^1^, Alessandro Tavelli^2^, Romina Salpini^3^, Anna Carraro^4^, Marta Camici^5^, Chiara Papalini^6^, Gregorio Recchia^7^, Carmen Rita Santoro^8^, Maria Vittoria Cossu^9^, Alessandra Guida^10^, Annalisa Mondi^5^, Giulia Marchetti^11^, Giovanni Guaraldi^12^, Antonella D'Arminio^2^, Silvia Nozza^1^

^1^Infectious Diseases Unit, Vita‐Salute San Raffaele University, Milan, Italy. ^2^ICONA Foundation, Milan, Italy. ^3^Department of Biology, University of Rome Tor Vergata, Rome, Italy. ^4^Infectious Diseases Unit, SM Goretti Hospital, Sapienza University of Rome, Latina, Italy. ^5^Clinical and Research Infectious Diseases Department, National Institute for Infectious Diseases Lazzaro Spallanzani IRCCS, Rome, Italy. ^6^Infectious Diseases Clinic, Santa Maria della Misericordia Hospital, Università degli Studi di Perugia, Perugia, Italy. ^7^Department of Public Health and Infectious Diseases, Sapienza University of Rome, Rome, Italy. ^8^Infectious Diseases Clinic, University of Bari, Bari, Italy. ^9^Department of Infectious Diseases, ASST Fatebenefratelli Sacco University Hospital, Milan, Italy. ^10^Infectious Diseases and Gender Medicine Unit, Cotugno Hospital, Napoli, Italy. ^11^Clinic of Infectious Diseases, ASST Santi Paolo e Carlo, Department of Health Sciences, University of Milan, Milan, Italy. ^12^Department of Surgical, Medical, Dental and Morphological Sciences, University of Modena, Modena, Italy

**Background**: The prevalence of metabolic syndrome (MetS) in people with HIV (PWH) on ART is higher than in people without. Nevertheless, the role of CD4 count at diagnosis has not been investigated yet.

**Material and methods**: Primary endpoint is time to diagnosis of MetS, as defined by modified National Cholesterol Education Program Adult Treatment Panel III (NCEP ATP III) criteria [1], in PWH enrolled in the Italian Cohort of Patients Naive from Antiretrovirals (ICONA cohort) who started ART with a diagnosis of recent HIV infection (RHI), chronic HIV infection with CD4 count above (CHI) or below 200 cells/mm^3^ (advanced, AHI). Abdominal fat accumulation was assessed by waist circumference or, if missing, by formula as in Taramasso et al. [2] study. Incidence rates (IRs) of MetS were calculated as the number of events per 100 person‐years of follow‐up (PYFU) with 95% confidence intervals (95% CIs). Kaplan−Meier curves estimated cumulative probabilities of the first incident MetS. Univariable and multivariable Cox proportional hazard models were applied to estimate factors associated with MetS, for age, sex, risk factor for HIV acquisition, ethnicity, year of ART start, HCV and HBV coinfection, first‐line ART regimen, HIV‐RNA and CD8 count at ART start.

**Results**: Among 13,034 PWH starting ART after 2008 enrolled in ICONA, 11,137 were included in the analysis after excluding those with a diagnosis of MetS (974, 7.47%) or major adverse cardiac event (MACE) (63, 0.48%) and those lost to follow‐up. Six hundred and eighty‐five (6.15%) were diagnosed with RHI, 7253 (65.1%) with CHI and 3199 (28.72%) with advanced HIV disease (Table 1). Overall, IR of MetS was 3.96 × 100 PYFU (3.8–4.1) with an overall prevalence of 18.5% (17.8–19.2), more frequent in AHI (*p* < 0.001). Cumulative 5‐year probability of MetS in AHI (25.6% [23.8–27.4%]) was higher than in CHI (15.7% [14.8–16.7%]) and RHI (12.9% [10.2–16.3%], *p* < 0.001) (Figure 1). At multivariable analysis, a higher adjusted hazard of MetS was found for advanced HIV disease versus CHI (aHR 1.39 [1.23–1.57]) (*p* < 0.001), whereas no difference was observed between CHI and RHI and when comparing calendar period 2008–2015 to 2016–2023 (*p* = 0.420).

**P289: Table 1:** Demographical and clinical characteristics according to stage of HIV infection

	Chronic HIV infection	Advanced HIV infection	Recent HIV infection	Total	*p*‐value
	7253 (65.1%)	3199 (28.7%)	685 (6.2%)	11,137 (100.0%)	
Male sex	5942 (81.9%)	2493 (77.9%)	649 (94.7%)	9084 (81.6%)	<0.001
Age	38 [30−46]	43 [35−51]	34 [27−43]	39 [31−47]	<0.001
Ethnicity					<0.001
Caucasian	6087 (83.9%)	2533 (79.2%)	609 (88.9%)	9229 (82.9%)	
Black	619 (8.5%)	398 (12.4%)	36 (5.3%)	1053 (9.5%)	
Transmission mode					<0.001
MSM	3805 (52.5%)	1049 (32.8%)	506 (73.9%)	5360 (48.1%)	
Heterosexual	2507 (34.6%)	1629 (50.9%)	115 (16.8%)	4251 (38.2%)	
PWID	472 (6.5%)	215 (6.7%)	31 (4.5%)	718 (6.4%)	
Positive HCV status	687 (10.1%)	291 (9.8%)	43 (6.6%)	1021 (9.8%)	0.019
Positive HBsAg status	325 (5.0%)	170 (5.9%)	18 (2.9%)	513 (5.1%)	0.007
Year^a^	2015 [2012−2018]	2016 [2012−2018]	2017 [2015−2019]	2015 [2012−2018]	<0.00
Months to ART start	7.8 [2.5−60.9]	1.9 [1.0−4.0]	1.6 [0.7−3.2]	4.0 [1.6−29.9]	<0.001
AIDS	208 (2.9%)	1032 (32.3%)	17 (2.5%)	1257 (11.3%)	<0.001
Log HIV RNA >5log^a^	1941 (26.9%)	2113 (67.3%)	353 (52.3%)	4407 (40.0%)	<0.001
CD4 count^a^	413 [313−554]	73 [30−136]	484 [347−646]	335 [165−492]	<0.001
<200	0 (0.0%)	3199 (100.0%)	45 (6.6%)	3244 (29.1%)	
200‐350	2466 (34.0%)	0 (0.0%)	130 (19.0%)	2596 (23.3%)	
350‐500	2428 (33.5%)	0 (0.0%)	183 (26.7%)	2611 (23.4%)	
>500	2359 (32.5%)	0 (0.0%)	327 (47.7%)	2686 (24.1%)	
Abacavir exposure	1638 (22.6%)	815 (25.5%)	140 (20.4%)	2593 (23.3%)	0.001
First anchor drug NNRTI	2362 (32.6%)	370 (11.6%)	82 (12.0%)	2814 (25.3%)	<0.001
First anchor drug INSTI	2877 (39.7%)	1480 (46.3%)	356 (52.0%)	4713 (42.3%)	<0.001
First anchor drug PI	1880 (25.9%)	1288 (40.3%)	159 (23.2%)	3327 (29.9%)	<0.001
Years of follow‐up	5.4 [2.4−8.4]	4.8 [1.9−8.2]	4.2 [2.0−6.5]	5.1 [2.2−8.2]	<0.001
Diagnosis of MetS	1201 (16.6%)	775 (24.2%)	82 (12.0%)	2058 (18.5%)	<0.001

^a^ Demographic and immuno‐virological characteristics at ART start.

**P289: Figure 1:**
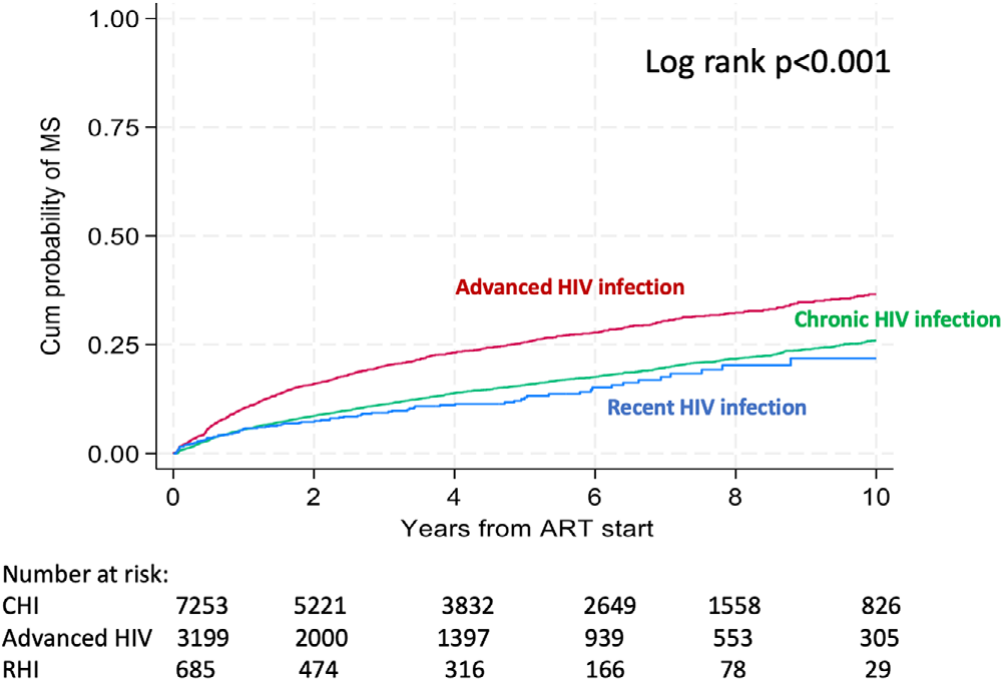
Cumulative 10‐year probability of developing metabolic syndrome according to stage of HIV infection.

**Conclusions**: Our findings show that PWH who start ART in AHI are at higher risk of developing MetS, being independent of calendar period and other key confounders.

**References**

1. National Cholesterol Education Program Expert Panel on Detection Evaluation and Treatment of High Blood Cholesterol in Adults (Adult Treatment Panel III). Third Report of the National Cholesterol Education Program (NCEP) Expert Panel on Detection, Evaluation, and Treatment of High Blood Cholesterol in Adults (Adult Treatment Panel III)—Final Report. Circulation. 2002;106:3143‐421.

2. Taramasso L, Bonfanti P, Ricci E, Maggi P, Orofino G, Squillace N, et al; CISAI study group. Metabolic syndrome and body weight in people living with HIV infection: analysis of differences observed in three different cohort studies over a decade. HIV Med. 2022;23(1):70‐9.

### P290 The effect of bictegravir/emtricitabine/tenofovir alafenamide (B/F/TAF) on whole‐body insulin sensitivity in volunteers without HIV

Joseph Heskin ^1^, Roya Movahedi^1^, Sujin Kang^2^, Ruth Byrne^2^, Krestine Elecito^2^, Perrine Gridel^2^, Paola Marchesani^2^, Graeme Moyle^1^, Ana Milinkovic^1^, Marta Boffito^1^

^1^Directorate of Sexual Health and HIV Medicine, Chelsea and Westminster NHS Foundation Trust, London, UK. ^2^Clinical Research Facility, Chelsea and Westminster NHS Foundation Trust, London, UK.

**Background**: Following the reports of weight gain and metabolic changes during INSTI therapy, we investigated the impact of B/F/TAF on total body glucose disposal over 28 days in HIV‐negative, metabolically healthy volunteers. Previous studies [1] had not found an effect of TAF regimens on glucose disposal. Clamps were performed, and glucose disposal rate (GDR) was calculated using the method of Fronzo et al. [2].

**Method**: This was a 72‐day, open‐label, two‐arm, crossover, single‐centre study. Participants were randomized 1:1 to either start with 28 days of B/F/TAF followed by 44 days without treatment (Group 1), or no treatment for 43 days followed by 28 days of B/F/TAF (Group 2). A hyperinsulinaemic‐euglycaemic clamp was carried out on days 1, 28 and 72. Group 1 underwent a 14‐day washout period following the second clamp. The primary study outcome was the change from baseline in total body glucose disposal by the euglycaemic clamp method after 28 days of treatment. Statistical assessments of the change in estimated GDR were performed using the Wilcoxon signed‐rank test (within‐group) and the two‐sample Wilcoxon rank‐sum (Mann–Whitney) test (between‐group).

**Results**: A total of 18 volunteers completed the study, with 11 in Group 1 and seven in Group 2. Within Group 1, the mean GDR was 7.52 mg/kg/min (SD 3.67) at baseline versus 8.50 mg/kg/min (SD 3.72) on day 28 (*p* = 0.32), with a mean change of 13%. Within Group 2, the mean GDR was 6.54 mg/kg/min (SD 1.86) on day 28 versus 5.85 mg/kg/min (SD 2.67) on day 72 (*p* = 0.38), with a mean change of ‐11%. There were no statistically significant changes in GDR between groups at baseline (mean 7.52 [SD 3.67] mg/kg/min vs. 6.11 [2.94] mg/kg/min, *p* = 0.31), at day 28 (8.50 [3.72] mg/kg/min vs. 6.54 [1.86] mg/kg/min, *p* = 0.27), or at day 72 (9.65 [SD 5.07] mg/kg/min vs. 5.85 [2.67] mg/kg/min, *p* = 0.13).

**Conclusion**: Treatment with Biktarvy for 28 days was not associated with a statistically significant impact on total body insulin sensitivity, as measured using the hyperinsulinaemic‐euglycaemic clamp method. Further data from the study will examine metabolic outcomes, pituitary and appetite hormones.

**References**

1. Spinner C, Avery A, Flamm JA, Crofoot G, Brinson C, Kronborg G, et al. Outcomes of participants switching from F/TDF to F/TAF for PrEP: week 48 results from the DISCOVER open label phase. J Int AIDS Soc. 2021;24(S4):29.

2. DeFronzo RA, Tobin JD, Andres R. Glucose clamp technique: a method for quantifying insulin secretion and resistance. Am J Physiol. 1979;237:E214−23.

### P291 Significant impact of previous major cardiovascular events (MACEs) and viraemia on risk of new MACEs in people living with HIV on antiretroviral therapy

Caterina Candela ^1^, Alessia Siribelli^1^, Tommaso Clemente^1^, Riccardo Lolatto^2^, Michele Bellomo^2^, Hamid Hasson^2^, Vincenzo Spagnuolo^3^, Antonella Castagna^3^, Silvia Nozza^3^, Camilla Muccini^2^

^1^Infectious Diseases, Vita‐Salute San Raffaele University, Milan, Italy. ^2^Infectious Diseases, IRCCS San Raffaele Scientific Institute, Milan, Italy. ^3^Infectious Diseases, Vita‐Salute San Raffaele University, IRCCS San Raffaele Scientific Institute, Milan, Italy

**Background**: Major cardiovascular events (MACEs) in people living with HIV (PLWH) may be related to the exposure to antiretroviral therapy (ART) and persistent inflammation, especially among those not achieving virological suppression [1–3]. The aim of the study was to evaluate the association between targeted variables and MACEs.

**Materials and methods**: This retrospective, observational, single‐centre study was conducted from January 2010 to April 2024 among PLWH receiving ART. Individuals were classified in three categories according to viraemia: virological suppression (<50 copies/ml), low‐level viraemia (divided into two subgroups based on HIV‐RNA 50–200 copies/ml and 200–1000 copies/ml) and virological non‐suppression (≥1000 copies/ml). Viraemia was considered as a time‐dependent variable and by cumulative years in each class since the start of follow‐up. A Cox proportional hazards model for recurrent events (Andersen−Gill model) was used to perform a multivariate time‐to‐event analysis, assessing the association between virological categories and MACEs.

**Results**: A total of 3349 PLWH were followed for a median time of 14 years (interquartile range [IQR] 11.2–14.2). At baseline, 2794 (83.4%) people were virologically suppressed, 189 (5.6%) and 90 (2.7%) presented 50–200 copies/ml and 200–1000 copies/ml, respectively, and 276 (8.2%) were non‐suppressed. During the follow‐up, virological suppression was documented at least once in 3295 (98.4%) cases, low‐level viraemia occurred in 1579 (47.1%) cases with 50–200 copies/ml and 794 (23.7%) with 200–1000 copies/ml and HIV‐RNA >1000 copies/ml was detected in 844 (25.2%) cases. Overall, 300 MACEs were recorded, including 53 (17.7%) repeated events, with a total incident rate of 0.00976 events per person‐year. Particularly, 247 (7.4%) individuals experienced at least one MACE, while 36 (1.1%) had recurrent events (Table 1). The risk of MACEs was significantly associated with previous MACEs (hazard ratio [HR] 3.385, *p* < 0.001), and viraemia >1000 copies/ml at baseline (HR 2.209, *p* = 0.039). Among targeted variables, the onset of MACEs was also significantly associated with greater age at baseline and years on ART, hypertension, diabetes, lower HDL and higher triglycerides (Figure 1).

**P291: Table 1:** Main characteristics at baseline and MACEs distribution

Demographics	Results
Males (*n*, %)	2532 (75.6%)
Age (median, IQR)	46.1 (41.8−50.8)
Time from HIV diagnosis (median, IQR)	14.2 (8.5−19.8)
Duration on ART (median, IQR)	11.6 (5.9−14.2)
CD4 copies/mL (median, IQR)	557 (394−734)
CD4 % (median, IQR)	26.3 (19.9−32.4)
CD4/CD8 ratio (median, IQR)	0.55 (0.37−0.78)
HDL mg/dl (median, IQR)	42 (35−52)
LDL mg/dl (median, IQR)	114 (91−137)
Triglycerides mg/dl (median, IQR)	129 (90−190)
Glucose mg/dl (median, IQR)	87 (81−96)
BP systolic mmHg (median, IQR)	120 (114−130)
BP diastolic mmHg (median, IQR)	80 (70−85)
Body mass index (median, IQR)	23.4 (21.3−25.8)

MACEs	Results
Myocardial infarction (*n*, %)	161 (53.7%)
Ischaemic strokes (*n*, %)	64 (21.3%)
Angina pectoris (*n*, %)	9 (3%)
Haemorrhagic strokes (*n*, %)	5 (1.7%)
Peripheral artery disease (*n*, %)	5 (1.7%)
Percutaneous coronary intervention (*n*, %)	39 (24%)
Coronary artery by‐pass graft (*n*, %)	8 (2.7%)
Dead (*n*, %)	23 (6.3%)

**P291: Figure 1:**
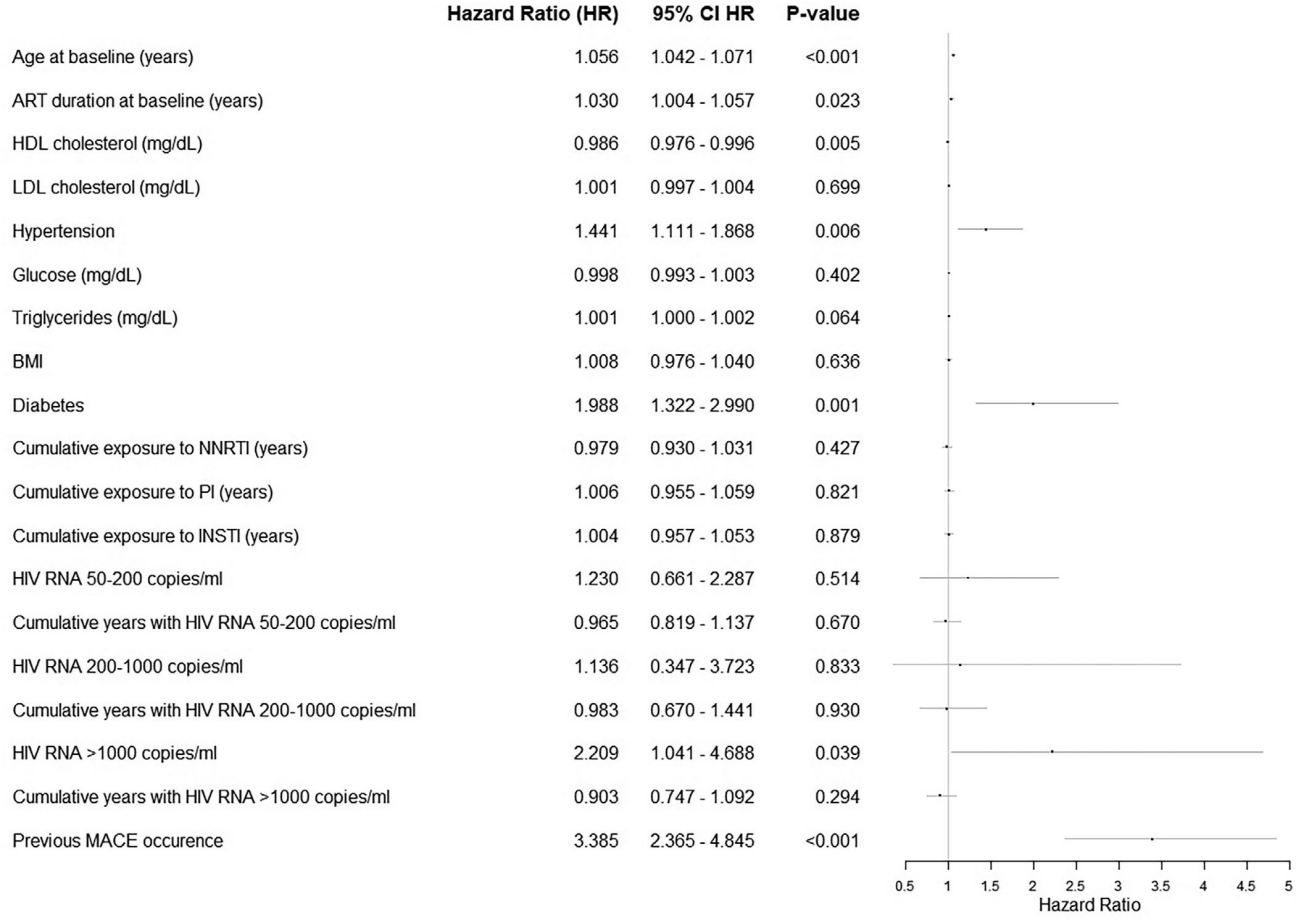
Forest plot of the targeted variables in the analysis (effect size per unit increase). ART, antiretroviral therapy; BMI, body mass index; BP, blood pressure; HDL, high density lipoprotein; INSTI, integrase strand transfer inhibitors; LDL, low density lipoprotein; NNRTI, non‐nucleoside reverse transcriptase inhibitors; PI, protease inhibitors.

**Conclusions**: Our study underlies the need of stringent cardiovascular monitoring and secondary prevention in PLWH on ART presenting previous MACEs and HIV‐RNA >1000 copies/ml, because of the high risk of developing new MACEs.

**References**

1. European AIDS Clinical Society. EACS Guidelines, version 12.0 [Internet]. 2023 [cited 2024 Jul 26]. Available from: https://www.eacsociety.org/media/guidelines‐12.0.pdf.

2. Grinspoon SK, Fitch KV, Zanni MV, Fichtenbaum CJ, Umbleja T, Aberg JA, et al. Pitavastatin to prevent cardiovascular disease in HIV infection. N Engl J Med. 2023;389(8):687‐99.

3. Elvstam O, Marrone G, Medstrand P, Treutiger CJ, Sönnerborg A, Gisslén M, et al. All‐cause mortality and serious non‐AIDS events in adults with low‐level human immunodeficiency virus viremia during combination antiretroviral therapy: results from a Swedish nationwide observational study. Clin Infect Dis. 2021;72(12):2079‐86.

### P292 Improved uric acid metabolism in virologically suppressed people with HIV‐1 switching to tenofovir DF‐containing, ainuovirine‐based compared to tenofovir alafenamide‐containing, boosted elvitegravir‐based antiretroviral regimen: the secondary analyses of 48‐week results of the SPRINT trial, a randomised, active‐controlled phase III study

Ping Ma^1^, Qingxia Zhao^2^, Hongxia Wei^3^, Hongzhou Lu^4^, Hui Wang^5^, Shenghua He^6^, Zhu Chen^7^, Yaokai Chen^8^, Min Wang^9^, Fujie Zhang^10^, Hao Wu^11^, Weiping Cai^12^, Wan Wan^13^, Heliang Fu^13^, Hong Qin ^13^

^1^Department of Infectious Diseases, Tianjin Second People's Hospital, Tianjin, China. ^2^Department of Infectious Diseases, The Sixth People's Hospital of Zhengzhou, Zhengzhou, China. ^3^Department of Infectious Diseases, The Second Hospital of Nanjing, Nanjing, China. ^4^National Clinical Research Centre for Infectious Diseases, Shenzhen Third People's Hospital, Shenzhen, China. ^5^Department of Infectious Diseases, Shenzhen Third People's Hospital, Shenzhen, China. ^6^Department of Infectious Diseases, Public Health Clinical Medical Center of Chengdu, Chengdu, China. ^7^Drug Clinical Trial Institution, Public Health Clinical Medical Center of Chengdu, Chengdu, China. ^8^Division of Infectious Diseases, Chongqing Public Health Medical Center, Chongqing, China. ^9^Institute of HIV/AIDS, The First Hospital of Changsha, Changsha, China. ^10^Clinical and Research Center for Infectious Diseases, Beijing Ditan Hospital Capital Medical University, Beijing, China. ^11^Clinical and Research Center for Infectious Diseases, Beijing Youan Hospital, Capital Medical University, Beijing, China. ^12^Infectious Disease Center, Guangzhou Eighth People's Hospital, Guangzhou Medical University, Guangzhou, China. ^13^Clinical Research & Development, Jiangsu Aidea Pharmaceutical Co., Ltd., Yangzhou, China

**Background**: ACC008, a novel antiretroviral fixed‐dose combination (FDC), contains ainuovirine (ANV), a new‐generation NNRTI, plus lamivudine and tenofovir DF (TDF). This FDC has shown noninferior virological efficacy but improved lipid profile compared to tenofovir alafenamide (TAF)‐containing, boosted elvitegravir (EVG/c) regimen among virologically suppressed people with HIV‐1 (PWHs) switching from NNRTI‐based regimen. We herein reported the secondary analyses of 48‐week glucose and uric acid metabolism as prespecified.

**Methods**: Virologically suppressed adult PWHs (*n* = 762) were randomized to ACC008 (*n* = 381) or comparator arm (*n* = 381) for 48 weeks. Changes from baseline (CFBs) in serum glucose and uric acid are primary safety outcomes of interest, including fasting glucose and uric acid, as prespecified. Other prespecified secondary safety outcomes of interest included glucose and uric acid metabolism adverse events (AEs) by MedDRA.

**Results**: Pre‐existing glucose or uric acid dysmetabolism conditions, including diabetes and gout, were uncommon (<5%) in the SPRINT study population. The two arms had a similar CFB in fasting serum glucose over 48‐week treatment (−0.25 ± 0.71 vs. −0.25 ± 0.72 mmol/l). However, PWHs on ACC008 showed significantly less CFB in serum uric acid compared to those on comparator at week 48 (least square mean ± standard error, −7.7 ± 3.1 vs. 49.8 ± 3.1 mmol/l, estimated treatment difference [95% CI], −57.5 [−66.1, −48.9], *p* < 0.001) (Figure 1). Glucose metabolism AEs were uncommon and comparable in the two arms. However, PWHs on ACC008 experienced significantly less frequent increased serum uric acid AEs (11.5% vs. 26.5%, −0.150 [0.094–0.204]), although hyperuricaemia or gout was occasionally reported in either arm. One PWH on ACC008 reported occurrence of gouty arthritis; a small proportion of PWHs reported use of anti‐gout medications (0.3% vs. 3.1%).

**P292: Figure 1:**
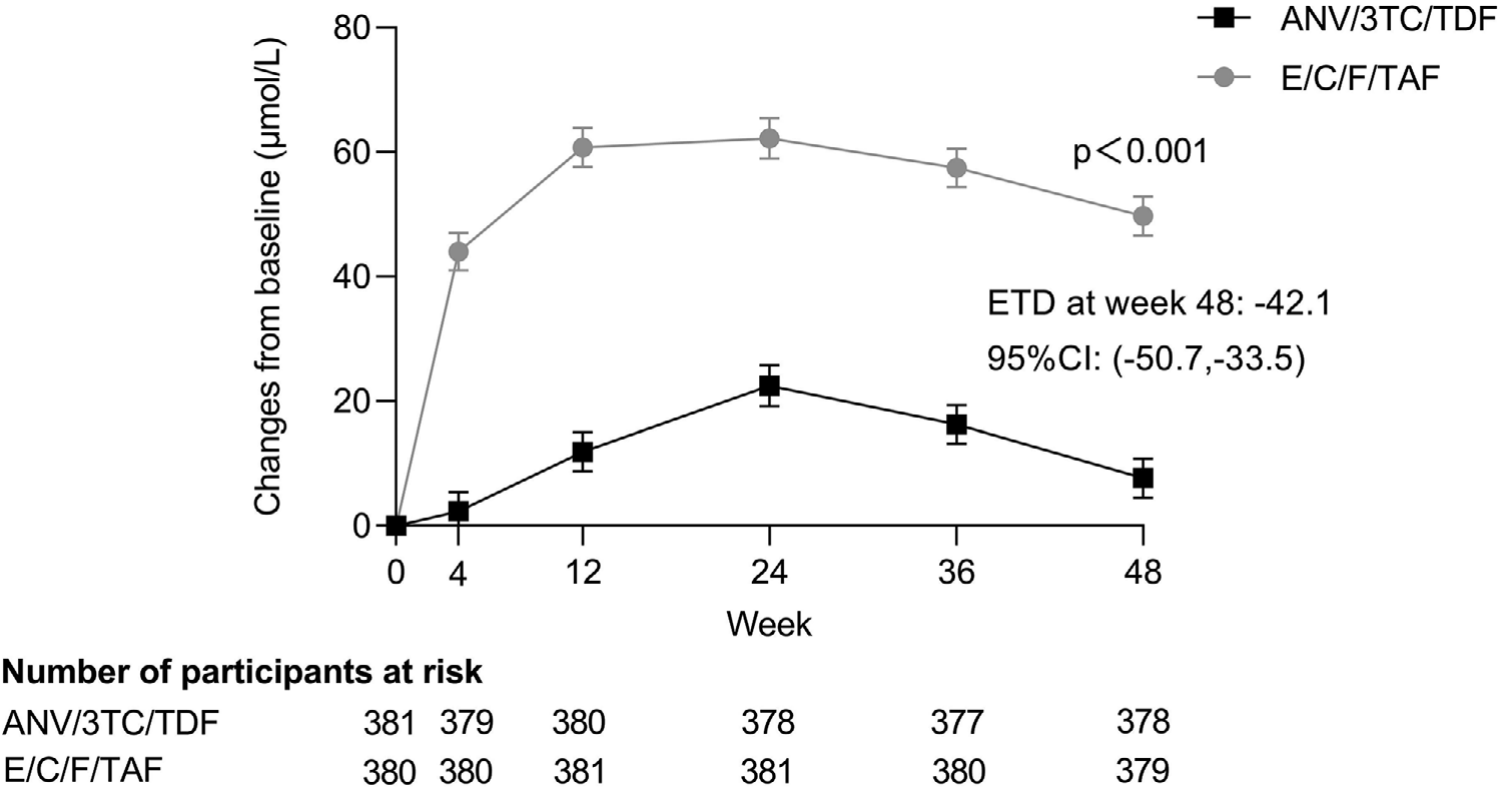
Mean changes from baseline in uric acid over 48 weeks. ANV/3TC/TDF, ainuovirine/lamivudine/tenofovir disoproxil; E/C/F/TAF, elvitegravir/cobicistat/emtricitabine/tenofovir alafenamide. Data are expressed in least square mean ± standard error.

**Conclusions**: Switching to tenofovir DF‐containing, ANV‐based regimen resulted in constant glucose but improved uric acid metabolism in virologically suppressed PWHs compared to that to TAF‐containing, boosted EVG‐based regimen. Glucose or uric acid dysmetabolism should be well addressed although diabetes or gout occasionally emerges in virologically suppressed PWHs.

### P293 Cytomegalovirus antibodies and coronary artery disease in people with HIV

Moises Alberto Suarez‐Zdunek ^1^, Andreas Dehlbæk Knudsen^1^, Andreas Fuchs^2^, Nikolai Søren Kirkby^3^, Thomas Benfield^4^, Jan Gerstoft^1^, Marius Trøseid^5^, Lars Valeur Køber^2^, Klaus Fuglsang Kofoed^2^, Susanne Dam Nielsen^1^

^1^Department of Infectious Diseases, Copenhagen University Hospital—Rigshospitalet, Copenhagen, Denmark. ^2^Department of Cardiology, Copenhagen University Hospital—Rigshospitalet, Copenhagen, Denmark. ^3^Department of Clinical Microbiology, Copenhagen University Hospital—Rigshospitalet, Copenhagen, Denmark. ^4^Department of Infectious Diseases, Copenhagen University Hospital—Amager and Hvidovre, Hvidovre, Denmark. ^5^Rikshospitalet, Research Institute of Internal Medicine, Oslo University Hospital, Oslo, Norway

**Background**: People with HIV (PWH) have twice the risk of cardiovascular disease, including coronary artery disease (CAD), compared to the general population. Cytomegalovirus (CMV) infection is associated with cardiovascular disease in the background population and PWH, possibly due to increased inflammation. Co‐infection with CMV is very common in PWH, but it is not known if CMV is associated with CAD in PWH who are not selected on a priori cardiovascular risk.

**Materials and methods**: From the Copenhagen Comorbidity in HIV Infection (COCOMO) study, we included PWH with measurements of CMV IgG and who underwent coronary CT angiography (CCTA). On CCTAs, we measured the maximum stenosis grade in any coronary vessel and the total plaque volume. We defined any atherosclerosis as a maximum stenosis ≥1% and obstructive CAD as a maximum stenosis ≥50%. Using linear and logistic regressions adjusted for age, sex, smoking, diabetes, dyslipidaemia and current CD4+ T cell count, we investigated if positive CMV serostatus and CMV IgG concentrations were associated with any atherosclerosis, obstructive CAD or total plaque volume. We also investigated if inflammation mediated the association.

**Results**: We included 620 PWH, of whom 95% had positive CMV serostatus, 45% had any atherosclerosis and 12% had obstructive CAD (Table 1). Positive CMV serostatus was not associated with any atherosclerosis (*p* = 0.91), obstructive CAD (*p* = 0.77) or total plaque volume (*p* = 0.34). However, higher IgG concentrations were associated with any atherosclerosis (OR 1.21 [1.06–1.39] per doubling, *p* < 0.01) and obstructive CAD (OR 1.31 [95% CI 1.07–1.59] per doubling, *p* < 0.01). Furthermore, per doubling of CMV IgG concentrations, total plaque volume was 56% higher (95% CI 21–101%, *p* < 0.001), and 10% (95% CI 3–38%, *p* = 0.03) of this effect was mediated by higher IL‐6 concentrations. After adjustment for age, sex, smoking, diabetes, dyslipidaemia and current CD4 count, no associations between CMV antibodies and CAD remained significant.

**P293: Table 1:** Participant characteristics at baseline (*N* = 620)

Age, median years (IQR)	50.1 (42.8−57.1)
Male sex, *n* (%)	553 (89.2)
Hypertension, *n* (%)	256 (41.3)
Diabetes mellitus, *n* (%)	33 (3.9)
Current smoking, *n* (%)	177 (28.5)
Dyslipidaemia, *n* (%)	118 (19.0)
Current use of ART, *n* (%)	612 (98.7)
CD4 count <350 cells/µL, *n* (%)	40 (6.5)
CD4 nadir <200 cells/µL, *n* (%)	235 (37.9)
HIV RNA ≥50 copies/mL, *n* (%)	29 (4.7)
Ever AIDS‐defining condition, *n* (%)	109 (17.6)

Abbreviations: ART, antiretroviral therapy; IQR, interquartile range.

**Conclusions**: We found a high seroprevalence of CMV in PWH. Higher CMV IgG concentrations were associated with CAD. However, this association was not significant after adjustment for traditional cardiovascular risk factors. This indicates that the association, in part, is explained by confounders.

### P294 N6‐methyladenine DNA and ABCA1 methylation association with coronary atherosclerosis in asymptomatic people with HIV with low‐intermediate cardiovascular risk

Jose I Bernardino ^1^, Regina Dalmau^2^, Diego Rodriguez‐Torres^3^, Gabriel Saiz‐Medrano^4^, María Fernandez‐Velilla^2^, Inmaculada Pinilla^2^, Javier Rodriguez‐Centeno^4^, Andres Esteban‐Cantos^4^, Tatiana Mata^5^, Carmen Ramos^3^, Berta Rodes^4^, Miriam Estebanez^5^

^1^HIV & Infectious Diseases Section, Hospital Universitario La Paz‐Carlos III, IdiPAZ, Madrid, Spain. ^2^Cardiology Image Unit, Hospital Universitario La Paz‐Carlos III, IdiPAZ, Madrid, Spain. ^3^Cardiology Image Unit, Hospital Universitario Central de la Defensa Gomez Ulla, Madrid, Spain. ^4^AIDS & Infectious Diseases Research Group, Hospital Universitario La Paz‐Carlos III, IdiPAZ, Madrid, Spain. ^5^Infectious Diseases Section, Hospital Universitario Central de la Defensa Gomez Ulla, Madrid, Spain

**Background**: Coronary artery disease (CAD) is more prevalent in low‐intermediate cardiovascular risk asymptomatic HIV individuals than in HIV‐negative individuals and is similar to cardiovascular high‐risk HIV‐negative individuals. Apolipoprotein A1 plays a key role in the reverse cholesterol transport, and this process is impaired in PWH. ABCA1 methylation and N6‐methyladenine DNA have been identified as epigenetic markers in coronary artery disease.

**Material and methods**: This was a cross‐sectional exploratory study to analyze whether N6‐adenine DNA methylation and ABCA1 proximal promoter methylation are associated with coronary atherosclerosis in asymptomatic HIV individuals without cardiovascular disease and low‐intermediate cardiovascular risk (SCORE‐2). Coronary atherosclerosis was defined as coronary plaque in at least one coronary segment on coronary CT angiography. Analyses of ABCA1 promoter methylation were performed in DNA from monocytes by NGS. Analyses of N6‐methyladenine DNA levels were performed in leukocyte DNA by ELISA.

**Results**: The study included 27 individuals with a median (IQR) age 57 years (52–64), 81.5% male, 85% Caucasian, CD4+ T cell count 659 cells/mm^3^ (480–794), CD4:CD8 ratio 0.76 (0.52–1.48) and 100% HIV‐RNA <50 cp/ml. Cardiovascular risk factors included hypertension (59.3%), dyslipidaemia (85%), diabetes (33%) and active smoking (26%). The median cardiovascular risk SCORE‐2 was 6% (4.05–8.80) (Table 1). Fifteen (55%) participants had coronary atherosclerosis. Mean (SD) CAC score (Agatston) was 188.5 (168.2), segment involvement scores 2.87 (1.85), segment involvement score 2.87 (1.85) and Leaman score 6.80 (2.68). Vulnerable plaque features were present in 60% of subjects. We found a moderate correlation between ABCA1 methylation and the Leaman score (Spearman rho = 0.37) and N6‐methyladenine DNA and Segment severity score (Spearman rho = 0.33). In the multivariate analysis, methylation in 6 positions in the ABCA1 gene was independently associated with coronary artery atherosclerosis (OR 1.06 [95% CI 1.01–1.12]; *p* = 0.046).

**P294: Table 1:** Characteristics of participants

	Overall *N* = 27	Without CAD *N* = 12	CAD *N* = 15	*p*‐value
Age, years	57 (52−64)	56 (52−61)	58 (56−65)	0.31
Male	22 (81.5)	11 (91.7)	11 (73.3)	0.47
Caucasian	23 (85.2)	11 (91.7)	12 (80)	0.59
CD4+ T cell/mm^3^	659 (480−794)	496 (429−828)	750 (575−794)	0.33
CD4:CD8 ratio	0.76 (0.52−1.48)	0.6 (0.5−1.39)	0.77 (0.53−1.42)	0.69
HIV RNA <50 cp/ml	27 (100)	12 (100)	15 (100)	1
ART duration, years	8 (5.6−18.9)	7 (4.7−10.9)	8.6 (7−20.7)	0.33
Cumulative exposure PI, months	0 (0−20)	0 (0−0)	3 (0−54)	0.09
Cumulative exposure ABC, months	0 (0−45)	0 (0−49.5)	0 (0−37.5)	0.97
Active smoking	7 (25.9)	3 (25)	4 (26.7)	0.25
Diabetes	9 (33.3)	2 (16.7)	7 (46.7)	0.29
Hypertension	16 (59.3)	6 (50)	10 (66.7)	0.63
Dyslipidaemia	23 (85.2)	8 (66.7)	15 (100)	0.06
BMI kg/m^2^	25.9 (23.5−27.3)	25.9 (24.6−27.5)	25.5 (23.4−26.7)	0.35
Cardiovascular risk SCORE‐2	6 (4.05−8.80)	5.5 (3.33−7.53)	6.75 (5.2−10.7)	0.14
Statin use, yes	20 (74)	6 (50)	14 (93)	0.03

Median (IQR) for quantitative variables, *n* (%) for qualitative variables.

Abbreviations: CAC, coronary artery calcium; ELISA, enzyme‐linked immunosorbent assay; NGS, next‐generation sequencing.

**Conclusion**: In this small exploratory study, ABCA1 methylation was independently associated with coronary artery disease in PWH. Further research is required to explore the role of epigenetic biomarkers in coronary artery disease.

### P295 Metabolic syndrome among people living with HIV (PLWH) in a specialist HIV clinic in London

Yomna Gharib, Irfaan Maan, Manik Kohli, Andrew Copas, Emmi Suonpera, Fowsiya Nur, Anya MacLaren, James Mason, Laura Waters, Richard Gilson, Sarah Pet, Alejandro Arenas‐Pinto

UCL Institute for Global Health, Mortimer Market Centre, Central and North West London NHS Foundation Trust, London, UK

**Background**: Metabolic abnormalities, including diabetes (DM), dyslipidaemia, hypertension (HTN) and obesity, are increasingly described among PLWH [1]. Some antiretroviral therapy (ART), including integrase strand‐transfer inhibitors (INSTI) and tenofovir alafenamide (TAF), is implicated in worsening of some of these parameters; in contrast, tenofovir disoproxil fumarate (TDF) seems protective against dyslipidaemia and abnormal weight gain [2,3]. We aimed to describe the prevalence of metabolic abnormalities and metabolic syndrome (MS) using the American Heart Association/National Heart Lung and Blood Institute definition in people attending our HIV clinic.

**Methods**: Cross‐sectional analysis was performed on 5372 people from 2017 to 2019 to capture the pre‐COVID‐19 impact of metabolic abnormalities and MS and to exclude the pandemic's impact on the availability of data. Metabolic abnormalities defined as having confirmed diagnosis and/or treatment of the following conditions: DM (HbA1c >48 mmol/mol), HTN (systolic BP ≥130 mmHg), dyslipidaemia (triglyceride >1.7mmol/L and/or low HDL (men <1.0 mmol/L; women <1.3), obesity (BMI ≥30 kg/m^2^). MS was defined as having ≥3 of the following: HTN, DM, hypertriglyceridaemia, low HDL and obesity. Chi‐square test and logistic regression explored association between MS with demographics and ART using 95% CI, OR and *p*‐value.

**Results**: Dyslipidaemia was highly prevalent (53%), followed by HTN (45%), obesity (16%) and DM (8%); 11% met criteria for MS. MS was more prevalent among older age and black ethnicity (15% vs 12.6% respectively; *p* < 0.05) (Table 1). Protease inhibitor (PI) use and TAF were associated with higher odds of MS (OR 1.6 and OR 1.89, respectively; *p* < 0.001), whereas TDF was protective (OR 0.73, 95% CI 0.61–0.87; *p* < 0.001). INSTI use was not associated with MS (OR 1.1, *p* = 0.276). After adjusting for confounders, age, use of PI, TAF and INSTI remained a significant predictor of MS (AOR 2.7, *p* < 0.001; AOR 1.6, *p* < 0.001; AOR 1.53, *p* < 0.001; AOR 1.33, *p* = 0.003 respectively), while sex, ethnicity, alcohol intake, smoking and being on TDF were not associated with MS (*p* > 0.05).

**P295: Table 1:** Demographics, HIV parameters, ART association with metabolic syndrome in this population of people living with HIV (PLWH)

	No metabolic syndrome, *N* (%)	Metabolic syndrome, *N* (%)	Chi^2^ *p*‐value
Age: <50, >50	2366 (94.15%), 2430 (84.99%)	147 (5.85%), 429 (15.01%)	<0.001
Gender: female, male	829 (88%), 3960 (89.53%)	113 (12%), 463 (10.47%)	0.169
Ethnicity: White, Black, others	2842 (89.17%), 1031 (87.37%), 923 (91.84%)	345 (10.83%), 149 (12.63%), 82 (8.16%)	0.003
Viral load: <50 copies/mL, >50 copies/mL	4435 (89%), 296 (91.36%)	548 (11%), 28 (8.64%)	0.187
CD4 count: <200 cells/mm^3^, 200 cells/mm^3^	177 (92.19%), 4502 (89.04%)	15 (7.81%), 554 (10.96%)	0.169
Alcohol intake: yes, no	3360 (89.91%), 1020 (87.86%)	377 (10.09%), 141 (12.14%)	0.047
Alcohol units/week: 0 units, 1‐14 units, >14 units	1020 (87.86%), 2742 (89.96%), 504 (90%)	141 (12.14%), 306 (10.04%), 56 (10%)	0.125
Smoking: ex‐smoker, non‐smoker, smoker	862 (88.32%), 2343 (88.68%), 1359 (91.52%)	114 (11.68%), 299 (11.32%), 126 (8.48%)	0.008
PI: no, yes	3749 (90.45%), 1047 (85.33%)	396 (9.55%), 180 (14.67%)	<0.001
INSTI: no, yes	2737 (89.68%), 2059 (88.75%)	315 (10.32%), 261 (11.25%)	0.276
TAF: no, yes	4017 (90.51%), 4796 (83.4%)	421 (9.49%), 155 (16.60%)	<0.001
TDF: no, yes	2522 (87.87%), 2274 (90.89%)	348 (12.13%), 228 (9.11%)	<0.001

Abbreviations: INSTI, integrase strand‐transfer inhibitors; PI, protease inhibitors; TAF, tenofovir alafenamide; TDF, tenofovir disoproxil fumarate.

**Conclusion**: Our study shows the complex metabolic profile of PLWH on ART. Future research should investigate the pathogenesis of MS, and ways to optimise the prevention and treatment of MS in PLWH.

**References**

1. Pao V, Lee GA, Grunfeld C. HIV therapy, metabolic syndrome, and cardiovascular risk. Curr Atheroscler Rep. 2008;10(1):61‐70.

2. Summers NA, Lahiri CD, Angert CD, Aldredge A, Mehta CC, Ofotokun I, et al. Metabolic changes associated with the use of integrase strand transfer inhibitors among virally controlled women. J Acquir Immune Defic Syndr. 2020;85(3):355‐62.

3. Surial B, Mugglin C, Calmy A, Cavassini M, Günthard HF, Stöckleet M, et al. Weight and metabolic changes after switching from tenofovir disoproxil fumarate to tenofovir alafenamide in people living with HIV: a cohort study. Ann Intern Med. 2021;174(6):758‐67.

### P296 Prevalence of metabolic syndrome in people living with HIV and its relationship with fatty liver and cardiovascular risk

Adrian Rodriguez ^1^, Ignasi Merino^2^, Aroa Villoslada^2^, Patricia Sorni^2^, Marta Molero^2^, Araceli Serrano^2^, Andrea Salom^2^, Francisco Homar^2^, Antoni Payeras^2^

^1^Infectious Disease, IDISBA—Hospital Universitari Son Llatzer, Palma, Spain. ^2^Internal Medicine—Infectious Disease, Hospital Universitari Son Llatzer, Palma, Spain

**Background**: Metabolic syndrome (MS) is a set of medical conditions that, when present in a person, increase the risk of developing certain pathologies, such as fatty liver disease (FLD) or cardiovascular diseases (CVD). Some studies indicate that people living with HIV (PLWH) have a higher risk of MS, and by extension, higher risk of CVD and FLD. The aim of this study is to assess the prevalence of MS, using three different methods, in our cohort of PLWH, and to study its relationship with FLD and CVD.

**Methods**: MS was defined as having three or more of these five criteria: waist circumference (WC) >102 cm (men) or 88 cm (women); treated hypertension or systolic blood pressure (SBP) >130 or diastolic blood pressure (DBP) >85; treated type 2 diabetes mellitus or fasting glucose >100 mg/dl; treated dyslipidaemia or HDL <50 mg/dl (men) or <45 mg/dl (women); treated hypertriglyceridaemia or triglyceride levels (TG) >150 mg/dl. Additionally, two new classifications were made: MS with altered WC; MS with overweight/obesity (BMI >25 kg/m^2^). The prevalence of MS was calculated, and in each group, the hepatic steatosis index (HSI), the percentage of patients with HSI >36, and the Framingham and Regicor cardiovascular risk scores were compared. Furthermore, we also compared the available controlled attenuation parameter (CAP) measurements, measured with a Fibroscan^®^.

**Results**: Out of 1096 PLWH in our cohort, there were 751 measurements of WC. Of these, 153 were women (20.4%). Figure 1 shows the prevalence of MS using the three different methods. The overall percentage of MS, using the usual criteria, was 41.9%. Table 1 shows that patients with MS have, by all three methods, higher HSI values, a higher percentage of people with HSI >36, higher CAP values, and higher cardiovascular risk scores (*p* < 0.001 in all cases). MS with altered WC or with overweight/obesity had a higher difference in CAP values and detected a higher percentage of people with HSI >36 than the usual MS criteria.

**P296: Figure 1:**
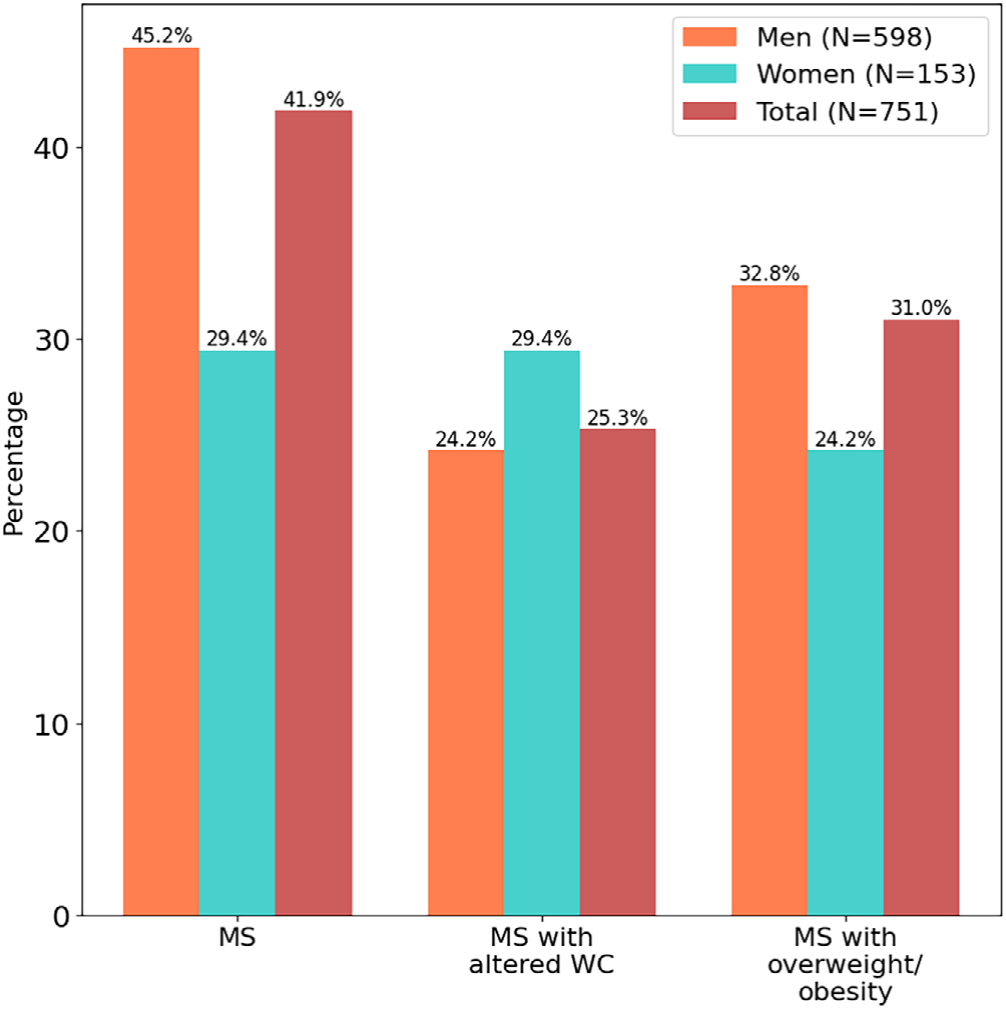
Prevalence of MS using three different methods, according to gender.

**P296: Table 1:** FLI, CAP, Framingham and Regicor according to MS. Results are expressed as *N* (%) or median [p25–p75]

		FLI	FLI >36	CAP (dB/m) (*N* = 55)	Framinghan	Regicor
	Yes (*N* = 315)	37 [34−41]	207 (66%)	280 [239−314]	12 [8−19]	4 [3−7]
MS	No (*N* = 436)	35 [31−38]	65 (15%)	211 [200−237]	6 [3−10]	2 [1−4]
	*p*‐value	<0.001	<0.001	<0.001	<0.001	<0.001
	Yes (*N* = 190)	39 [37−43]	159 (84%)	296 [270−340]	12 [8−18]	4 [3−7]
MS with altered WC	No (*N* = 561)	34 [31−37]	113 (20%)	214 [203−237]	8 [4−12]	3 [1−4]
	*p*‐value	<0.001	<0.001	<0.001	<0.001	<0.001
	Yes (*N* = 233)	39 [36−42]	201 (86%)	289 [254−332]	12 [8−19]	4 [3−7]
MS with overweight/obesity	No (*N* = 518)	34 [31−37]	71 (14%)	214 [204−237]	7 [4−12]	3 [1−4]
	*p*‐value	<0.001	<0.001	<0.001	<0.001	<0.001

Abbreviations: CAP, controlled attenuation parameter; FLI, fatty liver index.

**Conclusions**: Our cohort of PLWH has a very high prevalence of MS. MS is associated with higher rates of FLD and CVD. The use of MS with altered WC or with overweight/obesity may be a good alternative in PLWH.

### P297 Cardiovascular disease risk according to SCORE2 and the potential need for cholesterol and blood pressure lowering therapy in persons with HIV without established cardiovascular disease, Croatia

Josip Begovac, Vanja Romih Pintar, Sanja Belak Škugor, Ivana Benković, Ivana Javorić, Sime Zekan

HIV/AIDS Department, University Hospital for Infectious Diseases, Zagreb, Croatia

**Background**: The European Society for Cardiology (ESC) and European AIDS Clinical Guidelines (EACS) recommend assessing cardiovascular disease (CVD) risk using the SCORE2 algorithm [1,2]. The SCORE2 is applied to CVD‐free, apparently healthy persons. We examined the distribution of CVD risk in people living with HIV (PLWH) from 40 to 69 years old and assessed the proportions of those who could be eligible and are using lipid‐lowering therapy (LLT) according to the ESC and 2024 interim EACS guidelines.

**Method**: Included were all PLWH seen at the Outpatient HIV Department in the period 2019 to 2023 who were of European origin and had available data for the SCORE2 calculation. We excluded individuals with previously known atherosclerotic CVD, diabetes mellitus, or chronic kidney disease with eGFR <60 mL/min/1.73 m^2^, and pregnant women. We used the SCORE2 algorithm for countries with a high CVD risk and categorised study participants based on the ESC and EACS criteria [1,2].

**Results**: Of 1116 PLWH of European origin, 1000 had available data for SCORE2 determination. Two hundred and nine of 1000 (20.9%) were not eligible for the SCORE2 calculations: 56 had a past CVD event, 130 had diabetes and 23 had an Estimated Glomerular Filtration Rate (eGFR) <60 mL/min/1.73 m^2^. The remaining 791 persons had a median age of 46.3 (Q1–Q3, 41.7–52.6) years, 658 (83.2%) had an HIV‐1 RNA <50 c/ml and 694 (87.7%) had <200 c/ml. The median 10‐year risk of CVD according to SCORE2 was 3.9 (Q1–Q3, 2.4–6.1), and 292 (36.9%) were classified as low‐moderate risk, 427 (54.0%) as high risk and 72 (9.1%) as very‐high risk. Two hundred and seventy‐nine (35.3%) had a SCORE ≥5% a level at which EACS guidelines indicate or recommend LLT (Figure 1) [2]. All of those with a high and very high risk had LDL levels above optimal thresholds, and 34/791 (4.3%) had below 2.6 mmol/L. The proportion of individuals with blood pressure ≥140/90 mmHg was 16.4%. The use of blood pressure‐lowering therapy was 12.7% for those with blood pressure <140/80 mmHg and 35.4% for those ≥140/90 mmHg.

**P297: Figure 1:**
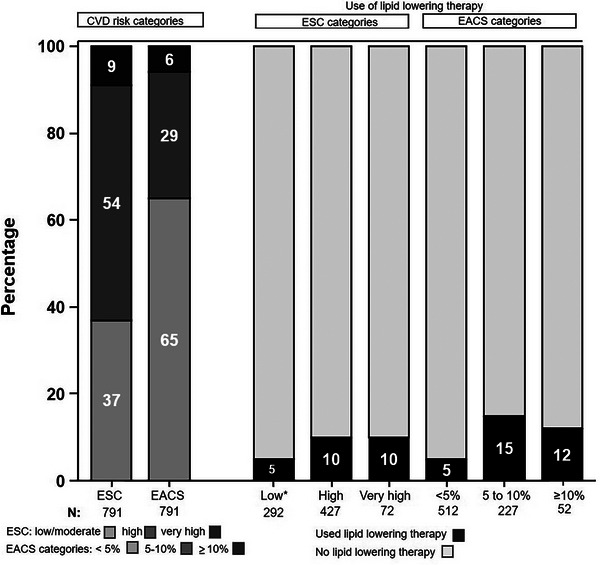
Distribution of SCORE2 risk categories according to ESC and EACS, and proportion receiving lipid lowering therapy (LLT) in PLWH in Croatia. ESC guidelines generally do not recommend LLT to persons at low/moderate risk, treatment should be considered for those with a high risk and is recommended for those at very high risk. The 2024 interim EACS guidelines indicate LLT for those with a SCORE2 ≥10%, recommend for those with 5 to 10% and consider for those <5%. *Indicates low and moderate risk. EACS, European AIDS Clinical Society; ESC, European Society for Cardiology.

**Conclusions**: A little more than one‐third of PLWH had indicated or recommended LLT by EACS guidelines. LLT and antihypertensive therapy were largely underused in PLWH in Croatia.

**References**

1. Visseren FLJ, Mach F, Smulders YM, Carballo D, Koskinas KC, Bäck M, et al. 2021 ESC Guidelines on cardiovascular disease prevention in clinical practice. Eur Heart J. 2021;42:3227‐337.

2. European AIDS Clinical Society. Interim guidance on the use of statin therapy for the primary prevention of cardiovascular disease in people with HIV [Internet]. 2024 [cited 2024 Jun 30]. Available from: https://www.eacsociety.org/guidelines/interim‐guidance/.

### P298 Online education yields significant gains in physicians' knowledge of the complexities of cardiometabolic disease in people living with HIV

Shanthi Voorn ^1^, Alessia Piazza^1^, Gill Adair^1^, Patrick Mallon^2^, Carlos Diego Malvestutto^3^, Esteban Martinez^4^

^1^Global Education, Medscape, London, UK. ^2^Centre for Experimental Pathogen Host Research, University College Dublin, Dublin, Ireland. ^3^Division of Infectious Diseases, Ohio State University Medical Center, Columbus, OH, USA. ^4^Infectious Diseases Unit, Hospital Clínic Barcelona, Barcelona, Spain

**Background**: As people living with HIV reach older age, they can be at the intersection of multiple comorbidities such as HIV infection cardiometabolic disease. Staying up‐to‐date with the latest research and accessing the ever‐growing field of knowledge is time‐consuming. Online education can make these clinician's tasks more efficient. We developed an online Continuing Medical Education (CME) activity titled: “Complexities of Cardiometabolic Disease in People Living With HIV”. The goal was to assess whether this online CME activity improves physicians’ knowledge of the influence of cardiometabolic conditions in people living with HIV.

**Methods**: Infectious disease (ID)/HIV specialists and primary care physicians participated in an online CME activity (https://www.medscape.org/viewarticle/999145) consisting of a 30‐minute video discussion between three experts with accompanying slides. Educational effect was assessed using a four‐question repeated pairs, pre‐/post‐assessment. A paired samples *t*‐test was conducted for significance testing on overall average number of correct responses and for confidence rating, and a McNemar's test was conducted at the question level (5% significance level). Cohen's *d* for paired samples estimated the effect size of the education (<0.20 modest, 0.20–0.49 small, 0.59–0.79 moderate, ≥0.80 large). The CME activity launched on 12/11/2023, and the data were collected through 3/18/2024.

**Results**: A total of 895 ID/HIV specialists and 3748 primary care physicians (PCPs) learners participated in the activity, of whom 34 and 132 completed the pre‐ and post‐activity questions, respectively. Overall 55% of ID/HIV specialists, and 47% of PCPs improved their knowledge of the factors that influence cardiometabolic conditions in people living with HIV (*p* < 0.001) indicating a considerable effect of the education (Cohen's *d* = 0.87; *d* = 0.81). The average percentage of correct responses rose from 30% to 55% for ID/HIV specialists; and 22% to 47% for PCPs pre‐activity to post‐activity. Almost half of physicians had a measurable improvement in confidence in their ability to manage multiple cardiometabolic disease in their HIV positive patients.

**Conclusions**: This online CME activity significantly improved knowledge of cardiometabolic disease in people living with HIV among ID/HIV specialists and PCPs. However, up to 75% of physicians still provided incorrect answers post‐education, indicating a need for further educational programmes to address this gap.

### P299 Carotid intima‐media thickness, dyslipidaemia, high cardiovascular risk and real‐life prescriptions of statins among people living with HIV enrolled in Archi Prevaleat cohort

Benedetto Maurizio Celesia ^1^, Salvatore Martini^2^, Elena Delfina Ricci^3^, Sergio Ferrara^4^, Laura Galli^5^, Addolorata Masiello^6^, Alessandra Tartaglia^7^, Michele Salvatore Paternò Raddusa^1^, Rosa Basile^8^, Antonella Castagna^5^, Paolo Maggi^9^

^1^Department of Clinical and Experimental Medicine, University of Catania, Unit of Infectious Diseases, ARNAS Garibaldi Catania, Catania, Italy. ^2^Department of Mental Health and Public Medicine, Section of Infectious Diseases, University of Campania, Luigi Vanvitelli, Napoli, Italy. ^3^Fondazione ASIA Onlus, Milan, Italy. ^4^Department of Medical and Surgical Sciences, Section of Infectious Diseases, University of Studies of Foggia, Foggia, Italy. ^5^Clinic of Infectious Diseases, Vita‐Salute San Raffaele University, San Raffaele Scientific Institute, Milan, Italy. ^6^Section of Infectious Diseases, AORN Sant'Anna e San Sebastiano of Caserta, Caserta, Italy. ^7^Unit of Infectious Diseases, Azienda Ospedaliera di Foggia, Foggia, Italy. ^8^Section of Infectious Diseases, Grande Ospedale Metropolitano, Bianchi Melacrino Morelli Reggio Calabria, Reggio Calabria, Italy. ^9^Department of Mental Health and Public Medicine, Section of Infectious Diseases, University of Campania, Luigi Vanvitelli, Caserta, Italy

**Background**: Preventive cardiovascular diseases (CVD) strategies are a growing concern in the era of the effective ARV treatment and ageing. In accord with EACS guidelines “statins should be used by all those with established vascular disease and in persons who are not at LDL‐c goals”. Archi Prevaleat is a multicentre, nationwide, prospective cohort aimed to evaluate the prevalence of carotid intima‐media thickness (IMT) in people living with HIV (PLWH). Aim of the study was to evaluate the concordance between guidelines indications and real‐life prescriptions of statins in this cohort.

**Materials and methods**: Cross sectional study: sex, age, BMI, clinical stage, use of statins and fasting total (TC), LDL and HDL cholesterol were recorded when a Doppler scan of the supra‐aortic vessels was performed. The 10‐year coronary heart disease risk was evaluated with ACC‐AHA score; a carotid IMT >1.2 mm or a plaque were considered for the study.

**Results**: Data of 1457 PLWH were analysed; 1170 (80.3%) male, median age 52 (IQR 45.9–58) years, median time from HIV diagnosis 17.8 (IQR 6.5–22.3) years, 598 (41%) had a previous diagnosis of dyslipidaemia. One hundred and forty‐seven (9.6%) had BMI >30. Median TC was 187 (IQR 160–214) mg/dL, 556 (38.1%) had a value >200 mg/dl; median LDLc was 115 (IQR 95–142) mg/dl, 412 (35.5%) had a value >130 mg/dl. Three hundred and seventy‐six had a CVD risk score >10. Any thickness or plaques were detected in 553 (36.6%) subjects. Four hundred and sixteen (31.5%) were on statins: respectively, 68% with previous dyslipidaemia, 24.6% with a BMI >30, 28% with TC >200 mg/dL, 28% with LDLc >130 mg/dL. 11.5% with high CVD risk had LDLc <70 mg/ml. Two hundred and eleven out of 376 with high CVD risk showed any IMT or plaque; 87 (41.7%) of them were on statins. One hundred and eighty‐nine with high CVD risk showed any IMT or plaque associated with LDLc value >70 mg/dl. Seventy‐four (37%) of them were on statin.

**Conclusions**: Only 42.3% of PLWH with statin indication were on treatment. A preventive action of information, aimed to PLWH refusing or delaying prescription of statins and a more stringent reconciliation between indications and real life prescriptions is increasingly desirable in all our clinical setting.

### P300 Association between high sensitivity troponin and NT‐pro brain natriuretic peptide and peripheral arterial disease in people living with HIV

Thomas Holtveg ^1^, Anne Marie Reimer Jensen^1^, Ask Bock^1^, Moises Alberto Suarez‐Zdunek^1^, Andreas Dehlbæk Knudsen^1^, Børge Nordestgaard^2^, Shoaib Afzal^2^, Thomas Lars Benfield^3^, Sisse Rye Ostrowski^4^, Tor Biering‐Sørensen^5^, Ruth Frikke‐Schmidt^6^, Susanne Dam Nielsen^1^

^1^Department of Infectious Diseases, Copenhagen University Hospital—Rigshospitalet, Copenhagen, Denmark. ^2^Department of Clinical Biochemistry, Copenhagen University Hospital—Herlev and Gentofte, Herlev, Denmark. ^3^Department of Infectious Diseases, Copenhagen University Hospital—Amager and Hvidovre, Hvidovre, Denmark. ^4^Department of Clinical Immunology, Copenhagen University Hospital—Rigshospitalet, Copenhagen, Denmark. ^5^Department of Cardiology, Copenhagen University Hospital—Herlev and Gentofte, Herlev, Denmark. ^6^Department of Clinical Biochemistry, Copenhagen University Hospital—Rigshospitalet, Copenhagen, Denmark

**Background**: People living with HIV (PWH) have high risk of peripheral artery disease (PAD). High sensitivity troponin (hsTnT) and NT‐pro B‐type natriuretic peptide (NT‐proBNP) are associated with PAD in the general population and could be useful screening tools for PAD in PWH. We aimed to assess the association between hsTnT and NT‐proBNP and both prevalent PAD and de novo PAD among PWH.

**Materials and methods**: PWH ≥18 years were examined at baseline (March 2015–November 2016) and after 2 years (April 2017–April 2019). Inclusion criteria were: (1) measurements of hsTnT and NT‐proBNP at baseline and (2) ankle brachial index (ABI) at baseline for prevalent PAD and at both visits for de novo PAD. PAD was defined as ABI ≤0.9. PWH with ABI ≥1.4 were excluded. Cross‐sectional analyses for prevalent PAD were performed in a base‐model with adjustments for age and sex and in an adjusted model further adjusting for smoking, hypertension, and diabetes. For de novo PAD due to low number of cases a completely unadjusted model was employed and an adjusted model adjusting for age, sex, and diabetes.

**Results**: Of 1011 PWH included, 88 (8.7%) had PAD at baseline (Table 1). Among 802 PWH with ABI measured at both visits and no PAD at baseline, 29 (3.6%) had de novo PAD at the 2‐year follow‐up. A doubling in hsTnT concentration was associated with prevalent PAD at baseline with an odds ratio of 1.41 (95% CI 1.02–1.96, p = 0.04) and 1.40 (95% CI 0.99–1.98, p = 0.05) in base model and adjusted model, respectively. High hsTnT was associated with a risk ratio of 3.39 (95% CI 1.24–9.27, p = 0.02) for de novo PAD in the unadjusted model and 3.44 (95% CI 0.98–12.10, p = 0.05) after adjustment (Figure 1). NT‐proBNP was not associated with prevalent PAD and was only associated with de novo PAD in the unadjusted model.

**P300: Table 1:** Baseline characteristics according to PAD‐status

Variable	Total (*n* = 1011)	No PAD at baseline (*n* = 923)	PAD at baseline (*n* = 88)
Age (years), mean ± SD	50.4 ± 11.2	49.9 ± 10.9	55.2 ± 13
Male, *n* (%)	862 (85)	787 (85)	75 (85)
Ethnicity			
Caucasian, *n* (%)	868 (88)	792 (88)	76 (91)
Other, *n* (%)	120 (12)	112 (12)	8 (10)
Smoking			
Never, *n* (%)	368 (36)	346 (38)	22 (25)
Current, *n* (%)	297 (29)	260 (28)	37 (42)
Previous, *n* (%)	346 (34)	317 (34)	29 (33)
Hypertension, *n* (%)	412 (44)	360 (42)	52 (61)
Diabetes, *n* (%)	42 (4)	38 (4)	4 (5)
BMI (kg/m^2^), mean ± SD	25 ± 3.9	25 ± 3.9	24.7 ± 3.8
Underweight, *n* (%)	26 (3)	23 (3)	3 (3)
Normal, *n* (%)	539 (54)	493 (54)	46 (52)
Overweight, *n* (%)	384 (35)	313 (34)	35 (40)
Obese, *n* (%)	94 (9)	90 (10)	4 (5)
Current CD4‐count (cells/µL), mean ± SD	718 ± 285	716 ± 288	740 ± 252
CD4 nadir <200 (cells/µL), n (%)	395 (40)	351 (39)	44 (51)
Viral load ≥50 (copies/mL), n (%)	53 (5)	48 (5)	5 (6)
Time with HIV (years), mean ± SD	14.4 ± 9.1	14.1 ± 9	18 ± 9.5
Use of ART, n (%)	996 (99)	910 (99)	86 (98)
NT‐proBNP (pmol/L), median [IQR]	5.2 [2.8‐9.0]	5.2 [2.8‐8.8]	5.3 [3‐12]
hsTnT (ng/L), median [IQR]	5.3 [3.8‐8.1]	5.2 [3.8‐7.9]	7.1 [4.7‐10.5]
LDL (mmol/L), mean ± SD	2.8 ± 0.9	2.8 ± 1	2.7 ± 0.9
hsCRP (mg/L), median [IQR]	1.2 [0.6‐2.4]	1.1 [0.6‐2.4]	1.5 [0.6‐2.7]
IL‐6 (pg/ml), median [IQR]	1.4 [0.9‐2.3]	1.4 [0.9‐2.2]	1.6 [1.1‐3.4]

Abbreviations: ART, antiretroviral therapy; BMI, body mass index; hsCRP, high sensitivity C‐reactive protein; hsTnT, high‐sensitivity troponin T; IDU, intravenous drug use; IL‐6, interleukin 6; INSTI, integrase strand transfer inhibitors; IQR, interquartile range; LDL, low density lipoprotein; NT‐proBNP, N‐terminal pro B‐type natriuretic peptide; SD, standard deviation.

**P300: Figure 1:**
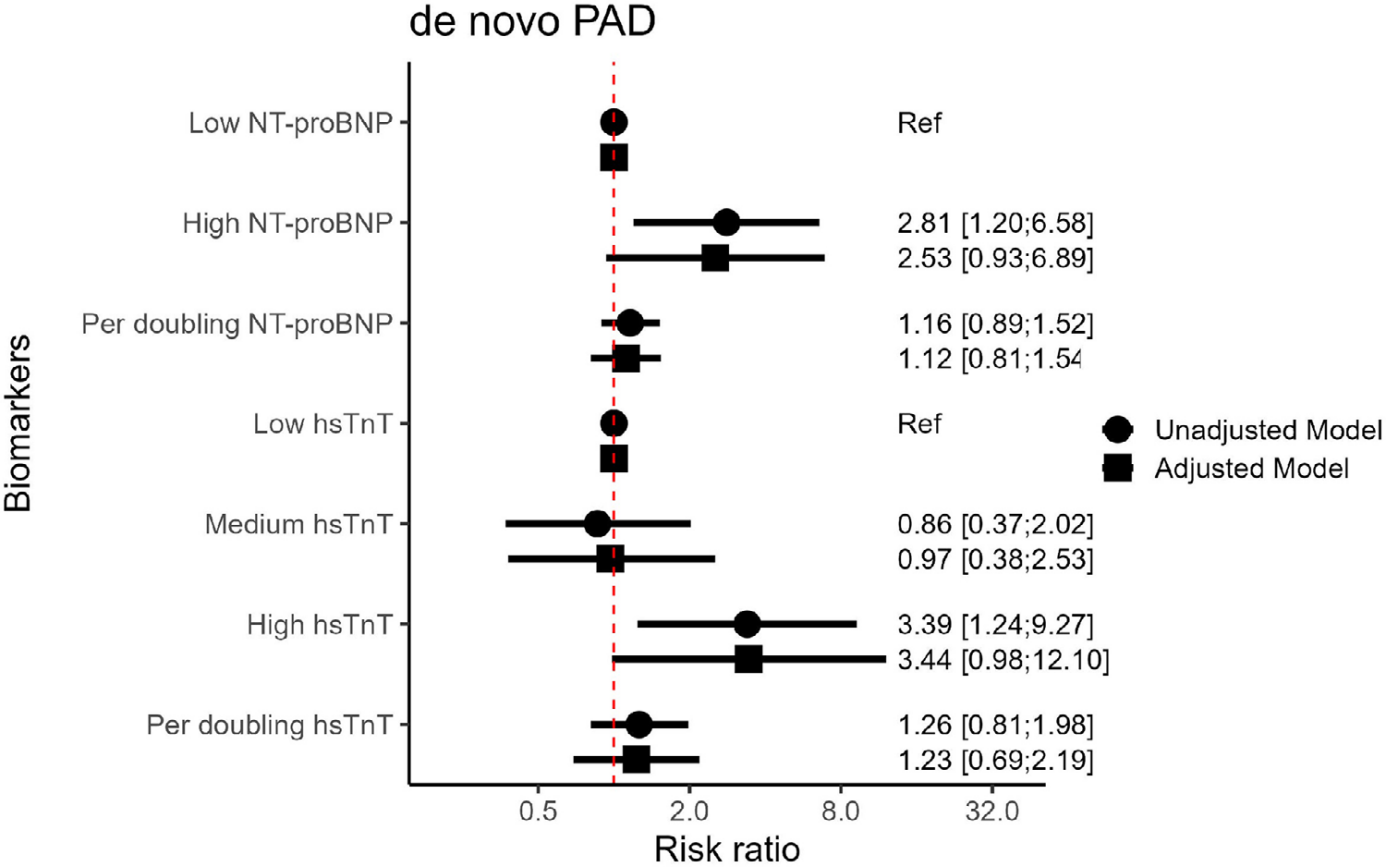
Association between hsTnT, NT‐proBNP and de novo PAD among 802 PWH. Unadjusted = no adjustments; adjusted model = adjusting for age per 10 years, smoking and diabetes. PAD, peripheral artery disease; PWH, people with HIV.

**Conclusions**: hsTnT was associated with higher odds of prevalent PAD and an increased risk of de novo PAD, albeit these associations were only borderline significant in adjusted analyses. hsTnT may be a potential biomarker for PAD in PWH.

### P301 Dolutegravir/lamivudine versus bictegravir/emtricitabine/tenofovir alafenamide fumarate: Real‐world Assessment of weight Gain in naïve people living with HIV of Asian OrigiN (DRAGON study)

Jang‐Pin Chen ^1^, Tsung‐Chia Chen^2^, Wen‐Hsin Hsih^3^, Mao‐Song Tsai^4^, Chia‐Jui Yang^4^, Shu‐Hsing Cheng^1^, Yi‐Chun Lin^1^, Ping Ma^5^, Qingxia Zhao^6^, Meiyin Zou^7^, Yi‐Wen Wu^8^, Miao‐Hui Huang^8^, Wang‐Huei Sheng^9^, Chien‐Yu Cheng^10^

^1^Department of Infectious Diseases, Taoyuan General Hospital, Ministry of Health and Welfare, Taoyuan, Taiwan. ^2^Department of Infectious Diseases, Taichung Hospital, Ministry of Health and Welfare, Taichung, Taiwan. ^3^Department of Infectious Diseases, China Medical University Hospital, Taichung, Taiwan. ^4^Department of Internal Medicine, Far Eastern Memorial Hospital, New Taipei City, Taiwan. ^5^Department of Infectious Diseases, Tianjin Second People's Hospital, Tianjin, China. ^6^Department of Infectious Diseases, Henan Infectious Diseases Hospital, Henan, China. ^7^Department of Infectious Diseases, Nantong Third People's Hospital, Jiangsu, China. ^8^Department of Infectious Diseases, Hualien Tzu Chi Hospital, Buddhist Tzu Chi Medical Foundation, Hualien, Taiwan. ^9^Department of Infectious Diseases, National Taiwan University Hospital, Taipei, Taiwan. ^10^Department of Infectious Diseases, Taoyuan General Hospital, Taoyuan City, Taiwan

**Background**: Studies have shown an increase in weight among people living with HIV (PLWH) who have took integrase strand‐transfer inhibitor (INSTI) containing antiretroviral therapy (ART). However, limited data are available in Asia. The aim was to assess effectiveness and weight change after initiating bictegravir/emtricitabine/tenofovir alafenamide fumarate (BIC/FTC/TAF) or lamivudine and dolutegravir (DTG/3TC).

**Methods**: This was a retrospective, multiple‐centre, observational cohort study conducted at nine HIV‐care‐designated hospitals from October 2019 to October 2023. Information on demographics, body weight, clinical characteristics, laboratory testing, HIV viral loads, and lipid profiles was collected and analysed. The proportion of participants with HIV‐1 RNA of less than 50 copies/mL and 200 copies/mL (Snapshot) at week 48 was analysed. Non‐inferiority margins for the adjusted difference in proportion of participants with HIV‐1 RNA of less than 50 copies/mL and 200 copies/mL at week 48 were ‐12% and ‐5%, respectively.

**Results**: A total of 381 patients were included, with 98 in the DTG/3TC group and 283 in the BIC/FTC/TAF group. CD4+ cell count was 322 (207–503) cells/mL and 190 (54–345) cells/mL in the DTG/3TC and BIC/FTC/TAF group, respectively (*p* < 0.01), and HIV RNA was 4.7 (4.16–5.39) log_10_ copies/mL and 4.51 (3.19–5.1) log_10_ copies/mL (*p* = 0.01), respectively (Table 1). At week 48, 87.8% and 98% of patients in the DTG/3TC group, and 92.6% and 97.9% in the BIC/FTC/TAF group achieved HIV RNA <50 copies/mL and <200 copies/mL (Figure 1). Absolute mean (SD) weight at baseline versus week 48 was 70.1 (11.0) versus 73.9 (12.8) kg in the DTG/3TC group and 66.9 (10.6) versus 70.1 (11.8) kg in the BIC/FTC/TAF group, respectively. Adjusted mean weight change from baseline to week 48 was 3.8 kg in the DTG/3TC group and 3.2 kg in the BIC/FTC/TAF group (adjusted difference, 0.6 kg; 95% CI ‐1.5, 2.7; *p* = 0.64). Additionally, 11 patients (23.9%) and 41 patients (18.6%) exhibited ≥10% weight gain in the DTG/3TC group and BIC/FTC/TAF group (*p* = 0.42).

**P301: Table 1:** Baseline characteristics of naïve PLWH in the study cohort

	DTG/3TC (*n* = 98)	BIC/FTC/TAF (*n* = 283)	*p*‐value
Age, y/o ± SD	35 ± 9.8	44.2 ± 13.7	<0.01
Male, *n* (%)	93 (94.9)	244 (86.2)	0.03
Route of transmission			<0.01
Men who have sex with men, *n* (%)	74 (75.5)	132 (46.6)	
Injection drug user, *n* (%)	8 (8.2)	75 (26.5)	
Heterosexual, *n* (%)	16 (17.4)	67 (23.7)	
Other, *n* (%)	0 (0)	9 (3.2)	
CD4 counts at baseline, cells/uL ± SD	362 ± 233	245 ± 230	<0.01
CD4 counts <200 cells/uL, *n* (%)	23 (25.3)	148 (52.3)	<0.01
HIV‐1 RNA at baseline, copies/mL, log10 ± SD	4.73 ± 0.94	4.38 ± 1.24	0.01
HIV‐1 RNA >100,000 copies/mL, *n* (%)	40 (40.8)	81 (28.6)	<0.01
Body weight at baseline, kg ± SD	70.1 ± 11.0	66.9 ± 10.6	0.07

**P301: Figure 1:**
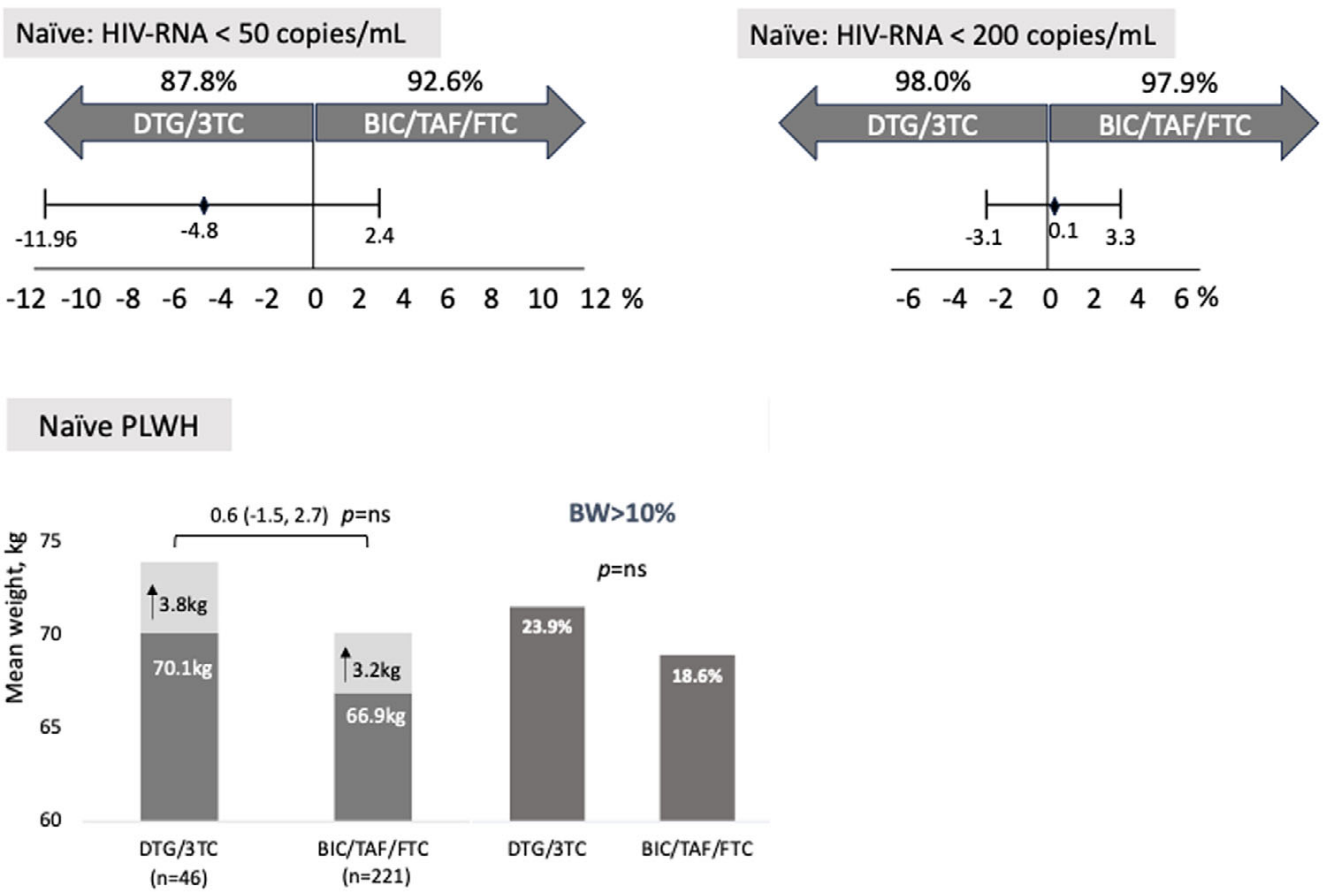
Effectiveness of viral suppression and weight change was shown among ART‐naïve PLWH in group of DTG/3TC and BIC/FTC/TAF at week 48.

**Conclusions**: In a real‐world setting, statistics analysis showed DTG/3TC was noninferior to BIC/FTC/TAF for achieving virological suppression at week 48. Furthermore. BIC/FTC/TAF is not associated with greater weight gain compared to DTG/3TC among naïve PLWH in Asia.

### P302 Evaluating the cardiovascular risk and the achievement of target levels in low‐density lipoprotein cholesterol in PLWH: insights from the DUALIS study

Florian Voit ^1^, Sven Breitschwerdt^2^, Annamaria Balogh^3^, Helen Bidner^4^, Silvia Egert‐Schwender^4^, Christoph Boesecke^2^, Laura Wagner^1^, Eva Wolf^3^, Christoph Spinner^1^

^1^Department of Clinical Medicine—Clinical Department for Internal Medicine II, University Medical Centre, Technical University of Munich, Munich, Germany. ^2^Department of Internal Medicine I, University Hospital Bonn, Bonn, Germany. ^3^MUC Research, Munich, Germany. ^4^School of Medicine and Health, Münchner Studienzentrum (MSZ), University Medical Centre, Technical University of Munich (TUM), Munich, Germany

**Background**: This post‐hoc evaluation of the DUALIS study aimed to examine cardiovascular risk and the achievement of low‐density lipoprotein cholesterol (LDL‐C) targets in a virologically suppressed cohort of people living with HIV (PLWH) in Germany.

**Methods**: Cardiovascular risk was assessed using the European Society of Cardiology (ESC)‐Systematic Coronary Risk Evaluation 2 (SCORE2)/SCORE2‐Older Persons (OP) and the current ESC guideline‐recommended LDL‐C targets among participants aged ≥40 years in the DUALIS study. Risk categorisation was based on the ESC‐SCORE2/SCORE2‐OP results and the presence of specific comorbidities indicative of high risk and very high risk of CVD.

**Results**: The use of lipid‐lowering therapy (LLT) was low in the DUALIS study, with only 12 out of 188 participants (6%) receiving the treatment. The median ESC‐SCORE2/SCORE2‐OP was 5.0. Overall, 92 participants (49%) had low‐to‐moderate CVD risk, 77 (41%) had high risk and 19 (10%) had very high risk. Only one participant in the high‐risk group and none in the very high‐risk group met the guideline‐recommended LDL‐C targets (Table 1). Even when using the less stringent LDL‐C targets valid at the time of data collection (2016 ESC guideline), only 19.7% of the high‐risk and none of the very high‐risk participants met these targets. In addition, a strong correlation regarding the estimated CVD risk was observed between the Data‐Collection on Adverse Effects of Anti‐HIV Drugs reduced model (D:A:D (R)) and ESC SCORE2/SCORE2‐OP scores (*r* = 0.95).

**P302: Table 1:** Achievement of LDL‐C target levels according to 2021 ESC guidelines on cardiovascular disease prevention in clinical practice. *N*, number of participants; %, percentage indicates the proportion of individuals within the respective risk group; LLT, lipid‐lowering therapy; Low/moderate risk <50 years, <2.5%/50–69 years, <5%/≥70 years, <7.5%; High risk <50 years, 2.5 to <7.5%/50–69 years, 5 to <10%/≥70 years, 7.5 to <15%; Very high risk <50 years, ≥7.5%/50–69 years, ≥10%/≥70 years, ≥15%

	None	LLT	Total	
	*N*	*N*	*N*	%
Low/moderate risk				
Above target levels	67	2	69	75
Below target levels	22	1	23	25
High risk				
Above target levels	68	8	76	98.7
Below target levels	1		1	1.3
Very high risk				
Above target levels	18	1	19	100
Below target levels	0	0	0	0
Total				
Above target levels	153	11	164	87.2
Below target levels	23	1	24	12.8
Total	176	12	188	100

**Conclusions**: The achievement of guideline‐recommended LDL‐C targets was low in the high‐ and very high‐CVD‐risk groups in the DUALIS study, reflecting low utilisation of LLT in clinical practice.

### P303 Prevalence and cardiovascular risk assessment in HIV patients: comparison between SCORE2 and REGICOR scales

Estefanía Díaz Martín ^1^, Inês Cachapa Viera^1^, Sonia Calzado Isbert^2^, María Del Carmen Navarro Saez^2^, Marc Pedrosa Aragón^2^, Marta Navarro Vilasaró^2^

^1^Servicio de Medicina Interna, Hospital Universitario Parc Taulí, Barcelona, Spain. ^2^Servicio de Enfermedades Infecciosas, Hospital Universitario Parc Taulí, Barcelona, Spain

**Background**: People living with HIV are at higher risk of cardiovascular disease (CVD) compared to the general population, due to complex factors related to the virus, host and antiretroviral therapy (ART) [1]. Our main objective was to determine the prevalence of CVD risk in our hospital cohort and to compare the agreement between the European SCORE2 (Systemic Coronary Risk Evaluation Score 2) and the Spanish REGICOR (Registro Gironí del Cor) risk scales [2,3].

**Materials and methods**: A retrospective, single‐centre cross‐sectional study was conducted on a sample of HIV‐positive patients from November 2023 to June 2024, attending our clinics. Inclusion criteria were aged 40 to 70 years and under follow‐up at our centre. Demographic, clinical and laboratory data were collected, and web‐based calculators for SCORE2 and REGICOR were used to categorise patients into high/very high, moderate and low risk of experiencing CVD in the next 10 years, as well as the need for lipid‐lowering therapy. Agreement between scores was assessed using Cohen's kappa statistic (*κ*).

**Results**: A total of 120 patients were analysed, of whom 76% (91/120) were male. Among them, 100 patients were of Spanish nationality, with a mean age of 54 years (range 40–69). The prevalence of CVD risk factors was: hyperlipidaemia 39%, hypertension 24%, diabetes mellitus 12%, active smoking 56% and obesity (BMI >30) 15%. Cardiovascular risk distributions according to the scales are shown in Figure 1. The agreement between SCORE2 and REGICOR was 0.68 (95% CI 0.58–0.76). Based on REGICOR and SCORE2, optimised lipid‐lowering treatment was recommended for 72% and 40% of the sample, respectively, although only 39% of our cohort was on statin therapy.

**P303: Figure 1:**
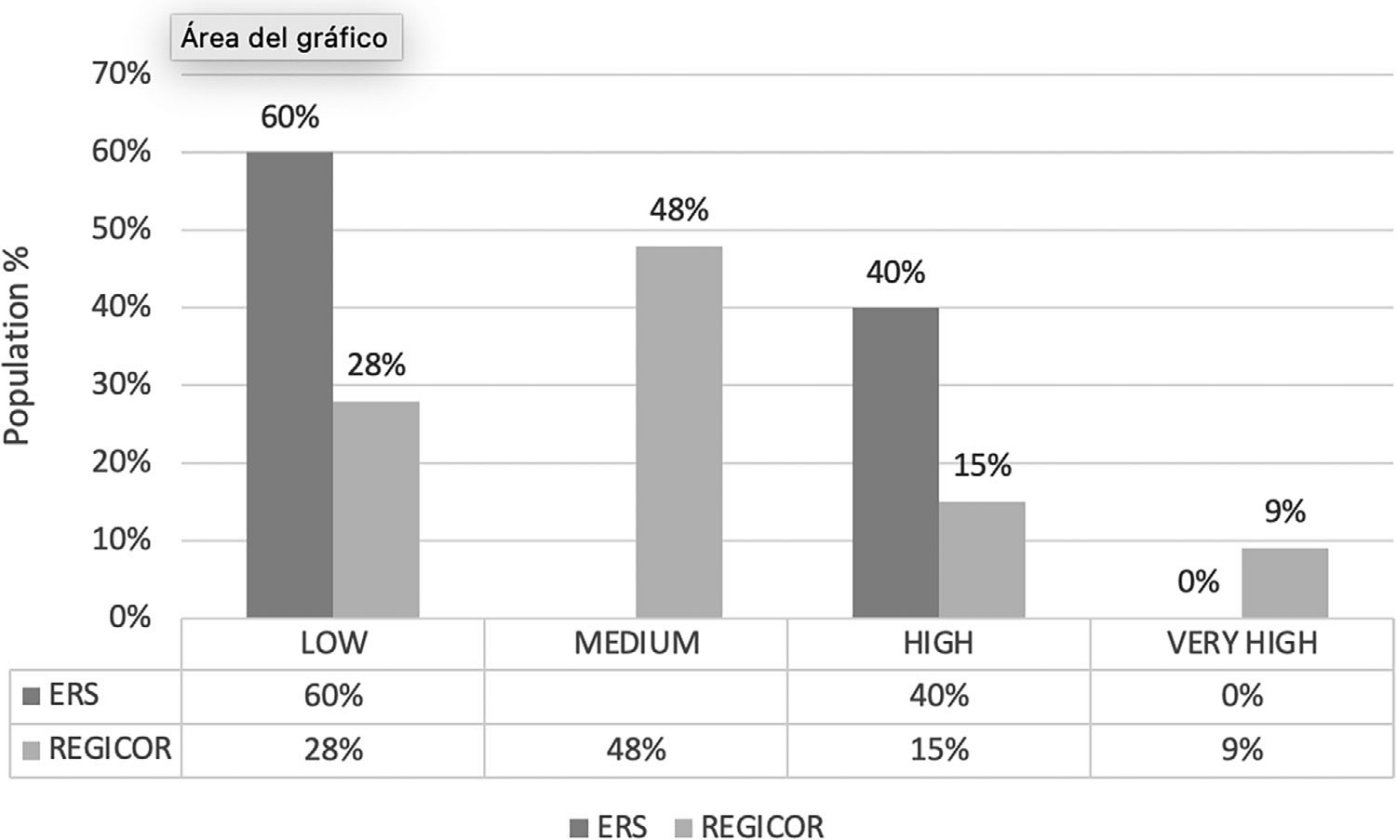
Prediction strata according to different CVD risk prediction.

**Conclusions**: Assessing and categorising CVD risk in people living with HIV is crucial for prevention and proper management. SCORE2 and REGICOR scales demonstrate satisfactory agreement in risk categorisation. SCORE2 identifies more high‐risk patients, needing urgent preventive treatment, while REGICOR identifies more patients who could benefit from preventive measures. The low treatment rate of statin therapy underscores the imperative for enhanced strategies to prevent CVD in our population.

**References**

1. Hemkens LG, Bucher HC, HIV infection and cardiovascular disease. Eur Heart J. 2014;35(21):1373‐81.

2. Marrugat J, Subirana I, Ramos R, Vila JS, Marin‐Ibañez A, Guembe MJ, et al, on behalf of the FRESCO Investigators. Derivation and validation of a set of 10‐year cardiovascular risk predictive functions in Spain: the FRESCO study. Prev Med 2014;61:66‐74.

3. Larrousse M, Martínez E. Enfermedad cardiovascular en el paciente infectado por el virus de la inmunodeficiencia humana. Hipertens Riesgo Vasc. 2010;27(4):162‐70.

### P304 A novel approach to weight loss: bringing I‐SatPro into the HIV clinic

Eleanor Hamlyn ^1^, Jorge Ferreira^1^, Sarah Edwards^1^, Tricia Tan^2^, Saira Hameed^2^, Fiona Burns^1^

^1^Ian Charleson Day Centre, Royal Free Hospital, London, UK. ^2^Metabolic Medicine and Endocrinology, Imperial Colleg Heathcare Trust, London, UK

**Background**: Around two thirds of the UK adult population, including people living with HIV (PLHIV), are living with overweight or obesity, with increasing morbidity and mortality risk. Helping PLHIV who would like to lose weight is an integral part of helping them to live well with HIV. The Imperial Satiety Protocol (I‐SatPro) [1] is an evidence‐based holistic approach to weight loss. Participants learn the scientific rationale behind making sustainable changes to eating habits, movement, sleep and selfcare which in combination result in clinically meaningful weight loss (≥10% body weight loss in previous work) as well as improvements in cardio‐metabolic health. Here we present preliminary results from the first PLHIV cohort of I‐SatPro participants delivered in a large urban HIV clinic. Participants were referred by their clinician (no specific referral criteria).

**Materials and methods**: Baseline data on demographics as well as starting weights and body mass index (BMI) were collected. Participants were asked to register weekly weights by email if they felt comfortable to do so. The program was delivered online—fortnightly over 12 weeks in January–April 2024. Daily emails were sent to reinforce learning.

**Results**: Thirty‐five patients enrolled in I‐SatPro (Table 1). Attendance at the sessions gradually decreased although some patients who did not attend the final session were still engaged with the program e.g. by email. There were no adverse events. Fifteen patients reported a weekly weight result, with seven reporting a weight at program completion (Figure 1). All reported weights demonstrated a loss. Of those patients who reported a weight result at program completion (12 weeks) (*n* = 7) the mean weight loss in women was 7 kg (range 6–8) and in men was 12.36 kg (range 5.6–22).

**P304: Table 1:** Baseline characteristics of enrolled participants

	Number of participants	Age, range (years)	Age, mean (years)	Weight, range (kg)	BMI, range	BMI, mean	Ethnicity, Black African	Ethnicity, White
Women	20	33–66	51.6	82–130	29–47	36	90%	10%
Men	15	41–68	54	87–149	29.5–41	34	20%	80%

**P304: Figure 1:**
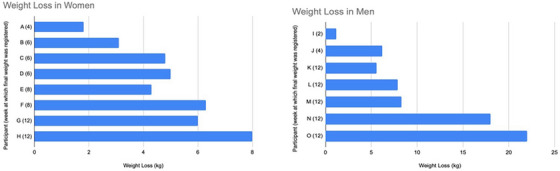
Individual reported weight loss and the week at which they last reported their weight.

**Conclusion**: In this delivery of I‐SatPro to PLHIV, patients who completed the program lost a clinically meaningful amount of weight suggesting significant potential benefits of I‐Satpro for PLHIV. Future work will focus on tailoring I‐SatPro to our patient population with the aim of increasing patient engagement. Participant feedback on improvements in quality of life was highly positive and will be formally assessed in future work alongside cardio‐metabolic benefits.

**Reference**

1. Hameed S, Salem V, Alessimii H, Scholtz S, Dar O, Miras AD, et al. Imperial Satiety Protocol: a new non‐surgical weight‐loss programme, delivered in a health care setting, produces improved clinical outcomes for people with obesity. Diabetes Obes Metab. 2021;23(1):270‐5.

### P305 Favourable cholesterol profile in virologically suppressed people with HIV‐1 switching to tenofovir DF‐containing, ainuovirine‐based compared to tenofovir alafenamide‐containing, boosted elvitegravir‐based antiretroviral regimen: normocholesterolemic subgroup analyses of the SPRINT trial, a randomised, active‐controlled phase III study

Weiping Cai^1^, Ping Ma^2^, Qingxia Zhao^3^, Hongxia Wei^4^, Hongzhou Lu^5^, Hui Wang^6^, Shenghua He^7^, Zhu Chen^8^, Yaokai Chen^9^, Min Wang^10^, Fujie Zhang^11^, Hao Wu^12^, Wan Wan^13^, Heliang Fu^13^, Hong Qin ^13^

^1^Infectious Disease Center, Guangzhou Eighth People's Hospital, Guangzhou Medical University, Guangzhou, China. ^2^Department of Infectious Diseases, Tianjin Second People's Hospital, Tianjin, China. ^3^Department of Infectious Diseases, The Sixth People's Hospital of Zhengzhou, Zhengzhou, China. ^4^Department of Infectious Diseases, The Second Hospital of Nanjing, Nanjing, China. ^5^National Clinical Research Centre for Infectious Diseases, The Third People's Hospital of Shenzhen, The Second Affiliated Hospital of Southern University of Science and Technology, Shenzhen, China. ^6^Department of Infectious Diseases, Shenzhen Third People's Hospital, Shenzhen, China. ^7^Department of Infectious Diseases, Public Health Clinical Medical Center of Chengdu, Chengdu, China. ^8^Drug Clinical Trial Institution, Public Health Clinical Medical Center of Chengdu, Chengdu, China. ^9^Division of Infectious Diseases, Chongqing Public Health Medical Center, Chongqing, China. ^10^Institute of HIV/AIDS, The First Hospital of Changsha, Changsha, China. ^11^Clinical and Research Center for Infectious Diseases, Beijing Ditan Hospital Capital Medical University, Beijing, China. ^12^Clinical and Research Center for Infectious Diseases, Beijing Youan Hospital, Capital Medical University, Beijing, China. ^13^Clinical Research & Development, Jiangsu Aidea Pharmaceutical Co., Ltd, Yangzhou, China

**Background**: In the SPRINT trial, virologically suppressed people with HIV‐1 (PWHs) switched to tenofovir DF (TDF) containing, ainuovirine (ANV, ACC008) based or tenofovir alafenamide (TAF) containing, boosted elvitegravir (EVG/c) based antiretroviral (ARV) regimen (comparator) from an efavirenz‐based regimen. ACC008 showed an improved serum lipid profile compared to the comparator. We herein reported subgroup analyses of serum low‐density lipoprotein cholesterol (LDL‐C) in PWHs with normocholesterolemia as prespecified.

**Methods**: Baseline normocholesterolaemia was defined as serum LDL‐C below 3.0 mmol/l; acceptable lipidaemic control was defined as post‐treatment serum LDL‐C below 3.0 mmol/l and good lipidaemic control as post‐treatment serum LDL‐C below 2.6 mmol/l as recommended by the European AIDS Clinical Society. Changes from baseline (CFBs) in LDL‐C (least square mean) at week 48 were compared using the mixed effects models for repeated measures with treatment as fixed factor and baseline LDL‐C measurement as a covariate, all nested within visits. The proportions of PWHs with good/acceptable lipidaemic control were analysed using the logistic regression method with baseline serum LDL‐C adjusted at week 48.

**Results**: The proportions of PWHs with normocholesterolaemia were comparable between the two arms (61.9% [236/381] vs 62.7% [239/381]), with a similar mean value of 2.35 ± 0.41 versus 2.36 ± 0.43 mmol/l at baseline. CFB in LDL‐C showed a significantly less increase in the ACC008 arm compared to that in the comparator arm at week 48 (0.02 vs 0.39 mmol/l, estimated treatment difference [95% CI] ‐0.37 [‐0.45, ‐0.29], *p* < 0.001) (Figure 1a). The proportions of PWHs with acceptable/good lipidaemic control were significantly greater in the ACC008 arm than those in the comparator arm (acceptable, 86.4% [204/236] vs 68.6% [164/239], 0.178 [0.104–0.250], *p* < 0.001; good, 64.8% [153/236] vs 38.1% [91/239], 0.268 [0.178–0.351], *p* < 0.001) at week 48 (Figure 1b).

**P305: Figure 1:**
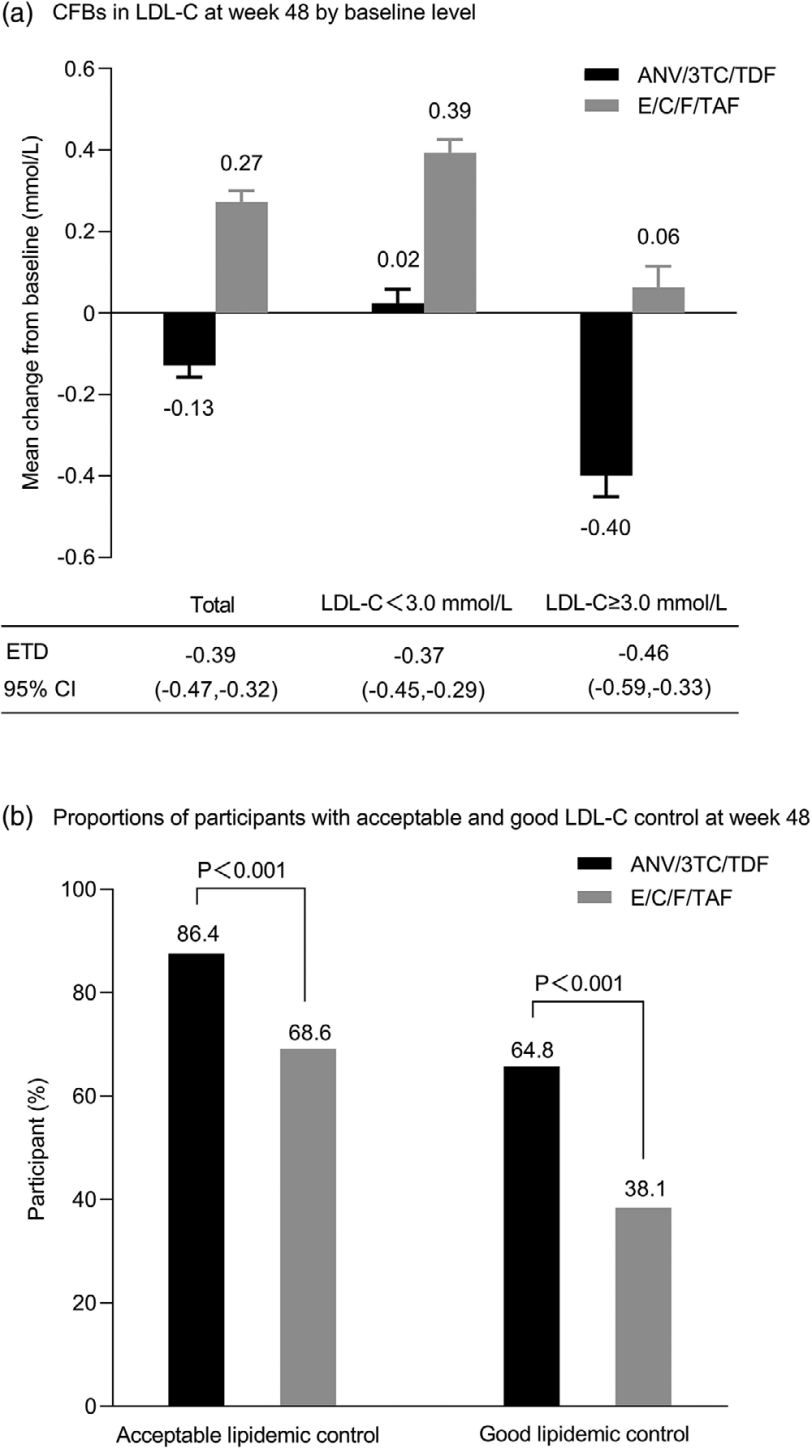
CFBs in LDL‐C and LDL‐C control at week 48. (a) CFBs in LDL‐C at week 48 by baseline level; (b) proportions of participants with acceptable and good LDL‐C control at week 48. ANV/3TC/TDF, ainuovirine/ lamivudine/tenofovir disoproxil; CFBs, changes from baseline; E/C/F/TAF, elvitegravir/cobicistat/emtricitabine/tenofovir alafenamide; LDL‐C, low density lipoprotein cholesterol.

**Conclusions**: In PWHs with baseline normocholesterolaemia, switching to a TDF‐containing, ANV‐based regimen resulted in less LDL‐C increase and better lipidaemic control compared to a TAF‐containing, EVG/c‐based regimen.

### P306 Immune and metabolic profile in people living with HIV initiating emtricitabine/tenofovir alafenamide/bictegravir

Nicola Squillace ^1^, Elena Delfina Ricci^2^, Paolo Maggi^3^, Lucia Taramasso^4^, Barbara Menzaghi^5^, Giuseppe Vittorio De Socio^6^, Eleonora Sarchi^7^, Stefania Piconi^8^, Giovanni Francesco Pellicanò^9^, Giordano Madeddu^10^, Alessandra Bandera^11^, Salvatore Martini^12^, Benedetto Maurizio Celesia^13^, Giancarlo Orofino^14^, Sergio Ferrara^15^, Emanuele Pontali^16^, Maria Aurora Carleo^17^, Elena Salomoni^18^, Giovanni Cenderello^19^, Antonio Cascio^20^, Goffredo Angioni^21^, Katia Falasca^22^, Olivia Bargiacchi^23^, Antonio Di Biagio^4^, Paolo Bonfanti^24^

^1^Infectious Diseases Clinic, Fondazione IRCCS San Gerardo dei Tintori, Monza, Italy. ^2^Fondazione ASIA Onlus, Buccinasco, Italy. ^3^Infectious Diseases Unit, AORN Sant'Anna e San Sebastiano, Caserta, Italy. ^4^Infectious Diseases Clinic, San Martino Hospital Genoa, University of Genoa, Genoa, Italy. ^5^Infectious Diseases Unit, ASST della Valle Olona, Busto Arsizio, Italy. ^6^Infectious Diseases Unit, Santa Maria Hospital, Perugia, Italy. ^7^Infectious Diseases Unit, S.Antonio e Biagio e Cesare Arrigo Hospital, Alessandria, Italy. ^8^Infectious Diseases Unit, A. Manzoni Hospital, Lecco, Italy. ^9^Infectious Diseases Unit, Department of Clinical and Experimental Medicine, AOU Policlinico G. Martino, Messina, Italy. ^10^Infectious Diseases Unit, Department of Medicine, Surgery and Pharmacy, University of Sassari, Sassari, Italy. ^11^Infectious Diseases Unit, Fondazione IRCCS Ca' Granda Ospedale Maggiore Policlinico, Milan, Italy. ^12^Infectious Diseases Unit, University Hospital Luigi Vanvitelli, Naples, Italy. ^13^Infectious Diseases Unit, Garibaldi Hospital, Catania, Italy. ^14^Division I of Infectious and Tropical Diseases, ASL Città di Torino, Torino, Italy. ^15^Infectious Diseases Unit, Department of Clinical and Experimental Medicine, University of Foggia, Foggia, Italy. ^16^Department of Infectious Diseases, Galliera Hospital, Genoa, Italy. ^17^Infectious Diseases and Gender Medicine Unit, Cotugno Hospital, AO dei Colli, Naples, Italy. ^18^Infectious Diseases Unit, Santa Maria Annunziata Hospital, Florence, Italy. ^19^Infectious Diseases Department, Sanremo Hospital, Sanremo, Italy. ^20^Infectious Diseases Unit, Department of Health Promotion, Mother and Child Care, Internal Medicine and Medical Specialties, University of Palermo, Palermo, Italy. ^21^Infectious Diseases Unit, SS Trinità Hospital, Cagliari, Italy. ^22^Infectious Diseases Clinic, Department of Medicine and Science of Aging, G. D'Annunzio University, Chieti‐Pescara, Chieti, Italy. ^23^Infectious Diseases Unit, Ospedale Maggiore della Carità, Novara, Italy. ^24^Infectious Diseases Clinic, Fondazione IRCCS San Gerardo dei Tintori‐University of Milano‐Bicocca, Monza, Italy

**Background**: Our aim was to investigate the role of emtricitabine/tenofovir alafenamide/bictegravir (FTC/TAF/BIC) regimen on metabolic and immune profile.

**Material and methods**: Consecutive people living with HIV (PWH) enrolled in SCOLTA project initiating antiretroviral treatment (ART) with FTC/TAF/BIC were included. T0 and T1 were defined as results at baseline and 96‐weeks follow‐up respectively. PWH with HBV co‐infection were excluded. PWH were classified according to naïve (N) and experienced (E) status. E PWH were divided into two groups according to previous ART including COBI (C‐E) or not (NC‐E).

**Results**: Eight hundred and thirteen PWH were enrolled (601 E and 212 naïve N to ART). C‐E and NC‐E were 267 and 334, respectively. PWH characteristics are depicted in Table 1. The following variables modified significantly at T1 both in C‐E and NC‐E: weight (mean change 1.5 kg [95% CI 0.5–2.5] and 1.1 [0.1–2.1]; Figure 1), CD4 cell count (45 cells/microl [13–78] and 46 [13–78]), CD4/CD8 ratio (0.08 [0.03–0.12] and 0.12 [0.07–0.16]), total cholesterol (TC) (‐14 mg/dl [‐20, ‐9] and ‐5 mg/dl [‐11, 0]), and triglycerides (‐19 mg/dl [‐30, ‐8] and ‐15 [‐26, ‐4]). In C‐E also LDL cholesterol (LDL‐c) (‐11 mg/dl [‐16, ‐6]) significantly changed at T1. The following variables were significantly different between N and C‐E and NC‐E at T1: TC (mean change 11 mg/dl [95% CI 3–19] vs ‐14 [‐20, ‐9] vs ‐5 [‐11, 0]; *p* < 0.0001), LDL‐c (6 mg/dl [‐1, 14] vs ‐11 [‐16, ‐6] vs 0 [‐5, 5]; *p* = 0.0002), ALT (‐8 IU/L [‐13, ‐2] vs 3 [‐1, 7] vs 0 [‐4, 4]; *p* = 0.008), CD4 cell count (292 cells/microl [243–340] vs 45 [13–78] vs 46 [13–78]; *p* < 0.0001), CD4/CD8 ratio (0.43 [0.36–0.50] vs 0.08 [0.03–0.12] vs 0.12 [0.07–0.16]; *p* < 0.0001).

**P306: Table 1:** Baseline characteristics of 813 PWH starting a regimen with FTC/TAF/BIC

	ART‐experienced *N* = 601 (73.9%)	ART‐naïve *N* = 212 (26.1%)	
Variables at enrolment	*N* or mean or median (% or SD or IQR)	*N* or mean or median (% or SD or IQR)	*p*‐value
Age, years	49.7 (12.1)	42.0 (12.4)	<0.0001
Male sex	445 (74.0%)	165 (77.8%)	0.27
Caucasian	527 (87.7%)	173 (81.6%)	0.03
Risk factor for HIV acquisition			
Sexual	373 (62.1%)	183 (86.3%)	<0.0001
IDU	89 (14.8%)	9 (4.2%)	<0.0001
Other/ND	139 (23.1%)	20 (9.4%)	<0.0001
BMI, kg/m^2^ (n = 408)	25.9 (4.7)	23.8 (3.6)	<0.0001
Weight, kg	75.5 (14.8)	70.8 (13.3)	<0.0001
HCV coinfection	110 (18.3%)	12 (5.7%)	<0.0001
HIV RNA >40 copies/microL	108 (18.0%)		
Previous ART			
FTC/TAF/EVG/COBI	196 (32.6%)		
FTC/TAF/DTG	90 (15.0%)		
Other DTG‐based	117 (19.5%)		
Any other TAF‐including	70 (11.6%)		
TDF‐including	19 (3.2%)		
Other	45 (7.5%)		
Unknown	64 (10.6%)		
CD4+, cells/mm^3^	603 (424‐834)	317 (124‐505)	<0.0001
Total cholesterol, mg/dl	193 (43)	166 (42)	<0.0001
HDL‐c, mg/dl	54 (18)	44 (16)	<0.0001
LDL‐c, mg/dl	112 (38)	97 (35)	<0.0001
TGL, mg/dl	114 (84‐167)	99 (72‐149)	0.002
BG (in 765 non‐diabetic pts), mg/dl	93 (16)	87 (14)	<0.0001
BG (in 48 diabetic pts), mg/dl	160 (66)	143 (40)	0.53
Diabetes	39 (6.5%)	9 (4.2%)	0.23
AST, IU/dl	22 (18‐27)	24 (19‐31)	0.003
ALT, IU/dl	22 (16‐31)	23 (17‐33)	0.12

Abbreviations: ALT, alanine aminotransferase; AST, aspartate aminotransferase; BG, blood glucose; BMI, body mass index; CI, confidence interval; HDL‐c, high density lipoprotein‐cholesterol; IDU, intravenous drug user; IQR, interquartile range; LDL‐c, low density lipoprotein‐cholesterol; ND, not defined; SD, standard deviation; TGL, triglycerides.

**P306: Figure 1:**
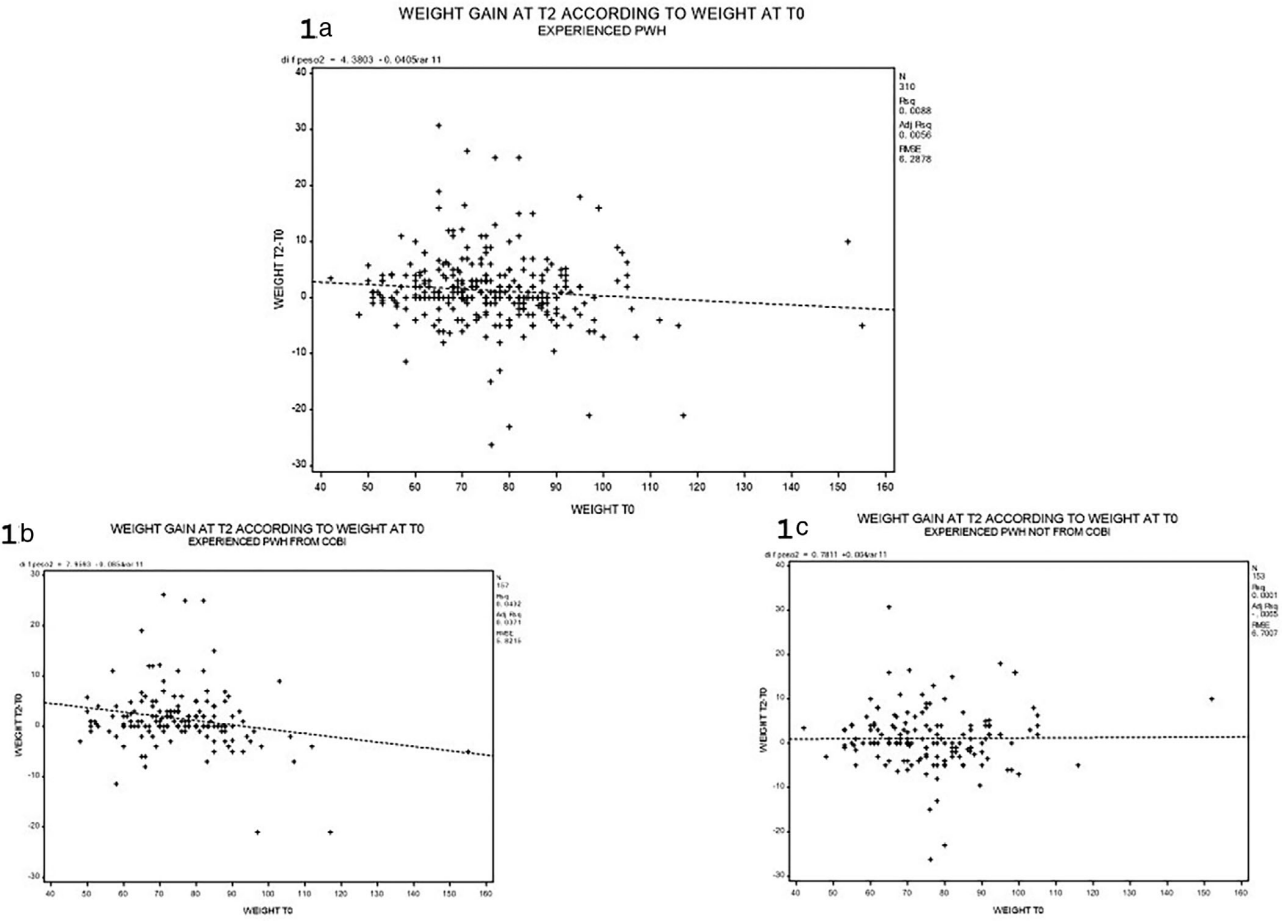
Weight gain at T2 according to weight at T0 in experienced PWH (1a), in experienced PWH from COBI‐including regimen (1b) and in experienced PWH not from COBI including regimen (1c).

**Conclusions**: FTC/TAF/BIC initiation is associated with weight gain and amelioration of immune profile both in N and E PWH. A significant reduction in LDL‐c was observed only in E with COBI in previous regimen.

## Co‐morbidities and complications of disease and/or treatment—Malignancies: non‐AIDS defining

### P307 Outcomes after the most common cancers in people with HIV

Alisa Timiryasova ^1^, Lauren Greenberg^1^, Pere Domingo^2^, Philip E Tarr^3^, Alexander Egle^4^, Charlotte Martin^5^, Cristina Mussini^6^, Ferdinand Wit^7^, Antonella Cingolani^8^, Jörg Janne Vehreschild^9,21,22^, Antonella Castagna^10^, Kathy Petoumenos^11^, Caroline Sabin^12^, Wafaa El‐Sadr^13^, Fabrice Bonnet^14^, Jens Lundgren^1^, Martina Botanelli^10^, Sean Hosein^15^, Christina Carlander^16^, Alain Amstutz^3^, Katharina Grabmeier‐Pfistershammer^17^, Harmony Garges^18^, Andrea Marongiu^19^, Lital A Young^20^, Lars Peters^1^, Lene Ryom^1,23,24^, for the RESPOND (International Cohort Consortium of Infectious Diseases) and D:A:D (The Data Collection on Adverse Events of Anti‐HIV Drugs Study) Study Groups

^1^Centre of Excellence for Health, Immunity and Infections (CHIP), Department of Infectious Diseases, Rigshospitalet, University of Copenhagen, Copenhagen, Denmark. ^2^Infectious Diseases, Hospital de la Santa Creu i Sant Pau, Barcelona, Spain. ^3^Swiss HIV Cohort Study (SHCS), University of Basel, Basel, Switzerland. ^4^3rd Medical Department, Paracelsus Medical University, Zalzburg, Austria. ^5^CHU Saint‐Pierre, Centre de Recherche en Maladies Infectieuses a.s.b.l., Brussels, Belgium. ^6^Modena HIV Cohort, Università degli Studi di Modena, Modena, Italy. ^7^AIDS Therapy Evaluation in the Netherlands (ATHENA) cohort, HIV Monitoring Foundation, Amsterdam, Netherlands. ^8^Italian Cohort Naive Antiretrovirals (ICONA), Fondazione Policlinico A. Gemelli, IRCCS, Rome, Italy. ^9^Department I of Internal Medicine, Faculty of Medicine and University Hospital Cologne, Cologne, Germany. ^10^San Raffaele Scientific Institute, Università Vita‐Salute San Raffaele, Milan, Italy. ^11^The Australian HIV Observational Database (AHOD), The University of New South Wales, Sydney, Australia. ^12^Centre for Clinical Research, Epidemiology, Modelling and Evaluation (CREME), Institute for Global Health, University College London, London, UK. ^13^Community Programs for Clinical Research on AIDS (CPCRA), CAP‐Columbia University and Harlem Hospital, New York, NY, USA. ^14^BPH, INSERM U1219, CHU de Bordeaux and Bordeaux University, Bordeaux, France. ^15^European AIDS Treatment Group (EATG), Brussels, Belgium. ^16^Department of Infectious Diseases, Karolinska University Hospital, Stockholm, Sweden. ^17^Center for Pathophysiology, Infectiology and Immunology, Medical University of Vienna, Vienna, Austria. ^18^Global Medical, ViiV Healthcare, Durham, NC, USA. ^19^Real World Evidence Virology, Gilead Sciences, London, UK. ^20^Global Medical Affairs HIV, Merck Sharp & Dohme, Zurich, Switzerland. ^21^German Centre for Infection Research, Partner Site Bonn‐Cologne, Cologne, Germany. ^22^Department II of Internal Medicine, Hematology/Oncology, Goethe University, Frankfurt, Frankfurt Am Main, Germany. ^23^Department of Clinical Medicine, University of Copenhagen, Denmark. ^24^Department of Infectious Diseases, Hvidovre University Hospital, Copenhagen, Denmark.

**Background**: While cancer is a leading cause of death in people with HIV, less is known about clinical outcomes after individual cancers. We compared incidence and risk factors for death and a composite clinical outcome (CCO; cardiovascular disease, diabetes, AIDS events, another cancer) after the most common cancers in participants in D:A:D and RESPOND.

**Materials and methods**: Participants with Kaposi's sarcoma (KS), non‐Hodgkin lymphoma (NHL), lung (LC), anal (AC) or prostate cancer (PC) were followed from first cancer diagnosis after 2006/2012 [D:A:D/RESPOND] until death, last follow‐up, or administrative censoring (1 Feb 2016/31 Dec 2021 [D:A:D/RESPOND]). Poisson regression assessed potential predictors of death and CCO.

**Results**: Two thousand, four hundred and eighty‐five individuals were included (602 KS, 599 NHL, 518 LC, 442 AC, 324 PC), with total follow‐up of 10,630 years (median 3.1 (IQR 0.8–7.1)). At diagnosis, median age was 55 years (43 years KS, 48 NHL, 57 LC, 52 AC, 64 PC), CD4 count was 399 cells/µl, and 88% were male. LC was predominantly disseminated at diagnosis (57%), while AC (66%) and PC (62%) localised. Mortality and CCO were highest after LC (mortality incidence/1000 person‐years 445.4 [95% CI 399.7–494.9]; CCO 117.1 [94.3–143.8]) compared to the other cancers (Table 1). CCO was predominantly AIDS events after NHL and KS (51%, 61%), diabetes after LC and PC (47%, 35%), and cancer after AC (36%). Mortality risk decreased over time (4–10%/calendar year) after LC, AC and NHL (Figure 1a). Participants with injecting drug use as HIV transmission category had three times higher mortality risk after AC versus men who have sex with men. Older age was associated with 24–40% higher mortality/10 years in those with NHL or AC. Increasing time‐updated CD4 count reduced mortality by 15–40%/100 cells/µl after LC, AC and NHL, and CCO by 17–28% after KS, NHL and AC. Smoking doubled CCO risks after KS and NHL; low BMI and multimorbidity increased CCO 2–3 times after KS and NHL; other risk factors were non‐significant, potentially due to low power (Figure 1b).

**P307: Table 1:** Crude incidence rates and 95% confidence intervals for death and composite clinical outcomes after individual cancers

	Kaposi's sarcoma (*n* = 602)	Non‐Hodgkin lymphoma (*n* = 599)	Lung cancer (*n* = 518)	Anal cancer (*n* = 442)	Prostate cancer (*n* = 324)
	IR/1000 PYFU [95% CI]; (*n* events)	IR/1000 PYFU [95% CI]; (*n* events)	IR/1000 PYFU [95% CI]; (*n* events)	IR/1000 PYFU [95% CI]; (*n* events)	IR/1000 PYFU [95% CI]; (*n* events)
Death	21.3 [16.9‐26.6]; (79)	87.8 [76.5‐100.23]; (218)	445.5 [399.7‐494.9]; (346)	48.2 [39.3‐58.4]; (103)	35.0 [26.3‐45.7]; (54)
Composite clinical outcome^a^	49.3 [37.5‐51.3]; (163)	64.0 [54.5‐74.8]; (159)	117.1 [94.3‐143.8]; (91)	54.2 [44.8‐65.1]; (116)	53.9 [42.9‐66.8]; (83)

Abbreviations: IR, incidence rates; PYFU, person‐years of follow‐up.

^a^ Composite clinical outcome: cardiovascular disease, diabetes, AIDS‐events, another primary cancer.

**P307: Figure 1:**
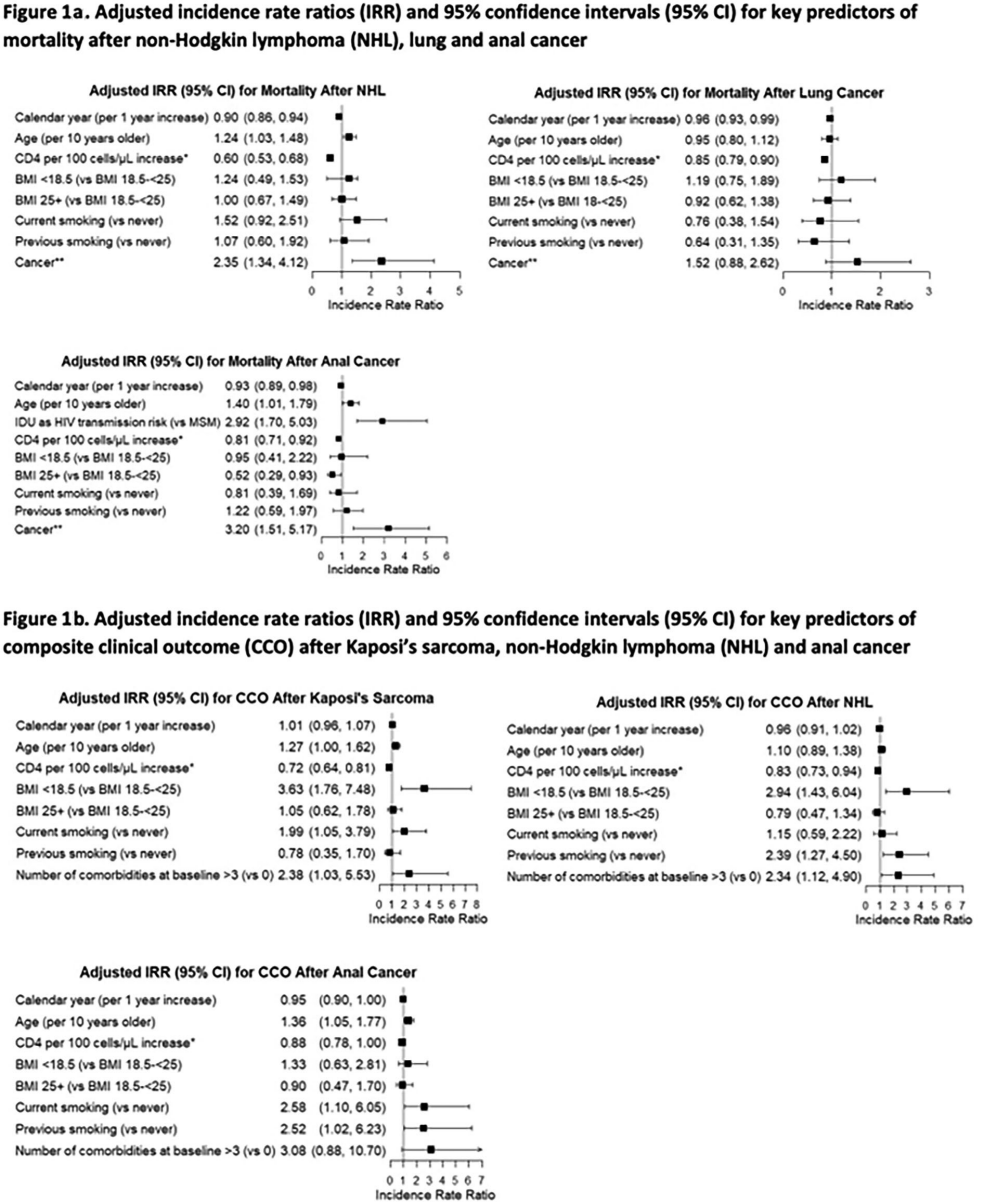
(a) In all models, age (fixed at baseline), gender/sex (fixed at baseline), ART status (fixed at baseline), BMI (fixed at baseline), calendar year (time‐updated), smoking status (time‐updated) were a priori included. Other risk factors were included in the multivariable model based on univariable models (*p* < 0.1 for inclusion). Therefore, the NHL model was also adjusted for hypertension. Due to low numbers cancer stage (disseminated vs localised) was not included in the final models. Predictors of mortality after KS and PC were not assessed due to low numbers and are not presented here. Baseline is the date of cancer diagnosis. (b) Models were a priori adjusted for the same variables as in Figure 1a. KS model was also adjusted for HIV transmission risk group and viral load at baseline. Number of comorbidities at baseline includes prior AIDS‐defining and non‐AIDS defining cancers, AIDS events, chronic kidney disease, cardiovascular disease, hypertension, diabetes, dyslipidaemia. There were no significant predictors of CCO after LC and PC, therefore these models are not presented here. *CD4 was time‐updated; **cancer: a different primary cancer during follow‐up. IDU, injecting drug use; MSM, men who have sex with men.

**Conclusions**: Mortality was high after LC and NHL but declined over time for LC, AC and NHL. Each cancer had a unique risk profile for mortality and CCO requiring careful monitoring.

### P308 High burden of non‐AIDS defining malignancies among adults with HIV in Thailand, 1996‐2023

Teton Avihingsanon ^1^, Rin Indarodom^1^, Pathaitorn Damtham^1^, Farsai Chiewbangyang^1^, Win Min Han^2^, Jiratchaya Sophonphan^3^, Haymar Su Lwin^2^, Anchalee Avihingsanon^2^, Napa Parinyanitikul^4^

^1^Medicine, Faculty of Medicine, Chulalongkorn University, Bangkok, Thailand. ^2^Medicine, HIV‐NAT, Thai Red Cross AIDS Research Center, Bangkok, Thailand. ^3^Statistics, HIV‐NAT, Thai Red Cross AIDS Research Center, Bangkok, Thailand. ^4^Division of Medical Oncology, Faculty of Medicine, Chulalongkorn University, Bangkok, Thailand

**Background**: With effective contemporary antiretroviral therapy (ART) and increasing longevity, main causes of death among people with HIV (PWH) have been shifted from AIDS‐defining malignancies (ADM) to non‐AIDS defining malignancies (NADM), recently. This epidemiological data is urgently needed to plan for effective cancer screening and treatment. We therefore examined the cancer incidence and age‐standardised incidence rate (ASR) among 3178 adult PWH in Thailand.

**Materials and methods**: PWH without known cancer prior to ART at HIV‐NAT, Thai Red Cross AIDS Research Centre, Thailand from 1 June 1996 to 31 December 2023, were retrospectively reviewed. Cancer crude incidence rate and ASR using the data from the general population were calculated. The incidence of ADM and NADM were also reported separately.

**Results**: There were 70.4% male, HBsAg positivity was 17.6%, anti‐HCV positivity was 12.3%. During a median follow‐up of 9.8 (IQR 3.4–18.5) years, 93 PWH developed cancers, resulting in crude incidence rate of 260 (95% CI 212–318.9) per 100,000 person‐year (PY). The incidence of NADM and ADM was 212.7 (170–266.3) per 100,000 PY and 47.6 (29.6–76.5) per 100,000 PY, respectively. The ASR for all cancers in total population, men and women was 232 and 242 per 100,000 PY, respectively. In men, the highest cancer ASR was lung cancer (116 per 100,000 PY), followed by hepatocellular carcinoma (HCC) (109 per 100,000 PY), and colon cancer (6.1 per 100,000 PY). In women, the highest cancer ASR was cervical carcinoma (401 per 100,000 PY), followed by breast cancer (220.2 per 100,000 PY), and lung cancer (98.7 per 100,000 PY) (Table 1). In multivariate analysis, age >35 years (aHR 2.66, 95% CI 1.75–4.03, *p* < 0.001), AIDS event (aHR 2.10, 95% CI 1.30–3.39, *p* = 0.002) and hepatitis C (aHR 1.46, 95% CI 0.85–3.39, *p* = 0.002) were significantly associated with an increased risk of overall cancer risk. Older age (aHR 4.93), AIDS event (aHR 2.96), HCV co‐infection (aHR 7.32) and HBV coinfection (aHR 4.78) were associated with an increased risk of HCC. The mortality rate was remarkably high (78.8% [95% CI 67.8–88.0]).

**P308: Table 1:** Data of age‐standardised incidence rates per 100,000 person‐years by gender and cancer sites among general population and people with HIV (PWH)

Cancer type	Male general population	Male PWH	Female general population	Female PWH
Any cancer	254	232	322	242
Cervical cancer			33.1	401
Non‐Hodgkin lymphoma	11.8		9.5	
Kaposi's sarcoma	0.1		0.1	
Hepatocellular carcinoma	23.2	109	8.9	7.3
Cholangiocarcinoma	8.7		5.8	
Lung cancer	29.1	116	16.7	98.7
Breast cancer			96.9	220.2
Hodgkin lymphoma	0.8		0.7	
Colon & rectum cancer	43.4	6.1	35.2	14.5
Leukaemia	13		10.2	
Melanoma	0.4		0.9	
Nasopharyngeal carcinoma	3.8		1.4	

Abbreviations: aHR, adjusted hazard ratio; HBsAg, hepatitis B surface antigen; HPV, human papillomavirus; IQR, interquartile range.

**Conclusions**: In this Asian PWH cohort, cancer risks and mortality rate, especially hepatitis virus‐ or HPV‐related cancer, were notably high. Efforts of cancer prevention, screening and better accessibility to treatment are warranted.

### P309 Screening for anal precancerous lesion and cancer in Japan

Naokatsu Ando, Daisuke Mizushima, Misao Takano, Hiroshi Kitamura, Daisuke Shiojiri, Seitaro Abe, Akira Kawashima, Ryo Kuwata, Takato Nakamoto, Takahiro Aoki, Shinichi Oka, Hiroyuki Gatanaga

AIDS Clinical Center, National Center for Global Health and Medicine, Tokyo, Japan

**Background**: Screening for anal cancer among patients living with HIV is increasingly recognised because HIV is one of the most significant risk factors, and interventions for precancerous lesions reduce the development of anal cancer [1‐3]. This study reports the results of initial screening with anal Pap smear (APS) and HPV genotype, including precancerous and cancerous lesions.

**Method**: Between May 2019 and July 2023, men who have sex with men (MSM) aged 18 and older underwent APS and HPV genotyping at the National Centre for Global Health and Medicine, Japan. If APS showed atypical squamous cells of undetermined significance (ASC‐US) or worse and high‐risk HPV was positive, participants were recommended to receive a biopsy under high‐resolution anoscopy (HRA). Comprehensive biopsies, defined as at least six different sites regardless of the presence or absence of visible abnormalities, were conducted in this study.

**Results**: A total of 842 individuals were included, with a median age of 44 (interquartile range 36–51). Among them, 612 (72.7%) participants were living with HIV, and 230 (27.3%) were without HIV. Initial screening showed that 353 (41.9%) had APS results of ASC‐US or worse. HPV genotyping revealed that 518 (61.5%) were positive for high‐risk HPV and 138 (16.5%) for HPV 16. Combined APS and HPV genotype screening indicated that 568 (67.5%) individuals had ASC‐US or more abnormalities or high‐risk HPV. Following the initial screening, 317 (37.6%) participants underwent biopsy with HRA, of whom 262 (82.6%) were living with HIV and 55 (17.4%) without HIV. Biopsy results showed one case (0.3%) of squamous cell carcinoma, 110 (34.7%) cases of anal intraepithelial neoplasia (AIN), 3, 134 (42.3%) cases of AIN2, 65 (20.5%) cases of AIN1 and seven (2.2%) with no abnormality as the worst result among the six biopsy sites.

**Conclusions**: Initial screening with combined APS and HPV genotype indicated that two in three people required detailed screening using HRA. Furthermore, secondary screening revealed that three in four people needed interventions to prevent the development of anal cancer. Given the high demand for testing, it is essential to establish a robust screening system to adequately address and manage this demand.

**References**

1. Stier EA, Clarke MA, Deshmukh AA, Wentzensen N, Liu Y, Poynten IM, et al. International Anal Neoplasia Society's consensus guidelines for anal cancer screening. Int J Cancer. 2024;154(10):1694‐1702.

2. Clifford GM, Georges D, Shiels MS, Engels EA, Albuquerque A, Poynten IM, et al. A meta‐analysis of anal cancer incidence by risk group: toward a unified anal cancer risk scale. Int J Cancer. 2021;148(1):38‐47.

3. Palefsky JM, Lee JY, Jay N, Goldstone SE, Darragh TM, Dunlevy HA, et al. Treatment of anal high‐grade squamous intraepithelial lesions to prevent anal cancer. N Engl J Med. 2022;386(24):2273‐82.

### P310 Effectiveness of trichloroacetic acid for the treatment of HPV‐related anal high‐grade squamous intraepithelial lesions

Jaime Vega‐Costa ^1^, Ana Silva‐Klug^1^, Mónica Sánchez^1^, Loris Trenti^2^, Maria Saumoy^1^

^1^HIV and STI Unit (Infectious Disease Department), Hospital Universitari de Bellvitge, Hospitalet de Llobregat, Spain. ^2^Colorectal Unit (General and Digestive Surgery Department), Hospital Universitari de Bellvitge, Hospitalet de Llobregat, Spain

**Background**: Treating anal high‐grade squamous intraepithelial lesions (H‐SIL) reduces the incidence of anal cancer (AC) among high‐risk population [1]. Currently, several treatments are available. The aim of our study is to describe the effectiveness of trichloroacetic acid 90% (TCA), a well‐tolerated therapy.

**Materials and methods**: Patients attending our anal dysplasia screening program with biopsy‐proven H‐SIL (index lesion/s) in the previous 3 months were eligible. Exclusion criteria: H‐SIL treatment in the previous 6 months, disseminated anogenital warts and/or H‐SIL (>2 quadrants) and active AC. Three doses of TCA were applied monthly through high‐resolution anoscopy (HRA). HRA with index lesion/s biopsy was carried out 6 and 12 months after initial treatment. If H‐SIL persisted at month 6, three additional doses of TCA were applied. Conversely, no HSIL detected in index lesion(s) was considered complete index H‐SIL clearance (CILC). Metachronous lesions were defined as incident H‐SIL in a different location. The primary endpoint was CILC at 12 months.

**Results**: Between January 2021 and December 2022, 63 participants were enrolled in Bellvitge Hospital (Spain). Mean age was 47, 10.9% were female, all had undetectable HIV viral load, and mean CD4 count was 836. Fifty‐eight patients received TCA and 54 reached month 6, where CILC was observed in 33/54 (61.1%); H‐SIL persisted in 21/54 cases (38.9%), requiring a second round of TCA. Among CILC cases, 27 reached month 12, of which 81.5% (22/27) showed maintained CILC, while 5/27 (18.5%) had developed a recurrent H‐SIL in the index lesion. Among 21 patients who underwent two rounds of TCA, 13 reached month 12 HRA, of whom 11/13 (84.6%) showed CILC after the second treatment and 2/13 (15.4%) had H‐SIL persistence. No cases of AC were diagnosed. To sum up, 33 out of 40 patients completing follow‐up (82.5%) experienced CILC after either one or two rounds of TCA. Fourteen metachronous lesions were diagnosed. TCA‐related adverse events were mild when present. There was 36.5% (23/63) of withdrawal mostly due to warts, H‐SIL extension, attrition and change of healthcare centre.

**Conclusion**: Up to 82.5% of H‐SIL treated either once or twice with three applications of TCA were cleared at 12 months.

**Reference**

1. Palefsky JM, Lee JY, Jay N, Goldstone SE, Darragh TM, Dunlevy HA, et al; ANCHOR Investigators Group. Treatment of anal high‐grade squamous intraepithelial lesions to prevent anal cancer. N Engl J Med. 2022;386(24):2273‐82.

### P311 Higher prevalence of high‐risk HPV genotypes and lesions in the youngest: do IANS guidelines align with clinical findings?

Elena Bruzzesi ^1^, Sara Diotallevi^2^, Federica Gandini^1^, Riccardo Lolatto^2^, Massimo Cernuschi^2^, Caterina Candela^1^, Angelo Roberto Raccagni^1^, Flavia Passini^1^, Andrea Marco Tamburini^3^, Roberto Burioni^4^, Antonella Castagna^1^, Silvia Nozza^1^

^1^Infectious Disease Unit, Vita‐Salute San Raffaele University, Milan, Italy. ^2^Infectious Disease Unit, IRCCS San Raffaele Scientific Institute, Milan, Italy. ^3^Gastrointestinal Surgery Unit, IRCCS San Raffaele Scientific Institute, Milan, Italy. ^4^Laboratory of Microbiology and Virology, IRCCS San Raffaele Scientific Institute, Milan, Italy

**Background**: International Anal Neoplasia Society (IANS) consensus guidelines suggest starting screening for anal cancer at age 35 for people with HIV (PWH) or at 45 for those without [1]. Aims of this study is to assess to determine prevalence and cytological abnormalities stratified by age category and to assess risk factors for infection from high‐risk (HR) human papillomavirus (HPV) genotype at anal sites.

**Methods**: Retrospective cohort analysis in men who have sex with men (MSM) living with and without HIV in care at IRCCS San Raffaele, Milan, Italy, with ≥1 HPV test on anal swabs collected from 2014–2023 before vaccine with Gardasil‐9. Twenty‐eight HPV genotypes detection was performed on anal samples by multiplex real‐time PCR (CFX96™, Seegene). Characteristics were compared using the Mann‐Whitney or Chi‐Square/Fisher's tests. Univariable and multivariable logistics regressions are used to estimate risk factors associated with presence of HR‐HPV.

**Results**: Among 1577 people who have performed at least one HPV test, 563 were to be vaccinated at testing; 506 (90%) are living with HIV and 57 (10.1%) in care for PreP, with a median age of 37.4 [31.8– 42.2] at testing. No substantial difference was found in terms of immunovirological characteristics comparing people <35 years to the older ones. HPV infection by HR serotypes (including HPV‐18) and by serotypes covered in Gardasil‐9 was more frequent in the younger; notably, HPV‐16 was detected in 162 (28.8%) with similar distribution. Furthermore, cytological abnormalities were more frequent among MSM <35 years; however, the prevalence of high‐grade lesion was higher among MSM >35 years (Table 1). At multivariate regression, the risk of ≥1 HPV‐HR genotype was associated with younger age and concomitant sexually transmitted infection, including HCV, HBV, gonorrhoea, ureaplasma, chlamydia, syphilis, mpox, HSV (Table 2).

**P311: Table 1:** Prevalence of HPV genotypes and of cytological abnormalities, stratified by age

	Total *N* = 563	<35 *N* = 217	≥35 *N* = 346	*p*‐value
Age	37.4 [31.8‐42.2]	30.2 [27.9‐32.7]	41.1 [38.2‐44.6]	<0.001
People with HIV	506 (89.9%)	192 (88.5%)	314 (90.8%)	0.468
PWH with nadir CD4+ ≤ 200 cells/microL (cells/microL)	74 (14.8%)	20 (10.6%)	54 (17.3%)	0.057
Test positive for HR‐HPV	521 (92.5%)	210 (96.8%)	311 (89.9%)	0.004
Test positive for HPV included in Gardasil‐9	448 (79.6%)	185 (85.3%)	263 (76.0%)	0.011
Cytological abnormalities				0.033
Negative	429 (83.5%)	157 (81.8%)	272 (84.5%)	
Atypical squamous cells of undetermined significance	12 (2.33%)	9 (4.69%)	3 (0.93%)	
Low grade squamous intraepithelial lesion	48 (9.34%)	19 (9.90%)	29 (9.01%)	
High grade squamous intraepithelial lesion	10 (1.78%)	1 (0.52%)	9 (2.60%)	
Condilomata/infection	15 (2.66%)	6 (3.12%)	9 (2.60%)	

**P311: Table 2:** Multivariable logistic regression models to assess risk factors for having at least one HR‐HPV genotype

Variable	Category	Adjusted odds ratio	95% CI	*p*‐value
Age	35 vs <35	0.34	0.18–0.58	<0.001
Smoke	Yes vs no	0.89	0.66‐1.21	0.46
Concomitant STI	Yes vs no	1.68	1.09‐2.67	0.023
Previous STI	Yes vs no	1.31	0.94‐1.84	0.12
CD4 nadir	Per 100 cells/µL higher	1.04	0.97‐1.12	0.27
CD4 count	Per 100 cells/µL higher	1.00	0.94‐1.06	0.95
HIV‐RNA	Detectable vs undetectable	1.05	0.6‐1.98	0.87

**Conclusions**: We showed a high prevalence of HR‐HPV serotypes and of low‐grade lesions before vaccination, especially among younger MSM. Given the benefit of early treatment, we believe that studies to better identify target populations that could benefit from a more personalised screening, especially in MSM without HIV, are warranted.

**Reference**

1. Stier EA, Clarke MA, Deshmukh AA, Wentzensen N, Liu Y, Poynten IM, et al. International Anal Neoplasia Society's consensus guidelines for anal cancer screening. Int J Cancer. 2024;154(10):1694‐702.

### P312 Cancers in people living with HIV: an observational study in the cohort of Modena over 27 years

Federica Prandini, Marianna Menozzi, Federica Casari, Deborah Lusetti, Beatrice Fontana, Martina Ricciardetto, Elena Ghidoni, Filippo Calandra Buonaura, Elena Martini, Giovanni Guaraldi, Cristina Mussini

Infectious Disease Clinic, University Hospital of Modena, Azienda Ospedaliero Universitaria Policlinico di Modena, Modena, Italy

**Background**: Nowadays, the life expectancy of people living with HIV (PLWH) is similar to the general population. Simultaneously, the prevalence of comorbidities increased, including neoplastic diseases, one of the main causes of death and more frequent in people living with HIV due to immunosuppression and inflammatory state. The aim of the study was to describe cancer prevalence, deaths and 5‐year survival in PLWH in our centre.

**Material and methods**: We retrospectively included all PLWH at Modena Clinic who developed cancer from 1996 to 2023, excluding those diagnosed with cancer prior to acquiring HIV. We analysed demographics, survival characteristics and tumour types, examining the frequency of cancer diagnosis over time. Following descriptive analysis, logistic regression identified factors contributing to death and 5‐year survival.

**Results**: In the study period, 258 people developed 309 tumours. The majority were men (71.5%) and the primary mode of HIV transmission was condomless sex (58.2%), followed by intravenous drug use (26.6%). Of these, (51.9%) had received an AIDS diagnosis, with prevalent AIDS‐defining conditions including Kaposi's sarcoma (KS) (31.3%), Hodgkin's lymphoma (18.7%) and Pneumocystis pneumonia (9%). The most frequently observed cancers were haematological malignancies (17.9%), KS (15.3%), and genitourinary tract tumours (14.9%). Ninety‐one deaths occurred (35.3%), predominantly due to cancer (80.2%). Among 309 total diagnosed tumours, 36% were AIDS‐defining cancers (ADM), and 64% were non‐AIDS‐defining cancers (NADM). Furthermore, a progressive increase in annual cancer diagnosis incidence was observed over time, with a higher incidence of NADM compared to ADM (Figure 1). Table 1 outlines the primary risk factors influencing mortality and 5‐year survival: individuals with AIDS diagnosis had elevated mortality risk (*p* = 0.009), while those with undetectable HIV RNA at cancer diagnosis showed improved 5‐year survival (*p* = 0.051). Lung cancer carried the highest mortality risk (*p* = 0.012), whereas human papillomavirus (HPV)‐related cancers presented the lowest risk (*p* = 0.004). The type of antiretroviral regimen administered at cancer diagnosis did not impact outcomes.

**P312: Figure 1:**
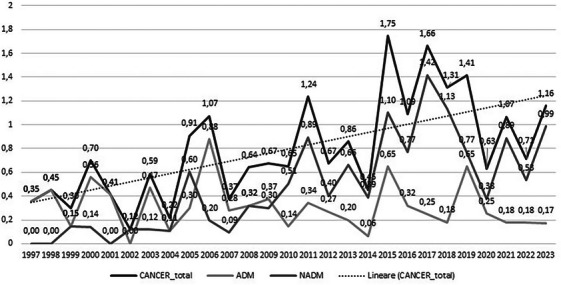
Cancer annual incidence. ADM, AIDS‐defining malignancy; NADM, non‐AIDS‐defining malignancy.

**P312: Table 1:** Logistic regression analysis shows factors contributing to mortality and 5‐year survival

	Outcome death: OR	Outcome death: 95% CI	Outcome death: *p*‐value	Outcome 5‐year survival: OR	Outcome 5‐year survival: 95% CI	Outcome 5‐year survival: *p*‐value
AIDS	3.42	1.36–8.62	0.009	1.32	0.45–3.86	0.613
Years of HIV	1.06	1.01–1.10	0.009	0.92	0.88–0.97	0.002
Undetectable HIV RNA at cancer diagnosis	0.41	0.16–1.05	0.061	3.48	0.97–12.5	0.051
CD4/CD8 ratio at cancer diagnosis	1.22	0.55–2.70	0.626	1.60	0.62–4.08	0.334
INSTI ARV at cancer diagnosis	0.51	0.07–3.88	0.518	0.30	0.04–2.49	0.266
PI ARV at cancer diagnosis	0.51	0.61–4.12	0.524	0.62	0.07–5.22	0.661
NNRTI ARV at cancer diagnosis	0.67	0.08–5.56	0.707	0.42	0.05–3.64	0.431
Kaposi's sarcoma	0.50	0.14–1.82	0.091	0.57	0.12–2.77	0.488
Gastro‐intestinal cancer	2.75	0.57–13.2	0.234	1.55	0.23–10.6	0.653
HPV‐related cancer	0.18	0.04–0.81	0.004	4.28	0.86–21.3	0.076
Liver cancer	0.77	0.09–6.86	0.223	2.75	0.25–32.3	0.420
Lung cancer	28.1	2.11–374.1	0.012	0.06	0.01–0.81	0.035
Genito‐urinary cancer	0.81	0.21–3.03	0.749	1.69	0.32–8.98	0.540
Breast cancer	0.65	0.13–3.23	0.600	3.61	0.43–30.7	0.239
Bone cancer	−	−	−	−	−	−
Head and neck cancer	0.63	0.12–3.30	0.284	9.97	1.15–86.8	0.121
Other	1.23	0.26–5.82	0.790	3.89	0.69–21.7	−
Years of cancer diagnosis	1.01	0.92–1.08	0.975	0.70	0.62–0.80	<0.001
Age at cancer diagnosis	1.03	0.99–1.06	0.162	0.99	0.96–1.03	0.824
Metastasis	6.50	2.99–14–1	<0.001	0.25	0.09–0.65	0.05

Abbreviations: ARV, antiretroviral; INSTI, integrase inhibitor; NNRTI, non‐nucleoside reverse transcriptase inhibitor; PI, protease inhibitor.

**Conclusions**: Consistent with the literature, our cohort observed an increasing incidence of cancer in PLWH over time, with a rise in NADM compared to ADM. These findings underscore the importance of implementing strategies to prevent cancer onset and enable early diagnosis.

### P313 Diagnostic accuracy of non‐invasive markers for biopsy‐proven high‐grade anal dysplasia

David Chromy ^1^, Steffi Silling^2^, Alexander Kreuter^3^, Anja Potthoff^4^, Nathalie Judith Auer^5^, Dirk Schadendorf^5^, Ulrike Wieland^2^, Stefan Esser^5^

^1^Department of Dermatology, Medical University of Vienna, Vienna, Austria. ^2^Institute of Virology, National Reference Center for Papilloma‐ and Polyomaviruses, Faculty of Medicine and University Hospital Cologne, University of Cologne, Cologne, Germany. ^3^Department of Dermatology, Venereology and Allergology, Helios St. Elisabeth Hospital Oberhausen, University of Witten/Herdecke, Oberhausen, Germany. ^4^Interdisciplinary Immunological Outpatient Clinic, Center for Sexual Health and Medicine, Department of Dermatology, Venerology and Allergology, Ruhr‐University Bochum, Bochum, Germany. ^5^Department of Dermatology and Venereology, University Hospital Essen, University Duisburg‐Essen, Essen, Germany

**Background**: The highest burden of anal cancers is observed among people with HIV and is mainly caused by high‐risk (HR) human papillomavirus (HPV) types. Men who have sex with men (MSM) experience an up to 100‐fold increased risk for anal cancer. Since 2024, international guidelines recommend screening and treatment of anal cancer precursor lesions, i.e. high‐grade squamous intraepithelial lesions (HSIL). The gold standard for diagnosing HSIL is biopsy/histology (hHSIL), which is obtained during high‐resolution anoscopy (HRA). However, the capacity for HRA is limited, and thus, screening recommendations include non‐invasive diagnostics via anal sampling for HR‐HPV and/or anal cytology, allowing a pre‐selection prior to HRA. Since the diagnostic performances of anal cytology and qualitative HR‐HPV testing are considered suboptimal, alternative non‐invasive markers are required. We, therefore, initiated in 2023 a diagnostic accuracy study to assess the composite diagnostic performance of anal cytology, HR‐HPV, oncogenic E6/E7‐mRNA expression, and host‐cell methylation markers for hHSIL.

**Materials and methods**: MSM with HIV undergoing HRA at the Department of Dermatology at three German and one Austrian University Hospital are included. Before HRA, anal swabs were obtained for cytology and non‐invasive markers. Abnormal findings detected during HRA were biopsied for histologic evaluation. In this abstract, we present preliminary data on the diagnostic performance of cytology and HPV‐typing for hHSIL comprising the first 155 individuals.

**Results**: Among 238 biopsies obtained from 155 individuals, 31% (74/238) hHSIL—including one American Joint Committee on Cancer (AJCC) stage I carcinoma—were diagnosed in 38% (59/155) of patients. The cytology cut‐off for abnormal findings at ‘ASC‐US’ (atypical cells of undetermined significance) achieved a sensitivity of 67.8% and specificity of 62.5% for hHSIL. Sixty‐six percent (102/155) of the swabs tested positive for at least one HR‐HPV‐type, demonstrating a sensitivity and specificity of 86.4% and 46.9% for solely HR‐HPV and 61.0% and 75.0% for the composite analysis of ASC‐US + HR‐HPV, respectively.

**Conclusions**: In this preliminary analysis, the currently established non‐invasive diagnostics (cytology and/or HR‐HPV) demonstrated unsatisfactory diagnostic accuracy for the detection of anal pre‐cancers in this highly‐burdened population. Further research on anal biomarkers is required to improve non‐invasive screening tools for anal cancer prevention.

### P314 High‐resolution anoscopy referral rates when adopting IANS consensus guidelines for anal cancer screening in men who have sex with men living with HIV

Christof Stingone ^1^, Maria Benevolo^2^, Francesca Rollo^2^, Eugenia Giuliani^1^, Massimo Giuliani^1^, Paolo Giorgi Rossi^3^, Laura Gianserra^1^, Mauro Zaccarelli^1^, Alessandra Latini^1^, Maria Gabriella Donà^1^

^1^STI and HIV Unit, San Gallicano Dermatological Institute IRCCS, Rome, Italy. ^2^Pathology Department, IRCCS Regina Elena National Cancer Institute, Rome, Italy. ^3^Epidemiology Unit, Azienda Unità Sanitaria Locale‐IRCCS di Reggio Emilia, Reggio Emilia, Italy

**Background**: The International Anal Neoplasia Society (IANS) has generated recommendations for anal cancer prevention, identifying MSM living with HIV (MSM‐LWH) ≥35 years as a priority group for the screening. Since high‐resolution anoscopy (HRA) availability is still limited across Europe, a retrospective study was conducted to estimate the potential HRA referral rates of our STI/HIV centre (Rome, Italy) using IANS‐recommended screening strategies.

**Materials and methods**: MSM‐LWH ≥35 years with valid results for liquid‐based anal cytology and human papillomavirus (HPV) tests were included. The strategies evaluated were: cytology alone or with high‐risk (hr) HPV triage; hrHPV (with/without HPV16 genotyping) alone or with cytology triage; co‐testing with cytology and hrHPV (with/without HPV16 genotyping).

**Results**: Of the 1400 MSM attending the centre during the study period (May 09–March 23), 244 MSM‐LWH were included [median age: 45 years (IQR 40–51); median time since HIV diagnosis: 6.5 years (IQR 2.8–12.9); median nadir and baseline CD4+: 308 (IQR 201–400) and 610 cells/mm^3^ (IQR 441–804), respectively]; 231 MSM (94.7%) were on cART, and 200 of them (86.6%) had undetectable HIV‐RNA. HrHPV as a standalone test led to the highest referral rate (74.6%). Strategies with triage resulted in the same and lowest referral rates (44.3%). In settings with insufficient HRA capacity, only atypical squamous cells—possible high‐grade lesion (ASC‐H)/High grade squamous intraepithelial lesion (HSIL) (4.9%) and HPV16+ MSM (27.0%) —would be referred to HRA.

**Conclusions**: Despite the high clinical sensitivity of the hrHPV test, its high referral rate is a matter of concern in settings with limited HRA capacity. Triage strategies with HPV genotyping would help prioritise MSM with the highest risk for anal pre‐cancer and avoid overwhelming HRA services.

### P315 Shifting patterns and outcomes of malignancies in people living with HIV: a 10‐year retrospective study

Susana Ramos Oliveira ^1^, Mafalda Teixeira Costa^2^, Cristina Soeiro^1^, Clara Bacelar^1^, Clara Batista^1^, Sara Vale Araújo^1^, Mário Guimarães^1^, Constança Azeredo^1^, Frederico Duarte^1^, Sónia Duarte Rocha^1^, Fábio Reis^1^, Eduarda Ruiz Pena^1^, Maria João Gonçalves^1^, Ricardo Correia de Abreu^1^, Sofia Jordão^1^

^1^Infectious Diseases Department, Pedro Hispano Hospital, Local Health Unit Matosinhos, Senhora da Hora, Portugal. ^2^Oncology Department, Pedro Hispano Hospital, Local Health Unit Matosinhos, Coimbra, Portugal

**Background**: People living with HIV (PLWH) are at greater risk of developing cancer, whether it is AIDS‐defining (ADC) or non‐AIDS‐defining (NADC), and the oncogenic role of HIV is not fully elucidated. The authors hypothesise that the epidemiology of malignancies in this population may have changed after the official recommendations of early initiation of antiretroviral therapy for all patients (2015).

**Materials and methods**: Retrospective analysis of the invasive malignancies diagnosed in PLWH under follow‐up in a tertiary hospital between 2014 and 2023. Statistical analysis was performed using SPSS, using Chi‐square and Mann‐Whitney tests accordingly.

**Results**: In a total of 1436 PLWH, 8% (*n* = 119) had at least one malignancy diagnosed between 2002 and 2024: 4% with HIV‐2 infection, median age at HIV diagnosis of 46 years [14–78], 80% *cis*‐man, and 60% current or previous smokers. Fourteen patients (12%) were diagnosed with two distinct malignancies, in an interval of less than 5 years in 79% of cases. The majority (82%) of the malignancies were NADC, with a significant decrease in the incidence of ADC in the period between 2015 and 2024 (37% vs 11%, *p* < 0.005). The median years between the diagnosis of HIV and the malignancy were significantly lower in the 2002–2014 period (4 vs 12, *p* < 0.005). There was no significant correlation between CD4+ count, CD4+ nadir, or viral suppression at the diagnosis and limitation to palliative treatment. Twenty‐three percent of the malignancies (31/133) received palliative treatment or best supportive care only. The patients who underwent curative treatment at diagnosis presented 1‐year and 5‐year mortality of 17% and 25%, respectively, and 45.5% were officially cured, while 28% are still under follow‐up. The global mortality for all causes was 33% at 1 year and 41% at 5 years. No significant differences in mortality were found between the period before and after 2015, ADCs and NADCs, and haemato‐oncology neoplasms and others.

**Conclusions**: PLWH have a high incidence of malignancies, with a significant increase of NADCs in the latter years. Even with virological suppression and better immunological status, morbimortality remains high, which may allude to the necessity of systematic cancer screening in this population.

## Co‐morbidities and complications of disease and/or treatment—Neurological

### P316 Cognitive performance and patient‐reported outcomes in older people with HIV in the GEPPO cohort

Federica Barrera ^1^, Andrea Calcagno^1^, Andrea Tommasi^2^, Lavinia Patetta^3^, Jovana Milic^4^, Alessandra Coin^5^, Chiara Mussi^6^, Stefano Calza^7^, Benedetto Maurizio Celesia^8^, Samuele Gardin^9^, Domenico Azzolino^10^, Elisa Lenotti^11^, Micol Ferrara^1^, Benedetta Fioretti^12^, Giordano Madeddu^13^, Giancarlo Orofino^14^, Emanuele Focà^12^, Giovanni Guaraldi^15^

^1^Unit of Infectious Diseases, Department of Medical Sciences, University of Turin, Turin, Italy. ^2^Department of Infectious Diseases, Azienda Ospedaliero‐Universitaria di Perugia, Perugia, Italy. ^3^Geriatric Unit, Fondazione IRCCS Ca' Granda Ospedale Maggiore Policlinico di Milano, Milan, Italy. ^4^Department of Biomedical and Metabolic Sciences and Neurosciences, University of Modena and Reggio Emilia, Modena, Italy. ^5^Geriatric Unit, University of Padova, Padova, Italy. ^6^Centre of Gerontological Evaluation and Research, University of Modena and Reggio Emilia, Modena, Italy. ^7^Unit of Biostatistics and Bioinformatics, Department of Molecular and Translational Medicine, University of Brescia, Brescia, Italy. ^8^Division of Infectious Diseases, Department of Clinical and Molecular Biomedicine, University of Catania, ARNAS Garibaldi, Catania, Italy. ^9^Unit of Infectious Diseases, Department of Internal Medicine, Azienda Ospedaliero‐Universitaria di Padova, Padova, Italy. ^10^Geriatric Unit, Fondazione IRCCS Ca' Granda Ospedale Maggiore Policlinico di Milano, Milano, Italy. ^11^Geriatric Unit, University of Brescia and ASST Spedali Civili Hospital, Brescia, Italy. ^12^Department of Infectious and Tropical Diseases, University of Brescia and ASST Spedali Civili Hospital, Brescia, Italy. ^13^Unit of Infectious and Tropical Diseases, Department of Medical, Surgical and Experimental Sciences, University of Sassari, Sassari, Italy. ^14^Unit of Infectious Diseases, ‘Divisione A’, Amedeo di Savoia Hospital, ASLTO2, Turin, Italy. ^15^Department of Surgical, Medical, Dental and Morphological Sciences, University of Modena and Reggio Emilia, Modena, Italy

**Background**: Older people living with HIV (OPWH) display multiple risk factors that may contribute to lower cognitive performance. Recent data reported a worrisomely higher risk of dementia in OPWH than the general population. This study aims to describe cognitive performance and patient‐reported outcomes (PROs) in OPWH in the GEPPO cohort.

**Methods**: This was a cross‐sectional study of participants enrolled in the GEPPO cohort who were assessed for cognitive performance with the Mini‐Addenbrooke Cognitive Examination test (MACE) and the Grooved Pegboard Test (GPT). Impaired cognitive performance (ICP) was defined as MACE score ≤21 and motor speed ≥2 SD of normative values of non‐dominant hand (ndh‐GPT). We measured anxiety and depressive symptoms (Hamilton Anxiety Rating Scale [HAM‐A] and Centre for Epidemiologic Studies Depression Scale [CES‐D]), quality of life (EuroQol 5‐Dimension 5‐Level [EQ‐5D‐5L]) and sleep quality (Pittsburgh Sleep Quality Index [PSQI]). Multimorbidity (MM) was defined as the presence of at least three comorbidities and polypharmacy (PP) as the use of >5 drugs. The anticholinergic burden (ACB) was measured using the ACB score and categorised as < or ≥3.

**Results**: We included 240 OPWH; mean age was 73.7 years (±5.3) and 204 were male (85%). The mean CD4 count was 647 (±307) and HIV RNA was <200 copies/mL in 228 (95%). Most used ARV classes were INIs (179, 74.5%), NNRTIs (33, 13.7%), or PIs (22, 9.1%). MM, PP and ACB ≥3 were observed in 40 (17%), 70 (29%), and 13 (5.4%) participants. Average PRO scores were: 7 (±8) for HAM‐A, 13 (±7) for CES‐D, 7.9 (±4.6) for PSQI and 0.77 (±0.23) for EQ‐5D‐5L. MACE and GPT were abnormal in 60 (25%) and 45 (18.7%) participants, respectively; 22 (9.2%) had both altered tests. ICP was more common in females (41 vs 13%, *p* = 0.001), with no other statistically significant differences. PRO scores (either as continuous or stratified variables) did not differ according to cognitive performance. No differences were observed among drug classes or single ARV drugs.

**Conclusion**: Despite the cross‐sectional design and the use of screening tests, preliminary data suggest that impaired cognitive or motor function affects approximately 1/4 of OPWH and that only 9.2% of participants had ICP. The finding of a higher prevalence of ICP in older female participants suggests that gender‐based screening programs need to be implemented. Longitudinal studies are needed in order to assess the effect of antiretroviral and non‐antiretroviral drugs on cognitive function.

### P317 The association between anticholinergic medication use and cognitive function in older people with HIV in the Pharmacokinetic and clinical Observations in PeoPle over fiftY (POPPY) study

Saumitro Deb ^1^, Jessica Doctor^2^, Jaime Vera^3^, Frank Post^4^, Jasmini Alagaratnam^5^, Patrick WG Mallon^6^, Jane Anderson^7^, Laura Waters^8^, Nicki Doyle^9^, Fiona Burns^10^, Memory Sachikonye^11^, Alan Winston^9^, Caroline Sabin^1^

^1^Institute for Global Health, University College London, London, UK. ^2^Guy's and St Thomas' NHS Foundation Trust, London, UK. ^3^University Hospitals Sussex NHS Foundation Trust, Brighton, UK. ^4^King's College Hospital NHS Foundation Trust, London, UK. ^5^Chelsea and Westminster Hospital NHS Foundation Trust, London, UK. ^6^University College Dublin, Dublin, Ireland. ^7^Homerton University Hospital, London, UK. ^8^Central and North West London NHS Foundation Trust, London, UK. ^9^Imperial College London, London, UK. ^10^Infection and Population Health, University College London, London, UK. ^11^UK Community Advisory Board (UK‐CAB), London, UK

**Background**: Evidence suggests that the use of anticholinergic medication (ACM) is associated with several negative health outcomes, including falls and frailty. As ACM are commonly used in people with HIV as they age, we examine associations of the use of these drugs with measures of cognitive function in people aged >50 years participating in the POPPY study.

**Methods**: At study baseline, participants completed a cognitive assessment covering six domains (visual learning, psychomotor, visual attention, executive function, verbal learning, working memory). Raw test scores were standardised into T‐scores (mean 0, SD 1) and averaged to obtain global/domain‐specific T‐scores. Co‐medications scoring ≥1 on the Anticholinergic Burden, Anticholinergic Risk, or Scottish Intercollegiate Guidelines Network scales were identified as ACM drugs. Associations of ACM use with global/domain‐specific T‐scores and binary classifications of cognitive impairment (Frascati, Gisslen, Global Deficit Score, Multivariable Normative Comparison) were assessed using linear/logistic regression before and after adjustment for age, sex, ethnicity, marital status, educational attainment, employment, alcohol use, recent recreational drug use, smoking, depressive symptoms (Patient Health Questionnaire, PHQ‐9), number of co‐medications and number of comorbidities.

**Results**: Among the 608 participants with a complete cognitive assessment (89.3% male, 87.8% white ethnicity, median age 57 (interquartile range 53–62)), 171 (28.1%) reported ACM use (median 1, range 1–6 drugs), with users being more likely to be of white ethnicity (*p* = 0.01), to not be working, to have depressive symptom scores and to be on a greater number of co‐medications with more comorbidities (*p* < 0.0001 for each) than non‐users. The mean [SD] global T‐score was 48.32 [5.66] with no significant difference between those reporting (48.00 [6.30]) and not reporting (48.50 [5.40]) ACM use, either before [mean difference ‐0.50 (95% CI ‐1.51, 0.50), *p* = 0.32] or after [‐0.06 (95% CI ‐1.15, 1.04), *p* = 0.94] adjustment for confounders. No significant associations were seen between ACM use and domain‐specific T‐scores, nor with any binary classification of impairment, after adjustment for confounders.

**Conclusion**: Our findings suggest no strong or consistent associations between commonly reported measures of cognitive function in people with HIV and ACM use; further analyses will explore the contribution of specific ACM drug types in this population.

### P318 Brain functional connectivity changes over 18 months in persons with acute and chronic HIV infection

Charalampos D Moschopoulos ^1^, Evangelia Stanitsa^2^, Efstratios Karavasilis^3^, Konstantinos Protopapas^1^, Dimitra Kavatha^4^, Antonios Papadopoulos^1^, Georgios Velonakis^5^, Sokratis G Papageorgiou^6^, Anastasia Antoniadou^7^

^1^4th Department of Internal Medicine, University General Hospital Attikon, Medical School, National and Kapodistrian University of Athens (NKUA), Athens, Greece. ^2^1st Department of Neurology, Eginition Hospital, Athens, Greece. ^3^School of Medicine, Democritus University of Thrace, Alexandroupolis, Greece. ^4^4th Department of Internal Medicine, University General Hospital Attikon, Athens, Greece. ^5^Research Unit of Radiology, Medical School, National and Kapodistrian University of Athens (NKUA), Athens, Greece. ^6^1st Department of Neurology, Eginition Hospital, Medical School, National and Kapodistrian University of Athens (NKUA), Athens, Greece. ^7^4th Department of Internal Medicine, Attikon University Hospital, Medical School, National and Kapodistrian University of Athens (NKUA), Athens, Greece

**Background**: HIV enters the central nervous system soon after infection and produces long term effects, even in the cART era, especially for persons who remain untreated for a long period of time. Longitudinal evidence on functional connectivity changes in the course of HIV infection are limited. In this study we evaluate pre‐post cART changes in treatment‐naïve persons with acute or chronic HIV infection.

**Materials and methods**: Newly diagnosed persons with HIV were prospectively enrolled in this study, excluding those with comorbidities relevant to brain or cognitive damage. Participants underwent a comprehensive neuropsychological evaluation covering seven cognitive domains, according to the Frascati criteria, and resting state functional MRI brain imaging at baseline and 18 months on a 3.0T MRI system. Composite z scores per cognitive domain were calculated by averaging individual test scores. Acute HIV was defined as evidence of seroconversion or documented negative testing up to 6 weeks before diagnosis. Neuroimaging functional data pre‐, post‐processing and statistical analyses (statistical threshold: cluster level <0.05 FWE; voxel level <0.001 uncorrected) were performed using the CONN toolbox of Matlab. For between groups, pre‐post analysis 2 x 2 mixed group factorial ANOVA was used.

**Results**: Fifteen individuals (14 males) of white ethnicity with a mean (±SD) age of 36 (±10) years were enrolled. Six persons showed evidence of acute HIV infection. At baseline CD4 count was 441 (±153) and log viral load 5.26 (±1). At follow‐up, all patients had undetectable viral load. Looking into intrinsic functional networks of the whole group, we found longitudinal alterations only in the dorsal attention network. The subgroup of chronic HIV did not show significant changes, whereas analysis between groups and across time identified functional differences in the salience, dorsal attention, and frontoparietal networks. Significant changes from baseline to follow‐up were detected for memory (z‐score 0.45 vs. 0.03, *p* = 0.047) and executive function (‐0.37 vs 0.50, *p* = 0.039).

**Conclusions**: To our knowledge this is the first study to explore longitudinal functional connectivity changes in persons with acute and chronic HIV after cART initiation. Despite the small number of participants, we found significant differences in intrinsic functional networks between the two groups and across time.

## Co‐morbidities and complications of disease and/or treatment—Renal

### P319 Long‐term change of renal function among people with HIV who received TAF‐containing ART

Guan‐Jhou Chen ^1^, Hsin‐Yun Sun^2^, Szu‐Min Hsieh^2^, Wang‐Huei Sheng^2^, Yu‐Chung Chuang^2^, Yu‐Shan Huang^2^, Kuan‐Yin Lin^2^, Wen‐Chun Liu^3^, Yi‐Ching Su^3^, Chien‐Ching Hung^2^

^1^Internal Medicine, Min‐Sheng General Hospital, Taoyuan, Taiwan. ^2^Internal Medicine, National Taiwan University College of Medicine, Taipei, Taiwan. ^3^Internal Medicine, National Taiwan University Hospital, Taipei, Taiwan

**Background**: Among people with HIV (PWH), the real‐world data evaluating the long‐term impact of tenofovir alafenamide (TAF) on renal function remain scarce.

**Methods**: PWH who were initiated or switched to a TAF‐containing antiretroviral therapy (ART) were included in this 5‐year longitudinal follow‐up study at a university hospital in Taiwan. Information was collected on the demographics, baseline CD4 counts, plasma HIV RNA load (PVL), concomitant medications and illnesses and prior exposure to tenofovir disoproxil fumarate (TDF). Estimated glomerular filtration rate (eGFR) in the study were estimated by the Chronic Kidney Disease Epidemiology Collaboration (CKD‐EPI) equation and the levels of proteinuria were estimated by urine protein‐to‐creatinine ratio (UPCR). The association between the duration of TAF exposure and evolution of eGFR was plotted in a locally estimated scatterplot smoothing (LOESS) regression. Factors associated with excess decline of eGFR (defined as an eGFR decline >2.5 ml/min/1.73 m^2^ per year or >25% throughout the observation) were also evaluated in a multivariate logistic regression.

**Results**: In total, 2475 PWH, 96.6% being male with a mean age of 40.6 years were included in the cohort (see Table 1). The median duration of TAF exposure was 226 weeks, and most included PWH had good HIV control. In the LOESS regression model, the estimated changes of eGFR at weeks 48, 96, 144, 192 and 240 were ‐3.8 (95% CI ‐2.3, ‐5.2), ‐5.5 (95% CI ‐4.4, ‐6.6), ‐7.0 (95% CI ‐5.9, ‐8.0), ‐7.3 (95% CI ‐6.3, ‐8.4), ‐8.4 (95% CI ‐7.5, ‐9.3) ml/min/1.73 m^2^, respectively (see Figure 1). The level of proteinuria remained stable throughout the observation and two PWH progressed to end‐stage renal disease during observation and were treated with renal replacement therapy. Higher baseline PVL (adjusted odds ratio [aOR] per 1‐log unit increase 1.42; 95% CI 1.15–1.77), old age (aOR per 1‐year increase 1.03; 95% CI 1.01–1.05), having higher baseline eGFR (aOR per 1‐ml/min/1.73 m^2^ increase 1.02; 95% CI 1.01–1.03) and the presence of proteinuria (aOR 1.56; 95% CI 1.14–2.12) were associated with an excessive decline of eGFR in our cohort.

**P319: Table 1:** Baseline characteristics of the included PWH

Characteristic	Included PWH (*N* = 2475)
Age, mean (SD), years	40.6 (10.7)
Male sex, *n* (%)	2392 (96.6)
PVL at switch, median (IQR), log10 copies/mL	1.3 (1.3‐1.3)
PVL >50 copies/mL at switch, *n* (%)	287 (11.6)
CD4 count at switch, median (IQR), cells/mm^3^	567 (410‐752)
Duration of HIV diagnosis before TAF, median (IQR), years	5.2 (2.4‐8.7)
Average length of TAF exposure in the cohort, median (IQR), weeks	226 (157‐265)
Previous exposure to TDF, *n* (%)	1815 (73.3)
Length of TDF exposure, median (IQR), years	2.4 (0.1‐4.3)
Baseline eGFR (CKD‐EPI) when starting TAF‐containing ART, mean (SD), mL/min/1.73 m^2^	95.8 (19.3)
Diabetes mellitus before enrolment, *n* (%)	269 (10.9)

**P319: Figure 1:**
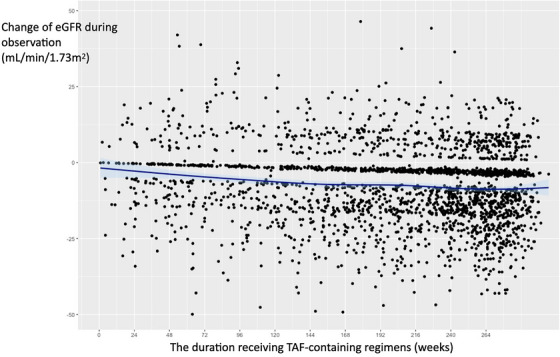
Correlation between the exposure to TAF‐containing regimens and the changes of eGFR among the included PWH.

**Conclusions**: Compared with previous findings associated with prolonged TDF exposure, our study demonstrated only modest reduction of eGFR during the 5‐year exposure to TAF‐containing ART.

### P320 Reduction in estimated glomerular filtration rate (eGFR) observed with doravirine (DOR) is caused by inhibition of organic cation transporter 2 (OCT2)

Yang Li^1^, Xiaoyan Chu^1^, Rosa I Sanchez^1^, Russ P Carstens^2^, Mary L Pisculli^2^, Stephanie Klopfer^3^, Zhi Jin Xu^3^, Rima Lahoulou^4^, Rebeca M Plank ^2^

^1^Pharmacokinetics, Merck & Co., Inc., Rahway, NJ, USA. ^2^Clinical Research, Merck & Co., Inc., Rahway, NJ, USA. ^3^Biostatistics, Merck & Co., Inc., Rahway, NJ, USA. ^4^Clinical Research, Merck Sharp & Dohme, Puteaux, France

**Background**: In three phase III studies of DOR in treatment‐naïve adults with HIV‐1, declines in creatinine‐based eGFR of ∼10 mL/min/1.73 m^2^ were observed within a few weeks of initiating DOR, regardless of concomitant tenofovir use, followed by stable eGFR for up to 3.5 years [1,2]. To assess the possible involvement of renal transporters, we investigated the in vitro interaction of DOR with OCT2 and with multidrug and toxin extrusion protein 1 (MATE1), which are responsible for active renal transport of creatinine.

**Materials and methods**: In vitro assays were established in OCT2‐ and MATE1‐transfected cells using [14C]‐creatinine and metformin as probes. The inhibitory effects of DOR on OCT2‐ and MATE1‐mediated uptake of creatinine and metformin were measured, and the FDA drug interaction risk assessment criteria were applied.

**Results**: Creatinine exhibited uptake ratios of 8‐ and 5‐fold in OCT2‐ and MATE1‐transfected cells, respectively, compared to parental cells. DOR inhibited OCT2‐mediated creatinine uptake (Figure 1a) with a half maximal inhibitory concentration (IC50) of 6.9 µm (∼12‐fold above DOR unbound peak concentration [C_max_]), suggesting that eGFR reduction observed after initiation of DOR may be associated with inhibition of OCT2. When metformin rather than creatinine was used as the OCT2 in vitro probe, a 10‐fold higher IC50 for OCT2 inhibition was observed for DOR (67 µm). As previously demonstrated, DOR does not affect the pharmacokinetics of metformin [3], suggesting that DOR inhibition of OCT2 is substrate dependent. Finally, DOR inhibited only ∼48% of MATE1‐mediated creatinine uptake at 100 µm, which is comparable to the magnitude of inhibition observed for metformin uptake (Figure 1b).

**P320: Figure 1:**
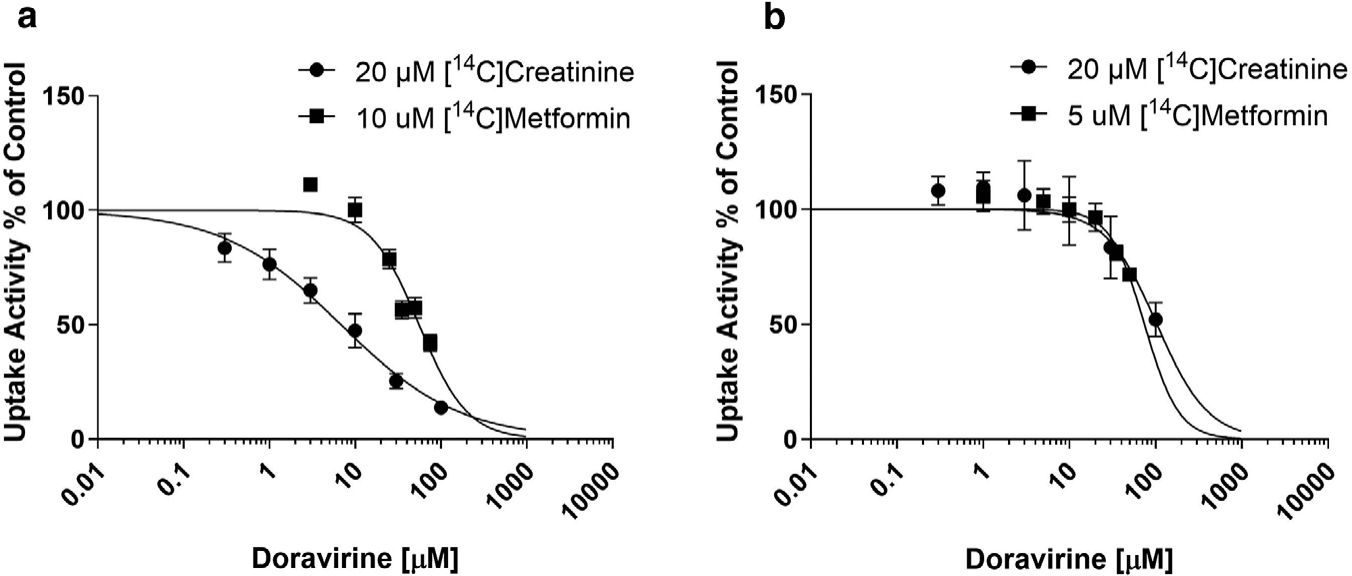
The inhibitory effect of doravirine on OCT2 (a) and MATE1 (b) mediated uptake of creatinine and metformin.

**Conclusions**: At clinically relevant exposures, DOR inhibits OCT2‐ but not MATE1‐mediated creatinine transport. Our in vitro observations mechanistically illustrate that the reduction in creatinine‐based eGFR calculations observed with DOR is caused by inhibition of renal creatinine transport by DOR and does not reflect a reduction in renal function. These findings are consistent with clinical data showing improvement in eGFR, when calculated using cystatin‐C, in treatment‐naïve individuals initiating treatment with the DOR/islatravir combination [2]. Inhibition of renal creatinine transporters has been seen with other drugs, including antiretrovirals, and such effects are reversible with removal of the drug [4].

**References**

1. Orkin C, Molina JM, Cahn P, Lombaard J, Supparatpinyo K, Kumar S, et al. Safety and efficacy of doravirine as first‐line therapy in adults with HIV‐1: week 192 results from the open‐label extensions of the DRIVE‐FORWARD and DRIVE‐AHEAD phase 3 trials. Lancet HIV. 2024;11(2):e75‐85.

2. Post FA, Di Perri G, Cunningham D, Mussini C, Rockstroh JK, Correll TA, et al. Renal safety parameters of doravirine/islatravir (100/0.75 mg) vs bictegravir/emtricitabine/tenofovir alafenamide once‐daily as initial HIV‐1 treatment: week 48 results from a randomized, double‐blind phase 3 trial. 19th European AIDS Conference; 2023 Oct 18‐21; Warsaw, Poland. Poster eP.A.096.

3. Sanchez RI, Yee KL, Fan L, Cislak D, Martell M, Jordan HR, et al. Evaluation of the pharmacokinetics of metformin following coadministration with doravirine in healthy volunteers. Clin Pharmacol Drug Dev. 2020;9(1):107‐14.

4. Chu X, Bleasby K, Chan GH, Nunes I, Evers R. The complexities of interpreting reversible elevated serum creatinine levels in drug development: does a correlation with inhibition of renal transporters exist? Drug Metab Dispos. 2016;44(9):1498‐509.

### P321 Profiles of cardiac, liver, renal, musculoskeletal, and pancreatic safety in virologically suppressed people with HIV‐1 switching to tenofovir DF‐containing, ainuovirine‐based antiretroviral regimen: the secondary analyses of 48‐week results of the SPRINT trial, a randomised, active‐controlled phase III study

Qingxia Zhao^1^, Hongxia Wei^2^, Hongzhou Lu^3^, Hui Wang^4^, Shenghua He^5^, Zhu Chen^6^, Yaokai Chen^7^, Min Wang^8^, Fujie Zhang^9^, Hao Wu^10^, Weiping Cai^11^, Ping Ma^12^, Wan Wan^13^, Heliang Fu^13^, Hong Qin ^13^

^1^Department of Infectious Diseases, The Sixth People's Hospital of Zhengzhou, Zhengzhou, China. ^2^Department of Infectious Diseases, The Second Hospital of Nanjing, Nanjing, China. ^3^National Clinical Research Centre for Infectious Diseases, The Third People' s Hospital of Shenzhen, The Second Affiliated Hospital of Southern University of Science and Technology, Shenzhen, China. ^4^Department of Infectious Diseases, Shenzhen Third People's Hospital, Shenzhen, China. ^5^Department of Infectious Diseases, Public Health Clinical Medical Center of Chengdu, Chengdu, China. ^6^Drug Clinical Trial Institution, Public Health Clinical Medical Center of Chengdu, Chengdu, China. ^7^Division of Infectious Diseases, Chongqing Public Health Medical Center, Chongqing, China. ^8^Institute of HIV/AIDS, The First Hospital of Changsha, Changsha, China. ^9^Clinical and Research Center for Infectious Diseases, Beijing Ditan Hospital Capital Medical University, Beijing, China. ^10^Clinical and Research Center for Infectious Diseases, Beijing Youan Hospital, Capital Medical University, Beijing, China. ^11^Infectious Disease Center, Guangzhou Eighth People's Hospital, Guangzhou Medical University, Guangzhou, China. ^12^Department of Infectious Diseases, Tianjin Second People's Hospital, Tianjin, China. ^13^Clinical Research & Development, Jiangsu Aidea Pharmaceutical Co., Ltd, Yangzhou, China

**Background**: ACC008, a novel antiretroviral fixed‐dose combination (FDC), contains ainuovirine (ANV) plus lamivudine and tenofovir DF (TDF). This FDC as switch therapy has shown noninferior virological efficacy but improved lipid profile compared to tenofovir alafenamide (TAF)‐containing, boosted elvitegravir (EVG/c) regimen among virologically suppressed people with HIV‐1 (PWHs). We herein reported the secondary analyses of profiles of cardiac, liver, renal, musculoskeletal, and pancreatic safety as prespecified.

**Methods**: Virologically suppressed adult PWHs (*n* = 762) were randomised to ACC008 (*n* = 381) or comparator arm (*n* = 381) for 48 weeks. Changes from baseline (CFBs) in QTcF interval on electrocardiography and clinical laboratory examinations were primary safety outcomes of interest, including blood biochemistry, as prespecified.

**Results**: The two arms showed no significant changes in QTcF interval at week 48 (mean ± SD, ‐0.1 ± 17.3 vs. 1.2 ± 16.8 msec); a very small proportion of PWHs had QTcF interval prolongation >60 msec (0.3% vs 0.5%). Liver enzymes decreased slightly in both arms, including alanine transaminase (ALT), aspartate aminotransferase (AST), gamma‐glutamyl transferase (GGT), alkaline phosphatase (ALP), and lactate dehydrogenase (LDH) (Figure 1a). Total bilirubin increased marginally in the ACC008 arm and slightly in the comparator arm (0.28 ± 3.82 vs 2.04 ± 4.08 µmol/l), and direct bilirubin increased slightly in both arms (0.19 ± 1.308 vs. 0.30 ± 1.16 µmol/l) (Figure 1a). Blood urea nitrogen decreased in the ACC008 arm but increased marginally in the comparator arm (‐0.20 ± 2.35 vs. 0.15 ± 1.20 mmol/l) (Figure 1b). Serum creatinine increased slightly in both arms (1.28 ± 8.29 vs. 5.52 ± 8.88 µmol/l), accompanied by a slight increase in the estimated glomerular filtration rate with the Chronic Kidney Disease Epidemiology Collaboration (CKD‐EPI) equation (2.3 ± 15.0 vs 9.9 ± 15.9 mL/min/1.73 m^2^) (Figure 1b). Serum phosphates remained constant (‐0.016 ± 0.176 vs. 0.004 ± 0.221 mmol/l) (Figure 1b); creatine kinase increased slightly (83.0 ± 758.0 vs 51.2 ± 1228.5 u/l); and amylase remained constant (‐0.05 ± 16.5 vs. ‐0.17 ± 17.0 u/l). Common organ‐associated adverse events (≥5% in either arm) included sinus bradycardia (8.1% vs 3.7%), elevated ALT (3.9% vs 6.0%), elevated AST (4.7% vs. 6.0%), elevated GGT (6.8% vs. 2.4%), urine protein detected (7.1% vs. 6.8%) and elevated creatine kinase (7.1% vs. 6.8%).

**P321: Figure 1:**
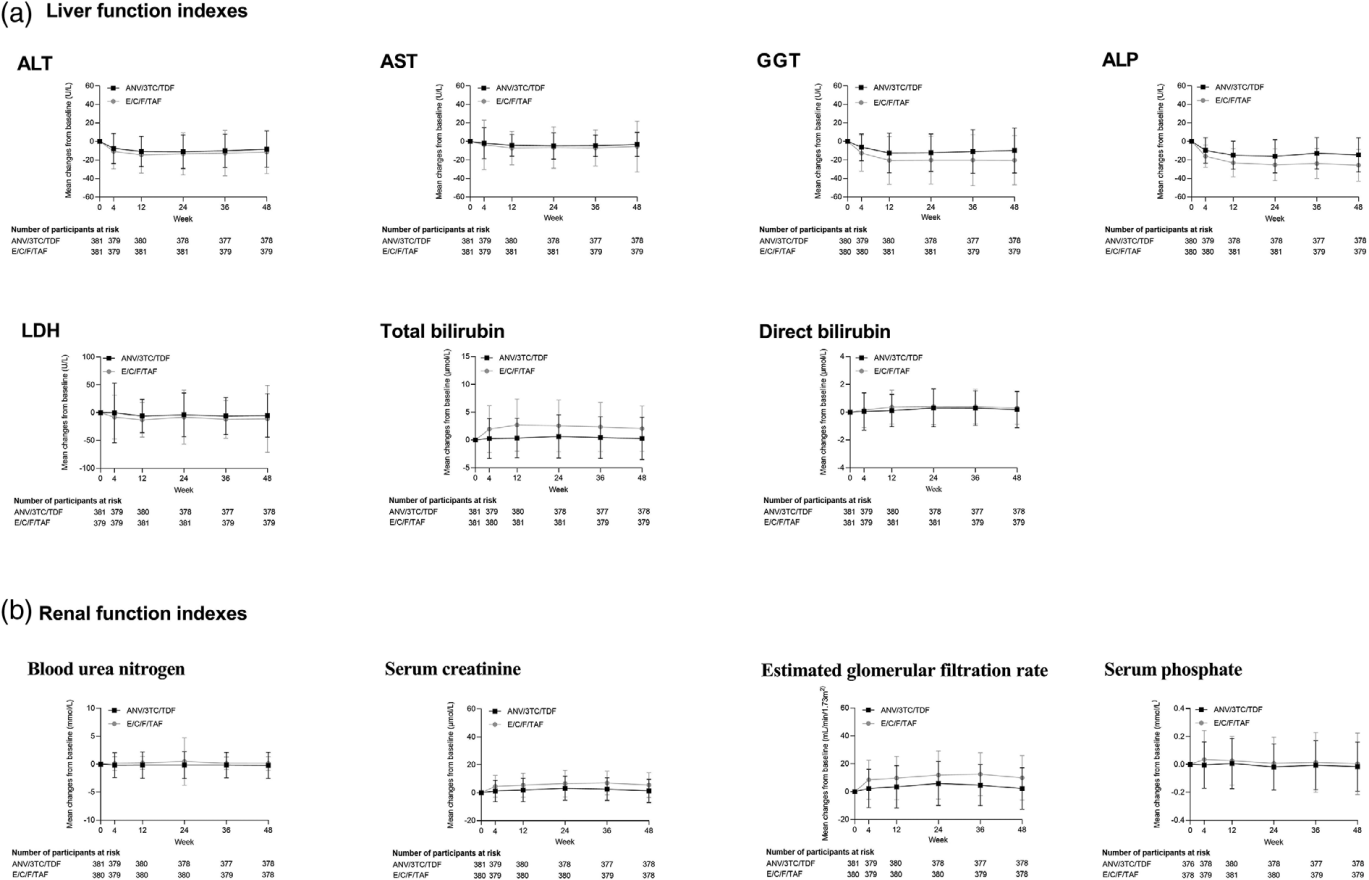
Mean changes from baseline in liver and renal function indexes. Error bars represent standard deviations.

**Conclusions**: Switches to ANV/3TC/TDF and EVG/c/FTC/TAF were both well tolerated in virologically suppressed PWHs. Liver and renal function tests showed an improving trend with both regimens, without clinically significant changes in cardiac, musculoskeletal and pancreatic measures observed.

### P322 Kidney transplantation in liver‐transplanted HIV‐positive recipients with end‐stage renal disease: two case reports

Daniela Malano Barletta^1^, Nuria Esforzado^2^, Pablo Ruiz^3^, Frederic Cofan^2^, Montserrat Laguno^1^, David Cucchiari^2^, Alejandro Forner^3^, Hugo López^4^, Mireia Musquera^2^, Asunción Moreno^1^, Antonio Rimola^3^, Fritz Diekmann^2^, Jose M. Miró ^5^

^1^Infectious Diseases—HIV/AIDS Unit, Hospital Clinic‐Institut d'Investigacions Biomèdiques August Pi i Sunyer (IDIBAPS)‐University of Barcelona, Barcelona, Spain. ^2^Nephrology Service—Kidney Transplant Unit, Hospital Clinic‐IDIBAPS‐University of Barcelona, Barcelona, Spain. ^3^Hepatology Service—Liver Transplant Unit, Hospital Clinic‐IDIBAPS‐University of Barcelona and CIBEREHD, Barcelona, Spain. ^4^Psychiatry Service, Hospital Clinic‐IDIBAPS‐University of Barcelona, Barcelona, Spain. ^5^Infectious Diseases—HIV/AIDS Unit, Hospital Clinic‐IDIBAPS‐University of Barcelona and CIBERINFEC, Barcelona, Spain

**Background**: Since the implementation of highly effective antiretroviral therapy (ART), solid organ transplantation (SOT) in people with HIV (PWH) has become part of routine clinical care [1]. Their short/medium‐term survival is comparable to that of the transplanted population not HIV‐infected, provided hepatitis C virus (HCV) has been eradicated [2,3]. In contrast, there is limited information on the efficacy of kidney transplantation (KT) in liver transplant (LT) HIV‐positive recipients with end‐stage renal disease. This study describes the outcomes of two PWH with successful LT who subsequently required KT due to developed end‐stage renal disease.

**Methods**: We describe the clinical characteristics and outcomes of two patients with HIV with successful LT who subsequently required KT.

**Results**: Case 1. A 62‐year‐old male, HIV‐positive since 1984, with a history of liver cirrhosis (LC) due to HCV infection and alcohol abuse, underwent LT in 2011 and immunosuppressed treatment (IST) with mycophenolate sodium, prednisone, and cyclosporine. In 2014, he had HCV reinfection, receiving treatment with sofosbuvir + daclatasvir with sustained virological response. In 2017 he developed end‐stage chronic kidney disease (CKD) secondary to calcineurin microangiopathy, started haemodialysis (HD) in 2019, and finally successfully underwent cadaveric KT in 2021. Three years later clinical progress is good on antiretroviral dual therapy with lamivudine/dolutegravir (since 2017), as is virological control with no interactions or complications after the second transplant (Table 1). Case 2. A 62‐year‐old male with HIV/HCV co‐infection for over 30 years, HCV eradication in 2011 after direct‐acting antivirals with ribavirin, and developed LC and CKD (type II hepatorenal syndrome), leading to LT in 2019. Subsequently, HD began in 2022 due to worsening renal function secondary to the use of calcineurin inhibitors, and deceased‐donor KT was performed in 2023. Currently, at 6 months post‐KT, he is virologically suppressed on ART with dolutegravir/darunavir/cobicistat (previous resistance), with increasing CD4 counts. He is on immunosuppressive therapy with mycophenolate, tacrolimus, and prednisone. However, acute graft rejection developed at 1 month (receiving methylprednisolone bolus, rituximab and apheresis) (Table 1).

**P322: Table 1:** Evolution of analytical parameters of the two described patients

	Case 1	Case 1	Case 1	Case 1	Case 2	Case 2	Case 2	Case 2
	Viral load HIV‐1 (copies/mL)	CD4/mm^3^	Creatinine (mg/dL)/eGFR (mL/min/1.73 m^2^)	Proteinuria (mg/gr creatinine)	Viral load HIV‐1 (copies/mL)	CD4/mm^3^	Creatinine (mg/dL)/eGFR (mL/min/1.73 m^2^)	Proteinuria (mg/gr cr)
Before KT	<50	322 (40%)	6.72/8	5184	<50	409 (29%)	5.47/10	364
+1 month	<50	−	1.10/73	305	<50	91 (24%)	4.40/13	390
+3 months	<50	514 (39%)	1.10/73	255	<50	201 (28%)	3.60/17	155
+6 months	<50	263 (26%)	0.93/90	850	<50	306 (28%)	3.51/18	290
+12 months	<50	401 (24%)	0.87/>90	2340	−	−	−	−
+2 years	<50	594 (40%)	1.09/73	5363	−	−	−	−
+3 years	<50	485 (40%)	1.11/71	1634	−	−	−	−

**Conclusion**: Kidney transplantation in PWH and LT with end‐stage CKD can be feasibly performed. Current non‐boosted integrase inhibitors‐based ART avoid drug‐drug interactions. Long‐term follow‐up of these patients is needed.

**References**

1. Miro JM, Torre‐Cisnero J, Moreno A, Tuset M, Quereda C, Laguno M, et al. GESIDA/GESITRA‐SEIMC, SPNS y ONT sobre trasplante de órgano sólido en pacientes infectados por el VIH en España (marzo 2005). Enferm Infecc Microbiol Clin. 2005;23:353‐62.

2. Agüero F, Forner A, Manzardo C, Valdivieso A, Blanes M, Barcenaet R, et al; FIPSE Investigators. Human immunodeficiency virus infection does not worsen prognosis of liver transplantation for hepatocellular carcinoma. Hepatology. 2016;63(2):488‐98.

3. Werbel WA, Durand CM. Solid organ transplantation in HIV‐infected recipients: history, progress, and frontiers. Curr HIV/AIDS Rep. 2019;16:191‐203.

### P323 Use of bictegravir/emtricitabine/tenofovir alafenamide (B/F/TAF) in kidney transplant recipients living with HIV‐1 receiving calcineurin and mTOR inhibitors: a pilot switch study

Sébastien Gallien ^1^, Lambert Assoumou^2^, Jacques Dantal^3^, Anne Hulin^4^, Aida Benalycherif^5^, Raida Ben Rayana^1^, Yasmine Tobbal^2^, Marie Matignon^6^, Giovanna Melica^1^, Clotilde Allavena^7^, Thomas Stehle^6^

^1^Infectious Diseases and Clinical Immunology, Hôpital Henri Mondor (APHP) / Université Paris Est Créteil, Créteil, France. ^2^Institut Pierre Louis d'Epidémiologie et de Santé Publique, Sorbonne Université, INSERM, Paris, France. ^3^Nephrology and Transplantation, CHU Nantes, Nantes, France. ^4^Pharmacology, Hôpital Henri Mondor (APHP) / Université Paris Est Créteil, Créteil, France. ^5^Clinical Research, Institute of Applied Medicine and Epidemiology (IMEA), Paris, France. ^6^Nephrology and Transplantation, Hôpital Henri Mondor (APHP) / Université Paris Est Créteil, Créteil, France. Infectious Diseases and Tropical Medicine, ^7^CHU Nantes, Nantes, France

**Background**: Management of ART in kidney transplant recipients living with HIV (HIV‐KTR) has historically been problematic because of nephrotoxicity of some antiretrovirals and drug–drug interactions between immunosuppressants and ART.

**Methods**: Prospective study to assess the safety and efficacy of B/F/TAF in virologically suppressed (<50 copies/mL) HIV‐KTR with sensitive virus (genotypic susceptibility score, GSS ≥2). Changes in pharmacokinetics of calcineurin and mammalian target of rapamycin (mTOR) inhibitors, estimated and measured glomerular filtration rate (eGFR and mGFR), bone mineral density (BMD), immunovirological responses and clinical outcomes were recorded through 48 weeks.

**Results**: Five HIV‐KTR were enrolled and were receiving 3TC/ABC+RAL (*n* = 4) or RPV+DTG (*n* = 1) before switching to B/F/TAF: two women, median (IQR) age 58 (48–66) years and BMI 25.0 kg/m^2^ (20.6–26.), two with AIDS. At baseline (BL): in median, CD4 count was 312/mm^3^ (296–499), time since HIV diagnosis 13.9 years (11.4–29.0), duration on last ART 5.7 years (5.1–8.9), duration of viral suppression 8.5 years (6.6–15.2), time since kidney transplantation 5.9 years (3.3–7.0). In addition to corticosteroids, the immunosuppressant regimen included calcineurin inhibitors (cyclosporine *n* = 1, mycophenolate mofetil *n* = 3, tacrolimus *n* = 1) and mTOR inhibitors (everolimus *n* = 2). Two patients had resistance‐associated mutations to NRTI or INSTI prior to switching (M184V for one, N155H for one). Variations of studied parameters from BL to week (W) 48 are presented in Table 1. No adjusting dose of calcineurin and mTOR inhibitors was needed through 48 weeks. Median mGFR measured (iohexol clearance) remained stable from 46.7 (45.4–52.3) at BL to 48.9 mL/min/1.73 m^2^ (37.3–58.0) at W48. Median eGFR decreased from 48 (35–49) at BL to 40 mL/min/1.73 m^2^ (33–51) at W48. Median change in BMD from BL to W48 was ‐0.04 g/cm^2^ (‐0.07, ‐0.04) at the lumbar spine and ‐0.01 g/cm^2^ (‐0.03−0.01) at the hip. All patients remained virologically suppressed during the study period. Median change in CD4 count was +38 mm^3^ (‐36−144) at 48 weeks. There was one grade 1 treatment‐related adverse event (nightmare). One patient died after intracranial haemorrhage (W15) and another developed autoimmune encephalitis (W42), both not related to the treatment.

**P323: Table 1:** Variations of studied parameters from baseline to week 48

Time from switch to B/F/TAF	D0	W2	W4	W12	W24	W36	W48	Δ (D0‐W48)
mGFR (iohexol clearance) mL/min/1.73 m^2^, median (IQR)	46.7 (45.4‐52.3)						48.9 (37.3‐58)	5.7 (2.2‐8.1)
Creatinine‐based eGFR (CKD‐EPI 2009) mL/min/1.73 m^2^, median (IQR)	48 (35‐49)	41 (34‐42)	43 (32‐43)	36 (35‐41)	39 (35‐49)	40.5 (35‐49.5)	40 (33‐51)	−1.5 (−5.0‐3.0)
Bone mineral density g/cm^2^, median (IQR), lumbar spine	0.82 (0.75‐1.01)						0.79 (0.68‐0.98)	−0.04 (−0.07, −0.04)
Bone mineral density g/cm^2^, median (IQR), hip	0.72 (0.72‐1.04)						0.71 (0.61‐1.07)	−0.01 (−0.03‐0.01)
CD4 count cells/L, median (IQR)	312 (296‐499)			315 (286‐452)			435 (342‐562)	38.5 (−36.5‐144.0)
CD4/CD8 ratio, median (IQR)	1.01 (0.84‐1.27)			1.24 (1.00‐1.37)			1.21 (0.61‐1.78)	0.09 (0.04‐0.23)

Abbreviations: CKD‐EPI 2009, Chronic Kidney Disease Epidemiology Collaboration equation developed in 2009 to estimate glomerular filtrate rate (eGFR); D, day; IQR, interquartile range.

**Conclusions**: This pilot study shows that B/F/TAF appears to be safe and effective in HIV‐KTR receiving calcineurin and mTOR inhibitors.

## Co‐morbidities and complications of disease and/or treatment—Mental health disorders

### P324 Resilience among people with HIV: results from the UK Positive Voices 2022 survey

Colette Smith ^1^, Janey Sewell^1^, Alex Sparrowhawk^2^, Annegret Pelchen‐Matthews^1^, Fumiyo Nakagawa^1^, Fiona Wilson^3^, Tracey Fong^4^, Hannah Kitt^5^, Carole Kelly^5^, Meaghan Kall^5^, Veronique Martin^5^, Adamma Aghaizu^5^, Alison Rodger^1^, Fiona Lampe^1^

^1^Global Health, UCL, London, UK. ^2^George House Trust, Manchester, UK. ^3^HIV, Addenbrooke's Hospital, Cambridge, UK. ^4^HIV, Homerton University Hospital, London, UK. ^5^STI and HIV, UK Health Security Agency, London, UK

**Background**: Resilience is the capacity to withstand or to recover from difficulties or stressors. Resilience is complex and multilevel, but higher levels of resilience and the resources associated with resilience (e.g. social support and financial stability) may protect against poor mental health in people with HIV.

**Materials and methods**: The Positive Voices 2022 survey (April 2022–March 2023) recruited people from 101 HIV clinics in England, Wales and Scotland using the HIV surveillance database (HARS) as a national sampling frame. Participants self‐completed a questionnaire asking about a wide range of topics. Resilience was measured using the 14‐item resilience scale (RS‐14), using a seven‐point Likert scale (overall score between 14 and 98), with higher scores indicating higher resilience. First, factors associated with improved resilience (with resilience as the dependent factor/“outcome”) were investigated using modified Poisson regression, adjusted for age, demographic group and year of diagnosis. Next, the association of resilience (as the independent factor/“predictor”) with a range of health and wellbeing outcomes was examined using modified Poisson regression.

**Results**: Four thousand, one hundred and thirty‐five of 4607 (89.8%) participants completed the resilience questions (60% GBMSM, 23% women, median age 53 years, 99% on ART for median duration of 13 years). The median (range) score was 80 (14–98). The numbers with low (≤73), moderate (74–81) and high (≥82) resilience were 1556 (37.6%), 653 (15.8%) and 1926 (46.6%), respectively. High levels of resilience ≥82 were more common among those of older age, heterosexual men and black African women (compared to GBMSM), greater social support, and lower levels of financial hardship (Table 1). Higher resilience scores were associated with a considerably lower prevalence of depressive symptoms. PHQ‐9 ≥ 10 (adjusted prevalence ratio [aPR] 0.16 high vs low resilience; 95% CI 0.13–0.20), lower anxiety symptoms GAD‐7 ≥ 10 (aPR 0.14, 0.11–0.18), recreational drug use (aPR 0.67; 0.60–0.76), and not being ashamed of one's HIV status (aPR 0.76; 0.71–0.81). There was no evidence of an association with alcohol dependency (aPR 1.00; 0.93–1.07).

**P324: Table 1:** Factors associated with high resilience (score ≥83 on RS‐14 scale)

	High resilience; % (*n*/*N*)	Adjusted prevalence ratio (95% CI)^a^
Age (years)		
18‐34	33.8% (105/311)	0.74 (0.62‐0.87)
35‐44	44.2% (352/797)	0.93 (0.83‐1.02)
45‐54	47.6% (582/1222)	1.00
55‐64	47.0% (595/1267)	1.02 (0.94‐1.11)
65+	54.2% (291/537)	1.21 (1.10‐1.34)
Demographic group		
GBMSM	43.1% (1068/2479)	1.00
Black African heterosexual men	58.2% (139/239)	1.33 (1.18‐1.49)
Other ethnicity heterosexual men	51.0% (176/345)	1.13 (1.01‐1.27)
Black African women	60.9% (323/530)	1.43 (1.31‐1.55)
Other ethnicity women	41.5% (182/439)	0.96 (0.86‐1.09)
Gender described as non‐binary or in another way	36.9% (38/103)	0.86 (0.67‐1.11)
Year of HIV diagnosis		
2019‐2021	37.5% (66/176)	0.84 (0.68‐1.03)
2014‐2018	44.1% (319/724)	0.96 (0.86‐1.07)
2009‐2013	48.8% (418/856)	1.00
2004‐2008	49.8% (479/961)	0.96 (0.87‐1.05)
2003 or earlier	45.0% (599/1332)	0.86 (0.79‐0.95)
Enough money for basic needs		
Yes always	55.8% (1234/2210)	1.00
Most of the time	36.7% (385/1048)	0.60 (0.55‐0.66)
Some of the time	36.6% (185/505)	0.53 (0.47‐0.60)
No	29.1% (76/261)	0.41 (0.34‐0.50)
Social support		
High	64.5% (1442/2235)	1.00
Low	25.5% (484/1900)	0.40 (0.37‐0.43)
Religious beliefs		
No religion	41.8% (623/1490)	1.00
Spiritual (no organised religion), religion important to me	36.4% (52/143)	0.87 (0.69‐1.09)
Spiritual (no organised religion), religion not very important to me	43.6% (95/218)	1.03 (0.88‐1.21)
Christian, religion important to me	53.9% (678/1259)	1.09 (0.98‐1.20)
Christian, religion not very important to me	49.7% (275/553)	1.13 (1.02‐1.25)
Other organised religion, religion important to me	49.2% (93/189)	1.09 (0.93‐1.27)
Other organised religion, religion not very important to me	36.1% (30/83)	0.85 (0.63‐1.10)

Abberviations: CI, confidence interval; GAD‐7, general anxiety disorder‐7; GBMSM, gay, bisexual and other men who have sex with men; PHQ‐9, patient health questionnaire‐9.

Note: ^a^Adjusted for age, demographic group, year of diagnosis; unknown categories included in analysis but not presented here.

**Conclusions**: Those with higher resilience had better mental health and wellbeing. Evaluation and implementation of successful strategies that potentially help to improve resilience, such as a health and wellbeing coaching, could help improve physical and mental health and wellbeing outcomes for people with HIV.

### P325 Mental health in people with HIV (PWH): patient‐reported outcomes in the DUALIS study

Eva Wolf ^1^, Annamaria Balogh^1^, Florian Voit^2^, Judith Laufenberg^3^, Christoph Boesecke^3^, Christine Koegl^1^, Christoph D Spinner^2^, Andreas Dinkel^4^, of the DUALIS study group on behalf^5^

^1^Clinical Research, MUC Research, Munich, Germany. ^2^Department of Clinical Medicine—Clinical Department for Internal Medicine II, Technical University of Munich, TUM School of Medicine and Health, Munich, Germany. ^3^Department of Internal Medicine I, University Hospital Bonn, Bonn, Germany. ^4^Clinical Department for Psychosomatic Medicine and Psychotherapy, Technical University of Munich, TUM School of Medicine and Health, Munich, Germany. ^5^Clinical Research, Münchner Studienzentrum, Munich, Germany

**Background**: Patient‐reported outcome measures (PROMs) are of increasing relevance in the assessment of patients’ health‐related quality of life and in identification of unnoticed symptoms. In this post‐hoc analysis of the DUALIS study [1], we focus on mental health in people with HIV (PWH) on stable suppressive ART.

**Methods**: The Hospital Anxiety and Depression Scale (HADS) was used to screen for anxiety and depression. For the corresponding subscales HADS/A and HADS/D, cut‐offs of ≥8 (mild to severe) and ≥11 (moderate to severe) were used. PROMs were evaluated with respect to socio‐demographic (age, HIV disclosure status, partnership, employment) and clinical variables (stage of HIV disease, presence of comorbidities).

**Results**: The analysis population consisted of 259 (out of 263) DUALIS participants who completed the HADS questionnaire: 90% male sex, 40% ≥50 years, 26% CDC C stage at HIV diagnosis, 46% with CD4‐nadir <200/µl; 51% in stable partnership, 75% having a full‐ or part‐time job. Overall, 10% of participants had disclosed their HIV status to an extended circle of people, 80% only to very close people; for a further 10%, only healthcare providers knew about it. The baseline prevalence of depression using an HADS/D score ≥8 [≥11] was 17% (*n* = 44/259) [5%]. Anxiety was highly correlated with depression: in those with HADS/D ≥8, the median HADS/A score (IQR, interquartile range) was 10 (7–12) (in comparison to 4 (2–6) in those with HADS/D <8). Fifteen percent of participants received psychotherapy or psychotropic treatment/pharmacotherapy, 36% of those with HADS/D ≥8, 11% of those with HADS/D <8. Independent factors associated with HADS/D scores ≥8 in logistic regression analysis were female sex (OR 3.3; 95% CI 1.2–9.5; *p* = 0.03) and age ≥50 years (OR 2.6; 95% CI 1.2–5.5; *p* = 0.01); there was a trend towards higher depression scores for people without stable partnership (OR 2.0; 95% CI 0.9–4.3, *p* = 0.07). Of note, HADS/D scores did not change significantly from baseline to week 48, neither in the continuous ART study arm (darunavir/ritonavir + 2NRTI) nor in the interventional arm with switch to darunavir/ritonavir + dolutegravir.

**Conclusions**: In the DUALIS study, other factors than disease related variables were associated with higher depression scores. Patient‐reported outcome measures may help identify PWH in need of therapeutic intervention.

**Reference**

1. Spinner CD, Kümmerle T, Schneider J, Cordes C, Heiken H, Stellbrink H‐J, et al. Efficacy and safety of switching to dolutegravir with boosted darunavir in virologically suppressed adults with HIV‐1: a randomized, open‐label, multicenter, phase 3, noninferiority trial: the DUALIS study. Open Forum Infect Dis.2020;7(9):ofaa356.

### P326 Anxiety and depression in people living with HIV/AIDS (PLHIV) and their impact on fatigue and health‐related quality of life (HRQOL)

Pulin Kumar Gupta ^1^, Akhil Goyal^2^, Atul Anand^1^

^1^HIV Clinic, Department of Medicine, ABVIMS Dr Ram Manohar Lohia Hospital, New Delhi, India. ^2^Department of Medicine, ABVIMS Dr Ram Manohar Lohia Hospital, New Delhi, India

**Background**: Neuropsychiatric diseases are quite common but under‐diagnosed entities in PLHIV and are associated with fatigue syndromes and hence poor HRQOL, especially in developing countries like India. We tried to determine the prevalence and predictors of anxiety neurosis (AN) and depression illness (DI) by hospital anxiety and depression score (HADS) in PLHIV their affect on fatigue and HRQOL and their correlation with various socio‐economic parameters and level of immunosuppression as assessed by CD4 counts and viral load.

**Methods**: It was a cross‐sectional observational study done in 200 PLHIV on ART for at least 2 years (clients) at a tertiary care centre in New Delhi, India. All cases with any comorbidities, organ dysfunction, endocrinopathies, hypovitaminosis‐D or B12 deficiency, recent opportunistic infection (OI) or any illness including COVID‐19 during the last 6 months were excluded.

**Results**: Thirty‐two percent of the clients were females, 60% males and 8% transgender. The mean age of the clients was 37.86 ± 11.79 years and 58% had CD4 counts between 250–499/µl and 34% had CD4 counts >500/µl. The mean duration of ART was 4.4 ± 1.9 years. Viral RNA was undetectable in 67% of clients. Using HADS score, 168 clients (84%) were found to be suffering with AN and DI. The mean HRQOL as assessed by 36‐Item Short Form Health Survey (SF‐36) was found to be 67.68 ± 20.45 (range 11.9–98.75). Seventy‐five percent of cases had SF‐36 <2.0 SD and HRQOL was low primarily in emotional and mental health aspects rather than physical health. Fatigue was assessed by multidimensional assessment of fatigue (MAF, 0–50) and significant fatigue (values >27) was found in 73% of cases (mean 32.47 ± 10.39, range 10–50). Age >40 years, male sex, anaemia, high creatinine, longer duration of ART and viral load (*p* < 0.005) were found to be significantly associated with AN and DI. Surprisingly, CD4 counts, poor socio‐economic status being transgender or female or type of ART were not found to have any association with AN or DI. Presence of anxiety, depression, high viral load and anaemia were found to be predictors of fatigue and poor HRQOL.

**Conclusion**: AN and DI are under‐diagnosed but alarmingly high (>80%) amongst PLHIV and are associated with fatigue and poor HRQOL. Viral load, haemoglobin, creatinine and 6‐monthly psychological evaluation should be a must in PLHIV.

### P327 No association of cART and HIV‐related parameters with health‐related quality of life (HR‐QoL) of PLWH: insights from a multidimensional screening

Stella Capodieci, Mauro Zaccarelli, Alessandra Latini, Christof Stingone, Laura Gianserra, Maria Gabriella Donà, Eugenia Giuliani, Valentina Cafaro, Massimo Giuliani

HIV/AIDS Unit, San Gallicano Dermatological Institute IRCCS, Rome, Italy

**Background**: Progress in HIV treatment has improved PLWH life expectancy and infection management. However, PLWH still faces more health challenges, including somatic and psychological symptoms and cognitive decline [1, 2]. Screening aimed to identify early signs of health vulnerability could improve the HIV‐continuum.

**Materials and methods**: Consecutive PLWH on ART attending the HIV/AIDS Unit of the San Gallicano Dermatological Institute (Rome, Italy) from January to August 2024 were screened for emotional vulnerability, subjective symptoms and cognitive functions using the Hospital Anxiety and Depression Scale [3], a PRO based on 28 qualitative‐quantitative items [4], and the Montreal Cognitive Assessment (MoCA) [5], respectively. A regression analysis was conducted to identify factors associated with the outcomes of interest, adjusting for clinical values, polypharmacy, current ART regimen and number of ART classes experienced.

**Results**: Two hundred and six PLWH were screened [182 men (88.3%), 24 women (11.7%), median age of 53 (IQR 44–71) and 56 (IQR 41–81), respectively]. Almost all had undetectable HIV‐RNA (94.2%) and CD4+ counts >200 cells/mm^3^ (97.6%). Fifty‐five (26.7%) and 30 subjects (14.6%) reported anxiety and depression symptoms, respectively. Twenty‐three of 203 (12.8%) Italian patients had a MoCA score under normative cut‐off [6]. At PRO, PLWH reported sleep disturbances (28.2%), fatigue (27.8%) and pain (26.2%) with a remarkable intensity. High educational level and polypharmacy were protective for cognitive decline, but not age, symptoms of depression and separated/widowed status. Anxiety predicted several symptoms at PRO, such as pain, fatigue, sleep and sexual disorders (Table 1). Moreover, female sex and comorbidities were associated with pain. Depression was associated with fatigue and a MoCA score under cut‐off. A manager‐level employment was protective for depression. No associations between any covariates and current ART and clinical/immunological/virological parameters (nadir or current CD4+ count, HIV‐RNA, clinical stage, time from diagnosis) emerged.

**P327: Table 1:** Statistically significant associations between individual factors and cognitive, psychological and physical outcomes in 206 PLWH

	Covariates	AOR	95% CI	*p*‐value
MoCA <22	Age (per year)	1.07	1.00–1.13	0.041
	Educational level (high school or higher versus middle school or lower)	0.14	0.05–0.42	0.001
	Polypharmacy (≥2 non ART drugs)	0.20	0.05–0.79	0.021
	Time from HIV diagnosis (per month)	1.01	1.00–1.02	0.023
	Marital status (separated/widowed versus married)	8.91	1.43–55.39	0.019
	HADS‐Depression	3.61	1.04–12.53	0.044
HADS‐Anxiety ≥8	Age (per year)	0.96	0.93–1.00	0.034
	Marital status (separated/widowed versus married)	4.34	1.23–15.27	0.022
	Educational level (high school or higher versus middle school or lower)	0.34	0.15–0.76	0.008
HADS‐Depression ≥8	Marital status (separated/widowed versus married)	6.66	1.73–25.59	0.006
	Employment status (manager‐level versus unemployed/retired/office workers)	0.25	0.07–0.89	0.033
PRO‐Pain	Comorbidities (≥2)	2.17	1.06–4.47	0.035
	Sex (F versus M)	3.23	1.24–8.39	0.016
	HADS‐Anxiety^a^	3.69	1.82–7.47	<0.001
PRO‐Fatigue	HADS‐Anxiety^a^	3.70	1.63–8.40	0.020
	HADS‐Depression^a^	3.48	1.22–10.00	0.002
PRO‐Sleep Disturbances	HADS‐Anxiety^a^	5.68	2.88–11.23	<0.001
PRO‐Sexual Disorders^b^	Comorbidities (≥2)	3.59	1.66–7.76	0.001
	HADS‐Anxiety^a^	3.90	1.92–7.96	<0.001

*Note*: Results from logistic regressions analysis adjusted for current and nadir CD4^+^ Tcell count, HIV‐RNA, current ART regimen and number of ART classes experienced.

Abbreviations: AOR, adjusted odds ratio; CI, confidence interval; HADS, Hospital Anxiety and Depression Scale; MoCA, Montreal Cognitive Assessment; PRO, patient reported outcome.

^a^ The scores were used as dichotomous variables. Clinical cut‐off is ≥8;

^b^ PROs include: decreased sexual interest, erection disorders and delayed ejaculation.

**Conclusions**: Cognitive impairment and psychological disorders did not show associations with parameters of HIV infection, or with current or past treatments. The improvement of ART tolerability and the long‐term‐associated stability of disease have certainly contributed to this outcome. However, the relevant proportion of PLWH with psychological and cognitive issues highlights the need for further investigations and specific interventions for these conditions.

**References**

1. Miners A, Phillips A, Kreif N, Rodger A, Speakman A, Fisher M, et al. Health‐related quality‐of‐life of people with HIV in the era of combination antiretroviral treatment: a cross‐sectional comparison with the general population. Lancet HIV. 2014;1(1):e32‐40.

2. Chaudhury S, Bakhla AK, Saini R. Prevalence, impact, and management of depression and anxiety in patients with HIV: a review. Neurobehav HIV Med. 2016;7:15‐30.

3. Bjelland I, Dahl AA, Haug TT, Neckelmann D. The validity of the Hospital Anxiety and Depression Scale. An updated literature review. J Psychosom Res. 2002;52(2):69‐77.

4. Bucciardini R, Murri R, Guarinieri M, Starace F, Martini M, Vatrella A, et al. ISSQoL: a new questionnaire for evaluating the quality of life of people living with HIV in the HAART era. Qual Life Res. 2006;15(3):377‐90.

5. Nasreddine ZS, Phillips NA, Bédirian V, Charbonneau S, Whitehead V, Collin I, et al. The Montreal Cognitive Assessment, MoCA: a brief screening tool for mild cognitive impairment. J Am Geriatr Soc. 2005;53(4):695‐9.

6. Santangelo G, Siciliano M, Pedone R, Vitale C, Falco F, Bisogno R, et al. Normative data for the Montreal Cognitive Assessment in an Italian population sample. Neurol Sci. 2015;36(4):585‐91.

### P328 Predictors of mortality among tuberculosis patients with mental health disorders: a retrospective study at a national referral mental hospital in Uganda

Isaiah Aryatuha ^1^, Patrick Kazooba^2^, Gertrude Namale^1^, Emmanuel Sendaula^3^, Josephine Kaleebi^4^

^1^Medical, Reach Out Mbuya Community Health Initiatives, Kampala, Uganda. ^2^Research and Innovation, Reach Out Mbuya Community Health Initiatives, Kampala, Uganda. ^3^Monitoring and Evaluation, Reach Out Mbuya Community Health Initiatives, Kampala, Uganda. ^4^Programmes, Reach Out Mbuya Community Health Initiatives, Kampala, Uganda

**Background**: Tuberculosis (TB) remains a significant global health challenge, particularly among vulnerable populations. The intersection of TB and mental health presents unique challenges in limited healthcare resources settings. We aimed to identify mortality predictors among TB patients with mental health disorders admitted at Butabika National Referral Mental Hospital (BNRMH) in Central Uganda.

**Methods**: A retrospective study was conducted on patients receiving TB treatment at BNRMH between January 2020 and December 2023. Data were collected on demographic information, HIV status, treatment details, diagnostic methods, TB classification, comorbidities and points of entry into care from patient records and facility registers. Data was analysed in Stata. Multivariate logistic regression was performed to identify independent predictors of TB‐associated mortality, adjusting for confounders such as age, gender, area of residence, HIV status and mental health conditions.

**Results**: The study included 150 patients with mental health disorders who received TB treatment from January 2020 to December 2023. The majority were male (64.67%), with 73% aged between 20 and 40 years (median age 35.5 years, IQR 29–45). The mean weight at diagnosis was 50.8 kg (SD 12.52). Most patients (95.21%) were newly diagnosed with TB, and 44% were HIV‐positive. Pulmonary clinically diagnosed tuberculosis (PCD) was the most common TB classification (50.67%), with X‐ray being the most common diagnostic tool (46%). A total of 55 out of 150 patients (36.67%) died, with a median time to death of 27 days post‐diagnosis. Adjusted logistic regression analysis identified advanced age above 50 years (aOR 16.88, 95% CI 1.66–171.57, p = 0.017) and HIV‐positive status (aOR 3.57, 95% CI 1.57–8.15, p = 0.002) as significant predictors of mortality.

**Conclusions**: A high mortality rate of 36.67%, well above the World Health Organization threshold of <5%, was observed among TB patients with mental health disorders, particularly soon after diagnosis. Mortality was significantly associated with advanced age and HIV positivity. Targeted interventions focussing on early detection and tailored management for elderly patients, those with HIV co‐infection, and individuals with severe mental health conditions are crucial to reduce these high mortality rates.

### P329 Mental health service access barriers and facilitators among people living with HIV: a qualitative study in Georgia

Esma Imerlishvili ^1^, Maia Kajaia^1^, Mariam Pashalishvili^2^, Eka Chkhonia^3^, Tamar Zurashvili^1^, Ramesh Raghavan^4^, Mamuka Djibuti^1^

^1^Public Health and Epidemiology, Partnership for Research and Action for Health, Tbilisi, Georgia. ^2^Public Health and Epidemiology, Partnership for Research and Action for Health, Mtskheta, Georgia. ^3^Department of Psychiatry, Tbilisi State Medical University, Tbilisi, Georgia. ^4^Silver School of Social Work, New York University, New York, NY, USA

**Background**: While people living with HIV (PLWH) are disproportionately affected by mental health disorders, their needs are commonly unmet, especially in low‐resource settings [1]. This exacerbates the burden of mental health among PLWH, expressed in poor health outcomes and quality of life [2]. We explored the perceived mental health needs, barriers and facilitators for accessing mental health services among older PLWH.

**Materials and methods**: We collected data using semi‐structured in‐depth interviews with 28 PLWH in four Georgian regions (February–April 2024). Participants were purposively selected from a pilot cross‐sectional study on mental and cognitive health among older PLWH. We ensured diversity in gender, mental health needs and location. Data were analysed using a deductive‐inductive approach. We employed the Consolidated Framework for Implementation Research (CFIR) to interpret the data on barriers and facilitators.

**Results**: Participants reported diverse mental health needs, varying by gender, duration of HIV infection and membership in subgroups affected by the intersection of HIV and mental health challenges. Using the CFIR, we presented four key domains of barriers and facilitators to mental healthcare access. (1) Individual barriers to care included misconceptions about mental health that problems should be self‐managed, fear of re‐traumatization from discussing HIV and stigma (both HIV‐ and mental health‐related). Facilitators included awareness of mental health, family support and social status. (2) Healthcare setting barriers included provider's lack of knowledge about the importance of mental healthcare and discriminatory attitudes; however, person‐centred care and community involvement facilitated access. (3) Outer setting barriers were the scarcity of funded services and cultural stigma, while financial and geographical accessibility facilitated care. (4) Preferred interventions varied based on gender, age, key population status, geographic location and duration of living with HIV.

**Conclusions**: This first study focusing on the mental health needs of PLWH revealed multifaced factors that hinder or facilitate PLWH from accessing mental health services. The findings highlight the need for tailored interventions that address individual, interpersonal, organizational and societal factors to improve mental health service utilisation and outcomes for this vulnerable population. These findings may inform policies to develop person‐oriented integrated mental healthcare for PLWH from low‐resource highly stigmatised settings.

**References**

1. Bernard C, Dabis F, De Rekeneire N. Prevalence and factors associated with depression in people living with HIV in sub‐Saharan Africa: a systematic review and meta‐analysis. PLoS One. 2017;12(8):e0181960.

2. Institute for Health Metrics and Evaluation. Global Burden of Disease (GBD) [Internet]. 2021 [cited 2024 Jul 5]. Available from: https://www.healthdata.org/research‐analysis/gbd.

## Co‐morbidities and complications of disease and/or treatment—Other

### P330 Treatment‐emergent integrase strand transfer inhibitor (INSTI) resistance‐associated mutations among people living with HIV‐1 treated with dolutegravir (DTG) + lamivudine (3TC) with pre‐existing M184V/I from real‐world and interventional studies

Dainielle Fox ^1^, Gary Blick^2^, Thibault Mesplède^3^, Gustavo Verdier^4^, Mónica Calderón^1^, Chris M Parry^5^, Richard Grove^6^, Emilio Letang^7^, Julie Priest^8^, Bryn Jones^9^

^1^Medical Affairs, ViiV Healthcare, Durham, NC, USA. ^2^HIV/AIDS, Health Care Advocates International, Stratford, CT, USA. ^3^Viroscience, Erasmus University Medical Center, Rotterdam, Netherlands. ^4^Medical Affairs, ViiV Healthcare, Montréal, Canada. ^5^Translational Medicine, ViiV Healthcare, Brentford, UK. ^6^Statistics, GlaxoSmithKline, Brentford, UK. ^7^Medical Affairs, ViiV Healthcare, Madrid, Spain. ^8^Health Outcomes, ViiV Healthcare, Durham, NC, USA. ^9^Medical Affairs, ViiV Healthcare, Brentford, UK

**Background**: The two‐drug regimen DTG + 3TC has a high barrier to resistance and is recommended as first‐line and maintenance therapy for people living with HIV‐1. Previous meta‐analysis estimates of virological failure were low among people using DTG + 3TC in real‐world and interventional settings regardless of baseline M184V/I presence [1]. We report INSTI mutation incidence in populations using DTG + 3TC, including those with pre‐existing M184V/I, from real‐world evidence (RWE) and interventional studies.

**Materials and methods**: A systematic literature review was conducted following Preferred Reporting Items for Systematic Reviews and Meta‐Analyses (PRISMA) guidelines, searching Ovid MEDLINE^®^, Embase^®^, PubMed, Cochrane library databases and relevant congresses for RWE and interventional studies reporting DTG + 3TC use in people living with HIV‐1 published between January 2013 and March 2024. All publications with ≥10 people using DTG + 3TC (all studies group) and all publications with ≥10 people using DTG + 3TC and reporting baseline M184V/I (pre‐existing M184V/I group) were included. To avoid double counting people from studies with multiple publications or overlap with another study population, unique populations were collectively represented by a “lead” publication with most recent study data and/or highest N.

**Results**: Overall, 300 publications (*n* = 249 RWE, *n* = 51 interventional) reporting DTG + 3TC use were identified, representing 108 discrete cohorts and trials and 47,350 unique people living with HIV‐1 after accounting for population overlap. Of those, 30 lead studies (*N* = 10,383) reported ≥10 people and M184V/I at baseline (Table 1). M184V/I prevalence was 5% among all lead studies reporting people with known baseline M184V/I and 20% among interventional studies, with many of the latter focused on populations with M184V/I. Treatment‐emergent INSTI resistance was reported in 6/47,350 (0.01%) people across all lead studies and 2/10,383 (0.02%) among the sub‐set of studies reporting people with baseline M184V/I. None of the treatment‐emergent INSTI resistance cases had known M184V/I at baseline.

**P330: Table 1:** Summary of systematic literature review–identified publications reporting data from people living with HIV‐1 with M184V/I at baseline by study type

	Lead^a^ RWE studies	Lead^a^ interventional studies	Total
Studies with *N* ≥10 people using DTG + 3TC and reporting pre‐existing baseline M184V/I			
Studies, *n*	22	8	30
People using DTG + 3TC, *N*	9287	1096	10,383
People with baseline M184V/I, n (%)	280 (3)^b^	219 (20)	499 (5)
RNA or unspecified	152 (2)^b^	186 (17)	338 (3)
DNA	128 (1)^c^	33 (3)	161 (2)
Resistance at virological failure, *n* (%)	5 (0.05)	0	5 (0.05)
INSTI resistance at virological failure, *n* (%)	2 (0.02)	0	2 (0.02)
With baseline M184V/I, *n*	0	0	0
All studies with *N* ≥10 people using DTG + 3TC			
Studies, *n*	84	24	108
People using DTG + 3TC, *N*	44,436	2914	47,350
People with baseline M184V/I, *n* (%)	172 (0.4)^b^	221 (8)	393 (0.8)
RNA or unspecified	149 (0.3)^b^	187 (6)	336 (0.7)
DNA	23 (0.05)^c^	34 (1)	57 (0.1)
Resistance at virological failure, *n* (%)	6 (0.01)	9 (0.3)	15 (0.03)
INSTI resistance at virological failure, *n* (%)	3 (0.01)	3 (0.1)	6 (0.01)
With baseline M184V/I, *n*	0	0	0

Abbreviations: 3TC, lamivudine; DTG, dolutegravir; INSTI, integrase strand transfer inhibitor; RWE, real‐world evidence.

^a^ The publication with the largest *N* for each RWE cohort or interventional trial was selected to represent that cohort/trial, to account for overlapping populations. Lead studies were selected after all systematic literature review–identified publications were screened either for studies reporting *N* ≥10 people using DTG + 3TC (all studies group) or for studies reporting both *N* ≥10 people using DTG + 3TC and people with M184V/I at baseline (pre‐existing M184V/I group). Four lead studies in the pre‐existing M184V/I group are sub‐studies of the lead studies in the all studies group, which therefore provides a higher estimate of people with baseline M184V/I;

^b^ one study only reported baseline resistance for the people using DTG + 3TC who experienced virological failure, not the full population;

^c^ *n* = 2 confirmed through personal communications.

**Conclusions**: No cases of treatment‐emergent INSTI resistance development were identified among people living with HIV‐1 who had M184V/I before DTG + 3TC therapy (0/499). Results indicate that using DTG + 3TC when M184V/I is present may not increase the risk of INSTI mutation development.

**Reference**

1. Kabra M, Barber TJ, Allavena C, Marcelin A‐G, Di Giambenedetto S, Pasquau J, et al. Virologic response to dolutegravir plus lamivudine in people with suppressed human immunodeficiency virus type 1 and historical M184V/I: a systematic literature review and meta‐analysis. Open Forum Infect Dis. 2023;10(11):ofad526.

### P331 Use of lipid‐lowering drugs, even when associated with polypharmacy, reduces risk of hospitalisation in PWH >50 years

Jovana Milic^1^, Alessandra Carobbio^2^, Antonia Pugliese^3^, Pierluigi De Cosmo^4^, Lorenzo Federici^5^, Federico Motta^6^, Filippo Calandra Buonaura^7^, Matteo Mantovani^7^, Francesca Gandolfi^3^, Chiara Mussi^1^, Cristina Mussini^7^, Giovanni Guaraldi ^7^

^1^Department of Biomedical and Metabolic Sciences and Neurosciences, University of Modena and Reggio Emilia, Modena, Italy. ^2^Department of Medical and Surgical Sciences for Children and Adults, University of Modena and Reggio Emilia, Modena, Italy. ^3^Dipartimento Farmaceutico Interaziendale, AUSL Modena, Distribuzione Diretta AUSL Modena, Modena, Italy. ^4^Infologic Srl, Padova, Italy. ^5^School of Medicine, University of Modena and Reggio Emilia, Modena, Italy. ^6^Computer and Mathematical Sciences, University of Modena and Reggio Emilia, Modena, Italy. ^7^Department of Surgical, Medical, Dental and Morphological Sciences, University of Modena and Reggio Emilia, Modena, Italy

**Background**: The objective of the study was to compare most commonly used drug classes and polypharmacy in people with and without HIV (PWH and PWoH) >50 years in relation to risk of same‐year hospitalisation.

**Materials and methods**: This was a cross‐sectional study of ART experienced PWH and PWoH >50 years in Modena, Italy. Inclusion criteria were: being resident and having a general practitioner in Modena, taking at least one drug class prescribed for chronic use according to the Anatomical Therapeutic Chemical (ATC) classification (including ART for PWH). Groups were matched in a 1:10 ratio for age and sex. A dedicated software (Navapharma) was able to trace any dispensing medication from both hospitals and all local pharmacies in Modena province in 2023. Polypharmacy was defined as chronic use of at least five drug classes. The risk of hospitalisation was defined as at least one hospitalisation for any cause in Modena province in 2023. Logistic regression model (LRM) was used to determine risk factors associated with hospitalisation.

**Results**: Three hundred and seventeen PWH and 3170 PWoH were included, median age was 60 years, 2409 (69.1%) were males. The comparison between the groups is summarised in Table 1. In the unadjusted model, PWH were at higher risk of 1‐year hospitalisation when compared to PWoH (OR 2.50; 95% CI 1.70–3.52; *p* < 0.001). The same was confirmed in LRM (OR 1.69; 95% CI 1.17–2.45; *p* = 0.005), after adjustment for polypharmacy (OR 1.29; 95% CI 1.21–1.37; *p* < 0.001), use of proton‐pump inhibitors (OR 1.80; 95% CI 1.31–2.47; *p* < 0.001) and lipid‐lowering drugs (LLD) (OR 0.62; 95% CI 0.45–0.85; *p* = 0.003). In LRM with interactions, both PWH (OR 3.48; 95% CI 1.94–6.25; *p* < 0.001) and PWoH (OR 1.58; 95% CI 1.13–2.21; *p* = 0.007) without use of LLD were at higher risk of hospitalisation, while PWH with polypharmacy and LLD had reduced risk of hospitalisation (OR 0.88; 95% CI 0.80–0.99; *p* = 0.02). Figure 1 represents adjusted predictions for hospitalisation according to polypharmacy, HIV status and use of LLD.

**P331: Table 1:** The differences according to polypharmacy, drug classes and hospitalisation between PWH and PWoH

	People with HIV (PWH) *N* = 317	People without HIV (PWoH) *N* = 3170	*p*‐value
Male sex, *N* (%)	219 (69.1%)	2190 (69.1%)	1.00
Age, years, median (Q1‐Q3)	60.0 (57.0–65.0)	60.0 (57.0–65.0)	1.00
Polypharmacy, *N* (%)	100 (31.5%)	396 (12.5%)	<0.001
Use of LLD, *N* (%)	194 (61.2%)	1321 (41.7%)	<0.001
Use of proton‐pump inhibitors, *N* (%)	66 (20.8%)	697 (22.0%)	0.63
Use of antidepressants, *N* (%)	52 (16.4%)	297 (9.4%)	<0.001
Use of antipsychotics, *N* (%)	13 (4.1%)	81 (2.6%)	0.11
Hospitalisation, *N* (%)	45 (14.2%)	199 (6.3%)	<0.001

**P331: Figure 1:**
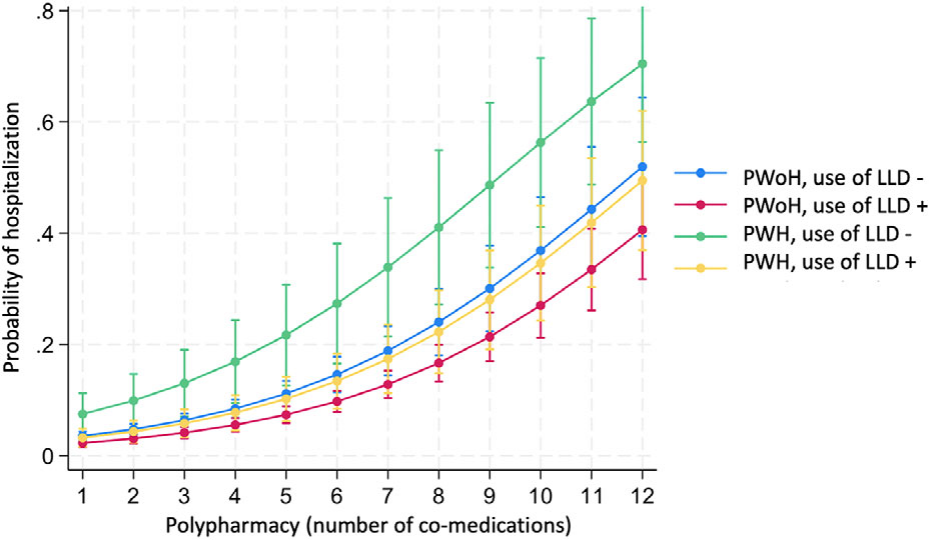
Adjusted predictions for hospitalisation according to polypharmacy, HIV status and use of lipid‐lowering drugs (LLD).

**Conclusions**: Prevalence of polypharmacy was higher in PWH and was associated with an increased risk for hospitalisation. Use of LLD, even when associated with polypharmacy, significantly reduced probability of hospitalisation, highlighting the importance of cardiovascular disease prevention and appropriate prescription in PWH.

### P332 Penile vascular status in young men living with HIV experiencing erectile dysfunction: a comparative cross‐sectional study

Giorgio Tiecco ^1^, Marco Di Gregorio^1^, Cosimo Colangelo^1^, Andrea Delbarba^2^, Matteo Riva^3^, Carlo Cappelli^4^, Emanuele Focà^1^, Francesco Castelli^1^, Eugenia Quiros‐Roldan^1^

^1^Clinical and Experimental Sciences, Infectious and Tropical Diseases Unit, University of Brescia, Brescia, Italy. ^2^Clinical and Experimental Sciences, Endocrinology and Metabolism, ASST Spedali Civili di Brescia, Brescia, Italy. ^3^Medical and Surgical Specialties, Radiological Sciences and Public Health Unit, University of Brescia, Brescia, Italy. ^4^Clinical and Experimental Sciences, Endocrinology and Metabolism Unit, University of Brescia, Brescia, Italy

**Background**: Erectile dysfunction (ED) is a prevalent concern among young men living with HIV (yMLWH). We aimed to assess the penile vascular status of yMLWH experiencing ED using dynamic penile color‐Doppler echography (dpCDE).

**Materials and methods**: This is a monocentric comparative cross‐sectional study in which we enrolled yMLWH attending our Unit of Infectious Diseases in Brescia, Italy. Inclusion criteria were a HIV infection and age between 18 and 50 years old. All yMLWH from June 2023 to December 2023 were asked for symptoms of ED during the routinely follow‐up visits. For those reporting ED, dpCDE was performed by an Andrology Specialist. Given that people living with HIV (PLWH) often experience age‐related comorbidities approximately 10 years earlier than the general population [1], we employed a comparative group of HIV‐negative individuals experiencing ED under the age of 60, referred to the same medical Andrology Unit during the same period. This comparative group was stratified by age into two cohorts: young (yC, under 50 years old), and middle‐aged controls (maC, aged 51–60 years old).

**Results**: In the study period, 310 yMLWH were assessed for eligibility, 50 (50/310, 16.1%) reported ED and were enrolled. A comprehensive summary of the viro‐immunological characteristics of the sample is presented in Table 1. Regarding the dpCDE results, the proportion of yMLWH with pathological intima‐media thickness (IMT) was significantly higher compared to that of the yC (76.0% vs. 61.0%, *p* = 0.004) (Figure 1a). Although, not statistically significant (*p* = 0.053), yMLWH exhibited a higher prevalence of pathological end‐diastolic velocity (EDV) compared to both the yC and maC (Figure 1b). Furthermore, yMLWH showed more frequently altered peak systolic velocity (PSV) when compared to the yC, although this difference was not observed when compared to the maC (*p* = 0.074) (Figure 1c).

**P332: Table 1:** Viro‐immunological characteristics of the yMLWH included

Demographic	
Sample size, *n* (%)	50 (100)
Age (years), mean (SD)	44.4 (4.34)
Viro‐immunological parameters	
Years living with HIV, mean (SD)	13.9 (6.77)
CD4 nadir, mean (SD)	285.7 (202.53)
HIV‐RNA zenith, mean (SD)	377,176.6 (863,099.40)
Patients with HIV‐RNA <20 copies/mL, *n* (%)	50 (100)
Current CD4 cells count, mean (SD)	837.9 (417.57)
Current CD4 cells %, mean (SD)	32.1 (12.05)
Current CD8 cells count, mean (SD)	1073.4 (477.60)
Current CD8 cells %, mean (SD)	40.0 (10.35)
Current CD4/CD8 ratio, mean (SD)	0.9 (0.45)
Patients on dolutegravir‐based dual therapy, *n* (%)	28 (56)

**P332: Figure 1:**
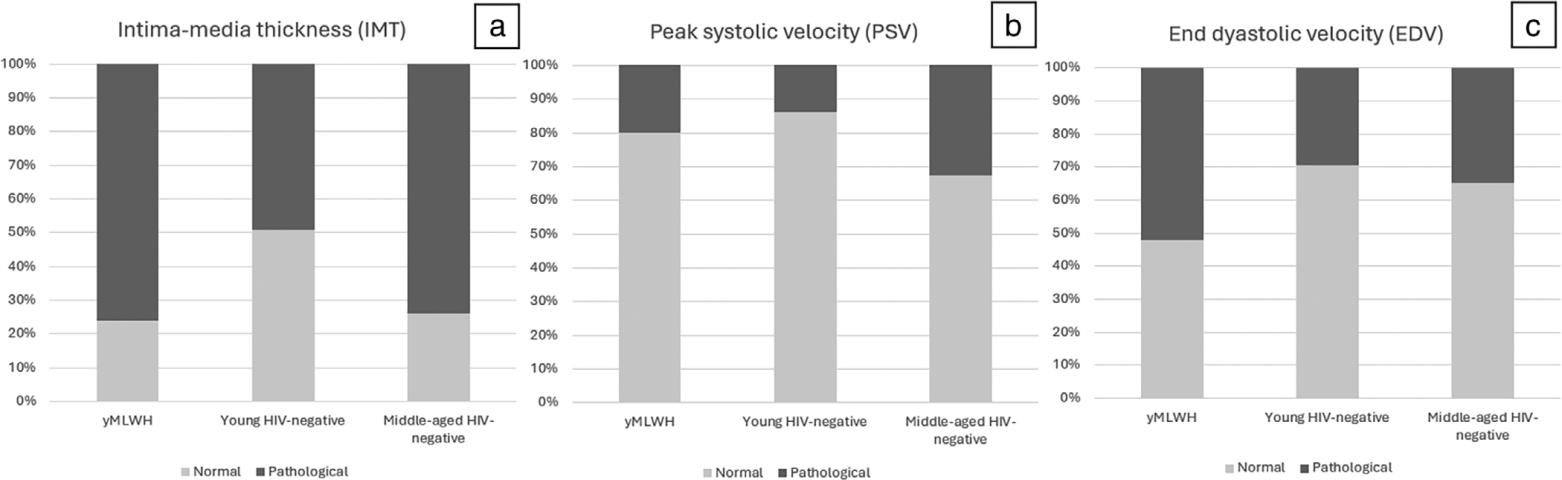
Comparison of different dpCDE parameters among yMLWH, young HIV‐negative individuals (under the age of 50), and middle‐aged HIV‐negative individuals (between 50 and 60 years old). (a) Bilateral IMT (normal/pathological) among the three populations; (b) bilateral PSV (normal/pathological) among the three different populations; (c) bilateral EDV (normal/pathological) among the three different populations. Cut off used: according to the International Andrology Guidelines, IMT was considered pathological if it was ≥0.3 mm; PSV was considered pathological if it was ≤35 cm/s; EDV was considered pathological if it was ≥5 cm/s. yMLWH, young men living with HIV.

**Conclusions**: In our cohort, the proportion of yMLWH exhibiting pathological penile IMT was similar than that observed in a population 10 years older. IMT is considered as an early predictor of major cardiovascular events in specific populations, such as individuals with diabetes. All yMLWH with pathological IMT were instructed to undergo a carotid artery echocardiogram and a treadmill.

**Reference**

1. Guaraldi G, Orlando G, Zona S, Menozzi M, Carli F, Garlassi E, et al. Premature age‐related comorbidities among HIV‐infected persons compared with the general population. Clin Infect Dis. 2011;53(11):1120‐6.

### P333 The prevalence and factors associated with polypharmacy in participants with HIV in the Pharmacokinetic and clinical Observations in PeoPle over fiftY (POPPY) study over a 3‐5 year period

Luxsena Sukumaran ^1^, Alan Winston^2^, Patrick W G Mallon^3^, Frank A Post^4^, Memory Sachikonye^5^, Marta Boffito^2^, Nicki Doyle^2^, Jaime Vera^6^, Jane Anderson^7^, Fiona M Burns^1^, Caroline A Sabin^1^

^1^Institute for Global Health, University College London, London, UK. ^2^Department of Infectious Disease, Imperial College London, London, UK. ^3^School of Medicine, University College Dublin, Dublin, Ireland. ^4^Department of Inflammation Biology, School of Immunology & Microbial Sciences, King's College London, London, UK. ^5^UK Community Advisory Board (UK‐CAB), London, UK. ^6^Brighton and Sussex Medical School, University of Brighton and the University of Sussex, Brighton and Hove, UK. ^7^HIV Medicine, Homerton University Hospital NHS Trust, London, UK

**Background**: Polypharmacy is increasingly common among people living with HIV and associated with adverse outcomes, such as organ injury and hospitalisation. However, the progression of polypharmacy and its contributing factors remain poorly understood. We compared baseline characteristics and the onset of polypharmacy over 2–3 years in individuals with and without polypharmacy.

**Methods**: Information on POPPY participant's medication usage was obtained at baseline and visit 3 by trained study staff. Polypharmacy at baseline was defined as the concurrent use of five or more non‐antiretroviral (non‐ART) medications. Incident polypharmacy was determined as participants who transitioned from using fewer than five non‐ART medications at baseline to five or more at the third visit. Demographic (age, sex, ethnicity, UK‐born status), social (education, employment, smoking, recreational drug use) and clinical (number of antiretrovirals [ARVs], ART duration, CD4+ Tcell count, number of comorbidities) factors were compared between groups using Kruskal‐Wallis and *χ2* tests.

**Results**: Analysis included 834 POPPY participants with HIV who attended their third study visit. The majority of participants were male (85.5%), men having sex with men (MSM, 77.2%) and of white ethnicity (85.7%). The median (interquartile range [IQR]) number of non‐ART medications increased from 2 [1–4] at baseline to 3 [1–6] at visit 3. Baseline polypharmacy was observed in 22.2% of participants, who were older (median [IQR] age: 55 [50–60] vs. 52 [46–59] years), had more comorbidities (9 [7–11] vs. 5 [3–7]), longer ART duration (median 12.3 vs. 8.6 years) and lower rates of employment (26.0% vs. 55.2%) and university education (33.0% vs. 50.4%), compared to those without polypharmacy (all *p* < 0.001) (Table 1). Incident polypharmacy was reported by 21.7% of participants at visit 3, who were more likely to be male (90.1% vs. 9.9%, *p* = 0.03), UK‐born (74.5% vs. 25.5%, *p* < 0.001) and MSM (83.7% vs. 16.3%, *p* = 0.02).

**P333: Table 1:** Demographic, social and clinical characteristics of POPPY participants with HIV with polypharmacy at baseline or incident polypharmacy at visit 3

	Baseline polypharmacy (*n* = 834)			Incident polypharmacy at visit 3 (*n* = 649)		
	No	Yes	*p*‐value	No	Yes	*p*‐value
*N* (%) or median (IQR)	649 (77.8)	185 (22.2)		508 (78.3)	141 (21.7)	
Age	52 (46–59)	55 (50–60)	<0.001	52 (46–58)	55 (50–61)	<0.001
Gender						
Female	102 (15.7)	19 (10.3)	0.06	88 (17.3)	14 (9.9)	0.03
Male	547 (84.3)	166 (89.7)		420 (82.7)	127 (90.1)	
Ethnicity						
Black African	95 (14.6)	24 (13.0)	0.57	82 (16.1)	13 (9.2)	0.04
White	554 (85.4)	161 (87.0)		426 (83.9)	128 (90.8)	
Born in the UK						
No	243 (37.4)	62 (33.5)	0.33	207 (40.8)	36 (25.5)	<0.001
Yes	406 (62.6)	123 (66.5)		301 (59.3)	105 (74.5)	
Sexuality						
Heterosexual	156 (24.0)	34 (18.4)	0.11	133 (26.2)	23 (16.3)	0.02
MSM	493 (76.0)	151 (81.6)		375 (73.8)	118 (83.7)	
Employed	358 (55.2)	48 (26.0)	<0.001	291 (57.3)	67 (47.5)	0.04
Completed university education	327 (50.4)	61 (33.0)	<0.001	257 (50.6)	70 (49.7)	0.84
Current smoker	151 (41.3)	43 (36.1)	0.32	118 (42.0)	33 (38.8)	0.60
Recreational drug use	181 (27.9)	48 (26.0)	0.60	133 (26.2)	48 (34.0)	0.07
Number of ARVs	3 (3–4)	3 (3–4)	0.22	3 (3–4)	3 (3–4)	0.84
Years since ART	8.6 (4.2–15.3)	12.3 (5.6‐17.5)	<0.001	8.1 (3.8–14.8)	11.3 (5.6‐17.4)	<0.001
CD4+ T cell count	628 (484–810)	620 (470–835)	0.64	628 (490–810)	630 (473–810)	0.62
Number of comorbidities	5 (3–7)	9 (7–11)	<0.001	4 (3–7)	9 (7–11)	<0.001

**Conclusions**: The prevalence of polypharmacy had increased within this cohort, with over 20% of participants developing incident polypharmacy over a 2–3 year period. These findings provide insights into identifying subgroups who may be at a higher risk of developing polypharmacy. Future research will explore the impact of polypharmacy burden on health outcomes, healthcare use and quality of life.

### P334 Mortality using raltegravir versus other integrase inhibitors in people with HIV in Europe and Australia

Erich Tusch ^1^, Lene Ryom^2^, Christian Hoffmann^3^, Olaf Degen^4^, Robert Zangerle^5^, Huldrych Günthard^6^, Ferdinand Wit^7^, Cristina Mussini^8^, Antonella Castagna^9^, Charlotte Martin^10^, Andrea Giacomelli^11^, Jörg Janne Vehreschild^12^, Josip Begovac^13^, Vani Vannappagari^14^, Jim Rooney^15^, Lital Young^16^, Joan Tallada^17^, Justyna Kowalska^18^, Elmar Wallner^5^, Katharina Kusejko^19^, Nadine Jaschinski^1^, Jens Lundgren^1^, Lars Peters^1^, Joanne Reekie^1^

^1^Centre of Excellence for Health, Immunity and Infections, Rigshospitalet, Copenhagen, Denmark. ^2^Department of Infectious Diseases, Hvidovre University Hospital, Copenhagen, Denmark. ^3^ICH Study Center, Hamburg, Germany. ^4^I. Medical Clinic and Polyclinic, University Clinic Hamburg Eppendorf, Hamburg, Germany. ^5^Medical University of Vienna, Vienna, Austria. ^6^Institute of Medical Virology, University of Zurich, Zurich, Switzerland. ^7^AIDS Therapy Evaluation in the Netherlands Cohort, HIV Monitoring Foundation, Amsterdam, Netherlands. ^8^Modena HIV Cohort, Università degli Studi di Modena, Modena, Italy. ^9^San Raffaele Scientific Institute, Università Vita‐Salute San Raffaele, Milan, Italy. ^10^CHU Saint‐Pierre, Centre de Recherche en Maladies Infectieuses a.s.b.l., Brussels, Belgium. ^11^Italian Cohort Naive Antiretrovirals (ICONA), ASST Santi Paolo e Carlo, Milan, Italy. ^12^Department I of Internal Medicine, Faculty of Medicine and University Hospital Cologne, Cologne, Germany. ^13^University Hospital Zagreb, Zagreb, Croatia. ^14^ViiV Healthcare, Durham, NC, USA. ^15^Gilead Science, Foster City, CA, USA. ^16^Merck Sharp & Dohme, Rahway, NJ, USA. ^17^European AIDS Treatment Group, Brussels, Belgium. ^18^Department of Adults' Infectious Diseases, Medical University of Warsaw, Warsaw, Poland. ^19^Swiss HIV Cohort Study (SHCS), University of Zurich, Zurich, Switzerland

**Background**: Integrase inhibitors (INSTI) are recommended as part of first‐line antiretroviral therapy (ART). Raltegravir (RAL) was the first INSTI approved and remains recommended for specific clinical indications. A prior study among ART‐naïve [1] found higher all‐cause mortality for RAL‐based first‐line ART compared with other regimens, including INSTI based regimens dolutegravir (adjusted hazard ratios [aHR] 1.49) and elvitegravir [aHR 1.86]. We investigated all‐cause mortality between RAL‐based ART versus other INSTIs in the RESPOND cohort consortium.

**Methods**: ART naïve and experienced participants enrolled in the RESPOND cohort who started their first INSTI during follow‐up between 2012 and 2021 were included. Survival was compared between those starting RAL as their first INSTI versus any other INSTI using Cox proportional hazards regressions, one model adjusting for age and another weighted by inverse propensity of treatment weights (IPTW) to estimate the average treatment effect. Predictors of RAL assignment were estimated by logistic regression after lasso regression variable selection.

**Results**: A total of 20,349 participants were included with 94,677 person‐years of follow‐up (PYFU); median 4.8 years (2.9–6.4); 938 died during follow‐up. See Table 1 for baseline characteristics. Crude mortality rates (MR) were higher for participants starting RAL (*n* = 312; MR 12.9 per 1000 PYFU; 95% CI 11.5–14.5) than other INSTIs (*n* = 626; MR 9.1 per 1000 PYFU; 95% CI 8.4–9.8). Starting RAL as first INSTI was associated with increased mortality when controlling for age (aHR 1.43; 95% CI 1.25–1.65) (Figure 1a). However, after applying IPTW, there was no difference between starting RAL and other INSTIs in the full sample (HR 1.13; 95% CI 0.93–1.34) (Figure 1b) or among ART‐naïve (HR 1.20; 95% CI 0.67–2.03). Starting RAL was associated with CD4 nadir ≤200 cells/mm^3^ (aOR vs. >500 1.41; 1.04–1.90) and higher HIV viral load. The clinical risk factors for mortality that were most strongly associated with starting RAL versus other INSTIs were prevalent end‐stage renal disease (aOR 2.58; 95% CI 1.58–4.20), cardiovascular disease (aOR 1.57; 95% CI 1.30–1.90), and hepatitis C (HCV) positive status (aOR vs. anti‐HCV negative 2.07; 95% CI 1.81–2.36).

**Conclusions**: While there was an age‐adjusted association between starting RAL and mortality, this was no longer the case after accounting for confounding at baseline using IPTW.

**P334: Table 1:** Characteristics at baseline

Baseline covariate	All participants	Other INSTI group	RAL group
Age in years (median (IQR))	47 (38–54)	47 (38–55)	48 (39–54)
Sex/gender			
Male	15,429 (75.8%)	12,378 (76.6%)	3051 (72.9%)
Female	4879 (24%)	3750 (23.2%)	1129 (27%)
Transgender	41 (0.2%)	37 (0.2%)	4 (0.1%)
HIV exposure group			
MSM	9606 (47.2%)	7743 (47.9%)	1863 (44.5%)
IDU	2608 (12.8%)	1986 (12.3%)	622 (14.9%)
Heterosexual contact	6857 (33.7%)	5444 (33.7%)	1413 (33.8%)
Other/unknown	1278 (6.3%)	992 (6.1%)	286 (6.8%)
Time period			
Early (2012–2016)	11,656 (57.3%)	8312 (51.4%)	3344 (79.9%)
Late (2017–2021)	8693 (42.7%)	7853 (48.6%)	840 (20.1%)
Indication of pregnancy at baseline	231 (1.1%)	139 (0.9%)	92 (2.2%)
HIV viral load (copies/ml)			
≤50	13,307 (65.4%)	10,740 (66.4%)	2567 (61.4%)
50–200	1054 (5.2%)	751 (4.6%)	303 (7.2%)
200–10,000	1669 (8.2%)	1241 (7.7%)	428 (10.2%)
10,000–100,000	2143 (10.5%)	1745 (10.8%)	398 (9.5%)
>100,000	2176 (10.7%)	1688 (10.4%)	488 (11.7%)
CD4 cell count (cells/mm^3^)			
≤50	648 (3.2%)	465 (2.9%)	183 (4.4%)
50–200	1571 (7.7%)	1142 (7.1%)	429 (10.3%)
200–350	2544 (12.5%)	1928 (11.9%)	616 (14.7%)
350–500	3726 (18.3%)	2951 (18.3%)	775 (18.5%)
>500	11,860 (58.3%)	9679 (59.9%)	2181 (52.1%)
CD4 cell count nadir (cells/mm^3^)			
≤200	2581 (12.7%)	1852 (11.5%)	729 (17.4%)
200–500	7397 (36.4%)	5806 (35.9%)	1591 (38%)
>500	10,371 (51%)	8507 (52.6%)	1864 (44.6%)
Prior AIDS diagnosis	4600 (22.6%)	3432 (21.2%)	1168 (27.9%)
AIDS‐defining malignancy	1001 (4.9%)	745 (4.6%)	256 (6.1%)
Non‐AIDS‐defining malignancy	926 (4.6%)	685 (4.2%)	241 (5.8%)
Use of chemotherapy near baseline	127 (0.6%)	91 (0.6%)	36 (0.9%)
Tuberculosis history	826 (4.1%)	599 (3.7%)	227 (5.4%)
End‐stage liver disease	148 (0.7%)	92 (0.6%)	56 (1.3%)
Chronic kidney disease	693 (3.4%)	495 (3.1%)	198 (4.7%)
End‐stage renal disease	95 (0.5%)	42 (0.3%)	53 (1.3%)
Diabetes mellitus	1374 (6.8%)	1007 (6.2%)	367 (8.8%)
Cardiovascular disease	804 (4%)	564 (3.5%)	240 (5.7%)
ART‐experienced pre‐baseline	15,745 (77.4%)	12,380 (76.6%)	3365 (80.4%)
Reason for discontinuation of prior ART regimen			
Patient/physician choice	3357 (16.5%)	2693 (16.7%)	664 (15.9%)
Treatment failure	950 (4.7%)	638 (3.9%)	312 (7.5%)
Treatment simplification	2972 (14.6%)	2824 (17.5%)	148 (3.5%)
Toxicity	3915 (19.2%)	2780 (17.2%)	1135 (27.1%)
Unknown	1960 (9.6%)	1376 (8.5%)	584 (14%)
Other (not including pregnancy‐related)	3455 (17%)	2751 (17%)	704 (16.8%)

Abbreviations: ART, antiretroviral therapy; IDU, injection drug use; INSTI, integrase inhibitor; MSM, men who have sex with men; RAL, raltegravir.

**P334: Figure 1:**
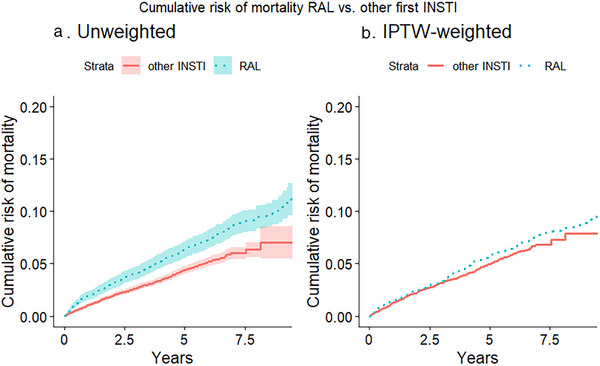
Cumulative risk of mortality in unweighted and weighted population.

**Reference**

1. Trickey A, Zhang L, Gill MJ, Bonnet F, Burkholder G, Castagna A, et al. Associations of modern initial antiretroviral drug regimens with all‐cause mortality in adults with HIV in Europe and North America: a cohort study. Lancet HIV. 2022;9(6):e404‐13.

### P335 Stigma in older people with HIV: disclosure concerns and self‐image do not improve over time in older AGEhIV cohort study participants

Kevin Moody ^1^, Myrthe L Verburgh^1^, Ferdinant WNM Wit^2^, Sarah Stutterheim^3^, Maarten F Schim van der Loeff^4^, Peter Reiss^5^, Pythia T Nieuwkerk^6^, Marc van der Valk^2^

^1^Division of Infectious Diseases, Amsterdam University Medical Center, University of Amsterdam, Amsterdam Institute for Immunology and Infectious Diseases, Amsterdam, Netherlands. ^2^Division of Infectious Diseases, Amsterdam University Medical Center, University of Amsterdam, Amsterdam Institute for Immunology and Infectious Diseases, Stichting HIV Monitoring, Amsterdam, Netherlands. ^3^Department of Health Promotion & Care and Public Health Research Institute, Maastricht University, Maastricht, Netherlands. ^4^Department of Infectious Diseases, Public Health Service of Amsterdam, Amsterdam, Netherlands. ^5^Department of Global Health, Amsterdam University Medical Center, University of Amsterdam, Amsterdam Institute for Immunology and Infectious Diseases, Amsterdam, Netherlands. ^6^Department of Medical Psychology, Amsterdam University Medical Center, University of Amsterdam, Amsterdam Institute for Immunology and Infectious Diseases, Amsterdam, Netherlands

**Background**: Stigma impedes the HIV care continuum and contributes to problematic mental health outcomes, non‐adherence and reduced quality of life [1–3]. We prospectively analysed changes in HIV stigma over a 10‐year period among older people with HIV (PWH), and its associations with socio‐demographic and clinical factors.

**Materials and methods**: PWH participating in the AGEhIV Cohort Study (*n* = 116, 93% male, 98% Caucasian, and at baseline, median age 55.5 years and 96% suppressed HIV‐1 RNA) completed the full Berger HIV Stigma Scale (HSS) between 2012–2014 (baseline) and two of the HSS subscales—i.e., disclosure concerns and negative self‐image (range per subscale 3–12) – 10 years later (T1) as a part of routine clinical care. Changes in scores over time were assessed using paired samples *t*‐tests. The influence of demographic factors (age, sex, ethnicity, marital status, household size, education, work type, sexual orientation, tobacco and alcohol use) and clinical factors (years since diagnosis, pre‐1996 and pre‐2010 diagnosis, mode of transmission, HIV‐1 RNA, CD4, CD8, nadir CD4, AIDS diagnosis, depressive symptoms) were assessed at T1 using linear regression.

**Results**: Disclosure concerns increased significantly, albeit minimally, between baseline and T1 (mean change 0.40, SD 1.43, *p* = 0.003), whereas negative self‐image scores remained stable (mean change 0.10, SD 1.73, *p* = 0.521). Higher disclosure concerns over time were linked to unsuppressed HIV (mean increase 3.68, 95% CI 0.93–6.43, *p* = 0.009) and being married or cohabitating (mean increase 0.52, 95% CI 0.08–1.03, *p* = 0.047). Women experienced a significant increase in negative self‐image scores (mean increase 1.32, 95% CI 0.08–2.56, *p* = 0.037). Increased disclosure concerns were also correlated with higher negative self‐image scores (r = 0.39, *p* < 0.001).

**Conclusions**: In this cohort of predominately older, white Dutch men with mostly suppressed HIV, disclosure concerns and self‐image did not improve over time, which is disconcerting. These HIV‐related stigma domains worsened mostly among women, those with unsuppressed HIV, and those who were married or cohabitating. The results provide insights for the development of targeted interventions to address stigma in these areas. Future longitudinal studies should explore specific aspects of stigma and their relationships with socio‐demographic and clinical factors.

**References**

1. van der Kooij YL, Kupková A, den Daas C, van den Berk GEL, Kleene MJT, Jansen HSE, et al. Role of self‐stigma in pathways from HIV‐related stigma to quality of life among people living with HIV. AIDS Patient Care STDS. 2021;35(6):231‐8.

2. Stutterheim SE, Kuijpers KJR, Waldén MI, Finkenflügel RNN, Brokx PAR, Bos AER. Trends in HIV stigma experienced by people living with HIV in the Netherlands: a comparison of cross‐sectional surveys over time. AIDS Educ Prev. 2022;34(1):33‐52.

3. Zhabokritsky A, Klein M, Loutfy M, Guaraldi G, Andany N, Guillemi S, et al. Non‐AIDS‐defining comorbidities impact health related quality of life among older adults living with HIV. Front Med (Lausanne). 2024;11:1380731.

### P336 Prevalence of unmet need for support managing long‐term conditions among people with HIV

Adamma Aghaizu ^1^, Hannah Kitt^1^, Carole Kelly^1^, Alison Brown^1^, Alex Sparrowhawk^2^, Fiona Wilson^3^, Judith Zhou^4^, Meaghan Kall^5^, Alison Rodger^6^, Janey Sewell^6^, Annegret Pelchen‐Matthews^6^, Colette Smith^7^, Valerie Delpech^8^, Veronique Martin^1^, Clare Humphreys^9^

^1^Blood Safety, Hepatitis, Sexually Transmitted Infections and HIV Division, UK Health Security Agency, London, UK. ^2^HIV Advice, Support and Information Services, George House Trust, Manchester, UK. ^3^HIV and Infectious Diseases, Addenbrooke's Hospital, Cambridge, UK. ^4^Worthing Sexual Health Clinic, University Hospitals Sussex, Worthing, UK. ^5^COVID‐19 Vaccines and Epidemiology Division, UK Health Security Agency, London, UK. ^6^Institute for Global Health, University College London, London, UK. ^7^Infection and Population Health, University College London, London, UK. ^8^Population and Public Health, NSW Health, Sydney, Australia. ^9^Health Protection Operations, UK Health Security Agency, Oxford, UK

**Background**: The population of people with HIV is ageing and consequently age‐related comorbidities are on the rise. We assessed the need for long‐term condition management support and the most prevalent diagnosed comorbidities among those with unmet need.

**Methods**: Positive Voices 2022 surveyed 4540 people with HIV aged 18+ years between April 2022 and March 2023 from 101 HIV clinics in England, Wales and Scotland, representing ∼5% of people with HIV in the UK. Data collected included need and unmet need for support managing long‐term conditions in addition to HIV, prevalence of diagnosed non‐HIV long‐term conditions and socio‐demographic factors. Data were compared to Positive Voices 2017.

**Results**: Despite increases in the prevalence of comorbidities among people with HIV from 62% (2699/4377) in 2017 to 69% (3052/4404) in 2022, the proportion of participants that reported a need for long‐term condition management support decreased over that period from 42% (1721/4130) in 2017 to 33% (1383/4205) in 2022. However, the proportion of this need that was unmet increased from 33% (563/1721) to 47% (646/1383). Of those that had an unmet need for long‐term condition management support, 78% (497/634) had at least one diagnosed long‐term condition in addition to their HIV and 53% (336/634) had two or more. In people aged 65+ years, 89% (509/574) had at least one diagnosed comorbidity compared to 42% (141/336) aged <35 years. Participants did not indicate for which conditions they had an unmet need. However, among those with an unmet need, the diagnosed conditions with highest prevalences were high cholesterol (35%, 208/508), hypertension (30%, 179/595), asthma (20%, 117/581) and erectile dysfunction (19%, 111/575).

**Conclusions**: Whilst the overall need for support with long‐term condition management declined over the previous 5 years, the proportion of people with unmet need increased and affected approximately one in seven of all people with HIV in the UK. Further work is needed to better characterise people with this unmet needs and understand ways in which they can be better supported.

### P337 Profile of suicide cases in the Spanish CoRIS cohort between 2004‐2022

Julián Olalla^1^, Alfonso Del Arco ^1^, Melchor Riera^2^, Joaquim Peraire^3^, Enrique Bernal^4^, Javier De la Torre^5^, Antonia Castillo‐Navarro^6^, Lorena Martínez‐Fernández^7^, Javier Pérez‐Stachowski^5^, Santiago Moreno^8^

^1^Internal Medicine, Hospital Universitario Costa del Sol, Marbella, Spain. ^2^Medicina Interna‐Enfermedades Infecciosas, Hospital Universitario Son Espases, IDISBA, CIBERINFEC, Carlos III Health Institute, Palma, Spain. ^3^Enfermedades Infecciosas, Hospital Universitari Joan XXIII, IISPV, CIBERINFEC, Carlos III Health Institute, Tarragona, Spain. ^4^Enfermedades Infecciosas, Hospital General Universitario Reina Sofía, Murcia, Spain. ^5^Medicina Interna, Hospital Universitario Costa del Sol, Marbella, Spain. ^6^UNITS‐Medicina Interna, Hospital Clínico Universitario Virgen de la Arrixaca, Murcia, Spain. ^7^Medicina Interna‐Infecciosas, Hospital General Universitario Santa Lucia, Cartagena, Spain. ^8^Servicio de Enfermedades Infecciosas, Hospital Universitario Ramón y Cajal—CIBERINFEC, Carlos III Health Institute, Madrid, Spain

**Background**: Suicide is a relevant cause of death in different cohorts of people living with HIV (PLHIV). The objective of this work is to review mortality due to suicide in PLHIV in Spain, members of the CoRIS cohort between 2004 and 2022.

**Methods**: Socio‐demographic and viro‐immunological characteristics, along with the presence of comorbidities were collected from patients with a death diagnosis of suicide.

**Results**: In a total of 134,375 person‐years between 2004 and 2022, 22 suicides were recorded, with an incidence rate of 16.37 cases/100,000 inhabitants. Of these, 63.6% were Spanish, with an average age of 37.7 years and 90.9% were men, ​​similar to the distribution of the entire cohort. Among suicides, injection drug users were 6.4% compared to 18.2% in the total cohort. In terms of education level, 4.5% did not complete primary education, 45.5% completed compulsory education, 27.3% completed high school and 22.7% completed higher education. The median baseline CD4 lymphocyte count was 414 cells/µl (IQR 207–613) and the median baseline viral load was 76,768 copies/ml (IQR 41,182–423,000). Three patients were not receiving ART, six received triple therapy based on integrase inhibitors, three on protease inhibitors (PI), six on non‐nucleoside reverse transcriptase inhibitors, two monotherapy with PI, one dual therapy based on PI and one whose treatment was not stated. Before the suicide, 16 had viral load <50 copies/ml, two had unavailable data, and four had detectable viral load (>50 copies/ml). Two patients presented AIDS events; 15 ≥1 non‐AIDS event. Of the 21 non‐AIDS events, 10 were suicide attempts, four depression, three psychosis, one diabetes mellitus, one bone fracture, one pulmonary hypertension, and one renal tubulopathy. Of the 10 suicide attempts, two had depression. Five patients were taking medications other than antiretroviral treatment, two of them with a number of drugs ≥5. Of the total of 19 registered medications, 14 were related to central nervous system drugs.

**Conclusions**: The suicides rate in PLHIV in the CoRIS cohort doubles that of the Spanish population. In this population, there is a higher percentage of injecting drug users and the main non‐AIDS event was attempted.

### P338 The clinical utility of targeted screening for liver fibrosis/cirrhosis among people living with HIV (PLWH)

Izuchukwu Ezeh ^1^, Amy Hardy^1^, Martin Prince^2^, Vincent Lee^1^

^1^The Northern Integrated Contraception, Sexual Health and HIV Service, Manchester University NHS Foundation Trust, Manchester, UK. ^2^Department of Hepatology, Manchester University NHS Foundation Trust, Manchester, UK

**Background**: The Community Liver Health Checks Programme offers population‐level screening to identify those with fibrosis or cirrhosis. In this study, we describe the population characteristics and results for our cohort to ascertain the clinical utility of routine FibroScan.

**Methods**: Prospective observational study of PLWH who had a FibroScan as part of the community liver health screen. All participants had identified risk factor(s) for liver fibrosis.

**Results**: Three hundred and eighty‐two were included in the study. Seventy‐six percent (*n* = 291) were male, with a median age of 48 years (range 26–75). Fifty percent (*n* = 191) were white British and 31.4 % (*n* = 120) were Black African. Sixty‐two percent (*n* = 237) were men who have sex with men (MSM). Median time since HIV diagnosis was 16 years, with a median CD4 of 642. Ninety‐five percent were undetectable. The most common indication for FibroScan referral was BMI >30 in 47.4% (*n* = 181). Other common indications include non‐alcoholic fatty liver disease (NAFLD) (31%, *n* = 118), alcohol (27.1%, *n* = 103). 24.9% (*n* = 95) had more than two risk factors. Overall, 53.7% (*n* = 205) had evidence of steatosis (≥S1), 19.4% (*n* = 74) had evidence of fibrosis (≥F1) and 1% (*n* = 4) had evidence of cirrhosis. In bivariate analysis, BMI >30 and a known diagnosis of NAFLD were associated with steatosis (≥S1) (OR 3.1 and 1.8 respectively). The antiretroviral combination of TAF and INI (excluding Genvoya) had statistically significant association with ≥S1 steatosis (OR 3.4). Of those with multiple risk factors (≥2), 74.7% (*n* = 71) had evidence of steatosis, 20% (*n* = 19) had evidence of fibrosis, 2.1% (n = 2) had evidence of cirrhosis. Bivariate analysis shows a significant correlation between the presence of multiple risk factors and ≥F1 fibrosis (OR 2.3) and ≥S1 steatosis (OR 3.2).

**Conclusions**: Our data show a low prevalence of liver cirrhosis. Routine FibroScan may not be cost effective in diagnosing cirrhosis in PLWH with a single liver risk factor; however, prevalence increases in the presence of multiple risk factors. We recommend fibrosis‐4 index (FIB‐4) score as an inexpensive primary screening tool for fibrosis/cirrhosis, and FibroScan for those with multiple risk factors or high FIB‐4 score.

### P339 Patient‐reported outcome measures among people with HIV on antiretroviral therapy in Kunming, China

Yongmei Jin, Jun Liu, Bo Tian, Chongxi Li

HIV/AIDS Department, Third People's Hospital of Kunming, Kunming, China

**Background**: Patient‐reported outcome measures (PROMs) have become important indicators among outcomes of antiretroviral therapy (ART) regimens. Existing studies of PROMs on current ART in China, however, are limited. This study was aimed to access PROMs among people living with HIV (PLWH) receiving different ART regimens in Kunming, China.

**Methods**: A cross‐sectional study was conducted at Third People's Hospital of Kunming from December 2021 to April 2022. A structured questionnaire was used to assess socio‐demographic characteristics and PROMs, including HIV‐related symptoms, anxiety, depression, sleep quality and health‐related quality of life (HRQOL). PROMs were compared across efavirenz (EFV)‐containing, integrase strand transfer inhibitor (INSTI)‐containing versus other ART regimens. A multivariate linear model adjusted for confounders estimated the association between ART regimens and the total HRQOL.

**Results**: Among 1200 participants (mean age 42.4 years, 63.6% male), 46.4% received EFV‐containing regimen, 29.7% INSTIs‐containing regimen, and 23.9% other ART regimens. HIV‐related symptoms were reported by 83.5% of participants. Individuals on INSTIs reported significantly fewer neuropsychiatric symptoms, anxiety, depression, and sleep quality compared to EFV and other regimens (*p* < 0.001) (Figure 1). Those on INSTIs had the highest total HRQOL score (72.7 ± 11.1), followed by the other ART group (67.9 ± 8.8) and EFV group (64.8 ± 9.3) (Figure 2). After adjusting for variables, INSTIs group showed better HRQOL compared to EFV and other ART group (*p* < 0.05).

**P339: Figure 1:**
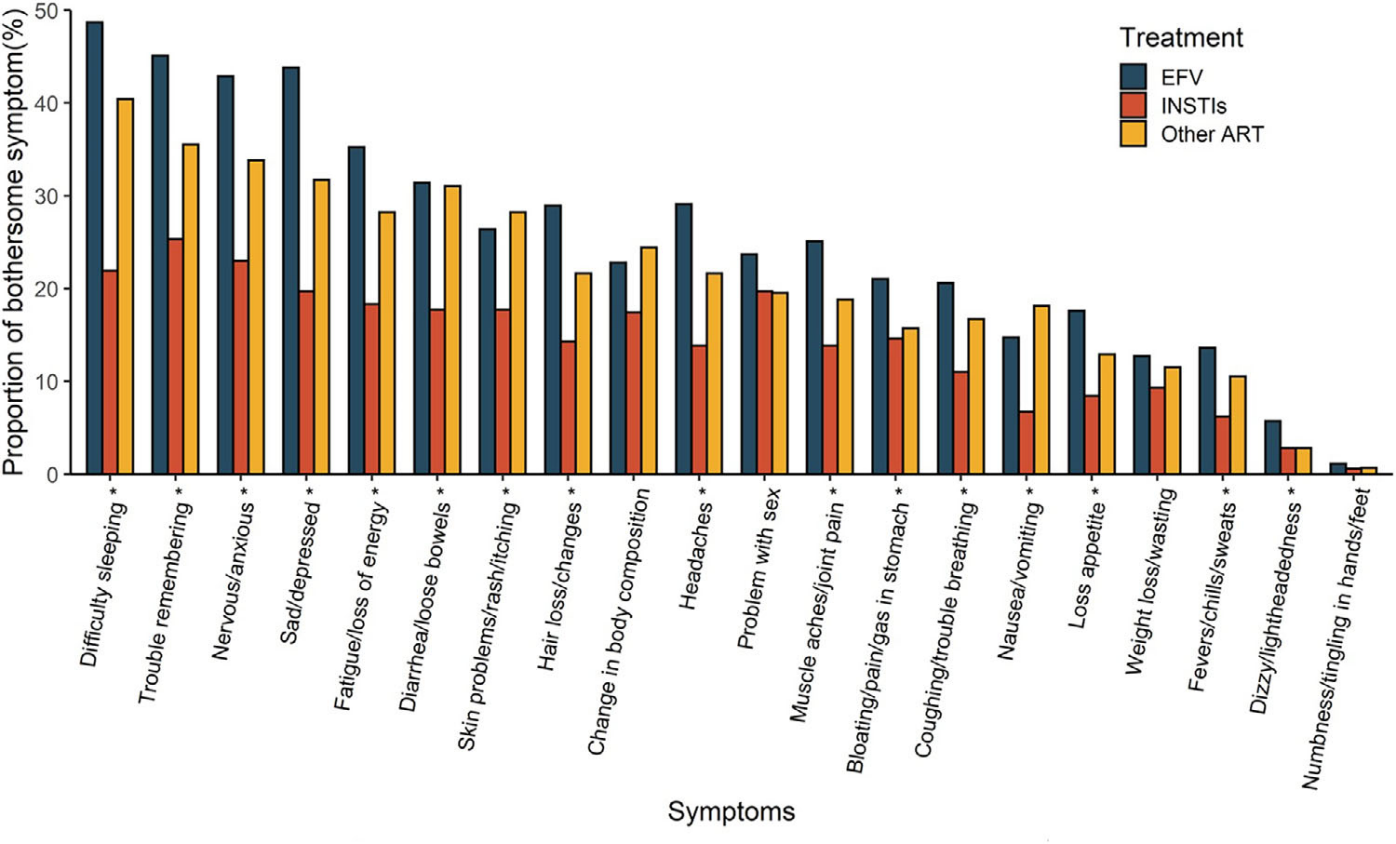
Patient‐reported bothersome symptoms (HIV‐symptom index, HIV‐SI) on different ART groups among PLWH in Kunming, China. *Note*: Asterix (*) indicates statistical significance at *p* < 0.05 level.

**P339: Figure 2:**
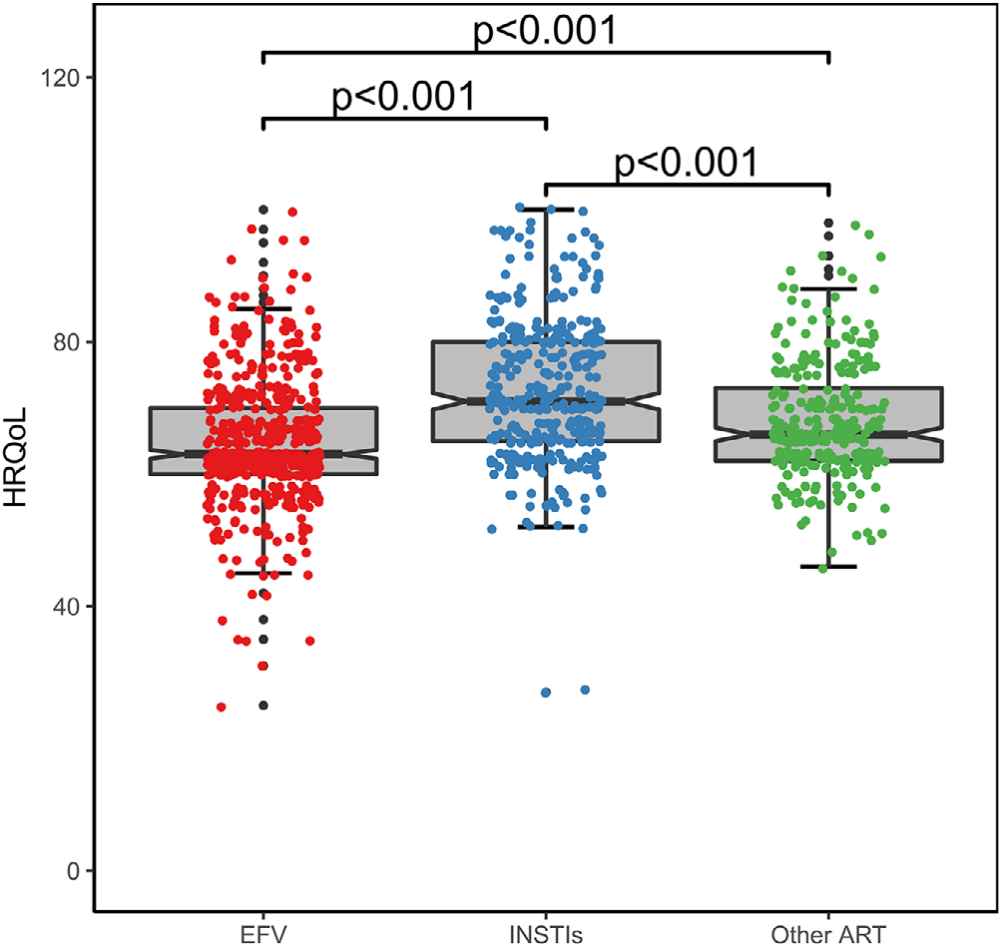
Boxplot of total HRQOL score across different ART regimen groups among PLWH in Kunming, China.

**Conclusions**: These findings suggest recommending INSTIs based regimens to enhance PROMs among PLWH and improve the HRQOL.

### P340 Virological failure and patterns of resistance‐associated mutations in previously untreated HIV‐positive participants in the RESINA cohort

Smaranda Gliga ^1^, Michael Böhm^2^, Nadine Lübke^3^, Alexander Killer^1^, Falk Hüttig^1^, Claudia Müller^2^, Eva Heger^2^, Joachim Büch^2^, Gerd Fätkenheuer^4^, Clara Lehmann^4^, Mark Oette^5^, Martin Hower^6^, Heribert Knechten^7^, Niels Schübel^8^, Stefan Esser^9^, Stephan Schneeweiß^10^, Nazifa Qurishi^11^, Katja Römer^11^, Rolf Kaiser^2^, Tom Luedde^1^, Björn Jensen^1^

^1^Department of Gastroenterology, Hepatology and Infectious Diseases, Medical Faculty and University Hospital Düsseldorf, Heinrich Heine University, Düsseldorf, Germany. ^2^Institute of Virology, German Center for Infection Research (DZIF), partner site Bonn‐Cologne, Faculty of Medicine and University Hospital of Cologne, University of Cologne, Cologne, Germany. ^3^Institute of Virology, Medical Faculty and University Hospital Düsseldorf, Heinrich Heine University, Düsseldorf, Germany. ^4^Division of Infectious Diseases, Department I of Internal Medicine, German Center for Infection Research (DZIF), partner site Bonn‐Cologne, Medical Faculty and University Hospital Cologne, University of Cologne, Cologne, Germany. ^5^Internal Medicine, Gastroenterology and Infectious Diseases, Krankenhaus der Augustinerinnen, Cologne, Germany. ^6^Pneumology, Infectious Diseases and Intensive Care, Klinikum Dortmund gGmbH, Hospital of University Witten / Herdecke, Dortmund, Germany. ^7^Private Practice Dr. Knechten, Aachen, Germany. ^8^Infectious Diseases, Klinikum Osnabrück, Osnabrück, Germany. ^9^Department of Dermatology and Venerology, Faculty of Medicine and University Hospital of Essen, University of Duisburg‐Essen, Essen, Germany. ^10^Private Practice Hohenstaufenring, Cologne, Germany. ^11^Private Practice Gotenring, Cologne, Germany

**Background**: Despite improvement of antiretroviral therapy (ART), virological failure (VF) remains a concern among people living with HIV. This study aimed to determine the frequencies and risk factors of VF and characterise the patterns of resistance mutations.

**Material and methods**: RESINA is a multicentre prospective cohort study examining the epidemiology of transmitted HIV drug resistance in North Rhine‐Westphalia, Germany. We performed a descriptive analysis of study participants with VF, defined as HIV‐RNA above 200 copies/ml after an episode of viral suppression. Frequency of VF was stratified by year of HIV diagnosis (1: 2001–2007; 2: 2008–2013; 3: ≥2014).

**Results**: From 2001 to 2022, 4983 participants were enrolled, of whom 103 (2.06%) (38 women, 65 men) experienced a VF (59/1368 [4.31%], 40/1835 [2.18%] and 4/1780 [0.22%] in groups 1–3 respectively). Their average age was 36 years. The most common transmission routes were heterosexual contacts (*n* = 49, 47.6%) and MSM (*n* = 28, 28.2%). The mean CD4 cell count and baseline HIV‐1 RNA of participants with VF were 70/µl and 76,708 copies/ml, respectively. At ART initiation, participants received: 2NRTI+PI (*n* = 31, 30.1%), 2NRTI+NNRTI (*n* = 30, 29.1%), 2NRTI+PI/r (*n* = 26, 25.2%) and 2NRTI+INI (*n* = 5, 4.9%). Baseline ART regimens included: NRTIs (45 FTC/TDF, 28 3TC/AZT), NNRTI (19 NVP, 13 EFV), PI (31 LPV, 12 DRV) and INI (3 DTG, 1 RAL, 1 EVG/c). At the time of VF, 52 (50.5%) participants were on their first ART‐regimen, while 29 (28.2%) and 14 (13.6%) were on their second and third ART‐regimen. Non‐compliance was given as the reason for VF in 47 (45.6%) participants. Of these 103 participants, 29 (28.2%) developed resistance‐associated mutations, 21 of whom against ≥2 drug classes. Resistance‐associated mutations occurred most frequently for NNRTI (n = 22) or NRTI classes (*n* = 20), while mutations against PIs and INIs were observed in four and five participants each (Table 1). Twenty‐two people did not achieve subsequent viral suppression until the end of the follow‐up period.

**P340: Table 1:** Major resistance mutations in participants experiencing virological failure

Patient ID	NRTI	NNRTI	PI	INI
48	M41L, M184V, T215YC	K101EK, K103N, Y188HY	V82A	
697			V82A	
10235	M184V	P236LP		H51HY, E92EQ^a^
10418	M184I	Y181C		
10783		K101EK		
13104	M184V	Y181C		
10952		K103NK		
964	M184V	K101E, V106I, H190S		
1022	M184VM	K103N		
1336				N155H^a^
1340	T69NT, K70EK	Y181CY	N88NS	
1362	K70R, M184V, T215F, K219EK	K103NK		
1383	M184IMV			
1576	M184V, T215F			
1595	M184MV			
1602	M184V, K219Q, K70R	V106IMV		
1701				N155H^a^
2175		K103N		
2244				Y143CPRS^a^
2268	M41L, M184V	K103N	V82A	
2767	M184V, L74I	Y181I, G190EG		
4400	M184IMV	Y181CY		
11499	M184MV	Y181CY		
11744	M184IMV			
11912	M184MV	K101EK, Y181C		
12619	M184IM			
12652	M184V	K101E		
13719	A62VA, M184IM	K101EK		
18613	M184V	V179D	R263K, G118R^a^	

^a^ 10235 also failed under regimens containing RAL and EVG/c; 1336, 1701, 2244 also failed under regimens containing RAL; 18613 failed under DTG.

**Conclusions**: The frequency of VF decreased over time. Better tolerability and a high genetic barrier to resistance second‐generation PIs and INIs are crucial for sustained viral suppression. Lack of treatment adherence remains one of the main causes of VF.

### P341 Online education yields significant gains in physicians' knowledge of the physical implications of stigma in people living with HIV

Shanthi Voorn ^1^, Alessia Piazza^1^, Gill Adair^1^, Chloe Orkin^2^, Maile Young Karris^3^

^1^Global Education, Medscape LLC, London, UK. ^2^Barts Health NHS Trust, Queen Mary University of London, London, UK. ^3^Department of Medicine, University of California San Diego, San Diego, CA, USA

**Background**: ​Stigma in people living with HIV impacts societal practices and experiences, contributes to ongoing health disparities and healthcare outcomes, and can lead to concealment, social exclusion and isolation. Chronic stress can have far‐reaching outcomes, including an increased risk of accumulating comorbidities and demonstrate reduced life expectancy. Here, we assessed if online independent medical education could increase HIV and infectious disease (ID) physician's knowledge and confidence regarding the impact of stigma‐induced stress. We developed an online Continuing Medical Education (CME) activity titled: “The Physical Implications of Stigma in People Living with HIV”. The goal was for learners to increase their knowledge on the impact of stigma, and its interventions.

**Methods**: ID/HIV specialists participated in an online CME activity (https://www.medscape.org/viewarticle/1000379) consisting of a 30‐minute discussion with two experts and accompanying slides. Educational effect was assessed using a four‐question repeated pairs, pre‐/post‐assessment. A paired samples *t*‐test was conducted for significance testing on overall average number of correct responses and for confidence rating, and a McNemar's test was conducted at the question level (5% significance level). Cohen's d for paired samples estimated the effect size of the education (<0.20 modest, 0.20–0.49 small, 0.59–0.79 moderate, ≥0.80 large). The CME activity launched on 3/13/2024, and the data were collected through 5/24/2024.

**Results**: A total of 478 ID/HIV specialists participated in the activity, of whom 34 completed the pre‐ and post‐activity questions. Overall, 76% of ID/HIV specialists improved their knowledge on the physical implications of stigma (*p* < 0.01) indicating a considerable effect of the education (Cohen's *d* = 0.55). The average percentage of correct responses rose from 64% to 76% for ID/HIV specialists pre‐activity to post‐activity. Thirty‐two percent of ID/HIV specialists had a measurable improvement in confidence in their ability to communicate the impact of stigma on health, and its interventions.

**Conclusions**: This online CME activity significantly improved ID/HIV specialists' knowledge about the impact of stress and stigma on people living with HIV and boosted physician's confidence in discussing these issues. However, 44% of physicians still provided incorrect answers post‐education, indicating a need for further educational efforts to better equip physicians in caring for HIV patients.

### P342 Identification of quality‐of‐life alterations among people living with HIV in Romania: ROCTAVE self‐administered questionnaire

Oana Sandulescu^1^, Victor Daniel Miron ^2^, Irina Magdalena Dumitru^3^, Manuela Arbune^4^, Anca Streinu‐Cercel^1^

^1^Infectious Diseases, Carol Davila University of Medicine and Pharmacy, Bucharest, Romania. ^2^Pediatrics and Infectious Diseases, Carol Davila University of Medicine and Pharmacy, Bucharest, Romania. ^3^Infectious Diseases, Faculty of Medicine, Ovidius University, Constanta, Romania. ^4^Infectious Diseases, Faculty of Medicine, Dunarea de Jos University, Galati, Romania

**Introduction**: Despite the advances in antiretroviral therapy, people living with HIV (PLWH) have many quality of life problems. Thus, knowledge of the difficulties they face is a necessity in order to be able to provide care tailored to their needs. Our aim was to detect alterations in the quality of life of PLWH in Romania using the OCTAVE questionnaire, adapted for our country (ROCTAVE).

**Methods**: We present preliminary data from a study conducted among PLWH using a previously validated self‐administered questionnaire (OCTAVE) [1] in which we assessed eight dimensions: mental health, physical health, sexuality, emotional well‐being, other non‐HIV‐related health issues, social and professional life, antiretroviral therapy. sleep quality. Data collection was done during the hospital visit, between July 2023 and February 2024, through the participation of three HIV care centres from different regions of Romania.

**Results**: A total of 426 questionnaires were analysed. The mean age of respondents was 38.9 years (min. 18, max. 70), and male sex was slightly predominant (56.1%). Length of time since diagnosis ranged from 1 to 33 years, with a mean of 15.5 years. The three main dimensions in which the quality of life of people with HIV was altered were social and professional life (31.3%), sexuality (29.0%) and sleep (25.5%) (Figure 1). The highest level of satisfaction was regarding antiretroviral therapy (94.1%) and aspects of non‐HIV‐related health care (87.4%). By gender, women reported significantly lower levels of satisfaction compared to men in sexuality (64.5% vs. 73.8%, OR 1.6, *p* = 0.037), mental health (78.4% vs. 87.0%, OR 1.8, *p* = 0.018) and emotional well‐being (73.3% vs. 81.9%, OR 1.1, *p* = 0.033).

**P342: Figure 1:**
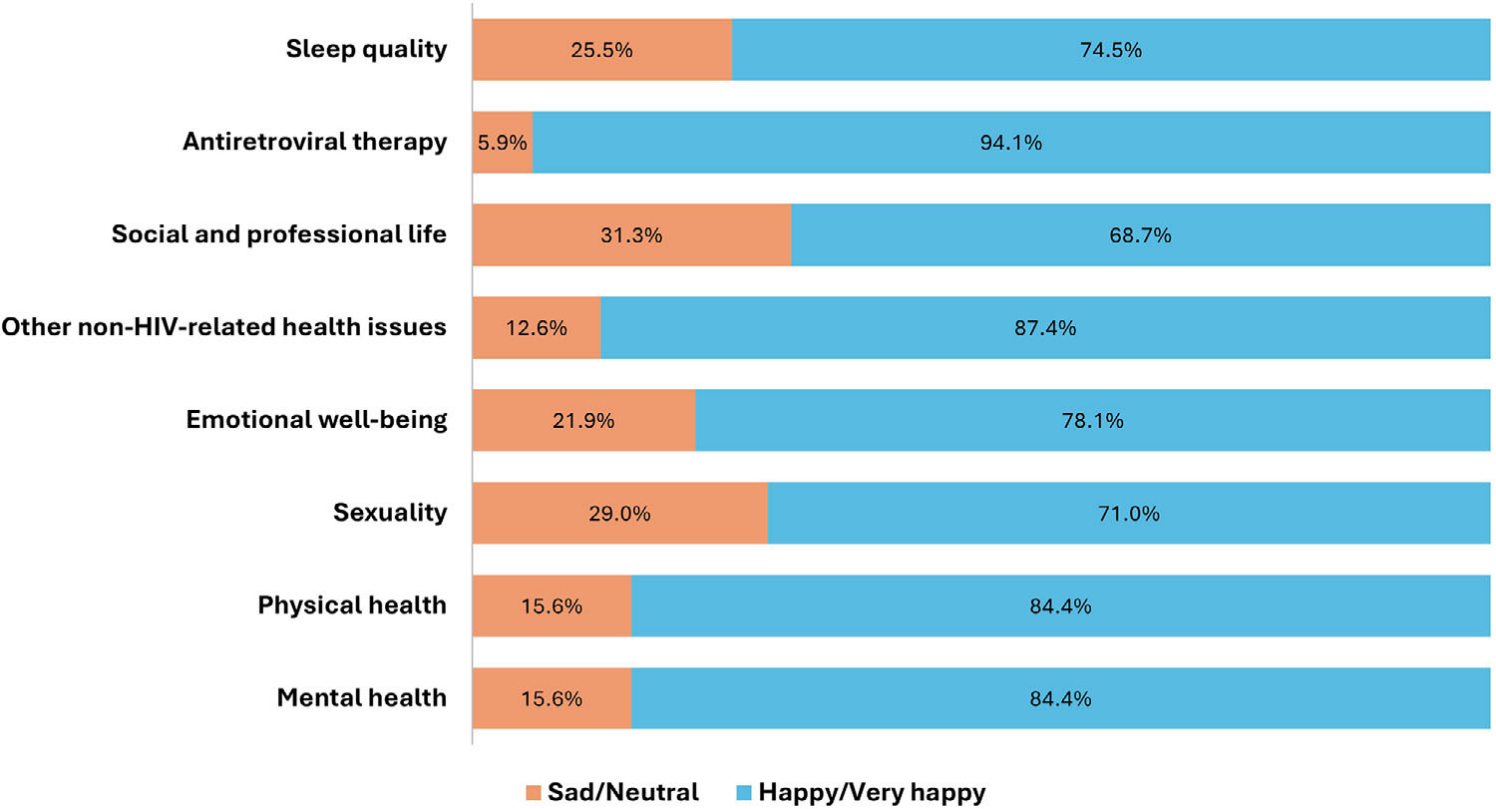
The level of satisfaction or affectation according to dimensions.

**Conclusions**: We have presented a preliminary analysis of the issues faced by PLWH in Romania by analysing the eight dimensions. Alteration was more frequently related to social and professional life alongside sexuality. Our data shows a reality that clinicians need to be aware of in order to increase the quality of life of PLWH.

**Reference**

1. Slama L, Bastides F, Robineau O, Leporrier J, Barriere G, Estrabaud E, et al. Identification of quality‐of‐life alterations in PLWHIV in routine practice: benefit of the OCTAVE self‐administered questionnaire [Abstract P188]. J Int AIDS Soc. 2022;25 (Suppl. 6):e26009.

### P343 Anti‐retroviral regimen change and its predictors among people living with HIV in Hawassa University Comprehensive Specialized Hospital, southern Ethiopia, retrospective cohort study

Endrias Makos Woldesemayat ^1^, Wonago Petros^2^, Jaime Vera^3^

^1^School of Public Health, Hawassa University, Hawassa, Ethiopia. ^2^Sidama Region Public Health Institute, Sidama Region Health Department, Hawassa, Ethiopia. ^3^Brighton and Sussex Medical School, Sussex University, Brighton, UK

**Background**: Anti‐retroviral regimen change is among the major challenges for the success of treatment among people living with human immunodeficiency virus (PLHIV), affecting its sustainability and outcome among ART patients. This study aimed to determine the incidence and its predictors of anti‐retroviral regimen changes among PLHIV at Hawassa University Comprehensive Specialized Hospital (HUCSH).

**Methods**: An institutional‐based retrospective cohort study was conducted among PLHIV who started ART from 1 January 2006 to 31 December 2023. Regimen change was defined as changing the ART regimen due to adverse events. Records were reviewed and a standard data extraction form was used to collect data on a Kobo Toolkit. A Kaplan‐Meier plot with a log‐rank test at *p* < 0.05 was considered to estimate the median follow‐up time and compare the survival difference between the covariates. Bi‐variable Cox regression analysis was done and variables with *p* ≤ 0.25 were entered into the multivariable Cox regression model. Predictors with *p*‐values <0.05 reported with their adjusted hazard ratios and the 95% confidence intervals.

**Result**: A total of 3856 patients were followed for 39,350 person‐years of observation with the median follow‐up period of 11.2 years. The incidence rate of anti‐retroviral regimen change was 12.1 (95% CI 11.5–12.7) per 100 person‐years. Female gender (adjusted hazard ratio (AHR) 2.9, 95% CI 2.6–3.4), occurrence of TB (AHR 4.6, 95% CI 2.9–7.4), occurrence of side effects on first regimen (AHR 3.4, 95% CI 2.9–3.8), baseline CD4 count <100 cells/mm^3^ (AHR 1.3, 95% CI 1.1–1.6) and availability of social support (AHR 0.47, 95% CI 0.38–0.58) were predictors of regimen change among the studied PLHIV (Table 1).

**P343: Table 1:** Multi‐variable analysis of predictors among PLHIV at Hawassa University Comprehensive Specialized Hospital from January 2006‐December 2023

		Survival status	Survival status			
Characteristics	Category	Regimen changed	Person‐years	CHR (95% CI)	AHR (95% CI)	*p*‐value
TB status test	Positive	991	9632.75	4.7 (2.9–7.4)	4.6 (2.9–7.4)	0.001^*^
	Negative	427	28988.8	0.56 (0.35–0.9)	0.62 (0.4–1.0)	0.06
	Not done	18	729.25	1	1	Ref.
Side effect occurred	Yes	321	2660.9	4.7 (4.1–5.3)	3.4 (2.9–3.8)	0.001^*^
	No	1115	36,689.9	1	1	Ref.
Sex	Male	335	20,292.8	1	1	Ref.
	Female	1101	19,058	4.9 (4.3–5.6)	2.9 (2.6–3.4)	0.001^*^
Social support availability	Yes	123	10,682.5	0.21 (0.18–0.26)	0.47 (0.38–0.58)	0.001^*^
	No	1313	28,668.3	1	1	Ref.
Baseline CD4 (cells/mm^3^)	<100	397	8838.2	1.4 (1.3–1.7)	1.3 (1.1–1.6)	0.001^*^
	100‐199	401	9759.8	1.3 (1.2–1.6)	1.1 (0.9–1.3)	0.20
	200‐349	381	12,342.4	0.98 (0.8–1.2)	1.0 (0.8–1.2)	0.56
	350	257	8404.6	1	1	Ref.

* Significant at *p* < 0.05.

**Conclusions**: The incidence of regimen change in this study was comparable to other findings. A number of variables predicted regimen change. Early identification and proper management of these predictors could minimise the problem.

### P344 The impact of cART on the intracellular antioxidant capacity in peripheral blood mononuclear cells of people living with HIV

Radoslava Emilova ^1^, Yana Todorova^1^, Reneta Dimitrova^2^, Victor Manolov^3^, Nina Yancheva^4^, Milena Aleksova^1^, Damian Vangelov^1^, Maria Nikolova^1^

^1^Department of Immunology, National Center of Infectious and Parasitic Diseases (NCIPD), Sofia, Bulgaria. ^2^National Reference Confirmatory Laboratory of HIV, National Center of Infectious and Parasitic Diseases (NCIPD), Sofia, Bulgaria. ^3^Clinical Laboratory, University Hospital Tsaritsa Yoanna—ISUL, Sofia, Bulgaria. ^4^Department of Acquired Deficiencies, Specialized Hospital for Active Treatment of Infectious and Parasitic Diseases, Sofia, Bulgaria

**Background**: A number of recent data reveal that continuous oxidative stress increases the rate of comorbidities in people living with HIV regardless of successful cART and sustained HIV viral suppression. Our aim was to evaluate the antioxidant capacity in peripheral blood mononuclear cells (PBMCs) from people living with HIV on cART using the glutathione/glutathione disulfide (GSH/GSSG) ratio, intracellular catalase and superoxide dismutase (SOD) levels.

**Material and methods**: Peripheral blood samples were obtained from HIV‐positive individuals on long‐term cART, with persistently suppressed HIV viral load and recovered CD4 absolute count (AC), (A, *n* = 29), untreated HIV+ individuals (B, *n* = 11) and gender‐ and age‐matched HIV‐ volunteers (C, *n* = 28) after informed consent. The absolute count of CD4+ and CD8+ T cells were determined by flow cytometry. PBMCs were isolated by density gradient separation, and cell lysates were prepared at 1x10^6 PBMCs in 200 µl PBS by sonication. Concentrations of GSH and GSSG [nmol/mg], SOD and catalase [U/L] were determined by spectrophotometry.

**Results**: In our hands, cART seemed to restore GSH/GSSG ratio, which was significantly decreased only in group B (mean 2.8 vs. 4.9 and 5.5 for A and C, *p* < 0.05). Intracellular GSH was lower in group B as compared to A and C though without statistical significance (12.4 vs. 16.3 and 17.3, *p* > 0.05). Noteworthy, GSSG levels remained significantly higher in both HIV+ groups (8.45 and 16.4 vs. 3.54 for controls, *p* < 0.01), in direct correlation with CD8AC (R = 0.40, *p* < 0.05). The mean intracellular catalase and SOD concentrations were lower in controls as compared to groups A and B (36.5 vs. 41.0 and 40.5, *p* > 0.001 and 18.9 vs. 32.5 and 47.8, *p* > 0.01, respectively).

**Conclusion**: In spite of restored GSH/GSSG balance, the persisting high GSSG levels in the settings of long‐term cART indicate possible disturbances in glutathione reductase activity in PBMCs of HIV‐positive individuals. Affected cellular redox status, in line with increased catalase and SOD levels may signal immune activation in the settings of undetectable HIV viral load and changes in share of lymphocyte subpopulations. These results reevaluate oxidative stress in the settings of long‐term cART monitoring.

### P345 Unmasked immune reconstitution syndrome diagnoses among people living with HIV admitted in a low‐resource hospital, Homabay, Kenya 2021‐2022

Edward Corneleous Odhiambo Okal ^1^, Thaddeus Owiti^1^, James Wagude^2^, Gordon Okomo^1^

^1^Health, Ministry Of Health, Homabay, Kenya. ^2^Health, Ministry Of Health, Siaya, Kenya

**Background**: Immune reconstitution inflammatory syndrome (IRIS) in people living with HIV (PLHIVs) is an “unmasking” or paradoxical worsening of a pre‐existing infection following initiating antiretroviral therapy (ART) [1]. Studies show that up to 25–30% starting ART get IRIS and a mortality of 4.9 deaths per 100 persons [2]. Most IRIS cases occur in the first 6 months of treatment of HIV/AIDS clients. Diagnoses of IRIS depend on criteria that consider ART duration, CD4 count, viral load (VL), and the presence of a new infection following ART initiation. Most facilities lack CD4 testing and do not do baseline VL making diagnoses a challenge. We assessed unmasked IRIS diagnoses amongst patients admitted to the Homabay County Teaching and Referral Hospital (HBCTRH) medical wards.

**Methods**: We retrospectively reviewed records of PLHIVs on ART admitted in the medical wards at HBCTRH, Homabay County in 2021 and 2022. All patients who had been on ART for less than 6 months were included in the study. Demographic and clinical variables were abstracted from the Ministry of Health register into a database with enforced data quality and consistency checks. We assessed IRIS diagnoses using the general IRIS cases definition proposed by French et al, 2004 [3] and low CD4 (<100), which is a risk factor for IRIS. Case definition A included a recent ART initiation and identification of new AIDs defining disease while B included case definition A + CD4 <100

**Results**: We included all the 252 patients on ART <6 months admitted in the medical wards between 2021 and 2022. Median age 35 range 85 (range 13–98). Males accounted for 56% and those on ART <3 months were 77% (194). Mortality was at 17% (43). Median CD4 86 IQR (0–2106). IRIS diagnoses using case definition A was at 64.7% (163) while B was at 35.3% (89) with a mortality of 7.9% (11).

**Conclusion**: At least three out of every 10 PLHIV on ART less than 6 months admitted in the inpatient had unmasked IRIS. We recommend the use of a more simplified IRIS clinical guide to help in case identification.

**References**

1. Murdoch DM, Venter WD, Van Rie A, Feldman C. Immune reconstitution inflammatory syndrome (IRIS): review of common infectious manifestations and treatment options. AIDS Res Ther. 2007;4:9.

2. Thapa S, Shrestha U. Immune Reconstitution Inflammatory Syndrome. [Updated 2023 Jan 2]. In: StatPearls [Internet]. 2024 [cited 2024 Jul 26]. Available from: https://www.ncbi.nlm.nih.gov/books/NBK567803/.

3. French MA, Price P, Stone SF. Immune restoration disease after antiretroviral therapy. AIDS. 2004;18:1615‐27.

## People living with HIV and COVID‐19: Outcomes

### P346 Proteic and mRNA XBB.1.5 SARS‐CoV‐2 vaccines elicit a close neutralising response against JN.1 in PWH

Alessandra Vergori^1^, Giulia Matusali^2^, Eleonora Cimini^3^, Alessandro Cozzi‐Lepri^4^, Federico Cecilia^5^, Davide Mariotti^2^, Francesca Colavita^2^, Simona Gili^3^, Flavia Cristofanelli^3^, Marisa Fusto^1^, Valentina Mazzotta^1^, Jessica Paulicelli^1^, Roberta Gagliardini^1^, Enrico Girardi^6^, Fabrizio Maggi^2^, Andrea Antinori ^1^

^1^Viral Immunodeficiency Unit, National Institute for Infectious Diseases L. Spallanzani, IRCCS, Rome, Italy. ^2^Virology Unit, National Institute for Infectious Diseases L. Spallanzani, IRCCS, Rome, Italy. ^3^Laboratory of Cellular Immunology and Pharmacology, National Institute for Infectious Diseases L. Spallanzani, IRCCS, Rome, Italy. ^4^Clinical Research, Epidemiology, Modelling and Evaluation (CREME) Centre, Institute of Global Health, University College London, London, UK. ^5^Viral Immunodeficiency Unit and Infectious Disease Unit, National Institute for Infectious Diseases L. Spallanzani, IRCCS and Department of Systems Medicine, University of Rome PTV, Rome, Italy. ^6^Scientific Direction, National Institute for Infectious Diseases L. Spallanzani, IRCCS, Rome, Italy

**Background**: A new Novavax‐CoV2373 (XBBPro) monovalent protein‐based and an mRNA vaccine (XBBmRNA) (Pfizer‐BioNTech raxtozinameran) specifically targeting the subvariant Omicron XBB.1.5 were available during the most recent vaccination campaign for frail individuals including persons with HIV (PWH). The level of immunogenicity achievable in humans with immune dysregulation by these vaccines is currently unknown.

**Material and methods**: We included PWH on ART enrolled in the HIV‐VAC cohort who received a booster vaccination with XBBPro or XBBmRNA vaccines after ≥4 doses of mRNA vaccines. Samples were collected before the booster (day 0, T0) and 1 month after vaccination (T1). Neutralising antibodies (nAbs) titers were measured by a micro‐neutralisation assay based on live SARS‐CoV‐2 virus against D614G, XBB.1.16 (XBB.1.5 like umbrella variant), and JN.1; interferon gamma (IFN‐γ) release from T cell‐specific response to both vaccines was analysed by an enzyme‐linked immunosorbent assay (ELISA) test after Spike‐peptides stimulation. Unadjusted T0–T1 geometric mean nAbs titers (GMTs) and mean IFN‐γ values were compared using paired and unpaired t‐tests. A counterfactual linear marginal model was used to compare nAbs titers after weighting for potential confounders.

**Results**: We included 51 PWH (*n* = 26 received the XBBmRNA and *n* = 25 the XBBPro), median age was 57 years (IQR 51–65), the median count of CD4 at T0 was 652/mmc (503–935), CD4 nadir was 226/mmc (95–340), 65%, 25% and 10% received five, four and six vaccine doses, respectively (Table 1). Regardless of the vaccine used, we observed a significant increase of nAbs titers from T0 to T1 against all the subvariants but no evidence for a change in IFN‐γ release (Figure 1). After controlling for confounders, there was no evidence for a difference in the T0–T1 change in nAbs titers by the type of vaccination strategy except for nAbs against D614G, which were lower for XBBPro than for XBBmRNA [average treatment effect (ATE) ‐1.30 log_2_ (‐2.27, ‐0.34); *p* = 0.008].

**P346: Table 1:** Potential average change at post vaccine dose and ATE from fitting a linear regression model (log_2_ scale)

Vaccine type				
XBBPro vs XBBmRNA	Mean (log2) in XBBPro (95% CI)	Mean (log2) in XBBmRNA (95% CI)	ATEa (95% CI)	*p*‐value
		**nAbs against D614G**		
IPWs	1.14 (0.70–1.58)	2.72 (1.73–3.71)	−1.58 (−2.66, −0.49)	0.004
Double robust	1.25 (0.42–2.08)	2.56 (1.08–4.03)	−1.30 (−2.27, −0.34)	0.008
Regression adjustment	1.30 (0.79–1.81)	2.49 (1.49–3.50)	−1.19 (−2.32, −0.06)	0.040
		**nAbs against XBB.1.16**		
IPWs	2.94 (2.27–3.61)	3.43 (2.42–4.44)	−0.49 (−1.71, 0.73)	0.433
Double robust	3.21 (1.68–4.75)	3.24 (1.47–5.01)	−0.03 (−1.22, 1.17)	0.965
		**nAbs against JN.1**		
IPWs	2.16 (1.67–2.65)	2.52 (2.02–3.03)	−0.37 (−1.06, 0.33)	0.301
Double robust	2.24 (1.52–2.96)	2.43 (0.98–3.88)	−0.19 (−0.90, 0.52)	0.599
		**IFN‐γ**		
IPWs	0.49 (0.01–0.96)	0.22 (−0.25, 0.70)	0.26 (−0.42, 0.95)	0.451
Double robust	0.57 (−0.21, 1.35)	0.30 (−1.10, 1.71)	0.27 (−0.66, 1.19	0.574
Regression adjustment	0.58 (0.21–0.96)	0.36 (−0.86, 1.58)	0.22 (−1.07, 1.51)	0.735

Abbreviations: IPWs, inverse probability weighting; nABs, neutralising antibodies; XBBmRNA, Pfizer‐BioNTech raxtozinameran; XBBPro, Novavax‐CoV2373 monovalent XBB.1.5.

^a^Average treatment effect; weighted for age, current and nadir CD4 count, year of booster, year of HIV diagnosis, days from previous vaccine dose, number of doses previously received, anti‐N IgG positivity at T0.

**P346: Figure 1:**
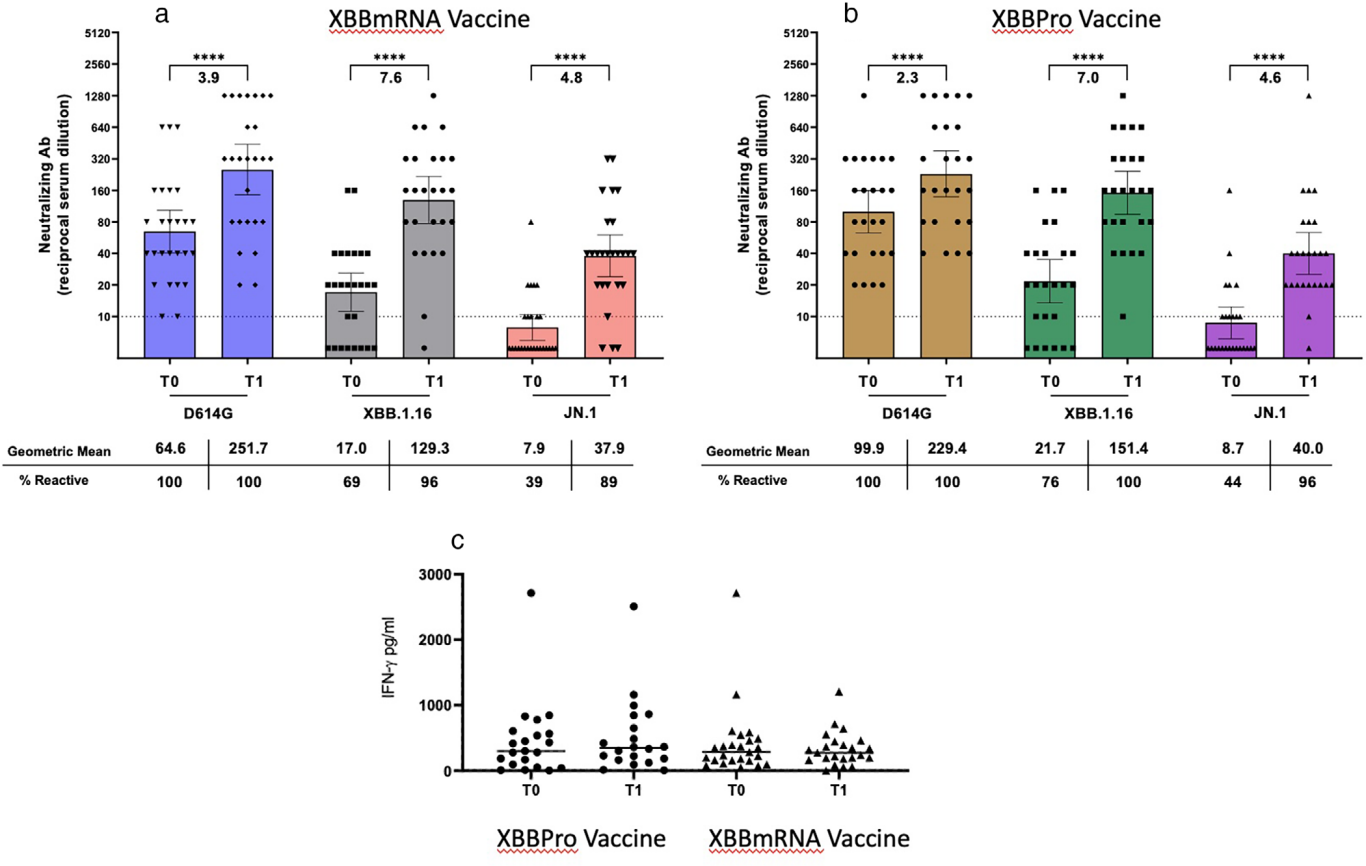
Comparison between T0 and T1 of geometric mean neutralising antibodies titers of XBBmRNA vaccine (a), XBBPro (b) and of mean values of IFN‐γ (c).

**Conclusions**: In our cohort of PWH, XBBPro and XBBmRNA elicited similar neutralising and T cell‐specific responses, while XBBmRNA induced higher nAbs titers against D614G than XBBPro. Our data also confirms that newly developed vaccines are unlikely to further modify the level of T‐cell immunity achieved with previous vaccination.

### P347 No changing incidence of type 2 diabetes in PWH before and after the first wave of the COVID‐19 pandemic

Alessandra Vergori^1^, Alessandro Tavelli^2^, Sara De Benedittis^2^, Nicola Squillace^3^, Lucia Taramasso^4^, Roberto Rossotti^5^, Eugenia Quiros Roldan^6^, Andrea Santoro^7^, Andrea Calcagno^8^, Simone Lanini^9^, Giuseppe Lapadula^10^, Giovanni Guaraldi^11^, Antonella Castagna^12^, Andrea Antinori^13^, Alessandro Cozzi‐Lepri ^14^

^1^Clinical and Research Infectious Disease Department, Viral Immunodeficiency Unit, National Institute for Infectious Diseases L.Spallanzani, IRCCS, Rome, Italy. ^2^ICONA Foundation, Milan, Italy. ^3^Infectious Diseases Unit, IRCCS San Gerardo dei Tintori, Monza, Italy. ^4^Infectious Disease Clinic, IRCCS Ospedale Policlinico San Martino, Genova, Italy. ^5^Department of Infectious Diseases, ASST Grande Ospedale Metropolitano Niguarda, Milan, Italy. ^6^Department of Clinical and Experimental Sciences, Unit of Infectious and Tropical Diseases, University of Brescia and ASST Spedali Civili di Brescia, Brescia, Italy. ^7^Clinic of Infectious Diseases, ASST Santi Paolo e Carlo, Department of Health Sciences, University of Milan, Milan, Italy. ^8^Unit of Infectious Diseases, Department of Medical Sciences, University of Turin, Amedeo di Savoia Hospital, Torino, Italy. ^9^Department of Medicine, Infectious Diseases Clinic, ASUFC, University of Udine, Udine, Italy. ^10^Department of Infectious Diseases, Infectious Diseases Unit, IRCCS San Gerardo dei Tintori, University of Milano‐Bicocca, Milan, Italy. ^11^Department of Surgical, Medical, Dental and Morphological Sciences, Department of Infectious Diseases, University of Modena, Azienda Ospedaliero‐Universitaria Policlinico of Modena, Modena, Italy. ^12^Infectious Diseases Unit, IRCCS San Raffaele Scientific Institute, Vita Salute San Raffaele University, Milan, Italy. ^13^Clinical and Research Infectious Diseases Department, Viral Immunodeficiency Unit, National Institute for Infectious Diseases L.Spallanzani, IRCCS, Rome, Italy. ^14^Centre for Clinical Research, Epidemiology, Modelling and Evaluation, Institute for Global Health, London, UK

**Background**: Previous studies suggest a rise in type‐2 diabetes (T2D) diagnoses around or after the time of COVID‐19 diagnosis in the general population. Explanations for the elevated T2D incidence have been attributed to several factors, such as acute infection, stress, or direct pancreatic beta‐cell damage. mRNA vaccination was associated with attenuated risk. Whether this was also seen in people with HIV (PWH), who are predisposed to diabetes, remains to be established.

**Material and methods**: PWH enrolled in the ICONA Foundation Study cohort under active follow‐up and free from T2D in January 2016 were included. T2D was defined as a reported and/or new prescription of antidiabetic medications and/or hyperglycaemia (fast glucose: >2 values ≥126 mg/dl or one value ≥200 mg/dl) or clinical diagnosis. Participants were followed until they were diagnosed with T2D or their last clinical visit. Incidence of T2D has been calculated per year over the period 2016–2023. Relative rates of T2D with 95% CI were calculated using a Poisson regression model after controlling for age, gender, and current use of TAF and INSTI. A nested case‐control study has also been conducted, matching case (T2D) and controls (T2D‐free) by age, gender, body mass index (BMI) and calendar year of enrolment. History of SARS CoV‐2 vaccination has been retrospectively collected in cases and controls. A conditional logistic regression was used to estimate the OR of T2D according to previous vaccination (≥2 doses vs. <2).

**Results**: Overall, 15,773 PWH were included, 47% enrolled before 2020: 22% female, median age 46 years (IQR 38–54), and 12% with AIDS. In January 2016, the median fast glucose was 87 mg/dl (80–95), and 86% were receiving a triple‐drug regimen (3DR) with a previous average duration of viral suppression of 32 months. The incidence of T2D remained stable over the study period (Figure 1). The case‐control study carried inconclusive results regarding the association between having completed a primary vaccination cycle and risk of T2D (Table 1).

**P347: Figure 1:**
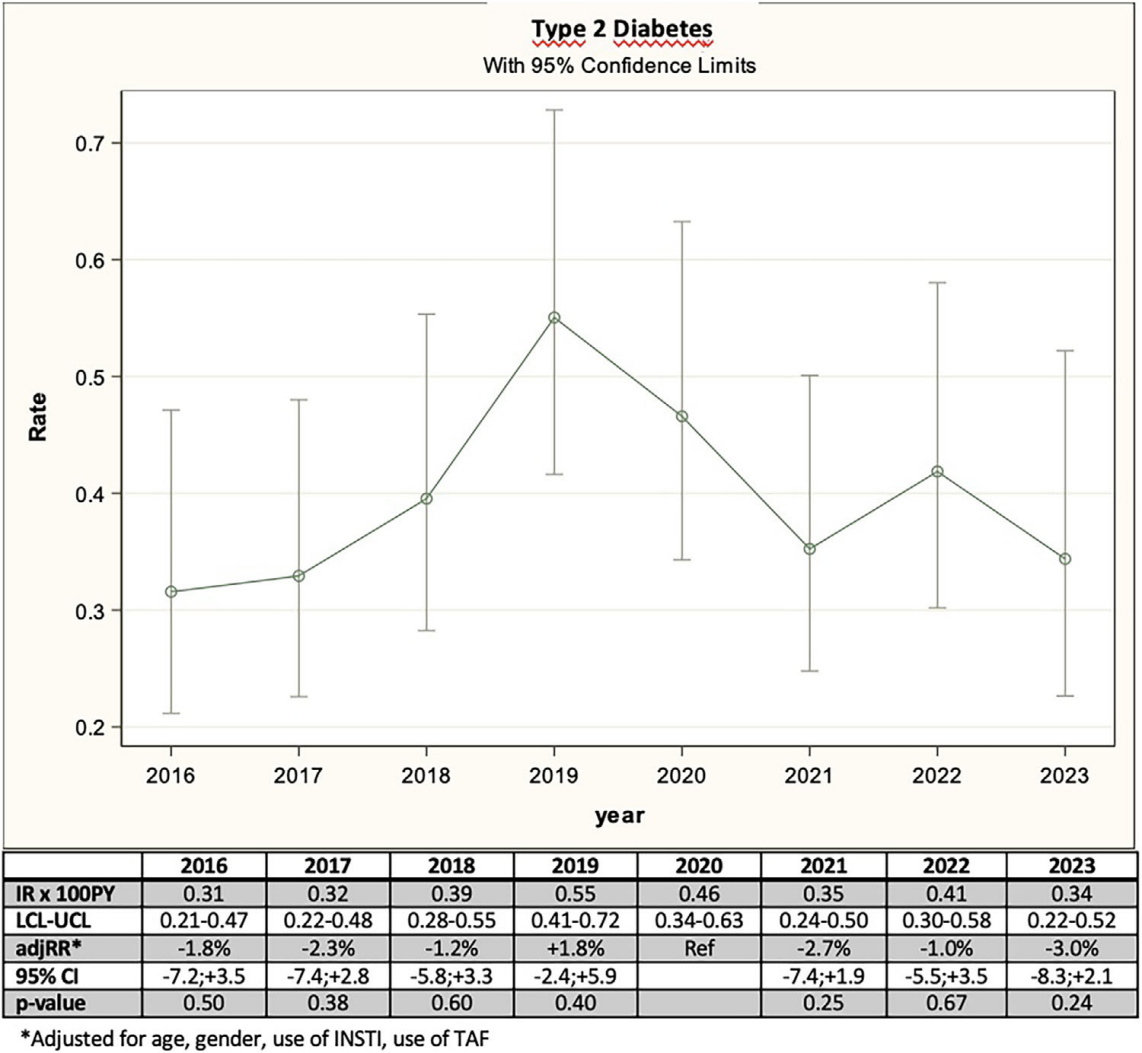
Rates of type 2 diabetes (T2D) x 100 person‐year follow up (PYFU) from 2016 to 2023.

**P347: Table 1:** ORs of T2D from fitting a conditional logistic regression

Exposure (mRNA vaccination)	Unadjusted^a^ OR (95% CI)	*p*‐value	Adjusted^b^ OR (95% CI)	*p*‐value
<2 doses	1		1	
≥2 doses	1.08 (0.42–2.79)	0.87	0.55 (0.14–2.19)	0.40

^a^ Matched for age, sex at birth and calendar year;

^b^ Further adjusted for geographical region, HCVAb status, ethnicity, BMI and CD4/CD8 ratio.

**Conclusions**: Our data provide poor evidence for and show no change in the incidence of T2D in PWH before and after the first wave of the COVID‐19 pandemic, regardless of vaccination.

### P348 “Don't turn that patient away, they must be seen.” A qualitative exploration of the views of London HIV clinical service providers on HIV care engagement challenges (the SHIELD study)

Rageshri Dhairyawan^1^, Shiraaz Sidat^2^, Rebecca Mbewe ^3^, Silvia Petretti^4^, Jane Anderson^5^, Vanessa Apea^6^, Sara Paparini^3^, Chloe Orkin^7^

^1^Sexual Health and HIV Medicine, Barts Health NHS Trust, London, UK. ^2^Doctorate Student, UCL, London, UK. ^3^Wolfson Institute for Population Health, Queen Mary University London, London, UK. ^4^CEO, Positively UK, London, UK. ^5^Centre for the Study of Sexual Health and HIV, Homerton Healthcare NHS Foundation Trust, London, UK. ^6^Consultant Physician in Sexual Health and HIV, Barts Health HNS Trust, London, UK. ^7^SHARE Collaborative, Queen Mary University London, London, UK

**Background**: Lifelong engagement in care with effective HIV therapy is essential to improve health outcomes for individuals and populations. This can be challenging for some people at different times in their lives. Supporting engagement is vital for individuals and for efforts to achieve global targets to end AIDS. During the COVID‐19 pandemic, people who had not been in regular care pro‐actively contacted their HIV clinic for treatment. The SHIELD study is an ongoing, mixed methods study aiming to understand shifts in re‐engagement (and dis‐engagement) during the pandemic to generate generalisable learnings to improve engagement.

**Methods**: We present workstream 1 findings derived from 11 semi‐structured qualitative interviews exploring staff perspectives (six doctors, two specialist nurses, two health advisors, one clinic manager) from 11 London‐based HIV clinics. We sampled staff based on their involvement in engagement tasks in the clinic. Interviews explored existing service‐level policies to support engagement in HIV clinical care; changes made during COVID‐19; staff's views on dis‐engagement and strategies to address it. Interviews were analysed thematically.

**Results**: Participants discussed interactions at person, service and system levels they regarded as protective or harmful to engagement. Person‐level: participants described how social and economic factors, treatment fatigue, competing priorities, stigma and shame, low self‐esteem and past trauma negatively affect engagement. Service‐level: protective values included accessibility, flexibility, non‐judgemental and personalised approaches, all considered central to building trust and long‐lasting patient‐centred relationships; processes to identify struggling patients; providing mental health, peer and non‐clinical support services. System‐level: participants described harms related to under‐prioritisation of engagement and heightened focus on testing, precarious funding for support services, and a lack of reliable data systems and data sharing across clinics.

**Conclusions**: Staff in HIV services advocated for the need to invest time and resources to identifying, supporting, and re‐engaging patients who might be facing challenges in making the most of available care.

### P349 SARS‐CoV‐2 vaccination and coronaviral disease 2019 in virologically suppressed people with HIV‐1 previously on NNRTI‐based antiretroviral regimen during the pandemic: The secondary longitudinal analysis of the SPRINT study, a multi‐centre, randomised, active‐controlled phase III trial

Hongzhou Lu^1^, Hui Wang^2^, Shenghua He^3^, Zhu Chen^4^, Yaokai Chen^5^, Min Wang^6^, Fujie Zhang^7^, Hao Wu^8^, Weiping Cai^9^, Ping Ma^10^, Qingxia Zhao^11^, Hongxia Wei^12^, Wan Wan^13^, Heliang Fu^13^, Hong Qin ^13^

^1^National Clinical Research Centre for Infectious Diseases, The Third People' s Hospital of Shenzhen, The Second Affiliated Hospital of Southern University of Science and Technology, Shenzhen, China. ^2^Department of Infectious Diseases, Shenzhen Third People's Hospital, Shenzhen, China. ^3^Department of Infectious Diseases, Public Health Clinical Medical Center of Chengdu, Chengdu, China. ^4^Drug Clinical Trial Institution, Public Health Clinical Medical Center of Chengdu, Chengdu, China. ^5^Division of Infectious Diseases, Chongqing Public Health Medical Center, Chongqing, China. ^6^Institute of HIV/AIDS, The First Hospital of Changsha, Changsha, China. ^7^Clinical and Research Center for Infectious Diseases, Beijing Ditan Hospital Capital Medical University, Beijing, China. ^8^Clinical and Research Center for Infectious Diseases, Beijing Youan Hospital, Capital Medical University, Beijing, China. ^9^Infectious Disease Center, Guangzhou Eighth People's Hospital, Guangzhou Medical University, Guangzhou, China. ^10^Department of Infectious Diseases, Tianjin Second People's Hospital, Tianjin, China. ^11^Department of Infectious Diseases, The Sixth People's Hospital of Zhengzhou, Zhengzhou, China. ^12^Department of Infectious Diseases, The Second Hospital of Nanjing, Nanjing, China. ^13^Clinical Research & Development, Jiangsu Aidea Pharmaceutical Co., Ltd, Yangzhou, China

**Background**: Pandemic coronaviral disease 2019 (COVID‐19) prevails between December 2019 and May 2023. People with HIV‐1 (PWH), including those virologically suppressed, are vaccinated against SARS‐CoV‐2, and inevitably afflicted with COVID‐19. We conducted the SPRINT study, a multi‐centre, randomized, active‐controlled phase III trial, between November 2021 and March 2023. This study compared efficacy and safety of switch to ainuovirine plus lamivudine and tenofovir DF (ANV/3TC/TDF) and that to cobicistat‐boosted elvitegravir plus emtricitabine and tenofovir alafenamide (E/C/F/TAF) in virologically suppressed PWH previously on NNRTI‐based antiretroviral (ARV) regimen. This secondary longitudinal analysis aimed to evaluate SARS‐CoV‐2 vaccination and COVID‐19 profile in virologically suppressed PWH during the pandemic.

**Methods**: Out of 923 PWH screened, 762 participants, aged 18–65 years, were enrolled. Eligible participants were randomised to ANV/3TC/TDF (N = 381) or E/C/F/TAF (N = 381) arm for 48 weeks. Participants were followed up at outpatients visits of baseline, and weeks 4, 12, 24, 36, and 48. Adverse events and concomitant medications, including medical events of special interest, and vaccination, were coded using MedDRA and WHODD, respectively.

**Results**: The SPRINT study population was at a median age of 33.0 years (Q1–Q3, 28.0–39.0 years), 97.1% being male, and with 98.3% HIV‐1 RNA below 50 copies/ml. At baseline, the proportion of vaccination against SARS‐CoV‐2 was comparable between the two arms (79.8% vs. 77.7%), and occasionally against influenza (0.3%). Post‐baseline 23.6% of participants were (re)vaccinated against SARS‐CoV‐2 (23.9% vs. 23.4%) (Figure 1a). None of participants was diagnosed with COVID‐19 prior to, and at baseline. Over 48‐week treatment SARS‐CoV‐2 was tested positive in 21.8% versus 18.6% of participants, respectively; diagnosis of COVID‐19 was established in 20.2% versus 18.6% of participants, respectively; and COVID‐19 was suspected in 13.1% versus 13.9% of participants, respectively (Figure 1b). No severe or critical illness was attributed to COVID‐19. None of participants was exposed to approved or investigational antiviral drug or any other therapies against COVID‐19.

**P349: Figure 1:**
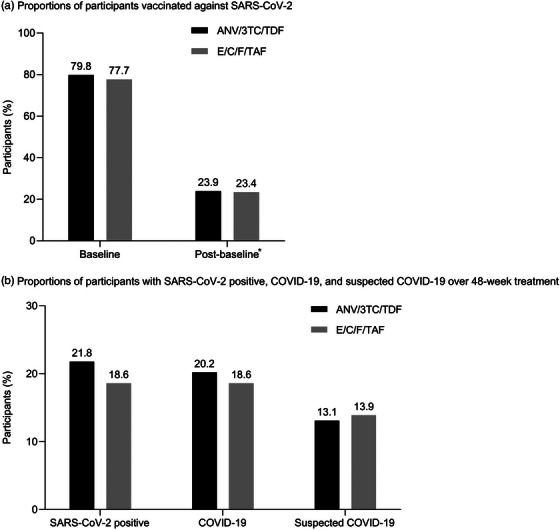
SARS‐CoV‐2 vaccination and proportions of participants with COVID‐19. (a) Proportions of participants vaccinated against SARS‐CoV‐2; (b) proportions of participants with SARS‐CoV‐2 positive, COVID‐19, and suspected COVID‐19 over 48‐week treatment. *Post‐baseline included revaccination. ANV/3TC/TDF, ainuovirine/lamivudine/tenofovir disoproxil; E/C/F/TAF, elvitegravir/cobicistat/emtricitabine/tenofovir alafenamide.

**Conclusions**: The SPRINT study population, a representative subset of virologically suppressed PWH, was well vaccinated against SARS‐CoV‐2 during the pandemic. No severe or critical illness of COVID‐19 occurred in this subpopulation, at a low incidence of testing positivity, and established or suspected diagnosis.

## People living with HIV and mpox virus

### P350 Mpox clade II in France in 2023‐2024: an endemic situation?

Mayda Rahi^1^, Sebastien Fouere^2^, Marie Gilbert^1^, Antoine Bachelard^1^, Fabien Taieb^3^, Baptiste Sellem^4^, Florian Herms^5^, Charles Cazanave^6^, Gentiane Monsel^7^, Yazdan Yazdanpanah^1^, Nathan Peiffer‐Smadja^1^, Jade Ghosn ^1^

^1^Infectious Diseases, Bichat University Hospital, Paris, France. ^2^Sexual Health Clinic, Saint Louis University Hospital, Paris, France. ^3^Centre Médical, Pasteur Institute, Paris, France. ^4^Infectious Diseases, Pitié‐Salpétrière University Hospital, Paris, France. ^5^Dermatology, Saint‐Louis University Hospital, Paris, France. ^6^Infectious Diseases, Bordeaux University Hospital, Bordeaux, France. ^7^Sexual Health Clinic, Pitié‐Salpétrière University Hospital, Paris, France

**Background**: The World Health Organization declared the end of clade IIb mpox virus (MPV) infection emergency on 11 May 2023. Since then, few cases have been reported in Europe. Third‐generation smallpox vaccination (MVA‐BN) for at‐risk population helped decreasing the number of new cases, but lack of data on long‐term vaccine effectiveness and vaccination coverage, especially for those living with HIV (PLWHIV), raise concerns about persistent circulation of MPV. The aim of this study was to describe laboratory‐confirmed cases of mpox in France since August 2023.

**Methods**: Demographic, clinical, and virological characteristics of PCR‐confirmed cases of mpox since August 2023 were collected from a national call via Infectio‐flash.

**Results**: Between August 2023 and June 2024, 31 laboratory‐confirmed cases of clade IIb MPV infection were identified in France. Median age was 34 [IQR 30–40], 94% men of whom 26 (90%) were men who have sex with men (MSM) with a median of 3 sexual partners in the previous month. Six (19%) were fully‐suppressed PLWHIV and three were diagnosed concomitantly with HIV and mpox. Two (6%) patients were immunocompromised (one solid‐organ transplant‐recipient and one with disseminated Kaposi's sarcoma, stage CDC C3 and CD4 <200/mm^3^). Nine (29%) and one (3%) patients had received two doses and one dose of MVA‐BN, respectively. Four (13%) patients were vaccinated during childhood. At the time of mpox diagnosis, 12 (39%) patients had typical genital and cutaneous lesions, 16 (52%) had isolated genital/anal lesions, and three (9%) had atypical lesions. Unvaccinated patients had significantly more lesions compared to vaccinated patients (median of 8 [IQR 3–14] vs. 4 lesions [2–6]). Median number of lesions (7) was similar in vaccinated (*n* = 5) and unvaccinated (*n* = 4) PLWHIV. Regardless of vaccination, PLWHIV exhibited a median of 7 [IQR 3–10] lesions versus 4 [IQR 2–11] in HIV‐negative patients. No patient showed critical complications.

**Conclusions**: This study shows persistent transmission of clade IIb MPV in France that could be explained by asymptomatic carriers and insufficient vaccination in high‐risk MSM. PLWHIV accounted for one‐third of mpox cases, including 9% with mpox revealing HIV infection, emphasising the need for targeted prevention messages and vaccination for MSM.

### P351 One‐year review of mpox in Hong Kong

Laura Pui Yee To ^1^, Ching See Leung^1^, Yau Chung Choi^1^, Alicia Wing Tung Lau^1^, Sophia Sharon Lamb^1^, Daphne Pui Ling Lau^1^, Thomas Shiu Hong Chik^1^, Ki Wai Heather To^2^, Bonnie Chun Kwan Wong^2^, Jacky Man Chun Chan^1^, Owen Tak Yin Tsang^1^

^1^Infectious Disease Centre, Department of Medicine and Geriatrics, Princess Margaret Hospital, Hong Kong Special Administrative Region, China. ^2^Special Preventive Programme, Department of Health, Hong Kong Special Administrative Region, China

**Introduction**: Hong Kong introduced the mpox vaccination programme, and saw its first mpox case in September 2022. Contrary to the global data, we did not observe a surge until July 2023. The delay was likely contributed by the strict travel restrictions implemented locally during the COVID‐19 pandemic. This study aims to describe our experience with the mpox cases in Hong Kong.

**Methods**: This retrospective descriptive study was performed in Princess Margaret Hospital, the designated Infectious Disease Centre in Hong Kong which provided inpatient care for all laboratory confirmed cases of mpox, between January 2023 and December 2023.

**Results**: Fifty‐two patients were confirmed positive for mpox PCR on lesion swabs. All cases were men (median age of 37 years). Ninety percent were Chinese. All but one reported sexual encounter prior to symptom onset, of which 88% reported sex with male, 6% with female and the rest with inconsistent history. Seventy‐three percent (*n* = 37) were casual sexual encounters, 33% (*n* = 17) had multiple sexual partners, and one reported encounters with different commercial sex workers. One case reported no sexual encounter, but was speculated to have acquired the infection via fomites during his holiday in Thailand. The majority of the cases (71%) were unvaccinated. Out of the 52 cases, 15 had known HIV and were on ART, while 5 were newly diagnosed with HIV upon their presentation with mpox. The median MPOX Severity Score System (MPOX‐SSS) were 5.5 and 7.5 for non‐HIV and HIV patients (*p* = 0.10); whereas for those who completed the two‐dose regime with JYNNEOS compared to the unvaccinated, the median was 4 and 7, respectively. All three patients treated with tecovirimat were unvaccinated and HIV positive, of which two patients were newly diagnosed with CD4 below 200 cells/µl. One case had relapsed mpox infection and received concomitant cidofovir with a second course of tecovirimat. There was no fatality.

**Conclusion**: Patients with HIV, especially those unvaccinated and not yet started on ART, are at risk of severe or disseminated mpox. Screening for HIV in those presenting with mpox infection is crucial. It is also imperative to sustain efforts to enhance vaccination coverage among vulnerable groups.

### P352 Exploring synergistic social media effects for people with HIV during the mpox epidemic: the evolution of the HIV case manager into a podcaster

Chung‐Ching Shih ^1^, Chin‐Fang Huang^2^, Wei‐Ming Chen^2^

^1^Infection Control Center, National Taiwan University Hospital, Taipei, Taiwan. ^2^Taiwan Positively Association, Taipei, Taiwan

**Introduction**: Mpox, an emergency transmitted disease among men who have sex with men, has created a situation of urgency, leading to severe illness among people with HIV (PWH). The mpox vaccine is the most effective strategy for ending the epidemic. As key populations get vaccinated quickly, PWH could prevent infection and stop the outbreak. Whether social media could be a promoter to accelerate vaccination coverage is crucial.

**Method**: The “SJ 愛 Talking” podcast is a social media platform for PWH and their loved ones. During the mpox epidemic, the podcast quickly delivered information on mpox vaccination, methods to prevent mpox, safe sexual behaviour, and guidelines for treatment in case of infection. We launched our episodes in February 2023, coinciding with the first mpox case in Taiwan. We track the vaccination coverage rate and mpox cases by group, evaluating the effectiveness of social media initiatives like podcasts.

**Results**: A total of 2819 PWH, 654 of whom listened to the podcast, 1805 PWH were vaccinated, and 20 were diagnosed with mpox. The results show they were getting the mpox vaccine simultaneously, and the podcast audience group increased the vaccine coverage rate faster. By the end of 2023, compared to the control group, the first vaccine coverage rate was significantly high, 68.99% versus 62.3%, and the second dose coverage was 56.2% and 50.9%, respectively (Figure 1). Regarding the outcome, the mpox infection rate in the podcast group was 0.465% lower than the control group's 0.782%. The OR of the podcast group was 0.593, meaning that people who listen to the podcasts would decrease their risk of being infected with mpox by 41%. However, in the control group, the chance of being infected with mpox is 1.69 times higher than listening to podcasts.

**P352: Figure 1:**
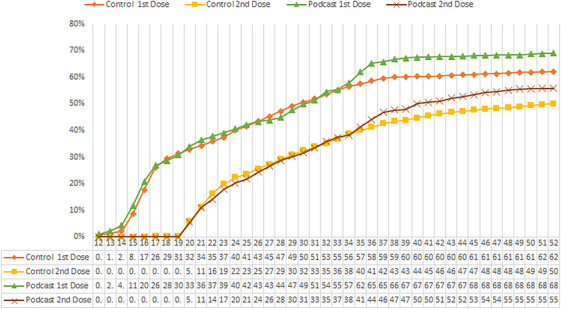
Comparing the effectiveness of “SJ 愛 Talking” podcast on mpox vaccine coverage by group in Taiwan 2023.

**Conclusions**: The “SJ 愛 Talking” podcast utilises the power of social media to disseminate vital prevention guidelines, counteract misinformation and boost the pace of vaccination coverage. Integrating social media into epidemic control amplifies and speeds up healthcare's effectiveness and makes it more beneficial for PWH. Therefore, further research is needed to integrate social media into healthcare strategies.

### P353 Exploring mpox vaccination uptake and tolerability among individuals living with HIV: a single‐centre study in an infectious disease unit in Italy

Samuele Gaggioli^1^, Giuseppe Formica^1^, Valentina Petrini^1^, Alessandra Russo Krauss^2^, Costanza Fiorelli^2^, Paola Corsi^2^, Alessandro Di Felice^1^, Costanza Malcontenti^2^, Michele Trotta^2^, Emanuela Francalanci^1^, Giulia Modi^1^, Francesca Costa^3^, Seble Tekle Kiros^1^, Lorenzo Zammarchi^1^, Alessandro Bartoloni^1^, Filippo Lagi ^2^

^1^Dipartimento di Medicina Sperimentale e Clinica, Università degli Studi di Firenze, Firenze, Italy. ^2^SOD Malattie Infettive Tropicali, AOU Careggi, Firenze, Italy. ^3^Dipartimento di Scienze della Salute (DISSAL), Università di Genova, Genova, Italy

**Introduction**: A live attenuated, non‐replicating vaccine (MVA‐BN) is available for vaccination against mpox. Initially administered intradermally, starting in January 2024 it has been available in Italy for subcutaneous administration only. This study aims to provide an overview of mpox vaccination uptake among people living with HIV (PLWH) in a single centre in Italy and explore its tolerability compared to individuals without HIV.

**Materials and methods**: We retrospectively collected routine data of people vaccinated with intradermal and subcutaneous MVA‐BN in a tertiary‐level hospital in Florence, Italy, from 15 September 2022 to 15 May 2024. Data were collected from pre‐vaccination screening questionnaires. Adverse events data were collected before the second dose through a dedicated questionnaire. Participants could report any adverse events anytime through phone or email contact.

**Results**: We vaccinated 332 subjects: 50.6% (*n* = 168) were already on follow‐up at our local HIV/STDs clinic. Overall, 36.1% (*n* = 120) were PLWH, who had a higher median age, higher rate of previous smallpox vaccination and a higher proportion of transgender individuals compared to people without HIV (Table 1). The first vaccine shot was administered intradermally in 208 persons (62.6%) and subcutaneously in 124 (37.5%). As for vaccine tolerability, subcutaneous administration was associated with significantly fewer adverse events than intradermal (13 [12.6%] vs 47 [30.1%], respectively; *p* = 0.002). This difference was observed in both PLWH (9.5% vs 27.2%) and individuals without HIV (12.9% vs 32.3%). All events were Grade 1 in severity. Overall, among people who were supposed to receive two doses of the vaccine, 18 (5.8%) did not complete the cycle, with a high proportion of migrants (83.4%) and sex workers (77.7%). All individuals who did not return for the second dose did so due to personal choice rather than adverse reactions. One individual developed mpox 10 days after the first dose; none of the others routinely followed up at our centre contracted mpox.

**P353: Table 1:** Baseline characteristics of a group of people vaccinated for mpox from 15/09/2022 to 15/05/2024 at the outpatient clinic of the Infectious and Tropical Diseases Unit, Careggi Teaching Hospital, Florence, Italy

	Without HIV (*n* = 212)	With HIV (*n* = 120)	*p*‐value^a^
Gender; *N* (%)			0.007
Cisgender male	194 (91.5)	103 (85.8)	
Cisgender female	6 (2.8)	0 (0)	
Transgender female	12 (5.7)	17 (14.2)	
Median age in years [IQR]	37 [31–45]	43 [38–52]	<0.001
Country of origin; *N* (%)			
Italy	186 (87.7)	89 (74.2)	
Peru	12 (5.7)	19 (15.8)	
Brazil	7 (3.3)	2 (1.7)	
Other	7 (3.3)	10 (8.3)	
Self‐defining MSM; *N* (%)	185 (87.3)	103 (85.8)	0.025
Self‐defining sex worker; *N* (%)	13 (6.1)	17 (14.2)	0.025
No. of expected doses; *N* (%)			<0.001
1 dose	23 (10.9)	31 (25.8)	
2 doses	189 (89.2)	89 (74.2)	
Number of individuals who did not complete the second dose; *N* (%)	8 (3.7)	10 (8.3)	0.078
Intradermal administration; *N* (%)	115 (54.2)	93 (77.5)	<0.001
Adverse event (AE) after the first intradermal dose^b^; *N* (%)			0.164
Local	31 (32.9)	16 (26.6)	
Systemic	0 (0)	2 (3.3)	
Adverse event (AE) after the first subcutaneous dose^b^; *N* (%)			0.698
Local	11 (12.9)	2 (9.5)	
Systemic	2 (2.3)	0 (0)	
Reported any allergies; *N* (%)			0.299
None	160 (75.5)	96 (80)	
Environmental	36 (17)	13 (10.8)	
Pharmacological	16 (7.6)	11 (9.2)	
Cardiovascular diseases; *N* (%)	7 (3.3)	6 (5)	0.443
Asthma; *N* (%)	7 (3.3)	5 (4.2)	0.691
Other vaccination received between 15 and 30 days before vaccination for mpox; *N* (%)	13 (6.1)	7 (5.8)	0.912

^a^ Differences between the two groups were described using chi‐square test, Fisher's exact test for categorical variables, and Mann–Whitney test for continuous variables.

^b^ Evaluated only in the person who got two doses (*n* = 260). Persons with only one dose were excluded.

**Conclusions**: In conclusion, our limited experience suggests that mpox vaccination has been accepted and well‐tolerated in individuals with and without HIV. More work is needed to promote awareness and immunization campaigns in vulnerable populations such as migrants and sex workers.

### P354 The frequency of scars following mpox: an aftermath of the 2022 outbreak

David Chromy ^1^, Nikolaus Urban^1^, Wolfgang Michael Bauer^1^, Alexander Kreuter^2^, Robert Strassl^3^, Katharina Grabmeier‐Pfistershammer^1^

^1^Department of Dermatology, Medical University of Vienna, Vienna, Austria. ^2^Department of Dermatology, Venereology and Allergology, Helios St. Elisabeth Hospital Oberhausen, University of Witten/Herdecke, Oberhausen, Germany. ^3^Division of Clinical Virology, Department of Laboratory Medicine, Medical University of Vienna, Vienna, Austria

**Introduction**: Mpox incidence of the 2022 epidemic among men who have sex with men (MSM) caused by the monkeypox virus (MPXV) has substantially decreased, yet new cases are still occurring. The epidemic caused by MPXV clade IIb is usually a self‐limiting disease with low mortality, which contrasts with 1–10% mortality rate reported for endemic mpox caused by clade I. However, individuals infected with the epidemic MPXV clade IIb are frequently concerned about potential scarring as permanent sequelae. While scar formations are a common feature of smallpox and endemic mpox caused by MPXV clade I, it is unclear whether lesions of the less virulent clade IIb 2022 outbreak resolve without scars. We thus aimed to investigate the long‐term outcome defined as the incidence of scarring following mpox infections of the 2022 outbreak.

**Materials and methods**: All individuals diagnosed with mpox at the Department of Dermatology at the Medical University of Vienna in 2022 were included in this analysis. Follow‐up data was collected throughout November 2023. “Scarring/scar formation” was defined as having at least one scar at the former active mpox lesions.

**Results**: Twenty‐eight cases of mpox were detected between June 2022 and October 2022. All occurred among MSM 100% (28/28), 46% (13/28) were living with HIV, whereas 32% (9/28) were using PrEP. All patients were symptomatic: pain (68%, 19/28), lymphadenopathy (54%, 15/28), papules (54%, 15/28), pustules (43%, 12/28) and ulcers (68%, 19/28), whereas three patients also presented with a generalized rash. Three individuals had a coinfection with gonorrhoea, and in a single person early syphilis was diagnosed. Secondary bacterial infection of mpox lesions was suspected in six individuals, and all received systemic antibiotics. Twenty‐one patients had follow‐up available (median time of follow‐up 15 months), whereas seven individuals were lost to follow‐up. Of those 21 individuals, 43% (9/21) showed scarring at least at one site of previous mpox lesions.

**Conclusions**: Our study provides clinically relevant new data on the long‐term lesional outcome following mpox and thereby offers insights on late sequelae. Almost half of all patients experienced residual scar formation. This underlines the importance of further improving prevention strategies to contain the epidemic.

## People living with HIV and sexually‐transmitted diseases

### P355 Knowledge, attitudes and behaviours in relation to doxycycline post‐exposure prophylaxis (dPEP) use amongst attendees and healthcare workers at a large sexual health and HIV service in Ireland

Amy Keane ^1^, Fiona Lyons^1^, Diego Caixeta^2^, Colette Smith^3^, Jean‐Michel Molina^4^, Giovanni Villa^1^

^1^Department of Genitourinary Medicine and Infectious Diseases, St James Hospital, Dublin, Ireland. ^2^HIV Ireland, Dublin, Ireland. ^3^Institute for Global Health, University College London, London, UK. ^4^Department of Infectious Diseases, Saint‐Louis and Lariboisiere Hospitals, Paris, France

**Introduction**: The study aimed to assess the knowledge, attitudes, and behaviours concerning dPEP among clinic attendees and healthcare workers (HCWs) in Ireland.

**Methods**: Following ethical approval two anonymous surveys were conducted at two sexual health/PrEP services in Ireland, targeting attendees and HCWs. The surveys collected demographic data, knowledge, attitudes and behaviours around dPEP. Survey data was analysed using Stata (version 16).

**Results**: Three hundred and seventy‐eight attendees responded: median age 32 (IQR 26–39); 303 (80%) male; 64 (17%) female and 10 (3%) non‐binary/trans/gender fluid. The majority of respondents were gbMSM (259, 66%). Respondent characteristics by sexual behaviour/sexuality are shown in Table 1. STIs in the previous year: gonorrhoea (117/378 [31%]); chlamydia (106/378 [28%]); and syphilis (42/378 [11%]). One hundred and sixteen of 378 (31%) heard of dPEP: 39% of gbMSM; 7% heterosexual; and 36% other. Sixty of 378 (16%) took antibiotics after sex to prevent STIs in the past 1 year, 35 (9%) in the past 3 months. Thirty‐five of 378 (9.3%) respondents took doxycycline after sex in the past 3 months, all but two gbMSM. Partner number (median 30, IQR 10–60) and STI in the previous year (27 [77%]) was highest in this group. One hundred and seventy‐three (46%) would prefer testing and treatment to dPEP. Respondents would like dPEP to provide 85% STI protection (median 85%, IQR 70–99). One hundred and sixty‐four of 273 (60%) ranked additional real‐world data as the most important information to them prior to using dPEP. Fifty‐two HCWs responded: 22 doctors, 26 nurses and 4 pharmacists. Thirty‐two of 52 (62%) consider dPEP a positive tool for STI reduction; 46 of 52 (86%) have concerns; 20 of 52 (39%) would be happy to prescribe dPEP with the current evidence available.

**P355: Table 1:** Respondent characteristics by sexual behaviour/sexuality

Variable	Total	gbMSM	Heterosexual	Other
*n* (%)	378	259	95	24
Number of sexual partners in the previous year, median (IQR)	6 (2–20)	12 (4–30)	2 (1–5)	4.5 (2.5–8)
Tested for STI in the previous year, *n* (%)				
Yes	314 (83.1)	231 (89.2)	66 (69.5)	17 (70.8)
No	62 (16.4)	27 (10.4)	28 (29.5)	17 (70.8)
Missing	2 (0.5)	1 (0.4)	1 (1.1)	0 (0.00)
Diagnosed with STI in the previous year, *n* (%)				
Yes	201 (53.2)	157 (60.6)	34 (35.8)	10 (41.7)
No	177 (46.8)	102 (39.4)	61 (64.2)	14 (58.3)
Number of STI diagnoses in the previous year in those that answered	2 (1–2)	2 (1–2)	1 (1–1)	1 (1–2)
Attending HIV clinic, *n* (%)				
Yes	208 (55)	153 (59.1)	43 (45.7)	12 (50)
No	169 (44.7)	105 (40.5)	52 (54.7)	12 (50)
Missing	1 (0.3)	1 (0.4)	0 (0.00)	0 (0.00)

**Conclusion**: A significant proportion of attendees knew about dPEP, almost 1 of 10 had taken doxycycline for STI prophylaxis. Those reporting recent doxycycline prophylactic use had higher partner number and STI diagnoses, suggesting appropriate self‐selection. Almost one of two would prefer a test‐and‐treat approach. Notably, respondents desired substantial protection from dPEP and prioritised additional real‐world data to inform their dPEP use in the future. Whilst the majority of HCWs see a role for dPEP, high levels of concern remain and less than half would be happy to prescribe with current evidence. Findings suggest need for continued data collection and monitoring of the role of dPEP in preventing STIs.

### P356 Incidence and clearance of high‐grade anal squamous intraepithelial lesions in men who have sex with men living with HIV

Maria Saumoy ^1^, Ana Silva‐Klug^1^, Jaime Vega^1^, Monica Sánchez^1^, Joao Carmezin^2^, Miguel Angel Pavon^3^, Laia Alemany^3^, Núria Baixeres^4^, Loris Trenti^5^, Sónia Paytubi^6^

^1^HIV and STD Unit, Infectious Disease Service, Hospital Universitari de Bellvitge, Hospitalet de Llobregat, Spain. ^2^Biostatistics Support and Research Unit, Germans Trias i Pujol Research Institute and Hospital, Badalona, Spain. Cancer Epidemiology Research Program, Infections and Cancer, ^3^Catalan Institute of Oncology, Hospitalet de Llobregat, Spain. ^4^Anatomy Pathology Service, Hospital Universitari de Bellvitge, Hospitalet de Llobregat, Spain. ^5^Surgery Service, Hospital Universitari de Bellvitge, Hospitalet de Llobregat, Spain. ^6^Infections and Cancer Laboratory, Catalan Institute of Oncology, Hospitalet de Llobregat, Spain

**Introduction**: Anal cancer is preceded by high‐grade‐intraepithelial lesions (HSIL). The detection and treatment of HSIL reduce the incidence of anal cancer. However, spontaneous regression of HSIL can occur. We aim to describe HSIL incidence and clearance rate in the ELAVI cohort. Clinical and human papillomavirus (HPV) biomarkers were assessed.

**Methods**: The ELAVI is a prospective cohort of men who have sex with men living with HIV, participating in an anal screening programme from June 2016 to March 2021 in the Bellvitge Hospital of Barcelona. Participants underwent an anal cytology and high‐resolution anoscopy with biopsy of suspected dysplasia areas every 6 (if HSIL) or 12 months, and did not receive treatment for HSIL. Composite cytological and histological results were used. HPV‐DNA detection performed by Linear Array^®^ (LA) and Hybrid Capture^®^2 (HC2), and E6/E7‐mRNA tested using Aptima^®^. Independent proportional hazards models were performed for each outcome (HSIL incidence and clearance).

**Results**: Three hundred and fifty‐four participants make up the ELAVI cohort, of which 291 have been included in the study because they have a follow‐up >2 years. Median follow‐up of 35.2 months (IQR 27.3–41.1). Mean age 45.6, mean CD4 T‐cell count 793 cells/mm^3^ and 87.6% had undetectable HIV‐1 RNA viral load. At baseline, 78 (26.8%) participants had HSIL. The cumulative incidence of HSIL was 27.2% (58/213) with a rate of 10.7 (8.1–13.9) per 100 person‐years (PY). The cumulative clearance HSIL was 54.6% (59/108) with a rate of 25 (19–32.3) per 100 PY. Baseline HIV‐1 RNA (HR 2.40 [1.29–4.49]), LSIL cytology (HR 2.48 [1.23–5.01]) and the presence of HPV (measured by HC2 (HR 2.20 [1.30–3.72]) and E6/E7‐mRNA (HR 2.98 [1.42–6.26])) were associated with increased risk of incident HSIL. Only the absence of HPV‐16 (measured by LA (HR 0.44 [0.24–0.81]) and E6/E7‐mRNA (HR 0.47 [0.25–0.89])) was associated with HSIL clearance. One case of superficial anal cancer was diagnosed.

**Conclusions**: Incident HSIL occurred at 10.7 per 100 PY and was associated with HIV‐1 RNA viral load and HPV‐biomarkers. More than half of HSIL cleared spontaneously during follow‐up. Detection of HPV‐16 can help determine whether HSIL treatment is necessary.

### P357 Characterisation of specific T‐cell responses to a three‐doses nonavalent HPV vaccine schedule in PLWH on ART

Eeva Tortellini ^1^, Mariasilvia Guardiani^1^, Federica Dominelli^1^, Carmen Falvino^1^, Anna Carraro^1^, Sara Corazza^2^, Sara Giovanna De Maria^2^, Silvia Garattini^2^, Blerta Kertusha^1^, Lorenzo Ansaldo^2^, Maria Antonella Zingaropoli^1^, Fabio Mengoni^1^, Cristina Giambi^3^, Cosmo Del Borgo^2^, Raffaella Marocco^2^, Miriam Lichtner^4^

^1^Public Health and Infectious Diseases, Sapienza University of Rome, Rome, Italy. ^2^Infectious Diseases Unit, Santa Maria Goretti Hospital, Sapienza University of Rome, Latina, Italy. ^3^UOS Profilassi e Sorveglianza Malattie Infettive, Santa Maria Goretti Hospital, Sapienza University of Rome, Latina, Italy. ^4^Neuroscienze Salute Mentale e Organi di Senso (NESMOS), Sapienza University of Rome, Rome, Italy

**Introduction**: PLWH have a higher incidence and persistence of human papillomavirus (HPV) infection, leading to an augmented risk of HPV‐related cancers. A nonavalent vaccine has been approved for the prevention of HPV in PLWH. Since most studies are focused on the antibody response, we investigated specific T‐cell responses in PLWH after nonavalent HPV vaccination, with a focus on its functional profile.

**Material and methods**: PLWH on ART under routine follow‐up at the Infectious Diseases Unit of S.M. Goretti Hospital, Italy, were enrolled. T‐cell responses were assessed by intracellular cytokine flow cytometry assay at the moment of the first‐dose (T0), at 6 (T1) and 12 months (T2) from it after stimulation of whole blood with a pool of HPV16 and HPV18 L1 peptide libraries. Through Boolean‐gating, we identified T cells producing all possible combinations of interferon gamma (IFNg), interleukin‐2 (IL2) and tumor necrosis factor alpha (TNFa), defining those producing any of them as responding T cells and those simultaneously producing all three as polyfunctional. The population was then stratified according to previous HPV infection and current CD4 cell‐count.

**Results**: Thirty‐eight PLWH (29 male/nine female, median age [IQR] of 41 [34–50] years, median CD4 T‐cell count [IQR] 716 [419–881] cells/µl) were enrolled between September 2022 and May 2023. HPV infection had previously been diagnosed in 12 of 38 patients (32%). Overall, an increase in the percentage of both responding and polyfunctional T cells was found at T3 compared to T (CD4: *p* < 0.0001 and *p* = 0.0041, respectively; CD8: *p* = 0.0065 and *p* = 0.0392, respectively). Stratifying the population according to previous HPV infection, an increase in the percentage of responding and polyfunctional T cells was observed in HPV− group (CD4: *p* < 0.0001 and *p* = 0.0019, respectively; CD8: *p* = 0.0135 and *p* = 0.05, respectively) as well as in and HPV+ group, although not significant. Stratifying according to current CD4 cell‐count, the increase was found only in CD4 >500 group (CD4: *p* < 0.0001 and *p* = 0.0015, respectively; CD8: *p* = 0.0020 and *p* = 0.0097, respectively).

**Conclusions**: Overall, a very good specific T‐cell response after HPV vaccination was observed, with an increase in the percentage of both responding and polyfunctional T cells. Vaccination improves the quality and magnitude of the response in HPV+ group, supporting the utility of vaccination in this category of people. PLWH with current CD4 <500 seems to have a lower polyfunctional capacity at T2, confirming that it remains a group that needs to be attentioned and who may benefit of ad hoc vaccination strategies.

### P358 Study on the prevalence and incidence of dysplasia and HPV infection of the oropharyngeal, cervical and anal mucosa (oropharyngeal) of people with HIV

Carmen Hidalgo Tenorio ^1^, Inmaculada Calle‐Gómez^2^, Raquel Moya‐Mejias^3^, Mohamed Omar^4^, Javier Lopez Hidalgo^5^, Javier Rodriguez Granges^6^, Carmen Garcia Martinez^7^

^1^Infectious Diseases, Hospital Universitario Virgen de las Nieves, Granada, Spain. ^2^Internal Medicine, Hospital Básico Baza, Granada, Spain. ^3^Internal Medicine, Complejo Hospitalario de Jaen, Jaen, Spain. ^4^Infectious Diseases, Complejo Hospitalario de Jaen, Jaen, Spain. ^5^Pathology Service, Hospital Universitario Virgen de las Nieves, Granada, Spain. ^6^Microbiology Service, Hospital Universitario Virgen de las Nieves, Granada, Spain. ^7^Internal Medicine, Hospital Universitario Virgen de las Nieves, Granada, Spain

**Introduction**: The main objective was to describe the prevalence and incidence of human papillomavirus (HPV) infection in the oropharyngeal mucosa as well as that of dysplasia in this mucosa in 12 months of follow‐up, together with the rate of clearance and acquisition of this infection; and to compare it with the infection and dysplasia in the anal mucosa of men and women, and genital mucosa only in women.

**Material and methods**: Longitudinal, prospective (period from December 2022 to April 2024). At the baseline and 12‐month visits, clinical and laboratory variables related to HIV were collected, and oropharyngeal mucosal swabs were collected for PCR for HPV and other sexually transmitted infections; together with mucosal samples from the anal canal and female genital mucosa by autotomy.

**Results**: Two hundred and seventy‐six participants were included with a mean age of 45.3 years, 79% were men, 24.3% had history of AIDS, 100% were taking ART and 0.7% were in virological failure. 30.1% had received the full course of HPV vaccine. The prevalence of HPV infection in the oropharyngeal region was 11.2%, the most frequent genotype was 16 (2.2%); and none had dysplasia. Fifty percent of women had genital HPV infection, HPV‐16 was the most prevalent (11%), 1.8% had CIN1. 79.8% of the PLHIV had anal HPV infection, the most frequent genotypes were 44 of 55 (16.9%), 62 of 81 (19.5%) and 16 (12.3%); 29.9% had low squamous intraepithelial lesion (LSIL [AIN1]) and 1.5% high squamous intraepithelial lesion (HSIL [AIN2/3]). At 12 months, the incidence of oropharyngeal dysplasia was zero, 5.5% cleared high risk human papillomavirus (HR‐HPV) and 4.4% acquired it; the incidence of anal HSIL was 1811.6 cases × 100,000 person‐years, the clearance rate of HR‐HPV was 16.2% and that of acquisition 25.6%; the incidence of CIN2/CIN3 or cervical cancer was zero, 11.3% cleared HR‐HPV and 7.5% acquired it. At 1‐year follow‐up, VL <50 cop/ml was the only factor related to oropharyngeal mucosa infection due to HPV (97.2% vs 87%, HR 0.044; 95% CI [0.042–0.956]).

**Conclusions**: In PLHIV the highest prevalence and incidence rate of HPV infection and HSIL lesions are found in the anal canal, and the lowest in the oropharyngeal region. Undetectability was a protective factor against HPV infection in oropharyngeal mucosa.

### P359 Exploring interest in long‐acting antiretroviral injections: perspectives of people living with HIV in Egypt and Saudi Arabia

Salma M. A. Mansour^1^, Roaa Alosaimi ^2^, Heba Fahmy^3^, Ahmed Cordie^3^, Rahma Mohammed^3^, Batool Ali^2^, Ganna Essam Elnahas^3^, Amany A. Salem^4^, Ibrahim Ali Kabbash^5^

^1^Egyptian Patent Office, Academy of Scientific Research and Technology (ASRT), Cairo, Egypt. ^2^Infectious Diseases, HIV Centre of Excellence, East Jeddah Hospital, Jeddah, Saudi Arabia. ^3^Endemic Medicine Department, Cairo University Hospitals, HIV Clinic, Cairo, Egypt. ^4^Public Health Department, Cairo University Hospitals, Cairo, Egypt. ^5^Public Health & Community Medicine, Tanta University, Tanta, Egypt

**Introduction**: Receiving a daily oral dosage of antiretroviral treatment (ART) could be challenging for people living with HIV (PLHIV) in the Middle East and North Africa (MENA) region. Therefore, a cross‐sectional study was carried out to identify the willingness of PLHIV to use the long‐acting antiretroviral injections (LAI), explore the factors associated with interest in trying LAI among PLHIV, and to evaluate perceived benefits and constraints of using LAI from PLHIV's perspective.

**Methods**: A validated survey was used after customisation to fit PLHIV in Egypt and Saudi Arabia (KSA). An overview of the LAI was provided, consent form was taken, and data was gathered from 624 PLHIV; 363 Egypt and 261 KSA, between October 2023 and June 2024 at Cairo University Hospitals HIV Clinic, Egypt and East Jeddah Hospital, KSA. Descriptive statistics was performed via Excel and SPSS V.23.

**Results**: Of the enrolled PLHIV, 79.6% (275 Egypt, 222 KSA) were males, 70.8% were in the age group of 18–39 years, and 60.7% (240 Egypt, 139 KSA) were diagnosed with HIV in the last 4 years. All participants were on ART, of them, 90.9% (328 Egypt, 239 KSA) expressed a high interest in the LAI. The majority of study participants had confidentiality concerns about their condition; as 68.6% of PLHIV (313 Egypt, 115 KSA) informed only their close‐knit circle about their condition, and 27.1% (42 Egypt, 127 KSA) were uncomfortable with self‐disclosure. Moreover, 29.2% (117 Egypt, 62 KSA) reported negative feelings towards ART, and 45.3% (193 Egypt, 84 KSA) were neutral. Participants from both countries agreed on the top four perceived benefits (Figure 1), while each cohort reported various constraints; the main constraint among PLHIV in Egypt was the injections' pain, while in KSA was fear of missing dose due to lack of LAI availability at any time.

**P359: Figure 1:**
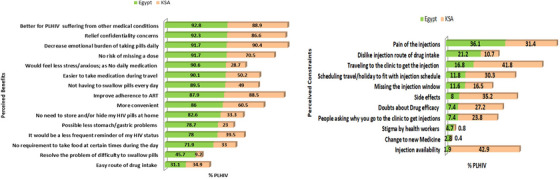
Perceived benefits and constraints to using LAI, PLHIV's perspective.

**Conclusion**: Our findings indicate that LAI may help address PLHIV unmet needs and may be more convenient for most of them.

### P360 Treatment of *Mycoplasma genitalium* and detection of macrolide resistance mutations (23S rRNA) in PHIV and HIV‐negative individuals

Elena Suanzes‐Martín^1^, María Muñoz‐Algarra^2^, Sara de la Fuente Moral^3^, Ilduara Pintos‐Ramos^1^, Natalia Vicente‐López^1^, Alberto Díaz de Santiago ^3^

^1^Internal Medicine, Puerta de Hierro University Hospital, Madrid, Spain. ^2^Microbiology, Puerta de Hierro University Hospital, Madrid, Spain. ^3^HIV‐AIDS Medicine (Internal Medicine), Puerta de Hierro University Hospital, Madrid, Spain

**Introduction**: *Mycoplasma genitalium* (MG) is rapidly developing resistance to antimicrobials. Diagnostic tests must include detection of macrolide resistance mutations (MRM) in 23S rRNA to guide treatment. Our objective is to evaluate the characteristics of MG cases and the detection of macrolide resistance mutations (23S rRNA).

**Materials and methods**: A retrospective observational study of MG cases from 1 March 2023 to 31 January 2024 (11 months) was conducted. Statistical analyses were performed using STATA v12.0.

**Results**: Sixty‐four patients were identified with a positive nucleic‐acid amplification test (NAAT) for MG: 35 cases in individuals on HIV‐PrEP, 15 cases in people with HIV (PHIV) and 14 cases in the general population (from emergency services). Two hundred and eight PrEP‐HIV users (100% STI screening). MG prevalence: 16.8% (35/208). One hundred and thirty‐five patients referred from emergency services for possible STIs or risky sexual behaviour (80% STI screening). MG prevalence: 10.3% (14/135). Excludes people living with HIV and those on PrEP. PHIV (N = 750). MG NAAT detected in 15 PHIV but calculating a denominator is challenging as screening is not routinely performed (Table 1). 46.7% of patients showed different symptoms of active infection. Localisation of symptomatic MG infection: rectum 31%, urine/urethra 55%, rectum and urine/urethra 3.5%, pelvic inflammatory disease 10.5%. Detection of macrolide resistance mutations in 23S rRNA: performed: 81.4%; no resistance: 36.7%; resistance: 59.3%; invalid: 4%; no resistance in symptomatic/asymptomatic: 34.8%/38.5%, *p* = 0.96; no resistance in rectum/urethritis: 25%/35%, *p* = 0.56; negative NAAT confirmed after treatment: 40%; days until negative NAAT after treatment (median): 90 (IQR 16–90); patients who received moxifloxacin after doxycycline despite macrolide being useful according to 23S rRNA: 24%. Ninety percent received sequential treatment with doxycycline and moxifloxacin; 8.3% with doxycycline followed by azithromycin, 1.7% doxycycline only. We did not identify hepatitis C or B. One patient was simultaneously diagnosed with primary HIV infection. There was a high coinfection rate with chlamydia (37.5%). Gonorrhoea was identified in 22% of cases.

**P360: Table 1:** Epidemiological characteristics

Characteristics	Details
Gender	Male 90.63% (58), female 9.38% (6)
Age (median)	31.5 years (IQR 27‐42.5)
MSM (among males)	78.13% (50), 82.8% (48/58)
Country of origin	Spain 72%, Latin America 23.5%, others 4.5%
PrEP‐HIV users	Daily PrEP 54.7%, on‐demand PrEP 79.5%, duration on PrEP (months): 9 (IQR 4–21)
PHIV (previously known)	Duration of HIV infection (years): 8 (IQR 1–12), AIDS 20%, on ART 100%, virally suppressed (HIV VL <50 copies/ml) 71.4%
Chemsex	14.75%
Slamsex	1.67%

**Conclusions**: Observed MG prevalence is higher (14%) than expected. Symptomatic MG achieved almost half of all positive NAAT. Two‐thirds showed 23S rRNA macrolide resistance mutations (MRM). Twenty‐four percent could receive azithromycin after doxycycline instead of moxifloxacin if MRM test was available before.

### P361 Antimicrobial resistance pattern in *Neisseria gonorrhoeae* isolates from men having sex with men (MSM): results from the first prospective cohort in Poland

Bartosz Szetela ^1^, Beata Maczynska^2^, Konrad Starzynski^2^, Daria Zareba^2^, Danuta Ruranska‐Smutnicka^2^, Anna Sacewicz^2^, Paulina Szuba^2^, Martyna Biala^1^

^1^Infectious Diseases, Liver Disease and Acquired Immune Deficiencies, Wroclaw Medical University, Wroclaw, Poland. ^2^Department of Pharmaceutical Microbiology and Parasitology, Wroclaw Medical University, Wroclaw, Poland

**Introduction**: The rate of gonorrhoea is much higher in men having sex with men (MSM) than in general population [1]. *Neisseria gonorrhoeae* (NG) has developed resistance to nearly all antibiotics used for its treatment [2–4]. Little up‐to‐date data is available regarding antimicrobial resistance of NG isolates among MSM in Poland [3]. The aim of this study was to evaluate the susceptibility of NG isolates in this key population.

**Methods**: Two hundred MSM living with HIV or using HIV pre‐exposure prophylaxis (PrEP) from Wroclaw All Saints' outpatient clinic were included between October 2022 and April 2024. Inclusion criteria were age over 18 years, identifying as MSM, having symptoms suggesting NG infection or PCR smears positive for NG, having sexual partner with positive PCR results and/or symptoms or having multiple sexual partners in the last 3 months. We investigated antimicrobial susceptibility of NG isolates to six antimicrobials (ceftriaxone, cefixime, azithromycin, ciprofloxacin, tetracycline, and benzylpenicillin). Minimum inhibitory concentrations (MICs; mg/L) were determined using Etest on gonococcal isolates. Patients filled out an online behavioural questionnaire before the swabs collection.

**Results**: Two hundred high‐risk MSM were included in the study (67 living with HIV and 133 HIV‐negative using PrEP). The rate of NG infection was 23% (46 isolates/200 cultures) with positive cultures from urethral (25), oropharyngeal (12) and rectal (9) sites. However, we were able to obtain resistance profiles for 43 isolates (Table 1). All NG isolates were susceptible to cefixime and ceftriaxone. Susceptibility to azithromycin was found in 69.7% (30/43) of the NG isolates and resistance in 30.3% (13/43). Susceptibility to tetracycline was found in 51% (22/43) and resistance in 49% (21/43) of the isolates.

**P361: Table 1:** Antimicrobial susceptibility of NG isolates

	Penicillin isolates, *n* [%]	Cefixime isolates, *n* [%]	Ceftriaxone isolates, *n* [%]	Ciprofloxacin isolates, *n* [%]	Tetracycline isolates, *n* [%]	Azithromycin isolates, *n* [%]
S—susceptible	12 [27.9]	43 [100]	43 [100]	10 [23]	22 [51]	30 [69.7]
I—intermediate	23 [53.5]	0	0	3 [7]	0	0
R—resistant	8 [18.6]	0	0	30 [70]	21 [49]	13 [30.3]
Total	43 [100]	43 [100]	43 [100]	43 [100]	43 [100]	43 [100]

**Conclusions**: All isolates remain susceptible to cephalosporins. Increasing azithromycin resistance is especially concerning for future treatment options, especially if ceftriaxone/cefixime resistance starts to develop. Resistance to azithromycin in 30% of isolates should prompt changes in the combined treatment guidelines for NG [5]. Easy testing for populations at risk is needed including monitoring of resistance patterns, their dynamics and spread. DoxyPEP partial efficacy for NG might decrease soon as rapid increase in doxycycline resistance in this group of patients is highly probable.

**References**

1. World Health Organization. Gonorrhoeae [Internet]. 2024 [cited 2024 Feb 6]. Available from: https://www.who.int/news‐room/fact‐sheets/detail/gonorrhoea‐(neisseria‐gonorrhoeae‐infection).

2. Golparian G, Cole MJ, Sánchez‐Busó L, Day M, Jacobsson S, Uthayakumaran T, et al. Antimicrobial‐resistant *Neisseria gonorrhoeae* in Europe in 2020 compared with in 2013 and 2018: a retrospective genomic surveillance study. Lancet Microbe. 2024;5:e478–88.

3. European Centre for Disease Prevention and Control. Gonococcal antimicrobial susceptibility surveillance in the European Union/European Economic Area, 2022. Stockholm: ECDC; 2024.

4. Day MJ, Jacobsson S, Spiteri G, Kulishev C, Sajedi N, Woodford N, et al. Significant increase in azithromycin “resistance” and susceptibility to ceftriaxone and cefixime in *Neisseria gonorrhoeae* isolates in 26 European countries, 2019. BMC Infect Dis. 2022;22(1):524.

5. Kenyon C, Herrmann B, Hughes G, de Vries HJC. Management of asymptomatic sexually transmitted infections in Europe: towards a differentiated, evidence‐based approach. Lancet Reg Health Eur. 2023:34:100743.

### P362 Syphilis incidence among HIV‐infected MSM and IDUs is as high before and after achieving U═U in Japan

Hauka Uemura, Daisuke Mizushima, Naokatsu Ando, Takato Nakamoto, Takahiro Aoki, Katsuji Teruya, Hiroyuki Gatanaga

AIDS Clinical Center, National Center for Global Health and Medicine, Tokyo, Japan

**Introduction**: There is accumulating evidence that the risk of HIV transmission is dependent on the viral load of the source of exposure and that condomless sex does not result in transmission to the partner if the blood viral load of the exposed source is below the detection limit (U═U). Therefore, we hypothesized that after achieving U═U, there may be a change in PWHIV behaviour and an increase in STIs. The aim of the study was to assess the actual changes in syphilis incidence after ART initiation.

**Method**: A single‐centre, retrospective study was conducted in Tokyo, Japan, where MSM or IDU cases who started initial ART between January 2017 and December 2021 were observed for syphilis incidence from ART initiation to the end of 2023. Syphilis testing was performed in all cases at the start of treatment, but thereafter when suspected by the practitioner; U═U was defined as achieving a blood HIV‐RNA level of less than 200/ml for a sustained period of 6 months. The first objective was to observe syphilis incidence during the entire study period and the second objective was to observe syphilis incidence from the start of ART up to 2 years.

**Result**: Of the 585 treatment‐naïve PWHIV, 271 had a history of syphilis. Of the 413 patients who started ART, 389 (94.2%) achieved U═U within 1 year of starting ART. During the entire study period, 413 patients were observed for 1457.2 person‐years and 80 cases of syphilis were diagnosed (incidence rate of 5.95/100 person‐years, 95% CI 4.35–6.83); the syphilis incidence rates for each year from the first year after ART initiation were 4.37, 6.45, 7.28, 4.17 and 10.14/100 person‐years, respectively, with no statistically significant difference. In an observation limited to the first 2 years of ART, 413 patients were observed for 711.5 person‐years and 37 cases of syphilis were diagnosed (incidence rate 5.20/100 person‐years, 95% CI 3.66–7.17, Figure 1).

**P362: Figure 1:**
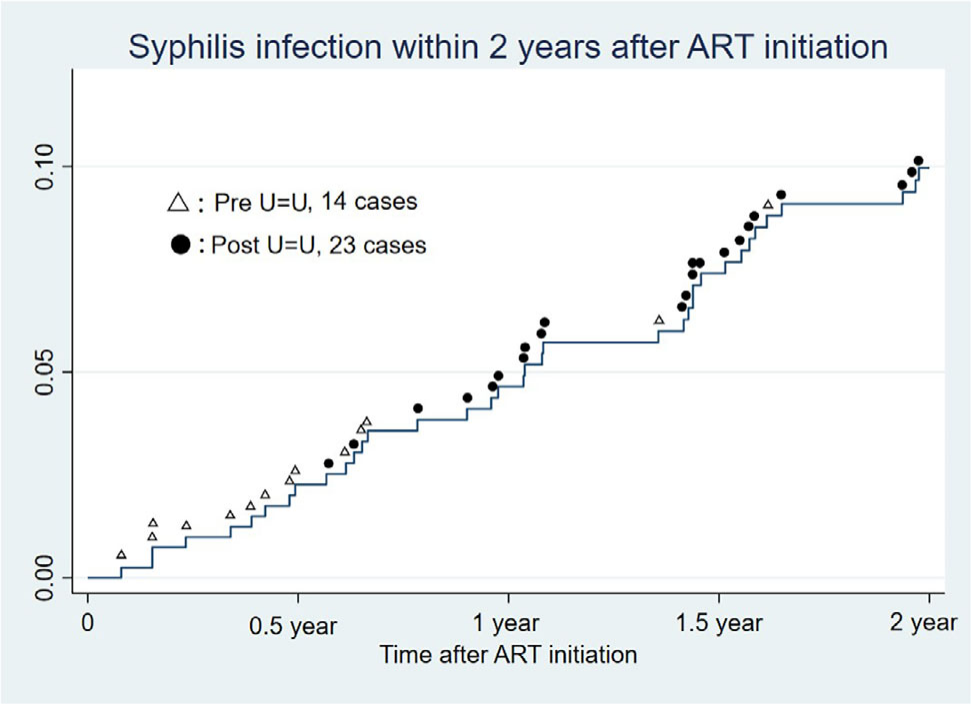
Syphilis infection within the first 2 years after ART initiation. Kaplan–Meier curve for cases of syphilis infection obsereved within the first 2 years after ART initiation (total *n* = 37). △ indicates cases of syphilis infection before achieving U═U (*n* = 14) and ● indicates cases of syphilis infection after achieving U═U (*n* = 23).

**Conclusions**: The incidence of syphilis was found to be consistently high after initial HIV treatment in Japan, regardless of the timing of U═U, suggesting the need for caution in sexual behaviour regardless of timing after HIV treatment.

### P363 Epidemiological study of the prevalence of STIs and HIV among men who have sex with men in the Russian Federation

Dmitrii Korenev ^1^, Kirill Barskiy^2^

^1^Steps, Moscow, Russian Federation. ^2^Public Health, Moscow, Russian Federation

**Introduction**: MSM are socially marginalized and highly stigmatized in Russia, even in the medical community. Tightening laws against LGBT+ people are the reason for the lack of real epidemiological data on the prevalence of diseases in this key group. In light of this, NGOs that promotes HIV care for marginalized populations decided that a research to address the topic was necessary.

**Methods**: The Foundation “Steps” and the Central Research Institute of Epidemiology carried out a biobehavioural study on a cohort of MSM in five regions of Russia in 2021. In addition to taking exams, participants completed a questionnaire. PCR investigations for the following STIs were conducted in samples obtained from three loci (oropharynx, rectum, and urethra): *N. gonorrhoeae*, *C. trachomatis*, *M. genitalium*, *T. vaginalis*, *T. pallidum*, herpes simplex virus type I (HSVI) and herpes simplex virus type II (HSVII). Serum enzyme‐linked immunosorbent assay (ELISA) tests (rapid testing) were also performed for syphilis, hepatitis B/C, and HIV.

**Results**: Six hundred and forty‐four MSM participated in the study. Male and female sexual partners were reported by 15% of the subjects. Participants who are not in committed relationships make up about 60% of the group. Only 34% of participants, meanwhile, consistently use condoms. Of the 158 (24.5%) individuals, 14 (8.9%) were found to be HIV‐positive during the study. STIs were found in 215 (33.4%) of the participants. Three‐quarters of the 325 cases of STIs detected were located in the rectum 166 (51%), the oral cavity 104 (32%), and the urethra 55 (17%). There was simultaneous detection of STIs at the three loci in 16% and 4% of HIV‐positive and HIV‐negative participants, respectively. Twenty percent of participants mentioned utilising psychoactive drugs or chemsex of which 33% were HIV positive and 16% HIV negative. The study revealed that substance abuse and HIV status are risk factors that more than double the likelihood of contracting an STI as shown in Table 1 and the predictor of acquiring an STI is presented in Figure 1.

**P363: Table 1:** Factors that increase a chance of having an STI

Predictors	Odds ratios	CL	*p*‐value
(Intercept)	0.16	0.12–0.23	<0.001
Sexparthm 1 (more 5)	1.89	1.25–2.88	0.003
Drug use (yes)	2.05	1.27–3.28	0.003
HIV (1)	2.33	1.52–3.56	<0.001
Observations	540		
R2Tjur	0.086		

Abbreviations: CL, confidence level; R2Tjur, coefficient of determination; Sexparthm, sex partners male.

**P363: Figure 1:**
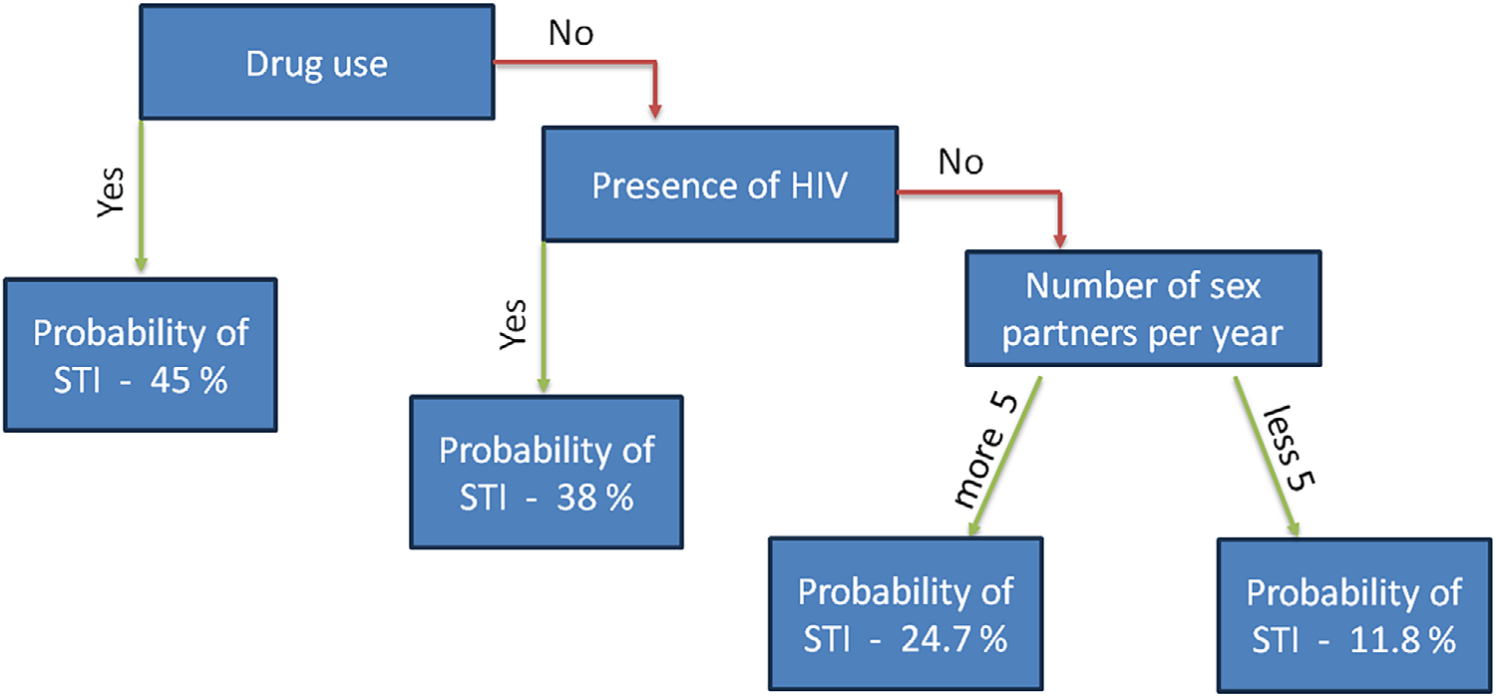
Predictors of having an STI.

**Conclusions**: The three main predictors of STIs among MSM were revealed: substance use, HIV status and number of sexual partners. Collaboration between NGOs and health institutions has been highly effective, and community‐based studies have shown unique results. Such practices also serve as tools for community integration.

### P364 *Neisseria gonorrhoeae* (NG) infection in MSM: clinical and microbiological characteristics—culture is essential but provides limited assistance

Cristina Gómez‐Ayerbe ^1^, María López‐Jódar^1^, Salvador Martin‐Cortes^1^, Marina Villalobos^1^, Isabel Ascensión Pérez‐Hernández^1^, María Victoria García‐López^2^, Rosario Palacios^1^, Jesús Santos^1^

^1^Infectious Unit, Hospital Universitario Virgen de la Victoria, Málaga, Spain. ^2^Microbiologia, Hospital Universitario Virgen de la Victoria, Málaga, Spain

**Introduction**: NG infection is the most frequent STI among MSM. Its clinical relevance is questioned in some scenarios, even suggesting the unnecessary screening of asymptomatic individuals [1]. The objective of our study is to characterize NG infection in a cohort of MSM who undergo regular STI screening.

**Materials and method**: All diagnosed NG infections in our Unit were analysed in a cohort of subjects on PrEP and PLHIV at risk of STIs from October 2023 to June 2024 undergoing regular STI screening. Cases without culture were excluded. STI screenings was performed by taking two swabs from each site (pharyngeal, rectal and urethral/urine). Samples were transported to the Microbiology Service in Amies transport medium (Deltalab^©^) and inoculated onto Martin‐Lewis agar. NG identification was performed using the Malditof system. Susceptibility testing of isolates was carried out using E‐test strips following European Committee on Antimicrobial Susceptibility Testing (EUCAST) recommendations. Real‐time PCR was also performed using the automated Cobas 4800 system (Roche CT/NG). Epidemiological and clinical characteristics were collected. Statistical programme: SPSS v25.

**Results**: Among the 362 NG infections with positive PCR assay, cultures were performed in 349 episodes occurring in 289 MSM, of which 215 were on PrEP, 65 were PLHIV, and nine MSM without PrEP. Mean age was 38.2 years (SD 10.5). Cultures were positive in 68 cases (19.4%). By site: rectal 56 out of 218 positive PCR, pharyngeal one out of 102, and urethral/urine 11 out of 29 (*p* < 0.001). 80.8% of episodes were asymptomatic, and 65 (18.6%) had concurrent STIs (chlamydia 44 and syphilis 17). In 41 episodes, subjects were on doxycycline post‐exposure prophylaxis (Doxy PEP). Positive cultures were not associated with symptoms, HIV infection, Doxy PEP status, or concurrent STIs. All isolates were sensitive to ceftriaxone, and 55.2% to tetracycline. No seroconversions occurred in subjects on PrEP during this period.

**Conclusions**: Most NG infections in MSM are asymptomatic. Cultures is not very effective in this scenario and is more frequently recovered from rectal or urethral samples. All isolates were sensitive to ceftriaxone, and half to tetracyclines. The transmissibility of a positive PCR assay but negative culture result, especially pharyngeal, should be investigated.

**Reference**

1. Kenyon C, Herrmann B, Hughes G, de Vries HJC. Management of asymptomatic sexually transmitted infections in Europe: towards a differentiated, evidence‐based approach. Lancet Health Reg Eur. 2023;34:100743.

### P365 Sexually transmitted infections in virologically suppressed people with HIV‐1: the secondary longitudinal analysis of the SPRINT study, a multi‐centre, randomised, active‐controlled phase III trial

Shenghua He^1^, Zhu Chen^2^, Yaokai Chen^3^, Min Wang^4^, Fujie Zhang^5^, Hao Wu^6^, Weiping Cai^7^, Ping Ma^8^, Qingxia Zhao^9^, Hongxia Wei^10^, Hongzhou Lu^11^, Hui Wang^12^, Wan Wan^13^, Heliang Fu^13^, Hong Qin ^13^

^1^Department of Infectious Diseases, Public Health Clinical Medical Center of Chengdu, Chengdu, China. ^2^Drug Clinical Trial Institution, Public Health Clinical Medical Center of Chengdu, Chengdu, China. ^3^Division of Infectious Diseases, Chongqing Public Health Medical Center, Chongqing, China. ^4^Institute of HIV/AIDS, The First Hospital of Changsha, Changsha, China. ^5^Clinical and Research Center for Infectious Diseases, Beijing Ditan Hospital Capital Medical University, Beijing, China. ^6^Clinical and Research Center for Infectious Diseases, Beijing Youan Hospital, Capital Medical University, Beijing, China. ^7^Infectious Disease Center, Guangzhou Eighth People's Hospital, Guangzhou Medical University, Guangzhou, China. ^8^Department of Infectious Diseases, Tianjin Second People's Hospital, Tianjin, China. ^9^Department of Infectious Diseases, The Sixth People's Hospital of Zhengzhou, Zhengzhou, China. ^10^Department of Infectious Diseases, The Second Hospital of Nanjing, Nanjing, China. ^11^National Clinical Research Centre for Infectious Diseases, The Third People' s Hospital of Shenzhen, The Second Affiliated Hospital of Southern University of Science and Technology, Shenzhen, China. ^12^Department of Infectious Diseases, Shenzhen Third People's Hospital, Shenzhen, China. ^13^Clinical Research & Development, Jiangsu Aidea Pharmaceutical Co., Ltd, Yangzhou, China

**Introduction**: People with HIV‐1 (PWH) are susceptible to sexually transmitted infections (STIs). We performed the secondary longitudinal analysis of the SPRINT study, a multi‐centre, randomized, active‐controlled phase III trial. This study compared efficacy and safety of switch to ainuovirine plus lamivudine and tenofovir DF and that to cobicistat‐boosted elvitegravir plus emtricitabine and tenofovir alafenamide. The secondary analysis aimed to evaluate baseline and post‐baseline STIs in virologically suppressed PWH.

**Methods**: PWH, aged 18–65 years, were enrolled if remaining virologically suppressed while on NNRTI‐based antiretroviral regimen for at least 12 months. Any participant would be excluded if presenting with active syphilis or concurrent viral hepatitis. Medical history and comedications were coded using MedDRA and WHODD, respectively. STIs of special interest included syphilis, gonorrhoea, genital herpes, urogenital mycoplasma or chlamydial infection and anogenital warts.

**Results**: A total of 762 participants were on switch therapy, and followed up at outpatient clinic visits for 48 weeks. The study population was at a median (Q1, Q3) age of 33.0 (28.0, 39.0) yr, with 97.1% being male. More than 99% of participants reported current practice of protective sex. Previous history of STIs included syphilis (21.9%), gonorrhoea (0.1%), genital herpes (0.1%), urogenital mycoplasma or chlamydial infection (0.1%) and anogenital warts (1.6%) (Figure 1). Serological test showed 16.5% of participants positive for syphilis (rapid plasma reagin) at baseline. Post‐baseline newly‐emerging or recurrent STIs included syphilis (2.5%), gonorrhoea (0.1%), genital herpes (0.1%), urogenital mycoplasma or chlamydial infection (0.1%) and anogenital warts (0.9%) (Figure 1). Secondary syphilis occurred in two participants post‐baseline (one per arm). Prior prescription of beta‐lactam antibiotics was common (5.2%), while on‐trial use of broad‐spectrum penicillins and third‐generation cephalosporins was common, 8.5% and 5.5%, respectively.

**P365: Figure 1:**
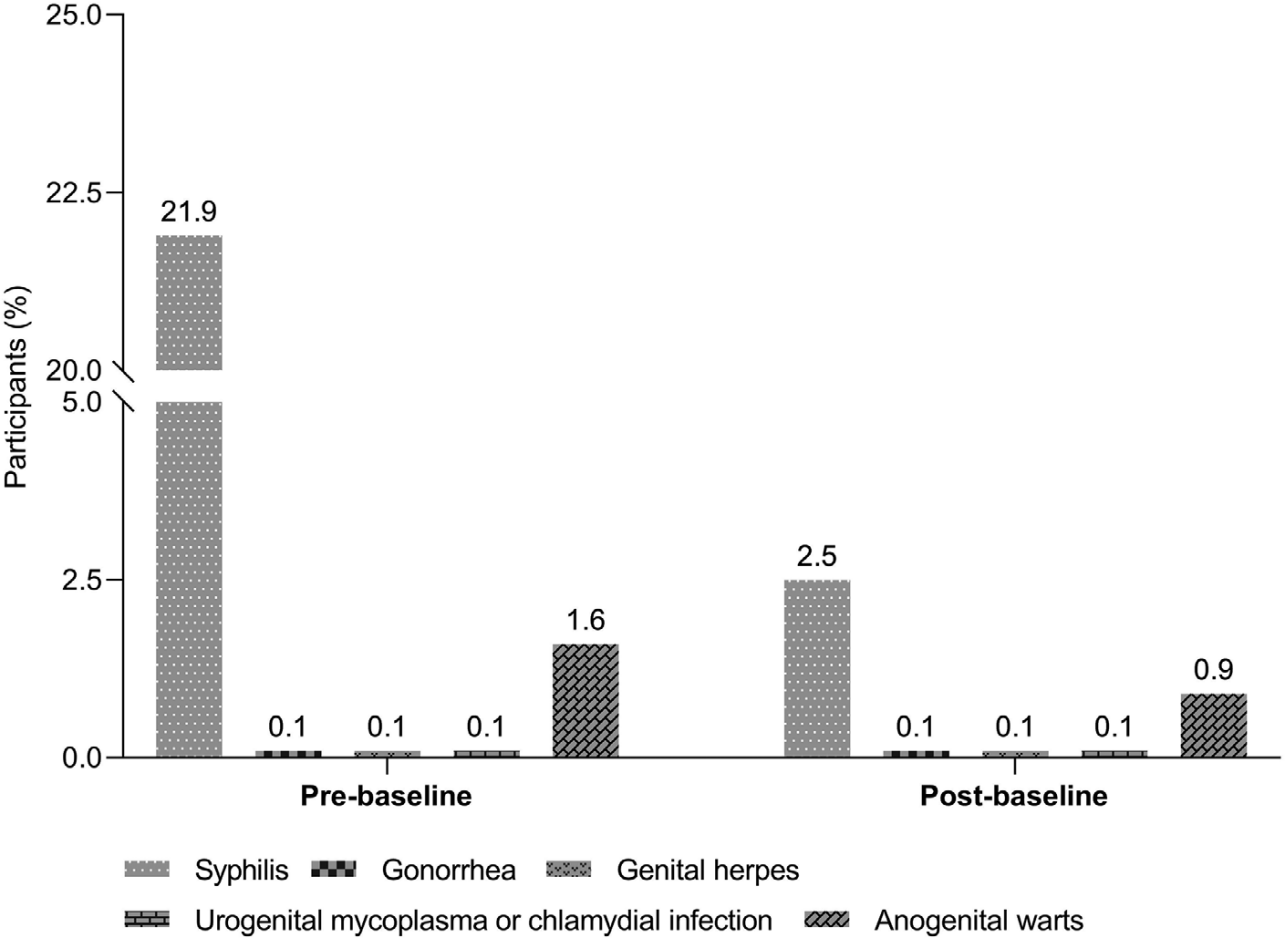
Proportions of participants with pre‐baseline and post‐baseline newly‐emerging sexually transmitted infections.

**Conclusions**: Syphilis is prevalent among virologically suppressed PWH. Other STIs are relatively less common probably due to inadequate testing and monitoring. Detection and multidisciplinary care of STIs is warranted in virologically suppressed PWH who are sexually active.

### P366 Malignant syphilis as the initial manifestation of HIV: a case report

Filippo Calandra Buonaura, Marco Visicaro, Antonella Santoro, Cristina Mussini

Infectious Diseases Clinic, Azienda Ospedaliero‐Universitaria di Modena, Modena, Italy

**Introduction**: Malignant syphilis (MS) is a rare form of secondary syphilis, more common among people living with HIV (PLWH) [1]. This report describes MS as the initial expression of HIV infection.

**Case report**: In December 2023, a 38‐year‐old man presented to the emergency department with a 30‐day history of painful erythematous skin lesions with papules, nodules, and crusts secreting serous‐haematic fluid. These lesions began on the lower limbs and spread to the trunk and upper limbs, with palmoplantar sparing (Figure 1). He also experienced fever, otalgia, and unilateral hearing loss. Blood tests showed pancytopenia, elevated liver enzymes, and increased C‐reactive protein. He reported recent condomless sexual intercourse. Sexually transmitted infection screening showed positive HIV‐1 serology with a viral load of 609,656 copies/mL, a CD4+ count of 107/mm^3^, and a CD4/CD8 ratio of 0.26. Serology for *Treponema pallidum* was also positive, with an rapid plasma reagin (RPR) of 1:128 and *T. pallidum* particle agglutination test (TPPA) of 1:40,960. Real‐time PCR resulted positive for *T. pallidum* on lesion biopsy, with histopathological examination consistent with MS [2]. The patient began antiretroviral therapy with BIC/TAF/FTC, along with cotrimoxazole prophylaxis. A lumbar puncture confirmed CNS involvement, leading to a 14‐day course of intravenous ceftriaxone (2 g/day), preceded by methylprednisolone to prevent a Jarisch–Herxheimer reaction (JHR). Skin lesions totally resolved after 4 months.

**P366: Figure 1:**
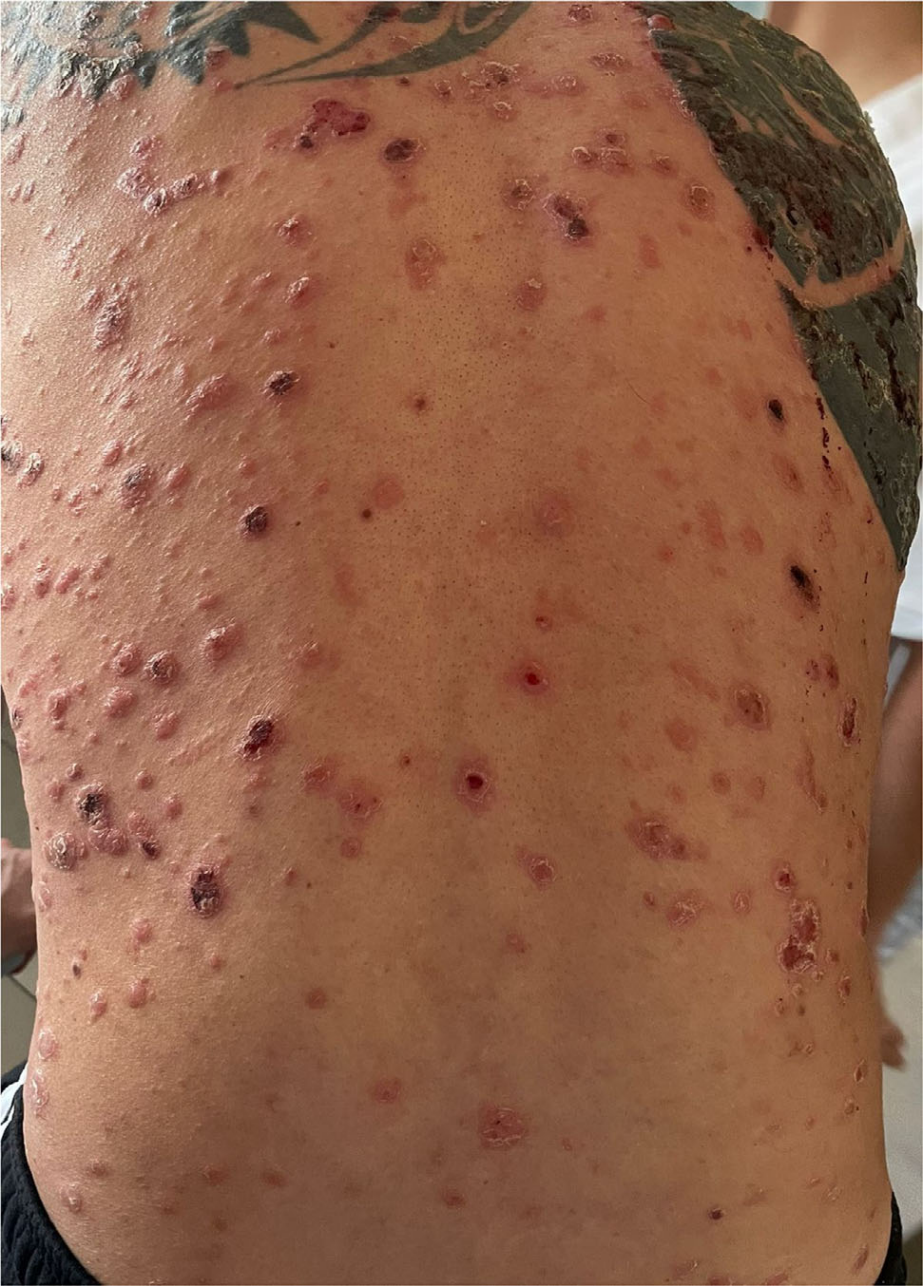
Skin lesions presentation at admission.

**Discussion**: Consistent with literature, this case met Fisher's criteria for MS except for JHR, due to corticosteroid use [3]. Fast diagnosis of MS is crucial to avoid complications, such as superinfection or permanent CNS damage. MS presentation should prompt HIV testing, as HIV increases the risk of developing MS by 60 times. Awareness of MS is essential due to its ability to mimic other conditions, particularly in PLWH with risk factors [4–6].

**Conclusion**: We described a rare case of MS. In HIV patients, syphilis can present atypically. Therefore, it is crucial to include syphilis testing in the differential diagnosis of skin lesions, since early identification is mandatory to prevent severe complications.

**References**

1. Bayramgürler D, Bilen N, Yildiz K, Sikar A, Yavuz M. Lues maligna in a chronic alcoholic patient. J Dermatol. 2005;32:217–9.

2. Cid PM, Cudós ES, Zamora Vargas FX, Beato Merino MJ, Herranz Pintoet P. Pathologically confirmed malignant syphilis using immunohistochemical staining: report of 3 cases and review of the literature. Sex Transm Dis. 2014;41:94–7.

3. Fisher DA, Chang LW, Tuffanelli DL. Lues maligna. Presentation of a case and a review of the literature. Arch Dermatol. 1969;99:70–3.

4. Tucker JD, Shah S, Jarell AD, Tsai KY, Zembowicz A, Kroshinsky D. Lues maligna in early HIV infection case report and review of the literature. Sex Transm Dis. 2009;36:512–4.

5. Sands M, Markus A. Lues maligna, or ulceronodular syphilis, in a man infected with human immunodeficiency virus: case report and review. Clin Infect Dis. 1995;20:387–90.

6. Samaan F. The great imitator: a rare case of Lues maligna in an HIV‐positive patient. Brown Hospital Medicine. 2023;2(2).

### P367 Syphilis profile in individuals on post‐exposure prophylaxis with doxycycline (Doxy PEP)

Rosario Palacios ^1^, María López‐Jódar^1^, Salvador Martin‐Cortés^1^, Andrea Prolo^1^, Isabel Ascensión Pérez‐Hernández^1^, Isabel Viciana^2^, Cristina Gómez‐Ayerbe^1^, Jesús Santos^1^

^1^Infectious Unit, Hospital Universitario Virgen de la Victoria, Málaga, Spain. ^2^Microbiology Department, Hospital Universitario Virgen de la Victoria, Málaga, Spain

**Introduction**: One limitation of Doxy PEP is its potential to alter the serological diagnosis of syphilis in infected individuals. The objective of our study was to analyse the characteristics of syphilis cases diagnosed in individuals enrolled in a Doxy PEP programme.

**Method**: Subjects enrolled in a Doxy PEP programme, similar inclusion criteria as on IPERGAY and DoxiPEP were included [1, 2]. The diagnosis of syphilis was made following commonly accepted criteria. Adherence to Doxy was defined as poor <1 pill a week, and regular <2 pills. Study period: March 2023 to June 2024.

**Results**: During this period, six cases of syphilis were diagnosed among the 240 subjects included in Doxy PEP (prevalence rate 2.5%, 95% CI 0.52–4.48%). All were MSM, five on PrEP and one PLHIV. Four had a history of syphilis. Baseline rapid plasma regain (RPR) at the beginning of the Doxy PEP programme: five negative and one at 1/4. There were two cases of secondary syphilis, and four latent syphilis with minimal RPR increase. In four cases, adherence to Doxy was poor/regular. All subjects responded well to a single dose of benzathine penicillin G, with RPR negativization in all cases (Table 1).

**P367: Table 1:** Characteristics of syphilis case

Case	Age	PEP date	Syphilis date	RPR at PEP date	RPR at syphilis	Clinical	Other STIs	Adherence	Response
1	41	05/10/23	08/04/23	Neg	8	Latent	NG^b^	Regular	Yes
2	34	05/18/23	08/10/23	4	32	Latent	No	Poor	Yes
3	37	06/28/23	08/08/23	Neg	4	Latent	No	Regular	Yes
4	44	07/0723	04/12/24	Neg	4	Secondary	NG^b^	Good	Yes
5	49	07/02/23	02/22/24	Neg	2	Secondary^a^	No	Good	Yes
6	47	08/01/23	11/30/23	Neg	8	Latent	No	Regular	Yes

^a^ Cutaneous lesions and genital chancre with positive polymerase chain reaction (PCR) assay for *Treponema pallidum* (TP);

^b^ *Neisseria gonorrhoeae*.

**Conclusions**: In real‐world settings, the prevalence rate of syphilis in individuals in a Doxy PEP programme is low. Most cases are asymptomatic, and the minimal RPR elevation is notable. Response to benzathine penicillin G is adequate. Proper adhesion to Doxy may be a key factor in reducing the incidence of syphilis.

**References**

1. Molina JM, Charreau I, Chidiac C, Pialoux G, Cua E, Delaugerre C, et al. Post‐exposure prophylaxis with doxycycline to prevent sexually transmitted infections in men who have sex with men: an open‐label randomized substudy of the ANRS IPERGAY trial. Lancet Infect Dis. 2018;18(3):308–17.

2. Luetkemeyer AF, Donnell D, Dombrowski JC, Cohen S, Grabow C, Brownet CE, et al. Postexposure doxycycline to prevent bacterial sexually transmitted infections. N Engl J Med. 2023;388(14):1296–306.

### P368 Acceptability and real‐life experience of doxyPEP in MSM at a combined HIV/PrEP clinic in a Southeast Asian university hospital

Dariusz Olszyna ^1^, Eon Tat Gan^2^, Tiane Le^1^, Sophia Archuleta^1^

^1^Medicine, National University Hospital, Singapore, Singapore. ^2^Pharmacy, National University Hospital, Singapore, Singapore

**Introduction**: Bacterial sexually transmitted infections (STIs) have been consistently rising among MSM. In Singapore, comprehensive STI screening can cost in excess of GBP200 (EUR240) and its uptake remains low. Recent studies showed a single 200‐mg dose of doxycycline (doxyPEP) to be effective in preventing syphilis, chlamydia and to a lesser extent gonorrhoea in MSM and transgender women [1]. We hypothesized that discussing doxyPEP with MSM at high risk of bacterial STIs could encourage STI screening.

**Materials and methods**: Between 1 January and 14 June 2024, 121 (HIV positive or HIV negative MSM on PrEP) were screened for doxyPEP eligibility. Those who met one or more of the following criteria were offered doxyPEP: bacterial STI within last 12 months; condomless anal sex outside a monogamous relationship; participation in threesomes or groupsex; attendance of circuit parties. Clinicians provided patients with information about doxyPEP, including efficacy, safety, potential concerns, and information on how to use it. Before initiating doxyPEP, patients were required to undergo full STI screening (blood test for syphilis and chlamydia/gonorrhoea PCR in appropriate exposure sites).

**Results**: Thirty‐two patients met eligibility criteria and were offered doxyPEP. Twenty‐eight (88%) patients accepted and four (12%) declined. Twenty (71%) of those who accepted doxyPEP, completed STI screening and were given a doxycycline prescription. Among 20 patients screened, eight were positive for an STI, five of whom had multiple STIs. Although our clinic offers full STI screening every 6–12 months, none of the patients diagnosed with STIs before starting doxyPEP, had undergone such testing in the past 6 or 12 months.

**Conclusions**: Real‐life experience in our clinic shows high acceptability of doxyPEP among carefully pre‐selected MSM at high risk of bacterial STIs. Discussion of doxyPEP can lead to high levels of detection of STIs in MSM who previously may have declined STI screening. DoxyPEP may not only be a tool to prevent future STIs in MSM, but also an opportunity to detect existing ones in MSM who otherwise would not be screened. Our experience highlights importance of STI screening before starting doxyPEP.

**Reference**

1. Luetkemeyer AF, Donnell D, Dombrowski JC, Cohen S, Grabow C, Brown CE, et al. Postexposure doxycycline to prevent bacterial sexually transmitted infections. N Engl J Med. 2023;388(14):1296–306.

## People living with HIV and tuberculosis

### P369 Long‐term efficacy of tuberculosis preventive treatment in people living with HIV: a single centre prospective cohort study

Napon Hiranburana ^1^, Haymar Su Lwin^1^, Jiratchaya Sophonphan^1^, Win Min Han^1^, Chuleeporn Khongpetch^2^, Anchalee Avihingsanon^2^

^1^Research, HIV Netherlands Australia Thailand Research Collaboration (HIV‐NAT), Thai Red Cross AIDS Research Centre, Bangkok, Thailand. ^2^Research, HIV Netherlands Australia Thailand Research Collaboration (HIV‐NAT), Bangkok, Thailand

**Introduction**: As a high tuberculosis (TB) prevalence country, the current national Thai guidelines recommend tuberculosis preventive treatment (TPT) for people with HIV (PWH) who have a history of TB exposure, low CD4 count, or positive interferon‐gamma release assay (IGRA)/tuberculin skin test (TST) tests. We investigated the long‐term efficacy of TPT in this specific population.

**Methods**: We retrospectively analysed the data of PWH aged ≥18 years who were on antiretroviral therapy (ART) at the HIV‐NAT long term cohort in Bangkok, Thailand during 1996 and 2023. The study aimed to investigate the incidence of new TB infections after initiating ART. PWH were categorized into three groups: those who voluntarily received TPT with isoniazid 9 months (9H), those who received a combination of isoniazid and rifapentine (1HP/3HP), and those who did not receive TPT.

**Results**: Out of 1909 participants with active follow‐up status, 142 who had active TB before starting ART were excluded. Among the remaining 1767 participants, 1031 (58.3%) did not receive TPT, 85 (4.8%) received isoniazid, and 651 (36.8%) received the combination of isoniazid and rifapentine (as shown in Figure 1). The median CD4 count (cells/mm^3^) at ART initiation for no TPT, 9H and 1HP/3HP group were 268 (IQR 163–384), 304 (IQR 170–403) and 308 (IQR 199–444), respectively. The median of duration of ART (years) for no TPT, isoniazid and isoniazid and rifapentine group was 16.7 (IQR 7.1–23.5), 24 (IQR 18.5–27.2) and 3.8 (IQR 1.9–4.6), respectively. The probability of TB incidence was higher in the no TPT group (7.5%), compared to 9H group (2.4%) and 1HP/3HP (0%) (log rank *p* < 0.001). The TB incidence for no TPT, isoniazid and isoniazid and rifapentine group was 428.3, 105.7 and 0 per 100,000 person‐years respectively (as shown in Table 1).

**P369: Figure 1:**
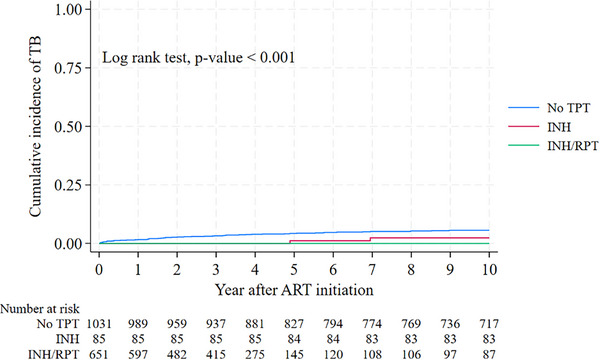
Cumulative incidence of TB in different groups over time, following the initiation of antiretroviral therapy (ART).

**P369: Table 1:** The number of individuals who developed tuberculosis (TB) and the rate of TB onset, along with 95% confidence intervals (CI)

			Incidence TB (100,000/person year)		
	Person year	Number of TB onset	Incidence rate	95% CI	
No TPT	15878.3	68	428.3	337.7	543.2
INH	1891.4	2	105.7	26.4	422.8
1HP/3HP	3128.2	0	0	−	−

**Conclusion**: The study highlights a significant reduction in TB incidence among PWH who received TPT, especially with the combination of isoniazid and rifapentine. This supports the recommendation for TPT to prevent TB infection in PWH. Further follow‐up time in the HP group is needed to demonstrate efficacy.

### P370 Impact of injection drug use on tuberculosis outcomes in people with HIV: a nationwide cohort study

Naila Bugyi‐Bozo ^1^, Josep M. Llibre^2^, Iben Ørsted^3^, Nina Breinholt Stærke^4^, Johanna Åhsberg^5^, Gitte Kronborg^6^, Steffen Leth^7^, Lars Haukali Omland^8^, Niels Obel^8^, Line Dahlerup Rasmussen^1^, Isik Somuncu Johansen^1^, Raquel Martin‐Iguacel^1^

^1^Department of Infectious Diseases, Odense University Hospital, Odense, Denmark. ^2^Infectious Diseases Department and Fight Infections Foundation, University Hospital Germans Trias i Pujol, Badalona, Spain. ^3^Department of Infectious Diseases, Aalborg University Hospital, Aalborg, Denmark. ^4^Department of Infectious Diseases, Aarhus University Hospital, Aarhus, Denmark. ^5^International Reference Laboratory of Mycobacteriology, Statens Serum Institut, Copenhagen, Denmark. ^6^Department of Infectious Diseases, Copenhagen University Hospital, Amager Hvidovre Hospital, Hvidovre, Denmark. ^7^Department of Infectious Diseases & Internal Medicine, Gødstrup Hospital, Herning, Denmark. ^8^Department of Infectious Diseases, Copenhagen University Hospital, Copenhagen, Denmark

**Introduction**: Tuberculosis (TB) significantly impacts morbidity and mortality among people with HIV (PWH), particularly people with injection drug use (IDU). Factors like poor treatment adherence and social instability play a significant role. In European countries with low TB incidence, understanding the differential treatment outcomes in PWH with IDU is crucial for tailored interventions. We aimed to investigate the impact of IDU on TB treatment completion and mortality rates (MR) among PWH in Denmark.

**Materials and methods**: In this nationwide, population‐based cohort study, we included PWH aged ≥18 years from the Danish HIV Cohort (1995–2017) who developed TB during the study period. Medical records were reviewed by physicians. We compared clinical and TB characteristics and treatment outcomes between PWH with and without IDU (Table 1). We used Poisson regression to estimate all‐cause and TB‐related MR and mortality rate ratio (MRR). Social burden was defined as homelessness, stay in shelter or prison facilities and/or prostitution.

**P370: Table 1:** Baseline characteristics

	HIV/TB without IDU *N* = 182	HIV/TB with IDU, *n* = 35	*p*‐value
Male, *n* (%)	104 (57.1)	23 (65.7)	0.35
Age at TB diagnosis, median years (IQR)	37 (31‐45)	42 (34‐47)	0.05
Route of HIV transmission			
MSM, *n* (%)	30 (16.5)		
Heterosexual, *n* (%)	127 (69.8)		
IDU, *n* (%)		29 (24.6)	
Unknown, *n* (%)	25 (13.7)		
Caucasian, n (%)	50 (28.1)	31 (91.2)	<0.0001
Region of origin			<0.001
Denmark	43 (23.6)	27 (77.1)	
Greenland	6 (3.3)	<3	
Africa/Asia	115 (63.2)	3 (8.6)	
Other	15 (8.2)	<3	
HCV, *n* (%)	26 (14.3)	28 (80)	<0.0001
HBV, *n* (%)	14 (7.7)	5 (14.3)	0.21
HIV diagnosis before 1995, *n* (%)	24 (13.2)	14 (40.0)	<0.0001
Emigration during the study period, *n* (%)	13 (7.1)	0	0.103
Non‐TB AIDS before TB diagnosis, *n* (%)	30 (16.5)	3 (8.6)	0.23
Time of TB diagnosis			
Concomitant HIV/TB diagnosis^a^	92 (50.6)	7 (20.0)	0.001
Subsequent TB diagnosis^b^	90 (49.5)	28 (80.0)	
HIV infection			
CD4+T count at TB diagnosis, median cells/µl (IQR)	147 (62‐370)	197 (84‐440)	0.35
HIV VL <50 copies/ml at TB diagnosis	42 (23.1)	9 (25.7)	0.74
On ART at TB diagnosis, *n* (%)	50 (27.5)	11 (31.4)	0.63
Comorbidity			
Charlson comorbidity score at TB diagnosis			
0	147 (80.8)	11 (31.4)	<0.0001
1	19 (10.4)	17 (48.6)	<0.0001
2–5	15 (8.2)	6 (17.1)	0.10
≥6	<3	<3	0.19
Social burden^c^	15 (8.2)	27 (77.1)	<0.0001
Delay in TB diagnosis			
Patient delay (days, IQR)	35 (14‐73)	21 (14‐30)	0.16
Doctor delay (days, IQR)	10 (3‐28)	11 (3‐30)	0.77
TB diagnosis based on:			0.63
Microbiology (PCR or culture)	149 (82.8)	31 (88.6)	
Histopathology	29 (16.1)	4 (11.4)	
Clinical suspicion	<3	0	
Resistance			
Any resistance	9 (5.0)	5 (14.3)	0.10
Multidrug resistance	<3	<3	−
TB localization			
Pulmonary	79 (43.3)	23 (65.7)	
Extrapulmonary	43 (23.6)	4 (11.4)	
Disseminated	60 (33.0)	8 (22.9)	
Treatment outcome^d^			<0.0001
Cure	5 (2.8)	0	
Treatment completed	166 (91.2)	21 (60.0)	
Treatment failure	0	0	
Treatment interrupted	4 (2.2)	6 (5.1)	
Relapse	13	5	

^a^ Concomitant TB/HIV: TB diagnosed within 3 months after HIV diagnosis.

^b^ subsequent TB: TB diagnosed 3 months after HIV diagnosis.

^c^ social burden defined as homelessness, stay in shelter or prison facilities and/or prostitution.

^d^ outcome for TB treatment according to WHO guidelines.

**Results**: Two hundred and seventeen PWH developed TB during the study period, and 35 (16.1%) had a history of IDU. No significant differences were found in CD4+ T counts or viral suppression at TB diagnosis, microbiological confirmation of TB diagnosis or delays in diagnosis by patients or doctors between both groups. However, the IDU group exhibited higher rates of social burden (77.1% vs 8.2%, *p* < 0.001), higher Charlson comorbidity scores, and higher rates of pulmonary TB (65.7% vs 43.3%, *p* = 0.048). Treatment completion rates were significantly lower for IDU (60.0% vs 94%, *p* < 0.001). All‐cause MR in IDU was 165.3/1000 person‐years (95% CI 110.8–246.6; *n* = 24) compared to 17.0/1000 person‐years (95% CI 11.9–24.1; *n* = 31) in non‐IDU (MRR 9.75 [95% CI 5.72–16.61]) (Figure 1). TB‐related MR was also higher for IDU (86.7 [95% CI 45.1–166.6] vs 5.5 [95% CI 2.6–11.6], MRR 15.7 [95% CI 5.9–42.2]).

**P370: Figure 1:**
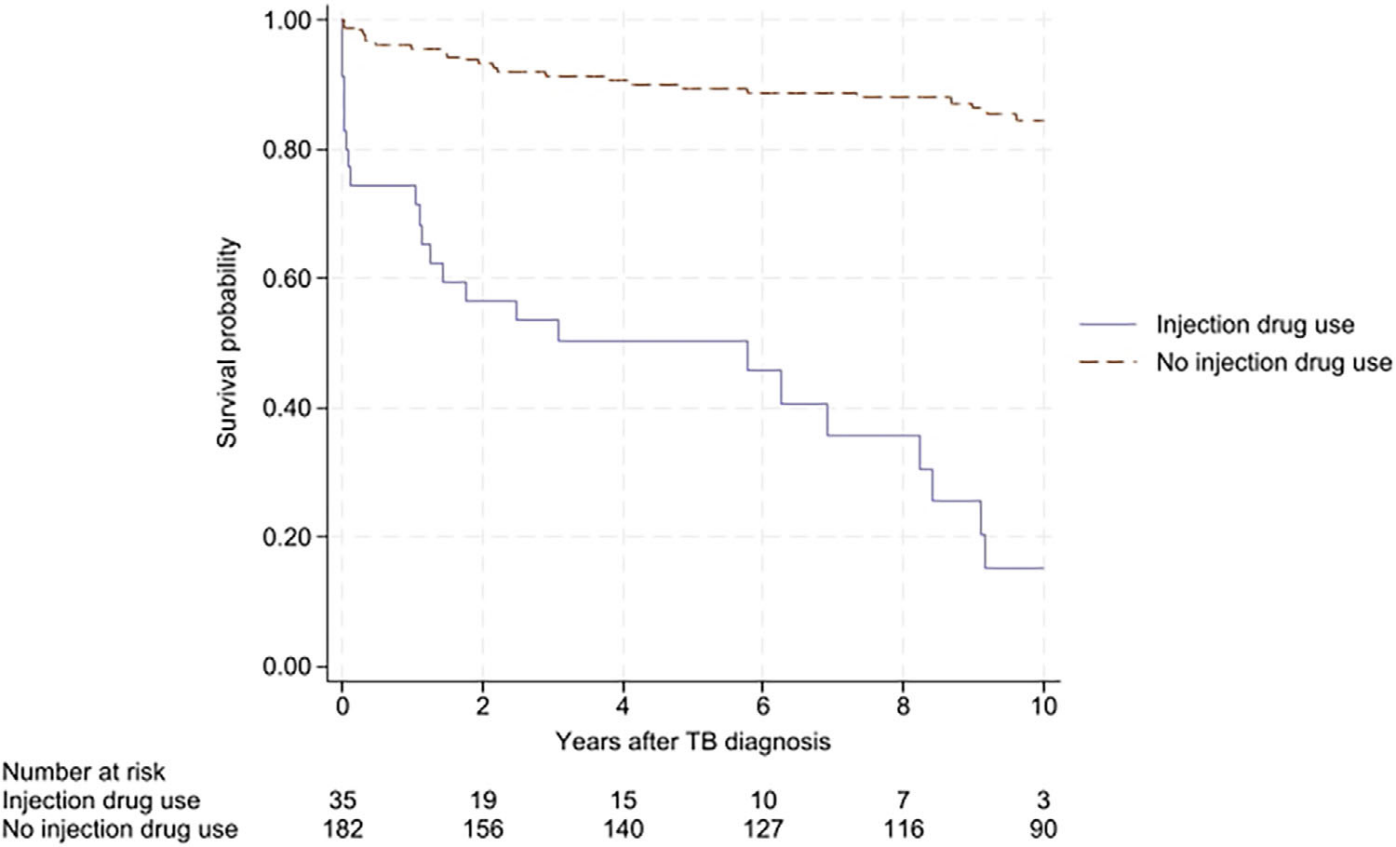
Survival probability after TB diagnosis.

**Conclusion**: In Denmark, a country with low TB incidence, TB treatment completion rates among PWH were significantly lower for those with a history of IDU compared to non‐IDU. TB‐related MR were 15 times higher among the IDU group. These findings underscore the urgent need for improved TB management strategies, such as shortened treatment courses, integrated care programmes and enhanced social support for PWH with IDU and TB to enhance treatment outcomes and reduce mortality.

### P371 Evaluating tuberculosis prevalence in key populations with advanced HIV diseases in Nigeria

Mark Akhigbe ^1^, Roger Abang^2^, Abiye Kalaiwo^3^, Paul Umoh^2^

^1^KP CARE 1 Project, Heartland Alliance Nigeria, Uyo, Nigeria. ^2^Programs Department, Heartland Alliance Nigeria, Abuja, Nigeria. ^3^Programs Department, United States Agency for International Development (USAID), Abuja, Nigeria

**Introduction**: The dual epidemic of TB and HIV remains a significant public health challenge globally, particularly in regions with high HIV prevalence. Nigeria, a region ranking fourth worldwide in terms of HIV burden, faces a generalized HIV epidemic. The country has approximately 1.9 million people living with HIV (PLHIV), and it accounted for 38% of new HIV infections in the West and Central African sub‐region. Addressing the co‐morbidity of TB and advanced HIV disease (AHD) is imperative for improving health outcomes and reducing mortality. This study aims to evaluate the prevalence of TB among key populations (KP) such as men who have sex with men (MSM), people who inject drugs (PWID), and female sex workers (FSW) living with AHD.

**Methods**: To evaluate TB prevalence in KP with AHD, we conducted a retrospective analysis. The study population consisted of clients diagnosed with AHD, defined by CD4 counts below 200 cells/mm^3^ or presenting with an AIDS‐defining condition based on World Health Organization guideline and tested for TB using the lipoarabinomannan assay (TB‐LAM), data was extracted from electronic medical records (EMR) of 19 One Stop Shops (OSS) under the United States Agency for International Development (USAID)‐funded KP CARE 1 project between 2022 to March 2024.

**Results**: Out of the 22,686 PLHIV that were analysed, 2% (276 of 22,686) were diagnosed with AHD having a CD4 <200 cells/mm^3^. The overall prevalence of TB among those who had AHD was 27% (66/241). A deep dive among key populations subgroup shows that, FSW who tested positive for TB, accounted for 58% (38/66), MSM accounted for 30% (20/66), PWID 8% (5/66) and transgender 5% (3/66) of the total positive cases.

**Conclusions**: This study demonstrates a substantial burden of TB among key populations with AHD. Targeted TB screening strategies are crucial for early diagnosis and treatment within these high‐risk groups especially the FSW as more studies will be needed to further understand this. Integrating TB screening into routine HIV care for key populations can significantly improve TB control efforts. In addition, promoting early ART initiation remains critical in reducing their susceptibility to TB.

### P372 Abstract withdrawn

## People living with HIV and viral hepatitis

### P373 Bictegravir/emtricitabine/tenofovir alafenamide (B/F/TAF) in treatment‐naïve people with both HIV‐1 and hepatitis B: 3‐year outcomes from ALLIANCE

Anchalee Avihingsanon ^1^, Hongzhou Lu^2^, Chee Loon Leong^3^, Chien‐Ching Hung^4^, Sasisopin Kiertiburanakul^5^, Man‐Po Lee^6^, Khuanchai Supparatpinyo^7^, Sharline Madera^8^, Hongyuan Wang^9^, Jason Hindman^10^, Taisheng Li^11^

^1^HIV Netherlands Australia Thailand Research Collaboration (HIV‐NAT), Thai Red Cross AIDS Research Centre, Bangkok, Thailand. ^2^National Clinical Research Center for Infectious Disease, Shenzhen Third People's Hospital, Shenzhen, China. ^3^Department of Medicine, Kuala Lumpur General Hospital, Kuala Lumpur, Malaysia. ^4^Division of Infectious Diseases, Department of Internal Medicine, National Taiwan University Hospital Yunlin, Yunlin, Taiwan. ^5^Faculty of Medicine, Ramathibodi Hospital, Mahidol University, Bangkok, Thailand. ^6^Department of Medicine, Queen Elizabeth Hospital, Kowloon, Hong Kong. ^7^Department of Medicine, Chiang Mai University, Chiang Mai, Thailand. ^8^Virology, Gilead Sciences, Inc., Foster City, CA, USA. ^9^Clinical Data Science, Gilead Sciences, Inc., Foster City, CA, USA. ^10^Clinical, Gilead Sciences, Inc., Foster City, CA, USA. ^11^Infectious Diseases Department, Peking Union Medical College Hospital, Beijing, China

**Introduction**: The ALLIANCE study showed that bictegravir/emtricitabine/tenofovir alafenamide (B/F/TAF) was non‐inferior to dolutegravir + emtricitabine/tenofovir disoproxil fumarate at achieving HIV‐1 RNA suppression, and superior at achieving HBV DNA suppression at week 48 in treatment‐naïve adults with both HIV‐1 and hepatitis B virus (HBV), with high rates of HIV‐1 and HBV suppression observed at week 96 [1]. Here, we report longer‐term efficacy and safety of B/F/TAF in adults with HIV‐1 and HBV through 3 years of treatment.

**Materials and methods**: ALLIANCE (NCT03547908) was a randomized, double‐blind, active‐controlled phase III clinical study. This analysis reports data from participants who had received B/F/TAF in the 96‐week randomization phase, plus 48 weeks of open‐label extension (participating countries only). Baseline demographics and clinical characteristics, HIV‐1 suppression (HIV‐1 RNA <50 copies/mL), HBV suppression (HBV DNA <29 IU/mL), alanine aminotransferase (ALT) normalization (2018 American Association for the Study of Liver Diseases criteria), hepatitis B e antigen (HBeAg) and surface antigen (HBsAg) loss/seroconversion at week 144, and treatment‐emergent adverse events (TEAEs) through to end of study (EOS) are reported.

**Results**: At baseline, most participants receiving B/F/TAF (*N* = 121) were male at birth (92.6%) and Asian (89.3%), with a median (range) age of 31 (19–65) years; 49.6% had ALT levels above the upper limit of normal and 76.0% were HBeAg positive. In total, 106 participants received B/F/TAF through week 144. High levels of HIV‐1 RNA (99.0%) and HBV DNA (80.2%) suppression were maintained after 144 weeks of treatment (Table 1). ALT normalization was maintained and HBeAg and HBsAg loss/seroconversion continued through week 144, indicating sustained anti‐HBV activity (Table 1). Safety data through to EOS are shown (Table 1). By EOS, 8.3% of participants had discontinued treatment prematurely.

**P373: Table 1:** Virological and immunological outcomes at week 144 and safety outcomes through end of study in participants randomized to B/F/TAF

*n*/*N* (%)	B/F/TAF (*N* = 121)
**Virological and immunological outcomes at week 144**	
HIV‐1 RNA <50 copies/ml	104/105 (99.0)
HBV DNA <29 IU/ml	85/106 (80.2)
HBeAg loss^a^/seroconversion^b^	26/58 (44.8)/17/58 (29.3)
HBsAg loss^a^ / seroconversion^b^	16/71 (22.5)/11/71 (15.5)
ALT normalization (2018 AASLD criteria)^c^	41/52 (78.8)
**Safety up to week 144**	
≥1 study drug‐related TEAE^d^	39/121 (32.2)
≥1 study drug‐related TEAE leading to premature discontinuation	1/121 (0.8)^e^
≥1 serious TEAE	20/121 (16.5)
Death	3/121 (2.5)

*Note*: Safety outcomes were assessed in the safety analysis set (*N* = 121), which included all randomly assigned participants who received ≥1 dose of study drug. All virological and immunological outcomes are from a missing = excluded analysis; all except HBeAg and HBsAg loss/seroconversion were assessed in the full analysis set (*N* = 119), which included all randomized participants who received ≥1 dose of study drug and had ≥1 post‐baseline HIV‐1 RNA or HBV DNA result while on study drug. The serologically evaluable full analysis set, defined as all participants in the full analysis set who were HBe/sAg positive and HBe/sAb negative or missing at baseline, was used for assessment of HBsAg and HBeAg loss/seroconversion. The denominator is the number of participants with non‐missing data for the endpoint at the week 144 visit.

Abbreviations: AASLD, American Association for the Study of Liver Diseases; ALT, alanine aminotransferase; B/F/TAF, bictegravir/emtricitabine/tenofovir alafenamide; HBeAg, hepatitis B e antigen; HBsAg, hepatitis B surface antigen; HBV, hepatitis B virus; TEAE, treatment‐emergent adverse event; ULN, upper limit of normal.

^a^ Changes from HBe/sAg positive at baseline to negative at a post‐baseline visit with HBe/sAb negative or missing at baseline.

^b^ HBe/sAg loss and HBe/sAb changes from negative or missing at baseline to positive at a post‐baseline visit.

^c^ Reduction in ALT to ULN at baseline based on 2018 AASLD criteria, where ULN is 25 U/L for females and 35 U/L for males.

^d^ Weight increase was the most commonly reported study drug‐related TEAE (7.4%), followed by abnormal weight gain, ALT increases, dyslipidaemia, and headache (all 3.3%).

^e^ Hepatocellular carcinoma.

**Conclusions**: At year 3, B/F/TAF maintained high rates of HIV‐1 and HBV virological suppression, with favourable HBV treatment outcomes and HBsAg and HBeAg loss/seroconversion persisting into year 3. Safety findings through 3 years were consistent with the established profile of B/F/TAF; most TEAEs were mild to moderate, and rates of study drug discontinuation were low. These results further support the longer‐term use of B/F/TAF in people with both HIV‐1 and HBV.

**Reference**

1. Avihingsanon A, Lu H, Leong CL, Hung C‐C, Koenig E, Kiertiburanakul S, et al. Bictegravir, emtricitabine, and tenofovir alafenamide versus dolutegravir, emtricitabine, and tenofovir disoproxil fumarate for initial treatment of HIV‐1 and hepatitis B coinfection (ALLIANCE): a double‐blind, multicentre, randomized controlled, phase 3 non‐inferiority trial. Lancet HIV. 2023;10(10):e640–52.

### P374 Long‐term outcomes of people living with HCV/HIV with advanced hepatic fibrosis treated with direct‐acting antivirals: a retrospective study

João Matos, Inês Caetano, Flavia Faria, Francisco Novela, António Ludgero Vasconcelos, Josefina Méndez

Infectious Diseases, Centro Hospitalar Universitário de Santo António, Porto, Portugal

**Introduction**: HIV infection significantly impacts the clinical progression of hepatitis C virus (HCV) infection. Direct‐acting antivirals (DAA) have revolutionized HCV treatment with outstanding success rates [1]. Nonetheless, long‐term follow‐up data, particularly in people living with HCV/HIV (PLWHIV/HCV) and advanced hepatic fibrosis (F3/F4), remain limited [2]. This study aims to evaluate liver disease status and overall survival in this population 5 years post‐DAA treatment.

**Materials and methods**: A retrospective, observational, single‐centre study was conducted. PLWHIV/HCV were included if presenting with F3/F4 stage at the time of DAA treatment and subsequently achieved sustained virological response (SVR). Clinical data were collected at baseline and 5 years post‐treatment, or at the last follow‐up for those lost to follow‐up (LTF).

**Results**: A total of 234 patients was included, with a median age of 47.3 years. The cohort comprised 88.9% males and 11.1% females. Portuguese nationality represented most patients (96.6%). Modes of HIV/HCV acquisition included 95.7% people who inject drugs and 4.3% via sexual transmission. HIV infection was also characterized: 94.9% had undetectable RNA, and 88.5% had CD4 counts >200/mm^3^ (mean of 582/mm^3^). Mean baseline aspartate aminotransferase to platelet ratio index (APRI) and fibrosis‐4 index (FIB‐4) scores were 1.40 and 3.08, respectively, with 20.5% having an APRI score >2 and 33.3% a FIB‐4 score >3.25. Mean Fibroscan score was 20.0 kPa, with 40.2% having scores >10 but <13 kPa, and 59.8% >13 kPa. Over the 5‐year follow‐up, HCV reinfection occurred in 3.0% of patients. Regarding HIV infection, 90.2% (*p* = 0.082) had undetectable RNA, and the percentage of patients with CD4 counts >200/mm^3^ remained unchanged (mean of 597/mm^3^; *p* = 0.447). Mean 5‐year APRI and FIB‐4 scores were 0.97 (*p* = 0.433) and 2.09 (*p* < 0.001), respectively, with 1.7% (*p* < 0.001) having an APRI score >2 and 9.4% (*p* < 0.001) a FIB‐4 score >3.25. Mean Fibroscan score was 12.0 kPa (*p* < 0.001), with 61.7% having scores <10 kPa, 11.2% >10 but <13 kPa, and 27.1% >13 kPa. Outcomes reported that 57.3% of patients were stable without cirrhosis, 30.8% had cirrhosis, 1.7% developed hepatocarcinoma, 0.4% underwent liver transplant, and 9.8% deceased (17.4% of hepatic causes).

**Conclusions**: This study highlights the long‐term effectiveness of DAAs in improving liver status disease outcomes in PLWHIV/HCV with advanced hepatic fibrosis at baseline.

**References**

1. European Association for the Study of the Liver. EASL recommendations on treatment of hepatitis C: final update of the series. J Hepatol. 2020;73(5):1170–218.

2. European AIDS Clinical Society. EACS Guidelines version 12.0: pp. 127–32 [Internet]. 2023 [cited 2024 Jul 26]. Available from: https://www.eacsociety.org/media/guidelines‐12.0.pdf.

### P375 Abstract withdrawn

### P376 Acute/recent infections and reinfections by HCV in MSM with and without HIV in the region of Madrid (ATHENS study)

Pablo Ryan^1^, Juan Berenguer ^2^, Luis Ramos Ruperto^3^, Mar Vera^4^, Leire Pérez‐Latorre^2^, Ignacio De los Santos^5^, Adriana Pinto^6^, Santos Del Campo^7^, Eva Orviz^8^, Beatriz Álvarez‐Álvarez^9^, José Sanz^10^, Pilar Ruiz‐Seco^11^, Rafael Torres^12^, Beatriz Brazal^13^, Beatriz López‐Centeno^14^, José M Bellón^2^, Luz Martín‐Carbonero^3^, Juan González‐García^3^

^1^Infectious Diseases, Hospital Infanta Leonor, Madrid, Spain. ^2^Infectious Diseases, Hospital Gregorio Marañón, Madrid, Spain. ^3^HIV Unit/Internal Medicine, Hospital La Paz, Madrid, Spain. ^4^HIV Unit, Centro Sanitario Sandoval, Madrid, Spain. ^5^Infectious Diseases, Hospital de La Princesa, Madrid, Spain. ^6^HIV Unit, Hospital Doce de Octubre, Madrid, Spain. ^7^Infectious Diseases, Hospital Ramón y Cajal, Madrid, Spain. ^8^HIV Unit/Internal Medicine, Hospital Clínico San Carlos, Madrid, Spain. ^9^Infectious Diseases, Hospital Fundación Jiménez Díaz, Madrid, Spain. ^10^Infectious Diseases, Hospital Príncipe de Asturias, Alcalá de Henares, Spain. ^11^Internal Medicine, Hospital Infanta Sofía, San Sebastián de los Reyes, Spain. ^12^Infectious Diseases, Hospital Severo Ochoa, Leganés, Spain. ^13^Clinical Research, Fundación SEIMC‐GeSIDA, Madrid, Spain. ^14^Sub. Gral. Farmacia y Productos Sanitarios, Servicio Madrileño de Salud, Madrid, Spain

**Introduction**: Ongoing high‐risk transmission behaviour among MSM challenges HCV elimination goals in many settings. We examined the epidemiology of acute/recent HCV infections and reinfections in MSM with and without HIV in the Madrid region.

**Methods**: This prospective study (2021–2023) enrolled MSM with HIV included in the Cohort of the Spanish Network of AIDS Research (CoRIS) at several clinical centres in Madrid and from those with previous HCV treated with DAA in the Madrid Coinfection Registry (Madrid‐CoRE). MSM without HIV were recruited among those receiving PrEP in one public STI clinic. Participants were assessed at baseline and follow‐up visits at months 6 and 12 (±2). Assessment in all visits included sexual behaviour, drug use, syphilis serology, HCV (serology and HCV‐RNA) and nucleic acid testing (NAT) for gonorrhoea and chlamydia on pharyngeal, urethral, and rectal swabs. Primary outcomes (reported herein) were the prevalence of active HCV infection at baseline and the incidence of new infections during follow‐up, stratified by previous HCV infection and HIV status. For the incidence analysis, people with prevalent HCV infections were excluded.

**Results**: A total of 1372 MSM (733 HIV‐positive and 639 HIV‐negative) were enrolled. HIV‐positive participants were slightly older (41 vs 37 years), and both groups were mainly native‐born Spaniards. At baseline, both HIV‐positive and negative participants reported high rates of condomless anal intercourse in the past 2 months (66% vs 98%) and had high rates of previous STIs (75% vs 68%). Chemsex use was 33% HIV‐positive and 27% HIV‐negative participants, respectively. Table 1 shows the prevalence of active HCV at baseline and incidence rates of new HCV infections during follow‐up. The prevalence ratio for those with versus those without prior HCV was 6.93 (95% CI 3.07–15.66). The incidence rate ratio for those with versus those without prior HCV was 8.41 (95% CI 2.96–23.88).

**P376: Table 1:** Active HCV prevalence at baseline and HCV incidence during follow‐up (FU)

Participant category	*N*	*N* with prevalent HCV	HCV prevalence % (95% CI)	Years FU	*N* with incident HCV	HCV incidence per 100 person years (95% CI)
No prior HCV infections						
HIV‐positive MSM	481	5	1.04 (0.34–2.41)	460.6	3	0.65 (0.21‐2.02)
HIV‐negative MSM	625	4	0.64 (0.17–1.63)	425.8	2	0.47 (0.12‐1.88)
Prior HCV infections						
HIV‐positive MSM	252	12	4.76 (2.48–8.17)	244.7	10	4.09 (2.2‐7.59)
HIV‐negative MSM	14	3	21.43 (4.66–50.8)	8.2	2	24.53 (6.14‐98.1)

**Conclusions**: Low HCV prevalence and incidence were found among MSM without prior HCV infection. However, these figures were significantly higher in MSM with a history of HCV, regardless of HIV status. Targeted interventions for HCV screening, rapid treatment initiation, and harm reduction strategies are crucial for MSM with prior HCV infection to achieve HCV elimination goals.

### P377 HIV/HBV coinfection in Portugal today: data from a multicentric approach

Ruben Carvalho^1^, Ana Martins^1^, João Matos^2^, Vanda Castro^3^, Joana Martinez^4^, António Ferreira^5^, Catarina Esteves^6^, Ana Parente^5^, Jorge Velez^4^, Josefina Mendez^2^, Cristina Valente ^1^

^1^Infectious Diseases, ULS Coimbra, Coimbra, Portugal. ^2^Infectious Diseases, ULS Santo António, Porto, Portugal. ^3^Internal Medicine, Hospital Cascais, Lisboa, Portugal. ^4^Infectious Diseases, ULS Região Aveiro, Aveiro, Portugal. ^5^Internal Medicine, ULS Alto Minho, Viana do Castelo, Portugal. ^6^Internal Medicine, Hospital de Cascais, Cascais, Portugal

**Introduction**: Recent data on the prevalence of HIV and hepatitis B virus (HBV) coinfection in Europe is scarce. The aim of this study was to assess the seroprevalence of HBV in HIV+ coinfected patients, as well as characterize these individuals according to their current status regarding HBV (HBV DNA and treatment) and HIV (HIV RNA and treatment), grade of fibrosis and presence of hepatitis C (HCV) and hepatitis delta viruses (HDV).

**Methods**: The study was carried out in five centres in Portugal, from north to south, and HIV/HBV population was analysed in a cross‐sectional study, in a sample size of 8415 HIV+ infected individuals, during 1 year period (2023).

**Results**: Overall 146 patients were included (1.73%), 113 (77.4%) were male, with an average age of 51.6 years. Mean time of diagnosis was 13.3 years for HIV and 10.9 years for HBV. Portuguese citizens represented 52% of the total, followed by those from Angola and Guinea Bissau (11.6%), Brazil (10.2%), Mozambique (8.2%). HBV was most commonly transmitted by sexual route (83.5%, heterosexual 42.4% and men who have sex with men 41.1%), followed by former drug users. HBeAg was positive in 41.4% and HBsAg loss occurred in 11 patients (7.5%) over the years. HBV DNA load <10 IU/ml was found in 71.9% and the current therapy for HBV include TDF/TAF + XTC in 87.6% of the cases. The most recent fibrosis evaluation, according to transient elastography, evidenced cirrhosis in 10.3% and 8.9% (n = 13) tested positive for anti‐HDV virus, 30.7% with detectable HDV RNA. Triple infection (HIV/HBV/HCV) represented 12.3% and 6.1% patients had a quadruple coinfection (HIV/HBV/HDV/HCV). Regarding HIV, 85.6% remained viral suppressed and ART regimens included INSTI in 55.5%, followed by NNRTI (22%).

**Conclusions**: Based on this sample, the prevalence of HIV/HBV coinfection in Portugal is 1.73% and 8.9% had HDV antibodies. Almost half are people originally from Portuguese speaking countries. National programmes to control HBV, mainly in migrant population, need to be improved. This study has some limitations, as estimates point to higher prevalence of HIV/HBV coinfection in the south of the country and only one centre from this region was represented.

### P378 HCV cure with direct‐acting antivirals in HIV/HCV coinfected patients belonging to key populations

Cristiana Oprea ^1^, Irina Ianache^1^, Cristiana Costescu^2^, Sebastian Piscu^2^, Sorina Vasile^2^, Luminita Ene^2^, Roxana Radoi^2^, Ionut Popa^2^

^1^Infectious Diseases, HIV, Carol Davila University of Medicine and Pharmacy, Bucharest, Romania. ^2^Infectious Diseases, HIV, Victor Babes Hospital for Infectious and Tropical Diseases, Bucharest, Romania

**Introduction**: Direct‐acting antiviral (DAA) treatment is a priority as HIV/HCV coinfection accelerates liver disease progression. In our Romanian tertiary care centre 21.5% of people living with HIV (PLH) have serological markers for HCV. This study aimed to assess the response to DAA treatment in HIV/HCV coinfected patients belonging to key populations.

**Methods**: Prospective study performed on PLH, with confirmed HCV infection, in active care at “Victor Babes” Hospital for Infectious and Tropical Diseases, Bucharest, between 1 January 2017 and 31 October 2023. Patients were stratified by modes of HCV acquisition in: injecting drug use (PWIDs), sexual transmission, and parenteral mode, during early childhood.

**Results**: Out of 122 HIV/HCV coinfected patients who started treatment with DAAs, 72.7% were males, with a median age (IQR) of 41 years (35–48), 69.6% being PWIDs. HCV viral load 12 weeks after treatment was available for 111 cases and 92.6% (103/111) were cured (sustained virological response at 12 weeks [SVR12]). One woman stopped DAA due to pregnancy and seven were non‐responders, two with genotype 3. Five of the non‐responders, treated initially with genotype‐specific DAA were re‐treated with pan‐genotypic (sofosbuvir/velpatasvir/voxilaprevir) and achieved SVR (total rate 97.2%) (Figure 1). All patients were on ART, with a median CD4 cell count (IQR) of 663 cells/µl (395–878) and 78.8% had undetectable HIV‐VL. PWIDs were younger at treatment initiation and less stable on ART (Table 1). Socio‐economic barriers and the lack of pan‐genotypic DAAs limited outcomes in the first years of the study. The availability of DAAs varied during the years: ombitasvir/paritaprevir/ritonavir (4.1%), grazoprevir/elbasvir (14.0%), sofosbuvir/ledipasvir (34.7%), sofosbuvir/velpatasvir (47.1%), sofosbuvir/velpatasvir/voxilaprevir (4.9%). HCV reinfection was diagnosed in one case. There was a marked increase in the use of DAAs during the years, from 4.5% in 2017 to 100% of the newly diagnosed cases in 2023.

**P378: Figure 1:**
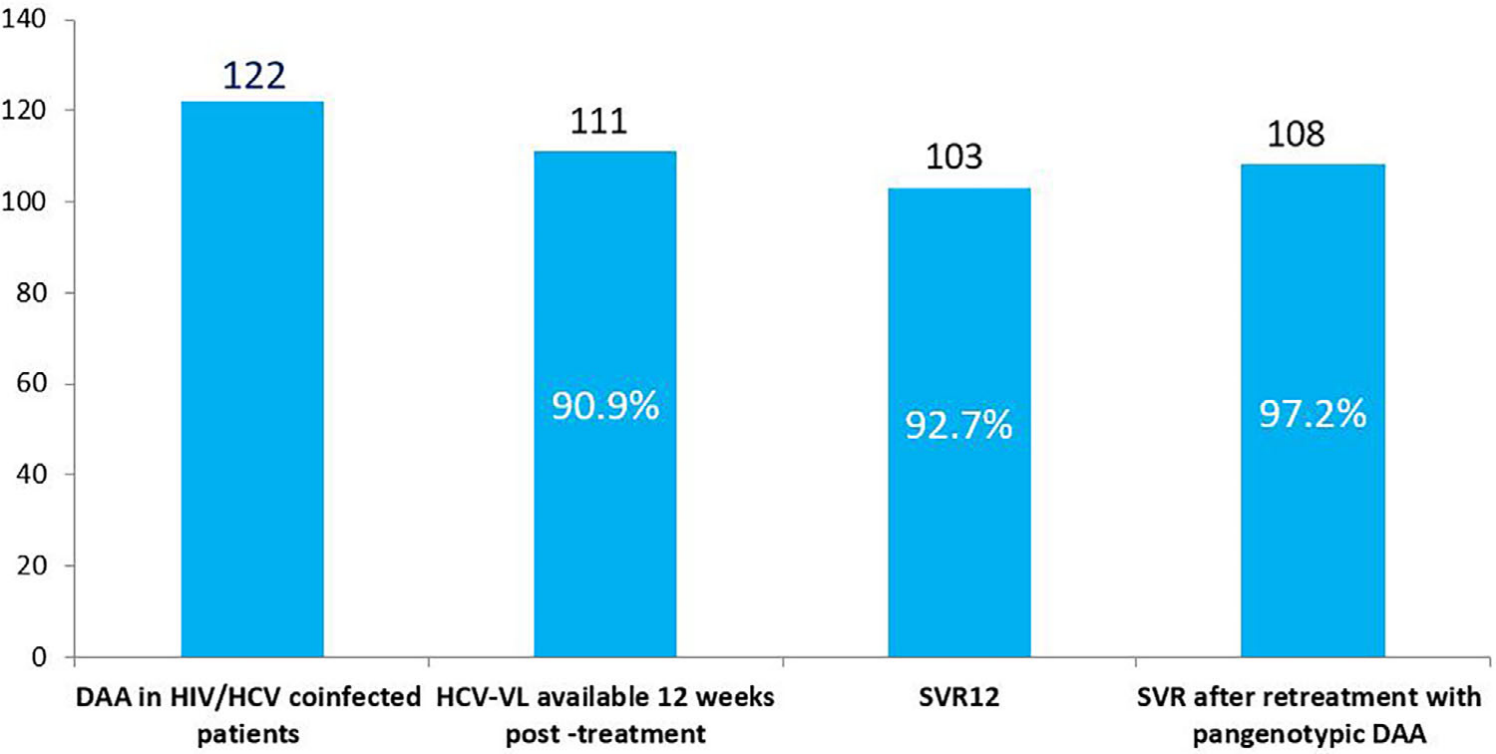
Cascade of care in HIV/HCV coinfected patients treated with DAA.

**P378: Table 1:** Demographic and clinical characteristics—comparison by modes of HCV acquisition

Characteristics		Total *n* = 122	PWIDs *n* = 85	Non‐PWIDs *n* = 37	*p*‐value
Gender (male)	*n* (%)	88 (72.7)	67 (79.7)	21 (56.7)	0.007
Age (years)	Median (IQR)	41 (36–45)	40 (35–43)	42 (36–52)	0.005
CD4 cell count/µl	Median (IQR)	663 (385–882)	660 (423–867)	665 (336–955)	0.764
HIV‐RNA <50 copies/ml	*n* (%)	95 (80.5)^a^	65 (76.4)^b^	31 (88.5)^c^	0.044
Previous IFN treatment	*n* (%)	8 (6.5)	3 (3.5)	5 (13.5)	0.039
Fibrosis stage					
F0‐F1	*n* (%)	55 (46.6)	38 (41.1)	17 (45.9)	0.886
F1‐F2	*n* (%)	43 (36.4)	29 (34.1)	15 (40.5)	0.886
F3‐F4	*n* (%)	20 (16.9)	14 (16.4)	5 (13.5)	0.886

^a^ Out of 118 available.

^b^ Out of 85 available.

^c^ Out of 35 available.

**Conclusions**: DAA treatment success rate in HIV/HCV coinfected patients from key population was high and comparable to those monoinfected. The SVR rates were similar in PLH infected by sexual mode or in PWIDs, irrespective of the CD4 cell count or HIV‐VL. Elimination of HCV requires a targeted scale‐up of DAA treatment and behavioural interventions in particular among high‐risk populations.

### P379 “What we wanted was what we needed”—collaborative community advocacy for inclusive clinical trials in emerging hepatitis C research: lessons from the EATG Sitges model

Rocco Pignata, Ian Hodgson, Giorgio Barbareschi, Apostolos Kalogiannis, Fiona Greenhalgh, Brian West, Sean Hosein, Alain Volny‐Anne, Luis Mendão, Memory Sachikonye

European AIDS Treatment Group (EATG), Brussels, Belgium

**Introduction**: Between 2007 and 2017, European AIDS Treatment Group (EATG) organized a series of community‐owned multi‐stakeholder meetings in Sitges, Spain, promoting the inclusion of people with HIV and hepatitis C virus (HCV) coinfection in clinical trials for emerging HCV treatments, and advocacy for rapid access. Stakeholders included community representatives, medicines regulators, physicians, researchers, and pharmaceutical industry. In 2023 EATG Belong project, focusing on the inclusion of people living with HIV in non‐HIV clinical trials, developed a case study documenting experiences, results, methodologies, and lessons learned from the Sitges meetings to provide examples and guidance for use in other disease areas.

**Material and methods**: The case study is based on documentary analysis of materials from the meetings and 19 semi‐structured interviews with participants. It includes verbatim reflections from five community participants, presents the nature and characteristics of the Sitges Model and its outcomes, and the transferability of this model to advocate for the inclusion of people living with HIV in non‐HIV clinical trials.

**Results**: Characterized by a strong egalitarian multistakeholder ambiance, the Sitges meetings built a platform of credibility and raised awareness of HIV/HCV coinfection, combining human experience with advocacy in medical research. Key outcomes included the amendment of exclusion criteria to include people with coinfection in clinical trials, reworking EMA guidelines, and building meaningful bridges between stakeholders. Factors to consider when applying the Sitges Model in other disease areas include close involvement of community leaders and ensuring all current scientific information is available. Meetings must also aim for the co‐creation of solutions and collaborative agreements. Efficient organization, clear agendas, detailed post‐event reporting with recommendations, and unanimous joint statements are crucial. The Sitges case study is a resource to increase the overall effectiveness of community advocacy.

**Conclusions**: The case study explored the Sitges meetings, their key outcomes and characteristics that could be applied in other health advocacy settings. Reflecting on the concept of ‘patient expert’, the case study offers an example of a successful advocacy model highlighting the value of community advisory settings. It shows how community advocates can effectively collaborate with other stakeholders towards a shared vision and common goals.

### P380 HIV/HCV: barriers to treatment in the era of direct acting antivirals in a low‐middle income country

Raquel Zhumi, Cruz Molina Neizly, Falak Adriana, Alicia Sisto, Maria Rolon, Natalia Laufer

Hospital Juan A. Fernandez, Buenos Aires, Argentina

**Introduction**: HIV‐HCV coinfection is a frequent event. Direct acting antivirals (DAA) are associated with higher rates of HCV clearance and low rate of adverse effects. However, it is reported that only 5% of these patients receive treatment in Argentina. To describe limiting barriers in HCV treatment initiation for people living with HIV and HCV on follow up at a tertiary center in Argentina.

**Materials and methods**: A cross sectional study was carried out, which included people older than 18 years of age living with HIV, with reactive HCV serology and a follow up for ≥12 months. Sociodemographic, clinical and virological variables were recorded at the moment of DAA initiation and in the last clinical control for individuals in whom HCV treatment was not prescribed. To evaluate possible differences between groups in the variables of interest, a comparative analysis was performed (*t* test and chi2).

**Results**: Among individuals on follow‐up, 368 fulfil inclusion criteria. Of these, 40 (10%) were excluded from the analysis due to spontaneous HCV clearance. Of the remaining 328 people, 168 (51%) received DAA with documented HCV clearance in 105 (63%). In the remaining 63 (37%) post treatment studies are still pending. Among those people who did not receive treatment, 80 (50%) had not completed HCV RNA viral tests. When comparing the variables of interest between both groups, we evidenced significant differences, in median CD4 and APRI score, and time from diagnosis to HCV treatment (Table 1). Of the 80 people who completed their HCV studies, nine of them were transferred to another centre, eight were lost to follow‐up, nine are awaiting for DAA authorization, eight had active use of illicit drugs or psychiatric comorbidity that jeopardize treatment initiation and 12 deaths (causes are unknown).

**P380: Table 1:** General information of the population

	Total (*n* = 328)	Treatment group^a^ (*n* = 168)	Non treatment group^a^ (*n* = 160)	*p*‐value^a^
Age, years (IQR)	55 (50–58)	56 (51–56)	53 (49–58)	0.002
Men cis, % (*n*)	74% (242)	73% (122)	75% (120)	0.63
Tertiary/university education	15% (51)	16% (27)	15% (24)	0.79
Years since HIV diagnosis	25 (19–30)^a^	26 (20–30)	25 (19–29)	0.19
Years since HCV diagnosis	18 (12–25)^a^	19 (13–26)	15.5 (11–24)	0.003
Psychiatric history, % (*n*)	36% (119)	35% (59)	37% (60)	0.65
History of drug use/alcohol, % (*n*)	62% (203)	63% (107)	60% (96)	0.49
Alcohol	31% (101)	34% (58)	27% (44)	0.17
Drug	30% (100)	32% (54)	30% (49)	0.76
APRI index	1.035 (0.53–2.1)	1.125 (0.63–2.29)	0.90 (0.44–1.878)	0.008
CD4 cell/µl (IQR)	445 (244–727)	534 (276–785)	385 (199–601)	0.002
HIV VL <40, % (*n*)	71%	80%	61%	0.0002

*Note*: Disaggregates between people who received or not treatment for HCV.

^a^ The cut‐off of the median number of years since HIV and HCV diagnosis was made with the date of the last available control.

**Conclusions**: Access to hepatitis C treatment was mainly balked because of the difficulty to complete diagnosis tests. Individuals that accessed treatment had higher known time of infection and higher degree of liver fibrosis which reflects the priority given in previous guidelines to the treatment of individuals with advanced liver disease. On the other hand, individuals who did not receive treatment presented indirect markers of lower adherence to HIV treatment. These findings reinforce the importance of improving access and linkage to the health system and to promote HCV treatment at early stages.

### P381 The era of DAAs: assessing the patients' characteristics, clinical impact, and emergence of comorbidities in HIV/HCV‐coinfected versus HIV‐infected individuals

Beatriz Álvarez‐Álvarez^1^, Laura Prieto‐Pérez^1^, Alberto de la Cuadra‐Grande^2^, Miguel Ángel Casado^2^, Alfonso Cabello Úbeda^1^, Aws W Al‐Hayani^1^, Irene Carrillo Acosta^1^, Ignacio Mahillo‐Fernández^3^, Miguel Górgolas Hernández‐Mora^1^, José M Benito^4^, Norma Rallón ^4^

^1^Division of Infectious Diseases, Hospital Universitario Fundación Jiménez Díaz, Madrid, Spain. ^2^Pharmacoeconomics & Outcomes Research, Iberia (PORIB), Madrid, Spain. ^3^Biostatistics and Epidemiology Unit, Instituto de Investigación Sanitaria‐Hospital Universitario Fundación Jiménez Díaz, Universidad Autónoma de Madrid, Madrid, Spain. ^4^HIV and Viral Hepatitis Research Laboratory, IIS‐FJD, UAM, Hospital Universitario Rey Juan Carlos, Madrid, Spain

**Introduction**: HIV/HCV coinfection establishes interactions between viruses that can modify the clinical course of both diseases. The aim of this study was to determine whether HIV‐infected individuals versus individuals with HIV/HCV coinfection, in the era of interferon‐free therapies, exhibit an increased incidence of comorbidities and non‐AIDS‐related events.

**Materials and methods**: A retrospective analysis was conducted by collecting data from clinical records of Spanish patients at a tertiary hospital involving HIV/HCV‐coinfected and HIV‐infected patients, all with effectively controlled HIV. Coinfected patients underwent HCV clearance using direct‐acting antivirals (DAAs) and had no history of interferon treatment. The incidences of hypertension, diabetes mellitus, cardiovascular disease, kidney disease, liver disease, non‐AIDS cancer, and death were compared between the groups. Multivariate adjustments for all factors potentially impacting outcomes were used to assess the risk of clinical event onset. Propensity score (PS) analyses were also conducted to support the multivariate model results.

**Results**: Data were available from 229 HIV/HCV‐coinfected patients and 229 HIV‐infected patients. Both cohorts were comparable in terms of age, gender distribution, follow‐up, and HIV‐related characteristics. Multivariate models and PS showed that previous exposure to HCV was not associated with the onset of any clinical events studied. Significant differences between HIV/HCV‐coinfected and HIV‐infected were not found for survival according to the log‐rank test (*p* = 0.402).

**Conclusions**: Successful HCV elimination using DAAs improved the outlook regarding comorbidities and survival across HIV/HCV‐coinfected cohorts. Early HCV detection and DAA therapy could enhance clinical results. These findings provide an optimistic perspective for those living with HIV/HCV coinfection and underscore the importance of continuing efforts toward early detection and DAA treatment initiation.

### P382 Predictors of hepatitis C treatment failure in people living with HIV

Alan Sinuhe Medina Valle ^1^, Elizabeth Mendoza Portillo^2^, Humberto Gudiño Solorio^3^

^1^Internal Medicine, Hospital General Ticoman, Mexico, Mexico. ^2^Internal Medicine, Hospital General Ruben Leñero, Mexico, Mexico. ^3^Internal Medicine, Clinica Especializada Condesa, Mexico, Mexico

**Introduction**: Hepatitis C is an inflammation of the liver caused by the hepatitis C virus. The people living with HIV have an increased risk of acquiring hepatitis C virus (HCV), and it has recently been observed that this population group may have an increased risk of virological failure with the use of direct‐acting antivirals (DAA) and not achieve a sustained viral response (SVR) [1, 2].

**Materials and methods**: We conducted a cross‐sectional analytical study of people living with HIV, from paired groups, at the Clinica Especializada Condesa at Mexico City, in the period from January 2020 to December 2023. We included people over 18 years old, all of them were people living with HIV (PLWH) and hepatitis C (people with HCV RNA greater than 1000 IU/ml). They received treatment with DAA, specifically Sofosfuvir with Velpatasvir by availability in our care center, the viral HCV RNA was measured at 12 weeks after finishing treatment to determine whether sustained virological response existed or not. Once selected, they were assigned to one of two groups: people with 100% adherence and another group of people with poor adherence. We intended to determine predictors of treatment adherence.

**Results**: 92 people were included: 46 in each group, all of them PLWH and hepatitis C. A statistically significant difference was found in patients with prior HIV treatment failure (31% vs 51%, *p* = 0.03) or low‐level viraemia (2% vs 4%, *p* = 0.02). On the other hand, psychiatric disorders were shown to decrease treatment adherence (17% vs 68%, *p* < 0.001) (see Table 1).

**P382: Table 1:** Comparison between people SVR and people who failed to treatment with DAA

Viral response	SVR	Failure to treatment to DAA	*p*‐value
	46 (50%)	46 (50%)	
Adherence percent %			
25%	0	0	0.432
50%	0	39%	<0.001
75%	0	61%	<0.001
100%	100%	0%	<0.001
Men cisgender and men who have sex with men (MSM)	97%	100%	0.01
People drugs users	22%	47%	0.058
Alcohol consumption	54%	46%	0.04
Smoking	28%	72%	0.05
PLWH	100%	100%	0.52
Status HIV			
Undetectable	98%	93%	
Low level viraemia (LLV)	0%	3%	0.02
Virological failure	2%	4%	0.04
Antiretroviral therapy			
BIC/TAF/FTC	79%	81%	0.245
TDF/FTC+DRV/cob	21%	16%	0.001
TDF/FTC+DRV+rit	0%	3%	0.001
People with virological failure prior to HIV	31%	51%	0.03
Syphilis the last 6 months	17%	68%	<0.001
Adverse effects to DAA treatment	15%	13%	0.773

**Conclusions**: Patients living with HIV infection who have experienced previous treatment failures are likely to show lower adherence to hepatitis C virus infection treatment. Also patients with psychiatric disorders are in risk of lower adherence to treatment of hepatitis C. This information would support us to pay more attention to these population groups to avoid treatment failures and reduce the potential transmission risk of resistant strains of hepatitis C.

References

1. Manns MP, Buti M, Gane E, Pawlotsky JM, Razavi H, Terrault N, Younossi Z. Hepatitis C virus infection. Nat Rev Dis Primers. 2017;3:17006.

2. Gower E, Estes C, Blach S, Razavi‐Shearer K, Razavi H. Global epidemiology and genotype distribution of the hepatitis C virus infection. J Hepatol. 2014;61(1 Suppl):S45–57.

## People living with HIV and other diseases

### P383 Preliminary results of a pilot study to evaluate the usefulness of using patient‐reported outcomes (PROs) in the follow‐up of patients living with HIV

Sara de la Fuente Moral ^1^, María Pilar Corrales Rodríguez^2^, Ana Belén Hernández López^2^, Carlos Folguera Olías^2^, Belén Menchén Viso^2^, Miriam Redondo^3^, Victoria Ayala Vargas^4^, María Sainz Guerra^5^, Alberto Díaz de Santiago^1^

^1^HIV Unit, Puerta de Hierro University Hospital, Madrid, Spain. ^2^Puerta de Hierro University Hospital, Madrid, Spain. ^3^HOPES (HOH HEALTH SL), Madrid, Spain. ^4^Government Affairs Spain, Gilead Sciences, Madrid, Spain. ^5^Medical, Gilead Sciences, Madrid, Spain

**Introduction**: The follow‐up of patients living with HIV includes the evaluation of multiple clinical and psychosocial aspects. Patient‐reported outcomes (PROs) could be a valuable tool. The main objective of this study is to evaluate the detection of solvable problems using PROs, comparing it with a control group managed in accordance with usual practice.

**Materials and methods**: Prospective, randomized study, with an intervention (active) and a control group. The active group completed validated quality‐of‐life questionnaires (WHOQOL‐BREF and Clinic Screening Tool [CST]), quarterly for 1 year. When the score exceeded established thresholds, a nurse contacted the patient, and when necessary the nurse informed the patient's doctor.

**Results**: After signing the informed consent, 100 individuals were included in the study: 50 in the surveillance group using PROs, and 50 in the control group. Active and control groups were similar in age (51.52 vs 49.34 years old; *p* = 0.5), sex (84% vs 76% men; *p* = 0.45), immunosuppression (100% in both groups) and CD4+ T cells level (650.63 vs 727.41; *p* = 0.8). In the active group, only 20 individuals (40%) responded at least once to the questionnaires sent. The response rate was affected by technical problems, loss of interest and stigma, compared to that shown by other patient populations. Eighty percent of patients reported a good perceived quality of life in the WHOQOL‐BREV questionnaire. However, in the CST questionnaire, 18 of 20 (91.95%) patients revealed at least one problem in some dimension, and some of them, especially cognitive, sexual and stigma problems, were not detected in consultation (Table 1). The detection rate of health problems was higher in the active group (28/50 = 56%) than in the control group (11/50 = 22%). This could be explained by the greater self‐perception of problems thanks to the questionnaires, and the intervention of the nurse (Figure 1).

**P383: Table 1:** Quality of life and problems reported by patients

	Men, *n* (%)	Women, *n* (%)	Total, *n* (%)
Quality of life (WHOQOL‐BREV)			
<15	4 (22.22)	0	4 (20.00)
>15	14 (77.77)	2 (100)	16 (80)
Problems (CST)			
Cognitive alteration	7/15 (46.66)	1/3 (33.33)	8/18 (44.44)
Anxiety/depression	7/15 (46.66)	1/3 (33.33)	8/18 (44.44)
Sleep disorder/fatigue	12/15 (80)	2/3 (66.66)	14/18 (77.77)
Physical discomfort	7/15 (46.66)	2/3 (66.66)	9/18 (50)
Sexual function	10/15 (66.66)	2/3 (66.67)	12/18 (66.66)
Stigma	12/15 (80)	2/3 (66.66)	14/18 (77.77)
Perceived social support	6/15 (40)	0	6/18 (33.33)
Material deprivation	3/15 (20)	1/3 (33.33)	4/18 (22.22)

**P383: Figure 1:**
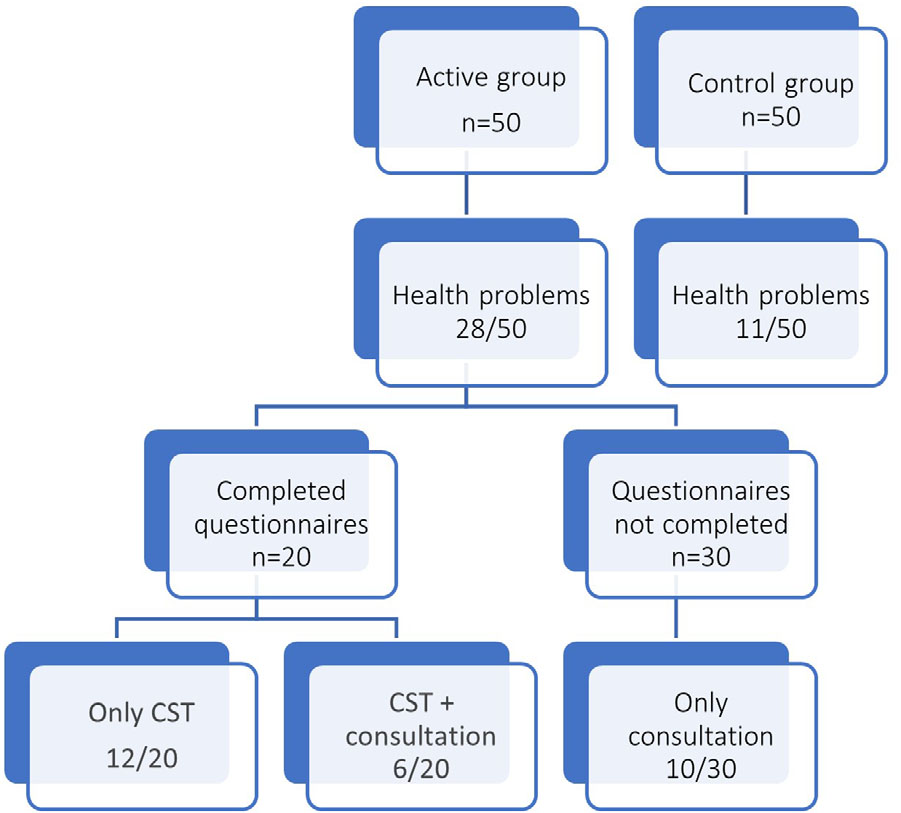
Summary of study results.

**Conclusions**: PROs are a useful tool as a complement to in‐person consultations. Their use could contribute to the detection of hidden problems, and to the exploration of areas which usually go unnoticed in clinical practice. The main limitation in this study was the high rate of non‐completion of questionnaires.

### P384 Preferences for a psychosocial intervention based on health and wellbeing coaching, for people living with HIV in a secondary care setting in England, Wales and Scotland: a discrete choice experiment

Janey Sewell^1^, Meaghan Kall^2^, Adamma Aghaizu^2^, Alec Miners^3^, Alex Sparrowhawk^4^, Jane Holder^5^, Farai Mukazi^6^, Alison Rodger^1^, Fiona Lampe ^1^, Valentina Cambiano^1^

^1^Institute for Global Health (IGH), University College London, London, UK. ^2^UK Health Security Agency (UKHSA), London, UK. ^3^Source Health Economics, London, UK. ^4^George House Trust, Manchester, UK. ^5^The Starling Clinic, Musgrove Park Hospital, Taunton, UK. ^6^Crawley Sexual Health, Crawley Hospital, Crawley, UK

**Introduction**: Psychosocial support may help to improve mental health and wellbeing among people living with HIV (PLHIV). We assessed preferences for a psychosocial intervention based on health and wellbeing coaching among PLHIV.

**Materials and methods**: We conducted a discrete choice experiment (DCE) among PLHIV (aged 18+) who participated in Positive Voices 2022 survey and agreed to further contact. One thousand were invited via email in August–September 2023 and a further 250 in May–June 2024. Two reminders were sent. The online DCE was designed based on prior literature reviews and qualitative work and assessed preferences across four intervention characteristics: intervention deliverer; delivery mode; session number and frequency. Participants indicated their preference using nine questions concerning two different ways of receiving the coaching intervention (programme A or B), or not to receive it. Data were analysed using a conditional logit model.

**Results**: Three hundred and ten PLHIV completed the DCE: 74% (227/307) white ethnicity; 84% (257/307) men (including transmen); median age 54 years (range 23–78). There was a strong preference to opt‐into the intervention over not taking the intervention (OR programme A vs neither 4.41; 95% CI 3.29–5.92; OR programme B vs neither 4.58; 95% CI 3.42–6.12; Figure 1). There was a slight positive preference for (in order of strength): flexibility between face to face (f2f) and online versus always f2f (OR 1.32; 95% CI 1.11–1.58); meeting 2 weekly versus weekly (OR 1.23; 95% CI 1.10–1.38). There was no preference for number of sessions or type of staff delivering the intervention: trained peer or healthcare worker or psychologist/counsellor. Restricting to those reporting depressive symptoms (PHQ‐9 ≥10; 28%, 88/310) the strength of preference for wanting the intervention increased (OR programme A vs neither 5.50; 95% CI 3.05–9.91; OR programme B vs neither 6.50; 95% CI 3.60–11.74). The direction of preference for other attribute levels was unchanged, but only having 12 sessions (OR 1.42; 95% CI 1.05–1.92) versus fewer sessions was statistically significant.

**P384: Figure 1:**
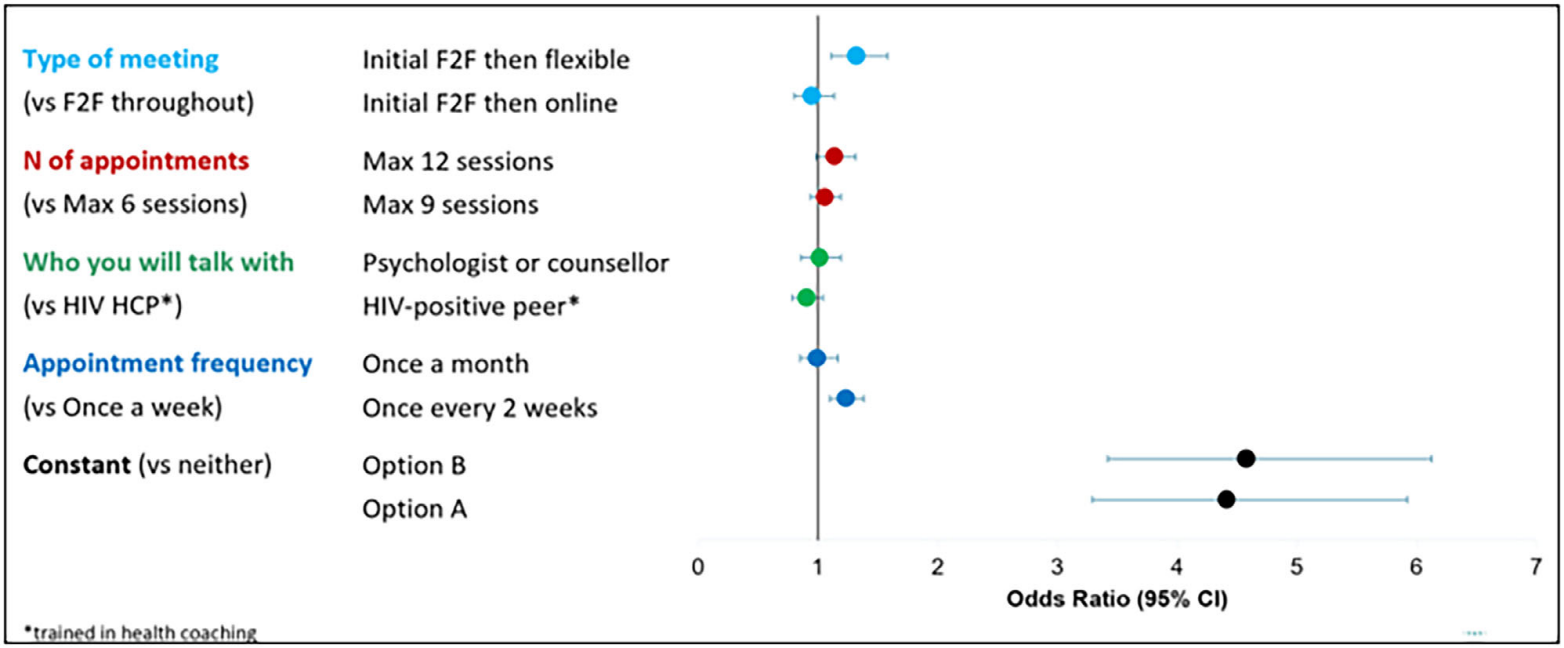
Preferences for a psychosocial intervention based on health and wellbeing coaching among PLHIV.

**Conclusions**: PLHIV showed a strong interest for a psychosocial intervention (particularly in those reporting depressive symptoms) with preference for flexibility between f2f and online, meetings every 2 weeks. These findings have been used to develop a coaching intervention to be evaluated in the SPHERE randomized controlled trial.

### P385 Developing the person‐centred needs informed model of care for people with HIV (NICHE) programme: findings from formative qualitative research

Vasiliki Papageorgiou ^1^, Lucy Cullen^2^, T Charles Witzel^1^, Flavien Coukan^1,5^, Alex Sparrowhawk^6^, Jonathan William Shaw^3^, Janey Sewell^1^, Alison Rodger^1^, Fiona Burns^1^, Carl May^4^

^1^Institute for Global Health, University College London, London, UK. ^2^Department of Health Services Research and Policy, London School of Hygiene & Tropical Medicine, London, UK. ^3^Devon Sexual Health, Royal Devon University Healthcare NHS Foundation Trust, Exeter, UK. ^4^Faculty of Public Health and Policy, London School of Hygiene & Tropical Medicine, London, UK. ^5^Patient Experience Research Centre, School of Public Health, Imperial College London, London, UK. ^6^George House Trust, Manchester, UK

**Introduction**: Many people living with HIV are impacted by unique and interlinked adverse psychosocial factors including high levels of depression, stigma and socioeconomic disadvantage which are known to affect mental health and well‐being. Person‐centred care, an approach supported by the NHS Long Term Plan, offers an opportunity to better address these factors.

**Materials and methods**: The NICHE (a person‐centred Needs Informed model of Care for people with HIV) research programme has developed, and will evaluate, a health coaching with social prescribing intervention. We conducted in‐person focus group discussions (FGDs) in June to September 2023 with people living with HIV to shape NICHE's intervention design and explore what “living well” meant to participants, including challenges and concerns that can negatively impact quality of life.

**Results**: Thirty‐seven people living with HIV from a diverse range of backgrounds (gay and bisexual men who have sex with men; people of Black ethnicities; women; people aged 60+) attended nine FGDs (Table 1). Five FGDs were held in London and four in clinics outside of London (Devon, Sheffield, Sussex). We identified three interconnected narratives of “living well”: 1) an unrestricted living; 2) emotional sexual health and intimacy well‐being; and 3) autonomy of health and healthcare. Unrestricted living referred to the desire to not have to navigate life and negotiate decisions differently to others (for example, having to think about things that other people do not have to think about, not having the same opportunities available to them in the same way). The second area centred around desires of sex and intimate relationships, feelings of deservedness and broader emotional needs. Participants also shared feelings of a limited sense of autonomy relating to health and challenges accessing healthcare. Narratives were underpinned by pervasive HIV‐related stigma, the social isolation and loneliness stigma reinforces, and a sense of erosion and increasing fatigue.

**P385: Table 1:** Summary characteristics of focus group participants

Characteristic	Focus group participants (*N* = 37) *n* (%)
**Age**	
18–34	5 (13.5)
35–44	4 (10.8)
45–54	8 (21.6)
55–64	16 (43.2)
65+	4 (10.8)
**Gender**	
Cisgender woman	11 (29.7)
Cisgender man	23 (62.2)
Non‐binary	2 (5.4)
Non‐binary transgender	1 (2.7)
**Ethnicity**	
White British	13 (35.1)
White other	8 (21.6)
Black African	8 (21.6)
Black other	4 (10.8)
Mixed/multiple	3 (8.1)
Other ethnic group	1 (2.7)
**Year of diagnosis**	
1982–1995	7 (18.9)
1996–2005	13 (35.1)
2006–2015	13 (35.1)
2016–2021	4 (10.8)
**Sexuality**	
Gay or lesbian/homosexual	18 (48.6)
Heterosexual/straight	12 (32.4)
Bisexual	4 (10.8)
Queer	2 (5.4)
Asexual	1 (2.7)
**Country of birth**	
Born in UK	20 (54.1)
Not born in UK	17 (45.9)

**Conclusions**: Our formative research highlights the importance of an approach to HIV care which considers broader physical, psychological, and social needs. Past and current experiences of stigma and discrimination continue to shape perceptions of self, relationships with others and experiences of healthcare.

### P386 Inflammatory biomarkers decay after first‐line antiretroviral treatment initiation with dolutegravir/lamivudine or bictegravir/emtricitabine/tenofovir alafenamide. What puts the fire out faster?

Analuz Fernandez ^1^, Sofia Scevola^1^, Raul Rigo Bonin^2^, Maria Saumoy^1^, Arkaitz Imaz^1^, Daniel Podzamczer^3^, Juan Manuel Tiraboschi^1^

^1^Infectious Diseases, Hospital Universitari de Bellvitge, L´Hospitalet de Llobregat, Spain. ^2^Pharmacology Service, Hospital Universitari de Bellvitge, L´Hospitalet de Llobregat, Spain. ^3^Infectious Diseases, Fight Infections Foundation, Barcelona, Spain

**Introduction**: The aim of this study was to compare short‐term decay in several blood biomarkers (BM) after first‐line treatment initiation in a group of people with HIV‐1 receiving bictegravir/emtricitabine/tenofovir alafenamide (BIC/FTC/TAF) or dolutegravir/lamivudine (DTG/3TC).

**Materials and methods**: This is a sub‐study of an open‐label, multicentre, randomized, pilot clinical trial [1]. Asymptomatic, ART–naive males with plasma HIV‐1 RNA >1000 copies/ml and <500,000 copies/ml, CD4 cell count >200/µl, negative for HBV and no resistance associated mutations were eligible. One participant who met acute HIV‐1 infection criteria was excluded from the main study but participated in this analysis. Participants were randomized 2:1 to initiate BIC/F/TAF or DTG/3TC. Inflammatory BM CRP, IL‐6, D‐dimer, sCD163, sCD14, TNF‐α, cell activation BM VCAM1 and FABP2 and HIV‐1 RNA (LOQ 20 copies/ml) were measured in blood plasma (BP) at baseline (BL), days 3, 7, 14, 28, and week 12 and 24. We used a linear mixed effects model to test the time trend and the interaction between time and treatment adjusting by baseline viral load.

**Results**: Twenty‐five participants were included (16 in the DTG/3TC arm and nine in the BIC/FTC/TAF). Median (range) age was 31 (20–60) years; BL CD4 count and HIV‐1 RNA in BP were 398 (216–716) cells/µl and 4.56 (3.09–5.65) log_10_ copies/ml, respectively. No statistically significant differences were observed between treatment groups in HIV‐1 RNA at BL. By week 24, undetectable viral load was achieved in seven out of nine subjects and 14 out of 16 participants in triple and dual therapy, respectively. There was a high level of intra‐subject variability (intra‐class coefficient >0.7) in all the BM analysed. No interaction between BL HIV‐1 RNA and BM decline (*p* = 0.1–0.7) was detected. We observed no significant changes in CRP, IL‐6, D‐dimer, sCD163, sCD14 and TNF‐α between groups (Table 1).

**P386: Table 1:** Median (IQR) biomarkers at each timepoint

Biomarker	Treatment group	Baseline	Day 3	Day 7	Day 14	Day 28	Week 12	Week 24
CRP (mg/L)	DTG+3TC	1.7 [1‐2.5]	2.3 [1–3.7]	3.8 [0.5–7.1]	2.6 [0.7–4.4]	1.9 [0.8–3.0]	2 [0.9–3.0]	1.6 [1.03–2.21]
CRP (mg/L)	BIC/F/TAF	7.8 [0–17.4]	4.6 [0–9.7]	2.9 [0.7–5.0]	1.6 [0.7–2.4]	1.1 [0.4–1.8]	1.2 [0.2–2.2]	1.43 [0.15–2.72]
*p*‐value			0.1	0.08	0.11	0.13	0.32	0.37
IL‐6 [subjects >3.5 ng/L (%)]	DTG+3TC	1 (6.25%)	2 (12.5%)	3 (18.75%)	2 (14.29%)	3 (18.75%)	0 (0%)	1(6.25%)
IL‐6 [subjects >3.5 ng/L (%)]	BIC/F/TAF	2 (22.22%)	2 (22.22%)	2 (25%)	2 (22.22%)	1 (11.11%)	0 (0%)	1 (14.2%)
DD (µg/L)	DTG+3TC	79.6 [49.1–110.2]	119 [74.1–163.9]	99 [60.5–137.4]	82.2 [42.8–121.7]	59.6 [36.9–82.3]	51.9 [34.9–68.9]	48.9 [33.9–63.9]
DD (µg/L)	BIC/F/TAF	186.2 [53.3–319]	214.6 [54.2–375]	183 [50.5–315.4]	142.2 [42.9–241.5]	141.3 [41.9–240.7]	121.5 [36.1–206.8]	111.1 [15.5–206.7]
*p*‐value			0.6	0.29	0.23	0.41	0.75	0.47
sCD14 (µg/L)	DTG+3TC	2231.5 [1810.5–2652.5]	2339.3 [1913.35–2765.2]	2240.7 [1858–2623.4]	2262.9 [1822–2703.7]	2261.2 [1953.9–2568.6]	2271.2 [1931.3–2611]	2097.02 [1825.2–2368.7]
sCD14 (µg/L)	BIC/F/TAF	2518.1 [1874.3–3162]	2581.9 [2015.2–3148.5]	2584.6 [2193.9–2975.2]	2294.9 [1777.1–2812.7]	2390.1 [1826.2–2954]	2343.5 [1794.5–2892.6]	2360.8 [1996.5–2725]
*p*‐value			0.65	0.57	0.08	0.32	0.710	0.64
sCD163 (ng/L)	DTG+3TC	926.8 [773.3–1080.3]	946 [819.8–1072.2]	918.3 [836–1000.6]	860.1[776.26–944]	808 [706.3–909.7]	740.61 [628.92–852.3]	706.7 [606.02–807.3]
sCD163 (ng/L)	BIC/F/TAF	1010.5 [705.8–1315.3]	985.2 [723.7–1246.8]	973.1 [745.8–1200.4]	886.3 [732.4–1040.2]	812.44[653.4–971.4]	735.6 [506.7–964.5]	759.1 [451.8–1066.5
*p*‐value			0.34	0.38	0.41	0.33	0.7	0.83
TNF‐α (ng/L)	DTG+3TC	20.8 [16.2–25.5]	21.7 [16.7–26.7]	20.2 [15.9–24.6]	20.6 [16.1–25]	21.2 [16.9–25.6]	19.6 [15–24.2]	18.2 [14.6–21.8]
TNF‐α (ng/L)	BIC/F/TAF	22.8 [16.1–29.5]	22.3 [15.6–29]	23.1 [17.1–29.1]	20.7 [15.8–25.6]	21 [16.2–25.8]	21.1 [17.5–24.7]	19.3[15.5–23.1]
*p*‐value			0.07	0.73	0.18	0.16	0.8	0.68
FABP‐2 (ng/L)	DTG+3TC	2078.56 [1787.09–2370.03]	2161.56 [1857.12–2466.01]	2121.12 [1788.8–2453.45]	1998.5 [1679.16–2317.84]	1977.06 [1680.58–2273.55]	1861.13 [1535.06–2187.2]	1782.3 [1474.8–2089.9]
FABP‐2 (ng/L)	BIC/F/TAF	2511.22 [1696.41–3326.04]	2466.78 [1751.74–3181.82]	2488.12 [1693.52–3282.73]	2331.11 [1735.74–2926.48]	2495.67 [1913.22–3078.11]	2268.38 [1606.6–2930.15]	2072.4 [1416.5–2728.3]
*p*‐value			0.21	0.05	0.34	0.57	0.43	0.79
VCAM‐1 (µg/L)	DTG+3TC	452 [376.1–527.9]	447.7 [375.7–519.8]	422 [356.1–487.9]	437.4 [354.7–520.1]	428.3[353.8–502.8]	453.6 [364.2–542.9]	447.5 [366.6–528.5]
VCAM‐1 (µg/L)	BIC/F/TAF	507.1 [378.5–635.7]	499.8 [387.3–612.4]	473.1 [353.6–592.6]	468.8 [366.9–570.8]	462.1 [368–556.2]	427.2 [325.6–528.8]	431.4 [326.3–536.5]
*p*‐value^a^			0.91	0.98	0.81	0.61	0.2	0.37

Abbreviations: CD4, cluster of differentiation‐4; CRP, C‐reactive protein; DD, D‐dimer; FABP2, fatty acid binding protein‐2; HBV, hepatitis B virus; IL‐6, interleukin‐6; LOQ, limit of quantification; sCD14, human soluble cluster of differentiation‐14; sCD163, soluble‐CD163; TNF‐αTNF‐a, tumour necrosis factor alpha; VCAM‐1, vascular cell adhesion molecule‐1.

^a^ *p*‐values of the time trend effect and the interaction effect between time and treatment estimated using a linear mixed effects model.

**Conclusions**: No differences in most inflammatory BM were found after 24 weeks between treatment groups. Larger studies are needed to confirm the clinical impact of our findings.

**Reference**

1. Scévola S, Ghinelli F, Sartori C, Galli L, Cafaro V, Fanti I, et al. Decay of HIV RNA in seminal plasma and rectal fluid in treatment‐naive adults starting antiretroviral therapy with dolutegravir plus lamivudine or bictegravir/emtricitabine/tenofovir alafenamide. J Infect Dis. 2023;228(7):919–25.

### P387 Assessing the utility of a simple respiratory symptom screening tool in an outpatient population of people with HIV

Andrew Read ^1^, Rob F Miller^1^, Fiona M Burns^2^, Jane Akodu^2^, Sarah Edwards^2^, James Brown^1^, Marc Lipman^1^, Tristan J Barber^2^

^1^Respiratory Medicine, Royal Free Hospital, London, UK. ^2^Ian Charleson Day Centre, Royal Free Hospital, London, UK

**Introduction**: People with HIV (people) experience greater morbidity and mortality from age‐associated, non‐communicable pulmonary conditions such as chronic obstructive pulmonary disease (COPD) and lung cancer. This results from higher rates of smoking than in the general population, the HIV associated pro‐inflammatory state in the lungs, and increasing life expectancy [1]. Whilst current European HIV guidelines suggest annual screening for respiratory symptoms [2], there is a lack of similar guidance in the UK.

**Materials and methods**: We adapted a simple questionnaire taken from European guidelines to assess the burden of respiratory symptoms in people attending ambulatory care at our London HIV clinic. The questionnaire was distributed in the waiting room of an ambulatory centre over a 3‐week period and could be completed in under 5 minutes; participation was optional. Service users were asked whether they regularly experienced shortness of breath on moderate exertion; cough and/or sputum production, or recurrent wheezing over the last few months. We also assessed smoking status, current respiratory infections and previous respiratory diagnoses. Results were collated after 3 weeks. Data from questionnaires was cross‐referenced with electronic patient and GP records to assess for previous respiratory investigations including thoracic CT, lung function testing, and echocardiography; as well as current follow up with a respiratory or cardiology doctor.

**Results**: Forty‐one of 76 people (54%) who answered the questionnaire reported at least one respiratory symptom with 20 of these (26% of total participants) reporting multiple symptoms (Figure 1). Symptomatic participants ranged in age from 31 to 80 years old (median 60 years old), and over half (56%) were current‐ or ex‐smokers (seven current‐, 16 ex‐ and 18 never‐smokers). Of 41 symptomatic participants, 21 (28% of total) were not being followed up by a respiratory or cardiology doctor for their symptoms, and 16 (21%) had never received any of the respiratory investigations assessed.

**P387: Figure 1:**
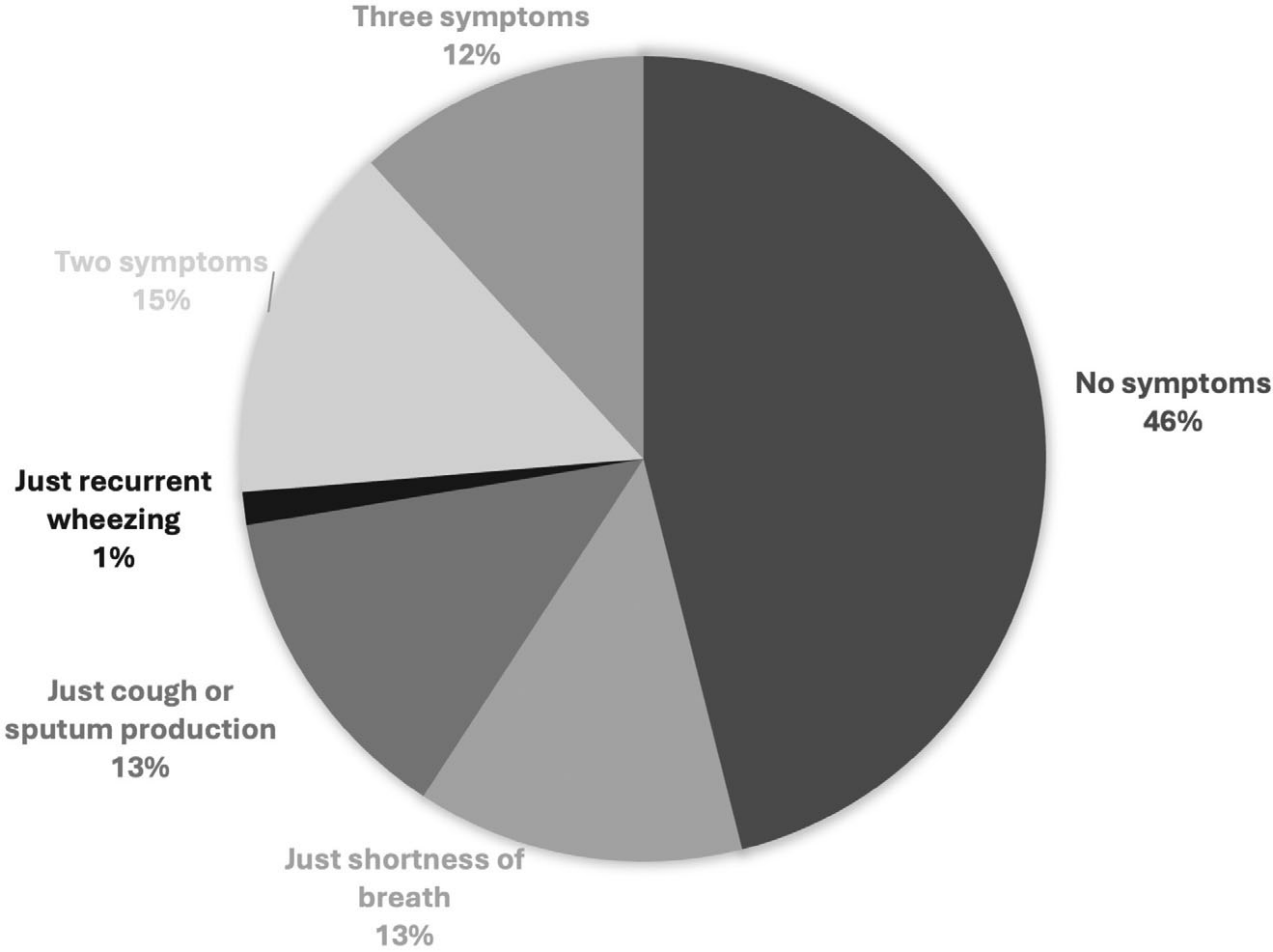
The proportion of people reporting respiratory symptoms (total *n* = 76).

**Conclusions**: In line with previous reports, we continue to identify a high burden of respiratory symptoms in people with HIV, many of which remain un‐investigated. Further work will look to identify the aetiology of these symptoms, and develop an appropriate pathway to efficiently manage and investigate this unmet need.

**References**

1. Konstantinidis I, Crothers K, Kunisaki KM, Drummond MB, Benfield T, Zar HJ, et al. HIV‐associated lung disease. Nat Rev Dis Primers. 2023;9(1):39.

2. European AIDS Clinical Society. EACS guidelines, version 12.0 [Internet]. 2023 [cited 2024 Jul 4]. Available from: https://www.eacsociety.org/guidelines/eacs‐guidelines/eacs‐guidelines.html.

### P388 Additional vaccination in PLWH: adherence in a central hospital in Lisbon

António Moreno‐Marques, Fátima Gonçalves, Zélia Rodrigues Sobral, Fábio Cota‐Medeiros

Infectious Diseases, ULSSM Hospital Santa Maria, Lisboa, Portugal

**Introduction**: Vaccination remains essential for protecting people living with HIV (PLWH) from infections and comorbidities. While immunological status generally does not contraindicate most vaccines, it is advisable to vaccinate primarily during periods of viral suppression and immune reconstitution. In Portugal, data on vaccination adherence among PLWH is scarce. This study aims to assess vaccination adherence and identify factors associated with adherence in an urban centre in Portugal.

**Materials and methods**: This observational study reviewed medical records of 150 PLWH attending the Infectious Diseases Department of Hospital Santa Maria between April and June 2024. Data collected included demographics, infection‐related parameters and vaccination history. Information was collected for the following vaccines: tetanus and pertussis, measles‐mumps‐rubella (MMR), hepatitis B, varicella‐zoster, annual influenza, COVID‐19 (seasonal booster) and *Streptococcus pneumoniae*. In addition, information regarding hepatitis A, human papillomavirus (HPV), mpox and *Neisseria meningitidis* was considered according to risk exposure. High adherence was defined as having completed over 70% of the recommended vaccinations, adjusted for risk exposure.

**Results**: The cohort consisted of 74.7% males with a mean age of 48.4 years. The majority were Portuguese (72%) with HIV‐1 infection (97.3%). Primary transmission routes were sexual (MSM 49.3%, heterosexual 36%). Mean infection duration was 13.7 years. All participants were on antiretroviral therapy (ART) with a median CD4 count of 588/mcL and 90% had HIV RNA <50 c/ml. Overall vaccination adherence was 60.1% (95% CI 56.1–64.1), with the highest rates observed for tetanus and pertussis (84.5%), MMR (73%), and hepatitis B (66%). The lowest adherence rates were for varicella‐zoster (0%) and HPV (1.3%). Logistic regression analysis revealed that age over 50 years (aOR 5.11, 95% CI 2.15–12.15, *p* < 0.001) was significantly associated with higher vaccination adherence. Other factors, including gender, nationality, transmission route, initial our current CD4 count and viral load, did not show statistically significant associations with vaccination adherence.

**Conclusion**: While vaccination adherence among PLWH in this cohort is moderately high, there is a significant need to improve coverage for vaccines such as varicella‐zoster and HPV. Targeted strategies to enhance adherence should consider age‐related factors and focus on patient and physician education and support mechanisms.

## AUTHOR INDEX

### A

A. Salem, A P359

Abang, R P371

Abate, M P161

Abbar, B O41

Abberbock, J P037, P208

Abdool Karim, Q O48

Abdou, F P023

Abdulmumin, I P237

Abe, S P309

Abecasis, A O31

Abela, I P246, P253

Abgrall, S P071, P277

Abulizi, D P277

Abusamra, L P256

Acevedo‐Quiñones, M O49

Achayo, H P176

Acsai, M P098

Adair, G P174, P192, P298, P341

Adamis, G P089, P124

Adedeji, O P237, P260

Adelakun, A P160*, P264*

Adriana, F P380

Adriantsoanirina, V P277

Adriaque Lozano, A O47, P014, P015

Adrover, A P106

Aebi‐Popp, K P002

Afani, A P105*, P181

Afzal, S P300

Aghaizu, A O42, P019, P324, P336*, P384

Aguilera García, M P064, P065, P079, P081, P096, P104

Agwu, A O49

Agyemang, Z P127

Ahmad, S P286

Åhsberg, J P370

Ainemukama, J P184*

Aissi, E P136

Ait‐Khaled, M P047

Aizen, K P283

Ajeh, R P161, P190

Akhigbe, M P371*

Akodu, J P127*, P387

Akolo, M P167

Aksak‐Was, B P180

Al‐Shakarchi, Y P279

Alagaratnam, J P317

Albendin‐Iglesias, H P151

Albertini, M P132

Albini, L P121

Alcamí, J P129

Aldamiz‐Echevarría Lois, T P065

Aldebes, P P280

Alejos, B P048

Aleksova, M P344

Alemany, L P356

Alessandri‐Gradt, E P133

Alexiev, I O31

Algar, C P099

Algarte Genin, M O41

Al‐Hayani, A P099, P154, P381

Ali, B P191, P359

Alkhalaf, A P191

Allavena, C P041, P059*, P122, P123, P277*, P323

Allegre, T O41

Alleman, L P061, P083

Allerton, L P257

Almahfoud, M P031, P111, P141, P259

Almeida, M P085*, P250*, P274

Almeida, M P085, P274

Almeida, S P032

Almeida, V P024

Almutairi, R P191

Alonso, G O45

Alonso Moreno, B P104

Alonso Serena, M P026, P239

Alosaimi, R P191*, P359*

Alqurashi, M P191

Alrowais, H P012

Alsaeed, A P191

Alshelawi, M P191

Alsubaie, A P191

Altclas, I P239

Álvarez‐Álvarez, B P099, P376, P381

Álvarez‐Ríos, A P223

Alves, H P158

Alves, L P220

Amador, C P048

Amara, A P196

Amari, S P102

Ambe Chenwi, C P053, P161

Ambler, W P095, P165

Ambrosioni, J P075, P080, P125, P129, P228, P244, P269

Ameil, L P282

Amoabeng Ortsin, E P231*

Amondarain, N O46B

Amstutz, A P307

Amundaba, N P217

Anand, A P326

Anderson, J P317, P333, P348

Anderson, K P047

Anderson, P P018

Anderson, S P264

Ando, N P309*, P362

Andreu, A P106

Angioni, G P066, P137, P306

Ansaldo, L P107, P357

Antinori, A P006, P030, P041, P077, P143, P177*, P178, P179, P346*, P347

Antinori, S P177

Antoniadou, A P124, P318

Antoniak, S P008, P268

Antunes, C P085, P250, P274*

Aoki, T P309, P362

Apea, V P348

Appiah, A O47, P014, P015, P029

Aquilini, D P086

Araújo, S P034

Arbonés, L O45

Arbune, M P342

Archuleta, S P025, P368

Arcos, M O45, P065

Arena, D P147

Arenas García, V P065, P079, P104, P189

Arenas‐Pinto, A P295

Arevalo Calderon, G P044

Arístegui, I P230

Armenia, D P130, P161

Armiñanzas Castillo, C P064, P079

Arnaiz de las Revillas Almajano, F P065

Arns‐Glaser, L P248*

Arora, P P049*

Arreba, P P075, P080

Arribas, J P179

Arribas Lopez, J P043

Artigues Serra, F O43

Aryatuha, I P328*

Asante‐Appiah, E P042

Asensi, V P215

Ashinyo, A P231

Aslangul, E P102

Assoumou, L O41, P008, P041, P122, P133, P179, P323

Astriti, M P089, P124

Ataide de Lima Nascimento, N P146

Ateba, F P161, P190

Attala, L P284

Atujuna, M P060

Auer, N P017, P313

Augello, M P113

Augustovski, F P265

Avettand‐Fenoel, V P133

Avihingsanon, A O32, O49, P149, P283, P308, P369, P373*

Avihingsanon, T P308*

Ávila‐Funes, J P198

Awosiku, O P233

Ayafor, C P161

Ayakaka, I P005

Ayala Vargas, V P383

Ayer, T P263

Ayisi Addo, S P231

Ayres, S P286

Azeredo, C P315

Azwa, I P149

Azzolino, D P316

### B

Babatunde, A P233*, P260*

Babatunde, Y P237*, P260

Bacelar, B P076, P093, P100

Bacelar, C P034, P109, P315

Bacha, A P161

Bachelard, A P350

Badalucco Ciotta, F P288

Baeten, J O21, O49

Baiguera, C P202

Bailey, H P002

Baixeres, N P356

Bajda, T P229

Bakaya, M P194

Balayan, T P168*

Balcells, M P251

Baldé, A P133

Baldé, R O41

Baldin, G P186

Ballivian, J P044, P265*

Balogh, A P302, P325

Bame, B P196

Bamford, A P001, P003

Ban, J P160

Bana, N P074, P202

Bandera, A P306

Bang, A P225

Bani‐Sadr, F P122

Bannister, W P272

Bapabi, M P196

Baptista, T P250, P274

Baquero, L P214

Barbareschi, G P379

Barbaro, F P030

Barber, T P127, P387

Barbini, B P278

Barbosa, T P203

Bareng, O P131, P150

Bargiacchi, O P066, P306

Barkat, S P041

Barkins, R P163

Barletta, J P092*, P183, P185, P239

Baron, M O41

Barrera, F P316*

Barskiy, K P270, P363

Bartalesi, F P086

Bartalucci, C P074

Bartoloni, A P086, P353

Basile, R P299

Basoulis, D P207

Basova, A P094*, P108*

Bassini, A P212

Batista, C P010*, P034, P109, P315

Bauer, W P017, P354

Baumgartner, C P115*

Bécares, J P099

Beer, D P063

Begovac, J O31, P155, P216, P297, P334

Behrens, G P179*

Bekker, L O48*, P060

Belak Škugor, S P297

Belinchon, O O45

Bellagamba, R P066

Bellocchi, M P130

Bellomo, M P120, P291

Bellón, J P376

Beloukas, A P207

Beloumou, G P053, P161

Beltrán, C P181

Belus, J P194

Belvisi, V P007

Ben Rayana, R P323

Benabadji, E P263

Benalycherif, A P323

Bendezu Mejía, K P110

Benevolo, M P314

Benfield, T P281, P293, P300

Beniguel, L P041

Benito, J P381

Benkouiten, S P206

Benković, I P216, P297

Benson, C P211

Benson, P O49

Bento, D P024

Berenguer, J O45*, P376*

Bergeron, A P271

Bergin, C P203

Bergman, M P093, P100

Berhe, M O21

Bering, L P281

Berini, C P038

Bermejo Plaza, L P073, P104

Bernal, E O45, P048, P056, P064, P065, P079, P081, P096, P104, P337

Bernardini, C P016

Bernardino, J P081, P294*

Bernasconi, E P016, P115

Berrocal, L P075, P080, P125, P129, P228, P244, P269

Bertheau, M O41

Berzow, D P141

Bessissow, T P038

Betkova, S P024

Biala, M P361

Bickel, M P139

Bidner, H P302

Bienkowski, C P261

Biering‐Sørensen, T P300

Bihan, K O41

Bikhezar, F O31

Bini, T P074, P103

Bisbal‐Pardo, O P073, P189

Bitira, L P162*

Blain, H P277

Blais, L P271

Blanc, P P086

Blanco, S O46A

Blanco, J P096

Blanco‐Arevalo, J P043, P048, P075, P080, P129, P228, P244, P269

Blankenberger, J P194*

Bletsa, M P124

Blick, G P330

Bloch, M P050

Bloch, M P102

Blomme, E P054

Blumenthal, J O49

Bobbio, N P143

Bochena, L P118

Bociąga Jasik, M P180

Bock, A P300

Bocket, L P133, P136

Boehm, M P039

Boesecke, C P302, P325

Boffito, M P068*, P279, P290, P333

Bogart, M P163

Bognar, F P154

Böhm, M O31, P340

Boit, B P195*

Bonafede, M P067

Bonfanti, P P066, P074, P306

Bonilla, M P099

Bonnafous, P P133

Bonnet, F P061*, P078*, P083, P277, P307

Bono, V P113*, P178

Bonura, C P147

Borghetti, A P287

Borghi, F P128

Borghi, V P245

Borjabad, B P129*

Borralho, J P250

Borse, R P149

Bortkiewicz, J P097

Boschi, C P206

Botanelli, M P307

Bottaro, E P076, P093, P100

Bouba, Y P053, P161, P190

Bouteira, G P102

Boutselakou, E P089

Bowonwatanuwong, C O32

Boyachuk, B P280

Bozejko, M P022, P097

Brady, M P249

Braun, D P016, P115, P254

Braun, P P142

Bravo, J P048

Brawley, D P257*

Brazal, B P376

Bregigeon‐Ronot, S P071, P206

Breitschwerdt, S P302

Bresser, M P193

Breuer, S P218

Brillat, S P242

Brinson, C O49, P211

Brites, C O24, O49

Brizuela, M P076

Brochot, E P133

Brogan, A P101

Brooks, G P243

Brown, A P336

Brown, J P005, P193, P194

Brown, J P387

Brown, K P037

Brown, K P040

Brown, L O49

Brunet, L P285

Brunet, M P125

Brunetta, J P068, P098

Bruno, R P090

Bruno Pena Gralle, A P271

Bruun, T P011

Bruzzesi, E P077, P289*, P311*

Bruzzone, B P121

Büch, J P340

Buchholz, A P259

Buech, J P039

Bugyi‐Bozo, N P370*

Bukasa, L P003

Bukusi, E P174

Burgard, M P058

Buria, L P229

Burillo, I P099

Burioni, R P311

Burk, C P213

Burns, F P020, P127, P227, P304, P317, P333, P385, P387

Buzon Martin, L P056*, P065, P079*, P081, P104, P189

Byrne, L P003, P004

Byrne, R P290

### C

Cabello, A P057, P081, P099*

Cabello Clotet, N O43, P056, P064, P065, P079, P081, P104, P151

Cabello Úbeda, A P056, P064, P065, P079, P104, P189, P381

Cabie, A P277

Cabrera, P P266

Cabrera, T P175*

Caby, F P071

Cachapa Viera, I P303

Cacopardo, B P090

Caetano, I P374

Cafaro, V P327

Cahn, P O24*, O49, P021, P209, P230

Cai, L P156*

Cai, W P045, P046, P052, P055, P197, P292, P305, P321, P349, P365

Caixeta, D P355

Calandra Buonaura, F P312, P331, P366*

Calcagno, A P205, P316, P347

Caldas, C P112

Calderón, M P330

Calderón Hernáiz, R P056, P064, P065, P079, P081, P104

Calì, C P137

Callaghan, L P163

Callebaut, C P035, P119

Calle‐Gómez, I P358

Calleja, M P151

Calmy, A P016, P115, P204

Calvo, J P075, P080, P129, P228, P244, P269

Calza, L P120

Calza, S P316

Calzado Isbert, S P215, P303

Camacho Espejo, A P215

Cambiano, V P011, P384

Camici, M P289

Campbell, K P264

Campbell, L P278

Cañas Ruano, E P043

Canavesi, G P074*

Candela, C P291*, P311

Canetti, D P041

Cantos, V O49

Capelli, C P016

Capodieci, S P327*

Cappell, K P067

Cappelli, C P332

Caraballo, J P099

Caracik de Camargo, L P146

Cardoso, O P024

Cardozo, F P026, P239

Cardozo, N P230

Caria, J P024

Carioti, L P130

Carlander, C P307

Carleo, M P066, P306

Carles, M P282

Carlos, M P024

Carmezin, J P356

Carobbio, A P331

Caro‐Vega, Y P198

Carraro, A P107*, P289, P357

Carrillo Acosta, I P099, P381

Carrozzo, G P077

Carstens, R P320

Carter, C O48, O49

Carter, D P262

Cart‐Richter, E P255

Carvalho, R P112, P377

Casado, J P069, P072, P144, P145

Casado, M P381

Casari, F P171, P312

Cascio, A P066, P147, P306

Casseb, J P146

Cassetti, I P076, P093, P100, P181, P209

Cassidy, T P068, P095, P098

Castagna, A P030, P041, P120, P130, P135*, P205, P284, P288, P291, P299, P307, P311, P334, P347

Castaneda, J P023

Castañeda, F P251

Castel, M P125

Castelli, A P125

Castelli, F P332

Castillo‐Navarro, A P337

Castro, A P266

Castro, J P067

Castro, V P377

Catalano, G P288

Catania, J P013

Cattelan, A P170

Cavassini, M P115, P246, P253

Cavazza, G P074, P202

Cazanave, C P061, P083, P350

Ceballos, M P251*

Ceccherini‐Silberstein, F P077, P161, P178, P205

Cecchini, D O24, P076*, P093*, P100*, P239

Cecere, S P030, P240

Cecilia, F P346

Cecilio, Á P056, P064, P065, P079, P081, P104

Celemente, T P120

Celesia, B P090, P137, P299*, P306, P316

Cenderello, G P066, P284, P306

Centorrino, F P074

Ceppi, C P282

Cerezales Calviño, A P056, P064, P065, P079

Cernuschi, M P030, P311

Cervo, A P077, P132*, P171*, P245

Cesar, C P021, P230

Ceschel, M P230

Chahin, C O44

Chahroudi, A P192

Chaiwarith, R P149

Chaiyakunapruk, N P051

Chakela, M P005

Chakona, S P196

Chakraborty, S P208

Chammartin, F P194

Chan, J P351

Chang, S P119

Charlton, R P165

Charpentier, C P082, P133

Chebani, T P118

Chen, B P160

Chen, G P319*

Chen, H P149

Chen, J P219*

Chen, J P301*

Chen, P P152

Chen, T P301

Chen, W P208

Chen, W P352

Chen, Y P045, P046, P052, P055, P292, P305, P321, P349, P365

Chen, Z P045, P052, P055, P292, P305, P321, P349, P365

Chen, Z P070

Cheng, C P152, P301

Cheng, D P159

Cheng, F P267

Cheng, S P152, P301

Chetchotisakd, P O32

Chiewbangyang, F P308

Chik, T P351

Chini, M P124

Chirio, D P282

Chivite, I P075, P080, P129, P228, P244, P269

Chkhonia, E P329

Choga, O P117, P118*

Choga, W P117, P118, P131, P150

Choi, J P149

Choi, S P198

Choi, Y P351

Cholewik, M P261

Chomont, N P038

Chow, J P154

Christinet, V P016

Chromy, D P017, P313*, P354*

Chrysos, G P124

Chu, D P148

Chu, X P320

Chuang, Y P319

Ciccullo, A P287

Cielniak, I P180

Cimini, E P346

Cingolani, A P006, P143, P205, P307

Cipriano, A P032

Cirelli, T P090

Cirioni, O P006

Ciullini, L P204*

Clark, A P116, P135

Clark, J O49

Clavero Olmos, M O45, P056, P064, P065, P079, P104

Clement, M O49

Clemente, T P130, P284*, P291

Clutterbuck, D P249

Coco, V P090

Cofan, F O46B, P125, P322

Coghlan, M P203

Cohen, C P283

Coin, A P316

Cola, M P170

Colagrossi, L P205*

Colangelo, C P332

Colavita, F P346

Cole, S P177

Cole‐Haley, S P174

Colizzi, V P053, P161, P190

Collado, A P151

Collins, S O23

Colson, A O21*

Colson, P P206

Comi, L P128

Conti, F P121*

Conti, G P090, P137

Converso, D O24

Conway, B P187, P188

Cook, P O23*

Cooper, C P280

Copas, A P295

Copertari, G P076

Corazza, S P007, P107, P357

Corda, L P137

Cordel, H P063

Cordie, A P359

Cordova, E P044*

Corkin, H P019

Corona Mata, D O43, P215

Corps, D O45

Corrales Rodríguez, M P383

Correia de Abreu, R P010, P034, P109, P112, P315

Corsi, P P086, P353

Corsini, D P121

Cortes, C P209

Cossu, M P132, P287, P289

Costa, C P178

Costa, F P353

Costagliola, D O41, P071

Costantini, A P006

Costarelli, S P086

Costescu, C P134, P378

Costiniuk, C P038

Cota‐Medeiros, F P388

Cottrell, M O22

Coukan, F P020*, P227*, P385

Coulon, P P172

Courvoisier, N P173

Coutinho, D P112

Cox, S O49

Cozzi‐Lepri, A P121, P143, P177, P178, P205, P346, P347*

Cozzolino, C P170

Crabtree‐Ramírez, B P198*, P209

Craik, J P262

Crespo, G O46A, P125

Creticos, C O49

Crisinel, P P246, P253

Cristofanelli, F P346

Cristóvão, G P024

Crofoot, G O21, O23, O49

Cruces, L P214*, P256*

Crusells‐Canales, M O43, P047, P048, P056, P057, P065, P104, P189

Cruz, A P175

Cruz, C P024

Cruz, G P236

Cua, E P282

Cucchiari, D O46B, P322

Cuevas Tascón, G P104

Cullen, L P385

Cuomo, G P245

Cureg, B P213

Curran, A P048, P179

Curtis, A P249

Curtis, H P249

Czernikier, A P214

### D

D:A:D (The Data Collection on Adverse Events of Anti‐HIV Drugs Study) P307

Dächert, C P142

Dai, L P114, P154

Dakic, Z P222

Dalmau, R P294

Damas, J P173, P246, P253

D'Amico, F P202

Dams, M P163

Damtham, P P308

Danchenko, N P264

Daneluzzi, V P102

Dannenberg, C P111*

Dantal, J P323

D'Antoni, M P119*

Darcis, G P201, P232

Darling, K P016, P173, P246, P253, P254, P255*

d'Arminio Monforte, A P006, P030, P077, P143, P178, P205, P289

Das, M O48, O49

Däumer, M P126, P142

D'Avanzo, P P166

Davis, A P264

Dawaher, J P181

Dawiec, M P097*

De Barra, E P068

De Benedittis, S P077, P347

De Castro, N P071

De Cosmo, P P331

De Gennaro, M P086

de la Calle Riaguas, B P056, P064, P065, P079, P104

de la Cuadra‐Grande, A P381

de la Fuente Moral, S O43, P027, P048, P065, P079, P104, P360, P383*

de la Mora, L P075, P080, P129, P228, P244, P269

de la Rosa, G O46B

De la Torre, J P337

de Lagarde Sebastian, M P043*

de Lazzari, E O46B, P075, P080, P125, P129, P228, P244, P269

de los Rios, P P040

de los Santos, I P056, P096, P151, P376

De Maria, S P007*, P107, P357

de Mendonça Melo, M P210

De Miguel, M O45, P043, P048

de Miguel Buckley, R P043

De Monte, A P133

de Oliveira Nunes, F P220

de Oliveira Pereira, L P146*

De Pokomandy, A P148*

de Ruiter, A P285

De Scheerder, M P054

De Socio, G P066, P130, P306

De Vito, A P074, P137*, P143*, P287

de Vries, C P211*

De Wit, S P008, P179

de Zárraga Fernández, M P056, P079, P081, P104

Deaton, C O49

Deb, S P317*

Decosterd, L P204

Degen, O P031, P111, P141, P259, P334

Degrendel, M P136, P172

Degroote, S P054*

Dehlbæk Knudsen, A P281, P293, P300

DeJesus, E O23, P211

Del Arco, A P337*

Del Borgo, C P007, P107, P357

Del Campo, S P069, P072, P144, P145, P376

Del Negro, L P240

Del Punta, V P170

Delbarba, A P332

Delesie, L P054

Delgado Vazquez, R P043

Delobel, P O41, P122

Delpech, V P336

Dema, E P001*

Derdour, V P136

Deschanvres, C P071, P123

Desclaux, A P078

Destordeur, L P201, P232*

Devendra, A P194

Devisich, G P209

Devlin, S P023

Dhairyawan, R P348

Dhippayom, T P051

Di Biagio, A P006, P066, P074, P284, P306

Di Felice, A P353

Di Gennaro, E P202

Di Giambenedetto, S P186, P287

Di Gregorio, M P332

Di Gregorio, S P048

Di Rosolini, M P137

Diarra, A P136*

Dias, D P212

Diaz, R O31, O49, P212*

Diaz Almidon, M O43

Díaz de Santiago, A P027*, P056, P064, P081, P189*, P360*, P383

Díaz Martín, E P303*

Diaz‐Brito, V P048

Dickinson, L P196

Diekhoff, P P031

Diekmann, F P322

Dieterich, D P285

Diez Romero, C P056, P079

Digaetano, M P245

Dimitrova, R P344

DiMondi, V P047

Dinkel, A P325

Diotallevi, S P284, P288, P311

Ditangco, R P149

Ditshwanelo, D P131, P150

Djeneba, F P133

Djibuti, M P329

Djupsa, S P161

Dmitriev, I P271

Doblecki‐Lewis, S O49

Doctor, J P317

Dolph, M P264

Dominelli, F P357

Domingo, P O45, P041, P048, P179, P307

Domínguez de Landa, A P251

Donà, M P314, P327

Dorama, W P182

Dos Santos, M P206

Doutre, M P271

Doyle, N P317, P333

Dragne, M P134

Dretler, A O21

Dronda, F P072, P144

Drumel, T P123*

Du, F P116, P135

DUALIS Study Group P325

Duarte, F P010, P034*, P109*, P112, P315

Duarte Rocha, S P315

Dube, K P213

Dubois, S P102

Ducci, F P120

Dudoit, Y P041, P122

Dueñas, C P048

Duffau, P P061, P078, P083

Dumitru, I P342

Dunbar, M P163*

Dundas, P P257

Dunham, R P208

Duppenthaler, A P246, P253

Duracinsky, M P213

Durán, A P230

Durant, J P282

Duvivier, C P041, P058, P063, P071, P122

Dvory‐Sobol, H O21

Dyson, A P116, P135

### E

Eberle, J P142

Ebrahimi, R O48, O49

Edali, M P263

Edwards, J P177

Edwards, S P304, P387

Egert‐Schwender, S P302

Egido Murciano, M P056, P065, P079, P104, P189

Egle, A P307

Ehret, R P141, P142, P218

Ekundayo, I P233

Ekwoge Mejane, R P161

El Khalili, O P205

El Moussaoui, M P201, P232

El Sakka, N P257

Eland, N P163

Elbirt, D P212

Elecito, K P290

Elias, A P062*, P088*

Ellis, R P225

Elnahas, G P359

El‐Sadr, W P307

Else, L P196

Elsner, C P142

Elzi, L P246, P253

Emilova, R P344*

Endo, T P068

Ene, L P002, P378

Engel, N P126

Engler, K P148

Ensor, S P196

Eron, J O21, O23

Esforzado, N P322

Eshetu, A P217

Esparis, M P099

Espinosa, N P084, P223

Espinoza, M P251

Essamba, S P161

Esser, S P142, P313, P340

Esteban, S P265

Esteban‐Cantos, A P294

Estébanez, M P056, P064, P065, P079, P104, P294

Esteves, C P377

Ezeh, I P338*

### F

Fabbiani, M P086, P287

Fahmy, H P359

Fainguem Nguendjoung, N P190*

Falasca, K P066, P306

Falkard, B P035*

Falvino, C P357

Fanciulli, C O45, P064, P215

Fanjul, F P048

Faqih, L P191

Faria, F P032, P374

Fariñas, C O45

Farnham, A P016, P248

Fätkenheuer, G P340

Fearnley, N O42

Feasi, M P120

Federici, L P331

Fehr, J P016, P248

Fernandes, G P002

Fernández, A O22, P386*

Fernandez‐Velilla, M P294

Fernsler, D O44

Ferra, S P048

Ferrand, H P078

Ferrando, S P282

Ferrante, E P103

Ferrara, M P130, P316

Ferrara, S P066, P299, P306

Ferrari, A P170

Ferraris, C P166

Ferreira, A P377

Ferreira, J P304

Ferreira Pasos, E P056, P064, P065, P079, P081

Ferrero, O O45

Ferrés, M P251

Fidler, S P286

Figueroa, M O24, P021, P209*, P230*

Fijołek, F P261*

Fink, V P021, P230

Finkelshtein, E P212

Fiorelli, C P353

Fioretti, B P316

Fisicaro, V P090

Fletcher, C P008, P179

Fletcher, D P098

Floridia, M P002

Flower, K P211

Focà, E P074, P120, P316, P332

Fognani, M P086

Fokam, J O31, P053, P161, P190

Folguera Olías, C P383

Fombellida, K P201, P232

Foncillas, A P075, P080, P129, P228, P244, P269

Fondevila, C O46A

Fong, T P324

Fontana, B P132, P245, P312

Fontana Del Vecchio, R P006

Forgwei, L P161

Formica, G P086, P287, P353

Fornabaio, C P130

Forner, A O46A, P125, P322

Foster, C P192, P286

Fouere, S P350

Fox, D P330*

Francalanci, E P086, P353

Francavilla, A P128

Franch, R O45

Francis, K P001, P004

Francisci, D P287

Franco, C P238

Franco‐Rodríguez, J P198

Francos, J O24

Franklin, D P225

Friedman, E P023

Frikke‐Schmidt, R P300

Fu, H P045, P052, P055, P292, P305, P321, P349, P365

Fu, Y P087

Fuchs, A P281, P293

Fuglsang Kofoed, K P281, P293

Furdal, M P097

Fursa, O P272*

Fusco, F P030

Fusco, G P285

Fusco, J P285

Fuster, J O46A

Fusto, M P346

### G

Gabisa Jeremiah, E P161

Gaggioli, S P353

Gagliardini, R P077, P130, P177, P346

Galarza, P P021

Galindo Puerto, M O45, P048, P056, P064, P065*, P079, P081, P104*, P151

Galinskas, J P212

Gallardo‐Cartagena, J O49

Gallego, B P099

Galli, L P130, P299

Gallien, S P323*

Gallo, M O43, P215

Gamell, A P002

Gan, E P368

Gan, L P087

Ganci, I P147

Gandhi, M P154

Gandhi‐Patel, B P154

Gandini, F P311

Gandolfi, F P331

Gani, Y P149

Garattini, S P357

Garcia, B O22

Garcia, F O31

Garcia, J P021

Garcia, M P069

García, C O45

García, L P099

García Abellán, J P104

García López, M P081

Garcia Martearena, M P288

Garcia Martinez, C P358

García Navarro, M P056, P064, P065, P079, P081

Garcia‐Fraile, L P048

García‐López, M P364

García‐López, V O25

Garcia‐Marti, S P265

García‐Ruiz de Morales, A P215

Garcia‐Valdecasas, J O46A

García‐Vallecillos, C P096, P151

Garcinuño Jiménez, M P056, P064, P065, P079, P081

Gardin, S P316

Garges, H P307

Garner, A P276

Garnier, E P123

Garrote, R P024

Garside, L P040

Gartland, M P037*, P208

Garvey, L P091

Gaseitsiwe, S P117, P118, P131, P150

Gasiorowski, J P022, P097

Gasparro, G P086

Gasperin, A P007, P107

Gatanaga, H P309, P362

Gattiker, M P254

Gaudiano, R P147

Gaur, A O49

Gauthier, M P213

Gazzola, L P074, P103

Gemmell, O P012

Gennari, W P121

Gérard, Y P061, P078, P083

Geringer, E P142

Gerstoft, J P293

Gervasoni, C P132

Gharib, Y P295*

Ghidoni, E P312

Ghiglione, Y P214

Ghosn, J P071, P122, P154*, P350*

Giachè, S P086

Giacomelli, A P120, P132, P143, P177, P284, P287, P334

Giambi, C P357

Giannou, F P124

Gianserra, L P314, P327

Giarratana, C P090

Gibowski, S O41

Giglia, M P030

Gil, P O43, P048

Gilbert, M P350

Gilbert, V P019

Gili, S P346

Gilles, I P255

Gillespie, G P224*

Gilson, R P295

Giner, L O45

Gioè, C P147

Giolma, K P098

Giorgi Rossi, P P314

Girardi, E P006, P346

Girometti, N P009*, P279

Githaiga, J P247

Giuliani, E P314, P327

Giuliani, M P314, P327

Glass, T P193

Glaum, L P242

Glenn, J P286

Gliga, S P340*

Gmizic, I P222

Gnambi, A P161

Gobe, I P118

Godemert, M P282

Godin, M P271

Gomes, P O31

Gomes‐Pires, E P058

Gómez‐Ayerbe, C O25, P084, P364*, P367

Gonçalves, A P024

Gonçalves, F P238

Gonçalves, F P388

Gonçalves, M P109, P315

Gonzalez, A O46B, P125

Gonzalez, A P175, P181

González‐Cordón, A P075*, P080*, P129, P228, P244, P269

González‐García, J O45, P376

Gordon, L P040

Górgolas Hernández‐Mora, M P057, P099, P154, P381

Gorgos, L O23

Gori, A P178

Gorska, A P210

Görzer, I P142

Gouissi Anguechia, DH P053*, P161, P190

Goyal, A P326

Grabar, S P071*

Grabinger, T P254*

Grabmeier‐Pfistershammer, K P017, P307, P354

Greco, M P181*

Greenberg, D O44

Greenberg, L P307

Greenhalgh, F P379

Gregori, N P116, P135

Greillier, L O41

Gridel, P P290

Grier‐Gavin, R P091

Griesel, R P037

Grijalva, C O44

Grillo, C P074

Grimaldi, A P007, P107

Grinsztejn, B O49

Grove, R P047, P330

Gruber, J P067, P095

Gruters, R P210

Guaraldi, G P074, P132, P171, P245, P289, P312, P316, P331*, P347

Guardiani, M P357

Gudiño Solorio, H P382

Guebiapsi Tameza, D P161

Guerrero‐Tobias, M P198

Guerri‐Fernandez, R P048

Guida, A P289

Guillaume, X P163

Guilleminot, J P023

Guilorit, L O31

Guimarães, M P315

Guimond, T P012

Guinan, K P153

Gullotta, C P090

Gulminetti, R P120

Gun, A P230

Günthard, H P115, P334

Guo, L P070

Gupta, N P249

Gupta, P P326*

Gupta, S P067

Gursel, E P263

Gutiérrez Cuadra, M P056, P104

Gutiérrez Liarte, A P043

Gutierrez‐Valencia, A P223

Guze, M P243

### H

Haberl, A P057, P139

Hachfeld, A P115, P246, P253

Haenggi, K P005, P193

Haerry, D P016, P254

Hakozaki, T P038

Hameed, S P304

Hamlyn, E P278, P304*

Hampel, B P016*, P248

Hamzah, L P179

Hansen, U P242

Hanssen, J P182

Hardy, A P338

Hardy, I P264

Harper, G P101

Hart, J P127

Hartard, C P133

Hasson, H P288, P291

Hauser, A P142

He, Q P070

He, S P045, P052, P055, P292, P305, P321, P349, P365

He, Y P159

He, Y P197

Hedgcock, M P050

Heekin, A P203*

Heger, E P142, P340

Heinzkill, M P063

Henderson, H P234*

Henderson, M P286*

Henegar, C P285

Hennessy, F P029, P095, P165*

Hensley, K P210*

Herieka, E O42

Herms, F P350

Hernández Gutiérrez, C P043, P215

Hernández López, A P383

Hernandez Rendon, J P044

Hernández‐Ruiz, V P198

Hernando, A O45

Hernando, M P073

Herrero, M P223

Herrmann, E P139

Heskin, J P290*

Hessamfar, M P061, P078, P083

Hetman, L P272

Hickens, N P148

Hidalgo, A P084

Hidalgo Tenorio, C P057, P096*, P151*, P358*

Higgen, S P259

Hindman, J P036, P283, P373

Hinojosa, J O49

Hiranburana, N O32*, P369*

Hirota, K P226

Hlasoa, M P193

Hlebowicz, M P180

Ho, B P225*

Hoan, D P122

Hocqueloux, L P059, P082

Hodge, T O49

Hodgson, I P379

Hoerst, C P019*

Hoffmann, C P142, P334

Holbrook, T P029, P095, P165

Holder, J P384

Holtveg, T P300*

Homar, F P106, P296

Hosein, S P307, P379

Hower, M P340

Hrytsaiuk, N P272

Hsieh, S P319

Hsih, W P301

Hsue, P P283*

Htun, W P276*

Hua, W P114

Huang, C P352

Huang, H O23

Huang, J P197

Huang, K P197

Huang, M P301

Huang, S P152

Huang, S P211

Huang, X P013

Huang, X P221

Huang, Y P152*, P319

Huber, R P194

Huefner, A P031, P259

Hulin, A P323

Humphreys, C O42, P019, P336

Hung, C P152, P319, P373

Hunstig, F P259

Hunter, A P127

Hunter, J P212

Hurtado, C P129

Hutchinson, S P262

Hüttig, F P340

Hutton, C P249

### I

Ianache, I P134*, P378

Iannantuono, M P021

Ianniello, A P143

Iannone, V P186, P287*

Ida Penda, C P190

Idelovici‐Marchal, C P263

Iglesias, J P073

Illán Ramos, M P002

Imaz, A O22*, P043, P386

Imerlishvili, E P329*

Inciarte, A P043, P075, P080, P129, P228*, P244*, P269*

Indarodom, R P308

Inglot, M P097

Iribarren, J O45

Isari, A P124

Ishak, J P148

Isnard, S P038

Izadifar‐Legrand, F P206

Izuzquiza, I P144, P145

### J

Jaber, M P035

Jablonowska, E P180

Jackson‐Perry, D P255

Jadayel, H P286

Jakobsen, M P011*

Jakubowski, P P180

Jarrett, J P213, P263

Jarrin, I O45, P215

Jaschinski, N P334

Javier, R P151

Javorić, I P297

Jayaweera, I P091*

Jennings, L P060*, P166

Jensen, A P300

Jensen, B P142, P340

Jiamsakul, A P149

Jin, Y P339*

Jirajariyave, S O32

Johansen, I P370

Johnson, A P023

Jonas, K O47, P014, P015*

Jones, B P047, P094, P108, P135, P285, P330

Jones, H P095

Jones, S O26

Jooss, K P211

Jordan, S P031, P259

Jordans, C P182*

Jordão, S P010, P109, P315

Jovanovic, S P222

### K

Ka'e, A P053, P161, P190

Kaba, A P122

Kabbash, I P359

Kagia, S P235

Kahlert, C P246, P253

Kaiser, E P154

Kaiser, R O31, P039, P142, P340

Kajaia, M P329

Kalaiwo, A P371

Kaleebi, J P169, P176, P184, P328

Kalinowska‐Nowak, A P180

Kall, M O42, P324, P336, P384

Kalogiannis, A P379

Kamgaing, N P161, P190

Kamgaing, R P161, P190

Kandlen, K P242*

Kang, S P290

Kang, Z P219

Kannike, K P279

Kao, M P005

Kaplan, R O49

Karavasilis, E P318

Karmochkine, M P071, P277

Karris, M P341

Katlama, C O41, P041, P071, P122, P179, P277

Kavatha, D P258, P318

Kawashima, A P309

Kayembe, B P193

Kazali, E P136

Kazooba, P P162, P169, P176, P184, P328

Keane, A P355*

Keegan, M P208*

Kelley, C O49

Kelly, C O42, P324, P336

Kengni Ngueko, A P161

Kerr, S O32

Kertusha, B P357

Ketchaji, A P161, P190

Khan, I P278

Khan, M O26*

Khaykin, P P139

Khodus, I P272

Khol, V P149

Khongpetch, C P369

Khoo, S P196

Khusuwan, S P149

Kiertiburanakul, S P149, P373

Killer, A P340

Kim, C P163

Kim, Y O44

King, H O42

Kintu, A O48

Kirkby, N P293

Kiros, S P353

Kitamura, H P309

Kitt, H O42, P019, P324, P336

Kiwanuka, N O48

Klastrup, V P286

Klein, F P039

Klein, M P038, P280

Klimkait, T P142, P193

Klopfer, S O21, P224, P320

Knechten, H P340

Knops, E P142

Knox, D P098*

Knysz, B P097

Køber, L P281, P293

Koch, T P031

Koduah Owusu, K P231

Koegl, C P325

Kohli, M P295

Kohns, M P246, P253

Kokeyo, A P243*

Koki Ndombo, P P161, P190

Kolstee, J O47, P014, P015

Koofhethile, C P118, P131

Kopo, M P005

Korenev, D P270*, P363*

Korn, K P142

Kosmopoulou, O P124

Kostaki, E P124*

Kottanattu, L P246, P253

Kouyos, R P115

Kovacs, C P098

Koval, A P259*

Koval, T P229*

Kovalevska, M P272

Kowalska, J P008*, P261, P268*, P334

Krabek, R P281

Kreuter, A P313, P354

Kronborg, G P370

Krüdewagen, B P141

Kryshchuk, A P272

Krzyzanowsky, C P078

Ku, T P164

Kulzer, J P243

Kumarasamy, N P149

Kumwimba, Y P033*

Kundro, M P026, P239

Kusejko, K P246, P253, P334

Kuwata, R P309

Kyokunda, L P217

### L

Labhardt, N P005*, P193, P194

Lacerda, M O24, O49

Lachaine, J P153

Lackey, P P285

Lacombe, K P122

Lada, M P124

LaForty, C P160

Lagadinou, M P207

Lagarde, M P073*

Lagi, F P066, P086*, P120*, P287, P353*

Lagiou, P P124

Laguno, M O46A, P075, P080, P125, P129, P179, P228, P244, P269, P322

Lahoulou, R P042, P320

Lakatos, P P038

LaMarca, A O49

Lamb, S P351

Lambotte, O O41

Lampe, F O42*, P324, P384*

Lander, F P009

Langarica, E P175

Langer, T P214

Lanini, S P347

Lanoy, E P071

Lanzafame, M P170

Lapadula, G P077, P205, P347

Lapinski, L P022

LAPTOP Study Group P179

Lara, G P064

Laroche, H P206

Latham, C P040

Latifovic, L P160

Latini, A P314, P327

Lau, A P351

Lau, D P351

Laufenberg, J P325

Laufer, N P209, P214, P256, P380

Launay, O O44, P071

Lavole, A O41

Laymouna, M P148

Lazaro, E P061, P078, P083

Lazo, E P265

Le, T P025, P368

Le Guillou‐Guillemette, H P133

Le Moal, G P061, P078, P083

Le Saux, C P173, P255

Leal, P P250

Leal Santos, M P024

Lebouché, B P038, P148, P153*

Lee, C P164*

Lee, M P149, P373

Lee, M P154

Lee, N P152

Lee, T P194

Lee, V P338

Lee, Y P152

Lehmann, C P340

Lei, Y P197

Leleux, O P061, P078, P083*

Lemee, N P172*

Lemire, B P271*

Lemogang, G P118, P150

Lemos Correia, G P220*

Lengauer, T P039, P142

Lenotti, E P316

Leone, P P037, P208

Leonidou, L P207*

Leporrier, J P102*

Lertpiriyasuwat, C O32

Leserri, F P030

Letang, E P047, P330

Letendre, S P225

Leth, S P370

Leung, C P351

Leverette, B P047*

Levett, T P278

Levi, I O31

Levy, I P068

Li, A P018

Li, A P114

Li, B P116, P135

Li, C P339

Li, J O44

Li, J P159

Li, K P095

Li, L P070, P197

Li, L P114

Li, Q P046

Li, R P114

Li, S P221*

Li, T P373

Li, Y P320

Lichtner, M P007, P107, P178, P357

Liebana, A P106

Liegeon, G P023

Lin, K P319

Lin, S P152

Lin, W P197

Lin, Y P164

Lin, Y P301

Lino, S P024, P112

Liou, B P152

Lipman, M P387

Lisicar, I P155

Lisovoder, N P212

Little, F P166

Little, S O23

Liu, A P224

Liu, J P339

Liu, L P159

Liu, S O21

Liu, W P319

Liu, Y P114

Llamoso, C P224

Llenas‐García, J P056, P064*, P065, P079, P104

Llibre, J O31, P370

Lligoña, A O46A

Llobet, R P075, P080

Lo Caputo, S P074, P177, P205, P284

Łojewski, W P180

Lolatto, R P120, P130, P284, P288, P291, P311

Lombaard, J O44

Lombardi, F P287

Londero, A P170

Long, H P070, P087*

Longo, D P224

Loon Leong, C P373

Lopardo, G P092

Lope, S P081

Lopes, M P158

Lopez, H O46A, P322

Lopez, J P057

Lopez, M P073

Lopez, T P096

López Caballero, M P081

López Cárdenas, S P096

López De Las Heras, M P099

Lopez Delhoulle, V P201*, P232

Lopez Hidalgo, J P358

Lopez Lirola, A P096

Lopez Pineiro, J P110

López‐Centeno, B P376

López‐Cortés, L P223

López‐Cruz, I O45

López‐Íñiguez, A P198

López‐Jódar, M O25, P364, P367

López‐Ruz, M P151

Loring, M P084

Losa, J O45, P048

Losa García, J P056, P064, P065, P079, P081, P104

Losos, J P037, P208

Losso, M O49, P026, P239

Lourida, G P207

Lourida, P P089

Lourinho, J P238*

Loutfy, M P098, P230, P280

Low, N P016

Lozano, A P084

Lu, H P045, P052, P055, P200, P292, P305, P321, P349, P365, P373

Lu, P P152, P164

Lu, Y P163

Lübke, N P142, P340

Lucas Dato, A P064

Ludgero Vasconcelos, A P374

Luedde, T P340

Lundgren, J P272, P307, P334

Lungu, C P210

Luoga, E P193

Lusetti, D P312

Lv, S P114

Lyall, H P001, P003

Lyons, F P355

### M

M. A. Mansour, S P359

Ma, P P045, P052, P055, P157, P292, P301, P305, P321, P349, P365

Ma, Q P225

Ma, S P070, P087

Maan, I P295

Mabanga, T P038

Macias, J P048

Mackie, N P091

MacLaren, A P295

MacPherson, P P018

Maczynska, B P361

Madeddu, G P066, P074, P137, P143, P287, P306, P316

Madera, S O21, P373

Madimabe, M P060

Maes, N P201

Magalhães, S P024*

Mager, D P225

Maggi, F P346

Maggi, P P299, P306

Magiorkinis, G P124

Mahillo, B O46B

Mahíllo‐Fernández, I P099, P381

Maillard, A P133

Mainardi, I P288

Makhesi, T P194

Makinson, A O41, P059, P277

Malano Barletta, D O46A, O46B, P125, P322

Malcontenti, C P353

Maldonado‐Doblado, A P094, P108

Malebranche, D P154

Malena, M P170

Mallolas, J P075, P080, P129, P228, P244, P269

Mallon, P P298, P317, P333

Maltez, F P024, P112

Malvestutto, C P298

Manata, M P024

Mancarella, G P107

Mancheño De Real, V P079

Manchon, R P058

Manda, V P023*

Manfrin, V P170

Mangin‐Ratana, S P282

Manolov, V P344

Mansinho, K P085, P250, P274

Mantilla, M P181

Mantovani, M P331

Manzanares, E P048

Manzardo, C O46A, O46B, P125

Maphorisa, S P150

Marake‐Raleie, N P194

Marangos, M P207

Marañón‐Solorio, K P198

Marathe, D P035, P049, P224

Marcelin, A O41, P041, P133*

Marchegiani, G P130

Marchenko, O P229

Marchesani, P P290

Marchetti, G P077, P103, P113, P130, P177, P178, P205, P289

Marcon, I P116, P135

Marelli, C P006

Marette, A P038

Mariaca, A P092

Maricato, J P212

Marinho Silva, J P236*, P273*

Marino, A P090, P137

Mariotti, D P346

Marocco, R P007, P107, P357

Marongiu, A P063, P068, P095*, P283, P307

Marozin, S P113

Marque Juillet, S P133

Marques, N P238

Marshall, G P127

Marshall, N P249*

Martel‐Laferriere, V P280

Martin, J P073

Martin, P P044

Martin, V P324, P336

Martin, C O44, P307, P334

Martín, C O45

Martin Rico, P P056, P064, P065, P079, P104

Martín‐Carbonero, L P043, P104, P215, P376

Martin‐Cortés, S O25, P364, P367

Martinez, E P041, P048*, P075, P080, P129, P298

Martinez, J P072, P144, P145

Martinez, J P377

Martinez, O P096, P151

Martínez, R O45

Martínez Buitrago, E P181

Martinez‐Chamorro, E P228, P244, P269

Martínez‐Fernández, L P337

Martinez‐Rebollar, M P075, P080, P129, P228, P244, P269

Martini, E P132, P312

Martini, S P066, P299, P306

Martin‐Iguacel, R P370

Martín‐Rico, P P189

Martins, A P377

Maruapula, D P117, P118, P131, P150

Marzolini, C P115, P204

Masari, S P176

Mashele, N P060

Masia, M P048

Masiá Canuto, M P056, P079

Masiello, A P299

Masoero, T P030

Mason, J P295

Massimiliani, E P245

Mastrogianni, E P124, P207

Mata, T P294

Matarranz del Amo, M P064

Mateos, F O45

Mathurin, K P153

Matignon, M P323

Matlosz, B P268

Matos, J P112, P374*, P377

Matsumura, T P226

Matthews, E P264

Matthews, H P031, P111, P259

Matthews, R P224

Mattioli, S P240*

Matusali, G P346

Mauas, R P076

Maulen, C P105

May, C P385

Mayer, K O49

Mayorga, M P084

Mazzotta, V P006, P030, P077, P177, P346

Mbewe, R P348*

Mburu, M P243

McAuley, A P262

McCluskey, S O31

McCormack, S P009

McCoy, B P234

McCully, J P098

McDuling, C P166*

McGarrity, M P018*

McMillan, A P101

McQueen, L P278

Mechie, I P196

Medina, D O22

Medina Valle, A P382*

Meixenberger, K P142

Melica, G P323

Mena, A O43

Mena, M P074

Mena‐de‐Cea, A P266

Ménard, A P059

Menchén Viso, B P383

Menchi, M P104

Mendão, L P379

Mendes, D P274

Méndez, J P032, P112, P374, P377

Mendoza Portillo, E P382

Menegotto, G P132

Meng, X P200

Meng, Y P197

Mengoni, F P357

Menozzi, M P030, P074, P132, P245*, P312

Menzaghi, B P006, P066, P120, P306

Merino, D P048

Merino, I P296

Merkley, B P098

Merli, M P202

Merlin, C P173*, P246*, P253*

Mernies, G O24, P021

Merrill, D P047, P101

Meseiro, A P274

Mesple, T O31, P210, P330

Messeri, D P086

Metallidis, S P089

Metcalfe, B P262

Metzner, K P142

Meurer, A P063

Meybeck, A P136, P172

Meyer, L P277

Mezzio, D P013, P263*

Mhishi, S P160

Mi, Y P267

Mican, R O43, P057, P179

Mielczak, K O31, P180

Migaud, P P218

Migazzi, C P093, P100

Miguel, M P238

Milano, E P030, P178

Milic, J P316, P331

Milinkovic, A O47, P008, P014, P015, P290

Miller, R P387

Mills, A O49, P211

Min Han, W P308, P369

Minaeva, S P094, P108

Miners, A P384

Mingrone, V P044

Minisci, D P284

Mirabile, A P090

Miranda, A P085, P250, P274

Miranda, M O31*

Miras, H O26

Mirera, I P167*

Miró, J O46A*, O46B*, P075, P080, P125*, P129, P228, P244, P269, P322*

Miron, V P342*

Mirza, H P009

Mizushima, D P309, P362

Mnzava, D P193

Modi, G P353

Moghadassi, M P243

Mohammed, H P020, P227

Mohammed, R P359

Mohammed, T P150

Moi, G P137

Moioli, M P202

Mokebe, M P005

Mokgethi, P P117, P118, P131, P150

Mokhele, K P193

Moko, L P161

Molatelle, M P193

Molero, M O43, P106, P296

Molina, J‐M P042*, P179, P355

Mondi, A P178, P289

Monroy, V P012

Monsel, G P350

Monteiro, M P146

Montejano Sanchez, R P043, P048

Montenegro, D O24

Montero, M O45, P151

Montero Hernández, C P056, P064, P065, P079, P104

Montes, B P133

Montes, M P056, P079

Montezuma‐Rusca, J P049, P050*

Montshosi, P P118

Moodley, R P047

Moody, J P179

Moody, K P335*

Mor, O O31

Moraka, N P117, P118, P131, P150*

Morano, L O45, P056, P064, P065, P079, P081, P104

Mora‐Peris, B P091

Morenilla, S O22

Moreno, A P069*, P072*, P144*, P145*

Moreno, A O46A, O46B, P125, P322

Moreno, C P265

Moreno, S P069, P072, P096, P144, P145, P337

Moreno‐Marques, A P388*

Moriarty, M P203

Morroni, C P196

Morse, G P225

Mortensen, E P035

Moschese, D P030, P132, P287

Moschopoulos, C P089, P207, P258, P318*

Moshashane, N P196

Mosquera, M P080, P129

Motaboli, L P005

Mothobi, B P193

Mothudi, M P150

Motswaledi, M P118

Motta, F P331

Motte, A P206

Mounzer, K O49, P050

Mousadifar, N P098

Moussallem, A P163

Movahedi, R P290

Moya‐Mejias, R P358

Moyle, G P290

Moyo, S P117, P118, P131, P150

Moyo Tetang, S P161

Mponponsuo, K O23

Muccini, C P077, P284, P288, P291

Muhairwe, J P193

Muhumuza, M P162

Mukazi, F P384

Mularska, E P180

Mullen, D P019

Mumm, D P098

Munk, K P281*

Muñoz‐Algarra, M P360

Muñoz‐Andrés, R P027

Muñoz‐Muela, E P223

Murphy, G P020, P227

Musonda, R P118

Musquera, M O46B, P322

Mussa, A P196

Mussi, C P316, P331

Mussini, C P121, P171, P177, P245, P307, P312, P331, P334, P366

Muzanywa, G P118

Müller, A P139*

Müller, A P139

Müller, C P340

Münchhoff, M P142

Mwamba, D P028*

Mwanga, P P162

Mwangi, D P218

Mycock, K P101

### N

Nabipoor, M P280

Naccarato, M P018

Naftali, E P212

Nagimova, F P094, P108

Naguleswaran, N P082

Nahass, R O21

Najarro Cermeño, J P013

Nakagawa, F O42, P324

Nakamoto, T P309, P362

Nakauchi, T P226

Nakyomu, D P169*

Namale, G P169, P176*, P184, P328

Naomi‐Karell, E P161

Naous, N P279

Naqvi, A P282

Nava, A P021, P230

Nava, C P175

Navadeh, S P013, P051, P067, P098

Navarro, C P064

Navarro, J O45

Navarro, R P073

Navarro Saez, M P303

Navarro Vilasaró, M P303

Navarro‐Soler, R P043, P057

Nawa, J P118

Nawej Tshikung, O P246, P253, P255

Ndege, R P193

Ndembi, N P053

Ndjolo, A P053, P161, P190

Neau, D P061, P078, P083

Negredo, E P057

Neidhart, T P017

Neizly, C P380

Nencioni, C P086

Neo, NP196

Neophytou, O P258

Neves, T P158*

Newman Lobato Souza, T O24

Ng, R P160

Nga Motaze, A P161, P190

Ngoufack Jagni Semengue, E P161

Nguendjoung Fainguem, N P161

Nguisseu, G P102

Nicholls, J P249

Nielsen, S P281, P293, P300

Nielson, C P283

Nieuwkerk, P P335

Nijhuis, M O31

Nikolova, M P344

Nishida, Y P226

Niubó, J O22

Njom Nlend, A P161

Nka, A P161*, P190

Nkosiphile, N O49

Nkwe, D P117

Noe, S P057

Nokuthula, NP117*, P131

Noknoi, S O32

Norcross, C P278*

Nordestgaard, B P300

Notter, J P016

Noukayo, F P161

Novak, R O49

Novela, F P032*, P374

Novitsky, V P117

Nozza, S P030*, P177, P288*, P289, P291, P311

Nunes, S P112

Nuñez, J P076

Nunnari, G P090*, P137

Nur, F P295

Nwanbila, M P279

Nyakane, M P005

Nyzhnyk, A P272

### O

Obel, N P370

Oberle, C P254

Obermeier, M P141*, P142*, P218*

Obieta, E P181

Ocampo Hermida, A P043

Odorizzi, P P211

Oette, M P340

Ogaz, D P020, P227

Ogbuagu, O O49*

Oglesby, A P264

Oh, E P049

Ohrtmann, L P241*

Oka, S P309

Okal, E P345*

Okhai, H P249

Okoli, C P040

Okomo, G P345

Olajuwon, F P233

Olalla, J P337

Olatokun, S P233

Olawuyi, D P233

Olczak, A P180

Olive, D P206

Oliveira, S P109

Olszyna, D P025*, P368*

Omar, M P096, P151, P358

Omiste Sanvicente, T P064, P081

Omland, L P370

Omri, A P163

Oprea, C P134, P378*

Orellano, G P230

Orenstein, W O44

Orkin, C O26, P042, P062, P088, P283, P341, P348

Orlova‐Morozova, E P094, P108

Orofino, G P066, P074, P306, P316

Orrell, C P060, P166

Ortiz, V P183, P185

Ortiz, Z P021, P230

Orviz, E P376

Osbak, K P182

Osewe, E P235*

Ostrowski, SR P281, P300

Otter, A O26

Overmars, R P210

Owino, M P163

Owiti, T P345

Ørsted, I P370

### P

Padilla, S P064, P065

Paggi, R P086

Pagliuzza, A P038

Paioni, P P246, P253

Palacios, C P122

Palacios, R O25*, O45, P084*, P364, P367*

Palaparthy, R P036*, P049, P224

Palich, R P041*, P058*, P059, P122*

Panagopoulos, P P124, P138

Panese, S P170

Papachristou, E P124

Papadopoulos, A P089, P124, P207, P258, P318

Papadopoulos, I P232

Papageorgiou, S P318

Papageorgiou, V P385*

Papaioannu, R P120

Papaioannu Borjesson, R P284

Papalini, C P287, P289

Paparello, J P249

Paparini, S P348

Paparizos, V P089

Papastamopoulos, V P124, P207

Papazoglou, D P138

Paraskeva, D P124

Paraskevis, D O31, P124

Parczewski, M O31, P022, P180*

Paredes, D O46B

Paredes, R P042

Parente, A P377

Parenti, P O24

Parinyanitikul, N P308

Parry, C P330

Pascual‐Bernaldez, M P047

Pashalishvili, M P329

Passini, F P311

Pastor, I P026*, P239*

Pastori, L P265

Paterno Raddusa, M P090, P299

Patetta, L P316

Pathirana, J O44

Patton, G P257

Paulicelli, J P346

Pavlica, B P222

Pavon, M P356

Payeras, A P106, P296

Paytubi, S P356

Pedrero Tomé, R P056, P064, P065, P079, P081, P104, P189

Pedrosa Aragón, M P303

Peiffer‐Smadja, N P350

Pelchen‐Matthews, A O42, P324, P336

Pellegrino, M P171

Pellegrino, P P246, P253

Pellicanò, G P066, P090, P306

Pema, M P131*, P150

Pena, E P034, P109

Peng, J P219

Penini, V P265

Peracchi, F P202

Peraire, J P048, P337

Pereira, M P085

Pereira Cruz, G P273

Perera, S P247*

Perez, C P021, P230

Perez, J O43

Pérez, E P175

Pérez‐Hernández, I O25, P364, P367

Perez Martinez, D P065

Perez Rios, A O49

Perez Trejo, M P160

Perez Valero, I O43*, P215*

Perez‐Elias, M P069, P072, P144, P145

Pérez‐Latorre, L P376

Pérez‐Martínez, L O45

Perez‐Stachowski, J P048, P337

Perno, C P053, P161, P178, P190, P205

Perrier, A P061, P083

Perry, M P249

Pet, S P295

Peters, H P001, P002, P003, P004*

Peters, L P272, P307, P334

Peters, S P262*

Petoumenos, K P307

Petrakis, V P138*

Petretti, S P348

Petrini, V P353

Petros, W P343

Peyrouny‐Mazeau, A P061, P078, P083

Peytavin, G P041, P058

Pezzati, L P121

Pham, M P224

Phanuphak, N O49

Philibert, P P082*

Philippe, C P082

Phillips, A P011

Piatti, C O22

Piazza, A P174, P192, P298, P341

Piccica, M P086

Pichon‐Riviere, A P265

Piconi, S P178, P306

Pierone Jr, G P285

Piet, E P204

Pignata, R P379

Pineiro, C P112

Pinheiro, H P112

Pinho, R P112

Pinilla, I P294

Pinnetti, C P006*, P177

Pino, E P064

Pinto, A P043, P073, P376

Pinto, R P010, P024

Pinto, V P183, P185*

Pintos‐Ramos, I P027, P360

Pipitò, L P147

Pirkl, M P039*

Piscu, S P378

Pisculli, M P320

Pistarà, E P090

Pitchford, A P047

Plank, R P042, P320*

Platt, H O44

Podzamczer, D P386

Pollich, S P031

Ponta, G P143

Pontali, E P066, P121, P306

Popa, I P378

Porteiro, N P044

Portilla, J P048, P068

Pos, E P032

Post, F P179, P317, P333

Potthoff, A P313

Poughias, L P089

Pousada, G P056

Póvoas, D P024

Pozniak, A P008, P179

Pozzi, M P086

Pradier, C P071

Prakash, M P116*, P135

Prandini, F P312*

Prelutsky, D P050

Prentice, M P218

Previlon, M P277

Price, K P067*, P213*

Priest, J P330

Prieto‐Pérez, L P099, P381

Prince, M P338

Prolo, A O25, P367

Protopapas, K P207, P258*, P318

Prouvost‐Keller, B P282

Psichogiou, M P124, P207

Puchulu, M P076

Pugliese, A P331

Puglièse, P P122

Puig, J P057

Pujari, S P149

Pulido Ortega, F P043, P073

Puoti, M P202

Pusterla, L P066

Putcharoen, O O32

Puzzolante, C P171

Pylli, M P124

### Q

Qin, H P045*, P046*, P052*, P055*, P070*, P197*, P200*, P267*, P292*, P305*, P321*, P349*, P365*

Quiros Roldan, E P332, P347

Qurishi, N P340

### R

Rabbione, A P132, P287

Raccagni, A P311

Racha‐Pacheco, R P112

Radoi, R P134, P378

Radzikhovskaya, M P094, P108

Rafailidis, P P138

Raffi, F P123

Raghavan, R P329

Ragone, L P002, P008

Rahi, M P350

Rahman, H O26

Raimondi, A P177, P202

Raimondo, V P137

Rajkovic, I P222*

Rakhuba, V P272

Ralegoreng, C P118, P150

Rallón, N P381*

Ramgopal, M O21, O44*, O49, P050, P051*, P211

Ramirez, F P024

Ramos, C P294

Ramos, U P175

Ramos Oliveira, S P315*

Ramos Ruperto, L P064, P376

Ramos Vicente, N P056, P064, P065, P079

Ramos‐Font, C P151

Ramroth, J P063

Ranin, J P222

Ranin, J P222

Rantshabeng, P P217*

Ranzani, A P006

Ranzenigo, M P288

Rashid, I P051

Rasi, M P016

Rasmussen, L P370

Ratanasuwan, W O44

Rauch, A P115

Raymond, S P133

Read, A P387*

Recchia, G P289

Redondo, M P383

Reekie, J P272, P334

Regan, S P036

Reis, F P034, P109, P315

Reiss, P P335

RELATIVITY Cohort P064, P065, P081, P104

Remien, R P166

Revollo, B P048

Rey, D P059

Reynes, J P071

Rezzonico, L P103

Rhee, E O21, P224

Rhee, M O21

Rial, D P073

Ricci, E P066, P299, P306

Ricciardetto, M P312

Riché, A P061, P083

Ridgway, J P023

Ridsdill Smith, E P009

Riera, M P337

Rigo, F P170

Rigo Bonin, R P386

Riguccini, E P086

Rimola, A O46A, O46B, P125, P322

Ring, K P062, P088

Ringera, I P193

Ripamonti, D P128*

Riva, M P332

Rivas, A P105

Rivas‐Jeremias, I P223

Rivero Juarez, A P215

Rivero Roman, A O45, P215

Roberts, D P179

Robineau, O P068, P122, P172

Roca, C P223

Rocha, S P109

Rocha, S P250, P274

Rocha Veiga, A P146

Rockstroh, J P057, P179, P283

Rodallec, A P123

Rodes, B P294

Rodger, A O42, P324, P336, P384, P385

Rodrigues, P P220

Rodrigues Sobral, Z P388

Rodriguez, A P106*, P296*

Rodriguez, A P183*, P185

Rodríguez, S O45, P057

Rodriguez Granges, J P358

Rodriguez‐Centeno, J P294

Rodriguez‐Torres, D P294

Roedling, S P008

Roel, M P076, P093, P100

Rogati, C P202

Roggelin, L P259

Rokka, C P124

Rokx, C P182, P210

Rollo, F P314

Rolón, M O24, P092, P183, P185, P256, P380

Roman, O P125

Romanin, B P147*

Römer, K P340

Romero, A P096, P151

Romero, M P230

Romih Pintar, V P155, P297

Ron, R P144

Rooney, J P334

Ross, J P149

Rossetti, B P086, P121

Rossi, M P170

Rossotti, R P030, P074, P132, P143, P202*, P347

Roukens, A P182

Roustand, L P059, P082

Routy, B P038

Routy, J‐P P038*

Rovira, C P129

Rovito, R P113

Royston, L P038

Rozpłochowski, B P180

Ruane, P O21, O49, P050

Rubin, L P225

Rubinstein, A P265

Rubio, R P073

Rugemalila, M P217

Ruiz, P O46A, P125, P322

Ruiz Pena, E P315

Ruiz‐Seco, P P376

Ruiz‐Tagle, C P251

Rupasinghe, D P149

Ruranska‐Smutnicka, D P361

Rusconi, S P074, P077, P121, P205

Russo Krauss, A P353

Ruxrungtham, K O32

Ryan, P O43, O45, P048, P151, P376

Ryan, R P196*

Ryan‐Murua, P P189

Rybak, T P008

Ryom, L P307, P334

### S

Sabin, C P307, P317, P333

Saborido‐Alconchel, A P223*

Sacewicz, A P361

Sachikonye, M P317, P333, P379

Sadou, A P282

Safran, R P051

Sahni, A P279

Saifi, T P098

Sainz Guerra, M P383

Saiz‐Medrano, G P294

Sakar, S P014, P015

Salazar, E P038

Salmon, D P071

Salom, A P296

Salomoni, E P066, P306

Salpini, R P289

Salusso, D P021

Salvador, S P242

Salvo, P P186*

Samarina, A P002

Samba, B P243

Sambatakou, H P089, P124

Samsonova, O P268

San Joaquín Conde, I P064, P079, P096

Sanchez, R P320

Sánchez, M P310, P356

Sánchez Guirao, A P056, P064, P065, P079

Sanchez Thomas, D P092

Sánchez‐Morales, R P198

Sanchez‐Palomino, S P129

Sando, D P193

Sandoval, M P230

Sandulescu, O P342

Sani, S P086

Sanna, G P137

Santacreu, M P056, P065, P073

Santini, M P216*

Santoro, A P103, P113, P347

Santoro, A P265

Santoro, A P366

Santoro, C P289

Santoro, M P130*, P161

Santos, B O49

Santos, I O45

Santos, J O25, P084, P364, P367

Santos Ramalho Teixeira Benvenuto, A O24

Sanz, J O45, P376

Sánz‐Pérez, M O45

Saracino, A P006, P121

Sarchi, E P066, P306

Sarkar, S O47

Sarmati, L P068

Sarracino, A P077

Sarteschi, G P086

Sasset, L P170*

Saucedo, C P163

Saumoy, M O22, P310, P356*, P386

Saunders, J P020, P227

Sayan, M O31

Scévola, S O22, P386

Schadendorf, D P313

Schaefer, G P031

Schauer, A O22

Schechter, M P212

Scherzer, J P029*

Schim van der Loeff, M P335

Schine, P O49

Schmid, P P115, P246, P253

Schmidt, K P017

Schmiedel, S P031, P259

Schmutz, H P051

Schneeweiß, S P340

Schoeni, S P013

Schommers, P P039

Schönenberger, C P193*

Schreuder, C P060

Schreibman, T O49

Schroeder, M O47, P014, P015, P029

Schübel, N P340

Schuettfort, G P057*, P139

Schulze zur Wiesch, J P031, P111

Schuster, T P148

Sconza, R P001, P002*, P003*, P004

Scott, K P013

Seang, S P058

Seatla, K P117

Sebastiani, G P038

Sedgley, R P067

Sedó, A O22

Seery, P P286

Segal‐Maurer, S P050, P067, P213

Seguin‐Devaux, C O31

Seixas, D P024

Sekayi, W P184

Seleme, S P044

Selepe, P O48

Sellem, B P350

Semengue, E P053

Sempere, A P075, P080, P129, P228, P244, P269

Sendaula, E P169, P176, P184, P328

SenGupta, D P211

Sepúlveda, M P056, P064, P065, P079, P081, P104

Sequera‐Arquelladas, S P151

Serna‐Gallego, A P223

Serrano, A P296

Serrano, C P076

Serrano, J P106

Serrano, L O46A, O46B

Serrao, R P057

Seru, K P118

Sérvio, R P220

Serwin, K O31, P022

Sewell, J O42, P324, P336, P384, P385

Shadreck, T P118, P150

Shah, A P019

Shaik, N P036

Shallvari, A P121

Shao, Y P211

Sharp, H P213

Shaw, J P385

Sheng, W P301, P319

Shenkerman, A O44

Sherman, E P051

Shevchenko, V P094, P108

Shih, C P352*

Shihadeh, F O21

Shiojiri, D P309

Shirasaka, T P226

Shongwe, M P249

Shoukry, A P275*

Sidat, S P348

Sierra‐Madero, J P198

Signori‐Schmuck, A P133

Silling, S P313

Silva‐Klug, A P310, P356

Silvariño, R O45

Simba, B P193

Simoes, P P127

Simões Bassini, A O31

Simon, S P196

Simonsen, C P281

Sinclair, G O21

Singh, R O49

Sipsas, N P124

Siribelli, A P284, P291

Sisto, A P256, P380

Siwak, E P180

Sizova, N P094, P108

Sklar, P P049, P050

Skrzat‐Klapaczynska, A P261

Slim, J P050, P101

Small, M P165

Smith, C O42, P324*, P336, P355

Smith, G P098

Smith, N P225

Smuk, M P062, P088

Soares Laranjeira, F P220

Sobhie Diaz, R P212*

Socrate Adzesi, R P231

Soeiro, C P109, P315

Soffritti, A P245

Sogga Alfano, M P092

Soler‐González, J O45, P056, P064, P081

Somia, I P149

Sophonphan, J O32, P308, P369

Soriano, I O22

Sorni, P P096, P106, P151, P296

Sosso, S P161, P190

Sotiriadou, T P124

Soto, A P175

Sotomayor, C P223

Soulie, C O41, P041, P122, P133

Sourice, J P123, P133

Spadaccini, L P021*

Spagnuolo, V P116, P130, P284, P291

Spampinato, S P090, P137

Spano, J O41*

Sparrowhawk, A O42, P324, P336, P384

Spencer, L O49

Spinner, C P302, P325

Spreen, W P040

Sprinz, E O24

Squillace, N P066*, P074, P306*, P347

Stærke, N P370

Stagnaro, J P110*

Stanitsa, E P318

Starychenko, O P272

Starzynski, K P361

Steffy, T P194

Stehle, T P323

Steinhaus, N P031*

Stelling, A P242

Stephan, C P057, P139

Sterrantino, G P086

Stetsenko, O P229

Stingone, C P314*, P327

Stoch, A P224

Stoeckle, M P016, P246, P253

Stoffels, K O31

Stover, S P214

Strassl, R P017, P354

Streinu‐Cercel, A P342

Stürmer, M P142

Stutterheim, S P335

Su, F P042

Su, Y P319

Su Lwin, H P308, P369

Suanzes‐Martín, E P360

Suarez‐Zdunek, M P281, P293*, P300

Sued, O O24, P209

Sukumaran, L P333*

Sullivan, A P011

Sun, H P152, P319

Sun, L P114

Suonpera, E P295

Supparatpinyo, K P373

Surial, B P016, P115

Swartz, A P247

Sykes, C O22

Szetela, B P022*, P097, P180, P361*

Szuba, P P361

Szymczak, A P022, P097, P180

### T

Tacconi, D P086

Taieb, F P350

Takano, M P309

Takou, D P053, P161

Tallada, J P334

Tamburini, A P311

Tan, D P018

Tan, T P304

Tang, H P152

Tanuma, J P149

Tao, L P013*

Tao, X P219

Taramasso, L P006, P030, P066, P306, P347

Tardei, G P134

Tariq, S P174

Tarr, P P307

Tartaglia, A P299

Tarumbiswa, T P005

Tatipamula, V P196

Tavares, R P220

Tavelli, A P030, P077*, P143, P178, P205, P289, P347

Tavio, M P284

Taylor, G P003

Tchendjou, P P161

Teira, R O45

Teixeira Costa, M P315

Tekle Kiros, S P120

Téllez, F P084

Terrón, A P151

Teruya, K P362

Tesoro, D P030

Tesoro, E P030

Tetart, M P136

Tetteh‐Kwao Teye, J P231

Thahane, L P193

Thielen, A P126, P142

Thill, P P172

Thomas, K P258

Thorne, C P001, P002, P003, P004, P008

Thornhill, J O26, P040*

Thorpe, D P063, P068

Thoueille, P P204

Tian, B P339

Tian, Y P283

Ticchioni, M P282

Tiecco, G P074, P332*

Tieghi, T P007

Timiryasova, A P307*

Tincati, C P103*, P113, P178*

Tiraboschi, J O22, O43, P041, P048, P064, P065, P079, P266*, P283, P386

Tisné, A P093, P100

Tittle, V P009

To, K P351

To, L P351*

Tobbal, Y P323

Tobias, A P181

Todorova, Y P344

Toiber, M P175

Tomás, J P076

Tommasi, A P287, P316

Tommo Tchouaket, M P161, P190

Tong, T P279

Tookes, H P154

Torralba, M P048, P057, P064, P065, P079, P081, P104

Torralba González, M P056

Torres, B P075, P080, P129, P228, P244, P269

Torres, R P376

Tortellini, E P357*

Torti, C P130

Totsikas, C P089, P124, P207

Touzeau‐Roemer, V P017

Trabaud, M P133

Trenti, L P310, P356

Trigo, D P057

Trimis, G P089

Trizzino, M P147

Trøseid, M P293

Trotta, M P353

Trottier, B P063

Troya, J P065, P079, P081*, P104

Troya Garcia, J P056, P266

Trujillo‐Rodríguez, M P223

Truong, D P187*, P188*

Trypsianis, G P138

Tsai, C P164

Tsai, M P301

Tsang, O P351

Tsang, W P098

Tschumi, N P005, P193, P194

Tsekes, G P124

Tsiara, C P124

Tsokos, D P089*

Tsuaneng, M P196

Tumbarello, M P086

Turk, G P209, P214

Turner, L P029

Tusch, E P334*

Tuset, M O46A, P125

### U

Uehira, T P226

Ueji, T P226

Uemura, H P362*

Umoh, P P371

Underhill, A P098

Unger, N P051

Urban, N P017*, P354

Urbanska, A P022

Urioste, A P214

### V

Vacchino, M P021

Vachon, M P280

Valantin, M P058

Valdenmaiier, O P272

Valdez Madruga, J O24, O49

Vale, F P238

Vale Araújo, S P315

Valente, C P112*, P158, P236, P273, P377*

Valenti, D P128

Valiente‐Echeverria, F P209

van Beek, J P182

Van Dam, C P047

van de Vijver, D P210

van der Sluis, D P182

van der Valk, M P335

Van Gerwen, O O49

van Holten, N P182

van Kampen, J O31, P210

van Nood, E P210

van Welzen, B O31, P068

Vandekerckhove, L P054

Vandenhende, M P061, P078, P083

VanderVeen, L P119

Vangelov, D P344

Vannappagari, V P002, P008, P285*, P334

Vanspranghels Gibert, R P172

Varadarajan, M P279*

Vargo, R P224

Varin, T P038

Vasalou, V P089

Vasconcelos, R O49

Vasilakis, A P124

Vasile, S P378

Vasquez, J O49

Vassallo, M P282*

Vassao de Almeida Baptista, M P212

Vaz Pinto, I P112

Vazquez, M P048

Vega, J P092, P183, P185

Vega, J P356

Vega‐Costa, J P310*

Vehreschild, J P307, P334

Velasco Arribas, M O43

Velez, J P377

Velez, M P069, P072, P144, P145

Velez de Mendizabal, N P049

Velonakis, G P318

Veloso, M O31

Veloso, S O45

Venanzi Rullo, E P090

Venturelli, S P030, P128

Vera, J P278, P317, P333, P343

Vera, M P376

Verbon, A O31, P182

Verburgh, M P335

Verdier, G P101*, P330

Vergori, A P205, P346, P347

Verheyen, J P126, P142

Veyri, M O41

Vicente‐López, N P027, P360

Vicenti, I P121

Viciana, I P367

Vidal‐Vilar, N P266

Viget, N P136

Villa, G P355

Villalobos, M O25, P364

Villari, N P090

Villaverde, M P092

Villoslada, A O45, P048, P106, P296

Viloria, G P026, P239

Vincenti, A P086

Vinuesa, D P096, P151

Visconti, E P186

Visicaro, M P366

Vivancos, M O45, P057, P069, P072, P144, P145, P151

Vivancos Gallego, M P056, P064, P065, P079, P081

Viveros, P P175

Vliegenthart‐Jongbloed, K P182

Vock, F P016

Vogelmann, R P068, P126*

Voit, F P302*, P325

Volij, C P265

Volny‐Anne, A P379

Volpe, A P121

Vong, K P213

Voorn, S P174*, P192*, P298*, P341*

Vouillot, C P059, P082

Vriesde, M P182

Vrsaljko, N P216

Vu, T P149*

### W

Wagner, L P302

Wagner, N P246, P253

Wagude, J P345

Wallner, E P334

Walmsley, S P230, P280*

Walsh, J P091

Walter, H P142

Wan, W P045, P052, P055, P292, P305, P321, P349, P365

Wandeler, G P115

Wang, B P156

Wang, F P035, P036

Wang, H O47*, P014, P015

Wang, H P045, P052, P055, P292, P305, P321, P349, P365

Wang, H P373

Wang, M P045, P046, P052, P055, P070, P292, P305, P321, P349, P365

Wang, M P116, P135

Wang, R P114*

Wang, W P221

Wang, X P114

Wang, Y P156

Wannamaker, P P037, P208

Ward, A O48

Watanabe, D P226

Waters, L P249, P283, P295, P317

Weeks, I P213

Wei, H P045, P046, P052, P055, P292, P305, P321, P349, P365

Weimann, L P031, P111

Weinberg, A P051

Weisser, M P193

Welbers, H P242

Weninger, W P017

Wensing, A O31

West, B P379*

Weston, R P091

White, J P249

Whiteman, A P283

Wiadamong, A P161

Wieland, U P313

Wiesmann, C P187, P188

Wiesmann, F P142

Wilches, V P037

Willinger, B P017

Willis, C P051

Wilson, F P324, P336

Wilson, J P234

Winston, A P091, P317, P333

Wirden, M P058

Wit, F O31, P307, P334, P335

Witak‐Jedra, M P180

Witor, A P180

Witzel, T P385

Wohlfeiler, M P285

Woldesemayat, E P343*

Wolf, E P142, P302, P325*

Wome, B P161

Wong, A P063*

Wong, B P351

Wong, P O49

Woodward, K P012*

Wu, H P045, P046, P052, P055, P221, P292, P305, P321, P349, P365

Wu, S P070

Wu, S P219

Wu, Y P301

### X

Xia, H P157*

Xiao, D P036, P049

Xiao, L P197

Xie, X P087

Xu, J P264

Xu, Z P042, P320

### Y

Yager, J O48

Yagura, H P226*

Yakouba, L P161

Yancheva, N P344

Yang, C P068, P152, P301

Yang, C P267

Yang, J P013

Yang, J P199*

Yang, T P159*

Yang, X P087

Yared, N P051

Yazdanpanah, Y P350

Yi, S P187, P188

Yienya, N P243

Yildirim, Ö P141, P218

Yimga, J P161

Yin, M P140*

Yoshino, M P226

Young, L P307, P334

Yu, F P046

Yuan, Y P221

Yuan, Z P140

Yunihastuti, E P149

### Z

Zabek, P P180

Zaccarelli, M P314, P327

Zaegel‐Faucher, O O41, P206*

Zalazar, V P230

Zammarchi, L P353

Zamora‐Clemente, E P228, P244, P269

Zampetas, G P258

Zangerle, R P334

Zaninetta, E P074

Zareba, D P361

Zazzi, M P121, P130

Zeggagh, J P058

Zekan, S P155*, P216, P297*

Zeng, F P102

Zeng, H P267

Zeng, Y P267

Zhai, Y P114

Zhang, C P070, P197, P200, P267

Zhang, F P045, P046, P052, P055, P292, P305, P321, P349, P365

Zhang, H P035, P224

Zhang, M P225

Zhang, Q P070

Zhang, X P050

Zhao, Q P045, P046, P052, P055, P292, P301, P305, P321, P349, P365

Zhao, Y O48

Zhao, Y P197

Zheng, Y P070, P197, P200, P267

Zhou, J P336

Zhou, M P267

Zhou, R P159

Zhu, Y P156

Zhumi, R P380*

Zielinska, K P022, P097

Zimmermann, H O47, P014*, P015

Zinczuk, A P022, P097

Zingaropoli, M P357

Zisaki, S P138

Zitko, P P105, P181

Zorz, M P026, P239

Zou, M P301

Zoulas, D P207

Zuccalà, P P007, P107

Zurashvili, T P329

Zuze, B P118, P131

Zwane, Z O49

